# UBE2T promotes breast cancer tumor growth by suppressing DNA replication stress

**DOI:** 10.1093/narcan/zcac035

**Published:** 2022-11-02

**Authors:** Roshan Dutta, Praveen Guruvaiah, Kiran Kumar Reddi, Suresh Bugide, Dhana Sekhar Reddy Bandi, Yvonne J K Edwards, Kamaljeet Singh, Romi Gupta

**Affiliations:** Department of Biochemistry and Molecular Genetics, The University of Alabama at Birmingham, Birmingham, AL 35233, USA; Department of Biochemistry and Molecular Genetics, The University of Alabama at Birmingham, Birmingham, AL 35233, USA; Department of Biochemistry and Molecular Genetics, The University of Alabama at Birmingham, Birmingham, AL 35233, USA; Department of Biochemistry and Molecular Genetics, The University of Alabama at Birmingham, Birmingham, AL 35233, USA; Department of Biochemistry and Molecular Genetics, The University of Alabama at Birmingham, Birmingham, AL 35233, USA; Department of Biochemistry and Molecular Genetics, The University of Alabama at Birmingham, Birmingham, AL 35233, USA; Department of Pathology and Laboratory Medicine, Brown University, Providence, RI 02912, USA; Department of Biochemistry and Molecular Genetics, The University of Alabama at Birmingham, Birmingham, AL 35233, USA; O’Neal Comprehensive Cancer Center, The University of Alabama at Birmingham, Birmingham, AL 35233, USA

## Abstract

Breast cancer is a leading cause of cancer-related deaths among women, and current therapies benefit only a subset of these patients. Here, we show that ubiquitin-conjugating enzyme E2T (UBE2T) is overexpressed in patient-derived breast cancer samples, and UBE2T overexpression predicts poor prognosis. We demonstrate that the transcription factor AP-2 alpha (TFAP2A) is necessary for the overexpression of UBE2T in breast cancer cells, and UBE2T inhibition suppresses breast cancer tumor growth in cell culture and in mice. RNA sequencing analysis identified interferon alpha–inducible protein 6 (IFI6) as a key downstream mediator of UBE2T function in breast cancer cells. Consistently, UBE2T inhibition downregulated IFI6 expression, promoting DNA replication stress, cell cycle arrest, and apoptosis and suppressing breast cancer cell growth. Breast cancer cells with IFI6 inhibition displayed similar phenotypes as those with UBE2T inhibition, and ectopic IFI6 expression in *UBE2T*-knockdown breast cancer cells prevented DNA replication stress and apoptosis and partly restored breast cancer cell growth. Furthermore, UBE2T inhibition enhanced the growth-suppressive effects of DNA replication stress inducers. Taken together, our study identifies UBE2T as a facilitator of breast cancer tumor growth and provide a rationale for targeting UBE2T for breast cancer therapies.

## INTRODUCTION

Breast cancer is the most commonly diagnosed cancer type and the second-leading cause of cancer-related deaths among women (1,2). The current 5-year overall survival (OS) rate is estimated at 27% for locally advanced, unresectable disease (3). Breast cancer is categorized based on appearance as tubular, mucinous, medullary, or papillary (4,5). Breast cancer subtypes are also classified based on the presence or absence of hormone receptors (HRs), including estrogen receptor (ER) and progesterone receptor (PR) (6). In addition to differences in HR expression, breast cancers are classified based on the overexpression of human epidermal growth factor receptor 2 (HER2), which is detected in 10–15% of breast cancer cases (6). Approximately 15%–20% of breast cancers are classified as triple-negative breast cancer (TNBC), characterized by the absence of ER, PR and HER2 expression (6).

Therapeutic options for breast cancer include surgery combined with chemotherapy (7), although locally advanced, unresectable, or metastatic disease is typically treated using chemotherapy combined with radiotherapy, hormonal therapy, targeted therapy, or immunotherapy (8,9). However, existing treatment options do not provide long-term survival benefits in patients with metastatic disease, and in many cases, the disease becomes resistant to therapy or relapses after an initial response (10–12). Because the TNBC subtype does not express any HRs, patients with TNBC do not respond to hormonal therapies, making these cases difficult to treat and leading to a generally poor prognosis (6,13). To achieve better clinical outcomes, an improved understanding of the underlying breast cancer pathogenesis remains necessary to identify novel breast cancer drivers for the development of targeted therapies.

Diverse cellular pathways are regulated by the covalent conjugation of ubiquitin to proteins through the concerted actions of a ubiquitin-activating enzyme (E1), a ubiquitin-conjugating enzyme (E2), and a ubiquitin ligase (E3). Previous studies have shown that E2 enzymes play important roles in the ubiquitination of cellular proteins, with effects on the genesis and progression of various cancer types (14,15). Ubiquitin-conjugating enzyme E2T (UBE2T) is an essential E2 enzyme (16,17) involved in the efficient repair of damaged DNA in the Fanconi anemia pathway (18), and the regulation of this pathway may involve a UBE2T self-inactivation mechanism (18). In addition, UBE2T-mediated ubiquitination regulates several cancer-relevant pathways, including the ubiquitination of AKT, which activates the AKT/β-catenin pathway (16), and the ubiquitination of β-catenin, which induces its nuclear translocation (19). UBE2T also promotes autophagy via the p53/AMP-activated protein kinase (AMPK)/mammalian target of rapamycin (mTOR) signaling pathway in lung adenocarcinoma (20).

In this study, we found that UBE2T is overexpressed in breast cancer and that UBE2T overexpression predicts poor prognosis. UBE2T expression is regulated by transcription factor AP-2 alpha (TFAP2A), and UBE2T inhibition suppresses breast cancer tumor growth. Mechanistically, UBE2T inhibition in breast cancer cells suppresses interferon alpha–inducible protein 6 (IFI6) expression, resulting in DNA replication stress, cell cycle arrest, and apoptosis induction. However, the ectopic expression of IFI6 in *UBE2T*-knockdown cells prevented DNA replication stress and apoptosis and partly restored breast cancer cell growth. Furthermore, UBE2T inhibition enhanced the growth-suppressive effects of DNA replication stress inducers. Therefore, our studies indicate that UBE2T acts as a facilitator of breast cancer growth and may represent a novel target for breast cancer therapy.

## MATERIALS AND METHODS

### Cell culture

The MCF7, T47D and HEK293T cell lines were obtained from American Type Culture Collection (Manassas, VA, USA) and maintained in a humidified atmosphere containing 5% CO_2_ at 37°C in Dulbecco's modified Eagle's medium (Life Technologies, Carlsbad, CA, USA) or Roswell Park Memorial Institute (RPMI)-1640 medium (Life Technologies), as recommended. All media were supplemented with 10% fetal bovine serum (Life Technologies) and 1% penicillin/streptomycin (Life Technologies).

### Mouse tumorigenesis experiments

Female NSG mice (Jackson Laboratory, Stock No. 005557, Bar Harbor, ME, USA), 5–6 weeks of age, were subcutaneously injected in the flank with 5 × 106 breast cancer cells (MCF7 and T47D) expressing either non-specific (NS) or UBE2T small hairpin RNAs (shRNAs). Tumor volumes were measured every week. Tumor sizes were calculated using the following formula: length × width2 × 0.5. All protocols for mouse experiments were approved by the Institutional Animal Care and Use Committee of the University of Alabama at Birmingham.

### shRNA and lentivirus preparation

pLKO.1 lentiviral vector–based shRNAs targeting specific candidate genes and NS control shRNAs were obtained from Horizon Discovery. Details regarding the shRNAs are provided in Supplementary Table S5. Lentiviral particles were prepared by transfecting HEK293T cells with either gene-specific shRNA plasmids or NS shRNA plasmids, together with lentiviral packaging plasmids (described in detail at https://portals.broadinstitute.org/gpp/public/resources/protocols). All lentiviral transfections were performed using Effectene Transfection Reagent (Qiagen, Hilden, Germany). Stable cell lines were generated by infecting breast cancer cells seeded in 12-well plates with shRNA lentiviral particles, followed by selection with appropriate concentrations of puromycin (0.5–1.5 μg/ml) to enrich infected cells. For the pLX304-Blast-V5–based lentivirus, infected breast cancer cells were selected using 2 μg/ml blasticidin (ThermoFisher Scientific, Waltham, MA, USA).

### Plasmids

V5-tagged *IFI6* lentiviral expression construct (plasmid pLX304-Blast-V5) was purchased from Horizon Discovery (Waterbeach, UK) and is listed in Supplementary Table S5.

### RNA preparation, cDNA preparation, reverse transcription and quantitative PCR (qPCR) analysis

Total RNA was extracted with TRIzol Reagent (Invitrogen, Carlsbad, CA, USA) and purified using the RNeasy Mini Kit (Qiagen). cDNA was generated using the M-MuLV First Strand cDNA Synthesis Kit (New England Biolabs, Ipswich, MA, USA), according to the manufacturer's instructions. qPCR was performed using gene-specific primers with the Power SYBR-Green Master Mix (Applied Biosystems, Foster City, CA, USA), according to the manufacturer's instructions. The beta-actin (*ACTB*) gene was used as a normalization control. The primer sequences for all genes analyzed in this study are provided in Supplementary Table S5.

### Immunoblot analysis

Whole-cell protein extracts were prepared using IP Lysis Buffer (Pierce Chemical, Rockford, IL, USA) containing Protease Inhibitor Cocktail (Roche, Basel, Switzerland) and Phosphatase Inhibitor Cocktail (Sigma-Aldrich, St. Louis, MO, USA). Lysed samples were centrifuged at 12 000 rpm for 40 min, and the clarified supernatants were stored at −80°C. Protein concentrations were determined using Bradford Protein Assay Reagent (Bio-Rad Laboratories, Hercules, CA, USA). Equal amounts of protein samples were electrophoresed on 10% or 12% sodium dodecyl sulfate (SDS)-polyacrylamide gels and transferred onto polyvinylidene difluoride (PVDF) membranes (Millipore, Burlington, MA, USA) using a wet transfer apparatus (Bio-Rad). Membranes were blocked in 5% skim milk prepared in Tris-buffered saline containing 0.1% Tween-20 (TBST) and probed with primary antibodies. After washing, the membranes were incubated with the appropriate horseradish peroxidase (HRP)-conjugated secondary antibodies (1:2000; GE Healthcare Life Sciences, Marlborough, MA, USA). The blots were developed using SuperSignal West Pico or Femto Chemiluminescent Substrate (ThermoFisher Scientific). All antibodies used for immunoblotting are listed in Supplementary Table S5.

### Flow cytometry analysis

MCF7 and T47D cells expressing either NS or *UBE2T* shRNA were analyzed for cell cycle phases using fluorescence-activated cell sorting (FACS). Cells were collected, washed twice with ice-cold 1 × phosphate-buffered saline (PBS), and fixed in 70% ethanol overnight. The cells were then washed three times with 1× PBS and resuspended in 400 μl propidium iodide/Triton X-100 staining solution [0.1% (v/v) Triton X-100 in PBS with 2 mg DNAse-free RNase and 0.40 ml 500 μg/ml propidium iodide]. The samples were incubated for 15 min at 37°C. Flow cytometry analysis was performed using a BD LSRFortessa (BD Biosciences, San Jose, CA, USA), and samples were analyzed using FlowJo software (Ashland, OR, USA).

### DNA fiber assay

DNA fiber assays were performed as described previously (21). Briefly, cells were plated in the appropriate medium until they reached 30–40% confluency. After 48 h, iododeoxyuridine (IdU; Sigma-Aldrich: I7125) was added to exponentially growing cells (final concentration: 25 μM), and the cells were incubated for 30 min at 37°C in 5% CO_2_. Then, cells were washed with PBS and incubated with a second label, chlorodeoxyuridine (CldU; Sigma-Aldrich: C6891), at a final concentration of 250 μM for an additional 30 min at 37°C. The cells were then trypsinized and counted. A 3-μl cell suspension containing 2 × 10^3^ cells were applied to the end of a glass slide and air-dried for 5 min. The cells were lysed by adding 7 μl lysis solution (50 mM ethylenediaminetetraacetic acid and 0.5% SDS in 200 mM Tris–HCl, pH 7.6). The glass slides were placed at a 15° angle to allow the DNA fibers to spread across the length of the slide and then placed horizontally to air dry. The slides were fixed with methanol:acetic acid (3:1) for 10 min, washed with double-distilled water, and treated with 2.5 M HCl for 30 min. The fixed cells were blocked with 5% bovine serum albumin (BSA) for 30 min at room temperature and incubated with primary antibodies (anti-BrdU [mouse antibody, BD Biosciences #347580] for IdU at a 1:25 dilution and anti-BrdU [rat antibody, Abcam, Cambridge, UK #ab6326] for CldU at a 1:400 dilution, each in 5% BSA) for 1 h at room temperature in a humidified chamber. The slides were then washed three times with 1× PBS for 5 min and incubated with secondary antibodies (1:500 sheep anti-mouse Cy3, Sigma, Cat# C218-M for IdU; and 1:400 goat anti-rat Alexa Fluor 488, Invitrogen, cat A11006 for CldU) in 5% BSA for 1 h at room temperature in the dark. The glass slides were then washed and visualized at 60× magnification to locate the fibers. Pictures were captured with one color channel, and data were analyzed with ImageJ software.

### Immunohistochemistry (IHC)

Formalin-fixed, paraffin-embedded tissue microarray (TMA) slides containing breast cancer samples and matched normal breast tissues were obtained from US Biomax (#BC081120f). Briefly, following slide deparaffinization, antigen retrieval was performed in citrate buffer (pH 6.0) at 97°C for 20 min, using the Lab Vision PT Module (ThermoFisher Scientific). Endogenous peroxides were blocked by incubation in hydrogen peroxide for 30 min, followed by washing with 1× Tris-buffered saline, and proteins were blocked by incubation with 0.3% BSA for 30 min. Slides were incubated in anti-UBE2T antibody (dilution 1:1000) or anti-IFI6 antibody (dilution 1:1000), followed by secondary anti-rabbit HRP-conjugated antibody (Dako, Jena, Germany). Slides were then stained using the Dako Liquid DAB+ Substrate Chromogen System and counterstained with Dako Automation Hematoxylin Histological Staining Reagent. UBE2T and IFI6 staining was scored by Dr Kamaljeet Singh, who was blinded to the identity of each slide. All antibodies used for IHC analyses are listed in Supplementary Table S5.

### Breast cancer dataset UBE2T and IFI6 expression analyses

Datasets comparing gene expression between breast cancer and normal breast tissues were identified by searching the Oncomine cancer profiling database. The Cancer Genome Atlas (TCGA) breast (22), Curtis breast (23) and Zhao breast (24) datasets were analyzed. The TCGA breast dataset includes 389 invasive ductal breast carcinoma samples and 61 normal breast duct samples. The Curtis breast dataset includes 114 normal breast samples and 1556 invasive ductal breast carcinoma samples. The Zhao breast dataset includes three normal breast samples and 36 invasive ductal breast carcinoma samples. All three datasets were analyzed for UBE2T gene expression. The Curtis breast and TCGA breast datasets were used to analyze the co-expression of various transcription factors and *UBE2T* at the mRNA level. The TCGA breast invasive carcinoma PanCancer dataset (1084 samples) was analyzed for UBE2T alterations (https://www.cbioportal.org). Gene Expression Profiling Interactive Analysis (GEPIA), a web server for cancer and normal gene expression profiling and interactive analyses, was used to analyze IFI6 and UBE2T expression and identify associations between overall survival (OS) and high and low UBE2T expression levels (25). Breast invasive carcinoma samples were analyzed for TFAP2A transcript levels using the UALCAN bioinformatics tool (http://ualcan.path.uab.edu/index.html). Km plotter was used to plot OS and relapse-free survival (RFS) among patients with high and low UBE2T expression levels (https://kmplot.com/analysis/). DepMap portal was used to analyze the transcript levels of various genes in breast cancer samples (https://depmap.org/portal/).

### Transcription factor analysis using PROMO

Transcription factors binding with 100% sequence identity on the promoter region of *UBE2T* were identified using the PROMO tool (http://alggen.lsi.upc.es/cgi-bin/promo_v3/promo/promoinit.cgi?dirDB=TF_8.3) (26). We selected a 2-kB upstream promoter region of the *UBE2T* gene to identify human transcription factors able to bind with 0% dissimilarity using PROMO.

### Sample preparation for RNA sequencing

MCF7 and T47D cells expressing NS or *UBE2T* shRNA were used to prepare total RNA, which was then used for gene expression analysis on an Illumina HiSeq 2500 system. Total RNA was extracted using TRIzol^®^ reagent (Invitrogen), according to the manufacturer's instructions, and purified on RNeasy mini columns (Qiagen), according to the manufacturer's instructions. mRNA was purified from ∼500 ng total RNA using oligo-dT beads and sheared by incubation at 94°C. Following first-strand synthesis with random primers, second-strand synthesis was performed with dUTP to generate strand-specific sequencing libraries. The cDNA libraries were then end-repaired and A-tailed. Adapters were ligated, and second-strand digestion was performed using uracil–DNA–glycosylase. Indexed libraries that met appropriate cutoffs for both were quantified by qPCR using a commercially available kit (KAPA Biosystems, Wilmington, MA, USA). The insert size distribution was determined using LabChip GX or an Agilent Bioanalyzer. Samples with a yield ≥0.5 ng/μl were used for sequencing on the Illumina HiSeq 2500 system. Images generated by the sequencers were converted into nucleotide sequences by the base-calling pipeline RTA 1.18.64.0 and stored in fastq format.

### RNA sequencing and data analysis

RNA sequencing was performed for two cell lines (MCF7 and T47D) expressing *UBE2T*-specific shRNAs or NS shRNA. RNA sequencing was carried out for 15 samples. The MCF7 samples (*n* = 9) comprise three biological replicates for three groups (control, shRNA1, shRNA2). The T47D samples (*n* = 6) consist of two biological replicates for three groups (control, shRNA1, shRNA2). Single-end 75-bp reads were generated using the Illumina NextSeq500 sequencing instrument. Pre-alignment quality assessments of the raw fastq sequences were conducted using FastQC (version 0.11.7) (27). The raw fastq sequences were aligned to the human hg38 reference genome (GenBank assembly accession: GCA_000001405.28) using STAR (version 2.7.1a) (28) with default parameters. Post-alignment quality assessments were performed using RSeQC (version 2.6.3) (29). Samtools (version 0.0.19) (30) and IGV (version 2.6.2) (31) were used to index and view the alignments, respectively. Gene expression was quantified as gene-level counts using the htseq-count function (version 0.12.3) and the UCSC gene annotations for the human genome. The htseq-count default parameters were used, except for the strand parameter, which was set to ‘reverse’ to account for the strandedness of the library. Differentially expressed genes were identified using DESeq2 (version 1.28) with default parameters (32). Genes with *p*-values less than 0.05 were considered differentially expressed. Interactivenn was used to generate Venn diagrams (33). The normalized gene expression data were used for downstream analyses. The complex heatmap package version 1.12.0 (34) was used to generate heatmaps. To determine altered cellular functions in both cell lines under different treatment conditions, over-representation enrichment analysis was performed using the WEB-based GEne SeT AnaLysis Toolkit (Webestalt) (35), with the genome as the reference set and the community-contributed_Hallmark50 database as the functional database. A hypergeometric test was used to test for the over-representation of functions among the differentially expressed genes common to both cell lines. The Benjamini and Hochberg method was used to calculate adjusted *P*-values (*q*) with the significance cutoff filter set to *q* < 0.05.

### Soft-agar assay

Soft-agar assay was performed by seeding 5 × 10^3^ breast cancer cells stably expressing the indicated shRNA or cDNA constructs onto 0.4% low-melting-temperature agarose (Sigma-Aldrich) layered on top of 0.8% agarose. After 3–4 weeks of incubation, colonies were stained with 0.005% crystal violet and imaged using a microscope. Colony sizes were measured using ImageJ software (https://imagej.nih.gov/ij/) and plotted. Statistical analyses were performed using Student's *t*-test in GraphPad Prism 7 software (GraphPad, San Diego, CA, USA).

### Cleavage under targets & release using nuclease (CUT&RUN) assay

CUT&RUN assays were performed in MCF7 cells using the CUT&RUN Assay Kit (Cat#86652; Cell Signaling Technology Danvers, MA, USA), according to the manufacturer's instructions. Briefly, 2 × 10^5^ cells were harvested, washed, bound to activated concanavalin A–coated magnetic beads, and permeabilized. The bead–cell complexes were incubated overnight with the appropriate antibody at 4°C. The complexes were washed three times, and the cells were resuspended in 100 μl protein A and G/micrococcal nuclease (pAG/MNase) and incubated for 1 h at room temperature. The samples were then washed three times with digitonin buffer containing protease inhibitors, resuspended in 150 μl digitonin buffer, and incubated for 5 min on ice. MNase was activated by adding calcium chloride, and the samples were incubated at 4°C for 30 min. The reaction was stopped by adding 150 μl stop buffer, and the samples were incubated at 37°C for 10 min to release the DNA fragments. DNA was extracted using the DNA purification columns included in the CUT&RUN Assay Kit. qPCR was performed using *UBE2T* promoter–specific primers, and relative fold change was calculated as the ratio of immunoprecipitated DNA to IgG-precipitated DNA. The primer sequences and antibodies used for the CUT&RUN assays are listed in Supplementary Table S5.

### Apoptosis measurement via annexin V/propidium iodide (PI) FACS assay

Annexin V binding to cells (MCF7 and T47D cells expressing either NS, *UBE2T*, or *IFI6* shRNA) was measured using an Annexin V staining kit (BD Pharmingen™ #556547, BD Pharmingen, San Diego, CA, USA), according to the manufacturer's protocol. In brief, after 24 h of plating, cells were collected, washed twice with 1 × PBS, resuspended in 1× binding buffer, and stained with 5 μl FITC-Annexin V and 5 μl PI. After incubation for 15 min in the dark, cells were analyzed with FACS using LSR Fortessa (BD Biosciences, Franklin Lakes, NJ, USA).

### 3-(4,5-Dimethylthiazol-2-yl)-2,5-diphenyltetrazolium bromide (MTT) assay

To perform the MTT assay, 5 × 10^3^ MCF7 cells expressing either NS or *UBE2T* shRNA were plated in a final volume of 100 μl in 96-well plates. After 24 h, hydroxyurea and aphidicolin were added to 100 μl media at a range of concentrations as shown in figure and were then added to the cells. After 72 h of inhibitor treatment, cell viability was evaluated by adding 20 μl of 5 mg/ml MTT solution dissolved in 1 × PBS to each well, followed by incubation for 1 h in a 37°C incubator. The MTT solution was gently removed, and 100 μl dimethyl sulfoxide was added to each well. After mixing by pipetting, absorbance was measured at 590 nm and 630 nm using the Biotek Synergy MX Multi-Format Microplate Reader (Winooski, VT, USA). The average measurement at 630 nm was subtracted from the average measurement at 590 nm, and the relative cell viability at each concentration was plotted with respect to vehicle-treated cells.

### Statistical analysis

All experiments were conducted in at least three biological replicates. The results for individual experiments are expressed as the mean ± standard error of the mean (SEM). To assess measurements of tumor progression in mice and MTT assays, statistical analyses were performed by analyzing the area under the curve using GraphPad Prism software, version 7.0, for Macintosh. In all other experiments, *P*-values were calculated using a two-tailed unpaired Student's *t*-test in GraphPad Prism software, version 7.0, for Macintosh.

## RESULTS

### UBE2T is overexpressed in breast cancer patient samples and predicts poor prognosis

**Figure 1.**
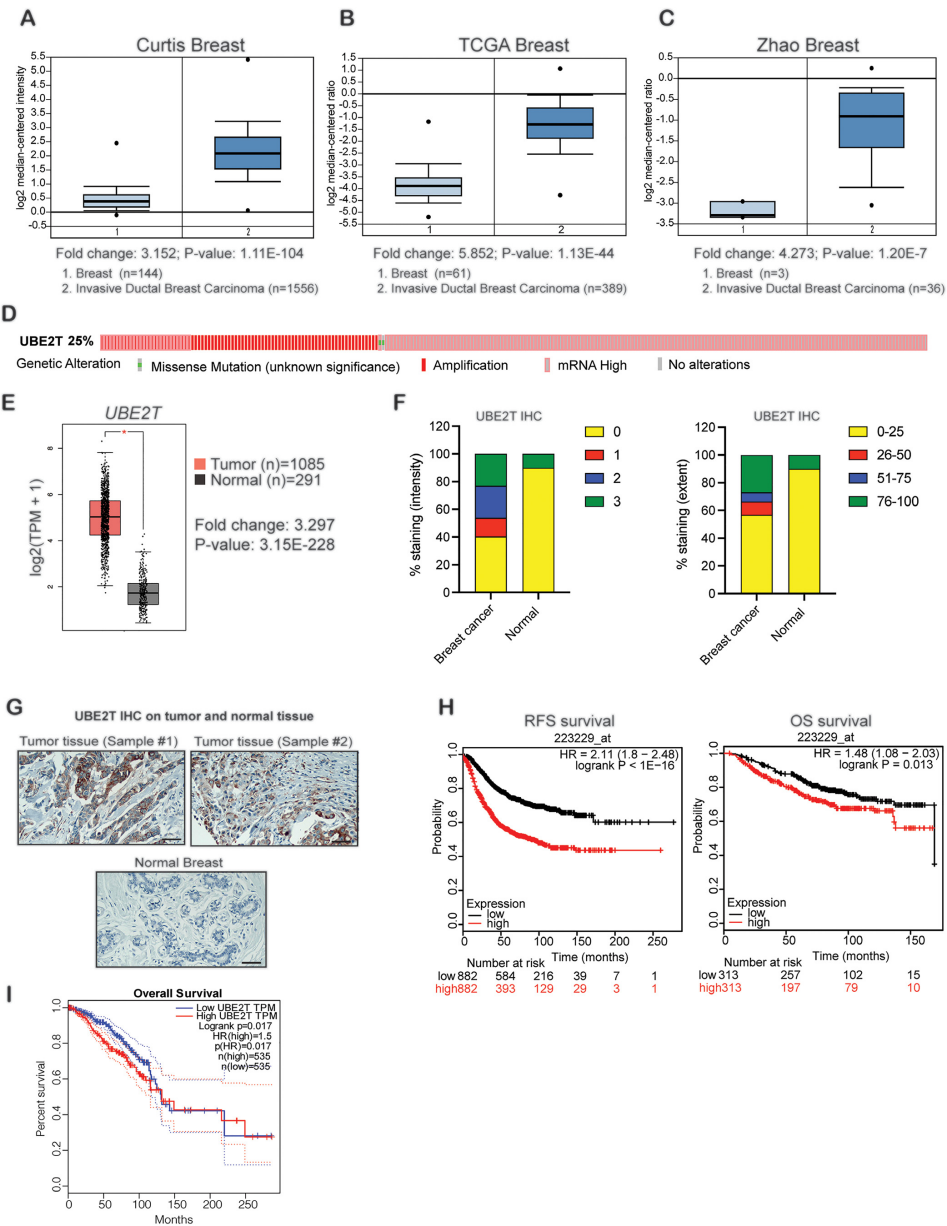
Ubiquitin-conjugating enzyme E2T (UBE2T) is overexpressed in patient-derived samples of invasive ductal breast carcinoma (breast cancer). (**A**–**C**) The indicated patient-derived breast cancer datasets were analyzed for *UBE2T* mRNA expression. Upregulation of *UBE2T* mRNA in breast cancer samples was observed compared with normal breast samples. (**D**) *UBE2T* mRNA alterations were analyzed using cBioPortal. Representative schematic showing the amplification and mutational status of *UBE2T* mRNA in patient-derived breast cancer samples. (**E**) *UBE2T* mRNA expression in normal breast tissues compared with breast tumor samples was analyzed using Gene Expression Profiling Interactive Analysis (GEPIA). (**F**) Analysis of immunohistochemical (IHC) staining data from a tissue microarray (TMA) featuring breast cancer samples and matched adjacent normal breast tissues. The average intensities and the extents of UBE2T staining in breast cancer samples and normal breast tissues are shown. (**G**) Protein expression was measured *via* IHC staining in a TMA containing breast cancer samples and matched normal adjacent breast tissues (20 × magnification). Representative images are shown. Scale bar, 50 μm. (**H**) The effects of *UBE2T* expression levels in patient-derived breast cancer samples on relapse-free survival and overall survival were analyzed using a Kaplan–Meier plot. (**I**) Overall survival was analyzed in patient-derived breast cancer samples with both high and low UBE2T expression levels using GEPIA.

UBE2T has been implicated as an important mediator of cancer growth and metastasis in several cancer types (16,36–38). Based on these findings, we examined whether UBE2T is overexpressed in breast cancer and whether its overexpression predicts poor prognosis. We analyzed publicly available breast cancer datasets and found that *UBE2T* mRNA was significantly overexpressed in breast cancer samples due to both gene amplification and transcriptional upregulation [TCGA breast (22), Curtis breast (23), and Zhao breast (24) datasets] (Figure 1A–E). To confirm UBE2T overexpression at the protein level, we analyzed a breast cancer TMA (#BC081120f), which included 100 cases of invasive breast cancer tissue and 10 adjacent normal breast tissues (Supplementary Table S1). We performed IHC analysis, which showed the significant elevation of UBE2T protein expression in the large majority of patient-derived breast cancer samples relative to normal breast tissue samples, consistent with gene amplification and transcriptional upregulation (Figure 1F, G). We next examined whether UBE2T overexpression was associated with prognosis and found that breast cancer samples with higher UBE2T expression levels were associated with shorter on relapse-free survival and (RFS) and shorter overall survival (OS) (Figure 1H, I) among breast cancer patients. Collectively, these results demonstrate that UBE2T is overexpressed in breast cancer and predicts poor prognosis.

### UBE2T is necessary for breast cancer tumor growth

**Figure 2.**
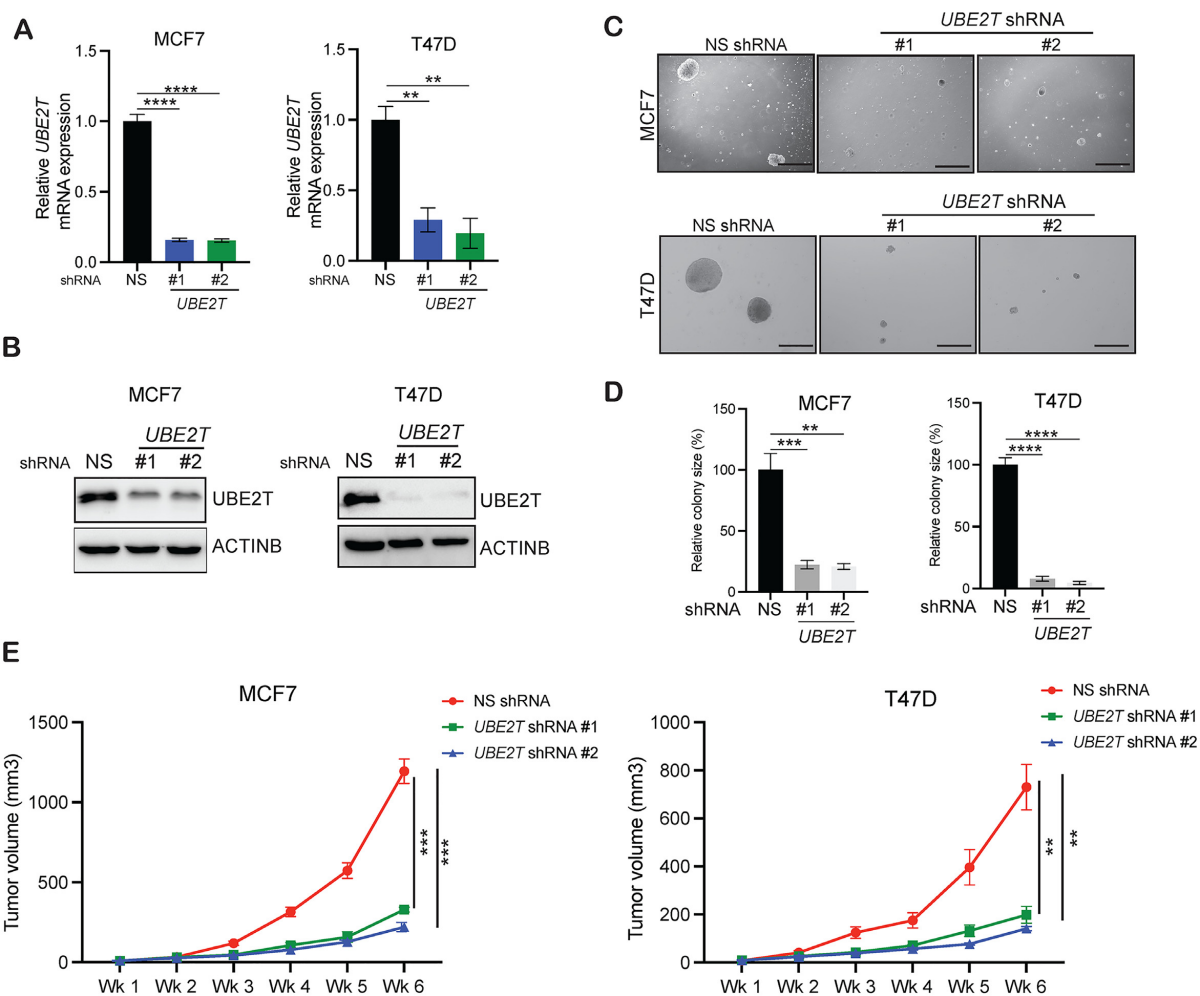
Loss of *UBE2T* expression inhibits breast cancer tumor growth. (**A**) MCF7 and T47D cells expressing either non-specific (NS) small hairpin RNA (shRNA) or *UBE2T* shRNAs were analyzed by quantitative reverse-transcriptase polymerase chain reaction (qPCR) to examine *UBE2T* mRNA expression. *UBE2T* mRNA expression levels are presented relative to levels in cells expressing NS shRNA. (**B**) MCF7 and T47D cells expressing *UBE2T* or NS shRNA were analyzed for UBE2T protein expression by immunoblotting. Beta-actin (ACTINB) was used as the loading control. (**C**) MCF7 and T47D cells expressing *UBE2T* or NS shRNA were analyzed using soft-agar assays. Representative images are shown. Scale bar, 500 μm. (**D**) Colony sizes for the indicated breast cancer cell lines expressing *UBE2T* or NS shRNA from the experiment shown in panel C. (**E**) MCF7 and T47D cells expressing *UBE2T* or NS shRNA were injected subcutaneously into the flanks of mice (n = 5) and analyzed for tumor formation. Average tumor volumes measured at the indicated time points are shown. Data represent the mean ± SEM. **P< 0.01, ***P< 0.001, ****P< 0.0001.

Breast cancer can be classified into several different subtypes based on the presence or absence of HRs (6). The luminal A breast cancer subtype, which expresses ER or PR but not HER2, accounts for 73% of all breast cancer cases and is the most common and slowest growing subtype. Because *UBE2T* is overexpressed in breast cancer, we rationalized that *UBE2T* might be involved in breast cancer tumor growth. Therefore, we examined whether the inhibition of *UBE2T* expression prevents tumor growth in the highly prevalent luminal A breast cancer subtype. We used two sequence-independent shRNAs to knock down *UBE2T* expression in the luminal A breast cancer cell lines MCF7 and T47D (Figure 2A, B). After *UBE2T* knockdown was validated in these breast cancer cell lines via qPCR and immunoblotting, these cells were tested for their abilities to grow in an anchorage-independent manner using soft-agar assays. Breast cancer cells expressing an NS shRNA were used as a negative control. Anchorage-independent growth in soft agar serves as a reliable surrogate assay for *in vivo* tumorigenesis (39). We found that *UBE2T* knockdown in breast cancer cells significantly reduced their abilities to form colonies in soft agar (Figure 2C, D).

Based on these results, we examined whether *UBE2T* knockdown also inhibits breast cancer tumor growth using an *in vivo* xenograft mouse model of breast cancer. We subcutaneously injected breast cancer cells (MCF7 and T47D) expressing either *UBE2T* or NS shRNA into the flanks of immunodeficient female NSG mice. Consistent with the cell culture studies, *UBE2T* knockdown inhibited breast cancer tumor growth *in vivo* (Figure 2E). Collectively, these results demonstrate that UBE2T inhibition prevents breast cancer tumor growth in both cell culture and mice.

### Transcription factor TFAP2A activates UBE2T overexpression in breast cancer cells

**Figure 3.**
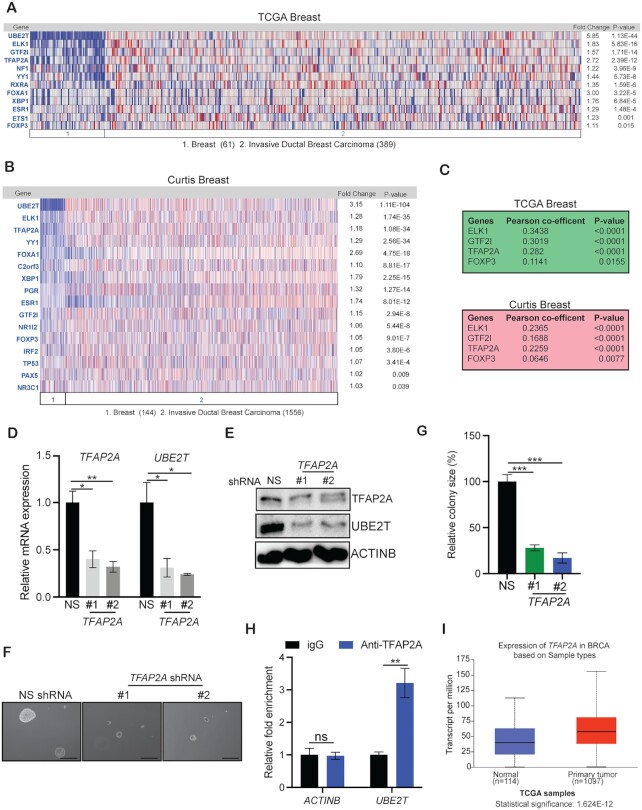
TFAP2A regulates UBE2T expression in breast cancer cells. (**A**, **B**) The Cancer Genome Atlas (TCGA) and Curtis breast datasets were analyzed for the co-expression of *UBE2T* mRNA with the indicated genes. (**C**) The genes significantly co-expressed with *UBE2T* in both the TCGA and Curtis breast datasets are listed. (**D**) MCF7 cells expressing either non-specific (NS) small hairpin RNA (shRNA) or *TFAP2A* shRNAs were analyzed by quantitative reverse-transcriptase polymerase chain reaction (qPCR) to assess the expression of *TFAP2A* and *UBE2T* mRNA. (**E**) MCF7 cells expressing NS or *TFAP21* shRNAs were analyzed for the expression of TFAP2A and UBE2T protein by immunoblotting. ACTINB was used as the loading control. (**F**) MCF7 cells expressing *UBE2T* or NS shRNA were analyzed using soft-agar assays. Representative images are shown. Scale bar, 500 μm. (**G**) Colony sizes for MCF7 cells expressing *TFAP2A* or NS shRNA from the experiment shown in panel F. (**H**) CUT&RUN assay was performed to analyze the recruitment of TFAP2A to *UBE2T* or *ACTINB* promoters. The relative enrichment of TFAP2A on *UBE2T* or *ACTINB* promoters is shown. (**I**) TCGA patient-derived breast cancer datasets were analyzed for *TFAP2A* mRNA expression in normal breast tissues compared with primary breast tumors via the UALCAN bioinformatics tool. Data represent the mean ± SEM. *P< 0.05, **P< 0.01, ***P< 0.001.

We next attempted to determine the mechanism driving UBE2T overexpression in breast cancer cells to promote cell growth. Because UBE2T was overexpressed at the transcriptional level, we performed experiments to identify transcriptional regulators of UBE2T. We first analyzed the DNA sequence of the *UBE2T* promoter (∼2 kb) to identify transcription factor consensus DNA-binding sites using the PROMO search tool for putative transcription factor identification (26,40). This analysis identified 25 transcription factors that displayed perfect matches for the consensus DNA-binding sequence (Supplementary Figure S1). To prioritize transcription factors for further analysis, we examined whether any of these potential transcription factors were also overexpressed in patient-derived breast cancer samples using publicly available breast cancer datasets. We identified eight transcription factors that were overexpressed in both the TCGA breast cancer dataset and the Curtis breast cancer dataset (Figure 3A, B). However, only four of these transcription factors were significantly co-overexpressed with UBE2T in patient-derived breast cancer samples (Figure 3C). Based on these analyses, we used shRNAs targeting all four shortlisted transcription factors and tested whether their knockdown had any effects on UBE2T expression. We used breast cancer cells expressing NS shRNA as controls. Our results showed that TFAP2A knockdown reduced UBE2T expression at the mRNA and protein levels in breast cancer cells (Figure 3D, E). However, the knockdown of the other three transcription factors (ELK1, GTF2I and FOXP3) did not reduce UBE2T expression levels in breast cancer cells (Supplementary Figure S2). TFAP2A knockdown also inhibited the abilities of breast cancer cells to grow in an anchorage-independent manner in soft-agar assays, mimicking the growth inhibitory phenotype observed with UBE2T inhibition in breast cancer cells (Figure 3F, G). To determine whether UBE2T is a direct target of TFAP2A, we performed CUT&RUN assays and observed the significant enrichment of TFAP2A on the UBE2T promoter (Figure 3H). We also found that *TFAP2A* mRNA was significantly overexpressed in breast cancer tumor tissue, similar to the overexpression of *UBE2T* mRNA (Figure 3I). Collectively, these results demonstrated that the transcription factor TFAP2A is overexpressed in breast cancer cells and is necessary for the transcriptional overexpression of UBE2T.

### UBE2T loss results in the activation of a tumor-suppressive signaling pathway leading to breast cancer growth inhibition

**Figure 4.**
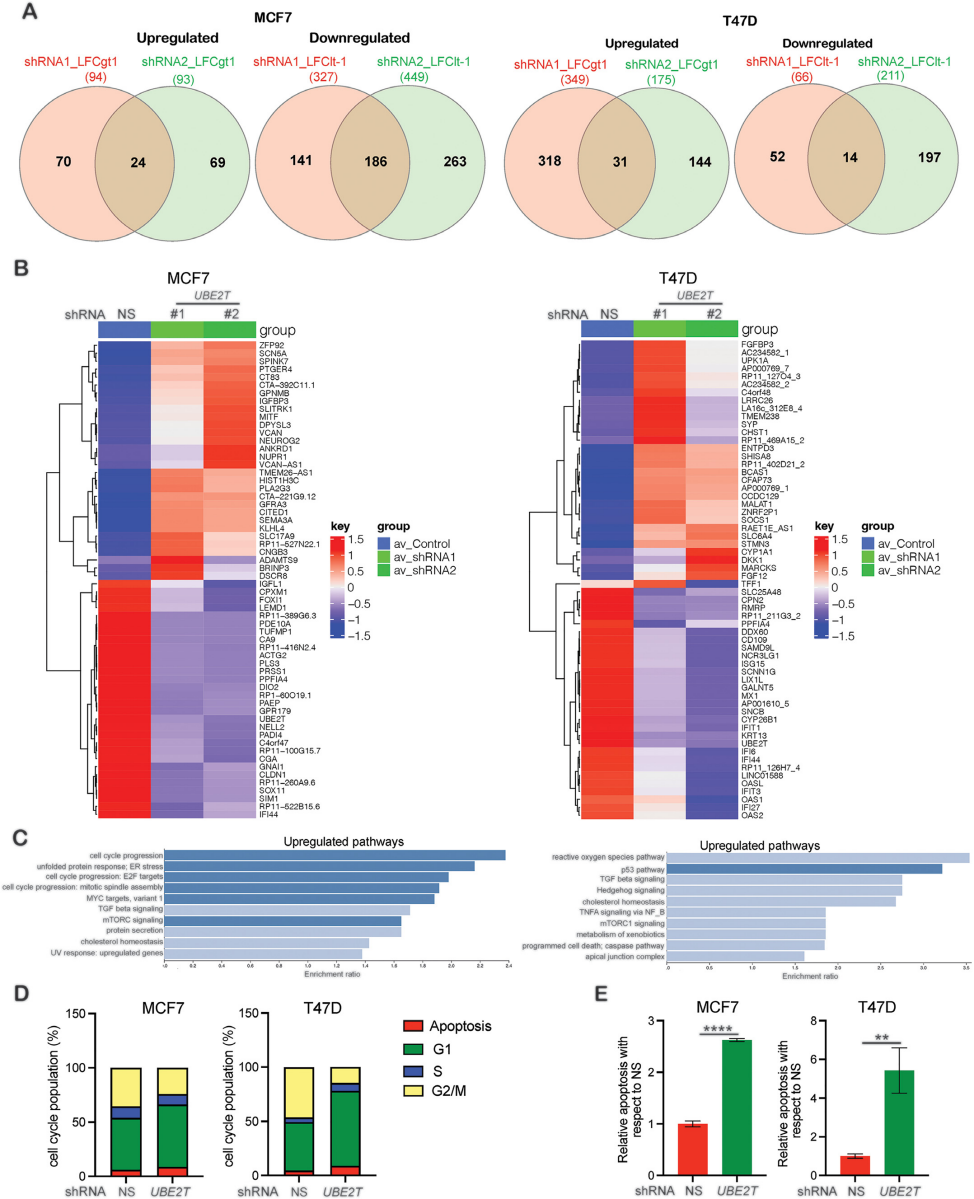
Loss of UBE2T results in cell cycle inhibition and apoptosis induction. (**A**) Venn diagram showing overlap among the most differentially expressed genes (upregulated and downregulated) in MCF7 and T47D cells expressing *UBE2T* small hairpin RNA (shRNA) compared with non-specific (NS) shRNA. Cutoff values of fold change >2 were used. Reads from three biological replicates in each group were used for analysis (Supplementary Tables S2 and S3). Additional cutoff values of absolute log_2_ FC >1. (**B**) Heatmaps showing the top upregulated and downregulated genes in MCF7 and T47D cells expressing NS or *UBE2T* shRNA based on gene expression Z-scores for each gene. For MCF7, the average log_2_ FC was calculated for the two contrasts (Supplementary Table S2) using the average log_2_ FC to rank these genes. Thirty of the most upregulated genes and thirsty of the most downregulated genes were identified. The normalized gene expression data from the sixty most regulated genes were used as input to generate the heatmaps. A heatmap for T47D (Supplementary Table S3) was generated using similar methods as described for MCF7. (**C**) The top 10 biological pathways enriched among gene signatures from MCF7 (Supplementary Table S2) and T47D (Supplementary Table S3) cells expressing *UBE2T* shRNA compared with NS shRNA. (**D**) Flow cytometry analysis of MCF7and T47D cells expressing NS or *UBE2T* shRNA. The percentages of cells in each phase of the cell cycle are shown. (**E**) Apoptosis was measured in MCF7 and T47D cells expressing NS or *UBE2T* shRNA. Apoptosis rates in cells expressing *UBE2T* shRNA were plotted relative to those in cells expressing NS shRNA.

To more comprehensively determine the mechanisms through which UBE2T contributes to breast cancer growth and identify new mediators of UBE2T function, we performed transcriptome-wide mRNA expression profiling of breast cancer cell lines (MCF7 and T47D) expressing either *UBE2T* or NS shRNA. We identified 186 commonly downregulated genes (≥−1.0-fold) and 24 commonly upregulated genes (≥1.0-fold) in MCF7 cells expressing either *UBE2T* shRNA relative to NS shRNA expressing cells (Supplementary Table S2). We also identified 14 commonly downregulated genes (≥−1.0-fold) and 31 commonly upregulated genes (≥1.0-fold) in T47D cells expressing either *UBE2T* shRNA relative to NS shRNA expressing cells (Supplementary Table S3 and Figure 4A, B). We analyzed these significantly altered genes in MCF7 and T47D cells for biological pathway enrichment and found that *UBE2T* knockdown resulted in the significant upregulation of pathways associated with cell cycle progression, including the p53 pathway (Figure 4C). Based on these results, we performed a cell cycle analysis of MCF7 and T47D cells expressing *UBE2T* shRNA. Compared with NS shRNA expressing control cells, *UBE2T* shRNA expressing cells showed an increased proportion of cells in the G1 phase and a decreased proportion of cells in the G2/M phase (Figure 4D and Supplementary Figure S3), which led to increased apoptosis (Figure 4E and Supplementary Figure S4). We also observed several significantly downregulated oncogenic pathways in *UBE2T*-knockdown cells compared with control cells, including the mTOR complex 1 (mTORC1) and tumor necrosis factor-alpha (TNFA) signaling pathways (Supplementary Figure S5A). Collectively, these results show that *UBE2T* knockdown leads to cell cycle arrest and apoptosis induction, which inhibit breast cancer cell growth.

### UBE2T modulates interferon-stimulated *IFI6* expression to regulate DNA replication stress and promote breast cancer growth

**Figure 5.**
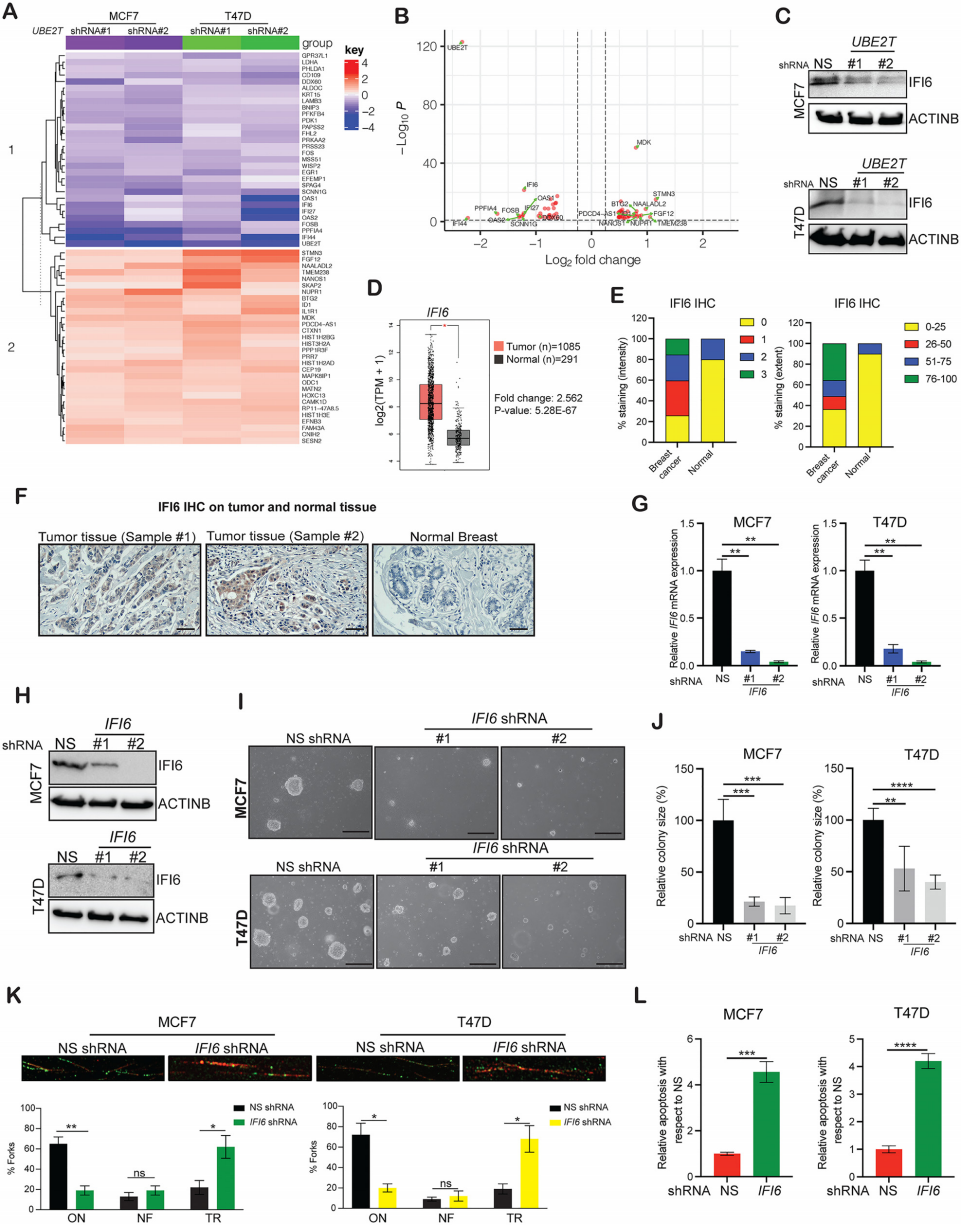
UBE2T regulates DNA replication stress in breast cancer cells. (**A**) Heat maps showing commonly altered genes (Supplementary Table S4) in both MCF7 and T47D cells expressing *UBE2T* small hairpin RNA (shRNA) compared with non-specific (NS) shRNA expressing cells. The respective average log_2_ fold changes from the sixty most highly regulated genes were used as input to generate heatmaps; the input data was not scaled. (**B**) Volcano plot showing upregulated and downregulated genes in *UBE2T* shRNA expressing cells compared with NS shRNA expressing cells (Supplementary Table S4). (**C**) UBE2T and IFI6 protein expression were assessed by immunoblotting in MCF7 and T47D cells expressing NS or *UBE2T* shRNA, using ACTINB as the loading control. (**D**) *IFI6* mRNA expression in normal breast tissues compared with breast tumor samples was analyzed using Gene Expression Profiling Interactive Analysis (GEPIA). (**E, F**) Analysis of immunohistochemical data from a tumor microarray (TMA) featuring breast cancer samples and matched adjacent normal breast tissues. The average intensity and extent of IFI6 staining are plotted (**E**). Representative images of immunohistochemical IFI6 staining are shown at 20 × magnification (**F**). Scale bar, 50 μm. (**G**) MCF7 and T47D cells expressing either NS or *IFI6* shRNAs were analyzed by qPCR. *IFI6* mRNA expression is plotted relative to expression levels in NS shRNA expressing cells. (**H**) IFI6 protein expression was assessed by immunoblotting in MCF7 and T47D cells expressing *IFI6* or NS shRNA, using ACTINB as the loading control. (**I**, **J**) Representative images (I) and colony size (J) of MCF7 and T47D cells expressing *IFI6* or NS shRNA were analyzed using soft-agar assays. Scale bar, 500 μm. (**K**) Representative images of DNA fiber assays assessing MCF7 and T47D cell lines expressing *IFI6* or NS shRNA (scale bar, 5 μm). Bar diagram showing the percentage of DNA forks (ON, ongoing; NF, newly fired; TR, truncated/stalled). (**L**) Apoptosis was measured in MCF7 and T47D cells expressing NS or *IFI6* shRNA. The apoptosis rate in *IFI6* shRNA cells is plotted relative to that in NS shRNA expressing cells. Data represent the mean ± SEM. **P< 0.01, ***P< 0.001.

We identified 118 commonly downregulated genes and 144 commonly upregulated genes in *UBE2T* shRNA expressing cells as compared to control cells expressing NS shRNA (Supplementary Table S4) in both MCF7 and T47D cells. The downregulated genes included several interferon-stimulated genes (Figure 5A), among which *IFI6* was the most significantly repressed gene identified in *UBE2T* shRNA expressing cells (Figure 5B, C). We also found that IFI6 was one of the most significantly expressed genes next to UBE2T in breast cancer cell lines (Supplementary Figure S5B). Based on these results, we next investigated the role of UBE2T-regulated IFI6 expression in breast cancer cell growth. To explore whether IFI6 plays a role in breast cancer cell growth downstream of UBE2T, we first examined whether IFI6 is overexpressed in patient-derived breast cancer samples compared with normal breast tissues, similar to UBE2T overexpression. We analyzed publicly available breast cancer datasets (25) and found that, similar to *UBE2T* mRNA, *IFI6* mRNA was significantly overexpressed in breast cancer samples relative to normal breast samples (Figure 5D). Similarly, IFI6 protein expression assessed by IHC using a breast cancer TMA (#BC081120f) demonstrated the significant elevation of IFI6 expression in a large majority of patient-derived breast cancer samples compared with normal breast tissue samples (Figure 5E, F). To study the role of IFI6, we knocked down *IFI6* expression using two shRNAs with independent sequences in MCF7 and T47D cells (Figure 5G–H). After *IFI6* knockdown was validated, these breast cancer cell lines were tested for their abilities to grow in an anchorage-independent manner using soft-agar assays. Breast cancer cells expressing NS shRNA were used as a negative control. We found that *IFI6* knockdown in breast cancer cells resulted in significant reductions in the abilities of these cells to form colonies in soft agar (Figure 5I–J), mimicking the growth inhibitory phenotype observed in *UBE2T*-knockdown cells.

**Figure 6.**
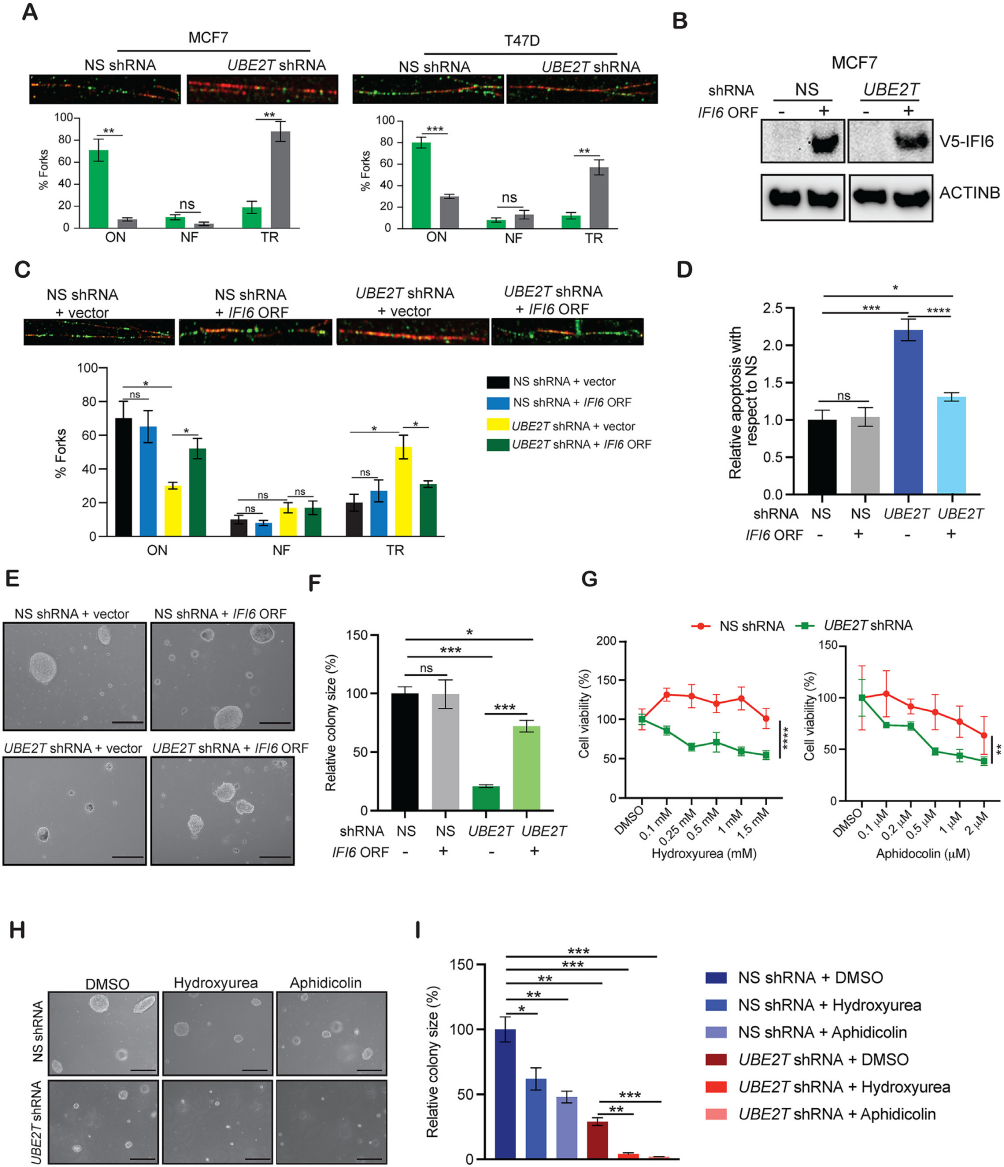
Overexpression of IFI6 rescues UBE2T loss–mediated tumor inhibition. (**A**) MCF7 cells expressing non-specific (NS) or *UBE2T* small hairpin RNA (shRNA) were subjected to DNA fiber assays; representative images of DNA fibers are shown (scale bar, 5 μm). Bar diagram showing the percentage of DNA forks (ON, ongoing; NF, newly fired; TR, truncated/stalled). (**B**) MCF7 cells expressing NS or *UBE2T* shRNA alone or with IFI6 overexpression were analyzed for V5-IFI6 protein expression by immunoblotting. ACTINB was used as a loading control. (**C**) MCF7 cells expressing NS or *UBE2T* shRNA alone or with IFI6 overexpression were subjected to DNA fiber assays; representative images of DNA fibers are shown (scale bar, 5 μm). Bar diagram showing the percentage of DNA forks (ON, ongoing; NF, newly fired; TR, truncated/stalled). (**D**) Apoptosis was measured in MCF7 cells expressing NS or *UBE2T* shRNA alone or with IFI6 overexpression. Apoptosis rates in *IFI6*-overexpressing cells are plotted relative to those in vector-expressing cells. (**E**) MCF7 cells expressing NS or *UBE2T* shRNA alone or with IFI6 overexpression were analyzed using soft-agar assays. Representative images are shown. Scale bar, 500 μm. (**F**) Colony sizes for the experiment shown in panel E. (**G**) Relative cell survival (%) was measured by MTT assay after MCF7 cells expressing NS or *UBE2T* shRNA were treated with the indicated concentrations of hydroxyurea or aphidicolin for 3 days. (**H**) MCF7 cells expressing NS or *UBE2T* shRNA were treated with 1.5 mM hydroxyurea and 0.5 μM aphidicolin and analyzed using soft-agar assays. Representative images are shown. Scale bar, 500 μm. (**I**) Colony sizes for the experiment shown in panel H.

Our previous study in melanoma cells showed that IFI6 regulates DNA replication stress pathways to promote melanoma growth (41). Based on these findings, we examined whether *IFI6* knockdown resulted in DNA replication stress in breast cancer cells, leading to growth inhibition. We found that the loss of *IFI6* expression resulted in dysregulated DNA replication, as indicated by the detection of significantly fewer ongoing DNA replication forks and more stalled forks in *IFI6* shRNA expressing breast cancer cells than in NS shRNA expressing cells (Figure 5K and Supplementary Figure S6). In addition, increased DNA replication stress in *IFI6* knockdown cells compared with control cells was associated with increased apoptosis (Figure 5L and Supplementary Figure S7). Guided by these results, we also examined DNA replication stress in *UBE2T* shRNA expressing breast cancer cells and found that inhibition of *UBE2T* expression also led to the activation of DNA replication stress (Figure 6A and Supplementary Figure S8A).

Based on these results, we performed experiments to determine whether IFI6 acts as a downstream mediator of UBE2T-induced tumor growth in breast cancer. We ectopically expressed IFI6 in breast cancer cells expressing *UBE2T* shRNA (Figure 6B) and tested whether IFI6 overexpression restored normal DNA replication by preventing DNA replication stress and apoptosis. We found that the ectopic expression of IFI6 in *UBE2T*-knockdown cells partially rescued *UBE2T* knockdown–induced DNA replication stress and apoptosis (Figure 6C, D and Supplementary Figure S8B). Consistent with this finding, the ectopic expression of IFI6 in *UBE2T* shRNA expressing breast cancer cells also rescued their abilities to grow in soft-agar assays (Figure 6E, F). These results demonstrate that UBE2T activates IFI6 expression to suppress DNA replication stress and prevent apoptosis induction, facilitating breast cancer cell growth. However, we would also like to note that it is also possible that IFI6 may also influence cell growth and other tumor phenotypes by regulating alternative UBE2T-independent pathways to promote breast cancer cell growth.

### UBE2T inhibition enhances the sensitivity of breast cancer cells to DNA replication stress–inducing drugs

Finally, we asked whether our results were clinically significant. We first tested the effects of DNA replication stress inducers on the growth of MCF7 and T47D cells expressing *UBE2T* shRNA using cell-based assays. We treated NS shRNA expressing and *UBE2T* shRNA expressing breast cancer cells with the DNA replication stress–inducing agents’ hydroxyurea and aphidicolin (42–44) and measured cell viability using the MTT assay. We found that hydroxyurea and aphidicolin induced more potent inhibitory effects on the growth of breast cancer cells expressing *UBE2T* shRNA than on the growth of cells expressing NS shRNA, and these effects increased in a concentration-dependent manner (Figure 6G). Similar results were obtained in the soft-agar assay (Figure 6H, I). These results demonstrate that *UBE2T* suppression might enhance the therapeutic benefits of drugs that function by inducing DNA replication stress, such as hydroxyurea and aphidicolin.

## DISCUSSION

UBE2T belongs to the family of E2 ubiquitin-conjugating enzymes and has previously been implicated in DNA repair and carcinogenesis (18,45–47). Previous studies have reported that UBE2T is overexpressed in several cancer types, such as melanoma, ovarian cancer, renal cancer and hepatocellular carcinoma, and its overexpression correlates with cancer progression and poor prognosis (16,37,38). UBE2T expression has also been considered to serve as an early biomarker for cancer prognosis (36).

**Figure 7.**
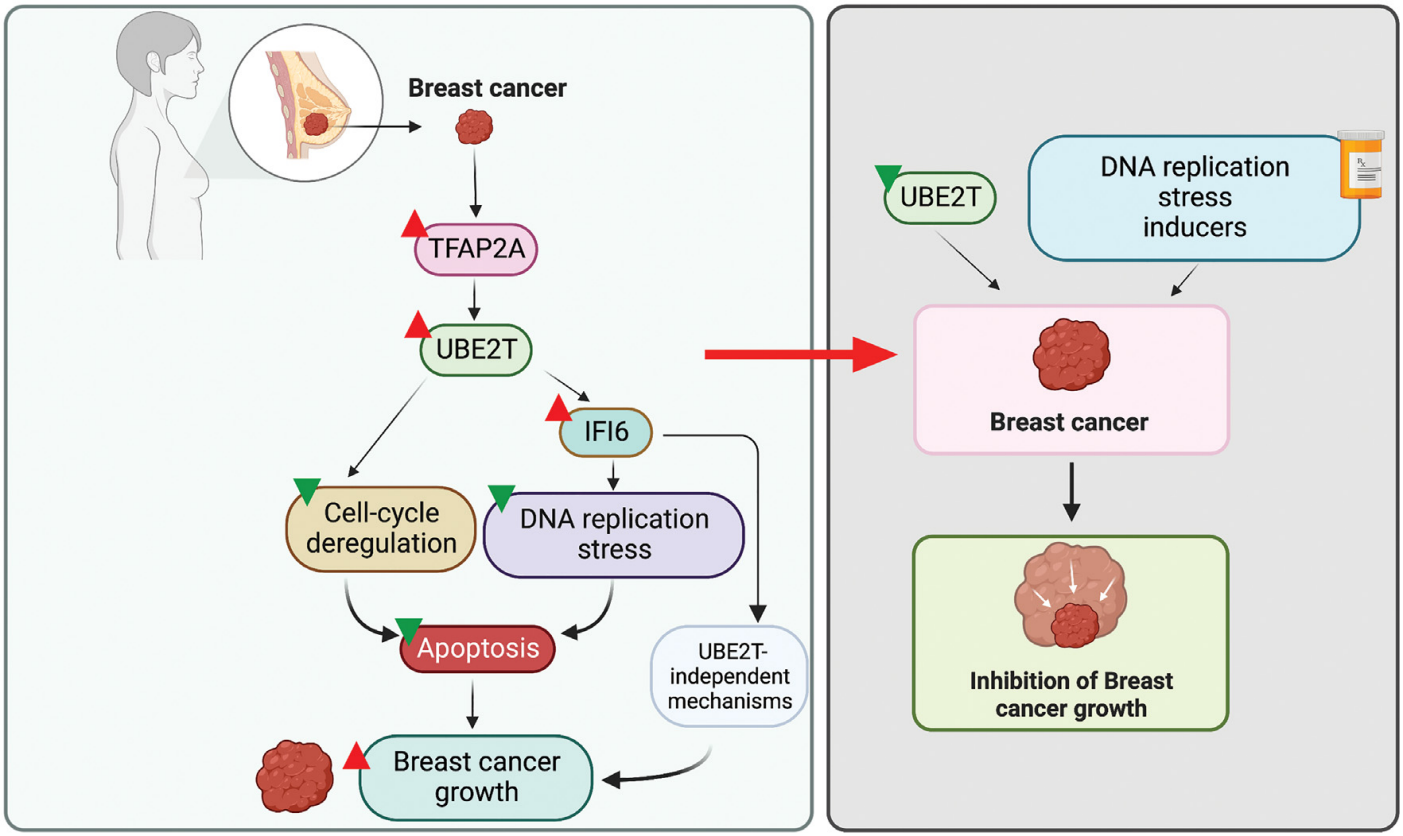
Model showing the mechanism of action for UBE2T in breast cancer progression. Model depicting the proposed mechanism through which UBE2T plays a role in breast cancer tumor progression by regulating DNA replication stress and apoptosis.

In this study (Figure 7), we found that UBE2T is overexpressed at both the mRNA and protein levels in patient-derived breast cancer samples, and UBE2T overexpression predicted poor survival among patients with breast cancer. The transcription factor TFAP2A regulates UBE2T expression in breast cancer cells, and previous study has shown that TFAP2A regulates the expression of several genes that promote cancer growth and contribute to determining anticancer therapy resistance and sensitivity (48).

Deregulation of cell cycle and apoptotic pathways has been associated with aberrant cell proliferation and cancer development (49,50). In our study, we found that UBE2T inhibition leads to upregulated cell cycle progression as observed in MCF7 cell expressing UBE2T shRNA and further G1 cell cycle arrest and apoptosis induction. During the G1 phase of the cell cycle, major regulatory events occur, including cell growth, increasing protein contents, and organelle doubling, which serve as signals for the cell to enter the cell division stage (51). Therefore, cell cycle arrest in the G1 phase, such as that observed in *UBE2T* shRNA expressing breast cancer cells, prevents progression to the S and G2 phases and inhibits cell division. Moreover, prolonged cell cycle arrest leads to cell death, further inhibiting cancer cell proliferation (52,53). Upregulated cell cycle progression observed in MCF7 cell expressing UBE2T shRNA was associated with genes such as transcription factor E2F targets CKS1B, KPNA2, LDLR, minichromosome maintenance proteins (MCM2, MCM7) etc. that leads to DNA hyper-replication induced cycle arrest stress and apoptosis induction (41,54). UBE2T is also involved in activating oncogenic signaling pathways, such as the mTOR and TNFA signaling pathways, which are known to modulate cancer growth and progression (55–57). Additionally, we found that UBE2T inhibits DNA replication stress pathways and is essential for the Fanconi anemia pathway, which is involved in the maintenance of genome stability through DNA damage repair, replication fork stabilization, and the alleviation of oxidative and mitotic stress (58). We identified that UBE2T regulates DNA replication stress in breast cancer cells by stimulating the expression of IFI6, suppressing DNA replication stress, cell cycle arrest, and apoptosis induction. *IFI6* is an interferon-stimulated gene that belongs to the FAM14 gene family (59). IFI6 is overexpressed in many cancer types and is involved in stabilizing mitochondrial function, resulting in apoptosis inhibition and tumor growth promotion (60). Our previous study also showed that IFI6 is a target of NRAS and promotes tumor growth by suppressing DNA replication stress in melanoma cells (41). The DNA replication stress pathway also regulates the cell cycle transition (61,62). The inhibition of the DNA replication stress pathway prevents the progression from G1 to S phase, resulting in the accumulation of cells in the G1 phase (63). In this study, we showed that the inhibition of *UBE2T* expression correlated with the repression of *IFI6* expression, the activation of the DNA replication stress pathway, G1 cell cycle arrest, and apoptosis induction, leading to the inhibition of breast cancer cell growth and proliferation. We further showed that IFI6 overexpression effectively reverses DNA replication stress and the inhibition of colony-forming growth induced by UBE2T knockdown in breast cancer cells, partially restoring breast cancer cell proliferation.

The DNA replication stress pathway can be pharmacologically activated by a group of small-molecule DNA replication stress inducers, such as hydroxyurea and aphidicolin (43), which have been used for cancer treatment (64,65). We show that *UBE2T* knockdown increases the sensitivity of breast cancer cells to DNA replication stress inducers. Thus, the UBE2T→IFI6→DNA replication stress→apoptosis pathway represents a potential new therapeutic target for breast cancer cases associated with high UBE2T expression levels. A previous study reported the identification of UBE2T inhibitors, including M435-1279 (66), which may potently inhibit breast cancer growth when combined with DNA replication stress inducers. Collectively, these studies identify UBE2T as a facilitator of breast cancer tumor growth through the suppression of IFI6 expression, DNA replication stress, and apoptosis induction. These studies indicate that UBE2T may serve as a potential therapeutic target for breast cancer, either alone or in combination with other anticancer agents, such as DNA replication stress inducers.

### Limitations of the study

Although, in our study we find that UBE2T mediates its function through IFI6 in UBE2T-IFI6-DNA replication-apoptosis pathway dependent manner in breast cancer cells, it is possible that IFI6 could influence cell growth and other phenotypes by alternative UBE2T-independent mechanisms. Another observation in our study was that UBE2T regulates mRNA expression level of IFI6. However, it is possible that UBE2T might affect ubiquitination of IFI6 and other proteins including transcription factors that may be involved in regulating IFI6 transcription. Future studies are required to address both these aspects more comprehensively.

## MATERIALS AND CORRESPONDENCE

All correspondence and materials should be requested from Dr Romi Gupta.

## DATA AVAILABILITY

These data were submitted to and are available from the Gene Expression Omnibus (Accession# GSE156603 for *UBE2T*-knockdown RNA-seq). Additional data related to any experiments presented in this article are available from the corresponding author upon request.

## Supplementary Material

zcac035_Supplemental_Files
