# Supplementary material for: UBE2T promotes breast cancer tumor growth by suppressing DNA replication stress
Source: NAR Cancer. 2022 Nov 2;4(4):zcac035. doi: 10.1093/narcan/zcac035 (PMC9629447; doi:10.1093/narcan/zcac035)
Supplement: zcac035_Supplemental_Files [file zcac035_supplemental_files.zip › Supplementary Figures.pdf]

Supplementary Data

Supplementary Figures and Figure Legends

NAME	MATRIX_WIDTH
C/EBPbeta [T00581]	4
Elk-1 [T00250]	9
TFII-I [T00824]	6
GR-alpha [T00337]	5
YY1 [T00915]	4
GR-beta [T01920]	5
FOXP3 [T04280]	6
STAT4 [T01577]	6
TFIID [T00820]	7
HNF-3alpha [T02512]	8
GR [T05076]	7
RXR-alpha [T01345]	7
IRF-2 [T01491]	6
ER-alpha [T00261]	5
PXR-1:RXR-alpha [T05671]	8
XBP-1 [T00902]	6
GCF [T00320]	9
PR B [T00696]	7
PR A [T01661]	7
AP-2alphaA [T00035]	6
NF-1 [T00539]	8
SRY [T00997]	9
c-Ets-1 [T00112]	7
Pax-5 [T00070]	7
p53 [T00671]	7

Supplementary Figure S1. Analysis of the *UBE2T* promoter region for transcription factor–binding sites. Analysis of the *UBE2T* promoter DNA sequence (~2 kb) for transcription factor consensus DNA-binding sites using the PROMO search tool for putative transcription factor identification with 0% dissimilarity.

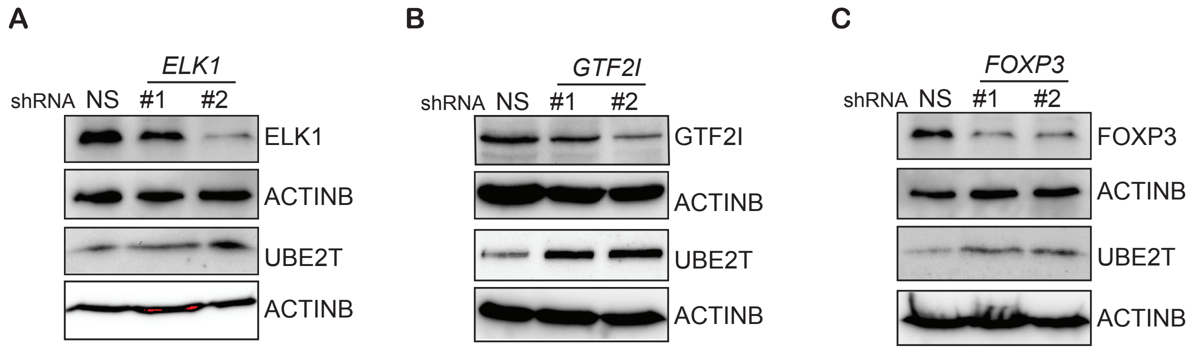

Supplementary Figure S2. Analysis of the UBE2T expression in breast cancer cells expressing *ELK1*, *GTF2I* and *FOXP3* shRNA. A. MCF7 cell line expressing NS or *ELK1* shRNA was analyzed for the expression of ELK1 and UBE2T protein by immunoblotting. ACTINB was used as the loading control. **B.** MCF7 cell line expressing NS or *GTF2I* shRNA was analyzed for the expression of GTF2I and UBE2T protein by immunoblotting. ACTINB was used as the loading control. **C.** MCF7 cell line expressing NS or *FOXP3* shRNA was analyzed for the expression of FOXP3 and UBE2T protein by immunoblotting. ACTINB was used as the loading control.

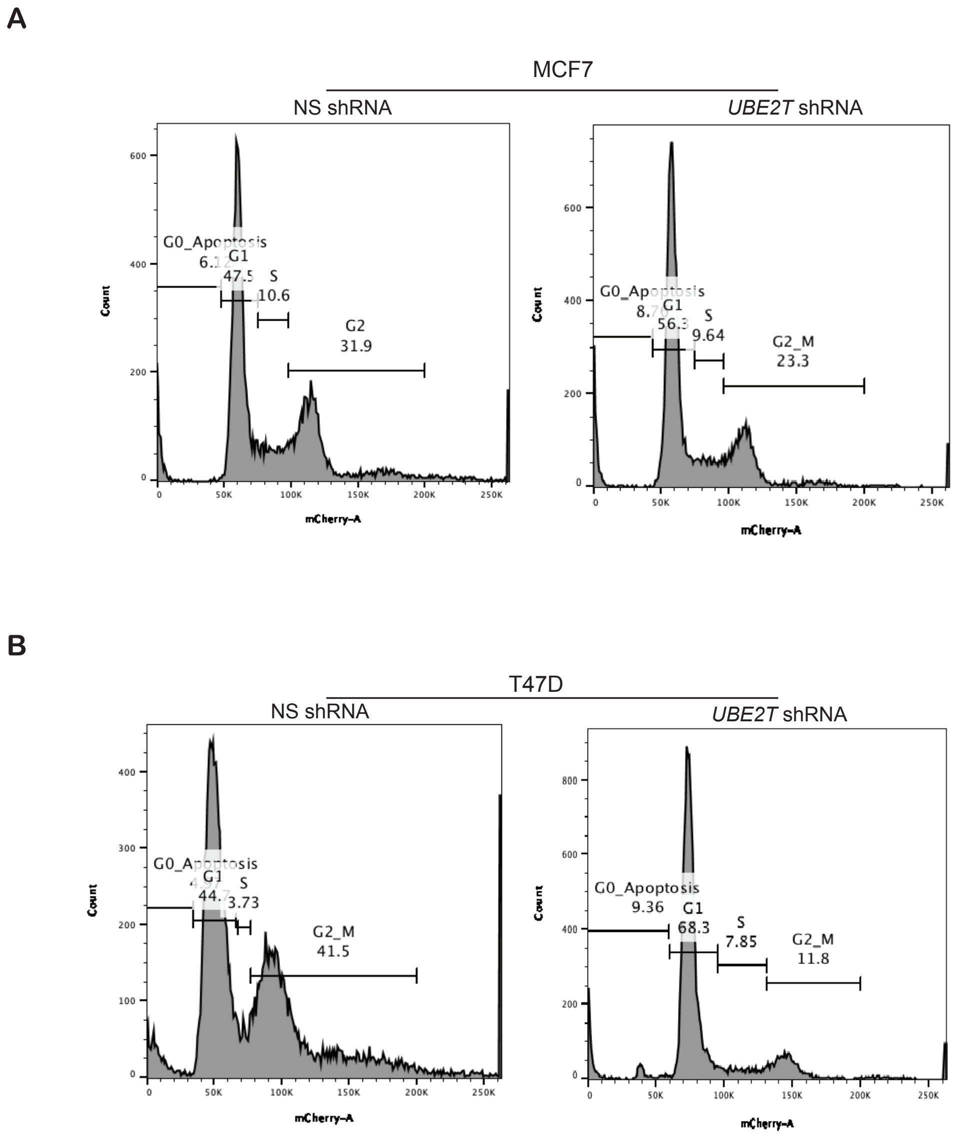

Supplementary Figure S3. Loss of UBE2T in breast cancer cell lines results in cell cycle defect. Raw images for flow cytometry analysis of MCF7 (**A**) and T47D (**B**) cells expressing NS or *UBE2T* shRNA. The percentages of cells in each cell cycle phase are shown. Data plotted for percentages of cells in each cell cycle phase is shown in Figure 4D.

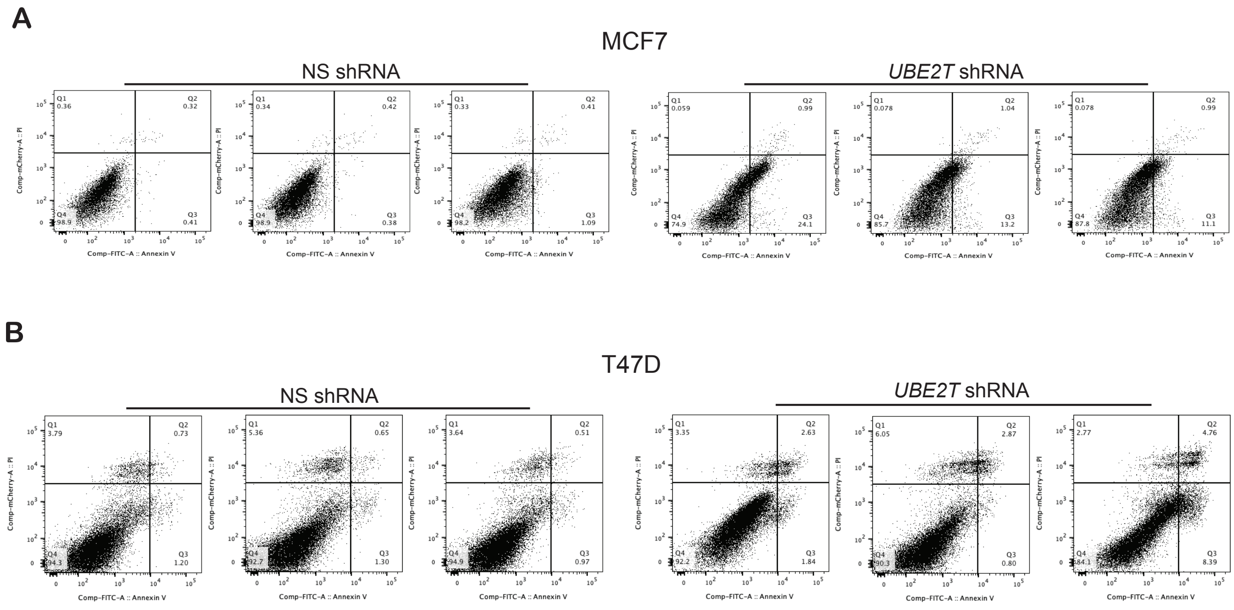

Supplementary Figure S4. Loss of UBE2T in breast cancer cell lines results in increased apoptosis. Apoptosis was measured in MCF7 (**A**) and T47D (**B**) cells expressing NS or *UBE2T* shRNA. Original images are shown here and relative apoptosis (Q2 population) in cells expressing *UBE2T* shRNA is plotted relative to cells expressing NS shRNA and is shown in Figure 4E.

A

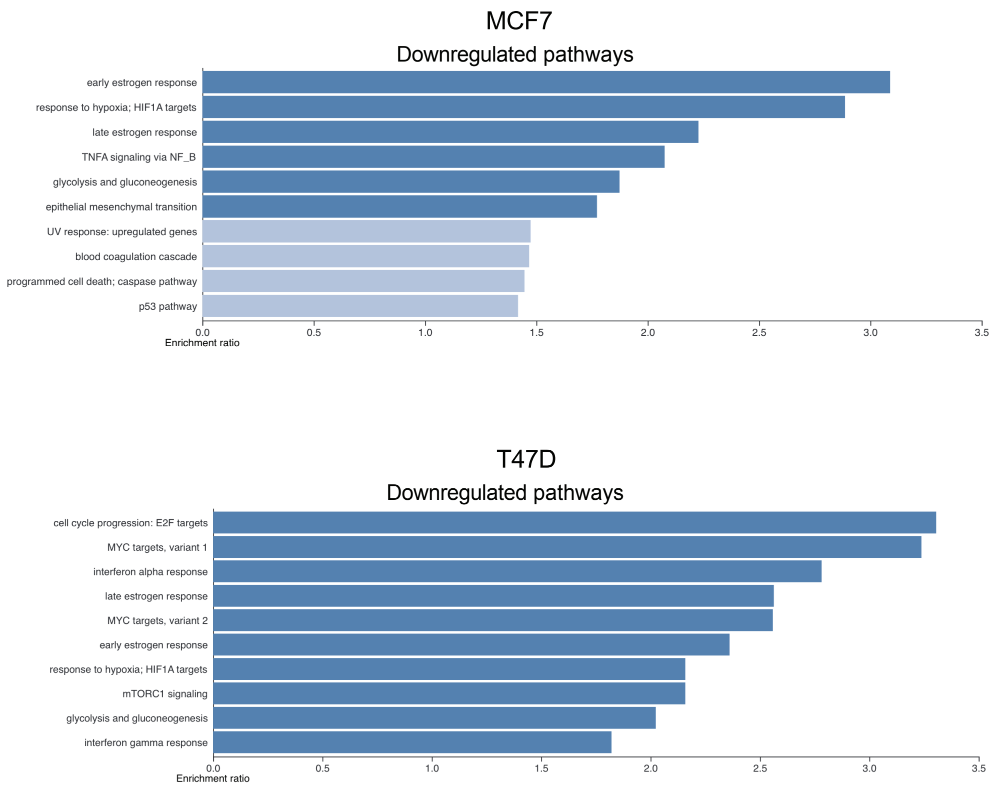

B

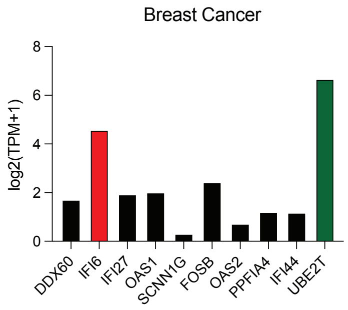

Supplementary Figure S5. Pathways downregulated in *UBE2T* knockdown cells. A. The top 10 downregulated biological pathways based on gene signatures from MCF7 (Supplementary Table S2) and T47D (Supplementary Table S3) cells expressing *UBE2T* small hairpin RNA (shRNA) compared with cells expressing non-specific (NS) shRNA is shown. **B.** *UBE2T*, *IFI6*, and other indicated gene transcript expression levels (expression 22Q1 Public) in breast cancer patient samples using deepmap portal is presented.

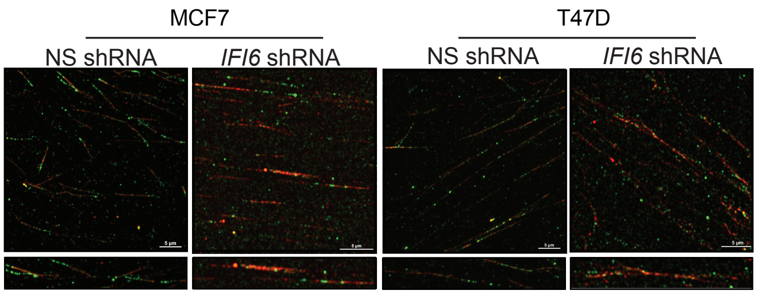

Supplementary Figure S6. DNA fiber assay performed in *UBE2T* knockdown cells. MCF7 and T47D cells expressing non-specific (NS) or *IFI6* small hairpin RNA (shRNA) were subjected to DNA fiber assays; representative images of DNA fibers are shown (scale bar, 5 μm). Original images are shown here and relative % forks (ON, NF and TR) plotted for these samples are shown in Figure 5K.

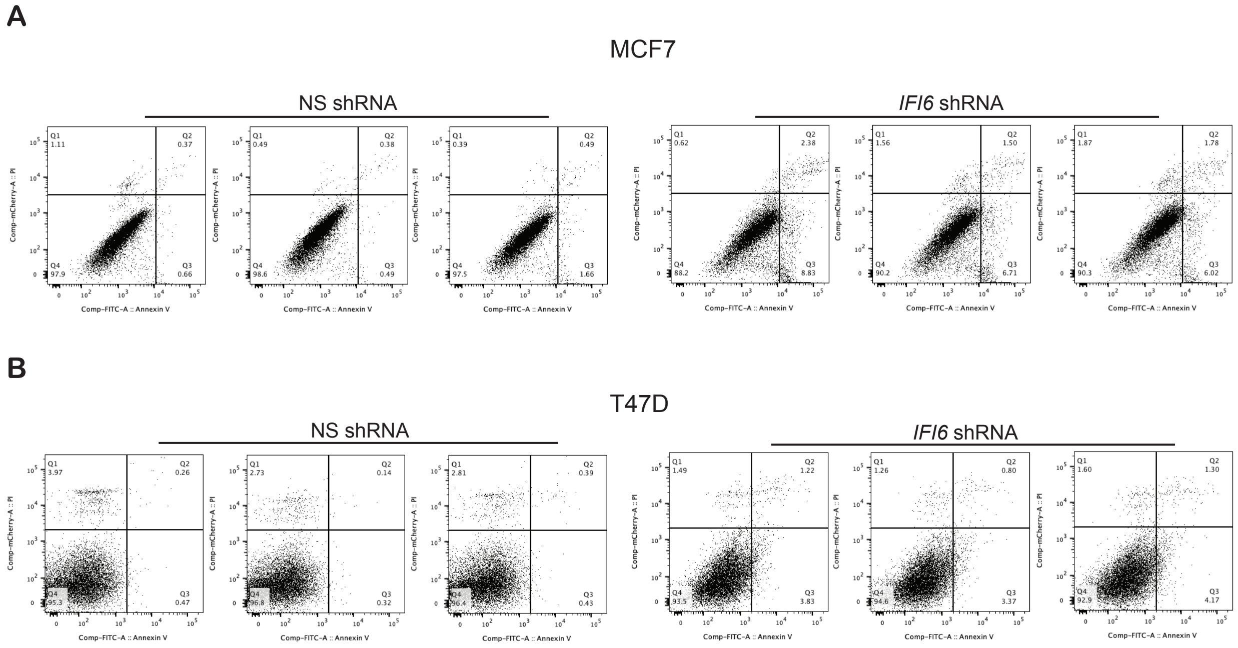

Supplementary Figure S7. Loss of IFI6 in breast cancer cell lines results in increased apoptosis. Apoptosis was measured in MCF7 (A) and T47D (B) cells expressing NS or *IFI6* shRNA. Original images are shown here and relative apoptosis (Q2 population) in cells expressing *IFI6* shRNA is plotted relative to cells expressing NS shRNA and is shown in Figure 5L.

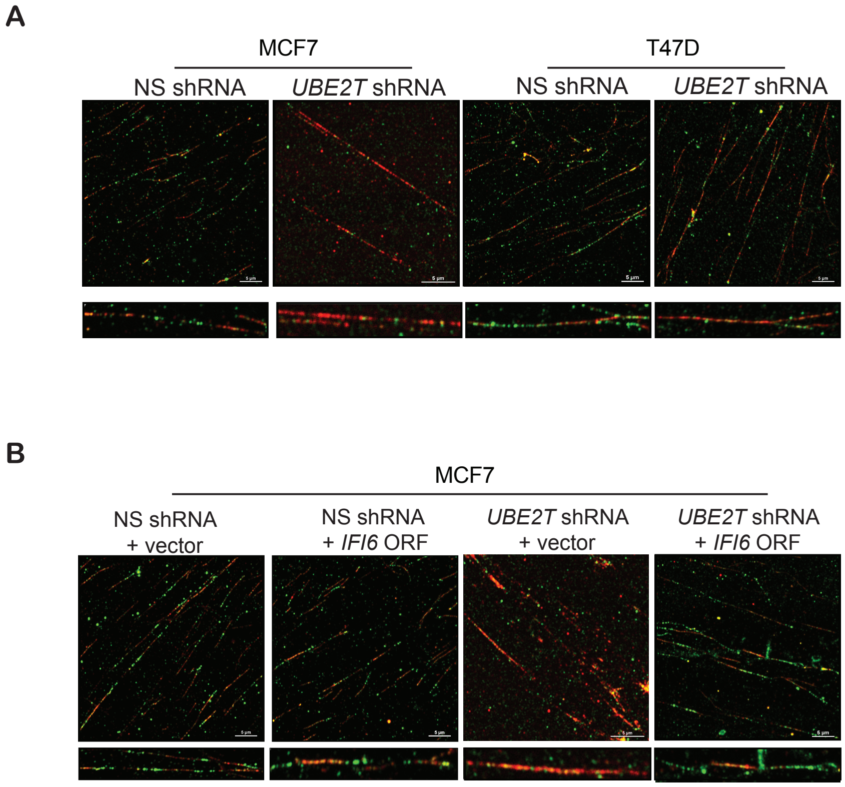

Supplementary Figure S8. DNA fiber assay performed in *UBE2T* knockdown cells. A. MCF7 and T47D cells expressing non-specific (NS) or *UBE2T* small hairpin RNA (shRNA) were subjected to DNA fiber assays; representative images of DNA fibers are shown (scale bar, 5 µm). Original images are shown here and relative % forks (ON, NF and TR) plotted for these samples are shown in Figure 6A **B.** MCF7 cells expressing NS or *UBE2T* shRNA alone or with IFI6 overexpression were subjected to DNA fiber assays; representative images of DNA fibers are shown (scale bar, 5 µm). Original images are shown here and relative % forks (ON, NF and TR) plotted for these samples are shown in Figure 6C.
