# Supplementary material for: UBE2T promotes breast cancer tumor growth by suppressing DNA replication stress
Source: NAR Cancer. 2022 Nov 2;4(4):zcac035. doi: 10.1093/narcan/zcac035 (PMC9629447; doi:10.1093/narcan/zcac035)
Supplement: zcac035_Supplemental_Files [file zcac035_supplemental_files.zip › Supplementary Tables.pdf]

Supplementary Table Legends

Supplementary Table S1: Related to Figure 1F and G and Figure 5E and F.

Specification sheet for the BC081120f tissue array, containing 110 breast cancer samples and 110 matched adjacent normal breast tissue, including information regarding pathology grade; TNM and clinical stage; and IHC results for estrogen receptor, progesterone receptor, human epidermal growth factor 2.

Supplementary Table S2: Related to Figure 4A-C.

All significantly altered (p<0.05) genes in MCF7 cells expressing *UBE2T* shRNA compared with cells expressing non-specific shRNA. The MCF7 samples (n=9) include 3 biological replicates in each of these 3 groups (control, shRNA1, shRNA2). For MCF7, differentially expressed genes in the shRNA1 treated versus the control were identified. Differentially expressed genes for the shRNA2 treated versus the control group were also identified. The differentially expressed genes common to both contrasts that are regulated in the same direction comprise a first set of genes of interest.

Supplementary Table S3: Related to Figure 4A-C.

All significantly altered (p<0.05) genes in T47D cells expressing *UBE2T* shRNA compared with cells expressing non-specific shRNA. The T47D samples (n=6) include 2 biological replicates in each of the 3 groups (control, shRNA1, shRNA2). For T47D, differentially expressed genes in the shRNA1 treated versus the control were identified. Also, for T47D, differentially expressed genes in the shRNA2 treated versus the control were identified. The differentially expressed genes

common to both contrasts that are regulated in the same direction comprise a second set of genes of interest.

Supplementary Table S4: Related to Figure 5A-B.

All significantly altered (p<0.05) genes common to both MCF7 and T47D cells expressing *UBE2T* shRNA compared with cells expressing non-specific shRNA. A third set of genes of interest comprise those differentially expressed in MCF7 (Supplementary Table S2) and shared in common with those differentially expressed in T47D (Supplementary Table S3). The genes regulated in the opposite directions between the two comparisons were filtered out.

Supplementary Table S5: List of reagents, chemical primers, and antibodies used for this study.

Supplementary Table S1

Name	BC081120f
Description	Breast cancer with adjacent normal breast tissue array, including pathology grade, TNM and clinical stage, with IHC results ER/PR/Her2, 110 cases/110 cores, replacing BC081120e
Panel	Breast carcinoma with adjacent normal breast tissue microarray, containing 100 cases of invasive carcinoma of no special type, 10 adjacent normal breast tissue, single core per case
Price	306
Cases	110
Cores	110
Diameter	5
Rows	11
Columns	10

Position	No.	Age	Sex	Organ/Anatomic Site	Pathology diagnosis	TNM	Grade	Stage	Type	Tissue ID.	ER	PR	HER2
A1	1	40	F	Breast	Invasive carcinoma of no	T2N0M0	2	IIA	Malignant	Fmg020761	+	-	3+
A2	2	45	F	Breast	Invasive carcinoma of no	T2N0M0	2	IIA	Malignant	Fmg010622	++	+++	0
A3	3	46	F	Breast	Invasive carcinoma of no	T2N0M0	2	IIA	Malignant	Fmg040237	+++	++	0
A4	4	69	F	Breast	Invasive carcinoma of no	T2N1M0	2	IIB	Malignant	Fmg130003	*	*	*
A5	5	45	F	Breast	Invasive carcinoma of no	T1N0M0	2	IA	Malignant	Fmg100001	++	++	0
A6	6	44	F	Breast	Invasive carcinoma of no	T1N0M0	2	IA	Malignant	Fmg010733	+++	+++	0
A7	7	45	F	Breast	Invasive carcinoma of no	T2N1M0	2	IIB	Malignant	Fmg120154	++	-	3+
A8	8	53	F	Breast	Invasive carcinoma of no	T2N0M0	2	IIA	Malignant	Fmg020031	-	-	0
A9	9	46	F	Breast	Invasive carcinoma of no	T2N0M0	2	IIA	Malignant	Fmg020090	+++	+++	0
A10	10	41	F	Breast	Invasive carcinoma of no	T2N0M0	2	IIA	Malignant	Fmg020037	+	-	0
B1	11	43	F	Breast	Invasive carcinoma of no	T1N1M0	3	IB	Malignant	Fmg080098	-	-	0
B2	12	45	F	Breast	Invasive carcinoma of no	T2N1M0	2	IIB	Malignant	Fmg020179	+++	++	3+
B3	13	37	F	Breast	Invasive carcinoma of no	T2N0M0	2	IIA	Malignant	Fmg030639	+++	+++	0
B4	14	63	F	Breast	Invasive carcinoma of no	T2N0M0	1–2	IIA	Malignant	Fmg020081	+++	+++	0
B5	15	65	F	Breast	Invasive carcinoma of no	T2N0M0	1–2	IIA	Malignant	Fmg020082	+++	-	0
B6	16	43	F	Breast	Invasive carcinoma of no	T1N1M0	2	IB	Malignant	Fmg100066	+++	+	0
B7	17	44	F	Breast	Invasive carcinoma of no	T2N0M0	2	IIA	Malignant	Fmg080091	-	-	3+
B8	18	34	F	Breast	Invasive carcinoma of no	T4N0M0	2	IIIB	Malignant	Fmg020190	++	-	0
B9	19	48	F	Breast	Invasive carcinoma of no	T2N0M0	3	IIA	Malignant	Fmg040118	+++	++	1+
B10	20	62	F	Breast	Invasive carcinoma of no	T3N1M0	2	IIIA	Malignant	Fmg140037	+++	-	0
C1	21	44	F	Breast	Invasive carcinoma of no	T2N1M0	2	IIB	Malignant	Fmg020770	-	-	3+
C2	22	67	F	Breast	Invasive carcinoma of no	T2N0M0	2	IIA	Malignant	Fmg010624	+++	++	0
C3	23	55	F	Breast	Invasive carcinoma of no	T2N0M0	2	IIA	Malignant	Fmg040104	+++	+++	0
C4	24	57	F	Breast	Invasive carcinoma of no	T2N0M0	2	IIA	Malignant	Fmg040001	+++	+++	3+
C5	25	63	F	Breast	Invasive carcinoma of no	T2N0M0	2	IIA	Malignant	Fmg110044	+++	-	0
C6	26	39	F	Breast	Invasive carcinoma of no	T2N1M0	2	IIB	Malignant	Fmg130073	+++	-	3+
C7	27	42	F	Breast	Invasive carcinoma of no	T2N0M0	2	IIA	Malignant	Fmg030482	+++	+++	0
C8	28	40	F	Breast	Invasive carcinoma of no	T2N0M0	2	IIA	Malignant	Fmg030496	+++	+++	0
C9	29	42	F	Breast	Invasive carcinoma of no	T2N0M0	2	IIA	Malignant	Fmg030501	+	+	0
C10	30	32	F	Breast	Invasive carcinoma of no	T2N0M0	2	IIA	Malignant	Fmg010613	-	-	0
D1	31	52	F	Breast	Invasive carcinoma of no	T3N0M0	2	IIB	Malignant	Fmg130041	+++	+++	0
D2	32	48	F	Breast	Invasive carcinoma of no	T2N0M0	2	IIA	Malignant	Fmg030645	+++	+++	0
D3	33	54	F	Breast	Invasive carcinoma of no	T2N1M0	2	IIB	Malignant	Fmg100050	+	-	0
D4	34	47	F	Breast	Invasive carcinoma of no	T3N2M0	2	IIIA	Malignant	Fmg040661	-	-	0
D5	35	46	F	Breast	Invasive carcinoma of no	T3N0M0	2	IIB	Malignant	Fmg040074	+++	++	3+
D6	36	68	F	Breast	Invasive carcinoma of no	T2N0M0	2	IIA	Malignant	Fmg010735	+++	+++	0
D7	37	53	F	Breast	Invasive carcinoma of no	T2N0M0	2	IIA	Malignant	Fmg010310	-	-	0
D8	38	58	F	Breast	Invasive carcinoma of no	T2N1M0	2	IIB	Malignant	Fmg090029	++	+	0
D9	39	42	F	Breast	Invasive carcinoma of no	T3N0M0	2	IIB	Malignant	Fmg050032	-	-	3+
D10	40	34	F	Breast	Invasive carcinoma of no	T3N0M0	2	IIB	Malignant	Fmg040048	+++	+	3+
E1	41	47	F	Breast	Invasive carcinoma of no	T3N0M0	2	IIB	Malignant	Fmg010671	+++	+++	0
E2	42	42	F	Breast	Invasive carcinoma of no	T3N0M0	2	IIB	Malignant	Fmg040768	-	-	3+
E3	43	48	F	Breast	Invasive carcinoma of no	T4N2M0	2	IIIB	Malignant	Fmg010531	++	++	0
E4	44	32	F	Breast	Invasive carcinoma of no	T2N1M0	2	IIB	Malignant	Fmg030523	++	+	0
E5	45	52	F	Breast	Invasive carcinoma of no	T2N1M0	2	IIB	Malignant	Fmg060191	-	-	0
E6	46	37	F	Breast	Invasive carcinoma of no	T2N1M0	3	IIB	Malignant	Fmg010612	-	-	0
E7	47	74	F	Breast	Invasive carcinoma of no	T2N0M0	3	IIA	Malignant	Fmg030477	*	*	*
E8	48	46	F	Breast	Invasive carcinoma of no	T1N0M0	2	IA	Malignant	Fmg010358	+	-	0
E9	49	48	F	Breast	Invasive carcinoma of no	T2N1M0	2	IIB	Malignant	Fmg050026	++	+++	0
E10	50	51	F	Breast	Invasive carcinoma of no	T3N1M0	3	IIIA	Malignant	Fmg010604	-	-	3+
F1	51	52	F	Breast	Invasive carcinoma of no	T2N2M0	2	IIIA	Malignant	Fmg010536	+++	+	2+
F2	52	51	F	Breast	Invasive carcinoma of no	T2N2M0	2	IIIA	Malignant	Fmg010643	-	-	0
F3	53	46	F	Breast	Invasive carcinoma of no	T4N2M0	2	IIIB	Malignant	Fmg030516	+++	-	0
F4	54	49	F	Breast	Invasive carcinoma of no	T2N0M0	2	IIA	Malignant	Fmg060181	+++	+++	0
F5	55	46	F	Breast	Invasive carcinoma of no	T4N0M0	2	IIIB	Malignant	Fmg040082	+++	+++	0
F6	56	69	F	Breast	Invasive carcinoma of no	T3N0M0	2	IIB	Malignant	Fmg020184	+++	-	0
F7	57	45	F	Breast	Invasive carcinoma of no	T4N1M0	2	IIIB	Malignant	Fmg020562	++	+	0
F8	58	50	F	Breast	Invasive carcinoma of no	T4N1M0	3	IIIB	Malignant	Fmg040675	-	-	0
F9	59	49	F	Breast	Invasive carcinoma of no	T2N0M0	3	IIA	Malignant	Fmg040125	*	*	*
F10	60	40	F	Breast	Invasive carcinoma of no	T1N0M0	2	IA	Malignant	Fmg020028	++	+	3+
G1	61	60	F	Breast	Invasive carcinoma of no	T1N0M0	2	IA	Malignant	Fmg100010	++	+	0
G2	62	49	F	Breast	Invasive carcinoma of no	T2N0M0	2	IIA	Malignant	Fmg010492	-	-	0
G3	63	50	F	Breast	Invasive carcinoma of no	T2N1M0	2	IIB	Malignant	Fmg080055	+++	+	0
G4	64	64	F	Breast	Invasive carcinoma of no	T2N0M0	2	IIA	Malignant	Fmg010540	-	-	3+
G5	65	59	F	Breast	Invasive carcinoma of no	T2N0M0	3	IIA	Malignant	Fmg040132	-	-	0
G6	66	38	F	Breast	Invasive carcinoma of no	T3N1M0	2	IIIA	Malignant	Fmg100103	*	*	*
G7	67	56	F	Breast	Invasive carcinoma of no	T2N1M0	2	IIB	Malignant	Fmg020026	-	-	0
G8	68	45	F	Breast	Invasive carcinoma of no	T4N2M0	2	IIIB	Malignant	Fmg010767	+++	+++	0
G9	69	58	F	Breast	Invasive carcinoma of no	T3N1M0	2	IIIA	Malignant	Fmg140019	++	-	0
G10	70	29	F	Breast	Invasive carcinoma of no	T2N0M0	2	IIA	Malignant	Fmg010729	-	-	3+
H1	71	47	F	Breast	Invasive carcinoma of no	T2N1M0	2	IIB	Malignant	Fmg010642	++	+	0
H2	72	55	F	Breast	Invasive carcinoma of no	T2N0M0	2	IIA	Malignant	Fmg010943	++	-	3+
H3	73	45	F	Breast	Invasive carcinoma of no	T1N0M0	2	IA	Malignant	Fmg010547	-	-	0
H4	74	55	F	Breast	Invasive carcinoma of no	T3N2M0	2	IIIA	Malignant	Fmg080090	+++	+++	0
H5	75	54	F	Breast	Invasive carcinoma of no	T2N2M0	2	IIIA	Malignant	Fmg100104	+	-	3+
H6	76	40	F	Breast	Invasive carcinoma of no	T2N0M0	3	IIA	Malignant	Fmg020035	++	+	0
H7	77	53	F	Breast	Invasive carcinoma of no	T2N0M0	3	IIA	Malignant	Fmg020765	-	-	0
H8	78	46	F	Breast	Invasive carcinoma of no	T2N0M0	*	IIA	Malignant	Fmg030512	-	-	0
H9	79	52	F	Breast	Invasive carcinoma of no	T4N2M0	3	IIIB	Malignant	Fmg050004	-	-	3+
H10	80	48	F	Breast	Invasive carcinoma of no	T4N0M0	2–3	IIIB	Malignant	Fmg030528	+++	+++	0
I1	81	27	F	Breast	Invasive carcinoma of no	T2N1M0	3	IIB	Malignant	Fmg040182	+++	-	3+
I2	82	70	F	Breast	Invasive carcinoma of no	T2N0M0	2–3	IIA	Malignant	Fmg030520	-	-	0
I3	83	42	F	Breast	Invasive carcinoma of no	T2N1M0	3	IIB	Malignant	Fmg130067	-	-	0
I4	84	31	F	Breast	Invasive carcinoma of no	T2N0M0	3	IIA	Malignant	Fmg040162	-	-	3+
I5	85	47	F	Breast	Invasive carcinoma of no	T2N0M0	3	IIA	Malignant	Fmg120001	+++	+	0
I6	86	34	F	Breast	Invasive carcinoma of no	T2N0M0	3	IIA	Malignant	Fmg110075	-	-	0
I7	87	56	F	Breast	Invasive carcinoma of no	T2N0M0	3	IIA	Malignant	Fmg020038	+++	-	0
I8	88	37	F	Breast	Invasive carcinoma of no	T2N1M0	3	IIB	Malignant	Fmg080106	-	-	2+
I9	89	43	F	Breast	Invasive carcinoma of no	T1N0M0	3	IA	Malignant	Fmg020443	+++	+	0
I10	90	45	F	Breast	Invasive carcinoma of no	T2N0M0	3	IIA	Malignant	Fmg110115	-	-	0
J1	91	48	F	Breast	Invasive carcinoma of no	T2N1M0	3	IIB	Malignant	Fmg100067	+++	-	0
J2	92	38	F	Breast	Invasive carcinoma of no	T2N0M0	3	IIA	Malignant	Fmg030644	-	-	0
J3	93	47	F	Breast	Invasive carcinoma of no	T2N0M0	3	IIA	Malignant	Fmg110037	-	-	2+
J4	94	51	F	Breast	Invasive carcinoma of no	T3N1M0	3	IIIA	Malignant	Fmg100037	-	-	3+
J5	95	52	F	Breast	Invasive carcinoma of no	T3N0M0	3	IIB	Malignant	Fmg010755	-	-	3+
J6	96	43	F	Breast	Invasive carcinoma of no	T2N1M0	3	IIB	Malignant	Fmg080053	*	*	*
J7	97	68	F	Breast	Invasive carcinoma of no	T2N0M0	3	IIA	Malignant	Fmg030432	+++	+	0
J8	98	30	F	Breast	Invasive carcinoma of no	T2N2M0	3	IIIA	Malignant	Fmg090030	-	-	3+
J9	99	46	F	Breast	Invasive carcinoma of no	T4N0M0	3	IIIB	Malignant	Fmg021845	-	-	0
J10	100	45	F	Breast	Invasive carcinoma of no	T3N1M0	3	IIIA	Malignant	Fmg040344	++	+	3+
K1	101	43	F	Breast	Adjacent normal breast tis	-	*	-	NAT	Fmg010001	+	+	0
K2	102	48	F	Breast	Adjacent normal breast tis	-	-	-	NAT	Fmg110129	+	+	0
K3	103	40	F	Breast	Adjacent normal breast tis	-	-	-	NAT	Fmg021239	+	+	0
K4	104	41	F	Breast	Adjacent normal breast tis	-	-	-	NAT	Fmg140091	+	+	0
K5	105	38	F	Breast	Adjacent normal breast tis	-	*	-	NAT	Fmg021401	+	+	0
K6	106	29	F	Breast	Adjacent normal breast tis	-	-	-	NAT	Fmg140093	+	+	0
K7	107	41	F	Breast	Adjacent normal breast tis	-	*	-	NAT	Fmg021705	*	*	*
K8	108	42	F	Breast	Adjacent normal breast tis	-	*	-	NAT	Fmg020849	+	+	0
K9	109	35	F	Breast	Adjacent normal breast tis	-	-	-	NAT	Fmg060379	*	*	*
K10	110	43	F	Breast	Adjacent normal breast tis	-	-	-	NAT	Fmg100278	*	*	*

Gene	baseMean	log2FoldChai	lfcSE	stat	pvalue	padj
44626	12491.131	-0.2023191	0.03245201	-6.2344071	4.53E-10	7.95E-09
44628	993.50298	0.61850871	0.06938334	8.91436901	4.91E-19	2.13E-17
44630	460.292295	0.72838907	0.09637942	7.55751673	4.11E-14	1.19E-12
44813	20100.6858	0.17782472	0.0376653	4.72118132	2.34E-06	2.19E-05
44814	546.515807	0.45300193	0.08778592	5.16030273	2.47E-07	2.79E-06
A2M	15.3676347	-2.725503	0.5956175	-4.5759283	4.74E-06	4.19E-05
AAGAB	5928.1698	0.1145719	0.04147053	2.76273044	0.00573201	0.0219206
AARD	253.43961	0.68395878	0.1273314	5.37148581	7.81E-08	9.61E-07
AARS	18536.7426	0.51667225	0.03258113	15.8580193	1.24E-56	3.37E-54
ABAT	7199.10851	-0.2200451	0.0393475	-5.5923539	2.24E-08	3.05E-07
ABCA10__AE	3690.48101	-0.5594281	0.05552928	-10.07447	7.16E-24	4.28E-22
ABCA12	9967.15617	0.54841516	0.04224236	12.9825878	1.54E-38	2.00E-36
ABCA2	13417.1011	-0.2536029	0.05110315	-4.9625694	6.96E-07	7.26E-06
ABCC2	264.719964	-0.6018567	0.12595837	-4.7782191	1.77E-06	1.69E-05
ABCC3	4211.74629	-0.6224847	0.04327936	-14.382949	6.62E-47	1.27E-44
ABCC4	573.379869	0.41829279	0.08723292	4.79512553	1.63E-06	1.57E-05
ABCD3	1786.62906	-0.1691228	0.05442289	-3.1075668	0.00188634	0.00851631
ABCD4	1771.8437	-0.1310432	0.05332041	-2.4576563	0.0139847	0.04546086
ABCF1	6802.46225	0.10122194	0.03705771	2.73146752	0.0063053	0.02370043
ABCF2	4037.64125	0.16034873	0.04634121	3.46017617	0.00053982	0.00290357
ABCG2	1335.30669	-0.6627208	0.06041603	-10.969288	5.37E-28	4.11E-26
ABHD11	3678.58988	-0.2139106	0.04401582	-4.8598575	1.17E-06	1.17E-05
ABL1	5532.31536	0.2203512	0.04184574	5.26579743	1.40E-07	1.66E-06
ABLIM1	4346.25173	0.30401066	0.04281901	7.09989926	1.25E-12	3.01E-11
ABR	7038.6747	0.18260803	0.03778691	4.83257404	1.35E-06	1.32E-05
ABRACL	2185.06378	0.2593293	0.05814596	4.45997127	8.20E-06	6.85E-05
ABTB1	673.244541	-0.3362747	0.08032753	-4.1862941	2.84E-05	0.00020881
AC002310.11	7152.23356	0.13709055	0.0377246	3.63398276	0.00027908	0.00163205
AC003002.4_	1504.45102	-0.2943918	0.05864363	-5.0200126	5.17E-07	5.55E-06
AC005077.14	70.9237934	-0.7076562	0.23324944	-3.0339029	0.00241412	0.01048323
AC005253.2	222.154287	-0.5051381	0.14612238	-3.4569523	0.00054632	0.00293075
AC005523.2	1557.35784	0.22464619	0.05652708	3.97413377	7.06E-05	0.00047579
AC005538.3_	1086.97482	0.1825666	0.06776368	2.69416598	0.0070565	0.02610433
AC005785.2	25.3254803	-1.3483146	0.40279349	-3.3474093	0.00081571	0.00413156
AC006128.2	191.950708	-0.5849031	0.15412493	-3.7949935	0.00014765	0.0009242
AC006486.9_	7598.13751	0.25100597	0.0377905	6.64203906	3.09E-11	6.40E-10
AC007040.11	6505.65474	0.28565228	0.03792627	7.53177814	5.01E-14	1.43E-12
AC007192.4_	10683.3159	0.24895272	0.03736775	6.66223413	2.70E-11	5.60E-10
AC007325.2	26.2687524	-0.9472656	0.39161275	-2.4188834	0.01556823	0.04960181
AC007563.5	1938.77262	1.33664823	0.13608063	9.82247275	9.01E-23	5.04E-21
AC008074.1	1341.04679	0.23000337	0.06073939	3.78672484	0.00015265	0.00095157
AC009095.4	30.8361936	-0.9322749	0.37627103	-2.4776686	0.01322439	0.04343965

AC009133.12	11941.5695	0.1561229	0.03391669	4.60312971	4.16E-06	3.72E-05
AC009133.15	3980.09841	0.35714697	0.05831611	6.12432755	9.11E-10	1.54E-08
AC009133.21	842.566867	-0.4054931	0.075105	-5.3990163	6.70E-08	8.34E-07
AC009133.22	3839.98151	0.44248442	0.04584991	9.65071454	4.88E-22	2.59E-20
AC009237.8	684.068818	0.20541628	0.0797283	2.57645378	0.00998196	0.03478396
AC010203.1_	819.347637	-0.4232669	0.0761316	-5.5596735	2.70E-08	3.63E-07
AC011530.4_	3921.74348	-0.2259049	0.04662658	-4.8449811	1.27E-06	1.25E-05
AC011747.4	533.425275	-0.2394084	0.08917273	-2.6847714	0.00725794	0.0266871
AC016629.7_	1636.68038	0.34335607	0.06042975	5.68190439	1.33E-08	1.89E-07
AC024560.2	17.6774046	-1.397632	0.48902089	-2.858021	0.00426292	0.01702741
AC034220.3	66.679506	-0.7065672	0.24041511	-2.9389465	0.0032933	0.01366948
AC037459.4_	722.287723	-0.1969994	0.07876205	-2.501197	0.01237743	0.0412898
AC058791.1_	70.2122793	-0.7934198	0.23710282	-3.3463112	0.00081894	0.00414565
AC068533.7_	5015.9792	0.29398626	0.03907239	7.52414435	5.31E-14	1.50E-12
AC068831.15	827.051661	-0.4337738	0.07275702	-5.961951	2.49E-09	3.98E-08
AC073610.5_	17280.2181	0.10263647	0.03435271	2.98772525	0.00281062	0.01196106
AC093838.4	1539.44375	-0.2980056	0.06327103	-4.709985	2.48E-06	2.31E-05
AC114730.7	158.640361	-0.8110542	0.1585208	-5.1163901	3.11E-07	3.47E-06
AC138969.4	478.227381	-0.3361776	0.10228905	-3.2865455	0.00101424	0.0049808
AC159540.1	902.216675	-0.4747309	0.07757876	-6.1193402	9.40E-10	1.58E-08
AC240274.1	680.655263	-0.4824188	0.08553067	-5.6403023	1.70E-08	2.36E-07
ACAD11__NF	2597.47672	-0.314715	0.04807664	-6.5461104	5.91E-11	1.17E-09
ACADM__RA	3983.61761	-0.2504631	0.04559602	-5.4930901	3.95E-08	5.13E-07
ACADVL	8836.31715	-0.4984736	0.03738766	-13.332569	1.50E-40	2.15E-38
ACAP1	116.949642	-0.8140083	0.19087238	-4.2646733	2.00E-05	0.00015286
ACAP3	2657.61354	-0.3107004	0.06641396	-4.6782392	2.89E-06	2.66E-05
ACAT1	1062.00069	0.30917296	0.07253475	4.2624114	2.02E-05	0.00015429
ACBD7	341.020243	0.48087465	0.10938068	4.39633989	1.10E-05	8.93E-05
ACCS	531.011278	-0.3847013	0.08809219	-4.3670305	1.26E-05	0.00010042
ACKR3	4230.72446	-0.5646611	0.04636702	-12.178075	4.07E-34	4.26E-32
ACLY	11785.3226	0.23207521	0.03395117	6.83555938	8.17E-12	1.79E-10
ACO1	1817.97541	0.17341089	0.05288948	3.278741	0.00104271	0.00508909
ACOT7	2569.61047	0.15235336	0.04908967	3.1035728	0.00191199	0.00862098
ACOT8	1126.18297	-0.2182039	0.0649208	-3.3610775	0.00077639	0.0039688
ACP6	2345.27303	-0.1878271	0.04947257	-3.796591	0.0001467	0.0009189
ACSBG1	1952.26442	-0.2003483	0.06301499	-3.1793755	0.00147593	0.00688993
ACSL1	942.025463	-0.3091853	0.0705976	-4.3795434	1.19E-05	9.54E-05
ACSL6__CTB-	46.0404811	-1.3505122	0.31433157	-4.2964574	1.74E-05	0.00013483
ACTA2	358.836314	0.5782748	0.10986124	5.26368366	1.41E-07	1.67E-06
ACTG2	120.407946	-3.6498334	0.25572591	-14.272443	3.25E-46	6.05E-44
ACTL8	138.298107	1.24124727	0.17992006	6.89888205	5.24E-12	1.17E-10
ACTN4	29404.1663	0.10979444	0.03335303	3.29188835	0.00099517	0.00489769
ACTR10	2823.0131	-0.1779177	0.04818067	-3.6927185	0.00022187	0.00132772

ACTR1A	5774.34219	0.1768581	0.03899234	4.53571431	5.74E-06	4.98E-05
ACVR2B	1723.20477	0.19545252	0.06140371	3.1830736	0.00145721	0.00681824
ADAM22	732.491164	0.31303033	0.08798075	3.55794106	0.00037377	0.00210937
ADAM8	390.888198	-0.5094187	0.12428745	-4.0987138	4.15E-05	0.00029499
ADAMTS1	30.765226	-1.5670062	0.38170024	-4.105332	4.04E-05	0.0002879
ADAMTS13	294.9718	-0.4338074	0.12014145	-3.6108058	0.00030525	0.00176582
ADAMTS9	650.538008	2.76184475	0.09655175	28.6048117	5.85E-180	1.33E-176
ADCY3	3331.19948	0.15533022	0.04652732	3.33847384	0.0008424	0.00424201
ADCY4__RIPI	24.2288311	-1.6501046	0.42287847	-3.9020777	9.54E-05	0.00062396
ADD1	6898.75611	0.12859495	0.03701281	3.47433657	0.00051212	0.00277173
ADGRD1	90.343176	0.64616914	0.21605719	2.99073187	0.0027831	0.01186509
ADGRE5	922.333343	0.21427208	0.07114569	3.01173656	0.00259758	0.01116818
ADGRF2__AI	515.444338	-0.4914421	0.09061976	-5.423123	5.86E-08	7.38E-07
ADGRG1	9009.89357	0.18768406	0.04359983	4.30469701	1.67E-05	0.00013052
ADGRG5	106.885487	0.74614105	0.1922139	3.88182663	0.00010368	0.00067155
ADGRL1	4315.02191	0.1462408	0.04151028	3.52300232	0.00042669	0.00236481
ADH5	5212.19198	0.11507837	0.03875479	2.96939726	0.00298385	0.01256523
ADI1	5184.28283	0.17440074	0.04125119	4.22777448	2.36E-05	0.00017688
ADIPOR1	8722.05269	0.2624245	0.03958237	6.62983367	3.36E-11	6.91E-10
ADIRF__FAM	310.961869	-0.582109	0.12039473	-4.8350043	1.33E-06	1.31E-05
ADM	107.569511	-0.7786859	0.19570511	-3.9788737	6.92E-05	0.00046727
ADM5__PRM	11788.4616	0.28227299	0.03629989	7.77613885	7.48E-15	2.33E-13
ADNP	18844.417	0.23951711	0.03332841	7.18657496	6.64E-13	1.65E-11
ADO	2361.16768	0.18798783	0.05184428	3.62600924	0.00028784	0.00167787
ADORA2A__	3184.34486	0.20991153	0.04989635	4.20695139	2.59E-05	0.0001924
ADRBK2	578.497645	0.48000104	0.08579611	5.59467167	2.21E-08	3.01E-07
AES	6202.55006	0.14918141	0.03950287	3.77646979	0.00015907	0.0009857
AF011889.5_	7945.95149	-0.5221348	0.03772039	-13.842242	1.42E-43	2.33E-41
AFAP1	2273.82226	0.37717551	0.05948642	6.34053116	2.29E-10	4.20E-09
AFG3L1P__R	2662.92365	-0.1923792	0.04954651	-3.8828006	0.00010326	0.00066958
AGAP2-AS1	1352.18991	0.42741557	0.07270844	5.87848644	4.14E-09	6.42E-08
AGAP4	167.207974	-0.4434687	0.15344442	-2.8900933	0.00385128	0.01561578
AGAP5	446.207664	-0.331574	0.09547783	-3.4727853	0.00051509	0.00278532
AGAP6	451.396757	-0.4086623	0.10117972	-4.0389744	5.37E-05	0.00037108
AGAP7P	83.1209627	-0.5533845	0.2191402	-2.5252532	0.0115615	0.03911174
AGAP9	307.640465	-0.5076132	0.11975533	-4.2387523	2.25E-05	0.00016964
AGER	158.808945	-0.9948325	0.16263975	-6.1167857	9.55E-10	1.60E-08
AGMAT	522.837494	0.803133	0.08933232	8.99039664	2.46E-19	1.10E-17
AGO1	2711.49059	0.27476665	0.05137307	5.34845664	8.87E-08	1.08E-06
AGO4	1198.98939	-0.357947	0.06206131	-5.7676353	8.04E-09	1.19E-07
AGPAT1	2722.47051	0.1506127	0.04874819	3.08960589	0.00200422	0.0089593
AGPAT3	7011.42451	0.29376687	0.03786992	7.75726073	8.68E-15	2.68E-13
AGR3	810.264182	-0.2080762	0.07526463	-2.7645948	0.00569936	0.02181861

AGTRAP	1342.72037	0.3426341	0.06952903	4.92792883	8.31E-07	8.52E-06
AHCY	10318.3365	0.35964422	0.0386212	9.31209307	1.25E-20	6.05E-19
AHNAK2	1402.15097	0.22862905	0.07005798	3.26342612	0.00110074	0.00534084
AHRR__PDCI	6047.3202	-0.1651688	0.03728065	-4.4304166	9.41E-06	7.74E-05
AHSA1	12703.7086	0.09462638	0.03417455	2.76891361	0.00562436	0.02158133
AHSA2	2497.59721	-0.7043457	0.04762311	-14.789997	1.70E-49	3.60E-47
AIF1L	5722.24388	0.3143361	0.03882042	8.09718441	5.62E-16	1.96E-14
AIFM1	4898.47888	0.11252259	0.04009019	2.80673592	0.00500463	0.01954892
AIM1	2744.84856	-0.4692113	0.05068062	-9.2581987	2.08E-20	9.85E-19
AJUBA__HAU	4722.04948	0.11659989	0.04208068	2.77086507	0.00559076	0.02147049
AK4	3400.52334	-0.2719289	0.04402914	-6.1761119	6.57E-10	1.13E-08
AKAP13	5171.81953	0.163762	0.05642484	2.90230314	0.0037043	0.01511394
AKNAD1__CL	3561.92006	-0.1389602	0.04558815	-3.0481652	0.00230243	0.01007772
AKR1A1	2834.09435	0.20437575	0.04684744	4.3625813	1.29E-05	0.00010235
AKR1C2	9583.59594	-0.1116868	0.03556713	-3.140169	0.0016885	0.00775326
AKR7A2	2666.45692	0.19330697	0.04847362	3.98787941	6.67E-05	0.00045156
AKR7A3	47.7166427	-1.0475475	0.31180202	-3.3596559	0.0007804	0.00398592
AKT2	8053.64201	0.19502304	0.03963055	4.9210278	8.61E-07	8.80E-06
AL449212.1_	673.916672	-0.3999725	0.09509279	-4.2061284	2.60E-05	0.00019303
ALAD	2358.20094	-0.3063899	0.04852089	-6.3145972	2.71E-10	4.91E-09
ALDH16A1__	91725.0505	0.18169517	0.03102161	5.8570512	4.71E-09	7.23E-08
ALDH18A1	7438.74686	0.28747853	0.03576231	8.03858899	9.09E-16	3.13E-14
ALDH1A3	587.525374	-0.6031322	0.08768638	-6.8782887	6.06E-12	1.34E-10
ALDH1B1	1713.6219	0.31600289	0.05525774	5.71870804	1.07E-08	1.55E-07
ALDH3A2	3693.87855	-0.1201265	0.0472313	-2.5433665	0.010979	0.03760163
ALDH3B1	314.933287	-0.5401867	0.11400797	-4.7381486	2.16E-06	2.04E-05
ALDH4A1__II	2636.72164	0.23256441	0.0509239	4.56690111	4.95E-06	4.35E-05
ALDH5A1	4715.81701	0.3074148	0.0422647	7.27356008	3.50E-13	9.07E-12
ALDH6A1	2986.82265	0.20278693	0.05009138	4.0483402	5.16E-05	0.0003579
ALDH7A1	2952.31596	0.1828072	0.04602927	3.97154282	7.14E-05	0.00048047
ALDOA	95839.8394	-0.5453123	0.0370497	-14.718399	4.91E-49	1.03E-46
ALDOC	749.38081	-0.5425046	0.07629789	-7.1103495	1.16E-12	2.80E-11
ALG13	2401.91535	-0.1451104	0.04835391	-3.0010058	0.00269089	0.01151508
ALG8	3633.50755	0.14801324	0.04559531	3.24623803	0.00116941	0.00563207
ALKBH4	1005.17349	0.23731891	0.0681413	3.48274698	0.0004963	0.00269728
ALOX12P2	566.110035	-0.4289491	0.09913219	-4.3270414	1.51E-05	0.00011868
ALS2CL	440.327024	-0.9037878	0.1012452	-8.9267225	4.39E-19	1.92E-17
ALYREF	6532.4833	0.26214462	0.0371302	7.0601455	1.66E-12	3.96E-11
AMBRA1	2552.77897	0.18164037	0.04803017	3.7817976	0.0001557	0.00096796
AMER1	638.014239	0.22022058	0.08560504	2.57251875	0.01009615	0.03507873
AMER2	185.178597	-0.8003633	0.15056817	-5.3156211	1.06E-07	1.28E-06
AMFR	6271.38928	0.28273732	0.04187511	6.75191836	1.46E-11	3.12E-10
AMH	655.313811	-0.6077591	0.10506115	-5.784813	7.26E-09	1.08E-07

AMIGO2	10379.1241	-0.4802898	0.03921371	-12.248007	1.72E-34	1.84E-32
AMIGO3__G	2303.47127	0.17167271	0.05268537	3.25845151	0.00112022	0.00542238
AMOT	941.615817	-0.2343071	0.07489433	-3.128503	0.00175699	0.00801586
AMOTL2	4137.40397	-0.332201	0.04207194	-7.8960236	2.88E-15	9.53E-14
AMT	140.477793	-1.7298133	0.18018852	-9.6000196	7.99E-22	4.19E-20
AMTN	14.757623	-1.5360064	0.56255513	-2.7304104	0.00632555	0.0237659
AMY2B__RN	570.885482	-0.498403	0.09613077	-5.1846347	2.16E-07	2.46E-06
ANAPC7__AI	14577.2335	0.21210301	0.03301758	6.42394107	1.33E-10	2.52E-09
ANGPTL6	146.940698	-0.5246606	0.17783053	-2.9503404	0.00317424	0.01324464
ANG__RNAS	115.029664	-1.5521755	0.20588782	-7.5389382	4.74E-14	1.35E-12
ANK3	4372.35592	0.15040079	0.04572156	3.28949366	0.00100368	0.00493556
ANKRD1	43.3831961	0.94940817	0.30252145	3.13831687	0.00169921	0.00778861
ANKRD10	955.834461	-0.3234423	0.06925836	-4.6700841	3.01E-06	2.75E-05
ANKRD11	11966.7832	0.18652599	0.03860053	4.83221298	1.35E-06	1.32E-05
ANKRD13B	821.076855	0.30388001	0.08347121	3.64053674	0.00027207	0.00159618
ANKRD18A	756.913698	-0.4992222	0.07668132	-6.5103498	7.50E-11	1.47E-09
ANKRD18B	618.8053	-0.5293756	0.0846159	-6.256219	3.94E-10	6.98E-09
ANKRD23__/	2015.96295	-0.1371648	0.05174659	-2.6507019	0.00803247	0.02907947
ANKRD24	154.899511	-0.7676877	0.1813414	-4.2333836	2.30E-05	0.00017324
ANKRD27	4069.01294	0.31783055	0.04380874	7.25495717	4.02E-13	1.03E-11
ANKRD34B	363.454244	-0.3236106	0.10470382	-3.0907243	0.00199669	0.00892782
ANKRD35	273.604397	-0.4081911	0.1256985	-3.247382	0.00116472	0.00561393
ANKRD36	511.154796	-0.6200741	0.0941082	-6.5889494	4.43E-11	8.99E-10
ANKRD36B	239.85098	-0.7765422	0.13021585	-5.9635	2.47E-09	3.95E-08
ANKRD36BP2	28.2111479	-1.5331732	0.41254643	-3.7163653	0.00020211	0.00121988
ANKRD36C	374.697277	-0.3801372	0.11533389	-3.2959709	0.00098082	0.00484315
ANKRD40	2999.29588	0.25265465	0.04456941	5.66879023	1.44E-08	2.03E-07
ANKRD52	11000.9452	0.23788746	0.04955559	4.80041663	1.58E-06	1.53E-05
ANKS1A	2778.77055	0.21056809	0.05263207	4.00075612	6.31E-05	0.00042927
ANKUB1__PI	10982.6383	-0.2356288	0.04069123	-5.7906527	7.01E-09	1.05E-07
ANKZF1	2983.42053	-0.5509613	0.05143083	-10.712667	8.88E-27	6.42E-25
ANLN	7029.95839	0.24437987	0.04128196	5.91977446	3.22E-09	5.07E-08
ANO10	2127.37557	0.17735947	0.05423702	3.27008145	0.00107517	0.00523067
ANO7	323.930449	-0.5557144	0.11682949	-4.7566277	1.97E-06	1.87E-05
ANO9	1315.07792	-0.7284589	0.06046971	-12.046674	2.02E-33	2.06E-31
ANP32A	9811.91098	0.15271175	0.04458316	3.42532367	0.00061407	0.003237
ANXA1	315.030993	-0.4736004	0.1161909	-4.0760542	4.58E-05	0.00032149
ANXA11	5412.80352	0.12161814	0.03998194	3.04182676	0.00235147	0.01025269
ANXA3	1695.86205	-0.2607541	0.06237122	-4.1806793	2.91E-05	0.00021378
ANXA6	1814.0545	-0.2504659	0.05394018	-4.6434008	3.43E-06	3.11E-05
ANXA9	2561.11659	0.23255971	0.04857545	4.78759762	1.69E-06	1.62E-05
AP000295.9_	1869.57645	0.27327806	0.05553593	4.92074321	8.62E-07	8.81E-06
AP000769.1	7787.01142	-0.8316932	0.04715557	-17.63722	1.28E-69	5.06E-67

AP000866.1	25.2897925	-1.4723045	0.41829632	-3.5197645	0.00043193	0.00239168
AP001062.7	899.055447	-0.2226916	0.07525715	-2.9590755	0.00308564	0.01293116
AP003391.1	574.613854	0.50336515	0.10513598	4.78775331	1.69E-06	1.62E-05
AP1B1	8792.853	0.25449502	0.04893568	5.20060246	1.99E-07	2.28E-06
AP1G2__JPH	5120.48969	-0.4596607	0.04367863	-10.523698	6.72E-26	4.64E-24
AP1M2	9625.04774	0.22586607	0.03478624	6.49297257	8.42E-11	1.64E-09
AP1S1	10048.2752	0.26742278	0.03808886	7.02102367	2.20E-12	5.14E-11
AP2A2	2679.87993	0.19512691	0.05591628	3.48962623	0.0004837	0.00263985
AP2B1	10721.8113	0.2931925	0.03754159	7.80980503	5.73E-15	1.82E-13
AP2M1	25157.4993	0.09584843	0.03727378	2.57147078	0.01012676	0.03516497
AP3B1	4199.49859	0.14799043	0.04090245	3.61813098	0.00029674	0.00172371
AP3D1	7280.87064	0.10698847	0.04057946	2.63651802	0.00837618	0.0301045
AP4M1	1220.0254	0.30405526	0.06508451	4.67169948	2.99E-06	2.73E-05
AP5B1	2264.79306	0.3019804	0.05641532	5.3528089	8.66E-08	1.06E-06
APBB3__SRA	2204.72043	-0.1597257	0.0487187	-3.2785288	0.0010435	0.00509155
APEH	6207.10581	0.280895	0.04298847	6.53419349	6.40E-11	1.26E-09
APEX2	2295.72172	0.13081712	0.05398956	2.42300745	0.01539261	0.04913671
APH1A	14926.5468	0.15174332	0.03149139	4.81856564	1.45E-06	1.41E-05
APOA1BP	3071.34373	0.2503712	0.04474638	5.59533942	2.20E-08	3.01E-07
APOBEC3A__	641.278692	0.50191063	0.08781393	5.71561545	1.09E-08	1.58E-07
APOL1	92.6389393	-0.5822623	0.20626787	-2.8228451	0.00475996	0.01872154
APOL2	726.188322	-0.4501449	0.08173135	-5.5076168	3.64E-08	4.73E-07
APOLD1__DD	3622.52294	0.10939206	0.04301298	2.54323389	0.01098317	0.03760884
APP	17551.5424	0.0993629	0.03299016	3.01189481	0.00259623	0.01116499
APRT	7720.27222	0.17418971	0.03509939	4.96275532	6.95E-07	7.26E-06
AQP1__FAM	308.083683	0.32890513	0.11642036	2.8251514	0.00472583	0.01861541
AQP3	2183.33919	-0.7173382	0.05073519	-14.138869	2.19E-45	3.87E-43
ARCN1	13982.0513	0.24495042	0.03291982	7.44081791	1.00E-13	2.74E-12
ARF5__FSCN	6669.17503	0.14821546	0.03987952	3.71658054	0.00020194	0.00121925
ARFGAP3__F	3045.82744	0.2113657	0.04591864	4.60304791	4.16E-06	3.72E-05
ARG2	81.2812768	0.56172619	0.22530708	2.49315816	0.01266124	0.04197548
ARGLU1	2806.28743	-0.1763905	0.04707076	-3.7473486	0.00017871	0.00109425
ARHGAP1	4594.61554	0.15879118	0.04204798	3.77642871	0.00015909	0.0009857
ARHGAP10	1337.34825	0.168636	0.06062244	2.78174225	0.0054068	0.02090047
ARHGAP11A	4523.12154	0.23280188	0.04192192	5.55322534	2.80E-08	3.75E-07
ARHGAP12	3676.96015	-0.1440078	0.04531691	-3.1777948	0.001484	0.00692052
ARHGAP18	800.025427	0.32082107	0.073178	4.38411904	1.16E-05	9.39E-05
ARHGAP19__	1600.34386	0.30320512	0.05759738	5.26421725	1.41E-07	1.67E-06
ARHGAP29	601.87198	-0.6147117	0.08398943	-7.3189178	2.50E-13	6.55E-12
ARHGAP30	108.294211	-0.4566533	0.18782904	-2.431218	0.01504816	0.04819745
ARHGAP32	2339.93747	0.1873283	0.06969517	2.68782336	0.00719194	0.0264925
ARHGAP33	942.181872	-0.3684151	0.0728269	-5.0587784	4.22E-07	4.59E-06
ARHGAP36	906.579231	0.6822472	0.0827469	8.24498764	1.65E-16	5.93E-15

ARHGAP39_	4303.18472	0.25384636	0.05772896	4.39720986	1.10E-05	8.90E-05
ARHGAP4__I	6549.57655	-0.2608165	0.0357922	-7.2869636	3.17E-13	8.23E-12
ARHGDIA	17199.6162	0.09948902	0.03921601	2.53694879	0.01118233	0.03808342
ARHGDIB	76.9108382	-1.2668071	0.24349476	-5.2026051	1.97E-07	2.26E-06
ARHGEF10L	2310.35865	-0.2733902	0.05714829	-4.7838738	1.72E-06	1.65E-05
ARHGEF18_	1435.42717	0.45584988	0.06559221	6.94975698	3.66E-12	8.32E-11
ARHGEF19	1577.43052	0.18105739	0.06252633	2.89569853	0.00378316	0.01537376
ARHGEF2	1212.96242	0.25724764	0.06312408	4.07526963	4.60E-05	0.00032245
ARHGEF26	828.553097	-0.372458	0.08238828	-4.5207641	6.16E-06	5.30E-05
ARHGEF40	301.128528	-0.2993896	0.11972641	-2.5006144	0.01239781	0.04134466
ARID1A	7592.83858	0.16668518	0.04057475	4.10810141	3.99E-05	0.00028497
ARID2	4718.64226	0.19245427	0.04260644	4.51702258	6.27E-06	5.39E-05
ARID5B	1796.55467	-0.2675475	0.05524547	-4.8428857	1.28E-06	1.26E-05
ARL1	3679.37889	-0.1065792	0.04318086	-2.4682047	0.01357926	0.04446916
ARL2__ARL2	3484.20293	0.21702178	0.04252858	5.10296279	3.34E-07	3.71E-06
ARL3	2887.97127	0.13484701	0.04782042	2.81986237	0.00480443	0.01886786
ARL8A	2810.64189	0.13948815	0.04603149	3.03027645	0.0024433	0.01060741
ARMC10	4225.82057	0.12663614	0.04557927	2.77837152	0.00546321	0.02107018
ARMC6	2308.91599	0.28497092	0.05124947	5.56046533	2.69E-08	3.62E-07
ARMCX3	5923.8887	0.15767312	0.03804594	4.14428203	3.41E-05	0.00024773
ARNT	4273.01828	0.12949564	0.04159931	3.11292792	0.00185241	0.0083911
ARNTL	464.789497	0.37372662	0.09522584	3.92463448	8.69E-05	0.00057344
ARPC1A__AF	21045.9108	0.25006911	0.03304826	7.56678622	3.83E-14	1.11E-12
ARRB1	926.87912	-0.1986966	0.07356793	-2.7008594	0.00691606	0.02565804
ARRDC1	5570.59225	-0.2568858	0.05434063	-4.7273256	2.27E-06	2.14E-05
ARRDC3	5282.66098	-0.2901862	0.04128777	-7.0283805	2.09E-12	4.90E-11
ARRDC4	3049.44998	-0.1632116	0.04810393	-3.3928959	0.00069158	0.00357637
ASAP1	4011.35141	0.2913957	0.04329461	6.73053047	1.69E-11	3.60E-10
ASB9	174.243164	-0.4826224	0.15737838	-3.0666374	0.00216481	0.00956231
ASCL1	652.423248	-1.3606252	0.08544621	-15.923764	4.33E-57	1.22E-54
ASF1B	4860.45378	0.22622662	0.0445471	5.07836955	3.81E-07	4.20E-06
ASIC3	109.826584	-0.5150096	0.19649483	-2.6209828	0.00876767	0.03121845
ASIC4	47.975882	-1.0151499	0.30397183	-3.3396185	0.00083894	0.0042273
ASMTL-AS1	80.5725537	-1.4136172	0.24286091	-5.8206863	5.86E-09	8.89E-08
ASNA1	4680.89065	0.28179313	0.04006778	7.0329103	2.02E-12	4.75E-11
ASNS	3070.51827	0.83717802	0.04968266	16.8505072	1.04E-63	3.51E-61
ASPM	5142.5226	0.16415159	0.05122095	3.20477458	0.00135168	0.00640005
ASPRV1	668.658125	-0.3862255	0.08209385	-4.704683	2.54E-06	2.36E-05
ATAD3B	1238.28998	-0.3372136	0.07005804	-4.8133465	1.48E-06	1.44E-05
ATAD3C	91.0177869	-0.6251906	0.20736848	-3.0148778	0.00257083	0.01107667
ATAD5	752.009297	-0.4949922	0.07683299	-6.4424437	1.18E-10	2.24E-09
ATF2	2040.33841	0.16948629	0.05265497	3.21880892	0.00128724	0.00614439
ATF4	8708.21818	0.4503677	0.03671538	12.2664576	1.37E-34	1.48E-32

ATF5	3073.63332	0.41634694	0.04391637	9.48044885	2.53E-21	1.27E-19
ATF7IP	4435.67859	0.18125785	0.04186249	4.32983965	1.49E-05	0.00011728
ATG10	585.208046	-0.2409208	0.0864924	-2.7854566	0.00534524	0.02069762
ATG13	4394.88311	0.27777817	0.04553964	6.09970004	1.06E-09	1.77E-08
ATG16L2	1278.00054	-0.9081114	0.063699	-14.256289	4.10E-46	7.55E-44
ATHL1	771.657184	-0.8033269	0.07901938	-10.166201	2.81E-24	1.71E-22
ATIC	15761.2328	0.30332068	0.03269286	9.27788899	1.73E-20	8.25E-19
ATN1	8479.20109	0.13060586	0.04216312	3.09763266	0.00195073	0.00874806
ATP13A1	5161.40288	0.16666815	0.05373378	3.10173865	0.00192388	0.008666
ATP13A3	6301.09949	0.28428849	0.03700473	7.68248997	1.56E-14	4.73E-13
ATP1A3	374.721093	0.55208789	0.10913339	5.0588359	4.22E-07	4.59E-06
ATP1B1	6678.75737	-0.5474507	0.03893296	-14.061368	6.56E-45	1.13E-42
ATP2A1	87.8559694	-1.0691843	0.21562243	-4.958595	7.10E-07	7.37E-06
ATP2A3	6420.81696	-0.27542	0.0444471	-6.19658	5.77E-10	1.00E-08
ATP2B1	7795.90549	0.10529051	0.03727875	2.8244107	0.00473677	0.01864641
ATP2C2	1851.49738	0.54087615	0.06131487	8.82128875	1.13E-18	4.75E-17
ATP5B	32101.9533	0.16406505	0.03151988	5.20513	1.94E-07	2.23E-06
ATP5S	1386.25624	-0.3219273	0.05971781	-5.3908087	7.01E-08	8.68E-07
ATP6AP1L	132.022024	-0.4768116	0.17688195	-2.6956488	0.00702517	0.02600425
ATP6V0A4	132.283354	-0.7494034	0.17859619	-4.1960771	2.72E-05	0.0002009
ATP6V0D1	5145.75695	0.14633501	0.04011631	3.64776814	0.00026453	0.00155579
ATP6V1B1	206.669188	-1.0974292	0.1640072	-6.6913475	2.21E-11	4.65E-10
ATP6V1C1	6469.18248	0.10504514	0.03841186	2.7347058	0.00624361	0.02350246
ATP6V1G2__	19230.2273	-0.1658271	0.03097748	-5.3531517	8.64E-08	1.06E-06
ATP8A2	979.696036	1.34787017	0.07555248	17.8401845	3.45E-71	1.40E-68
ATP8B1	3986.04595	0.26109666	0.04184198	6.24006435	4.37E-10	7.68E-09
ATP8B2	2555.21837	0.15471542	0.04901322	3.15660619	0.00159617	0.00738131
ATP8B3	40.4256476	-0.7816912	0.30779537	-2.5396459	0.01109648	0.03786174
ATRNL1	1710.93034	0.61159871	0.05979673	10.2279632	1.49E-24	9.25E-23
ATXN2L	11089.5585	0.15713449	0.03751178	4.18893729	2.80E-05	0.00020656
ATXN7L3	3264.87612	0.3099579	0.04410463	7.0277861	2.10E-12	4.92E-11
ATXN7L3B	14491.7422	0.09128993	0.03318888	2.75061828	0.00594829	0.02256317
AUP1	6530.90995	-0.1248593	0.04001652	-3.1201933	0.00180732	0.00821404
AURKA	7146.73583	0.35407855	0.03915222	9.04364025	1.52E-19	6.86E-18
AVIL	143.790116	-0.6177094	0.17233192	-3.5844166	0.00033783	0.00192682
AZGP1	80.9427412	-0.9832175	0.2244955	-4.3796758	1.19E-05	9.54E-05
AZIN1	18829.6348	-0.1394693	0.03034919	-4.5954869	4.32E-06	3.83E-05
AZU1	24.6051203	-1.1016158	0.42868201	-2.5697738	0.01017649	0.03530407
B2M	11362.5445	-0.3060701	0.03302962	-9.2665337	1.92E-20	9.15E-19
B3GNT9	851.4321	0.42603382	0.07266873	5.86268407	4.55E-09	7.00E-08
B3GNTL1	622.245013	-0.2185204	0.08266932	-2.6433069	0.00821006	0.02959711
B4GALNT4	2337.68228	-0.2480177	0.06350493	-3.9054881	9.40E-05	0.00061656
B4GALT2	1970.52212	0.16307118	0.06360803	2.56368849	0.01035665	0.03577929

B4GALT3	1547.27246	0.24024686	0.05893847	4.07623154	4.58E-05	0.00032137
B4GALT5	3793.00928	0.3108315	0.04748372	6.54606411	5.91E-11	1.17E-09
BACE2	1828.81385	0.18866559	0.05338465	3.5340796	0.0004092	0.00227479
BACH1-AS1	62.6340605	0.76938885	0.25905527	2.96997957	0.0029782	0.01254433
BAG1	4611.15046	-0.3949923	0.042983	-9.1895013	3.95E-20	1.83E-18
BAG3	8098.08175	-0.6321351	0.04164228	-15.180128	4.79E-52	1.16E-49
BAG6	13420.3881	0.12548536	0.04046215	3.10130255	0.00192671	0.00866807
BAIAP2	1972.93692	0.2180924	0.06169715	3.53488605	0.00040795	0.00226994
BAIAP2-AS1	1208.30579	0.20523954	0.06390277	3.21174721	0.0013193	0.00627444
BAIAP3	884.306025	-0.4217588	0.08683546	-4.8569884	1.19E-06	1.19E-05
BAMBI	12730.7209	0.57297916	0.03344139	17.1338302	8.30E-66	3.03E-63
BANP	1200.58651	0.37808048	0.06504969	5.81217967	6.17E-09	9.29E-08
BAX	4070.67084	0.28589007	0.04537053	6.3012283	2.95E-10	5.30E-09
BAZ1B	10080.3139	0.128228	0.04376606	2.92985047	0.00339125	0.01400274
BAZ2A	8725.90308	0.19071994	0.04603195	4.14320771	3.42E-05	0.0002487
BBIP1	1555.56736	-0.2866216	0.06261489	-4.5775305	4.70E-06	4.16E-05
BBS12	162.069491	0.71027238	0.15905357	4.46561745	7.98E-06	6.69E-05
BBS1__CTD-:	8075.82914	0.23546104	0.03582591	6.57236728	4.95E-11	9.99E-10
BBS9	859.779358	0.27219969	0.07543864	3.60822621	0.0003083	0.00178121
BBX	4468.3274	-0.2541479	0.04401661	-5.7739093	7.75E-09	1.15E-07
BCAR3	658.562345	-0.4033301	0.08494459	-4.7481558	2.05E-06	1.94E-05
BCAS4	1062.85008	0.1698878	0.06678368	2.54385195	0.01096375	0.03756264
BCAT2	2612.22814	0.16517436	0.04818512	3.42791192	0.00060824	0.00321004
BCCIP	3226.31429	-0.1218706	0.0442475	-2.7542947	0.00588188	0.02237175
BCL2	3340.05012	0.74114189	0.04659283	15.9067787	5.69E-57	1.57E-54
BCL2L11	4504.03043	-0.3084258	0.04043659	-7.6273941	2.40E-14	7.14E-13
BCL2L12	1726.61477	0.18796828	0.06134377	3.06417888	0.00218268	0.00962958
BCL2L2__BCI	12553.8848	-0.2178173	0.03262189	-6.6770294	2.44E-11	5.10E-10
BCL6	639.677651	-0.7068502	0.08482117	-8.3334176	7.85E-17	2.88E-15
BCL9P1	14.9908198	-1.7236017	0.56211502	-3.0662794	0.00216741	0.00957145
BCLAF1	10432.1121	-0.1477121	0.03628913	-4.0704234	4.69E-05	0.00032886
BCOR	3118.39833	0.24128019	0.05450275	4.4269364	9.56E-06	7.85E-05
BCORL1	1263.0728	0.41052014	0.06417131	6.39725334	1.58E-10	2.97E-09
BCR	4344.13407	0.25819953	0.05482225	4.70975796	2.48E-06	2.31E-05
BDKRB1__BI	530.909451	-0.5568765	0.09162208	-6.0779732	1.22E-09	2.01E-08
BHLHE40	3318.14033	-0.3491991	0.044238	-7.8936458	2.93E-15	9.69E-14
BID	2738.87256	0.24877221	0.04732239	5.25696661	1.46E-07	1.73E-06
BIN1	97.3839062	-0.488319	0.1989271	-2.4547634	0.01409774	0.04573988
BIRC2	1654.03904	0.13293303	0.05444615	2.44155058	0.01462434	0.04709647
BIRC3	202.759019	-0.3697258	0.1451544	-2.5471212	0.01086157	0.03725543
BIRC5	3784.40961	0.34944368	0.04277751	8.16886505	3.11E-16	1.10E-14
BLM	2177.94277	0.12666096	0.04896263	2.58689073	0.00968463	0.03389435
BLOC1S5__B	21465.016	0.08565882	0.03170779	2.70150704	0.0069026	0.02561817

BLVRA	3611.26309	0.27279934	0.04229414	6.45005103	1.12E-10	2.14E-09
BMP6	243.784514	0.46231056	0.12975632	3.56291371	0.00036676	0.00207494
BMP7	17939.4088	0.53374052	0.03475641	15.3566083	3.20E-53	8.21E-51
BMPR1B	337.874375	0.66406535	0.10923789	6.07907544	1.21E-09	2.00E-08
BMPR2	5537.74252	0.12080879	0.03755861	3.2165401	0.00129746	0.00618509
BMS1P1	579.293688	-0.3696795	0.08752752	-4.2235799	2.40E-05	0.00017954
BNIP3	5036.81991	-0.8704427	0.03940234	-22.091144	3.85E-108	3.69E-105
BNIP3L	4561.29523	-0.4231573	0.04722962	-8.9595757	3.26E-19	1.44E-17
BOD1	7351.16937	0.15763623	0.03599067	4.37991958	1.19E-05	9.54E-05
BPTF	21614.2684	0.18691344	0.04252583	4.39529212	1.11E-05	8.96E-05
BRD3	5129.52137	0.19564876	0.0396304	4.93683593	7.94E-07	8.17E-06
BRD8	4385.1735	0.16367128	0.04195184	3.90140915	9.56E-05	0.00062524
BRI3	1801.32558	0.21951585	0.05572681	3.93914261	8.18E-05	0.0005422
BRINP3	82.8608687	2.28481964	0.24835571	9.19978708	3.59E-20	1.67E-18
BRPF3	3707.81673	0.21498809	0.0429973	5.00003692	5.73E-07	6.09E-06
BSCL2__HNR	18211.439	0.17810503	0.03378179	5.27222074	1.35E-07	1.60E-06
BSDC1	3149.98573	0.19082005	0.04607129	4.14184328	3.45E-05	0.00024998
BSPRY	2208.52422	-0.2402039	0.05158983	-4.6560327	3.22E-06	2.93E-05
BTAF1	3600.75939	-0.3466963	0.05398102	-6.4225587	1.34E-10	2.54E-09
BTBD10	1666.6262	0.18732656	0.06015586	3.11401992	0.00184557	0.00836493
BTBD2	4673.40968	0.24476775	0.05107627	4.79220061	1.65E-06	1.59E-05
BTG2	2428.9314	0.91125981	0.04903274	18.5847225	4.27E-77	1.95E-74
BTN2A2	355.257308	-0.4826411	0.11042464	-4.370774	1.24E-05	9.90E-05
BTN3A1	119.655995	-0.5439425	0.18535019	-2.9346745	0.00333898	0.01382446
BUB1	3877.16767	0.35012421	0.04527821	7.7327313	1.05E-14	3.23E-13
BUB1B__PAI	2704.28947	0.2155286	0.04760276	4.52764961	5.96E-06	5.15E-05
BUB3	11531.7227	-0.1965049	0.03480183	-5.646396	1.64E-08	2.29E-07
BX004987.4	74.9308885	-0.9620527	0.23235286	-4.1404815	3.47E-05	0.00025117
BYSL	1692.29066	0.26672564	0.05573079	4.78596533	1.70E-06	1.63E-05
C10orf2	1775.55992	0.18172585	0.05438443	3.34150526	0.00083325	0.00420525
C10orf82	129.110821	0.50837949	0.171129	2.97073842	0.00297085	0.01252495
C11orf30	2998.16476	0.23355447	0.04849091	4.81645932	1.46E-06	1.42E-05
C11orf31__C	15138.5363	0.19743034	0.03108651	6.35099793	2.14E-10	3.93E-09
C11orf45	124.48057	0.62135908	0.18353043	3.38559151	0.00071025	0.0036646
C11orf72	134.293179	-0.4425988	0.17070677	-2.5927433	0.00952138	0.03342566
C11orf80__R	3828.15581	-0.1754289	0.04699595	-3.7328515	0.00018932	0.00115187
C11orf84	1802.36129	0.41383593	0.05732474	7.21915082	5.23E-13	1.32E-11
C11orf91__C	6215.33396	-0.3942234	0.03861268	-10.209689	1.79E-24	1.11E-22
C12orf49	5063.70625	0.42176586	0.03899969	10.8145958	2.94E-27	2.18E-25
C12orf65	1346.17823	0.15648433	0.06188592	2.52859349	0.01145206	0.03881575
C12orf66	1101.80314	0.22567267	0.06715731	3.36035912	0.00077841	0.0039769
C14orf119	3597.25834	0.1084426	0.04316558	2.51224695	0.01199651	0.04025476
C14orf28	445.659791	-0.3305402	0.09476347	-3.4880555	0.00048655	0.00265381

C14orf37	3103.27389	-0.5022186	0.04780866	-10.504763	8.21E-26	5.63E-24
C15orf39	2078.52451	0.18491266	0.05516811	3.35180347	0.00080287	0.00407982
C15orf40	1598.42197	-0.1883612	0.06577767	-2.8636034	0.00418852	0.01677166
C15orf48	397.70547	-0.467285	0.10938981	-4.2717415	1.94E-05	0.00014853
C15orf59	65.8015912	-1.3821606	0.24996478	-5.5294214	3.21E-08	4.23E-07
C16orf70	1060.39758	0.20206677	0.06881751	2.93626955	0.00332186	0.01375982
C17orf67	219.527531	0.53037154	0.14015631	3.78414305	0.00015424	0.00096085
C17orf80	1772.23991	0.18096815	0.05322689	3.39993871	0.00067401	0.00350106
C17orf82	351.503605	0.60318604	0.11471096	5.25831198	1.45E-07	1.71E-06
C19orf12	873.384496	0.18081244	0.07181783	2.51765387	0.01181394	0.03980327
C19orf25	733.175477	0.30985684	0.08031153	3.85818648	0.00011423	0.00073341
C19orf48	6000.39109	0.23101115	0.04328471	5.33701493	9.45E-08	1.15E-06
C19orf66	648.168271	-0.4705619	0.08425865	-5.5847312	2.34E-08	3.18E-07
C1QTNF6	5546.76402	0.27500473	0.04310328	6.38013476	1.77E-10	3.30E-09
C1QTNF9__F	6751.77105	-0.4159647	0.03928008	-10.589709	3.33E-26	2.35E-24
C1orf132__M	226.389228	-0.5881845	0.14222941	-4.1354635	3.54E-05	0.00025591
C1orf186	13.248723	-2.2133501	0.61284068	-3.6116239	0.00030429	0.00176194
C1orf21	3446.24086	-0.4240895	0.04337867	-9.7764533	1.42E-22	7.73E-21
C1orf228	58.8978533	-1.2428256	0.27166962	-4.5747683	4.77E-06	4.21E-05
C1orf43	12752.7928	0.19997998	0.03329631	6.00606986	1.90E-09	3.09E-08
C21orf58	1650.71052	-0.35953	0.05519865	-6.5133841	7.35E-11	1.45E-09
C22orf39__H	3531.38055	0.29089856	0.04545564	6.39961384	1.56E-10	2.93E-09
C2CD2	775.184952	0.68705276	0.07449395	9.22293375	2.89E-20	1.37E-18
C2CD5	2309.27786	0.14091578	0.05027554	2.8028694	0.00506502	0.01974675
C2orf61__CA	31730.4534	-0.2417914	0.03890067	-6.2156099	5.11E-10	8.91E-09
C3orf62__US	2990.93047	0.15410185	0.04840267	3.18374727	0.00145382	0.0068063
C4A__STK19	911.967625	-0.1921558	0.07255137	-2.6485485	0.00808382	0.02922293
C4orf3	4321.21041	-0.4275037	0.05731655	-7.4586432	8.74E-14	2.42E-12
C4orf47	18.821567	-2.0303515	0.49512297	-4.1007015	4.12E-05	0.00029315
C5orf30	1760.09525	-0.2313524	0.05456714	-4.2397745	2.24E-05	0.00016907
C5orf38	981.72597	-0.201415	0.07922407	-2.5423464	0.0110111	0.03766205
C6orf141	7401.18485	-0.6097111	0.03591205	-16.977899	1.20E-64	4.28E-62
C6orf62	12526.7264	-0.1161229	0.03738765	-3.1059168	0.0018969	0.00855717
C7orf50	6911.4472	0.14705279	0.0402231	3.65592896	0.00025625	0.00151214
C7orf61__TS	3868.19657	0.15249461	0.05548068	2.74860742	0.0059849	0.02268317
C8orf88	747.230192	-1.1862233	0.08363859	-14.182728	1.17E-45	2.12E-43
C9orf106	273.735659	-1.1935292	0.13515453	-8.8308489	1.04E-18	4.38E-17
C9orf152	349.86233	-0.4672164	0.11310499	-4.1308206	3.61E-05	0.00026041
C9orf16	2811.98426	0.3532922	0.04802225	7.35684342	1.88E-13	5.00E-12
C9orf172__M	5549.60521	-0.1254615	0.03940707	-3.1837297	0.00145391	0.0068063
C9orf173	34.3129286	-1.4046183	0.35038417	-4.0087948	6.10E-05	0.00041632
C9orf173-AS	36.9537428	-1.5379336	0.34228866	-4.4930897	7.02E-06	5.95E-05
C9orf40	851.926553	0.25972539	0.07411395	3.50440621	0.00045763	0.00251943

C9orf47__S1	1778.95262	-0.881105	0.05527386	-15.940719	3.31E-57	9.57E-55
C9orf91	1838.44752	0.17445864	0.05268297	3.31148071	0.00092804	0.0046184
CA12	9050.98952	-0.6014816	0.03347769	-17.966641	3.56E-72	1.51E-69
CA2	2993.89844	-0.8101161	0.04508778	-17.967532	3.50E-72	1.51E-69
CA5B	888.045625	0.19396449	0.07263304	2.67047175	0.00757447	0.0276946
CA9	73.0159257	-4.521326	0.39483038	-11.451312	2.32E-30	2.02E-28
CABIN1	4004.60619	0.16106641	0.046135	3.4911974	0.00048086	0.00262752
CACNA1H	5212.46418	0.23232849	0.0530444	4.37988704	1.19E-05	9.54E-05
CACNG6	93.6814629	0.73618493	0.20173926	3.64919023	0.00026307	0.00154785
CAD	7479.03395	0.2223991	0.04761669	4.67061229	3.00E-06	2.75E-05
CADM4	524.888545	0.25896809	0.08907587	2.9072756	0.00364592	0.01489908
CADPS2	1176.6943	0.40047313	0.0642821	6.22993232	4.67E-10	8.17E-09
CALCOCO1	2184.75096	-0.2680908	0.05044154	-5.3148815	1.07E-07	1.29E-06
CALCR	6574.63498	-0.2861371	0.03680506	-7.7743952	7.58E-15	2.35E-13
CALHM2	1853.5077	0.30050012	0.05640967	5.32710346	9.98E-08	1.21E-06
CALM1	30069.1071	-0.2285352	0.03205702	-7.12902	1.01E-12	2.46E-11
CALM3	13043.3944	0.23639316	0.03395544	6.96186504	3.36E-12	7.66E-11
CALML3-AS1	152.989733	-0.8019179	0.1651403	-4.8559792	1.20E-06	1.19E-05
CALML5	1847.87184	-0.7014785	0.05400191	-12.989883	1.40E-38	1.83E-36
CALR	29252.5151	0.1471997	0.03513882	4.18909109	2.80E-05	0.00020651
CALR3__CHE	16127.9088	0.08608317	0.0329504	2.61250802	0.00898806	0.03185869
CAMK1D	697.11482	0.34207005	0.07966063	4.29409172	1.75E-05	0.00013616
CAMK2B	706.605844	-0.725513	0.08264677	-8.7784798	1.66E-18	6.91E-17
CAMK2N1	1905.04159	-0.3901215	0.05401223	-7.2228376	5.09E-13	1.28E-11
CAMKK1	267.746473	-0.5211432	0.12968185	-4.0186286	5.85E-05	0.00040127
CAMSAP1	4786.65854	0.13875652	0.04158184	3.33695027	0.00084703	0.00426298
CAMSAP2	6940.24974	0.13974788	0.04296923	3.25227806	0.00114484	0.00553127
CAND1	17010.1034	0.10748827	0.03409558	3.15255737	0.00161847	0.00747352
CANT1	6481.44114	0.15942405	0.03747327	4.25434109	2.10E-05	0.00015949
CANX	52052.2039	0.07979665	0.03210175	2.48574138	0.01292819	0.04269744
CAP1	11058.2912	0.12016086	0.03439238	3.49382265	0.00047616	0.00260415
CAPN1	14368.2304	0.1641806	0.04577627	3.58658765	0.00033503	0.00191565
CAPN2	3731.9717	-0.4131145	0.0436031	-9.474428	2.68E-21	1.34E-19
CAPN5	539.087752	-0.2861175	0.08912322	-3.2103585	0.0013257	0.00630155
CAPN8	620.00271	-0.9336474	0.08451036	-11.047727	2.25E-28	1.74E-26
CAPN9	460.105976	-0.2586045	0.10013202	-2.582635	0.0098049	0.03424297
CAPRIN1	17113.4508	0.12159828	0.03330377	3.65118659	0.00026103	0.00153785
CARD14	428.670303	-0.3466389	0.09746507	-3.5565446	0.00037577	0.00211864
CARD19	986.608892	-0.2006557	0.07069046	-2.8385121	0.00453244	0.01794279
CARF	608.642151	-0.4754328	0.08383239	-5.6712305	1.42E-08	2.00E-07
CARNS1	87.1197188	-1.353987	0.21654412	-6.2527075	4.03E-10	7.12E-09
CASC5	2244.34087	0.22099245	0.05395333	4.09599267	4.20E-05	0.00029811
CASK	3580.16097	-0.2524234	0.04330515	-5.8289457	5.58E-09	8.49E-08

CASP14	58.5876831	-1.0706468	0.27765375	-3.85605	0.00011523	0.00073881
CASP6	1308.40053	0.31137879	0.06245082	4.98598449	6.16E-07	6.51E-06
CAST	4496.77627	-0.4073073	0.03917909	-10.396037	2.58E-25	1.72E-23
CASZ1	1744.19497	0.18341378	0.05950263	3.08244817	0.00205306	0.00913504
CAT	2246.64302	0.41663616	0.05034511	8.2756039	1.28E-16	4.62E-15
CATSPER2	220.791814	-0.720746	0.13561487	-5.314653	1.07E-07	1.29E-06
CATSPERG	241.570603	-0.6490967	0.13163277	-4.931118	8.18E-07	8.39E-06
CAV2	1426.0399	-0.203873	0.05884625	-3.4645029	0.00053121	0.00286064
CBFB	3355.87433	0.17633518	0.04894695	3.6025777	0.00031508	0.00181406
CBL	2746.12115	0.24960889	0.05457773	4.5734569	4.80E-06	4.23E-05
CBLL1	2828.54707	0.21804097	0.05207872	4.1867577	2.83E-05	0.00020847
CBS	360.042818	0.64241014	0.10814546	5.94024152	2.85E-09	4.52E-08
CBWD1	1067.98303	-0.3431581	0.06483912	-5.2924544	1.21E-07	1.45E-06
CBWD5	492.240541	-0.5148961	0.10240238	-5.0281656	4.95E-07	5.33E-06
CBX1	14323.0685	0.27106346	0.04000375	6.77595086	1.24E-11	2.67E-10
CBX2	4689.42694	0.25794107	0.04402117	5.85947843	4.64E-09	7.13E-08
CBX6	4957.72754	0.1261737	0.03960156	3.18607876	0.00144215	0.00676373
CC2D2A	607.165585	0.28540393	0.08277172	3.44808528	0.00056458	0.00301096
CCAR2	4118.93765	0.19898503	0.04024815	4.94395506	7.66E-07	7.91E-06
CCBL1	184.802468	-0.4651274	0.15454185	-3.0097178	0.00261491	0.01123473
CCDC102A	357.049816	0.4545865	0.1150319	3.95182987	7.76E-05	0.00051668
CCDC106__Z	3808.38286	0.19291982	0.04740929	4.06924078	4.72E-05	0.00033028
CCDC114	76.0547463	-1.0516695	0.23806555	-4.4175626	9.98E-06	8.15E-05
CCDC117	5258.58314	0.27156255	0.04142488	6.55554176	5.54E-11	1.11E-09
CCDC120	1298.90094	-0.1905589	0.06429456	-2.9638416	0.00303825	0.01275602
CCDC125	2284.37559	-0.433593	0.05791339	-7.486921	7.05E-14	1.96E-12
CCDC130	979.785466	-0.2098417	0.06986614	-3.0034824	0.00266909	0.01144057
CCDC136	195.067503	0.36972791	0.14126414	2.61728067	0.00886334	0.03150374
CCDC137	2580.54913	0.24346047	0.04906005	4.96249989	6.96E-07	7.26E-06
CCDC14	4451.11767	-0.2085088	0.04387869	-4.7519371	2.01E-06	1.91E-05
CCDC146	408.807494	-0.3875536	0.11058307	-3.5046381	0.00045723	0.002518
CCDC162P	172.075142	-0.5786584	0.15275162	-3.7882309	0.00015172	0.00094647
CCDC180	262.408563	-0.3437832	0.13112012	-2.6218951	0.00874423	0.03114717
CCDC183-AS	197.809575	-0.3601411	0.14023933	-2.5680464	0.01022735	0.03545351
CCDC191	541.551014	-0.4629503	0.09395912	-4.9271454	8.34E-07	8.55E-06
CCDC57	1622.28657	-0.2800306	0.0630507	-4.4413554	8.94E-06	7.39E-05
CCDC64B	2024.09047	-0.2559249	0.05401692	-4.737865	2.16E-06	2.04E-05
CCDC69	392.506489	0.61818014	0.10255403	6.02784851	1.66E-09	2.72E-08
CCDC71	1069.78817	0.24217318	0.06820631	3.55059805	0.00038436	0.00215843
CCDC74A	820.341789	0.33064767	0.07686839	4.30147768	1.70E-05	0.00013215
CCDC77	381.768754	0.49237558	0.10354521	4.75517498	1.98E-06	1.88E-05
CCDC84	901.725766	-0.8392287	0.08275497	-10.141128	3.63E-24	2.19E-22
CCDC85C	8610.35786	0.09011568	0.03584661	2.51392481	0.01193959	0.04012283

CCDC88C	4964.40074	0.50023028	0.04278437	11.691892	1.40E-31	1.32E-29
CCDC89	45.3966445	-1.0189334	0.31912687	-3.1928787	0.00140862	0.00663005
CCDC90B	2623.55576	0.32275706	0.05072308	6.36312026	1.98E-10	3.66E-09
CCER2	22.292466	-1.8320977	0.4532769	-4.0418951	5.30E-05	0.00036676
CCL22	24.263414	-2.2026568	0.45069727	-4.8872202	1.02E-06	1.03E-05
CCNA2	3587.14037	0.19937844	0.04422925	4.50784154	6.55E-06	5.60E-05
CCNB1	9036.50766	0.32992485	0.03613944	9.12921755	6.90E-20	3.16E-18
CCNB2	3456.16261	0.35746784	0.04726438	7.56315569	3.93E-14	1.14E-12
CCNF	2156.359	0.24785025	0.05540708	4.47325998	7.70E-06	6.48E-05
CCNK	3549.33022	0.11624209	0.04582191	2.53682358	0.01118633	0.03808993
CCNL1	7033.19757	-0.5782322	0.03560722	-16.239182	2.66E-59	8.30E-57
CCNL2	8293.48512	-0.4946312	0.03558359	-13.900545	6.29E-44	1.04E-41
CCNT1	3833.60074	0.1624176	0.04574376	3.55059595	0.00038436	0.00215843
CCNYL1	1191.97958	-0.4048164	0.06389457	-6.3356931	2.36E-10	4.33E-09
CCPG1__DYX	4728.59392	-0.1617719	0.0396393	-4.0810993	4.48E-05	0.00031568
CCT2	18775.1914	0.10612065	0.03495315	3.03608237	0.00239674	0.01042016
CCT3	28081.0404	0.09061008	0.03069122	2.95231324	0.00315403	0.01317704
CCT5	22299.0996	0.08422738	0.03347024	2.51648598	0.01185316	0.03990594
CCT6P3__RP	685.750629	-0.4790857	0.08109982	-5.9073591	3.48E-09	5.45E-08
CCT7	22684.2754	0.15447227	0.03158131	4.89125582	1.00E-06	1.01E-05
CD109	967.825345	-0.5209039	0.07016562	-7.4239195	1.14E-13	3.10E-12
CD22	56.8428702	-1.0390155	0.26582113	-3.9087018	9.28E-05	0.00060929
CD24	55070.2915	-0.268138	0.03154386	-8.5004797	1.89E-17	7.26E-16
CD276	8490.30211	0.26984128	0.04102392	6.57765661	4.78E-11	9.65E-10
CD320__CTD	1573.26201	0.25092321	0.06202068	4.04579912	5.21E-05	0.00036153
CD44	7000.58795	-0.2176919	0.03557679	-6.1189306	9.42E-10	1.59E-08
CD46	6849.78533	-0.169059	0.03619877	-4.670297	3.01E-06	2.75E-05
CD47	6682.06225	-0.463277	0.04020626	-11.52251	1.02E-30	9.17E-29
CD58	700.244421	-0.3658607	0.08276417	-4.4205207	9.85E-06	8.05E-05
CD81	7749.46266	-0.1243075	0.04387121	-2.8334633	0.00460466	0.01820498
CDC14A	722.134872	0.71336448	0.08007348	8.90887315	5.16E-19	2.23E-17
CDC20	3806.72511	0.27454067	0.05144461	5.33662711	9.47E-08	1.15E-06
CDC25C	833.400203	0.4586356	0.07505879	6.11035176	9.94E-10	1.66E-08
CDC27	4993.96757	0.16725796	0.03902968	4.28540398	1.82E-05	0.00014071
CDC42BPB	14320.598	0.1066113	0.0436311	2.44347046	0.01454676	0.04689633
CDC42EP4	2974.25664	0.23014693	0.04807981	4.78676888	1.69E-06	1.63E-05
CDCA5	4030.12308	0.18279145	0.04235078	4.31612926	1.59E-05	0.00012437
CDCA7	591.180914	-0.507307	0.08426696	-6.0202363	1.74E-09	2.84E-08
CDCA8	2420.98424	0.33799934	0.04869753	6.94079017	3.90E-12	8.79E-11
CDK10	3263.18194	-0.335991	0.04633159	-7.2518767	4.11E-13	1.05E-11
CDK14__CLD	3839.88018	0.20487502	0.04218793	4.85624727	1.20E-06	1.19E-05
CDK18	1966.23953	0.20586857	0.06161193	3.34137483	0.00083365	0.00420606
CDK19	3135.5931	0.17653706	0.04457658	3.9603093	7.49E-05	0.00050216

CDK2	4361.57333	0.13927675	0.04025992	3.45943919	0.0005413	0.00290896
CDK2AP1	4958.33872	0.23098181	0.05393023	4.28297439	1.84E-05	0.000142
CDK4	11383.2581	0.26788091	0.03444554	7.77694001	7.43E-15	2.32E-13
CDK5RAP3__	16313.3141	-0.252242	0.03222656	-7.8271456	4.99E-15	1.60E-13
CDK9	3194.43546	0.20124675	0.0467521	4.30455029	1.67E-05	0.00013055
CDKL1	138.536704	-0.7452847	0.17587032	-4.2376947	2.26E-05	0.00017022
CDKN1B	6099.07779	0.3036416	0.03786602	8.01884201	1.07E-15	3.67E-14
CDON	628.648478	0.24377071	0.0825895	2.95159461	0.00316138	0.01320306
CDRT1__RP1	2816.3532	-0.5615794	0.04672677	-12.018365	2.85E-33	2.88E-31
CDRT4__TVP	503.973743	-0.3420391	0.08976327	-3.8104571	0.00013871	0.00087517
CDYL	2760.84112	0.28617414	0.04732629	6.04683213	1.48E-09	2.43E-08
CEBPG	2394.94128	0.19747469	0.04994542	3.95380971	7.69E-05	0.00051298
CELF6__HEX/	12110.5253	-0.0969501	0.0339964	-2.8517747	0.00434759	0.01728608
CEMP1	484.657235	-0.2899165	0.10757295	-2.6950686	0.00703741	0.026039
CENPE	2799.27913	0.25467207	0.04744145	5.36813437	7.96E-08	9.77E-07
CENPF	5208.13001	0.31469175	0.04920159	6.39596663	1.60E-10	2.99E-09
CENPM	855.849694	0.38325928	0.07618971	5.03032879	4.90E-07	5.28E-06
CENPN	3298.66579	0.17226653	0.04600298	3.74468193	0.00018062	0.00110482
CENPT	1911.37858	-0.3146551	0.05355233	-5.8756563	4.21E-09	6.52E-08
CEP126	292.724736	0.36335066	0.1181408	3.075573	0.00210099	0.00932456
CEP152	1202.50716	0.16326762	0.06337115	2.57637156	0.00998433	0.03478396
CEP162	620.223364	0.28582334	0.08161743	3.50198918	0.0004618	0.00253628
CEP164P1__I	332.369093	0.49390495	0.11019029	4.48229094	7.38E-06	6.24E-05
CEP170	975.674985	0.19372331	0.06908901	2.80396708	0.00504781	0.01969228
CEP170B	6430.30023	-0.2433	0.05363863	-4.5359107	5.74E-06	4.98E-05
CEP19	123.011639	0.66851579	0.17865472	3.74194297	0.0001826	0.00111506
CEP290	1550.28447	-0.2107311	0.05639789	-3.7365076	0.00018659	0.00113753
CEP295	1104.38845	0.19328281	0.07210387	2.68061622	0.00734867	0.02695549
CEP55	3561.82825	0.17457437	0.04709725	3.70667842	0.00021	0.00126455
CEP95	4136.75812	-0.4014881	0.04085502	-9.8271434	8.60E-23	4.83E-21
CEP97	1072.03119	0.24653881	0.06548731	3.76468075	0.00016676	0.00102797
CERS1	759.937959	0.27422849	0.08818862	3.10956777	0.00187361	0.0084724
CERS2	20269.201	0.23698733	0.03242988	7.30768441	2.72E-13	7.07E-12
CERS6	2825.29144	0.27188075	0.05575181	4.87662668	1.08E-06	1.08E-05
CFAP44	1984.8414	-0.4409873	0.05301009	-8.3189312	8.88E-17	3.25E-15
CFAP97	1446.18568	0.1943595	0.05864631	3.31409588	0.0009194	0.00457917
CFL1	47966.3458	0.14127353	0.03301645	4.27888339	1.88E-05	0.00014433
CFL2	833.844135	-0.1846794	0.0728121	-2.5363839	0.01120039	0.03813068
CFLAR	2871.58194	-0.1474102	0.05114139	-2.8824052	0.00394652	0.01596646
CGA	23.3926389	-1.829294	0.46866691	-3.9031857	9.49E-05	0.00062178
CGGBP1	6578.96167	0.12563313	0.03622512	3.46812175	0.00052411	0.00282824
CGN	4631.81192	-0.2791966	0.04144033	-6.7373168	1.61E-11	3.44E-10
CH17-140K2⁄	2122.47918	0.24757665	0.0546568	4.52965896	5.91E-06	5.10E-05

CHCHD2	9698.42676	0.26455564	0.03443895	7.68187297	1.57E-14	4.74E-13
CHD3	14328.9343	0.11198976	0.03414451	3.27987622	0.00103853	0.00507137
CHD4	21475.7372	0.21507768	0.03772052	5.70187433	1.18E-08	1.70E-07
CHD5	659.307053	0.24792337	0.0847931	2.92386258	0.00345717	0.0142234
CHEK1__STT:	7869.16192	0.2192588	0.03607462	6.0779247	1.22E-09	2.01E-08
CHKB__CHKE	1500.46794	-0.6793069	0.05977456	-11.364483	6.28E-30	5.33E-28
CHMP5	3877.08608	-0.1409492	0.04328732	-3.2561306	0.00112942	0.00546255
CHMP7	1956.64828	0.23922333	0.05261662	4.54653523	5.45E-06	4.76E-05
CHN1	630.976572	0.22547284	0.08362843	2.69612664	0.0070151	0.0259828
CHN2	108.874918	-0.6796361	0.1909253	-3.5596966	0.00037128	0.00209597
CHP1	4456.94573	0.15011817	0.03974865	3.7766859	0.00015893	0.00098535
CHPT1	1808.87725	-0.1947118	0.05791331	-3.3621256	0.00077345	0.00395865
CHRNA10__I	4086.92437	0.12470673	0.0433885	2.87418872	0.00405067	0.01631902
CHRNA5	835.109418	0.2590849	0.07589895	3.41355041	0.00064122	0.00335882
CHST10	615.100953	0.31963758	0.09033389	3.53840148	0.00040256	0.00224472
CHST14	1053.0686	0.26912175	0.07345216	3.66390536	0.0002484	0.00147199
CHTF18	2559.38737	-0.2526509	0.05648047	-4.4732428	7.70E-06	6.48E-05
CHTF8	7619.59042	0.41335024	0.04575147	9.03468694	1.64E-19	7.35E-18
CIAPIN1	2379.50362	0.20182844	0.05349705	3.77270198	0.00016149	0.00099918
CIART	669.227899	-0.307812	0.08096353	-3.80186	0.00014361	0.00090299
CICP14	391.715641	-0.3762905	0.11216834	-3.3546942	0.00079453	0.00404692
CICP27	274.049525	-0.4434269	0.13379952	-3.314114	0.00091934	0.00457917
CILP	19.5487083	-1.2246343	0.45227287	-2.7077332	0.00677445	0.02522983
CILP2	251.090456	0.58701996	0.12825989	4.57680076	4.72E-06	4.17E-05
CIRBP	6100.59651	-0.1179102	0.04745007	-2.4849329	0.01295759	0.04275966
CIRH1A	4355.9708	0.11167878	0.04203449	2.65683653	0.00788777	0.0286277
CISD3	672.694596	0.22651645	0.08535996	2.65366165	0.00796236	0.0288697
CIT	3289.37558	0.24338056	0.04858355	5.00952634	5.46E-07	5.81E-06
CITED1	74.311143	1.97206057	0.2581192	7.64011572	2.17E-14	6.48E-13
CKAP2	5033.35455	0.24478195	0.03872479	6.32106544	2.60E-10	4.73E-09
CKAP2L	998.177645	0.29770116	0.06837656	4.35384787	1.34E-05	0.0001061
CKAP5	14365.6756	0.30467785	0.03867399	7.8781082	3.32E-15	1.09E-13
CKMT2-AS1	332.302698	-0.3797864	0.1205219	-3.1511818	0.00162611	0.00750325
CKS1B	3907.727	0.15253913	0.04223068	3.61204527	0.00030379	0.00175964
CLASRP	1657.41317	-0.2315735	0.05669936	-4.0842354	4.42E-05	0.00031193
CLCN2	397.059037	-0.380585	0.10070541	-3.779191	0.00015734	0.00097782
CLDN1	48.1906727	-3.3751445	0.37996932	-8.8826764	6.53E-19	2.81E-17
CLEC2D	419.471409	-0.5773724	0.11868361	-4.8648036	1.15E-06	1.14E-05
CLIC4	9225.31416	0.08459159	0.03476931	2.43293859	0.01497684	0.04803654
CLIC6	90.7530682	-0.5746597	0.20970056	-2.7403824	0.00613677	0.0231481
CLINT1	6380.21025	0.10349757	0.03868515	2.67538222	0.00746441	0.02732504
CLIP3	225.995998	-0.5712774	0.13595064	-4.2020943	2.64E-05	0.0001961
CLK1	1854.61533	-0.3135416	0.05341363	-5.8700678	4.36E-09	6.74E-08

CLK2	2866.58255	-0.2312583	0.04708366	-4.9116455	9.03E-07	9.21E-06
CLNS1A	5828.17929	0.10253322	0.04051938	2.53047355	0.01139087	0.03867804
CLPTM1	9336.78937	0.13387135	0.04347186	3.07949425	0.00207352	0.00921488
CLSTN2	1291.39701	-0.5733086	0.06939625	-8.2613771	1.44E-16	5.19E-15
CLSTN3	1163.9187	0.23219352	0.06404938	3.62522642	0.00028871	0.00168243
CLTB	4628.59876	-0.2077641	0.04147233	-5.0097041	5.45E-07	5.81E-06
CLU	9064.21334	-0.5884692	0.0364312	-16.152891	1.08E-58	3.29E-56
CLUHP3	1210.26071	-0.2399155	0.06420463	-3.7367323	0.00018643	0.00113689
CMB9-22P13	93.4597049	-0.6574135	0.2121767	-3.0984244	0.00194553	0.00873117
CMC2	2963.62851	-0.2413079	0.05178893	-4.6594484	3.17E-06	2.89E-05
CMIP	3559.98498	0.13287241	0.04478462	2.96692043	0.00300799	0.01265228
CMTM3	340.321443	0.96021418	0.11420456	8.40784427	4.18E-17	1.57E-15
CMTM4	5443.649	0.25440541	0.04451956	5.71446418	1.10E-08	1.59E-07
CMTM6	5611.25989	0.21000462	0.045858	4.57945425	4.66E-06	4.13E-05
CMTR2	3116.11874	0.1232114	0.04883105	2.52321815	0.01162862	0.03928781
CMYA5	1092.28527	-0.3305426	0.08474377	-3.9004941	9.60E-05	0.00062694
CNBP	18317.7184	-0.0918426	0.0355116	-2.5862698	0.00970209	0.03394895
CNDP2	4419.58579	0.11688352	0.04021665	2.90634668	0.00365676	0.01493669
CNGB3	26.100393	1.27044761	0.39745827	3.19643018	0.0013914	0.00656422
CNIH2	407.265083	0.75775446	0.1015556	7.46147401	8.56E-14	2.37E-12
CNKSR1	1007.64232	-0.222401	0.06789822	-3.2755059	0.00105473	0.00513673
CNN2	1362.48589	0.37615997	0.06112086	6.15436321	7.54E-10	1.29E-08
CNNM4	3221.10002	0.12730527	0.04481436	2.84072492	0.00450111	0.01783814
CNOT1	13213.8142	0.17059767	0.03867617	4.4109246	1.03E-05	8.38E-05
CNOT3	4522.56424	0.13205507	0.04693269	2.81371224	0.0048973	0.01917489
CNOT4	1894.70434	0.25608048	0.05911843	4.33165186	1.48E-05	0.00011637
CNOT6	5962.70535	0.10195056	0.03765728	2.70732639	0.00678275	0.02524018
CNP	2277.85415	0.34214695	0.05027087	6.80606827	1.00E-11	2.18E-10
CNTNAP2	81.836687	1.06561206	0.22986817	4.63575298	3.56E-06	3.21E-05
COA7	1981.86147	0.15319889	0.05109189	2.99849707	0.00271315	0.0115967
COBL	2791.08766	0.31425325	0.05047269	6.22620363	4.78E-10	8.35E-09
COCH	467.337123	0.80603469	0.09837667	8.19335234	2.54E-16	9.01E-15
COG4	5719.05203	0.1983282	0.04336486	4.57347769	4.80E-06	4.23E-05
COG8__PDF_	6711.41158	0.11901466	0.0408361	2.91444698	0.00356319	0.01459045
COL11A2	304.855646	-1.2455161	0.1213828	-10.261059	1.06E-24	6.59E-23
COL12A1	5501.83484	0.16569511	0.05288386	3.13318863	0.00172918	0.00790422
COL13A1	25.4787812	-1.4538983	0.41204784	-3.5284698	0.00041797	0.00231931
COL18A1	11976.3497	-0.4259701	0.051789	-8.2251074	1.95E-16	6.96E-15
COL1A1	1552.97254	0.17747256	0.06532248	2.71686817	0.00659029	0.02460931
COL21A1	421.664639	0.4838182	0.10479456	4.61682556	3.90E-06	3.49E-05
COL27A1	857.556609	-1.0125972	0.07765368	-13.039913	7.25E-39	9.73E-37
COL4A5	5046.32553	-0.4144988	0.04139936	-10.012202	1.35E-23	7.87E-22
COL4A6	569.216364	-0.2701383	0.0887695	-3.043143	0.00234121	0.01021527

COL5A1	5223.44616	-0.6321056	0.04445817	-14.217986	7.09E-46	1.29E-43
COL5A3	48.4847396	-0.9629915	0.3049265	-3.1581104	0.00158795	0.00735079
COL6A1	437.753294	-0.3355586	0.09806141	-3.4219236	0.0006218	0.00327306
COL6A2	1478.88689	-0.4874105	0.06492744	-7.5070033	6.05E-14	1.70E-12
COL7A1	332.775049	-1.6408489	0.12494665	-13.132396	2.15E-39	2.97E-37
COL9A3	395.237199	1.50762637	0.11058601	13.6330663	2.55E-42	4.07E-40
COPB1__PSN	15075.7507	0.0866186	0.03502038	2.47337698	0.01338429	0.04389372
COQ2	1097.82102	0.19344777	0.06523849	2.9652397	0.00302447	0.01270989
COQ4	2817.81092	-0.1609317	0.04597712	-3.5002555	0.00046481	0.00255129
COQ9__POLI	4954.67461	0.13227238	0.04276914	3.09270635	0.0019834	0.00888345
CORO1B__P	9766.20477	0.26010127	0.04852844	5.35976998	8.33E-08	1.02E-06
CORO1C	4630.49973	0.18409203	0.04063189	4.5307277	5.88E-06	5.08E-05
COX10	1077.41234	0.24607674	0.0661238	3.72145519	0.00019808	0.00119952
CP	572.012244	-0.2635463	0.08569531	-3.0753871	0.0021023	0.00932456
CPA6	56.9391746	-0.7073889	0.27150335	-2.6054517	0.00917532	0.03238537
CPAMD8	246.645395	-0.7040777	0.12986198	-5.4217388	5.90E-08	7.42E-07
CPNE1__NFS	13110.3665	0.12687986	0.03410994	3.71973293	0.00019943	0.00120693
CPNE2	1211.27133	0.32432758	0.06768194	4.79193696	1.65E-06	1.59E-05
CPS1	199.551947	-0.5499242	0.14051102	-3.9137445	9.09E-05	0.00059756
CPT2	1282.00805	0.28153291	0.06708726	4.19651844	2.71E-05	0.00020066
CPXM1	15.3357198	-1.5527821	0.53179094	-2.9199109	0.00350132	0.01437905
CRABP2	9018.49339	-0.3905451	0.03766135	-10.36992	3.40E-25	2.21E-23
CRACR2A	622.489526	0.3049872	0.08788632	3.47024655	0.00051998	0.00280762
CRACR2B	491.297972	-0.3333622	0.1108191	-3.0081657	0.0026283	0.01128429
CRAMP1__HI	17977.9146	0.08390941	0.03172488	2.64490841	0.0081713	0.02946895
CREB3L1	429.059736	0.63229942	0.09771689	6.47072819	9.75E-11	1.88E-09
CREBBP	7663.91574	0.17686495	0.04022607	4.39677389	1.10E-05	8.91E-05
CREBL2	2832.85757	0.34708632	0.04655697	7.45508799	8.98E-14	2.48E-12
CREBZF	7071.82304	-0.4842386	0.03606342	-13.427418	4.18E-41	6.19E-39
CREG1	3989.61277	0.16214987	0.04935648	3.28527993	0.00101881	0.0049965
CREG2	367.083501	-0.5375296	0.11309097	-4.753073	2.00E-06	1.90E-05
CRIM1	1458.00056	-0.237433	0.0594038	-3.9969321	6.42E-05	0.00043561
CRIP3__ZNF:	2417.29931	0.12568078	0.05061822	2.48291573	0.01303119	0.04295982
CRISPLD2	467.245091	0.27862871	0.09340266	2.98309192	0.00285352	0.01211435
CROCC	1423.57988	-0.1575085	0.06331267	-2.4877872	0.01285406	0.04249111
CROCCP3	234.157497	-0.478554	0.13838301	-3.4581844	0.00054383	0.00292168
CROT	1956.689	-0.3178729	0.05154647	-6.1667239	6.97E-10	1.20E-08
CRTAP	9472.50553	0.25703958	0.0381808	6.73216762	1.67E-11	3.56E-10
CRTC3	2124.77891	0.13893305	0.0525298	2.64484265	0.00817289	0.02946895
CRY2	1910.15992	0.19520494	0.05414487	3.60523428	0.00031187	0.00179787
CRYZL1	615.649046	-0.2562985	0.08304092	-3.0864118	0.00202588	0.00903839
CSAD	1158.35284	-0.7793094	0.06712393	-11.610008	3.67E-31	3.39E-29
CSF1R	21.3326265	-1.1358398	0.46652033	-2.4347059	0.0149039	0.04786152

CSGALNACT1	360.127243	-0.7221308	0.10622671	-6.7980153	1.06E-11	2.30E-10
CSK	8510.57345	0.20538138	0.04219723	4.8671771	1.13E-06	1.13E-05
CSNK1A1	16757.432	-0.1021591	0.03229837	-3.1629813	0.00156162	0.00724875
CSNK1D	8958.66535	0.09106768	0.0349338	2.60686431	0.00913756	0.03229583
CSNK1G2	2748.07198	0.15973295	0.05644818	2.82972709	0.00465877	0.01839752
CSNK1G3	2663.87418	0.16667481	0.04990652	3.33974043	0.00083857	0.0042273
CSPG4P10__	189.413886	-0.3807245	0.14313685	-2.6598634	0.00781724	0.0284965
CSRP2BP__P	2148.68023	0.29248439	0.05109978	5.72378968	1.04E-08	1.51E-07
CT83	24.3661326	1.57244743	0.42678702	3.6843844	0.00022926	0.00136699
CTA-221G9.1	17.9301834	1.13102575	0.46509248	2.43182981	0.01502276	0.04814149
CTA-228A9.3	57.336083	-1.0554474	0.2675006	-3.9455889	7.96E-05	0.00052917
CTA-280A3.2	16.6716371	-1.7530968	0.52886145	-3.3148508	0.00091692	0.00457141
CTA-392C11.	40.2494705	0.93790226	0.3238811	2.89582275	0.00378166	0.01537111
CTA-392E5.1	134.499003	-1.1836275	0.18187963	-6.5077518	7.63E-11	1.50E-09
CTB-131B5.5	44.7024979	-1.7799764	0.33131563	-5.3724491	7.77E-08	9.57E-07
CTB-33O18.1	295.723491	0.60652602	0.12510388	4.84817917	1.25E-06	1.24E-05
CTB-50L17.8	116.09619	-0.4978046	0.18468738	-2.6953905	0.00703062	0.02601914
CTC-326K19.(	7010.4668	0.17028992	0.04036602	4.21864491	2.46E-05	0.00018329
CTC-429P9.3	119.548797	-0.5883619	0.19546442	-3.0100716	0.00261186	0.0112243
CTC-432M15	3883.45918	-0.1329852	0.04226941	-3.1461342	0.00165444	0.0076141
CTC-471F3.5	151.14929	-0.5544307	0.16837478	-3.2928367	0.00099182	0.00488384
CTC-490E21.	64.3989698	-0.7752901	0.24732448	-3.1347084	0.00172025	0.00787325
CTC-510F12.(	1613.24533	-0.2848998	0.07665333	-3.7167314	0.00020182	0.00121892
CTC-518B2.8	35.3783955	-0.8220269	0.33262555	-2.4713282	0.01346122	0.04413015
CTC-523E23.	211.513998	0.59609376	0.13623264	4.37555745	1.21E-05	9.70E-05
CTC-523E23.(	88.9042251	0.92556521	0.2138938	4.32721853	1.51E-05	0.00011863
CTCF	7487.636	0.22579089	0.03847164	5.86902243	4.38E-09	6.77E-08
CTD-2006C1.	1458.8243	-0.4421985	0.06364518	-6.9478711	3.71E-12	8.41E-11
CTD-2012K1(	161.013217	0.48237028	0.156298	3.08622166	0.00202718	0.00904196
CTD-2013N1	113.125784	-0.6842328	0.184758	-3.7034004	0.00021273	0.00127959
CTD-2017D1	59.5027926	-0.6229206	0.2555337	-2.4377238	0.01478006	0.04752243
CTD-2017F1	252.45035	-0.37173	0.12722715	-2.9217822	0.00348035	0.01430583
CTD-2033A1(	1878.64985	-0.4567788	0.05322586	-8.5818954	9.33E-18	3.72E-16
CTD-2132N1	9422.39863	0.18315236	0.03334803	5.49214955	3.97E-08	5.15E-07
CTD-2147F2.	105.454586	0.63148173	0.19084389	3.30889156	0.00093666	0.00465751
CTD-2192J16	7811.46284	0.26197429	0.03578478	7.32083011	2.46E-13	6.47E-12
CTD-2196E1(	67.3705872	-1.0557271	0.24061396	-4.3876385	1.15E-05	9.26E-05
CTD-2207O2	120.493666	0.62930691	0.20868848	3.01553262	0.00256528	0.01106062
CTD-2228K2.	1107.01809	-0.6824527	0.07128188	-9.5739985	1.03E-21	5.34E-20
CTD-2240E1(	47.3098035	-0.8353726	0.29241001	-2.8568536	0.00427863	0.01707893
CTD-2336O2.	109.224867	-0.6009142	0.20184127	-2.9771623	0.0029093	0.01230538
CTD-2369P2.	7070.08929	0.21138533	0.0460445	4.59089189	4.41E-06	3.92E-05
CTD-2510F5.	583.982626	0.31318591	0.09503837	3.29536301	0.00098295	0.00484931

CTD-2510F5.	12330.4081	0.2938755	0.03474563	8.45791272	2.72E-17	1.04E-15
CTD-2540F13	84.2054962	-0.7854555	0.21645541	-3.6287172	0.00028483	0.00166332
CTD-2547G2:	200.627324	-0.4898356	0.15374309	-3.1860659	0.00144222	0.00676373
CTD-2547L24	97.7848967	0.70512352	0.20097134	3.50857741	0.00045051	0.00248551
CTD-2574D2:	131.445899	-0.5207659	0.18148437	-2.8694806	0.00411147	0.01652016
CTD-2653D5.	2256.16079	-0.1461001	0.0581336	-2.5131784	0.01196488	0.0401782
CTD-3032H1:	18.2338646	-1.3029127	0.49024565	-2.6576731	0.00786822	0.02860229
CTD-3092A1:	362.816135	-0.5000202	0.1050785	-4.7585393	1.95E-06	1.86E-05
CTD-3184A7.	352.788206	-0.6790958	0.10633304	-6.3864986	1.70E-10	3.17E-09
CTD-3247F14	215.075363	-1.4647343	0.14412075	-10.163244	2.89E-24	1.75E-22
CTD-3252C9.	450.112765	-0.4044469	0.09675502	-4.1801128	2.91E-05	0.00021422
CTDP1	1061.34491	0.2488467	0.08022111	3.10201031	0.00192211	0.00866019
CTDSP1	4898.57117	0.15683823	0.0387855	4.04373325	5.26E-05	0.00036431
CTF1	52.3379818	0.85884628	0.28392131	3.02494477	0.00248679	0.01077056
CTNNA1	24819.0187	0.09551507	0.03534375	2.70245974	0.00688285	0.02555527
CTNNB1	18621.927	0.16373996	0.03118678	5.25029942	1.52E-07	1.78E-06
CTPS1	3791.50455	0.114874	0.04446413	2.58352066	0.00977976	0.03416826
CTPS2	2787.16142	0.13365921	0.04646494	2.87656045	0.00402035	0.01621838
CTSH	3050.47438	0.21599068	0.0453687	4.76078585	1.93E-06	1.84E-05
CTSK	263.324071	-0.6345331	0.1239996	-5.1172189	3.10E-07	3.46E-06
CTSL	4282.94009	-0.2445616	0.04842345	-5.0504787	4.41E-07	4.79E-06
CTSZ	352.46018	0.31854788	0.11385801	2.7977644	0.00514576	0.02000594
CTTNBP2NL	1292.38494	0.2870228	0.06387742	4.49333754	7.01E-06	5.95E-05
CTU2	1378.57675	0.22674388	0.06024155	3.76391195	0.00016728	0.00103044
CTXN1	1144.14635	0.34750878	0.0687002	5.05833689	4.23E-07	4.60E-06
CUEDC2	2146.77137	0.19054996	0.05226938	3.64553701	0.00026683	0.00156748
CXCL17	28.6510703	-1.0727195	0.37772987	-2.8399119	0.0045126	0.01786813
CXCL8	37.7173696	-1.4283994	0.33743792	-4.2330731	2.31E-05	0.0001734
CXCR4	612.373219	0.60713421	0.08702628	6.9764466	3.03E-12	6.98E-11
CYB5B	6667.05876	0.13145364	0.03824032	3.43756681	0.00058697	0.00311398
CYB5R1	2575.9872	-0.2329311	0.04639203	-5.0209289	5.14E-07	5.53E-06
CYB5R4	1367.12064	0.26368111	0.05947036	4.43382384	9.26E-06	7.63E-05
CYBA	8726.58556	0.25127286	0.04261981	5.89568288	3.73E-09	5.83E-08
CYFIP1	7738.08333	0.17750905	0.04071248	4.36006502	1.30E-05	0.00010335
CYFIP2	2134.34032	0.39877907	0.05929646	6.7251747	1.75E-11	3.72E-10
CYP1A1	386.2716	-0.5016786	0.11249896	-4.459407	8.22E-06	6.86E-05
CYP1B1	24087.6747	-0.3797208	0.03042982	-12.478576	9.77E-36	1.13E-33
CYP24A1	728.650664	0.90552154	0.07835586	11.5565255	6.84E-31	6.27E-29
CYP27B1__M	1368.23795	0.2261145	0.05997207	3.77032987	0.00016303	0.0010077
CYP2C8	87.7084701	-1.0057005	0.21463276	-4.6856805	2.79E-06	2.57E-05
CYP2E1	36.0680624	-1.4539686	0.35902747	-4.049742	5.13E-05	0.00035603
CYP2J2	542.813457	0.26319029	0.08924056	2.94922273	0.00318574	0.01328352
CYP4F23P	31.5075397	-1.2837879	0.36312993	-3.5353405	0.00040725	0.00226716

CYP4F33P__I	405.777909	-0.2984145	0.10286545	-2.9010182	0.00371952	0.01516587
CYP4V2__KLI	325.200196	-0.2703225	0.11021242	-2.452741	0.01417724	0.04591612
CYSRT1	158.23483	-0.5568544	0.16307341	-3.4147465	0.00063841	0.00334738
D2HGDH	2296.92668	-0.2326367	0.05205105	-4.4693945	7.84E-06	6.58E-05
DAB2IP	2641.34709	0.38734616	0.04949795	7.82549947	5.06E-15	1.62E-13
DAD1	4293.04837	-0.1214432	0.04530572	-2.680526	0.00735066	0.02695733
DAG1	7365.38859	0.11600374	0.03707989	3.12848113	0.00175712	0.00801586
DAGLB__KDE	16393.7219	0.11763044	0.03225708	3.6466551	0.00026568	0.00156118
DANCR	1391.99115	0.18771453	0.06317695	2.9712503	0.0029659	0.01250699
DAP	7925.12628	0.1051053	0.03656115	2.87478087	0.00404308	0.01629565
DAPK2	242.935855	-0.7352894	0.1378806	-5.3327985	9.67E-08	1.17E-06
DAPK3	1756.97836	0.16799242	0.05951107	2.82287691	0.00475948	0.01872154
DARS	5479.42522	-0.2253562	0.03819562	-5.9000522	3.63E-09	5.68E-08
DARS-AS1	95.3461057	-0.6551548	0.22251493	-2.9443183	0.00323667	0.01346815
DARS2	6926.93374	0.15104985	0.03890985	3.8820465	0.00010358	0.00067118
DAXX	4364.11524	0.13101503	0.0407049	3.21865496	0.00128793	0.00614608
DAZAP2	19171.3517	0.17942395	0.03324341	5.39727844	6.77E-08	8.42E-07
DBF4	2902.2501	0.15381437	0.0483741	3.17968439	0.00147436	0.00688435
DBF4B	923.970438	0.31363778	0.0704039	4.45483547	8.40E-06	7.00E-05
DBN1	3831.52418	0.39009454	0.05003926	7.79577013	6.40E-15	2.02E-13
DBNDD2__SY	2768.82692	0.39784301	0.04748972	8.37745463	5.41E-17	2.00E-15
DBP	1171.97365	-0.3056361	0.07010774	-4.3595206	1.30E-05	0.00010356
DBT	948.601643	0.24807429	0.07207347	3.44196381	0.00057751	0.00307005
DCAF12	4064.46939	0.32655764	0.04209488	7.75765608	8.65E-15	2.68E-13
DCAF16	1725.81378	-0.1521303	0.05408041	-2.8130399	0.00490756	0.01920678
DCAF6	2892.3054	0.2800833	0.04682381	5.98164307	2.21E-09	3.55E-08
DCAF7	18884.0531	0.34811953	0.03361388	10.3564233	3.91E-25	2.54E-23
DCBLD1	984.310036	0.23429479	0.06711574	3.4909069	0.00048138	0.00262946
DCLK1	2236.22688	0.53602711	0.05097133	10.5162484	7.27E-26	5.00E-24
DCP1B	733.918087	0.40394249	0.07720161	5.23230634	1.67E-07	1.95E-06
DCP2	4380.35312	0.131057	0.05171606	2.53416445	0.01127158	0.03832296
DCST2	94.3702301	-1.1322183	0.21623042	-5.2361655	1.64E-07	1.91E-06
DCTD	2030.33949	0.17232785	0.05627534	3.06222659	0.00219697	0.00968324
DCTN1__RP1	9068.89135	0.22545214	0.03829574	5.88713321	3.93E-09	6.12E-08
DCTN4	5251.92473	0.12630546	0.04063415	3.10835723	0.00188131	0.00850154
DCTPP1	6596.24064	0.1044722	0.03987569	2.61994743	0.00879433	0.03128284
DCUN1D3__I	3058.69961	-0.1627349	0.04853017	-3.3532716	0.00079862	0.00406422
DDAH1	2356.13123	-0.2438161	0.05370025	-4.5403163	5.62E-06	4.88E-05
DDB1	18054.4416	0.19560605	0.03697735	5.2898886	1.22E-07	1.47E-06
DDOST	14611.3812	0.21260306	0.0332098	6.40181717	1.54E-10	2.89E-09
DDR1	19868.2347	0.18381627	0.03744247	4.90929854	9.14E-07	9.31E-06
DDX10	1256.35013	0.35855413	0.06372578	5.626516	1.84E-08	2.54E-07
DDX11	2422.80396	-0.1517251	0.05338394	-2.8421477	0.00448107	0.01777231

DDX23	8757.13292	0.13768161	0.03570386	3.85621059	0.00011516	0.00073859
DDX31	1494.18348	0.16326102	0.05785053	2.8221182	0.00477076	0.01875624
DDX54	7736.90729	0.25054392	0.04716209	5.31240113	1.08E-07	1.30E-06
DDX60	476.10267	-1.6087077	0.10412138	-15.450311	7.51E-54	2.01E-51
DECR2__NM	6368.74173	0.23954956	0.04172806	5.74073079	9.43E-09	1.38E-07
DEDD2	1275.90575	0.39252613	0.06850171	5.73016514	1.00E-08	1.46E-07
DENND6B	257.514619	-0.5950711	0.1335602	-4.4554527	8.37E-06	6.98E-05
DENR	6229.17619	0.11870853	0.04331671	2.7404787	0.00613498	0.02314611
DEPDC1	1890.08442	0.29943645	0.05463188	5.48098373	4.23E-08	5.45E-07
DEPDC1B	1423.4859	0.41237207	0.06228548	6.62067795	3.58E-11	7.33E-10
DEPTOR	2993.45955	0.32612175	0.04652219	7.01002505	2.38E-12	5.53E-11
DFFA	3667.67073	0.12222781	0.04482376	2.72685283	0.00639416	0.02398499
DGCR2	4770.15962	0.22618916	0.04304971	5.2541386	1.49E-07	1.75E-06
DHCR7	11729.8153	0.30569367	0.04409418	6.93274451	4.13E-12	9.27E-11
DHRS1	1486.42162	-0.3242778	0.0613213	-5.2881754	1.24E-07	1.48E-06
DHRS2	1566.7995	-0.6372737	0.05752632	-11.077949	1.61E-28	1.26E-26
DHTKD1	6068.68111	0.15006888	0.03816611	3.93199297	8.42E-05	0.00055717
DHX30	4794.96324	0.21238187	0.04400898	4.82587579	1.39E-06	1.36E-05
DHX37	3082.18192	0.31050504	0.0476811	6.51211995	7.41E-11	1.46E-09
DHX8	2802.43027	0.34169241	0.04921482	6.94287618	3.84E-12	8.68E-11
DICER1-AS1	350.076347	-0.5098009	0.11761007	-4.3346707	1.46E-05	0.00011483
DIDO1	10654.3256	0.09546143	0.03788221	2.5199541	0.01173701	0.03960265
DIO2	16.0174475	-3.2744393	0.65969858	-4.9635385	6.92E-07	7.24E-06
DIP2A	4860.5805	-0.18511	0.04390256	-4.2163817	2.48E-05	0.00018491
DIP2B	6522.96516	0.17270276	0.04363952	3.95748546	7.57E-05	0.00050681
DIRAS2	105.310952	1.11861993	0.19457407	5.74907003	8.97E-09	1.32E-07
DIRC2	986.665384	0.2527052	0.06830356	3.69973703	0.00021582	0.00129494
DIXDC1	258.10052	0.33177133	0.12525252	2.64881956	0.00807734	0.02921109
DLD	7740.56904	0.13157153	0.03653538	3.6012088	0.00031674	0.00182249
DLGAP1-AS2	124.081932	-0.8292853	0.17895705	-4.6339907	3.59E-06	3.24E-05
DLGAP5	3984.56889	0.34069854	0.04449674	7.65670804	1.91E-14	5.71E-13
DMTF1	3490.58035	-0.2467576	0.04243319	-5.8152034	6.06E-09	9.15E-08
DMXL2	5680.7666	-0.1898661	0.05156375	-3.6821617	0.00023127	0.00137807
DNAAF1	319.49957	-0.7620716	0.11499765	-6.6268452	3.43E-11	7.04E-10
DNAAF5	4417.07424	0.13796273	0.05210079	2.64799713	0.00809702	0.02926483
DNAH1	205.885153	-0.5025097	0.13917567	-3.6106148	0.00030547	0.00176657
DNAJB1	10103.2257	0.2961543	0.03407905	8.69021597	3.62E-18	1.47E-16
DNAJB12	3122.3651	0.132527	0.04696818	2.82163364	0.00477797	0.0187688
DNAJC14__R	6379.02494	0.14296516	0.03941003	3.62763374	0.00028603	0.00166895
DNAJC22	2931.81708	0.33241043	0.04720477	7.04188214	1.90E-12	4.48E-11
DNAJC3	3540.84801	-0.2745555	0.04271293	-6.4279262	1.29E-10	2.46E-09
DNAJC3-AS1	325.58984	-0.2752975	0.11205823	-2.4567361	0.01402056	0.04554622
DNAJC5	9720.76302	0.16713775	0.03775576	4.42681494	9.56E-06	7.85E-05

DNAJC6	661.140561	0.42828004	0.08357476	5.12451447	2.98E-07	3.35E-06
DNAJC7	3827.0125	0.19875796	0.04212448	4.71834757	2.38E-06	2.22E-05
DNASE1	2244.55449	-0.2460695	0.05155304	-4.7731324	1.81E-06	1.73E-05
DNASE2	3382.89799	0.47564486	0.04406391	10.7944313	3.66E-27	2.71E-25
DNER	49.0237505	-1.3117955	0.29205225	-4.4916467	7.07E-06	5.99E-05
DNMT1__S1	13067.6507	0.14293855	0.03783154	3.77829018	0.00015791	0.00098036
DNMT3A	4762.4754	0.31089153	0.04201368	7.3997683	1.36E-13	3.70E-12
DNMT3B	1333.42968	0.33894778	0.0626437	5.410724	6.28E-08	7.84E-07
DNPEP	7913.78057	0.20586028	0.04140253	4.97216725	6.62E-07	6.95E-06
DNPH1	2014.77244	0.2782118	0.05669408	4.90724596	9.24E-07	9.40E-06
DNTTIP2	2283.874	-0.20079	0.05001466	-4.0146221	5.95E-05	0.00040738
DOC2A	1925.35349	-0.190863	0.05501396	-3.4693561	0.00052171	0.00281611
DOCK7	2385.87855	0.13309368	0.05190712	2.56407372	0.01034516	0.03576738
DOCK8	565.122425	0.68690692	0.0931436	7.37470857	1.65E-13	4.42E-12
DOK3	220.674606	-0.5029648	0.13717268	-3.6666539	0.00024575	0.00145768
DOK4	910.789115	0.48166534	0.07215434	6.67548689	2.46E-11	5.14E-10
DOK7	2220.43512	-0.2311187	0.06311757	-3.661717	0.00025053	0.00148269
DOPEY2	4381.48037	0.14406679	0.04523037	3.18517834	0.00144665	0.00678102
DPAGT1	4904.72592	0.34773271	0.04938853	7.04075833	1.91E-12	4.51E-11
DPF2	3678.85855	0.20924996	0.04281013	4.88786062	1.02E-06	1.03E-05
DPM2	3395.0719	-0.1275674	0.04641407	-2.7484633	0.00598753	0.02268842
DPY19L1	1937.95346	0.149604	0.05256437	2.84611041	0.00442569	0.01757069
DPY19L3	3210.33658	0.3067602	0.04698189	6.52932865	6.61E-11	1.31E-09
DPYD	394.693607	-0.4955262	0.10951906	-4.5245656	6.05E-06	5.21E-05
DPYSL3	39.1340905	1.44369835	0.33253858	4.34144623	1.42E-05	0.00011159
DRAM1	2379.9291	0.47001542	0.04789741	9.81296188	9.90E-23	5.50E-21
DRAM2	1322.2837	-0.1887751	0.06117895	-3.0856224	0.00203127	0.00905577
DRG2	2074.95891	0.24692213	0.05207846	4.74134849	2.12E-06	2.01E-05
DRICH1	608.701434	-0.3819314	0.08532354	-4.4762727	7.60E-06	6.40E-05
DSCAM	8850.89451	0.3535074	0.04280532	8.25849193	1.48E-16	5.31E-15
DSCAM-AS1	55398.2293	-0.1818319	0.03133627	-5.8026007	6.53E-09	9.82E-08
DSCR8__KCN	80.6299588	1.38844633	0.25707314	5.40097785	6.63E-08	8.26E-07
DTX2	1107.05141	0.27009403	0.07617715	3.54560432	0.00039171	0.00219432
DTYMK	3110.36631	0.32835226	0.05023544	6.53626673	6.31E-11	1.25E-09
DUBR	246.146923	0.34091528	0.1298668	2.62511501	0.00866197	0.03087827
DUSP1	2187.77164	-0.21064	0.05215657	-4.0386084	5.38E-05	0.00037152
DUSP16	4820.98521	0.44864916	0.04358832	10.2928752	7.59E-25	4.84E-23
DUSP18	359.731427	0.33259857	0.10556903	3.15053157	0.00162974	0.00751311
DUSP4	4406.69219	-0.2239591	0.03988146	-5.6156197	1.96E-08	2.69E-07
DVL2	2269.9077	0.29099133	0.05142349	5.65872431	1.53E-08	2.14E-07
DYNLT3	4504.96896	-0.1650544	0.04095685	-4.0299584	5.58E-05	0.00038429
DYRK1B	1562.41392	0.16495336	0.06565657	2.51236633	0.01199245	0.04024855
DYRK2	2719.34457	0.12202254	0.04561824	2.67486294	0.00747598	0.02736191

DYRK3	115.136034	0.89524908	0.19665492	4.55238583	5.30E-06	4.64E-05
E2F2	1170.35635	-0.1688609	0.06737726	-2.5062	0.01220366	0.04081465
E2F8	1516.45752	0.30249249	0.06163206	4.90803777	9.20E-07	9.37E-06
EAF1	4023.85403	0.20582331	0.04590517	4.48366297	7.34E-06	6.20E-05
EBAG9	2168.80781	-0.1850407	0.0545213	-3.3939163	0.00068901	0.0035661
EBF4	188.747613	-0.7869398	0.15589808	-5.047784	4.47E-07	4.85E-06
EBLN2	99.1066455	-1.1881447	0.22463217	-5.2892898	1.23E-07	1.47E-06
EBPL	2218.74664	0.18842557	0.0553409	3.40481593	0.00066209	0.00345224
ECD	2162.95772	0.14193089	0.05865257	2.41985798	0.01552657	0.04951233
ECHDC2	2179.55158	-0.2986431	0.05509451	-5.4205606	5.94E-08	7.46E-07
ECHS1	9830.56882	0.12611731	0.03414288	3.69380965	0.00022092	0.00132247
ECI1	3454.29231	0.30718361	0.05840789	5.25928309	1.45E-07	1.71E-06
ECM1	70.9238811	-1.0784301	0.2373602	-4.5434325	5.53E-06	4.82E-05
ECT2	6432.88314	0.1638956	0.0381403	4.29717688	1.73E-05	0.00013451
EDA2R	619.16302	0.43152353	0.08601972	5.01656509	5.26E-07	5.63E-06
EDC3	3985.36127	0.14995298	0.04182531	3.58522134	0.00033679	0.00192329
EDC4__NRN:	6205.32771	0.19010002	0.04745041	4.00628804	6.17E-05	0.00041997
EDN1	605.678014	0.32614905	0.08936448	3.64964964	0.0002626	0.00154608
EEF1A2	85931.1325	0.22507079	0.04101724	5.487224	4.08E-08	5.28E-07
EEF1B2	16290.9989	0.21439219	0.05129901	4.17926604	2.92E-05	0.00021494
EEF1GP4	25.8258709	-1.5823653	0.41705374	-3.794152	0.00014815	0.00092702
EEF1G__RP1	78831.7306	0.23831341	0.02788634	8.54588482	1.28E-17	5.00E-16
EEF2	184842.05	0.34389057	0.03777898	9.1026956	8.81E-20	4.01E-18
EFEMP1	670.77593	-1.1723336	0.0815121	-14.382326	6.68E-47	1.27E-44
EFEMP2	120.534432	-2.0549532	0.19597362	-10.485866	1.00E-25	6.83E-24
EFHC1	1230.16904	-0.2967988	0.06501248	-4.5652581	4.99E-06	4.38E-05
EFHD1	1115.00285	-0.6996437	0.06414095	-10.907911	1.06E-27	7.99E-26
EFNA1	2272.72697	-0.2606734	0.05443305	-4.7888816	1.68E-06	1.61E-05
EFNB3	882.818437	0.55887804	0.07290021	7.66634354	1.77E-14	5.32E-13
EFR3B	1359.81944	-0.3664533	0.05994376	-6.1132842	9.76E-10	1.63E-08
EFTUD2	5958.26799	0.15429321	0.04091049	3.7714833	0.00016228	0.0010034
EGLN1	1963.2829	-0.2867768	0.06272284	-4.5721276	4.83E-06	4.25E-05
EGLN1P1__S	234.055822	-0.4085337	0.1340245	-3.048202	0.00230215	0.01007772
EGR1	8385.21649	-0.9315206	0.03416498	-27.265366	1.09E-163	1.99E-160
EGR3	3021.05837	-0.3218723	0.04490307	-7.1681581	7.60E-13	1.87E-11
EHD2	1112.50256	0.42290429	0.06427902	6.57919586	4.73E-11	9.56E-10
EHMT2	6312.09404	0.14194943	0.04138423	3.4300364	0.0006035	0.00318802
EI24	4498.26887	0.16663388	0.0400831	4.15721086	3.22E-05	0.00023544
EID1	12387.4669	-0.1642038	0.03674782	-4.4683975	7.88E-06	6.61E-05
EID3	15.9570683	-1.4514009	0.51924747	-2.7952007	0.00518675	0.02014811
EIF2AK1	15377.2354	0.14349541	0.0316686	4.53115693	5.87E-06	5.08E-05
EIF2D	3154.60105	0.11737595	0.04376231	2.68212442	0.00731562	0.02686669
EIF3F	4906.08951	0.2389177	0.0415804	5.74592062	9.14E-09	1.34E-07

EIF3H	15329.7391	0.12020915	0.03431141	3.50347464	0.00045923	0.00252674
EIF3L	9723.78918	0.24881336	0.03642986	6.82992943	8.50E-12	1.86E-10
EIF4B	45337.2233	0.15810236	0.0283922	5.56851412	2.57E-08	3.47E-07
EIF4E3	1044.71675	-0.2347025	0.07106513	-3.3026391	0.0009578	0.00474835
EIF4EBP1	1447.36642	0.29699848	0.0618879	4.79897466	1.59E-06	1.55E-05
EIF4EBP2	10166.8027	0.24877855	0.03426809	7.25977273	3.88E-13	9.97E-12
EIF4G2	49621.0113	0.15878685	0.03009909	5.27547006	1.32E-07	1.58E-06
EIF4G3	7212.89285	0.16456298	0.04256754	3.86592629	0.00011067	0.00071179
ELAC2	5812.71131	0.16593462	0.03789625	4.37865521	1.19E-05	9.58E-05
ELAVL1	4991.36393	0.16067666	0.04800413	3.34714211	0.00081649	0.00413439
ELF1	13366.4769	0.4340105	0.03521882	12.3232546	6.79E-35	7.46E-33
ELF3	4737.57835	-0.510351	0.0395082	-12.917598	3.58E-38	4.54E-36
ELK1	2548.33504	0.26667689	0.04735539	5.63139523	1.79E-08	2.48E-07
ELMO3	1923.72135	0.31425634	0.06478259	4.85093861	1.23E-06	1.22E-05
ELOVL2	3217.56723	0.64813149	0.04535951	14.2887681	2.57E-46	4.83E-44
ELOVL5	3265.31866	-0.4060781	0.04677215	-8.6820492	3.89E-18	1.56E-16
ELP6	1284.13675	0.19563099	0.06210727	3.14988874	0.00163333	0.00752453
EMC9	1272.6685	-0.1878998	0.06055707	-3.1028544	0.00191664	0.0086398
EME1	713.215356	-0.1955073	0.07764645	-2.5179164	0.01180513	0.03978097
EME2	1594.19383	-0.3229742	0.06442765	-5.0129748	5.36E-07	5.72E-06
EMILIN3	397.42055	-0.8005461	0.10351015	-7.7339863	1.04E-14	3.20E-13
EML1	2638.95562	0.36994342	0.05057337	7.31498452	2.57E-13	6.72E-12
EML3	3039.56729	0.17889687	0.05505777	3.24925727	0.00115707	0.00558296
EML5	1725.89222	-0.2104421	0.05632241	-3.7363822	0.00018669	0.00113772
ENO1	81297.799	-0.2271108	0.03552144	-6.3936263	1.62E-10	3.03E-09
ENO2__LRR(	2252.63689	-0.6160848	0.04982553	-12.364842	4.05E-35	4.56E-33
ENOSF1	4097.54042	-0.3625908	0.04135868	-8.7669815	1.84E-18	7.62E-17
ENOX2	1690.27691	0.26224625	0.05924314	4.42660949	9.57E-06	7.86E-05
ENTPD4__LC	2252.02599	-0.1791755	0.05079014	-3.5277614	0.00041909	0.00232482
ENTPD8	374.054658	-1.0794773	0.1083257	-9.9651078	2.17E-23	1.26E-21
EP300	5492.0771	0.16841411	0.05341294	3.15305811	0.0016157	0.00746405
EP400	7921.98176	0.13278647	0.0460511	2.88345927	0.00393334	0.01592372
EP400NL	1295.5615	-0.5860933	0.06258829	-9.3642646	7.66E-21	3.75E-19
EPAS1	451.096447	-0.5312296	0.09545834	-5.5650418	2.62E-08	3.53E-07
EPB41	2089.46107	0.22321587	0.05907138	3.77874814	0.00015762	0.00097922
EPB41L2	2947.35559	0.3771669	0.04476939	8.42466118	3.62E-17	1.36E-15
EPB41L4B	2595.64056	-0.1924154	0.04874496	-3.947392	7.90E-05	0.00052575
EPC2	1972.70545	0.2261463	0.05737031	3.9418697	8.08E-05	0.00053666
EPG5	1902.52448	0.22027117	0.06109692	3.60527439	0.00031182	0.00179787
EPHB4	12108.0524	0.25861986	0.03944885	6.555828	5.53E-11	1.11E-09
EPN1	7227.35501	0.19980827	0.05138387	3.88854113	0.00010085	0.00065478
EPN2	2097.80113	0.17912115	0.05519853	3.24503492	0.00117436	0.00565294
EPN3__SPAT	9727.29635	0.15922937	0.04301477	3.7017369	0.00021413	0.00128647

EPRS	10584.1529	0.218823	0.0364745	5.99934287	1.98E-09	3.21E-08
EPS8L2	4596.31616	-0.2587923	0.0493029	-5.2490294	1.53E-07	1.79E-06
ERAL1	2710.33734	0.14628497	0.05096728	2.87017419	0.00410246	0.01649123
ERAP2	36.6945013	-0.9232316	0.32491706	-2.8414378	0.00449106	0.01780297
ERC1	2123.33313	0.30434158	0.06248063	4.87097497	1.11E-06	1.11E-05
ERCC6L	1190.09627	0.28956731	0.06640001	4.36095309	1.29E-05	0.00010298
ERGIC3	9377.14557	0.16156699	0.03418652	4.72604396	2.29E-06	2.15E-05
ERMARD	1087.92762	-0.3161404	0.07439957	-4.2492238	2.15E-05	0.00016277
ERMP1	9061.00736	-0.212428	0.03591049	-5.9154858	3.31E-09	5.20E-08
ERO1A	5768.71255	-0.2854419	0.03918188	-7.2850483	3.22E-13	8.34E-12
ERP29	7206.20744	0.30506937	0.04211981	7.24289537	4.39E-13	1.11E-11
ERV3-1__ZN	4670.16194	-0.1163288	0.04738571	-2.454933	0.01409109	0.04573331
ESCO1	2069.16482	0.17121864	0.05154804	3.32153531	0.00089524	0.00447594
ESPL1	3523.65507	0.37046702	0.05119231	7.23677068	4.59E-13	1.16E-11
ESPN	2062.04689	-0.1999879	0.06280895	-3.1840669	0.00145222	0.00680187
ESR1	20164.4499	0.18238583	0.03539042	5.1535366	2.56E-07	2.88E-06
ESRP2	11216.3488	-0.1254718	0.03270947	-3.8359475	0.00012508	0.00079496
ESYT1__ZC3I	12524.3811	0.12620691	0.03860159	3.26947471	0.00107747	0.00523911
ETHE1	607.849101	0.41694661	0.08897898	4.68590018	2.79E-06	2.57E-05
ETS2	1583.6793	-0.3451956	0.0629277	-5.48559	4.12E-08	5.32E-07
EVL	26271.3068	-0.4079578	0.03110524	-13.115404	2.69E-39	3.68E-37
EXD3	938.140331	-0.5132214	0.08546225	-6.0052418	1.91E-09	3.10E-08
EXOC4	4129.79547	0.20335385	0.04054314	5.01573998	5.28E-07	5.64E-06
EXTL2	348.377154	0.32959015	0.11008852	2.99386468	0.00275468	0.01175239
EXTL3	1056.31942	0.1944815	0.07615733	2.55368063	0.0106591	0.03667976
EXTL3-AS1	46.0115314	-0.7979969	0.29324842	-2.7212318	0.00650391	0.02433226
EYA2	536.099909	0.29500016	0.08734727	3.37732538	0.00073194	0.00376694
EYA3	1602.50994	0.22853271	0.05718232	3.99656218	6.43E-05	0.00043613
EZR	8964.97439	-0.0976513	0.03403784	-2.8689038	0.00411897	0.01654666
F11R__RP11	12718.4957	0.0831629	0.03294506	2.52429041	0.01159321	0.03919722
F12	801.291381	0.38472785	0.0843693	4.56004562	5.11E-06	4.48E-05
F2RL1	523.260772	0.2400384	0.09290724	2.58363515	0.00977652	0.03416347
F3	78.2505588	-0.6646345	0.21975915	-3.0243772	0.00249146	0.01078566
FAM102B	9902.21669	0.10541885	0.03282084	3.21194859	0.00131838	0.00627168
FAM106A	985.195135	-0.3999778	0.07200782	-5.5546432	2.78E-08	3.72E-07
FAM106DP	29.3320123	-1.18055	0.38462572	-3.0693477	0.00214527	0.00948516
FAM110B	329.43431	0.5673566	0.11181531	5.07405111	3.89E-07	4.29E-06
FAM111A	3065.10278	-0.1498802	0.04747275	-3.1571839	0.00159301	0.00736857
FAM117A	541.712155	0.26529664	0.09021253	2.94079605	0.0032737	0.01360361
FAM120B	2990.42946	0.13950886	0.04570172	3.05259531	0.00226872	0.00995854
FAM120C	1534.89204	0.25223258	0.05818341	4.33512848	1.46E-05	0.00011464
FAM13A	98.2326711	-0.9596023	0.20502571	-4.6804	2.86E-06	2.63E-05
FAM155B	646.470847	0.29122394	0.08446597	3.44782573	0.00056512	0.00301297

FAM160B2	2015.15779	-0.3946476	0.05661528	-6.9706913	3.15E-12	7.24E-11
FAM162A	1944.17966	-0.4293394	0.05560673	-7.7209971	1.15E-14	3.53E-13
FAM167A-AS	97.0840019	-0.565521	0.20354998	-2.7782905	0.00546458	0.02107018
FAM169A	257.336765	-0.3961978	0.13228569	-2.9950167	0.0027443	0.01171612
FAM171A2	1007.11569	0.38126423	0.09342598	4.08092294	4.49E-05	0.00031568
FAM178B	124.055877	-0.9668612	0.18884603	-5.1198387	3.06E-07	3.42E-06
FAM189A2	1466.46936	-0.3555323	0.06237168	-5.7002205	1.20E-08	1.71E-07
FAM189B	1741.56098	0.3321169	0.06671248	4.9783323	6.41E-07	6.74E-06
FAM193B	2396.63874	-0.1845952	0.05208393	-3.5441865	0.00039383	0.00220412
FAM217B	2788.65857	-0.1907328	0.04733069	-4.0297913	5.58E-05	0.00038442
FAM219A	1157.83271	-0.3706724	0.0629444	-5.888886	3.89E-09	6.06E-08
FAM21A	1453.89238	0.27301076	0.05771437	4.73037734	2.24E-06	2.11E-05
FAM222B	2158.45874	0.25431293	0.05004981	5.08119657	3.75E-07	4.15E-06
FAM228A__I	276.699085	-0.5760876	0.12005403	-4.7985693	1.60E-06	1.55E-05
FAM229A	136.628767	-0.9358181	0.17268049	-5.4193622	5.98E-08	7.51E-07
FAM234B	1922.1475	0.14652164	0.05432453	2.69715436	0.00699349	0.025908
FAM32A	4695.32208	0.24857141	0.04259754	5.83534615	5.37E-09	8.19E-08
FAM43A	485.770945	0.84197664	0.11809169	7.12985485	1.00E-12	2.45E-11
FAM46A	1998.48212	-0.44834	0.0517636	-8.661299	4.66E-18	1.87E-16
FAM46C	623.457474	-0.5699107	0.08650365	-6.5882851	4.45E-11	9.02E-10
FAM53C	3268.19327	0.36267691	0.04407286	8.22903157	1.89E-16	6.75E-15
FAM60A	2976.33255	0.2622181	0.0505994	5.18223742	2.19E-07	2.49E-06
FAM64A	1041.3272	0.53480331	0.0656693	8.1438867	3.83E-16	1.34E-14
FAM65A	2367.9275	0.18809707	0.05119552	3.67409261	0.0002387	0.00142003
FAM71F2	54.7683297	-0.8099417	0.2662582	-3.0419409	0.00235058	0.01025125
FAM83A	1075.45903	-0.5171868	0.0716026	-7.2230166	5.08E-13	1.28E-11
FAM83A-AS1	34.2335621	-0.8212908	0.33833106	-2.4274769	0.01520425	0.04862055
FAM83D	2259.56851	0.41592993	0.05277707	7.88088365	3.25E-15	1.07E-13
FAM83G	2383.25081	0.18124492	0.05537625	3.27297224	0.00106423	0.00518162
FAM86B3P	205.562637	-0.7084733	0.13829171	-5.1230355	3.01E-07	3.37E-06
FARP1	2082.03593	-0.1935335	0.05435532	-3.5605246	0.00037012	0.00209131
FARP2	2224.07934	0.13238873	0.05023925	2.63516561	0.00840962	0.03017302
FASN	50639.2083	0.23046675	0.04726079	4.87649003	1.08E-06	1.08E-05
FASTK	3427.71016	-0.1893808	0.04995152	-3.7912926	0.00014987	0.00093648
FBL	10385.7662	0.10981716	0.03511428	3.1274217	0.00176347	0.00803676
FBRS	7416.62104	0.15252028	0.03702079	4.11985441	3.79E-05	0.00027237
FBXL14	585.776127	0.35184839	0.08640104	4.07227017	4.66E-05	0.00032638
FBXL18	962.829573	0.22906997	0.07192531	3.18483119	0.00144839	0.00678568
FBXL19__OR	5326.64	0.24171631	0.04618132	5.2340707	1.66E-07	1.93E-06
FBXL20	764.808868	-0.2595894	0.07700719	-3.3709755	0.00074903	0.00384616
FBXL8__HSF	2376.53797	-0.7693883	0.06141974	-12.526725	5.33E-36	6.27E-34
FBXO21	4252.8833	0.2043162	0.04035939	5.0624201	4.14E-07	4.52E-06
FBXO27	1183.09712	0.61803854	0.06640577	9.30700051	1.31E-20	6.31E-19

FBXO32	290.037125	-0.5665676	0.11836711	-4.7865295	1.70E-06	1.63E-05
FBXO41	1238.21764	0.38382044	0.0701758	5.46941299	4.52E-08	5.79E-07
FBXO44	727.356201	-0.2716149	0.07786673	-3.4882024	0.00048628	0.00265315
FBXO7	4388.83041	0.10121233	0.04080763	2.48023075	0.01312974	0.04319099
FBXW11	4972.39251	0.18796562	0.0402923	4.66505073	3.09E-06	2.81E-05
FBXW2	5090.82232	-0.1217675	0.03855126	-3.1585869	0.00158536	0.007344
FBXW9	804.229333	0.3094737	0.07808207	3.96344113	7.39E-05	0.00049633
FCGR2A__FC	90.5547062	-0.7005415	0.22234308	-3.1507232	0.00162867	0.00751255
FCHO1	1432.70965	0.2770626	0.06325088	4.38037584	1.18E-05	9.53E-05
FCHSD1	1555.16777	-0.233153	0.06077078	-3.8365972	0.00012475	0.00079313
FDXR	1354.96524	0.40564946	0.07441014	5.45153496	4.99E-08	6.36E-07
FER1L4	1398.52446	-1.5663963	0.06498392	-24.104367	2.25E-128	3.16E-125
FGD2	20.4792003	-1.1229755	0.44909275	-2.5005424	0.01240033	0.04134466
FGD5-AS1	13465.4506	0.16419152	0.03207636	5.11877079	3.08E-07	3.44E-06
FGF12	594.599395	0.33411914	0.09021535	3.70357317	0.00021258	0.00127929
FGFR1	856.63174	-0.5224201	0.07513808	-6.9528007	3.58E-12	8.15E-11
FGGY	452.551003	0.25821045	0.09570004	2.69812263	0.00697318	0.02583802
FHL1	1506.41077	0.65978463	0.05833265	11.3107256	1.16E-29	9.67E-28
FHL2	340.462758	-0.7950149	0.11047919	-7.1960607	6.20E-13	1.55E-11
FIBP	3696.99269	0.15655897	0.04765236	3.28543994	0.00101823	0.00499501
FJX1	462.606546	0.32324608	0.09508845	3.39942523	0.00067528	0.00350498
FKBP10	186.528712	0.69469336	0.14653298	4.74086709	2.13E-06	2.01E-05
FKBP4	23916.1926	0.36646996	0.03206347	11.4295156	2.98E-30	2.57E-28
FKBP8	11633.0314	0.15884698	0.04448534	3.57077119	0.00035593	0.00201805
FLCN__PLD6	3077.84695	0.21139676	0.04400742	4.80366176	1.56E-06	1.51E-05
FLJ31356	68.6370841	-1.1908189	0.24377483	-4.8849132	1.03E-06	1.04E-05
FLNB-AS1	640.607468	-0.3485454	0.08229521	-4.2353067	2.28E-05	0.00017197
FLOT2	4801.08382	0.1896831	0.0421585	4.49928458	6.82E-06	5.80E-05
FMN1	2994.80591	0.2718305	0.05909126	4.60018137	4.22E-06	3.76E-05
FMR1	5150.86255	-0.1449603	0.04050472	-3.5788502	0.00034511	0.00196218
FNBP4	5932.2185	-0.2777108	0.04112401	-6.7530085	1.45E-11	3.10E-10
FNDC3A	3713.42262	0.18289197	0.04726214	3.86973505	0.00010895	0.000702
FNIP2	797.655393	0.69846463	0.07453006	9.37158261	7.15E-21	3.51E-19
FOCAD	2404.19413	0.19017608	0.05686352	3.3444302	0.00082452	0.00416939
FOS	17253.108	-0.7292092	0.0372962	-19.551838	3.98E-85	2.34E-82
FOSB	1787.48831	-1.9927567	0.05870585	-33.94477	1.46E-252	8.86E-249
FOSL2	6706.53747	-0.483399	0.03838717	-12.592723	2.32E-36	2.80E-34
FOXC1	1325.6362	-0.1915845	0.06424309	-2.9821814	0.00286202	0.01214479
FOXH1	1017.9673	-0.7075556	0.06870234	-10.298857	7.13E-25	4.56E-23
FOXI1	73.8364492	-1.5197215	0.24677621	-6.1582982	7.35E-10	1.26E-08
FOXJ2	1560.33887	0.15447709	0.05585456	2.76570245	0.00568003	0.02175378
FOXK2	5899.70225	0.24138099	0.03933886	6.13594213	8.47E-10	1.44E-08
FOXM1	5966.77949	0.33981425	0.04055489	8.37911811	5.33E-17	1.98E-15

FOXP4	4931.53648	0.16554912	0.05170425	3.20184735	0.00136549	0.00645873
FRA10AC1	1352.97772	-0.4102317	0.06336238	-6.4743732	9.52E-11	1.84E-09
FRAS1	2321.99171	0.28635777	0.06929323	4.13255059	3.59E-05	0.00025867
FRAT1	584.447779	0.49759835	0.08462208	5.88024271	4.10E-09	6.36E-08
FRAT2	2335.07736	0.42541842	0.0603833	7.04529916	1.85E-12	4.39E-11
FREM2	6980.93274	0.22619255	0.05203022	4.34733054	1.38E-05	0.00010882
FRS2	2970.36784	0.17151329	0.04464895	3.84137311	0.00012235	0.0007784
FRS3	103.298986	-0.6509113	0.19429271	-3.3501582	0.00080765	0.00410102
FSD2	197.982413	-0.8064969	0.16452789	-4.9018858	9.49E-07	9.64E-06
FSTL4	241.257925	-0.6407407	0.13160098	-4.8688143	1.12E-06	1.12E-05
FTCD	87.4166067	-0.8892	0.22096533	-4.0241606	5.72E-05	0.00039299
FTH1	104101.7	-0.0735118	0.02852914	-2.5767265	0.00997408	0.03477389
FTL	30530.2606	0.33217681	0.03744462	8.87115007	7.24E-19	3.09E-17
FTSJ3	14176.6841	0.11287137	0.03277447	3.44388067	0.00057343	0.00305371
FUT10__TTI2	759.357988	0.25626799	0.08198332	3.12585536	0.00177289	0.00807162
FUT11	767.640858	-0.2317564	0.08348747	-2.7759426	0.00550419	0.02119161
FXR1	8336.65654	0.09824919	0.03859892	2.54538716	0.01091566	0.03741282
FZD2	643.601558	0.58318192	0.0836106	6.9749759	3.06E-12	7.03E-11
G3BP2	5456.56352	0.17182417	0.03962382	4.33638608	1.45E-05	0.00011409
G6PC3	2369.33383	0.46577367	0.05790793	8.04334833	8.74E-16	3.02E-14
GABBR1__U	575.11413	-1.0193868	0.09064347	-11.246113	2.42E-29	1.97E-27
GABPB1-AS1	4732.08917	-0.615965	0.04088648	-15.065249	2.74E-51	6.33E-49
GAK	5814.15635	0.13434206	0.04569788	2.93978736	0.00328438	0.01364176
GAL	279.076725	-0.7501897	0.1200682	-6.2480298	4.16E-10	7.32E-09
GALC	5311.83373	1.15017231	0.03901949	29.4768698	5.70E-191	1.48E-187
GALM	247.094727	1.004591	0.1347807	7.4535227	9.09E-14	2.50E-12
GALNT14	157.153165	0.5084437	0.17090959	2.97492776	0.00293058	0.0123752
GALNT16	417.66115	0.43855939	0.11010896	3.98295826	6.81E-05	0.00046016
GALNT4__PC	6564.677	0.10402537	0.03943371	2.63798058	0.00834014	0.02999488
GALNT6	4695.034	-0.2469469	0.04005598	-6.1650438	7.05E-10	1.21E-08
GALNT7	3796.96906	0.15860915	0.0425015	3.73184837	0.00019008	0.00115604
GALT__IL11F	399.938075	-0.3479154	0.10640455	-3.2697421	0.00107646	0.00523556
GAN	3854.58056	-0.286018	0.05831894	-4.904376	9.37E-07	9.53E-06
GANAB	22099.5506	0.18843616	0.03472354	5.42675551	5.74E-08	7.26E-07
GAPDH	111152.661	-0.2792176	0.02888192	-9.6675582	4.14E-22	2.21E-20
GAREML	62.4753183	0.65868684	0.25572963	2.57571574	0.01000329	0.0348253
GARS	6805.24021	0.45773975	0.03740638	12.236943	1.97E-34	2.09E-32
GART	4555.11835	0.1719304	0.04097416	4.19606889	2.72E-05	0.0002009
GAS2L1	1337.2481	0.24350908	0.07273765	3.34777242	0.00081464	0.00412729
GAS2L3	1372.4145	0.16851922	0.05848813	2.88125524	0.00396095	0.01601417
GAS8	2192.97111	-0.3280676	0.05035877	-6.5146081	7.29E-11	1.44E-09
GAS8-AS1	43.2653545	-1.0132583	0.35660233	-2.8414236	0.00449126	0.01780297
GATA3-AS1_	3345.00874	0.48042736	0.0447949	10.7250459	7.76E-27	5.65E-25

GATA6	493.319635	0.37681288	0.09916379	3.79990398	0.00014475	0.0009092
GBA	2592.79273	0.43509739	0.05545941	7.84533013	4.32E-15	1.40E-13
GBE1	1378.95477	-0.3227697	0.06241679	-5.1712002	2.33E-07	2.63E-06
GBF1	5884.27612	0.16025698	0.04342852	3.69013256	0.00022414	0.00133953
GCA	2976.36944	0.21070292	0.04938462	4.26656959	1.99E-05	0.00015166
GCAT	448.502397	0.46245179	0.10036866	4.60753184	4.07E-06	3.65E-05
GCFC2	709.653846	-0.2422153	0.07765848	-3.1189806	0.00181478	0.0082429
GCH1	2131.30954	-0.1955231	0.05296366	-3.6916466	0.00022281	0.00133246
GCLM	1260.22645	-0.3958809	0.07248574	-5.4615007	4.72E-08	6.04E-07
GCN1	12963.6918	0.16426705	0.04324387	3.79862083	0.0001455	0.00091329
GCOM1__MY	2208.67943	0.14458458	0.0523493	2.76191996	0.00574626	0.02195667
GDAP1	1235.12085	0.17543899	0.06119846	2.86672235	0.00414747	0.01662817
GDF11	2091.06172	0.44460831	0.05471135	8.12643658	4.42E-16	1.55E-14
GDPD3	266.558936	-0.7819804	0.12406444	-6.3030182	2.92E-10	5.25E-09
GEMIN4	2795.5075	0.19453309	0.05180751	3.7549208	0.0001734	0.00106491
GEMIN5	3730.27302	0.12313286	0.04783684	2.5740174	0.01005252	0.03495379
GFM2	2600.81935	0.12857226	0.04838936	2.6570355	0.00788311	0.02861651
GFOD2	1658.08134	0.2282274	0.05491989	4.15564181	3.24E-05	0.00023697
GFPT1	4740.58882	0.20674433	0.0407936	5.06805833	4.02E-07	4.41E-06
GFRA1	46513.5388	0.12138668	0.02799701	4.33570181	1.45E-05	0.00011439
GFRA3	115.537148	1.20745832	0.19761779	6.11006892	9.96E-10	1.66E-08
GID4	753.887481	0.30095067	0.07563656	3.97890498	6.92E-05	0.00046727
GID8	14314.4838	0.13211626	0.03316089	3.98409872	6.77E-05	0.0004583
GIGYF1	5641.17372	-0.1892082	0.04218732	-4.4849552	7.29E-06	6.17E-05
GIPR	135.486901	-1.0874109	0.18505781	-5.8760606	4.20E-09	6.51E-08
GIT1	4196.65085	0.19248003	0.04789921	4.01843862	5.86E-05	0.00040144
GIT2	1819.73996	0.15742998	0.05757521	2.7343361	0.00625062	0.02352401
GJA9__MYCE	3774.62929	0.1306095	0.0467025	2.79662771	0.0051639	0.02006362
GJB3	447.650133	0.28672325	0.09406013	3.04829732	0.00230142	0.01007772
GJD3	445.93122	0.58519591	0.09855678	5.93765242	2.89E-09	4.58E-08
GK5	1439.76656	-0.3393215	0.06024261	-5.6325822	1.78E-08	2.46E-07
GLA	5902.47951	0.3663892	0.04002404	9.15422878	5.47E-20	2.52E-18
GLB1L	182.882911	-0.5885044	0.14842481	-3.9650001	7.34E-05	0.00049329
GLDN	410.293404	-0.8970816	0.10464505	-8.5726138	1.01E-17	4.02E-16
GLI3	1012.52318	0.19151103	0.06824599	2.8061872	0.00501316	0.01956966
GLIPR1	58.9424355	-1.13687	0.26136464	-4.3497468	1.36E-05	0.00010782
GLMP	1331.23557	0.49610784	0.06364202	7.79528739	6.43E-15	2.02E-13
GLRA3	1029.7683	0.30965198	0.07560019	4.0959154	4.21E-05	0.00029811
GLS2	492.135952	0.34130045	0.09226436	3.69915799	0.00021632	0.00129704
GLTSCR1	1675.08828	0.14635574	0.06035077	2.42508462	0.01530482	0.04889259
GLTSCR2	14699.0363	0.2117735	0.04781298	4.42920508	9.46E-06	7.78E-05
GLUD1	6989.7956	0.16324947	0.03802857	4.29281116	1.76E-05	0.00013672
GLUD2	291.11861	0.68823371	0.11618629	5.92353655	3.15E-09	4.97E-08

GLYR1	5334.47135	0.13973827	0.03904284	3.57910092	0.00034478	0.00196091
GMEB2	2615.60852	0.17158928	0.05229351	3.28127293	0.0010334	0.00505173
GMIP	2268.02831	0.33462033	0.05429759	6.16271066	7.15E-10	1.23E-08
GNA12	6672.78205	0.45696218	0.04045202	11.2963997	1.37E-29	1.13E-27
GNA14	2179.30025	0.70432019	0.05620684	12.5308631	5.06E-36	5.99E-34
GNAI1	56.1383354	-3.6325764	0.37102448	-9.7906648	1.23E-22	6.78E-21
GNAL	35.9652998	-0.8557239	0.34507583	-2.4798142	0.01314509	0.04323368
GNAS	125031.661	0.12144126	0.02817171	4.31075305	1.63E-05	0.00012738
GNB1	18768.2442	0.11341936	0.03057533	3.709505	0.00020767	0.00125134
GNB2	23973.3449	0.25986107	0.05113146	5.0822152	3.73E-07	4.13E-06
GNB2L1	76328.5795	0.08827659	0.02915384	3.02795731	0.00246213	0.01067391
GNPDA1	2541.22482	0.21360054	0.04877035	4.37972124	1.19E-05	9.54E-05
GNPTAB	4666.29049	0.14032378	0.04003694	3.50485787	0.00045685	0.00251744
GOLGA2	8317.25302	-0.1344541	0.03486211	-3.8567412	0.00011491	0.00073724
GOLGA6L10	229.588115	-0.6303005	0.13448652	-4.6867188	2.78E-06	2.56E-05
GOLGA6L4	68.2202257	-0.9821275	0.24065871	-4.0809971	4.48E-05	0.00031568
GOLGA6L5P_	93.1883192	-1.4303569	0.22873499	-6.2533367	4.02E-10	7.11E-09
GOLGA6L9	903.117694	-0.6700076	0.0748985	-8.9455405	3.70E-19	1.63E-17
GOLGA8A	984.908551	-0.888328	0.07486424	-11.865851	1.78E-32	1.76E-30
GOLGA8B	390.388331	-0.789866	0.10270519	-7.6906141	1.46E-14	4.44E-13
GOLGA8N	258.558458	-1.1003416	0.13121612	-8.3857197	5.04E-17	1.88E-15
GOLGA8R	38.8236708	-1.0328373	0.32334345	-3.1942421	0.00140199	0.00660581
GOLGA8S	19.7286875	-1.1261041	0.44839502	-2.5114108	0.01202497	0.04029645
GOLIM4	1589.23204	0.36741825	0.05857397	6.27272254	3.55E-10	6.31E-09
GOLM1	751.510896	-0.7151433	0.07965471	-8.9780412	2.76E-19	1.22E-17
GOLPH3L	2965.10615	0.20868666	0.04939204	4.22510735	2.39E-05	0.00017862
GORASP1	2984.28024	0.12988282	0.04637182	2.80089997	0.00509603	0.01985068
GORASP2	6580.1315	0.15565667	0.03658715	4.2544082	2.10E-05	0.00015949
GOSR2__RP:	1497.24478	0.26084549	0.05780243	4.51270814	6.40E-06	5.49E-05
GOT1	2367.52047	0.12891192	0.0494472	2.60706213	0.00913228	0.03228344
GOT2	7258.50201	0.17200587	0.03796305	4.53087631	5.87E-06	5.08E-05
GPAT3	394.155441	-0.3465482	0.10197543	-3.3983494	0.00067794	0.00351706
GPATCH2	2743.1785	0.38814155	0.04947431	7.84531473	4.32E-15	1.40E-13
GPBP1L1	5400.91569	0.18576782	0.03759612	4.94114385	7.77E-07	8.01E-06
GPC1	3528.64408	-0.4064724	0.05470287	-7.4305488	1.08E-13	2.96E-12
GPCPD1	789.854839	-0.5793121	0.08305108	-6.9753711	3.05E-12	7.02E-11
GPER1	3665.77635	0.55531827	0.04867747	11.4081176	3.81E-30	3.28E-28
GPI__PDCD2I	27506.0599	-0.253067	0.03367664	-7.5146165	5.71E-14	1.61E-12
GPN1__ZNF!	3256.07608	0.14669412	0.04701103	3.12041901	0.00180594	0.0082098
GPN3	1249.6725	0.19002832	0.06821948	2.78554328	0.00534381	0.02069648
GPNMB	285.793844	0.94998128	0.11981388	7.92880796	2.21E-15	7.44E-14
GPR137B	1353.48737	0.30616073	0.06193269	4.94344304	7.68E-07	7.92E-06
GPR155	149.081741	-0.45404	0.16317548	-2.7825257	0.00539376	0.02086777

GPR179	253.676602	-3.2726068	0.16664367	-19.63835	7.27E-86	4.57E-83
GPR21__RAE	7050.72387	0.15706906	0.03786897	4.14769784	3.36E-05	0.00024445
GPR37L1	1148.38459	-0.2791475	0.06333075	-4.4077718	1.04E-05	8.50E-05
GPR68	574.346409	0.37548757	0.0857711	4.37778652	1.20E-05	9.61E-05
GPRC5A	13823.4955	0.26295768	0.03526753	7.45608461	8.91E-14	2.46E-12
GPRIN1	1368.55275	0.26129067	0.06353217	4.11273038	3.91E-05	0.0002801
GPT2	1767.58597	0.38400006	0.05602138	6.85452718	7.15E-12	1.58E-10
GPX3	1943.5181	0.47484164	0.05420664	8.75984341	1.96E-18	8.08E-17
GPX4	8532.58303	0.28018317	0.03612243	7.75648647	8.73E-15	2.69E-13
GPX8	772.833681	0.67910302	0.08311144	8.17099379	3.06E-16	1.08E-14
GRB2	17330.4646	0.19470039	0.03029007	6.42786152	1.29E-10	2.46E-09
GREB1L	820.75779	-0.2359557	0.07631963	-3.0916783	0.00199028	0.00890573
GRHL2	12312.8883	0.21812143	0.03772393	5.7820448	7.38E-09	1.10E-07
GRIN1	555.538566	-0.2368269	0.09556579	-2.4781561	0.01320634	0.04338816
GRIN2C	134.45525	-0.5779758	0.18636494	-3.1013121	0.00192665	0.00866807
GRIP1	594.428667	0.25248384	0.08711064	2.89842698	0.0037504	0.01526786
GRIPAP1	3571.11686	-0.1178227	0.04538975	-2.5958002	0.00943709	0.03317447
GRK6	4317.0818	0.22933655	0.05423637	4.22846437	2.35E-05	0.00017656
GRN	15169.0827	0.33960659	0.04412319	7.69678214	1.40E-14	4.24E-13
GRPR	25.1427578	1.04844299	0.39859519	2.63034529	0.00852982	0.03053253
GS1-114I9.1	149.314795	-0.4776853	0.16025044	-2.9808674	0.00287433	0.01219134
GS1-114I9.3_	2035.23605	0.34907432	0.05721689	6.10089647	1.05E-09	1.75E-08
GS1-124K5.1	354.256201	-0.4971041	0.11936335	-4.1646297	3.12E-05	0.00022837
GS1-358P8.4	1152.62834	-0.39644	0.08883314	-4.4627486	8.09E-06	6.77E-05
GSDMB	240.916639	-0.7177506	0.13357594	-5.3733524	7.73E-08	9.53E-07
GSTM3	6036.65484	-0.2105564	0.04042921	-5.2080264	1.91E-07	2.20E-06
GTF2A1	3766.96449	0.15457442	0.04842463	3.19206188	0.00141261	0.00664368
GTF2I	5152.25735	0.17165428	0.05858287	2.93011064	0.00338841	0.01400053
GTF2IP13	353.785011	-0.3659464	0.11820963	-3.0957411	0.00196322	0.00879757
GTF2IP20	424.301114	-0.3286564	0.1183531	-2.7769139	0.00548777	0.02113525
GTF2IRD2	310.589693	-0.6822225	0.11791977	-5.7854805	7.23E-09	1.08E-07
GTF3C1	10095.999	0.26332724	0.03874011	6.79727634	1.07E-11	2.31E-10
GTPBP2	2607.15869	-0.1829528	0.05222313	-3.5032914	0.00045955	0.00252695
GTPBP3	1142.26989	-0.2118694	0.07231303	-2.9298919	0.0033908	0.01400274
GTSE1	1322.54056	0.37433729	0.05931708	6.3107842	2.78E-10	5.01E-09
GUSBP3	30.4153799	-0.9376953	0.35890196	-2.6126782	0.00898359	0.03185661
H19__MIR67	1200.08352	-0.898982	0.06671403	-13.475157	2.19E-41	3.27E-39
H1F0	12655.3539	-0.2131684	0.03856603	-5.5273629	3.25E-08	4.27E-07
H2AFX	10303.9132	0.45564573	0.09666842	4.71349099	2.44E-06	2.27E-05
H2AFY	15324.8668	-0.1074005	0.03294014	-3.2604756	0.00111226	0.00538955
H2AFY2	2346.37588	0.23602693	0.049064	4.81059295	1.50E-06	1.46E-05
H2AFZ	20035.3997	0.11670597	0.03699501	3.15464102	0.00160696	0.00742932
HACD2	12574.3705	-0.2846095	0.03325418	-8.5586086	1.14E-17	4.50E-16

HADHA	11245.4468	0.11696057	0.03419569	3.42033046	0.00062545	0.00329039
HADHB	5997.74541	0.16683735	0.04302823	3.87739284	0.00010558	0.0006822
HAUS5	1366.9311	-0.2322589	0.06448294	-3.6018664	0.00031594	0.00181845
HAUS7__TRI	1547.70215	0.15924602	0.05555616	2.86639724	0.00415173	0.01663999
HAX1	3608.15372	0.17025609	0.04303408	3.95630817	7.61E-05	0.00050821
HBP1	2357.54278	0.23278932	0.05182373	4.49194453	7.06E-06	5.98E-05
HCFC1	9248.49664	0.12027964	0.04195566	2.86682761	0.00414609	0.0166263
HCFC1R1	1788.35879	-0.2385381	0.05352401	-4.4566562	8.32E-06	6.95E-05
HCK	441.406913	0.38939068	0.10272871	3.7904757	0.00015036	0.00093892
HCN2	2084.7203	0.16841057	0.06673355	2.52362678	0.01161512	0.03925673
HCN3	596.765282	-0.5640661	0.08745101	-6.4500808	1.12E-10	2.14E-09
HDAC10__M	1224.76112	-0.3435006	0.06667751	-5.1516706	2.58E-07	2.91E-06
HDAC2	8196.74119	0.09251619	0.03673117	2.51873754	0.01177764	0.03971034
HDAC5	1264.45535	0.35966719	0.06792476	5.29508202	1.19E-07	1.43E-06
HDAC6	1857.52995	-0.2786752	0.05566166	-5.0065922	5.54E-07	5.90E-06
HDGF	26658.6902	0.1117104	0.03293193	3.39216051	0.00069344	0.00358445
HDLBP	40249.3603	0.21707505	0.03291136	6.59574774	4.23E-11	8.62E-10
HELLS	3549.61738	-0.126145	0.05203292	-2.4243299	0.01533667	0.04897526
HELZ	7482.52537	0.14581673	0.03840153	3.79715889	0.00014636	0.00091749
HEMK1	869.899992	-0.4917329	0.07207668	-6.8223579	8.96E-12	1.95E-10
HENMT1	2909.5253	0.21580821	0.04919448	4.38683799	1.15E-05	9.29E-05
HERC2P9	533.621736	-0.3482962	0.08976536	-3.8800736	0.00010443	0.00067569
HES1	5515.90586	-0.2896131	0.05290317	-5.4743992	4.39E-08	5.63E-07
HES4	1620.35411	-0.191493	0.07321581	-2.6154604	0.00891073	0.03163515
HGFAC	43.5699018	-1.2090462	0.306296	-3.947313	7.90E-05	0.00052575
HHIPL2	59.5153139	-0.8289077	0.26134846	-3.171657	0.00151572	0.00705762
HID1	6059.6718	-0.1049908	0.03950619	-2.6575785	0.00787043	0.02860461
HIP1	5759.39939	0.51124245	0.0409243	12.4923947	8.21E-36	9.54E-34
HIST1H2AC	2596.8453	0.25412655	0.05311986	4.78402199	1.72E-06	1.65E-05
HIST1H2AD_	178.786391	0.54331317	0.15828533	3.43249223	0.00059806	0.00316272
HIST1H2AG	535.067741	0.40836414	0.08799649	4.64068685	3.47E-06	3.14E-05
HIST1H2AI	106.369522	0.66041446	0.19366156	3.41014746	0.00064928	0.00339614
HIST1H2APS	49.4160256	0.74252865	0.28098525	2.64258943	0.00822747	0.02965403
HIST1H2BG	950.796331	0.40440479	0.07194105	5.62133594	1.89E-08	2.61E-07
HIST1H2BH	269.933366	0.31740823	0.12409664	2.55775032	0.01053517	0.03631348
HIST1H2BJ	202.762208	0.73049	0.13951645	5.23587021	1.64E-07	1.91E-06
HIST1H3C	23.1483862	1.21158243	0.42625219	2.84240755	0.00447742	0.01776356
HIST1H3E	847.576227	0.43029913	0.07640565	5.63177145	1.78E-08	2.47E-07
HIST1H3G	356.407742	0.48433663	0.11125861	4.35325096	1.34E-05	0.00010628
HIST1H3H	1126.81925	0.60800192	0.06738965	9.02218604	1.84E-19	8.22E-18
HIST2H2AA3_	5374.72809	0.13584308	0.04106062	3.30835419	0.00093846	0.00466518
HIST3H2A	1072.1286	0.27213684	0.06464073	4.20999049	2.55E-05	0.00018999
HIVEP1	1483.26974	0.15849017	0.06151534	2.57643312	0.00998255	0.03478396

HIVEP3	817.285882	0.50572591	0.07727496	6.54449938	5.97E-11	1.19E-09
HK2	2986.15947	-0.4301003	0.04603005	-9.3439016	9.28E-21	4.50E-19
HLA-A	4465.17101	-0.1497709	0.04656642	-3.2162852	0.00129862	0.00618897
HLA-B	2192.87569	-0.3097437	0.04913165	-6.3043607	2.89E-10	5.21E-09
HLA-DQB1	1062.18401	0.23941734	0.06532567	3.66498103	0.00024736	0.00146677
HLA-DRB5	1554.89481	0.34524045	0.05598796	6.16633378	6.99E-10	1.20E-08
HLA-F	406.222193	-0.2737087	0.10382278	-2.6363071	0.00838139	0.03011342
HLA-L	193.637759	-0.5426982	0.14362589	-3.778554	0.00015774	0.00097965
HMCN1	2057.64749	-1.3610265	0.05767946	-23.596379	4.20E-123	5.10E-120
HMG20A	1725.97386	0.26279524	0.05875112	4.47302487	7.71E-06	6.48E-05
HMGB2	11336.8485	0.11868569	0.04347651	2.72988111	0.00633572	0.02379515
HMGCR	5880.26522	0.1180315	0.03697186	3.19246844	0.00141062	0.00663776
HMGN2	36219.1945	0.10499608	0.03037369	3.45681079	0.00054661	0.00293143
HMMR	2847.90061	0.23975072	0.04650809	5.15503263	2.54E-07	2.86E-06
HNRNPA0	10197.177	0.1275688	0.03873355	3.29349598	0.0009895	0.00487466
HNRNPA3	25775.2068	-0.1895113	0.04921318	-3.8508242	0.00011772	0.00075238
HNRNPC	35558.6041	0.08219251	0.03171378	2.59169685	0.00955039	0.03350814
HNRNPD	11376.5785	-0.4021653	0.04436563	-9.0647936	1.25E-19	5.66E-18
HNRNPDL	9590.43903	-0.3574174	0.04175321	-8.5602369	1.13E-17	4.46E-16
HNRNPF	23566.7293	0.15124282	0.03049326	4.95987784	7.05E-07	7.33E-06
HNRNPH1	29288.1608	-0.3687639	0.03674176	-10.036643	1.05E-23	6.19E-22
HNRNPLL	2824.02812	0.14913671	0.05112319	2.91720249	0.00353186	0.01448493
HNRNPM	16256.554	0.1831908	0.03151585	5.81265605	6.15E-09	9.27E-08
HNRNPUL1	20441.4025	0.16691362	0.03208064	5.20293911	1.96E-07	2.25E-06
HOMER2	3620.03108	0.25655866	0.04331134	5.92359098	3.15E-09	4.97E-08
HOMEZ__PPI	2359.87576	-0.2707974	0.05043156	-5.3696024	7.89E-08	9.70E-07
HOXA10__HO	413.612553	0.5550353	0.10442264	5.3152775	1.06E-07	1.29E-06
HOXB7	578.469365	0.3956981	0.08604473	4.59874871	4.25E-06	3.78E-05
HOXC10__HO	713.63459	0.38714328	0.07899411	4.90091356	9.54E-07	9.68E-06
HOXC13	1099.01073	0.41652613	0.06632779	6.27981355	3.39E-10	6.05E-09
HOXC13-AS	233.918623	0.44349004	0.13375764	3.31562397	0.00091439	0.00456043
HPN	197.421984	1.41451279	0.14745459	9.59287057	8.57E-22	4.48E-20
HPRT1	4521.4048	-0.2700558	0.04402748	-6.1338006	8.58E-10	1.46E-08
HPS6	1822.62568	0.35357895	0.06671322	5.29998345	1.16E-07	1.39E-06
HPSE	243.898777	0.64168887	0.12956074	4.95280357	7.32E-07	7.59E-06
HS2ST1__RP	2345.18261	0.19687182	0.04869243	4.04317081	5.27E-05	0.00036491
HS3ST3B1	443.748332	-0.2996965	0.09892692	-3.0294734	0.00244981	0.01062806
HS6ST2	222.973762	-0.9925144	0.13857242	-7.1624237	7.93E-13	1.95E-11
HSD11B2	205.147459	0.53137912	0.14286379	3.7194809	0.00019963	0.00120773
HSD17B10	3933.19068	0.19517637	0.0415519	4.69717078	2.64E-06	2.45E-05
HSD17B12	1126.76705	-0.3473562	0.06404555	-5.4235801	5.84E-08	7.37E-07
HSD17B8	406.55342	-0.3107358	0.10430144	-2.9792088	0.00288994	0.01224329
HSP90AA1	183278.049	0.07389031	0.0297993	2.4795984	0.01315304	0.04325205

HSP90AB1	124087.485	0.18249397	0.0281081	6.49257608	8.44E-11	1.64E-09
HSP90B1	29960.0539	0.10919189	0.02965345	3.68226561	0.00023117	0.00137795
HSPA6	96.9968621	-0.7107153	0.20318123	-3.497938	0.00046887	0.00257036
HSPA8	99734.1237	0.18824567	0.03152736	5.9708672	2.36E-09	3.78E-08
HSPB1	84885.8757	0.2102958	0.0403505	5.2117274	1.87E-07	2.16E-06
HSPB8	1158.96779	-1.0033634	0.06937399	-14.463106	2.07E-47	4.02E-45
HSPD1	42567.0205	0.09839502	0.02981321	3.30038329	0.00096553	0.00478279
HSPD1P11	236.077375	-1.0150795	0.14000729	-7.2501907	4.16E-13	1.06E-11
HSPH1	17746.8429	0.14674007	0.03092735	4.74466953	2.09E-06	1.98E-05
HTT	9417.58262	0.1542614	0.05051538	3.0537509	0.00226	0.00992743
HYOU1	11164.7365	0.17588403	0.03435378	5.11978725	3.06E-07	3.42E-06
IARS	13255.3607	0.09296754	0.0334343	2.78060409	0.00542579	0.02096499
ICA1	3031.59964	0.11722102	0.04533266	2.585796	0.00971544	0.0339761
ID1	3968.57449	0.78538761	0.05385142	14.5843438	3.53E-48	7.24E-46
ID2	4505.26446	0.3433069	0.04134329	8.30381284	1.01E-16	3.68E-15
ID3	4258.67394	0.28093304	0.04967372	5.6555665	1.55E-08	2.17E-07
IDH2	11070.8886	0.16601608	0.03435145	4.83287017	1.35E-06	1.32E-05
IDNK	281.407624	-0.3557811	0.12155721	-2.9268611	0.00342402	0.01410609
IDUA	1304.48025	-0.3113406	0.0692087	-4.4985758	6.84E-06	5.82E-05
IER5L	5061.76257	0.17759229	0.05811285	3.0559901	0.00224319	0.00986548
IFFO1	40146.0595	-0.3437468	0.09509496	-3.6147741	0.00030061	0.00174453
IFI27	6009.7513	-1.397802	0.03784726	-36.932717	1.38E-298	2.52E-294
IFI44	111.561328	-3.3550208	0.24650636	-13.610281	3.48E-42	5.42E-40
IFI44L	41.3819409	-1.8264722	0.32593199	-5.6038446	2.10E-08	2.86E-07
IFI6	6364.4924	-1.4470091	0.04346924	-33.288121	5.74E-243	2.61E-239
IFRD1__LSM	3141.75753	-0.1505031	0.04501113	-3.343686	0.00082673	0.00417812
IFT172	859.227489	-0.2353498	0.0708805	-3.3203738	0.00089897	0.00449102
IFT46	2374.27513	0.28141131	0.05010228	5.61673618	1.95E-08	2.67E-07
IGDCC3	231.036191	-1.0271758	0.14135807	-7.2664817	3.69E-13	9.50E-12
IGF1R	39551.9977	0.09611835	0.03641151	2.63977927	0.008296	0.02985972
IGF2R	7918.55011	0.31222905	0.05028627	6.20903174	5.33E-10	9.26E-09
IGFBP2	4354.90662	0.600936	0.05902514	10.1810172	2.41E-24	1.47E-22
IGFBP3	865.901211	1.04450568	0.07546261	13.8413666	1.43E-43	2.34E-41
IGFBP4	17901.5846	0.30729199	0.03289251	9.34231012	9.43E-21	4.56E-19
IGFBP5	67888.1022	1.14283829	0.03447754	33.1473236	6.19E-241	2.26E-237
IGFL1	79.9844984	-1.1221145	0.22530198	-4.9804912	6.34E-07	6.68E-06
IGHE	30.492058	-1.0194435	0.37486927	-2.7194639	0.00653878	0.02444703
IGHG1	11.9037632	-2.1579706	0.65523267	-3.2934417	0.00098969	0.00487466
IGSF1	9458.4593	0.15576757	0.03617735	4.30566564	1.66E-05	0.00013012
IGSF3	10575.469	0.35035034	0.03722922	9.41062723	4.93E-21	2.43E-19
IGSF9	1204.20479	0.30111696	0.06612807	4.55354225	5.28E-06	4.62E-05
IKBIP	882.95755	0.23528745	0.07229328	3.254624	0.00113543	0.00549015
IL18BP	352.570013	-0.4871197	0.1101923	-4.4206333	9.84E-06	8.05E-05

IL1R1	176.728138	0.53848257	0.14910569	3.61141518	0.00030453	0.00176224
IL20	344.697881	0.53590683	0.10889415	4.9213556	8.59E-07	8.79E-06
IL27RA	1142.5546	0.37532467	0.06517684	5.75855905	8.48E-09	1.25E-07
IL4R	2789.97432	0.22514245	0.04888272	4.60576742	4.11E-06	3.68E-05
IL6ST	1777.45045	-0.3508416	0.0536654	-6.5375744	6.25E-11	1.24E-09
IL9R	1174.67377	-0.3391458	0.06492085	-5.2239884	1.75E-07	2.03E-06
ILF2	15189.5983	0.11569143	0.0346222	3.34153885	0.00083315	0.00420525
IMP3	2936.29391	0.16285894	0.05424288	3.00240196	0.00267858	0.01147317
IMPACT	2068.90134	-0.12604	0.05143345	-2.4505459	0.01426398	0.04612331
IMPDH2	12596.466	0.13991886	0.03328383	4.20380874	2.62E-05	0.00019478
INADL	4125.09794	-0.1084912	0.04302561	-2.5215503	0.0116839	0.03945547
INCENP	3311.69998	0.3231105	0.0515098	6.27279737	3.55E-10	6.31E-09
INF2	8894.56353	-0.3451078	0.04822252	-7.1565702	8.27E-13	2.03E-11
ING5	1961.09323	-0.1635246	0.05448991	-3.0010063	0.00269089	0.01151508
INHA	412.518546	-0.5062961	0.10526459	-4.8097472	1.51E-06	1.47E-05
INHBB	699.811577	-0.9608838	0.07938575	-12.103984	1.01E-33	1.04E-31
INO80	2603.70904	0.11378353	0.04643044	2.45062352	0.0142609	0.04612331
INPP5J	316.473863	0.38347713	0.11903303	3.22160261	0.00127476	0.00609118
INSIG1	6451.5239	0.19582601	0.03866934	5.06411544	4.10E-07	4.49E-06
INTS9	582.459287	0.230823	0.08444014	2.73356975	0.00626519	0.02356864
IP6K1	2413.89747	0.30371076	0.04821268	6.29939622	2.99E-10	5.36E-09
IPO5	6441.74509	0.10709166	0.036011	2.9738599	0.00294079	0.01240973
IPO8	3299.85903	0.20307274	0.0466053	4.35728889	1.32E-05	0.00010458
IPO9	4092.26121	0.19175795	0.04248784	4.51324304	6.38E-06	5.48E-05
IRAK1	16873.0768	0.12390239	0.03956658	3.13149102	0.00173921	0.00794607
IRF3	4897.35876	-0.1392655	0.04453829	-3.1268714	0.00176677	0.00804779
IRF9__RNF3	7243.61137	-0.4197725	0.04170057	-10.06635	7.78E-24	4.64E-22
IRS1	20677.5715	0.17744756	0.03770405	4.70632606	2.52E-06	2.34E-05
IRX2	1490.51864	-0.4033503	0.06400075	-6.3022742	2.93E-10	5.27E-09
IRX3	1781.64943	0.24438183	0.05583354	4.37697167	1.20E-05	9.64E-05
ISG20L2	4239.51149	0.15364943	0.04046513	3.79708198	0.00014641	0.00091749
ISL2	55.6574869	0.96344749	0.281889	3.41782584	0.00063124	0.00331507
ISOC2	3343.60584	0.25253612	0.04485588	5.62994403	1.80E-08	2.49E-07
ISYNA1	3535.0562	0.50502547	0.05179438	9.75058376	1.83E-22	9.89E-21
ITGA2B	102.161361	-0.9036153	0.20811107	-4.3419856	1.41E-05	0.00011136
ITGAM	58.5394288	-0.8107626	0.26013946	-3.1166461	0.00182921	0.0083011
ITGAV	7658.1929	-0.3275187	0.03973862	-8.2418246	1.70E-16	6.08E-15
ITGB1BP1	4515.86266	-0.1858868	0.04525456	-4.1075823	4.00E-05	0.00028545
ITGB5	6961.32858	0.27430649	0.04906842	5.59028629	2.27E-08	3.08E-07
ITGB8	304.243531	-0.4076163	0.11653025	-3.4979439	0.00046886	0.00257036
ITM2C	553.148609	0.58555645	0.09809571	5.96923619	2.38E-09	3.82E-08
ITPR1	512.747728	0.76819247	0.09125109	8.41844733	3.82E-17	1.43E-15
IVD	5319.90776	0.11542442	0.04293461	2.68837711	0.00718003	0.02646464

IVNS1ABP	8372.23697	0.09351104	0.03438481	2.71954489	0.00653718	0.02444606
JADE1	1298.71734	0.16440161	0.06062208	2.71190958	0.00668968	0.02493787
JADE2	5334.97321	0.40673156	0.04149033	9.80304526	1.09E-22	6.04E-21
JAG1	1123.54127	-0.3345192	0.07389914	-4.5266996	5.99E-06	5.17E-05
JAK1	12526.8634	0.33344202	0.03567125	9.34764187	8.96E-21	4.36E-19
JAK2	1552.66017	-0.1879604	0.05602924	-3.3546835	0.00079456	0.00404692
JAK3	89.933903	-0.7203596	0.20729853	-3.4749863	0.00051088	0.00276749
JAKMIP3	176.420957	-0.3821455	0.14918688	-2.5615224	0.01042145	0.0359623
JDP2	633.031459	0.89121978	0.0861695	10.3426357	4.52E-25	2.91E-23
JMJD1C-AS1	124.949206	0.45828262	0.17645031	2.59723324	0.00939781	0.03305549
JMJD7__JMJ	929.405825	-0.3098914	0.07222337	-4.2907357	1.78E-05	0.00013788
JTB	8616.07998	0.19553309	0.03472772	5.63046065	1.80E-08	2.49E-07
JUN	6645.66345	-0.7102878	0.04374052	-16.23867	2.69E-59	8.30E-57
JUND	10390.5459	0.37092487	0.11615089	3.19347413	0.00140572	0.00661981
JUP	27260.9042	0.27542209	0.04298985	6.40667706	1.49E-10	2.80E-09
KANK1	676.522073	0.68004863	0.08068844	8.42807989	3.51E-17	1.33E-15
KANSL1	2135.50655	0.15562036	0.05075496	3.0661114	0.00216863	0.00957451
KAT7	5014.9441	0.22890843	0.04106744	5.57396344	2.49E-08	3.37E-07
KB-1562D12.	157.468128	0.50125121	0.16450581	3.04701226	0.00231128	0.01010404
KBTBD7	1161.41827	0.25997548	0.06275971	4.14239475	3.44E-05	0.00024948
KCNG1	282.318707	0.60113928	0.12975848	4.63275512	3.61E-06	3.25E-05
KCNJ11	514.906133	0.32045205	0.09220382	3.47547477	0.00050995	0.0027641
KCNJ3	234.585746	-1.553878	0.13644866	-11.388005	4.80E-30	4.11E-28
KCNK2	370.456943	-1.1047846	0.1072265	-10.303279	6.81E-25	4.37E-23
KCNK5	883.843751	-0.5758099	0.07509063	-7.6681993	1.74E-14	5.26E-13
KCNMA1	341.505688	0.4122922	0.10981626	3.75438195	0.00017377	0.00106684
KCNMB3	974.561709	-0.2446781	0.06888747	-3.5518519	0.00038253	0.00215014
KCNN4	403.12591	-1.6646354	0.11389339	-14.615733	2.23E-48	4.62E-46
KCNQ1OT1	2411.3752	-0.6834496	0.05361232	-12.747996	3.20E-37	3.91E-35
KCTD10	2886.41268	0.26062591	0.04570704	5.70209522	1.18E-08	1.70E-07
KCTD2	2160.49027	0.22498619	0.05717328	3.93516362	8.31E-05	0.00055066
KCTD3	7230.8545	-0.15054	0.03710496	-4.0571405	4.97E-05	0.00034645
KDELC2	1023.99258	-0.3605479	0.07004922	-5.1470653	2.65E-07	2.98E-06
KDELR3	1024.96406	0.34597415	0.06885669	5.02455362	5.05E-07	5.43E-06
KDM1A	7870.66639	0.19603601	0.03548604	5.52431357	3.31E-08	4.33E-07
KDM1B	3104.35472	0.17195792	0.04766492	3.60764125	0.00030899	0.00178466
KDM2B	3531.43366	0.15961304	0.04181977	3.81668859	0.00013526	0.00085514
KDM3B	7654.48815	0.20213928	0.03863586	5.23190836	1.68E-07	1.95E-06
KDM4A	3914.87091	0.4728228	0.04355503	10.8557564	1.87E-27	1.41E-25
KDM4A-AS1	539.83473	0.32162457	0.09099161	3.5346618	0.0004083	0.00227061
KDM4B	2627.69997	0.18408066	0.04967801	3.70547582	0.00021099	0.00127015
KDM4C__RP:	2130.17896	-0.1611812	0.05004982	-3.2204153	0.00128005	0.00611326
KDM5B	16604.1556	0.19265333	0.03198009	6.024165	1.70E-09	2.78E-08

KHDRBS1	14163.6122	0.12958804	0.03145725	4.11949677	3.80E-05	0.00027269
KHSRP	8484.08407	0.19764295	0.03636393	5.43513729	5.48E-08	6.94E-07
KIAA0100	16303.5182	0.17353836	0.04458918	3.8919389	9.94E-05	0.00064669
KIAA0226L	280.831151	0.30112391	0.11809	2.54995268	0.01077375	0.03698901
KIAA0232	3097.30647	0.13190058	0.04906747	2.68814684	0.00718498	0.02647755
KIAA0319	143.29078	-0.574082	0.16519971	-3.4750789	0.0005107	0.00276736
KIAA0368	9688.5843	-0.2122158	0.04187966	-5.0672745	4.04E-07	4.42E-06
KIAA0391__F	16143.4972	-0.1501098	0.03641725	-4.1219419	3.76E-05	0.00027013
KIAA0586	1797.29726	0.31212487	0.0541592	5.76309973	8.26E-09	1.22E-07
KIAA0895L	1030.08011	-0.2146057	0.0682765	-3.1431853	0.0016712	0.00767767
KIAA0907	2568.72208	-0.3494806	0.04841781	-7.2180182	5.28E-13	1.32E-11
KIAA1191	6616.16749	0.18641573	0.03588218	5.19521684	2.04E-07	2.34E-06
KIAA1549	1536.65912	0.29720931	0.06374094	4.66276932	3.12E-06	2.84E-05
KIAA1551	1612.07999	0.37676981	0.0581033	6.48448273	8.90E-11	1.73E-09
KIAA1683	1357.1907	-0.8378586	0.06208411	-13.49554	1.66E-41	2.52E-39
KIAA1715	2796.34418	-0.2603609	0.04751116	-5.4799955	4.25E-08	5.47E-07
KIDINS220	5280.50639	0.14371586	0.04316559	3.32940777	0.00087031	0.00437168
KIF11	5277.47783	0.10410574	0.04015175	2.5928073	0.00951961	0.03342566
KIF12	437.990401	-0.8496835	0.09921024	-8.5644736	1.09E-17	4.30E-16
KIF13A	3533.09732	0.13235533	0.05263374	2.51464802	0.01191513	0.04006833
KIF13B	1398.56604	0.26912823	0.05877088	4.57927845	4.67E-06	4.13E-05
KIF14	3283.18213	0.29221992	0.04718609	6.19292554	5.91E-10	1.02E-08
KIF15	1723.70787	0.22864468	0.05777888	3.95723629	7.58E-05	0.00050681
KIF18A	993.763875	0.29124412	0.07354439	3.96011325	7.49E-05	0.00050239
KIF20A	4553.89998	0.51253636	0.04033932	12.7056284	5.50E-37	6.69E-35
KIF21A	3744.45834	0.17610548	0.04389027	4.01240393	6.01E-05	0.00041077
KIF2C	2620.32395	0.39464003	0.05687387	6.93886374	3.95E-12	8.89E-11
KIF4A	2708.07006	0.35848958	0.04657615	7.69684891	1.39E-14	4.24E-13
KIF5C	1559.27366	1.02206548	0.05865018	17.4264671	5.20E-68	1.97E-65
KIFC1	3569.04496	0.13718797	0.04742311	2.89285059	0.00381763	0.01550003
KIFC2	3067.98624	-0.6545359	0.0631187	-10.369921	3.40E-25	2.21E-23
KIFC3	334.404362	-0.4828832	0.10972426	-4.4008789	1.08E-05	8.75E-05
KIT	63.1663435	-1.0382797	0.2502304	-4.1492949	3.34E-05	0.00024295
KLF12	256.933907	-0.4512684	0.12839533	-3.5146791	0.00044029	0.00243131
KLF16	1146.96335	0.2183753	0.0640396	3.41000423	0.00064962	0.00339695
KLF3-AS1	113.270921	-0.9325218	0.19591532	-4.759821	1.94E-06	1.84E-05
KLF4	5635.49053	-0.2375003	0.0418214	-5.6789168	1.36E-08	1.92E-07
KLF7	775.206229	-0.7447681	0.07615557	-9.7795615	1.38E-22	7.52E-21
KLHDC3	5874.41213	0.19502807	0.03884418	5.02077993	5.15E-07	5.53E-06
KLHDC7B	78.1936365	-1.3892969	0.2376828	-5.8451723	5.06E-09	7.75E-08
KLHL12	3913.36033	0.23042409	0.04813683	4.78685659	1.69E-06	1.63E-05
KLHL13	434.863417	-0.5483722	0.11480791	-4.7764327	1.78E-06	1.71E-05
KLHL17	684.945706	-0.5802805	0.08786296	-6.6043821	3.99E-11	8.14E-10

KLHL25	949.217125	0.24575174	0.07107385	3.45769565	0.00054482	0.00292511
KLHL4	741.542303	1.23149541	0.08124845	15.1571555	6.79E-52	1.63E-49
KLHL8	1451.10365	0.18387313	0.05975621	3.07705478	0.00209057	0.00928836
KLHL9	2108.89025	0.19339331	0.0516299	3.74576202	0.00017985	0.00110082
KLK10	181.490506	-0.775311	0.14864238	-5.2159486	1.83E-07	2.11E-06
KLK6	187.693697	-0.6735439	0.14543947	-4.6310939	3.64E-06	3.28E-05
KMT2B	4905.18508	0.13774459	0.04936772	2.79017549	0.00526795	0.0204374
KMT2E	5926.27831	0.10656848	0.03797141	2.80654501	0.00500759	0.01955631
KNSTRN	1978.77699	0.25502339	0.05347237	4.76925496	1.85E-06	1.77E-05
KNTC1	4540.28364	-0.1921428	0.04613804	-4.1645197	3.12E-05	0.00022839
KPNA2	29279.477	0.34084567	0.02965529	11.493589	1.42E-30	1.26E-28
KRT15	814.379877	-0.7336456	0.07477122	-9.8118713	1.00E-22	5.55E-21
KRT16	178.087022	-0.6139164	0.15308513	-4.0102943	6.06E-05	0.00041384
KRT17	615.048325	-1.24967	0.09071793	-13.775336	3.59E-43	5.79E-41
KRT18P28	177.190036	-0.5402717	0.1569057	-3.4432891	0.00057469	0.0030595
KRT19	138125.426	-0.1158284	0.04406721	-2.6284496	0.00857751	0.0306611
KRT32	12.5407769	-1.9770383	0.68279826	-2.8954941	0.00378562	0.01538035
KRT6A	292.985886	0.73820405	0.11869975	6.21908663	5.00E-10	8.73E-09
KRT7	380.350365	-0.6289939	0.10723241	-5.8657069	4.47E-09	6.90E-08
KRT81	1606.26212	-0.7189264	0.05728169	-12.550719	3.94E-36	4.69E-34
KRT86	292.456695	-0.4273564	0.12003097	-3.5603844	0.00037031	0.00209178
KRTAP5-10	20.3461806	-1.1465044	0.45569673	-2.5159373	0.01187163	0.03996074
KRTCAP2__M	6481.44998	-0.4983323	0.04528549	-11.004238	3.65E-28	2.81E-26
KTI12__TXNI	3658.03912	0.24647513	0.05395156	4.56845278	4.91E-06	4.32E-05
KTN1	14170.125	-0.1337646	0.0332549	-4.0224033	5.76E-05	0.00039534
KYNU	8141.01882	-0.3184947	0.04089441	-7.7882214	6.80E-15	2.13E-13
L3MBTL1__R	204.744802	-0.8619602	0.14447357	-5.9662141	2.43E-09	3.88E-08
LA16c-358B7	919.94784	-0.3714009	0.07320203	-5.0736423	3.90E-07	4.29E-06
LA16c-380H5	106.99456	-0.9180429	0.19952899	-4.60105	4.20E-06	3.74E-05
LAGE3	2420.5241	0.37493599	0.05011044	7.48219312	7.31E-14	2.03E-12
LAMA3	590.369809	-0.4699885	0.08753924	-5.368889	7.92E-08	9.73E-07
LAMB3	629.923311	-0.9082621	0.08840427	-10.273962	9.23E-25	5.81E-23
LAMC1	6376.11557	0.15193132	0.04341296	3.49967683	0.00046582	0.0025553
LAMP3	69.7430723	-1.2503511	0.24029442	-5.2034132	1.96E-07	2.25E-06
LAMTOR2	1856.78397	0.30629629	0.06137495	4.99057518	6.02E-07	6.37E-06
LAMTOR5	3349.91348	-0.16429	0.05072871	-3.2385996	0.00120118	0.00577136
LAPTM4B	17144.4424	0.35743274	0.03717705	9.61433957	6.96E-22	3.65E-20
LARP1	22484.7908	0.19421303	0.04009164	4.84422706	1.27E-06	1.26E-05
LARP4	6408.09516	0.14814755	0.03864157	3.83389112	0.00012613	0.00080136
LARP7	2570.935	-0.2045319	0.04825849	-4.2382571	2.25E-05	0.00016987
LARS2	1272.8666	0.42409823	0.06348536	6.6802521	2.39E-11	4.99E-10
LCA5	229.967954	0.41040995	0.13366272	3.07048938	0.00213708	0.00945585
LCN12	61.5900595	-1.2368786	0.26141778	-4.7314248	2.23E-06	2.10E-05

LCN2	621.230708	0.46330853	0.08449499	5.48326605	4.18E-08	5.39E-07
LDB1	3772.79397	0.22127098	0.04226974	5.23473678	1.65E-07	1.92E-06
LDHA	27760.003	-0.657292	0.03205582	-20.504609	1.96E-93	1.32E-90
LDHAP4	312.264552	-0.4828267	0.11523932	-4.1897739	2.79E-05	0.00020597
LDHD	710.362855	-0.2225621	0.07808706	-2.8501795	0.00436946	0.01736923
LDLR	6361.96057	0.29921709	0.04077362	7.3384979	2.16E-13	5.72E-12
LDLRAD4	1404.02994	0.24773763	0.06023757	4.11267614	3.91E-05	0.0002801
LDOC1	6631.33055	0.14973583	0.03697692	4.04944038	5.13E-05	0.00035635
LEMD1	24.2126078	-1.433847	0.4168641	-3.4396029	0.00058257	0.00309335
LENG8	6219.77217	-0.738152	0.04836495	-15.262127	1.37E-52	3.46E-50
LGALS1	1695.33218	-0.4742711	0.05880871	-8.0646416	7.35E-16	2.56E-14
LGALS3	15849.8098	-0.4371547	0.03416597	-12.795032	1.75E-37	2.15E-35
LGALS8	2432.16877	-0.3936459	0.04950125	-7.9522419	1.83E-15	6.21E-14
LGMN	2205.32456	0.27104819	0.05286431	5.12724369	2.94E-07	3.30E-06
LHFPL2	1401.49148	-0.1579899	0.0642629	-2.4584928	0.01395216	0.04539679
LHX1	275.373709	-0.4202244	0.12317498	-3.4116054	0.00064582	0.00337996
LIF	304.053114	-0.8303179	0.11965469	-6.9392841	3.94E-12	8.87E-11
LIFR	535.921369	0.23168907	0.0929149	2.49356201	0.01264685	0.04194301
LIG3	1602.77369	0.23577978	0.05750668	4.10004155	4.13E-05	0.00029376
LIMCH1	170.995036	0.44115639	0.15993705	2.75831262	0.00581006	0.02215868
LIME1__RP4	13302.304	-0.1085425	0.04314799	-2.5155872	0.01188343	0.03998744
LIMK1	2574.96917	0.28468879	0.05000142	5.693614	1.24E-08	1.77E-07
LIN7A	544.823369	-0.9742627	0.10245109	-9.5095395	1.92E-21	9.75E-20
LINC-PINT	165.496971	-0.5127197	0.15621019	-3.2822425	0.00102985	0.00503845
LINC00052	3887.45131	-1.0685944	0.04808509	-22.222986	2.06E-109	2.09E-106
LINC00094	1418.12151	0.19920775	0.05795188	3.43746803	0.00058718	0.00311421
LINC00173	97.6159819	-1.8386841	0.23410895	-7.8539675	4.03E-15	1.31E-13
LINC00174	527.337311	-0.2936588	0.10045975	-2.9231489	0.00346511	0.01425282
LINC00294	217.999472	-0.4333938	0.13491735	-3.2122911	0.00131681	0.00626584
LINC00342	436.634622	-0.6284162	0.10157236	-6.1868825	6.14E-10	1.06E-08
LINC00461__	162.270355	-0.4321186	0.15866942	-2.7233894	0.00646158	0.02420307
LINC00482	64.699588	-1.2343086	0.25492881	-4.8417777	1.29E-06	1.27E-05
LINC00504	912.329062	-0.2944288	0.0795517	-3.7011008	0.00021467	0.00128842
LINC00511	510.62094	0.71519052	0.09411136	7.59940708	2.97E-14	8.79E-13
LINC00641	502.759411	-1.0010323	0.0979283	-10.222094	1.58E-24	9.79E-23
LINC00672	46.5522505	-1.374043	0.29682578	-4.6291228	3.67E-06	3.31E-05
LINC00847	1462.43359	0.26247921	0.0592647	4.42893028	9.47E-06	7.78E-05
LINC00865	95.1609617	1.110078	0.22933435	4.84043502	1.30E-06	1.28E-05
LINC00894	104.585163	-1.1193978	0.1982521	-5.646335	1.64E-08	2.29E-07
LINC00992	447.539246	0.35071173	0.10094041	3.4744434	0.00051191	0.00277145
LINC01002__	403.039612	-0.7225189	0.11448731	-6.3109078	2.77E-10	5.01E-09
LINC01003	747.829796	0.373378	0.08172873	4.56850369	4.91E-06	4.32E-05
LINC01006	2293.2429	0.28260551	0.05239393	5.39385989	6.90E-08	8.57E-07

LINC01016	579.786255	0.35282786	0.08585986	4.10934595	3.97E-05	0.00028372
LINC01030	16.7719904	-1.7487117	0.53082569	-3.2943239	0.00098659	0.00486464
LINC01087	571.625407	-0.7854146	0.09634808	-8.151845	3.58E-16	1.26E-14
LINC01125	149.319345	-0.5400597	0.18292246	-2.9523967	0.00315318	0.01317704
LINC01132	23.9656094	-1.2435736	0.42567995	-2.9213818	0.00348483	0.01432101
LINC01291	171.338226	-1.5849016	0.16601212	-9.546903	1.34E-21	6.88E-20
LINC01293	356.074572	-1.1386501	0.10890265	-10.455669	1.38E-25	9.32E-24
LINC01341	69.1665793	-1.0034909	0.24518677	-4.0927612	4.26E-05	0.00030185
LINC01355	240.3736	-1.1783901	0.14084242	-8.3667274	5.92E-17	2.19E-15
LINGO4__RC	403.518653	-0.475547	0.10538176	-4.5126117	6.40E-06	5.49E-05
LL22NC03-2H	144.598505	-0.7943521	0.17669979	-4.4954902	6.94E-06	5.89E-05
LLGL1	1878.19712	0.34542987	0.06223191	5.55068745	2.85E-08	3.80E-07
LMBR1	23233.1592	0.23909961	0.03125108	7.65092203	2.00E-14	5.96E-13
LMCD1	744.92305	-0.4103234	0.07705849	-5.3248313	1.01E-07	1.22E-06
LMNA	13509.2917	-0.1658534	0.04243989	-3.9079606	9.31E-05	0.00061072
LMNB1	11497.4909	0.23467583	0.03256311	7.20679955	5.73E-13	1.43E-11
LMNB2__TIN	7616.88237	0.28630224	0.03579721	7.99789354	1.27E-15	4.32E-14
LMNTD2	209.387516	-0.6149908	0.14862002	-4.1380078	3.50E-05	0.00025359
LMO7__UCH	1665.32962	-0.2078465	0.06037732	-3.44246	0.00057645	0.00306711
LMTK2	3519.83452	0.19723761	0.04561917	4.32356824	1.54E-05	0.00012046
LMTK3	1260.77492	-0.4491443	0.06101389	-7.3613444	1.82E-13	4.85E-12
LNX2	2211.52786	0.22186845	0.04896907	4.53078742	5.88E-06	5.08E-05
LOX	41.0504415	-1.5565876	0.33551057	-4.6394593	3.49E-06	3.16E-05
LOXL1-AS1	1602.5303	-0.1945794	0.0584873	-3.3268654	0.00087829	0.00440206
LPAR2	2270.06605	0.2539387	0.06509575	3.90100272	9.58E-05	0.00062607
LPCAT3	1498.84411	0.15421128	0.05769674	2.67279012	0.00752233	0.02751498
LPIN3	2116.92653	-0.5459399	0.05110134	-10.683476	1.22E-26	8.73E-25
LRFN3	1015.0916	0.42674822	0.08106919	5.264	1.41E-07	1.67E-06
LRFN4	2631.19753	0.49010152	0.06898087	7.10489087	1.20E-12	2.91E-11
LRIF1	1574.97833	0.29074945	0.05903069	4.92539453	8.42E-07	8.62E-06
LRIG2	874.141494	-0.4104083	0.07199844	-5.7002385	1.20E-08	1.71E-07
LRP10	15332.3091	0.28757471	0.03675687	7.82369903	5.13E-15	1.64E-13
LRP1B	169.385194	-0.6858278	0.16345894	-4.1957191	2.72E-05	0.00020113
LRP8	2906.87965	-0.1156877	0.04630812	-2.4982169	0.01248198	0.04151704
LRPPRC	15841.0892	0.10087738	0.03169767	3.18248534	0.00146017	0.00683003
LRRC14	1430.18443	-0.1473712	0.06010893	-2.4517355	0.01421691	0.04600742
LRRC37A4P_	90.0854963	0.73968351	0.21715851	3.40619166	0.00065876	0.00344002
LRRC37A6P	187.564375	0.61733515	0.15619117	3.95243307	7.74E-05	0.00051557
LRRC45	1852.28806	-0.2158873	0.0635747	-3.3958048	0.00068427	0.00354561
LRRC58	6504.87901	-0.2669487	0.03735772	-7.1457428	8.95E-13	2.19E-11
LRRC59	14066.7482	0.10687535	0.03149975	3.39289572	0.00069158	0.00357637
LRRC61	981.937339	0.42655655	0.08637462	4.93844771	7.87E-07	8.11E-06
LRRC75A	10742.5517	0.13577923	0.04657027	2.91557778	0.00355031	0.01454421

LRRC8A	3310.07966	0.32127123	0.04574712	7.02276461	2.18E-12	5.08E-11
LRRC8C__LRI	1755.96159	0.12908245	0.05303359	2.43397529	0.01493402	0.04794134
LRRFIP2	3356.03032	0.19236249	0.0450825	4.26689911	1.98E-05	0.0001516
LRRN2	224.158522	-0.345371	0.13405523	-2.5763335	0.00998543	0.03478396
LRWD1	2221.27127	0.29785102	0.05466362	5.44879742	5.07E-08	6.46E-07
LSM14B	7557.30788	0.26408456	0.04048756	6.52260956	6.91E-11	1.36E-09
LSM4	6407.73896	0.2734874	0.04296834	6.36485785	1.95E-10	3.63E-09
LSM8	1485.98572	-0.227245	0.07955919	-2.8563011	0.00428608	0.0171012
LSS	7421.9918	0.21028018	0.04108871	5.11771162	3.09E-07	3.45E-06
LTB4R__LTB4	5295.27095	-0.1786919	0.04565423	-3.914028	9.08E-05	0.00059707
LTBP1	4758.76776	0.28982328	0.04028104	7.19502945	6.24E-13	1.56E-11
LTBP3	4178.33143	0.31684307	0.05436981	5.82755494	5.62E-09	8.55E-08
LTC4S__MAN	6073.46173	0.32709194	0.04613975	7.08915798	1.35E-12	3.24E-11
LUC7L3	10235.75	-0.3366725	0.03754906	-8.9662038	3.07E-19	1.36E-17
LUZP1	1828.84845	0.2631281	0.06014853	4.37463881	1.22E-05	9.74E-05
LY6G6C	32.8177957	-1.3180131	0.3552225	-3.7103873	0.00020694	0.00124761
LYPD3	3733.22075	-0.2814153	0.04744901	-5.9309002	3.01E-09	4.76E-08
LYPD6B	1201.38574	-0.1597455	0.06163068	-2.5919809	0.00954251	0.03348693
LYPLA1	5740.10114	0.13796877	0.04324848	3.19014122	0.00142203	0.0066811
LYSMD2	602.874756	0.25777571	0.08480039	3.03979407	0.0023674	0.01030981
LYSMD3	2241.85498	0.23191585	0.0524025	4.42566409	9.61E-06	7.88E-05
LYSMD4	692.282542	-0.2635754	0.08574448	-3.0739635	0.00211235	0.00936689
M6PR	7139.23867	0.19704296	0.0382478	5.15174655	2.58E-07	2.91E-06
MACC1	1134.56789	-0.8849532	0.07837351	-11.291484	1.45E-29	1.19E-27
MACF1	10365.6995	0.43260353	0.0556392	7.77515775	7.54E-15	2.34E-13
MACROD2	462.327102	-0.2698	0.09736775	-2.770938	0.00558951	0.02147049
MAD2L1	2917.94343	0.13255821	0.04612604	2.8738263	0.00405532	0.01633415
MADD	5707.76846	0.32563901	0.04961228	6.56367831	5.25E-11	1.05E-09
MAF	159.444957	0.5532781	0.16071212	3.44266573	0.00057601	0.00306567
MAF1	6413.0171	0.2291231	0.04084881	5.60905149	2.03E-08	2.79E-07
MAFG-AS1	128.73493	0.52835142	0.18973583	2.78466868	0.00535824	0.02073917
MAGED1	8145.86712	0.26964168	0.03517888	7.66487309	1.79E-14	5.38E-13
MAGED2	13315.4583	0.34330211	0.04035753	8.50651875	1.79E-17	6.92E-16
MAL2	17203.8869	-0.3408973	0.03617901	-9.4225177	4.40E-21	2.18E-19
MALAT1	50032.1757	-0.8778202	0.16317478	-5.3796315	7.46E-08	9.22E-07
MALL	375.289442	-0.3901994	0.10943048	-3.5657287	0.00036285	0.0020547
MAML3	730.361429	0.42304599	0.08830383	4.7907998	1.66E-06	1.60E-05
MAN1B1	5623.94728	-0.1119192	0.04318709	-2.5914963	0.00955596	0.03352122
MAN2A2	4386.93119	-0.275847	0.04356416	-6.3319706	2.42E-10	4.43E-09
MAN2C1	4541.35186	-0.568797	0.04808496	-11.829	2.76E-32	2.70E-30
MANEA	851.31659	0.19424175	0.07154653	2.71490119	0.00662956	0.02472557
MANSC1	1070.03085	0.46725935	0.07070262	6.60879781	3.87E-11	7.92E-10
MAOB	235.765032	-0.7029253	0.13396993	-5.2468888	1.55E-07	1.81E-06

MAP2K3	1972.59428	0.1667097	0.05378412	3.09960856	0.00193777	0.00870491
MAP3K11	5791.00322	0.20832768	0.04740073	4.39503086	1.11E-05	8.97E-05
MAP3K12	2931.48321	-0.3425769	0.06100862	-5.615222	1.96E-08	2.69E-07
MAP3K14	325.083916	-0.5590877	0.12029166	-4.6477677	3.36E-06	3.04E-05
MAP3K3	4182.4843	0.16103568	0.05362031	3.00325913	0.00267105	0.01144628
MAP3K5	1047.96209	0.3811044	0.07097481	5.36957267	7.89E-08	9.70E-07
MAP7	5177.02868	0.16592969	0.04067094	4.07980947	4.51E-05	0.00031695
MAP7D3	1575.43193	0.14255943	0.05845032	2.43898442	0.0147286	0.04739876
MAPK13	1208.11011	0.38055065	0.06659593	5.7143234	1.10E-08	1.59E-07
MAPK15	317.151561	-1.3383137	0.11651875	-11.485823	1.55E-30	1.37E-28
MAPK8IP1	827.173235	0.41549993	0.0732657	5.67113869	1.42E-08	2.00E-07
MAPK8IP3	2482.23336	-0.4505225	0.06063506	-7.4300658	1.09E-13	2.97E-12
MAPK9	5793.47112	-0.1817962	0.04063554	-4.4738229	7.68E-06	6.47E-05
MAPKAPK2	8626.74538	0.12041233	0.03787711	3.1790262	0.00147771	0.00689647
MAPKAPK3	1588.64615	0.25278451	0.05728654	4.41263371	1.02E-05	8.32E-05
MAPKBP1	566.772066	0.21266231	0.08545753	2.48851462	0.0128278	0.04241332
MARCKSL1	9413.87576	0.24494961	0.03747713	6.53597538	6.32E-11	1.25E-09
MARK2	4830.40965	0.22323813	0.0392234	5.6914533	1.26E-08	1.79E-07
MARS	8018.20442	0.4071571	0.03716377	10.9557545	6.24E-28	4.76E-26
MARS2	912.691555	0.1906586	0.07330538	2.60088148	0.00929846	0.03275026
MARVELD1	2944.89866	0.3826161	0.05191351	7.37026045	1.70E-13	4.55E-12
MARVELD2	3321.13045	0.11595038	0.04499416	2.57700964	0.00996592	0.03475206
MASP2	62.7504528	-0.9282909	0.26148513	-3.5500716	0.00038513	0.00216207
MAST3	1301.34182	0.35923137	0.06777566	5.30030022	1.16E-07	1.39E-06
MAST4	2919.75698	-0.3652889	0.05955435	-6.1337062	8.59E-10	1.46E-08
MATN2	1093.48384	0.36391399	0.06545057	5.56013463	2.70E-08	3.62E-07
MB	795.779397	-0.3285557	0.07646692	-4.2967037	1.73E-05	0.00013474
MB21D1	754.212218	0.2229053	0.07608616	2.92964334	0.00339351	0.01400653
MBD6	3836.03456	0.30692569	0.04938349	6.21514751	5.13E-10	8.93E-09
MCCC1	2346.91392	0.12136875	0.04847179	2.50390505	0.0122831	0.04105775
MCFD2	7138.50119	0.18812761	0.03777551	4.98014793	6.35E-07	6.68E-06
MCM2	8900.51855	0.25342562	0.04579005	5.53451317	3.12E-08	4.12E-07
MCM7	20820.0067	0.21279658	0.03366616	6.32078647	2.60E-10	4.74E-09
MDH1	7511.02387	0.14839919	0.03553247	4.17643839	2.96E-05	0.00021754
MDH2	12919.214	0.25360562	0.03326207	7.62446978	2.45E-14	7.29E-13
MDK	5952.79853	0.80194356	0.03820904	20.9883185	8.39E-98	6.12E-95
MDM4	4611.658	-0.4368998	0.05945255	-7.3487149	2.00E-13	5.30E-12
ME1	5392.16765	-0.2989498	0.04077183	-7.3322632	2.26E-13	5.96E-12
MECP2	4984.47743	0.17585165	0.04136441	4.25127889	2.13E-05	0.00016143
MED12	3077.07386	0.29659092	0.05347468	5.54638059	2.92E-08	3.89E-07
MED13	23524.5791	0.10586529	0.03908279	2.70874406	0.00675384	0.02515823
MED14	4358.37382	0.11952118	0.04093916	2.9194828	0.00350613	0.01439233
MED16	1608.3176	0.23584929	0.0695636	3.3904125	0.00069788	0.00360483

MED20	1219.06523	0.16808502	0.06860503	2.45003946	0.01428406	0.04618005
MED21	1187.59312	0.29880293	0.06630255	4.50665805	6.59E-06	5.63E-05
MED25	2328.00967	0.19469427	0.05695215	3.41855893	0.00062954	0.00330711
MED30	557.734077	0.49439311	0.09149136	5.40371365	6.53E-08	8.15E-07
MEGF6	805.172614	-0.6417471	0.08960067	-7.1623026	7.93E-13	1.95E-11
MEIS1	743.592384	-0.5711321	0.07948746	-7.185186	6.71E-13	1.66E-11
MEIS3	1768.3727	-0.3157209	0.05752618	-5.4882996	4.06E-08	5.25E-07
MEPCE	5292.32252	0.3138868	0.04162709	7.54044628	4.68E-14	1.34E-12
MESDC1	3720.40314	0.37457197	0.06228521	6.01381918	1.81E-09	2.95E-08
MEST	4984.13936	0.15275422	0.04125562	3.70262845	0.00021338	0.0012828
MET	2600.61133	0.88706077	0.05882254	15.0802858	2.18E-51	5.10E-49
METAP1	3878.94783	0.13077739	0.04300653	3.04087265	0.00235894	0.01028103
METTL13	4565.74329	0.21418594	0.04130912	5.18495547	2.16E-07	2.46E-06
METTL17	1892.94718	-0.308335	0.05411753	-5.6975077	1.22E-08	1.73E-07
METTL7A	380.817124	0.44665018	0.10274015	4.34737693	1.38E-05	0.00010882
METTL7B	538.856034	0.489632	0.08934241	5.48039846	4.24E-08	5.46E-07
METTL9	7233.67718	0.18218161	0.03776569	4.8239978	1.41E-06	1.37E-05
MEX3A	1845.89159	0.63667418	0.05631482	11.3056248	1.23E-29	1.02E-27
MEX3B	870.682932	0.55905883	0.07458858	7.49523383	6.62E-14	1.85E-12
MEX3C	1955.45485	0.31544428	0.05145009	6.13107353	8.73E-10	1.48E-08
MFAP2	520.918971	0.96480235	0.10220483	9.43989002	3.73E-21	1.85E-19
MFI2-AS1	167.815091	-0.505015	0.15200445	-3.32237	0.00089256	0.00446496
MFSD6	1881.22303	0.40628741	0.05555514	7.31322788	2.61E-13	6.80E-12
MFSD8	1010.83674	-0.3598447	0.07079727	-5.0827479	3.72E-07	4.12E-06
MGA	4142.1853	0.13599917	0.0544493	2.49772128	0.01249944	0.04155996
MGAT3	31.2979685	-1.3866397	0.37570122	-3.6908044	0.00022355	0.00133644
MGAT4B	8439.48973	0.14876187	0.04798406	3.10023524	0.00193367	0.00869079
MGAT4EP	81.5802965	-0.9952895	0.22919393	-4.3425646	1.41E-05	0.00011112
MGAT5	5958.28047	0.21938308	0.05148824	4.26083871	2.04E-05	0.00015518
MGEA5	10827.5585	-0.2725864	0.03438931	-7.9264879	2.25E-15	7.57E-14
MGLL	1635.93351	-0.6085046	0.06169372	-9.8633158	6.00E-23	3.41E-21
MGRN1	4308.42314	0.16893646	0.04545079	3.71690905	0.00020168	0.00121847
MGST1	10395.5654	-0.2215227	0.03869696	-5.7245511	1.04E-08	1.50E-07
MIB2	1398.62099	-0.4293423	0.07028477	-6.1086113	1.01E-09	1.68E-08
MICA	1454.33722	0.24536924	0.0633454	3.87351303	0.00010728	0.00069243
MICALL1	1240.44538	0.21251951	0.06507022	3.26600288	0.00109077	0.00529529
MICALL2	3362.8681	-0.5829574	0.04905923	-11.882726	1.46E-32	1.45E-30
MICB	1032.19683	0.28225483	0.06671698	4.23062974	2.33E-05	0.00017501
MICU1	2458.24557	0.20439233	0.04747349	4.30539901	1.67E-05	0.00013016
MIEN1	1184.18327	0.29517718	0.06761926	4.36528295	1.27E-05	0.00010118
MIF4GD	1205.84418	0.18108899	0.06362721	2.8460936	0.00442592	0.01757069
MIOS	2003.73115	0.18049241	0.05389504	3.34896156	0.00081115	0.0041164
MIR193BHG	207.578147	-0.6151898	0.14037164	-4.382579	1.17E-05	9.45E-05

MIR210HG	234.943466	-1.8080037	0.14748095	-12.259235	1.50E-34	1.61E-32
MIR22__MIR	475.690497	-0.4405785	0.09574903	-4.6013885	4.20E-06	3.74E-05
MIR34A__MI	479.628709	-0.3684446	0.09565903	-3.8516449	0.00011733	0.00075038
MIR3916__R	1860.52532	-0.44393	0.05628298	-7.8874643	3.08E-15	1.02E-13
MIR424__MI	341.328813	-1.6559351	0.11443735	-14.470233	1.87E-47	3.66E-45
MIR6511B2_	886.743776	-0.4047891	0.07478149	-5.4129583	6.20E-08	7.76E-07
MIR9-3HG	375.597449	-0.3231827	0.10521537	-3.0716297	0.00212894	0.00942438
MIR92B__RF	44.673464	-0.8519355	0.29461736	-2.8916678	0.00383203	0.0155494
MIRLET7DHG	153.426979	-0.5402278	0.1606936	-3.3618504	0.00077422	0.00395919
MITF	394.985377	1.41414641	0.10582765	13.3627314	9.98E-41	1.46E-38
MKI67	21367.1376	0.23554713	0.04982867	4.72714063	2.28E-06	2.14E-05
MKNK1__MC	1911.94513	-0.1823561	0.05233481	-3.4844125	0.00049322	0.00268538
MLKL	935.404869	0.25263861	0.06811262	3.70913085	0.00020797	0.00125278
MLLT1	3561.09894	0.2457164	0.04945339	4.9686467	6.74E-07	7.07E-06
MLPH	25146.3627	-0.3215034	0.03269989	-9.83194	8.20E-23	4.62E-21
MLX	2029.9815	0.22438268	0.05291153	4.24071424	2.23E-05	0.00016844
MLXIP	5917.83805	0.24680351	0.04176206	5.9097539	3.43E-09	5.37E-08
MLXIPL	448.039056	-0.4742738	0.0971892	-4.8799026	1.06E-06	1.07E-05
MMACHC	612.241412	0.40318179	0.08424414	4.78587371	1.70E-06	1.63E-05
MMD	307.011403	0.6326528	0.11984529	5.27891229	1.30E-07	1.55E-06
MMEL1	500.082796	-0.2859856	0.09857783	-2.9011146	0.00371838	0.0151646
MMP13	92.8713439	-1.5581443	0.2164131	-7.1998613	6.03E-13	1.51E-11
MMP16	152.473299	-0.7888441	0.16863426	-4.6778399	2.90E-06	2.66E-05
MMP19	654.564173	-0.6283514	0.08659837	-7.2559266	3.99E-13	1.02E-11
MMP25-AS1	686.386888	-0.6690655	0.08057448	-8.3036909	1.01E-16	3.68E-15
MMP9	68.0263991	-0.6751086	0.24626184	-2.7414259	0.00611732	0.02309382
MMRN2	787.549398	-0.7036035	0.07516075	-9.3613151	7.88E-21	3.85E-19
MOCOS	1340.5417	0.24631108	0.05987114	4.11402012	3.89E-05	0.00027869
MOK	514.159201	-0.2952111	0.08982275	-3.2865963	0.00101406	0.0049808
MON1B__SY	4688.41374	0.21074805	0.03998305	5.27093447	1.36E-07	1.61E-06
MORC2	4684.59773	0.23990362	0.04280728	5.60427111	2.09E-08	2.86E-07
MORF4L1	23235.5116	0.0909124	0.03738064	2.43207198	0.01501272	0.04811777
MORF4L2	24841.6368	-0.1398977	0.03502862	-3.9938125	6.50E-05	0.00044106
MORF4L2-AS	77.7704206	-0.7959188	0.22722063	-3.5028457	0.00046032	0.00253042
MPC2	1813.89075	0.31998726	0.05499964	5.81798842	5.96E-09	9.01E-08
MPDZ	2000.76102	0.16246336	0.06165352	2.63510276	0.00841118	0.03017302
MPG	1194.26013	0.16647143	0.06560797	2.53736602	0.01116901	0.03805227
MPP2	492.416223	0.56748289	0.09458851	5.99949076	1.98E-09	3.21E-08
MPP3	324.518872	-0.4724379	0.11356389	-4.160107	3.18E-05	0.00023257
MPP5	3073.89969	0.14164325	0.04453859	3.18023676	0.00147155	0.00687476
MPZL1	3180.00128	0.1304553	0.04965443	2.62726392	0.00860746	0.030732
MPZL2	1955.55435	-0.7221694	0.05300721	-13.623984	2.88E-42	4.57E-40
MRC2	1093.25314	0.86427799	0.07292306	11.8519155	2.10E-32	2.06E-30

MREG__PEC	9334.07086	0.12106773	0.03473531	3.48543725	0.00049133	0.00267672
MRGBP	3630.94093	0.17189815	0.04472724	3.84325384	0.00012141	0.00077408
MROH1	4242.97379	-0.1750082	0.04976115	-3.5169652	0.00043651	0.00241339
MRPL10	4964.71489	0.16460811	0.03875208	4.24772274	2.16E-05	0.00016373
MRPL11	2756.80643	0.1838527	0.05180258	3.54910348	0.00038655	0.00216803
MRPL12__RF	8198.68675	0.13123285	0.04311698	3.04364654	0.0023373	0.01020796
MRPL27	2465.42924	0.14776185	0.04752576	3.10908991	0.00187665	0.00848259
MRPL37	3782.12809	0.15254947	0.04377777	3.4846331	0.00049281	0.00268397
MRPL4	3332.67152	0.12914203	0.04735697	2.72699111	0.00639148	0.02397987
MRPS12	2564.36263	0.1381064	0.05179749	2.66627581	0.00766967	0.02801459
MRPS27	3796.16462	0.14536475	0.04351652	3.34044976	0.00083643	0.00421776
MRPS28__RI	10637.5877	-0.3155652	0.03712537	-8.4999895	1.90E-17	7.28E-16
MRPS6__SLC	2818.90501	0.19735592	0.04675109	4.22141847	2.43E-05	0.00018112
MRPS7	3796.02242	0.15497031	0.04254758	3.64228264	0.00027023	0.0015859
MSANTD2	401.308311	-0.4321436	0.10378084	-4.1640018	3.13E-05	0.00022882
MSH5__MSH	856.650415	-0.8965815	0.07800121	-11.494456	1.41E-30	1.25E-28
MSRB1	2267.35508	-0.2115035	0.05578829	-3.7911808	0.00014993	0.00093658
MSS51	78.660822	-0.8507772	0.22851038	-3.7231447	0.00019676	0.0011931
MST1	343.504623	-0.8084782	0.10845492	-7.454509	9.02E-14	2.48E-12
MST1L	34.3339516	-0.9527584	0.35202735	-2.7064898	0.00679987	0.02528835
MST1P2	65.873176	-0.8368927	0.24234613	-3.4532949	0.00055378	0.00296325
MST1R	342.779832	-0.4274985	0.12867556	-3.3222973	0.0008928	0.00446496
MT-ND6	137358.157	-0.2506292	0.03901398	-6.4240869	1.33E-10	2.52E-09
MTA2	8104.91033	0.15612777	0.03542797	4.40690732	1.05E-05	8.53E-05
MTF1	1501.6967	0.2430086	0.06226555	3.90277798	9.51E-05	0.00062238
MTFP1__RP4	1309.41113	-0.1547287	0.06308045	-2.4528789	0.01417181	0.04590668
MTHFD2	7945.63973	0.33654949	0.03520515	9.55966685	1.18E-21	6.10E-20
MTMR11	1197.7429	-0.3576783	0.06302066	-5.675572	1.38E-08	1.96E-07
MTSS1	1921.71587	-0.72078	0.05580973	-12.914953	3.71E-38	4.66E-36
MTURN	1070.69681	0.7032684	0.07093989	9.91358217	3.63E-23	2.09E-21
MUC20	1914.4674	-0.2968927	0.05340825	-5.5589292	2.71E-08	3.64E-07
MUC5AC	517.493258	-1.7961699	0.09989023	-17.981436	2.72E-72	1.21E-69
MXD1__SNR	3800.38012	-0.5864653	0.04508134	-13.009048	1.09E-38	1.45E-36
MXD3__RAB	2634.97744	-0.1895762	0.04793651	-3.9547338	7.66E-05	0.00051119
MXD4	3612.59639	0.24815856	0.05598354	4.43270595	9.31E-06	7.67E-05
MXRA7	527.828231	0.29735332	0.08811607	3.3745641	0.00073933	0.00380172
MYADML2__	3004.59352	0.58468336	0.04954275	11.8015922	3.83E-32	3.69E-30
MYBL2	12074.5972	0.22299125	0.03788962	5.88528547	3.97E-09	6.18E-08
MYCL	293.961812	0.5101926	0.11622279	4.38978101	1.13E-05	9.18E-05
MYEOV	154.522391	-0.7139324	0.16641267	-4.2901323	1.79E-05	0.00013819
MYH10	4545.43738	0.15856824	0.0440763	3.59758542	0.00032119	0.00184514
MYL5	346.449844	-0.5592548	0.11538202	-4.8469837	1.25E-06	1.24E-05
MYO15A	46.80893	-0.7532715	0.29630377	-2.5422272	0.01101485	0.03766783

MYO15B	1793.01269	-1.6577452	0.06184766	-26.803686	2.93E-158	4.85E-155
MYO1C	11150.9568	0.11496824	0.03741119	3.07309767	0.00211849	0.00938934
MYO5C	11540.0759	-0.250386	0.03745351	-6.6852487	2.31E-11	4.84E-10
MYO7A	80.6573686	0.6307657	0.22005823	2.86635821	0.00415224	0.01663999
MYO9B	2985.03674	0.1298225	0.04716523	2.75250413	0.00591414	0.0224757
MYSM1	1984.88614	-0.3651101	0.05171383	-7.0602027	1.66E-12	3.96E-11
MZT1	1401.92931	0.16451917	0.06200632	2.65326464	0.00797173	0.02888645
N4BP2L1__N	4362.48101	-0.2260639	0.04143406	-5.4559929	4.87E-08	6.22E-07
NAA20	4336.47205	-0.173931	0.04562385	-3.812283	0.00013769	0.00086903
NAAA__SDA	2722.54736	0.15028186	0.04786959	3.13940152	0.00169293	0.00777164
NAALADL2	1072.99167	0.37235344	0.06492636	5.7350121	9.75E-09	1.42E-07
NAB2	6417.09274	0.51171221	0.03992045	12.818297	1.30E-37	1.61E-35
NABP2	3061.51996	0.11980387	0.04535967	2.64119792	0.00826134	0.02975259
NACC1	8686.56544	0.24826365	0.04304416	5.76765015	8.04E-09	1.19E-07
NACC2	3680.87644	0.34414171	0.04582785	7.50944487	5.94E-14	1.68E-12
NADSYN1	8004.3721	-0.2357798	0.04229078	-5.5752051	2.47E-08	3.34E-07
NAGA	1186.99665	0.30632643	0.06818007	4.4929027	7.03E-06	5.96E-05
NAIF1	777.203305	0.49344995	0.0781836	6.3114256	2.76E-10	5.00E-09
NAIP	540.972723	-0.5048142	0.09003331	-5.6069721	2.06E-08	2.82E-07
NALT1	95.285656	-1.3267798	0.20629376	-6.4315074	1.26E-10	2.41E-09
NANOS1	283.753296	0.4250791	0.123488	3.4422706	0.00057685	0.00306836
NANS	2812.91737	-0.2299229	0.04907987	-4.6846687	2.80E-06	2.58E-05
NAPEPLD	1423.49776	0.16782123	0.05930242	2.82992237	0.00465593	0.01839173
NARF	2788.81479	-0.1561282	0.04646118	-3.3604017	0.00077829	0.0039769
NARS	6544.47403	0.14279801	0.03702235	3.8570756	0.00011475	0.0007365
NAT10	4727.81282	0.22039628	0.04481193	4.91825007	8.73E-07	8.91E-06
NAT8L	835.853151	0.2899356	0.07446549	3.8935566	9.88E-05	0.00064284
NAV2-AS2	108.77604	0.92486564	0.2158188	4.28538031	1.82E-05	0.00014071
NBN	10572.0431	0.21165908	0.03281679	6.44971926	1.12E-10	2.14E-09
NBPF1	3617.53736	-0.1732796	0.04210242	-4.1156677	3.86E-05	0.00027682
NBPF11	918.087064	-0.292579	0.07217961	-4.0534858	5.05E-05	0.00035131
NBPF12	772.534558	-0.2075021	0.07803149	-2.6592095	0.00783243	0.02852335
NBPF8	330.250945	-0.4824935	0.12469339	-3.8694391	0.00010909	0.00070236
NBPF9	1516.1851	-0.3870908	0.06087051	-6.3592495	2.03E-10	3.75E-09
NBR1	6307.69825	0.18499315	0.04038964	4.58021333	4.65E-06	4.12E-05
NCAM2	3264.91513	0.56107914	0.04543223	12.3498029	4.88E-35	5.46E-33
NCAPD2	8552.70873	0.33967611	0.03692648	9.19871255	3.62E-20	1.68E-18
NCAPG2	6398.79524	0.10915935	0.03812624	2.86310327	0.00419514	0.01679345
NCAPH	2291.36104	0.24651629	0.05255311	4.69080324	2.72E-06	2.52E-05
NCDN	2722.79496	0.22243442	0.05238072	4.24649382	2.17E-05	0.00016456
NCK2	1438.84344	0.22769189	0.06535697	3.48381977	0.00049431	0.00268893
NCKAP5L	1932.23926	0.30299898	0.05764815	5.25600529	1.47E-07	1.73E-06
NCOA3	80110.8538	0.16009974	0.03959491	4.04344235	5.27E-05	0.00036463

NCOR1	7760.08509	0.13240712	0.0412938	3.20646473	0.00134377	0.00636919
NCS1	3168.54946	0.16809122	0.04613258	3.64365579	0.00026879	0.00157799
NCSTN	3080.14027	0.24772559	0.04586893	5.40072688	6.64E-08	8.27E-07
NDC80	1504.93067	0.1623552	0.05681046	2.85783985	0.00426536	0.0170334
NDE1	1491.19673	0.2964746	0.05950951	4.98196979	6.29E-07	6.63E-06
NDFIP1	4288.06792	-0.2303263	0.04628274	-4.9765053	6.47E-07	6.80E-06
NDRG1	2421.2649	-1.2088576	0.0517733	-23.349058	1.41E-120	1.51E-117
NDRG4	732.091345	0.29250679	0.07913295	3.69639711	0.00021868	0.00130993
NDST1	4056.64744	0.28145783	0.04567354	6.16238292	7.17E-10	1.23E-08
NDUFA4L2	28.0163181	1.20147704	0.38128098	3.15115912	0.00162624	0.00750325
NDUFAF1	629.380384	0.34490525	0.09249489	3.72891142	0.00019231	0.00116769
NDUFAF5	581.267485	-0.2258414	0.0911959	-2.476443	0.01326988	0.04356555
NDUFS2	4062.82637	0.13425071	0.04092071	3.28075234	0.00103531	0.00505835
NDUFS4	1241.11504	-0.1773704	0.07287696	-2.4338343	0.01493983	0.04795158
NEAT1__ma	23884.8423	-0.9615761	0.04353485	-22.087505	4.17E-108	3.80E-105
NEB	158.914568	0.52059764	0.17074175	3.04903544	0.00229577	0.01006036
NECAB1	7336.70181	-1.2421283	0.03952055	-31.429939	7.89E-217	2.40E-213
NECAB3	1152.11662	-0.2552608	0.06681641	-3.8203305	0.00013327	0.0008432
NEDD1	3080.68045	0.23350129	0.0468599	4.98296664	6.26E-07	6.60E-06
NEDD9	4234.18166	-0.4156206	0.04071986	-10.206829	1.85E-24	1.14E-22
NEIL1	425.731063	-0.6555511	0.10320575	-6.3518858	2.13E-10	3.92E-09
NEK2	953.792215	0.18577438	0.06994705	2.65592867	0.00790903	0.02869346
NEK8	409.535836	-0.322859	0.09890663	-3.2642809	0.00109742	0.00532617
NELL2	140.072746	-2.2812138	0.19333757	-11.799123	3.94E-32	3.78E-30
NEO1	6275.16014	0.24055084	0.04557089	5.27860783	1.30E-07	1.55E-06
NES	1018.28407	0.77143217	0.06744797	11.4374413	2.72E-30	2.36E-28
NET1	12086.9241	-0.4323156	0.03256044	-13.277326	3.13E-40	4.43E-38
NETO2	2138.40617	0.13596626	0.05108583	2.6615261	0.00777873	0.02836748
NEU1	4332.6685	0.29533802	0.04192476	7.04447705	1.86E-12	4.41E-11
NEU3	1385.48432	0.24867186	0.06177348	4.02554422	5.68E-05	0.00039128
NEURL1B	2165.06548	0.26667951	0.06418928	4.1545802	3.26E-05	0.00023797
NEUROG2	17.4841923	1.66945873	0.48890137	3.41471476	0.00063849	0.00334738
NFAT5	3570.37795	-0.399182	0.04831499	-8.2620739	1.43E-16	5.17E-15
NFATC2	2095.29119	0.23042763	0.05789828	3.97987025	6.90E-05	0.00046566
NFATC2IP	4285.12227	-0.1923879	0.04166804	-4.617157	3.89E-06	3.49E-05
NFATC4	422.89002	-0.6760966	0.10790439	-6.2657008	3.71E-10	6.58E-09
NFE2L1	18414.5214	0.28489023	0.03650405	7.80434555	5.98E-15	1.90E-13
NFE2L2	5003.2617	0.12913495	0.04136571	3.12178723	0.00179757	0.00817378
NFIB	2531.85461	0.13727027	0.05462972	2.5127399	0.01197976	0.04022077
NFIX	765.360427	0.36085689	0.07739798	4.6623554	3.13E-06	2.85E-05
NFKB1	2760.66761	0.28477786	0.04805807	5.92570282	3.11E-09	4.91E-08
NFX1	3254.83753	-0.1493166	0.04491925	-3.324112	0.00088701	0.00444087
NFYA	2577.75889	0.28939181	0.04736922	6.10927897	1.00E-09	1.67E-08

NFYC	1963.13569	0.2043607	0.0571084	3.57846987	0.00034561	0.00196443
NHEJ1__RP1	692.387689	0.27013125	0.07870987	3.43198721	0.00059918	0.00316769
NHLRC3	1861.99654	-0.2105279	0.05327704	-3.9515689	7.76E-05	0.00051706
NHP2	13393.4749	0.18936724	0.03414937	5.54526313	2.94E-08	3.91E-07
NHSL1	1447.07779	0.19830046	0.06393628	3.1015327	0.00192522	0.00866636
NICN1	372.744428	-0.2759675	0.10567118	-2.6115687	0.00901279	0.03192912
NIPA1	2201.70051	0.14605957	0.05066033	2.88311519	0.00393763	0.01593759
NIPA2	2505.51873	0.17118504	0.04738057	3.6129799	0.0003027	0.00175442
NIPSNAP1__	8214.28406	0.19852746	0.03782912	5.24800566	1.54E-07	1.80E-06
NIPSNAP3A	1235.24286	-0.2867103	0.06422557	-4.464115	8.04E-06	6.73E-05
NKAIN1	916.281986	0.61387172	0.07067997	8.68522937	3.78E-18	1.52E-16
NKIRAS2	1913.29722	0.34000832	0.05493762	6.18898848	6.06E-10	1.05E-08
NKPD1	105.9198	-0.6640087	0.19494709	-3.406097	0.00065899	0.00344002
NKTR	5060.34546	-0.4547944	0.05196701	-8.7515992	2.10E-18	8.68E-17
NLN	4269.02723	0.12591236	0.04259847	2.95579523	0.00311864	0.01304549
NLRX1	581.439192	0.2840844	0.0915956	3.10150717	0.00192538	0.00866636
NMB	493.283204	0.37647236	0.09466307	3.97697187	6.98E-05	0.00047068
NME1__NMI	54532.656	0.10509139	0.03681624	2.8544843	0.00431068	0.01718052
NMRK1	681.154928	-0.2947904	0.08381042	-3.5173479	0.00043588	0.00241064
NMT1	5187.60178	0.156913	0.04147438	3.7833719	0.00015472	0.00096284
NNT	3374.31139	0.29851118	0.04699765	6.35161978	2.13E-10	3.92E-09
NOB1	5082.77016	0.22639928	0.04178921	5.41764893	6.04E-08	7.57E-07
NOL6	3392.00556	-0.1289901	0.04803781	-2.6851789	0.0072491	0.0266761
NOL9	1362.70049	-0.2125051	0.06546945	-3.2458665	0.00117094	0.00563794
NONO	24678.2	0.15145494	0.0305173	4.96292146	6.94E-07	7.26E-06
NOP14	3678.6546	0.15165499	0.04280243	3.54314007	0.00039539	0.00221085
NOP14-AS1	1057.82529	0.22389957	0.06518099	3.43504391	0.00059246	0.00313855
NOTCH1	3118.92465	-0.1548306	0.05079811	-3.0479598	0.00230401	0.01007948
NOTCH2	16882.1199	0.35559113	0.04571982	7.77761449	7.39E-15	2.31E-13
NOTCH3	13852.7454	0.29021273	0.05018405	5.78296722	7.34E-09	1.10E-07
NOXA1	800.662408	-1.024545	0.07756511	-13.208838	7.80E-40	1.09E-37
NPAS2	683.872317	0.46577827	0.08637435	5.39255337	6.95E-08	8.61E-07
NPC1	2997.92456	0.23227083	0.04620211	5.02727821	4.97E-07	5.35E-06
NPEPL1__ST.	28789.5006	-0.1347181	0.02901971	-4.6422974	3.45E-06	3.12E-05
NPIPA1__NP	1471.94244	-0.4383957	0.05984189	-7.3259011	2.37E-13	6.23E-12
NPIPB15	171.232446	-1.2191632	0.15412759	-7.9100909	2.57E-15	8.59E-14
NPIPB3	419.971087	-0.3421221	0.10285812	-3.3261553	0.00088053	0.00441119
NPIPB4	2242.42248	-0.6017829	0.05385245	-11.174662	5.43E-29	4.36E-27
NPIPB5	1842.66241	-0.5271231	0.05251961	-10.03669	1.05E-23	6.19E-22
NPLOC4	8083.42466	-0.1029329	0.03860728	-2.6661518	0.0076725	0.02801932
NPM3	1539.94054	0.18676919	0.06189366	3.01758175	0.002548	0.01100173
NPNT	3021.77981	-0.5110423	0.04710015	-10.850121	1.99E-27	1.49E-25
NPR3	153.126246	-0.4755235	0.15905662	-2.9896494	0.00279298	0.01189609

NPTXR	1505.23046	0.15273668	0.06140033	2.48755465	0.01286247	0.04251119
NQO1	23111.8754	-0.3046482	0.03096521	-9.8384003	7.69E-23	4.36E-21
NQO2	2032.45385	-0.2137872	0.05049746	-4.2336224	2.30E-05	0.00017312
NR2C2	4867.01266	-0.2600684	0.04424086	-5.8784658	4.14E-09	6.42E-08
NR2F2	26916.7828	0.10277928	0.03667653	2.80231774	0.00507369	0.01977632
NR2F2-AS1_	122.848699	-0.876112	0.1814281	-4.8289762	1.37E-06	1.34E-05
NR4A1	1428.76003	-1.2849932	0.06239435	-20.594704	3.06E-94	2.15E-91
NR5A2	656.872584	0.3919989	0.08110403	4.83328504	1.34E-06	1.32E-05
NRARP	1474.1657	0.24298063	0.06313759	3.84843047	0.00011888	0.00075923
NRAV	474.845447	0.43137657	0.10548052	4.08963243	4.32E-05	0.00030547
NRBF2	1118.9281	0.20164788	0.06684751	3.01653522	0.00255682	0.01102933
NRBP1	13124.6617	0.19941509	0.03506582	5.68687876	1.29E-08	1.84E-07
NRBP2	1097.38442	-0.9089078	0.06671789	-13.623151	2.92E-42	4.59E-40
NRCAM	14335.2857	-0.1964462	0.03318859	-5.9190895	3.24E-09	5.09E-08
NRDE2	4248.44298	-0.1943288	0.04160466	-4.6708417	3.00E-06	2.74E-05
NRG4	220.046598	-0.8326919	0.13464659	-6.1842776	6.24E-10	1.08E-08
NRGN	375.572839	0.32913898	0.10667201	3.08552354	0.00203194	0.00905657
NRIP1	15358.8203	0.1060793	0.03827595	2.77143467	0.00558099	0.02144874
NRK	68.8200286	-1.6586364	0.25575298	-6.4853063	8.86E-11	1.72E-09
NRP1	4503.45193	-0.7839687	0.04141915	-18.927686	6.75E-80	3.32E-77
NSD1	10535.8055	0.15704051	0.05259925	2.98560388	0.00283019	0.0120293
NSMCE2	1846.10933	-0.222513	0.05431807	-4.0964812	4.19E-05	0.00029771
NSRP1	1159.0165	-0.21371	0.06676827	-3.2007724	0.0013706	0.00647951
NSUN4	1589.76388	0.15103028	0.05559559	2.71658759	0.00659587	0.02461508
NSUN5P2	633.51048	-0.4940755	0.09105499	-5.4261215	5.76E-08	7.28E-07
NT5C3B	1223.94696	0.34841327	0.06423578	5.42397556	5.83E-08	7.36E-07
NT5DC2	3158.02811	0.34099257	0.051986	6.55931502	5.41E-11	1.08E-09
NT5DC3	1133.30312	-0.2668812	0.07517824	-3.5499798	0.00038526	0.00216216
NT5E	516.6669	-0.8199599	0.091867	-8.9255105	4.44E-19	1.94E-17
NTN4	171.662272	-0.6806498	0.15953245	-4.2665285	1.99E-05	0.00015166
NTN5	91.0771191	-0.7542836	0.20620009	-3.6580181	0.00025417	0.0015023
NUDT13	215.210928	-0.6515064	0.13768824	-4.7317506	2.23E-06	2.10E-05
NUDT19	1866.8183	0.30271332	0.05423602	5.58140713	2.39E-08	3.24E-07
NUDT3__RPS	37815.5323	0.11609914	0.03333011	3.48331062	0.00049525	0.00269325
NUFIP2	7971.00847	0.13211389	0.03826057	3.45300398	0.00055438	0.00296439
NUP160	5942.9339	0.12812905	0.03767898	3.40054434	0.00067252	0.00349464
NUP188__PH	8672.31113	0.14600943	0.03858665	3.78393679	0.00015437	0.00096131
NUP210	13658.1303	0.11387891	0.04633087	2.45794913	0.0139733	0.04543986
NUP43	2281.65511	-0.2195091	0.05192765	-4.2272108	2.37E-05	0.00017725
NUP88	3458.7967	0.1591562	0.04433307	3.59001103	0.00033066	0.00189244
NUP93	2814.61276	0.2080119	0.0506594	4.10608681	4.02E-05	0.00028708
NUPR1	93.7193926	0.74807138	0.21742689	3.44056517	0.0005805	0.00308416
NUS1P2	150.308922	0.51067431	0.16141593	3.1637169	0.00155768	0.00723269

NUSAP1	4162.12716	0.10712802	0.0407743	2.6273416	0.00860549	0.030731
NUTF2	6842.8271	0.10565458	0.03728576	2.83364426	0.00460205	0.01819862
NXF1	3785.64382	0.13355028	0.04353176	3.06788192	0.00215582	0.0095295
NXNL2	497.16573	-0.6178376	0.09373147	-6.5915706	4.35E-11	8.86E-10
NXPH3	1734.0706	0.75860364	0.05380112	14.100145	3.79E-45	6.58E-43
NYNRIN	758.248505	0.57314821	0.08462849	6.77252117	1.27E-11	2.72E-10
OAF	182.646159	0.48862368	0.14946928	3.26905752	0.00107906	0.00524402
OAS1	803.864435	-0.9328525	0.07484762	-12.463356	1.18E-35	1.36E-33
OAS2	425.967035	-1.672458	0.11230819	-14.891683	3.73E-50	8.10E-48
OAT	2724.84831	0.26251823	0.04713937	5.56898017	2.56E-08	3.46E-07
OBFC1	838.263757	-0.2580318	0.07176438	-3.5955412	0.00032372	0.00185619
OCLN	3287.60119	-0.199767	0.05088418	-3.9259166	8.64E-05	0.0005706
OCRL	4451.47694	0.19802697	0.04135494	4.78847208	1.68E-06	1.62E-05
ODC1	4446.13493	0.40538119	0.041212	9.83648316	7.84E-23	4.43E-21
ODF2	6173.9301	0.09105116	0.0366461	2.48460742	0.01296944	0.04278718
OFD1	2205.71692	-0.3346944	0.04876579	-6.8633023	6.73E-12	1.49E-10
OGDH	6698.67072	0.27328922	0.04295765	6.3618298	1.99E-10	3.69E-09
OGFOD1	2629.74199	0.19048623	0.05036326	3.78224574	0.00015542	0.00096655
OIP5	654.065795	0.24696315	0.09013072	2.74005515	0.00614289	0.02316637
OPHN1	1784.51453	0.22214424	0.05541664	4.0086201	6.11E-05	0.00041647
OPN3	1068.83283	-0.6843766	0.07068283	-9.6823601	3.58E-22	1.93E-20
OPRL1	254.2388	0.64678748	0.12462727	5.18977495	2.11E-07	2.40E-06
OR2AE1__TF	2318.11404	0.16768107	0.04940385	3.39408926	0.00068857	0.00356486
ORAI2	3289.43372	0.49042014	0.04702912	10.42801	1.85E-25	1.24E-23
ORC3	1729.43981	-0.243812	0.05475674	-4.4526387	8.48E-06	7.06E-05
ORMDL1	2012.93783	-0.1803703	0.05905511	-3.0542703	0.00225609	0.00991743
OSBP	2977.63683	0.25062502	0.04704398	5.32746187	9.96E-08	1.21E-06
OSBP2	617.243451	0.23488281	0.08232632	2.85307059	0.0043299	0.01723765
OSBPL7	1916.67783	-0.2325263	0.05793199	-4.0137814	5.98E-05	0.00040868
OSBPL9	4669.11478	-0.2238033	0.04043621	-5.5347245	3.12E-08	4.12E-07
OSGEP	1529.58162	-0.5290698	0.05951312	-8.8899684	6.11E-19	2.64E-17
OSR2	2109.60282	0.1419195	0.0540597	2.6252365	0.00865888	0.03087329
OSTC	3574.78243	0.12200182	0.04909726	2.48490086	0.01295875	0.04275966
OTUB2	355.540344	0.34423426	0.11109349	3.0985999	0.00194437	0.00872814
OXTR	421.326568	-0.2443442	0.09942641	-2.4575381	0.0139893	0.04546086
P2RX2	111.788438	-1.4901503	0.19404472	-7.679417	1.60E-14	4.82E-13
P2RX5__P2R	3811.13868	0.14327444	0.0490261	2.92241153	0.00347332	0.01428274
P2RY2	836.529624	-0.3896401	0.07815586	-4.9854243	6.18E-07	6.52E-06
P3H4	1326.83238	0.34255835	0.06254643	5.47686493	4.33E-08	5.56E-07
P4HA1	3480.88076	-0.4979172	0.04628532	-10.757562	5.46E-27	4.01E-25
P4HA2	2813.25201	-0.1627676	0.04601431	-3.5373253	0.0004042	0.00225251
P4HB	31222.2492	0.13500956	0.03398223	3.97294655	7.10E-05	0.000478
P4HTM__WD	13381.9949	0.11528262	0.03677076	3.13517063	0.00171754	0.00786481

PABPC1	195217.043	0.21931155	0.0272403	8.05099623	8.21E-16	2.85E-14
PABPC1L	2658.39226	-0.3236895	0.04637008	-6.9805677	2.94E-12	6.79E-11
PABPN1L	27.2475378	-1.5177122	0.4190319	-3.6219491	0.00029239	0.00170171
PACS1	10207.4141	0.12322282	0.03955044	3.1155869	0.00183579	0.0083289
PADI2	379.647299	-0.7041374	0.10616475	-6.6324975	3.30E-11	6.80E-10
PADI4	10.9891805	-1.9121882	0.6593553	-2.9000877	0.00373058	0.01520417
PAEP	15.6807073	-3.2248969	0.64709643	-4.983642	6.24E-07	6.58E-06
PAF1	4926.18153	0.13291853	0.04003182	3.32032206	0.00089914	0.00449102
PAIP2	4179.68323	0.3079208	0.04234789	7.27121868	3.56E-13	9.21E-12
PAK1	1741.48729	0.34373051	0.05396764	6.36919674	1.90E-10	3.54E-09
PAK2	15561.2246	0.18395943	0.03171318	5.80072488	6.60E-09	9.91E-08
PALM3	747.567269	-0.4003755	0.08217096	-4.8724692	1.10E-06	1.10E-05
PAM	2572.90755	-0.6011956	0.05200271	-11.560851	6.51E-31	5.99E-29
PAN3	3116.3014	-0.3026561	0.04505105	-6.7180691	1.84E-11	3.90E-10
PANK1	1118.39982	0.2347903	0.06402762	3.66701608	0.0002454	0.00145657
PANX1	888.176334	0.21041476	0.07183178	2.92927117	0.00339758	0.01401934
PAPD5	1530.06257	0.1806883	0.06020025	3.00145441	0.00268693	0.01150353
PAPLN	156.720848	-0.4693	0.17101232	-2.7442468	0.00606499	0.02292476
PAPOLG	1031.35166	0.2858707	0.07002481	4.08242048	4.46E-05	0.00031414
PAPSS1	3697.08843	0.22502212	0.04317591	5.21175131	1.87E-07	2.16E-06
PAPSS2	5094.66307	-0.480036	0.04000089	-12.000633	3.53E-33	3.55E-31
PAQR4	3043.77086	-0.1420179	0.05389058	-2.6353018	0.00840625	0.0301687
PAQR6	594.700577	-1.0813886	0.08944932	-12.0894	1.20E-33	1.23E-31
PARD3	4021.02305	0.14844158	0.04514613	3.28802453	0.00100893	0.00495872
PARD6A	301.573021	0.43490465	0.11961428	3.63589228	0.00027702	0.00162209
PARD6B	14181.4469	-0.4755404	0.10061738	-4.7262247	2.29E-06	2.15E-05
PARG	1810.73943	0.15048698	0.05371835	2.80140711	0.00508803	0.01982374
PARP1	10559.3772	0.41536484	0.03503067	11.8571759	1.98E-32	1.95E-30
PARP10	1469.98894	-0.4919146	0.06590701	-7.463767	8.41E-14	2.33E-12
PARP2	2572.60342	-0.2711506	0.05357021	-5.0615925	4.16E-07	4.53E-06
PARP3	131.827675	-0.5900714	0.18160195	-3.2492569	0.00115707	0.00558296
PARP4	4372.79517	0.24769682	0.05120876	4.8370008	1.32E-06	1.30E-05
PARP8	950.597548	0.29499461	0.07022874	4.2004827	2.66E-05	0.00019734
PARP9	1513.90077	-0.9711652	0.06098856	-15.923728	4.34E-57	1.22E-54
PARS2	415.729853	0.30125313	0.10289191	2.92786012	0.00341304	0.01407038
PARVA	1826.40815	0.17291599	0.05825579	2.96821999	0.0029953	0.01260764
PAX8-AS1__I	1840.19253	-0.2380081	0.05379633	-4.4242449	9.68E-06	7.93E-05
PBX2	3654.98995	-0.2053551	0.05057202	-4.0606461	4.89E-05	0.00034202
PBXIP1	2935.64665	0.4703975	0.04482785	10.4934206	9.26E-26	6.32E-24
PCBP3	518.775713	-0.4305101	0.09695043	-4.4405175	8.97E-06	7.41E-05
PCCB	3681.50523	0.23979714	0.04335431	5.53110278	3.18E-08	4.19E-07
PCDH10	470.670219	-0.8358962	0.09851988	-8.4845442	2.17E-17	8.30E-16
PCDH7	1757.16922	0.43874751	0.05638868	7.78077306	7.21E-15	2.26E-13

PCDHA1__PC	6134.83615	0.21662843	0.0377714	5.73525011	9.74E-09	1.42E-07
PCDHB4	418.935099	0.43362571	0.10416905	4.16271146	3.14E-05	0.00023002
PCED1A	1505.49986	-0.1840543	0.05884175	-3.1279548	0.00176027	0.00802621
PCED1B	798.396656	-0.6160309	0.0792196	-7.7762438	7.47E-15	2.33E-13
PCGF3	4136.16085	0.13364333	0.04437645	3.01158228	0.0025989	0.01117123
PCK2	4050.38913	0.28555494	0.0451272	6.3277785	2.49E-10	4.55E-09
PCNT	7075.2014	0.12565198	0.04553822	2.75926432	0.00579317	0.02210812
PCNXL3	9295.1731	0.1612195	0.04449394	3.62340365	0.00029075	0.00169325
PCP2	101.015348	-0.7413825	0.19645599	-3.773784	0.00016079	0.0009952
PCSK4	143.278183	-0.9139483	0.17951881	-5.0911004	3.56E-07	3.94E-06
PCTP	1341.0816	0.21182146	0.06168971	3.43365934	0.00059549	0.00315187
PCYT2	2738.07945	0.22394995	0.05794828	3.86465241	0.00011125	0.00071526
PDAP1	9071.35186	0.11869585	0.04296237	2.76278616	0.00573103	0.0219206
PDCD4-AS1	195.932552	0.54844679	0.14141161	3.87837175	0.00010516	0.00067995
PDDC1	1397.7299	-0.227948	0.0604611	-3.77016	0.00016314	0.00100805
PDE10A	30.6007565	-2.9343358	0.4381623	-6.6969154	2.13E-11	4.49E-10
PDE4A	132.76143	0.61787242	0.17468022	3.53716309	0.00040445	0.0022532
PDE4C	64.238361	-1.0896794	0.25996335	-4.1916653	2.77E-05	0.00020451
PDE5A	570.398684	-0.5187423	0.08737275	-5.9371179	2.90E-09	4.59E-08
PDE6B	213.551456	0.36094452	0.13458338	2.68193984	0.00731966	0.0268761
PDE7A	2468.14677	-0.2454289	0.04861375	-5.0485481	4.45E-07	4.83E-06
PDIA2	99.1761529	-1.4296127	0.22354985	-6.3950511	1.60E-10	3.01E-09
PDIA4	11383.3251	0.26928247	0.03420825	7.87185796	3.49E-15	1.14E-13
PDIA5__SEC2	2115.26263	0.14919187	0.05085148	2.93387455	0.0033476	0.01385699
PDK1	828.336024	-0.9684479	0.07401313	-13.084812	4.02E-39	5.47E-37
PDK2	1069.53977	0.51439341	0.06509289	7.90245182	2.73E-15	9.10E-14
PDK4	199.018126	-0.6858584	0.14516003	-4.7248434	2.30E-06	2.16E-05
PDS5A	12192.5342	0.08935437	0.0331054	2.69908721	0.006953	0.02577372
PDXDC2P__R	2151.26436	-0.9064927	0.05173881	-17.520556	9.98E-69	3.87E-66
PDZD7	262.172716	-0.4892632	0.1257543	-3.8906279	1.00E-04	0.00064973
PEBP1	13068.194	0.13875807	0.03518794	3.94334221	8.04E-05	0.00053357
PER1__RP11	2321.50849	-0.3312869	0.05028891	-6.5876723	4.47E-11	9.04E-10
PER3	1117.70711	-0.3822164	0.0689888	-5.5402679	3.02E-08	4.01E-07
PERM1	132.100509	-0.7301558	0.17807289	-4.1003198	4.13E-05	0.00029352
PEX11A	1343.90045	-0.1749305	0.05875302	-2.9773881	0.00290716	0.01230195
PEX26__TUB	3130.53012	0.1740194	0.04463072	3.89909478	9.66E-05	0.00063012
PEX3	1205.07558	-0.1789364	0.06502181	-2.7519449	0.00592425	0.02249537
PEX5	1499.35419	0.27667827	0.0608032	4.55039024	5.35E-06	4.68E-05
PFKFB3	4073.41182	-0.1275858	0.04283598	-2.9784733	0.00289688	0.01226416
PFKFB4__UC	666.315404	-0.9770156	0.0851997	-11.46736	1.92E-30	1.69E-28
PFKM	2874.4317	0.17628941	0.04587358	3.84293964	0.00012157	0.0007748
PFKP	15726.3515	-0.1425912	0.03369386	-4.2319648	2.32E-05	0.00017412
PFN1P2	453.903255	-0.7017142	0.12226221	-5.7394201	9.50E-09	1.39E-07

PFN1P6	131.493826	-0.4720199	0.17934374	-2.6319286	0.00849017	0.03042645
PGAM1	15535.2053	-0.1758573	0.03164987	-5.5563375	2.75E-08	3.69E-07
PGAM5__PX	5383.72784	0.12952521	0.04038557	3.20721481	0.00134027	0.00635734
PGAP2	863.446269	0.18195339	0.07243649	2.51190229	0.01200823	0.04027927
PGD	14250.5332	0.22415933	0.03115689	7.19453408	6.27E-13	1.56E-11
PGK1	28060.9843	-0.3724471	0.0306631	-12.146427	5.99E-34	6.24E-32
PGR	7584.41299	-0.3967383	0.04387385	-9.0427052	1.53E-19	6.90E-18
PHACTR4	2475.71921	0.17559263	0.04882277	3.59653126	0.00032249	0.00185089
PHB	10716.9575	0.13297641	0.03436251	3.86981021	0.00010892	0.000702
PHB2	8082.80428	0.1163772	0.04094744	2.84211176	0.00448158	0.01777231
PHF11	1124.91215	-0.270787	0.06388649	-4.2385639	2.25E-05	0.00016971
PHF14	3541.99235	0.2711993	0.04532258	5.98375682	2.18E-09	3.51E-08
PHF19	2570.10533	0.14109967	0.04905817	2.87617053	0.00402532	0.01623125
PHF20	2160.88656	0.14921749	0.05083901	2.93509812	0.00333442	0.01380873
PHF23	2361.55661	0.22915068	0.0488478	4.69111554	2.72E-06	2.52E-05
PHF2P2	91.9018219	-1.1497845	0.21205213	-5.4221786	5.89E-08	7.41E-07
PHGDH	1700.0354	0.8348547	0.06445952	12.9516127	2.30E-38	2.95E-36
PHLDA1	4968.48956	-0.4837876	0.04162758	-11.621804	3.19E-31	2.99E-29
PHLDB1	1695.05801	0.53803206	0.05435483	9.89851477	4.23E-23	2.41E-21
PHLPP1	895.183162	0.25190158	0.07280934	3.45974276	0.00054069	0.00290654
PHRF1	2442.6996	0.24505931	0.05116669	4.78943027	1.67E-06	1.61E-05
PHTF2	2680.32244	0.2889047	0.0482452	5.98825806	2.12E-09	3.42E-08
PI4KA	5774.65429	0.17395552	0.0490385	3.54732538	0.00038916	0.00218137
PI4KAP1	191.540905	-0.3801295	0.14154431	-2.6855867	0.00724026	0.02665433
PI4KAP2	294.176814	-0.309618	0.12394572	-2.4980127	0.01248917	0.04153339
PI4KB	7826.88277	0.20430189	0.03695816	5.5279234	3.24E-08	4.26E-07
PIAS4	1883.54795	0.33736814	0.0545172	6.1882876	6.08E-10	1.05E-08
PIDD1	725.943154	-0.2705691	0.0795818	-3.399887	0.00067414	0.00350106
PIGA	896.597156	-0.289919	0.06996733	-4.1436343	3.42E-05	0.00024834
PIGBOS1__R	1491.1859	-0.1774796	0.05839339	-3.0393781	0.00237067	0.01031913
PIGH	877.366665	-0.2228406	0.07045399	-3.1629231	0.00156194	0.00724875
PIGM	1592.66433	0.20328363	0.05671191	3.58449609	0.00033773	0.00192682
PIGS	3570.66138	0.14850029	0.04290922	3.46080121	0.00053857	0.0028977
PIGT	6537.23586	0.31948634	0.04455195	7.17109668	7.44E-13	1.84E-11
PIK3CA	1911.94007	0.20706073	0.05524574	3.74799422	0.00017825	0.0010929
PILRA__PILR	3773.85828	-0.4059182	0.04280707	-9.4825029	2.48E-21	1.25E-19
PIM1	1722.36318	-0.5706224	0.05489078	-10.395597	2.60E-25	1.72E-23
PIP5K1A	8873.23295	0.12802899	0.03546229	3.61028574	0.00030586	0.00176825
PIP5KL1	241.55242	-0.3962989	0.13085144	-3.0286167	0.00245676	0.01065571
PJA1	892.837289	0.26041154	0.07163755	3.63512627	0.00027785	0.00162578
PKD1	3669.36704	-0.3591254	0.06028444	-5.9571819	2.57E-09	4.09E-08
PKD2	1075.77873	-0.2169202	0.06477376	-3.3488899	0.00081136	0.0041164
PKIA	426.743358	-0.8908948	0.10033601	-8.8791134	6.74E-19	2.90E-17

PKIB	8668.79172	0.3434823	0.03567296	9.6286465	6.05E-22	3.20E-20
PKN3	570.453587	0.36510021	0.09600482	3.80293622	0.00014299	0.0009
PKP3	5338.21429	0.20922496	0.05437171	3.84804853	0.00011906	0.00076015
PLA2G3	23.8451054	1.25933456	0.40979077	3.07311597	0.00211836	0.00938934
PLA2G4F	358.493596	-0.2908132	0.10834112	-2.6842362	0.00726957	0.02671909
PLAGL2	1813.69017	0.15191838	0.05399625	2.81349893	0.00490056	0.0191835
PLAU	91.5286032	-0.8348501	0.20439852	-4.0844233	4.42E-05	0.0003118
PLCG1	12995.5233	-0.2550279	0.03634244	-7.0173586	2.26E-12	5.26E-11
PLCG2	966.502193	0.22564086	0.06710155	3.36267754	0.00077191	0.00395363
PLCXD3	244.158786	-1.9940162	0.15407303	-12.94202	2.61E-38	3.32E-36
PLD1	869.800822	-0.2082299	0.07292812	-2.8552755	0.00429995	0.01714527
PLEK2	462.704231	0.5995126	0.09848763	6.08718683	1.15E-09	1.91E-08
PLEKHA1	1759.5833	-0.1530255	0.05492633	-2.7860127	0.00533608	0.02067532
PLEKHA8	1385.62852	0.33547972	0.06720187	4.99211911	5.97E-07	6.32E-06
PLEKHB1	960.238418	0.93026483	0.06990611	13.3073466	2.10E-40	2.99E-38
PLEKHB2	8403.79054	0.1315956	0.03562398	3.69401785	0.00022074	0.00132182
PLEKHF2	7673.27512	0.28507782	0.03898728	7.3120728	2.63E-13	6.85E-12
PLEKHH1	2825.32634	-0.3633983	0.0465836	-7.8009925	6.14E-15	1.94E-13
PLEKHH3	2482.4035	0.22213575	0.06937922	3.20176183	0.0013659	0.00645897
PLEKHM1P__	2493.36398	-0.4409192	0.0512315	-8.6064093	7.54E-18	3.01E-16
PLEKHM2	2952.31568	0.13042799	0.05339491	2.4427046	0.01457766	0.0469739
PLEKHN1	300.403198	-0.6962993	0.1158919	-6.0081791	1.88E-09	3.05E-08
PLIN3	3337.53306	0.28261551	0.04579737	6.1709989	6.79E-10	1.17E-08
PLK1	4754.86027	0.42273962	0.04112792	10.2786522	8.79E-25	5.57E-23
PLK2	11769.8713	-0.3232993	0.03312916	-9.758751	1.69E-22	9.16E-21
PLK3	197.633522	-0.5260979	0.14231692	-3.6966646	0.00021845	0.00130898
PLLP	2132.28521	0.27258736	0.05488814	4.96623463	6.83E-07	7.15E-06
PLOD3	7093.19882	0.12796425	0.04529948	2.82485038	0.00473027	0.01862889
PLP2	3349.8027	-0.2705881	0.04961213	-5.4540706	4.92E-08	6.28E-07
PLPP2	2441.42281	0.49426806	0.0563371	8.773403	1.73E-18	7.22E-17
PLPP6	1633.27326	0.17075195	0.05624979	3.03560169	0.00240056	0.01043181
PLPPR2	3302.8767	0.30273123	0.05292963	5.71950448	1.07E-08	1.54E-07
PLS3	43.5613627	-2.6690126	0.35589162	-7.4995096	6.41E-14	1.80E-12
PLXNA1	8332.41668	0.14108789	0.04831121	2.9203964	0.00349586	0.0143599
PLXNA3	7496.45476	-0.3393402	0.04709769	-7.205029	5.80E-13	1.45E-11
PLXNA4	220.958759	-1.3862874	0.13997269	-9.9039848	4.00E-23	2.29E-21
PMS2	1113.07558	0.20283897	0.06595508	3.07541088	0.00210213	0.00932456
PMS2CL	1082.2317	-0.1914544	0.06649398	-2.8792741	0.00398592	0.01609758
PNISR	6641.78223	-0.5404599	0.03812962	-14.174279	1.32E-45	2.36E-43
PNN	12566.8422	-0.3631607	0.03536721	-10.268288	9.79E-25	6.14E-23
PNPLA7	480.765983	-0.6231053	0.10796511	-5.7713578	7.86E-09	1.17E-07
PODXL	6854.02102	-0.7327681	0.03841512	-19.074994	4.07E-81	2.19E-78
PODXL2	2042.49436	0.47888593	0.05221506	9.17141373	4.67E-20	2.16E-18

POFUT2	1701.29952	-0.2276508	0.05891097	-3.8643184	0.0001114	0.00071599
POGK	6126.48194	0.10488931	0.03806108	2.75581534	0.0058546	0.02229482
POGZ	10192.2268	0.10601098	0.03482758	3.04388013	0.00233548	0.01020249
POLDIP3	2902.54029	0.22158212	0.04487207	4.93808519	7.89E-07	8.12E-06
POLI	1550.20889	-0.1996868	0.05713407	-3.4950555	0.00047396	0.00259449
POLR1B	3006.19967	0.2807372	0.05066544	5.54100013	3.01E-08	4.00E-07
POLR1E	1378.899	0.15251571	0.06089089	2.50473765	0.01225423	0.04097626
POLR2A	18053.1391	0.24109164	0.03671178	6.56714631	5.13E-11	1.03E-09
POLR2G	4244.88088	0.19581164	0.04547616	4.30580818	1.66E-05	0.00013009
POLR2J2__P(	2343.66211	-0.1387006	0.0567954	-2.4421107	0.01460167	0.04704008
POLR3C	2745.36031	0.12223211	0.047633	2.56612247	0.01028425	0.0356042
POLR3D	1342.49456	0.19941407	0.06117746	3.25960052	0.00111569	0.00540333
POLR3H	4440.83094	0.23731061	0.04717617	5.03030688	4.90E-07	5.28E-06
POM121	2186.1101	0.17689139	0.05095337	3.47163257	0.0005173	0.00279565
POM121C	3060.77024	0.26986221	0.04793187	5.6301206	1.80E-08	2.49E-07
POMP	5169.23461	-0.2210199	0.05143234	-4.2972932	1.73E-05	0.0001345
POMT2	4095.41254	0.24522046	0.04687942	5.23087725	1.69E-07	1.96E-06
POP1	1602.12704	0.33189733	0.05949649	5.57843549	2.43E-08	3.29E-07
POR	7320.2087	0.21827195	0.05319025	4.10360799	4.07E-05	0.00028972
POTEI	231.959307	-0.5448161	0.15297779	-3.5614065	0.00036887	0.00208624
POTEKP__RP	477.271535	-0.6379894	0.10775885	-5.9205293	3.21E-09	5.06E-08
PP14571	296.557348	-0.7550887	0.11725381	-6.4397793	1.20E-10	2.28E-09
PPARG	361.433605	-0.6028316	0.10981794	-5.4893721	4.03E-08	5.22E-07
PPFIA3	1707.80477	0.15535415	0.06287513	2.47083621	0.01347975	0.04418297
PPFIA4	935.112111	-2.2942784	0.08288942	-27.678783	1.26E-168	2.55E-165
PPIA	51174.4402	0.13929555	0.03327604	4.18606182	2.84E-05	0.00020894
PPIB	14634.2341	0.13932481	0.03669285	3.79705609	0.00014643	0.00091749
PPIP5K1	844.751287	-0.2299022	0.07162149	-3.2099612	0.00132753	0.00630862
PPM1B	3268.30175	0.14967839	0.04558758	3.28331554	0.00102594	0.0050274
PPM1D	17045.0071	0.16861972	0.03214288	5.24594378	1.55E-07	1.82E-06
PPM1E	1010.76885	-0.1713202	0.06821074	-2.5116305	0.01201749	0.04028722
PPM1G	13466.0498	0.09137967	0.03167738	2.88469745	0.0039179	0.0158718
PPM1H	3729.12774	0.27276251	0.04227501	6.45209859	1.10E-10	2.12E-09
PPM1K	1154.00013	-0.289915	0.06824527	-4.248133	2.16E-05	0.0001635
PPOX	457.870185	-0.3020725	0.09528964	-3.1700455	0.00152415	0.00709325
PPP1CA	14865.9325	0.15610505	0.03613732	4.31977411	1.56E-05	0.0001224
PPP1R15A	1744.13678	-0.1978801	0.05833521	-3.3921216	0.00069354	0.00358445
PPP1R3C	741.146889	0.509354	0.08080647	6.30338157	2.91E-10	5.24E-09
PPP1R3F	509.467079	0.3694963	0.09758101	3.78655946	0.00015275	0.00095188
PPP1R3G	50.2629331	-1.255254	0.29458334	-4.2611167	2.03E-05	0.00015506
PPP1R9B	6612.66349	0.26571509	0.03938777	6.74613213	1.52E-11	3.25E-10
PPP2R1A	14977.2579	0.24022823	0.03808249	6.30810158	2.82E-10	5.09E-09
PPP2R2A	3975.09063	0.1689667	0.04768477	3.54341031	0.00039499	0.00220927

PPP2R4	14946.8226	0.16660657	0.03235093	5.14997695	2.61E-07	2.93E-06
PPP2R5D	2725.08757	0.18284935	0.04721514	3.87268454	0.00010764	0.00069454
PPP4R1L	1290.40354	-0.5531556	0.0661543	-8.3615972	6.19E-17	2.28E-15
PPRC1	3429.67141	0.13339024	0.04789118	2.78527796	0.00534819	0.02070463
PRC1	10123.0703	0.19805615	0.03324968	5.95663412	2.57E-09	4.10E-08
PRCC	4596.36487	0.11109356	0.04177932	2.6590559	0.007836	0.02852497
PRCP	1928.24255	0.2627143	0.05366312	4.89562097	9.80E-07	9.94E-06
PRDM15	1372.45702	0.21026448	0.05996116	3.50667832	0.00045374	0.0025018
PRDM2	2722.78201	0.13115638	0.04686639	2.79851689	0.00513379	0.01996364
PRDX2	7920.50631	0.30865655	0.03590018	8.59763251	8.14E-18	3.25E-16
PRDX3	8322.24607	0.1075063	0.04221754	2.54648435	0.01088141	0.03731646
PRDX6	13791.4298	0.12717715	0.03295143	3.85953419	0.0001136	0.00072964
PRELID1	7818.00724	0.12398056	0.03482554	3.56004675	0.00037079	0.00209383
PREX1	17242.2645	-0.3811367	0.03904715	-9.7609343	1.66E-22	8.99E-21
PRG4	102.044651	-1.7697222	0.22450762	-7.8826823	3.20E-15	1.05E-13
PRICKLE2	6472.20651	-0.2499859	0.03734486	-6.6939843	2.17E-11	4.57E-10
PRKAA2	76.207868	-0.7339746	0.22755261	-3.2255161	0.00125746	0.00602114
PRKACA	6106.22702	0.12977621	0.03814857	3.40186278	0.00066928	0.0034818
PRKACB	873.886318	-0.1889282	0.07095256	-2.6627403	0.00775072	0.0282823
PRKAR2B	794.0752	0.71751893	0.07453865	9.6261324	6.20E-22	3.27E-20
PRKCH__RP1	3009.85348	0.19500474	0.04443319	4.38871776	1.14E-05	9.22E-05
PRKCI	5631.20869	0.17395706	0.03719984	4.67628575	2.92E-06	2.68E-05
PRKCSH	17825.1099	0.12489882	0.0315622	3.95722745	7.58E-05	0.00050681
PRKD2	3966.68609	0.1299882	0.04632809	2.80581851	0.0050189	0.01958787
PRKX	1881.34807	0.18996978	0.05546161	3.42524818	0.00061424	0.003237
PRMT2	4827.90828	0.15729401	0.03852774	4.08261706	4.45E-05	0.00031399
PROB1	402.770811	0.31043796	0.10382078	2.99013317	0.00278856	0.0118856
PROCA1	326.564278	-0.4689963	0.10976438	-4.2727547	1.93E-05	0.00014798
PRODH	76.8931359	-1.265083	0.23981049	-5.275345	1.33E-07	1.58E-06
PROSC	1724.03098	0.22380819	0.05542392	4.03811572	5.39E-05	0.00037201
PROSER1	5045.22965	0.19171887	0.03928171	4.88061406	1.06E-06	1.06E-05
PROSER2	1459.90968	0.8639171	0.06865854	12.5828061	2.63E-36	3.15E-34
PRPF3	6553.62429	-0.2624742	0.03621665	-7.2473342	4.25E-13	1.08E-11
PRPF40B	637.420807	-0.3153072	0.08472165	-3.7216836	0.0001979	0.0011991
PRPF8	22461.3934	0.10282592	0.04079244	2.52071057	0.01171181	0.03953225
PRR12	3961.39427	0.22007614	0.04947832	4.44793041	8.67E-06	7.19E-05
PRR15	1037.05721	0.38067312	0.0689589	5.52029025	3.38E-08	4.43E-07
PRR7	1625.53016	0.24597278	0.08051166	3.05512008	0.0022497	0.00989176
PRRC2A	23490.9275	0.14978337	0.03722412	4.02382523	5.73E-05	0.00039325
PRRC2B	20457.2503	0.18733726	0.05418914	3.45709946	0.00054602	0.00293001
PRRG1__RP5	578.702421	0.45613417	0.08681945	5.25382505	1.49E-07	1.75E-06
PRSS1__PRS	29.1807761	-3.1079735	0.46636445	-6.664259	2.66E-11	5.53E-10
PRSS22	1081.72743	-0.6068982	0.06632498	-9.1503716	5.67E-20	2.61E-18

PRSS23	11422.7922	-0.6809301	0.03470501	-19.620514	1.03E-85	6.28E-83
PRSS27	138.002159	-0.9817149	0.17465334	-5.6209342	1.90E-08	2.62E-07
PRSS53__RP	2675.65074	-0.2375303	0.05536773	-4.2900486	1.79E-05	0.00013819
PRTG	393.195997	-0.4528957	0.10032799	-4.514151	6.36E-06	5.46E-05
PRUNE	3160.43265	0.2138998	0.04706953	4.5443365	5.51E-06	4.80E-05
PSAP	29097.2373	0.24181413	0.03060595	7.90088737	2.77E-15	9.20E-14
PSAPL1	16.9298477	-1.6585004	0.50258868	-3.2999159	0.00096714	0.00478556
PSCA	88.0633787	-1.1195314	0.21466827	-5.2151695	1.84E-07	2.12E-06
PSD	233.574042	-0.3810183	0.12924947	-2.9479295	0.0031991	0.01333007
PSKH1	2396.49305	0.36229488	0.05374925	6.74046331	1.58E-11	3.37E-10
PSMA5	9591.24259	-0.2168406	0.03763616	-5.7614952	8.34E-09	1.23E-07
PSMB1	8894.74999	-0.1034035	0.03640544	-2.8403324	0.00450666	0.01785039
PSMB7	7594.93879	-0.0971828	0.03715452	-2.6156398	0.00890605	0.03163086
PSMC1	5931.19657	-0.1610759	0.04430816	-3.6353553	0.0002776	0.00162495
PSMC1P7	56.5949519	-0.8078135	0.2672871	-3.022269	0.00250888	0.01085076
PSMC2	9316.42997	-0.1656136	0.03705501	-4.4693995	7.84E-06	6.58E-05
PSMC4	8160.2565	-0.1136475	0.03561169	-3.1912978	0.00141635	0.00665613
PSMC5	20570.6414	-0.0783951	0.03180645	-2.4647536	0.01371075	0.04479516
PSMC6	7337.38798	-0.3094842	0.0416695	-7.4271155	1.11E-13	3.03E-12
PSMD1	6797.48037	-0.1446822	0.03840014	-3.7677511	0.00016473	0.00101713
PSMD11	4506.12772	-0.1881432	0.03998541	-4.7052949	2.53E-06	2.35E-05
PSMD12	14635.7181	-0.2244394	0.03627697	-6.1868284	6.14E-10	1.06E-08
PSMD13	3266.56191	-0.1100166	0.04433938	-2.4812398	0.01309263	0.04310779
PSMD5-AS1	2281.37042	-0.2802213	0.05164911	-5.4254819	5.78E-08	7.30E-07
PSMD6	60222.958	-0.2355595	0.03246581	-7.2556186	4.00E-13	1.03E-11
PSMD6-AS2_	252.63715	-0.9467521	0.14163216	-6.6845842	2.32E-11	4.86E-10
PSME2	6235.15365	-0.2970586	0.03851292	-7.7132184	1.23E-14	3.75E-13
PSME3	6842.21017	0.13548154	0.03631575	3.73065558	0.00019098	0.00116079
PSPH	886.18599	0.28683459	0.07007918	4.09300744	4.26E-05	0.00030164
PTBP1	18676.4262	0.21420201	0.0412638	5.19103959	2.09E-07	2.38E-06
PTDSS1	7034.42236	0.18591371	0.03993865	4.65498211	3.24E-06	2.95E-05
PTGER2	45.0826027	0.85186447	0.29958307	2.84350002	0.0044621	0.01770663
PTGER4	1809.03047	1.06921482	0.05567377	19.2050023	3.36E-82	1.86E-79
PTGR1	2577.55071	-0.1974489	0.05036329	-3.9204922	8.84E-05	0.00058233
PTK2B	391.67961	0.30881679	0.10446944	2.95604897	0.00311608	0.01304374
PTK6	605.539778	-0.5100712	0.08616247	-5.9198768	3.22E-09	5.07E-08
PTK7	3226.54011	0.23334507	0.05135426	4.54383115	5.52E-06	4.81E-05
PTMS	6557.86294	0.16468045	0.03851074	4.27622144	1.90E-05	0.00014594
PTOV1-AS2	341.065037	-0.4003364	0.11733002	-3.4120542	0.00064475	0.00337634
PTP4A1	20104.668	0.07862223	0.03052667	2.57552587	0.01000878	0.03483491
PTPN1	10394.1436	0.18628411	0.03246397	5.73817979	9.57E-09	1.40E-07
PTPN23	3736.93464	0.14715608	0.05112786	2.87819736	0.00399955	0.01614518
PTPN9__SNU	2963.19392	0.1880779	0.04578265	4.10806027	3.99E-05	0.00028497

PTPRF	27629.1207	0.15686687	0.0383631	4.08900404	4.33E-05	0.00030618
PTPRH	46.4113571	-0.7889389	0.31936554	-2.470332	0.01349877	0.04423735
PTPRU	2906.56486	-0.2395614	0.04978429	-4.8119876	1.49E-06	1.45E-05
PTPRVP	36.6821908	-2.1647473	0.3605553	-6.0039257	1.93E-09	3.12E-08
PTTG1	1913.26229	0.21456805	0.05681183	3.7768201	0.00015884	0.00098515
PUM1	5661.22082	0.20684788	0.04179737	4.94882553	7.47E-07	7.74E-06
PUM2	10046.7248	0.12704777	0.03338832	3.80515598	0.00014172	0.00089258
PURA	2552.08468	0.13201865	0.04966974	2.65792898	0.00786225	0.02858978
PVRL1	3726.20693	0.16123655	0.04650613	3.46699603	0.00052631	0.00283843
PXDN	8505.1824	0.30973527	0.04062813	7.62366574	2.47E-14	7.32E-13
PYGM	51.184402	-1.1741909	0.28353423	-4.1412668	3.45E-05	0.00025051
PYGO2__SH(	8218.16669	0.12478534	0.03448751	3.61827573	0.00029657	0.0017233
PYROXD2	1357.65031	-0.1952166	0.06468531	-3.0179439	0.00254496	0.01099119
QRICH1	4055.79279	0.10549092	0.04173008	2.52793452	0.01147358	0.03886479
QSOX1	7307.45992	-0.3491704	0.03680713	-9.4864879	2.39E-21	1.21E-19
QSOX2	2170.92821	0.16346171	0.04962226	3.29412052	0.0009873	0.00486596
R3HDM1	3910.91753	0.13975018	0.04349398	3.21309256	0.00131314	0.00625001
RAB11FIP1P:	658.096787	-0.7801984	0.08223634	-9.487271	2.37E-21	1.20E-19
RAB11FIP2	665.082602	-0.2046304	0.08015539	-2.5529212	0.01068237	0.03673058
RAB11FIP5_	2016.78959	-0.1337813	0.05287689	-2.530052	0.01140456	0.03870292
RAB12	1646.65557	-0.3554487	0.05831449	-6.095375	1.09E-09	1.81E-08
RAB13	5244.60014	0.26945119	0.04564857	5.90273069	3.58E-09	5.59E-08
RAB1B	12836.5168	0.18610192	0.03214653	5.78917651	7.07E-09	1.06E-07
RAB26	576.299719	-0.4064327	0.09075162	-4.4785179	7.52E-06	6.34E-05
RAB27B	1432.24692	-0.2984468	0.05830181	-5.1189974	3.07E-07	3.44E-06
RAB29	2952.55704	0.43855538	0.04481623	9.7856366	1.30E-22	7.11E-21
RAB35	3837.76594	0.19456782	0.04289214	4.53621162	5.73E-06	4.97E-05
RAB39B	570.194017	0.46177002	0.08660295	5.3320355	9.71E-08	1.18E-06
RAB5B	8118.38266	0.0938525	0.03503471	2.67884352	0.00738769	0.02708226
RAB6C-AS1	108.887622	-0.6555199	0.19729972	-3.3224573	0.00089228	0.00446485
RABEP1	6247.02814	0.22536251	0.03980582	5.66154719	1.50E-08	2.11E-07
RABIF	2206.42978	0.18481128	0.05246349	3.52266475	0.00042723	0.0023671
RABL2A	317.886528	-0.5470539	0.11659791	-4.6917988	2.71E-06	2.51E-05
RABL2B	658.755109	-0.2769753	0.08630467	-3.2092733	0.00133071	0.00632044
RABL3	1548.30876	-0.1754481	0.05832026	-3.0083564	0.00262665	0.01127987
RACGAP1	7200.73297	0.2798499	0.03683444	7.59750713	3.02E-14	8.91E-13
RAD21	22153.1901	0.11876626	0.03038952	3.90813255	9.30E-05	0.00061051
RAD51-AS1	171.377322	-0.7039803	0.15133335	-4.6518515	3.29E-06	2.99E-05
RAD52	583.886955	0.25738952	0.08480976	3.03490428	0.00240612	0.01045347
RAD54L	1159.47169	0.24881008	0.06764716	3.67805624	0.00023502	0.00139906
RAD9A	1497.95873	-0.3099172	0.05998412	-5.1666538	2.38E-07	2.70E-06
RAI14	533.056862	0.48763511	0.09264082	5.26371734	1.41E-07	1.67E-06
RALGAPA2	4102.28203	0.2121081	0.05537338	3.83050635	0.00012788	0.00081189

RALGPS1	1506.92353	-0.2664904	0.0596374	-4.4685105	7.88E-06	6.61E-05
RALY	6321.54839	0.13843465	0.04035323	3.43057167	0.00060231	0.00318334
RAN	27532.4428	0.17091021	0.033648	5.07935739	3.79E-07	4.19E-06
RANBP9	4808.82141	0.16073377	0.04125189	3.89639781	9.76E-05	0.00063649
RANGAP1	6918.72654	0.21900956	0.04364913	5.01750145	5.23E-07	5.60E-06
RAP1GAP	916.890936	-0.4050978	0.0719674	-5.6289076	1.81E-08	2.51E-07
RARG	4194.78271	0.15727168	0.04602738	3.41691592	0.00063335	0.00332378
RASEF	1761.63405	0.32204368	0.05580232	5.77115174	7.87E-09	1.17E-07
RASGRP1	1247.35868	-0.5658746	0.06151001	-9.1997149	3.59E-20	1.67E-18
RASL10B	68.0072782	0.59232803	0.24241199	2.44347665	0.01454651	0.04689633
RASSF2	159.250599	1.36701677	0.1602838	8.5287272	1.48E-17	5.77E-16
RASSF8	2342.02165	0.17688044	0.05084178	3.47903739	0.00050322	0.00272923
RB1	4296.4779	0.11920219	0.04138724	2.88016779	0.00397464	0.01606595
RBBP5	3136.76514	0.21118079	0.04466374	4.72823818	2.26E-06	2.13E-05
RBBP6	6699.55167	-0.2375502	0.0356124	-6.670435	2.55E-11	5.31E-10
RBBP8	2398.24973	0.14876812	0.04820594	3.08609512	0.00202804	0.0090436
RBCK1	3655.80412	-0.193953	0.04788373	-4.0505001	5.11E-05	0.00035542
RBL1	1243.09841	-0.1885707	0.0657525	-2.8678861	0.00413224	0.01658171
RBL2	5510.43379	0.27039965	0.04324255	6.25309263	4.02E-10	7.11E-09
RBM10	5101.26357	0.14262516	0.03881712	3.67428454	0.00023852	0.00141942
RBM14__RB	15019.3567	0.19609043	0.03355484	5.84387927	5.10E-09	7.80E-08
RBM15B	3420.85875	0.15769925	0.047279	3.33550322	0.00085145	0.00428404
RBM3	5978.2229	-0.3881607	0.04047797	-9.5894324	8.86E-22	4.61E-20
RBM38	5087.92982	0.28145928	0.06214274	4.52923836	5.92E-06	5.11E-05
RBM39	13126.5314	-0.0801452	0.03260548	-2.4580292	0.01397018	0.04543923
RBMS1	1754.66544	-0.4440606	0.05846441	-7.5953995	3.07E-14	9.04E-13
RBPJ	3534.86257	-0.1103006	0.04399294	-2.507234	0.01216801	0.0407029
RBPMS	2141.41648	-0.1399525	0.05554479	-2.5196339	0.0117477	0.03963135
RCAN3	767.964609	0.3067872	0.08379456	3.66118295	0.00025105	0.0014853
RCC2	15098.0481	0.17951715	0.03132731	5.7303722	1.00E-08	1.46E-07
RCCD1	2340.63808	-0.2573587	0.0550614	-4.6740306	2.95E-06	2.71E-05
RCOR2	198.0993	0.36482794	0.14360649	2.54046983	0.01107036	0.0378151
RDX	15100.9577	-0.6582099	0.03134137	-21.001312	6.38E-98	4.85E-95
REC8	256.228461	-1.9232002	0.14725083	-13.060709	5.52E-39	7.46E-37
REPIN1__RP	8461.12036	0.17092246	0.04490141	3.80661704	0.00014088	0.00088794
RERE	4198.71234	0.20042026	0.04973131	4.03006202	5.58E-05	0.00038429
RET	7368.3756	-0.1380671	0.04056781	-3.4033662	0.00066561	0.00346673
REV3L	4939.37571	0.13517171	0.04580738	2.95087192	0.00316878	0.01322793
RFNG	1451.51424	-0.204672	0.07077249	-2.8919711	0.00382833	0.01554003
RFWD3	7469.3239	0.09811888	0.03476809	2.82209555	0.0047711	0.01875624
RFX1	1481.50839	0.26936844	0.06287436	4.28423327	1.83E-05	0.00014132
RFX5	4510.10759	0.22495578	0.04069326	5.52808507	3.24E-08	4.26E-07
RFXANK	1875.81992	0.19039967	0.05359641	3.55247075	0.00038163	0.0021484

RGAG4	353.445872	0.33991153	0.10620226	3.20060553	0.00137139	0.00648158
RGL2	6761.33019	-0.1849614	0.03820791	-4.8409203	1.29E-06	1.27E-05
RGPD5	43.620078	-0.8106528	0.31419788	-2.5800709	0.009878	0.03448507
RGS11	117.24063	-0.9311608	0.18742345	-4.9682192	6.76E-07	7.08E-06
RGS16	358.82184	-0.9643172	0.10769139	-8.9544509	3.41E-19	1.50E-17
RGS19	1883.26429	0.36616758	0.06020948	6.08156067	1.19E-09	1.97E-08
RGS9BP	85.584603	0.91030389	0.21391249	4.25549673	2.09E-05	0.0001588
RHBDF2	1907.89612	0.14493771	0.05905287	2.45437206	0.01411309	0.04577012
RHBDL1	121.720956	-1.0789627	0.20506167	-5.2616501	1.43E-07	1.69E-06
RHBG	241.191547	-0.5168008	0.12958551	-3.9881063	6.66E-05	0.0004513
RHNO1__TU	3309.54526	0.26569608	0.0453054	5.86455677	4.50E-09	6.94E-08
RHOD	3534.3325	-0.2192648	0.04524352	-4.8463249	1.26E-06	1.25E-05
RHOT2	4893.50674	-0.2839437	0.0474761	-5.9807712	2.22E-09	3.57E-08
RHOU	2028.99188	0.44699116	0.05271496	8.47939826	2.26E-17	8.65E-16
RHOV	716.09813	-0.2463604	0.07746784	-3.1801629	0.00147192	0.00687476
RHPN1	3679.97354	-0.3181495	0.052653	-6.0423802	1.52E-09	2.49E-08
RILPL2	542.296438	0.54518041	0.09121406	5.97693384	2.27E-09	3.65E-08
RIMKLA	320.742779	-0.3970734	0.11673118	-3.4016052	0.00066991	0.00348309
RIN3	647.417083	0.67833955	0.08421596	8.054762	7.96E-16	2.77E-14
RIOK3	2237.92797	-0.305008	0.05165986	-5.9041592	3.54E-09	5.55E-08
RITA1	2272.33508	0.18551537	0.0580782	3.19423445	0.00140202	0.00660581
RLN2	362.388194	0.58604693	0.11067793	5.29506605	1.19E-07	1.43E-06
RMND5A	4135.46907	0.15489737	0.04261207	3.63505895	0.00027792	0.00162578
RMND5B	10226.1869	-0.1171965	0.03606981	-3.2491582	0.00115747	0.00558342
RN7SL674P	60.5055794	-0.875177	0.2606856	-3.3572126	0.00078733	0.00401457
RNASEH2A	3640.10754	0.13889041	0.04278355	3.24635114	0.00116895	0.00563132
RNASET2__F	3586.51567	-0.2160095	0.04269788	-5.0590215	4.21E-07	4.59E-06
RNF10	9182.48589	0.16750836	0.03765918	4.44800914	8.67E-06	7.19E-05
RNF111	1553.54246	0.20927214	0.05804132	3.60557176	0.00031147	0.00179667
RNF125__RN	1631.44826	0.14530805	0.05691829	2.55292365	0.01068229	0.03673058
RNF130	5638.76174	0.22881011	0.03944037	5.80141935	6.58E-09	9.88E-08
RNF139-AS1	310.855098	-0.7160017	0.12415845	-5.7668384	8.08E-09	1.19E-07
RNF150	2157.63089	0.36999326	0.05511477	6.71314224	1.90E-11	4.03E-10
RNF183	147.730874	-1.4761342	0.17363879	-8.5011779	1.88E-17	7.23E-16
RNF187	6127.80016	0.19201532	0.04051224	4.73968616	2.14E-06	2.02E-05
RNF207	410.469983	-0.8776406	0.10094333	-8.6943888	3.49E-18	1.42E-16
RNF223	1423.311	-0.3974755	0.06286035	-6.3231522	2.56E-10	4.67E-09
RNF224	63.8417216	-0.6260795	0.24328503	-2.5734404	0.0100693	0.03500545
RNF24	3188.87039	0.48558234	0.0437019	11.1112406	1.11E-28	8.73E-27
RNF26	3222.85992	0.278055	0.05434404	5.1165684	3.11E-07	3.47E-06
RNF34	2179.98194	0.2929706	0.05072213	5.7759917	7.65E-09	1.14E-07
RNF40	5875.74185	0.15810378	0.04397471	3.59533436	0.00032398	0.00185708
RNF41	2765.00532	0.35535396	0.04587395	7.74631219	9.46E-15	2.91E-13

RNF6	4053.77209	0.11449527	0.04302592	2.66107649	0.00778913	0.02839971
RNPEP	5106.11167	0.22417763	0.04455054	5.03198415	4.85E-07	5.24E-06
RORA	190.601764	-0.6375959	0.15349166	-4.1539451	3.27E-05	0.00023844
RP1-266L20.!	45.1792534	-0.9682901	0.30847147	-3.1389941	0.00169529	0.00777462
RP1-60O19.1	13.825047	-2.4045535	0.60770064	-3.956806	7.60E-05	0.00050752
RP11-1000B(	42.9704518	-0.8614859	0.31313478	-2.7511665	0.00593835	0.02253482
RP11-100G1!	9.00459871	-2.3251953	0.73890683	-3.1468045	0.00165065	0.00759859
RP11-1020A:	170.413019	-0.5422908	0.16070898	-3.3743652	0.00073986	0.00380339
RP11-104N1(	583.479742	-0.4994557	0.09866267	-5.062256	4.14E-07	4.52E-06
RP11-1055B8	129.543595	-0.9288362	0.18820556	-4.935222	8.01E-07	8.23E-06
RP11-106D4.	16.1002123	-1.8478571	0.55604806	-3.3231967	0.00088992	0.00445425
RP11-1072A:	82.6106849	-0.7501977	0.21894754	-3.4263809	0.00061168	0.00322633
RP11-1100L3	618.596786	-1.443471	0.08956375	-16.116688	1.95E-58	5.82E-56
RP11-127I20	45.5299935	-1.4260951	0.31375834	-4.5452022	5.49E-06	4.79E-05
RP11-1281K2	142.947439	0.56907575	0.18353685	3.10060761	0.00193124	0.00868244
RP11-1379J2	30.0415947	-1.176211	0.36898755	-3.1876714	0.00143423	0.00673322
RP11-138H1(	23.2246381	-1.0840002	0.41597376	-2.6059342	0.00916241	0.03234846
RP11-1399P1	245.516079	-0.6590033	0.13167566	-5.0047465	5.59E-07	5.95E-06
RP11-142E9.	154.053424	-0.5421622	0.16359952	-3.3139598	0.00091985	0.00458015
RP11-147L13	6502.90187	0.11912797	0.03603868	3.30555851	0.00094787	0.00470172
RP11-147L13	710.649103	-0.283088	0.10345789	-2.7362632	0.00621413	0.02341083
RP11-147L13	12.0698089	-1.7929231	0.64566174	-2.7768769	0.0054884	0.02113525
RP11-147L13	17.1098677	-1.4324887	0.50856596	-2.8167215	0.00485166	0.01902067
RP11-156E6.	499.874103	-0.357263	0.10032783	-3.5609567	0.00036951	0.002089
RP11-156L14	135.5641	0.9193771	0.18985376	4.84255408	1.28E-06	1.26E-05
RP11-156P1.	407.862469	0.3138377	0.10546776	2.97567422	0.00292345	0.01235251
RP11-158K1.	549.68482	-0.3638904	0.09634724	-3.7768637	0.00015882	0.00098515
RP11-180C16	691.900421	-0.393641	0.08075032	-4.8747917	1.09E-06	1.09E-05
RP11-192H2:	5382.12033	0.15566031	0.0394711	3.94365326	8.02E-05	0.00053326
RP11-192H2:	124.487444	-0.8221134	0.19794563	-4.1532286	3.28E-05	0.0002391
RP11-192H2:	1529.70803	0.2454691	0.07755256	3.16519657	0.00154978	0.00720285
RP11-196G1	187.064642	-0.6319693	0.17418004	-3.6282535	0.00028535	0.00166549
RP11-196H1	100.416333	-0.8272575	0.22538404	-3.6704352	0.00024214	0.00143909
RP11-199F11	90.4660171	-0.959119	0.21035704	-4.5594812	5.13E-06	4.49E-05
RP11-203J24	581.340225	-0.5620568	0.08650521	-6.4973752	8.17E-11	1.60E-09
RP11-203M5	65.8908763	-0.831756	0.24840007	-3.3484533	0.00081264	0.0041206
RP11-205M5	1569.99806	-0.3511725	0.06206824	-5.6578464	1.53E-08	2.14E-07
RP11-21L23.:	228.940388	-0.5877304	0.13681435	-4.2958239	1.74E-05	0.00013516
RP11-244F12	1273.12882	-0.7856219	0.06934754	-11.328764	9.45E-30	7.91E-28
RP11-244F12	155.338793	-0.8169743	0.1662179	-4.91508	8.87E-07	9.05E-06
RP11-248C1.	70.5317907	1.08818739	0.24627335	4.41861611	9.93E-06	8.12E-05
RP11-252E2.	560.736469	-0.4060394	0.09016044	-4.5035208	6.68E-06	5.70E-05
RP11-259O2.	2107.24166	0.1607458	0.05622399	2.85902543	0.00424945	0.01698103

RP11-260A9.	18.413166	-4.7307403	0.82642046	-5.7243746	1.04E-08	1.50E-07
RP11-265D1	65.9064741	-0.7565684	0.26813245	-2.8216219	0.00477815	0.0187688
RP11-274B2	273.222507	-0.4068034	0.13440498	-3.0266987	0.0024724	0.01071335
RP11-274B2	371.350412	-0.6166387	0.11324374	-5.445234	5.17E-08	6.58E-07
RP11-274B2	109.823517	-0.7951519	0.19730777	-4.0300079	5.58E-05	0.00038429
RP11-290L1.	38.9749518	-0.8444889	0.31327534	-2.6956764	0.00702459	0.02600425
RP11-293I14	1954.34647	0.24439498	0.05223328	4.678913	2.88E-06	2.65E-05
RP11-308D1	148.076365	-0.5965214	0.17073129	-3.4939196	0.00047598	0.00260399
RP11-319G9.	17.6115963	-1.8041786	0.503275	-3.5848762	0.00033724	0.00192468
RP11-321G1	38.897774	-0.9250972	0.31673527	-2.9207268	0.00349216	0.01434791
RP11-330H6.	1326.56846	0.20274542	0.06301499	3.21741573	0.00129351	0.00616785
RP11-332H1	283.559799	-0.5580657	0.12792414	-4.3624741	1.29E-05	0.00010235
RP11-334C1	349.221711	-0.5846938	0.1147232	-5.096561	3.46E-07	3.84E-06
RP11-342D1	358.609793	-0.6730083	0.11060168	-6.0849739	1.17E-09	1.93E-08
RP11-342K2.	133.312859	-0.7422037	0.18053526	-4.1111286	3.94E-05	0.00028187
RP11-347C1	227.963324	0.34259661	0.1328763	2.57831239	0.00992842	0.03464118
RP11-347E1	193.682503	-0.9285966	0.14871396	-6.2441791	4.26E-10	7.50E-09
RP11-347I19	350.691894	-0.6224503	0.11659282	-5.3386679	9.36E-08	1.14E-06
RP11-34P13.	439.47708	-0.9917022	0.11192879	-8.860117	7.99E-19	3.39E-17
RP11-354E1	23.3948831	-1.5562608	0.43161413	-3.6056762	0.00031134	0.00179652
RP11-358L2	116.934716	-0.5804581	0.21425506	-2.7091919	0.00674473	0.02512943
RP11-366L5.	208.186632	-0.8678831	0.14115143	-6.148596	7.82E-10	1.33E-08
RP11-367J11	1904.15702	0.23670982	0.0534328	4.43004744	9.42E-06	7.75E-05
RP11-379B1	227.376717	-0.8501154	0.13539242	-6.2788993	3.41E-10	6.08E-09
RP11-379F1	332.040821	0.49809888	0.13852442	3.59574783	0.00032346	0.0018553
RP11-379H1	1094.11179	0.22340343	0.06963941	3.20800278	0.0013366	0.00634347
RP11-379K1	904.729203	-0.2029529	0.07978354	-2.5437945	0.01096556	0.03756264
RP11-37B2.1	539.429277	-0.4563768	0.09004133	-5.0685258	4.01E-07	4.40E-06
RP11-386G1	32843.2586	0.23026445	0.03038699	7.57773056	3.52E-14	1.02E-12
RP11-386M2	51.278684	-1.6978519	0.29693013	-5.7180181	1.08E-08	1.55E-07
RP11-389G6.	11.2998158	-2.466795	0.71927733	-3.4295464	0.00060459	0.00319262
RP11-395N3.	785.668898	-0.3464422	0.0778414	-4.4506162	8.56E-06	7.12E-05
RP11-400F1	897.467902	0.48619753	0.07400154	6.57010037	5.03E-11	1.01E-09
RP11-416N2.	10.9077674	-2.2870009	0.67888321	-3.3687693	0.00075505	0.0038749
RP11-427L1	34.0627257	-0.8848941	0.33674888	-2.6277567	0.008595	0.03069955
RP11-434D9.	546.851143	-0.3256423	0.09094721	-3.580564	0.00034285	0.00195301
RP11-438D1	236.181747	0.62868375	0.13977205	4.49792192	6.86E-06	5.83E-05
RP11-438J1.	6333.50966	0.16499811	0.03766385	4.38080891	1.18E-05	9.52E-05
RP11-439E1	48.2991679	-0.9670554	0.28641766	-3.3763817	0.00073446	0.00377882
RP11-440D1	437.633243	0.47017605	0.09619097	4.88794372	1.02E-06	1.03E-05
RP11-449P1	600.671972	-0.3443016	0.08934507	-3.8536167	0.00011639	0.00074489
RP11-44F14.	47.5098616	-0.8032226	0.28543007	-2.8140784	0.00489173	0.01915717
RP11-452H2	26.72305	-1.4440655	0.40771525	-3.5418481	0.00039733	0.00222103

RP11-455O6.	349.55659	0.50034329	0.12576473	3.97840711	6.94E-05	0.00046802
RP11-457M1	98.6700998	-0.7537239	0.20823397	-3.6196011	0.00029506	0.00171559
RP11-458F8.	748.073409	-0.430865	0.08047815	-5.3538137	8.61E-08	1.05E-06
RP11-459E5.	465.964695	-0.4766416	0.09837107	-4.8453433	1.26E-06	1.25E-05
RP11-463D1	4470.69113	0.10868068	0.04131585	2.63048422	0.00852633	0.03052605
RP11-465B2	232.393166	-0.5420843	0.13318668	-4.0701086	4.70E-05	0.00032917
RP11-465B2	140.18571	-0.5045084	0.17866779	-2.8237234	0.00474693	0.01868241
RP11-468E2.	261.230107	-0.712053	0.12577169	-5.6614725	1.50E-08	2.11E-07
RP11-471B2	30.5202472	-1.5151521	0.36959615	-4.0994801	4.14E-05	0.00029425
RP11-479O9.	10.8136936	-1.5763787	0.64620049	-2.4394577	0.01470933	0.04734508
RP11-47A8.5	282.440995	0.40978466	0.12973867	3.15853908	0.00158562	0.007344
RP11-480A1	942.011409	-0.6196417	0.07802999	-7.9410711	2.00E-15	6.77E-14
RP11-481C4.	226.302409	-0.6667014	0.13517117	-4.9322748	8.13E-07	8.35E-06
RP11-483I13	675.586534	-0.3645015	0.09203814	-3.9603316	7.48E-05	0.00050216
RP11-486L1	15.9864388	-1.6192608	0.54089113	-2.9936908	0.00275625	0.01175339
RP11-486O1	240.953846	-0.6431001	0.13574996	-4.7373872	2.16E-06	2.04E-05
RP11-488L1	450.163079	-0.4459512	0.100623	-4.4319007	9.34E-06	7.69E-05
RP11-48B3.4	62.4461943	-1.5073115	0.2694329	-5.5943857	2.21E-08	3.02E-07
RP11-496H1.	231.678678	-0.942806	0.14593154	-6.4606044	1.04E-10	2.00E-09
RP11-496I9.1	254.526808	-1.4238022	0.13054006	-10.907014	1.07E-27	8.04E-26
RP11-498C9.	858.76999	0.56946003	0.1046561	5.44125003	5.29E-08	6.72E-07
RP11-498C9.	91.109167	-0.5692256	0.22574657	-2.521525	0.01168474	0.03945547
RP11-4K16.2	950.106247	-0.3556821	0.07584801	-4.689405	2.74E-06	2.53E-05
RP11-500C1	258.608857	0.34090593	0.12820421	2.65908542	0.00783531	0.02852497
RP11-504P2	127.481995	-1.1124322	0.19511615	-5.7013849	1.19E-08	1.70E-07
RP11-522B1	301.676578	-1.5153448	0.12821606	-11.818682	3.13E-32	3.03E-30
RP11-522B1	11.161083	-3.3243636	0.77867275	-4.2692693	1.96E-05	0.00015006
RP11-524H1	553.526454	0.55833192	0.08825459	6.3263785	2.51E-10	4.58E-09
RP11-527N2	43.6018934	1.31156465	0.31823928	4.12131602	3.77E-05	0.00027075
RP11-539I5.1	338.126115	0.37483722	0.11337409	3.30619829	0.00094571	0.00469227
RP11-563J2.2	43.1890772	-1.291576	0.31207129	-4.1387211	3.49E-05	0.00025291
RP11-563J2.3	48.3340687	-2.2343249	0.31902824	-7.0035333	2.50E-12	5.78E-11
RP11-564A8.	35.8472728	0.93981544	0.33488784	2.8063588	0.00501049	0.01956343
RP11-56G10.	920.563809	0.31411859	0.08047103	3.90349905	9.48E-05	0.00062142
RP11-574K1	348.999305	-0.8564138	0.11405396	-7.5088475	5.97E-14	1.68E-12
RP11-609D2	108.208348	-1.2421498	0.2022343	-6.142132	8.14E-10	1.38E-08
RP11-609N1	1018.57825	-1.4290438	0.07267701	-19.662942	4.48E-86	2.92E-83
RP11-617F2	524.533483	-0.325417	0.09387242	-3.466588	0.00052711	0.00284138
RP11-624G1	34.4203443	0.88958754	0.33770026	2.63425184	0.00843229	0.03024139
RP11-631N1	151.241309	-0.433981	0.1663855	-2.6082861	0.00909969	0.03218998
RP11-637O1	4160.57713	-0.1102784	0.04162225	-2.6495062	0.00806095	0.02916915
RP11-638I2.6	1377.3741	-0.2617353	0.06357995	-4.1166324	3.84E-05	0.00027588
RP11-657O9.	77.3422575	0.6372548	0.2261652	2.81765195	0.00483762	0.0189738

RP11-662G2:	2499.291	-1.1632024	0.04918306	-23.650466	1.17E-123	1.52E-120
RP11-666A8.	236.561103	-0.5113322	0.13919015	-3.673623	0.00023914	0.00142218
RP11-66N24.	939.560561	-0.6697921	0.07232229	-9.2612117	2.02E-20	9.60E-19
RP11-68L18.	658.081164	-0.4241046	0.08380985	-5.0603194	4.19E-07	4.56E-06
RP11-705C1!	170.384823	-0.459064	0.15596344	-2.9434077	0.00324621	0.01350167
RP11-707P17	113.620995	-0.7016013	0.18909333	-3.7103442	0.00020698	0.00124761
RP11-72I8.1	568.775633	-1.2264673	0.09897	-12.392313	2.88E-35	3.26E-33
RP11-734I18	354.494174	0.47939853	0.11717606	4.09126689	4.29E-05	0.00030356
RP11-747H7.	137.606762	-0.9130933	0.18611307	-4.9061211	9.29E-07	9.45E-06
RP11-75C10.	44.7014061	-1.0059218	0.31759311	-3.1673289	0.00153846	0.00715438
RP11-77P16.	67.1796534	-0.6417251	0.23838043	-2.6920208	0.00710205	0.02623026
RP11-798M1	49.329535	-1.0607752	0.29540067	-3.5909708	0.00032945	0.00188608
RP11-7K24.3	62.8217565	-0.7888498	0.25670786	-3.0729476	0.00211956	0.00938934
RP11-800A3.	621.561518	-0.802861	0.08705518	-9.2224377	2.90E-20	1.37E-18
RP11-801F7.	263.688455	-0.3979751	0.12166621	-3.2710402	0.00107153	0.00521436
RP11-817O1:	336.745238	0.34218959	0.11010609	3.10781719	0.00188475	0.00851287
RP11-81A1.6	436.674968	-0.4506718	0.10080786	-4.4706017	7.80E-06	6.55E-05
RP11-829H1(	303.683994	0.77078127	0.13201273	5.83868887	5.26E-09	8.04E-08
RP11-841O2(	173.750933	-0.6301259	0.17314419	-3.6393128	0.00027337	0.00160327
RP11-848G1	38.5930521	-0.9174081	0.31507323	-2.9117296	0.00359434	0.01470476
RP11-849H4.	7010.48634	0.30015392	0.05461864	5.49544837	3.90E-08	5.06E-07
RP11-855A2.	83.3090293	-0.8998674	0.2192782	-4.1037704	4.06E-05	0.00028963
RP11-865I6.2	637.58772	0.57271272	0.08731974	6.55880032	5.42E-11	1.09E-09
RP11-87H9.4	192.819857	-0.6744502	0.1445115	-4.6671039	3.05E-06	2.79E-05
RP11-88H9.2	378.182597	-0.4084742	0.10684636	-3.823005	0.00013184	0.00083497
RP11-95D17.	125.086587	-0.5322539	0.20059937	-2.653318	0.00797048	0.02888645
RP11-981P6.	49.1619646	-0.9235835	0.31049225	-2.9745783	0.00293392	0.01238357
RP13-1032I1	1487.85578	-0.1740349	0.06393381	-2.7221101	0.00648665	0.02427702
RP13-104F2	395.071445	-0.5179883	0.10595845	-4.8885986	1.02E-06	1.03E-05
RP13-39P12.	198.579246	-1.0914244	0.15888719	-6.8691781	6.46E-12	1.43E-10
RP13-39P12.	150.242788	-1.2848001	0.16601868	-7.7388891	1.00E-14	3.08E-13
RP13-516M1	41.0160948	-0.8718481	0.3084688	-2.8263736	0.00470783	0.01855655
RP3-368A4.5	967.028871	-1.0643437	0.07124047	-14.940156	1.81E-50	4.01E-48
RP3-368A4.6	364.483308	-0.9551557	0.10821989	-8.8260643	1.08E-18	4.57E-17
RP3-406P24.	39.9374273	-1.2839876	0.32729988	-3.9229699	8.75E-05	0.00057695
RP3-416H24.	194.920682	-0.5374726	0.14727706	-3.6493977	0.00026286	0.0015471
RP4-635E18.	67.5718749	-0.7289421	0.23910931	-3.0485729	0.00229931	0.01007344
RP4-669L17.	101.813092	-0.7567701	0.20537101	-3.6848927	0.0002288	0.00136471
RP4-717I23.3	361.794932	-1.3071052	0.11348011	-11.518364	1.07E-30	9.58E-29
RP4-724E16.	342.524676	-0.4600249	0.12209085	-3.7678905	0.00016463	0.00101691
RP4-734G22.	875.418278	0.20362731	0.08034584	2.53438534	0.01126448	0.03830595
RP4-740C4.7	26.9370461	-1.5821579	0.41763527	-3.7883724	0.00015164	0.00094626
RP4-798A10.	67.0769833	-0.7718128	0.24224474	-3.1860868	0.00144211	0.00676373

RP4-798A10.	254.897905	-0.3732964	0.12625236	-2.9567476	0.00310903	0.01302619
RP4-816N1.7	1878.45668	0.41210866	0.05578825	7.38701584	1.50E-13	4.05E-12
RP5-1024G6.	491.714512	0.38300522	0.09084706	4.21593434	2.49E-05	0.0001852
RP5-1074L1.4	245.494053	-1.1008633	0.14894989	-7.3908296	1.46E-13	3.94E-12
RP5-1085F17	42.986422	-1.0712019	0.32925687	-3.2533928	0.00114036	0.00551253
RP5-1120P11	19.2827525	-1.5821203	0.49358724	-3.2053509	0.00134898	0.00639057
RP5-1125A11	139.404895	-0.695266	0.16537156	-4.204266	2.62E-05	0.00019454
RP5-1142A6.	2222.92597	-0.3374148	0.05874912	-5.7433164	9.28E-09	1.36E-07
RP5-1159O4.	206.980171	-0.6985259	0.14575821	-4.7923606	1.65E-06	1.59E-05
RPIA	1640.3929	0.31186584	0.05541813	5.62750556	1.83E-08	2.52E-07
RPL10	56240.3614	0.1222683	0.03587934	3.40776374	0.00065498	0.003423
RPL13	76008.9454	0.09976441	0.02947435	3.38478681	0.00071234	0.00367328
RPL15	44777.4093	0.07747693	0.03037829	2.55040503	0.01075978	0.036948
RPL27A	13695.6448	0.12900016	0.04535672	2.84412453	0.00445336	0.0176758
RPL3	64780.2986	0.16786238	0.02763888	6.07341404	1.25E-09	2.07E-08
RPL4	94644.6952	0.08112239	0.02943736	2.75576309	0.00585554	0.02229482
RPL6	38156.6407	0.12212834	0.0317841	3.84243542	0.00012182	0.00077585
RPLP0	78343.3021	0.18917	0.0292795	6.46083376	1.04E-10	2.00E-09
RPN1	15496.6396	0.11149786	0.03313024	3.36544049	0.00076422	0.00391644
RPN2	18251.0103	0.18531441	0.03957237	4.68292401	2.83E-06	2.60E-05
RPP25	1765.46762	0.43983424	0.05509939	7.98256081	1.43E-15	4.88E-14
RPP25L	886.033692	0.24335041	0.0710911	3.4230785	0.00061916	0.00326012
RPRD1A	5853.44799	0.13575273	0.03772422	3.59855608	0.00031999	0.00183885
RPRD2	8214.0762	0.26123026	0.03671847	7.11440993	1.12E-12	2.73E-11
RPS2	74922.7782	0.1958041	0.03273032	5.9823467	2.20E-09	3.54E-08
RPS2P5	5454.08918	0.29655019	0.04767818	6.21983016	4.98E-10	8.69E-09
RPS3	101272.369	0.21654337	0.03680349	5.88377234	4.01E-09	6.23E-08
RPS3A	33347.2657	0.07648761	0.03116414	2.45434673	0.01411408	0.04577012
RPS4X	61461.8565	0.08185186	0.03200754	2.55726783	0.0105498	0.03635015
RPS5	32039.3878	0.18713156	0.03115802	6.00588662	1.90E-09	3.09E-08
RPS6	63143.2071	0.10002185	0.0387233	2.58298907	0.00979484	0.0342144
RPS6KA3	2418.52349	-0.5930001	0.05219155	-11.361994	6.47E-30	5.46E-28
RPS9	26465.2531	0.08491279	0.0342784	2.47715187	0.01324355	0.04349477
RPSA	44400.6484	0.20184439	0.02861938	7.05271748	1.75E-12	4.17E-11
RRAGD	522.568112	-0.4392837	0.09066015	-4.8453884	1.26E-06	1.25E-05
RRM1	5470.03093	0.149753	0.0405971	3.68876133	0.00022535	0.00134545
RRM2B	5780.48555	0.17288021	0.03836221	4.50652331	6.59E-06	5.63E-05
RRP1B	4779.43541	0.10310694	0.04081619	2.52612813	0.01153274	0.03902895
RTKN	644.748922	0.39464817	0.08417444	4.68845615	2.75E-06	2.55E-05
RTN3	11746.0571	0.10209816	0.03552031	2.87436049	0.00404847	0.01631375
RUNX1	3334.37215	-0.5671722	0.04532108	-12.514535	6.22E-36	7.27E-34
RUNX2	348.503475	-0.7286815	0.10923734	-6.6706266	2.55E-11	5.31E-10
RUSC1-AS1	1969.21388	-0.2548892	0.05414058	-4.7079145	2.50E-06	2.33E-05

RUVBL1	4993.99693	0.12158834	0.03903514	3.11484349	0.00184043	0.00834576
S100A10	14022.9948	-0.5928718	0.09670358	-6.1308152	8.74E-10	1.48E-08
S100A14	5071.5625	-0.8204988	0.05342738	-15.357273	3.17E-53	8.21E-51
S100A16	3534.36955	-0.9732706	0.04549569	-21.392588	1.57E-101	1.30E-98
S100A6	790.937315	-0.7038738	0.2108287	-3.3386053	0.000842	0.00424117
S100A7	64.5659017	-1.0596021	0.25646571	-4.1315547	3.60E-05	0.00025969
S100A7A	20.4477946	-1.2294002	0.44566385	-2.7585818	0.00580528	0.02214531
S100A9	2327.79093	-0.2505506	0.0542232	-4.6207275	3.82E-06	3.43E-05
S100P	1425.57129	-0.5257687	0.07875377	-6.6761086	2.45E-11	5.12E-10
S1PR5	195.516109	0.62704611	0.14777582	4.24322536	2.20E-05	0.00016677
SACS	1028.52383	0.53904228	0.07864407	6.85420107	7.17E-12	1.58E-10
SAE1	9419.74509	0.22466521	0.03391503	6.62435578	3.49E-11	7.15E-10
SALL2	736.041027	0.41461129	0.08508272	4.87303745	1.10E-06	1.10E-05
SALL4	1515.47031	-0.2737098	0.05866471	-4.6656633	3.08E-06	2.81E-05
SAMD11	1691.68955	0.36806703	0.07451612	4.93942832	7.84E-07	8.07E-06
SAMD12	1227.77542	-0.349689	0.0752143	-4.6492353	3.33E-06	3.02E-05
SAMD14	872.483735	0.36584545	0.08016377	4.5637259	5.03E-06	4.41E-05
SAMD15	618.867845	0.29846482	0.0899345	3.31869115	0.0009044	0.00451353
SAMD4B	5185.64224	0.20805072	0.04243963	4.90227467	9.47E-07	9.62E-06
SAP130	3613.00572	0.15197478	0.04353531	3.49083958	0.00048151	0.00262946
SAR1B	4521.81718	-0.1260552	0.04278292	-2.9463919	0.00321505	0.01338734
SART3	4153.61156	0.1810012	0.0445786	4.06027139	4.90E-05	0.0003423
SAT1	1476.87771	-0.381666	0.05900346	-6.4685364	9.90E-11	1.91E-09
SBNO1	6852.95182	0.17984321	0.03870866	4.64607144	3.38E-06	3.07E-05
SBSN	29.572514	-1.5028532	0.37342131	-4.0245512	5.71E-05	0.00039263
SCAMP2	7560.24332	0.16019628	0.03898522	4.10915387	3.97E-05	0.00028385
SCAMP3	3484.26013	0.2638416	0.05364002	4.91874579	8.71E-07	8.89E-06
SCAMP5	2075.67609	0.49367098	0.05560366	8.87838989	6.78E-19	2.91E-17
SCAP	3912.44112	0.17937489	0.04624915	3.87844703	0.00010513	0.00067995
SCAPER	899.627929	-0.2067402	0.07136399	-2.8969815	0.00376772	0.01532812
SCARA3	774.29051	-0.2823402	0.08688071	-3.2497456	0.00115508	0.0055778
SCARB1	6835.7551	0.34387931	0.04398963	7.81728076	5.40E-15	1.72E-13
SCARB2	7579.86754	-0.1251957	0.03809836	-3.2861165	0.00101579	0.00498571
SCARNA15__	206.795421	-0.6289928	0.13935187	-4.5137021	6.37E-06	5.47E-05
SCART1	159.580846	-1.4628263	0.17437711	-8.3888662	4.91E-17	1.83E-15
SCCPDH	5245.02308	-0.3781591	0.04239518	-8.9198606	4.67E-19	2.03E-17
SCD	43572.6858	0.28885479	0.03488598	8.2799689	1.23E-16	4.48E-15
SCIN	51.0510033	-1.4109176	0.31083699	-4.5390917	5.65E-06	4.91E-05
SCML1	861.784286	-0.5946501	0.07851658	-7.573561	3.63E-14	1.06E-12
SCN5A	33.6944385	1.101392	0.34197506	3.22067931	0.00127887	0.00610923
SCNN1A	473.251971	-0.8352157	0.11003142	-7.5907021	3.18E-14	9.35E-13
SCNN1B	109.979414	-1.4372558	0.20462349	-7.0239043	2.16E-12	5.05E-11
SCNN1D	120.071057	-1.0568337	0.19464578	-5.4295226	5.65E-08	7.15E-07

SCNN1G	41.2349822	-1.0227778	0.32383899	-3.1582912	0.00158697	0.0073481
SCRN1	10692.7169	0.14343121	0.03831237	3.74373097	0.00018131	0.00110753
SCRN2	1061.86642	-0.3630299	0.07298236	-4.9742145	6.55E-07	6.88E-06
SCX	366.701507	-0.738695	0.12261763	-6.0243789	1.70E-09	2.78E-08
SCYL2	5390.23661	0.10371811	0.03887147	2.66823245	0.00762515	0.0278687
SDR39U1	2731.17143	-0.3063101	0.04720445	-6.4890092	8.64E-11	1.68E-09
SEC22B	7866.84283	-0.1114571	0.03818693	-2.9187226	0.00351469	0.01442422
SEC24C	4767.75446	0.22322113	0.05162125	4.32420975	1.53E-05	0.00012016
SEC31A	9782.37546	0.13068463	0.03717548	3.51534479	0.00043918	0.00242595
SEC61A1	20073.1763	0.2229505	0.03555299	6.27093507	3.59E-10	6.37E-09
SECTM1	171.275095	-0.5529065	0.16232714	-3.406125	0.00065892	0.00344002
SELENBP1	3558.4721	-0.1888156	0.04651169	-4.0595311	4.92E-05	0.00034313
SEMA3A	16.6726524	1.69175069	0.52212134	3.24014853	0.00119468	0.00574162
SEMA3B	2817.43995	-1.0970833	0.05760803	-19.043931	7.38E-81	3.74E-78
SEMA3F	3312.36532	-0.2584699	0.04718079	-5.4782861	4.29E-08	5.52E-07
SEMA4B	4833.01738	0.23370862	0.04390326	5.32326355	1.02E-07	1.23E-06
SEMA4C	8118.52257	-0.3305574	0.04509187	-7.3307535	2.29E-13	6.02E-12
SEMA4D	1514.71861	0.15533395	0.05785716	2.68478343	0.00725768	0.0266871
SENP1	1720.0298	0.20505021	0.05485621	3.73795816	0.00018552	0.00113175
SENP7	1477.40188	-0.2924233	0.05826295	-5.0190269	5.19E-07	5.57E-06
SEPHS1	6370.55014	0.2557012	0.0368263	6.94343958	3.83E-12	8.66E-11
SEPHS2	7602.96606	0.24150288	0.03465256	6.96926469	3.19E-12	7.31E-11
SERINC2	636.749315	0.45392823	0.08966011	5.06276675	4.13E-07	4.52E-06
SERPINB5	95.0498701	0.58019368	0.20511135	2.82867664	0.00467409	0.0184395
SERPINE1	211.263374	0.36387362	0.13686012	2.65872635	0.00784366	0.02854718
SERPINH1	11222.0735	0.3570487	0.0373056	9.57091384	1.06E-21	5.49E-20
SERTAD1	985.206238	0.31629377	0.0747561	4.23100962	2.33E-05	0.00017479
SERTAD2	951.52657	0.4572917	0.0703949	6.49609138	8.24E-11	1.61E-09
SESN1	1493.48268	0.27240709	0.06350632	4.28944871	1.79E-05	0.00013851
SESN2	1777.44301	0.62554623	0.0560609	11.1583344	6.52E-29	5.19E-27
SETD2	5420.41813	0.12048093	0.04535805	2.65621939	0.00790222	0.02867444
SETD7	9686.40083	0.17224401	0.03960378	4.34918161	1.37E-05	0.000108
SEZ6L2	4556.46655	0.19398187	0.04518721	4.29284906	1.76E-05	0.00013672
SF1	12476.5799	0.0975041	0.03389736	2.87645149	0.00402174	0.0162204
SF3A1	6153.40843	0.14408426	0.04103468	3.51128054	0.00044595	0.00246186
SF3B3	27542.0984	0.15330999	0.03305255	4.63837107	3.51E-06	3.17E-05
SF3B4	10921.7319	0.1590422	0.03365571	4.72556346	2.29E-06	2.15E-05
SFMBT2	843.422522	-0.2222085	0.07497223	-2.963877	0.0030379	0.01275602
SFN	4165.51004	-0.205503	0.04266467	-4.8167011	1.46E-06	1.42E-05
SFR1	360.264488	-0.3121386	0.10802529	-2.8894953	0.00385861	0.01563855
SFXN2	4718.3171	0.47394481	0.04163806	11.38249	5.11E-30	4.36E-28
SFXN3	1638.78683	-0.4406395	0.05622542	-7.8370163	4.61E-15	1.49E-13
SGCG	1452.03444	0.31864176	0.06061259	5.25702246	1.46E-07	1.73E-06

SGK223	1413.38305	-0.152408	0.06241838	-2.441717	0.0146176	0.04708309
SGMS1	3185.51186	0.17428723	0.0473404	3.68157542	0.0002318	0.00138079
SGOL1	1517.64217	0.23905576	0.05964162	4.00820391	6.12E-05	0.00041705
SGOL2	2157.10522	0.15996479	0.05215457	3.06712884	0.00216126	0.00955123
SGPL1	6764.81117	0.24719988	0.03887149	6.35941433	2.03E-10	3.75E-09
SGPP1	1813.41443	0.40847938	0.05487445	7.44389055	9.78E-14	2.68E-12
SGSM2	6565.37161	-0.1847122	0.03995715	-4.6227556	3.79E-06	3.40E-05
SGTA	2509.87524	0.16007835	0.06086504	2.63005423	0.00853713	0.03055269
SH2B3	400.662796	0.34590773	0.10423099	3.31866506	0.00090449	0.00451353
SH3BGRL	5001.89129	0.23599724	0.04556073	5.17983949	2.22E-07	2.52E-06
SH3BGRL3	2644.39174	0.14177536	0.05063812	2.79977533	0.00511382	0.01990721
SH3BP2	3027.8214	-0.3572578	0.04928031	-7.2495036	4.18E-13	1.07E-11
SH3BP5-AS1	635.338784	-0.7980847	0.08658174	-9.2177026	3.04E-20	1.43E-18
SH3BP5L	1657.51557	0.32440815	0.05984687	5.42063699	5.94E-08	7.46E-07
SH3D19	6252.78463	0.12414671	0.04116291	3.01598453	0.00256146	0.01104677
SH3D21	782.932217	-0.9283526	0.08178642	-11.350939	7.34E-30	6.17E-28
SH3GL1P3	449.53614	-0.3041963	0.1140676	-2.6668071	0.00765756	0.02797595
SH3GLB2	10285.9691	-0.3186717	0.04414586	-7.2186098	5.25E-13	1.32E-11
SH3PXD2B	1274.52276	0.25893573	0.06330764	4.09011841	4.31E-05	0.00030495
SH3RF1	953.917313	0.3079402	0.06950262	4.43062754	9.40E-06	7.73E-05
SHANK3	404.237718	0.28538319	0.10959311	2.60402482	0.00921361	0.03248906
SHC2	261.875311	-0.3080811	0.1273974	-2.4182682	0.01559458	0.04966839
SHCBP1	1955.45445	0.20405232	0.05368845	3.80067469	0.0001443	0.000907
SHISA5	7121.30337	0.10662214	0.04200517	2.53830973	0.01113894	0.03796399
SHISA9	596.496271	-0.6459326	0.0843071	-7.6616622	1.84E-14	5.50E-13
SHKBP1	4521.2015	0.25064554	0.05226567	4.79560575	1.62E-06	1.57E-05
SHMT2	6883.01702	0.50870089	0.0396086	12.8431942	9.39E-38	1.17E-35
SHOC2	2950.89364	0.11925299	0.04532289	2.6311867	0.00850873	0.03048097
SHROOM3	2677.37738	0.26820599	0.04988775	5.37618915	7.61E-08	9.39E-07
SIGLEC15	100.265475	0.4982566	0.1951085	2.55374117	0.01065725	0.03667976
SIGMAR1	1313.50569	0.21710739	0.06641435	3.26898322	0.00107935	0.00524402
SIKE1	7313.76527	-0.1429029	0.03984767	-3.586231	0.00033549	0.00191767
SIM1	19.0615875	-3.699795	0.63980732	-5.7826707	7.35E-09	1.10E-07
SIN3A	9039.34059	0.25923341	0.04101785	6.32001541	2.62E-10	4.75E-09
SIN3B	3058.98336	-0.1476618	0.0448014	-3.2959197	0.000981	0.00484315
SIPA1L2	3045.55197	0.19806608	0.04836616	4.09513756	4.22E-05	0.000299
SIRT1	2244.52561	0.16038882	0.05090768	3.15058215	0.00162945	0.00751311
SIX2	233.044058	0.75219282	0.13565289	5.5449821	2.94E-08	3.91E-07
SKAP2	2387.17443	0.1375124	0.04926286	2.79140103	0.00524804	0.0203645
SKOR1	30.7786399	-0.9270806	0.37283114	-2.4865965	0.01289716	0.0426104
SLC10A4	9.4708443	-2.02385	0.70558739	-2.8683194	0.00412659	0.01656996
SLC12A6	1148.43393	0.170995	0.06278092	2.723678	0.00645594	0.0241869
SLC15A3	59.9528801	-0.965405	0.27740375	-3.480144	0.00050114	0.00271879

SLC16A1	441.220836	-0.2926152	0.10090121	-2.900017	0.00373142	0.0152042
SLC16A2	1105.96654	0.2254266	0.07078112	3.18484098	0.00144834	0.00678568
SLC16A8	45.0065173	-0.9857977	0.29778867	-3.3103937	0.00093165	0.00463385
SLC17A9	83.4858991	1.32765795	0.22360608	5.93748596	2.89E-09	4.59E-08
SLC20A2	1519.56218	0.14257114	0.05868038	2.42962195	0.01511458	0.0483847
SLC22A5	2902.09812	-0.3800459	0.04500015	-8.4454348	3.03E-17	1.15E-15
SLC25A15	1168.7606	0.22780316	0.06545097	3.48051618	0.00050045	0.00271583
SLC25A16	1029.34694	-0.3572161	0.07324323	-4.8771205	1.08E-06	1.08E-05
SLC25A24	13158.0784	-0.2910836	0.03333375	-8.7323972	2.49E-18	1.02E-16
SLC25A25-AS	1096.98037	-0.3462507	0.06882687	-5.030749	4.89E-07	5.27E-06
SLC25A28	948.365585	-0.2784305	0.06836143	-4.0729176	4.64E-05	0.0003256
SLC25A29	7168.25421	-0.1779967	0.04071658	-4.3716039	1.23E-05	9.86E-05
SLC25A30	2089.423	0.14845093	0.05188164	2.86133836	0.00421857	0.01687462
SLC25A36	4913.46313	-0.294533	0.03930993	-7.4925857	6.75E-14	1.88E-12
SLC25A38	3215.81182	0.19166073	0.0439393	4.36194293	1.29E-05	0.00010256
SLC25A39	11679.0389	0.17018438	0.03894146	4.37026148	1.24E-05	9.92E-05
SLC25A46	2710.08747	-0.213776	0.04700519	-4.5479238	5.42E-06	4.73E-05
SLC25A5	35593.0266	0.08873887	0.02934584	3.02389938	0.00249539	0.01079758
SLC25A6	23295.1243	0.2750137	0.03352657	8.20285764	2.35E-16	8.34E-15
SLC26A2	2301.01841	-0.5220462	0.05246332	-9.9506905	2.50E-23	1.45E-21
SLC28A3	26.3150631	-1.1713336	0.41407538	-2.828793	0.00467239	0.01843678
SLC29A1	2019.55049	0.16743217	0.05664046	2.95605263	0.00311604	0.01304374
SLC29A3	1281.66976	0.40279541	0.0614094	6.5591816	5.41E-11	1.08E-09
SLC2A12	86.0262258	-0.7918976	0.23162023	-3.4189483	0.00062864	0.00330428
SLC30A1	3158.74918	0.15649611	0.04431713	3.53127839	0.00041356	0.00229692
SLC30A5	4907.66575	0.13002079	0.04149057	3.1337435	0.00172592	0.00789126
SLC30A7	988.729499	-0.2752731	0.07404431	-3.7176811	0.00020106	0.00121556
SLC34A3	46.1620011	-0.7873894	0.30267638	-2.6014235	0.00928378	0.03270487
SLC35B2	4510.94918	0.31808341	0.0401029	7.93168161	2.16E-15	7.29E-14
SLC35C1	4505.43507	0.69742002	0.04693753	14.8584725	6.13E-50	1.32E-47
SLC35D2	1533.20482	0.17266163	0.0634261	2.72224901	0.00648393	0.0242718
SLC35E2B	7876.7512	0.15897146	0.03911475	4.06423314	4.82E-05	0.00033693
SLC37A1	4198.06195	0.29479963	0.04206521	7.00815704	2.41E-12	5.60E-11
SLC38A7	898.940976	0.33719704	0.07381205	4.5683196	4.92E-06	4.32E-05
SLC39A1	9535.22526	0.21405733	0.03412979	6.27186174	3.57E-10	6.34E-09
SLC39A4	1241.24945	0.49118647	0.06672233	7.36165083	1.82E-13	4.85E-12
SLC39A6	150756.202	0.32138262	0.0286893	11.2021779	3.98E-29	3.21E-27
SLC39A8	1392.31595	-0.5592765	0.06137581	-9.1123276	8.06E-20	3.68E-18
SLC41A1	3107.33167	0.36511743	0.04839427	7.54464159	4.54E-14	1.30E-12
SLC43A1	159.610414	1.0583289	0.164227	6.4443051	1.16E-10	2.22E-09
SLC46A1	1587.09289	0.25629331	0.06269195	4.08813787	4.35E-05	0.00030721
SLC46A3	984.332667	-0.2490263	0.0743755	-3.348231	0.00081329	0.00412276
SLC47A1	162.515156	0.48910182	0.15870664	3.08179804	0.00205754	0.00914609

SLC4A10__TI	1248.69784	0.69486931	0.06288676	11.0495321	2.20E-28	1.72E-26
SLC7A1	7549.06487	0.34610822	0.03973663	8.71005455	3.04E-18	1.24E-16
SLC8A1	270.866863	0.45306869	0.12452981	3.63823485	0.00027451	0.00160844
SLC9A2	2799.17344	-0.1753158	0.05126303	-3.4199259	0.00062638	0.00329433
SLC9A3	162.907338	-0.5249494	0.16389399	-3.2029815	0.00136013	0.00643836
SLC9A6	1450.25038	0.2363027	0.05819518	4.06052019	4.90E-05	0.00034207
SLC9A7	3456.89918	-0.2432261	0.04692754	-5.1830151	2.18E-07	2.48E-06
SLC9A8	2063.32748	-0.3369503	0.05079424	-6.6336324	3.28E-11	6.76E-10
SLCO3A1	1163.67545	-0.45588	0.06375242	-7.1507868	8.63E-13	2.11E-11
SLFN5	1888.62262	0.32856501	0.05644642	5.82082958	5.86E-09	8.89E-08
SLITRK1	61.1876671	0.83701744	0.25545301	3.27660047	0.00105065	0.00511823
SLK	3670.25966	-0.1835341	0.04649796	-3.9471427	7.91E-05	0.00052594
SLPI	67.2585269	-1.3191549	0.25697315	-5.1334348	2.85E-07	3.20E-06
SLX1A__SUL	129.452602	-0.6611804	0.17704947	-3.7344388	0.00018813	0.00114577
SMAD1	433.302387	0.32205699	0.10401353	3.09629913	0.00195953	0.00878529
SMAD1-AS1	90.1368601	0.85702757	0.21650409	3.95848219	7.54E-05	0.00050527
SMAD6	1399.29543	0.26041963	0.06237224	4.17524925	2.98E-05	0.0002185
SMAGP	859.287845	0.22842295	0.07310994	3.12437599	0.00178183	0.00810827
SMARCA2	4353.78853	0.17629482	0.04542537	3.88097736	0.00010404	0.00067366
SMARCA5	8454.72493	0.12658392	0.03583458	3.53245134	0.00041173	0.00228815
SMARCA5-AS	355.122345	0.41334372	0.12432048	3.32482389	0.00088474	0.00443077
SMARCAL1	1312.47747	0.25841214	0.06148559	4.20280808	2.64E-05	0.00019556
SMARCB1	4386.77872	0.24891024	0.04252024	5.85392352	4.80E-09	7.36E-08
SMARCC1	8648.41691	0.27985984	0.0397605	7.03864068	1.94E-12	4.57E-11
SMARCC2	9790.45075	0.15417102	0.0354864	4.34450985	1.40E-05	0.00011018
SMARCD2	27207.1367	0.10321945	0.03526746	2.9267619	0.00342511	0.0141074
SMCR8	3340.58169	0.34967011	0.05160803	6.77549798	1.24E-11	2.67E-10
SMG6	2145.64007	0.16531768	0.05374218	3.07612554	0.0020971	0.00931055
SMG7	8407.18972	0.12794239	0.03836833	3.33458315	0.00085427	0.00429587
SMOC2	106.172625	1.00578506	0.20149185	4.99169112	5.99E-07	6.33E-06
SMPD2	854.821551	0.30293545	0.07197904	4.20866177	2.57E-05	0.00019103
SMPDL3B	541.054724	1.32684954	0.09403965	14.1094685	3.32E-45	5.82E-43
SMS	7087.36986	0.12606931	0.03754831	3.35752308	0.00078644	0.00401231
SMTN	2274.44644	0.37201553	0.05132973	7.24756418	4.24E-13	1.08E-11
SMTNL2	253.18882	-0.4185492	0.13175918	-3.1766227	0.00149001	0.00694499
SMURF1	4850.01326	0.20635314	0.03978289	5.18698214	2.14E-07	2.43E-06
SMURF2	3430.13234	0.12100798	0.04225655	2.86365013	0.0041879	0.01677166
SNAI1	151.619285	-0.4611478	0.16016144	-2.8792682	0.00398599	0.01609758
SNAP47	1321.13463	0.1601173	0.06308899	2.53795948	0.01115009	0.0379949
SND1	10833.4984	0.12481947	0.03290121	3.79376561	0.00014838	0.00092815
SNED1	642.171202	-0.2578667	0.08896221	-2.8986093	0.00374822	0.01526239
SNHG11__SI	356.31375	-0.4334439	0.11020236	-3.9331632	8.38E-05	0.00055466
SNHG12__SI	531.466251	-0.6143282	0.0913066	-6.7281905	1.72E-11	3.65E-10

SNHG16__SN	2879.56494	0.17335295	0.05805433	2.98604671	0.0028261	0.01201469
SNHG1__SN	4541.63844	-0.4141483	0.04866702	-8.5098347	1.74E-17	6.76E-16
SNHG7__SN	3207.57461	-0.2164099	0.04550611	-4.7556224	1.98E-06	1.88E-05
SNHG8__SN	1567.99387	0.17963231	0.06070372	2.95916472	0.00308474	0.01293039
SNN	1497.88641	0.14389322	0.05829721	2.46826955	0.0135768	0.04446909
SNRNP70	9726.49687	-0.1138757	0.03816048	-2.9841269	0.00284389	0.0120847
SNRPA1	5539.17569	-0.3084919	0.03813031	-8.0904645	5.94E-16	2.07E-14
SNX1	6710.89228	0.10290502	0.03754568	2.74079534	0.00612907	0.02313339
SNX17	5364.31978	0.15356014	0.04129752	3.7183862	0.0002005	0.00121257
SNX18	1438.32008	0.20697859	0.06335273	3.26708226	0.00108662	0.00527655
SNX25	337.492526	0.33027628	0.10854203	3.04284228	0.00234355	0.01022304
SNX29	1581.39242	0.20919895	0.05834313	3.58566535	0.00033622	0.00192123
SNX33	2886.0881	0.29472205	0.05245659	5.61839942	1.93E-08	2.65E-07
SNX8	2000.04402	0.20660516	0.05267661	3.92214208	8.78E-05	0.00057857
SOAT1	1651.84022	0.46565314	0.06166154	7.55176038	4.29E-14	1.24E-12
SOCS2	1846.39871	0.41781395	0.05277258	7.91725502	2.43E-15	8.12E-14
SOD2	3427.78287	-0.4657054	0.04486856	-10.379326	3.08E-25	2.02E-23
SOGA1	5762.31561	0.35917449	0.04574768	7.85120655	4.12E-15	1.34E-13
SORBS3	1030.8043	-0.31346	0.06954745	-4.5071383	6.57E-06	5.62E-05
SORCS1	151.513457	-0.6947638	0.16535301	-4.2017002	2.65E-05	0.00019636
SORCS2	592.177814	-0.2519263	0.09374763	-2.6872813	0.00720363	0.02653018
SORD	1620.00923	0.44790001	0.05593383	8.00767665	1.17E-15	4.00E-14
SORD2P	138.793648	0.40808767	0.16839477	2.42339871	0.01537604	0.04909241
SORL1	444.604002	-0.3317971	0.09744785	-3.4048686	0.00066196	0.00345224
SOWAHC	724.77058	0.28503715	0.0785177	3.63022799	0.00028317	0.00165545
SOX11	49.578565	-2.6209634	0.33091683	-7.9203085	2.37E-15	7.94E-14
SOX12	4989.10712	0.16420422	0.0448128	3.66422598	0.00024809	0.00147062
SOX2	2386.21151	0.18795143	0.05019412	3.74449091	0.00018076	0.00110529
SPAG4	309.013147	-1.1555124	0.12354169	-9.3532186	8.50E-21	4.15E-19
SPAST	2049.11364	0.21345151	0.05354683	3.98625833	6.71E-05	0.00045432
SPATA17	190.67978	0.80464389	0.14490812	5.55278664	2.81E-08	3.76E-07
SPATA2	2604.72846	0.27698827	0.04911893	5.63913475	1.71E-08	2.38E-07
SPATA9	200.195914	-0.4327356	0.14569227	-2.9702031	0.00297603	0.0125381
SPATS2	3644.48067	0.16185585	0.04298225	3.76564399	0.00016612	0.00102471
SPEN	6346.11306	0.14422534	0.05915543	2.4380742	0.01476574	0.04748475
SPG7	5305.71774	-0.1220505	0.0425066	-2.8713297	0.00408749	0.01644193
SPIN4	1212.86086	0.16381296	0.06381072	2.56716982	0.01025324	0.0355321
SPINK7	15.3858879	2.80363915	0.60776998	4.61299381	3.97E-06	3.56E-05
SPINT1	6173.26862	-0.1138527	0.04554305	-2.499892	0.01242312	0.04138204
SPIRE2	920.250282	-0.262309	0.0717798	-3.6543574	0.00025783	0.00152094
SPNS2	221.395757	-1.2138809	0.14514854	-8.3630257	6.11E-17	2.26E-15
SPOCK1	244.485185	-0.328786	0.12778903	-2.5728809	0.01008559	0.03504872
SPOP	5418.91542	0.32560334	0.03808349	8.54972393	1.23E-17	4.85E-16

SPPL3	3396.11252	0.24894249	0.0490332	5.0770189	3.83E-07	4.22E-06
SPR	3714.71657	0.41478143	0.04284393	9.68121799	3.62E-22	1.94E-20
SPRYD3	3628.66031	0.31376525	0.04543781	6.90537821	5.01E-12	1.12E-10
SPTBN2	6149.8086	0.29199547	0.05145974	5.67425048	1.39E-08	1.97E-07
SQLE	11788.1511	-0.1309609	0.03342239	-3.9183581	8.92E-05	0.0005873
SRC	1987.10125	0.37940952	0.05259224	7.21417355	5.43E-13	1.36E-11
SRD5A1	1474.76467	0.24714647	0.06101791	4.05039254	5.11E-05	0.00035545
SREBF1	8557.09528	0.23151144	0.05083671	4.55402098	5.26E-06	4.61E-05
SREBF2	6434.30513	0.25769093	0.04038742	6.38047592	1.77E-10	3.29E-09
SRGAP1	1838.15708	-0.5291071	0.05853209	-9.0396072	1.57E-19	7.04E-18
SRGAP2	3782.55957	0.11018182	0.04153558	2.65270967	0.00798485	0.02892249
SRP14	7453.83892	0.17610025	0.04552388	3.86830476	0.0001096	0.00070538
SRP68	5585.85226	0.1029446	0.03950143	2.6060982	0.00915802	0.03234308
SRPK2	5395.0215	0.14924109	0.03829133	3.89751672	9.72E-05	0.00063378
SRPR	6212.75539	0.19836445	0.03714846	5.3397753	9.31E-08	1.13E-06
SRRD	624.680559	0.20698062	0.08531056	2.42620149	0.0152578	0.04875757
SRSF11	7305.90031	-0.3267958	0.03829829	-8.5329078	1.43E-17	5.57E-16
SRSF2	11216.5075	-0.1839945	0.04344718	-4.2349012	2.29E-05	0.00017221
SRSF4	4601.17802	-0.191037	0.03946432	-4.8407528	1.29E-06	1.27E-05
SRSF5	10801.2106	-0.5936074	0.03708883	-16.005019	1.18E-57	3.47E-55
SRSF6	12189.1047	-0.1240171	0.03323277	-3.7317724	0.00019014	0.00115604
SSBP1	4298.16134	-0.1555519	0.05262426	-2.9558976	0.00311761	0.0130445
SSBP2	721.893403	-0.3956065	0.08258621	-4.7902243	1.67E-06	1.61E-05
SSFA2	23290.0716	-0.2961052	0.03214144	-9.2125682	3.18E-20	1.49E-18
SSPO	322.90996	-0.7636687	0.12136831	-6.2921592	3.13E-10	5.61E-09
SSR2	8425.88312	0.1848622	0.03786292	4.88240692	1.05E-06	1.06E-05
SSRP1	7420.57361	0.10721692	0.03853668	2.78220457	0.0053991	0.020884
ST13	10507.1323	0.11754563	0.03527329	3.33242641	0.00086092	0.00432692
ST3GAL1	3114.5048	-0.3060357	0.04764543	-6.4231919	1.33E-10	2.53E-09
ST3GAL4	1347.42593	-0.4365265	0.06348457	-6.8761031	6.15E-12	1.36E-10
ST3GAL5	1488.65307	0.2595457	0.05941327	4.36848067	1.25E-05	9.98E-05
ST6GALNAC2	6339.38392	-0.1206766	0.04681229	-2.5778832	0.00994076	0.0346776
ST7L	570.812474	-0.2263043	0.08697077	-2.6020725	0.00926623	0.03264934
STAG2	10267.1562	0.09382227	0.03646374	2.57302966	0.01008126	0.03504034
STAG3L2	362.764209	-0.352187	0.12067789	-2.9184055	0.00351826	0.01443565
STAG3L3	575.670222	-0.268198	0.08688184	-3.0869285	0.00202236	0.00902832
STARD7	10701.5673	0.12233255	0.0361413	3.38484067	0.0007122	0.00367328
STAT1	13497.0259	-0.5390268	0.03285999	-16.403744	1.80E-60	5.85E-58
STAT3	5287.44404	0.17186523	0.04493651	3.82462331	0.00013097	0.00082979
STAT6	3566.01883	0.65119266	0.04317041	15.0842365	2.06E-51	4.87E-49
STC1	7297.99614	0.72023233	0.03870125	18.6100522	2.66E-77	1.25E-74
STC2	34978.587	-0.2242844	0.02959681	-7.5779951	3.51E-14	1.02E-12
STEAP3	1928.095	0.19430537	0.05419379	3.58538048	0.00033659	0.00192272

STEAP3-AS1	218.874032	0.36611143	0.13776926	2.65742467	0.00787402	0.02860755
STIP1	17813.4662	0.14619944	0.03292365	4.44055977	8.97E-06	7.41E-05
STK10	2518.51333	0.16538148	0.05321923	3.10755137	0.00188644	0.00851631
STK17A	1697.90252	0.23306044	0.06160111	3.78338045	0.00015471	0.00096284
STK26	5262.06572	0.20769012	0.04034898	5.14734551	2.64E-07	2.97E-06
STK38L	1140.61493	0.1945017	0.06305246	3.08476015	0.00203716	0.0090762
STMN1	11235.7481	0.27482868	0.03979768	6.90564547	5.00E-12	1.12E-10
STMN3	2737.43215	0.52606477	0.05025221	10.4684908	1.21E-25	8.17E-24
STOM	5310.913	-0.2247694	0.03839403	-5.8542791	4.79E-09	7.35E-08
STOML2	5297.24863	0.16125638	0.04094326	3.93853311	8.20E-05	0.00054338
STOX1	381.729816	0.40015321	0.10341209	3.86950122	0.00010906	0.00070236
STRA6	81.899271	-1.2917556	0.23620096	-5.4688836	4.53E-08	5.80E-07
STX1B	87.9985235	-1.0097244	0.2228777	-4.5303968	5.89E-06	5.09E-05
STX4	2711.12876	-0.2171664	0.05005029	-4.3389648	1.43E-05	0.0001128
STX6	5703.49304	0.13167585	0.0381225	3.45401953	0.0005523	0.00295846
STXBP3	4089.51994	-0.1175279	0.0427069	-2.7519654	0.00592388	0.02249537
STXBP4	1136.04557	0.16425844	0.06419523	2.55873259	0.01050545	0.03623159
SUFU	1222.16317	0.35547041	0.07055368	5.0382973	4.70E-07	5.08E-06
SULF1	2037.75864	-0.3228994	0.05664479	-5.7004255	1.20E-08	1.71E-07
SULF2	95829.4467	-0.2323779	0.03069165	-7.5713722	3.69E-14	1.07E-12
SULT1A1	724.842034	-0.2569397	0.07674119	-3.3481324	0.00081358	0.00412308
SUMO3	14050.9059	0.22959471	0.03191108	7.19482774	6.25E-13	1.56E-11
SUOX	1393.16723	0.22172497	0.06069864	3.6528818	0.00025931	0.00152921
SUPT6H	9379.94342	0.10539364	0.03783342	2.78572851	0.00534076	0.02068906
SUPT7L	3168.13335	-0.1486065	0.04322564	-3.437925	0.00058619	0.00311077
SURF4	16891.6561	0.21149598	0.03493461	6.0540532	1.41E-09	2.32E-08
SUSD3	106.502554	-0.4888153	0.19476371	-2.5097862	0.01208043	0.04044969
SUV39H2	1452.51405	0.21576825	0.06342468	3.40196047	0.00066904	0.00348155
SVIL	5950.90257	0.26006211	0.04715169	5.51543582	3.48E-08	4.54E-07
SWI5	960.111026	-0.1690525	0.06915605	-2.4445082	0.01450498	0.04678645
SYAP1	7468.93504	0.11431886	0.03633939	3.14586651	0.00165596	0.00761915
SYBU	586.405691	-0.4747657	0.08569262	-5.5403331	3.02E-08	4.01E-07
SYCP3	176.299768	-0.4542334	0.15520421	-2.9266821	0.00342599	0.01410783
SYNGR1	584.453728	-0.4486452	0.09201875	-4.8755844	1.08E-06	1.09E-05
SYNJ2	2481.67325	0.15150321	0.05322663	2.84637977	0.00442194	0.01756256
SYNPO	355.601868	-0.7682571	0.11276069	-6.813164	9.55E-12	2.07E-10
SYT10	1154.23967	0.55442278	0.06636788	8.35378197	6.61E-17	2.43E-15
SYT12	2796.04349	-0.3436266	0.04866539	-7.0610066	1.65E-12	3.95E-11
SYT8	55.8584616	-0.7758729	0.26376487	-2.9415325	0.00326593	0.0135744
SYTL1	2400.88215	-0.4591759	0.05751604	-7.9834404	1.42E-15	4.85E-14
SYTL2	26777.0007	-0.4728002	0.04055004	-11.659674	2.05E-31	1.93E-29
SYTL4	3071.61183	0.51260148	0.04825523	10.6227131	2.34E-26	1.66E-24
SZT2	1401.89571	-0.2029284	0.06710621	-3.0239881	0.00249466	0.01079698

TACC1	1945.40926	-0.3949623	0.05804325	-6.8046207	1.01E-11	2.20E-10
TACC3	5684.76073	0.10894798	0.03787225	2.87672287	0.00401828	0.01621363
TACSTD2	11528.135	-0.3084107	0.03230556	-9.5466766	1.34E-21	6.88E-20
TADA3	6585.42844	0.19251869	0.04134678	4.65619529	3.22E-06	2.93E-05
TAF1C	4026.63238	-0.4195032	0.04803746	-8.7328345	2.48E-18	1.02E-16
TAF4	4027.24266	0.11194376	0.04260015	2.62777834	0.00859445	0.03069955
TAF5	586.364954	0.2996269	0.08688728	3.44845523	0.0005638	0.00300772
TAF6	5490.53627	0.26404329	0.04540292	5.81555714	6.04E-09	9.14E-08
TAF7	11057.7222	0.10298934	0.03555351	2.89674193	0.0037706	0.01533298
TAGLN	103.121105	-0.7263469	0.20344437	-3.5702483	0.00035664	0.00202146
TANC2	28712.5133	0.32247691	0.04645531	6.94165836	3.88E-12	8.75E-11
TANGO6	485.869913	0.3136375	0.10385284	3.02001836	0.00252759	0.01092136
TAOK2	6754.52489	0.11319566	0.03810857	2.97034675	0.00297464	0.01253514
TAPBP__ZBT	8667.41939	-0.1381511	0.03889367	-3.5520188	0.00038229	0.00214944
TARBP1	2003.95244	-0.3232036	0.05119209	-6.3135465	2.73E-10	4.94E-09
TARS	7189.11563	0.33604724	0.03539503	9.49419267	2.22E-21	1.13E-19
TARS2	3905.10582	0.18240961	0.04316682	4.2256901	2.38E-05	0.00017831
TAT	75.7578847	-0.5764006	0.23124312	-2.4926173	0.01268054	0.04203182
TATDN1	3482.03483	-0.3465359	0.04608077	-7.5201847	5.47E-14	1.55E-12
TAZ	1469.61936	-0.4530713	0.06476296	-6.9958408	2.64E-12	6.10E-11
TBC1D1	2507.5386	0.2254908	0.05094933	4.4257854	9.61E-06	7.88E-05
TBC1D14	1205.89399	0.46687103	0.06313347	7.39498454	1.41E-13	3.83E-12
TBC1D16	5554.17471	0.54638683	0.04442086	12.3002305	9.03E-35	9.86E-33
TBC1D3B__T	25.6799732	-1.4701284	0.41091465	-3.5776977	0.00034663	0.00196962
TBC1D3C__T	79.6160393	-0.7437908	0.22182186	-3.3530996	0.00079912	0.00406561
TBC1D4	989.559831	0.19891819	0.07238043	2.74823178	0.00599176	0.02269502
TBC1D8B	1018.67773	0.19208801	0.06724213	2.85666138	0.00428122	0.01708554
TBCD	4777.33019	0.22009966	0.04815916	4.57025565	4.87E-06	4.29E-05
TBL1X	5249.38791	0.21132158	0.04133206	5.11277697	3.17E-07	3.53E-06
TBRG4	4270.57999	0.17410528	0.04826952	3.60694048	0.00030983	0.00178836
TCAF1	5767.15667	0.14422045	0.03984727	3.61933058	0.00029537	0.00171683
TCAIM	1516.7276	0.26507371	0.06465967	4.0995214	4.14E-05	0.00029425
TCF12	2495.80382	0.1503734	0.05018719	2.9962507	0.00273322	0.01167939
TCF20	2380.98536	0.20507843	0.06444375	3.18228601	0.00146117	0.00683155
TCF3	6412.12027	0.18682347	0.04230802	4.41579349	1.01E-05	8.20E-05
TCF7L1	438.593212	0.46676076	0.09753169	4.7857343	1.70E-06	1.63E-05
TCF7L2	1300.36448	0.47303589	0.06022376	7.85463849	4.01E-15	1.31E-13
TCHH	1034.2035	0.3006448	0.07501538	4.00777537	6.13E-05	0.00041749
TCOF1	5742.42499	0.12208651	0.0427216	2.85772333	0.00426692	0.01703592
TDG	4806.9229	0.11270373	0.04087128	2.7575289	0.00582401	0.02220722
TDRKH	3141.59295	0.16258875	0.04469281	3.63791768	0.00027485	0.00160991
TEAD1	5934.98225	0.2187966	0.04412656	4.95838847	7.11E-07	7.38E-06
TEAD2	2476.52476	0.63891736	0.04785562	13.350936	1.17E-40	1.69E-38

TEF	1138.12446	0.28916318	0.06961088	4.15399407	3.27E-05	0.00023844
TESC	181.551791	0.8026762	0.14981023	5.35795317	8.42E-08	1.03E-06
TET1	430.84652	0.26471449	0.09798713	2.70152308	0.00690227	0.02561817
TET2	10401.0301	0.22825044	0.04881523	4.67580402	2.93E-06	2.68E-05
TEX2	6830.93021	0.12780147	0.03839767	3.32836492	0.00087357	0.00438446
TFAMP1	17.5098076	-1.1553918	0.47434406	-2.4357675	0.01486023	0.04775493
TFDP1	5111.67328	0.17523009	0.04097647	4.27635929	1.90E-05	0.00014591
TFF1	50942.2062	-0.3116794	0.09931642	-3.1382463	0.00169962	0.00778861
TFF3	2664.20577	-0.3268221	0.05858391	-5.5787004	2.42E-08	3.28E-07
TFIP11	2402.74109	0.18547948	0.04741891	3.91150895	9.17E-05	0.00060268
TFPI	720.372189	-1.3181834	0.08653562	-15.232842	2.14E-52	5.35E-50
TFPI2	495.768261	0.43108527	0.10068535	4.28150931	1.86E-05	0.00014282
TFR2	145.191748	0.86311105	0.16419175	5.25672592	1.47E-07	1.73E-06
TFRC	20478.8996	0.32589455	0.03199503	10.185786	2.29E-24	1.41E-22
TGFB1	5061.48026	0.3439823	0.04833496	7.11663544	1.11E-12	2.69E-11
TGFB3	266.831022	-0.6979121	0.12367043	-5.6433223	1.67E-08	2.32E-07
TGFBI	1252.69913	0.36166427	0.06537201	5.53240276	3.16E-08	4.16E-07
TGFBRAP1	2491.39015	0.19912527	0.05131001	3.88082691	0.0001041	0.00067384
THAP1	487.2844	0.3315484	0.09210443	3.59970103	0.00031858	0.0018317
THAP10	603.65836	0.2953549	0.08806008	3.35401596	0.00079648	0.00405531
THAP11	2675.79302	0.29077054	0.05042952	5.76587961	8.12E-09	1.20E-07
THAP4	2638.84664	0.18413237	0.05477098	3.36185985	0.00077419	0.00395919
THBD	532.807315	-0.4054528	0.09120996	-4.4452693	8.78E-06	7.27E-05
THBS1	18123.6928	0.20218017	0.03617266	5.5893087	2.28E-08	3.10E-07
THBS3	1125.2823	-0.686696	0.06518131	-10.535167	5.95E-26	4.14E-24
THEMIS2	60.0021336	-1.0263156	0.25651831	-4.0009449	6.31E-05	0.00042909
THG1L	1403.73438	0.15890806	0.06139934	2.58810703	0.0096505	0.03379435
THNSL1	1483.38204	0.26057624	0.06054155	4.30408909	1.68E-05	0.00013077
THOC3	1051.99342	0.2244286	0.06807639	3.29671717	0.00097822	0.00483384
THOP1	2113.64106	0.15773633	0.06282981	2.51053317	0.0120549	0.04037639
THRA	722.598616	0.25517	0.08311158	3.07021012	0.00213908	0.0094624
THRAP3	9471.62085	0.1352045	0.03811766	3.54703024	0.0003896	0.00218315
THRB	2124.15336	0.53362551	0.05737403	9.30081974	1.39E-20	6.67E-19
TIAM1	2171.83837	0.72014536	0.05320403	13.5355422	9.65E-42	1.49E-39
TICRR	3056.80431	0.29426034	0.05193797	5.66561054	1.47E-08	2.06E-07
TIGAR	1236.90402	0.19310877	0.06327452	3.05192002	0.00227383	0.00997377
TIGD5	1075.03813	0.26071978	0.08192488	3.18242505	0.00146047	0.00683003
TIMELESS	8368.29308	0.25745958	0.03795079	6.78403759	1.17E-11	2.52E-10
TIMP1	3936.84232	-0.1942496	0.04362366	-4.4528504	8.47E-06	7.06E-05
TIMP3	1670.93563	-0.8764586	0.05852518	-14.975754	1.06E-50	2.38E-48
TINCR	2172.93837	-0.2183974	0.04945509	-4.4160741	1.01E-05	8.20E-05
TIPARP	2130.77504	-0.1674119	0.05219958	-3.2071501	0.00134057	0.00635734
TJP3	3204.41375	0.2113956	0.05237319	4.03633257	5.43E-05	0.00037457

TK1	5835.30727	0.22905096	0.05343815	4.28628194	1.82E-05	0.00014026
TKT	23012.0749	-0.2967971	0.03783992	-7.84349	4.38E-15	1.41E-13
TLE3	6908.87907	0.29297737	0.03805186	7.69942412	1.37E-14	4.17E-13
TLE6	70.9450215	-0.7120835	0.24445264	-2.912971	0.00358008	0.01465301
TLK2	7545.01466	0.09100837	0.03557831	2.55797337	0.01052842	0.03629706
TLN1	8006.08999	0.15081532	0.03921588	3.84577188	0.00012017	0.00076671
TM4SF1	34.5621476	-1.6192518	0.35621036	-4.5457742	5.47E-06	4.78E-05
TM7SF2__VI	9664.92065	0.25281371	0.04414117	5.72738967	1.02E-08	1.48E-07
TM7SF3	4432.23646	0.29210142	0.04145206	7.04672818	1.83E-12	4.35E-11
TM9SF2	8978.61028	0.11837594	0.0360644	3.28234853	0.00102946	0.0050379
TMBIM6	51691.052	0.19123139	0.03132719	6.10432557	1.03E-09	1.72E-08
TMC6	1896.76787	-0.2339436	0.05607818	-4.1717404	3.02E-05	0.00022171
TMED8	5718.81568	0.17354724	0.04221586	4.11094899	3.94E-05	0.00028198
TMEM101	1398.86327	0.27925074	0.0616083	4.53268024	5.82E-06	5.05E-05
TMEM102	1028.61005	0.35746763	0.08035773	4.44845372	8.65E-06	7.18E-05
TMEM105	178.70044	-0.651221	0.15387867	-4.2320419	2.32E-05	0.00017412
TMEM106C	6021.12595	0.25942301	0.03672198	7.06451674	1.61E-12	3.86E-11
TMEM107	478.810086	-0.2758564	0.09762363	-2.8257136	0.00471754	0.01859081
TMEM120B	1833.26767	-0.1750183	0.05900886	-2.9659668	0.00301733	0.01268573
TMEM127	4450.53733	0.11711193	0.04000446	2.927472	0.0034173	0.01408477
TMEM134	1721.74623	-0.2241526	0.05958868	-3.7616634	0.00016879	0.0010387
TMEM139	31.6981037	-1.2162633	0.37344582	-3.2568668	0.00112649	0.00544985
TMEM147-A	538.074999	-0.3359641	0.08942863	-3.7567849	0.00017211	0.00105737
TMEM150A	828.589175	0.2656343	0.07317626	3.6300615	0.00028335	0.00165598
TMEM161A	1576.71723	0.1498358	0.05997256	2.49840594	0.01247532	0.04150248
TMEM161B	1442.24722	-0.4002296	0.05753013	-6.9568691	3.48E-12	7.93E-11
TMEM161B-	204.181558	-0.5173147	0.15192372	-3.4050947	0.00066141	0.00345069
TMEM167A	6070.62018	-0.2473162	0.04042858	-6.1173611	9.51E-10	1.60E-08
TMEM167B	3147.53835	-0.1488043	0.0471079	-3.158798	0.00158421	0.00734279
TMEM173	60.6285919	-0.9142749	0.2564529	-3.5650794	0.00036375	0.00205916
TMEM175	801.776771	-0.2373666	0.0847912	-2.7994246	0.00511938	0.01992034
TMEM178A	305.576619	-0.7279837	0.12070191	-6.0312528	1.63E-09	2.67E-08
TMEM18	2728.09545	-0.1670672	0.04747469	-3.5190794	0.00043305	0.00239714
TMEM184B	3660.54211	0.36919632	0.04460848	8.27637062	1.27E-16	4.60E-15
TMEM185B	2001.07412	0.28230849	0.0511987	5.51397775	3.51E-08	4.58E-07
TMEM192	2289.55212	0.21355667	0.05056033	4.22379916	2.40E-05	0.00017951
TMEM198B	859.424891	-0.7885088	0.0737618	-10.689935	1.13E-26	8.18E-25
TMEM214	4370.81576	0.11207127	0.04479624	2.50180077	0.01235634	0.0412421
TMEM238	832.780709	0.92102468	0.09283894	9.92067185	3.38E-23	1.95E-21
TMEM259	3670.83095	-0.2265222	0.05360462	-4.2257954	2.38E-05	0.0001783
TMEM26	1230.77897	0.5549415	0.0638845	8.68663794	3.73E-18	1.51E-16
TMEM26-AS	65.9164496	1.10856849	0.2532107	4.37804749	1.20E-05	9.60E-05
TMEM41B	1220.87222	-0.1775486	0.06591735	-2.6935034	0.00707054	0.02614567

TMEM45B	203.29881	-0.7309398	0.13913367	-5.2535074	1.49E-07	1.75E-06
TMEM51-AS	134.203801	-0.4522386	0.17990877	-2.5137109	0.01194683	0.04013237
TMEM52	178.219948	0.44410678	0.14853875	2.98983783	0.00279126	0.01189396
TMEM54	2738.82639	0.38228474	0.05540759	6.89950182	5.22E-12	1.17E-10
TMEM59L	166.246627	-0.9373472	0.1606172	-5.8359077	5.35E-09	8.17E-08
TMEM63A	2326.35665	-0.1513329	0.05931952	-2.5511485	0.01073686	0.03690402
TMEM63B	3259.58486	0.20434406	0.04333617	4.71532335	2.41E-06	2.25E-05
TMEM64	42090.5765	-0.2863381	0.04920542	-5.8192382	5.91E-09	8.95E-08
TMEM74B	22.4854155	-2.0409838	0.45290528	-4.5064253	6.59E-06	5.63E-05
TMEM8A	5422.11787	0.30110195	0.05295266	5.68624818	1.30E-08	1.84E-07
TMEM8B	322.280746	-0.4187886	0.11069974	-3.7831036	0.00015489	0.00096355
TMOD4__VP	4743.50717	0.2060749	0.04080233	5.05056708	4.41E-07	4.79E-06
TMPO	14733.47	0.15413976	0.03349956	4.60124699	4.20E-06	3.74E-05
TMPRSS2	893.098772	0.52111773	0.07306675	7.13207727	9.89E-13	2.41E-11
TMPRSS6	57.0550866	-0.7098548	0.26370361	-2.691866	0.00710535	0.02623713
TMTC1	447.813617	-0.7151547	0.0970267	-7.3707003	1.70E-13	4.54E-12
TNFAIP1	3159.10936	0.16452318	0.04833752	3.40363337	0.00066496	0.00346623
TNFAIP3	815.004108	-0.2251727	0.07288918	-3.0892469	0.00200665	0.00896574
TNFAIP8L1	540.602753	0.45765397	0.0873705	5.23808342	1.62E-07	1.89E-06
TNFRSF11B	617.154402	-0.5395151	0.09487971	-5.6863061	1.30E-08	1.84E-07
TNKS2	5497.9948	0.10433874	0.03956733	2.6369921	0.00836448	0.03007056
TNPO2	6078.97039	0.20825902	0.04110386	5.06665347	4.05E-07	4.44E-06
TNPO3	5258.34677	0.13569756	0.03924136	3.45802434	0.00054415	0.00292255
TNRC18	16658.7509	0.34088585	0.04352284	7.83234319	4.79E-15	1.54E-13
TNRC6A	5427.29582	0.10657523	0.03893063	2.7375674	0.00618954	0.02332785
TNRC6B	1433.52509	0.2493014	0.05900302	4.22523148	2.39E-05	0.0001786
TNS2	543.766734	-0.4268252	0.08900673	-4.7954259	1.62E-06	1.57E-05
TOB2	4425.17595	0.4703833	0.04047844	11.620589	3.24E-31	3.01E-29
TOM1L1	4182.42899	0.33294753	0.04376333	7.60791107	2.79E-14	8.26E-13
TOP1MT	2554.11448	0.22291007	0.0472156	4.72111064	2.35E-06	2.19E-05
TOP2A	18019.9138	0.33947046	0.03147589	10.7850948	4.05E-27	2.99E-25
TOP3B	1157.72345	0.22238536	0.0638472	3.4830873	0.00049567	0.00269469
TOPBP1	6249.64422	0.09564776	0.03763018	2.54178334	0.01102885	0.0377006
TP53INP1	4282.72398	0.3734887	0.04268214	8.75046814	2.12E-18	8.75E-17
TP63	370.528087	0.30515009	0.10507035	2.90424541	0.0036814	0.01503394
TPBG	13147.3035	-0.1318656	0.04201373	-3.1386316	0.00169739	0.00778229
TPCN1	2186.8452	-0.2583929	0.0536685	-4.8146112	1.47E-06	1.44E-05
TPD52L2	15693.5673	0.17070877	0.03137463	5.44098051	5.30E-08	6.73E-07
TPGS2	3453.36978	0.18083599	0.04725147	3.82709792	0.00012966	0.00082207
TPM1	8371.04027	-0.6908383	0.03622322	-19.071698	4.34E-81	2.26E-78
TPM2	379.912733	-0.9273621	0.10834062	-8.5596902	1.13E-17	4.47E-16
TPM4	36883.5998	0.10082429	0.02923196	3.44911138	0.00056244	0.00300306
TPT1-AS1	275.561742	-0.4680841	0.1297592	-3.6073288	0.00030937	0.00178625

TPTE	798.687264	-0.2228436	0.07481085	-2.9787603	0.00289417	0.01225838
TPX2	10174.5816	0.366291	0.03474805	10.5413387	5.57E-26	3.89E-24
TRAF5	1298.6486	-0.555096	0.06090037	-9.1148213	7.88E-20	3.60E-18
TRAF7	7891.75687	0.15885597	0.04180283	3.80012525	0.00014462	0.0009087
TRAFD1	4064.45767	0.30623048	0.04083779	7.49870446	6.45E-14	1.81E-12
TRAM1	9418.51611	0.10188165	0.03837209	2.65509768	0.00792855	0.02875471
TRAM2	2504.37557	0.18234639	0.05462236	3.33831029	0.0008429	0.00424334
TRANK1	1605.54908	0.4926866	0.07195343	6.84729799	7.53E-12	1.65E-10
TRAPPC2	791.240267	-0.2981252	0.07862926	-3.79153	0.00014972	0.0009359
TRAPPC2L	3069.36157	0.20550003	0.04496081	4.57064782	4.86E-06	4.28E-05
TRAPPC6A	1972.3053	0.31406329	0.05499961	5.71028249	1.13E-08	1.62E-07
TRAPPC6B	2396.41924	-0.2803915	0.051093	-5.4878643	4.07E-08	5.26E-07
TRAV30	22.302192	-1.830693	0.44606926	-4.1040554	4.06E-05	0.00028939
TRIAP1	2032.80596	0.18090956	0.05377079	3.36445785	0.00076694	0.00392931
TRIB2	873.272285	-1.677782	0.07902492	-21.231049	4.93E-100	3.91E-97
TRIM16L	1137.31395	-0.6027101	0.07082781	-8.5095132	1.75E-17	6.76E-16
TRIM24	4363.00608	0.16630164	0.04116565	4.0398162	5.35E-05	0.00036989
TRIM28	32461.8406	0.24667915	0.04825864	5.11160644	3.19E-07	3.55E-06
TRIM29	204.416443	-0.4177179	0.14565016	-2.8679533	0.00413137	0.01658171
TRIM32	1657.59273	0.32540723	0.05659212	5.75004468	8.92E-09	1.31E-07
TRIM33	22900.899	-0.107568	0.03203449	-3.3578807	0.00078543	0.00400824
TRIM37	68202.6222	0.08994435	0.02892549	3.1095181	0.00187393	0.0084724
TRIM44	6221.95184	0.09578041	0.0393178	2.4360571	0.01484834	0.04772513
TRIM45	1227.08833	-0.2008669	0.06081206	-3.3030776	0.0009563	0.00474223
TRIM52	1711.00105	-0.404978	0.05493509	-7.3719356	1.68E-13	4.51E-12
TRIM65	1778.1281	0.13623417	0.05542715	2.45789609	0.01397536	0.04543986
TRIM66	1360.66378	-0.2791567	0.05910164	-4.7233325	2.32E-06	2.17E-05
TRIM73	1666.71846	-0.6250453	0.06147414	-10.167614	2.77E-24	1.69E-22
TRIM8	5462.72544	0.26556857	0.05109196	5.197855	2.02E-07	2.31E-06
TRIP12	13109.209	0.17676239	0.03983855	4.43696795	9.12E-06	7.53E-05
TRIP6	5411.19873	0.48582493	0.04616004	10.5247955	6.64E-26	4.60E-24
TRIQK	5142.39905	-0.1051834	0.03973178	-2.6473377	0.00811283	0.02931035
TRMT10B	427.601991	-0.2803153	0.10336625	-2.7118651	0.00669058	0.02493787
TRMT1L	1942.71799	0.22179504	0.05251274	4.22364269	2.40E-05	0.00017954
TRPC6	190.40814	0.51784475	0.14941663	3.46577722	0.0005287	0.00284861
TRPS1	4431.65407	0.17392635	0.04042404	4.30254742	1.69E-05	0.00013162
TRPV4	418.815587	0.73080764	0.1040908	7.02086668	2.20E-12	5.14E-11
TRRAP	12065.1619	0.29544758	0.05323668	5.54969919	2.86E-08	3.82E-07
TRUB2	4114.55569	0.21218627	0.04277143	4.96093471	7.02E-07	7.30E-06
TSC1	3072.90278	-0.1332825	0.04865461	-2.7393604	0.00615589	0.02321059
TSG101	3687.96237	0.21753297	0.04822879	4.51043763	6.47E-06	5.54E-05
TSKU	3066.95478	-0.2319205	0.05315934	-4.3627427	1.28E-05	0.00010232
TSPAN1	636.345096	-0.7280886	0.08264356	-8.8099861	1.25E-18	5.23E-17

TSPAN17	4054.01896	0.21788227	0.04360372	4.99687361	5.83E-07	6.18E-06
TSPAN33	541.315933	0.27783807	0.08963396	3.09969644	0.00193719	0.00870447
TSPAN9	880.804022	0.27069882	0.07216563	3.75107692	0.00017608	0.00108064
TSPO	536.956448	0.33190586	0.09320851	3.56089666	0.00036959	0.002089
TSPY26P	244.287425	0.78166148	0.13551048	5.7682733	8.01E-09	1.19E-07
TSPYL5	4999.33461	0.18545668	0.03889801	4.76776721	1.86E-06	1.78E-05
TSSK4	179.536418	-0.8257202	0.15550344	-5.3099801	1.10E-07	1.32E-06
TST	4569.82451	0.24563559	0.04803333	5.11385736	3.16E-07	3.52E-06
TSTA3	3567.44508	-0.1198382	0.04597667	-2.6064992	0.0091473	0.03231149
TTC14	2922.28626	-0.2048291	0.04785312	-4.2803707	1.87E-05	0.00014349
TTC32	306.664577	-0.4483198	0.11448971	-3.9158086	9.01E-05	0.00059311
TTC33	421.260354	0.28738324	0.09857657	2.91533002	0.00355313	0.01455249
TTC9	927.115071	-0.7020135	0.07167705	-9.794118	1.19E-22	6.57E-21
TTI1	2481.00955	0.17845848	0.056468	3.16034679	0.00157582	0.00730758
TTK	2239.32378	0.20684134	0.05211876	3.96865443	7.23E-05	0.00048597
TTLL12	2683.3321	0.15482933	0.04698791	3.29508834	0.00098391	0.00485274
TTYH3	4143.80323	0.44400676	0.05217366	8.51017019	1.74E-17	6.75E-16
TUBA1A	5560.43638	0.43084836	0.03911324	11.0154108	3.22E-28	2.49E-26
TUBA1B	13662.1346	0.41198153	0.04268854	9.65086978	4.87E-22	2.59E-20
TUBB	58410.2668	0.18422335	0.03270123	5.63352892	1.77E-08	2.45E-07
TUBB8	155.415924	-0.9111077	0.17058748	-5.3409997	9.24E-08	1.13E-06
TUBD1	8009.95999	0.35571524	0.03622577	9.81939642	9.29E-23	5.18E-21
TUBG2	438.706235	-0.3482065	0.09851239	-3.5346463	0.00040832	0.00227061
TUBGCP6	1338.60085	-0.2039081	0.06689837	-3.0480278	0.00230349	0.01007948
TUFMP1	16.2465442	-4.1929464	0.77493302	-5.410721	6.28E-08	7.84E-07
TXLNB	164.235654	1.03527842	0.1560096	6.6359917	3.22E-11	6.66E-10
TXN2	2187.79702	0.13070346	0.05165793	2.5301724	0.01140065	0.03869684
TXNDC11	2296.7718	-0.1459552	0.05868485	-2.487101	0.01287889	0.04255774
TXNDC16	1031.52818	0.27319705	0.06745271	4.05020106	5.12E-05	0.00035547
TXNL4B	1161.68875	0.22133913	0.0651833	3.39564157	0.00068468	0.00354672
TYRO3	700.247737	0.30267098	0.0795917	3.8027957	0.00014307	0.00090003
TYRP1	231.074748	0.62189103	0.13088968	4.75126095	2.02E-06	1.92E-05
TYW5	1091.7483	-0.2106214	0.06422259	-3.2795534	0.00103972	0.00507581
U2AF1	1780.14833	-0.4858616	0.0548173	-8.8632903	7.77E-19	3.30E-17
U2AF2	13978.8049	0.16290143	0.03366149	4.83940026	1.30E-06	1.28E-05
U73166.2	81.1047279	-0.7403277	0.22299515	-3.3199275	0.00090041	0.00449563
UBA1	24067.0204	0.10752862	0.03614029	2.97531177	0.00292691	0.01236258
UBA3	1246.99727	-0.2567668	0.06201342	-4.1405035	3.47E-05	0.00025117
UBAC1	2889.06786	0.11216242	0.04576991	2.45057121	0.01426298	0.04612331
UBAC2	2212.05285	0.17674033	0.05131693	3.44409421	0.00057298	0.00305219
UBAP1	2246.50298	0.1546791	0.05530211	2.79698381	0.00515821	0.02004579
UBAP1L	142.900145	-0.7584333	0.16991602	-4.4635772	8.06E-06	6.74E-05
UBAP2L	11394.9617	0.30092967	0.03372459	8.92315313	4.53E-19	1.98E-17

UBE2C	3654.70789	0.22535301	0.04444499	5.07038095	3.97E-07	4.36E-06
UBE2D1	1212.33843	0.18110612	0.06524574	2.77575381	0.00550739	0.02119944
UBE2G2	8238.64425	-0.2804515	0.03457178	-8.1121504	4.97E-16	1.74E-14
UBE2I	7047.93221	-0.1293648	0.0388818	-3.327129	0.00087746	0.00439911
UBE2Q1	5355.44181	0.09968558	0.03865594	2.57879084	0.00991468	0.03459986
UBE2QL1	167.234204	-0.9879252	0.15899547	-6.2135433	5.18E-10	9.01E-09
UBE2S	3980.90044	0.41635619	0.04605709	9.04000252	1.57E-19	7.04E-18
UBE2T	1873.20292	-2.2146889	0.06337759	-34.944352	1.58E-267	1.44E-263
UBE2Z	14614.4583	0.22207044	0.03337234	6.65432629	2.85E-11	5.90E-10
UBE3C	7644.90302	0.13938069	0.03672	3.79577074	0.00014719	0.00092163
UBE4A	5511.83036	0.15858629	0.05092131	3.11434047	0.00184357	0.00835793
UBE4B	5549.27796	0.23923081	0.04256265	5.62067416	1.90E-08	2.62E-07
UBL4A	4108.77796	0.13479304	0.04565735	2.95227438	0.00315443	0.01317704
UBL7	2374.86061	0.35537552	0.05153277	6.89610769	5.34E-12	1.19E-10
UBQLN4	4561.10092	0.24031272	0.04194347	5.72944242	1.01E-08	1.46E-07
UBTD2	2594.09557	0.2702561	0.04891532	5.52497864	3.30E-08	4.32E-07
UBXN4	12144.9263	-0.1565968	0.0339273	-4.6156583	3.92E-06	3.51E-05
UCHL5	2726.68896	-0.2254363	0.04678145	-4.8189259	1.44E-06	1.41E-05
UCK2	2391.83217	0.11594789	0.04769659	2.4309471	0.01505941	0.04821657
UCN	81.2075984	-0.5560629	0.22989145	-2.4188064	0.01557152	0.04960363
UFC1	3816.35602	0.25474546	0.0461567	5.51914373	3.41E-08	4.45E-07
UFM1	3504.81048	-0.1226129	0.04672109	-2.6243595	0.00868121	0.03093476
UFSP1	157.038133	0.66384875	0.17670339	3.75685355	0.00017206	0.00105737
UGDH	24084.9215	0.15748734	0.03423585	4.60007146	4.22E-06	3.76E-05
UGT2B15__[illegible]	845.205402	1.26355116	0.07443522	16.9751784	1.25E-64	4.40E-62
UIMC1	1983.012	0.15641528	0.05248906	2.97995986	0.00288286	0.01221899
UNC5B	568.865368	0.3654979	0.08992107	4.06465241	4.81E-05	0.00033645
UNKL	1891.47689	-0.3011573	0.05944593	-5.0660711	4.06E-07	4.45E-06
UPF1	9007.18546	0.12333123	0.03845757	3.20694337	0.00134153	0.00636025
UPP1	113.621472	-1.0718897	0.18990168	-5.6444456	1.66E-08	2.31E-07
UQCC1	1229.5307	-0.1732438	0.06634845	-2.6111209	0.0090246	0.03196475
UQCRHL	53.3457919	-0.9346959	0.2764791	-3.3807108	0.00072299	0.00372399
URAHP	973.764626	-0.3468448	0.0728258	-4.7626644	1.91E-06	1.82E-05
URB1	2988.99024	0.25823525	0.05071425	5.09196676	3.54E-07	3.93E-06
URI1	4635.22637	0.16605922	0.04069372	4.08070914	4.49E-05	0.00031585
USB1	1827.03504	0.29553956	0.056085	5.26949391	1.37E-07	1.63E-06
USF2	5925.71161	0.22013212	0.03753545	5.86464558	4.50E-09	6.94E-08
USO1	4273.56755	0.12216614	0.04034878	3.02775288	0.0024638	0.01067859
USP10	10313.2355	0.10983228	0.03652968	3.00665854	0.00264136	0.01132971
USP11	5303.3572	0.24217777	0.04353327	5.56304992	2.65E-08	3.57E-07
USP19	3421.04217	0.24144963	0.05003533	4.82558318	1.40E-06	1.36E-05
USP21	1102.65651	0.203709	0.0682929	2.98287208	0.00285557	0.01212023
USP22	13924.1464	0.28394111	0.03269977	8.68327432	3.85E-18	1.55E-16

USP32	29999.0571	0.25197907	0.03824894	6.58786987	4.46E-11	9.04E-10
USP54	1722.6056	0.15369772	0.05842728	2.63058134	0.0085239	0.03052332
USP8	9561.52412	0.08903361	0.03489586	2.55140904	0.01072883	0.0368834
UTS2	195.860535	-0.5691056	0.14254352	-3.992504	6.54E-05	0.00044317
VAPB	11947.6894	-0.1487188	0.03191051	-4.6604962	3.15E-06	2.87E-05
VASN	873.02379	-0.2035202	0.08141091	-2.4999134	0.01242237	0.04138204
VAT1	7072.93389	0.20130967	0.0440151	4.57365066	4.79E-06	4.23E-05
VCAN	316.498367	3.14791729	0.14400086	21.8604056	6.19E-106	5.37E-103
VCAN-AS1	14.0282255	2.65271106	0.63675958	4.16595389	3.10E-05	0.00022714
VCPKMT	908.470526	-0.6046999	0.07198789	-8.4000231	4.46E-17	1.67E-15
VEGFA	3729.40944	-0.4029869	0.04225095	-9.5379381	1.46E-21	7.46E-20
VEZF1	7587.20049	0.12195266	0.03533771	3.45106336	0.00055838	0.00298317
VGLL4	5081.31663	0.33085754	0.04161138	7.95113128	1.85E-15	6.25E-14
VHL	6586.85399	0.18664039	0.03972906	4.69783078	2.63E-06	2.44E-05
VIL1	21.9537517	-1.9022196	0.48160694	-3.9497347	7.82E-05	0.00052084
VIM	186.314716	-1.0340538	0.15968916	-6.4754163	9.46E-11	1.83E-09
VIM-AS1	291.64582	-1.8790739	0.12906819	-14.558769	5.14E-48	1.03E-45
VKORC1L1	3924.36609	0.24800403	0.04176211	5.9384943	2.88E-09	4.56E-08
VMP1	72157.0719	-0.378181	0.03675044	-10.290517	7.78E-25	4.94E-23
VOPP1	3650.09845	0.14297656	0.04569897	3.12866061	0.00175605	0.00801497
VPS18	1836.35967	0.35993594	0.05428084	6.63099469	3.33E-11	6.86E-10
VPS25	2903.35442	0.13991085	0.04602897	3.03962595	0.00236872	0.0103131
VPS41	3275.53996	0.10722486	0.04372588	2.45220573	0.01419835	0.04596815
VPS45	4520.44528	-0.1114621	0.04122878	-2.7035008	0.00686133	0.02549092
VPS4B	2136.68823	0.15191623	0.04977068	3.05232382	0.00227077	0.00996276
VPS9D1	1006.77653	-0.2873749	0.07696545	-3.733817	0.0001886	0.00114822
VSIG10	2656.04764	0.16577565	0.04900982	3.38249849	0.0007183	0.00370088
VTCN1	92.3912305	-1.6285842	0.22897584	-7.1124721	1.14E-12	2.76E-11
VWA7	279.976879	-0.6792532	0.1259459	-5.3932143	6.92E-08	8.59E-07
VWA9	3774.71598	0.26900678	0.04399017	6.11515605	9.65E-10	1.62E-08
VWDE	185.242328	0.85167904	0.14687102	5.79882305	6.68E-09	1.00E-07
WASF1	1001.46521	0.5226985	0.06886072	7.59066241	3.18E-14	9.35E-13
WBP11	7646.40993	0.10397161	0.03909836	2.65923199	0.0078319	0.02852335
WBP1L	3769.80281	0.19285486	0.04162368	4.63329631	3.60E-06	3.25E-05
WBP2	5718.82928	0.26171241	0.03961134	6.60700795	3.92E-11	8.01E-10
WBSCR16	3733.94448	0.25022045	0.05084611	4.92113219	8.60E-07	8.80E-06
WDR1	13468.1967	0.16641304	0.03550993	4.68638038	2.78E-06	2.57E-05
WDR13	2055.99931	-0.1404109	0.05121298	-2.7417047	0.00611213	0.02307901
WDR27	1035.9812	-0.417741	0.06879345	-6.0723948	1.26E-09	2.08E-08
WDR31	333.964741	-0.2899547	0.11038969	-2.6266467	0.00862308	0.03077574
WDR54	1846.56173	-0.3032448	0.05579706	-5.4347809	5.49E-08	6.95E-07
WDR60	1333.12746	-0.2970375	0.05988848	-4.9598435	7.06E-07	7.33E-06
WDR72	56.1602387	1.00197557	0.28230122	3.54931366	0.00038624	0.00216697

WDR76	1008.23587	-0.178937	0.06971345	-2.5667494	0.01026568	0.03555933
WDR82	4455.87474	0.17112307	0.04348413	3.93529975	8.31E-05	0.00055055
WDR97	361.923169	-0.5335244	0.10795552	-4.942076	7.73E-07	7.98E-06
WDTC1	3016.90361	0.27101713	0.0524794	5.1642574	2.41E-07	2.73E-06
WIPF1	1164.35305	-0.6490705	0.0642646	-10.099969	5.53E-24	3.31E-22
WISP2	1293.25927	-1.132114	0.06695892	-16.907591	3.96E-64	1.36E-61
WIZ	4570.00988	0.33412572	0.05611614	5.9541821	2.61E-09	4.16E-08
WNT2B	247.363859	-0.7365599	0.13417914	-5.4893768	4.03E-08	5.22E-07
WSB1	8736.03068	-0.8236959	0.03494886	-23.56861	8.09E-123	9.22E-120
WWC1	5270.39134	0.17729351	0.04193096	4.22822416	2.36E-05	0.0001766
WWP2	4362.96712	0.16936438	0.04654034	3.63908774	0.00027361	0.00160364
XIST__XIST_i	14157.5201	-1.2416955	0.0498542	-24.90654	6.32E-137	9.60E-134
XKRX	80.1088107	-0.6442723	0.23008214	-2.800184	0.00510735	0.01988934
XPA	553.174561	-0.276469	0.0915435	-3.0200837	0.00252705	0.01092136
XPC	4618.85705	0.28022958	0.04364024	6.4213569	1.35E-10	2.55E-09
XPO7	4373.21375	0.10366489	0.04093614	2.53235628	0.01132988	0.03848529
XPOT	16204.106	0.17223876	0.03158515	5.45315704	4.95E-08	6.31E-07
XRCC5	25258.0171	0.07863141	0.03228851	2.43527505	0.01488047	0.04779471
XRCC6	14428.8246	0.09131637	0.0329908	2.7679341	0.00564129	0.02164175
XRN1	1957.36531	-0.1655247	0.06118369	-2.7053731	0.00682277	0.02536748
XRN2	8479.98749	0.16878817	0.03709941	4.54961888	5.37E-06	4.70E-05
XXYLT1	1657.22396	0.17151492	0.05629644	3.04663878	0.00231416	0.01011417
XXbac-BPG1{	931.671563	0.28093285	0.08297233	3.38586179	0.00070955	0.00366203
XXbac-BPG2!	15554.7811	0.2825197	0.03938462	7.17335061	7.32E-13	1.81E-11
XYLT1	811.864159	0.54660907	0.07695375	7.10308582	1.22E-12	2.94E-11
XYLT2	3806.96023	0.24420522	0.04868222	5.01631278	5.27E-07	5.63E-06
YAP1	2953.31425	0.17773909	0.04591433	3.87110254	0.00010834	0.00069881
YARS	5363.39603	0.44235319	0.03962121	11.164555	6.08E-29	4.86E-27
YARS2	1091.0636	0.21143299	0.06793307	3.11237228	0.0018559	0.00840133
YBX1	32195.5879	0.1404064	0.0317437	4.42312708	9.73E-06	7.97E-05
YBX3	9482.62907	0.1225154	0.03894733	3.14566862	0.00165708	0.00762238
YDJC	2408.36958	0.26440099	0.06958661	3.79959576	0.00014493	0.00091002
YLPM1	8247.46228	0.15846332	0.04790179	3.30808791	0.00093935	0.00466835
YPEL2	2012.91213	0.383168	0.05668213	6.75994383	1.38E-11	2.96E-10
YPEL3	2045.3702	-0.2772638	0.05059019	-5.4805843	4.24E-08	5.46E-07
YPEL5	3177.29877	-0.2140756	0.04794742	-4.4647999	8.01E-06	6.71E-05
ZBED3	384.085287	0.38313609	0.10215347	3.75059316	0.00017642	0.00108236
ZBED4	1443.29285	0.30882589	0.06668991	4.63077403	3.64E-06	3.28E-05
ZBTB11-AS1	340.594139	0.31148205	0.11284077	2.760368	0.00577363	0.02204279
ZBTB12	824.894058	0.6148484	0.07711807	7.97281883	1.55E-15	5.27E-14
ZBTB21	1689.54952	0.15587474	0.05721983	2.72413859	0.00644695	0.02416312
ZBTB25	2053.52	-0.3173693	0.05605363	-5.6618867	1.50E-08	2.10E-07
ZBTB33	3930.06498	0.18539193	0.0411679	4.50331251	6.69E-06	5.70E-05

ZBTB37	1214.81574	-0.371431	0.06875692	-5.4020884	6.59E-08	8.22E-07
ZBTB38	2116.92654	0.17422831	0.05074862	3.43316378	0.00059658	0.00315581
ZBTB40	2537.47146	0.14522522	0.05242533	2.77013459	0.00560331	0.02151366
ZBTB41	2442.0955	0.15506058	0.05204285	2.97947899	0.00288739	0.01223534
ZBTB43	1088.52192	-0.1710021	0.06612405	-2.5860809	0.00970742	0.03395454
ZBTB45	1488.73577	0.39733863	0.07040208	5.64384789	1.66E-08	2.32E-07
ZBTB5	1648.88944	0.23609988	0.0556217	4.24474441	2.19E-05	0.00016571
ZBTB7A	6277.91418	0.10740998	0.04422923	2.42848392	0.0151621	0.04851126
ZC3H12A	1641.53488	-0.3788088	0.05598825	-6.7658627	1.33E-11	2.85E-10
ZC3H18	6075.23053	0.21668665	0.0391638	5.53282951	3.15E-08	4.16E-07
ZC3H4	3800.68088	0.2380601	0.04276765	5.56635923	2.60E-08	3.51E-07
ZC3H7A	4230.75526	0.11708735	0.04191652	2.79334618	0.00521658	0.02025536
ZC3H7B	4749.42309	0.15420259	0.04402692	3.50246153	0.00046098	0.00253331
ZCCHC14	3704.98766	0.18174029	0.04676986	3.88584211	0.00010198	0.00066148
ZDHHC17	1921.15113	-0.2611875	0.05391657	-4.84429	1.27E-06	1.26E-05
ZDHHC18	2001.27592	0.15205756	0.05409555	2.81090702	0.00494021	0.01933041
ZDHHC22	852.182621	0.7356272	0.07318601	10.0514723	9.05E-24	5.38E-22
ZDHHC3	3768.09912	0.11266707	0.0423897	2.65788776	0.00786321	0.02858978
ZDHHC7	6828.82811	0.09776835	0.03611252	2.70732598	0.00678276	0.02524018
ZDHHC8P1	115.699027	0.59618072	0.1901493	3.13532954	0.00171661	0.00786377
ZDHHC9	5217.19044	0.27924138	0.04062164	6.87420293	6.23E-12	1.38E-10
ZFAND5	5577.91137	-0.1411935	0.04297135	-3.2857601	0.00101708	0.00499068
ZFHX2	392.17615	0.59911302	0.11181556	5.35804685	8.41E-08	1.03E-06
ZFP64	1520.66588	0.22899769	0.06000239	3.81647614	0.00013537	0.00085558
ZFP92	34.5582411	1.11138393	0.33685373	3.29930721	0.00096924	0.00479465
ZFPM1	1465.17426	0.25994337	0.06115985	4.25022937	2.14E-05	0.00016211
ZFR	6316.36891	0.21688257	0.04089879	5.30290959	1.14E-07	1.37E-06
ZFYVE1	1892.05569	0.42917091	0.05432519	7.90003593	2.79E-15	9.24E-14
ZFYVE19	1218.6364	-0.1618877	0.06195482	-2.6129962	0.00897523	0.03183318
ZFYVE9	986.63038	0.3728599	0.07144525	5.21882014	1.80E-07	2.08E-06
ZGRF1	979.341082	-0.3050572	0.07123318	-4.2825151	1.85E-05	0.00014223
ZHX2	3581.89692	0.16864787	0.04471562	3.77156502	0.00016223	0.0010034
ZIC2	261.972516	-0.6365796	0.12571444	-5.0636955	4.11E-07	4.50E-06
ZIC5	113.587124	-0.8140441	0.18844735	-4.3197432	1.56E-05	0.0001224
ZKSCAN5	1351.48932	0.3481423	0.05887842	5.9129016	3.36E-09	5.27E-08
ZMAT1	1066.91374	-0.3126021	0.06587803	-4.7451649	2.08E-06	1.97E-05
ZMIZ1-AS1	29.4997764	-1.3741221	0.38625057	-3.5575925	0.00037427	0.00211152
ZMYM3	4372.49897	0.36443621	0.04322187	8.43175443	3.41E-17	1.29E-15
ZMYM4	3742.2586	0.19355956	0.04810255	4.02389382	5.72E-05	0.00039325
ZMYM5	1376.34537	-0.4495595	0.06143825	-7.3172577	2.53E-13	6.62E-12
ZMYND8	12714.141	0.21738526	0.0341341	6.3685653	1.91E-10	3.55E-09
ZNF106	4559.37411	0.19538708	0.04824033	4.05028527	5.12E-05	0.00035547
ZNF135	31.8584085	-1.3670588	0.36561221	-3.739095	0.00018468	0.00112702

ZNF146	8214.39047	0.12286046	0.03664343	3.35286506	0.0007998	0.00406792
ZNF160	2640.89437	-0.3328199	0.04651291	-7.1554294	8.34E-13	2.04E-11
ZNF185	3134.49158	-0.3662327	0.04656001	-7.8658218	3.67E-15	1.20E-13
ZNF204P	159.458408	-0.7750125	0.16541544	-4.6852488	2.80E-06	2.58E-05
ZNF213-AS1	544.603331	-0.2742395	0.10334716	-2.6535751	0.0079644	0.02887136
ZNF217	40098.9389	0.08797293	0.03249073	2.70763142	0.00677652	0.02523195
ZNF219	3608.00608	0.44340647	0.06493339	6.82863608	8.57E-12	1.87E-10
ZNF224	673.856839	-0.2905812	0.08302818	-3.4997906	0.00046562	0.00255498
ZNF24	6212.0857	0.10782631	0.03721309	2.89753752	0.00376105	0.01530438
ZNF251	1193.0911	-0.2458573	0.06414164	-3.8330376	0.00012657	0.00080386
ZNF253	1217.74582	0.23485284	0.06155169	3.81553839	0.00013589	0.00085824
ZNF254	2193.13943	0.20847754	0.05021571	4.15163992	3.30E-05	0.00024057
ZNF256	220.509467	-0.528428	0.13571699	-3.8936027	9.88E-05	0.00064284
ZNF257	575.62462	0.23720167	0.09130076	2.59802511	0.00937616	0.03298572
ZNF26	806.69359	-0.3953684	0.07515868	-5.260449	1.44E-07	1.70E-06
ZNF273	684.220935	0.2514	0.08149811	3.08473417	0.00203734	0.0090762
ZNF275	1567.80019	0.32126182	0.05592137	5.74488485	9.20E-09	1.35E-07
ZNF282	2511.76139	0.25060412	0.04793082	5.22845523	1.71E-07	1.98E-06
ZNF292	3165.3872	-0.3493771	0.04641222	-7.5276972	5.16E-14	1.47E-12
ZNF3	3174.02387	0.16874235	0.04386997	3.84642044	0.00011986	0.00076495
ZNF32	1314.1166	0.31347482	0.06800282	4.60973282	4.03E-06	3.61E-05
ZNF322	1461.60119	0.18806773	0.05797299	3.24405776	0.0011784	0.00566788
ZNF329	641.794555	-0.2631651	0.08572501	-3.0698759	0.00214148	0.0094707
ZNF333	401.241098	-0.3845873	0.10309919	-3.7302648	0.00019128	0.0011622
ZNF337	2171.13434	-0.3389265	0.05244181	-6.4629062	1.03E-10	1.98E-09
ZNF346	1337.27767	0.19259347	0.05929813	3.24788426	0.00116267	0.00560551
ZNF354B	881.760833	-0.3929052	0.071745	-5.4764123	4.34E-08	5.57E-07
ZNF365	96.8213088	-0.5623253	0.21199249	-2.6525719	0.00798811	0.02892854
ZNF367	2136.98336	-0.3768489	0.05184476	-7.2687943	3.63E-13	9.35E-12
ZNF37BP	1188.29747	-0.5140727	0.065892	-7.8017464	6.11E-15	1.93E-13
ZNF398	1400.57831	0.14633284	0.05787186	2.52856625	0.01145295	0.03881575
ZNF404	176.823559	-0.4566274	0.15152479	-3.013549	0.00258211	0.01111217
ZNF407	654.190266	0.20113389	0.08046891	2.49952297	0.01243606	0.0414172
ZNF418	295.928659	-0.654869	0.1226024	-5.3414041	9.22E-08	1.12E-06
ZNF432__ZN	1489.91386	-0.2231356	0.05906851	-3.7775723	0.00015837	0.00098285
ZNF436-AS1	63.5907831	-0.6365356	0.25313472	-2.514612	0.01191634	0.04006833
ZNF44	559.457588	-0.5439364	0.09495249	-5.7285111	1.01E-08	1.47E-07
ZNF473	1584.52874	0.31700538	0.05699185	5.5622929	2.66E-08	3.58E-07
ZNF486	755.027014	0.52650672	0.07704966	6.83334287	8.30E-12	1.82E-10
ZNF492	188.558136	-0.4083237	0.14910335	-2.7385281	0.00617149	0.02326461
ZNF507	2276.40468	0.14522791	0.0590491	2.45944323	0.01391527	0.04530103
ZNF516	2659.36533	0.2546631	0.04597551	5.53910322	3.04E-08	4.03E-07
ZNF517	751.726046	-0.2144509	0.08001587	-2.6801039	0.00735993	0.02698592

ZNF546	231.979359	-0.4029778	0.13254966	-3.0402029	0.00236419	0.01029828
ZNF550	491.172045	-0.4110496	0.09333918	-4.4038274	1.06E-05	8.64E-05
ZNF558	758.437608	-0.3967509	0.07911371	-5.0149455	5.30E-07	5.66E-06
ZNF561	1397.64498	0.20906779	0.05986494	3.49232467	0.00047884	0.00261723
ZNF592	5098.34841	0.13673767	0.04776985	2.86242619	0.00420411	0.01682567
ZNF609	5755.60225	0.28757108	0.04675329	6.15081965	7.71E-10	1.31E-08
ZNF610	278.883823	0.30936811	0.11770956	2.62823271	0.00858298	0.03067464
ZNF616__ZN	1323.42959	0.15225114	0.06080853	2.50377949	0.01228746	0.0410648
ZNF618	1718.77626	0.17041497	0.05862215	2.90700677	0.00364905	0.01490854
ZNF627	1646.41014	0.13622816	0.05625288	2.4217098	0.01544768	0.04928662
ZNF629	4484.34886	0.117277	0.04345713	2.69868258	0.00696145	0.02579983
ZNF638	10198.3694	0.11791016	0.0388122	3.03796667	0.0023818	0.01036016
ZNF641	1554.38654	0.19142746	0.05840593	3.2775347	0.00104718	0.00510301
ZNF644	1551.99188	0.28706271	0.0560793	5.11887128	3.07E-07	3.44E-06
ZNF654	1423.43356	-0.1575528	0.058904	-2.6747382	0.00747876	0.0273666
ZNF669	416.861673	0.28835078	0.10437739	2.76257887	0.00573467	0.02192619
ZNF682	411.764927	-0.5734535	0.10051053	-5.7054074	1.16E-08	1.67E-07
ZNF687	7192.32173	0.28845068	0.04153189	6.94528191	3.78E-12	8.56E-11
ZNF689	1268.4555	0.34055582	0.06444245	5.28465007	1.26E-07	1.50E-06
ZNF692	1168.0991	-0.316389	0.06343885	-4.9873065	6.12E-07	6.47E-06
ZNF7	1506.08659	-0.2698903	0.05730958	-4.7093402	2.49E-06	2.31E-05
ZNF74	1082.481	0.26122911	0.0683614	3.82129571	0.00013275	0.0008402
ZNF740	2370.19301	0.26390683	0.05759317	4.58225918	4.60E-06	4.08E-05
ZNF767P	476.193665	-0.5518689	0.09272039	-5.9519689	2.65E-09	4.22E-08
ZNF783	1517.34528	-0.203959	0.0581187	-3.5093522	0.0004492	0.00247903
ZNF789	1209.94153	-0.3407541	0.0615629	-5.5350558	3.11E-08	4.12E-07
ZNF827	41.304858	-0.9073331	0.32628874	-2.7807675	0.00542306	0.02095888
ZNF83	2826.84942	-0.4750833	0.04588813	-10.353076	4.05E-25	2.62E-23
ZNF839	772.013797	-0.2935802	0.07505118	-3.9117334	9.16E-05	0.00060234
ZNF84	2737.71911	-0.1411073	0.0467691	-3.0171061	0.00255201	0.0110164
ZNF845	1099.79514	0.17099431	0.06794233	2.51675664	0.01184406	0.0398831
ZNF850	421.069369	-0.3060293	0.09987877	-3.064008	0.00218393	0.00963275
ZNF91	1678.80174	0.16881861	0.05711263	2.95588936	0.00311769	0.0130445
ZNF93	730.479561	0.20969556	0.0783458	2.67653879	0.0074387	0.02724732
ZNRD1-AS1	368.982154	-0.5403513	0.10539732	-5.1268032	2.95E-07	3.31E-06
ZNRF1	6304.8206	-0.1522324	0.04923193	-3.092147	0.00198714	0.00889386
ZNRF3	979.601179	0.40512298	0.0694424	5.83394245	5.41E-09	8.25E-08
ZRANB2	3938.44354	-0.2428787	0.04184624	-5.8040733	6.47E-09	9.74E-08
ZRSR2	742.711625	-0.2164615	0.07702935	-2.8101173	0.00495235	0.01937375
ZSCAN18	456.69272	-0.601854	0.09614836	-6.2596381	3.86E-10	6.84E-09
ZSCAN2	499.645467	0.31139063	0.09113312	3.41687659	0.00063344	0.00332378
ZSCAN30	411.012545	-0.3846671	0.10220802	-3.7635702	0.00016751	0.0010315
ZSWIM5	1207.35317	0.2283234	0.0724916	3.14965305	0.00163465	0.0075287

ZSWIM6	2180.9668	0.33090836	0.05312727	6.22859695	4.71E-10	8.24E-09
ZZZ3	2894.16781	0.15708329	0.04873937	3.22292392	0.00126889	0.00606633

Control.0	Control.1	Control.2	shRNA1.0	shRNA1.1	shRNA1.2	Gene
13204.1137	13342.1572	13552.3805	11538.6909	11573.967	11735.4768	44626
747.365008	789.312467	815.288326	1191.65045	1174.07655	1243.32508	44628
326.286192	363.321718	349.239005	555.737444	573.601725	593.567685	44630
18743.9942	18355.6796	19495.4033	21301.4382	20570.8961	22136.7033	44813
450.015512	455.342066	480.352625	609.553916	651.441054	632.389668	44814
22.9497933	28.558039	28.6066079	4.39318137	4.63329342	3.06489338	A2M
5731.46143	5729.85322	5617.62263	6126.29143	6439.35119	5924.43891	AAGAB
201.559054	203.872667	176.407416	318.50565	297.457437	322.835436	AARD
14977.2347	15162.7322	15621.5918	21713.2989	21587.4407	22158.1575	AARS
7916.68087	7700.35791	7626.04489	6388.78401	6822.06123	6740.72218	ABAT
4461.24026	4373.34636	4354.16411	3285.00137	2909.70827	2759.42567	ABCA10__AE
7954.59792	8078.75192	8256.58221	11104.8642	12157.7619	12250.3788	ABCA12
14260.802	13900.6255	15622.7838	12094.4283	11467.4012	13156.5657	ABCA2
327.284009	310.965314	319.440455	195.496571	200.158276	234.975159	ABCC2
5084.87594	5042.08044	5194.48322	3236.67638	3257.20527	3455.15647	ABCC3
499.906367	504.525356	467.241263	623.831755	684.800767	659.973708	ABCC4
1840.97255	1920.52812	1913.0669	1639.75495	1730.99842	1674.45342	ABCD3
1837.9791	1879.27762	1839.1665	1672.70381	1725.43847	1676.49668	ABCD4
6581.60159	6634.19112	6474.62893	6874.23055	7083.37898	7166.74236	ABCF1
3617.08699	3821.22427	4004.92511	4235.02684	4200.54381	4347.04045	ABCF2
1610.4768	1665.88561	1632.96054	1020.31637	1015.61792	1066.5829	ABCG2
3949.36008	3927.52364	3975.12656	3534.31442	3392.49744	3292.71712	ABHD11
5019.02002	5057.94602	5257.65615	5865.99543	5798.10338	6195.17115	ABL1
3851.57401	3997.33218	3818.98216	4656.77226	4930.75085	4822.09892	ABLIM1
6473.83735	6627.05161	6680.83489	7401.41232	7343.77007	7705.14196	ABR
2084.43992	2047.45274	1832.01485	2358.0401	2481.59195	2306.84309	ABRACL
719.426129	746.475408	789.065602	596.374372	588.428264	599.697472	ABTB1
6761.20867	6696.86015	6982.39622	7647.43048	7320.6036	7504.90226	AC002310.11
1732.21049	1626.22167	1613.88946	1328.93737	1348.28838	1377.15876	AC003002.4_
78.8275509	94.4001845	90.5875917	52.7181765	52.8195449	56.189712	AC005077.14
298.347313	263.368582	219.317327	195.496571	190.891689	165.504243	AC005253.2
1412.90901	1421.55572	1475.62419	1697.9646	1640.18587	1695.90767	AC005523.2
1008.79309	1036.81547	1008.38293	1197.14192	1189.82975	1080.88573	AC005538.3_
33.9257814	35.6975488	39.3340859	17.5727255	11.1199042	14.3028358	AC005785.2
219.519762	227.671033	243.156167	179.022141	126.025581	156.309562	AC006128.2
7003.67823	6762.70229	7053.91274	8196.57815	8093.43694	8478.51672	AC006486.9_
5691.54874	5910.7208	5989.50853	7155.39416	7064.8458	7221.91044	AC007040.11
9492.23408	9705.76687	10095.7487	11846.2136	11295.9694	11663.9626	AC007192.4_
28.9366959	38.8706642	35.7582599	23.0642022	16.6798563	14.3028358	AC007325.2
1025.75598	1031.26252	1244.38744	2593.07531	2575.18448	3162.96997	AC007563.5
1284.19061	1189.12501	1231.27608	1460.73281	1455.78079	1425.17542	AC008074.1
26.9410617	46.8034528	47.6776799	17.5727255	19.4598324	26.5624093	AC009095.4

11068.7851	11289.9448	11530.8469	12787.4527	12473.7525	12498.6352	AC009133.12
3462.42534	3244.51054	3767.72865	4672.14839	4128.26443	4605.51312	AC009133.15
906.017927	986.838903	986.927973	758.922082	710.74721	705.947109	AC009133.21
3127.15879	3265.13579	3375.57973	4552.4342	4286.72307	4432.85746	AC009133.22
661.552738	644.935714	598.354882	730.366403	736.693653	732.509518	AC009237.8
980.85421	908.304296	928.522815	657.878911	727.427066	713.098527	AC010203.1_
4221.76415	4075.07351	4389.92237	3727.6144	3464.77682	3651.30965	AC011530.4_
596.694626	590.199473	544.717492	477.758474	486.495809	504.685777	AC011747.4
1499.7191	1360.47325	1470.85642	1912.13219	1812.54438	1764.35696	AC016629.7_
20.9541591	28.558039	27.4146659	6.58977206	9.26658683	13.2812047	AC024560.2
89.803539	80.9144438	77.4762298	46.1284044	54.6728623	51.0815564	AC034220.3
773.308253	754.408197	786.681718	706.203906	634.761198	678.363068	AC037459.4_
81.8210022	88.0539536	97.7392437	45.0301091	56.5261797	52.1031875	AC058791.1_
4402.36905	4553.42066	4563.9459	5534.31024	5482.11277	5559.71659	AC068533.7_
933.956806	982.872509	933.290583	702.90902	703.333941	705.947109	AC068831.15
16995.8187	16813.5455	16185.3804	17693.538	18332.0887	17660.9373	AC073610.5_
1698.28471	1594.49051	1803.40824	1434.37372	1317.70865	1388.3967	AC093838.4
204.552506	214.185293	187.134893	113.12442	120.465629	112.379424	AC114730.7
534.829966	499.765683	567.36439	418.450526	379.003401	469.950318	AC138969.4
1095.60318	1101.07106	948.785829	761.118673	779.319953	727.401362	AC159540.1
800.249315	753.614918	827.207746	562.327216	524.488815	616.04357	AC240274.1
2796.88133	2936.71834	2903.5707	2366.82647	2274.02041	2306.84309	ACAD11__NI
4344.49566	4466.15999	4169.4131	3780.33257	3607.48225	3533.82207	ACADM__RA
10112.8763	10322.1445	10608.2838	7481.58788	7115.81203	7377.19837	ACADVL
151.668199	146.756589	148.99275	93.3551042	94.5191857	66.4060233	ACAP1
2728.03195	2873.25604	3227.77893	2518.39122	2154.48144	2443.74166	ACAP3
1000.81055	953.521191	890.380671	1189.45386	1242.64929	1095.18857	ACAT1
267.414983	297.479573	288.449963	408.565868	382.710036	401.501033	ACBD7
604.677163	591.786031	607.890418	476.660179	459.622707	445.431171	ACCS
4925.22521	5052.39307	5167.06855	3372.865	3265.5452	3601.24972	ACKR3
10790.3941	10734.6496	10996.8569	12507.3874	12660.9376	13021.7103	ACLY
1710.25851	1724.58824	1690.17375	1929.70492	1888.5304	1964.59666	ACO1
2350.85709	2478.99644	2470.89576	2740.24688	2623.37073	2753.29589	ACOT7
1251.26264	1168.49976	1214.58889	1046.67546	1015.61792	1060.45311	ACOT8
2432.67809	2492.48218	2568.635	2229.53955	2150.7748	2197.52855	ACP6
2019.58181	2115.67472	2125.23258	2007.68389	1719.87852	1725.53497	ACSBG1
1006.79745	1052.68105	1067.98003	880.832865	836.772791	807.08859	ACSL1
68.8493799	71.3950975	58.4051578	23.0642022	36.1396887	18.3893603	ACSL6__CTB-
281.384422	299.85941	281.298311	418.450526	403.096527	468.928687	ACTA2
206.54814	248.296284	214.549559	14.2778395	20.386491	18.3893603	ACTG2
93.7948074	73.7749341	79.8601138	172.432369	216.838132	193.088283	ACTL8
28092.5426	27893.2713	28874.7949	30398.6185	29728.1372	31437.633	ACTN4
2986.46658	2998.5941	3004.88577	2754.52472	2665.07037	2528.53704	ACTR10

5294.41754	5449.0325	5519.88339	6074.67154	6036.25466	6271.79349	ACTR1A
1738.19739	1531.0282	1551.90848	1844.03788	1891.31037	1782.74632	ACVR2B
687.495982	694.119004	575.707984	751.234015	840.479426	845.910573	ADAM22
457.998049	426.784027	492.272045	389.894847	267.80436	310.575863	ADAM8
45.8995866	54.7362414	36.9502019	16.4744302	11.1199042	19.4109914	ADAMTS1
361.20979	310.172035	346.855121	235.035204	252.977821	263.580831	ADAMTS13
167.633273	169.761676	163.296054	1138.93227	1089.75061	1173.85417	ADAMTS9
3145.1195	3119.17248	3193.21261	3425.58318	3422.15052	3681.95858	ADCY3
41.9083182	39.6639431	28.6066079	10.9829534	12.9732216	11.2379424	ADCY4__RIPI
6527.71947	6544.55061	6705.86567	7041.17145	7250.17754	7323.05192	ADD1
73.8384654	62.66903	75.0923458	124.107374	89.8858923	116.465949	ADGRD1
887.059402	823.423458	852.238527	978.581151	961.871713	1030.82581	ADGRE5
572.747016	625.897022	607.890418	438.219842	429.04297	418.868762	ADGRF2__AI
8198.0653	8256.44639	8823.9466	9410.1945	9264.73352	10105.9751	ADGRG1
71.8428312	87.2606747	79.8601138	137.286918	125.098922	139.963464	ADGRG5
4152.91477	4110.77106	4022.80424	4579.89158	4409.04202	4614.7078	ADGRL1
4984.09642	4970.68534	5059.79378	5510.14774	5444.11976	5304.30881	ADH5
4776.55046	5046.04684	4788.031	5371.76253	5594.23847	5529.06766	ADI1
7546.49073	8032.74175	8214.86424	9576.0371	9373.15258	9589.02976	ADIPOR1
403.118109	361.735161	354.006773	253.706224	271.510994	221.693955	ADIRF__FAM
133.707491	151.516263	121.578084	82.3721508	70.4260599	85.8170147	ADM
10554.9093	10462.5549	10901.5015	13259.7197	12529.3521	13022.732	ADM5__PRM
16883.0653	17520.3569	17447.6469	20018.6292	20319.7716	20877.0321	ADNP
2136.32641	2249.73885	2234.89124	2633.71223	2478.81198	2433.52534	ADO
2961.52115	2987.48819	2909.53041	3236.67638	3385.08417	3625.76887	ADORA2A__
504.895453	484.693384	458.897669	678.746522	684.800767	658.952077	ADRBK2
5745.43086	5806.00799	6100.35914	6399.76697	6558.89016	6604.84524	AES
9205.86057	9550.28421	9346.01719	6478.84423	6365.2185	6729.48423	AF011889.5_
1977.67349	1995.88962	1961.93653	2339.36908	2607.61754	2760.44731	AFAP1
2806.8595	2813.76012	2902.37876	2513.99804	2572.40451	2368.14095	AFG3L1P__R
1040.72324	1156.60058	1264.65046	1546.39984	1465.04738	1639.71796	AGAP2-AS1
194.574335	180.074302	203.822081	151.564757	135.292168	137.920202	AGAP4
510.882355	492.626173	488.696219	381.108484	409.583138	394.349615	AGAP5
480.947842	510.871587	554.253028	358.044282	386.416671	417.847131	AGAP6
86.8100877	102.332973	107.27478	76.880674	62.0861318	63.3411299	AGAP7P
343.249083	402.985662	336.127643	246.018157	264.097725	253.36452	AGAP9
194.574335	235.603822	203.822081	102.141467	108.419066	108.2929	AGER
389.148669	385.533527	367.118135	674.353341	658.854324	662.01697	AGMAT
2449.64098	2563.084	2345.74185	2844.58494	3035.73385	3030.15792	AGO1
1346.05527	1356.50685	1338.55086	1021.41467	1063.80417	1067.60453	AGO4
2538.4467	2560.70416	2642.53541	2932.44857	2742.9097	2917.7785	AGPAT1
6392.01635	6113.00691	6400.72852	7582.63105	7752.42654	7827.73769	AGPAT3
849.142352	859.914286	898.724265	728.169813	794.146492	731.487887	AGR3

1173.43291	1109.79713	1271.80211	1556.2845	1389.06137	1555.94421	AGTRAP
8689.98913	9193.30872	9231.59076	11829.7391	11222.7633	11742.6282	AHCY
1286.18624	1208.95698	1383.84466	1386.04872	1542.88671	1604.9825	AHNAK2
6472.83953	6424.7655	6280.34238	5692.46477	5773.0836	5640.42545	AHRR__PDCI
12246.2093	12539.359	12071.9885	13214.6896	13314.232	12835.7735	AHSA1
3153.10204	3072.36903	3060.90705	1886.8714	1897.79698	1914.53673	AHSA2
5177.67293	5160.27899	4963.24647	6282.24936	6458.81102	6291.20448	AIF1L
4726.6596	4765.22612	4629.50271	5051.06028	5222.64834	4995.77621	AIFM1
3111.19372	3291.314	3158.64629	2191.09921	2312.94007	2403.89804	AIM1
4440.2861	4448.70785	4710.55477	4802.84554	4957.62396	4972.2787	AJUBA__HAU
3658.99531	3782.35361	3717.66709	3124.65025	3120.98645	2998.48736	AK4
5037.97854	4765.22612	4835.70868	4881.9228	5658.17792	5851.9031	AKAP13
3719.86215	3795.04607	3680.71689	3528.82294	3256.27861	3390.79371	AKNAD1__CL
2554.41178	2640.82533	2706.90027	3030.19685	2983.84096	3088.3909	AKR1A1
9963.20375	10118.2719	9779.88408	9116.94965	9449.13859	9074.12767	AKR1C2
2415.7152	2487.72251	2561.48335	2933.54686	2804.06918	2796.20439	AKR7A2
56.8755747	53.9429626	82.2439978	35.145451	21.3131497	36.7787206	AKR7A3
7555.47108	7329.89668	7646.30791	8928.04285	8282.47531	8579.6582	AKT2
805.2384	729.816552	767.610646	489.839723	632.907881	618.086832	AL449212.1_
2588.33756	2664.6237	2568.635	2083.46627	2130.38831	2113.7548	ALAD
87518.5379	85643.1791	84706.5499	99090.4025	98496.4048	94895.2289	ALDH16A1__
6637.47935	6677.02818	6786.91773	8157.03952	8061.00389	8313.01248	ALDH18A1
745.369374	721.090485	657.951982	475.561884	472.595928	452.582589	ALDH1A3
1554.59904	1534.9946	1488.73555	1885.77311	1856.09734	1961.53176	ALDH1B1
3661.98876	3993.36579	3886.92285	3422.28829	3605.62894	3593.07667	ALDH3A2
359.214156	361.735161	399.300569	265.787473	250.197845	253.36452	ALDH3B1
2384.78287	2428.22659	2460.16828	2877.5338	2695.65011	2973.96821	ALDH4A1__II
4087.05884	4167.88714	4395.88208	5134.53073	5243.03483	5266.50846	ALDH5A1
2772.93372	2801.86094	2754.57795	3388.24113	3170.09936	3033.22282	ALDH6A1
2778.92063	2835.97193	2678.29367	3125.74855	3183.07258	3111.88841	ALDH7A1
110530.196	112724.926	117968.883	75963.5974	76403.9351	81447.4983	ALDOA
898.03539	869.433632	899.916207	589.784599	629.201246	609.913783	ALDOC
2478.57768	2562.29072	2526.91703	2261.39011	2329.61993	2252.69664	ALG13
3378.6087	3479.32109	3481.66257	3971.43596	3664.00843	3826.00857	ALG8
933.956806	882.126094	954.745539	1088.41069	1108.28379	1063.518	ALKBH4
695.478519	623.517185	631.729258	412.959049	527.268791	505.707408	ALOX12P2
556.781942	566.401107	598.354882	336.078375	299.310755	285.035084	ALS2CL
5828.24968	5927.37965	6069.36865	7100.4794	7214.03785	7055.38456	ALYREF
2401.74576	2336.99953	2441.09721	2663.36621	2695.65011	2777.81503	AMBRA1
615.653151	593.372588	559.020796	634.814709	717.233821	707.990371	AMER1
240.473921	220.531523	245.540051	120.812488	132.512192	151.201407	AMER2
5617.71028	5472.83086	5890.57735	6999.43622	6650.62937	6997.15159	AMFR
745.369374	756.788034	871.309599	613.947097	443.869509	500.599252	AMH

11605.6107	12384.6696	12279.3865	8864.34172	8375.14118	8765.59507	AMIGO2
2209.16706	2074.42422	2218.20406	2523.8827	2346.29979	2448.84981	AMIGO3__G
1023.76035	1010.63727	1021.49429	800.657305	945.191857	847.953835	AMOT
4505.14421	4622.43592	4708.17089	3637.55418	3603.77562	3747.34297	AMOTL2
226.504482	195.1466	226.468979	65.8977206	68.5727426	60.2762365	AMT
18.9585249	32.5244333	14.303304	4.39318137	10.1932455	8.17304902	AMTN
608.668431	752.02836	642.456736	473.365293	461.476024	487.318048	AMY2B__RN
13333.8299	13841.1296	13343.7907	15504.6354	15816.2104	15623.8048	ANAPC7__AI
204.552506	168.968397	146.608866	114.222716	138.998803	108.2929	ANGPTL6
157.655102	180.074302	177.599358	53.8164718	75.0593533	45.9734007	ANG__RNAS
4139.94315	4233.72928	4059.75444	4344.85638	4758.39234	4697.45992	ANK3
22.9497933	32.5244333	33.3743759	53.8164718	58.379497	59.2546054	ANKRD1
1110.57043	1035.22891	1041.75731	855.572073	836.772791	855.105253	ANKRD10
11536.7613	10935.3491	11113.6672	12261.3692	12748.9702	13204.5823	ANKRD11
732.397752	708.398023	764.03482	1011.53001	828.432863	881.667663	ANKRD13B
861.116158	882.126094	917.795337	601.865848	632.907881	645.670872	ANKRD18A
751.356277	741.715735	698.47801	535.968128	493.909078	491.404572	ANKRD18B
2087.43337	2069.66455	2181.25385	1893.46117	1942.2766	1921.68815	ANKRD23__/
167.633273	183.247417	234.812573	126.303965	91.7392096	125.660629	ANKRD24
3527.28345	3704.61228	3635.42309	4359.13422	4554.52743	4633.09716	ANKRD27
410.102828	412.505008	388.573091	328.390308	313.210635	327.943592	ANKRD34B
306.32985	295.099736	334.935701	259.197701	213.131497	232.931897	ANKRD35
635.609493	605.271771	618.617896	382.20678	441.089533	384.133304	ANKRD36
310.321118	314.931708	282.490253	185.611913	175.138491	170.612398	ANKRD36B
55.8777576	36.4908276	33.3743759	18.6710208	16.6798563	8.17304902	ANKRD36BP
451.013329	402.985662	418.371641	278.967017	328.037174	368.808837	ANKRD36C
2746.99048	2778.06257	2684.25338	3282.80478	3259.98525	3243.67883	ANKRD40
10237.6035	10029.4246	10024.2322	10761.0978	12530.2787	12423.0345	ANKRD52
2468.59951	2559.11761	2704.51639	2865.45255	2937.50803	3137.42919	ANKS1A
11719.3618	12219.6676	11689.3752	10472.2461	10236.7985	9558.38082	ANKUB1__PI
3360.64799	3528.50438	3753.42535	2342.66397	2450.08556	2465.19591	ANKZF1
6344.12112	6688.13408	6270.80684	7380.54471	7615.28106	7880.86251	ANLN
1944.74553	2024.44765	2019.14974	2329.48442	2130.38831	2316.03777	ANO10
378.172681	399.812546	379.037555	228.445431	276.144288	281.970191	ANO7
1613.47025	1627.01494	1680.63822	1000.54706	975.771593	993.025455	ANO9
9584.03325	9329.75269	8965.7877	9940.67115	11019.8251	10031.396	ANP32A
381.166132	337.936795	381.421439	250.411338	280.777581	258.472675	ANXA1
5131.77335	5189.63031	5231.43342	5823.16191	5474.6995	5626.12262	ANXA11
1839.97473	1985.57699	1716.39648	1534.3186	1591.99962	1506.90591	ANXA3
1935.76518	1947.4996	2031.06916	1681.49017	1616.09274	1672.41016	ANXA6
2322.91821	2333.03313	2411.29866	2723.77245	2715.10994	2860.56716	ANXA9
1665.35674	1765.83875	1643.68801	2051.6157	1980.26961	2110.68991	AP000295.9_
10617.7718	10019.112	9274.50067	5641.94318	5709.14415	5459.59674	AP000769.1

44.9017695	35.6975488	30.9904919	8.78636275	12.9732216	18.3893603	AP000866.1
935.95244	951.141355	1017.91847	877.537979	770.980025	840.802417	AP001062.7
497.910733	425.19747	504.191465	762.216968	582.868312	675.298175	AP003391.1
7740.06725	7864.56663	8455.63652	9740.7814	8874.61021	10081.456	AP1B1
5817.2737	5815.52733	6157.57235	4300.92457	4157.91751	4473.72271	AP1G2__JPH
8757.84069	8767.31797	9098.09326	10401.9552	10264.5982	10460.4811	AP1M2
9360.52222	9210.76086	8784.61251	10833.5853	11301.5293	10798.641	AP1S1
2326.90948	2528.17973	2642.53541	2883.02528	2721.59655	2977.0331	AP2A2
9490.23844	9657.37686	9763.19689	11345.3909	11852.8912	12221.7732	AP2B1
23424.7543	24429.8158	25111.834	26713.8376	25056.8508	26207.9033	AP2M1
3940.37973	3951.32201	4063.33027	4386.5916	4367.34237	4488.02554	AP3B1
6767.19558	6999.09939	7268.46229	7614.48162	7310.41035	7725.57458	AP3D1
1079.6381	1061.40712	1135.92072	1405.81804	1280.6423	1356.72614	AP4M1
2011.59927	1948.29288	2128.80841	2534.86565	2351.85974	2613.33242	AP5B1
2311.94222	2343.34576	2324.28689	2077.97479	2088.68867	2082.08424	APBB3__SRA
5364.26473	5630.69336	5820.25277	6903.88453	6557.03684	6966.50265	APEH
2100.405	2206.10851	2268.26562	2462.37816	2274.02041	2463.15265	APEX2
13950.4809	14202.8647	14273.5054	15742.9655	15581.7658	15807.6984	APH1A
2762.95555	2813.76012	2840.39778	3379.45477	3368.40431	3263.08982	APOA1BP
478.952208	564.02127	548.293318	765.511854	714.453845	776.439656	APOBEC3A__
112.753332	111.059041	109.658664	62.6028346	75.986012	83.7737524	APOL1
861.116158	791.692304	865.349889	608.45562	653.294372	577.221587	APOL2
3402.55631	3568.9616	3481.66257	3772.64451	3714.97466	3794.33801	APOLD1__DD
16737.384	16986.4803	17118.671	17963.7186	17841.8863	18661.1142	APP
7230.18271	7235.49649	7298.26084	8303.1128	8060.07723	8194.50327	APRT
254.443361	283.200554	281.298311	366.830645	341.937054	320.792174	AQP1__FAM
2731.0254	2705.8742	2710.4761	1614.49416	1709.68527	1628.48002	AQP3
12571.4977	13050.2306	12769.2746	15001.6161	15142.5295	15357.1591	ARCN1
6204.42673	6319.25941	6456.7498	7293.77938	6859.12757	6881.70727	ARF5__FSCN
2758.96428	2843.90472	2866.6205	3230.08661	3208.09236	3367.29619	ARFGAP3__F
67.8515628	65.8421455	63.1729258	107.632944	106.565749	76.6223345	ARG2
3029.37272	2939.09818	2966.74363	2559.02815	2717.88992	2625.592	ARGLU1
4275.64628	4262.28732	4491.23744	4884.11939	4725.03263	4929.37019	ARHGAP1
1262.23863	1230.37551	1286.10541	1437.6686	1363.11492	1444.58641	ARHGAP10
4205.79908	4221.8301	4046.64308	4897.29894	5010.4435	4756.71453	ARHGAP11A
3937.38628	3924.35053	3714.09126	3577.14793	3384.15751	3524.62739	ARHGAP12
706.454507	719.503927	708.013546	870.948207	884.959043	910.273334	ARHGAP18
1404.92648	1489.7777	1401.72379	1732.01176	1824.59095	1749.03249	ARHGAP19_
709.447958	729.816552	746.15569	466.775521	491.129102	467.907056	ARHGAP29
119.738052	133.270849	122.770026	85.6670368	96.3725031	91.9468014	ARHGAP30
2268.03827	2149.78571	2149.07142	2147.1674	2633.56398	2691.99802	ARHGAP32
1022.76253	1028.88268	1135.92072	811.640259	798.779785	855.105253	ARHGAP33
602.681529	753.614918	730.660444	1137.83398	1078.63071	1136.05381	ARHGAP36

3787.71371	3804.56542	4186.10029	4992.85063	4253.36336	4794.51488	ARHGAP39_
7205.23728	7099.84581	7114.70178	5983.51303	5927.8356	5966.32578	ARHGAP4__I
16021.9492	16412.1464	17390.4337	17768.2221	17244.1914	18360.7546	ARHGDIA
90.8013561	102.332973	133.497504	38.440337	46.3329342	50.0599252	ARHGDIB
2488.55585	2436.15938	2662.79842	2131.79126	1952.46985	2190.37714	ARHGEF10L
1215.34123	1192.29813	1224.12443	1633.16518	1547.52	1800.11405	ARHGEF18_
1409.91556	1429.48851	1599.58616	1721.0288	1618.87272	1685.69136	ARHGEF19
1078.64029	1083.61892	1154.9918	1350.90327	1312.1487	1297.47153	ARHGEF2
956.906599	982.872509	864.157947	653.485729	769.126707	744.769092	ARHGEF26
323.292741	343.489747	330.167933	238.33009	275.217629	296.273027	ARHGEF40
7007.6695	7009.41202	7453.2133	7793.50376	8011.89098	8281.34192	ARID1A
4399.3756	4326.54291	4491.23744	4846.77735	5034.53663	5213.38364	ARID2
2035.54689	1970.50469	1880.88447	1602.41291	1661.49902	1628.48002	ARID5B
3776.73773	3934.66315	3730.77845	3516.74169	3562.07598	3555.27632	ARL1
3200.99726	3206.43316	3261.1533	3803.39677	3719.60795	3713.62915	ARL2__ARL2
2827.81366	2760.61044	2669.95007	3009.32924	3121.9131	2938.21112	ARL3
2599.31355	2720.15322	2704.51639	2943.43152	2934.72805	2961.70864	ARL8A
3976.30115	4078.24663	4064.52221	4663.36203	4316.37615	4256.11528	ARMC10
2056.50104	2101.3957	2084.70655	2640.30201	2428.77241	2541.81824	ARMC6
5556.84343	5717.16075	5524.65115	6252.59539	6343.90535	6148.17612	ARMCX3
4046.14834	4114.73745	4083.59328	4330.57854	4484.10137	4578.95071	ARNT
387.153035	396.639431	432.674945	516.198811	541.168671	514.902088	ARNTL
19044.3372	18942.7059	19695.6496	23355.2505	22421.4335	22816.088	ARPC1A__AF
939.943709	974.93972	1059.63644	822.623212	879.39909	884.732556	ARRB1
5968.94189	5873.43669	6353.05084	5471.7074	4626.80681	5129.60989	ARRDC1
5766.38502	5971.00999	5695.09886	4869.84155	4797.312	4596.31844	ARRDC3
3100.21773	3292.10727	3270.68884	2950.02129	2767.92949	2915.73524	ARRDC4
3576.17649	3637.97686	3609.20037	4248.20639	4445.1817	4551.36667	ASAP1
207.545957	174.52135	228.852863	136.188623	149.192048	149.158145	ASB9
954.910965	915.443806	946.401945	393.189733	352.1303	352.462739	ASCL1
4479.20096	4480.43901	4478.12608	5421.18582	4938.16412	5365.60668	ASF1B
128.718406	126.131339	132.305562	110.92783	75.0593533	85.8170147	ASIC3
82.8188193	49.1832894	60.7890418	29.6539743	29.6530779	35.7570894	ASIC4
138.696577	111.059041	101.31507	43.9318137	34.2863713	54.1464497	ASMTL-AS1
4156.90604	4290.05208	4226.62632	5227.88584	5141.10238	5042.77124	ASNA1
2103.39845	2198.969	2311.17553	4065.88936	3820.61375	3923.06353	ASNS
4860.3671	4908.80959	4783.26323	4948.91882	5737.87057	5615.90631	ASPM
792.266778	769.480495	709.205488	596.374372	564.335138	580.28648	ASPRV1
1339.07055	1311.28996	1499.46303	1153.21011	1089.75061	1036.95559	ATAD3B
102.775161	108.679204	120.386142	63.7011299	75.986012	74.5790723	ATAD3C
891.050671	860.707564	889.188729	601.865848	654.22103	615.021938	ATAD5
1936.76299	1976.05764	1845.12621	2155.95376	2118.34175	2209.78813	ATF2
7269.09758	7616.27035	7187.41024	10117.4967	10074.6332	9984.401	ATF4

2630.24588	2620.99336	2649.68706	3484.89113	3552.80939	3503.17313	ATF5
4070.09595	4297.19159	4100.28047	4792.96088	4700.01284	4653.52978	ATF7IP
602.681529	625.103743	675.831112	522.788584	549.508599	535.334711	ATG10
3837.60457	4052.06842	4027.57201	4840.18758	4601.78702	5010.07905	ATG13
1620.45497	1676.19823	1704.47706	930.256156	848.819354	887.797449	ATG16L2
978.858576	964.627095	998.847393	601.865848	520.78218	564.962013	ATHL1
13749.9196	14381.3525	14198.4131	17330.0022	17458.2496	17449.4597	ATIC
7762.01922	7993.87108	8535.49664	8841.27751	8633.67895	9108.86313	ATN1
4635.85825	4728.73529	5229.04954	5673.79374	5069.74966	5631.23077	ATP13A1
5676.58148	5756.03142	5611.66292	6834.69192	6890.63397	7036.9952	ATP13A3
332.273094	291.133342	288.449963	417.352231	442.016192	477.101736	ATP1A3
8063.35999	8088.27127	7636.77237	5476.10058	5450.60638	5357.43363	ATP1B1
106.76643	125.33806	125.15391	52.7181765	63.0127905	54.1464497	ATP2A1
6845.02531	6818.23181	7436.52612	5828.65339	5612.77164	5983.69351	ATP2A3
7459.68064	7540.11558	7536.64924	7796.79864	8183.32283	8258.86603	ATP2B1
1557.59249	1421.55572	1549.5246	2132.88956	2100.73524	2346.6867	ATP2C2
29415.6481	30792.7056	30626.9496	33632	33965.7474	34178.6694	ATP5B
1548.61214	1558.79296	1512.57439	1254.25328	1260.25581	1183.04885	ATP5S
136.700943	156.275936	168.063822	121.910783	112.125701	97.0549571	ATP6AP1L
175.61581	149.136426	174.023532	86.7653321	112.125701	96.0333259	ATP6V0A4
4903.27323	4742.22103	5014.49998	5365.17275	5366.28044	5483.09426	ATP6V0D1
264.421532	296.686294	282.490253	153.761348	94.5191857	148.136513	ATP6V1B1
6136.57517	6433.49156	6125.38992	6798.44818	6627.4629	6693.72714	ATP6V1C1
20020.2023	20453.9022	20527.6251	18275.6345	17927.1389	18176.861	ATP6V1G2__
544.808137	529.117	587.627404	1321.2493	1404.81456	1490.55981	ATP8A2
3533.27035	3675.26096	3669.98941	4325.08706	4304.32958	4408.33831	ATP8B1
2400.74794	2428.22659	2426.79391	2587.58383	2693.79679	2794.16113	ATP8B2
53.8821234	51.563126	47.6776799	34.0471557	27.7997605	27.5840404	ATP8B3
1336.0771	1439.80113	1278.95376	2092.25263	2000.6561	2117.84133	ATRNL1
10229.6209	10460.9683	10769.1959	11788.0039	11289.4827	12000.0792	ATXN2L
2898.65868	2864.52997	2985.8147	3638.65247	3557.44269	3644.15823	ATXN7L3
13711.0048	14053.7283	14339.0622	14785.2519	15102.6832	14958.723	ATXN7L3B
6573.61906	6844.41002	7022.92224	6404.16015	6209.53984	6130.80839	AUP1
6202.4311	6174.88266	6447.21426	8269.06564	7789.49289	7997.32846	AURKA
201.559054	168.175119	152.568576	104.338058	118.612312	117.48758	AVIL
97.7860758	102.332973	122.770026	51.6198811	51.8928863	59.2546054	AZGP1
19655.0012	19925.5784	19633.6686	18076.8431	17841.8863	17844.8309	AZIN1
25.9432446	44.4236162	29.7985499	23.0642022	11.1199042	13.2812047	AZU1
12442.7792	12738.472	12507.0474	10193.2791	10257.185	10036.5042	B2M
695.478519	745.68213	737.812096	996.153877	951.678468	981.787513	B3GNT9
654.568018	664.767686	688.942474	595.276076	578.235018	551.680809	B3GNTL1
2457.62352	2394.1156	2765.30543	2306.42022	2035.86913	2066.75977	B4GALNT4
1747.17774	1852.30614	1978.62371	2217.4583	1920.96345	2106.60338	B4GALT2

1352.04217	1444.56081	1458.937	1732.01176	1631.84594	1664.23711	B4GALT3
3555.22233	3324.63171	3279.03243	4039.53027	4332.12934	4227.5096	B4GALT5
1739.19521	1680.95791	1708.05288	2002.19241	1912.62352	1929.8612	BACE2
52.8843063	41.2505008	45.2937959	61.5045392	88.0325749	86.8386458	BACH1-AS1
5279.45028	5274.51115	5157.53302	4173.52231	3886.40652	3895.47949	BAG1
9615.9634	9766.05606	10154.1539	6125.19313	6255.87277	6671.25126	BAG3
12687.2444	12692.4618	13135.2008	13432.1521	13763.6614	14811.6081	BAG6
1801.05987	1762.66563	1909.49108	2249.30886	1944.12992	2170.96615	BAIAP2
1152.47875	1101.07106	1115.65771	1239.97544	1348.28838	1292.36338	BAIAP2-AS1
977.860758	924.963152	1137.11267	817.131736	696.84733	751.920509	BAIAP3
10126.8458	10190.4603	10390.1584	15550.7638	15039.6704	15086.4269	BAMBI
1020.76689	1064.58023	1045.33313	1391.5402	1280.6423	1400.65628	BANP
3763.7661	3591.96668	3650.91834	4637.00294	4510.97447	4269.39648	BAX
9817.52245	9667.68948	9413.95789	9807.77742	10966.0789	10808.8573	BAZ1B
8201.05875	8058.91995	8193.40928	8591.96447	9467.67177	9842.39428	BAZ2A
1717.24323	1734.10759	1674.67851	1518.94246	1370.52819	1317.90415	BBIP1
118.740235	123.751502	126.345852	221.855659	202.938252	178.785447	BBS12
7431.74176	7291.02601	7536.64924	8574.39175	8751.3646	8869.80144	BBS1__CTD-[illegible]
811.225303	776.620005	748.539574	950.025472	990.598132	881.667663	BBS9
4918.24049	4825.51531	4840.47645	3889.06381	4075.44489	4261.22343	BBX
737.386837	709.984581	805.75279	537.066423	592.134899	569.048538	BCAR3
1025.75598	975.732999	1001.23128	1108.18	1174.07655	1092.12368	BCAS4
2418.70865	2458.37119	2511.42179	2812.73437	2668.77701	2803.35581	BCAT2
3284.81389	3392.06041	3411.33799	3097.19287	3132.10635	3040.37423	BCCIP
2604.30263	2453.61152	2444.67304	4075.77402	4288.57639	4173.36315	BCL2
4878.3278	4988.93076	5086.0165	3983.51721	4075.44489	4011.94544	BCL2L11
1595.50954	1529.44164	1721.16424	1936.29469	1768.99143	1808.2871	BCL2L12
13478.5134	13569.8282	13451.0654	11603.4903	11804.705	11415.7062	BCL2L2__BC[illegible]
747.365008	831.356247	799.79308	506.314153	467.962635	485.274785	BCL6
24.9454275	28.558039	15.495246	7.6880674	10.1932455	3.06489338	BCL9P1
10752.4771	11355.7869	10782.3073	10031.8297	9780.8824	9889.38931	BCLAF1
2805.86169	2777.26929	2996.54218	3146.61616	3386.93749	3597.1632	BCOR
1139.50713	1059.02728	1056.06061	1377.26236	1473.38731	1473.19209	BCORL1
3884.50197	3759.34852	4230.20215	4691.91771	4438.69509	5060.13897	BCR
612.6597	674.287032	607.890418	418.450526	421.629701	450.539327	BDKRB1__B[illegible]
3642.03242	3743.48295	3768.92059	2851.17471	2928.24144	2974.98984	BHLHE40
2465.60606	2529.76629	2512.61373	3073.03037	2897.6617	2954.55722	BID
115.746784	103.126252	122.770026	83.4704461	81.5459641	77.6439656	BIN1
1599.50081	1573.07198	1560.25207	1749.58448	1705.05198	1736.77292	BIRC2
213.53286	214.978571	258.651413	161.449416	177.918467	190.02339	BIRC3
3325.7244	3308.76613	3351.74089	4297.62968	4313.59617	4109.00039	BIRC5
2082.44429	2068.07799	2097.81791	2259.19352	2286.99363	2273.12926	BLM
20466.2266	20742.6557	21277.3566	22410.7165	21932.1577	21960.9827	BLOC1S5__B[illegible]

3298.78333	3218.33234	3296.91156	4007.67971	3929.95948	3915.91211	BLVRA
196.569969	217.358408	200.246255	278.967017	264.097725	305.467707	BMP6
14449.3894	14436.0887	15093.5615	21544.1615	20628.349	21484.9026	BMP7
268.4128	252.262678	264.611123	398.68121	424.409677	418.868762	BMPR1B
5287.43282	5323.69444	5307.71771	5675.99034	5837.9497	5793.67012	BMPR2
621.640054	638.589483	700.861894	490.938019	507.808958	515.923719	BMS1P1
6561.64525	6458.87649	6515.15495	3622.17804	3583.38913	3479.67562	BNIP3
5049.95235	5305.44902	5318.44519	4147.16322	3788.1807	3758.58092	BNIP3L
6810.10171	7010.2053	7028.88195	7853.91	7697.75368	7706.16359	BOD1
20651.8205	20068.3686	19930.4621	21364.041	23668.7161	24002.2017	BPTF
4782.53736	4681.93184	4886.96219	5373.95912	5495.08599	5556.6517	BRD3
4062.11342	4247.21502	4099.08853	4512.89557	4715.76604	4673.96241	BRD8
1717.24323	1600.04346	1679.44627	1927.50833	1998.80278	1884.90943	BRI3
24.9454275	23.005087	38.1421439	142.778395	133.43885	134.855309	BRINP3
3413.5323	3393.64697	3491.19811	3922.01267	3931.81279	4094.69756	BRPF3
17325.0983	17119.7511	16821.8774	18857.7311	19902.7752	19241.4006	BSCL2__HNR
2862.73726	3020.8059	2939.32896	3384.94625	3265.5452	3426.5508	BSDC1
2406.73485	2329.86002	2439.90527	2090.05604	1951.54319	2033.04594	BSPRY
3905.45613	4113.1509	4077.63357	2888.51675	3283.15172	3336.64726	BTAF1
1550.60777	1550.86017	1576.93926	1674.9004	1901.50362	1744.94597	BTBD10
4166.88421	4132.18959	4537.72318	5268.52276	4737.07919	5198.05917	BTBD2
1659.36984	1664.29905	1737.85143	3200.43263	3120.05979	3191.57564	BTG2
421.078816	427.577306	393.340859	270.180655	296.530779	322.835436	BTN2A2
124.727138	148.343147	152.568576	98.8465809	86.1792575	107.271268	BTN3A1
3299.78115	3517.39847	3406.57023	4317.399	4224.63694	4497.22022	BUB1
2451.63662	2546.42514	2510.22984	2818.22585	2977.35435	2921.86502	BUB1B__PAI
12029.683	12575.0565	12340.1755	10914.8591	10786.3071	10544.2549	BUB3
95.7904416	109.472483	91.7795337	45.0301091	57.4528384	50.0599252	BX004987.4
1482.75621	1594.49051	1529.26158	1871.49527	1844.97744	1830.76298	BYSL
1684.31527	1650.81331	1657.99132	1837.44811	1955.24982	1867.5417	C10orf2
110.757698	103.126252	106.082838	148.269871	150.118707	156.309562	C10orf82
2664.17166	2841.52488	2760.53766	3154.30423	3196.0458	3372.40435	C11orf30
14187.9614	14037.8627	14088.7544	16031.8171	16327.726	16157.0963	C11orf31__C
97.7860758	92.0203479	104.890896	144.974985	132.512192	174.698923	C11orf45
141.690028	161.828888	160.91217	106.534648	121.392288	113.401055	C11orf72
3951.35572	4036.99613	4193.25194	3778.13598	3461.07018	3548.1249	C11orf80__R
1603.49208	1498.50377	1535.22129	2161.44524	2038.6491	1976.85623	C11orf84
6964.76336	7192.65944	7020.53836	5226.78754	5495.08599	5392.16909	C11orf91__C
4398.37778	4303.53782	4285.03148	5735.29828	5900.03584	5759.95629	C12orf49
1339.07055	1243.86125	1236.04385	1464.02769	1396.47464	1397.59138	C12orf65
999.812735	975.732999	1075.13168	1202.6334	1210.21624	1147.29176	C12orf66
3405.54976	3557.85569	3419.68159	3714.43485	3702.9281	3783.10006	C14orf119
503.897636	502.145519	482.736509	399.779505	391.049964	394.349615	C14orf28

3666.97784	3745.86278	3497.15782	2640.30201	2581.67109	2487.67179	C14orf37
1909.82193	1880.0709	2050.14023	2196.59069	2136.87492	2297.6484	C15orf39
1795.07296	1669.05872	1643.68801	1554.08791	1568.83315	1359.79103	C15orf40
442.032976	510.078308	430.291061	362.437463	330.81715	310.575863	C15orf48
100.779527	88.0539536	96.5473017	37.3420417	34.2863713	37.8003517	C15orf59
937.948074	978.906115	1042.94925	1167.48795	1088.82395	1146.27012	C16orf70
206.54814	160.24233	172.83159	250.411338	259.464431	267.667355	C17orf67
1679.32618	1653.98643	1649.64772	1922.01685	1858.87732	1869.58496	C17orf80
241.471738	291.926621	303.945209	427.236889	403.096527	441.344647	C17orf82
820.205657	850.394939	783.105892	925.862975	913.685462	947.052055	C19orf12
658.559286	633.82981	672.255286	837.999347	751.520192	844.888942	C19orf25
5379.23199	5429.20053	5757.07984	6654.57149	6203.97988	6578.28283	C19orf48
731.399935	765.514101	760.458994	583.194827	505.955641	542.486128	C19orf66
4788.52427	5076.19143	5194.48322	6233.92437	5904.66913	6082.79173	C1QTNF6
7750.04542	7697.97807	7709.48083	5543.0966	5841.65634	5968.36904	C1QTNF9__F
272.404068	276.061044	268.186949	148.269871	208.498204	184.915234	C1orf132__M
17.9607078	19.0386927	28.6066079	2.19659069	5.5599521	6.12978676	C1orf186
3917.42994	3970.3607	3959.63131	2979.67527	2985.69428	2864.65368	C1orf21
66.8537457	86.4673959	95.3553597	29.6539743	35.21303	39.843614	C1orf228
11797.1916	12138.7531	11670.3041	13637.5333	13760.8815	13512.0933	C1orf43
1837.9791	1841.20024	1886.84418	1475.01065	1409.44786	1453.78109	C21orf58
3238.91431	3142.97085	3146.72687	3902.24336	3736.28781	4021.14012	C22orf39__H
599.688077	594.165867	587.627404	971.991379	942.411881	955.225104	C2CD2
2206.17361	2172.7908	2213.43629	2327.28783	2497.34515	2438.6335	C2CD5
34008.6002	35165.2586	33970.3469	30686.3719	28840.3982	27711.7443	C2orf61__CA
2857.74818	2793.92815	2845.16555	2984.06845	3211.799	3252.87351	C3orf62__US
986.841112	973.353163	958.321365	839.097642	908.12551	806.066959	C4A__STK19
5104.83229	5172.17818	4590.16863	3767.15303	3912.35296	3380.5774	C4orf3
24.9454275	30.1445967	35.7582599	6.58977206	8.33992815	7.15141789	C4orf47
1906.82848	1918.94157	1876.1167	1580.447	1596.63291	1681.60484	C5orf30
1008.79309	1050.30121	1090.62693	1024.70956	881.252408	834.672631	C5orf38
9073.15089	8826.81389	8926.45361	5923.10679	5774.01026	5883.57366	C6orf141
13348.7972	13002.6338	12737.0922	12448.0794	12015.9832	11607.7729	C6orf62
6654.44224	6418.41927	6605.74254	7568.35321	7031.48609	7190.23987	C7orf50
3648.01932	3478.52781	3867.85178	4266.87741	3741.84776	4206.05535	C7orf61__TS
1064.67085	1033.64236	1014.34264	508.510744	429.04297	433.171598	C8orf88
432.054805	346.662862	364.734251	148.269871	166.798563	183.893603	C9orf106
427.065719	422.817633	367.118135	302.031219	310.430659	269.710618	C9orf152
2438.66499	2391.73577	2582.93831	3166.38548	3180.2926	3111.88841	C9orf16
5920.04886	5623.55385	5830.98025	5390.43355	5267.12796	5265.48683	C9orf172__M
55.8777576	46.010174	47.6776799	19.7693162	22.2398084	14.3028358	C9orf173
57.8733918	47.5967317	59.5970998	21.9659069	20.386491	14.3028358	C9orf173-AS
784.284241	768.687217	772.378414	977.482856	872.91248	935.814112	C9orf40

2245.08848	2335.41297	2336.20631	1260.84305	1212.99622	1283.1687	C9orf47__S1
1764.14063	1695.23693	1723.54813	1971.44014	1941.34994	1934.96935	C9orf91
10875.2086	10932.176	10925.3403	7155.39416	7181.6048	7236.21327	CA12
3836.60675	3828.36378	3773.68836	2144.97081	2162.82137	2216.93955	CA2
873.089963	819.457064	792.641428	926.96127	981.331546	934.792481	CA5B
137.69876	131.684291	150.184692	8.78636275	4.63329342	5.10815564	CA9
3678.95165	3759.34852	3907.18586	4196.58651	4068.03162	4417.53299	CABIN1
4607.91937	4690.65791	5084.82456	5700.15283	5210.60178	5980.62862	CACNA1H
66.8537457	74.568213	69.1326358	114.222716	116.758994	120.552473	CACNG6
6749.23487	6779.36115	7186.2183	7728.70433	7767.25308	8663.43196	CAD
464.982769	464.861413	505.383407	567.818693	568.041773	578.243218	CADM4
992.828015	1020.94989	1029.83789	1298.1851	1317.70865	1400.65628	CADPS2
2367.81998	2408.39462	2383.88399	1982.4231	1923.74343	2042.24062	CALCOCO1
7100.46649	7357.66144	7213.63296	5947.26928	5967.68192	5861.09778	CALCR
1595.50954	1661.91921	1727.12395	2037.33786	1970.07636	2129.07927	CALHM2
32783.2808	32367.3641	32184.8178	28373.3619	27796.0539	26909.7639	CALM1
12045.648	12058.632	11826.4485	14017.5435	14487.3819	13824.7124	CALM3
179.607078	194.353321	209.781791	101.043172	107.492407	125.660629	CALML3-AS1
2307.95095	2251.32541	2306.40776	1453.04474	1406.66788	1361.83429	CALML5
28489.6739	27988.4648	26803.1997	30494.1702	31645.394	30094.1881	CALR
15434.2349	15679.1567	15826.6058	16739.1193	16200.7738	16887.5625	CALR3__CHE
620.642236	621.930627	601.930708	764.413559	822.872911	750.898878	CAMK1D
838.166364	867.053795	938.058351	510.707335	521.708839	563.940382	CAMK2B
2173.24565	2218.0077	2088.28238	1703.45608	1619.79938	1627.45839	CAMK2N1
338.259997	272.887928	337.319585	211.971001	223.324743	222.715586	CAMKK1
4541.06562	4508.20377	4622.35106	4937.93586	4901.09778	5209.29712	CAMSAP1
6536.69982	6642.91718	6634.34915	6923.65385	7203.8446	7700.0338	CAMSAP2
15878.2635	16716.7654	16534.6194	17326.7073	17709.3741	17894.8908	CAND1
6168.50531	5959.11081	6248.15995	6834.69192	6802.60139	6875.57748	CANT1
51264.8492	51314.8297	49255.8111	53057.5497	54777.5747	52642.6087	CANX
10375.3022	10879.0263	10535.5753	11582.6227	11535.974	11441.247	CAP1
13108.3233	13135.1114	14415.3465	15579.3195	14297.4168	15673.8648	CAPN1
4215.77725	4163.92074	4411.37733	3242.16785	3180.2926	3178.29444	CAPN2
594.698992	590.992752	591.20323	473.365293	464.256	520.010244	CAPN5
797.255863	810.730996	834.359398	408.565868	421.629701	447.474434	CAPN8
507.888904	515.63126	479.160683	395.386324	395.683258	466.885425	CAPN9
15871.2788	16630.2981	16676.4605	17812.1539	17764.9736	17925.5398	CAPRIN1
469.971854	480.72699	488.696219	390.993142	359.543569	382.090042	CARD14
1007.79527	1067.75335	1089.43499	969.794788	906.272192	878.602769	CARD19
691.487251	692.532446	741.387922	498.626086	507.808958	520.010244	CARF
126.722772	121.371666	127.537794	51.6198811	45.4062755	50.0599252	CARNS1
2145.30677	2051.41914	2022.72557	2280.06113	2498.27181	2468.2608	CASC5
3970.31424	3904.51856	3802.29497	3204.82581	3316.51143	3282.50081	CASK

72.8406483	93.6069056	71.5165198	27.4573836	37.0663473	49.0382941	CASP14
1148.48748	1172.46616	1181.21452	1507.95951	1374.23483	1466.04067	CASP6
5144.74497	5151.55293	5084.82456	3865.99961	3869.72666	3863.80892	CAST
1592.51609	1574.65854	1737.85143	1775.94357	1853.31737	1930.88283	CASZ1
1860.92889	1972.88453	1938.09769	2561.22474	2534.4115	2612.31079	CAT
273.401886	283.200554	266.995007	183.415322	159.385294	158.352825	CATSPER2
301.340764	294.306458	288.449963	207.57782	167.725222	190.02339	CATSPERG
1500.71692	1564.34591	1512.57439	1350.90327	1285.27559	1342.4233	CAV2
3105.20682	3150.11036	3196.78843	3785.82405	3486.08997	3411.22633	CBFB
2390.76977	2644.79172	2489.96683	2810.53778	3033.88053	3106.78026	CBL
2620.26771	2580.53614	2648.49512	2934.64516	3256.27861	2931.0597	CBLL1
267.414983	287.166948	288.449963	461.284044	410.509797	445.431171	CBS
1196.3827	1185.15862	1201.47753	955.516949	919.245414	950.116948	CBWD1
608.668431	594.959146	531.60613	459.087454	388.269988	370.852099	CBWD5
13190.1443	13272.3486	12478.4408	15421.1649	16410.1986	15166.1141	CBX1
4193.82527	4275.77306	4343.43664	5209.21481	4852.91152	5261.4003	CBX2
4745.61813	4696.21086	4782.07129	5178.46254	5053.99646	5290.00598	CBX6
537.823417	545.775857	559.020796	667.763569	683.874108	648.735766	CC2D2A
3809.66569	3818.84444	3878.57926	4434.9166	4396.99545	4374.62449	CCAR2
179.607078	219.738245	244.348109	151.564757	160.311952	153.244669	CCBL1
293.358228	305.412362	303.945209	462.38234	361.396887	415.803869	CCDC102A
3531.27472	3509.46568	3621.11979	4232.83025	3826.1737	4129.43302	CCDC106__ZI
106.76643	95.9867422	104.890896	62.6028346	47.2595928	38.8219828	CCDC114
4774.55483	4835.03466	4681.94816	5960.44883	5748.99047	5550.52191	CCDC117
1406.92211	1444.56081	1298.02483	1257.54817	1187.97643	1198.37331	CCDC120
2725.0385	2710.63387	2436.32944	1982.4231	2010.84934	1840.97929	CCDC125
1016.77563	1043.1617	1093.01081	933.551042	864.572552	927.641063	CCDC130
170.626724	165.002003	175.215474	222.953955	205.718228	230.888635	CCDC136
2423.69774	2288.60952	2379.11623	2830.3071	2713.25663	2848.30758	CCDC137
4682.75565	4708.90332	4929.8721	3959.35471	4202.39713	4223.42308	CCDC14
491.923831	486.279942	411.219989	314.112468	374.370108	374.938624	CCDC146
186.591798	215.77185	215.741501	141.680099	130.658874	142.006727	CCDC162P
293.358228	306.20564	281.298311	194.398276	252.977821	246.213102	CCDC180
224.508848	213.392014	230.044805	165.842597	183.478419	169.590767	CCDC183-AS1
595.696809	663.181128	624.577606	419.548821	495.762396	450.539327	CCDC191
1714.24978	1711.89578	1916.64273	1409.11293	1538.25341	1443.56478	CCDC57
2158.27839	2230.70016	2218.20406	1913.23049	1750.45825	1873.67149	CCDC64B
313.31457	306.998919	308.712977	464.57893	457.76939	503.664146	CCDC69
971.873856	981.285951	986.927973	1154.30841	1095.31056	1229.02225	CCDC71
694.480702	732.196389	753.307342	977.482856	884.959043	879.6244	CCDC74A
317.305838	318.898102	315.864629	418.450526	462.402683	457.690745	CCDC77
1189.39798	1133.59549	1146.6482	725.973222	647.73442	567.005275	CCDC84
8488.43007	8184.25801	8353.12951	8903.88035	8702.25169	9030.19753	CCDC85C

4130.9628	3975.91365	4234.96991	5746.28124	5685.97768	6012.29918	CCDC88C
60.8668431	43.6303374	78.6681718	28.5556789	36.1396887	24.5191471	CCDC89
2256.06446	2325.89362	2412.4906	2986.26504	2784.60934	2976.01147	CCDC90B
37.9170498	28.558039	38.1421439	6.58977206	9.26658683	13.2812047	CCER2
36.9192327	41.2505008	41.7179699	6.58977206	12.9732216	6.12978676	CCL22
3334.70475	3396.02681	3287.37603	3700.15701	3914.20628	3890.37133	CCNA2
7796.94282	8136.66128	8088.51839	10205.3603	10134.866	9856.69711	CCNB1
2916.61938	3124.72543	3047.79569	3922.01267	3994.82558	3730.99688	CCNB2
1863.92234	1991.12994	2060.86771	2306.42022	2273.09375	2442.72002	CCNF
3544.24634	3338.91073	3337.43759	3652.93031	3804.86055	3617.59582	CCNK
8464.48246	8444.45348	8362.66505	5672.69545	5685.97768	5568.91127	CCNL1
9693.79313	9510.62027	9902.65411	6957.701	6826.69452	6869.4477	CCNL2
3561.20923	3735.55016	3555.56298	3980.22232	4225.5636	3943.49615	CCNT1
1308.13822	1416.79605	1348.0864	1015.92319	1034.15109	1028.78255	CCNYL1
4887.30816	5050.01323	5041.91465	4516.19045	4423.86855	4452.26845	CCPG1__DYX
17682.3168	18377.0981	18191.4188	20119.6724	18948.3168	19332.3258	CCT2
26729.5245	27565.6472	27300.2395	29204.7715	28512.361	29173.6985	CCT3
21170.6854	22022.2145	21752.9414	22334.9341	23445.3913	23068.4309	CCT5
801.247132	815.490669	777.146182	612.848802	555.068551	552.70244	CCT6P3__RP
20965.1351	21624.7818	21823.266	24160.301	23706.7091	23825.4595	CCT7
1172.43509	1105.83073	1145.45626	754.528901	805.266396	823.434688	CD109
73.8384654	80.121165	75.0923458	39.5386324	30.5797366	41.8868762	CD22
59570.6787	60830.2096	60114.4027	51559.4749	49196.3095	49150.6735	CD24
7528.53002	7533.76935	8037.26488	9460.71609	8918.16317	9463.36913	CD276
1546.61651	1346.19423	1419.60292	1728.71687	1709.68527	1688.75625	CD320__CTD
7469.65881	7601.99133	7510.42652	6426.12605	6447.69112	6547.63389	CD44
7151.35516	7337.03619	7263.69453	6384.39083	6541.28365	6420.95163	CD46
7528.53002	7952.62058	7754.77463	5695.75965	5737.87057	5422.81802	CD47
808.231851	792.485582	764.03482	652.387434	625.494611	558.832226	CD58
7710.13274	8037.50142	8503.3142	7640.8407	7129.71191	7475.27496	CD81
561.771028	551.328809	528.030304	842.392528	925.732025	923.554539	CDC14A
3224.94487	3505.49929	3606.81648	4165.83424	3979.99904	4357.25676	CDC20
654.568018	725.850158	725.892676	950.025472	987.818156	956.246735	CDC25C
4636.85607	4688.27807	4791.60683	5220.19777	5274.54123	5352.32547	CDC27
13640.1598	13704.6856	14033.9251	13861.5855	14886.7718	15796.4605	CDC42BPB
2692.11054	2676.52288	2846.35749	3315.75364	3119.13313	3195.66217	CDC42EP4
3780.72899	3791.07968	3752.23341	4246.0098	4182.9373	4427.7493	CDCA5
705.45669	698.085398	678.214996	477.758474	481.862515	505.707408	CDCA7
2093.42028	2125.19407	2199.13298	2698.51166	2660.43708	2749.20936	CDCA8
3598.12846	3602.27931	3723.6268	2986.26504	2773.48944	2895.30261	CDK10
3557.21796	3588.00029	3555.56298	4206.47117	4008.72546	4123.30323	CDK14__CLD
1780.10571	1715.0689	1988.15925	2136.18444	2008.99603	2168.92288	CDK18
2969.50369	2917.67965	2946.48062	3238.87297	3356.35775	3384.66392	CDK19

4109.01082	4201.20485	4143.19038	4516.19045	4654.60657	4545.23688	CDK2
4342.50002	4594.67116	4748.69691	5840.73464	5104.03603	5119.39358	CDK2AP1
10155.7825	10219.0183	10617.8193	12427.2118	12301.394	12578.3224	CDK4
17660.3649	17467.2073	18082.952	14948.8979	14691.2468	15029.2155	CDK5RAP3__
2931.58664	2935.13179	3051.37151	3367.37352	3324.85136	3556.29795	CDK9
193.576518	168.175119	158.528286	116.419306	105.63909	88.881908	CDKL1
5416.15122	5524.39399	5435.2555	6909.37601	6673.79584	6635.49417	CDKN1B
560.77321	584.646521	581.667694	653.485729	679.240815	712.076895	CDON
3367.63271	3418.23861	3283.8002	2305.32193	2219.34755	2303.77819	CDRT1__RP1
546.803771	570.367501	573.3241	439.318137	447.576144	446.452803	CDRT4__TVP
2429.68464	2500.41497	2534.06868	3004.93606	2971.7944	3124.14799	CDYL
2266.04264	2210.86819	2217.01211	2627.12246	2594.64431	2453.95797	CEBPG
12201.3075	12521.1136	12832.4475	11689.1573	11739.8389	11679.287	CELF6__HEX
494.917282	533.083395	570.940216	471.168702	367.883497	469.950318	CEMP1
2538.4467	2603.54122	2514.99761	2947.8247	3052.4137	3138.45082	CENPE
4630.86916	4693.83102	4603.27999	5312.45458	5910.22908	6098.1162	CENPF
747.365008	744.095572	735.428212	1044.47887	904.418875	959.311628	CENPM
3163.08021	3146.93724	2992.96635	3528.82294	3570.41591	3389.77208	CENPN
2052.50978	2125.98735	2178.86997	1704.55437	1662.42568	1743.92433	CENPT
234.487019	264.161861	269.378891	339.373261	316.91727	332.030116	CEP126
1138.50931	1108.21057	1158.56762	1236.68056	1250.06256	1323.01231	CEP152
549.797222	554.501924	573.3241	688.63118	683.874108	671.21165	CEP162
283.380057	277.647601	266.995007	374.518712	409.583138	382.090042	CEP164P1__
933.956806	877.366421	921.371163	1004.94024	1040.6377	1075.77758	CEP170
6671.40513	6961.81529	7279.18977	6153.74881	5334.77404	6180.86832	CEP170B
101.777344	93.6069056	89.3956497	161.449416	152.898683	138.941833	CEP19
1707.26506	1659.53938	1622.23306	1429.98054	1428.90769	1453.78109	CEP290
1016.77563	1043.1617	1032.22177	1071.93626	1185.19646	1277.03891	CEP295
3274.83572	3304.00646	3463.78344	3889.06381	3840.07358	3599.20646	CEP55
4744.62031	4762.84628	4616.39135	3582.63941	3584.31579	3529.73554	CEP95
991.830198	953.521191	998.847393	1135.63739	1190.75641	1161.59459	CEP97
662.550555	679.839984	721.124908	877.537979	724.64709	893.927236	CERS1
18208.1665	18750.7324	18867.2499	21525.4904	21956.2508	22307.3157	CERS2
2629.24806	2531.35285	2520.95732	2823.71733	3209.94568	3236.52741	CERS6
2251.07538	2232.28672	2376.73234	1706.75096	1690.22544	1651.97753	CFAP44
1383.97232	1370.78587	1289.68124	1534.3186	1547.52	1550.83605	CFAP97
46138.0649	45798.3685	44923.1019	49463.9274	52172.7372	49301.8749	CFL1
893.046305	914.650527	852.238527	779.789694	795.07315	768.266607	CFL2
3188.02564	2912.91998	2956.01615	2630.41735	2813.33576	2728.77674	CFLAR
23.9476104	47.5967317	38.1421439	8.78636275	15.7531976	6.12978676	CGA
6377.04909	6263.72989	6236.24053	6947.81634	6838.74108	6810.19309	CGGBP1
5191.64237	4955.61305	5091.97621	4072.47913	4230.19689	4248.96386	CGN
1962.70624	1928.46091	1932.13798	2181.21455	2290.70027	2439.65513	CH17-140K2

8937.44777	8708.61534	8789.38028	10774.2773	10560.2024	10420.6375	CHCHD2
14024.3194	13741.1764	13553.5724	14495.3019	14965.5377	15193.6981	CHD3
19828.6214	19820.8656	19987.6753	21822.0302	23477.8244	23917.4063	CHD4
571.749199	604.478492	632.9212	689.729476	686.654084	770.30987	CHD5
7184.28312	7417.95063	7210.05714	8590.86618	8314.90837	8496.90608	CHEK1__STT[illegible]
1812.03585	1790.43039	1941.67351	1155.4067	1131.45025	1171.8109	CHKB__CHKB[illegible]
4035.17235	4139.3291	4020.42035	3811.08484	3589.87574	3666.63411	CHMP5
1770.12754	1761.87235	1854.66175	2162.54353	2113.70846	2076.97608	CHMP7
567.75793	593.372588	582.859636	719.38345	643.101126	679.384699	CHN1
134.705309	147.549868	119.1942	86.7653321	84.3259402	80.708859	CHN2
4204.80126	4265.46044	4205.17136	4645.7893	4764.87895	4655.57305	CHP1
1941.75208	1881.65746	1969.08818	1800.10607	1648.5258	1612.13392	CHPT1
3913.43867	3886.27314	3933.40859	4102.13311	4262.62994	4423.66278	CHRNA10__[illegible]
753.351911	796.451977	728.27656	912.68343	863.645893	956.246735	CHRNA5
525.849612	599.718819	513.727001	638.109595	694.994012	718.206682	CHST10
1016.77563	870.226911	980.968263	1188.35556	1104.57715	1157.50807	CHST14
2716.05815	2674.93632	2959.59198	2465.67305	2203.59435	2336.47039	CHTF18
6115.62101	6648.47014	6842.939	8795.14911	8365.87459	8949.48867	CHTF8
2075.45957	2279.88345	2283.76087	2564.51963	2472.32537	2601.07285	CIAPIN1
732.397752	771.067053	716.35714	605.160734	616.228024	574.156693	CIART
457.998049	414.884844	456.513785	291.048266	354.910276	374.938624	CICP14
329.279643	322.071218	296.793557	188.906799	264.097725	243.148208	CICP27
26.9410617	30.1445967	25.0307819	12.0812488	13.8998803	9.19468014	CILP
203.554689	199.112994	199.054313	308.620992	273.364312	322.835436	CILP2
6637.47935	6284.35514	6126.58186	6008.77382	6071.46769	5474.92121	CIRBP
4048.14398	4186.13255	4330.32527	4542.54954	4490.58798	4538.08547	CIRH1A
626.629139	654.455061	576.899926	676.549932	729.280384	772.353132	CISD3
2988.46222	2997.00754	3054.94734	3359.68546	3610.26223	3725.88872	CIT
37.9170498	21.4185293	32.1824339	126.303965	107.492407	120.552473	CITED1
4537.07436	4632.74855	4653.34155	5435.46365	5534.00566	5407.49356	CKAP2
874.08778	870.226911	945.210003	1085.1158	1111.06376	1103.36162	CKAP2L
12787.0261	12963.7632	12813.3765	15032.3684	16105.3279	16492.1913	CKAP5
407.109377	357.768766	362.350367	321.800536	296.530779	248.256364	CKMT2-AS1
3692.92109	3709.37196	3699.78796	4213.06094	3996.6789	4134.54117	CKS1B
1738.19739	1838.8204	1791.48882	1580.447	1514.16029	1481.36513	CLASRP
442.032976	464.861413	439.826597	350.356215	351.203641	334.073379	CLCN2
78.8275509	90.4337902	94.1634177	12.0812488	6.48661078	7.15141789	CLDN1
470.969671	518.804375	516.110884	403.074391	328.963833	278.905298	CLEC2D
8908.51107	9046.55213	8906.1906	9716.6189	9375.0059	9399.00637	CLIC4
102.775161	124.544781	97.7392437	71.3891973	70.4260599	77.6439656	CLIC6
6106.64065	6291.49465	6053.8734	6423.92946	6673.79584	6731.5275	CLINT1
257.436812	263.368582	289.641905	196.594867	164.018587	184.915234	CLIP3
2074.46175	2008.58208	2084.70655	1680.39188	1673.54558	1606.00413	CLK1

3013.40764	3126.31199	3146.72687	2675.44746	2554.79799	2682.80334	CLK2
5683.5662	5588.64958	5592.59185	6062.5903	6242.89955	5798.77828	CLNS1A
8583.2227	8811.74159	9319.79447	9700.14447	9362.95934	10242.8737	CLPTM1
1498.72129	1470.73901	1667.52685	999.448763	1004.49801	1107.44814	CLSTN2
1071.65557	1040.78187	1101.35441	1216.91124	1258.40249	1294.40664	CLSTN3
4997.06804	4884.21795	5002.58056	4358.03592	4145.87095	4383.81917	CLTB
10855.2522	10625.1771	11185.1837	7336.61289	7112.10539	7270.94873	CLU
1286.18624	1305.73701	1342.12669	1047.77376	1141.6435	1138.09708	CLUHP3
135.703126	107.092646	100.123128	80.1755601	72.2793773	65.3843921	CMB9-22P13
3286.80953	3300.83334	3041.83598	2762.21279	2811.48245	2578.59696	CMC2
3425.50611	3274.65514	3493.58199	3648.53713	3733.50784	3784.12169	CMIP
250.452092	216.565129	226.468979	465.677226	417.923066	464.842163	CMTM3
4920.23612	4955.61305	5021.65163	5576.04546	5988.06841	6200.27931	CMTM4
5145.74279	5228.50097	5233.81731	6444.79708	5803.66333	5811.03785	CMTM6
2882.6936	3046.19083	3017.99714	3414.60022	3158.97945	3176.25117	CMTR2
1100.59226	1248.62093	1302.7926	861.063549	952.605126	1088.03715	CMYA5
18780.9135	19417.88	18498.9398	17829.7266	18204.2098	17174.6409	CNBP
4172.87111	4258.32093	4290.99119	4578.79329	4541.55421	4674.98404	CNDP2
12.9716223	18.2454138	14.303304	45.0301091	33.3597126	32.6921961	CNGB3
314.312387	283.200554	312.288803	508.510744	522.635497	502.642514	CNIH2
1089.61627	1050.30121	1116.84965	929.157861	901.638899	958.289997	CNKSR1
1205.36306	1140.735	1213.39695	1548.59643	1486.36053	1580.46335	CNN2
3010.41419	3083.47493	3144.34299	3334.42466	3310.02482	3443.91853	CNNM4
12108.5105	12552.8447	12641.7368	13339.8952	14179.7312	14460.167	CNOT1
4142.9366	4267.84027	4540.10706	4635.90464	4611.05361	4937.54324	CNOT3
1788.08824	1721.41513	1672.29462	1964.85037	2211.00762	2010.57006	CNOT4
5767.38284	5843.29209	5642.65341	6184.50108	6248.4595	6089.94315	CNOT6
1957.71715	2024.44765	2044.18052	2615.04121	2478.81198	2546.9264	CNP
66.8537457	47.5967317	44.1018539	112.026125	101.932455	118.509211	CNTNAP2
1861.92671	1889.59025	1878.50059	2102.13729	2113.70846	2045.30552	COA7
2440.66063	2507.55448	2517.3815	2956.61106	3056.12034	3268.19798	COBL
365.201059	346.662862	306.329093	596.374372	575.455042	614.000307	COCH
5201.62054	5345.90625	5431.67968	6130.68461	5821.26985	6383.15128	COG4
6487.80679	6231.20546	6587.86341	7258.63392	6810.01466	6892.94521	COG8__PDF_
414.094097	425.990749	446.978249	164.744302	176.06515	201.261332	COL11A2
5151.72969	5220.56819	5188.52351	5247.65515	5999.18832	6203.3442	COL12A1
39.912684	34.9042699	36.9502019	19.7693162	11.1199042	10.2163113	COL13A1
13273.9609	13405.6195	14515.4696	10657.858	9284.19335	10720.9971	COL18A1
1467.78896	1371.57915	1536.41323	1634.26347	1547.52	1760.27043	COL1A1
315.310204	356.975488	383.805323	461.284044	496.689054	515.923719	COL21A1
1136.51368	1109.00385	1196.70976	526.08347	567.115114	609.913783	COL27A1
5676.58148	5815.52733	5807.14141	4202.07798	4278.38314	4498.24185	COL4A5
593.701175	615.584396	657.951982	541.459604	485.56915	521.031875	COL4A6

6460.86573	6095.55477	6494.89194	4105.42799	3930.88613	4253.05038	COL5A1
85.8122706	58.7026357	47.6776799	32.9488603	36.1396887	29.6273027	COL5A3
453.008964	504.525356	507.767291	387.698256	387.34333	386.176566	COL6A1
1653.38294	1736.48743	1787.913	1285.00555	1131.45025	1279.08217	COL6A2
468.974037	510.078308	531.60613	187.808504	140.85212	157.331194	COL7A1
196.569969	223.704639	194.286545	629.323232	561.555162	565.983644	COL9A3
14128.0923	15066.7454	14672.806	15407.9854	15722.6179	15456.2573	COPB1__PSN
1041.72105	1015.39694	1016.72652	1155.4067	1218.55617	1139.11871	COQ2
2977.48623	2935.13179	3013.22937	2634.81053	2621.51742	2724.69022	COQ4
4573.99359	4736.66808	4872.65888	5353.0915	5035.46329	5156.1723	COQ9__POLF
8466.4781	8880.75685	9317.41059	11317.9335	9936.56106	10678.0885	CORO1B__PT
4235.73359	4393.17833	4378.00295	4817.12338	4963.18391	4995.77621	CORO1C
956.906599	992.391855	1008.38293	1196.04363	1187.97643	1122.77261	COX10
603.679346	638.589483	629.345374	540.361309	513.368911	506.729039	CP
55.8777576	68.2219821	88.2037077	40.6369277	41.6996408	46.9950318	CPA6
295.353862	303.032525	319.440455	168.039188	192.745006	201.261332	CPAMD8
12215.2769	12638.5188	12751.3955	13447.5282	13876.7138	13732.7656	CPNE1__NFS
1024.75816	1093.93155	1107.31412	1402.52315	1253.7692	1385.33181	CPNE2
248.456458	234.810543	227.660921	165.842597	151.972024	168.569136	CPS1
1115.55952	1147.87451	1208.62918	1513.45098	1333.46185	1373.07224	CPT2
26.9410617	19.0386927	22.6468979	6.58977206	5.5599521	11.2379424	CPXM1
10533.9551	10206.3258	9955.09955	7592.51571	7944.24489	7878.81925	CRABP2
531.836515	544.189299	597.16294	634.814709	700.553965	726.379731	CRACR2A
489.928196	575.127174	576.899926	478.85677	364.176863	462.798901	CRACR2B
17147.4869	17391.8458	17830.2603	18388.7589	18526.6871	18582.4486	CRAMP1__HI
352.229436	330.797285	326.592107	511.80563	531.902084	521.031875	CREB3L1
7236.16961	7142.68287	7208.86519	7717.72138	8364.02128	8314.03411	CREBBP
2441.65845	2565.46384	2470.89576	3224.59513	3167.31938	3127.21288	CREBL2
8178.10896	8275.48508	8288.76464	5869.29032	5811.0766	6008.21266	CREBZF
3724.85124	3733.9636	3836.86129	4512.89557	3987.41231	4141.69259	CREG1
451.013329	443.442883	407.644163	332.783489	263.171066	304.446076	CREG2
1586.52919	1588.14428	1559.06013	1268.53112	1369.60153	1376.13713	CRIM1
2287.99461	2325.10034	2324.28689	2400.87362	2550.1647	2615.37569	CRIP3__ZNF3
440.037341	414.091566	412.411931	519.493697	499.46903	517.966981	CRISPLD2
1451.82388	1462.01294	1591.24257	1388.24531	1275.08235	1373.07224	CROCC
259.432446	255.435793	303.945209	215.265887	199.231617	171.634029	CROCCP3
2147.3024	2202.9354	2162.18278	1773.74698	1716.17188	1737.79455	CROT
8448.51739	8526.1612	8920.4939	10636.9904	10057.9534	10244.9169	CRTAP
2006.61019	2013.34175	2050.14023	2116.41513	2286.99363	2275.17252	CRTC3
1715.2476	1805.50269	1823.67126	1973.63673	2051.62233	2091.27892	CRY2
692.485068	660.008013	657.951982	552.442558	547.655282	583.351374	CRYZL1
1476.76931	1423.14228	1491.11944	912.68343	821.946252	824.456319	CSAD
33.9257814	32.5244333	21.4549559	6.58977206	12.0465629	21.4542537	CSF1R

463.984952	447.409278	433.866887	267.984064	279.850922	267.667355	CSGALNACT1
7709.13492	7855.04728	8154.0752	9245.4502	8637.38559	9462.3475	CSK
17383.9695	17404.5382	17259.3201	16492.0029	15788.4106	16216.3509	CSNK1A1
8621.13975	8680.85058	8728.59124	9032.3809	9416.70554	9272.32411	CSNK1D
2516.49473	2548.80498	2723.58746	3057.65424	2677.11694	2964.77353	CSNK1G2
2483.56676	2591.64204	2453.01663	2817.12756	2925.46146	2712.43064	CSNK1G3
221.515396	210.218898	210.973733	161.449416	156.605318	175.720554	CSPG4P10__
1954.7237	1981.6106	1854.66175	2386.59578	2365.75962	2348.72996	CSRP2BP__P
15.9650736	11.1059041	9.53553597	46.1284044	28.7264192	34.7354583	CT83
12.9716223	11.1059041	9.53553597	25.2607929	23.1664671	25.5407782	CTA-221G9.1
68.8493799	80.9144438	82.2439978	45.0301091	34.2863713	32.6921961	CTA-228A9.3
31.9301472	28.558039	16.687188	4.39318137	9.26658683	9.19468014	CTA-280A3.2
35.9214156	22.2118081	25.0307819	48.3249951	63.0127905	46.9950318	CTA-392C11.
199.56342	189.593648	171.639648	68.0943113	96.3725031	81.7304902	CTA-392E5.1
77.8297338	71.3950975	58.4051578	14.2778395	16.6798563	29.6273027	CTB-131B5.5
258.434629	220.531523	225.277037	314.112468	379.003401	376.981886	CTB-33O18.1
149.672565	126.131339	132.305562	97.7482856	102.859114	87.8602769	CTB-50L17.8
6629.49682	6460.46305	6702.28985	7668.29809	7106.54544	7495.70758	CTC-326K19.
171.624541	131.684291	127.537794	102.141467	105.63909	78.6655968	CTC-429P9.3
4093.04575	4085.38614	4008.50093	3599.11384	3752.04101	3762.66744	CTC-432M15
206.54814	161.828888	171.639648	123.009079	132.512192	111.357793	CTC-471F3.5
83.8166364	75.3614918	84.6278818	54.9147672	41.6996408	45.9734007	CTC-490E21.
1795.07296	1797.5699	1721.16424	1655.13108	1453.92747	1256.60629	CTC-510F12.
46.8974037	41.2505008	47.6776799	26.3590882	29.6530779	20.4326225	CTC-518B2.8
159.650736	176.901186	168.063822	263.590882	246.49121	254.386151	CTC-523E23.
57.8733918	65.0488666	60.7890418	106.534648	109.345725	133.833678	CTC-523E23.
6779.16938	6902.31937	7031.26584	7798.99523	8298.22851	8115.83767	CTCF
1730.21485	1669.05872	1640.11219	1335.52714	1169.44326	1208.58962	CTD-2006C1.
125.724955	129.304454	148.99275	180.120436	190.891689	191.045021	CTD-2012K14
145.681297	132.47757	140.649156	81.2738554	90.812551	87.8602769	CTD-2013N1
77.8297338	76.1547707	61.9809838	51.6198811	44.4796168	44.9517696	CTD-2017D1
300.342947	287.166948	265.803065	237.231794	207.571545	216.585799	CTD-2017F17
2161.27184	2217.21442	2139.53588	1623.28052	1541.96005	1588.6364	CTD-2033A1
8803.74028	8819.67438	8851.36127	10115.3001	9918.02789	10026.2879	CTD-2132N1
81.8210022	78.5346073	88.2037077	136.188623	122.318946	125.660629	CTD-2147F2.
7148.36171	7107.7786	7055.10468	8452.48096	8384.40777	8720.6433	CTD-2192J16
93.7948074	92.0203479	87.0117657	45.0301091	44.4796168	41.8868762	CTD-2196E14
98.7838929	90.4337902	94.1634177	191.10339	107.492407	140.985096	CTD-2207O2
1415.90247	1268.4529	1408.87544	895.110705	809.899689	843.867311	CTD-2228K2.
60.8668431	67.4287032	53.6373898	28.5556789	41.6996408	31.6705649	CTD-2240E14
137.69876	126.924618	131.11362	75.7823787	110.272383	73.5574411	CTD-2336O2
6578.60814	6365.26958	6716.59315	7738.58899	7073.18573	7948.29017	CTD-2369P2.
589.709906	506.905192	464.857379	657.878911	664.414276	620.130094	CTD-2510F5.

11268.3485	11069.4132	10899.1176	13209.1981	13842.4274	13693.9436	CTD-2510F5.
114.748967	101.539694	103.698954	61.5045392	68.5727426	55.1680809	CTD-2540F13
272.404068	233.223985	196.670429	179.022141	173.285174	149.158145	CTD-2547G2
63.8602944	82.5010016	76.2842878	120.812488	118.612312	124.638998	CTD-2547L24
158.652919	172.141513	133.497504	91.1585135	116.758994	116.465949	CTD-2574D2
2309.94659	2352.07182	2451.82469	1976.93162	2152.62812	2293.56188	CTD-2653D5.
23.9476104	30.9378756	22.6468979	16.4744302	9.26658683	6.12978676	CTD-3032H1
427.065719	421.231075	426.715235	315.210764	296.530779	290.14324	CTD-3092A1
429.061353	443.442883	430.291061	267.984064	275.217629	270.732249	CTD-3184A7.
331.275277	321.277939	294.409673	106.534648	110.272383	126.68226	CTD-3247F14
540.816868	487.073221	511.343117	389.894847	396.609916	374.938624	CTD-3252C9.
913.002647	931.309383	1067.98003	1163.09477	1045.271	1247.41161	CTDP1
4618.89536	4641.47462	4637.84631	5078.51767	5171.68211	5243.01094	CTDSP1
39.912684	35.6975488	35.7582599	85.6670368	54.6728623	62.3194987	CTF1
23635.2937	24162.4808	24197.6145	24579.8498	25830.6108	26508.2629	CTNNA1
17325.0983	17585.4058	17790.9262	19424.4514	19735.9766	19869.7038	CTNNB1
3519.30091	3747.44934	3653.30222	3870.39279	3906.79301	4051.78905	CTPS1
2608.2939	2725.70617	2637.76764	2963.20084	2916.19488	2871.8051	CTPS2
2794.8857	2817.72652	2854.70108	3380.55307	3226.62554	3228.35436	CTSH
317.305838	333.970401	308.712977	208.676115	193.671665	217.60743	CTSK
4695.72727	4708.90332	4531.76347	3751.77689	4197.76384	3811.70573	CTSL
299.34513	295.099736	348.047063	427.236889	376.223425	368.808837	CTSZ
1188.40017	1108.21057	1199.09365	1379.45895	1393.69466	1485.45166	CTTNBP2NL
1244.27792	1266.86634	1300.40872	1503.56633	1427.98103	1528.36017	CTU2
980.85421	959.867422	1083.47528	1336.62543	1255.62252	1248.43324	CTXN1
1958.71497	2012.54847	2044.18052	2292.14238	2200.81437	2372.22748	CUEDC2
44.9017695	40.4572219	30.9904919	20.8676115	20.386491	14.3028358	CXCL17
64.8581115	57.116078	42.9099119	19.7693162	22.2398084	19.4109914	CXCL8
480.947842	521.184212	450.554075	761.118673	712.600527	747.833985	CXCR4
6455.87664	6451.73698	6177.83537	7121.34701	6933.26027	6862.29628	CYB5B
2813.84422	2785.99536	2749.81019	2369.02306	2362.97964	2374.27074	CYB5R1
1232.30412	1237.51502	1257.49881	1538.71178	1450.22084	1486.47329	CYB5R4
8239.97362	7625.78969	8043.22459	9874.77343	9485.27828	9090.47377	CYBA
6958.77646	7323.55045	7506.85069	8166.92417	8008.18434	8464.21389	CYFIP1
1768.1319	1880.0709	1874.92476	2339.36908	2313.86673	2629.67852	CYFIP2
465.980586	454.548788	436.250771	372.322121	298.384096	290.14324	CYP1A1
27159.5837	27207.0851	27350.3011	21119.1212	20531.9764	21157.9806	CYP1B1
530.838697	494.212731	497.039813	928.059565	956.311761	965.441415	CYP24A1
1233.30194	1295.42438	1252.73104	1502.46803	1429.83435	1495.66797	CYP27B1__M
117.742418	126.131339	107.27478	57.1113579	64.8661078	53.1248186	CYP2C8
38.9148669	58.7026357	60.7890418	25.2607929	19.4598324	13.2812047	CYP2E1
506.891087	499.765683	473.200973	587.588009	625.494611	563.940382	CYP2J2
40.9105011	45.2168951	47.6776799	24.1624976	15.7531976	15.3244669	CYP4F23P

458.995866	428.370585	456.513785	371.223826	385.490012	334.073379	CYP4F33P__I
343.249083	357.768766	365.926193	300.932924	299.310755	284.013453	CYP4V2__KLI
191.580883	176.901186	196.670429	149.368167	120.465629	114.422686	CYSRT1
2431.68027	2402.84167	2613.9288	2135.08615	2072.00882	2126.01438	D2HGDH
2292.9837	2219.59425	2357.66127	2940.13663	2931.94807	3105.75863	DAB2IP
4452.2599	4663.68643	4300.52672	4124.09901	4229.27023	3988.44792	DAD1
7257.12377	6878.52101	7075.36769	7723.21286	7655.12738	7602.97885	DAG1
15455.1891	15868.7503	15851.6366	17375.0323	16907.8143	16903.9086	DAGLB__KDE
1281.19716	1369.99259	1249.15521	1558.48109	1456.70745	1436.41336	DANCR
7489.61516	7582.95263	7839.40251	8294.32643	8070.27047	8274.1905	DAP
318.303655	296.686294	296.793557	155.957939	213.131497	176.742185	DAPK2
1596.50736	1595.28379	1775.99358	1920.91856	1806.05777	1847.10908	DAPK3
5808.29334	5937.69228	5972.82134	5131.23584	4991.91033	5034.59819	DARS
120.735869	107.092646	122.770026	75.7823787	93.592527	52.1031875	DARS-AS1
6297.22372	6649.26342	6748.77558	7258.63392	7282.61059	7325.09518	DARS2
4077.08067	4249.59486	4169.4131	4559.02397	4512.82779	4616.75106	DAXX
17450.8233	18462.7722	18024.5469	20476.6184	20201.1593	20412.1899	DAZAP2
2650.20222	2838.35177	2751.00213	3156.50082	2980.13433	3037.30934	DBF4
830.183828	802.798208	840.319108	992.85899	1010.05797	1067.60453	DBF4B
3234.92304	3183.42807	3536.4919	4513.99386	4187.57059	4332.73761	DBN1
2313.93786	2410.77446	2443.48109	3226.79172	3096.89332	3121.08309	DBNDD2__SY
1275.21025	1239.89486	1373.11718	1074.13285	970.211641	1099.27509	DBP
871.094329	893.231998	835.55134	991.760695	1001.71804	1098.25346	DBT
3491.36203	3670.50129	3656.87805	4496.42114	4499.85457	4571.79929	DCAF12
1862.92453	1804.70941	1781.95329	1627.6737	1622.57935	1655.04243	DCAF16
2552.41614	2618.61352	2668.75813	3078.52185	3247.01203	3188.51075	DCAF6
16656.5609	16477.9885	16719.3704	20453.5542	21337.2428	21659.6015	DCAF7
917.991732	893.231998	902.300091	1085.1158	1040.6377	1066.5829	DCBLD1
1872.9027	1819.78171	1783.14523	2576.60088	2638.19727	2726.73348	DCLK1
629.62259	629.070137	636.497026	851.178891	796.926468	860.213409	DCP1B
4300.5917	4098.0786	4145.57426	4890.70916	4626.80681	4220.35819	DCP2
146.679114	109.472483	133.497504	51.6198811	66.7194252	58.2329742	DCST2
1923.79137	1908.62894	1892.80389	2303.12534	2046.06237	2107.62502	DCTD
8204.0522	8315.9423	8568.87101	9481.5837	9711.383	10131.5159	DCTN1__RP1
4899.28196	5211.84212	4951.32705	5455.23297	5541.41893	5452.44532	DCTN4
6166.50968	6469.18911	6434.1029	7054.35099	6654.336	6798.95515	DCTPP1
3153.10204	3308.76613	3228.97087	3004.93606	2904.14831	2752.27426	DCUN1D3__I
2447.64535	2684.45567	2528.10897	2248.21057	2124.82836	2103.53849	DDAH1
16615.6504	16926.9843	16956.5668	18395.3487	19406.0861	20026.0134	DDB1
13310.8801	13641.2233	13656.0795	15978.0007	15351.0278	15731.0761	DDOST
18118.3629	18342.9871	19355.9461	20986.2274	20554.2163	21851.6682	DDR1
1163.45474	1059.02728	1081.09139	1421.19417	1377.94146	1435.39173	DDX10
2485.5624	2642.41189	2518.57344	2307.51852	2175.79459	2406.96294	DDX11

8240.97143	8413.5156	8365.04893	8928.04285	9255.46693	9339.75176	DDX23
1409.91556	1431.86834	1385.0366	1606.80609	1523.42688	1608.04739	DDX31
6964.76336	6971.33463	7263.69453	8881.91444	7777.44633	8562.29048	DDX54
763.330082	705.224908	682.982764	211.971001	232.59133	260.515937	DDX60
5974.9288	5676.70353	5876.27404	6910.4743	7152.87838	6621.19133	DECR2__NM
1071.65557	1043.1617	1199.09365	1471.71576	1375.16149	1494.64634	DEDD2
277.393154	303.032525	350.430947	208.676115	219.618108	185.936865	DENND6B
6175.49003	6070.96313	5668.87614	6456.87832	6693.25567	6309.59384	DENR
1698.28471	1735.69415	1646.0719	2174.62478	2030.30918	2055.52183	DEPDC1
1151.48093	1280.35208	1231.27608	1612.29756	1680.95885	1584.54988	DEPDC1B
2638.22841	2702.70108	2628.2321	3211.41558	3412.88393	3367.29619	DEPTOR
3648.01932	3498.35978	3388.6911	3871.49109	3852.12015	3747.34297	DFFA
4415.34067	4221.03682	4560.37008	5108.17164	5057.70309	5258.33541	DGCR2
10270.5314	10176.9745	11031.4232	13392.6134	12228.188	13279.1614	DHCR7
1684.31527	1599.25018	1674.67851	1404.71974	1271.37571	1284.19033	DHRS1
1922.79355	1909.42222	1889.22806	1263.03965	1244.50261	1171.8109	DHRS2
5797.31735	5662.42451	5802.37364	6319.59141	6282.74587	6547.63389	DHTKD1
4284.62663	4456.64064	4587.78474	5134.53073	4974.30381	5331.89285	DHX30
2697.09962	2670.96993	2891.65128	3465.12181	3349.87114	3418.37775	DHX37
2371.81125	2475.82333	2571.01889	3068.63719	3096.89332	3230.39762	DHX8
472.965306	391.086479	369.502019	294.343152	294.677461	277.883667	DICER1-AS1
10150.7934	10250.7494	10508.1606	10586.4688	11016.1184	11413.663	DIDO1
35.9214156	33.3177122	17.87913	1.09829534	2.77997605	5.10815564	DIO2
5205.61181	5156.3126	5156.34108	4295.43309	4637.00005	4712.78439	DIP2A
6211.41145	6022.57311	6169.49177	6488.72889	7117.66535	7127.92037	DIP2B
68.8493799	69.0152609	60.7890418	151.564757	142.705437	138.941833	DIRAS2
896.039756	909.890854	893.956497	1118.06466	1031.37111	1070.66942	DIRC2
247.458641	221.324802	216.933443	288.851675	300.237413	273.797142	DIXDC1
7192.26566	7444.92211	7527.11371	8184.4969	8115.67675	7978.9391	DLD
161.64637	167.38184	146.608866	95.5516949	83.3992815	89.9035392	DLGAP1-AS2
3389.58469	3673.67441	3481.66257	4395.37796	4483.17471	4483.93902	DLGAP5
3782.72463	3841.05625	3739.12204	3216.90706	3190.48585	3173.18628	DMTF1
5994.88514	6019.4	6151.61264	4811.6319	5463.5796	5643.49035	DMXL2
379.170498	404.572219	423.139409	226.248841	252.977821	230.888635	DNAAF1
4003.24221	4116.32401	4501.96492	4644.69101	4379.38894	4856.83438	DNAAF5
241.471738	252.262678	230.044805	158.154529	170.505198	182.871972	DNAH1
8949.42157	9061.62443	9199.40833	11328.9165	11069.8646	11010.1187	DNAJB1
3009.41638	2966.86294	2961.97586	3268.52694	3397.13073	3130.27777	DNAJB12
6141.56425	5947.21162	6103.93496	6772.08909	6852.64096	6456.70872	DNAJC14__R
2634.23715	2544.04531	2611.54491	3216.90706	3391.57078	3192.59727	DNAJC22
3931.39938	3879.92691	3818.98216	3155.40252	3253.49864	3205.87848	DNAJC3
374.181413	362.52844	332.551817	285.556789	308.577342	290.14324	DNAJC3-AS1
9116.05703	8852.99209	9512.88907	10224.0314	10157.1058	10461.5027	DNAJC5

604.677163	563.227992	522.070594	736.956175	764.493414	775.418025	DNAJC6
3492.35985	3634.01046	3563.90657	4028.54732	4109.73126	4133.51954	DNAJC7
2545.43142	2379.04331	2381.50011	2076.87649	2013.62932	2070.84629	DNASE1
2830.80711	2814.5534	2845.16555	4056.0047	3890.11315	3860.74403	DNASE2
70.8450141	80.121165	58.4051578	28.5556789	29.6530779	26.5624093	DNER
12351.9779	12332.3132	12578.5639	13648.5162	13164.1133	14330.4198	DNMT1__S1
4387.40179	4155.19468	4212.32302	5216.90288	5178.16872	5424.86128	DNMT3A
1191.39362	1123.28287	1221.74055	1429.98054	1505.82036	1528.36017	DNMT3B
7183.28531	7341.79586	7524.72982	8695.20423	8041.54405	8696.12415	DNPEP
1886.87214	1764.25219	1812.94378	2318.50147	2223.98084	2082.08424	DNPH1
2531.46198	2415.53413	2380.30817	2123.0049	2144.28819	2108.64665	DNTTIP2
2034.54907	2023.65438	2099.00986	1885.77311	1716.17188	1792.96263	DOC2A
2244.09066	2249.73885	2337.39826	2362.43328	2550.1647	2571.44555	DOCK7
422.076634	411.711729	468.433205	637.011299	720.013797	731.487887	DOCK8
253.445544	252.262678	270.570833	183.415322	163.091928	201.261332	DOK3
742.375923	802.798208	733.044328	1031.29933	1090.67727	1064.53963	DOK4
2306.95314	2313.20116	2574.59471	2206.47535	1890.38371	2031.00268	DOK7
4231.74232	4050.48187	4208.74719	4505.2075	4456.30161	4836.40176	DOPEY2
4006.23566	4435.22211	4507.92463	5494.7716	5296.78103	5687.42048	DPAGT1
3332.70912	3430.1378	3475.70286	3903.34165	3914.20628	4017.05359	DPF2
3401.5585	3589.58685	3643.76668	3344.30932	3210.87234	3180.3377	DPM2
1799.06423	1859.44565	1853.4698	2092.25263	2040.50242	1982.98602	DPY19L1
2827.81366	2914.50654	2869.00439	3383.84795	3618.60216	3648.24475	DPY19L3
441.035158	479.140432	466.049321	282.261903	350.276982	349.397845	DPYD
20.9541591	20.6252504	21.4549559	65.8977206	43.5529581	62.3194987	DPYSL3
1976.67568	2021.27454	1988.15925	2752.32813	2792.94927	2748.18773	DRAM1
1361.02253	1422.349	1442.24982	1249.8601	1194.46304	1263.7577	DRAM2
1905.83066	1911.00878	1874.92476	2336.0742	2249.92728	2171.98778	DRG2
710.445776	690.152609	665.103634	512.903925	509.662276	563.940382	DRICH1
7498.59551	7997.0442	7820.33144	9382.73712	10201.5854	10205.0733	DSCAM
59059.7964	60044.8636	57545.7677	51559.4749	53014.1433	51165.3301	DSCAM-AS1
57.8733918	38.0773853	38.1421439	95.5516949	151.972024	102.163113	DSCR8__KCN
988.836747	997.151529	1023.87818	1321.2493	1073.99741	1237.1953	DTX2
2706.07998	2724.11961	2843.9736	3603.50702	3260.91191	3523.60576	DTYMK
220.517579	228.464312	201.438197	264.689178	260.39109	301.381182	DUBR
2335.88983	2363.17773	2340.97408	2126.29979	2019.18927	1941.09914	DUSP1
3995.25967	4127.42992	4111.00795	5295.98015	5732.31061	5663.92297	DUSP16
330.27746	314.138429	311.096861	398.68121	416.996408	387.198197	DUSP18
4711.69235	4808.06318	4722.47419	4057.103	4022.62534	4118.19507	DUSP4
1984.65821	2007.7888	2135.96006	2482.14748	2453.79219	2555.09945	DVL2
4703.70981	4891.35746	4689.09981	4263.58252	4301.54961	4180.51457	DYNLT3
1460.80424	1404.10358	1556.67625	1690.27653	1519.72024	1742.9027	DYRK1B
2600.31136	2594.02188	2619.88851	2798.45654	2834.64891	2868.7402	DYRK2

69.847197	96.7800211	73.9004038	130.697146	161.238611	158.352825	DYRK3
1267.22772	1166.11993	1286.10541	1120.26125	1134.23023	1048.19354	E2F2
1320.11202	1366.81948	1388.61243	1571.66064	1680.03219	1771.50837	E2F8
3888.49324	3793.45951	3525.76443	4276.76207	4372.90233	4285.74258	EAF1
2358.83963	2388.56265	2170.52638	2066.99184	1967.29639	2060.62998	EBAG9
258.434629	217.358408	240.772283	162.547711	119.53897	133.833678	EBF4
132.709674	122.958224	158.528286	41.7352231	62.0861318	76.6223345	EBLN2
2171.25001	2091.08308	1955.97682	2468.96793	2339.81318	2285.38883	EBPL
1923.79137	2036.34684	2212.24435	2370.12135	2229.54079	2205.7016	ECD
2480.57331	2436.95266	2294.48834	1936.29469	2055.32896	1873.67149	ECHDC2
9190.89331	9524.89929	9486.66635	10308.6001	10228.4586	10243.8953	ECHS1
3072.27885	2927.99228	3265.92107	4058.20129	3482.38333	3918.977	ECI1
98.7838929	98.3665788	91.7795337	42.8335184	51.8928863	41.8868762	ECM1
6083.69086	6202.64742	5912.0323	6908.27771	6808.16135	6682.4892	ECT2
531.836515	543.39602	505.383407	722.678336	749.666875	662.01697	EDA2R
3771.74864	3708.57868	3858.31624	4141.67174	4163.47746	4268.37485	EDC3
5571.81069	5665.59763	6157.57235	6702.89648	6279.9659	6854.12323	EDC4__NRN1
501.902002	521.184212	591.20323	710.597087	653.294372	655.887184	EDN1
77367.7445	77228.077	83133.1865	95068.4449	87409.8603	95379.482	EEF1A2
15487.1192	15472.1109	14287.8087	16674.3199	19197.5879	16627.0466	EEF1B2
40.9105011	27.7647601	47.6776799	13.1795441	11.1199042	14.3028358	EEF1GP4
71525.5254	72322.4405	73161.3997	85048.6965	85754.8479	85177.4736	EEF1G__RP1
158118.089	160021.798	170612.194	208579.465	198662.649	213058.107	EEF2
944.932794	916.237085	927.330873	401.976096	423.483018	410.695713	EFEMP1
191.580883	198.319715	193.094603	45.0301091	48.1862515	46.9950318	EFEMP2
1336.0771	1333.50177	1399.3399	1126.85102	1036.93107	1148.31339	EFHC1
1401.93303	1376.33882	1362.3897	836.901052	855.305965	857.148516	EFHD1
2373.80688	2478.99644	2579.36248	2151.56058	1965.44307	2087.19239	EFNA1
705.45669	746.475408	687.750532	1013.7266	1055.46424	1088.03715	EFNB3
1536.63834	1523.09541	1536.41323	1137.83398	1210.21624	1214.71941	EFR3B
5521.91983	5602.9286	5797.60587	6170.22324	6140.96709	6515.96333	EFTUD2
2102.40063	2213.24802	2153.83919	1926.41003	1613.31277	1770.48674	EGLN1
281.384422	249.089562	271.762775	181.218732	219.618108	201.261332	EGLN1P1__S
10970.0012	10979.7727	11056.454	5774.83692	5740.65054	5789.5836	EGR1
3385.59342	3351.60319	3333.86176	2619.43439	2733.64312	2702.21433	EGR3
948.924062	954.31447	947.593887	1305.87316	1236.16268	1282.14706	EHD2
5998.87641	5784.58946	6226.70499	6702.89648	6420.81802	6738.67891	EHMT2
4182.84929	4216.27715	4318.40585	4829.20463	4705.57279	4737.30354	EI24
13274.9587	13267.589	12727.5566	12016.4494	11737.9855	11300.2619	EID1
27.9388788	18.2454138	23.8388399	12.0812488	6.48661078	7.15141789	EID3
14359.5859	14663.7598	14817.031	16172.3989	16060.8483	16189.7885	EIF2AK1
2991.45567	3058.88329	3027.53267	3277.31331	3247.01203	3325.40932	EIF2D
4380.41707	4452.67425	4672.41263	5343.20685	5206.89514	5380.93115	EIF3F

14402.492	15106.4094	14560.7634	16165.8092	16109.9612	15632.9995	EIF3H
8608.16813	8995.78229	9058.75917	10433.8058	10462.9032	10783.3165	EIF3L
43008.9105	43064.7295	42491.5402	47561.6799	48285.404	47611.0754	EIF4B
1100.59226	1181.19222	1103.73829	1032.39762	934.998611	915.38149	EIF4E3
1325.10111	1310.49668	1258.69075	1693.57142	1577.17308	1519.16549	EIF4EBP1
9245.77325	9427.32599	9200.60027	10957.6926	10904.9194	11264.5048	EIF4EBP2
46384.5257	47607.8376	46684.7922	51367.2732	52482.2412	53199.3977	EIF4G2
6789.14755	6919.77151	6696.33014	7196.03109	7723.70013	7952.37669	EIF4G3
5349.29748	5495.04267	5593.78379	6135.07779	6144.67373	6158.39243	ELAC2
4608.91719	4682.72512	4848.82004	5645.23807	5030.82999	5131.65315	ELAVL1
11093.7305	11656.4396	11360.3992	14992.8297	15439.987	15655.4754	ELF1
5526.90892	5602.13532	5569.94495	3992.30357	3873.4333	3860.74403	ELF3
2339.8811	2312.40788	2288.52863	2720.47757	2835.57557	2793.1395	ELK1
1680.324	1607.97625	1860.62146	2198.78728	1975.63631	2218.98281	ELMO3
2445.64971	2478.20316	2599.62549	3980.22232	3912.35296	3889.3497	ELOVL2
3752.79011	3820.431	3590.12929	2795.16165	2899.51502	2733.8849	ELOVL5
1213.34559	1181.9855	1195.51782	1431.07883	1314.92867	1367.96408	ELP6
1358.02907	1343.02111	1365.96553	1167.48795	1179.6365	1221.87083	EMC9
754.349728	737.749341	793.83337	662.272092	658.854324	672.233282	EME1
1712.25414	1700.78988	1904.72331	1444.25838	1327.90189	1475.23535	EME2
509.884538	523.564048	480.352625	283.360199	279.850922	307.510969	EMILIN3
2212.16051	2411.56774	2280.18504	2919.26902	2947.70127	3062.85012	EML1
2771.93591	2727.29273	3058.52316	3354.19398	3045.00043	3280.45755	EML3
1925.787	1853.8927	1772.41775	1634.26347	1566.05318	1602.93924	EML5
84743.6085	87288.4395	91020.2667	76499.5656	72545.3283	75689.5853	ENO1
2692.11054	2691.59518	2797.48787	1758.37085	1768.99143	1807.26546	ENO2__LRR(
4609.915	4566.9064	4652.14961	3655.1269	3500.91651	3600.22809	ENOSF1
1543.62305	1576.2451	1488.73555	1791.31971	1779.18467	1962.5534	ENOX2
2428.68682	2302.09526	2447.05692	2148.26569	2109.07516	2076.97608	ENTPD4__LC
495.915099	495.799288	531.60613	248.214748	221.471425	251.321257	ENTPD8
5091.86066	5178.52441	5248.12061	5247.65515	5948.22209	6238.07966	EP300
7442.71775	7473.48015	7761.92628	7674.88786	8511.36001	8667.51848	EP400
1584.53356	1530.23492	1551.90848	1042.28228	1079.55737	984.852406	EP400NL
523.853978	537.843068	537.56584	370.125531	353.056958	384.133304	EPAS1
2028.56217	1873.72467	1884.4603	2090.05604	2369.46625	2290.49699	EPB41
2533.45762	2591.64204	2567.44306	3283.90308	3352.65112	3355.03662	EPB41L2
2774.92936	2807.41389	2720.01164	2522.7844	2390.7794	2357.92464	EPB41L4B
1833.98783	1898.31632	1718.78036	2053.81229	2097.0286	2234.30728	EPC2
1709.26069	1795.19006	1768.84192	1879.18333	2069.22884	2193.44203	EPG5
10891.1737	10740.9958	11448.6029	13537.5884	12641.4778	13388.4759	EPHB4
6487.80679	6689.72064	7005.04311	8099.92816	7044.45931	8037.17208	EPN1
1945.74335	1957.81223	2001.27061	2113.12024	2201.74103	2367.11932	EPN2
8844.65078	9087.00936	9644.00269	10452.4768	9778.10243	10557.5361	EPN3__SPAT

9670.84334	9881.0815	9797.76321	10973.0688	11470.1812	11711.9792	EPRS
4847.39547	5008.76273	5164.68467	4362.4291	3886.40652	4308.21846	EPS8L2
2458.62134	2590.05548	2671.14201	2846.78153	2742.9097	2952.51396	ERAL1
52.8843063	43.6303374	47.6776799	27.4573836	25.0197845	23.4975159	ERAP2
1914.81102	1896.72976	1890.42001	2112.02195	2385.21945	2540.79661	ERC1
1141.50276	1010.63727	1063.21226	1303.67657	1335.31516	1286.23359	ERCC6L
8845.6486	8795.08273	8919.30196	9755.05924	9864.28168	10083.4992	ERGIC3
1175.42854	1190.71157	1253.92298	1064.24819	964.651689	878.602769	ERMARD
9733.70581	9854.11001	9591.55725	8169.12076	8430.7407	8586.80962	ERMP1
6474.83516	6330.36531	6205.25003	5308.0614	5215.23507	5078.52833	ERO1A
6474.83516	6536.61782	6329.212	7896.74352	8382.55445	7617.28168	ERP29
4825.4435	4991.3106	4758.23245	4185.60355	4662.01984	4598.3617	ERV3-1__ZN
1899.84376	1976.05764	1964.32041	2133.98785	2245.29399	2195.48529	ESCO1
2974.49278	3020.01263	3228.97087	4018.66266	3742.77442	4157.01706	ESPL1
2114.37444	2153.75211	2346.93379	1980.2265	1754.16489	2022.82963	ESPN
18964.5118	18922.874	18789.7736	20533.7297	22149.9225	21625.8877	ESR1
11780.2287	11665.1657	11666.7283	10589.7637	10736.2675	10859.9389	ESRP2
11752.2898	11797.6432	12385.4693	12762.1919	12822.1762	13626.516	ESYT1__ZC3I
510.882355	562.434713	486.312335	679.844818	739.473629	668.146757	ETHE1
1738.19739	1683.33774	1899.95554	1326.74078	1411.30118	1442.54315	ETS2
29753.9081	29775.7221	30355.1868	22498.5801	22194.4021	23050.0415	EVL
1158.46565	992.391855	1159.75956	862.161845	715.380504	740.682567	EXD3
3845.58711	3857.7151	3814.21439	4342.65979	4458.15493	4460.4415	EXOC4
296.351679	291.926621	339.703469	386.599961	376.223425	399.457771	EXTL2
987.838929	967.800211	1001.23128	1034.59421	1088.82395	1257.62792	EXTL3
67.8515628	59.4959146	47.6776799	32.9488603	33.3597126	34.7354583	EXTL3-AS1
466.978403	479.933711	498.231755	586.489713	589.354923	595.610947	EYA2
1474.77367	1508.81639	1442.24982	1675.99869	1716.17188	1797.04915	EYA3
9270.71868	9277.39628	9256.62154	8563.40879	8614.21912	8807.48195	EZR
12457.7465	12074.4976	12528.5023	13116.9413	13068.6674	13064.6189	F11R__RP11
640.598578	674.287032	772.378414	942.337405	842.332743	935.814112	F12
468.974037	456.928624	516.110884	530.476651	574.528384	592.546054	F2RL1
98.7838929	98.3665788	90.5875917	59.3079485	61.1594731	61.2978676	F3
9559.08782	9491.58157	9572.48617	10215.245	10264.5982	10310.3013	FAM102B
1170.43946	1081.23909	1113.27383	797.362419	898.858923	849.997098	FAM106A
31.9301472	53.1496837	36.9502019	15.3761348	22.2398084	16.346098	FAM106DP
283.380057	266.541697	245.540051	390.993142	384.563354	405.587557	FAM110B
3115.18499	3234.9912	3325.51817	2817.12756	2991.25423	2906.54056	FAM111A
464.982769	523.564048	486.312335	570.015283	589.354923	616.04357	FAM117A
2773.93154	2869.28964	2894.03517	3191.64627	3069.09356	3144.58061	FAM120B
1398.93958	1365.23292	1442.24982	1647.44302	1730.07176	1625.41512	FAM120C
128.718406	146.756589	113.23449	65.8977206	70.4260599	64.362761	FAM13A
589.709906	610.824723	541.141666	700.712429	687.580743	748.855616	FAM155B

2225.13213	2206.10851	2438.71333	1774.84528	1682.81217	1763.33533	FAM160B2
2268.03827	2266.39771	2159.7989	1591.42995	1745.82496	1633.58817	FAM162A
123.72932	103.919531	120.386142	84.5687414	82.4726228	67.4276544	FAM167A-AS
263.423715	282.407275	332.551817	236.133499	225.17806	204.326225	FAM169A
764.327899	851.188218	1010.76681	1236.68056	1010.98462	1168.74601	FAM171A2
157.655102	175.314628	159.720228	64.7994253	90.812551	96.0333259	FAM178B
1615.46589	1565.13919	1761.69027	1281.71067	1263.03579	1311.77437	FAM189A2
1490.73875	1488.19114	1649.64772	1914.32878	1796.79119	2109.66828	FAM189B
2485.5624	2500.41497	2665.1823	2260.29182	2155.4081	2312.97287	FAM193B
3080.26139	2956.55032	2879.73186	2604.05826	2581.67109	2629.67852	FAM217B
1320.11202	1314.46307	1282.52959	987.367514	1023.95785	1018.56623	FAM219A
1287.18406	1334.29505	1327.82338	1618.88734	1561.41988	1593.74456	FAM21A
1981.66476	1937.98026	1988.15925	2309.71511	2332.39991	2400.83315	FAM222B
340.255631	328.417449	325.400165	208.676115	231.664671	225.780479	FAM228A__I
193.576518	166.588561	178.7913	87.8636275	101.005797	91.9468014	FAM229A
1810.04022	1879.27762	1783.14523	1935.1964	2064.59555	2060.62998	FAM234B
4133.95625	4450.29441	4288.6073	5006.03018	5122.5692	5170.47513	FAM32A
322.294923	334.763679	387.381149	744.644243	541.168671	584.373005	FAM43A
2367.81998	2297.33558	2253.96232	1670.50722	1692.07876	1709.18888	FAM46A
713.439227	771.860332	748.539574	540.361309	497.615713	468.928687	FAM46C
2810.85077	2848.66439	2920.25789	3703.4519	3610.26223	3715.67241	FAM53C
2815.83986	2634.4791	2669.95007	3371.7667	3287.78501	3078.17459	FAM60A
847.146718	837.702477	867.733773	1232.28738	1216.70285	1246.38998	FAM64A
2131.33733	2225.14721	2284.95281	2576.60088	2433.4057	2556.12108	FAM65A
62.8624773	76.9480495	69.1326358	41.7352231	37.0663473	40.8652451	FAM71F2
1233.30194	1265.27978	1299.21678	894.01241	810.826348	950.116948	FAM83A
40.9105011	38.0773853	52.4454478	25.2607929	23.1664671	25.5407782	FAM83A-AS1
1976.67568	1875.31123	1957.16876	2666.66109	2462.13212	2619.46221	FAM83D
2082.44429	2227.52704	2394.61147	2507.40827	2514.02501	2573.48881	FAM83G
241.471738	260.195467	263.419181	158.154529	153.825341	156.309562	FAM86B3P
2183.22382	2187.06982	2295.68029	1911.0339	1870.92388	2044.28389	FARP1
2137.32423	2116.468	2113.31316	2246.01398	2340.73983	2390.61684	FARP2
45890.6063	44588.6182	49335.6712	53508.9491	51679.7548	58831.6501	FASN
3658.99531	3573.72127	3723.6268	3416.79681	3040.36714	3152.75366	FASTK
10153.7868	9910.43281	9909.80576	10653.4648	11075.4246	10611.6825	FBL
6949.7961	6985.61365	7139.73256	7960.44465	7598.6012	7865.53805	FBRS
515.871441	500.558961	528.030304	677.648227	616.228024	676.319806	FBXL14
911.007013	844.048708	907.067859	987.367514	1032.29777	1095.18857	FBXL18
4747.61376	4770.77907	5128.92641	5926.40167	5482.11277	5904.00628	FBXL19__OR
868.100877	794.865419	839.127166	686.43459	720.940456	679.384699	FBXL20
2951.54298	2904.19391	3130.03968	1935.1964	1629.06597	1709.18888	FBXL8__HSF
3913.43867	3915.62446	4029.95589	4548.04102	4522.09437	4588.14539	FBXO21
923.978635	958.280864	915.411453	1523.33564	1377.94146	1399.63464	FBXO27

337.26218	367.288113	333.743759	228.445431	244.637892	228.845372	FBXO32
1112.56607	1039.98859	1071.55586	1346.51009	1324.19526	1534.48995	FBXO41
785.282058	819.457064	781.91395	627.126641	680.167474	670.190019	FBXO44
4114.99772	4297.19159	4292.18313	4539.25465	4516.53442	4572.82092	FBXO7
4588.96085	4785.85137	4568.71367	5233.37731	5384.81361	5272.63825	FBXW11
5298.4088	5348.28608	5268.38362	4935.73927	4905.73107	4788.38509	FBXW2
718.428312	704.431629	731.852386	923.666384	817.312959	929.684326	FBXW9
99.78171	116.611993	120.386142	69.1926066	85.2525989	52.1031875	FCGR2A__FC
1261.24082	1325.56898	1298.02483	1571.66064	1472.46065	1667.302	FCHO1
1655.37857	1613.5292	1774.80163	1489.28849	1377.94146	1420.06727	FCHSD1
1125.53769	1128.04254	1244.38744	1692.47312	1384.42807	1554.92258	FDXR
2042.5316	2044.27963	2186.02162	721.580041	666.267593	730.466256	FER1L4
33.9257814	20.6252504	29.7985499	12.0812488	11.1199042	15.3244669	FGD2
12726.1593	12855.8772	12514.199	14309.69	14277.0303	14109.7475	FGD5-AS1
561.771028	498.179125	518.494768	714.990269	656.074348	618.086832	FGF12
991.830198	1000.32464	1038.18148	693.024362	663.487617	752.942141	FGFR1
406.11156	414.884844	415.987757	462.38234	504.102324	511.837195	FGGY
1174.43073	1161.36025	1168.10316	1880.28163	1884.82376	1769.46511	FHL1
431.056987	409.331892	456.513785	246.018157	246.49121	253.36452	FHL2
3319.73749	3522.95142	3648.53445	4013.17119	3897.52642	3780.03517	FIBP
410.102828	399.019267	424.331351	530.476651	485.56915	526.14003	FJX1
138.696577	138.030522	151.376634	218.560773	224.251401	248.256364	FKBP10
20592.9493	20987.7788	21106.9089	26332.7292	27548.636	26928.1532	FKBP4
10801.3701	10741.7891	11437.8754	12968.6714	11537.8273	12310.6551	FKBP8
2852.75909	2876.42915	2827.28642	3349.8008	3290.56498	3270.24124	FLCN__PLD6
107.764247	88.0539536	90.5875917	41.7352231	40.7729821	42.9085073	FLJ31356
744.371557	687.772773	721.124908	574.408465	544.875306	571.0918	FLNB-AS1
4302.58734	4524.86262	4631.8866	5085.10744	5066.04302	5196.01591	FLOT2
2713.0647	2691.59518	2739.08271	2924.7605	3466.63013	3433.70222	FMN1
5554.8478	5360.18527	5313.67742	4889.61087	5007.66352	4779.19041	FMR1
6420.95304	6596.11373	6488.93223	5133.43244	5562.73208	5391.14746	FNBP4
3393.57596	3526.12454	3517.42083	3730.90928	4099.53802	4012.96707	FNDC3A
634.611676	596.545704	593.587114	979.679446	981.331546	1000.17687	FNIP2
2224.13432	2253.70525	2261.11397	2377.80942	2541.82477	2766.57709	FOCAD
20921.2311	21788.1972	21855.4485	13383.8271	12434.8329	13135.1114	FOS
2825.81803	2950.99736	2793.91204	704.007315	722.793773	727.401362	FOSB
7694.16766	7770.16645	7999.12274	5431.07047	5686.90434	5657.79318	FOSL2
1363.01816	1373.95899	1505.42274	1276.21919	1189.82975	1245.36834	FOXC1
1235.29757	1253.3806	1300.40872	791.870943	792.293174	734.55278	FOXH1
124.727138	114.232156	89.3956497	34.0471557	40.7729821	39.843614	FOXI1
1481.75839	1492.15754	1455.36118	1636.46006	1619.79938	1676.49668	FOXJ2
5322.35641	5391.91642	5509.15591	6321.788	6261.43272	6591.56403	FOXK2
5163.70349	5287.20361	5353.01151	6434.91242	6661.74927	6900.09663	FOXM1

4456.25117	4558.18034	4934.63987	5308.0614	4861.25145	5470.83469	FOXP4
1583.53574	1550.0669	1495.88721	1239.97544	1118.47703	1129.92403	FRA10AC1
2111.38098	2099.01587	2068.01936	2199.88557	2669.70367	2783.94482	FRAS1
496.912916	485.486663	470.817089	700.712429	686.654084	666.103495	FRAT1
2027.56435	1903.07599	2050.14023	2856.66619	2447.30558	2725.71185	FRAT2
6572.62124	6473.94879	6258.88742	6797.34988	7888.64537	7894.14372	FREM2
2812.84641	2801.86094	2766.49737	3112.569	3125.61974	3202.81358	FRS2
132.709674	116.611993	129.921678	74.6840834	86.1792575	79.6872279	FRS3
256.438995	283.200554	214.549559	174.62896	143.632096	115.444317	FSD2
295.353862	317.311545	268.186949	181.218732	186.258395	199.21807	FSTL4
114.748967	127.717897	97.7392437	51.6198811	61.1594731	71.5141789	FTCD
106974.974	107006.972	106279.508	99994.2995	103353.95	101000.497	FTH1
26315.4304	27243.5759	27536.244	32593.0126	33942.5809	35550.72	FTL
13886.6206	13473.0482	13509.4706	14555.7082	14954.4178	14680.8393	FTSJ3
643.59203	729.023274	703.245778	831.409575	877.545773	771.331501	FUT10__TTI2
914.998281	805.178044	766.418704	732.562994	714.453845	672.233282	FUT11
7987.52589	8299.28345	7868.00912	8611.73379	8870.90358	8382.4834	FXR1
498.90855	510.871587	536.373898	796.264124	727.427066	791.764123	FZD2
5132.77116	5190.42359	5071.7132	5793.50794	5937.10218	5613.86304	G3BP2
1894.85467	1972.09125	2104.96957	2817.12756	2561.2846	2865.67531	G6PC3
785.282058	769.480495	756.883168	341.569852	392.903282	404.565926	GABBR1__U
5830.24532	5703.67501	5645.0373	3812.18314	3631.57538	3769.81886	GABPB1-AS1
5441.09665	5419.68118	5772.57509	6149.35563	5751.77045	6350.45909	GAK
359.214156	350.629257	339.703469	211.971001	197.3783	215.564168	GAL
3256.87502	3357.94942	3282.60826	7277.30495	7425.31603	7270.94873	GALC
151.668199	174.52135	166.87188	314.112468	367.883497	307.510969	GALM
124.727138	112.645598	153.760518	159.252825	187.185054	205.347857	GALNT14
379.170498	348.24942	337.319585	421.745412	539.315354	480.16663	GALNT16
6177.48567	6569.14225	6233.85664	6708.38796	6916.58041	6782.60905	GALNT4__PC
5142.74934	5056.35946	5089.59233	4206.47117	4272.82319	4402.20853	GALNT6
3591.14374	3588.79357	3586.55347	3884.67063	4042.08518	4088.56777	GALNT7
443.030793	413.298287	489.888161	327.292012	361.396887	364.722312	GALT__IL11R
4535.07872	4199.61829	3969.16685	3682.58429	3555.58937	3185.44585	GAN
20117.9884	20806.118	21054.4634	22762.171	23920.7673	23935.7957	GANAB
119160.316	123088.321	123377.916	100329.28	100477.601	100482.53	GAPDH
57.8733918	46.8034528	40.5260279	65.8977206	76.9126707	86.8386458	GAREML
5612.72119	5847.25849	5741.5846	7801.19182	7799.68614	8028.99903	GARS
4187.83837	4331.30258	4333.9011	4706.19555	4868.66472	4902.80778	GART
1215.34123	1204.99059	1252.73104	1544.20325	1277.86232	1528.36017	GAS2L1
1267.22772	1308.11684	1301.60066	1437.6686	1454.85413	1465.01904	GAS2L3
2405.73703	2405.22151	2514.99761	1962.65378	1960.80977	1908.40695	GAS8
45.8995866	45.2168951	83.4359398	15.3761348	37.993006	31.6705649	GAS8-AS1
2835.7962	2706.66747	2841.58972	3817.67461	3940.15272	3928.17168	GATA3-AS1_

388.150852	428.370585	472.009031	593.079485	523.562156	554.745702	GATA6
2151.29367	2138.67981	2329.05466	2831.4054	2940.288	3166.03486	GBA
1468.78677	1548.48034	1581.70703	1168.58625	1225.04278	1281.12543	GBE1
5498.97004	5475.2107	5703.44245	5923.10679	6191.00666	6513.92007	GBF1
2650.20222	2869.28964	2754.57795	3315.75364	3103.37993	3165.01323	GCA
354.225071	360.941882	418.371641	542.5579	504.102324	510.815564	GCAT
769.316984	779.793121	758.07511	623.831755	659.780983	667.125126	GCFC2
2261.05355	2214.0413	2352.8935	2071.38502	1942.2766	1946.2073	GCH1
1345.05745	1436.62802	1513.76634	1199.33852	1028.59114	1037.97723	GCLM
12290.1132	11795.2634	12600.0188	13035.6674	13527.3635	14533.7244	GCN1
2054.50541	2150.57899	2085.89849	2426.13441	2243.44067	2291.51862	GCOM1__MY
1188.40017	1158.98042	1132.3449	1299.28339	1315.85533	1315.86089	GDAP1
1721.2345	1857.06581	1733.08366	2311.9117	2453.79219	2469.28243	GDF11
341.253448	333.177122	337.319585	195.496571	211.27818	180.82871	GDPD3
2533.45762	2525.00662	2767.68932	2980.77356	2882.83516	3083.28274	GEMIN4
3601.12192	3564.20192	3549.60327	3654.02861	3927.1795	4085.50288	GEMIN5
2400.74794	2489.30907	2566.25112	2763.31108	2708.62333	2676.67355	GFM2
1552.60341	1520.71558	1507.80663	1819.87538	1800.49782	1746.98923	GFOD2
4481.1966	4382.07243	4342.24469	4962.09836	5219.86836	5056.05245	GFPT1
44817.9529	44207.0511	44650.1472	48612.7485	48346.5635	48446.7697	GFRA1
60.8668431	68.2219821	81.0520558	151.564757	144.558755	186.958496	GFRA3
680.511262	662.387849	684.174706	836.901052	805.266396	854.083622	GID4
13628.186	13624.5644	13725.2121	15361.857	14887.6984	14659.385	GID8
6019.83057	5840.91226	6174.25954	5207.01822	5118.86257	5486.15915	GIGYF1
163.642005	202.28611	187.134893	68.0943113	92.6658683	99.0982193	GIPR
3822.63731	3838.67641	4091.93687	4605.15238	4229.27023	4592.23192	GIT1
1607.48335	1780.91104	1772.41775	1920.91856	1879.26381	1957.44524	GIT2
3481.38386	3689.53998	3638.99892	4095.54334	3973.51243	3768.79723	GJA9__MYCE
407.109377	402.192383	400.492511	495.3312	488.349126	492.426203	GJB3
370.190144	340.316632	359.966483	562.327216	501.322348	541.464497	GJD3
1662.36329	1571.48542	1592.43451	1215.81295	1280.6423	1315.86089	GK5
5058.9327	5196.76982	5217.13012	6577.69081	6884.14736	6480.20624	GLA
202.556871	217.358408	239.580341	142.778395	141.778779	153.244669	GLB1L
540.816868	555.295203	505.383407	282.261903	311.357318	266.645724	GLDN
965.886953	925.756431	946.401945	1034.59421	1127.74362	1074.75595	GLI3
82.8188193	84.0875593	76.2842878	35.145451	42.6262994	32.6921961	GLIPR1
1077.64247	1155.8073	1076.32362	1629.87029	1492.84714	1554.92258	GLMP
914.998281	970.973326	871.309599	1035.69251	1175.00321	1210.63289	GLRA3
419.083182	442.649605	441.018539	551.344262	573.601725	525.118399	GLS2
1573.55757	1502.47016	1698.51735	1796.81118	1699.49203	1779.68142	GLTSCR1
13265.9784	13332.6378	14268.7376	16911.5517	14669.9336	15745.3789	GLTSCR2
6467.85045	6686.54752	6627.1975	7613.38332	7288.17054	7255.62426	GLUD1
218.521945	226.084475	224.085095	360.240873	353.056958	364.722312	GLUD2

5144.74497	4974.65174	5113.43117	5535.40853	5705.43751	5533.15418	GLYR1
2497.5362	2344.93231	2541.22034	2809.43949	2640.97725	2859.54552	GMEB2
2026.56653	1901.48943	2094.24209	2586.48553	2426.91909	2572.46718	GMIP
5612.72119	5460.93168	5805.94947	7643.0373	7540.22171	7973.83095	GNA12
1560.58595	1699.20332	1712.82065	2679.84064	2596.49763	2826.85333	GNA14
111.755515	109.472483	90.5875917	8.78636275	11.1199042	5.10815564	GNAI1
54.8799405	41.2505008	42.9099119	30.7522696	29.6530779	16.346098	GNAL
118616.506	118957.718	121746.148	130977.211	129241.087	130651.297	GNAS
17846.9567	18019.3293	18227.177	19592.4906	19324.5402	19598.9715	GNB1
20674.7703	21581.9447	23204.7268	27651.7819	23895.7475	26831.0983	GNB2
72477.4429	74596.771	74907.5948	79150.8505	77960.7217	78878.0961	GNB2L1
2315.93349	2321.13395	2426.79391	2713.88779	2803.14252	2666.45724	GNPDA1
4325.53713	4507.41049	4485.27773	4902.79041	4927.04422	4849.68296	GNPTAB
8682.00659	8573.75793	8862.08874	7895.64522	7988.72451	7901.29514	GOLGA2
306.32985	276.061044	253.883645	182.317027	176.06515	182.871972	GOLGA6L10
89.803539	85.674117	96.5473017	41.7352231	44.4796168	51.0815564	GOLGA6L4
132.709674	163.415445	110.850606	48.3249951	43.5529581	60.2762365	GOLGA6L5P_
1065.66866	1162.94681	1098.97052	664.468683	743.180264	683.471224	GOLGA6L9
1331.08801	1223.236	1281.33765	741.349357	635.687857	696.752429	GOLGA8A
504.895453	491.039615	487.504277	293.244857	272.437653	293.208133	GOLGA8B
326.286192	388.706642	342.087353	170.235778	167.725222	156.309562	GOLGA8N
45.8995866	53.1496837	57.2132158	30.7522696	20.386491	25.5407782	GOLGA8R
22.9497933	28.558039	29.7985499	9.88465809	13.8998803	13.2812047	GOLGA8S
1417.8981	1412.82965	1330.20727	1770.45209	1737.48503	1866.52007	GOLIM4
966.88477	910.684133	924.946989	591.98119	588.428264	526.14003	GOLM1
2705.08216	2713.01371	2836.82195	3317.95023	3188.63253	3029.13629	GOLPH3L
2893.66959	2828.83242	2826.09447	3234.47979	3050.56039	3072.0448	GORASP1
6138.5708	6205.82053	6335.17171	6850.06806	6999.97969	6951.17819	GORASP2
1401.93303	1366.81948	1317.09591	1612.29756	1667.05897	1618.26371	GOSR2__RP1
2269.03609	2321.13395	2191.98133	2443.70714	2453.79219	2525.47215	GOT1
6619.51864	6933.25725	6925.183	7671.59297	7552.26827	7849.19195	GOT2
437.04389	439.476489	446.978249	351.45451	325.257198	364.722312	GPAT3
2397.75449	2421.08708	2308.79165	3059.85083	3251.64532	3019.94161	GPATCH2
5027.00255	5074.60488	5058.60183	5816.57214	5744.35718	5684.35559	GPBP1L1
3918.42775	3946.56234	4201.59554	3255.3474	2800.36254	3049.56891	GPC1
928.96772	1011.43055	895.148439	660.075501	576.381701	667.125126	GPCPD1
2869.72198	2985.90163	3051.37151	4325.08706	4166.25744	4596.31844	GPER1
29381.7223	29459.9971	30898.7124	25349.7548	24535.142	25411.031	GPI__PDCD2I
2936.57573	3172.32217	3163.41406	3387.14284	3474.97006	3402.03165	GPN1__ZNF5
1172.43509	1176.43255	1151.41597	1457.43792	1280.6423	1259.67118	GPN3
195.572152	183.247417	207.397907	382.20678	373.443449	372.895361	GPNMB
1185.40672	1251.00076	1191.942	1517.84417	1427.98103	1546.74953	GPR137B
186.591798	176.107907	153.760518	129.598851	127.878898	120.552473	GPR155

466.978403	420.437796	492.272045	49.4232905	50.0395689	42.9085073	GPR179
6843.02967	6614.35915	6544.9535	7254.24074	7524.46851	7523.29162	GPR21__RAB
1236.29539	1244.65453	1298.02483	1034.59421	1048.97763	1027.76091	GPR37L1
516.869258	489.453057	493.463987	672.15675	626.42127	647.714135	GPR68
12902.7729	12318.0342	12480.8246	15499.1439	14917.3515	14822.846	GPRC5A
1226.31722	1258.93355	1249.15521	1438.7669	1437.24762	1600.89598	GPRIN1
1491.73657	1577.03838	1530.45352	1978.02991	1944.12992	2084.1275	GPT2
1582.53792	1691.27053	1601.97004	2292.14238	2192.47445	2300.7133	GPX3
7711.13055	7695.59823	7711.86472	9647.4263	9277.70674	9151.77164	GPX4
604.677163	646.522272	526.838362	967.598198	963.725031	927.641063	GPX8
16219.517	16062.3104	16208.0273	18453.5584	18449.7744	18589.6	GRB2
849.142352	915.443806	898.724265	708.400497	758.006803	794.829017	GREB1L
11305.2678	11284.3918	11566.6051	12639.1828	13412.4578	13669.4245	GRHL2
669.535274	537.843068	597.16294	510.707335	504.102324	513.880457	GRIN1
178.609261	136.443964	168.063822	128.500555	89.8858923	105.228006	GRIN2C
507.888904	581.473405	536.373898	644.699367	631.054563	665.081864	GRIP1
3824.63295	3619.73144	3708.13155	3342.11273	3386.01083	3546.08164	GRIPAP1
3832.61548	3900.55216	4192.06	4841.28587	4270.96987	4865.00743	GRK6
12887.8057	13079.5819	14212.7164	17382.7204	16074.7482	17376.9238	GRN
14.9672565	14.2790195	20.2630139	31.850565	29.6530779	39.843614	GRPR
177.611444	180.074302	163.296054	127.40226	126.95224	120.552473	GS1-114I9.1
1782.10134	1770.59842	1818.90349	2200.98387	2186.91449	2451.91471	GS1-114I9.3_
411.100645	421.231075	410.028047	338.274966	245.564551	299.33792	GS1-124K5.1
1349.04872	1249.41421	1330.20727	1187.25727	899.785581	900.057023	GS1-358P8.4
325.288375	301.445967	271.762775	186.710208	192.745006	167.547505	GSDMB
6559.64962	6594.52717	6273.19073	5487.08354	5780.49687	5524.98113	GSTM3
3702.89926	3541.99012	3448.2882	4179.01378	3928.10616	3801.48942	GTF2A1
4849.39111	5005.58961	4683.1401	4828.10633	5743.43052	5803.88643	GTF2I
360.211973	406.158777	429.099119	292.146561	282.630898	352.462739	GTF2IP13
387.153035	492.626173	537.56584	404.172686	349.350324	374.938624	GTF2IP20
411.100645	371.254507	365.926193	242.723271	253.904479	218.629061	GTF2IRD2
9162.95443	8959.29146	9415.14983	10633.6955	10969.7855	11435.1172	GTF3C1
2745.99266	2787.58192	2780.80068	2504.11338	2279.58036	2544.88314	GTPBP2
1214.34341	1204.99059	1257.49881	1156.505	961.871713	1058.40985	GTPBP3
1168.44383	1132.00894	1156.18374	1498.07485	1490.99382	1489.53818	GTSE1
42.9061353	38.8706642	38.1421439	17.5727255	19.4598324	25.5407782	GUSBP3
1630.43314	1481.84491	1576.93926	834.704461	866.425869	810.153484	H19__MIR67
13810.7865	13630.9107	13325.9115	11182.8432	12192.0483	11789.6232	H1F0
8238.9758	8369.88527	9462.82751	12801.7305	10774.2605	12175.7998	H2AFX
15622.8223	15841.7789	16223.5225	14588.6571	14661.5937	15010.8262	H2AFY
2114.37444	2192.62277	2157.41501	2474.45941	2576.11114	2563.2725	H2AFY2
19610.0995	19167.9971	18897.0484	21355.2547	21321.4896	19860.5091	H2AFZ
13902.5857	13935.5298	13590.5226	11510.1352	11374.7353	11132.7144	HACD2

10560.8962	10959.9408	10846.6722	11672.6829	11539.6806	11892.808	HADHA
5422.13812	5763.17093	5766.61538	6620.52433	6198.41993	6215.60378	HADHB
1443.84134	1481.05163	1504.2308	1278.41578	1163.88331	1330.16373	HAUS5
1480.76058	1458.04655	1448.20953	1628.77199	1642.96585	1627.45839	HAUS7__TRE
3339.69384	3461.86895	3383.92333	3738.59735	3854.90012	3869.93871	HAX1
2229.1234	2073.63094	2205.09269	2459.08327	2604.83756	2573.48881	HBP1
9026.25349	8452.38627	9117.16433	9391.52348	9452.84523	10050.807	HCFC1
1898.84594	1941.15337	1967.89624	1670.50722	1645.74582	1606.00413	HCFC1R1
341.253448	422.024354	381.421439	510.707335	488.349126	504.685777	HCK
1828.99875	1916.56173	2146.68754	2197.68898	2045.13571	2373.24911	HCN2
661.552738	729.023274	744.963748	495.3312	462.402683	487.318048	HCN3
1329.09238	1323.18914	1458.937	1131.2442	1041.56436	1064.53963	HDAC10__M/
7728.09344	8115.24275	7955.02088	8611.73379	8330.66156	8439.69474	HDAC2
1087.62064	1062.2004	1175.25481	1522.23735	1371.45485	1367.96408	HDAC5
1960.7106	2037.14012	2112.12122	1666.11404	1627.21265	1741.88107	HDAC6
25743.6812	26008.4407	25127.3292	27051.0143	28453.9815	27567.6943	HDGF
36815.4597	37020.7379	37848.9262	42032.8611	43135.9617	44642.2154	HDLBP
3710.8818	3809.32509	3588.93735	3654.02861	3327.63133	3206.90011	HELLS
7092.48395	7106.19204	7118.2776	7645.23389	8177.76288	7755.20188	HELZ
1033.73852	986.045625	1032.22177	695.220952	737.620312	734.55278	HEMK1
2594.32446	2793.13487	2685.44532	3245.46274	3042.22046	3096.56395	HENMT1
607.670614	574.333896	611.466244	497.527791	454.062755	456.669114	HERC2P9
5941.00302	6003.53442	6256.50354	5406.90798	4569.35397	4918.13225	HES1
1652.38512	1630.18806	1902.33943	1652.93449	1383.50141	1500.77613	HES4
52.8843063	59.4959146	70.3245778	24.1624976	25.9464431	28.6056716	HGFAC
78.8275509	79.3278861	70.3245778	43.9318137	50.9662276	33.7138272	HHIPL2
6138.5708	6312.91318	6387.61716	5941.77781	5651.69131	5925.46054	HID1
4879.32562	4679.552	4691.4837	6520.57945	6882.29404	6903.16152	HIP1
2311.94222	2490.10235	2298.06417	2955.51277	2706.77001	2818.68028	HIST1H2AC
149.672565	136.443964	151.376634	177.923846	213.131497	244.169839	HIST1H2AD_
452.011147	450.582393	477.968741	622.73346	612.52139	594.589316	HIST1H2AG
75.8340996	86.4673959	84.6278818	139.483509	114.905677	136.898571	HIST1H2AI
31.9301472	36.4908276	42.9099119	54.9147672	64.8661078	65.3843921	HIST1H2APS/
835.172913	816.283948	804.560848	1010.43172	1137.0102	1101.31836	HIST1H2BG
251.449909	250.67612	218.125385	282.261903	318.770587	298.316289	HIST1H2BH
146.679114	160.24233	150.184692	241.624976	260.39109	257.451044	HIST1H2BJ
15.9650736	11.8991829	14.303304	28.5556789	42.6262994	25.5407782	HIST1H3C
683.504714	698.878677	786.681718	997.252172	961.871713	957.268366	HIST1H3E
307.327667	299.85941	283.682195	386.599961	401.24321	459.734007	HIST1H3G
869.098694	856.74117	955.937481	1391.5402	1328.82855	1358.7694	HIST1H3H
4923.22957	5238.02032	5204.01876	5656.22102	5710.07081	5516.80809	HIST2H2AA3_
972.871673	961.45398	979.776321	1196.04363	1151.83674	1170.78927	HIST3H2A
1419.89373	1380.30522	1407.6835	1455.24133	1615.16609	1621.3286	HIVEP1

643.59203	674.287032	710.39743	901.700477	967.431665	1006.30666	HIVEP3
3532.27254	3392.06041	3360.08449	2527.17759	2589.08436	2516.27747	HK2
4472.21624	4746.98071	4870.275	4403.06603	4079.15152	4219.33655	HLA-A
2415.7152	2440.91906	2424.41002	1983.52139	1931.1567	1961.53176	HLA-B
961.895685	997.151529	961.897191	1175.17602	1128.67028	1148.31339	HLA-DQB1
1346.05527	1369.19931	1394.57214	1735.30664	1719.87852	1764.35696	HLA-DRB5
437.04389	438.68321	457.705727	390.993142	330.81715	382.090042	HLA-F
214.530677	248.296284	225.277037	161.449416	152.898683	159.374456	HLA-L
3014.40546	3008.90672	2864.23662	1067.54307	1194.46304	1196.33005	HMCN1
1585.53137	1587.351	1535.22129	1778.14016	1996.94946	1872.64986	HMG20A
11148.6105	11242.348	10216.1349	11629.8494	12305.1007	11479.0473	HMGB2
5591.76703	5682.25648	5645.0373	6060.39371	6103.90075	6198.23605	HMGCR
34433.6703	35289.8034	34979.9218	38105.3569	37690.9153	36815.4993	HMGN2
2685.12582	2606.71434	2541.22034	3131.24002	3035.73385	3087.36927	HMMR
10205.6733	9579.63553	9453.29197	10798.4398	10702.9078	10443.1134	HNRNPA0
28952.661	27756.8274	25686.35	23532.076	25910.3034	22813.0231	HNRNPA3
34063.4802	35324.7077	34245.6855	36878.561	37082.1005	35757.0894	HNRNPC
13574.3038	12991.5279	12288.922	9789.1064	10254.405	9361.20602	HNRNPD
10970.0012	11014.677	10330.5613	8289.93325	8813.45074	8124.01072	HNRNPDL
21961.9544	22423.6136	22612.3316	24962.0566	24555.5285	24884.891	HNRNPF
33763.1372	33067.8293	32201.505	25769.3036	26476.4919	24450.6978	HNRNPH1
2606.29827	2822.48619	2599.62549	3066.4406	2866.15531	2983.16289	HNRNPLL
15395.32	15174.6313	15105.4809	17548.563	17097.7794	17217.5494	HNRNPM
19554.2217	18755.4921	19476.3322	21733.0683	21699.5664	21429.7345	HNRNPUL1
3327.72003	3319.87203	3249.23388	3857.21325	4052.27842	3913.86885	HOMER2
2531.46198	2598.78155	2613.9288	2075.7782	2228.61413	2110.68991	HOMEZ__PP
321.297106	349.042699	333.743759	531.574946	498.542372	447.474434	HOXA10__H
488.930379	513.251423	495.847871	643.601071	637.541174	691.644273	HOXB7
612.6597	612.411281	630.537316	806.148782	770.053366	849.997098	HOXC10__H
909.011378	919.4102	998.847393	1245.46692	1256.54917	1264.77934	HOXC13
214.530677	184.833975	195.478487	294.343152	254.831138	259.494306	HOXC13-AS
109.759881	103.919531	109.658664	307.522696	269.657677	284.013453	HPN
4912.25359	5081.74439	4832.13285	4235.02684	4142.16431	3925.10679	HPRT1
1511.69291	1570.69215	1719.9723	2220.75318	1895.94367	2016.69985	HPS6
209.541591	186.420532	175.215474	291.048266	294.677461	306.489338	HPSE
2166.26093	2210.07491	2180.06191	2460.18157	2566.84455	2487.67179	HS2ST1__RP
530.838697	469.621086	468.433205	396.484619	386.416671	410.695713	HS3ST3B1
275.39752	285.58039	330.167933	149.368167	149.192048	148.136513	HS6ST2
152.666016	161.035609	190.710719	248.214748	227.958036	250.299626	HSD11B2
3687.932	3650.66932	3664.0297	4274.56548	4219.07699	4102.87061	HSD17B10
1273.21462	1270.83274	1239.61968	1022.51297	966.505007	987.9173	HSD17B12
425.070085	468.827807	455.321843	382.20678	329.890491	378.003517	HSD17B8
176518.834	181747.327	177484.931	192116.018	186425.194	185375.99	HSP90AA1

114451.617	117575.826	116720.92	130888.249	132299.987	132588.309	HSP90AB1
28640.3442	29320.38	28514.8284	31132.2798	30995.8063	31156.6845	HSP90B1
110.757698	128.511176	121.578084	83.4704461	72.2793773	65.3843921	HSPA6
90093.9038	95158.5591	94455.4435	106277.647	105248.04	107171.149	HSPA8
78081.1837	76099.2412	81953.1639	95618.6909	86539.7278	91023.2469	HSPB1
1476.76931	1574.65854	1587.66674	751.234015	733.913677	829.564475	HSPB8
40473.4572	41843.8734	41027.8355	44154.7677	44398.0708	43504.1183	HSPD1
328.281826	319.691381	300.369383	128.500555	175.138491	164.482611	HSPD1P11
16706.4517	17117.3713	16707.451	18720.4441	18639.7394	18589.6	HSPH1
8982.34954	8731.62043	9033.72839	9026.88943	10028.3003	10702.6077	HTT
10274.5227	10433.2036	10751.3168	11719.9096	11828.7981	11980.6682	HYOU1
12666.2903	12947.8976	12870.5897	13451.9214	13584.8163	14010.6493	IARS
2842.78092	2993.04114	2886.88352	3178.46672	3157.12613	3131.2994	ICA1
2865.73071	2851.83751	3026.34073	5365.17275	4625.88015	5076.48507	ID1
3921.4212	4021.92383	3969.16685	5201.52675	4958.55061	4958.99749	ID2
3830.61985	3743.48295	3963.20714	5012.61995	4530.4343	4471.67944	ID3
10229.6209	10479.2138	10595.1724	11730.8926	11491.4943	11898.9377	IDH2
345.244717	307.792198	294.409673	254.80452	250.197845	235.99679	IDNK
1391.95486	1377.1321	1567.40373	1176.27431	1095.31056	1218.80593	IDUA
4577.98486	4528.03574	5149.18943	5587.02841	4900.17112	5628.16588	IER5L
41844.4579	44947.1803	47926.7957	38265.7081	31851.1123	36041.1029	IFFO1
8697.97166	8697.50943	8741.7026	3365.17693	3256.27861	3299.86854	IFI27
206.54814	200.699552	202.630139	18.6710208	20.386491	20.4326225	IFI44
65.8559286	63.4623089	64.3648678	16.4744302	16.6798563	21.4542537	IFI44L
9490.23844	9128.25986	9318.60253	3618.88316	3353.57778	3277.39266	IFI6
3275.83354	3314.31908	3326.71011	3022.50879	3030.17389	2880.99978	IFRD1__LSM
931.961172	929.722825	926.138931	767.708445	797.853126	801.980435	IFT172
2065.4814	2176.7572	2187.21356	2668.85768	2548.31138	2599.02959	IFT46
295.353862	282.407275	352.814831	161.449416	139.925461	154.2663	IGDCC3
38890.9193	37580.7928	38237.4993	38886.2449	41417.9365	42298.5936	IGF1R
7138.38354	6879.31428	7182.64247	7964.83783	9134.07464	9212.04787	IGF2R
3349.67201	3338.91073	3696.21213	5673.79374	4768.58558	5302.26555	IGFBP2
605.67498	537.843068	554.253028	1131.2442	1183.34314	1183.04885	IGFBP3
16018.9557	15875.8899	16111.48	20271.2372	19274.5006	19857.4442	IGFBP4
42300.4603	42415.8274	42251.9599	89079.4404	95426.3845	95854.5405	IGFBP5
114.748967	97.5732999	116.810316	52.7181765	50.0395689	48.016663	IGFL1
42.9061353	46.010174	33.3743759	15.3761348	16.6798563	28.6056716	IGHE
22.9497933	22.2118081	13.111362	2.19659069	2.77997605	8.17304902	IGHG1
8739.87998	8975.95032	9131.46764	9778.12344	9916.17457	10209.1599	IGSF1
9316.61827	9194.89528	9385.35128	11381.6346	11937.2172	12237.0976	IGSF3
1041.72105	1055.06089	1141.88043	1361.88623	1264.8891	1359.79103	IGSF9
792.266778	785.346073	858.198237	967.598198	964.651689	929.684326	IKBIP
439.039524	403.77894	392.148917	285.556789	315.990611	278.905298	IL18BP

153.663834	134.064128	145.416924	203.184639	218.691449	205.347857	IL1R1
277.393154	280.027438	287.258021	415.15564	426.262994	382.090042	IL20
954.910965	993.185134	1036.98954	1327.83907	1260.25581	1282.14706	IL27RA
2512.50346	2565.46384	2640.15152	3096.09457	2879.12853	3046.50402	IL4R
1997.62984	2030.00061	1948.82516	1538.71178	1586.43967	1563.09562	IL6ST
1301.1535	1279.5588	1356.42999	1087.31239	1019.32455	1004.2634	IL9R
14122.1054	15034.221	14582.2184	15759.4399	15692.9648	15946.6403	ILF2
2697.09962	2721.73977	2894.03517	3242.16785	2877.27521	3185.44585	IMP3
2147.3024	2207.69507	2121.65675	1949.47424	2034.94247	1952.33708	IMPACT
11784.22	12208.5617	11963.5218	13207.0015	13375.3914	13040.0997	IMPDH2
4392.39088	4332.88914	4112.19989	3913.22631	4002.23885	3997.6426	INADL
2923.6041	2924.81916	2981.04693	3433.27124	3684.39493	3923.06353	INCENP
9683.81496	9742.2577	10434.2602	8129.58213	7238.13098	8139.33519	INF2
2083.44211	1991.92322	2143.11171	1880.28163	1782.89131	1884.90943	ING5
459.993683	480.72699	511.343117	366.830645	305.797366	350.419477	INHA
900.031025	920.996758	953.553597	471.168702	479.082539	474.036843	INHBB
2512.50346	2487.72251	2504.27013	2658.97303	2739.20307	2719.58206	INO80
261.42808	276.061044	286.066079	404.172686	329.890491	341.224796	INPP5J
5924.04013	6033.67902	6084.86389	7103.77428	6861.90755	6700.87856	INSIG1
524.851795	543.39602	539.949724	612.848802	650.514396	623.194987	INTS9
2126.34824	2193.41605	2162.18278	2611.74633	2695.65011	2694.04128	IP6K1
6208.418	6251.83071	6146.84488	6638.09706	6734.02865	6671.25126	IPO5
3102.21337	3116.79265	2983.43082	3384.94625	3577.82918	3633.94192	IPO8
3779.73118	3812.49821	3872.61955	4211.96264	4437.76843	4438.98725	IPO9
15872.2766	15867.9571	16708.6429	17980.1931	16703.0228	18106.3685	IRAK1
5226.56597	4950.85337	5224.28177	4850.07224	4465.5682	4666.81099	IRF3
8107.26394	8236.61442	8525.9611	6300.92039	5894.47588	6396.43249	IRF9__RNF3
19522.2916	19156.0979	19547.8487	20935.7058	21912.6979	22990.7869	IRS1
1641.40913	1644.46708	1808.17601	1311.36464	1206.50961	1331.18536	IRX2
1632.42878	1584.17789	1678.25433	1976.93162	1851.46405	1966.63992	IRX3
3932.39719	4048.89531	4060.94638	4434.9166	4463.71488	4496.19859	ISG20L2
46.8974037	28.558039	38.1421439	87.8636275	63.0127905	69.4709166	ISL2
3007.42074	3000.97393	3149.11076	3708.94338	3560.22266	3634.96355	ISOC2
2933.58228	2811.38028	3025.14879	4264.68082	3863.24005	4312.30499	ISYNA1
116.744601	138.823801	144.224982	73.585788	83.3992815	56.189712	ITGA2B
66.8537457	84.0875593	72.7084618	38.440337	47.2595928	41.8868762	ITGAM
8586.21615	8617.38827	8367.43282	6492.02378	6857.27426	7028.82215	ITGAV
4787.52645	4789.02449	4841.66839	4457.9808	4207.03042	4011.94544	ITGB1BP1
5961.95718	6281.18202	6662.95576	7820.96114	7142.68513	7898.23024	ITGB5
359.214156	328.417449	354.006773	247.116452	255.757797	280.94856	ITGB8
426.067902	429.163864	474.392915	650.190843	740.400288	598.67584	ITM2C
375.17923	380.773853	382.613381	611.750506	657.001006	669.168388	ITPR1
5118.80173	4983.37781	5223.08983	5386.04036	5414.46669	5793.67012	IVD

8063.35999	8141.42095	8096.86198	8763.29855	8632.75229	8535.72807	IVNS1ABP
1246.27356	1204.19731	1225.31637	1328.93737	1389.98803	1397.59138	JADE1
4561.02197	4589.91149	4614.00747	5857.20907	6056.64115	6331.04809	JADE2
1247.27138	1242.2747	1269.41823	985.170923	898.858923	1098.25346	JAG1
10811.3483	11143.1882	11304.3779	13678.1702	13850.7673	14373.3283	JAK1
1680.324	1625.42839	1655.60743	1458.53622	1427.98103	1468.08393	JAK2
116.744601	104.71281	114.426432	69.1926066	63.0127905	71.5141789	JAK3
206.54814	209.425619	182.367126	152.663053	159.385294	148.136513	JAKMIP3
470.969671	452.96223	404.068337	822.623212	800.633102	846.932204	JDP2
95.7904416	104.71281	115.618374	152.663053	139.925461	140.985096	JMJD1C-AS1
1012.78436	989.21874	1085.85916	855.572073	793.219833	839.780786	JMJD7__JMJ
7957.59138	8015.28961	8126.66053	9299.26667	9038.6288	9259.0429	JTB
8191.08058	8033.53503	8523.57722	5172.97107	4763.95229	5188.86449	JUN
8430.55668	8737.17338	10019.4644	12786.3544	10425.8369	11943.8895	JUND
23748.047	24161.6876	26093.9942	30644.6367	28253.8233	30663.2366	JUP
533.832149	514.044702	511.343117	866.555026	799.706444	833.651	KANK1
2034.54907	2050.62586	1973.85595	2227.34296	2214.71425	2311.95124	KANSL1
4514.12456	4736.66808	4600.89611	5282.8006	5400.56681	5554.60844	KAT7
147.676931	124.544781	119.1942	166.940892	206.644886	179.807078	KB-1562D12.
1053.69486	1075.68614	1040.56536	1279.51408	1278.78898	1240.26019	KBTBD7
214.530677	207.045783	252.691703	365.732349	298.384096	355.527632	KCNG1
452.011147	431.543701	492.272045	578.801646	550.435258	584.373005	KCNJ11
342.251265	343.489747	364.734251	117.517602	125.098922	114.422686	KCNJ3
509.884538	493.419452	514.918943	228.445431	249.271186	226.80211	KCNK2
1117.55515	1010.63727	1045.33313	728.169813	670.900887	730.466256	KCNK5
314.312387	273.681207	292.025789	393.189733	394.756599	381.06841	KCNMA1
1019.76908	1100.27778	1050.1009	874.243093	909.052168	893.927236	KCNMB3
623.635688	594.165867	619.809838	206.479525	162.16527	212.499274	KCNN4
3150.10859	2941.47802	2823.71059	1816.5805	1851.46405	1884.90943	KCNQ1OT1
2588.33756	2642.41189	2649.68706	3074.12867	3208.09236	3155.81855	KCTD10
1910.81975	1983.99043	2083.51461	2310.8134	2204.52101	2469.28243	KCTD2
7369.8771	7708.29069	7745.23909	6873.13226	6840.5944	6847.99344	KCTD3
1122.54424	1209.75026	1120.42548	848.9823	914.61212	927.641063	KDELC2
849.142352	917.823642	941.634177	1165.29136	1159.25001	1116.64282	KDELR3
7233.17616	7379.87325	7396.00009	8501.90425	8251.89557	8461.14899	KDM1A
2945.55608	2952.58392	2860.66079	3175.17184	3444.39033	3247.76535	KDM1B
3343.6851	3334.94433	3330.28594	3730.90928	3747.40772	3701.36957	KDM2B
7003.67823	7181.55353	7174.29888	7864.89295	8394.60101	8307.90432	KDM3B
3290.8008	3300.04006	3245.65806	4398.67285	4544.33418	4709.7195	KDM4A
501.902002	448.995835	489.888161	613.947097	566.188456	618.086832	KDM4A-AS1
2425.69337	2402.04839	2555.52364	2834.70028	2693.79679	2854.43737	KDM4B
2245.08848	2298.12886	2201.51687	2015.37196	2005.28939	2015.67821	KDM4C__RP1
15332.4576	15541.9195	15618.016	17468.3874	17593.5418	18070.6114	KDM5B

13405.6727	13469.8751	13710.9088	14794.0383	14828.3923	14772.7861	KHDRBS1
7866.79002	7954.20714	7890.65602	8810.52525	9059.94195	9322.38403	KHSRP
15172.8068	15198.4297	15606.0966	15917.5944	17827.9864	18098.1954	KIAA0100
256.438995	239.570216	259.843355	309.719287	315.990611	303.424445	KIAA0226L
2929.59101	3012.07984	2925.02566	3075.22696	3236.81878	3405.09655	KIAA0232
159.650736	168.968397	185.942951	117.517602	111.199042	116.465949	KIAA0319
10154.7846	10526.0172	10521.272	8450.28437	9122.02808	9357.11949	KIAA0368
17036.7292	17448.1686	16457.1431	15578.2212	15537.2861	14803.435	KIAA0391__F
1588.52482	1604.80314	1618.65723	1920.91856	2002.50941	2048.37041	KIAA0586
1162.45692	1059.82056	1098.97052	930.256156	965.578348	963.398153	KIAA0895L
2772.93372	2946.23769	2915.49012	2250.40716	2278.6537	2248.61011	KIAA0907
6143.55989	6162.98347	6263.65519	6987.35498	7066.69912	7072.75229	KIAA1191
1368.00725	1357.30013	1413.64321	1576.05382	1677.25222	1827.69809	KIAA1549
1351.04435	1466.77261	1388.61243	1769.3538	1859.80398	1836.89277	KIAA1551
1743.18647	1699.20332	1780.76134	999.448763	998.011402	922.532908	KIAA1683
3147.11514	3058.88329	2935.75314	2565.61792	2558.50462	2512.19094	KIAA1715
5099.8432	5013.5224	4940.59958	5260.8347	5626.67153	5741.56693	KIDINS220
5011.03748	5267.37164	4977.54978	5516.73751	5457.09299	5435.0776	KIF11
549.797222	543.39602	598.354882	307.522696	323.40388	305.467707	KIF12
3446.46027	3292.90055	3377.96362	3391.53602	3858.60676	3831.11673	KIF13A
1286.18624	1251.00076	1268.22628	1548.59643	1484.50721	1552.87931	KIF13B
2969.50369	2852.63079	3038.26015	3490.3826	3611.18889	3737.12666	KIF14
1514.68636	1578.62493	1672.29462	1801.20436	1908.91689	1866.52007	KIF15
949.92188	874.193305	855.814353	1174.07772	1057.31756	1051.25843	KIF18A
3670.96911	3750.62246	3840.43711	5350.89491	5306.04762	5404.42866	KIF20A
3624.07171	3473.76813	3451.86402	3886.86722	4075.44489	3954.73409	KIF21A
2100.405	2379.04331	2309.98359	3053.26106	2813.33576	3065.91501	KIF2C
2329.90293	2406.80807	2382.69205	2996.1497	3030.17389	3102.69373	KIF4A
1001.80837	1029.67596	1056.06061	2085.66286	2024.74922	2157.68494	KIF5C
3238.91431	3384.9209	3577.01793	3817.67461	3666.78841	3728.95361	KIFC1
3639.03897	3553.8893	4064.52221	2575.50258	2172.08795	2402.87641	KIFC2
408.107194	383.15369	377.845613	288.851675	270.584336	277.883667	KIFC3
88.8057219	87.2606747	78.6681718	43.9318137	43.5529581	36.7787206	KIT
315.310204	276.854323	299.177441	196.594867	218.691449	234.975159	KLF12
1048.70577	1070.13318	1060.82838	1282.80896	1195.3897	1223.91409	KLF16
146.679114	170.554955	127.537794	85.6670368	69.4994012	79.6872279	KLF3-AS1
6032.80219	6093.96821	6165.91595	5359.68128	4929.8242	5230.75137	KLF4
979.856393	947.968239	985.736031	597.472667	587.501605	552.70244	KLF7
5320.36078	5514.08136	5599.7435	6364.62152	6189.15335	6258.51228	KLHDC3
113.751149	103.919531	121.578084	52.7181765	34.2863713	42.9085073	KLHDC7B
3422.51265	3765.69475	3615.16008	4083.46209	4382.16891	4211.16351	KLHL12
550.795039	493.419452	506.575349	353.651101	410.509797	294.229765	KLHL13
822.201291	778.999842	861.774063	559.03233	492.055761	595.610947	KLHL17

879.076865	836.909199	891.572613	997.252172	1004.49801	1085.99389	KLHL25
438.041707	470.414365	417.179699	1025.80785	1012.83794	1084.97226	KLHL4
1350.04654	1354.12702	1373.11718	1563.97257	1600.33955	1465.01904	KLHL8
1914.81102	1960.19207	2031.06916	2198.78728	2300.89351	2247.58848	KLHL9
232.491384	217.358408	238.388399	126.303965	143.632096	130.768784	KLK10
224.508848	232.430706	234.812573	153.761348	132.512192	148.136513	KLK6
4629.87135	4538.34837	4850.01198	4856.66201	5056.77643	5499.44036	KMT2B
5783.34791	5725.88682	5612.85486	6027.44485	6255.87277	6152.26265	KMT2E
1835.98347	1848.33975	1725.93201	2138.38103	2208.22764	2115.79806	KNSTRN
4720.6727	4897.70369	4910.80103	3981.32062	4362.70908	4368.4947	KNTC1
25509.1942	26150.4377	25848.4541	32901.6336	32852.8303	32414.3124	KPNA2
989.834564	1005.8776	1057.25255	598.570962	631.981222	602.762365	KRT15
204.552506	200.699552	241.964225	146.073281	145.485413	129.747153	KRT16
849.142352	884.50593	862.966005	360.240873	329.890491	403.544295	KRT17
192.5787	203.872667	233.620631	151.564757	124.172264	157.331194	KRT18P28
139188.501	140766.541	151047.658	139342.927	123658.895	134748.038	KRT19
10.9759881	13.4857406	35.7582599	6.58977206	7.41326947	1.02163113	KRT32
217.524128	232.430706	207.397907	369.027235	348.423665	383.111673	KRT6A
473.963123	425.19747	488.696219	283.360199	309.504	301.381182	KRT7
1974.68004	1960.19207	2060.86771	1253.15499	1185.19646	1203.48147	KRT81
324.290558	326.830891	355.198715	271.27895	228.884695	248.256364	KRT86
20.9541591	35.6975488	27.4146659	8.78636275	13.8998803	15.3244669	KRTAP5-10
7803.92754	7296.57897	7674.91452	5051.06028	5542.34558	5519.87298	KRTCAP2__M
3247.89466	3449.17649	3337.43759	4314.10411	3740.9211	3858.70077	KTI12__TXNI
15041.095	14911.2628	14522.6213	13646.3196	13258.6324	13640.8188	KTN1
8808.72936	9524.10601	8767.92533	7326.72824	7153.80504	7264.81894	KYNU
283.380057	241.156774	268.186949	144.974985	133.43885	157.331194	L3MBTL1__R
1084.62719	1009.84399	1020.30235	752.33231	841.406084	811.175115	LA16c-358B7
136.700943	150.722984	132.305562	75.7823787	86.1792575	60.2762365	LA16c-380H5
2121.35916	2092.66964	2110.92928	2711.6912	2847.62213	2638.8732	LAGE3
645.587664	723.470321	687.750532	483.249951	518.928863	483.231523	LAMA3
803.242766	771.067053	892.764555	454.694272	422.55636	435.21486	LAMB3
5830.24532	6070.16985	6225.51305	6375.60447	6848.93433	6906.22642	LAMC1
99.78171	100.746415	94.1634177	39.5386324	45.4062755	38.8219828	LAMP3
1710.25851	1703.16972	1566.21178	1991.20946	2207.30098	1962.5534	LAMTOR2
3648.01932	3603.07259	3367.23614	3241.06956	3259.98525	2980.098	LAMTOR5
14504.2694	15441.9663	15143.6231	19948.3383	18854.7242	18973.7333	LAPTM4B
21373.2423	20565.7545	20986.5227	22530.4307	24504.5622	24948.2321	LARP1
6239.35033	6099.52116	5896.53706	6639.19535	6722.90875	6851.05834	LARP4
2790.89443	2713.80698	2753.38601	2475.5577	2346.29979	2345.66507	LARP7
1078.64029	1078.06597	1106.12217	1386.04872	1454.85413	1533.46832	LARS2
219.519762	191.180206	181.175184	271.27895	252.051162	264.602462	LCA5
85.8122706	91.227069	82.2439978	31.850565	32.4330539	45.9734007	LCN12

525.849612	491.832894	551.869144	727.071517	732.987018	697.77406	LCN2
3398.56504	3499.15306	3555.56298	4038.43198	4041.15852	4103.89224	LDB1
33217.3313	34506.0439	34203.9675	21964.8086	21506.8214	21161.0455	LDHA
377.174864	376.01418	338.511527	249.313043	276.144288	256.429413	LDHAP4
769.316984	752.02836	773.570356	679.844818	626.42127	660.995339	LDHD
5651.63606	5610.86139	5854.81909	6880.82033	6862.83421	7310.79234	LDLR
1319.11421	1285.90503	1245.57939	1470.61747	1522.50022	1580.46335	LDLRAD4
6326.16042	6183.60872	6354.24278	7100.4794	6860.05423	6963.43776	LDOC1
34.9235985	38.8706642	32.1824339	9.88465809	12.0465629	17.3677292	LEMD1
7643.27899	7546.46181	8143.34772	4619.43021	4397.92211	4968.19217	LENG8
2045.52506	1938.77354	1932.13798	1361.88623	1501.18707	1392.48323	LGALS1
18512.5007	18239.0676	17945.8787	13679.2685	13672.8489	13049.2944	LGALS3
2712.06688	2867.70308	2703.32445	2099.9407	2124.82836	2085.14913	LGALS8
1951.73025	2094.25619	1945.24934	2463.47646	2341.66649	2435.56861	LGMN
1517.67981	1506.43656	1410.06738	1235.58226	1393.69466	1345.48819	LHFPL2
287.371325	315.724987	343.279295	227.347136	235.371306	243.148208	LHX1
399.12684	398.225988	370.693961	200.988048	242.784575	212.499274	LIF
508.886721	473.58748	498.231755	523.886879	605.10812	605.827258	LIFR
1428.87409	1439.00785	1551.90848	1715.53733	1738.41169	1742.9027	LIG3
155.659468	145.963311	133.497504	164.744302	208.498204	217.60743	LIMCH1
13578.2951	13489.707	14340.2542	13357.468	12014.1298	13033.9699	LIME1__RP4
2329.90293	2271.15738	2364.81292	2952.21788	2725.30319	2806.42071	LIMK1
632.616042	733.782947	800.985022	343.766443	361.396887	396.392877	LIN7A
196.569969	193.560042	193.094603	146.073281	118.612312	145.07162	LINC-PINT
5095.85193	5546.6058	5149.18943	2537.06224	2569.62453	2426.37393	LINC00052
1306.14258	1351.74718	1301.60066	1521.13905	1525.28019	1502.81939	LINC00094
146.679114	153.896099	156.144402	60.4062439	28.7264192	39.843614	LINC00173
583.723004	550.53553	607.890418	537.066423	417.923066	466.885425	LINC00174
247.458641	262.575303	240.772283	195.496571	189.038371	172.65566	LINC00294
560.77321	541.016183	488.696219	322.898831	364.176863	342.246428	LINC00342
208.543774	167.38184	183.559067	142.778395	133.43885	137.920202	LINC00461__
104.770796	91.227069	76.2842878	37.3420417	40.7729821	37.8003517	LINC00482
995.821466	971.766605	1051.29284	746.840834	906.272192	801.980435	LINC00504
362.207607	418.05796	377.845613	622.73346	660.707641	622.173356	LINC00511
667.53964	693.325725	648.416446	372.322121	303.944048	331.008485	LINC00641
60.8668431	73.7749341	66.7487518	27.4573836	25.9464431	24.5191471	LINC00672
1303.14913	1356.50685	1329.01533	1540.90837	1581.80637	1663.21548	LINC00847
57.8733918	68.2219821	53.6373898	166.940892	124.172264	100.11985	LINC00865
139.694394	149.929705	139.457214	75.7823787	59.3061557	63.3411299	LINC00894
357.218522	403.77894	419.563583	526.08347	509.662276	468.928687	LINC00992
502.899819	478.347153	526.838362	251.509634	341.937054	316.705649	LINC01002__
625.631322	688.566052	637.688968	889.619228	781.17327	864.299933	LINC01003
2006.61019	2071.25111	2130.00035	2602.95996	2403.75262	2544.88314	LINC01006

486.934745	521.977491	518.494768	639.20789	634.761198	677.341437	LINC01016
32.9279643	30.1445967	14.303304	8.78636275	8.33992815	6.12978676	LINC01030
762.332265	714.744254	694.902184	376.715303	474.449246	406.609189	LINC01087
187.589615	139.61708	205.014023	108.731239	113.979018	140.985096	LINC01125
37.9170498	38.8706642	23.8388399	18.6710208	10.1932455	14.3028358	LINC01132
258.434629	253.849236	259.843355	70.290902	102.859114	82.7521213	LINC01291
505.89327	491.832894	470.817089	228.445431	217.764791	221.693955	LINC01293
78.8275509	99.9531365	97.7392437	50.5215858	38.9196647	49.0382941	LINC01341
359.214156	334.763679	305.137151	172.432369	139.925461	130.768784	LINC01355
425.070085	484.693384	498.231755	356.945987	326.183857	329.986854	LINGO4__RC
199.56342	172.934792	177.599358	125.205669	107.492407	84.7953835	LL22NC03-2H
1555.59686	1638.12085	1771.22581	2150.46228	1965.44307	2188.33387	LLGL1
20892.2945	21641.4406	21400.1266	25490.3366	24914.1454	25060.6115	LMBR1
884.065951	817.870506	849.854643	626.028346	654.22103	637.497823	LMCD1
14071.2168	13828.4371	14957.6801	13322.3225	12137.3754	12738.7185	LMNA
10606.7958	10640.2494	10443.7958	12344.8397	12390.3533	12558.9114	LMNB1
6884.93799	6715.89884	6994.31564	8415.13892	8347.34142	8343.66141	LMNB2__TIN
257.436812	230.844149	271.762775	183.415322	137.145485	175.720554	LMNTD2
1763.14282	1847.54647	1742.6192	1598.01973	1602.19286	1438.45663	LMO7__UCH
3385.59342	3165.97594	3290.95185	3654.02861	3787.25404	3835.20325	LMTK2
1470.78241	1441.38769	1454.16924	1091.70557	1066.58414	1040.02049	LMTK3
2055.50323	2063.31832	2004.84644	2383.3009	2378.73284	2383.46542	LNX2
55.8777576	53.9429626	73.9004038	24.1624976	13.8998803	24.5191471	LOX
1664.35892	1737.28071	1728.3159	1573.85723	1478.0206	1433.34847	LOXL1-AS1
2009.60364	1971.29797	2233.6993	2674.34916	2260.12053	2471.3257	LPAR2
1436.85663	1407.2767	1413.64321	1523.33564	1625.35933	1586.59314	LPCAT3
2476.58204	2475.03005	2588.89802	1696.86631	1716.17188	1748.01086	LPIN3
838.166364	836.909199	923.755047	1254.25328	1025.81116	1211.65452	LRFN3
2096.41373	2093.46292	2377.92428	3283.90308	2713.25663	3222.22457	LRFN4
1387.96359	1442.97425	1417.21903	1830.85834	1669.83895	1701.01583	LRIF1
961.895685	988.425461	1044.14119	739.152766	755.226827	756.007034	LRIG2
13505.4545	13719.7579	14204.3728	17020.2829	16240.6201	17303.3664	LRP10
199.56342	230.05087	196.670429	109.829534	126.95224	153.244669	LRP1B
2957.52989	3088.23461	3023.95685	2717.18268	2824.45567	2829.91822	LRP8
15009.1648	15513.3614	15337.9096	16396.4512	16324.0194	16465.6289	LRPPRC
1482.75621	1485.01803	1542.37294	1352.00157	1301.95545	1417.00237	LRRC14
67.8515628	71.3950975	63.1729258	91.1585135	132.512192	114.422686	LRRC37A4P_
145.681297	165.002003	132.305562	194.398276	232.59133	255.407782	LRRC37A6P
1951.73025	1928.46091	2091.8582	1818.77709	1559.56656	1763.33533	LRRC45
7033.61274	7237.08305	7043.18526	5782.52498	5978.80182	5954.06621	LRRC58
13433.6116	13582.5207	13621.5131	14579.8707	14453.0955	14729.8776	LRRC59
776.301704	817.870506	920.179221	1264.13794	1012.83794	1100.29672	LRRC61
10607.7936	10157.1425	9945.56402	11813.2647	11531.3407	10400.2049	LRRC75A

2887.68269	2879.60227	3065.67482	3685.87917	3606.5556	3735.0834	LRRC8A
1673.33928	1674.61168	1684.21404	1850.62765	1807.91109	1845.06582	LRRC8C__LR
3049.32906	3198.50037	3147.91881	3680.3877	3563.00264	3497.04335	LRRFIP2
241.471738	238.776937	272.954717	207.57782	189.038371	195.131545	LRRN2
1929.77827	2004.61568	2042.98858	2542.55372	2303.67349	2504.01789	LRWD1
7120.42283	6790.46705	6692.75431	8038.42362	8551.20633	8150.57313	LSM14B
5671.5924	5726.6801	6009.77155	7343.20267	6756.26846	6938.91861	LSM4
1732.21049	1623.04855	1452.97729	1292.69362	1551.22664	1263.7577	LSM8
6774.18029	6785.70738	7088.47905	7984.60715	7642.15416	8256.82277	LSS
5700.52909	5551.36547	5620.00651	4635.90464	5108.66932	5155.15067	LTB4R__LTB
4236.73141	4277.35962	4333.9011	5175.16766	5148.51564	5380.93115	LTBP1
3662.98658	3581.65406	3921.48917	4901.69212	4260.77663	4741.39006	LTBP3
5236.54414	5271.33803	5660.53254	6595.26354	6544.06362	7133.02853	LTC4S__MAN
11646.5212	11635.8143	10986.1294	9224.58259	9071.06185	8850.39045	LUC7L3
1693.29562	1607.97625	1690.17375	1839.6447	2080.34874	2061.65161	LUZP1
39.912684	48.3900105	52.4454478	17.5727255	22.2398084	16.346098	LY6G6C
4097.03701	4121.08368	4068.09803	3356.39057	3193.26582	3563.44937	LYPD3
1290.17751	1261.31339	1251.5391	1136.73568	1121.25701	1147.29176	LYPD6B
5458.05954	5456.96529	5480.5493	6366.81811	5891.69591	5786.5187	LYPLA1
585.718638	537.049789	524.454478	656.780615	663.487617	649.757397	LYSMD2
2091.42464	2137.88653	1952.40099	2381.1043	2424.13912	2464.17428	LYSMD3
769.316984	751.235082	743.771806	702.90902	584.721629	601.740734	LYSMD4
6441.9072	6876.14117	6635.54109	7660.61002	7656.05404	7565.1785	M6PR
1592.51609	1466.77261	1357.62193	747.939129	879.39909	763.158452	MACC1
8801.74464	8707.02878	8964.59576	10506.2933	12423.713	12790.8217	MACF1
512.87799	518.011096	485.120393	418.450526	451.282779	388.219828	MACROD2
2787.90098	2825.6593	2735.50688	3135.63321	3045.92709	2977.0331	MAD2L1
4849.39111	4995.27699	5358.97122	6065.88518	6216.95311	6760.13317	MADD
134.705309	115.818714	138.265272	199.889753	197.3783	170.612398	MAF
5863.17328	5844.87865	6006.19572	7213.60382	6655.26266	6894.98848	MAF1
82.8188193	105.506089	128.729736	165.842597	155.678659	133.833678	MAFG-AS1
7348.92294	7366.38751	7446.06165	8844.5724	8782.871	9086.38724	MAGED1
11245.3987	11807.1626	12163.7681	15260.8138	14256.6438	15158.9627	MAGED2
18904.6428	19651.104	19121.1335	15726.491	15175.8893	14644.0606	MAL2
73482.2447	64264.3138	56654.195	41273.939	33599.7172	30918.6444	MALAT1
452.011147	434.716816	388.573091	348.159624	299.310755	328.965223	MALL
620.642236	657.628176	592.395172	749.037424	835.846132	926.619432	MAML3
5748.42432	5751.27174	6027.65068	5451.93809	5158.70889	5605.68999	MAN1B1
4734.64214	4681.93184	5003.7725	3872.58938	3970.73246	4057.91884	MAN2A2
5198.62709	5271.33803	5809.52529	3734.20417	3626.01543	3608.40114	MAN2C1
817.212205	790.105746	774.762298	890.717524	914.61212	920.489645	MANEA
841.159816	902.751344	952.361655	1279.51408	1269.5224	1174.8758	MANSC1
286.373508	308.585477	281.298311	158.154529	188.111713	192.066652	MAOB

1822.01403	1853.8927	1899.95554	2166.93671	2002.50941	2090.25729	MAP2K3
5325.34987	5156.3126	5642.65341	6453.58344	5856.48288	6311.6371	MAP3K11
3178.04747	3211.19283	3443.52043	2812.73437	2335.17988	2608.22427	MAP3K12
344.2469	393.466315	424.331351	262.492587	240.931258	285.035084	MAP3K14
3914.43649	3774.42082	4163.45339	4281.15525	4199.61715	4761.82268	MAP3K3
868.100877	897.991671	967.856901	1115.86807	1202.80297	1235.15203	MAP3K5
4934.20556	4949.26682	4753.46468	5308.0614	5554.39215	5562.78149	MAP7
1492.73438	1465.18606	1537.60518	1583.74189	1645.74582	1727.57824	MAP7D3
1000.81055	1013.81039	1138.30461	1369.57429	1361.26161	1364.89919	MAPK13
461.989318	437.096653	464.857379	185.611913	180.698443	172.65566	MAPK15
704.458873	695.705561	727.084618	966.499902	909.978827	959.311628	MAPK8IP1
2892.67177	2704.28764	3003.69383	2249.30886	1925.59674	2117.84133	MAPK8IP3
5922.04449	6242.31136	6308.94899	5513.44262	5313.46089	5460.61837	MAPK9
8025.44294	8438.10725	8334.05844	9110.35987	8717.07823	9135.42554	MAPKAPK2
1410.91338	1424.72884	1516.15022	1732.01176	1693.93207	1754.14065	MAPKAPK3
511.880173	520.390933	543.52555	623.831755	602.328144	598.67584	MAPKBP1
8521.35804	8503.94939	8826.33048	10575.4859	10003.2805	10052.8503	MARCKSL1
4553.03943	4422.52965	4395.88208	5233.37731	5174.46209	5203.16733	MARK2
6693.35711	6880.90084	7111.12595	9228.97577	9034.92216	9159.94468	MARS
786.279875	890.852161	879.653193	982.974332	972.991617	963.398153	MARS2
2535.45325	2444.88545	2693.78891	3395.9292	3173.80599	3425.52917	MARVELD1
3278.82699	3198.50037	3083.55395	3488.18601	3487.94328	3389.77208	MARVELD2
102.775161	69.0152609	75.0923458	46.1284044	42.6262994	40.8652451	MASP2
1148.48748	1098.69122	1175.25481	1408.01463	1389.98803	1587.61477	MAST3
3295.78988	3277.03498	3292.14379	2271.27477	2638.19727	2744.10121	MAST4
965.886953	985.252346	915.411453	1211.41976	1232.45605	1250.4765	MATN2
923.978635	908.304296	823.63192	710.597087	704.260599	703.903846	MB
715.434861	663.974407	710.39743	820.426622	808.973031	806.066959	MB21D1
3401.5585	3454.72944	3432.79295	3991.20528	4207.03042	4528.89079	MBD6
2281.00989	2233.87327	2230.12348	2460.18157	2481.59195	2394.70336	MCCC1
6451.88537	6820.61165	6747.58364	7536.50265	7585.62798	7688.79586	MCFD2
7804.92536	7899.4709	8663.03443	9775.92685	9250.83364	10008.9202	MCM2
18943.5577	19124.3668	19796.9646	22459.0415	21811.6921	22784.4174	MCM7
7067.53852	7235.49649	7069.40798	7960.44465	7943.31823	7789.93734	MDH1
11714.3728	11632.6412	12015.9673	14148.2406	13814.6277	14189.4347	MDH2
4357.46728	4313.05717	4348.2044	7763.84978	7466.08901	7468.12354	MDK
5691.54874	5177.73113	5043.10659	4214.15923	4000.38554	3543.01675	MDM4
5810.28898	6181.22889	5851.24326	4779.78133	4850.13155	4880.33189	ME1
4667.7884	4680.34528	4697.44341	5066.43642	5409.83339	5385.01767	MECP2
2739.00794	2687.62878	2864.23662	3137.8298	3490.72326	3543.01675	MED12
23056.5597	22703.641	22226.1424	22988.4198	24856.6925	25316.0193	MED13
4134.95406	4117.91057	4285.03148	4463.47228	4563.79402	4585.0805	MED14
1421.88937	1373.95899	1640.11219	1762.76403	1633.69926	1817.48178	MED16

1158.46565	1216.09649	1066.78809	1267.43283	1361.26161	1244.34671	MED20
1003.804	1133.59549	1054.86867	1311.36464	1291.7622	1330.16373	MED21
2141.3155	2086.32341	2288.52863	2523.8827	2325.9133	2602.09448	MED25
454.006781	448.995835	488.696219	618.340278	707.040575	629.324774	MED30
930.963355	909.097575	1106.12217	682.041408	624.567953	578.243218	MEGF6
893.046305	873.400026	902.300091	560.130625	642.174468	590.502791	MEIS1
1904.83285	1948.29288	2029.87722	1636.46006	1491.92048	1598.85271	MEIS3
4588.96085	4666.85954	4903.64937	5961.54712	5715.63076	5917.28749	MEPCE
3229.93395	3072.36903	3419.68159	4576.5967	3758.52762	4265.30996	MESDC1
4530.08964	4801.71695	4829.74897	5297.07844	5243.03483	5203.16733	MEST
1804.05332	1784.87744	1891.61195	3069.73549	3508.32978	3545.06001	MET
3612.0979	3805.3587	3689.06048	4137.27856	4038.37854	3991.51281	METAP1
4239.72486	4093.31892	4354.16411	4939.03416	4870.51804	4897.69962	METTL13
2072.46612	2110.12177	2099.00986	1774.84528	1651.30577	1649.93427	METTL17
327.284009	332.383843	306.329093	430.531775	442.942851	445.431171	METTL7A
441.035158	442.649605	462.473495	591.98119	642.174468	652.82229	METTL7B
6650.45097	6909.45888	6769.0386	7911.02136	7605.08781	7557.00545	METTL9
1437.85444	1471.53229	1425.56263	2125.20149	2327.76661	2287.43209	MEX3A
704.458873	663.181128	747.347632	1066.44478	1002.6447	1040.02049	MEX3B
1772.12317	1742.04038	1712.82065	2168.03501	2194.32776	2143.3821	MEX3C
334.268729	385.533527	336.127643	769.905036	666.267593	633.411299	MFAP2
188.587432	201.492831	200.246255	148.269871	133.43885	134.855309	MFI2-AS1
1616.4637	1668.26545	1567.40373	2047.22252	2182.2812	2205.7016	MFSD6
1104.58353	1199.43764	1101.35441	929.157861	848.819354	881.667663	MFSD8
3988.27495	3981.4666	3872.61955	3917.61949	4489.66132	4603.46986	MGA
48.8930379	42.0437796	45.2937959	14.2778395	25.0197845	12.2595735	MGAT3
7594.38595	7977.21223	8443.7171	9180.65078	8306.56844	9134.40391	MGAT4B
131.711857	102.332973	91.7795337	53.8164718	54.6728623	55.1680809	MGAT4EP
5366.26037	5528.36038	5627.15817	5826.4568	6621.90295	6779.54416	MGAT5
11941.8751	11945.1931	11654.8088	9629.85357	10022.7403	9770.8801	MGEA5
1972.68441	1974.47109	1981.0076	1220.20613	1258.40249	1408.82932	MGLL
4156.90604	3912.45134	4102.66435	4509.60068	4408.11536	4760.80105	MGRN1
11171.5603	11316.123	11083.8686	10087.8427	9382.41917	9331.57871	MGST1
1606.48553	1513.57607	1696.13346	1280.61237	1083.264	1211.65452	MIB2
1266.2299	1437.4213	1284.91347	1583.74189	1585.51301	1568.20378	MICA
1120.5486	1139.94172	1187.17423	1406.91634	1263.03579	1325.05557	MICALL1
4018.20946	3848.19576	4236.16186	2773.19574	2619.6641	2681.78171	MICALL2
902.026659	953.521191	938.058351	1164.19306	1125.8903	1109.4914	MICB
2261.05355	2287.02296	2306.40776	2590.87872	2673.4103	2630.70015	MICU1
1035.73415	1109.79713	1044.14119	1247.66351	1392.768	1274.99565	MIEN1
1127.53332	1147.87451	1113.27383	1308.06975	1220.40949	1317.90415	MIF4GD
1918.80228	1861.82549	1854.66175	2083.46627	2068.30218	2235.32891	MIOS
274.399703	246.709726	232.428689	158.154529	172.358515	161.417718	MIR193BHG

409.105011	348.24942	338.511527	107.632944	91.7392096	114.422686	MIR210HG
529.84088	534.669953	579.28381	430.531775	395.683258	384.133304	MIR22__MIR
552.790674	510.871587	559.020796	440.416433	424.409677	390.263091	MIR34A__MI
2147.3024	2144.23276	2144.30365	1514.54928	1677.25222	1535.51158	MIR3916__R
518.864892	517.217818	518.494768	161.449416	160.311952	171.634029	MIR424__MI
1016.77563	986.045625	1027.454	818.230031	709.820551	762.136821	MIR6511B2_
430.05917	398.225988	425.523293	325.095422	354.910276	319.770543	MIR9-3HG
60.8668431	53.1496837	58.4051578	36.2437463	28.7264192	30.6489338	MIR92B__RF
182.600529	197.526436	164.487996	126.303965	126.025581	123.617366	MIRLET7DHG
214.530677	214.978571	216.933443	602.964144	550.435258	570.070169	MITF
19711.8768	19530.5256	19640.8202	20768.7649	23825.3214	24725.5165	MKI67
2058.49668	2033.967	2003.6545	1838.54641	1753.23823	1783.76795	MKNK1__MC
846.148901	859.914286	854.622411	1020.31637	1002.6447	1028.78255	MLKL
3266.85319	3203.26004	3305.25516	3972.53426	3604.70228	4013.9887	MLLT1
27637.5381	27698.1247	28475.4943	22520.546	21747.7526	22798.7202	MLPH
1922.79355	1850.71958	1845.12621	2109.82536	2261.04719	2190.37714	MLX
5481.00933	5333.21378	5426.91191	6163.63347	6391.16494	6711.09487	MLXIP
500.904184	515.63126	547.101376	390.993142	378.076743	355.527632	MLXIPL
559.775393	522.77077	498.231755	705.105611	695.920671	691.644273	MMACHC
240.473921	228.464312	253.883645	395.386324	333.597126	390.263091	MMD
524.851795	529.910279	594.779056	440.416433	416.069749	494.469465	MMEL1
125.724955	140.410358	150.184692	41.7352231	49.1129102	50.0599252	MMP13
196.569969	210.218898	171.639648	126.303965	113.052359	97.0549571	MMP16
728.406483	838.495756	816.480268	532.673242	489.275785	522.053506	MMP19
862.113975	799.625092	867.733773	534.869832	521.708839	532.269817	MMP25-AS1
83.8166364	75.3614918	91.7795337	64.7994253	45.4062755	46.9950318	MMP9
953.913148	974.93972	998.847393	597.472667	581.014994	619.108463	MMRN2
1219.3325	1220.06289	1240.81162	1411.30952	1453.00082	1498.73286	MOCOS
585.718638	562.434713	551.869144	444.809614	476.302563	463.820532	MOK
4259.6812	4364.62029	4417.33704	4941.23075	5076.23627	5071.37691	MON1B__SY
4279.63754	4130.60303	4484.08579	5070.8296	5027.12336	5115.30705	MORC2
22674.3958	22758.3773	22074.7658	24744.5941	24408.1897	22752.7468	MORF4L1
25559.085	26804.0994	25765.0182	24431.5799	23079.3612	23410.6773	MORF4L2
100.779527	99.1598577	96.5473017	47.2266998	66.7194252	56.189712	MORF4L2-AS
1625.44406	1631.77462	1580.51509	2090.05604	1935.78999	2019.76474	MPC2
1888.86777	1912.59533	1864.19728	1905.54242	2198.0344	2235.32891	MPDZ
1171.43728	1094.72483	1109.698	1333.33055	1232.45605	1223.91409	MPG
379.170498	424.404191	384.997265	573.310169	574.528384	618.086832	MPP2
398.129023	372.841065	359.966483	270.180655	254.831138	291.164871	MPP3
2867.72635	2970.03606	2930.98537	3179.56502	3234.0388	3261.04656	MPP5
2945.55608	3089.02789	3072.82647	3500.26726	3316.51143	3155.81855	MPZL1
2362.83089	2466.30398	2476.85547	1464.02769	1509.527	1453.78109	MPZL2
780.292973	727.436716	821.248036	1403.62145	1325.12192	1501.79776	MRC2

8838.66388	9107.63461	8879.96787	9596.90471	9881.8882	9699.36592	MREG__PEC
3429.49737	3408.71927	3404.18634	3987.91039	3694.58817	3860.74403	MRGBP
4371.43672	4424.11621	4705.787	4020.85925	3741.84776	4193.79578	MROH1
4598.93902	4727.94201	4717.70642	5295.98015	5228.20829	5219.51343	MRPL10
2651.20004	2613.85385	2478.04741	2872.04232	3097.81998	2827.87496	MRPL11
7655.25279	7751.12775	8071.8312	8915.9616	8081.39038	8716.55678	MRPL12__RI
2373.80688	2318.75411	2325.47884	2568.91281	2567.77121	2637.85157	MRPL27
3447.45808	3695.09294	3602.04871	4004.38482	3961.46587	3982.31813	MRPL37
3172.06056	3043.01771	3341.01342	3533.21612	3434.19708	3472.5242	MRPL4
2403.7414	2330.65329	2596.04967	2714.98609	2654.87713	2685.86823	MRPS12
3522.29436	3579.27422	3715.2832	4085.65868	3929.95948	3944.51778	MRPS27
11683.4404	12096.7094	11603.5553	9791.30299	9443.57864	9206.93972	MRPS28__RI
2631.24369	2638.44549	2610.35297	2936.84175	3115.42649	2981.11963	MRPS6__SLC
3545.24416	3617.35161	3612.77619	4109.82118	3918.83957	3972.10182	MRPS7
444.02861	452.96223	486.312335	366.830645	341.010395	316.705649	MSANTD2
1104.58353	1071.71974	1169.2951	618.340278	627.347929	548.615915	MSH5__MSH
2326.90948	2439.3325	2532.87674	2225.14637	1998.80278	2081.06261	MSRB1
89.803539	106.299367	107.27478	65.8977206	54.6728623	48.016663	MSS51
433.052622	450.582393	427.907177	246.018157	251.124503	252.342888	MST1
50.8886721	31.7311545	53.6373898	21.9659069	22.2398084	25.5407782	MST1L
85.8122706	88.8472325	78.6681718	42.8335184	50.0395689	49.0382941	MST1P2
354.225071	418.851239	405.260279	302.031219	238.151282	338.159903	MST1R
145604.465	153938.143	148228.715	132924.489	121976.083	121477.049	MT-ND6
7603.36631	7622.61658	7775.03764	8687.51617	8390.89438	8550.0309	MTA2
1449.82825	1303.35717	1376.69301	1578.25041	1703.19866	1598.85271	MTF1
1342.064	1378.71866	1417.21903	1303.67657	1179.6365	1235.15203	MTFP1__RP4
6885.93581	7137.12991	7044.3772	8877.52126	8886.65677	8842.2174	MTHFD2
1308.13822	1345.40095	1383.84466	1060.9533	1067.5108	1020.6095	MTMR11
2446.64753	2344.93231	2387.45982	1380.55725	1527.13351	1443.56478	MTSS1
833.177279	821.043621	790.257544	1248.76181	1424.2744	1306.66621	MTURN
2130.33951	2145.02604	2053.71606	1760.56744	1662.42568	1734.72965	MUC20
796.258046	774.240169	841.51105	209.774411	229.811353	253.36452	MUC5AC
4594.94775	4673.99905	4416.1451	3038.98322	3134.88633	2943.31928	MXD1__SNR
2777.92281	2760.61044	2886.88352	2523.8827	2455.64551	2404.91967	MXD3__RAB
3222.94923	3263.54924	3420.87353	4176.81719	3562.07598	4029.31316	MXD4
489.928196	467.241249	463.665437	573.310169	578.235018	594.589316	MXRA7
2376.80033	2412.36102	2420.8342	3781.43087	3417.51722	3618.61745	MYADML2__
10850.2632	11172.5395	11406.8849	13203.7066	12483.9458	13330.2429	MYBL2
248.456458	233.223985	246.731993	342.668147	358.61691	334.073379	MYCL
187.589615	201.492831	185.942951	139.483509	108.419066	104.206375	MYEOV
4230.74451	4271.80667	4388.73043	4564.51545	4854.76484	4962.06238	MYH10
383.161767	437.096653	418.371641	272.377245	312.283976	255.407782	MYL5
66.8537457	49.1832894	60.7890418	30.7522696	42.6262994	30.6489338	MYO15A

2676.14546	2603.54122	2890.45934	864.358435	826.579545	896.992129	MYO15B
10453.1319	10665.6343	11004.0085	11518.9216	11279.2895	11984.7548	MYO1C
12428.8098	12615.5137	12574.9881	10055.9922	10710.3211	10854.8307	MYO5C
72.8406483	58.7026357	58.4051578	101.043172	101.005797	91.9468014	MYO7A
2842.78092	2824.07275	2886.88352	3048.86787	3049.63373	3257.98166	MYO9B
2236.10812	2218.80098	2250.38649	1690.27653	1730.99842	1782.74632	MYSM1
1350.04654	1379.51194	1232.46802	1498.07485	1485.43387	1466.04067	MZT1
4698.72073	4827.89515	4579.44115	4079.06891	4001.31219	3988.44792	N4BP2L1__N
4635.85825	4712.07644	4442.36782	4088.95356	4244.09677	3895.47949	NAA20
2536.45107	2562.29072	2647.30317	2798.45654	2958.82118	2831.96148	NAAA__SDA
943.934977	925.756431	936.866409	1182.86409	1209.28958	1239.23856	NAALADL2
5163.70349	5369.70461	5336.32432	7795.70035	7316.89696	7520.22673	NAB2
2969.50369	2924.02588	2908.33847	3290.49285	3141.37294	3135.38593	NABP2
7983.53462	7571.05345	8273.2694	9798.99105	9071.06185	9421.48225	NACC1
3151.1064	3249.27022	3333.86176	3972.53426	4106.02463	4272.46137	NACC2
8423.57196	8591.21007	8956.25216	7548.5839	6973.10659	7533.50793	NADSYN1
1002.80619	1078.06597	1103.73829	1286.10385	1254.69586	1396.56975	NAGA
615.653151	654.455061	666.295576	920.371498	855.305965	951.138579	NAIF1
634.611676	627.483579	641.264794	484.348246	429.04297	429.085073	NAIP
135.703126	132.47757	140.649156	56.0130625	53.7462036	53.1248186	NALT1
248.456458	226.084475	252.691703	351.45451	292.824144	331.008485	NANOS1
3120.17407	2980.34868	3012.03743	2476.656	2688.23684	2600.05122	NANS
1372.99633	1340.64128	1308.75231	1449.74985	1536.4001	1532.44669	NAPEPLD
2902.64995	2916.88637	3000.11801	2640.30201	2578.89112	2694.04128	NARF
6123.60355	6364.4763	6170.68372	6932.44021	6857.27426	6818.36614	NARS
4243.71613	4375.7262	4486.46968	4830.30292	5215.23507	5215.4269	NAT10
720.423947	765.514101	769.99453	965.401607	909.052168	884.732556	NAT8L
56.8755747	84.8808382	83.4359398	113.12442	142.705437	171.634029	NAV2-AS2
9876.39366	9673.24243	9846.63283	11320.1301	11334.889	11380.9708	NBN
3784.72026	3891.03281	3826.13381	3401.42068	3370.25763	3431.65896	NBPF1
1077.64247	970.973326	984.544089	825.918098	832.139498	817.304902	NBPF11
776.301704	845.635266	862.966005	684.237999	727.427066	738.639305	NBPF12
443.030793	345.869584	367.118135	244.919862	286.337533	294.229765	NBPF8
1702.27597	1757.11268	1696.13346	1224.59931	1351.06836	1365.92082	NBPF9
5937.01175	5960.69736	5813.10112	6436.01071	6755.3418	6944.02677	NBR1
2598.31573	2687.62878	2627.04016	3761.66155	3944.78602	3970.05856	NCAM2
7376.86182	7552.80804	7724.97608	9337.70701	9551.99771	9771.90173	NCAPD2
6145.55552	6211.37348	6113.4705	6434.91242	6699.74228	6787.71721	NCAPG2
2057.49886	2096.63603	2135.96006	2362.43328	2494.56518	2601.07285	NCAPH
2421.7021	2468.68382	2652.07094	2967.59402	2797.58257	3029.13629	NCDN
1347.05309	1255.76044	1375.50106	1603.5112	1439.10094	1612.13392	NCK2
1681.32181	1733.31431	1775.99358	2094.44922	2032.16249	2276.19415	NCKAP5L
75929.8901	76067.51	75016.0615	78842.2295	87798.1303	87011.3015	NCOA3

7578.42088	7221.21747	7417.45504	7715.52479	8245.40896	8382.4834	NCOR1
2953.53862	3006.52688	2990.58247	3495.87408	3301.68489	3263.08982	NCS1
2751.97956	2882.77538	2811.79117	3335.52296	3270.17849	3428.59406	NCSTN
1404.92648	1417.58933	1438.67399	1627.6737	1582.73303	1557.98747	NDC80
1391.95486	1330.32865	1293.25707	1587.03677	1696.71205	1647.89101	NDE1
4730.65087	4716.83611	4437.60005	4071.38084	4013.35876	3758.58092	NDFIP1
3438.47773	3319.87203	3382.73139	1424.48906	1422.42108	1539.59811	NDRG1
642.594213	692.532446	636.497026	830.31128	768.200048	822.413057	NDRG4
3645.02587	3682.40047	3659.26193	4264.68082	4395.14214	4693.3734	NDST1
17.9607078	14.2790195	19.0710719	40.6369277	44.4796168	31.6705649	NDUFA4L2
611.661883	502.145519	550.677202	758.922082	675.53418	677.341437	NDUFAF1
602.681529	652.075224	624.577606	565.622102	562.481821	480.16663	NDUFAF5
3833.6133	3835.50329	3954.86354	4286.64673	4235.75684	4230.5745	NDUFS2
1380.97887	1364.43964	1205.05336	1126.85102	1263.96244	1105.40488	NDUFS4
33003.7984	31225.8358	30461.2697	15469.4899	17246.9714	15901.6885	NEAT1__ma
140.692211	125.33806	126.345852	151.564757	220.544767	189.001759	NEB
10508.0119	10474.4541	9952.71567	4474.45523	4273.74985	4336.82413	NECAB1
1225.3194	1211.33682	1326.63144	1053.26523	1009.13131	1087.01552	NECAB3
2766.94682	2940.68474	2783.18456	3399.22409	3255.35195	3338.69052	NEDD1
4880.32344	4839.00105	4801.14236	3547.49396	3670.49504	3666.63411	NEDD9
502.899819	492.626173	568.556332	317.407354	322.477222	350.419477	NEIL1
928.96772	884.50593	864.157947	977.482856	1048.05097	1019.58787	NEK2
442.032976	465.654692	457.705727	354.749396	364.176863	372.895361	NEK8
212.535042	250.67612	233.620631	53.8164718	50.9662276	38.8219828	NELL2
5797.31735	5818.70045	5643.84535	6331.67266	6970.32662	7089.09839	NEO1
761.334448	740.129178	756.883168	1269.62942	1325.12192	1256.60629	NES
13923.5398	14020.4106	13706.141	10299.8137	10186.7589	10384.8804	NET1
2046.52287	2033.17372	2032.2611	2327.28783	2200.81437	2190.37714	NETO2
3842.59365	3809.32509	4025.18812	4797.35406	4825.11176	4696.43829	NEU1
1233.30194	1275.59241	1292.06512	1439.8652	1584.58635	1487.49492	NEU3
1835.98347	1926.08108	2137.152	2378.90771	2198.0344	2514.2342	NEURL1B
7.9825368	8.72606747	8.34359398	28.5556789	27.7997605	23.4975159	NEUROG2
4163.89076	3988.60611	4034.72366	2904.99118	3229.40551	3100.65047	NFAT5
1855.93981	1967.33158	1961.93653	2136.18444	2223.98084	2426.37393	NFATC2
4574.99141	4502.65082	4635.46242	4084.56038	4031.89193	3881.17665	NFATC2IP
579.731735	479.933711	500.615639	349.257919	303.944048	323.857067	NFATC4
16473.9603	16450.2237	16887.4342	19348.6691	20396.6843	20930.1569	NFE2L1
4603.9281	4927.84829	4803.52625	5303.66821	5175.38875	5205.21059	NFE2L2
2522.48163	2365.55756	2348.12573	2575.50258	2825.38233	2554.07782	NFIB
695.478519	638.589483	678.214996	825.918098	859.012599	894.948867	NFIX
2498.53402	2406.01479	2567.44306	2959.90595	3037.58716	3094.52068	NFKB1
3428.49956	3433.31091	3408.95411	3017.01731	3198.82578	3042.4175	NFX1
2289.99025	2363.17773	2305.21582	2809.43949	2800.36254	2898.36751	NFYA

1820.01839	1792.01695	1860.62146	2197.68898	1958.95646	2149.51189	NFYC
654.568018	611.618002	617.425954	746.840834	751.520192	772.353132	NHEJ1__RP1
1988.64948	2014.92831	1988.15925	1778.14016	1733.7784	1668.32363	NHLRC3
12671.2794	12580.6095	12293.6898	14277.8395	14601.3609	13936.0702	NHP2
1370.00288	1295.42438	1381.46077	1433.27542	1579.95306	1622.35023	NHSL1
417.087548	403.77894	404.068337	319.603945	326.183857	365.743943	NICN1
2053.50759	2153.75211	2062.05965	2256.99693	2345.37313	2338.51365	NIPA1
2392.76541	2320.34067	2358.85321	2691.92189	2654.87713	2614.35405	NIPA2
7567.44489	7897.88434	7480.62797	8623.81504	8983.02928	8732.90287	NIPSNAP1__
1320.11202	1375.54555	1375.50106	1175.17602	1063.80417	1101.31836	NIPSNAP3A
716.432678	718.710648	737.812096	1155.4067	1092.53059	1076.79921	NKAIN1
1683.31745	1769.80514	1609.1217	2131.79126	2173.01461	2112.73317	NKIRAS2
130.71404	117.405272	141.841098	83.4704461	73.206036	88.881908	NKPD1
5894.10561	5832.18619	5830.98025	3863.80302	4409.04202	4531.95568	NKTR
3950.3579	4200.41157	4096.70464	4369.01888	4523.94769	4473.72271	NLN
523.853978	486.279942	566.172448	591.98119	644.027785	676.319806	NLRX1
457.000232	433.130258	396.916685	530.476651	578.235018	563.940382	NMB
54266.283	52567.417	50806.5276	56618.2233	58540.7357	54396.7494	NME1__NMI
806.236217	719.503927	725.892676	596.374372	591.20824	647.714135	NMRK1
4825.4435	4906.42976	4984.70143	5565.06251	5249.52144	5594.45205	NMT1
3054.31814	3020.8059	3006.07772	3529.92123	3856.75344	3777.99191	NNT
4533.08309	4824.72203	4692.67564	5543.0966	5331.06741	5571.97617	NOB1
3523.29218	3394.44025	3717.66709	3214.71047	3167.31938	3334.604	NOL6
1528.6558	1423.14228	1436.29011	1348.70668	1258.40249	1181.00558	NOL9
23622.322	23532.6174	22994.945	25704.5042	26273.5537	25941.2576	NONO
3535.26599	3407.13271	3516.22889	3853.91836	3816.90712	3942.47452	NOP14
982.849844	987.632182	955.937481	1135.63739	1121.25701	1163.63785	NOP14-AS1
3176.05183	3243.71726	3441.13654	2889.61505	2853.18209	3109.84515	NOTCH1
14939.3176	14554.2873	14946.9526	17411.2761	19340.2934	20100.5924	NOTCH2
12211.2857	11898.3896	13287.7694	15212.4888	14208.4576	16298.0814	NOTCH3
1054.69268	1055.85416	1109.698	552.442558	493.909078	537.377973	NOXA1
526.847429	565.607828	632.9212	778.691399	764.493414	834.672631	NPAS2
2670.15856	2745.53814	2858.27691	3224.59513	3223.84556	3265.13308	NPC1
30280.7555	30150.1497	29965.4218	27503.512	27212.2589	27624.9057	NPEPL1__ST)
1718.24105	1649.22675	1714.01259	1287.20214	1187.97643	1274.99565	NPIPA1__NP
233.489202	239.570216	245.540051	105.436353	99.1524791	104.206375	NPIPB15
463.984952	448.995835	497.039813	344.864738	361.396887	403.544295	NPIPB3
2735.01667	2658.27746	2715.24387	1887.9697	1686.5188	1771.50837	NPIPB4
2161.27184	2176.7572	2189.59745	1500.27144	1535.47344	1492.60308	NPIPB5
8220.01727	8295.31705	8601.05345	7852.81171	7530.95512	8000.39336	NPLOC4
1515.68418	1398.55063	1408.87544	1572.75893	1734.70506	1609.06903	NPM3
3628.06298	3476.14797	3553.17909	2393.18555	2546.45806	2533.6452	NPNT
168.63109	177.694465	188.326835	132.893737	130.658874	120.552473	NPR3

1421.88937	1360.47325	1498.27109	1537.61348	1554.00661	1659.12895	NPTXR
25521.168	25840.2656	25266.7864	20508.469	21071.2918	20463.2715	NQO1
2174.24346	2186.27654	2187.21356	1917.62367	1869.99722	1859.36865	NQO2
5273.46338	5349.07936	5292.22246	4185.60355	4475.76144	4625.94574	NR2C2
25593.0108	25719.6872	26562.4274	28832.4494	26605.2975	28187.8244	NR2F2
153.663834	158.655772	164.487996	92.2568088	75.0593533	92.9684326	NR2F2-AS1_
1980.66694	1983.19715	2114.5051	857.768663	799.706444	836.715893	NR4A1
588.712089	560.054876	555.44497	771.003331	747.813557	718.206682	NR5A2
1281.19716	1312.08324	1461.32089	1593.62654	1564.19986	1632.56654	NRARP
416.089731	402.985662	394.532801	466.775521	558.775186	609.913783	NRAV
1047.70796	1086.79204	985.736031	1158.70159	1235.23603	1199.39494	NRBF2
12106.5149	12156.9986	12393.8129	14160.3219	13567.2098	14363.112	NRBP1
1454.81733	1456.45999	1382.65272	788.576057	737.620312	764.180083	NRBP2
15259.6169	15410.2352	15260.4334	12993.9322	13517.1702	13570.3263	NRCAM
4468.22498	4520.10295	4614.00747	4073.57743	3894.74645	3919.99863	NRDE2
296.351679	272.887928	276.530543	157.056234	162.16527	155.287931	NRG4
326.286192	346.662862	324.208223	441.514728	383.636695	431.128336	NRGN
14579.1057	15010.4226	14795.576	15111.4456	16488.038	16168.3342	NRIP1
109.759881	102.332973	101.31507	30.7522696	26.8731018	41.8868762	NRK
5787.33918	5650.52533	5653.38089	3398.12579	3219.21227	3312.12811	NRP1
10017.0859	9889.01428	9986.09005	9915.41036	11536.9006	11870.3321	NSD1
1993.63857	2060.1452	1907.10719	1716.63562	1693.00541	1706.12398	NSMCE2
1260.243	1283.5252	1189.55811	1020.31637	1127.74362	1072.71268	NSRP1
1533.64488	1511.19623	1474.43225	1645.24642	1688.37212	1685.69136	NSUN4
739.382471	818.663785	661.527808	535.968128	524.488815	521.031875	NSUN5P2
1054.69268	1098.69122	1075.13168	1427.78395	1301.02879	1386.35344	NT5C3B
2714.06251	2800.27438	2843.9736	3637.55418	3278.51842	3673.78553	NT5DC2
1350.04654	1185.15862	1177.63869	1101.59023	1033.22443	952.16021	NT5DC3
632.616042	675.87359	669.871402	374.518712	355.836934	391.284722	NT5E
235.484836	188.00709	210.973733	147.171576	128.805557	119.530842	NTN4
123.72932	111.852319	107.27478	70.290902	64.8661078	68.4492855	NTN5
255.441178	248.296284	286.066079	173.530664	169.578539	158.352825	NUDT13
1641.40913	1691.27053	1680.63822	2123.0049	1978.41629	2086.17076	NUDT19
35303.7668	36308.3735	37273.2182	40158.0709	38422.9756	39426.7885	NUDT3__RPS
7726.09781	7603.57789	7490.16351	8015.35942	8531.7465	8459.10573	NUFIP2
5662.61205	5798.86848	5572.32883	6158.14199	6203.05323	6262.59881	NUP160
8026.44076	8287.38426	8388.88777	8899.48717	8967.27608	9464.39076	NUP188__PH
12445.7727	13035.1583	13881.3565	13702.3327	13809.9944	15074.1673	NUP210
2530.46417	2383.0097	2451.82469	2188.90262	2101.66189	2034.06757	NUP43
3156.09549	3332.5645	3315.98263	3592.52407	3669.56839	3686.04511	NUP88
2489.55367	2586.08909	2764.11349	2980.77356	2973.64771	3093.49905	NUP93
68.8493799	85.674117	53.6373898	120.812488	105.63909	127.703891	NUPR1
133.707491	112.645598	126.345852	171.334074	176.991809	180.82871	NUS1P2

3935.39064	4013.19776	4075.24969	4364.6257	4310.81619	4273.483	NUSAP1
6753.22614	6488.22781	6537.80185	7015.91065	7218.67114	7043.12499	NUTF2
3558.21578	3536.43716	3740.31399	4002.18823	3888.25984	3988.44792	NXF1
579.731735	640.176041	585.24352	390.993142	406.803162	380.046779	NXNL2
1274.21244	1304.15045	1286.10541	2172.42819	2192.47445	2175.05267	NXPH3
564.764479	624.310464	640.072852	859.965254	867.352528	993.025455	NYNRIN
161.64637	145.170032	148.99275	230.642022	191.818347	217.60743	OAF
1037.72978	1060.61384	1066.78809	543.656195	540.242012	574.156693	OAS1
624.633505	634.623089	686.55859	186.710208	186.258395	237.018421	OAS2
2458.62134	2413.1543	2565.05918	2985.16674	2974.57437	2952.51396	OAT
903.024476	909.890854	926.138931	771.003331	744.106923	775.418025	OBFC1
3602.11973	3495.18666	3444.71237	3271.82183	3014.4207	2897.34588	OCLN
4109.01082	4118.70385	4214.7069	4612.84044	4792.67871	4860.9209	OCRL
3707.88835	3908.48495	3858.31624	5110.36823	5013.22348	5078.52833	ODC1
5978.92007	6047.16476	5909.64842	6298.72379	6400.43153	6408.69206	ODF2
2474.58641	2445.67873	2461.36022	1951.67083	1949.68987	1951.31545	OFD1
5848.20603	6087.62198	6263.65519	7275.10836	7059.28585	7658.14693	OGDH
2570.37685	2463.13086	2332.63049	2783.0804	2770.70946	2858.52389	OGFOD1
625.631322	617.964233	548.293318	780.887989	697.773989	653.843921	OIP5
1695.29125	1640.50069	1605.54587	1962.65378	1958.0298	1845.06582	OPHN1
1353.03999	1307.32356	1290.87318	891.815819	790.439857	779.50455	OPN3
200.561237	189.593648	205.014023	317.407354	300.237413	312.619125	OPRL1
2214.15615	2187.06982	2147.87948	2443.70714	2394.48604	2521.38562	OR2AE1__TF
2722.04505	2658.27746	2829.6703	3986.8121	3746.48106	3793.31637	ORAI2
1826.00529	1934.80714	1863.00534	1577.15211	1570.68647	1604.9825	ORC3
2095.41591	2195.00261	2124.04064	1979.12821	1953.3965	1730.64313	ORMDL1
2649.2044	2716.9801	2795.10398	3144.41957	3227.55219	3332.56074	OSBP
581.72737	563.227992	556.636912	647.994253	685.727426	668.146757	OSBP2
2078.45302	2025.24093	2108.54539	1790.22141	1639.25921	1858.34702	OSBPL7
4889.30379	5139.65374	5060.98572	4337.16831	4298.76963	4288.80747	OSBPL9
1861.92671	1809.46908	1747.38697	1311.36464	1240.79598	1206.54636	OSGEP
1929.77827	1984.78371	2104.96957	2247.11227	2133.16829	2257.80479	OSR2
3500.34239	3490.42699	3275.45661	3920.91438	3700.14812	3561.40611	OSTC
294.356045	301.445967	345.663179	388.796552	379.003401	423.976918	OTUB2
466.978403	445.82272	457.705727	395.386324	359.543569	402.522664	OXTR
173.620176	163.415445	157.336344	61.5045392	55.599521	59.2546054	P2RX2
3497.34894	3653.04916	3714.09126	4235.02684	3799.3006	3968.0153	P2RX5__P2R
915.996098	930.516104	1002.42322	665.566978	739.473629	765.201714	P2RY2
1188.40017	1181.19222	1138.30461	1495.87826	1409.44786	1547.77116	P3H4
4088.05666	4136.94926	3998.9654	3010.42754	2888.39512	2762.49057	P4HA1
2911.6303	3000.97393	3003.69383	2602.95996	2693.79679	2666.45724	P4HA2
29314.8686	29172.0368	30806.9328	32306.3575	32271.8153	33461.4843	P4HB
12718.1768	12574.2632	13255.5869	13721.0037	13640.4158	14382.523	P4HTM__WD

179491.331	181478.405	180250.237	211488.85	207742.05	210851.383	PABPC1
2951.54298	2917.67965	2997.73412	2394.28385	2334.25322	2354.85975	PABPC1L
35.9214156	37.2841065	47.6776799	19.7693162	6.48661078	16.346098	PABPN1L
9389.45891	9708.1467	10222.0946	10529.3575	10482.363	10913.0637	PACS1
438.041707	496.592567	476.776799	278.967017	298.384096	289.121609	PADI2
13.9694394	26.1782024	11.91942	3.29488603	6.48661078	4.08652451	PADI4
32.9279643	29.3513179	22.6468979	2.19659069	1.85331737	5.10815564	PAEP
4667.7884	4766.81268	4662.87709	5009.32506	5203.18851	5247.09747	PAF1
3731.83596	3801.3923	3671.18135	4571.10522	4768.58558	4533.99894	PAIP2
1495.72783	1561.1728	1547.14071	1961.55548	1922.81677	1960.51013	PAK1
14360.5837	14668.5194	14681.1496	16657.8455	16332.3593	16666.8902	PAK2
898.03539	834.529362	820.056094	639.20789	695.920671	597.654209	PALM3
2938.57136	3145.35069	3220.62727	2025.25661	1996.94946	2110.68991	PAM
3424.50829	3497.5665	3401.80246	2847.87983	2810.55579	2715.49554	PAN3
1046.71014	1008.25743	1028.64594	1188.35556	1198.16968	1240.26019	PANK1
803.242766	859.121007	806.944732	919.273202	972.991617	967.484677	PANX1
1399.93739	1435.83474	1467.2806	1622.18222	1542.88671	1712.25377	PAPD5
183.598347	170.554955	191.902661	127.40226	107.492407	159.374456	PAPLN
880.074683	974.146442	932.098641	1146.62034	1082.33734	1172.83253	PAPOLG
3346.67856	3381.74779	3503.11753	3900.04676	4012.4321	4038.50785	PAPSS1
5855.19075	6003.53442	5943.02279	4238.32173	4153.28422	4374.62449	PAPSS2
3014.40546	3306.38629	3256.38553	3031.29515	2716.96326	2937.18949	PAQR4
809.229668	818.663785	793.83337	419.548821	373.443449	353.48437	PAQR6
3901.46486	3714.92491	3830.90158	4037.33368	4350.66252	4290.85073	PARD3
230.49575	256.229072	283.682195	336.078375	338.230419	364.722312	PARD6A
17355.0328	16427.2187	15709.7955	13197.1169	11789.8784	10609.6393	PARD6B
1730.21485	1675.40496	1746.19503	1856.11913	1915.4035	1941.09914	PARG
9198.87585	8948.97883	9002.7379	11818.7562	12015.9832	12370.9313	PARP1
1742.18866	1662.71249	1748.57891	1313.56123	1115.69706	1237.1953	PARP10
2839.78747	2943.85785	2654.45483	2240.5225	2419.50582	2337.49202	PARP2
174.617993	147.549868	152.568576	119.714192	86.1792575	110.336162	PARP3
3935.39064	3965.60103	4097.89658	4360.23251	4859.39814	5018.2521	PARP4
802.244949	881.332815	877.269309	1053.26523	1044.34434	1045.12864	PARP8
1939.75644	2073.63094	1998.88673	1064.24819	972.991617	1033.8907	PARP9
362.207607	351.422536	405.260279	486.544837	437.382899	451.560958	PARS2
1674.33709	1688.09742	1792.68076	1816.5805	1980.26961	2006.48353	PARVA
2000.62329	2009.37536	1963.12847	1749.58448	1682.81217	1635.63143	PAX8-AS1__I
4016.21383	3903.72528	3826.13381	3249.85592	3646.40192	3287.60897	PBX2
2484.56458	2475.82333	2422.02614	3376.15989	3417.51722	3437.78874	PBXIP1
608.668431	618.757512	559.020796	393.189733	455.916072	477.101736	PCBP3
3311.75496	3330.18466	3490.00617	4039.53027	3978.14573	3939.40963	PCCB
566.760113	602.891935	640.072852	360.240873	316.91727	337.138272	PCDH10
1523.66671	1490.57098	1461.32089	1946.17935	2003.43607	2117.84133	PCDH7

5723.47889	5578.33695	5724.89741	6668.84933	6451.39775	6662.05658	PCDHA1__PC
328.281826	385.533527	354.006773	471.168702	458.696048	515.923719	PCDHB4
1603.49208	1634.94773	1562.63596	1457.43792	1348.28838	1426.19705	PCED1A
914.998281	987.632182	995.271567	643.601071	583.79497	665.081864	PCED1B
4033.17672	3832.33018	3972.74267	4154.85128	4375.6823	4448.18193	PCGF3
3596.13283	3652.25588	3703.36378	4504.1092	4234.83018	4611.64291	PCK2
6755.22177	6698.4467	6851.2826	6911.5726	7375.27646	7859.40826	PCNT
8589.2096	8425.41479	9320.98641	9739.68311	9482.49831	10213.2464	PCNXL3
135.703126	122.164945	121.578084	73.585788	81.5459641	71.5141789	PCP2
185.593981	167.38184	208.589849	115.321011	80.6193054	102.163113	PCSK4
1200.37397	1289.87143	1237.23579	1384.95043	1465.97404	1468.08393	PCTP
2388.77414	2427.43332	2766.49737	3000.54288	2826.30898	3018.91998	PCYT2
9005.29933	8697.50943	8392.4636	9178.45419	9969.92077	9184.46383	PDAP1
147.676931	165.002003	164.487996	241.624976	227.958036	228.845372	PDCD4-AS1
1567.57067	1511.19623	1443.44176	1271.82601	1271.37571	1320.96905	PDDC1
54.8799405	49.1832894	58.4051578	7.6880674	8.33992815	5.10815564	PDE10A
113.751149	110.265762	89.3956497	161.449416	170.505198	151.201407	PDE4A
86.8100877	96.7800211	78.6681718	29.6539743	44.4796168	49.0382941	PDE4C
672.528726	680.633263	662.71975	436.023251	479.082539	491.404572	PDE5A
189.585249	185.627254	185.942951	238.33009	252.977821	228.845372	PDE6B
2634.23715	2762.197	2632.99987	2293.24068	2240.6607	2245.54522	PDE7A
143.685663	116.611993	174.023532	63.7011299	50.0395689	46.9950318	PDIA2
10240.5969	10284.0672	10447.3716	12673.23	12134.5955	12520.0895	PDIA4
1982.66258	1973.67781	2063.2516	2264.685	2183.20786	2224.09096	PDIA5__SEC2
1096.60099	1070.13318	1122.80936	565.622102	545.801964	569.048538	PDK1
864.109609	874.986584	904.683975	1247.66351	1252.84254	1272.95238	PDK2
262.425897	229.257591	245.540051	148.269871	168.65188	139.963464	PDK4
11851.0737	11929.3275	11663.1524	12338.2499	12647.9644	12725.4373	PDS5A
2790.89443	2768.54323	2860.66079	1443.16008	1536.4001	1507.92754	PDXDC2P__R
302.338581	288.753506	328.975991	200.988048	225.17806	226.80211	PDZD7
12408.8535	12671.0433	12236.4765	13971.4151	13854.474	13266.9018	PEBP1
2590.33319	2519.45366	2652.07094	2105.43217	1996.0228	2065.73814	PER1__RP11
1224.32158	1223.236	1349.27834	958.811835	934.071953	1016.52297	PER3
164.639822	151.516263	178.7913	110.92783	101.932455	84.7953835	PERM1
1441.84571	1427.10867	1406.49156	1263.03965	1271.37571	1253.54139	PEX11A
3005.42511	2924.81916	2894.03517	3372.865	3263.69188	3322.34443	PEX26__TUB
1252.26046	1358.09341	1225.31637	1136.73568	1124.03698	1134.01055	PEX3
1289.17969	1419.17588	1357.62193	1613.39586	1609.60613	1707.14561	PEX5
4274.64846	4142.50221	4348.2044	3816.57632	3995.75224	3862.78729	PFKFB3
822.201291	913.063969	916.603395	427.236889	465.182659	453.60422	PFKFB4__UC
2676.14546	2696.35485	2724.7794	3031.29515	2985.69428	3132.32104	PFKM
16311.3161	16263.8032	16936.3038	15217.9803	14689.3935	14939.312	PFKP
611.661883	578.30029	497.039813	272.377245	390.123306	373.916992	PFN1P2

163.642005	141.996916	153.760518	91.1585135	126.025581	112.379424	PFN1P6
16179.6043	16589.8408	16674.0766	14566.6911	14645.8405	14555.1787	PGAM1
5165.69913	5034.14765	5229.04954	5796.80282	5600.72508	5475.94284	PGAM5__PX
796.258046	820.250343	809.328616	956.615244	875.692456	922.532908	PGAP2
13065.4171	13097.8273	13275.85	15351.9723	15372.3409	15339.7914	PGD
31055.0616	32097.6493	31834.3868	24518.3453	24163.5518	24696.9109	PGK1
8629.12228	8607.86892	8628.46811	6095.53916	6711.78884	6833.69061	PGR
2337.88547	2391.73577	2242.0429	2619.43439	2603.9109	2659.30582	PHACTR4
10212.658	10395.1262	10058.7985	11043.3597	11159.7505	11432.0523	PHB
7422.76141	7874.08598	7972.90001	8703.9906	8181.46951	8341.61815	PHB2
1222.32595	1254.17388	1213.39695	1018.11978	1044.34434	997.11198	PHF11
3291.79861	3207.22644	3131.23163	3737.49905	3984.63234	3899.56601	PHF14
2470.59514	2384.59626	2479.23935	2795.16165	2621.51742	2669.52214	PHF19
2006.61019	2049.83258	2093.05015	2206.47535	2285.14031	2324.21081	PHF20
2197.19326	2117.26128	2211.0524	2587.58383	2539.97145	2516.27747	PHF23
113.751149	134.857406	131.11362	63.7011299	52.8195449	55.1680809	PHF2P2
1202.36961	1170.08632	1294.44901	2292.14238	2009.92268	2231.24238	PHGDH
5986.9026	5763.96421	5629.54205	4176.81719	4207.03042	4046.68089	PHLDA1
1407.91993	1368.40604	1371.92524	1979.12821	2009.92268	2033.04594	PHLDB1
849.142352	774.240169	829.59163	997.252172	934.998611	985.874038	PHLPP1
2277.01862	2154.54539	2277.80116	2665.5628	2547.38472	2733.8849	PHRF1
2340.87892	2429.01987	2468.51188	3040.08151	2905.07497	2898.36751	PHTF2
5186.65329	5515.66792	5580.67243	5711.13579	6171.54683	6482.2495	PI4KA
215.528494	222.91136	210.973733	165.842597	160.311952	173.677292	PI4KAP1
290.364776	318.104823	369.502019	259.197701	252.051162	275.840404	PI4KAP2
7057.56035	7380.66653	7381.69678	8396.4679	8250.04226	8494.86282	PI4KB
1662.36329	1642.08724	1688.98181	2112.02195	2013.62932	2182.20409	PIAS4
764.327899	795.658698	821.248036	667.763569	618.081342	688.57938	PIDD1
985.843295	985.252346	988.119915	822.623212	818.239617	779.50455	PIGA
1558.59031	1639.70741	1548.33265	1397.03168	1434.46764	1368.98571	PIGBOS1__R
931.961172	954.31447	948.785829	789.674352	809.899689	829.564475	PIGH
1498.72129	1521.50886	1418.41098	1704.55437	1689.29878	1723.49171	PIGM
3382.59997	3383.33434	3394.65081	3755.07178	3664.93509	3843.3763	PIGS
5576.79977	5768.72388	6106.31885	7317.94187	6951.79344	7501.83737	PIGT
1806.04895	1705.54955	1817.71155	1965.94867	2113.70846	2062.67325	PIK3CA
4424.32102	4237.69568	4242.12157	3218.00536	3230.33217	3290.67386	PILRA__PILR
2075.45957	2022.8611	2078.74684	1356.39475	1371.45485	1429.26195	PIM1
8253.94305	8576.93105	8609.39704	9236.66384	9365.73931	9196.7234	PIP5K1A
268.4128	282.407275	271.762775	233.936908	194.598324	198.196439	PIP5KL1
779.295155	831.356247	826.015804	977.482856	939.631905	1003.24177	PJA1
3996.25749	3892.61937	4485.27773	3259.74058	2958.82118	3423.48591	PKD1
1184.4089	1152.63419	1132.3449	985.170923	1009.13131	990.982193	PKD2
529.84088	587.026357	545.909434	296.539743	304.870707	296.273027	PKIA

7716.11964	7680.52593	7524.72982	9936.27797	9614.08384	9541.01309	PKIB
476.956574	515.63126	501.807581	672.15675	562.481821	693.687535	PKN3
4894.29288	4712.07644	5251.69644	6179.0096	5328.28743	5663.92297	PKP3
14.9672565	11.8991829	15.495246	37.3420417	29.6530779	33.7138272	PLA2G3
416.089731	373.634344	394.532801	306.424401	315.990611	344.28969	PLA2G4F
1710.25851	1738.07399	1706.86094	1840.743	1964.51641	1921.68815	PLAGL2
116.744601	118.19855	116.810316	68.0943113	63.9394491	65.3843921	PLAU
14208.9155	13932.3566	14285.4248	11490.3659	11735.2056	12320.8714	PLCG1
899.033207	890.852161	883.229019	1030.20103	1043.41768	1052.28006	PLCG2
345.244717	420.437796	406.452221	83.4704461	120.465629	88.881908	PLCXD3
900.031025	913.857248	985.736031	784.182875	813.606324	821.391426	PLD1
331.275277	389.499921	382.613381	563.425511	568.968432	540.442866	PLEK2
1837.9791	1853.8927	1865.38922	1739.69982	1619.79938	1640.73959	PLEKHA1
1244.27792	1285.11176	1143.07238	1454.14304	1538.25341	1648.91264	PLEKHA8
684.502531	634.623089	665.103634	1279.51408	1238.016	1259.67118	PLEKHB1
7867.78784	8170.77227	8021.76964	8685.31958	8743.95134	8933.14257	PLEKHB2
6822.07552	7080.80712	6844.13094	8720.46503	8161.08302	8411.08907	PLEKHF2
3184.03437	3190.56758	3163.41406	2399.77533	2462.13212	2552.03456	PLEKHH1
2263.04918	2095.84275	2519.76538	2896.20482	2430.62573	2688.93313	PLEKHH3
2879.70015	2848.66439	2888.07546	1996.70093	2133.16829	2213.87465	PLEKHM1P__
2798.87697	2708.25403	2952.44033	3129.04343	2894.88173	3230.39762	PLEKHM2
367.196693	368.081392	379.037555	243.821566	220.544767	223.737217	PLEKHN1
2942.56263	2977.17557	3118.12026	3747.38371	3681.61495	3558.34122	PLIN3
4039.16362	3996.5389	4157.49368	5347.60003	5385.74027	5602.6251	PLK1
12941.6878	13308.0462	12995.7436	10585.3705	10364.6774	10423.7024	PLK2
240.473921	231.637428	227.660921	151.564757	155.678659	178.785447	PLK3
1826.00529	1958.60551	2009.61421	2420.64294	2263.82716	2315.01613	PLLP
6645.46189	6566.76241	7127.81314	7571.6481	7013.87957	7633.62778	PLOD3
3602.11973	3835.50329	3549.60327	2909.38437	3171.02601	3031.17955	PLP2
1911.81756	2026.82749	2144.30365	2950.02129	2694.72345	2920.84339	PLPP2
1519.67544	1542.13411	1548.33265	1699.0629	1689.29878	1801.13568	PLPP6
2769.94027	2969.24278	3134.80745	3799.00359	3488.86994	3655.39617	PLPPR2
76.8319167	73.7749341	75.0923458	10.9829534	8.33992815	16.346098	PLS3
7727.09563	7649.58806	8404.38302	8538.148	8352.90137	9322.38403	PLXNA1
8251.94742	8065.26618	8809.6433	6398.66867	6405.99148	7047.21151	PLXNA3
298.347313	321.277939	339.703469	117.517602	123.245605	125.660629	PLXNA4
1057.68613	991.598577	1058.44449	1146.62034	1208.36292	1215.74104	PMS2
1121.54642	1151.84091	1188.36617	1029.10274	965.578348	1036.95559	PMS2CL
8022.44949	7900.26418	7691.6017	5337.71537	5543.27224	5355.39037	PNISR
14064.232	14384.5256	13970.7521	10650.1699	11039.2849	11292.0889	PNN
518.864892	564.814549	667.487518	345.963033	380.856719	406.609189	PNPLA7
8557.27945	8568.99826	8550.99188	4951.11541	5268.05461	5227.68648	PODXL
1666.35456	1695.23693	1759.30639	2441.51055	2330.54659	2362.01117	PODXL2

1867.91361	1741.2471	1901.14748	1494.77996	1605.8995	1596.80945	POFUT2
5790.33263	5887.71571	6037.18621	6231.72778	6432.86458	6379.06476	POGK
10029.0597	9605.81373	9822.79399	10404.1518	10668.6214	10622.9205	POGZ
2637.2306	2710.63387	2691.40503	3136.7315	3098.74664	3140.49408	POLDIP3
1668.35019	1699.9966	1601.97004	1418.99758	1472.46065	1439.47826	POLI
2608.2939	2713.80698	2824.90253	3145.51786	3300.75823	3443.91853	POLR1B
1283.19279	1331.91521	1302.7926	1388.24531	1451.1475	1516.10059	POLR1E
16166.6327	16482.7482	16999.4768	18925.8254	19451.4924	20292.6591	POLR2A
3981.29023	4123.46352	3761.76894	4445.89955	4645.33998	4511.52306	POLR2G
2646.21095	2356.8315	2366.00486	2280.06113	2268.46046	2144.40374	POLR2J2__P(
2533.45762	2706.66747	2646.11123	2821.52074	2877.27521	2887.12957	POLR3C
1196.3827	1274.79913	1278.95376	1391.5402	1449.29418	1463.99741	POLR3D
4003.24221	4071.9004	4152.72592	4989.55575	4496.14793	4931.41345	POLR3H
2022.57526	2038.72667	2096.62597	2293.24068	2265.68048	2399.81152	POM121
2811.84859	2718.56666	2798.67981	3192.74456	3370.25763	3472.5242	POM121C
5715.49635	5610.06811	5366.12287	4953.312	5003.95689	4366.45144	POMP
3566.19832	3740.30983	3940.56024	4461.27569	4311.74285	4552.3883	POMT2
1355.03562	1473.91212	1425.56263	1822.07198	1831.07756	1705.10235	POP1
6332.14732	6765.87541	7205.28937	8232.82189	7263.15076	8121.96746	POR
234.487019	295.099736	296.793557	149.368167	197.3783	218.629061	POTEI
611.661883	549.742251	582.859636	327.292012	355.836934	436.236491	POTEKP__RP
389.148669	359.355324	369.502019	211.971001	230.738012	218.629061	PP14571
434.050439	425.19747	448.170191	291.048266	258.537773	311.597494	PPARG
1551.60559	1537.37443	1762.88221	1853.92254	1733.7784	1807.26546	PPFIA3
1450.82606	1542.13411	1668.7188	304.22781	317.843928	326.921961	PPFIA4
48901.0205	49381.6091	47831.4404	54383.1922	54860.974	51688.4052	PPIA
14185.9657	14044.209	13552.3805	15275.0916	15882.0032	14865.7545	PPIB
910.009196	914.650527	910.643685	800.657305	776.539977	756.007034	PPIP5K1
3070.28322	3219.12562	3002.50189	3414.60022	3427.71047	3475.58909	PPM1B
15845.3356	16105.1474	16198.4917	18418.4129	17776.0935	17926.5614	PPM1D
1042.71887	1040.78187	1131.15296	943.4357	955.385102	951.138579	PPM1E
12939.6922	13198.5737	12979.0564	13830.8333	13970.3063	13877.8372	PPM1G
3335.70257	3410.30582	3387.49915	4006.58141	4163.47746	4071.20004	PPM1H
1330.0902	1216.88977	1262.26657	1092.80387	1045.271	976.679357	PPM1K
506.891087	508.49175	500.615639	438.219842	396.609916	396.392877	PPOX
13759.8978	14037.0695	14390.3157	16188.8734	15192.5691	15626.8697	PPP1CA
1826.00529	1782.4976	1985.77537	1634.26347	1587.36632	1648.91264	PPP1R15A
604.677163	652.075224	574.516042	918.174907	842.332743	855.105253	PPP1R3C
457.998049	428.370585	446.978249	639.20789	525.415473	558.832226	PPP1R3F
71.8428312	61.8757512	78.6681718	38.440337	23.1664671	27.5840404	PPP1R3G
5810.28898	5963.87048	6245.77606	7298.17256	7158.43833	7199.43455	PPP1R9B
13471.5287	13469.0818	14262.7779	16422.8103	15612.3455	16625.0033	PPP2R1A
3597.13065	3807.73853	3821.36604	4434.9166	4146.79761	4042.59437	PPP2R2A

14054.2539	14061.6611	14141.1999	15510.1268	16138.6876	15775.0062	PPP2R4
2530.46417	2613.85385	2511.42179	2830.3071	2897.6617	2966.81679	PPP2R5D
1482.75621	1481.05163	1642.49607	1055.46183	1000.79138	1079.8641	PPP4R1L
3227.93832	3211.98611	3375.57973	3589.22918	3424.93049	3748.36461	PPRC1
9480.26027	9493.16813	9312.64282	10708.3796	10859.5131	10884.458	PRC1
4442.28173	4343.20177	4473.35831	4929.1495	4631.4401	4758.75779	PRCC
1735.20394	1714.27562	1812.94378	2136.18444	2137.80158	2033.04594	PRCP
1329.09238	1236.72175	1252.73104	1475.01065	1476.16728	1465.01904	PRDM15
2606.29827	2610.68073	2580.55442	2756.72131	2870.7886	2911.64871	PRDM2
6979.73062	7189.48632	7058.6805	8767.69173	8918.16317	8609.28551	PRDX2
7544.4951	8240.58081	8251.81444	8741.33264	8731.90477	8423.34864	PRDX3
13198.1268	13342.1572	13010.0469	14215.2366	14699.5867	14283.4248	PRDX6
7540.50383	7479.82638	7424.6067	8249.29632	8059.15057	8154.65966	PRELID1
18971.4965	19579.7089	19969.7962	14631.4906	14538.3481	15762.7467	PREX1
159.650736	168.968397	145.416924	38.440337	63.0127905	36.7787206	PRG4
7041.59528	7025.2776	7026.49807	6070.27836	5886.13596	5783.45381	PRICKLE2
101.777344	92.8136268	90.5875917	63.7011299	49.1129102	59.2546054	PRKAA2
5824.25842	5827.42651	5845.28355	6191.09085	6428.23129	6521.07148	PRKACA
945.930611	945.588403	899.916207	834.704461	808.046372	809.131853	PRKACB
582.725187	618.757512	599.546824	969.794788	1002.6447	990.982193	PRKAR2B
2810.85077	2763.78355	2847.54943	3212.51388	3185.85255	3238.57067	PRKCH__RP1
5354.28656	5288.79017	5232.62536	5942.8761	5998.26166	5970.41231	PRKCI
17097.596	16723.905	17346.3319	18482.114	18661.0526	18639.6599	PRKCSH
3689.92764	3713.33835	3964.39908	4140.57345	4004.09217	4287.78584	PRKD2
1762.145	1700.78988	1814.13572	1914.32878	2034.01581	2062.67325	PRKX
4577.98486	4555.00722	4562.75396	5030.19267	5117.00925	5124.50173	PRMT2
370.190144	360.941882	346.855121	438.219842	415.14309	485.274785	PROB1
386.155218	383.946969	367.118135	270.180655	276.144288	275.840404	PROCA1
127.720589	107.092646	90.5875917	45.0301091	39.8463234	51.0815564	PRODH
1565.57503	1572.2787	1636.53636	1791.31971	1879.26381	1899.21227	PROSC
4651.82332	4693.83102	4786.83906	5382.74548	5279.17452	5476.96447	PROSER1
1008.79309	1016.9835	1081.09139	1991.20946	1705.97864	1955.40198	PROSER2
7220.20454	7131.57696	7093.24682	5917.61531	6041.81462	5917.28749	PRPF3
727.408666	713.950975	678.214996	542.5579	550.435258	611.957045	PRPF40B
20974.1155	21766.7787	22246.4054	22038.3944	23277.6661	24465.0006	PRPF8
3662.98658	3549.12963	3771.30448	4076.87232	4159.77083	4548.30178	PRR12
886.061585	909.890854	905.875917	1204.82999	1099.94386	1215.74104	PRR15
1418.89592	1400.93047	1643.68801	1958.2606	1536.4001	1795.00589	PRR7
22007.854	21814.3754	23000.9047	24153.7112	24175.5984	25793.1211	PRRC2A
19041.3437	18975.2304	19379.7849	19261.9037	22443.6733	23641.5659	PRRC2B
516.869258	492.626173	452.937959	667.763569	671.827545	670.190019	PRRG1__RP5
43.9039524	49.9765683	63.1729258	4.39318137	6.48661078	7.15141789	PRSS1__PRS
1338.07273	1277.97225	1301.60066	863.26014	824.726228	884.732556	PRSS22

13739.9415	14432.1223	14032.7331	8851.16217	8685.57184	8795.22237	PRSS23
167.633273	181.660859	200.246255	104.338058	85.2525989	88.881908	PRSS27
2763.95337	2913.71326	3007.26966	2624.92587	2316.64671	2427.39556	PRSS53__RP
457.998049	456.928624	448.170191	327.292012	332.670467	336.116641	PRTG
2804.86387	3033.49837	2938.13702	3437.66443	3323.9247	3424.50754	PRUNE
26767.4415	26347.9641	26881.8679	30977.4202	31510.1019	32098.6284	PSAP
26.9410617	26.1782024	23.8388399	10.9829534	6.48661078	7.15141789	PSAPL1
115.746784	124.544781	121.578084	49.4232905	53.7462036	63.3411299	PSCA
277.393154	252.262678	263.419181	205.381229	204.791569	198.196439	PSD
2051.51196	2120.4344	2119.27287	2605.15656	2606.69088	2875.89162	PSKH1
10359.3371	10590.2728	9976.55451	9051.05193	8929.28307	8640.95607	PSMA5
9188.89768	9441.60501	9003.92984	8746.82412	8566.95953	8420.28375	PSMB1
8008.48005	7837.59515	7705.90501	7282.79642	7527.24848	7207.6076	PSMB7
6383.03599	6406.52008	5994.2763	5490.37842	5876.86937	5436.09923	PSMC1
74.8362825	75.3614918	65.5568098	49.4232905	41.6996408	32.6921961	PSMC1P7
9737.69708	10134.9307	9672.6093	9026.88943	8636.45893	8689.99437	PSMC2
8395.63308	8396.06347	8654.69084	7972.5259	7759.83981	7782.78593	PSMC4
20901.2748	21477.2319	21007.9777	19986.7787	20379.0778	19671.5074	PSMC5
8112.25303	8465.87201	7779.80541	6678.73398	6632.0962	6355.56724	PSMC6
6980.72843	7300.54536	7132.58091	6334.96754	6628.38956	6407.67043	PSMD1
4824.44568	4720.00922	4855.97169	4271.27059	4207.03042	4158.03869	PSMD11
15707.6368	16179.7157	15423.7294	13742.9696	13739.5683	13020.6887	PSMD12
3303.77242	3385.71418	3485.2384	3168.58207	3133.95967	3122.10472	PSMD13
2558.40305	2549.59826	2394.61147	2125.20149	2019.18927	2041.21899	PSMD5-AS1
64749.3495	67022.5444	63607.9846	55812.0745	56094.3567	54051.438	PSMD6
351.231619	298.272852	349.239005	155.957939	154.752	206.369488	PSMD6-AS2_
6895.91398	6871.3815	6859.62619	5456.33127	5783.27684	5544.39213	PSME2
6432.92685	6619.9121	6508.0033	7178.45836	7068.55244	7245.40795	PSME3
815.216571	794.07214	785.489776	988.465809	977.624911	956.246735	PSPH
16745.3666	16980.9273	18155.6605	19975.7957	19252.2608	20948.5463	PTBP1
6309.19753	6756.35606	6677.25906	7580.43446	7315.04365	7568.24339	PTDSS1
37.9170498	24.5916447	34.5663179	58.2096532	62.0861318	53.1248186	PTGER2
1139.50713	1174.05272	1189.55811	2490.93384	2360.19967	2499.93137	PTGER4
2847.77	2804.24077	2605.5852	2408.56169	2426.91909	2372.22748	PTGR1
332.273094	350.629257	367.118135	429.433479	406.803162	463.820532	PTK2B
758.340996	676.666869	699.669952	515.100516	492.055761	491.404572	PTK6
2919.61284	2880.39555	3102.62502	3367.37352	3387.86415	3701.36957	PTK7
6323.16697	6020.98656	6212.40169	6775.38397	7042.60599	6972.63244	PTMS
406.11156	344.283026	414.795815	317.407354	270.584336	293.208133	PTOV1-AS2
19541.2501	19735.1915	19391.7043	20824.778	20752.5212	20382.5626	PTP4A1
9699.78003	9754.15688	9717.9031	11036.7699	11103.2243	11053.0272	PTPN1
3360.64799	3580.86078	3699.78796	3788.02064	3827.10036	4165.1901	PTPN23
2740.00576	2747.91798	2826.09447	3068.63719	3209.01902	3187.48912	PTPN9__SNU

25700.7751	25588.003	27102.3771	28377.7551	28474.368	30531.4462	PTPRF
38.9148669	63.4623089	73.9004038	43.9318137	29.6530779	28.6056716	PTPRH
3174.0562	3066.0228	3202.74814	2671.05428	2534.4115	2791.09624	PTPRU
63.8602944	60.2891935	56.0212738	10.9829534	16.6798563	12.2595735	PTPRVP
1744.18429	1827.7145	1741.42726	1953.86742	2171.1613	2041.21899	PTTG1
5296.41317	5272.13131	5200.44293	5774.83692	6155.79363	6267.70696	PUM1
9449.32794	9666.10292	9698.83203	10517.2762	10513.8694	10434.9403	PUM2
2535.45325	2429.01987	2339.78214	2635.90882	2645.61054	2726.73348	PURA
3544.24634	3363.50237	3653.30222	3812.18314	3957.75924	4026.24827	PVRL1
7492.60861	7379.87325	7921.64651	9142.21044	9355.54607	9739.20953	PXDN
66.8537457	67.4287032	78.6681718	28.5556789	27.7997605	37.8003517	PYGM
7820.89043	7783.65219	7987.20332	8598.55424	8550.27967	8568.42026	PYGO2__SH(
1418.89592	1475.49868	1454.16924	1175.17602	1269.5224	1352.63961	PYROXD2
3865.54345	3856.12854	4004.92511	4125.19731	4232.97687	4249.98549	QRICH1
8189.08494	8042.2611	8334.05844	6365.71981	6347.61198	6566.02325	QSOX1
2096.41373	2027.62077	2020.34168	2280.06113	2305.5268	2295.60514	QSOX2
3734.82941	3724.44425	3706.93961	3931.89733	4164.40412	4202.99046	R3HDM1
815.216571	838.495756	841.51105	507.412449	489.275785	456.669114	RAB11FIP1P1
733.395569	686.186215	717.549082	622.73346	602.328144	628.303143	RAB11FIP2
2065.4814	2068.07799	2200.32493	1887.9697	1916.33016	1962.5534	RAB11FIP5_
1830.99438	1865.79188	1846.31815	1536.51519	1385.35473	1414.95911	RAB12
4845.39984	4898.49697	4521.03599	5625.46875	6000.11497	5577.08432	RAB13
12023.6961	11857.9324	12151.8487	13562.8492	13764.5881	13658.1865	RAB1B
677.517811	613.20456	680.59888	527.181765	458.696048	500.599252	RAB26
1563.5794	1593.69723	1582.89897	1269.62942	1326.04858	1257.62792	RAB27B
2502.52529	2499.62169	2520.95732	3336.62125	3444.39033	3411.22633	RAB29
3579.16994	3504.70601	3656.87805	4093.34675	4006.87215	4185.62273	RAB35
461.989318	501.35224	474.392915	657.878911	643.101126	682.449593	RAB39B
7758.02796	7952.62058	7851.32193	8286.63837	8356.60801	8505.07913	RAB5B
114.748967	157.862493	126.345852	82.3721508	86.1792575	85.8170147	RAB6C-AS1
5707.51381	5858.36439	5714.16993	6473.35275	6897.12058	6831.64735	RABEP1
2014.59273	2054.59225	2128.80841	2388.79237	2409.31258	2242.48032	RABIF
399.12684	376.01418	357.582599	244.919862	284.484216	245.191471	RABL2A
686.498165	745.68213	733.044328	620.536869	627.347929	539.421235	RABL2B
1603.49208	1665.09233	1659.18326	1447.55326	1516.94026	1397.59138	RABL3
6425.94213	6664.33571	6419.79959	7882.46568	8013.74429	7798.11039	RACGAP1
20906.2639	21556.5598	21260.6694	23034.5482	23109.0142	23052.0848	RAD21
212.535042	209.425619	215.741501	121.910783	139.925461	128.725522	RAD51-AS1
533.832149	529.910279	532.798072	618.340278	666.267593	622.173356	RAD52
1024.75816	1027.29613	1131.15296	1198.24022	1297.32216	1278.06054	RAD54L
1631.43096	1660.33266	1681.83016	1368.476	1259.32915	1386.35344	RAD9A
422.076634	421.231075	491.080103	606.25903	627.347929	630.346405	RAI14
3852.57183	3800.59902	3751.04146	4042.82516	4340.46927	4826.18544	RALGAPA2

1572.55975	1648.43347	1717.58842	1341.01861	1351.06836	1410.87259	RALGPS1
6013.84366	5843.29209	6201.67421	6806.13624	6442.13117	6622.21297	RALY
25980.1638	26464.5761	25259.6348	29683.6283	29478.866	28327.7879	RAN
4457.24899	4446.32802	4724.85807	5093.8938	5042.87655	5087.72301	RANBP9
6344.12112	6220.89283	6621.23779	7607.89184	7041.67933	7676.53629	RANGAP1
1003.804	1034.43564	1096.58664	811.640259	758.006803	796.872279	RAP1GAP
3824.63295	3977.50021	4096.70464	4593.07113	4235.75684	4441.03051	RARG
1592.51609	1544.51394	1561.44402	1884.67481	1941.34994	2045.30552	RASEF
1467.78896	1522.30214	1475.62419	1015.92319	1015.61792	986.895669	RASGRP1
48.8930379	49.1832894	65.5568098	76.880674	89.8858923	77.6439656	RASL10B
91.7991732	88.0539536	87.0117657	231.740318	227.031377	229.867004	RASSF2
2173.24565	2229.1136	2194.36522	2384.39919	2585.37773	2485.62853	RASSF8
4113.00209	4234.52256	4004.92511	4495.32284	4475.76144	4455.33334	RB1
2918.61502	2901.81407	2902.37876	3311.36046	3322.07138	3464.35115	RBBP5
7274.08666	7228.35698	7247.00734	6144.96245	6096.48748	6206.4091	RBBP6
2280.01207	2293.36919	2250.38649	2460.18157	2547.38472	2558.16434	RBBP8
3807.67006	3842.6428	4054.98667	3399.22409	3262.76522	3567.5359	RBCK1
1310.13385	1329.53537	1334.97504	1076.32944	1229.67607	1177.94069	RBL1
4854.38019	5085.71078	5046.68241	5780.32839	5999.18832	6296.31264	RBL2
4854.38019	4823.92876	4869.08306	5435.46365	5235.62156	5389.10419	RBM10
13870.6555	13807.0186	14325.9509	16352.5194	15764.3175	15995.6786	RBM14__RB
3254.87938	3091.40772	3361.27643	3551.88714	3550.02942	3715.67241	RBM15B
6853.00785	6785.70738	6692.75431	5332.22389	5247.66812	4957.97586	RBM3
4296.60043	4520.89623	4963.24647	5950.56417	4987.27703	5808.99459	RBM38
13226.0657	13663.4351	13582.1791	12786.3544	12667.4242	12833.7302	RBM39
2040.53597	2052.21241	1971.47206	1589.23336	1418.71444	1455.82436	RBMS1
3699.90581	3622.90456	3686.6766	3520.03658	3347.09116	3332.56074	RBPJ
2195.19762	2214.83458	2325.47884	2128.49638	1912.62352	2071.86793	RBPMS
741.378106	657.628176	661.527808	786.379466	841.406084	919.468014	RCAN3
14355.5946	14012.4778	14112.5932	16035.112	15974.669	16097.8417	RCC2
2531.46198	2412.36102	2706.90027	2203.18046	2121.12173	2068.80303	RCCD1
163.642005	180.074302	175.215474	248.214748	214.058156	207.391119	RCOR2
18631.2409	18575.4178	18253.3997	11657.3068	11712.0391	11776.342	RDX
399.12684	455.342066	361.158425	119.714192	112.125701	89.9035392	REC8
7863.79657	7761.44038	8257.77415	9301.46326	8383.48111	9198.76667	REPIN1__RP
3883.50416	3816.4646	4027.57201	4177.91549	4553.60077	4733.21701	RERE
7518.55185	7653.55445	7994.35497	6789.66181	6994.41974	7259.71079	RET
4641.84515	4866.76581	4614.00747	4881.9228	5267.12796	5364.58505	REV3L
1505.706	1547.68706	1607.92975	1472.81406	1201.87631	1373.07224	RFNG
7205.23728	7277.54027	7161.18751	7750.67024	7687.56044	7733.74763	RFWD3
1323.10548	1343.02111	1363.58164	1677.09699	1496.55377	1685.69136	RFX1
4044.15271	4213.10403	4220.66661	4868.74326	4911.29102	4802.68793	RFX5
1727.2214	1729.34792	1801.02436	2045.02593	1937.64331	2014.65658	RFXANK

305.332033	326.830891	302.753267	392.091438	390.123306	403.544295	RGAG4
7141.37699	7072.08105	7370.96931	6406.35674	6161.35359	6415.84348	RGL2
44.9017695	57.9093569	64.3648678	25.2607929	41.6996408	27.5840404	RGPD5
143.685663	157.862493	159.720228	91.1585135	81.5459641	69.4709166	RGS11
453.008964	488.659779	481.544567	246.018157	244.637892	239.061684	RGS16
1562.58158	1651.60659	1722.35619	2251.50545	1998.80278	2112.73317	RGS19
56.8755747	62.66903	58.4051578	121.910783	108.419066	105.228006	RGS9BP
1840.97255	1711.1025	1886.84418	2072.48331	1887.60374	2048.37041	RHBDF2
165.637639	157.069215	172.83159	101.043172	80.6193054	53.1248186	RHBDL1
285.375691	291.133342	275.338601	195.496571	215.911473	183.893603	RHBG
2909.63467	3081.0951	3025.14879	3540.90419	3609.33557	3691.15326	RHNO1__TU
3724.85124	3787.90656	3894.0745	3387.14284	3167.31938	3244.70046	RHOD
5191.64237	5347.4928	5580.67243	4647.98589	4173.67071	4419.57626	RHOT2
1750.17119	1672.23184	1731.89172	2259.19352	2365.75962	2394.70336	RHOU
777.299521	761.547707	792.641428	679.844818	637.541174	647.714135	RHOV
3957.34262	4002.09186	4293.37507	3393.73261	3039.44048	3393.8586	RHPN1
458.995866	445.82272	417.179699	667.763569	665.340935	598.67584	RILPL2
373.183596	349.042699	371.885903	266.885768	253.904479	309.554232	RIMKLA
490.926013	515.63126	485.120393	836.901052	747.813557	808.110221	RIN3
2545.43142	2467.89054	2407.72283	1923.11515	2034.01581	2049.39204	RIOK3
2070.47048	2054.59225	2256.3462	2577.69917	2293.48024	2381.42216	RITA1
272.404068	310.172035	286.066079	428.335184	470.742611	406.609189	RLN2
3887.49542	4055.24154	3792.75943	4369.01888	4346.95588	4361.34328	RMND5A
10659.6801	10375.2942	10891.966	10019.7484	9604.81725	9805.61556	RMND5B
71.8428312	68.2219821	95.3553597	43.9318137	40.7729821	42.9085073	RN7SL674P
3495.3533	3523.7447	3373.19585	3768.25132	3842.85356	3837.24651	RNASEH2A
3870.53253	3841.05625	3851.16459	3402.51897	3298.90491	3254.91677	RNASET2__F
8576.23798	8561.06547	8816.79495	9392.62178	9698.40978	10049.7854	RNF10
1409.91556	1497.71049	1413.64321	1614.49416	1686.5188	1698.97256	RNF111
1553.60123	1606.38969	1485.15973	1701.25949	1750.45825	1691.82115	RNF125__RN
5155.72096	5286.41033	5131.3103	6269.06982	6028.84139	5961.21763	RNF130
349.235985	441.856326	367.118135	224.05225	242.784575	240.083315	RNF139-AS1
1956.71933	1857.06581	1834.39873	2294.33897	2478.81198	2524.45052	RNF150
209.541591	241.950053	200.246255	72.4874927	82.4726228	79.6872279	RNF183
5575.80196	5642.59254	5946.59862	6645.78512	6372.63177	6583.39098	RNF187
516.869258	535.463231	542.333608	287.75338	277.997605	302.402814	RNF207
1562.58158	1627.80822	1662.75909	1288.30044	1149.98343	1248.43324	RNF223
81.8210022	72.1883764	78.6681718	48.3249951	50.9662276	51.0815564	RNF224
2649.2044	2698.73469	2621.08045	3747.38371	3710.34137	3706.47773	RNF24
2735.01667	2866.11653	3141.9591	3607.9002	3376.74424	3609.42277	RNF26
2005.61237	1969.71141	1901.14748	2420.64294	2437.11234	2345.66507	RNF34
5513.9373	5381.60379	5772.57509	5976.92326	6126.14056	6483.27113	RNF40
2473.58859	2382.21642	2424.41002	3090.6031	3124.69308	3094.52068	RNF41

3858.55873	3968.77414	3851.16459	4109.82118	4359.92911	4174.38479	RNF6
4569.0045	4570.8728	4996.62085	5533.21194	5405.2001	5561.75986	RNPEP
246.460824	198.319715	252.691703	137.286918	146.412072	162.439349	RORA
68.8493799	61.0824723	48.8696219	37.3420417	22.2398084	32.6921961	RP1-266L20.[illegible]
19.956342	29.3513179	20.2630139	5.49147672	2.77997605	5.10815564	RP1-60O19.1
50.8886721	51.563126	64.3648678	20.8676115	33.3597126	36.7787206	RP11-1000B[illegible]
14.9672565	15.8655772	14.303304	1.09829534	3.70663473	4.08652451	RP11-100G1[illegible]
209.541591	185.627254	210.973733	164.744302	126.95224	124.638998	RP11-1020A[illegible]
774.30607	624.310464	653.184214	443.711319	497.615713	507.75067	RP11-104N1[illegible]
151.668199	152.309541	206.205965	96.6499902	81.5459641	88.881908	RP11-1055B[illegible]
29.934513	27.7647601	17.87913	8.78636275	10.1932455	2.04326225	RP11-106D4.[illegible]
105.768613	107.092646	97.7392437	68.0943113	64.8661078	52.1031875	RP11-1072A[illegible]
936.950257	930.516104	845.086876	347.061329	337.303761	314.662387	RP11-1100L3[illegible]
51.8864892	67.4287032	79.8601138	23.0642022	21.3131497	29.6273027	RP11-127I20[illegible]
133.707491	109.472483	102.507012	146.073281	207.571545	158.352825	RP11-1281K[illegible]
48.8930379	38.0773853	38.1421439	17.5727255	22.2398084	15.3244669	RP11-1379J2[illegible]
26.9410617	36.4908276	30.9904919	18.6710208	12.9732216	13.2812047	RP11-138H1[illegible]
303.336399	287.166948	311.096861	210.872706	169.578539	191.045021	RP11-1399P[illegible]
182.600529	180.074302	184.751009	140.581804	106.565749	129.747153	RP11-142E9.[illegible]
6268.28702	6216.13316	6218.3614	6836.88851	6684.91574	6792.82536	RP11-147L13[illegible]
880.074683	717.124091	742.579864	747.939129	615.301366	560.875489	RP11-147L13[illegible]
14.9672565	30.1445967	10.727478	8.78636275	3.70663473	4.08652451	RP11-147L13[illegible]
22.9497933	27.7647601	23.8388399	14.2778395	4.63329342	9.19468014	RP11-147L13[illegible]
555.784125	579.886848	547.101376	496.429495	431.822946	388.219828	RP11-156E6.[illegible]
118.740235	91.227069	70.3245778	194.398276	174.211832	164.482611	RP11-156L14[illegible]
389.148669	341.10991	362.350367	429.433479	492.98242	432.149967	RP11-156P1.[illegible]
659.557103	631.449974	563.788564	498.626086	511.515593	433.171598	RP11-158K1.[illegible]
800.249315	779.793121	777.146182	619.438574	619.008	555.767333	RP11-180C1[illegible]
4987.08987	5201.52949	5086.0165	5557.37444	5681.34439	5779.36729	RP11-192H2[illegible]
137.69876	188.00709	150.184692	94.4533995	71.3527186	105.228006	RP11-192H2[illegible]
1424.88282	1364.43964	1412.45127	1427.78395	1679.10553	1869.58496	RP11-192H2[illegible]
272.404068	213.392014	195.478487	183.415322	137.145485	120.552473	RP11-196G1[illegible]
160.648553	122.958224	101.31507	84.5687414	77.8393294	55.1680809	RP11-196H1[illegible]
119.738052	118.19855	120.386142	59.3079485	54.6728623	70.4925478	RP11-199F1[illegible]
719.426129	687.772773	672.255286	476.660179	486.495809	445.431171	RP11-203J24[illegible]
89.803539	80.121165	83.4359398	38.440337	46.3329342	57.2113431	RP11-203M5[illegible]
1837.9791	1754.73284	1686.59793	1429.98054	1422.42108	1288.27685	RP11-205M5[illegible]
268.4128	284.787111	271.762775	184.513618	204.791569	159.374456	RP11-21L23.[illegible]
1497.72347	1634.94773	1700.90123	974.18797	874.765797	956.246735	RP11-244F1[illegible]
210.539408	180.074302	205.014023	105.436353	128.805557	102.163113	RP11-244F1[illegible]
54.8799405	38.8706642	41.7179699	101.043172	82.4726228	104.206375	RP11-248C1.[illegible]
636.60731	641.762599	637.688968	530.476651	454.062755	463.820532	RP11-252E2.[illegible]
2046.52287	2042.69307	1878.50059	2112.02195	2301.82017	2261.89132	RP11-259O2[illegible]

39.912684	35.6975488	30.9904919	1.09829534	2.77997605	0	RP11-260A9.
75.8340996	65.8421455	107.27478	60.4062439	47.2595928	38.8219828	RP11-265D1
317.305838	303.825804	314.672687	186.710208	260.39109	256.429413	RP11-274B2
435.048256	427.577306	486.312335	299.834629	258.537773	320.792174	RP11-274B2
122.731503	130.097733	165.679938	84.5687414	74.1326947	81.7304902	RP11-274B2
53.8821234	47.5967317	48.8696219	26.3590882	30.5797366	26.5624093	RP11-290L1.
1760.14937	1768.21858	1841.55038	2135.08615	2146.14151	2074.93282	RP11-293I14
200.561237	180.86758	152.568576	129.598851	118.612312	106.249637	RP11-308D1
26.9410617	23.005087	32.1824339	8.78636275	5.5599521	9.19468014	RP11-319G9.
45.8995866	52.3564048	54.8293318	23.0642022	29.6530779	27.5840404	RP11-321G1
1215.34123	1174.84599	1314.71202	1411.30952	1435.3943	1407.80769	RP11-330H6.
351.231619	308.585477	355.198715	218.560773	260.39109	207.391119	RP11-332H1
407.109377	407.745335	443.402423	248.214748	275.217629	313.640756	RP11-334C1
422.076634	445.82272	454.129901	271.27895	253.904479	304.446076	RP11-342D1
177.611444	171.348234	151.376634	107.632944	111.199042	80.708859	RP11-342K2.
207.545957	206.252504	188.326835	253.706224	238.151282	273.797142	RP11-347C1
271.406251	249.089562	241.964225	116.419306	147.338731	135.87694	RP11-347E1
463.984952	394.259594	418.371641	246.018157	277.070946	304.446076	RP11-347I19
617.648785	543.39602	595.970998	253.706224	340.083737	286.056716	RP11-34P13.
26.9410617	34.9042699	42.9099119	14.2778395	11.1199042	10.2163113	RP11-354E1
163.642005	133.270849	123.961968	90.0602182	123.245605	67.4276544	RP11-358L2
290.364776	264.95514	251.499761	139.483509	145.485413	157.331194	RP11-366L5.
1753.16465	1736.48743	1754.53862	2147.1674	2011.776	2021.808	RP11-367J11
292.36041	287.166948	297.985499	176.82555	165.871904	144.049989	RP11-379B1
238.478287	272.887928	314.672687	451.399386	312.283976	402.522664	RP11-379F1
997.8171	992.391855	1040.56536	1098.29534	1175.92987	1259.67118	RP11-379H1
999.812735	911.477412	994.079625	929.157861	816.3863	777.461288	RP11-379K1
635.609493	648.10883	587.627404	433.826661	471.66927	459.734007	RP11-37B2.1
29731.9561	30553.1353	30395.7129	35818.706	34858.1197	35701.9214	RP11-386G1
82.8188193	88.8472325	63.1729258	26.3590882	25.0197845	21.4542537	RP11-386M2
18.9585249	21.4185293	16.687188	8.78636275	0.92665868	1.02163113	RP11-389G6.
865.107426	929.722825	842.702992	658.977206	722.793773	694.709166	RP11-395N3.
757.343179	756.788034	727.084618	1048.87205	983.184863	1111.53467	RP11-400F1
20.9541591	16.6588561	16.687188	5.49147672	4.63329342	1.02163113	RP11-416N2.
41.9083182	47.5967317	42.9099119	23.0642022	21.3131497	27.5840404	RP11-427L15
572.747016	641.762599	610.274302	472.266998	516.148887	467.907056	RP11-434D9.
178.609261	176.901186	201.438197	330.586898	255.757797	273.797142	RP11-438D1
5977.92225	5937.69228	6002.61989	6521.67775	6848.00767	6713.13814	RP11-438J1.
72.8406483	62.66903	56.0212738	32.9488603	30.5797366	34.7354583	RP11-439E1
360.211973	378.394017	361.158425	521.690288	497.615713	506.729039	RP11-440D1
606.672797	695.705561	713.973256	519.493697	522.635497	545.551022	RP11-449P15
63.8602944	57.9093569	59.5970998	35.145451	38.9196647	29.6273027	RP11-44F14.
39.912684	32.5244333	45.2937959	10.9829534	20.386491	11.2379424	RP11-452H2

287.371325	276.061044	305.137151	490.938019	356.763593	381.06841	RP11-455O6.
133.707491	126.924618	110.850606	77.9789694	84.3259402	58.2329742	RP11-457M1
840.161999	860.707564	874.885425	665.566978	581.014994	666.103495	RP11-458F8.
554.786308	516.424539	555.44497	427.236889	366.956839	374.938624	RP11-459E5.
4182.84929	4378.89931	4343.43664	4735.84952	4578.62055	4604.49149	RP11-463D19
295.353862	253.849236	277.722485	200.988048	182.551761	183.893603	RP11-465B22
150.670382	157.862493	184.751009	129.598851	93.592527	124.638998	RP11-465B22
325.288375	325.244333	323.016281	200.988048	214.058156	178.785447	RP11-468E2.
48.8930379	40.4572219	46.4857379	15.3761348	17.606515	14.3028358	RP11-471B22
18.9585249	9.51934634	20.2630139	6.58977206	6.48661078	3.06489338	RP11-479O9.
250.452092	245.123168	232.428689	269.082359	341.010395	356.549263	RP11-47A8.5
1088.61846	1179.60567	1156.18374	668.861864	749.666875	809.131853	RP11-480A16
285.375691	264.161861	283.682195	191.10339	175.138491	158.352825	RP11-481C4.
821.203474	748.061966	712.781314	574.408465	660.707641	536.356342	RP11-483I13
18.9585249	16.6588561	36.9502019	8.78636275	7.41326947	7.15141789	RP11-486L19
333.270912	283.200554	264.611123	193.29998	194.598324	176.742185	RP11-486O1
514.873624	533.083395	507.767291	412.959049	341.010395	391.284722	RP11-488L18
80.8231851	106.299367	89.3956497	40.6369277	26.8731018	30.6489338	RP11-48B3.4
315.310204	289.546784	308.712977	195.496571	138.998803	142.006727	RP11-496H1.
372.185778	360.941882	379.037555	148.269871	128.805557	137.920202	RP11-496I9.1
570.751381	774.240169	727.084618	1177.37261	918.318755	984.852406	RP11-498C9.
119.738052	126.924618	78.6681718	83.4704461	70.4260599	67.4276544	RP11-498C9.
1012.78436	1089.96516	1095.3947	903.897068	770.053366	828.542844	RP11-4K16.2
251.449909	214.185293	219.317327	272.377245	281.70424	312.619125	RP11-500C11
164.639822	192.766763	165.679938	59.3079485	83.3992815	99.0982193	RP11-504P24
463.984952	445.029441	432.674945	154.859643	180.698443	132.812047	RP11-522B15
21.9519762	19.8319715	19.0710719	1.09829534	0.92665868	4.08652451	RP11-522B15
436.046073	472.794201	432.674945	641.404481	658.854324	679.384699	RP11-524H19
20.9541591	34.110991	19.0710719	56.0130625	63.0127905	68.4492855	RP11-527N2
280.386605	314.931708	287.258021	349.257919	388.269988	408.652451	RP11-539I5.1
58.8712089	51.563126	73.9004038	25.2607929	25.0197845	24.5191471	RP11-563J2.2
80.8231851	69.0152609	89.3956497	18.6710208	15.7531976	16.346098	RP11-563J2.3
21.9519762	27.7647601	23.8388399	37.3420417	50.0395689	54.1464497	RP11-564A8.
800.249315	765.514101	901.108149	935.747633	1048.05097	1072.71268	RP11-56G10.
448.019878	450.582393	450.554075	262.492587	267.80436	214.542537	RP11-574K11
136.700943	159.449051	159.720228	77.9789694	60.2328144	55.1680809	RP11-609D2
1497.72347	1485.81131	1473.24031	570.015283	581.014994	503.664146	RP11-609N14
626.629139	548.155693	576.899926	461.284044	493.909078	440.323016	RP11-617F23
24.9454275	24.5916447	22.6468979	53.8164718	41.6996408	38.8219828	RP11-624G17
176.613627	153.10282	193.094603	115.321011	133.43885	135.87694	RP11-631N16
4326.53495	4353.51439	4277.87983	4024.15414	4093.0514	3888.32807	RP11-637O19
1490.73875	1519.9223	1493.50332	1344.3135	1239.86932	1175.89743	RP11-638I2.6
52.8843063	66.6354243	61.9809838	83.4704461	101.005797	98.0765882	RP11-657O9.

3476.39478	3435.69075	3455.43985	1560.67768	1579.0264	1488.51655	RP11-662G2
314.312387	280.820717	238.388399	195.496571	208.498204	181.850341	RP11-666A8.
1151.48093	1103.4509	1207.43724	753.430606	693.140695	728.422994	RP11-66N24.
697.474153	779.793121	785.489776	556.835739	578.235018	550.659177	RP11-68L18.
212.535042	176.107907	203.822081	152.663053	146.412072	130.768784	RP11-705C1
160.648553	137.237243	123.961968	86.7653321	85.2525989	87.8602769	RP11-707P1
820.205657	778.999842	791.449486	380.010189	354.910276	287.078347	RP11-72I8.1
312.316752	300.652688	275.338601	354.749396	446.649485	437.258122	RP11-734I18
176.613627	201.492831	159.720228	119.714192	84.3259402	83.7737524	RP11-747H7.
48.8930379	53.1496837	77.4762298	32.9488603	34.2863713	21.4542537	RP11-75C10.
78.8275509	85.674117	81.0520558	48.3249951	50.9662276	58.2329742	RP11-77P16.
50.8886721	72.9816552	76.2842878	34.0471557	35.21303	26.5624093	RP11-798M1
91.7991732	69.8085398	77.4762298	47.2266998	52.8195449	37.8003517	RP11-7K24.3
760.336631	799.625092	809.328616	488.741428	419.776384	451.560958	RP11-800A3.
295.353862	304.619083	299.177441	226.248841	218.691449	238.040053	RP11-801F7.
309.323301	302.239246	278.914427	362.437463	395.683258	371.87373	RP11-817O1
472.965306	526.737164	513.727001	353.651101	398.463234	354.506001	RP11-81A1.6
188.587432	266.541697	215.741501	410.762458	380.856719	359.614157	RP11-829H1
250.452092	167.38184	216.933443	126.303965	154.752	126.68226	RP11-841O2
50.8886721	49.1832894	51.2535059	28.5556789	24.0931258	27.5840404	RP11-848G1
6175.49003	6087.62198	6593.82312	7058.74417	7721.84681	8425.3919	RP11-849H4.
120.735869	103.126252	101.31507	62.6028346	52.8195449	59.2546054	RP11-855A2.
472.965306	517.217818	548.293318	812.738554	736.693653	737.617674	RP11-865I6.
249.454275	239.570216	221.701211	158.154529	150.118707	137.920202	RP11-87H9.4
398.129023	455.342066	439.826597	343.766443	322.477222	309.554232	RP11-88H9.2
181.602712	115.025435	147.800808	110.92783	109.345725	85.8170147	RP11-95D17.
61.8646602	47.5967317	84.6278818	24.1624976	38.9196647	37.8003517	RP11-981P6.
1467.78896	1617.4956	1647.26384	1454.14304	1411.30118	1329.1421	RP13-1032I1
481.94566	448.995835	464.857379	352.552805	329.890491	292.186502	RP13-104F2
255.441178	235.603822	320.632397	131.795441	112.125701	135.87694	RP13-39P12.
201.559054	217.358408	220.509269	84.5687414	92.6658683	84.7953835	RP13-39P12.
46.8974037	53.9429626	58.4051578	26.3590882	27.7997605	32.6921961	RP13-516M1
1313.1273	1323.18914	1288.4893	599.669258	611.594731	666.103495	RP3-368A4.5
502.899819	487.8665	451.746017	238.33009	255.757797	250.299626	RP3-368A4.6
53.8821234	58.7026357	57.2132158	28.5556789	25.9464431	15.3244669	RP3-406P24.
215.528494	220.531523	257.459471	161.449416	170.505198	144.049989	RP3-416H24.
89.803539	86.4673959	76.2842878	54.9147672	50.9662276	46.9950318	RP4-635E18.
107.764247	134.064128	141.841098	86.7653321	75.0593533	65.3843921	RP4-669L17.
499.906367	527.530443	519.68671	185.611913	231.664671	206.369488	RP4-717I23.
446.024244	361.735161	383.805323	251.509634	317.843928	294.229765	RP4-724E16.
737.386837	812.317554	892.764555	977.482856	889.592336	942.96553	RP4-734G22
46.8974037	26.9714813	47.6776799	13.1795441	16.6798563	10.2163113	RP4-740C4.7
91.7991732	75.3614918	87.0117657	45.0301091	49.1129102	54.1464497	RP4-798A10.

298.347313	291.926621	271.762775	241.624976	212.204839	213.520906	RP4-798A10.
1535.64052	1646.84692	1654.41549	2063.69695	2186.91449	2183.22572	RP4-816N1.7
413.09628	432.336979	435.058829	563.425511	558.775186	547.594284	RP5-1024G6.
357.218522	309.378756	337.319585	196.594867	142.705437	129.747153	RP5-1074L1.
51.8864892	62.66903	59.5970998	41.7352231	18.5331737	23.4975159	RP5-1085F17
32.9279643	38.0773853	15.495246	8.78636275	10.1932455	10.2163113	RP5-1120P11
173.620176	172.934792	170.447706	107.632944	106.565749	105.228006	RP5-1125A1
2429.68464	2419.50053	2596.04967	2043.92763	1805.13112	2043.26225	RP5-1142A6.
230.49575	268.921534	269.378891	157.056234	176.06515	139.963464	RP5-1159O4.
1493.7322	1418.3826	1481.5839	1813.28561	1830.1509	1805.2222	RPIA
55261.1067	54119.0705	52193.9481	58175.606	60920.3952	56772.0417	RPL10
73146.9782	72516.7938	74484.4554	80094.2862	77710.5238	78100.6348	RPL13
43470.8998	44111.0644	43138.7647	47042.1862	45575.854	45325.6866	RPL15
13272.9631	13215.2326	12764.5068	13448.6265	15405.7006	14066.839	RPL27A
60717.1706	60742.949	61590.0268	68617.0999	68595.909	68418.6366	RPL3
89985.1418	93441.9036	92525.6894	96232.638	97831.9905	97850.8077	RPL4
35957.337	37161.9415	36503.2236	40783.001	38960.4377	39573.9033	RPL6
71572.4228	74028.7833	74041.0529	83825.1955	82914.639	83677.7191	RPLP0
14715.8066	14896.9837	15081.6421	16129.5654	15719.8379	16436.0016	RPN1
16403.1153	17038.0434	17802.8457	19414.5668	18730.552	20116.9385	RPN2
1486.74748	1474.7054	1535.22129	2028.5515	1969.1497	2098.43034	RPP25
802.244949	799.625092	834.359398	948.927177	995.231426	935.814112	RPP25L
5593.76267	5561.6781	5579.48049	6224.03971	6178.03344	5983.69351	RPRD1A
7439.7243	7481.41294	7498.5071	8654.56731	9134.07464	9076.17093	RPRD2
69400.175	68855.0186	71285.283	82423.7706	77641.0244	79931.3977	RPS2
4797.50462	5025.42159	4864.31529	5682.58011	6428.23129	5926.48217	RPS2P5
95914.171	94283.5725	90860.5464	105361.669	114101.337	107112.916	RPS3
32011.9682	33196.3405	32176.4742	34783.0135	34152.9324	33762.8655	RPS3A
59168.5584	61291.1047	58692.4159	62851.0493	64714.1358	62053.8747	RPS4X
29363.7616	30311.1853	30215.7296	34553.4698	33455.1584	34337.0222	RPS5
61911.5576	61790.8703	59162.041	64423.8083	68987.8857	62583.0796	RPS6
2955.53425	2978.76212	2789.14427	1875.88845	1894.09035	2017.72148	RPS6KA3
26329.3998	25556.2718	25173.815	27359.6353	27959.1458	26413.2512	RPS9
41195.8768	41640.0007	41060.0179	48008.6861	47483.8443	47015.4645	RPSA
584.720821	607.651608	611.466244	474.463588	429.04297	428.063442	RRAGD
4982.10078	5316.55493	5260.04003	5718.82385	5828.68312	5713.98289	RRM1
5501.96349	5515.66792	5281.49499	6184.50108	6120.5806	6078.70521	RRM2B
4660.80368	4614.50314	4550.83454	4797.35406	4971.52384	5081.59323	RRP1B
573.744833	520.390933	579.28381	724.874927	707.040575	763.158452	RTKN
11007.9183	11603.2899	11377.0864	12414.0323	12098.4558	11975.5601	RTN3
3981.29023	4029.06334	3932.21665	2766.60597	2712.32997	2584.72675	RUNX1
457.000232	411.711729	436.250771	257.00111	270.584336	258.472675	RUNX2
2103.39845	2202.9354	2119.27287	1869.29867	1795.86453	1724.51334	RUSC1-AS1

4698.72073	4882.63139	4767.76799	5170.77448	5233.76824	5210.31875	RUVBL1
17399.9346	17567.1604	15626.3596	10476.6393	12435.7595	10632.1151	S100A10
6726.28507	6597.70029	6103.93496	3637.55418	3947.56599	3416.33449	S100A14
4525.10055	4756.50005	4766.57604	2441.51055	2325.9133	2390.61684	S100A16
1151.48093	984.459067	804.560848	555.737444	728.353725	521.031875	S100A6
103.772978	86.4673959	71.5165198	38.440337	46.3329342	40.8652451	S100A7
28.9366959	23.7983658	33.3743759	12.0812488	10.1932455	14.3028358	S100A7A
2555.40959	2632.89254	2396.99536	2116.41513	2215.64091	2049.39204	S100A9
1876.89397	1632.5679	1538.79712	1120.26125	1291.7622	1093.14531	S100P
163.642005	139.61708	158.528286	232.838613	215.911473	262.5592	S1PR5
833.177279	864.673959	817.67221	1074.13285	1267.66908	1313.81763	SACS
8571.24889	8754.62551	8736.93483	10095.5308	10077.4132	10282.7173	SAE1
595.696809	664.767686	631.729258	774.298217	830.28618	919.468014	SALL2
1640.41131	1603.21658	1735.46755	1394.83509	1372.38151	1346.50983	SALL4
1413.90683	1356.50685	1665.14297	2001.09412	1745.82496	1967.66155	SAMD11
1478.76494	1272.41929	1379.07689	1089.50898	1162.02999	984.852406	SAMD12
700.467605	752.821639	835.55134	1025.80785	921.098731	999.155242	SAMD14
576.738284	593.372588	492.272045	674.353341	714.453845	662.01697	SAMD15
4776.55046	4723.18234	4940.59958	5466.21592	5412.61337	5794.69175	SAMD4B
3431.49301	3443.62354	3393.45886	3681.48599	3824.32039	3903.65254	SAP130
4666.79058	4697.79742	4792.79877	4510.69898	4270.04321	4192.77415	SAR1B
3790.70716	4030.64989	3855.93236	4288.84332	4386.80221	4568.7344	SART3
1702.27597	1689.68397	1619.84917	1313.56123	1303.80877	1232.08714	SAT1
6539.69328	6446.97731	6292.2618	7047.76122	7457.74908	7333.26823	SBNO1
45.8995866	43.6303374	41.7179699	14.2778395	17.606515	14.3028358	SBSN
6908.8856	7239.46289	7273.23006	8043.9151	7741.30664	8154.65966	SCAMP2
3009.41638	3113.61953	3379.15556	3886.86722	3575.0492	3941.45289	SCAMP3
1620.45497	1729.34792	1824.8632	2380.00601	2415.79919	2483.58527	SCAMP5
3594.1372	3551.50946	3867.85178	4177.91549	4048.57179	4234.66102	SCAP
995.821466	951.141355	945.210003	803.952191	831.212839	870.42972	SCAPER
753.351911	843.25543	954.745539	697.417543	683.874108	713.098527	SCARA3
5747.4265	5934.51916	6398.34464	7587.02423	7513.3486	7833.86748	SCARB1
7634.29864	8169.97899	7918.07068	7254.24074	7221.45112	7281.16504	SCARB2
267.414983	234.810543	251.499761	170.235778	158.458635	158.352825	SCARNA15__
270.408434	232.430706	199.054313	94.4533995	82.4726228	78.6655968	SCART1
5752.41558	6105.07412	5923.95172	4733.65293	4481.32139	4473.72271	SCCPDH
38962.7621	39688.5347	39025.3729	45768.1636	49437.2408	48554.0409	SCD
98.7838929	64.2555878	59.5970998	21.9659069	25.9464431	35.7570894	SCIN
973.86949	1070.92646	1065.59615	724.874927	706.113917	629.324774	SCML1
23.9476104	22.2118081	17.87913	41.7352231	46.3329342	50.0599252	SCN5A
618.646602	616.377675	584.051578	304.22781	308.577342	407.63082	SCNN1A
175.61581	134.857406	171.639648	66.996016	55.599521	55.1680809	SCNN1B
138.696577	153.896099	194.286545	85.6670368	72.2793773	75.6007034	SCNN1D

61.8646602	44.4236162	59.5970998	35.145451	25.9464431	20.4326225	SCNN1G
10297.4725	10207.1191	9980.13034	10724.854	11345.0823	11601.6431	SCRN1
1167.44601	1225.61584	1188.36617	1015.92319	854.379306	919.468014	SCRN2
474.96094	429.163864	470.817089	307.522696	221.471425	296.273027	SCX
5062.92397	5295.92968	5229.04954	5590.3233	5534.00566	5629.18751	SCYL2
2962.51897	2989.07475	3109.77667	2464.57475	2390.7794	2470.30407	SDR39U1
8001.49533	8349.26001	8156.45908	7819.86285	7405.8562	7468.12354	SEC22B
4283.62881	4432.049	4485.27773	4741.341	5139.24906	5524.98113	SEC24C
9097.0985	9390.04188	9533.15209	10042.8126	10074.6332	10556.5144	SEC31A
18100.4022	18522.2681	18953.0697	22054.8688	20822.9473	21985.5019	SEC61A1
208.543774	216.565129	184.751009	165.842597	133.43885	118.509211	SECTM1
3706.89053	3751.41574	3915.52946	3367.37352	3183.07258	3426.5508	SELENBP1
9.978171	5.55295203	8.34359398	21.9659069	19.4598324	34.7354583	SEMA3A
3842.59365	3718.8913	3956.05549	1941.78617	1641.11253	1804.20057	SEMA3B
3563.20487	3522.95142	3740.31399	3081.81673	2894.88173	3071.02317	SEMA3F
4341.5022	4405.8708	4580.63309	5367.36934	4988.20369	5314.52512	SEMA4B
8702.96075	8834.74668	9598.7089	7365.16857	6871.17414	7338.37639	SEMA4C
1437.85444	1382.68506	1482.77584	1572.75893	1622.57935	1589.65803	SEMA4D
1546.61651	1640.50069	1605.54587	1833.05493	1821.81097	1872.64986	SENP1
1628.43751	1656.36626	1594.81839	1283.90726	1368.67488	1332.20699	SENP7
5771.37411	5863.91734	5786.87839	6858.85442	7066.69912	6875.57748	SEPHS1
7034.61056	6945.15643	6923.99106	8240.50996	8206.4893	8267.03908	SEPHS2
539.819051	511.664866	561.40468	776.494808	659.780983	771.331501	SERINC2
81.8210022	78.5346073	67.9406938	98.8465809	119.53897	123.617366	SERPINB5
179.607078	181.660859	193.094603	253.706224	221.471425	238.040053	SERPINE1
9800.55956	9675.62227	10048.071	12563.4004	12188.3417	13056.4458	SERPINH1
864.109609	861.500843	907.067859	1182.86409	1003.57135	1092.12368	SERTAD1
797.255863	827.389852	779.530066	1066.44478	1085.11732	1153.42154	SERTAD2
1304.14695	1462.01294	1287.29736	1677.09699	1625.35933	1604.9825	SESN1
1343.06182	1394.58424	1457.74506	2206.47535	2097.95526	2164.83636	SESN2
5191.64237	5280.0641	5111.04728	5284.99719	5791.61677	5863.14104	SETD2
9127.03302	9110.80772	9090.94161	9718.81549	10449.93	10620.8772	SETD7
4172.87111	4153.60812	4429.25646	4800.64895	4712.0594	5070.35528	SEZ6L2
12056.624	11836.5139	12278.1945	12634.7896	13011.2146	13042.143	SF1
5777.36101	5927.37965	5834.55607	6177.91131	6502.36398	6700.87856	SF3A1
25551.1025	26191.6882	26499.2545	28430.4733	28720.8592	29859.213	SF3B3
10214.6537	10473.6608	10268.5803	11704.5335	11356.2022	11512.7612	SF3B4
912.00483	878.952978	934.482525	779.789694	733.913677	821.391426	SFMBT2
4571.00014	4323.36979	4494.81327	3805.59337	3945.71267	3852.57098	SFN
399.12684	404.572219	393.340859	336.078375	337.303761	291.164871	SFR1
3888.49324	4073.48695	3883.34702	5503.55797	5363.50046	5597.51694	SFXN2
1852.94636	1907.83566	1899.95554	1433.27542	1398.32795	1340.38004	SFXN3
1266.2299	1358.09341	1249.15521	1643.04983	1634.62592	1561.05236	SGCG

1435.85881	1487.39787	1542.37294	1272.9243	1328.82855	1412.91585	SGK223
2874.71107	3011.28656	3094.28142	3445.35249	3271.10515	3416.33449	SGMS1
1394.94831	1428.69523	1351.66222	1625.47711	1723.58515	1581.48498	SGOL1
2084.43992	2079.97717	1945.24934	2299.83045	2311.08676	2222.0477	SGOL2
6402.99233	6123.31953	6033.61039	7198.22768	7439.21591	7391.5012	SGPL1
1500.71692	1592.90395	1580.51509	2066.99184	2016.4093	2122.94948	SGPP1
6876.95546	7011.79185	7064.64021	6247.10391	5885.2093	6306.52895	SGSM2
2265.04482	2291.78263	2557.90752	2756.72131	2442.67229	2745.12284	SGTA
325.288375	384.740248	346.855121	447.006205	438.309557	461.777269	SH2B3
4482.19442	4762.84628	4530.57153	5699.05454	5317.16752	5219.51343	SH3BGRL
2589.33538	2523.42006	2430.36973	2739.14859	2902.295	2681.78171	SH3BGRL3
3338.69602	3403.95959	3457.82373	2770.99915	2507.5384	2687.9115	SH3BP2
812.22312	832.942804	773.570356	450.301091	438.309557	504.685777	SH3BP5-AS1
1473.77586	1452.4936	1488.73555	1961.55548	1734.70506	1833.82787	SH3BP5L
5727.47016	6153.46413	6070.56059	6355.83515	6582.05663	6627.32112	SH3D19
1006.79745	1077.27269	995.271567	488.741428	552.288575	577.221587	SH3D21
476.956574	506.905192	505.383407	478.85677	391.976623	337.138272	SH3GL1P3
11121.6694	11128.1159	12004.0479	9496.95983	8655.91876	9309.10283	SH3GLB2
1181.41545	1116.93664	1185.98229	1324.54418	1403.88791	1434.3701	SH3PXD2B
881.0725	839.289035	837.935224	1026.90615	1096.23722	1042.06375	SH3RF1
390.146486	333.970401	369.502019	470.070407	393.82994	467.907056	SHANK3
291.362593	299.85941	276.530543	253.706224	206.644886	243.148208	SHC2
1869.90925	1815.81531	1765.2661	2132.88956	2133.16829	2015.67821	SHCBP1
6983.72189	6499.33371	7098.01459	7411.29698	7222.37778	7513.07531	SHISA5
725.413032	728.229995	730.660444	444.809614	480.935857	468.928687	SHISA9
4019.20728	3964.80775	4407.8015	5167.47959	4611.98027	4955.9326	SHKBP1
5523.91547	5663.21779	5860.7788	8152.64633	7847.87239	8249.67135	SHMT2
2754.97301	2874.04931	2857.08497	3078.52185	3043.14712	3097.58558	SHOC2
2452.63443	2360.79789	2476.85547	2865.45255	2846.69548	3061.82849	SHROOM3
80.8231851	78.5346073	90.5875917	113.12442	114.905677	123.617366	SIGLEC15
1121.54642	1265.27978	1257.49881	1438.7669	1448.36752	1349.57472	SIGMAR1
7912.68961	7703.53102	7409.11145	6932.44021	7147.31842	6777.5009	SIKE1
34.9235985	34.110991	36.9502019	4.39318137	0.92665868	3.06489338	SIM1
8211.03692	8303.24984	8175.53015	9286.08713	10225.6786	10034.4609	SIN3A
3236.91867	3139.79773	3271.88078	2901.6963	2873.56858	2930.03807	SIN3B
2765.949	2832.00554	2915.49012	3104.88094	3288.71167	3366.27456	SIPA1L2
2049.51632	2118.05456	2193.17327	2426.13441	2340.73983	2339.53528	SIRT1
150.670382	174.52135	196.670429	293.244857	290.970827	292.186502	SIX2
2245.08848	2310.82132	2264.68979	2432.72419	2577.96446	2491.75832	SKAP2
37.9170498	36.4908276	46.4857379	30.7522696	16.6798563	16.346098	SKOR1
13.9694394	10.3126252	21.4549559	3.29488603	3.70663473	4.08652451	SLC10A4
1060.67958	1098.69122	1081.09139	1222.40272	1202.80297	1224.93572	SLC12A6
67.8515628	99.9531365	69.1326358	51.6198811	33.3597126	37.8003517	SLC15A3

485.936928	519.597654	449.362133	415.15564	370.663473	406.609189	SLC16A1
1016.77563	1038.40203	1004.8071	1098.29534	1285.27559	1192.24353	SLC16A2
60.8668431	58.7026357	59.5970998	37.3420417	25.9464431	27.5840404	SLC16A8
47.8952208	42.0437796	53.6373898	110.92783	117.685653	128.725522	SLC17A9
1437.85444	1463.5995	1430.3304	1598.01973	1524.35353	1663.21548	SLC20A2
3267.851	3305.59302	3273.07272	2476.656	2535.33816	2554.07782	SLC22A5
1044.7145	1137.56189	1045.33313	1236.68056	1292.68886	1255.58466	SLC25A15
1201.37179	1135.18205	1131.15296	971.991379	910.905486	825.477951	SLC25A16
14251.8216	14669.3127	14519.0455	11900.0301	11598.9867	12009.2739	SLC25A24
1252.26046	1194.67797	1237.23579	1029.10274	966.505007	902.100285	SLC25A25-AS
1052.69704	1050.30121	1014.34264	870.948207	838.626108	863.278302	SLC25A28
7446.70902	7456.02802	7929.9901	6813.82431	6508.85059	6854.12323	SLC25A29
1962.70624	1973.67781	2009.61421	2284.45431	2173.94127	2132.14416	SLC25A30
5419.14467	5426.02741	5395.92142	4347.05297	4522.09437	4370.53796	SLC25A36
3039.35089	2997.00754	2971.5114	3401.42068	3500.91651	3384.66392	SLC25A38
10796.381	10982.9458	11192.3354	12757.7987	11759.2987	12585.4739	SLC25A39
2851.76127	2994.6277	2882.11575	2539.25883	2481.59195	2511.16931	SLC25A46
34210.1593	35008.9827	34274.2921	36334.9049	36815.2228	36914.5975	SLC25A5
21008.0412	20741.0691	21497.8659	26298.682	25023.4911	25201.5966	SLC25A6
2797.87915	2645.585	2697.36474	1876.98674	1972.85634	1815.43851	SLC26A2
35.9214156	44.4236162	28.6066079	23.0642022	16.6798563	9.19468014	SLC28A3
1864.92016	1811.05564	2037.02887	2092.25263	2125.75502	2186.29061	SLC29A1
1062.67521	1117.72992	1131.15296	1483.79701	1431.68767	1462.97577	SLC29A3
123.72932	119.785108	83.4359398	50.5215858	72.2793773	66.4060233	SLC2A12
2995.44694	2980.34868	2985.8147	3398.12579	3247.93869	3344.82031	SLC30A1
4584.96958	4808.85646	4661.68515	5280.60401	5005.81021	5104.06911	SLC30A5
1105.58135	1062.99367	1078.70751	982.974332	866.425869	835.694262	SLC30A7
71.8428312	46.8034528	57.2132158	27.4573836	38.9196647	34.7354583	SLC34A3
4039.16362	4033.82301	3973.93462	4920.36314	5107.74266	4990.66806	SLC35B2
3358.65236	3336.53089	3622.31173	5449.74149	5444.11976	5821.25416	SLC35C1
1421.88937	1524.68197	1373.11718	1729.81517	1584.58635	1565.13889	SLC35D2
7392.8269	7285.47306	7654.6515	8597.45595	8260.2355	8069.86427	SLC35E2B
3707.88835	3783.14689	3821.36604	4564.51545	4542.48087	4768.9741	SLC37A1
815.216571	747.268687	822.439978	1041.18399	958.165079	1009.37155	SLC38A7
8770.81231	8987.8495	8725.01541	10295.4206	10300.7379	10131.5159	SLC39A1
1046.71014	970.973326	1082.28333	1473.91235	1497.48043	1376.13713	SLC39A4
132000.226	136587.548	133509.423	166045.782	167667.769	168726.467	SLC39A6
1630.43314	1608.76953	1739.04337	1135.63739	1090.67727	1149.33502	SLC39A8
2668.16293	2767.74995	2712.85998	3316.85194	3564.85595	3613.5093	SLC41A1
101.777344	90.4337902	120.386142	207.57782	227.031377	210.456012	SLC43A1
1481.75839	1381.0985	1479.20002	1657.32767	1668.91229	1854.2605	SLC46A1
1057.68613	1093.13827	1053.67673	989.564104	840.479426	871.451351	SLC46A3
153.663834	132.47757	119.1942	180.120436	195.524982	194.109914	SLC47A1

964.889136	986.838903	905.875917	1555.18621	1553.07995	1526.3169	SLC4A10__TI
6652.44661	6548.517	6747.58364	8066.9793	8612.3658	8666.49685	SLC7A1
244.46519	238.776937	201.438197	310.817582	325.257198	304.446076	SLC8A1
2842.78092	2989.86803	3076.40229	2524.98099	2606.69088	2754.31752	SLC9A2
167.633273	195.1466	214.549559	117.517602	133.43885	149.158145	SLC9A3
1313.1273	1324.7757	1358.81388	1572.75893	1607.75282	1524.27364	SLC9A6
3716.8687	3804.56542	3720.05097	3263.03547	3241.45207	2995.42246	SLC9A7
2252.0732	2302.88853	2355.27739	1836.34981	1809.76441	1823.61156	SLC9A8
1322.10766	1389.82457	1324.24756	981.876037	962.798372	1001.1985	SLCO3A1
1663.36111	1715.86218	1643.68801	1978.02991	2172.08795	2158.70657	SLFN5
43.9039524	46.010174	41.7179699	65.8977206	79.6926468	89.9035392	SLITRK1
3869.53472	3856.92182	3988.23792	3255.3474	3556.51603	3495.00009	SLK
104.770796	107.885925	75.0923458	38.440337	42.6262994	34.7354583	SLPI
164.639822	156.275936	154.95246	107.632944	108.419066	84.7953835	SLX1A__SUL
358.216339	394.259594	404.068337	431.63007	516.148887	495.491097	SMAD1
58.8712089	76.1547707	56.0212738	128.500555	103.785773	117.48758	SMAD1-AS1
1255.25391	1247.03437	1319.47979	1494.77996	1465.04738	1614.17718	SMAD6
808.231851	786.139352	780.722008	880.832865	971.1383	928.662694	SMAGP
4090.05229	4145.67533	4028.76395	4356.93763	4743.5658	4757.73616	SMARCA2
8030.43202	8217.57572	8001.50662	8671.04174	8774.53107	9033.26242	SMARCA5
346.242534	265.748419	303.945209	353.651101	449.429461	411.717344	SMARCA5-AS
1190.3958	1177.22583	1220.5486	1365.18111	1468.75401	1452.75946	SMARCAL1
3871.53035	4052.06842	4103.85629	4852.26883	4654.60657	4786.34183	SMARCB1
7705.14365	7912.16336	7820.33144	9017.00477	9655.78348	9780.07478	SMARCC1
9190.89331	9263.91054	9350.78496	10035.1246	10312.7845	10589.2066	SMARCC2
25735.6987	25981.4693	26987.9507	28350.2977	27205.7723	28981.6318	SMARCD2
3000.43602	2881.9821	2933.36925	3478.30135	3794.66731	3954.73409	SMCR8
2049.51632	2000.64929	2017.9578	2249.30886	2163.74803	2392.6601	SMG6
7982.5368	8093.03094	8030.11323	8394.27131	8988.58923	8954.59683	SMG7
57.8733918	78.5346073	75.0923458	131.795441	134.365509	159.374456	SMOC2
735.391203	774.240169	786.681718	963.205016	938.705246	930.705957	SMPD2
290.364776	310.965314	324.208223	794.067533	723.720432	803.002066	SMPDL3B
6798.12791	6862.65543	6671.29935	7388.23278	7591.18793	7212.71576	SMS
1969.69096	1954.63911	2026.30139	2494.22873	2525.14491	2676.67355	SMTN
290.364776	308.585477	268.186949	240.52668	191.818347	219.650692	SMTNL2
4497.16167	4547.07443	4466.20666	5070.8296	5212.45509	5306.35207	SMURF1
3257.87283	3314.31908	3286.18408	3577.14793	3578.75584	3566.51426	SMURF2
161.64637	184.040696	181.175184	129.598851	130.658874	122.595735	SNAI1
1217.33686	1237.51502	1289.68124	1381.65554	1332.53519	1468.08393	SNAP47
10360.335	10307.8655	10429.4925	11150.9926	11318.2092	11434.0956	SND1
668.537457	720.297206	709.205488	542.5579	560.628503	651.800659	SNED1
434.050439	383.946969	411.219989	311.915878	317.843928	278.905298	SNHG11__SN
625.631322	669.527359	632.9212	434.924956	435.529581	390.263091	SNHG12__SN

2803.86605	2673.34976	2644.91929	2964.29913	3340.60455	2850.35084	SNHG16__S[illegible]
5059.93052	5430.78708	5071.7132	4098.83822	3908.64633	3679.91532	SNHG1__SN[illegible]
3555.22233	3346.84352	3442.32849	3017.01731	2956.0412	2927.99481	SNHG7__SN[illegible]
1532.64707	1481.84491	1395.76408	1652.93449	1744.8983	1599.87435	SNHG8__SN[illegible]
1373.99415	1413.62293	1483.96779	1562.87427	1554.00661	1598.85271	SNN
10268.5358	9915.19249	10145.8103	9701.24277	9009.90238	9318.29751	SNRNP70
6117.61664	6134.42543	6136.1174	4973.08132	5036.38994	4837.42339	SNRPA1
6284.2521	6625.46505	6503.23553	6928.04703	6942.52686	6981.82712	SNX1
4994.07459	5019.07536	5225.47371	5828.65339	5480.25945	5638.38219	SNX17
1421.88937	1300.97733	1283.72153	1465.12599	1571.61313	1586.59314	SNX18
301.340764	285.58039	311.096861	378.911894	366.956839	381.06841	SNX25
1460.80424	1487.39787	1452.97729	1650.7379	1787.5246	1648.91264	SNX29
2616.27644	2570.22351	2592.47384	2955.51277	3222.9189	3359.12315	SNX33
1887.86995	1808.6758	1877.30864	2075.7782	2147.99483	2202.63671	SNX8
1402.93084	1405.69014	1352.85417	1975.83332	2005.28939	1768.44348	SOAT1
1606.48553	1549.27362	1588.85868	2085.66286	2141.50822	2106.60338	SOCS2
4106.01737	3921.17741	3902.4181	2812.73437	2900.44168	2923.90829	SOD2
5176.67512	4904.04992	5069.32931	6103.22722	6513.48388	6807.1282	SOGA1
1166.44819	1116.14336	1146.6482	855.572073	959.091737	940.922268	SORBS3
183.598347	172.141513	207.397907	107.632944	126.95224	111.357793	SORCS1
597.692443	675.080311	659.143924	478.85677	567.115114	575.178324	SORCS2
1346.05527	1354.9203	1412.45127	1856.11913	1843.12412	1907.38531	SORD
116.744601	111.059041	131.11362	157.056234	168.65188	148.136513	SORD2P
494.917282	502.145519	489.888161	362.437463	409.583138	408.652451	SORL1
681.50908	650.488666	626.96149	831.409575	784.879905	773.374763	SOWAHC
83.8166364	84.8808382	87.0117657	13.1795441	10.1932455	18.3893603	SOX11
4626.87789	4733.49497	4754.65662	5284.99719	4998.39694	5536.21908	SOX12
2224.13432	2148.99244	2324.28689	2562.32304	2547.38472	2510.14768	SOX2
451.013329	385.533527	443.402423	195.496571	173.285174	205.347857	SPAG4
1913.8132	1938.77354	1836.78262	2250.40716	2106.29519	2248.61011	SPAST
133.707491	150.722984	131.11362	237.231794	250.197845	241.104946	SPATA17
2348.86145	2285.4364	2435.1375	2866.55085	2772.56278	2919.82176	SPATA2
211.537225	232.430706	246.731993	170.235778	189.038371	151.201407	SPATA9
3373.61962	3442.03698	3505.50141	3878.08086	3765.01423	3902.63091	SPATS2
6092.67122	5978.1495	6019.30708	5842.93123	6866.54084	7277.07852	SPEN
5406.17305	5373.67101	5815.485	5097.18869	5023.41672	5118.37195	SPG7
1190.3958	1135.97533	1104.93023	1312.46294	1298.24882	1235.15203	SPIN4
5.9869026	3.17311545	2.38388399	21.9659069	34.2863713	24.5191471	SPINK7
6233.36343	6373.99565	6643.88469	5901.14088	5626.67153	6260.55555	SPINT1
1022.76253	1034.43564	951.169713	844.589119	805.266396	863.278302	SPIRE2
287.371325	302.239246	338.511527	136.188623	114.905677	149.158145	SPNS2
278.390971	253.055957	286.066079	224.05225	215.911473	209.434381	SPOCK1
4854.38019	4853.28007	4718.89836	6092.24427	5993.62836	6001.06124	SPOP

3034.3618	3177.87512	3095.47337	3786.92234	3470.33677	3811.70573	SPPL3
3267.851	3149.31708	3135.99939	4265.77911	4295.98966	4173.36315	SPR
3189.02345	3191.36086	3327.90205	4091.15015	3860.46007	4112.06529	SPRYD3
5370.25163	5337.18018	5885.80958	6683.12717	6379.11838	7243.36469	SPTBN2
12274.1482	12355.3183	12336.5997	11518.9216	11065.2313	11178.6878	SQLE
1738.19739	1668.26545	1778.37746	2277.86454	2208.22764	2251.675	SRC
1330.0902	1385.06489	1327.82338	1683.68676	1516.94026	1604.9825	SRD5A1
7634.29864	7660.69396	8323.33096	9478.28881	8496.53347	9749.42584	SREBF1
5628.68626	5864.71062	6093.20749	6979.66691	6924.92034	7114.63917	SREBF2
2262.05137	2136.29997	2118.08093	1443.16008	1601.26621	1468.08393	SRGAP1
3629.06079	3652.25588	3631.84726	3958.25642	3854.90012	3969.03693	SRGAP2
7112.44029	7143.47615	6741.62393	7533.20776	8418.69414	7773.59125	SRP14
5219.58125	5494.24939	5445.98298	5686.97329	5828.68312	5839.64352	SRP68
5083.87813	5120.61505	5145.6136	5655.12272	5788.83679	5576.06269	SRPK2
5684.56402	5833.77275	5841.70772	6552.43002	6727.54204	6636.5158	SRPR
585.718638	576.713732	576.899926	719.38345	667.194252	622.173356	SRRD
8218.02164	8269.93213	7896.61573	6646.88342	6470.85759	6333.09136	SRSF11
11668.4732	12080.0505	12039.8061	11247.6426	10037.5669	10225.506	SRSF2
4943.18592	4894.53057	4878.61859	4232.83025	4290.4297	4367.47307	SRSF4
13357.7775	12945.5177	12675.1112	8474.44687	8882.02348	8472.38694	SRSF5
12716.1811	12966.143	12451.0261	11675.9778	11708.3325	11616.9675	SRSF6
4730.65087	4606.57035	4249.27322	4104.3297	4289.50305	3808.64084	SSBP1
813.220937	836.909199	811.7125	567.818693	676.460839	625.23825	SSBP2
25308.6329	25850.5783	25856.7977	20499.6826	20776.6143	21448.1239	SSFA2
408.107194	387.913363	424.331351	208.676115	232.59133	275.840404	SSPO
7692.17203	8041.46782	7922.83845	9204.81327	8698.54506	8995.46207	SSR2
7086.49705	7245.01584	7101.59042	7509.04526	7581.92135	7999.37172	SSRP1
10057.9964	10265.8217	9910.9977	10976.3637	11160.6772	10670.9371	ST13
3344.68292	3413.47894	3575.82599	2701.80655	2775.34276	2875.89162	ST3GAL1
1469.78459	1556.41313	1623.425	1151.01352	1100.87052	1183.04885	ST3GAL4
1323.10548	1320.01603	1425.56263	1635.36177	1649.45246	1578.42009	ST3GAL5
6719.30035	6662.74916	6425.7593	6531.56241	5828.68312	5868.24919	ST6GALNAC2
597.692443	654.455061	592.395172	532.673242	523.562156	524.096768	ST7L
9851.44823	10105.5794	9841.86506	10251.4887	10653.7949	10898.7609	STAG2
451.013329	387.913363	380.229497	367.92894	278.924264	310.575863	STAG3L2
646.585481	636.209647	604.314592	494.232905	548.581941	524.096768	STAG3L3
10267.538	10184.114	10291.2272	11580.4261	10888.2395	10997.8591	STARD7
15942.1238	16163.8501	15859.9802	10922.5472	10856.7331	11236.9208	STAT1
5004.05276	4898.49697	5020.45969	5253.14663	5791.61677	5756.8914	STAT3
2734.01886	2787.58192	2803.44758	4255.89446	4400.70209	4414.4681	STAT6
5607.7321	5596.58237	5331.55655	8903.88035	9312.91977	9035.30569	STC1
37988.8927	37824.3294	37262.4907	31764.8979	32546.1063	32484.8049	STC2
1795.07296	1840.40696	1756.9225	2046.12423	1987.68288	2142.36047	STEAP3

170.626724	206.252504	196.670429	240.52668	263.171066	235.99679	STEAP3-AS1
16775.3011	17195.1126	16762.2803	18278.9294	18756.4984	19112.6751	STIP1
2365.82435	2313.20116	2447.05692	2550.24179	2604.83756	2829.91822	STK10
1506.70382	1692.85709	1478.00808	1829.76004	1812.54438	1867.5417	STK17A
4775.55264	4946.88698	4927.48821	5817.67043	5565.51205	5539.28397	STK26
1050.70141	1088.3786	1051.29284	1205.92829	1220.40949	1226.97898	STK38L
9890.3631	10700.5386	9908.61382	12340.4465	12547.8852	12026.6416	STMN1
2173.24565	2195.79589	2366.00486	3325.6383	3168.24604	3195.66217	STMN3
5747.4265	5796.48864	5624.77428	4941.23075	4924.26424	4831.2936	STOM
5092.85848	4985.75764	4922.72045	5753.9693	5395.93351	5632.2524	STOML2
321.297106	330.797285	334.935701	451.399386	409.583138	442.366278	STOX1
117.742418	125.33806	104.890896	56.0130625	34.2863713	53.1248186	STRA6
126.722772	97.5732999	128.729736	68.0943113	53.7462036	53.1248186	STX1B
3060.30505	2874.84259	2809.40729	2448.10032	2564.99124	2509.12605	STX4
5489.98969	5476.00398	5361.3551	6083.45791	5950.07541	5860.07614	STX6
4381.41489	4143.29549	4244.50545	3998.89335	3912.35296	3856.6575	STXBP3
1086.62282	1066.96007	1060.82838	1216.91124	1231.52939	1153.42154	STXBP4
1005.79964	1065.37351	1149.03209	1272.9243	1398.32795	1441.52152	SUFU
2124.35261	2315.581	2356.46933	1763.86232	1877.41049	1788.8761	SULF1
102132.567	103036.611	105424.886	87274.9412	86755.6392	90352.0352	SULF2
766.323533	793.278861	808.136674	662.272092	646.807761	672.233282	SULT1A1
12817.9585	12982.8018	13005.2791	15264.1087	15300.0615	14935.2254	SUMO3
1258.24736	1251.00076	1352.85417	1473.91235	1485.43387	1537.55485	SUOX
9138.00901	9043.37902	8932.41332	9306.95474	9943.97433	9914.93009	SUPT6H
3308.76151	3351.60319	3332.66982	3002.73947	3003.30079	3009.7253	SUPT7L
15307.5121	15432.447	16231.8661	18383.2675	17815.9398	18178.9043	SURF4
109.759881	133.270849	129.921678	83.4704461	82.4726228	100.11985	SUSD3
1318.11639	1441.38769	1268.22628	1579.3487	1592.92628	1515.07896	SUV39H2
5457.06172	5262.61197	5534.18669	6020.85507	6705.30223	6725.39771	SVIL
1002.80619	993.185134	1054.86867	876.439684	901.638899	931.727588	SWI5
7235.1718	7300.54536	6978.82039	7804.48671	7770.03306	7724.55295	SYAP1
686.498165	696.49884	661.527808	503.019267	465.182659	505.707408	SYBU
230.49575	188.800369	191.902661	163.646006	139.925461	143.028358	SYCP3
638.602944	639.382762	748.539574	490.938019	515.222228	474.036843	SYNGR1
2335.88983	2344.93231	2373.15652	2587.58383	2468.61873	2779.8583	SYNJ2
430.05917	422.817633	492.272045	267.984064	244.637892	275.840404	SYNPO
927.969903	941.622008	936.866409	1292.69362	1440.95425	1385.33181	SYT10
3051.32469	3073.95559	3258.76942	2528.27588	2402.82597	2461.10939	SYT12
67.8515628	64.2555878	79.8601138	41.7352231	42.6262994	38.8219828	SYT8
2771.93591	2697.94141	2869.00439	2103.23558	1851.46405	2111.71154	SYTL1
31295.5355	31194.1047	30890.3688	20947.7871	22785.6104	23548.5975	SYTL2
2506.51656	2636.06566	2448.24886	3489.28431	3678.83497	3670.72064	SYTL4
1480.76058	1471.53229	1551.90848	1178.4709	1346.43507	1382.26692	SZT2

2231.11904	2234.66655	2165.75861	1552.98962	1773.62472	1714.29703	TACC1
5472.02898	5454.58545	5482.93318	6016.46189	5779.57021	5902.98465	TACC3
12863.8581	12644.0718	12759.7391	10369.0063	10283.1314	10249.0035	TACSTD2
5865.16892	6225.6525	6349.47502	7110.36405	6845.22769	7116.68243	TADA3
4406.36032	4616.08969	4801.14236	3544.19907	3277.59176	3514.41108	TAF1C
3758.77702	3905.31183	3950.09578	4118.60754	4155.13754	4275.52627	TAF4
513.875807	544.982578	517.302826	664.468683	672.754204	604.805627	TAF5
4892.29724	4865.97254	5213.55429	6042.82098	5701.73088	6226.84172	TAF6
10346.3655	10942.4886	10697.6794	11589.2125	11488.7144	11281.8725	TAF7
130.71404	132.47757	121.578084	96.6499902	65.7927665	71.5141789	TAGLN
25386.4627	25566.5844	25600.5302	29001.5868	33052.0619	33667.8538	TANC2
380.168315	445.82272	474.392915	519.493697	506.8823	588.459529	TANGO6
6515.74567	6305.77367	6652.22828	7006.026	6918.43373	7128.942	TAOK2
9425.38033	8780.01044	9042.07199	8456.87414	8143.47651	8156.70292	TAPBP__ZBT
2198.19107	2221.97409	2262.30591	1804.49925	1745.82496	1790.91937	TARBP1
6409.97705	6397.79402	6256.50354	7997.78669	8021.15756	8051.47491	TARS
3624.07171	3639.56342	3711.70738	4253.69787	4007.79881	4193.79578	TARS2
83.8166364	92.8136268	95.3553597	57.1113579	51.8928863	73.5574411	TAT
3911.44303	3948.94217	3832.09352	3170.77866	3107.08657	2921.86502	TATDN1
1667.35238	1613.5292	1816.5196	1298.1851	1177.78319	1244.34671	TAZ
2225.13213	2318.75411	2393.41953	2661.16962	2637.27061	2809.4856	TBC1D1
973.86949	1033.64236	1029.83789	1368.476	1399.25461	1430.28358	TBC1D14
4510.13329	4493.92475	4543.68289	6192.18915	6843.37438	6741.74381	TBC1D16
40.9105011	31.7311545	40.5260279	14.2778395	9.26658683	17.3677292	TBC1D3B__T
106.76643	88.8472325	103.698954	62.6028346	56.5261797	59.2546054	TBC1D3C__T
946.928428	943.208566	872.501541	1000.54706	1045.271	1128.9024	TBC1D4
990.832381	924.963152	938.058351	1073.03455	1107.35713	1077.82084	TBC1D8B
4396.38214	4174.23337	4675.98845	5237.77049	4933.53083	5246.07584	TBCD
4911.25577	4798.54383	4890.53801	5397.02332	5726.75066	5772.21587	TBL1X
3826.62858	4074.28023	4137.23067	4706.19555	4284.86975	4594.27518	TBRG4
5623.69718	5497.42251	5314.86936	5934.08974	6181.74008	6051.12117	TCAF1
1340.06837	1401.72375	1390.99631	1749.58448	1705.97864	1512.01407	TCAIM
2276.02081	2468.68382	2349.31768	2634.81053	2582.59775	2663.39235	TCF12
2207.17143	2243.39262	2187.21356	2246.01398	2743.83636	2658.28419	TCF20
5887.12089	5939.27883	6167.10789	6928.04703	6496.80403	7054.36293	TCF3
393.139938	352.215814	359.966483	499.724381	510.588935	515.923719	TCF7L1
1110.57043	1075.68614	1081.09139	1535.41689	1516.01361	1483.4084	TCF7L2
929.965538	937.655614	910.643685	1187.25727	1023.95785	1215.74104	TCHH
5551.85435	5457.75857	5488.89289	5876.97838	5768.4503	6310.61547	TCOF1
4570.00232	4723.18234	4560.37008	5135.62903	4956.6973	4895.65636	TDG
2981.4775	2972.41589	2940.52091	3225.69342	3339.67789	3389.77208	TDRKH
5442.09447	5343.52641	5678.41167	6045.01757	6524.60379	6576.23956	TEAD1
1893.85686	1953.84584	1963.12847	3008.23095	2988.47425	3051.61218	TEAD2

987.838929	1032.84908	1053.67673	1170.78284	1237.08934	1346.50983	TEF
143.685663	133.270849	119.1942	228.445431	220.544767	244.169839	TESC
412.098463	379.980575	382.613381	451.399386	471.66927	487.318048	TET1
9548.11183	9635.95833	9558.18287	10177.903	11645.3197	11840.7048	TET2
6575.61469	6534.23798	6475.82087	6922.55555	7099.13217	7378.22	TEX2
23.9476104	24.5916447	23.8388399	12.0812488	8.33992815	12.2595735	TFAMP1
4789.52208	4701.76381	4915.56879	5570.55398	5270.83459	5421.79639	TFDP1
61014.5201	55632.6465	52624.2391	43623.1927	50273.0869	42485.552	TFF1
3051.32469	3022.39246	2820.13476	2314.10829	2570.55119	2206.72323	TFF3
2237.10594	2237.83967	2271.84145	2543.65202	2577.0378	2548.96966	TFIP11
985.843295	1066.96007	1029.83789	459.087454	379.003401	401.501033	TFPI
455.004598	429.163864	380.229497	627.126641	546.728623	536.356342	TFPI2
105.768613	100.746415	102.507012	190.005094	176.991809	195.131545	TFR2
18239.0988	18446.9066	17837.412	22344.8188	22985.7686	23019.3926	TFRC
4287.62008	4382.07243	4716.51448	5680.38352	5382.96029	5919.33075	TGFB1
336.264363	306.998919	348.047063	205.381229	202.011593	202.282963	TGFB3
1114.5617	1093.13827	1081.09139	1373.96748	1345.50841	1507.92754	TGFBI
2215.15396	2318.75411	2427.98585	2602.95996	2646.5372	2736.94979	TGFBRAP1
419.083182	436.303374	439.826597	522.788584	565.261797	540.442866	THAP1
537.823417	548.155693	538.757782	717.186859	608.814755	671.21165	THAP10
2370.81343	2430.60643	2417.25837	3088.40651	2801.2892	2946.38417	THAP11
2442.65626	2436.95266	2531.6848	3001.64117	2617.81078	2802.33418	THAP4
598.69026	606.858329	615.04207	487.643133	420.703042	467.907056	THBD
17200.3712	17055.4955	16308.1504	18774.2606	19482.0722	19921.807	THBS1
1383.97232	1397.75735	1381.46077	869.849912	824.726228	893.927236	THBS3
81.8210022	78.5346073	81.0520558	38.440337	35.21303	44.9517696	THEMIS2
1287.18406	1407.2767	1281.33765	1489.28849	1488.21385	1469.10556	THG1L
1367.00943	1403.31031	1275.37794	1641.95154	1648.5258	1564.11726	THNSL1
1031.74288	955.901028	922.563105	1133.44079	1143.49682	1124.81587	THOC3
1919.8001	1911.80206	2165.75861	2359.1384	2075.71545	2249.63174	THOP1
678.515628	644.935714	654.376156	711.695383	805.266396	840.802417	THRA
9061.17709	8975.15704	9051.60752	9460.71609	10171.0057	10110.0616	THRAP3
1787.09043	1753.14628	1666.33491	2340.46738	2623.37073	2574.51044	THRB
1648.39385	1637.32757	1637.7283	2560.12645	2751.24963	2796.20439	TIAM1
2781.91408	2704.28764	2754.57795	3142.22298	3384.15751	3573.66568	TICRR
1133.52023	1163.74009	1164.52733	1388.24531	1296.3955	1274.99565	TIGAR
907.015744	1006.67088	1019.11041	1303.67657	1037.85773	1175.89743	TIGD5
7524.53875	7555.18787	7795.30066	8822.60649	9119.2481	9392.87658	TIMELESS
4129.96498	4180.5796	4296.9509	3666.10986	3789.10736	3558.34122	TIMP1
2211.16269	2065.69816	2217.01211	1137.83398	1213.92288	1179.98395	TIMP3
2290.98806	2352.07182	2368.38875	2017.56855	2006.21605	2002.39701	TINCR
2153.2893	2296.5423	2313.55942	2006.58559	2036.79579	1977.87786	TIPARP
2879.70015	2912.1267	3120.50415	3471.71158	3234.0388	3608.40114	TJP3

5172.68385	5271.33803	5676.02779	6740.23852	5788.83679	6362.71866	TK1
24677.0147	25203.2627	26232.2595	21275.0791	19762.8497	20921.9839	TKT
6357.09275	6007.50082	6268.42296	7681.47763	7523.54185	7615.23842	TLE3
96.7882587	74.568213	92.9714757	61.5045392	42.6262994	57.2113431	TLE6
7394.82253	7225.97715	7301.83667	7704.54183	7728.33342	7914.57634	TLK2
7416.77451	7525.04328	7826.29115	8205.36451	8345.4881	8717.57841	TLN1
54.8799405	52.3564048	48.8696219	23.0642022	13.8998803	14.3028358	TM4SF1
8564.26417	8638.01352	9261.38931	10805.0296	9927.29447	10793.5329	TM7SF2__VI
3868.5369	4067.934	4018.03647	4796.25577	4891.83119	4950.82444	TM7SF3
8562.26854	8759.38519	8504.50614	9611.18255	9190.60082	9243.71844	TM9SF2
47482.1245	48517.7284	48808.8328	56460.0687	53805.5098	55072.0475	TMBIM6
2041.53379	1983.99043	2126.42452	1788.02482	1656.86573	1783.76795	TMC6
5597.75393	5358.59871	5168.2605	5913.22213	6208.61318	6066.44563	TMED8
1187.40235	1293.04454	1311.1362	1568.36575	1515.08695	1518.14386	TMEM101
906.017927	831.356247	970.240785	1245.46692	1040.6377	1177.94069	TMEM102
212.535042	196.733158	246.731993	147.171576	136.218826	132.812047	TMEM105
5418.14686	5558.50498	5465.05405	6559.01979	6557.9635	6568.06652	TMEM106C
506.891087	552.122087	513.727001	441.514728	464.256	394.349615	TMEM107
2032.55343	1845.16663	1955.97682	1816.5805	1660.57236	1688.75625	TMEM120B
4257.68557	4252.76798	4299.33478	4729.25975	4573.98726	4590.18865	TMEM127
1814.03149	1782.4976	1971.47206	1647.44302	1524.35353	1590.67967	TMEM134
55.8777576	35.6975488	41.7179699	17.5727255	25.0197845	14.3028358	TMEM139
573.744833	618.757512	607.890418	484.348246	451.282779	492.426203	TMEM147-A
738.384654	748.061966	771.186472	936.845928	872.91248	904.143547	TMEM150A
1457.81078	1520.71558	1504.2308	1701.25949	1555.85993	1720.42682	TMEM161A
1644.40258	1642.88052	1635.34442	1237.77885	1223.18946	1269.88749	TMEM161B
251.449909	224.497918	246.731993	148.269871	202.938252	151.201407	TMEM161B-
6395.0098	6780.94771	6590.2473	5673.79374	5590.53184	5393.19072	TMEM167A
3378.6087	3338.91073	3209.8998	3024.70538	3065.38692	2867.71857	TMEM167B
80.8231851	87.2606747	69.1326358	46.1284044	42.6262994	37.8003517	TMEM173
839.164181	819.457064	945.210003	801.755601	674.607521	730.466256	TMEM175
412.098463	372.047786	358.774541	246.018157	238.151282	206.369488	TMEM178A
2957.52989	2874.84259	2824.90253	2584.28894	2627.07737	2499.93137	TMEM18
3149.11077	3168.35577	3269.4969	4004.38482	4100.46467	4271.43974	TMEM184B
1802.05768	1795.98334	1821.28737	2142.77422	2230.46745	2213.87465	TMEM185B
2132.33514	2122.81423	2106.16151	2486.54066	2532.55818	2356.90301	TMEM192
1101.59008	1067.75335	1096.58664	661.173797	633.834539	595.610947	TMEM198B
4009.22911	4215.48387	4380.38684	4564.51545	4430.35517	4624.92411	TMEM214
548.799405	557.675039	623.385664	934.649337	1118.47703	1213.69778	TMEM238
3830.61985	3903.72528	4140.8065	3516.74169	3113.57318	3519.51923	TMEM259
988.836747	1024.12301	976.200495	1414.6044	1447.44086	1533.46832	TMEM26
36.9192327	47.5967317	40.5260279	77.9789694	105.63909	86.8386458	TMEM26-AS
1344.05963	1291.45799	1250.34715	1216.91124	1132.37691	1090.08041	TMEM41B

253.445544	260.988745	246.731993	144.974985	159.385294	154.2663	TMEM45B
180.604895	130.097733	154.95246	120.812488	108.419066	110.336162	TMEM51-AS
152.666016	153.10282	146.608866	224.05225	193.671665	199.21807	TMEM52
2282.00771	2363.17773	2489.96683	3154.30423	2886.5418	3256.96003	TMEM54
239.476104	199.906273	215.741501	127.40226	105.63909	109.314531	TMEM59L
2344.87019	2457.57791	2543.60422	2091.15433	2121.12173	2399.81152	TMEM63A
2980.47968	3027.94541	3079.97812	3506.85703	3480.53001	3481.71888	TMEM63B
46660.9211	45807.0946	46290.2594	41968.0617	36249.0344	35568.0877	TMEM64
35.9214156	33.3177122	39.3340859	7.6880674	7.41326947	11.2379424	TMEM74B
4698.72073	4685.10495	5194.48322	6237.21926	5541.41893	6175.76016	TMEM8A
357.218522	368.874671	380.229497	283.360199	274.29097	269.710618	TMEM8B
4415.34067	4375.7262	4424.48869	5252.04833	5035.46329	4957.97586	TMOD4__VP
14007.3565	14091.8057	13737.1315	15936.2654	15333.4212	15294.8396	TMPO
749.360642	718.710648	733.044328	1021.41467	1018.39789	1117.66445	TMPRSS2
68.8493799	72.9816552	70.3245778	52.7181765	41.6996408	35.7570894	TMPRSS6
545.805954	541.809462	582.859636	334.98008	328.963833	352.462739	TMTC1
2850.76346	2977.17557	3110.96861	3395.9292	3233.11215	3386.70719	TNFAIP1
894.044122	879.746257	861.774063	731.464699	770.053366	752.942141	TNFAIP3
454.006781	462.481576	449.362133	646.895957	619.934659	610.935414	TNFAIP8L1
722.419581	813.110833	654.376156	532.673242	472.595928	507.75067	TNFRSF11B
5204.614	5284.0305	5412.60861	5539.80171	5757.3304	5789.5836	TNKS2
5640.66007	5609.27483	5676.02779	6203.1721	6627.4629	6717.22466	TNPO2
5005.05058	4950.06009	5082.44067	5361.87787	5576.63196	5574.01943	TNPO3
14663.9201	14046.5888	15395.1228	18415.118	17801.1133	19630.6421	TNRC18
5279.45028	5165.83194	5238.58507	5489.28013	5747.13715	5643.49035	TNRC6A
1339.07055	1269.24618	1324.24756	1516.74587	1593.85294	1557.98747	TNRC6B
646.585481	590.199473	635.305084	453.595977	457.76939	479.144999	TNS2
3729.84032	3732.37704	3667.60552	5026.89779	5220.79502	5173.54003	TOB2
3652.01059	3855.33527	3592.51318	4741.341	4680.55301	4572.82092	TOM1L1
2371.81125	2358.41805	2339.78214	2803.94801	2688.23684	2762.49057	TOP1MT
15854.3159	16045.6515	15827.7978	19749.5469	20228.9591	20413.2116	TOP2A
1034.73633	1104.24418	1065.59615	1233.38567	1233.38271	1274.99565	TOP3B
6057.74762	6113.80018	5954.94221	6305.31357	6544.99028	6521.07148	TOPBP1
3736.82504	3722.06442	3735.54622	4958.80348	4903.87775	4639.22695	TP53INP1
316.308021	341.903189	336.127643	390.993142	407.729821	430.106704	TP63
13399.6858	13678.5074	14163.8467	13140.0055	11822.3115	12679.4639	TPBG
2350.85709	2321.13395	2476.85547	1960.45719	1925.59674	2086.17076	TPCN1
14516.2432	14881.9114	14900.4669	16625.9949	16636.3033	16600.4842	TPD52L2
3096.22646	3392.85369	3218.24339	3683.68258	3598.21567	3730.99688	TPGS2
10441.1581	10313.4185	10260.2367	6396.47208	6572.79004	6242.16619	TPM1
532.834332	479.140432	482.736509	254.80452	281.70424	248.256364	TPM2
35697.9046	36041.8318	35041.9028	38148.1905	38370.1561	38001.613	TPM4
320.299289	322.864497	315.864629	265.787473	234.444647	194.109914	TPT1-AS1

864.109609	832.942804	885.612903	735.85788	763.566755	710.033633	TPTE
8753.84942	9029.1	8884.73564	11280.5915	11392.3419	11706.8711	TPX2
1553.60123	1573.86526	1507.80663	1058.75671	1072.1441	1025.71765	TRAF5
7212.222	7413.98424	7748.81492	8392.07472	7981.31124	8602.13409	TRAF7
3607.10882	3655.42899	3640.19086	4541.45125	4413.67531	4528.89079	TRAFD1
8832.67697	9141.7456	9282.84427	10115.3001	9506.59143	9631.93826	TRAM1
2321.92039	2341.7592	2377.92428	2457.98498	2744.76302	2781.90156	TRAM2
1384.97014	1243.06798	1377.88495	1701.25949	1854.24403	2071.86793	TRANK1
853.133621	882.919373	883.229019	693.024362	770.053366	665.081864	TRAPPC2
2817.83549	2910.54014	2823.71059	3222.39854	3338.75124	3302.93343	TRAPPC2L
1794.07515	1659.53938	1826.05514	2216.36	2153.55478	2184.24735	TRAPPC6A
2591.33101	2701.9078	2588.89802	2264.685	2132.24163	2099.45197	TRAPPC6B
37.9170498	28.558039	38.1421439	8.78636275	10.1932455	10.2163113	TRAV30
1960.7106	1889.59025	1866.58117	2164.74012	2244.36733	2070.84629	TRIAP1
1356.03344	1263.69323	1371.92524	430.531775	387.34333	430.106704	TRIB2
1337.07491	1331.12193	1447.01758	974.18797	889.592336	844.888942	TRIM16L
4047.14616	4114.73745	4175.37281	4509.60068	4609.20029	4721.97907	TRIM24
28540.5625	29072.877	31468.4607	37327.7638	32526.6464	35834.7334	TRIM28
222.513213	230.844149	247.923935	177.923846	150.118707	197.174808	TRIM29
1503.71037	1447.73392	1463.70477	1788.02482	1822.73763	1919.64489	TRIM32
23861.7981	23865.7945	23536.0867	21442.02	22455.7199	22243.9745	TRIM33
65350.0354	66227.679	66655.7803	69390.2998	70146.209	71445.7296	TRIM37
6039.78691	6021.77984	5985.93271	6186.69767	6477.3442	6620.1697	TRIM44
1323.10548	1301.77061	1312.32814	1148.81693	1133.30357	1143.20523	TRIM45
1941.75208	1970.50469	1934.52186	1526.63053	1441.88091	1450.7162	TRIM52
1686.3109	1668.26545	1728.3159	1941.78617	1794.93787	1849.15234	TRIM65
1460.80424	1497.71049	1517.34216	1249.8601	1229.67607	1208.58962	TRIM66
2152.29149	1903.86927	2013.19003	1261.94135	1330.68187	1338.33678	TRIM73
4968.13134	4731.11513	5187.33157	6358.03174	5566.43871	5965.30415	TRIM8
12111.504	12362.4578	12451.0261	13112.5481	14336.3365	14281.3815	TRIP12
4402.36905	4415.39014	4710.55477	6629.31069	6021.42812	6288.13959	TRIP6
5306.39134	5460.93168	5217.13012	4985.16256	4869.59138	5015.1872	TRIQK
511.880173	441.856326	454.129901	386.599961	410.509797	360.635788	TRMT10B
1822.01403	1829.30105	1727.12395	2071.38502	2109.07516	2097.4087	TRMT1L
158.652919	147.549868	164.487996	233.936908	243.711234	194.109914	TRPC6
4210.78816	4133.77615	4150.34203	4763.3069	4743.5658	4588.14539	TRPS1
309.323301	306.20564	330.167933	509.609039	486.495809	571.0918	TRPV4
10880.1977	10539.503	11087.4445	11907.7181	13608.9094	14367.1985	TRRAP
3731.83596	3773.62754	3934.60053	4519.48534	4396.06879	4331.71598	TRUB2
3203.99071	3149.31708	3295.71962	2778.68722	3035.73385	2973.96821	TSC1
3252.88375	3610.2121	3362.46837	4047.21834	3880.84657	3974.14508	TSG101
3249.8903	3177.08184	3515.03695	2839.09346	2675.26362	2945.36254	TSKU
792.266778	770.273774	818.864152	495.3312	474.449246	466.885425	TSPAN1

3771.74864	3720.47786	3753.42535	4288.84332	4241.31679	4548.30178	TSPAN17
463.984952	483.106827	522.070594	593.079485	572.675066	612.978676	TSPAN33
762.332265	831.356247	799.79308	986.269218	939.631905	965.441415	TSPAN9
472.965306	448.202557	506.575349	619.438574	551.361917	623.194987	TSPO
173.620176	205.459225	157.336344	326.193717	295.60412	307.510969	TSPY26P
4599.93683	4682.72512	4754.65662	5254.24492	5344.96729	5359.47689	TSPYL5
218.521945	225.291197	245.540051	133.992032	144.558755	109.314531	TSSK4
4150.91914	4060.79449	4335.09304	5256.44151	4713.91272	4901.78615	TST
3644.02805	3736.34344	3764.15283	3562.87009	3274.81179	3422.46428	TSTA3
3120.17407	3246.89038	3017.99714	2651.28496	2761.44288	2735.92816	TTC14
365.201059	358.562045	337.319585	271.27895	260.39109	247.234733	TTC32
375.17923	397.43271	364.734251	451.399386	479.082539	459.734007	TTC33
1101.59008	1173.25944	1169.2951	710.597087	676.460839	731.487887	TTC9
2182.226	2377.45675	2424.41002	2537.06224	2568.69787	2796.20439	TTI1
2082.44429	2122.02095	2028.68528	2507.40827	2312.94007	2382.44379	TTK
2496.53839	2528.97301	2593.66578	2895.10653	2796.65591	2789.05298	TTLL12
3477.3926	3407.92599	3649.72639	4959.90177	4400.70209	4967.17054	TTYH3
4638.8517	4787.43793	4780.87935	6540.34877	6279.9659	6335.13462	TUBA1A
12031.6786	11490.6443	11656.0008	14698.4866	16513.0577	15582.9396	TUBA1B
53044.9549	55522.3808	55488.4758	62849.951	60558.9983	62996.8402	TUBB
174.617993	218.944966	214.549559	125.205669	100.079138	99.0982193	TUBB8
6884.93799	7194.24599	6999.0834	9126.8343	8955.22952	8899.42875	TUBD1
511.880173	492.626173	468.433205	412.959049	373.443449	372.895361	TUBG2
1383.97232	1388.23801	1529.26158	1216.91124	1186.12312	1327.09883	TUBGCP6
30.9323301	29.3513179	32.1824339	0	0.92665868	4.08652451	TUFMP1
104.770796	105.506089	113.23449	220.757364	211.27818	229.867004	TXLNB
2106.3919	2094.25619	2064.44354	2367.92476	2306.45346	2187.31224	TXN2
2282.00771	2417.91397	2538.83645	2211.96682	2031.23583	2298.67004	TXNDC11
934.954623	908.304296	961.897191	1086.2141	1164.80997	1132.98892	TXNDC16
1033.73852	1084.4122	1100.16246	1297.0868	1258.40249	1196.33005	TXNL4B
590.707723	640.96932	649.608388	765.511854	759.86012	794.829017	TYRO3
168.63109	188.00709	189.518777	274.573836	281.70424	284.013453	TYRP1
1153.47657	1176.43255	1183.5984	1033.49592	996.158085	1007.32829	TYW5
2096.41373	2056.97209	2077.5549	1540.90837	1480.80058	1428.24032	U2AF1
13429.6204	12798.7612	13348.5584	14762.1877	14808.9324	14724.7694	U2AF2
106.76643	90.4337902	107.27478	65.8977206	51.8928863	64.362761	U73166.2
22809.1011	22816.2866	23888.9015	25267.3827	24005.0932	25615.3572	UBA1
1394.94831	1368.40604	1308.75231	1127.94932	1160.17667	1121.75098	UBA3
2828.81148	2720.94649	2781.99262	2985.16674	2959.74783	3057.74196	UBAC1
2013.59491	2117.26128	2097.81791	2427.23271	2325.9133	2290.49699	UBAC2
2114.37444	2263.22459	1995.3109	2373.41624	2407.45926	2325.23245	UBAP1
198.565603	177.694465	162.104112	115.321011	105.63909	98.0765882	UBAP1L
10086.9331	10214.2586	10332.9452	12405.2459	12489.5057	12840.8816	UBAP2L

3425.50611	3378.57467	3305.25516	3908.83313	4081.00484	3829.07346	UBE2C
1089.61627	1204.99059	1110.88994	1323.44589	1263.96244	1281.12543	UBE2D1
9177.92169	8956.11834	8976.51518	7462.91686	7398.44293	7459.95049	UBE2G2
7561.45799	7329.89668	7200.5216	6586.47717	6936.9669	6672.27289	UBE2I
5078.88904	5233.26065	5199.25099	5545.29319	5627.59818	5448.3588	UBE2Q1
210.539408	213.392014	243.156167	121.910783	101.005797	113.401055	UBE2QL1
3346.67856	3328.5981	3559.1388	4680.93475	4374.75564	4595.29681	UBE2S
3145.1195	3174.702	2928.60149	632.618118	713.527186	644.649241	UBE2T
13103.3342	13636.4636	13737.1315	15635.3325	15780.0707	15794.4172	UBE2Z
7206.2351	7314.82438	7306.60444	7865.99125	7922.00508	8253.75787	UBE3C
5189.64674	5049.21995	5394.72948	5331.1256	5981.5818	6124.67861	UBE4A
4900.27978	5118.23521	5255.27226	5805.58919	6034.40135	6181.88995	UBE4B
3855.56528	3995.74562	3895.26644	4470.06205	4082.85816	4353.17023	UBL4A
2009.60364	2158.51178	2079.93878	2716.08438	2570.55119	2714.4739	UBL7
4053.13306	4313.85045	4176.56476	4876.43133	4914.071	5032.55493	UBQLN4
2314.93567	2351.27855	2388.65176	2906.08948	2721.59655	2882.02141	UBTD2
12506.6395	12921.7194	12983.8242	11305.8523	11629.5665	11521.9559	UBXN4
2915.62157	3006.52688	2892.84323	2564.51963	2510.31837	2470.30407	UCHL5
2263.04918	2329.86002	2293.2964	2482.14748	2480.6653	2501.97463	UCK2
99.78171	77.7413284	113.23449	62.6028346	59.3061557	74.5790723	UCN
3527.28345	3522.95142	3389.88304	4077.97061	4359.92911	4020.11848	UFC1
3667.97566	3642.73653	3649.72639	3495.87408	3395.27742	3177.27281	UFM1
120.735869	99.9531365	145.416924	224.05225	173.285174	178.785447	UFSP1
22850.0116	22906.7204	22553.9265	26405.2167	25076.3106	24717.3435	UGDH
512.87799	489.453057	488.696219	1224.59931	1188.90309	1166.70275	UGT2B15__[illegible]
1918.80228	1886.41713	1818.90349	2144.97081	2047.91569	2081.06261	UIMC1
484.939111	505.318635	500.615639	633.716413	594.914875	693.687535	UNC5B
2093.42028	1969.71141	2203.90075	1745.1913	1604.97284	1731.66476	UNKL
8561.27072	8392.89035	8918.11002	9347.59167	9163.72772	9659.52231	UPF1
161.64637	159.449051	140.649156	66.996016	72.2793773	80.708859	UPP1
1215.34123	1341.43455	1354.04611	1110.37659	1185.19646	1170.78927	UQCC1
73.8384654	57.9093569	78.6681718	40.6369277	34.2863713	34.7354583	UQCRHL
1020.76689	1098.69122	1151.41597	876.439684	813.606324	881.667663	URAHP
2619.26989	2776.47601	2771.26514	3107.07753	3231.25883	3428.59406	URB1
4364.452	4481.23229	4255.23293	4959.90177	4843.64494	4906.8943	URI1
1583.53574	1674.61168	1661.56714	2094.44922	1929.30338	2018.74311	USB1
5358.27783	5518.84104	5546.10611	6437.10901	6328.15215	6365.78355	USF2
4010.22693	4109.1845	4159.87757	4454.68591	4467.42151	4440.00888	USO1
9646.89573	9847.76378	10272.1561	10629.3023	10601.902	10881.3931	USP10
4761.5832	4729.52857	5093.16815	5581.53694	5736.01725	5918.30912	USP11
3053.32033	3048.57066	3307.63904	3777.03769	3525.93629	3813.749	USP19
992.828015	1026.50285	1054.86867	1249.8601	1112.91708	1178.96232	USP21
12684.251	12356.1115	12639.3529	15043.3513	15498.3665	15323.4453	USP22

27223.444	27721.1298	27214.4197	30702.8463	33611.7638	33520.7389	USP32
1635.42223	1707.13611	1547.14071	1741.89642	1857.95066	1846.08745	USP54
9223.82128	9433.67222	9139.81123	9658.40925	9940.2677	9973.16306	USP8
224.508848	224.497918	253.883645	151.564757	160.311952	160.396087	UTS2
12452.7574	12636.9323	12598.8269	11320.1301	11340.449	11337.0406	VAPB
914.998281	911.477412	977.392437	806.148782	731.133701	896.992129	VASN
6462.86136	6489.02109	6789.30161	7941.77363	7154.73169	7599.91395	VAT1
68.8493799	67.4287032	54.8293318	539.263014	589.354923	579.264849	VCAN
6.9847197	3.17311545	1.191942	28.5556789	16.6798563	27.5840404	VCAN-AS1
1112.56607	1105.83073	1067.98003	763.315264	693.140695	707.990371	VCPKMT
4172.87111	4311.47061	4255.23293	3191.64627	3182.14592	3263.08982	VEGFA
7249.14123	7390.18587	7157.61169	7861.59807	7947.02487	7917.64123	VEZF1
4377.42362	4440.77507	4689.09981	5753.9693	5572.92532	5653.70666	VGLL4
6297.22372	6225.6525	5958.51804	6995.04304	7203.8446	6840.84203	VHL
30.9323301	35.6975488	36.9502019	12.0812488	2.77997605	13.2812047	VIL1
260.430263	245.123168	246.731993	99.9448763	149.192048	116.465949	VIM
428.063536	487.073221	460.089611	132.893737	123.245605	118.509211	VIM-AS1
3622.07607	3609.41882	3529.34025	4354.74104	4215.37035	4215.25003	VKORC1L1
81601.4825	83098.3406	79976.9241	65644.0144	62745.9128	59875.7571	VMP1
3317.74186	3531.67749	3560.33074	3763.85814	3933.66611	3793.31637	VOPP1
1680.324	1564.34591	1581.70703	2050.51741	2077.56877	2063.69488	VPS18
2727.03414	2751.88437	2810.59923	3087.30821	3086.70007	2956.60048	VPS25
3110.1959	3207.22644	3143.15105	3394.83091	3449.95028	3347.8852	VPS41
4556.03288	4830.27499	4696.25147	4330.57854	4334.90932	4374.62449	VPS45
2000.62329	2018.8947	2053.71606	2269.07818	2212.86094	2264.95621	VPS4B
1070.65775	1043.95498	1207.43724	937.944223	837.69945	942.96553	VPS9D1
2620.26771	2479.78972	2408.91478	2828.11051	2784.60934	2814.59376	VSIG10
151.668199	118.19855	148.99275	46.1284044	35.21303	54.1464497	VTCN1
330.27746	324.451054	380.229497	232.838613	215.911473	196.153176	VWA7
3327.72003	3526.12454	3413.72188	4227.33878	4049.49845	4103.89224	VWA9
140.692211	133.270849	121.578084	246.018157	229.811353	240.083315	VWDE
808.231851	803.591486	855.814353	1178.4709	1139.79018	1222.89246	WASF1
7122.41846	7568.67362	7421.03087	7704.54183	7967.41136	8094.38342	WBP11
3562.20705	3532.47077	3457.82373	4031.84221	3983.70568	4050.76742	WBP1L
5095.85193	5109.50915	5404.26501	6242.71073	6202.12657	6258.51228	WBP2
3312.75277	3284.17449	3640.19086	4168.03083	3866.02003	4132.49791	WBSCR16
12520.609	12625.0331	12931.3787	14545.8235	13750.6882	14435.6478	WDR1
2129.34169	2138.67981	2200.32493	1982.4231	1904.28359	1980.94276	WDR13
1171.43728	1178.01911	1207.43724	829.212984	926.658683	903.121916	WDR27
375.17923	348.24942	380.229497	286.655085	314.137294	299.33792	WDR31
1958.71497	2010.96191	2152.64725	1671.60551	1655.93907	1629.50165	WDR54
1476.76931	1473.11885	1460.12895	1192.74874	1233.38271	1162.61622	WDR60
37.9170498	46.010174	27.4146659	72.4874927	90.812551	62.3194987	WDR72

1116.55734	1005.8776	1091.81887	958.811835	950.751809	925.597801	WDR76
4135.95188	4171.06025	4271.92012	4516.19045	4754.6857	4885.44005	WDR82
453.008964	434.716816	395.724743	287.75338	292.824144	307.510969	WDR97
2770.93809	2605.12778	2832.05418	3149.91105	3253.49864	3489.89193	WDTC1
1433.86317	1416.00277	1417.21903	863.26014	932.218635	923.554539	WIPF1
1706.26724	1811.84892	1808.17601	846.78571	748.740216	837.737524	WISP2
4038.16581	3788.69984	4306.48643	5387.13866	4695.37955	5204.18896	WIZ
300.342947	279.234159	349.239005	182.317027	177.918467	195.131545	WNT2B
11057.8091	11234.4152	11198.2951	6398.66867	6182.66674	6344.3293	WSB1
4899.28196	4830.27499	5115.81505	5505.75456	5486.74606	5784.47544	WWC1
3992.26622	4071.9004	4258.80875	4634.80635	4397.92211	4822.09892	WWP2
19673.9598	20139.7637	19889.9361	7559.56685	8756.92456	8924.96953	XIST__XIST_i
105.768613	88.0539536	100.123128	49.4232905	75.986012	61.2978676	XKRX
629.62259	605.271771	582.859636	519.493697	528.195449	453.60422	XPA
4124.97589	4201.20485	4187.29223	5048.86369	4855.6915	5295.11413	XPC
4107.01519	4306.71094	4232.58603	4549.13931	4482.24805	4561.58298	XPO7
15101.9618	15491.1496	15116.2084	17348.6732	17053.2998	17113.343	XPOT
23866.7872	25088.2373	24751.8675	25972.4883	25673.0788	26195.6437	XRCC5
13677.079	14316.3036	13919.4986	14931.3252	14849.7054	14879.0357	XRCC6
2029.55998	2086.32341	2095.43403	1673.8021	1970.07636	1888.99595	XRN1
7702.1502	8179.49834	8069.44732	9037.87238	8880.17016	9010.78654	XRN2
1502.71255	1550.86017	1624.61694	1758.37085	1729.1451	1777.63816	XXYLT1
795.260229	806.764602	923.755047	1028.00444	925.732025	1110.51304	XXbac-BPG18
13714.996	13708.652	14691.8771	16975.2528	16547.3441	17690.5646	XXbac-BPG25
644.589847	708.398023	623.385664	942.337405	965.578348	986.895669	XYLT1
3441.47118	3352.39647	3666.41358	4280.05695	3948.49265	4152.93053	XYLT2
2767.94464	2823.27947	2722.39552	3067.53889	3214.57897	3124.14799	YAP1
4470.22061	4587.53165	4585.40086	6024.14996	6178.9601	6334.11299	YARS
968.880405	1048.71466	1014.34264	1172.97943	1117.55037	1223.91409	YARS2
30203.9236	31383.6983	30297.9736	33312.3961	33570.0641	34405.4715	YBX1
8830.68134	9071.14378	9340.05748	9898.93593	9542.73112	10212.2247	YBX3
2153.2893	2135.50669	2275.41727	2876.4355	2277.72704	2731.84163	YDJC
7860.80312	7793.17153	7733.31967	7950.55999	9004.34243	9142.57696	YLPM1
1823.01184	1738.07399	1680.63822	2173.52649	2397.26601	2264.95621	YPEL2
2255.06665	2237.83967	2230.12348	1863.8072	1810.69107	1874.69312	YPEL3
3489.3664	3388.8873	3357.7006	3087.30821	2920.82817	2819.70191	YPEL5
325.288375	330.004006	345.663179	420.647117	436.45624	446.452803	ZBED3
1356.03344	1179.60567	1337.35892	1525.53223	1603.11952	1658.10732	ZBED4
299.34513	290.340063	324.208223	344.864738	406.803162	378.003517	ZBTB11-AS1
692.485068	633.82981	629.345374	943.4357	1008.20465	1042.06375	ZBTB12
1589.52264	1584.17789	1623.425	1686.98165	1795.86453	1857.32539	ZBTB21
2352.85272	2343.34576	2134.76812	1893.46117	1808.83775	1787.85447	ZBTB25
3617.08699	3672.88113	3745.08175	4188.89844	4161.62415	4194.81741	ZBTB33

1378.98323	1416.79605	1314.71202	970.893084	1088.82395	1118.68608	ZBTB37
1973.68223	2041.89979	1950.01711	2204.27875	2255.48724	2276.19415	ZBTB38
2371.81125	2398.082	2462.55217	2499.7202	2707.69667	2784.96645	ZBTB40
2437.66718	2249.73885	2246.81066	2511.80145	2648.39052	2558.16434	ZBTB41
1137.51149	1122.48959	1200.28559	1058.75671	1011.91128	1000.17687	ZBTB43
1306.14258	1175.63927	1376.69301	1789.12311	1562.34654	1722.47008	ZBTB45
1504.70819	1495.33065	1544.75683	1732.01176	1784.74462	1831.78461	ZBTB5
6021.8262	5959.90408	6152.80459	6365.71981	6245.67953	6921.55089	ZBTB7A
1874.89833	1832.47417	1859.42951	1456.33963	1373.30817	1452.75946	ZC3H12A
5610.72556	5584.68318	5666.49225	6325.08288	6523.67713	6740.72218	ZC3H18
3588.15029	3444.41682	3431.60101	4042.82516	4162.55081	4134.54117	ZC3H4
4106.01737	4097.28532	3971.55073	4344.85638	4334.90932	4529.91242	ZC3H7A
4411.3494	4403.49096	4678.37234	4813.82849	5036.38994	5153.1074	ZC3H7B
3481.38386	3379.36795	3559.1388	3782.52916	3902.15972	4125.34649	ZCCHC14
2107.38972	2139.47309	2035.83693	1729.81517	1814.3977	1699.9942	ZDHHC17
1912.81538	1828.50778	1948.82516	2103.23558	2033.08915	2181.18246	ZDHHC18
660.55492	623.517185	635.305084	1032.39762	1061.02419	1100.29672	ZDHHC22
3691.92327	3641.14997	3526.95637	3956.05983	3872.50664	3919.99863	ZDHHC3
6509.75876	6597.70029	6685.60266	7104.87258	6967.54664	7107.48775	ZDHHC7
79.825368	88.0539536	109.658664	132.893737	151.972024	131.790415	ZDHHC8P1
4566.01105	4837.4145	4735.58555	5693.56306	5635.01145	5835.557	ZDHHC9
5657.62296	6082.86231	5807.14141	5467.31422	5320.87416	5131.65315	ZFAND5
295.353862	287.166948	356.390657	432.728365	492.055761	489.36131	ZFHX2
1396.94394	1380.30522	1424.37069	1603.5112	1579.0264	1739.83781	ZFP64
21.9519762	19.0386927	25.0307819	45.0301091	47.2595928	49.0382941	ZFP92
1296.16441	1301.77061	1405.29961	1576.05382	1547.52	1664.23711	ZFPM1
5731.46143	5955.93769	5840.51578	6494.22037	6974.95991	6901.11826	ZFR
1562.58158	1623.84183	1652.03161	2151.56058	2113.70846	2248.61011	ZFYVE1
1260.243	1274.79913	1326.63144	1166.38966	1132.37691	1151.37828	ZFYVE19
860.118341	899.578229	817.67221	1053.26523	1129.59694	1159.55133	ZFYVE9
1106.57916	1123.28287	1015.53458	844.589119	874.765797	911.294965	ZGRF1
3379.60652	3267.51563	3476.8948	3696.86213	3856.75344	3813.749	ZHX2
314.312387	321.277939	320.632397	224.05225	206.644886	184.915234	ZIC2
134.705309	145.963311	153.760518	91.1585135	83.3992815	72.53581	ZIC5
1202.36961	1178.01911	1187.17423	1531.02371	1489.1405	1521.20875	ZKSCAN5
1161.45911	1222.44273	1160.95151	932.452747	952.605126	971.571202	ZMAT1
46.8974037	36.4908276	44.1018539	23.0642022	11.1199042	15.3244669	ZMIZ1-AS1
3826.62858	3871.20084	3770.11254	4754.52054	4889.05121	5123.4801	ZMYM3
3375.61525	3637.18358	3459.01567	3853.91836	3940.15272	4187.66599	ZMYM4
1559.58813	1587.351	1619.84917	1226.7959	1150.91009	1113.57793	ZMYM5
11854.0672	11566.0058	11858.6309	13328.9123	13734.0083	13943.2216	ZMYND8
4314.56114	4264.66716	4175.37281	4515.09216	5053.99646	5032.55493	ZNF106
46.8974037	54.7362414	35.7582599	17.5727255	15.7531976	20.4326225	ZNF135

7698.15893	7996.25092	7900.19155	8345.94631	8667.03866	8678.75642	ZNF146
2920.61065	2992.24786	2917.87401	2330.58272	2313.86673	2370.18421	ZNF160
3467.41442	3542.78339	3580.59376	2691.92189	2668.77701	2855.459	ZNF185
208.543774	204.665946	189.518777	137.286918	98.2258204	118.509211	ZNF204P
553.788491	581.473405	654.376156	548.049376	505.955641	423.976918	ZNF213-AS1
39653.2516	38823.0675	38154.0633	40117.434	41990.6116	41855.2056	ZNF217
2923.6041	2904.98719	3348.16507	4494.22455	3746.48106	4230.5745	ZNF219
705.45669	774.240169	744.963748	582.096532	645.881102	590.502791	ZNF224
5982.91133	6069.37657	5885.80958	6379.99765	6547.77026	6406.6488	ZNF24
1265.23208	1347.78079	1268.22628	1081.82091	1066.58414	1128.9024	ZNF251
1129.52896	1092.34499	1135.92072	1324.54418	1300.10213	1324.03394	ZNF253
2018.58399	2082.35701	2002.46255	2320.69806	2407.45926	2327.27571	ZNF254
257.436812	258.608909	265.803065	182.317027	196.451641	162.439349	ZNF256
484.939111	557.675039	541.141666	651.289139	579.161677	639.541085	ZNF257
883.068134	943.208566	923.755047	671.058455	727.427066	691.644273	ZNF26
655.565835	604.478492	613.850128	784.182875	711.673869	735.574411	ZNF273
1390.95704	1409.65654	1380.26883	1717.73392	1725.43847	1782.74632	ZNF275
2263.04918	2314.78772	2304.02388	2699.60995	2682.67689	2806.42071	ZNF282
3595.13501	3581.65406	3463.78344	2708.39632	2892.10175	2751.25263	ZNF292
3012.40983	3008.11344	2942.90479	3411.30534	3317.43809	3351.97173	ZNF3
1125.53769	1268.4529	1116.84965	1528.82712	1447.44086	1397.59138	ZNF32
1328.09456	1386.65145	1385.0366	1546.39984	1591.99962	1531.42506	ZNF322
639.600761	736.162783	724.700734	565.622102	592.134899	592.546054	ZNF329
447.022061	445.82272	470.817089	370.125531	354.910276	318.748912	ZNF333
2381.78942	2501.20825	2389.8437	1962.65378	1953.3965	1837.9144	ZNF337
1260.243	1258.93355	1224.12443	1412.40781	1413.15449	1454.80273	ZNF346
967.882587	1043.1617	990.503799	777.593103	753.37351	758.050296	ZNF354B
99.78171	119.785108	126.345852	94.4533995	63.9394491	76.6223345	ZNF365
2486.56021	2393.32232	2362.42904	1856.11913	1807.91109	1915.55836	ZNF367
1423.885	1397.75735	1373.11718	924.764679	1039.71104	970.549571	ZNF37BP
1334.08146	1346.19423	1307.56037	1461.8311	1482.65389	1471.14882	ZNF398
199.56342	188.800369	226.468979	150.466462	155.678659	139.963464	ZNF404
631.618225	592.579309	601.930708	705.105611	683.874108	710.033633	ZNF407
356.220705	387.120084	342.087353	204.282934	233.517988	252.342888	ZNF418
1531.64925	1667.47217	1613.88946	1382.75384	1359.40829	1384.31018	ZNF432__ZN
80.8231851	77.7413284	73.9004038	46.1284044	62.0861318	40.8652451	ZNF436-AS1
676.519994	644.935714	668.67946	517.297107	425.336336	423.976918	ZNF44
1450.82606	1396.96408	1386.22854	1697.9646	1789.37792	1785.81121	ZNF473
640.598578	591.786031	625.769548	886.324342	918.318755	867.364827	ZNF486
242.469555	199.112994	203.822081	168.039188	167.725222	150.179776	ZNF492
2190.20854	2242.59934	2051.33218	2200.98387	2501.97845	2471.3257	ZNF507
2411.72393	2407.60134	2457.7844	2884.12357	2878.20187	2916.75687	ZNF516
836.17073	751.235082	836.743282	729.268108	685.727426	671.21165	ZNF517

273.401886	272.887928	245.540051	208.676115	208.498204	182.871972	ZNF546
576.738284	564.02127	541.141666	423.942003	444.796168	396.392877	ZNF550
824.196925	879.746257	883.229019	643.601071	698.700647	621.151725	ZNF558
1296.16441	1300.18405	1292.06512	1520.04076	1431.68767	1545.7279	ZNF561
4773.55701	4837.4145	4962.05453	5052.15858	5245.81481	5719.09105	ZNF592
5153.72532	5238.02032	5161.10884	5865.99543	6488.4641	6626.29949	ZNF609
243.467373	255.435793	247.923935	315.210764	305.797366	305.467707	ZNF610
1244.27792	1255.76044	1262.26657	1334.42884	1439.10094	1404.7428	ZNF616__ZN
1650.38948	1578.62493	1625.80888	1702.35778	1864.43727	1891.03922	ZNF618
1522.6689	1623.84183	1557.86819	1694.66972	1717.09854	1762.31369	ZNF627
4332.52185	4286.87897	4288.6073	4454.68591	4691.67291	4851.72622	ZNF629
9671.84115	10038.1507	9634.46716	10196.574	11006.8518	10642.3315	ZNF638
1520.67326	1423.93556	1410.06738	1610.10097	1680.95885	1680.5832	ZNF641
1407.91993	1382.68506	1404.10767	1740.79812	1705.05198	1671.38852	ZNF644
1470.78241	1482.63819	1550.71654	1371.77088	1313.07535	1351.61798	ZNF654
355.222888	408.538614	361.158425	432.728365	492.98242	450.539327	ZNF669
488.930379	480.72699	508.959233	332.783489	346.570348	312.619125	ZNF682
6505.76749	6259.76349	6665.33964	7983.50885	7582.84801	8156.70292	ZNF687
1169.44164	1053.47433	1138.30461	1390.44191	1400.18127	1458.88925	ZNF689
1273.21462	1279.5588	1336.16698	1011.53001	1051.75761	1056.36659	ZNF692
1594.51173	1675.40496	1669.91074	1346.51009	1376.08815	1374.09387	ZNF7
945.930611	1016.19022	990.503799	1181.76579	1121.25701	1239.23856	ZNF74
2266.04264	2150.57899	2045.37247	2426.13441	2740.12973	2592.8998	ZNF740
562.768845	553.708645	582.859636	378.911894	384.563354	394.349615	ZNF767P
1685.31308	1620.66871	1566.21178	1389.34361	1402.03459	1440.49989	ZNF783
1368.00725	1334.29505	1355.23805	1052.16694	1082.33734	1067.60453	ZNF789
68.8493799	42.8370585	50.0615639	34.0471557	30.5797366	21.4542537	ZNF827
3304.77024	3331.77122	3226.58698	2365.72817	2401.89931	2330.3406	ZNF83
838.166364	878.159699	833.167456	708.400497	696.84733	677.341437	ZNF839
2854.75472	2941.47802	2816.55894	2557.92986	2648.39052	2607.20264	ZNF84
986.841112	1097.89794	1016.72652	1146.62034	1147.20345	1203.48147	ZNF845
464.982769	458.515182	473.200973	403.074391	376.223425	350.419477	ZNF850
1620.45497	1600.83674	1517.34216	1846.23447	1723.58515	1764.35696	ZNF91
643.59203	674.287032	716.35714	769.905036	804.339737	774.396394	ZNF93
446.024244	439.476489	426.715235	282.261903	315.990611	303.424445	ZNRD1-AS1
6545.68018	6508.85306	6856.05036	6412.94651	5554.39215	5951.00131	ZNRF1
868.100877	798.038534	865.349889	1101.59023	1106.43047	1138.09708	ZNRF3
4188.83619	4347.96144	4268.34429	3671.60133	3574.12254	3579.79547	ZRANB2
814.218754	778.999842	803.368906	658.977206	717.233821	683.471224	ZRSR2
537.823417	541.809462	572.132158	382.20678	346.570348	359.614157	ZSCAN18
462.987135	441.063047	433.866887	548.049376	574.528384	537.377973	ZSCAN2
444.02861	463.274855	489.888161	338.274966	347.497006	383.111673	ZSCAN30
1031.74288	1107.41729	1200.28559	1201.53511	1356.62831	1346.50983	ZSWIM5

2031.55562	1930.04747	1832.01485	2425.03612	2461.20546	2405.9413	ZSWIM6
2720.04942	2745.53814	2746.23436	2889.61505	3100.59995	3162.96997	ZZZ3

baseMean	log2FoldChar	lfcSE	stat	pvalue	padj	Control.0
11907.5332	-0.120297	0.03444659	-3.4922747	0.00047893	0.00217321	12252.9386
887.803304	0.52318797	0.0714118	7.32635196	2.37E-13	4.46E-12	693.527621
400.002422	0.57113846	0.10206222	5.59598301	2.19E-08	2.20E-07	302.781752
18151.9374	0.10403369	0.02882964	3.60856676	0.00030789	0.00146611	17393.7468
480.663559	0.31828853	0.09128809	3.4866382	0.00048913	0.00221694	417.598074
16.6291846	-1.6135403	0.54537483	-2.9585896	0.0030905	0.01135724	21.2965758
5493.9844	0.11117712	0.03350343	3.31838001	0.00090541	0.00383386	5318.58833
226.170688	0.5929909	0.1342981	4.41548253	1.01E-05	6.55E-05	187.039492
15919.8916	0.32194504	0.0281339	11.4433127	2.54E-30	1.61E-28	13898.3306
6097.16908	-0.5209436	0.03494491	-14.90757	2.94E-50	4.02E-48	7346.39272
3398.14702	-0.5851139	0.03965601	-14.754735	2.87E-49	3.68E-47	4139.86915
8222.37675	0.24907511	0.03537295	7.04140078	1.90E-12	3.25E-11	7381.57837
12340.5439	-0.2813958	0.04120072	-6.8298756	8.50E-12	1.34E-10	13233.507
212.202817	-1.1909574	0.14024216	-8.4921495	2.03E-17	5.62E-16	303.70769
3814.44611	-0.7144467	0.03929288	-18.182601	7.09E-74	2.05E-71	4718.58045
614.640726	0.76062007	0.081391	9.34526002	9.17E-21	3.25E-19	463.894978
1638.47818	-0.2079038	0.05281315	-3.9365909	8.26E-05	0.00044491	1708.35576
1613.78902	-0.1876622	0.05165935	-3.632686	0.00028049	0.00134785	1705.57794
6570.13172	0.21034016	0.03236375	6.49925235	8.07E-11	1.14E-09	6107.48757
3802.06658	0.19989907	0.04383274	4.56049625	5.10E-06	3.50E-05	3356.52554
1004.35251	-1.6354502	0.07319946	-22.34238	1.43E-110	7.50E-108	1494.46406
3485.71281	-0.1470385	0.03894007	-3.7760204	0.00015935	0.00080845	3664.86292
5100.84504	0.20186254	0.03974482	5.07896444	3.79E-07	3.15E-06	4657.46854
4533.24184	0.59544498	0.03881112	15.3421248	4.00E-53	6.25E-51	3574.12099
6353.23391	0.1064617	0.03301811	3.22434293	0.00126262	0.00515638	6007.48626
1977.36781	0.19754784	0.06378399	3.09713841	0.00195399	0.00759992	1934.28465
592.622923	-0.511935	0.08256533	-6.2003623	5.63E-10	7.04E-09	667.601355
6526.80777	0.09159467	0.03291969	2.78236706	0.0053964	0.01845284	6274.15642
1439.71573	-0.1942972	0.05659467	-3.4331361	0.00059664	0.00265212	1607.42851
61.5686025	-0.9716903	0.25064842	-3.8767064	0.00010588	0.00055597	73.1491083
201.959893	-0.5734267	0.14708063	-3.8987233	9.67E-05	0.00051301	276.855486
1410.70914	0.16220431	0.05519934	2.9385191	0.00329784	0.01202571	1311.12832
1041.14383	0.27103161	0.06383188	4.24602284	2.18E-05	0.00013231	936.123398
24.5971702	-1.1152326	0.39656014	-2.8122661	0.00491938	0.01704113	31.4818947
182.873705	-0.4915283	0.14542815	-3.37987	0.0007252	0.00314638	203.706377
7157.31175	0.29141337	0.03285869	8.86868488	7.40E-19	2.27E-17	6499.15938
5900.2702	0.22408812	0.03970489	5.64384156	1.66E-08	1.70E-07	5281.5508
10277.8322	0.34448213	0.03134765	10.9890907	4.31E-28	2.46E-26	8808.44895
19.3950441	-2.2011746	0.49844633	-4.4160715	1.01E-05	6.53E-05	26.8522043
1260.3686	0.55415805	0.07961972	6.96006057	3.40E-12	5.60E-11	951.864345
1293.65659	0.32903832	0.06016673	5.46877529	4.53E-08	4.35E-07	1191.68231
26.281194	-1.2771033	0.41185564	-3.1008519	0.00192965	0.00751431	25.0003281

10947.3845	0.12427434	0.03018761	4.11673342	3.84E-05	0.00022151	10271.4311
3530.47239	0.24253272	0.04993587	4.8568841	1.19E-06	9.18E-06	3213.00513
800.571688	-0.3291536	0.07380313	-4.4598865	8.20E-06	5.43E-05	840.751776
3424.94567	0.34496907	0.04352946	7.92495665	2.28E-15	5.21E-14	2901.88994
643.60739	0.24181666	0.07918561	3.05379565	0.00225966	0.00861765	613.896946
754.045071	-0.4501857	0.07413625	-6.0724103	1.26E-09	1.49E-08	910.197132
3750.17698	-0.1318164	0.04052892	-3.2524039	0.00114433	0.00472479	3917.64401
496.925578	-0.2233098	0.08932468	-2.4999789	0.01242007	0.03792142	553.710971
1489.58587	0.29348248	0.05703091	5.14602507	2.66E-07	2.27E-06	1391.68493
15.0407668	-1.9611582	0.56135362	-3.4936235	0.00047651	0.00216531	19.4446997
56.1160433	-1.1167407	0.26731329	-4.1776474	2.95E-05	0.00017423	83.3344271
619.582926	-0.4506397	0.08005036	-5.6294525	1.81E-08	1.84E-07	717.602011
65.585122	-0.7498032	0.24569416	-3.0517748	0.00227493	0.00866905	75.9269225
4493.29643	0.20026535	0.03707172	5.40210535	6.59E-08	6.21E-07	4085.23881
788.134256	-0.3443751	0.07213293	-4.7741726	1.80E-06	1.34E-05	866.678042
16706.1454	0.21597403	0.03040998	7.10207636	1.23E-12	2.14E-11	15771.5033
1392.01502	-0.3776322	0.061626	-6.1278068	8.91E-10	1.09E-08	1575.94661
153.09758	-0.6678031	0.15865352	-4.2091916	2.56E-05	0.00015348	189.817306
440.434428	-0.3485693	0.10620903	-3.2819181	0.00103104	0.00430375	496.30281
800.520924	-0.6390931	0.07963948	-8.0248276	1.02E-15	2.40E-14	1016.68001
607.187649	-0.633433	0.09004362	-7.0347345	2.00E-12	3.39E-11	742.602339
2430.62855	-0.2861443	0.04499951	-6.3588311	2.03E-10	2.70E-09	2595.40444
3721.03136	-0.2302023	0.04084132	-5.6365052	1.74E-08	1.77E-07	4031.5344
8099.17046	-0.5408709	0.03245841	-16.663507	2.41E-62	5.44E-60	9384.38243
111.528037	-0.7068951	0.18599408	-3.8006321	0.00014433	0.00073879	140.742588
2549.39807	-0.2048468	0.05597734	-3.659459	0.00025275	0.00122734	2531.51471
1112.34236	0.61596352	0.06901808	8.92466856	4.47E-19	1.40E-17	928.715893
331.191082	0.59385482	0.1073701	5.53091441	3.19E-08	3.11E-07	248.151405
488.657198	-0.409789	0.0882398	-4.6440383	3.42E-06	2.42E-05	561.118476
4157.06332	-0.3692341	0.03938623	-9.374701	6.94E-21	2.49E-19	4570.43036
11217.0411	0.29876682	0.03143585	9.50401538	2.02E-21	7.63E-20	10013.0944
1706.19827	0.20240913	0.05161927	3.92119293	8.81E-05	0.00047132	1587.05787
2389.43591	0.16024215	0.04564285	3.510783	0.00044679	0.00204348	2181.51011
1053.89676	-0.1908404	0.06703848	-2.8467299	0.00441708	0.01552587	1161.12635
2188.63691	-0.172023	0.04790113	-3.5912107	0.00032915	0.00155896	2257.43704
1853.76776	-0.1326129	0.05337716	-2.4844497	0.01297518	0.03937444	1874.09867
890.126529	-0.2489485	0.06796321	-3.6629889	0.00024929	0.00121297	934.271522
40.2050279	-1.710959	0.32909173	-5.1990337	2.00E-07	1.74E-06	63.8897275
314.287542	0.4300482	0.11392717	3.77476428	0.00016016	0.00081189	261.114538
108.974866	-4.1642977	0.30253148	-13.764841	4.15E-43	4.44E-41	191.669182
103.703328	0.78200906	0.19224201	4.06783643	4.75E-05	0.00026701	87.0381794
27197.6369	0.10153237	0.02565407	3.95774835	7.57E-05	0.0004098	26068.8607
2661.94084	-0.1288008	0.04253088	-3.0284064	0.00245847	0.00928634	2771.33267

5311.09664	0.15275081	0.03495195	4.37030842	1.24E-05	7.92E-05	4913.02745
1592.87381	0.18832224	0.06436539	2.92583059	0.00343538	0.01245695	1612.98413
685.207944	0.33484637	0.08690816	3.852876	0.00011674	0.00060915	637.971337
366.045274	-0.4644882	0.10764394	-4.3150427	1.60E-05	9.98E-05	425.005578
30.4905248	-1.2513109	0.36928385	-3.38848	0.00070281	0.00305951	42.5931516
242.502159	-0.8797725	0.13579729	-6.4785716	9.26E-11	1.29E-09	335.189585
192.279843	0.55446515	0.14606813	3.79593527	0.00014709	0.00075113	155.557597
3198.2178	0.24874509	0.03967623	6.26937307	3.63E-10	4.65E-09	2918.55683
23.2619812	-1.4417785	0.42195957	-3.416864	0.00063347	0.00279531	38.8893993
6524.3616	0.17985485	0.03335598	5.39198248	6.97E-08	6.53E-07	6057.48691
82.2690723	0.61871	0.21673364	2.85470227	0.00430772	0.01519104	68.5194179
883.49406	0.29391228	0.07115488	4.13059911	3.62E-05	0.00020981	823.158952
433.887423	-0.8594901	0.09842882	-8.7320981	2.50E-18	7.39E-17	531.488457
8737.25143	0.30492735	0.03635796	8.38681233	5.00E-17	1.32E-15	7607.50726
92.8487605	0.58912078	0.20112386	2.92914409	0.00339897	0.01234339	66.6675417
4281.97011	0.32717399	0.03691443	8.86303907	7.79E-19	2.38E-17	3853.75429
4782.59866	0.0856742	0.03494697	2.45154899	0.01422428	0.04259813	4625.06071
4760.77777	0.14652514	0.03838508	3.81724206	0.00013495	0.00069557	4432.46558
7880.07039	0.19113023	0.03624323	5.27354304	1.34E-07	1.19E-06	7002.86969
276.564449	-0.7347228	0.1206858	-6.0878974	1.14E-09	1.37E-08	374.078984
108.852291	-0.4597661	0.18780766	-2.448069	0.01436242	0.04292998	124.075703
10869.9254	0.26660247	0.02927317	9.10740045	8.44E-20	2.78E-18	9794.573
18050.3762	0.32286335	0.03041404	10.6156016	2.52E-26	1.26E-24	15666.8723
2170.5821	0.16509771	0.0472664	3.4929192	0.00047777	0.00216899	1982.43343
2954.46052	0.20817451	0.04264903	4.8811081	1.05E-06	8.17E-06	2748.18422
538.471003	0.48089031	0.09124613	5.27025443	1.36E-07	1.21E-06	468.524668
5908.04374	0.2224422	0.03757535	5.91989765	3.22E-09	3.61E-08	5331.55146
8319.82414	-0.1298414	0.03154269	-4.1163698	3.85E-05	0.00022179	8542.70472
2004.57903	0.24088805	0.04844008	4.97290743	6.60E-07	5.26E-06	1835.20927
2458.70929	-0.2071888	0.04476992	-4.6278581	3.69E-06	2.60E-05	2604.66382
1200.94261	0.31481283	0.06873411	4.58015436	4.65E-06	3.21E-05	965.753417
144.180133	-0.7016713	0.16206346	-4.3296086	1.49E-05	9.38E-05	180.557925
407.651268	-0.3850421	0.09986596	-3.8555891	0.00011545	0.00060259	474.080297
413.85422	-0.4529213	0.10172888	-4.4522387	8.50E-06	5.60E-05	446.302154
75.0171486	-0.6506359	0.22437473	-2.8997734	0.00373433	0.01339561	80.5566129
281.926336	-0.5554089	0.12202232	-4.5516991	5.32E-06	3.64E-05	318.522699
163.741753	-0.5757494	0.15582625	-3.6948163	0.00022005	0.00108374	180.557925
480.837971	0.78713287	0.09118993	8.63179608	6.04E-18	1.74E-16	361.115851
2518.35102	0.27565342	0.04699369	5.86575361	4.47E-09	4.93E-08	2273.17798
1116.82497	-0.348226	0.06094568	-5.7137107	1.11E-08	1.16E-07	1249.09047
2558.92172	0.18379113	0.04596849	3.99819762	6.38E-05	0.0003507	2355.58647
6303.87486	0.21078132	0.03372902	6.24925659	4.12E-10	5.26E-09	5931.55933
680.241313	-0.5368184	0.07742137	-6.9337233	4.10E-12	6.69E-11	787.973305

1178.89446	0.19429398	0.06670444	2.91275929	0.00358251	0.01291795	1088.90318
9899.88924	0.44412541	0.03412562	13.0144281	1.01E-38	8.97E-37	8063.99473
1639.26716	0.79608594	0.05762603	13.8146938	2.08E-43	2.26E-41	1193.53418
5588.0173	-0.1756491	0.03524735	-4.9833272	6.25E-07	5.01E-06	6006.56032
12345.8606	0.22267629	0.03182374	6.99717563	2.61E-12	4.37E-11	11364.038
2422.02909	-0.5388154	0.04585415	-11.750636	7.01E-32	4.67E-30	2925.96433
5517.49984	0.41304338	0.03492035	11.8281561	2.79E-32	1.90E-30	4804.69269
4682.41985	0.19384401	0.03538646	5.47791486	4.30E-08	4.15E-07	4386.16868
2604.87165	-0.3954079	0.04721961	-8.3738065	5.58E-17	1.46E-15	2887.07493
4381.56362	0.11616137	0.04057674	2.86275775	0.00419972	0.01484798	4120.42445
3128.90057	-0.3018124	0.04388417	-6.8774779	6.09E-12	9.75E-11	3395.41494
4784.30265	0.1540032	0.04403861	3.49700422	0.00047051	0.00214207	4675.06136
3268.34537	-0.1713134	0.03983179	-4.3009219	1.70E-05	0.00010585	3451.89716
2613.49826	0.18862488	0.04381404	4.305124	1.67E-05	0.00010406	2370.40148
8233.88804	-0.3508007	0.0338059	-10.376909	3.16E-25	1.48E-23	9245.49172
2441.89942	0.16198623	0.04782691	3.3869264	0.0007068	0.00307551	2241.69609
41.2920788	-1.3819687	0.32458564	-4.2576398	2.07E-05	0.00012606	52.7784705
7348.04715	0.15068349	0.03190552	4.72280251	2.33E-06	1.70E-05	7011.20314
649.390753	-0.2751853	0.07983479	-3.4469346	0.00056699	0.00253308	747.23203
2268.55252	-0.1891123	0.04721133	-4.0056546	6.18E-05	0.00034069	2401.88338
89829.363	0.32563053	0.03512734	9.27000334	1.86E-20	6.45E-19	81214.0289
6668.53725	0.19616526	0.03173987	6.18040517	6.39E-10	7.94E-09	6159.3401
607.595159	-0.2329697	0.08505563	-2.7390276	0.00616212	0.02069879	691.675745
1664.18279	0.43238558	0.05155904	8.38622324	5.02E-17	1.33E-15	1442.61153
3404.75902	-0.1404204	0.04291821	-3.2718151	0.00106859	0.00443855	3398.19275
276.131277	-0.7642868	0.12463224	-6.1323361	8.66E-10	1.06E-08	333.337709
2579.53137	0.36916326	0.04574345	8.07029773	7.01E-16	1.68E-14	2212.99201
4094.1706	0.12964258	0.03951586	3.28077333	0.00103523	0.0043194	3792.64237
2711.45751	0.14336544	0.04206781	3.40796071	0.0006545	0.00287895	2573.18192
2708.74816	0.14994695	0.04526543	3.31261511	0.00092428	0.00390523	2578.73755
87155.2728	-0.6169161	0.02745268	-22.471984	7.80E-112	4.45E-109	102568.013
621.638224	-0.978165	0.08081898	-12.10316	1.02E-33	7.32E-32	833.344271
2227.67205	-0.1447462	0.04705097	-3.0763691	0.00209538	0.00805773	2300.03019
3428.80326	0.19276793	0.04070396	4.73585224	2.18E-06	1.61E-05	3135.22634
948.951846	0.28660518	0.06713841	4.26887044	1.96E-05	0.00012064	866.678042
487.98552	-0.7003648	0.09494152	-7.3768021	1.62E-13	3.10E-12	645.378841
395.750272	-1.0434458	0.10373672	-10.058596	8.42E-24	3.59E-22	516.673448
5948.87947	0.21473952	0.03729404	5.75801149	8.51E-09	9.05E-08	5408.40432
2352.44734	0.16124305	0.04680756	3.44480764	0.00057147	0.00255087	2228.73296
640.234825	0.42270659	0.07942248	5.32225404	1.02E-07	9.37E-07	571.303795
178.441862	-0.6442478	0.15190383	-4.2411555	2.22E-05	0.00013479	223.151077
5630.66357	0.19553723	0.03702743	5.28087584	1.29E-07	1.15E-06	5213.03139
576.716852	-0.8085879	0.0900299	-8.9813258	2.68E-19	8.47E-18	691.675745

9003.82052	-0.7252977	0.03556913	-20.391212	2.00E-92	8.08E-90	10769.5858
2124.80765	0.15814155	0.04885445	3.23699387	0.00120796	0.00495898	2050.02691
870.19983	-0.2525917	0.06856739	-3.6838461	0.00022974	0.00112776	950.012469
4021.73027	-0.1842461	0.03781188	-4.8727028	1.10E-06	8.52E-06	4180.61043
134.036654	-1.5829908	0.18915376	-8.3688044	5.82E-17	1.52E-15	210.187944
13.6033452	-1.6068845	0.57472021	-2.7959422	0.00517487	0.01779893	17.5928235
545.234556	-0.4011876	0.09723165	-4.1261004	3.69E-05	0.00021357	564.822228
13729.5659	0.25285281	0.030091	8.40293753	4.35E-17	1.16E-15	12373.3106
137.510488	-0.4760957	0.18649148	-2.5529086	0.01068275	0.03328273	189.817306
134.164106	-0.5325469	0.17319985	-3.0747537	0.00210677	0.00809668	146.298217
4161.25501	0.22049123	0.03831859	5.75415897	8.71E-09	9.24E-08	3841.71709
68.4823051	1.998795	0.25795282	7.74868457	9.28E-15	2.01E-13	21.2965758
891.516778	-0.3005796	0.07016234	-4.2840593	1.84E-05	0.00011355	1030.56908
10762.3013	0.1006031	0.03209654	3.13439128	0.00172211	0.00678519	10705.6961
795.447481	0.41723037	0.07210861	5.7861383	7.20E-09	7.74E-08	679.63855
753.320766	-0.2626395	0.07425029	-3.5372183	0.00040437	0.00187299	799.084562
592.403748	-0.4219772	0.08256468	-5.1108677	3.21E-07	2.70E-06	697.231374
1730.94933	-0.3805702	0.05213264	-7.3000366	2.88E-13	5.38E-12	1937.06246
141.187074	-0.838293	0.17676157	-4.7425071	2.11E-06	1.56E-05	155.557597
3769.11788	0.31335523	0.03927865	7.97774902	1.49E-15	3.46E-14	3273.19111
341.837791	-0.2853935	0.10601788	-2.6919372	0.00710383	0.0233601	380.560551
213.455415	-1.0735607	0.1384429	-7.7545376	8.87E-15	1.92E-13	284.26299
495.646368	-0.4691639	0.090122	-5.2058753	1.93E-07	1.69E-06	589.822556
249.22508	-0.3591621	0.12611809	-2.847824	0.00440193	0.01548944	287.966743
29.2536072	-0.9556438	0.37953148	-2.5179565	0.01180379	0.03627401	51.8525324
354.036139	-0.3124111	0.10790978	-2.8951139	0.00379021	0.01356846	418.524012
2804.10559	0.27393761	0.04335755	6.3181059	2.65E-10	3.46E-09	2549.10753
9787.37509	0.12387141	0.03193291	3.87911437	0.00010484	0.00055135	9500.12469
2659.65134	0.29116151	0.05076677	5.73527701	9.74E-09	1.03E-07	2290.77081
10646.1225	-0.1003532	0.03141373	-3.1945661	0.00140041	0.00564856	10875.1427
2683.15225	-0.6652945	0.04961843	-13.408214	5.41E-41	5.22E-39	3118.55945
6547.91396	0.25343243	0.036533	6.93708143	4.00E-12	6.54E-11	5887.11431
1969.36611	0.17099155	0.05010819	3.41244716	0.00064382	0.00283713	1804.65332
281.254705	-0.7997719	0.12372132	-6.4643019	1.02E-10	1.41E-09	350.930532
1208.41508	-0.761098	0.0602722	-12.62768	1.49E-36	1.18E-34	1497.24187
9376.9363	0.23421121	0.03884278	6.02972372	1.64E-09	1.92E-08	8893.63525
277.854696	-0.6448822	0.12226871	-5.2743034	1.33E-07	1.19E-06	353.708346
5125.62487	0.17919805	0.03534708	5.06967036	3.99E-07	3.30E-06	4762.09954
1528.00753	-0.3528863	0.06208711	-5.6837295	1.32E-08	1.37E-07	1707.42982
1604.61539	-0.4020227	0.05359196	-7.5015499	6.31E-14	1.26E-12	1796.31987
2444.69537	0.30705051	0.04432375	6.92744911	4.28E-12	6.97E-11	2155.58385
1700.48526	0.21978217	0.05350424	4.10775236	4.00E-05	0.00022941	1545.39065
7871.74108	-0.5099229	0.11361774	-4.4880571	7.19E-06	4.82E-05	9852.9071

25.6563135	-1.0183604	0.3946989	-2.5800942	0.00987734	0.03113369	41.6672136
779.93535	-0.437362	0.07444069	-5.8753086	4.22E-09	4.66E-08	868.529918
493.120464	0.30287314	0.09864835	3.07023002	0.00213894	0.00820569	462.043101
8290.32405	0.29653204	0.03863687	7.67484689	1.66E-14	3.50E-13	7182.50168
4848.03937	-0.3906036	0.03713187	-10.519364	7.03E-26	3.43E-24	5398.219
8884.9685	0.21377462	0.03179807	6.72287959	1.78E-11	2.71E-10	8126.95852
9096.06432	0.20285746	0.03489715	5.81301017	6.14E-09	6.66E-08	8686.22512
2470.86702	0.17855731	0.05007627	3.56570672	0.00036288	0.00169707	2159.2876
9989.70625	0.30313682	0.03308559	9.1622017	5.08E-20	1.70E-18	8806.59707
24203.8994	0.19594601	0.02961806	6.61576166	3.70E-11	5.38E-10	21737.3223
4076.17805	0.26970229	0.03684585	7.31974747	2.48E-13	4.67E-12	3656.52947
7151.55862	0.26164518	0.03485166	7.50739634	6.03E-14	1.21E-12	6279.71205
1077.77624	0.17384172	0.06281774	2.76739847	0.00565056	0.01916308	1001.865
2081.09252	0.27487437	0.05383343	5.10601628	3.29E-07	2.76E-06	1866.69117
1991.87272	-0.2405328	0.04749526	-5.0643528	4.10E-07	3.38E-06	2145.39853
5725.46888	0.26530523	0.03738778	7.09604191	1.28E-12	2.23E-11	4977.84311
2179.34494	0.19255416	0.05063219	3.80299903	0.00014296	0.00073234	1949.09966
13535.8074	0.0886575	0.02828268	3.13469276	0.00172034	0.0067796	12945.5403
2818.94067	0.22256337	0.04177429	5.3277597	9.94E-08	9.11E-07	2563.92254
560.726354	0.35210933	0.08810158	3.99662888	6.43E-05	0.00035283	444.450278
74.07776	-1.1987044	0.23020103	-5.2072071	1.92E-07	1.67E-06	104.631003
686.059297	-0.3917086	0.07816181	-5.0115092	5.40E-07	4.37E-06	799.084562
3442.23346	0.17350026	0.04087078	4.24509293	2.19E-05	0.00013278	3157.44885
16689.501	0.16696711	0.02687678	6.21231932	5.22E-10	6.55E-09	15531.6853
7237.34185	0.20476554	0.03629365	5.64191036	1.68E-08	1.72E-07	6709.34732
286.047254	0.32994216	0.11686404	2.82329927	0.00475322	0.01654239	236.11421
1992.08123	-0.7835365	0.04904592	-15.97557	1.89E-57	3.70E-55	2534.29252
12976.8478	0.24525455	0.02979445	8.23155194	1.85E-16	4.63E-15	11665.8939
6099.03891	0.10948606	0.03306965	3.31077167	0.00093039	0.00392762	5757.48298
2776.91724	0.16328617	0.04251666	3.84052241	0.00012277	0.00063737	2560.21879
79.4902204	0.69399288	0.21522546	3.2244925	0.00126196	0.00515556	62.9637894
2633.10755	-0.1422414	0.04304342	-3.3046025	0.00095111	0.00400637	2811.14801
4162.63758	0.09385956	0.03780354	2.48282461	0.01303453	0.03952358	3967.64467
1244.42283	0.17786057	0.05975959	2.9762682	0.0029178	0.01080655	1171.31167
4463.18632	0.3916284	0.0378982	10.3336944	4.96E-25	2.28E-23	3902.829
3447.25948	-0.114299	0.04238245	-2.6968481	0.00699992	0.02309282	3653.75166
715.582382	0.21863205	0.07615174	2.87100546	0.00409169	0.01450307	655.56416
1473.94783	0.28142804	0.05792954	4.85810924	1.19E-06	9.13E-06	1303.72082
590.423752	-0.4230361	0.08945047	-4.7292779	2.25E-06	1.66E-05	658.341974
93.0589454	-0.7419194	0.20546149	-3.61099	0.00030503	0.00145497	111.11257
2213.73483	0.23733105	0.0488706	4.85631512	1.20E-06	9.20E-06	2104.65725
815.544164	-0.6112611	0.07391165	-8.2701589	1.34E-16	3.39E-15	949.086531
713.577038	0.27658413	0.08632802	3.20387441	0.00135592	0.00549194	559.2666

3822.29298	0.13720471	0.04367552	3.14145545	0.0016811	0.00664387	3514.86095
6433.47416	-0.0835838	0.0335235	-2.49329	0.01265654	0.03855234	6686.19887
16045.5629	0.11504353	0.03252875	3.53667239	0.0004052	0.00187556	14867.7877
75.0017454	-1.0452136	0.23844459	-4.3834654	1.17E-05	7.50E-05	84.2603652
2052.46825	-0.4169834	0.05156685	-8.086269	6.15E-16	1.48E-14	2309.28957
1221.26147	0.23172509	0.05881446	3.93993399	8.15E-05	0.00043912	1127.79258
1469.12707	0.19211589	0.06071144	3.16441024	0.00155398	0.00620087	1308.35051
1091.50521	0.17464603	0.0626001	2.78986823	0.00527295	0.01808495	1000.93906
747.29593	-0.4723879	0.07753822	-6.0923242	1.11E-09	1.34E-08	887.974618
274.083854	-0.3600985	0.11670524	-3.0855385	0.00203184	0.00785075	300.003938
7118.4575	0.19422902	0.03683606	5.27279523	1.34E-07	1.20E-06	6502.86313
4628.98726	0.33853073	0.03739546	9.0527227	1.39E-19	4.53E-18	4082.46099
1664.45946	-0.2749053	0.05752931	-4.7785264	1.77E-06	1.32E-05	1888.91368
3240.77826	-0.2671198	0.04118662	-6.4855975	8.84E-11	1.24E-09	3504.67563
3294.83665	0.26963211	0.03978524	6.77718916	1.23E-11	1.90E-10	2970.40936
2708.62934	0.16835134	0.04965554	3.39038401	0.00069795	0.00304175	2624.10852
2603.14641	0.13559819	0.0439033	3.08856502	0.00201126	0.00779453	2412.0687
4115.86414	0.2568086	0.0380664	6.74633316	1.52E-11	2.33E-10	3689.86325
2230.96635	0.38858473	0.04640859	8.37312119	5.61E-17	1.47E-15	1908.35838
5477.34078	0.1481726	0.03484432	4.25241738	2.11E-05	0.00012883	5156.54916
4172.06969	0.26474774	0.03951605	6.69975201	2.09E-11	3.15E-10	3754.67891
409.469346	0.23390222	0.09810382	2.38423159	0.01711483	0.04992039	359.263975
19101.3352	0.19204708	0.02795406	6.87009637	6.42E-12	1.03E-10	17672.4542
821.976657	-0.3388743	0.07380653	-4.5913866	4.40E-06	3.06E-05	872.233671
4995.21772	-0.3645721	0.03887177	-9.3788407	6.67E-21	2.41E-19	5538.96159
5162.72229	-0.1297124	0.03623588	-3.5796663	0.00034403	0.0016191	5350.99616
2717.89092	-0.2900746	0.04554635	-6.3687788	1.91E-10	2.55E-09	2876.88961
3788.27585	0.3365653	0.03852205	8.73695301	2.39E-18	7.10E-17	3318.56208
162.737652	-0.4611422	0.15859747	-2.9076264	0.00364183	0.01311235	192.59512
663.554129	-0.9413	0.08064579	-11.672029	1.77E-31	1.16E-29	886.122742
4485.33899	0.21110427	0.03651644	5.78107453	7.42E-09	7.95E-08	4156.53604
97.955757	-0.6627567	0.19621292	-3.3777425	0.00073084	0.00316799	119.446012
43.8454817	-1.0757234	0.31599985	-3.4041895	0.00066361	0.00291305	76.8528606
75.2353772	-1.3793273	0.23926239	-5.764915	8.17E-09	8.70E-08	128.705393
4046.26737	0.09189824	0.03802927	2.41651347	0.01566995	0.04631223	3857.45804
2477.37099	0.50986333	0.04773447	10.6812405	1.25E-26	6.41E-25	1951.87747
5336.08327	0.45353385	0.03713497	12.2131199	2.65E-34	1.95E-32	4510.24438
577.208166	-0.6417309	0.08557732	-7.4988428	6.44E-14	1.29E-12	735.194835
1184.78892	-0.2384251	0.06506859	-3.664212	0.0002481	0.00120749	1242.6089
80.4251333	-0.7855699	0.23149179	-3.3935108	0.00069003	0.00301078	95.3716221
726.17722	-0.3682604	0.07829411	-4.7035516	2.56E-06	1.86E-05	826.862705
1961.50441	0.26497327	0.04952986	5.3497685	8.81E-08	8.14E-07	1797.24581
7137.82218	0.12695466	0.03715725	3.41668655	0.00063388	0.0027965	6745.45891

2926.84914	0.48500561	0.04825474	10.0509419	9.10E-24	3.87E-22	2440.77278
4344.76255	0.32322106	0.04482664	7.21046781	5.58E-13	1.01E-11	3776.90142
543.120953	-0.2442638	0.08725413	-2.7994527	0.00511893	0.01762844	559.2666
3954.68694	0.1951539	0.03966803	4.91967706	8.67E-07	6.80E-06	3561.15785
1189.62831	-0.8858557	0.06292091	-14.078875	5.12E-45	5.74E-43	1503.72344
784.908055	-0.4653059	0.0714711	-6.5104058	7.49E-11	1.06E-09	908.345256
14703.4867	0.31775778	0.02841161	11.1840838	4.88E-29	2.93E-27	12759.4267
8201.81476	0.24305916	0.03867141	6.28524232	3.27E-10	4.21E-09	7202.87232
4742.23305	0.14024142	0.04465291	3.1407006	0.00168544	0.00665831	4301.90832
5883.96028	0.30016618	0.03451508	8.69666687	3.42E-18	1.00E-16	5267.66173
353.031834	0.58341498	0.10769938	5.41706917	6.06E-08	5.74E-07	308.33738
6919.50666	-0.1830117	0.03425509	-5.3426115	9.16E-08	8.44E-07	7482.50562
88.1123999	-0.7371741	0.20896019	-3.5278209	0.000419	0.00193111	99.0753745
5707.63872	-0.4156046	0.03937453	-10.555163	4.81E-26	2.38E-24	6351.93522
7586.17128	0.23390462	0.03318883	7.04769121	1.82E-12	3.11E-11	6922.31308
1553.24663	0.28802784	0.05780241	4.98297252	6.26E-07	5.02E-06	1445.38934
31039.6194	0.27499938	0.02650962	10.3735688	3.27E-25	1.53E-23	27296.6546
1327.58154	-0.2159952	0.05886773	-3.6691622	0.00024335	0.00118674	1437.0559
107.41463	-0.9730904	0.19407492	-5.0139938	5.33E-07	4.32E-06	126.853517
115.588053	-1.0123246	0.18674376	-5.4209288	5.93E-08	5.63E-07	162.965102
4709.40948	0.10886808	0.0364853	2.9838887	0.0028461	0.01056929	4550.05972
180.831889	-1.3711114	0.15110586	-9.0738468	1.15E-19	3.75E-18	245.373591
5985.06455	0.09628752	0.03539001	2.72075406	0.00651332	0.02169801	5694.51919
18235.8584	-0.099442	0.02610572	-3.8092015	0.00013942	0.00071687	18578.0216
593.651076	0.39730072	0.08289941	4.79256422	1.65E-06	1.24E-05	505.562191
3581.2938	0.17231036	0.04481222	3.84516499	0.00012047	0.00062711	3278.74674
2732.40712	0.52041011	0.0422217	12.3256541	6.59E-35	5.01E-33	2227.80702
35.3962368	-1.0328383	0.32849965	-3.1441077	0.00166594	0.00659067	50.0006563
1543.23587	0.54247669	0.05760413	9.41732363	4.63E-21	1.69E-19	1239.83109
10300.2128	0.1596197	0.03269023	4.88279493	1.05E-06	8.11E-06	9492.71719
2991.55809	0.27904434	0.04223351	6.60717929	3.92E-11	5.69E-10	2689.85012
13974.4022	0.19724928	0.02968894	6.64386374	3.06E-11	4.49E-10	12723.3151
6087.34623	-0.1099082	0.03702812	-2.9682372	0.00299513	0.01105917	6100.08007
6810.50808	0.42286899	0.03272211	12.9230351	3.34E-38	2.90E-36	5755.6311
133.113435	-0.6368373	0.1785214	-3.5672885	0.00036069	0.00168809	187.039492
61.726336	-2.073009	0.27611287	-7.507832	6.01E-14	1.21E-12	90.7419318
17637.2183	-0.1107861	0.02625282	-4.2199717	2.44E-05	0.00014687	18239.1283
22.2848166	-1.1892913	0.43060139	-2.761931	0.00574606	0.01945632	24.0743901
11149.0842	-0.1304508	0.02899772	-4.4986562	6.84E-06	4.60E-05	11546.4479
731.162683	0.22663245	0.07453044	3.04080366	0.00235948	0.00894902	645.378841
531.1439	-0.4988514	0.0869854	-5.7348868	9.76E-09	1.03E-07	607.41538
2198.47733	-0.202514	0.05501745	-3.6809049	0.00023241	0.00113999	2280.58549
1867.12605	0.2187835	0.0556365	3.93237338	8.41E-05	0.00045204	1621.31758

1463.52139	0.2889271	0.05707436	5.06229237	4.14E-07	3.42E-06	1254.6461
3473.73916	0.2773247	0.04308623	6.43650474	1.22E-10	1.68E-09	3299.11738
1724.0946	0.23488779	0.05142655	4.56744175	4.94E-06	3.39E-05	1613.91007
59.9215981	0.84795632	0.25495684	3.32588182	0.00088139	0.00374379	49.0747182
3786.02326	-0.8410238	0.03826723	-21.977653	4.71E-107	2.34E-104	4899.13838
7672.31664	-0.5563355	0.03517888	-15.814474	2.47E-56	4.52E-54	8923.26527
12338.8902	0.10053933	0.02862172	3.51269338	0.00044359	0.00203141	11773.3027
1847.57256	0.2405497	0.05508	4.36727827	1.26E-05	8.02E-05	1671.31823
1114.74844	0.19366634	0.06262072	3.09268764	0.00198353	0.00769938	1069.45848
867.443227	-0.2408905	0.08009654	-3.0075021	0.00263404	0.00986875	907.419318
13038.1159	0.80500823	0.0289346	27.8216477	2.37E-170	3.54E-167	9397.34556
1092.06278	0.32800573	0.0612991	5.35090604	8.75E-08	8.09E-07	947.234655
3584.71293	0.14967579	0.04498108	3.32752797	0.0008762	0.00372645	3492.63843
9690.36641	0.22489351	0.03138195	7.16633375	7.70E-13	1.38E-11	9110.30476
8189.71171	0.22159371	0.03101017	7.14584002	8.94E-13	1.58E-11	7610.28507
1464.0641	-0.2385492	0.05758119	-4.1428316	3.43E-05	0.0002	1593.53943
138.020448	0.50135088	0.1643857	3.0498448	0.0022896	0.00871734	110.186631
7272.48309	0.15667688	0.03479596	4.50273143	6.71E-06	4.52E-05	6896.38681
797.564676	0.26789363	0.07990303	3.35273439	0.00080018	0.00343324	752.787658
4160.41609	-0.2453801	0.03874866	-6.3326078	2.41E-10	3.17E-09	4563.94879
561.651338	-0.7082674	0.08882558	-7.9736874	1.54E-15	3.58E-14	684.26824
1003.0717	0.22100252	0.06672759	3.31201116	0.00092628	0.00391111	951.864345
2528.2086	0.27938361	0.04409156	6.33644228	2.35E-10	3.10E-09	2244.4739
2999.6897	-0.1130787	0.04234656	-2.6703165	0.00757798	0.02471384	3048.18816
2869.49069	0.56053734	0.04393365	12.758724	2.79E-37	2.30E-35	2416.69839
3843.55102	-0.5933818	0.038958	-15.23132	2.19E-52	3.24E-50	4526.91127
1572.40044	0.14049997	0.0571074	2.46027632	0.01388301	0.04167913	1480.57499
11810.2837	-0.1737215	0.02896309	-5.9980287	2.00E-09	2.30E-08	12507.5716
630.960643	-0.4820549	0.08249798	-5.8432327	5.12E-09	5.59E-08	693.527621
14.287803	-1.553009	0.54744184	-2.8368476	0.00455614	0.01593949	23.148452
9807.95774	-0.1087212	0.03485163	-3.1195435	0.00181132	0.00710058	9977.90874
2861.73695	0.21122439	0.04495582	4.69848861	2.62E-06	1.90E-05	2603.73788
1172.94044	0.41035064	0.06333723	6.47882194	9.24E-11	1.29E-09	1057.42129
4023.9407	0.25395052	0.04509621	5.63130532	1.79E-08	1.82E-07	3604.67694
490.102928	-0.5641826	0.09535973	-5.9163609	3.29E-09	3.68E-08	568.525981
2860.21267	-0.6007881	0.04327709	-13.882359	8.10E-44	8.93E-42	3379.67399
2484.45776	0.19062681	0.04420992	4.31185571	1.62E-05	0.00010117	2287.99299
75.1968133	-1.252018	0.23422253	-5.3454209	9.02E-08	8.32E-07	107.408817
1582.89692	0.21367988	0.05452833	3.91869487	8.90E-05	0.00047544	1484.27874
150.691587	-1.263385	0.17001263	-7.4311243	1.08E-13	2.09E-12	198.150749
3369.26965	0.24405708	0.04068265	5.99904574	1.98E-09	2.29E-08	3086.15162
2030.31005	0.1362073	0.04934523	2.76029326	0.00577495	0.01954389	1932.43277
20102.2627	0.11166799	0.02780428	4.01621551	5.91E-05	0.00032727	18991.9159

3332.70443	0.26087827	0.04366011	5.97520943	2.30E-09	2.62E-08	3061.15129
227.278744	0.4663426	0.13131343	3.55137016	0.00038323	0.00178451	182.409802
15861.825	0.41496054	0.02826122	14.6830364	8.28E-49	1.03E-46	13408.5093
324.049382	0.73562376	0.1101037	6.68119017	2.37E-11	3.55E-10	249.077343
5196.56861	0.15115384	0.03399875	4.44586473	8.75E-06	5.76E-05	4906.54588
561.146086	-0.2269893	0.08675151	-2.6165463	0.00888243	0.02836671	576.859423
4629.67829	-0.9076751	0.03990849	-22.743911	1.65E-114	9.68E-112	6088.96881
4246.62089	-0.4130275	0.04023066	-10.266487	9.98E-25	4.51E-23	4686.17262
7004.8195	0.23128405	0.034584	6.68760329	2.27E-11	3.41E-10	6319.52739
21103.206	0.32189521	0.03014376	10.6786698	1.28E-26	6.57E-25	19164.1404
4669.17341	0.1424968	0.03675734	3.87668981	0.00010589	0.00055597	4438.02121
4159.42244	0.22403212	0.03793569	5.90557667	3.51E-09	3.90E-08	3769.49392
1696.28665	0.26344172	0.05693172	4.62732771	3.70E-06	2.61E-05	1593.53943
46.2717364	1.33532891	0.30079374	4.43935075	9.02E-06	5.92E-05	23.148452
3534.06495	0.28398816	0.04288083	6.62272984	3.53E-11	5.14E-10	3167.63417
16840.7841	0.16977324	0.02755028	6.16230588	7.17E-10	8.84E-09	16077.0629
2967.21041	0.23152036	0.04290323	5.39633827	6.80E-08	6.38E-07	2656.51635
1987.15815	-0.3371859	0.04849776	-6.9526083	3.59E-12	5.90E-11	2233.36265
3492.49297	-0.2083178	0.04507479	-4.6216046	3.81E-06	2.67E-05	3624.12164
1566.28831	0.21867162	0.05539647	3.94739244	7.90E-05	0.0004265	1438.90778
4548.36549	0.3714143	0.04130573	8.99183425	2.43E-19	7.73E-18	3866.71742
2180.60193	0.83711881	0.0471656	17.7485049	1.77E-70	4.57E-68	1539.83503
340.71601	-0.3663466	0.10845852	-3.3777577	0.0007308	0.00316799	390.745869
112.080102	-0.5045503	0.18446103	-2.7352676	0.00623296	0.02091499	115.74226
3798.89559	0.485397	0.04344018	11.1739173	5.47E-29	3.27E-27	3062.07723
2613.42772	0.32037432	0.04464024	7.17680526	7.14E-13	1.28E-11	2275.02986
10663.9519	-0.2056436	0.03168681	-6.4898794	8.59E-11	1.20E-09	11163.1095
76.3326631	-0.584845	0.2253283	-2.595524	0.00944468	0.02995993	88.8900556
1601.3463	0.31966439	0.05365945	5.95728016	2.56E-09	2.91E-08	1375.94399
1669.03947	0.21539187	0.05154642	4.17860029	2.93E-05	0.00017361	1562.98348
126.210678	0.64151133	0.17486966	3.66851128	0.00024397	0.00118886	102.779127
2933.61541	0.37110713	0.04408113	8.41873007	3.81E-17	1.02E-15	2472.25467
14258.5726	0.23801434	0.02701244	8.81128699	1.24E-18	3.74E-17	13165.9136
118.468364	0.68001384	0.17853115	3.80893671	0.00013957	0.00071741	90.7419318
123.371167	-0.4826257	0.17418833	-2.7707122	0.00559339	0.01898578	131.483207
3442.03329	-0.2736425	0.04069277	-6.7245992	1.76E-11	2.69E-10	3666.71479
1619.90962	0.33365486	0.05291151	6.30590309	2.87E-10	3.72E-09	1487.98249
6026.93846	-0.2514631	0.03357208	-7.490247	6.87E-14	1.37E-12	6463.04779
4682.14091	0.41214574	0.03601902	11.4424472	2.57E-30	1.63E-28	4081.53505
1254.73926	0.16820582	0.06227402	2.70105928	0.0069119	0.02283743	1242.6089
1025.05491	0.23038701	0.06606896	3.48706884	0.00048835	0.00221389	927.789955
3351.3247	0.12015163	0.04018541	2.98993159	0.0027904	0.01038631	3160.22666
412.701982	-0.3346462	0.09579649	-3.4933036	0.00047708	0.00216689	467.59873

2906.91534	-0.4645208	0.05011133	-9.2697766	1.87E-20	6.46E-19	3402.82244
1966.66423	0.23529162	0.05344008	4.40290587	1.07E-05	6.91E-05	1772.24548
1493.123	-0.165813	0.05665009	-2.9269678	0.00342284	0.01241846	1665.7626
332.475339	-0.8453447	0.11628758	-7.2694317	3.61E-13	6.69E-12	410.190569
59.0854658	-1.5545519	0.26517096	-5.8624514	4.56E-09	5.01E-08	93.519746
999.341708	0.24249977	0.06816171	3.55771238	0.0003741	0.0017466	870.381794
198.435017	0.47579323	0.14805122	3.2137069	0.00131033	0.00533183	191.669182
1732.00871	0.3142709	0.05859106	5.36380285	8.15E-08	7.55E-07	1558.35379
297.363765	0.382424	0.12120135	3.15527853	0.00160345	0.00637336	224.077015
816.448822	0.19960121	0.07121758	2.80269581	0.00506774	0.01748322	761.121101
654.781787	0.20519823	0.07954666	2.57959591	0.0098916	0.03116932	611.119132
5648.5469	0.27002702	0.03632557	7.43352403	1.06E-13	2.06E-12	4991.73218
619.186025	-0.374519	0.08144406	-4.5984813	4.26E-06	2.96E-05	678.712612
4844.6097	0.11076488	0.0399093	2.77541546	0.00551312	0.01876741	4443.57684
6091.76949	-0.5131951	0.03357753	-15.283883	9.79E-53	1.49E-50	7191.76106
200.316995	-0.7780651	0.13817001	-5.6312153	1.79E-08	1.82E-07	252.781096
13.5000907	-1.5828945	0.58835497	-2.6903732	0.00713721	0.02345794	16.6668854
3100.25753	-0.5305154	0.04044652	-13.116467	2.65E-39	2.42E-37	3635.2329
55.0058811	-1.2104701	0.27432827	-4.4124875	1.02E-05	6.63E-05	62.0378513
12006.9107	0.2400329	0.02981624	8.05040733	8.25E-16	1.96E-14	10947.3659
1468.63454	-0.499743	0.05475966	-9.1261162	7.10E-20	2.36E-18	1705.57794
3234.28318	0.25698063	0.04073554	6.30851243	2.82E-10	3.66E-09	3005.59501
701.564644	0.62950359	0.0762217	8.25884955	1.47E-16	3.71E-15	556.488786
2243.05469	0.25932566	0.05213684	4.9739427	6.56E-07	5.24E-06	2047.24909
30685.8581	-0.1140293	0.0259763	-4.3897421	1.13E-05	7.30E-05	31558.7476
2803.83232	0.18241185	0.04165272	4.37935004	1.19E-05	7.63E-05	2651.88666
828.157542	-0.2522276	0.0749627	-3.3647082	0.00076625	0.00330376	915.75276
3823.62142	-0.5895729	0.05401037	-10.915921	9.67E-28	5.42E-26	4737.09921
15.8928262	-2.972014	0.61509641	-4.8317856	1.35E-06	1.03E-05	23.148452
1640.83184	-0.2185171	0.05229405	-4.1786216	2.93E-05	0.00017361	1769.46767
877.080135	-0.3177295	0.07518813	-4.2257937	2.38E-05	0.00014339	936.123398
6948.97385	-0.5662815	0.0317047	-17.861121	2.37E-71	6.38E-69	8419.55495
11700.3425	-0.0948673	0.03078939	-3.0811696	0.00206189	0.00794944	12387.1996
6325.75934	0.11117749	0.03418529	3.25220238	0.00114515	0.00472714	6175.08105
3549.41255	0.12398259	0.04414772	2.80835772	0.00497949	0.01721551	3385.22962
780.344177	-0.6760118	0.08001912	-8.4481281	2.96E-17	8.06E-16	987.97593
265.361435	-0.9918997	0.12990062	-7.6358353	2.24E-14	4.67E-13	400.931188
274.944777	-1.1159726	0.12732897	-8.7644831	1.88E-18	5.60E-17	396.301498
2468.03359	0.2092251	0.04712686	4.4396148	9.01E-06	5.91E-05	2262.99267
4973.82138	-0.2281504	0.04374603	-5.2153402	1.83E-07	1.61E-06	5493.59062
33.3657477	-1.2021919	0.34569879	-3.4775706	0.00050598	0.00228368	51.8525324
32.3709207	-1.8620997	0.37077535	-5.0221778	5.11E-07	4.15E-06	53.7044086
773.348444	0.20153357	0.07099805	2.83857882	0.00453149	0.0158762	727.78733

1693.90838	-0.7780163	0.05226745	-14.885291	4.11E-50	5.57E-48	2083.36068
1741.56823	0.23074753	0.05031829	4.58575888	4.52E-06	3.14E-05	1637.05852
9513.70495	-0.1862359	0.03120989	-5.9672101	2.41E-09	2.74E-08	10091.7991
3240.26664	-0.2646649	0.04088458	-6.4734642	9.58E-11	1.33E-09	3560.23191
872.221752	0.33959092	0.07118794	4.77034333	1.84E-06	1.37E-05	810.195819
67.0528941	-4.8230801	0.45016844	-10.713945	8.75E-27	4.54E-25	127.779455
3709.98384	0.15674226	0.03960043	3.95809458	7.56E-05	0.00040932	3413.9337
5023.88437	0.33222361	0.04275722	7.7700007	7.85E-15	1.71E-13	4275.98205
80.8316306	0.57115718	0.21255613	2.68708877	0.00720778	0.0236698	62.0378513
7250.84472	0.33796784	0.03665048	9.22137559	2.93E-20	1.00E-18	6263.04517
503.607522	0.3435991	0.09276371	3.70402514	0.00021221	0.00104805	431.487145
1044.51875	0.28369753	0.06296258	4.50581167	6.61E-06	4.47E-05	921.308389
1877.23163	-0.5258773	0.04925823	-10.675928	1.32E-26	6.75E-25	2197.25106
5554.55872	-0.6080607	0.03979746	-15.278883	1.06E-52	1.60E-50	6588.97537
1701.72164	0.27520586	0.05518867	4.98663737	6.14E-07	4.94E-06	1480.57499
28204.4948	-0.1937491	0.02559347	-7.5702544	3.72E-14	7.63E-13	30421.6956
12258.373	0.27132959	0.02907282	9.33275732	1.03E-20	3.64E-19	11177.9245
151.579131	-0.555344	0.15818418	-3.5107427	0.00044686	0.00204348	166.668854
1658.16817	-0.8270435	0.05336113	-15.498988	3.52E-54	5.89E-52	2141.69478
26899.299	0.12337644	0.03276321	3.76570096	0.00016608	0.00083929	26437.384
14981.3187	0.08986235	0.02735598	3.28492504	0.0010201	0.00426359	14322.4102
721.479867	0.61158606	0.07386497	8.27978453	1.23E-16	3.13E-15	575.933485
620.054336	-0.9446446	0.08429108	-11.206934	3.77E-29	2.28E-27	777.787986
1485.57421	-1.0478971	0.05863234	-17.87234	1.94E-71	5.29E-69	2016.69314
258.353897	-0.3869489	0.13164041	-2.9394385	0.00328808	0.01199912	313.893009
4428.94248	0.1308695	0.03620239	3.61494078	0.00030042	0.00143508	4213.9442
6709.48951	0.24974152	0.03388252	7.37080734	1.70E-13	3.23E-12	6065.82036
16553.2098	0.23679286	0.03229542	7.33208908	2.27E-13	4.29E-12	14734.4527
5934.77318	0.12236147	0.03493067	3.50298138	0.00046008	0.00209753	5724.14921
49709.6579	0.1599488	0.02572858	6.21677433	5.07E-10	6.40E-09	47571.9207
10199.7688	0.10372333	0.03046444	3.40473487	0.00066228	0.0029079	9627.90415
13345.0477	0.16734406	0.0372586	4.49142045	7.07E-06	4.75E-05	12164.0485
3132.54987	-0.7761543	0.04252151	-18.253216	1.95E-74	5.82E-72	3912.08838
404.331872	-1.0720076	0.10551311	-10.159948	2.99E-24	1.31E-22	551.859095
480.298811	-1.8939179	0.10015793	-18.909315	9.56E-80	3.14E-77	739.824525
360.689271	-0.8554039	0.10539643	-8.1160616	4.82E-16	1.18E-14	471.302482
16359.3251	0.20307983	0.02944763	6.89630391	5.34E-12	8.61E-11	14727.9711
409.144987	-0.2586076	0.09770309	-2.6468724	0.008124	0.02624249	436.116835
850.192491	-0.4339976	0.07135995	-6.0818092	1.19E-09	1.42E-08	935.19746
557.588042	-0.5269659	0.08642888	-6.0971038	1.08E-09	1.30E-08	641.675089
81.3624279	-1.3530909	0.23625196	-5.7273215	1.02E-08	1.07E-07	117.594136
2292.48156	0.46745973	0.04702478	9.94071175	2.77E-23	1.15E-21	1990.76687
3407.6396	-0.1750181	0.04115128	-4.2530423	2.11E-05	0.00012851	3684.30762

44.506801	-2.3427371	0.35086225	-6.6770852	2.44E-11	3.64E-10	67.5934798
1142.5952	0.1485209	0.06011955	2.47042598	0.01349522	0.04068506	1065.75473
3936.99526	-0.6097614	0.03743053	-16.290482	1.15E-59	2.43E-57	4774.13674
1693.86157	0.30425966	0.05486892	5.54521006	2.94E-08	2.89E-07	1477.79717
1940.73374	0.23057618	0.0492471	4.68202534	2.84E-06	2.04E-05	1726.87452
208.079499	-0.6656481	0.13493798	-4.9329931	8.10E-07	6.38E-06	253.707034
228.951656	-0.5547604	0.13076275	-4.2424957	2.21E-05	0.00013403	279.6333
1198.66031	-0.5299009	0.06003284	-8.8268497	1.08E-18	3.27E-17	1392.61087
3116.21889	0.18132754	0.04287393	4.22931971	2.34E-05	0.00014142	2881.5193
2523.62696	0.22039892	0.05572631	3.95502418	7.65E-05	0.00041415	2218.54764
2673.57743	0.26705759	0.04230471	6.3127146	2.74E-10	3.57E-09	2431.5134
320.021972	0.54346795	0.10892565	4.98934772	6.06E-07	4.87E-06	248.151405
1020.77125	-0.2529475	0.06536082	-3.8700177	0.00010883	0.00057001	1110.19976
482.493564	-0.3345937	0.09451424	-3.540141	0.00039991	0.00185418	564.822228
13446.467	0.30299004	0.03520169	8.60725928	7.48E-18	2.14E-16	12239.9755
4198.20235	0.16162616	0.03690344	4.37970402	1.19E-05	7.62E-05	3891.71775
4802.55116	0.2452503	0.03746637	6.54587881	5.91E-11	8.42E-10	4403.7615
602.042635	0.4577535	0.08275072	5.53171641	3.17E-08	3.10E-07	499.080625
3806.23016	0.18748155	0.03802432	4.93056983	8.20E-07	6.46E-06	3535.23159
175.706339	-0.3785047	0.15513138	-2.4398975	0.01469143	0.04377882	166.668854
322.208877	0.38496551	0.10735635	3.58586629	0.00033596	0.00158496	272.225795
3689.25542	0.31019596	0.03933744	7.88551376	3.13E-15	7.07E-14	3276.89486
73.1446267	-0.8805337	0.22827745	-3.8572958	0.00011465	0.00059888	99.0753745
4988.33141	0.32896015	0.03511087	9.3691823	7.31E-21	2.61E-19	4430.61371
1214.58676	-0.1692297	0.06309827	-2.6820024	0.00731829	0.02398396	1305.57269
2128.73002	-0.4182861	0.05511944	-7.5887208	3.23E-14	6.65E-13	2528.73689
884.855575	-0.291162	0.07031614	-4.1407568	3.46E-05	0.00020157	943.530903
181.952365	0.38123396	0.14448389	2.6385914	0.00832513	0.02679764	158.335412
2383.05695	0.23123181	0.04704112	4.91552471	8.85E-07	6.94E-06	2249.10359
4176.84682	-0.176409	0.04298098	-4.104351	4.05E-05	0.00023246	4345.42741
371.440254	-0.4538815	0.11163918	-4.0656116	4.79E-05	0.0002691	456.487473
149.871442	-0.8238982	0.16216323	-5.080672	3.76E-07	3.13E-06	173.150421
218.149625	-0.738414	0.13329896	-5.5395334	3.03E-08	2.98E-07	272.225795
183.509274	-0.3605806	0.14293172	-2.5227471	0.01164421	0.03586203	208.336068
469.848858	-0.7096369	0.09338286	-7.5992199	2.98E-14	6.15E-13	552.785033
1515.85406	-0.2545047	0.06130437	-4.1514944	3.30E-05	0.00019316	1590.76162
1865.80956	-0.2759506	0.04933302	-5.5936296	2.22E-08	2.23E-07	2002.80407
356.657869	0.56503729	0.10384404	5.44121045	5.29E-08	5.06E-07	290.744557
995.081247	0.24870329	0.06482387	3.8366006	0.00012475	0.00064711	901.863689
754.35024	0.31046696	0.0741121	4.18915348	2.80E-05	0.00016639	644.452903
355.619413	0.50226623	0.10380783	4.83842357	1.31E-06	1.00E-05	294.448309
850.816476	-0.7655172	0.07340221	-10.429075	1.83E-25	8.70E-24	1103.71819
8153.04303	0.14805273	0.03212009	4.60934934	4.04E-06	2.82E-05	7876.95524

4372.63744	0.36774453	0.04296774	8.55861975	1.14E-17	3.22E-16	3833.38365
36.3945259	-1.7908492	0.36327761	-4.9296987	8.24E-07	6.48E-06	56.4822228
2344.33356	0.22608303	0.04631719	4.88119028	1.05E-06	8.17E-06	2093.546
20.355196	-1.9315505	0.47024848	-4.1075104	4.00E-05	0.00022958	35.185647
23.8831205	-1.7426652	0.44134705	-3.9485145	7.86E-05	0.00042474	34.2597089
3443.90717	0.28957243	0.03907341	7.4109845	1.25E-13	2.42E-12	3094.48506
8652.09964	0.41095595	0.03176038	12.939266	2.70E-38	2.37E-36	7235.28015
3407.01617	0.5093346	0.04165334	12.2279412	2.20E-34	1.63E-32	2706.51701
2084.82297	0.35438575	0.05139508	6.89532388	5.37E-12	8.66E-11	1729.65233
3336.71069	0.15199093	0.04140201	3.67110056	0.00024151	0.00117836	3288.93206
6783.92963	-0.4428243	0.0322985	-13.710368	8.80E-43	9.17E-41	7854.73273
7595.89347	-0.5378947	0.0331592	-16.221582	3.55E-59	7.32E-57	8995.48844
3675.5494	0.24865874	0.0417861	5.95075317	2.67E-09	3.02E-08	3304.673
1078.9149	-0.4936506	0.06578167	-7.5043795	6.17E-14	1.24E-12	1213.90482
4386.89596	-0.161929	0.03686265	-4.3927669	1.12E-05	7.21E-05	4535.24471
17926.3043	0.18495017	0.02772396	6.6711307	2.54E-11	3.77E-10	16408.5487
27706.5859	0.25814774	0.02643161	9.76663001	1.57E-22	6.26E-21	24804.0293
21349.5017	0.1712172	0.02646549	6.46945207	9.84E-11	1.36E-09	19645.6282
648.737996	-0.4120567	0.07773472	-5.3008057	1.15E-07	1.04E-06	743.528278
21505.0961	0.21300862	0.0271374	7.84926285	4.18E-15	9.30E-14	19454.885
807.162947	-0.9431467	0.08489202	-11.109958	1.12E-28	6.59E-27	1087.97724
50.1665726	-1.231318	0.28372017	-4.3399028	1.43E-05	8.99E-05	68.5194179
52408.6171	-0.1881637	0.02371315	-7.9349966	2.11E-15	4.83E-14	55279.4293
7508.59753	0.14001719	0.03670575	3.81458502	0.00013641	0.00070235	6986.20281
1460.54599	0.25360364	0.06123659	4.14137402	3.45E-05	0.00020109	1435.20402
5787.69143	-0.60724	0.03511488	-17.292956	5.32E-67	1.30E-64	6931.57246
6104.48675	-0.2966341	0.03357629	-8.8346285	1.00E-18	3.06E-17	6636.19821
6063.67674	-0.5410064	0.03488142	-15.509875	2.97E-54	5.06E-52	6986.20281
674.215093	-0.2423539	0.07691442	-3.1509557	0.00162737	0.00645521	750.009844
7272.06463	-0.0901968	0.0377808	-2.3873725	0.01696929	0.04957794	7154.72354
564.268503	0.29047698	0.08315119	3.49335948	0.00047698	0.00216689	521.303139
3792.47327	0.45718865	0.04404306	10.3804934	3.04E-25	1.43E-23	2992.63187
740.432522	0.35001675	0.07633964	4.58499361	4.54E-06	3.15E-05	607.41538
4860.88293	0.29363008	0.03738249	7.85474922	4.01E-15	8.93E-14	4302.83425
13490.6704	0.14808372	0.0325998	4.54247362	5.56E-06	3.79E-05	12657.5735
2653.7265	0.12439752	0.04442894	2.79992097	0.00511151	0.01760602	2498.18094
3687.58612	0.14403241	0.03792722	3.79760042	0.0001461	0.0007465	3508.37938
575.267612	-0.3442214	0.08210222	-4.1925963	2.76E-05	0.00016414	654.638222
2309.78297	0.40853844	0.04638156	8.80820865	1.27E-18	3.83E-17	1942.61809
2990.24928	-0.3762097	0.04136769	-9.0942876	9.52E-20	3.13E-18	3338.93271
3578.65168	0.2164968	0.03820216	5.66713537	1.45E-08	1.49E-07	3300.96925
1875.5784	0.27900377	0.05751057	4.85134759	1.23E-06	9.41E-06	1651.87353
2888.80631	0.15572561	0.04192332	3.71453448	0.00020358	0.00100956	2755.59172

4017.98659	0.11783298	0.03945529	2.98649354	0.00282197	0.01049172	3813.01301
4675.07355	0.27547805	0.03948079	6.97752045	3.00E-12	4.99E-11	4029.68252
10494.2686	0.25143183	0.03014033	8.34203987	7.30E-17	1.88E-15	9424.19777
14763.5512	-0.3313886	0.02729356	-12.141642	6.35E-34	4.60E-32	16388.1781
2906.11554	0.14689424	0.04216277	3.48397967	0.00049402	0.00223751	2720.40608
137.796546	-0.4951932	0.16790473	-2.9492509	0.00318545	0.01167297	179.631987
5661.05072	0.30585092	0.03333691	9.17454366	4.53E-20	1.53E-18	5025.99189
611.057064	0.35929037	0.0848308	4.2353763	2.28E-05	0.00013796	520.3772
2639.93504	-0.5260637	0.04376666	-12.019736	2.80E-33	1.99E-31	3125.04102
474.131864	-0.3038593	0.09330389	-3.2566627	0.0011273	0.00466283	507.414067
2610.01596	0.33411744	0.04389487	7.6117659	2.70E-14	5.60E-13	2254.65922
2172.27928	0.13447634	0.04624245	2.90807105	0.00363666	0.01310346	2102.80538
11232.5777	-0.0982804	0.03080769	-3.1901246	0.00142212	0.00572657	11322.3708
456.050969	-0.2423858	0.09515099	-2.5473804	0.01085351	0.03376046	459.265287
2819.21879	0.46421206	0.04260452	10.8958418	1.21E-27	6.72E-26	2355.58647
5109.85187	0.45453459	0.03795091	11.9769092	4.70E-33	3.31E-31	4297.27863
740.936305	0.20421562	0.07224463	2.82672377	0.00470269	0.01639892	693.527621
2985.80757	0.10488937	0.04235469	2.4764522	0.01326954	0.04012949	2935.22371
1815.60172	-0.2387968	0.0507921	-4.7014546	2.58E-06	1.87E-05	1904.65463
266.468672	0.31141758	0.12197151	2.55319942	0.01067384	0.03326565	217.595449
1125.66071	0.18494362	0.0611413	3.02485585	0.00248752	0.0093796	1056.49535
572.334939	0.26907268	0.0833885	3.22673589	0.00125211	0.00512287	510.191882
309.568355	0.50886951	0.11173223	4.55436643	5.25E-06	3.60E-05	262.966415
961.890122	0.35090656	0.06641215	5.28377041	1.27E-07	1.14E-06	866.678042
6131.37061	-0.1586033	0.03841935	-4.1282136	3.66E-05	0.00021181	6190.822
115.609629	0.68623918	0.18923169	3.62644964	0.00028735	0.00137705	94.4456841
1421.99055	-0.2498059	0.05709888	-4.3749697	1.21E-05	7.77E-05	1584.28005
1053.56122	0.26259301	0.06369677	4.12254818	3.75E-05	0.00021631	943.530903
3334.8204	0.1981022	0.04178271	4.74124859	2.12E-06	1.57E-05	3038.92878
3971.6834	-0.2895131	0.03768036	-7.6833968	1.55E-14	3.28E-13	4402.83557
988.772888	0.22856478	0.06514698	3.50844759	0.00045073	0.00205877	920.382451
706.842047	0.28474296	0.07950738	3.58134017	0.00034184	0.00161033	614.822885
18754.8756	0.23003709	0.02667005	8.62529615	6.39E-18	1.84E-16	16896.5181
2555.47242	0.20077639	0.04571768	4.39165781	1.12E-05	7.24E-05	2439.84684
1891.23132	-0.3524466	0.05220806	-6.7508088	1.47E-11	2.26E-10	2088.91631
1383.71861	0.27621337	0.05650668	4.88815414	1.02E-06	7.91E-06	1284.27612
43854.0909	0.1013192	0.03314534	3.05681617	0.00223701	0.00854642	42814.4508
770.299773	-0.1968125	0.0728929	-2.7000226	0.00693348	0.02289701	828.714581
2581.32072	-0.2466795	0.04770483	-5.1709542	2.33E-07	2.01E-06	2958.37216
19.4804726	-2.852454	0.56545538	-5.0445254	4.55E-07	3.72E-06	22.2225139
6007.54067	0.08073342	0.03304264	2.4433102	0.01455322	0.04341555	5917.67026
4347.00849	-0.2441777	0.03773918	-6.4701365	9.79E-11	1.36E-09	4817.65583
1897.72958	0.14519644	0.04930334	2.94496134	0.00322995	0.01180924	1821.3202

8687.56917	0.17231534	0.03413745	5.04769166	4.47E-07	3.67E-06	8293.62737
13125.688	0.07618883	0.02797557	2.7234061	0.00646126	0.02156534	13014.0597
20850.422	0.33449082	0.02590582	12.9118025	3.86E-38	3.34E-36	18400.2415
704.577283	0.60206913	0.08044941	7.48382279	7.22E-14	1.44E-12	530.562519
7215.54781	0.18715759	0.03198813	5.85084445	4.89E-09	5.35E-08	6666.75417
1419.31744	-0.6109652	0.05733671	-10.655741	1.64E-26	8.31E-25	1683.35543
3491.58	-0.2325612	0.04421275	-5.2600481	1.44E-07	1.28E-06	3744.49359
1741.24011	0.1297108	0.05359039	2.42041174	0.01550294	0.0458606	1642.61415
716.370697	0.72507558	0.07479185	9.69458015	3.18E-22	1.25E-20	526.858767
102.526999	-0.6288773	0.20034365	-3.138993	0.0016953	0.00669041	125.001641
4151.60002	0.16103953	0.03623293	4.44456333	8.81E-06	5.79E-05	3901.90307
1697.43862	-0.1592523	0.05158699	-3.0870639	0.00202144	0.00782628	1801.8755
3963.33769	0.24384895	0.04023923	6.0599811	1.36E-09	1.61E-08	3627.82539
790.947911	0.31420466	0.07224295	4.34927798	1.37E-05	8.64E-05	699.08325
555.101054	0.24294279	0.08962107	2.7107777	0.00671256	0.02227752	487.969368
964.108482	0.23335611	0.0720868	3.2371546	0.00120728	0.00495724	943.530903
2369.38702	-0.2562258	0.05086525	-5.0373452	4.72E-07	3.86E-06	2520.40345
7062.13002	0.41039293	0.04053086	10.1254443	4.26E-24	1.85E-22	5675.07449
2166.39088	0.14971331	0.05121799	2.92306086	0.00346609	0.01254952	1925.9512
589.365644	-0.4789159	0.08297874	-5.77155	7.85E-09	8.39E-08	679.63855
356.141329	-0.4497534	0.10902994	-4.125045	3.71E-05	0.00021442	425.005578
253.721136	-0.4593673	0.12550302	-3.6602089	0.00025201	0.00122436	305.559566
16.3769887	-1.829998	0.51583897	-3.5476148	0.00038874	0.00180581	25.0003281
220.650196	0.46120433	0.1308154	3.525612	0.00042251	0.00194543	188.891368
5540.8563	-0.1796897	0.04601816	-3.9047555	9.43E-05	0.00050107	6159.3401
4232.90895	0.23611768	0.04069251	5.80248569	6.53E-09	7.05E-08	3756.53079
652.674017	0.34559894	0.08142687	4.24428636	2.19E-05	0.00013313	581.489114
3053.9896	0.24328553	0.04819799	5.04762793	4.47E-07	3.67E-06	2773.18455
68.6882665	1.9741028	0.26407476	7.47554516	7.69E-14	1.52E-12	35.185647
4991.80843	0.41698496	0.03533338	11.8014448	3.84E-32	2.58E-30	4210.24045
915.460792	0.26579508	0.06775901	3.9226529	8.76E-05	0.0004686	811.121757
13738.3325	0.38195978	0.03294402	11.5942069	4.41E-31	2.86E-29	11865.8965
314.654085	-0.3120797	0.11171446	-2.793548	0.00521333	0.01792122	377.782736
3678.02565	0.19471422	0.04149941	4.69197562	2.71E-06	1.95E-05	3426.89683
1549.31418	-0.2073443	0.05361633	-3.8671848	0.0001101	0.00057636	1612.98413
380.190632	-0.2758855	0.10031177	-2.7502805	0.00595443	0.02008117	410.190569
48.9491895	-2.2976827	0.31598186	-7.2715651	3.55E-13	6.60E-12	73.1491083
398.944883	-0.4858737	0.10605443	-4.5813619	4.62E-06	3.19E-05	437.042773
8942.02969	0.20448164	0.02992033	6.83420309	8.25E-12	1.30E-10	8266.77517
74.2123258	-1.1074021	0.24070036	-4.6007497	4.21E-06	2.93E-05	95.3716221
5919.86612	0.10277933	0.03432601	2.99421146	0.00275155	0.01026339	5666.74104
213.21221	-0.5151071	0.13685579	-3.7638676	0.00016731	0.00084459	238.892024
1775.73547	-0.2183263	0.05496494	-3.9721002	7.12E-05	0.00038749	1925.02527

2705.16263	-0.1789845	0.04381188	-4.0852963	4.40E-05	0.00025003	2796.333
5621.50534	0.2101071	0.03382578	6.21144937	5.25E-10	6.58E-09	5274.1433
8548.71347	0.09688262	0.03585563	2.70201978	0.00689197	0.0227871	7964.91936
1173.9137	-0.6482701	0.06551238	-9.8953835	4.36E-23	1.78E-21	1390.759
1055.05117	0.16861881	0.06250577	2.69765182	0.00698304	0.02304107	994.457497
4316.42834	-0.1887955	0.03953819	-4.7750163	1.80E-06	1.34E-05	4637.0979
8668.97184	-0.4801321	0.03239771	-14.819937	1.09E-49	1.44E-47	10073.2804
1119.84946	-0.2467166	0.06231096	-3.9594418	7.51E-05	0.00040713	1193.53418
83.3691901	-0.8093488	0.2203447	-3.6731033	0.00023962	0.00117123	125.927579
2837.29529	-0.143193	0.04585057	-3.1230351	0.00178996	0.00702256	3050.04003
3373.51355	0.18952249	0.04226376	4.48427899	7.32E-06	4.90E-05	3178.74543
277.279729	0.66143646	0.12237639	5.40493509	6.48E-08	6.12E-07	232.410458
5126.10401	0.29182837	0.03746229	7.78992359	6.70E-15	1.46E-13	4565.80067
5211.98177	0.21328711	0.03381338	6.30777302	2.83E-10	3.68E-09	4775.06267
2998.94607	0.22158976	0.04320686	5.12857814	2.92E-07	2.47E-06	2675.03511
992.650155	-0.4015966	0.07375937	-5.444686	5.19E-08	4.97E-07	1021.3097
17026.88	-0.0853425	0.03184312	-2.6800933	0.00736017	0.02410081	17428.0065
4245.37878	0.21171057	0.03628604	5.83449119	5.40E-09	5.87E-08	3872.27305
21.6869635	1.03913666	0.41666194	2.49395626	0.01263281	0.03849215	12.037195
339.800311	0.49731727	0.11284068	4.40725146	1.05E-05	6.78E-05	291.670495
925.820576	-0.2541471	0.06667753	-3.8115855	0.00013808	0.00071036	1011.12438
1314.53523	0.47676766	0.05881239	8.10658557	5.21E-16	1.26E-14	1118.5332
3084.50295	0.21075372	0.0442998	4.75744126	1.96E-06	1.46E-05	2793.55519
13014.3397	0.32817568	0.03659787	8.96707015	3.05E-19	9.57E-18	11236.2586
4217.78839	0.14677803	0.04088077	3.59039308	0.00033018	0.00156301	3844.4949
1769.44644	0.27013377	0.05267238	5.12856559	2.92E-07	2.47E-06	1659.28104
5728.09947	0.19588055	0.03535677	5.54011432	3.02E-08	2.97E-07	5351.9221
2149.84065	0.3851364	0.05144233	7.48676011	7.06E-14	1.41E-12	1816.69051
63.7739146	0.67950207	0.25046266	2.71298748	0.00666796	0.02215226	62.0378513
1929.70717	0.27989069	0.05058205	5.53339996	3.14E-08	3.07E-07	1727.80046
2471.51617	0.18981543	0.04382991	4.33072882	1.49E-05	9.34E-05	2264.84454
398.356086	0.61026524	0.10114504	6.0335658	1.60E-09	1.88E-08	338.893337
5221.228	0.15460154	0.03531305	4.37802849	1.20E-05	7.67E-05	4826.91521
6231.39274	0.12303386	0.03521541	3.49375045	0.00047629	0.00216478	6020.44939
290.107281	-1.1255013	0.11908118	-9.4515464	3.34E-21	1.24E-19	384.264303
7071.97765	0.95437	0.03584963	26.6214734	3.83E-156	4.64E-153	4780.6183
20.8270575	-2.3383586	0.48873461	-4.784516	1.71E-06	1.28E-05	37.0375232
11716.295	-0.2527491	0.03725003	-6.7852067	1.16E-11	1.81E-10	12317.7543
1554.20473	0.37785075	0.05646962	6.69122212	2.21E-11	3.33E-10	1362.05491
371.072165	0.34314004	0.10799022	3.17751038	0.00148545	0.00595441	292.596433
805.346154	-0.9654082	0.07416109	-13.017718	9.70E-39	8.63E-37	1054.64347
4703.59515	-0.4007781	0.03761841	-10.653775	1.67E-26	8.45E-25	5267.66173
485.369841	-0.5575404	0.09274679	-6.011425	1.84E-09	2.12E-08	550.933157

5518.35766	-0.1961265	0.03962404	-4.9496846	7.43E-07	5.89E-06	5995.44906
45.2605608	-0.9078118	0.31874674	-2.8480661	0.00439858	0.01548047	79.6306748
416.068115	-0.24943	0.1002428	-2.4882582	0.01283705	0.03900411	420.375888
1370.97882	-0.4958792	0.06310205	-7.8583684	3.89E-15	8.71E-14	1534.2794
333.114541	-1.2177742	0.11647127	-10.455576	1.38E-25	6.65E-24	435.190897
231.672422	0.51600609	0.12964103	3.98026827	6.88E-05	0.000376	182.409802
13990.1142	0.08694378	0.03115059	2.7910799	0.00525325	0.01803334	13110.3573
1036.51877	0.24312235	0.06612223	3.67686261	0.00023612	0.00115644	966.679355
2561.45571	-0.221914	0.04418709	-5.0221461	5.11E-07	4.15E-06	2762.99923
4610.30097	0.14155946	0.03779946	3.74501303	0.00018038	0.00090496	4244.50015
8687.59742	0.14706654	0.03666287	4.01132141	6.04E-05	0.00033318	7856.5846
4384.16039	0.23810357	0.03748045	6.35274086	2.12E-10	2.80E-09	3930.60715
990.654073	0.22654093	0.06599847	3.43251818	0.000598	0.00265696	887.974618
518.923778	-0.3307792	0.08671646	-3.8144916	0.00013646	0.00070243	560.192538
51.9242792	-0.7807389	0.28154719	-2.7730303	0.0055537	0.01886757	51.8525324
245.095955	-0.4603048	0.1253093	-3.6733488	0.00023939	0.0011704	274.077671
12541.7373	0.21046875	0.03031042	6.94377623	3.82E-12	6.26E-11	11335.334
1160.10912	0.40638128	0.0610153	6.66031806	2.73E-11	4.05E-10	950.938407
164.154371	-1.0397406	0.15889838	-6.5434309	6.01E-11	8.53E-10	230.558582
1207.52454	0.32171386	0.06077294	5.29370233	1.20E-07	1.08E-06	1035.19877
11.7925385	-3.1092205	0.72980629	-4.2603367	2.04E-05	0.00012482	25.0003281
8276.1482	-0.4253812	0.03600327	-11.815072	3.26E-32	2.20E-30	9775.1283
570.984364	0.26527464	0.09089939	2.91833255	0.00351909	0.01271292	493.524996
432.061431	-0.5084448	0.10515766	-4.835071	1.33E-06	1.02E-05	454.635597
17148.4626	0.16067876	0.02743031	5.85770795	4.69E-09	5.15E-08	15912.2459
341.936485	0.25126989	0.1048439	2.39660957	0.01654755	0.04852128	326.856142
7361.63365	0.26879708	0.03212056	8.36838099	5.84E-17	1.52E-15	6714.90295
2414.90607	0.12393624	0.0463405	2.6744693	0.00748476	0.02445923	2265.77048
6816.438	-0.3569266	0.03307088	-10.792777	3.72E-27	1.97E-25	7588.9885
3731.01525	0.18395446	0.0381519	4.82163258	1.42E-06	1.08E-05	3456.52685
307.881811	-0.9300125	0.11728614	-7.9294322	2.20E-15	5.04E-14	418.524012
1289.81923	-0.3920662	0.05685665	-6.895697	5.36E-12	8.64E-11	1472.24155
2293.17803	0.18771354	0.04503701	4.16798372	3.07E-05	0.00018085	2123.17602
456.192551	0.40575333	0.0929569	4.36496204	1.27E-05	8.09E-05	408.338693
1256.96102	-0.3135904	0.0613635	-5.1103725	3.22E-07	2.70E-06	1347.23991
215.127332	-0.5231607	0.14030483	-3.7287432	0.00019244	0.0009602	240.743901
1734.55161	-0.4668342	0.05250315	-8.8915465	6.03E-19	1.87E-17	1992.61875
9515.10816	0.46054897	0.03356668	13.72042	7.66E-43	8.03E-41	7839.91772
2140.54338	0.35725191	0.0462516	7.72409861	1.13E-14	2.41E-13	1862.06148
1794.81277	0.22627744	0.05258931	4.30272732	1.69E-05	0.00010506	1591.68756
576.986565	-0.224245	0.08256636	-2.7159367	0.00660886	0.0219785	642.601027
1066.18327	-0.8028238	0.06692041	-11.996697	3.70E-33	2.63E-31	1370.38836
19.0431191	-1.3247129	0.46671713	-2.8383635	0.00453455	0.01588404	31.4818947

353.757693	-0.5257889	0.1080107	-4.8679329	1.13E-06	8.71E-06	430.561207
7924.48582	0.21549935	0.03229161	6.67354034	2.50E-11	3.72E-10	7153.7976
15537.6905	-0.1044211	0.0275197	-3.7944128	0.00014799	0.00075556	16131.6932
8294.65748	0.08633157	0.03026518	2.85250532	0.00433761	0.01529366	8000.105
2519.46465	0.12813643	0.04608061	2.78070142	0.00542416	0.01852817	2335.21584
2575.25705	0.27523885	0.04463756	6.1660818	7.00E-10	8.64E-09	2304.65988
172.789906	-0.4409866	0.14736532	-2.992472	0.00276728	0.01030817	205.558254
1902.2485	0.16825082	0.0503575	3.34112763	0.00083439	0.00356819	1813.9127
28.1937764	1.98617065	0.39238088	5.06184362	4.15E-07	3.42E-06	14.8150093
17.4105938	1.23429887	0.47590622	2.59357582	0.00949836	0.03010557	12.037195
53.4875961	-1.0316489	0.27023469	-3.8176037	0.00013475	0.00069474	63.8897275
16.6872166	-1.3367073	0.49984394	-2.6742493	0.00748968	0.02447116	29.6300185
46.1557731	1.36259089	0.30575417	4.45649163	8.33E-06	5.51E-05	33.3337709
123.079627	-1.219702	0.19486167	-6.2593222	3.87E-10	4.95E-09	185.187616
43.3493151	-1.5009676	0.31882583	-4.7077981	2.50E-06	1.82E-05	72.2231702
248.205329	0.35912044	0.12508258	2.87106683	0.00409089	0.01450291	239.817963
103.428233	-0.657702	0.19370499	-3.3953798	0.00068534	0.00299621	138.890712
6322.19762	0.09282293	0.03269169	2.83934375	0.00452064	0.01584677	6151.9326
105.569368	-0.7696061	0.19815006	-3.8839558	0.00010277	0.00054151	159.26135
3392.5095	-0.3241946	0.04095241	-7.9163739	2.45E-15	5.58E-14	3798.198
140.188027	-0.5439825	0.17214127	-3.1600934	0.00157719	0.00627798	191.669182
58.4738339	-0.8188887	0.27247197	-3.0054052	0.00265227	0.00992746	77.7787986
1452.82261	-0.3807872	0.06307428	-6.0371229	1.57E-09	1.84E-08	1665.7626
24.7736937	-2.4418934	0.45061434	-5.4190317	5.99E-08	5.69E-07	43.5190897
188.415494	0.49035173	0.14212557	3.45013021	0.00056032	0.00250559	148.150093
73.2440541	0.65361354	0.22240662	2.9388223	0.00329462	0.01201622	53.7044086
6889.86675	0.20338083	0.03371008	6.03323441	1.61E-09	1.88E-08	6290.82331
1359.04769	-0.4298928	0.05736147	-7.4944538	6.66E-14	1.33E-12	1605.57663
153.233181	0.54433445	0.16010396	3.39988132	0.00067415	0.00295265	116.668198
104.894433	-0.6818995	0.18864589	-3.6147066	0.00030069	0.00143603	135.18696
53.8127646	-0.7318622	0.26703295	-2.7407187	0.0061305	0.02060327	72.2231702
209.819258	-0.7539128	0.1367519	-5.5129967	3.53E-08	3.43E-07	278.707362
1700.99562	-0.5411562	0.05131165	-10.546459	5.27E-26	2.60E-24	2005.58188
8930.70097	0.24095003	0.02888541	8.34158324	7.33E-17	1.88E-15	8169.55167
95.4979667	0.57703336	0.19498387	2.95939029	0.00308248	0.01133206	75.9269225
7174.47011	0.2354805	0.03166119	7.43751275	1.03E-13	2.01E-12	6633.4204
67.268922	-0.7510456	0.2355588	-3.1883573	0.00143084	0.00575929	87.0381794
104.765426	0.46129507	0.19282064	2.39235312	0.01674073	0.04900619	91.6678698
1002.72877	-0.7705744	0.06958319	-11.074145	1.67E-28	9.72E-27	1313.90613
44.6516854	-0.7624506	0.29121254	-2.618193	0.00883968	0.02824414	56.4822228
102.761546	-0.5544108	0.18973617	-2.9220089	0.00347782	0.01258728	127.779455
6347.3648	0.12273752	0.03394993	3.61525123	0.00030006	0.00143372	6104.70976
603.97465	0.58440658	0.09206709	6.34761673	2.19E-10	2.89E-09	547.229405

11878.2383	0.39076611	0.02842623	13.7466749	5.33E-43	5.68E-41	10456.6187
76.0404174	-0.9277721	0.23794493	-3.8991043	9.65E-05	0.00051235	106.482879
191.329305	-0.3914731	0.15191037	-2.5770006	0.00996618	0.03137291	252.781096
112.514877	1.17132927	0.18848783	6.21434975	5.15E-10	6.48E-09	59.2600371
121.775132	-0.5292113	0.17988349	-2.9419672	0.00326135	0.01191317	147.224155
2109.62478	-0.1250257	0.05033202	-2.4840196	0.01299087	0.03940364	2143.54665
15.4496895	-1.782153	0.53109235	-3.3556368	0.00079183	0.00340043	22.2225139
322.071466	-0.6487721	0.11030356	-5.8816968	4.06E-09	4.49E-08	396.301498
326.515455	-0.673513	0.12305775	-5.4731457	4.42E-08	4.26E-07	398.153374
195.462116	-1.560576	0.15510577	-10.061367	8.19E-24	3.50E-22	307.411442
425.920221	-0.3423695	0.09803471	-3.4923294	0.00047883	0.00217321	501.858439
956.899438	0.17660763	0.07313208	2.41491313	0.01573896	0.04647369	847.233342
4477.42626	0.11675741	0.03543583	3.29489712	0.00098458	0.00413028	4286.16737
47.7012725	0.84637191	0.28963206	2.92223142	0.00347533	0.01258064	37.0375232
23178.5741	0.11374022	0.0255321	4.45479307	8.40E-06	5.55E-05	21932.6953
18444.9013	0.33726073	0.02688147	12.5462156	4.17E-36	3.27E-34	16077.0629
3699.51592	0.25168352	0.03997386	6.29620236	3.05E-10	3.95E-09	3265.78361
2660.36292	0.20927083	0.04405953	4.74972874	2.04E-06	1.51E-05	2420.40214
2727.41065	0.11307725	0.0443751	2.54821394	0.01082761	0.0336853	2593.55256
243.831911	-0.6424828	0.12609182	-5.0953565	3.48E-07	2.91E-06	294.448309
3768.93436	-0.4161792	0.03922489	-10.610078	2.68E-26	1.34E-24	4357.4646
346.589918	0.47324266	0.11014318	4.29661355	1.73E-05	0.00010779	277.781424
1193.71315	0.27262752	0.0642108	4.2458204	2.18E-05	0.00013239	1102.79225
1262.68833	0.18856356	0.05972146	3.15738344	0.00159192	0.00632882	1154.64479
1135.99844	0.51557243	0.06765957	7.62009636	2.53E-14	5.26E-13	910.197132
2005.29469	0.21241881	0.05186479	4.0956263	4.21E-05	0.00024012	1817.61645
23.8941758	-1.6237449	0.42568909	-3.8143917	0.00013652	0.00070253	41.6672136
40.8025889	-0.7396709	0.31014247	-2.384939	0.01708195	0.04983512	60.1859751
532.710961	0.44448795	0.09196565	4.83319543	1.34E-06	1.03E-05	446.302154
6106.08939	0.09505692	0.03547761	2.67934921	0.00737654	0.02413813	5990.81937
2144.47904	-0.5953522	0.04708252	-12.644867	1.19E-36	9.53E-35	2611.14538
1270.74391	0.26988179	0.05696537	4.73764647	2.16E-06	1.60E-05	1143.53353
7748.63531	0.13490089	0.04551965	2.96357474	0.00304088	0.01120462	7646.39666
7185.54113	0.17916275	0.03633553	4.93078708	8.19E-07	6.45E-06	6457.49216
2050.11075	0.48508534	0.0489964	9.90042862	4.14E-23	1.70E-21	1640.76228
252.922092	-2.2751261	0.13979052	-16.275253	1.48E-59	3.08E-57	432.413083
20533.7357	-0.6780043	0.02603304	-26.043991	1.57E-149	1.79E-146	25203.1086
552.679206	0.42993996	0.08815394	4.87714982	1.08E-06	8.34E-06	492.599058
1254.08422	0.19143552	0.05904554	3.24216731	0.00118624	0.00487809	1144.45947
81.4154821	-1.0077237	0.22336665	-4.5115226	6.44E-06	4.35E-05	109.260693
34.6611557	-1.275487	0.34868777	-3.6579632	0.00025423	0.00123328	36.1115851
506.323726	0.28339551	0.09009199	3.14562404	0.00165733	0.00656195	470.376544
25.7070658	-1.9616796	0.43147841	-4.5464144	5.46E-06	3.73E-05	37.9634612

375.612794	-0.3193177	0.11128202	-2.8694454	0.00411192	0.01457214	425.931516
293.734135	-0.3546578	0.1126975	-3.1469891	0.00164961	0.0065354	318.522699
148.304104	-0.5243486	0.16381283	-3.2009008	0.00136999	0.00553967	177.780111
2171.61787	-0.1724875	0.05228195	-3.2991789	0.00096968	0.00407485	2256.5111
2326.32417	0.25041523	0.04735579	5.28795371	1.24E-07	1.11E-06	2127.80571
3877.61819	-0.1999488	0.04685991	-4.2669479	1.98E-05	0.0001216	4131.53571
6768.8564	0.08871417	0.03457517	2.56583477	0.01029279	0.03224917	6734.34765
15477.5346	0.16536351	0.02779031	5.95040239	2.67E-09	3.02E-08	14341.8549
1374.78192	0.35759691	0.06256895	5.71524503	1.10E-08	1.15E-07	1188.90449
7431.37552	0.13431685	0.03241055	4.14423238	3.41E-05	0.00019902	6950.09122
221.858948	-0.8118575	0.13600204	-5.9694507	2.38E-09	2.71E-08	295.374247
1664.59895	0.22580057	0.05569578	4.05417753	5.03E-05	0.00028163	1481.50093
5125.02661	-0.1998823	0.03647031	-5.4806856	4.24E-08	4.09E-07	5389.88556
87.9819326	-0.6869284	0.207956	-3.3032392	0.00095575	0.00402415	112.038508
7201.0314	0.44718837	0.03823666	11.6952798	1.35E-31	8.88E-30	5843.59522
4105.80338	0.16917321	0.03724959	4.541613	5.58E-06	3.81E-05	3783.38299
17305.7274	0.1040315	0.0294133	3.53688662	0.00040487	0.00187449	16193.7311
2792.27437	0.25222731	0.04499836	5.60525524	2.08E-08	2.10E-07	2459.29154
825.22801	0.21241617	0.06919869	3.0696561	0.00214305	0.00821822	770.380482
3759.54136	0.53143445	0.04370571	12.159383	5.11E-34	3.74E-32	3001.89125
2579.19798	0.40945747	0.04441029	9.21987767	2.97E-20	1.02E-18	2147.25041
1086.01816	-0.3091504	0.06532691	-4.7323598	2.22E-06	1.63E-05	1183.34887
907.117274	0.3246594	0.06763016	4.80051231	1.58E-06	1.19E-05	808.343943
3733.1627	0.29959394	0.04185631	7.15767683	8.21E-13	1.46E-11	3239.85734
1599.20252	-0.1536732	0.05288455	-2.905825	0.00366286	0.01317828	1728.72639
2863.4088	0.44843882	0.04369783	10.2622684	1.04E-24	4.69E-23	2368.54961
17636.763	0.36500877	0.02700347	13.5171054	1.24E-41	1.23E-39	15456.6844
935.687659	0.29950036	0.06647268	4.5056158	6.62E-06	4.47E-05	851.863033
2009.88358	0.45564871	0.04874683	9.34724841	9.00E-21	3.20E-19	1737.98577
670.241692	0.3590729	0.07795519	4.60614513	4.10E-06	2.86E-05	584.266928
4078.39266	0.14023636	0.03833126	3.65853781	0.00025366	0.00123114	3990.79312
89.0383223	-1.0734904	0.21912831	-4.8989125	9.64E-07	7.51E-06	136.112898
1915.41457	0.21684401	0.05031476	4.30974958	1.63E-05	0.00010204	1785.20862
8323.11382	0.19692382	0.03098022	6.35643797	2.06E-10	2.74E-09	7613.06289
4901.55552	0.14253268	0.03718565	3.83300269	0.00012659	0.00065578	4546.35597
6223.24227	0.15278381	0.03585017	4.26173218	2.03E-05	0.00012416	5722.29733
2691.04158	-0.3333629	0.04574899	-7.2867811	3.17E-13	5.91E-12	2925.96433
2208.94463	-0.2110916	0.04973697	-4.2441588	2.19E-05	0.00013317	2271.32611
16960.0098	0.22896473	0.02627521	8.71409721	2.93E-18	8.62E-17	15418.7209
13253.1532	0.15116861	0.02800861	5.39721906	6.77E-08	6.36E-07	12352.014
18163.2648	0.14376286	0.03092558	4.64867095	3.34E-06	2.38E-05	16813.1836
1163.83933	0.35408098	0.06304277	5.6165198	1.95E-08	1.97E-07	1079.6438
2256.88772	-0.1488563	0.04727532	-3.1487105	0.00163993	0.00649969	2312.06738

7954.79408	0.07853805	0.03033719	2.58883704	0.00963007	0.03045828	7647.3226
1377.66145	0.14790656	0.05568185	2.65627977	0.0079008	0.0256287	1308.35051
6879.44454	0.1363137	0.03296782	4.13474999	3.55E-05	0.00020624	6463.04779
605.91253	-0.287243	0.08260066	-3.4774907	0.00050613	0.00228383	708.342631
6024.54516	0.29306291	0.03927822	7.46120608	8.57E-14	1.68E-12	5544.51722
1096.17827	0.19289348	0.06638445	2.90570275	0.00366429	0.01318099	994.457497
252.101557	-0.4058611	0.1283779	-3.1614559	0.00156983	0.00625896	257.410786
5989.59962	0.2168429	0.03883441	5.58378284	2.35E-08	2.36E-07	5730.63077
1824.21694	0.40076957	0.05088381	7.87617041	3.38E-15	7.60E-14	1575.94661
1348.21423	0.4647824	0.05917159	7.85482329	4.00E-15	8.93E-14	1068.53254
3415.37285	0.82462111	0.03941746	20.9202002	3.51E-97	1.51E-94	2448.18028
3609.96163	0.28186032	0.04402622	6.40210171	1.53E-10	2.07E-09	3385.22962
4278.45818	0.13388352	0.04007251	3.3410313	0.00083468	0.00356865	4097.276
10308.6478	0.16107903	0.03568025	4.51451507	6.35E-06	4.30E-05	9530.68065
1399.08857	-0.272976	0.05674798	-4.8103211	1.51E-06	1.14E-05	1562.98348
1091.70651	-2.092131	0.06943721	-30.129824	1.97E-199	6.37E-196	1784.28268
5921.34679	0.28523692	0.03286204	8.67983072	3.96E-18	1.16E-16	5379.70024
4455.75132	0.21599148	0.03855051	5.60281809	2.11E-08	2.12E-07	3975.97811
2838.92102	0.28945412	0.04690105	6.17159155	6.76E-10	8.37E-09	2502.81063
2553.54066	0.29317964	0.04635186	6.32508969	2.53E-10	3.32E-09	2200.95481
315.307824	-0.608615	0.12167483	-5.0019794	5.67E-07	4.58E-06	438.89465
9894.96691	0.09739086	0.03147172	3.09455125	0.00197111	0.00765884	9419.56808
15.203066	-2.9493185	0.63708648	-4.6293849	3.67E-06	2.59E-05	33.3337709
4630.78535	-0.1063313	0.04010641	-2.6512295	0.00801993	0.0259565	4830.61896
6197.96106	0.23737786	0.03392513	6.99711095	2.61E-12	4.37E-11	5763.96454
91.2687851	0.97273138	0.20205889	4.81409842	1.48E-06	1.12E-05	63.8897275
890.353834	0.17747758	0.06819586	2.60246831	0.00925554	0.02943218	831.492395
291.489104	0.80797263	0.11572963	6.98155375	2.92E-12	4.86E-11	229.632644
7466.90349	0.2365071	0.03337153	7.0870909	1.37E-12	2.37E-11	6674.16167
128.059213	-0.4257869	0.1752293	-2.4298844	0.01510364	0.04481617	150.001969
3874.50291	0.45881521	0.04118525	11.1402798	7.99E-29	4.72E-27	3145.41166
3342.34802	-0.1522347	0.04443607	-3.425926	0.00061271	0.0027142	3510.23126
5289.50781	-0.1814844	0.04485956	-4.0456139	5.22E-05	0.00029137	5563.03598
297.189757	-0.7478017	0.11593283	-6.4503016	1.12E-10	1.54E-09	351.85647
4077.00801	0.12310511	0.04597908	2.6774158	0.00741925	0.0242615	3714.86357
190.024526	-0.5313075	0.14250633	-3.728308	0.00019277	0.00096162	224.077015
9222.27568	0.25411675	0.03003562	8.460513	2.66E-17	7.30E-16	8304.73863
2863.78449	0.10073468	0.04134385	2.43650954	0.01482978	0.0441234	2792.62925
5993.88219	0.17878813	0.03396702	5.26358073	1.41E-07	1.26E-06	5699.14888
2750.66427	0.36021525	0.04246136	8.4833652	2.19E-17	6.04E-16	2444.47653
3326.25178	-0.2372445	0.04035064	-5.8795725	4.11E-09	4.54E-08	3648.19603
299.001719	-0.310543	0.11329732	-2.7409565	0.00612606	0.02059194	347.22678
8784.8276	0.09459348	0.03365099	2.81101636	0.00493853	0.0170983	8459.37029

603.680634	0.38876106	0.08602045	4.51940291	6.20E-06	4.21E-05	561.118476
3595.65307	0.23371681	0.03923445	5.95692842	2.57E-09	2.91E-08	3240.78328
2076.56265	-0.2520095	0.05002311	-5.037862	4.71E-07	3.85E-06	2362.06804
2814.09084	0.192916	0.04153677	4.64446317	3.41E-06	2.42E-05	2626.88633
44.0112156	-1.4466539	0.32213771	-4.4907936	7.10E-06	4.76E-05	65.7416036
12258.0567	0.17243077	0.03028172	5.69422071	1.24E-08	1.29E-07	11462.1875
4117.22449	0.12063789	0.04006321	3.0111891	0.00260227	0.00976102	4071.34973
1317.14509	0.49177789	0.06304575	7.80033331	6.17E-15	1.36E-13	1105.57007
7127.88018	0.12552345	0.03266887	3.8422959	0.00012189	0.00063312	6665.82823
1857.69625	0.26700808	0.05522776	4.83467135	1.33E-06	1.02E-05	1750.94891
2160.80217	-0.1402917	0.05275416	-2.6593485	0.00782919	0.02543516	2349.10491
1775.35664	-0.2086713	0.04995397	-4.1772724	2.95E-05	0.00017447	1887.98774
2280.69682	0.21170964	0.04718059	4.48721922	7.22E-06	4.84E-05	2082.43474
463.143609	0.37799739	0.09630013	3.92520123	8.67E-05	0.0004643	391.671807
210.479925	-0.4123231	0.13493401	-3.0557392	0.00224507	0.00857211	235.188272
786.796634	0.303496	0.07437952	4.08037046	4.50E-05	0.00025486	688.897931
1862.98039	-0.5693757	0.05735477	-9.9272594	3.17E-23	1.31E-21	2140.76884
4058.84192	0.13891008	0.0395035	3.51639937	0.00043744	0.00200469	3926.90339
4666.03156	0.41102912	0.04437514	9.26259846	2.00E-20	6.87E-19	3717.64139
3426.52364	0.21692403	0.04293131	5.05281701	4.35E-07	3.58E-06	3092.63318
3007.75054	-0.2704295	0.04299546	-6.289722	3.18E-10	4.10E-09	3156.52291
1873.17933	0.25651784	0.05173115	4.95867239	7.10E-07	5.63E-06	1669.46636
2842.19421	0.17898129	0.04600263	3.89067516	1.00E-04	0.0005286	2624.10852
369.472136	-0.4633877	0.10233441	-4.5281711	5.95E-06	4.05E-05	409.264631
56.4567063	2.26240906	0.28259092	8.00595088	1.19E-15	2.79E-14	19.4446997
2278.31357	0.54556134	0.04581571	11.9077359	1.08E-32	7.56E-31	1834.28334
1243.22756	-0.1502898	0.05934634	-2.5324184	0.01132788	0.03500572	1262.97954
1869.1365	0.16903394	0.04870254	3.47074191	0.00051902	0.0023344	1768.54173
589.280886	-0.2446336	0.08130722	-3.0087555	0.0026232	0.00983193	659.267912
7808.73326	0.2204418	0.03490223	6.31598022	2.68E-10	3.50E-09	6958.42466
49678.4945	-0.2880323	0.03267261	-8.8157122	1.19E-18	3.60E-17	54805.349
57.7051932	0.84718113	0.26662397	3.17743797	0.00148583	0.00595466	53.7044086
999.671845	0.19754128	0.0641386	3.07991257	0.00207061	0.00797513	917.604636
2655.46826	0.10727262	0.04419041	2.42750899	0.01520291	0.04507623	2511.14407
227.358835	0.32670741	0.12853306	2.54181626	0.01102781	0.03421481	204.632316
2063.59128	-0.1602825	0.04960138	-3.2314114	0.00123181	0.00504832	2167.62104
4287.29114	0.33965192	0.03831043	8.86578283	7.60E-19	2.33E-17	3707.45607
336.063453	0.3457885	0.10707792	3.22931669	0.00124086	0.00508222	306.485504
4095.22881	-0.2189359	0.0366556	-5.9727829	2.33E-09	2.66E-08	4372.27961
2016.79088	0.17843467	0.05201486	3.4304554	0.00060257	0.00267371	1841.69084
3938.65508	-0.3513958	0.03914921	-8.9758067	2.81E-19	8.88E-18	4364.87211
1453.81498	0.17441145	0.05703891	3.05776256	0.00222996	0.00852364	1355.57335
2639.51743	0.24151814	0.04545268	5.31361729	1.07E-07	9.78E-07	2412.99463

104.864305	0.86601379	0.20443397	4.23615402	2.27E-05	0.00013753	64.8156655
1049.04034	-0.2787778	0.06791052	-4.1050761	4.04E-05	0.00023188	1175.94136
1369.78565	0.23092996	0.05578744	4.13946152	3.48E-05	0.00020265	1225.01608
3703.87708	0.18440029	0.04484699	4.11176473	3.93E-05	0.00022593	3608.38069
2033.2044	-0.1507892	0.05239202	-2.8780949	0.00400085	0.01422793	2188.91762
180.509757	-0.6606966	0.14827777	-4.4558039	8.36E-06	5.53E-05	239.817963
99.6668046	-0.8265271	0.20139521	-4.1040059	4.06E-05	0.00023268	123.149765
2036.10453	0.16289324	0.05696058	2.85975421	0.0042397	0.01496476	2014.84126
2032.99837	0.17962225	0.05700153	3.15118295	0.00162611	0.00645151	1785.20862
1980.13602	-0.3630969	0.05452328	-6.6594838	2.75E-11	4.07E-10	2301.88207
9210.45068	0.15377138	0.03085097	4.98432873	6.22E-07	4.99E-06	8528.81565
3156.86661	0.26935979	0.04539139	5.93416047	2.95E-09	3.32E-08	2850.96335
63.0012582	-1.3081187	0.25928427	-5.045114	4.53E-07	3.72E-06	91.6678698
6124.34402	0.23261699	0.03454276	6.73417494	1.65E-11	2.52E-10	5645.44447
534.53104	0.24565359	0.08575119	2.86472519	0.00417371	0.01476144	493.524996
3664.42075	0.12460236	0.04083964	3.05101488	0.00228069	0.00868931	3500.04594
5696.18821	0.16103678	0.04091874	3.93552633	8.30E-05	0.00044651	5170.43823
746.662607	0.99962567	0.078199	12.7831006	2.04E-37	1.70E-35	465.746854
87463.8778	0.4643811	0.02995913	15.5004846	3.44E-54	5.80E-52	71794.4608
15534.1805	0.28800859	0.04312481	6.67848993	2.41E-11	3.61E-10	14371.4849
26.4664534	-1.0147182	0.41397529	-2.4511565	0.0142398	0.04263803	37.9634612
75285.5263	0.31490381	0.02469416	12.7521552	3.03E-37	2.48E-35	66373.0934
176055.164	0.411261	0.02938316	13.9964879	1.64E-44	1.82E-42	146727.852
629.722688	-1.1202522	0.08267592	-13.549922	7.93E-42	7.88E-40	876.863361
118.169291	-1.6896123	0.18926546	-8.9272089	4.37E-19	1.37E-17	177.780111
1196.434	-0.1498673	0.06005609	-2.4954556	0.01257955	0.03836001	1239.83109
1116.7126	-0.4268297	0.06121378	-6.9727717	3.11E-12	5.14E-11	1300.943
1994.01591	-0.445033	0.05072352	-8.7737004	1.73E-18	5.18E-17	2202.80669
750.356086	0.34057737	0.07443242	4.5756587	4.75E-06	3.27E-05	654.638222
1337.05691	-0.1831893	0.05663489	-3.2345659	0.00121828	0.00499922	1425.94464
5810.10488	0.28737591	0.03444642	8.34269342	7.26E-17	1.87E-15	5124.14133
1806.35833	-0.3149091	0.05265808	-5.9802627	2.23E-09	2.55E-08	1950.95153
223.044519	-0.3262409	0.13103927	-2.4896421	0.01278718	0.03888916	261.114538
8504.39865	-0.5855661	0.03015022	-19.421619	5.07E-84	1.82E-81	10179.7632
2617.84162	-0.5583076	0.04592139	-12.157899	5.21E-34	3.80E-32	3141.7079
1055.46967	0.47778505	0.06200061	7.70613451	1.30E-14	2.75E-13	880.567113
5920.04662	0.17164634	0.03694193	4.64638274	3.38E-06	2.40E-05	5566.73973
4251.55787	0.21696306	0.03656797	5.93314468	2.97E-09	3.34E-08	3881.53243
11269.7429	-0.2238807	0.03215802	-6.9618911	3.36E-12	5.54E-11	12318.6802
15.092701	-1.3069678	0.52963228	-2.4676891	0.01359884	0.04097194	25.9262662
14520.0971	0.19221562	0.02739094	7.01748978	2.26E-12	3.81E-11	13325.1749
3023.72965	0.20494961	0.04174723	4.90929806	9.14E-07	7.14E-06	2775.96236
4468.78226	0.1880311	0.03971787	4.73416853	2.20E-06	1.62E-05	4064.86817

14482.2915	0.16941201	0.0311825	5.43292019	5.54E-08	5.29E-07	13364.9902
9296.4501	0.32718376	0.03189878	10.2569351	1.10E-24	4.93E-23	7988.06781
43522.2878	0.25039802	0.02324362	10.7727643	4.63E-27	2.43E-25	39910.709
952.216814	-0.2906009	0.06667326	-4.3585828	1.31E-05	8.31E-05	1021.3097
1295.58266	0.20389481	0.05786248	3.52378301	0.00042543	0.00195752	1229.64577
9185.09702	0.17665637	0.03042675	5.80595576	6.40E-09	6.92E-08	8579.74224
45426.5705	0.12187341	0.02770559	4.39887528	1.09E-05	7.03E-05	43043.1575
6983.01878	0.27793344	0.03321691	8.36722647	5.90E-17	1.53E-15	6300.08269
5403.58336	0.1704683	0.03522976	4.83875894	1.31E-06	9.99E-06	4963.95404
4627.77463	0.15951515	0.0363566	4.38751512	1.15E-05	7.37E-05	4276.90799
11943.806	0.33742222	0.03347502	10.0798202	6.79E-24	2.92E-22	10294.5796
4419.43799	-0.4928132	0.03660536	-13.46287	2.59E-41	2.52E-39	5128.77102
2330.16941	0.22723359	0.04486379	5.06496596	4.08E-07	3.37E-06	2171.3248
1697.13979	0.18077679	0.05856001	3.08703464	0.00202164	0.00782628	1559.27973
3095.01721	0.73243993	0.04189466	17.4828948	1.93E-68	4.87E-66	2269.47423
2939.10707	-0.510735	0.04322193	-11.816572	3.20E-32	2.17E-30	3482.45312
1183.29533	0.18214085	0.06030751	3.02020157	0.00252607	0.00951015	1125.9407
1150.04114	-0.2659725	0.06391229	-4.1615234	3.16E-05	0.00018548	1260.20173
662.476834	-0.1970374	0.07911268	-2.4905921	0.01275304	0.03879751	700.009188
1447.61356	-0.3912297	0.05971051	-6.5521076	5.67E-11	8.10E-10	1588.90974
381.046896	-0.669409	0.10226778	-6.5456494	5.92E-11	8.42E-10	473.154358
2406.49512	0.32351002	0.04861644	6.65433435	2.85E-11	4.20E-10	2052.80472
2827.60636	0.18573798	0.04914113	3.77968498	0.00015703	0.00079769	2572.25598
1515.99996	-0.3884619	0.05635235	-6.8934472	5.45E-12	8.77E-11	1787.06049
75387.9867	-0.2283363	0.02818937	-8.1000867	5.49E-16	1.33E-14	78638.9951
2046.20359	-0.6927985	0.04834635	-14.329902	1.42E-46	1.65E-44	2498.18094
3870.9239	-0.3031186	0.03714891	-8.1595562	3.36E-16	8.27E-15	4277.83393
1550.52838	0.2281379	0.0559626	4.07661331	4.57E-05	0.00025833	1432.42621
2012.17597	-0.2972903	0.04895508	-6.0727161	1.26E-09	1.49E-08	2253.73328
325.415295	-1.4090502	0.12058303	-11.685311	1.52E-31	9.96E-30	460.191225
5241.18671	0.24211784	0.04405614	5.49566581	3.89E-08	3.77E-07	4725.06202
7456.69231	0.17116289	0.03671515	4.6619141	3.13E-06	2.24E-05	6906.57213
1226.5399	-0.5123503	0.0600347	-8.5342365	1.41E-17	3.95E-16	1470.38967
427.892231	-0.4525721	0.09465954	-4.7810511	1.74E-06	1.31E-05	486.117492
2045.29036	0.36231326	0.05006736	7.23651614	4.60E-13	8.44E-12	1882.43212
2664.06504	0.31001982	0.04286112	7.23312427	4.72E-13	8.64E-12	2350.95678
2060.00956	-0.7249877	0.04787363	-15.143779	8.33E-52	1.20E-49	2575.0338
1901.91382	0.32347783	0.05543676	5.83507854	5.38E-09	5.85E-08	1701.87419
1795.31653	0.26080962	0.05308062	4.91346253	8.95E-07	7.01E-06	1586.13193
11168.0089	0.24297044	0.0326958	7.43124223	1.08E-13	2.09E-12	10106.6141
6769.86235	0.22552525	0.03607252	6.25199526	4.05E-10	5.17E-09	6020.44939
1968.41678	0.2076771	0.04908862	4.23065663	2.33E-05	0.00014071	1805.57925
9120.07918	0.18859045	0.03456753	5.45571161	4.88E-08	4.67E-07	8207.51513

9489.15878	0.12485135	0.02953409	4.22736465	2.36E-05	0.00014243	8974.19186
4342.18297	-0.2030875	0.03974065	-5.1103201	3.22E-07	2.70E-06	4498.20719
2501.37063	0.1316932	0.04723304	2.78815861	0.00530086	0.01817102	2281.51143
32.1272919	-1.2327249	0.36154562	-3.4095972	0.00065059	0.0028638	49.0747182
1957.48988	0.28391224	0.04950872	5.73459021	9.77E-09	1.03E-07	1776.87517
1183.53261	0.46407524	0.06420397	7.22813952	4.90E-13	8.95E-12	1059.27316
8545.89263	0.11278486	0.0300718	3.75051876	0.00017647	0.00088715	8208.44107
1013.01758	-0.3052951	0.06452447	-4.7314625	2.23E-06	1.64E-05	1090.75506
8431.89077	-0.2051259	0.0360975	-5.6825512	1.33E-08	1.37E-07	9032.52596
5468.02324	-0.2180583	0.03579297	-6.09221	1.11E-09	1.34E-08	6008.4122
6393.25177	0.1880803	0.03875274	4.8533422	1.21E-06	9.33E-06	6008.4122
4099.09874	-0.2896708	0.0379947	-7.6239795	2.46E-14	5.11E-13	4477.83655
1978.39496	0.2486636	0.04980001	4.9932436	5.94E-07	4.78E-06	1762.9861
3369.29133	0.4448846	0.04939748	9.00622138	2.13E-19	6.85E-18	2760.22141
1776.97378	-0.4381339	0.05430157	-8.0685312	7.11E-16	1.70E-14	1962.06279
18574.5951	0.16270079	0.02535642	6.41655114	1.39E-10	1.90E-09	17598.3791
10612.6233	-0.0667735	0.02788852	-2.3943012	0.01665207	0.04876138	10931.625
11827.6225	0.17549312	0.0302739	5.79684468	6.76E-09	7.29E-08	10905.6987
522.164853	0.2224717	0.09290227	2.39468527	0.01663464	0.04873242	474.080297
1539.20119	-0.1995531	0.05928472	-3.3660133	0.00076263	0.0032889	1612.98413
22886.4401	-0.6285164	0.02496207	-25.178859	6.83E-140	6.02E-137	27611.4735
842.234003	-0.6281285	0.07768788	-8.0852831	6.20E-16	1.49E-14	1075.01411
3820.39455	0.19497023	0.03718417	5.24336612	1.58E-07	1.40E-06	3568.56536
316.901169	0.28137583	0.11159269	2.52145403	0.01168709	0.03597814	275.00361
977.314296	0.19171879	0.06577008	2.91498487	0.00355706	0.01284052	916.678698
43.1306197	-0.7575937	0.30231748	-2.5059541	0.01221215	0.03739259	62.9637894
578.04274	0.66810743	0.08271103	8.07761006	6.60E-16	1.58E-14	433.339021
1471.00319	0.1966149	0.05565765	3.53257653	0.00041153	0.00190203	1368.53648
8150.56132	-0.1582124	0.02979318	-5.3103557	1.09E-07	9.95E-07	8602.89069
11823.6581	0.08929339	0.02863623	3.11819602	0.00181962	0.00712736	11560.3369
773.989762	0.49021689	0.08011213	6.11913412	9.41E-10	1.14E-08	594.452247
511.820149	0.37518975	0.0888768	4.22145893	2.43E-05	0.00014599	435.190897
73.497718	-0.6066838	0.22597865	-2.684695	0.0072596	0.02380368	91.6678698
9905.50414	0.30752701	0.02939998	10.4601083	1.32E-25	6.37E-24	8870.4868
966.582746	-0.2260292	0.07206859	-3.1363066	0.0017109	0.00674651	1086.12537
27.6619516	-1.1403762	0.39130148	-2.9143162	0.00356469	0.01286326	29.6300185
316.333421	0.6507163	0.11118209	5.85270777	4.84E-09	5.29E-08	262.966415
2860.96123	-0.1320297	0.0446457	-2.9572764	0.0031037	0.01140356	2890.77868
531.059457	0.40296337	0.08837851	4.55951771	5.13E-06	3.51E-05	431.487145
2777.35977	0.14035567	0.04424044	3.17256492	0.00151099	0.00604302	2574.10786
1425.0749	0.25030392	0.06155672	4.06623224	4.78E-05	0.00026854	1298.16519
101.891557	-0.5394381	0.19770716	-2.72847	0.00636289	0.02128509	119.446012
584.119489	0.22192327	0.08408191	2.63937014	0.00830602	0.02675067	547.229405

1909.69007	-0.3239771	0.05287084	-6.127709	8.92E-10	1.09E-08	2064.84192
1685.07567	-0.6686652	0.05389873	-12.405954	2.43E-35	1.86E-33	2104.65725
90.4111173	-0.5449319	0.20652703	-2.6385502	0.00832614	0.02679764	114.816322
242.485196	-0.3443851	0.13052913	-2.6383776	0.00833038	0.02680683	244.447653
908.184448	0.31003875	0.08350157	3.71296926	0.00020484	0.00101453	709.268569
94.2181122	-2.0913669	0.22279018	-9.3871592	6.16E-21	2.23E-19	146.298217
1380.56125	-0.3091262	0.06154532	-5.0227404	5.09E-07	4.14E-06	1499.09375
1975.51207	0.81797674	0.05312019	15.3986027	1.67E-53	2.68E-51	1383.35149
2203.90731	-0.2097878	0.04832274	-4.3413885	1.42E-05	8.94E-05	2306.51176
2549.16747	-0.2358124	0.04528055	-5.207808	1.91E-07	1.67E-06	2858.37085
1051.69823	-0.4399968	0.06263925	-7.0242982	2.15E-12	3.64E-11	1225.01608
1324.85293	0.22271159	0.05821808	3.82547154	0.00013052	0.00067471	1194.46012
2023.81817	0.28029009	0.04845878	5.78409263	7.29E-09	7.83E-08	1838.91303
263.710768	-0.4902462	0.12052907	-4.0674519	4.75E-05	0.00026737	315.744885
137.697875	-0.6039151	0.16716394	-3.6127115	0.00030301	0.00144606	179.631987
1823.98487	0.2053248	0.05133439	3.99975119	6.34E-05	0.00034851	1679.65168
4182.94051	0.13946027	0.03953138	3.52783745	0.00041897	0.00193111	3836.16146
393.84455	0.52641701	0.10323461	5.09922997	3.41E-07	2.85E-06	299.078
1969.19849	-0.2533585	0.04960656	-5.1073581	3.27E-07	2.74E-06	2197.25106
476.839625	-1.3976338	0.09600633	-14.557726	5.22E-48	6.28E-46	662.045727
3189.81937	0.48885868	0.04283461	11.4127043	3.61E-30	2.28E-28	2608.36757
2754.51038	0.25699784	0.04470612	5.74860575	9.00E-09	9.52E-08	2612.99726
880.842259	0.30348072	0.06751668	4.49490019	6.96E-06	4.68E-05	786.121429
2288.08186	0.29572125	0.04986193	5.93080194	3.01E-09	3.38E-08	1977.80374
47.3772511	-1.1342792	0.29318236	-3.8688521	0.00010935	0.00057259	58.334099
804.8754	-1.4282742	0.07501928	-19.038762	8.14E-81	2.77E-78	1144.45947
24.5078896	-2.2580816	0.44588756	-5.06424	4.10E-07	3.38E-06	37.9634612
2115.45236	0.43982264	0.04816515	9.13155401	6.75E-20	2.25E-18	1834.28334
2255.68457	0.23372793	0.05559565	4.20406855	2.62E-05	0.00015663	1932.43277
203.377416	-0.4623372	0.14019916	-3.2977172	0.00097474	0.00409345	224.077015
1903.95993	-0.243024	0.05360398	-4.533693	5.80E-06	3.94E-05	2025.95252
2157.42761	0.25018325	0.04765894	5.24945036	1.53E-07	1.35E-06	1983.35937
48046.0236	0.28999604	0.03739114	7.75574255	8.78E-15	1.91E-13	42584.8182
3161.34662	-0.2076309	0.04138569	-5.0169727	5.25E-07	4.26E-06	3395.41494
9919.34381	0.1904184	0.03414666	5.57648625	2.45E-08	2.45E-07	9422.34589
6792.21688	0.11762007	0.03159894	3.7222786	0.00019743	0.0009821	6449.15872
525.290999	0.2605455	0.08644888	3.01386781	0.0025794	0.00968087	478.709987
877.169556	0.17887338	0.06905276	2.59038728	0.0095868	0.03035612	845.381466
4990.25473	0.26742768	0.03932534	6.80039148	1.04E-11	1.63E-10	4405.61338
707.10452	-0.2680667	0.07633363	-3.5117779	0.00044512	0.00203794	805.566129
2156.10857	-0.8539028	0.05043114	-16.932054	2.61E-64	6.03E-62	2738.92484
3832.19156	0.12401822	0.03779757	3.28111597	0.00103397	0.00431508	3631.52915
1016.26461	0.43164331	0.06419557	6.72387978	1.77E-11	2.70E-10	857.418661

250.971402	-0.816533	0.12827926	-6.3652769	1.95E-10	2.60E-09	312.967071
1087.6512	0.23905173	0.06348105	3.76571784	0.00016607	0.00083929	1032.42096
665.144801	-0.3118676	0.07923883	-3.935793	8.29E-05	0.00044627	728.713268
4181.59077	0.17407203	0.03745013	4.64810265	3.35E-06	2.38E-05	3818.56864
4715.50699	0.24514491	0.03750776	6.5358448	6.33E-11	8.96E-10	4258.38923
4693.45659	-0.141238	0.03529543	-4.0015957	6.29E-05	0.000346	4916.7312
734.452714	0.26233238	0.07758356	3.38128808	0.00072147	0.00313369	666.675417
83.1583643	-0.750622	0.22297495	-3.3663959	0.00076157	0.0032858	92.5938079
1332.62253	0.28132875	0.05777722	4.86919822	1.12E-06	8.66E-06	1170.38573
1432.16705	-0.251719	0.05968805	-4.2172428	2.47E-05	0.00014856	1536.13127
1204.76644	0.29600819	0.06260206	4.72840953	2.26E-06	1.66E-05	1044.45815
1306.68412	-1.5273672	0.06224508	-24.537958	5.82E-133	4.70E-130	1895.39525
16.6109563	-1.9630956	0.55952309	-3.5085157	0.00045062	0.00205872	31.4818947
12730.0353	0.21486488	0.02781743	7.72410941	1.13E-14	2.41E-13	11809.4143
561.828649	0.38345087	0.08718525	4.39811618	1.09E-05	7.05E-05	521.303139
818.366693	-0.4231515	0.07348226	-5.7585534	8.48E-09	9.02E-08	920.382451
423.222486	0.27025627	0.09730182	2.77750485	0.0054778	0.01866669	376.856798
1464.52338	0.77012571	0.05643988	13.6450636	2.16E-42	2.18E-40	1089.82912
294.135983	-1.0925987	0.11854995	-9.2163574	3.07E-20	1.04E-18	400.00525
3432.55545	0.15902272	0.04387345	3.62457709	0.00028944	0.00138604	3080.59599
528.855368	0.823365	0.08822521	9.33253614	1.03E-20	3.64E-19	380.560551
169.1517	0.63068931	0.15151616	4.16252177	3.15E-05	0.00018478	128.705393
22779.1221	0.43345014	0.02551827	16.9858773	1.04E-64	2.44E-62	19109.5101
10853.5571	0.17570937	0.03241182	5.42115129	5.92E-08	5.63E-07	10023.2797
2819.66672	0.17738196	0.04146128	4.27825553	1.88E-05	0.00011621	2647.25697
72.9700201	-0.6134139	0.22908165	-2.6777085	0.00741277	0.0242444	100.001313
581.660105	-0.4189176	0.08566343	-4.8902741	1.01E-06	7.83E-06	690.749807
4568.49495	0.2577603	0.03808723	6.76763016	1.31E-11	2.03E-10	3992.645
2779.8815	0.27001573	0.04487914	6.01650862	1.78E-09	2.06E-08	2517.62564
4854.69151	-0.0980133	0.03583739	-2.7349458	0.00623906	0.02093182	5154.69729
5634.53055	-0.205821	0.03460916	-5.9470093	2.73E-09	3.08E-08	5958.41154
3523.41713	0.24044376	0.04103513	5.85946189	4.64E-09	5.10E-08	3149.11541
637.877343	0.32888231	0.08064015	4.0783943	4.53E-05	0.00025674	588.896618
2359.73691	0.3341477	0.05958342	5.60806509	2.05E-08	2.07E-07	2063.91598
16092.8343	-0.7092423	0.03052459	-23.235114	2.01E-119	1.30E-116	19414.1437
1809.08925	-1.4509213	0.05678204	-25.55247	5.15E-144	5.00E-141	2622.25664
6091.47765	-0.5596362	0.03667364	-15.259901	1.41E-52	2.13E-50	7139.90853
1125.84074	-0.4738667	0.0660406	-7.1753838	7.21E-13	1.29E-11	1264.83142
984.020345	-0.5460624	0.07342141	-7.437373	1.03E-13	2.01E-12	1146.31134
57.3110004	-2.9921019	0.33130953	-9.0311375	1.70E-19	5.48E-18	115.74226
1502.623	0.25406329	0.05394813	4.70939944	2.48E-06	1.81E-05	1375.01805
5497.85368	0.25267887	0.03407736	7.41486132	1.22E-13	2.36E-12	4938.95371
5872.05222	0.48721308	0.03547399	13.7343746	6.32E-43	6.69E-41	4791.72956

4528.34922	0.13602329	0.04486999	3.03149835	0.00243343	0.00920431	4135.23946
1238.51381	-0.4507289	0.06079651	-7.4137302	1.23E-13	2.37E-12	1469.46373
2264.29042	0.40990051	0.05487324	7.46995322	8.02E-14	1.58E-12	1959.28498
514.66754	0.3749815	0.09060968	4.1384264	3.50E-05	0.00020339	461.117163
2103.16824	0.35173833	0.04866704	7.22744465	4.92E-13	8.98E-12	1881.50618
6539.67771	0.25022208	0.04057607	6.16673999	6.97E-10	8.61E-09	6099.15413
2708.6123	0.12383822	0.04201164	2.94771219	0.00320135	0.01171793	2610.21945
99.683605	-0.5359387	0.20119277	-2.6638068	0.00772619	0.02513379	123.149765
190.834175	-0.6670173	0.15053042	-4.4311131	9.37E-06	6.13E-05	237.966086
210.197809	-0.8879663	0.1410464	-6.2955617	3.06E-10	3.96E-09	274.077671
85.614779	-0.6832811	0.21364135	-3.1982626	0.00138258	0.00558362	106.482879
91605.8227	-0.2342491	0.02913419	-8.0403533	8.96E-16	2.12E-14	99268.8955
26359.4319	0.14093595	0.02813826	5.00869406	5.48E-07	4.43E-06	24419.765
13091.0143	0.1003532	0.02909978	3.44859017	0.00056352	0.00251876	12886.2803
687.853392	0.19119314	0.07829425	2.44198194	0.01460688	0.0435555	597.230061
674.938436	-0.3970893	0.0890825	-4.4575456	8.29E-06	5.49E-05	849.085219
8032.03315	0.20178825	0.03246897	6.21480302	5.14E-10	6.47E-09	7412.13432
567.63825	0.4619361	0.08285387	5.57531107	2.47E-08	2.46E-07	462.96904
4975.05946	0.122265	0.03575066	3.41993722	0.00062636	0.00276896	4763.02548
2103.0081	0.352665	0.05188096	6.79758013	1.06E-11	1.66E-10	1758.35641
589.301127	-0.6242901	0.08151233	-7.6588419	1.88E-14	3.94E-13	728.713268
4623.52998	-0.431695	0.03708026	-11.642179	2.52E-31	1.64E-29	5410.2562
5356.9178	0.11576143	0.03703113	3.12605732	0.00177167	0.00696065	5049.14035
261.065138	-0.7136317	0.12592469	-5.6671307	1.45E-08	1.49E-07	333.337709
3839.60961	0.5910804	0.03972406	14.8796573	4.47E-50	6.01E-48	3022.26189
214.382331	0.8508788	0.1358952	6.26128674	3.82E-10	4.89E-09	140.742588
189.008012	1.09693352	0.15412363	7.11723139	1.10E-12	1.93E-11	115.74226
446.754097	0.77666368	0.10173505	7.63417991	2.27E-14	4.73E-13	351.85647
6089.5217	0.10212053	0.03691015	2.76673267	0.00566212	0.01919555	5732.48265
3932.96879	-0.5896238	0.03790369	-15.555843	1.45E-54	2.54E-52	4772.28486
3512.12736	0.14829435	0.03932924	3.77058817	0.00016286	0.00082452	3332.45115
335.988921	-0.6835267	0.11921447	-5.7335884	9.83E-09	1.03E-07	411.116507
3525.86555	-0.3320178	0.04756434	-6.9803937	2.94E-12	4.89E-11	4208.38857
20558.48	0.19543626	0.02873109	6.8022568	1.03E-11	1.62E-10	18668.7636
103642.031	-0.2628526	0.02515572	-10.44902	1.48E-25	7.10E-24	110576.451
58.8829395	0.69608025	0.25354076	2.74543731	0.00604303	0.02034813	53.7044086
6173.40129	0.40024286	0.03517075	11.3799928	5.26E-30	3.30E-28	5208.4017
4395.00738	0.2772652	0.03700335	7.49297617	6.73E-14	1.34E-12	3886.16212
1248.59827	0.26238539	0.05875861	4.46547983	7.99E-06	5.30E-05	1127.79258
1343.39962	0.30996539	0.05750104	5.39060458	7.02E-08	6.57E-07	1175.94136
2036.02317	-0.3207251	0.05234822	-6.1267636	8.97E-10	1.09E-08	2232.43671
41.4196381	-0.8764564	0.33227441	-2.6377487	0.00834584	0.02683877	42.5931516
3172.76858	0.53769857	0.04516142	11.906149	1.10E-32	7.67E-31	2631.51602

470.693532	0.44280944	0.09941831	4.45400266	8.43E-06	5.57E-05	360.189913
2293.28779	0.31014605	0.05296295	5.85590612	4.74E-09	5.20E-08	1996.3225
1217.75967	-0.4916677	0.06109685	-8.0473494	8.46E-16	2.01E-14	1362.98085
5401.50369	0.12905719	0.03768736	3.42441541	0.00061612	0.00272684	5102.84475
3106.91207	0.51443201	0.04581432	11.228628	2.95E-29	1.80E-27	2459.29154
398.404324	0.3544319	0.10182689	3.48072982	0.00050005	0.00226046	328.708018
671.425031	-0.1821978	0.0758564	-2.4018782	0.01631114	0.04792958	713.898259
2009.11924	-0.1438112	0.04935391	-2.9138759	0.00356972	0.01287902	2098.17569
1199.2421	-0.3069323	0.06372999	-4.8161364	1.46E-06	1.11E-05	1248.16453
12461.305	0.25929319	0.0341208	7.59927011	2.98E-14	6.15E-13	11404.7793
2127.41017	0.24389131	0.04894012	4.98346378	6.25E-07	5.01E-06	1906.50651
1231.26508	0.36366537	0.05854401	6.21182872	5.24E-10	6.57E-09	1102.79225
2139.33781	0.68016675	0.04918313	13.8292692	1.70E-43	1.86E-41	1597.24319
241.082961	-0.8906415	0.1264519	-7.0433222	1.88E-12	3.21E-11	316.670823
2617.04525	0.21641443	0.05060784	4.27630274	1.90E-05	0.00011716	2350.95678
3668.41387	0.27746561	0.04171524	6.65142002	2.90E-11	4.28E-10	3341.71053
2515.43097	0.24012419	0.04556625	5.2697818	1.37E-07	1.22E-06	2227.80702
1560.0944	0.26588534	0.05434212	4.89280422	9.94E-07	7.73E-06	1440.75965
4505.83751	0.26910348	0.03744999	7.18567476	6.69E-13	1.20E-11	4158.38791
43971.5915	0.17296566	0.02362134	7.32243138	2.44E-13	4.58E-12	41589.4348
109.363212	1.25232694	0.1964898	6.37349601	1.85E-10	2.48E-09	56.4822228
722.693278	0.38614251	0.07381771	5.23102798	1.69E-07	1.49E-06	631.48977
13307.732	0.13766716	0.02701849	5.09529383	3.48E-07	2.91E-06	12646.4623
5143.80291	-0.2425492	0.03650842	-6.6436529	3.06E-11	4.50E-10	5586.18443
118.724323	-1.3798105	0.18993461	-7.2646605	3.74E-13	6.92E-12	151.853845
3961.41622	0.23872587	0.04038527	5.91121118	3.40E-09	3.78E-08	3547.26878
1744.8613	0.24372885	0.05629845	4.32922823	1.50E-05	9.40E-05	1491.68625
3538.75138	0.16135692	0.04116088	3.92015236	8.85E-05	0.00047296	3230.59796
422.96269	0.33138531	0.09531698	3.47666594	0.00050769	0.0022898	377.782736
380.16042	0.37971898	0.10022891	3.78851756	0.00015155	0.00077269	343.523027
1374.7462	-0.2504932	0.0579478	-4.3227394	1.54E-05	9.66E-05	1542.61284
5758.87328	0.49197479	0.03401683	14.4626888	2.08E-47	2.46E-45	4694.50606
180.259819	-0.3743233	0.14873816	-2.5166592	0.01184734	0.03637899	187.96543
408.147743	-0.6372865	0.10076512	-6.3244751	2.54E-10	3.33E-09	501.858439
948.507733	0.2166064	0.06657202	3.25371541	0.00113906	0.00470404	896.308061
60.1114978	-0.7515667	0.24855049	-3.0237989	0.00249622	0.00940949	76.8528606
1150.10596	0.31839506	0.0624093	5.10172491	3.37E-07	2.82E-06	1000.01313
1022.17541	0.47748614	0.06634896	7.19658829	6.17E-13	1.11E-11	849.085219
476.228864	0.44405184	0.09039345	4.91243393	9.00E-07	7.04E-06	388.893993
1633.66992	0.27655463	0.06185823	4.47078164	7.79E-06	5.18E-05	1460.20435
13508.075	0.18719469	0.03330676	5.62032137	1.91E-08	1.93E-07	12310.3468
6538.9615	0.18563454	0.03309654	5.60888089	2.04E-08	2.06E-07	6001.93063
258.42084	0.58230162	0.11967216	4.86580691	1.14E-06	8.80E-06	202.780439

4872.02647	0.09779625	0.03604311	2.71331336	0.00666141	0.02213808	4774.13674
2456.63129	0.2065495	0.04653708	4.43838512	9.06E-06	5.94E-05	2317.62301
2012.33884	0.21760911	0.0509409	4.27179529	1.94E-05	0.00011933	1880.58024
5725.22587	0.25601722	0.03682979	6.95136324	3.62E-12	5.94E-11	5208.4017
1773.92398	0.38711562	0.05349841	7.23602153	4.62E-13	8.47E-12	1448.16716
58.5810214	-2.2504815	0.2920019	-7.7070782	1.29E-14	2.73E-13	103.705065
32.5452458	-0.9656333	0.34476929	-2.8008101	0.00509745	0.01757007	50.9265944
118060.518	0.17023569	0.02381643	7.14782469	8.82E-13	1.56E-11	110071.815
17890.4689	0.18814032	0.02595822	7.24781362	4.24E-13	7.81E-12	16561.3285
21545.0712	0.1747472	0.03656601	4.778952	1.76E-06	1.32E-05	19185.437
73734.3244	0.19961141	0.02383064	8.37625159	5.46E-17	1.44E-15	67256.4383
2439.2258	0.30507779	0.04501807	6.77678504	1.23E-11	1.91E-10	2149.10228
4590.64746	0.29693885	0.03710177	8.00335993	1.21E-15	2.84E-14	4013.94157
7546.54056	-0.2018282	0.03173273	-6.3602525	2.01E-10	2.68E-09	8056.58723
217.792878	-0.5634398	0.13967973	-4.0337984	5.49E-05	0.00030545	284.26299
67.9565767	-0.6883458	0.23525868	-2.9259102	0.0034345	0.01245609	83.3344271
92.1830524	-1.1036704	0.21941494	-5.0300607	4.90E-07	4.00E-06	123.149765
867.991737	-0.5453305	0.0738368	-7.38562	1.52E-13	2.91E-12	988.901868
934.360428	-0.7950065	0.06847575	-11.610044	3.66E-31	2.38E-29	1235.2014
378.276769	-0.6299131	0.10143307	-6.2101357	5.29E-10	6.63E-09	468.524668
248.018968	-0.9459053	0.13126158	-7.2062622	5.75E-13	1.04E-11	302.781752
35.2929751	-1.1547923	0.34428767	-3.3541495	0.00079609	0.003418	42.5931516
17.4634678	-1.3219425	0.5038629	-2.6236155	0.00870019	0.02784895	21.2965758
1415.17111	0.26137564	0.05627612	4.64452171	3.41E-06	2.42E-05	1315.75801
643.786843	-1.0426116	0.08078295	-12.906333	4.15E-38	3.57E-36	897.233999
2770.31285	0.22635162	0.0437551	5.17314875	2.30E-07	1.98E-06	2510.21813
2825.25953	0.18586745	0.04149207	4.4795892	7.48E-06	4.99E-05	2685.22043
6038.27503	0.12521481	0.03320625	3.77082125	0.00016271	0.00082397	5696.37106
1355.02324	0.19319717	0.05656387	3.41555778	0.00063652	0.00280684	1300.943
2281.0402	0.22966775	0.04701768	4.8847108	1.04E-06	8.03E-06	2105.58319
7096.2917	0.31130843	0.03296212	9.44443124	3.57E-21	1.32E-19	6142.67322
340.591465	-0.5872607	0.10685508	-5.4958613	3.89E-08	3.77E-07	405.560879
2740.02239	0.56951686	0.04323604	13.1722706	1.27E-39	1.17E-37	2225.0292
5107.7917	0.23588919	0.03524233	6.69334798	2.18E-11	3.29E-10	4664.87604
3260.86193	-0.4202404	0.04336975	-9.6897123	3.33E-22	1.30E-20	3636.15884
770.079941	-0.4094796	0.07633258	-5.3644142	8.12E-08	7.53E-07	862.048352
3928.7928	0.88899049	0.03971315	22.3852924	5.47E-111	3.03E-108	2662.99792
25816.6363	-0.2160694	0.0280215	-7.7108437	1.25E-14	2.67E-13	27265.1727
3004.33948	0.12972532	0.04501513	2.88181586	0.00395391	0.01408943	2725.03577
1147.38132	0.16051724	0.06016403	2.66799353	0.00763057	0.02486446	1087.97724
336.671926	1.44289894	0.11154247	12.9358709	2.82E-38	2.47E-36	181.483864
1197.97261	0.18026109	0.05958356	3.0253494	0.00248346	0.00936977	1100.01444
135.585804	-0.5188923	0.16953087	-3.060754	0.0022078	0.00844981	173.150421

248.915023	-2.6065019	0.1566223	-16.641959	3.46E-62	7.71E-60	433.339021
6387.19362	0.09054014	0.03341887	2.7092516	0.00674352	0.02236494	6350.08335
991.835685	-0.5172533	0.06469123	-7.9957258	1.29E-15	3.01E-14	1147.23728
595.068946	0.64940989	0.08388731	7.74145563	9.83E-15	2.12E-13	479.635925
12367.9535	0.16570663	0.02991655	5.53896208	3.04E-08	2.99E-07	11973.3053
1270.01505	0.25758944	0.06047871	4.25917581	2.05E-05	0.00012527	1137.9779
1602.68357	0.32390696	0.0532341	6.08457716	1.17E-09	1.40E-08	1384.27743
1691.16205	0.3089683	0.05477236	5.64095285	1.69E-08	1.73E-07	1468.53779
7851.64099	0.26085228	0.03499966	7.45299467	9.12E-14	1.79E-12	7155.64948
606.643641	0.26184941	0.08888617	2.94589598	0.00322021	0.01177806	561.118476
15802.407	0.14773933	0.0257586	5.73553377	9.72E-09	1.02E-07	15051.1235
732.901019	-0.3551016	0.07688547	-4.6185785	3.86E-06	2.70E-05	787.973305
11730.1455	0.28774034	0.02924897	9.8376216	7.75E-23	3.13E-21	10490.8784
504.352296	-0.3021963	0.09715877	-3.1103352	0.00186875	0.00729622	621.304451
125.623773	-0.5336575	0.18264488	-2.9218312	0.0034798	0.01259211	165.742916
576.789095	0.36179864	0.09312952	3.88489734	0.00010237	0.00053971	471.302482
3155.95006	-0.2676056	0.04147152	-6.4527562	1.10E-10	1.52E-09	3549.12066
3884.21447	0.14622085	0.04219479	3.46537729	0.00052949	0.00237706	3556.52816
13149.9365	0.15967952	0.03564898	4.47921667	7.49E-06	5.00E-05	11959.4162
23.9856025	1.10118953	0.39817083	2.76562079	0.00568146	0.01925774	13.8890712
141.951736	-0.4057292	0.16296046	-2.4897404	0.01278365	0.03888451	164.816978
1800.19599	0.22193596	0.05279116	4.20403629	2.62E-05	0.00015663	1653.72541
338.324476	-0.3989308	0.10609518	-3.7601223	0.00016983	0.000856	381.486489
1062.35878	-0.4123846	0.06761641	-6.0988833	1.07E-09	1.29E-08	1251.86828
222.710572	-0.7142811	0.1419021	-5.0336189	4.81E-07	3.93E-06	301.855814
5604.7129	-0.2063098	0.03799785	-5.4295114	5.65E-08	5.39E-07	6087.11693
3452.68306	0.12173082	0.04140166	2.94023998	0.00327958	0.01197128	3436.15621
5069.96165	0.32576354	0.04255087	7.65586152	1.92E-14	4.02E-13	4500.05906
317.849955	-0.4876714	0.11997181	-4.0648832	4.81E-05	0.00026987	334.263647
384.40771	-0.4124403	0.12002095	-3.4364022	0.0005895	0.00262577	359.263975
278.41807	-0.823485	0.12080398	-6.8167041	9.32E-12	1.47E-10	381.486489
9439.52285	0.28272415	0.03441817	8.21438685	2.13E-16	5.31E-15	8502.88938
2458.0922	-0.1336435	0.04375454	-3.0543912	0.00225518	0.00860225	2548.18159
1060.85487	-0.2032683	0.06263242	-3.2454171	0.00117279	0.00482788	1126.86664
1231.95141	0.38032113	0.06098584	6.23622053	4.48E-10	5.69E-09	1084.27349
28.3443047	-0.8851947	0.36759111	-2.4080961	0.01603596	0.04726427	39.8153374
1130.61376	-0.8308704	0.06743934	-12.320262	7.05E-35	5.32E-33	1512.98282
12110.6351	-0.1168905	0.03476741	-3.3620716	0.0007736	0.0033325	12815.909
8844.15446	0.25670858	0.04331119	5.92707222	3.08E-09	3.45E-08	7645.47072
14202.3101	-0.1102624	0.02771137	-3.9789585	6.92E-05	0.00037765	14497.4125
2256.73521	0.33151493	0.04693392	7.06344032	1.62E-12	2.79E-11	1962.06279
18399.3244	0.08931393	0.0310256	2.87871736	0.00399296	0.0142077	18197.4611
12044.181	-0.1852267	0.02975716	-6.2246098	4.83E-10	6.10E-09	12901.0953

10831.6922	0.2183566	0.03114405	7.01118193	2.36E-12	3.98E-11	9800.12863
5555.95516	0.16196914	0.03725183	4.34795179	1.37E-05	8.69E-05	5031.54752
1293.65145	-0.167149	0.05725863	-2.9191927	0.00350939	0.01268735	1339.8324
1423.84673	0.13403222	0.05471966	2.44943426	0.01430808	0.042783	1374.09211
3282.06513	0.11808917	0.04120692	2.86576063	0.00416009	0.01471861	3099.11475
2097.34295	0.11777729	0.04915715	2.39593405	0.01657807	0.04859609	2068.54567
8870.70738	0.21059357	0.03697537	5.69550977	1.23E-08	1.28E-07	8376.03586
1703.64522	-0.151245	0.054267	-2.7870522	0.00531899	0.01821386	1762.06016
496.654182	0.84230465	0.10049171	8.38183256	5.21E-17	1.37E-15	316.670823
2078.64136	0.35777697	0.05800633	6.16789577	6.92E-10	8.56E-09	1697.2445
581.151401	-0.3972592	0.08466547	-4.6921038	2.70E-06	1.95E-05	613.896946
1163.72057	-0.2680119	0.06887051	-3.891534	9.96E-05	0.00052702	1233.34952
7858.35649	0.18245644	0.03281123	5.56079323	2.69E-08	2.66E-07	7171.39042
1103.33657	0.19665785	0.06578761	2.98928386	0.00279632	0.01040436	1009.27251
1602.11377	-0.5236496	0.05467842	-9.5768969	1.00E-21	3.82E-20	1819.46833
25806.1268	0.22776875	0.0281514	8.09085107	5.92E-16	1.43E-14	23889.2024
38124.5373	0.27228524	0.02467141	11.0364683	2.55E-28	1.46E-26	34163.4114
3162.48628	-0.2505873	0.04172603	-6.0055392	1.91E-09	2.20E-08	3443.56372
7097.38618	0.20395731	0.03446041	5.91859846	3.25E-09	3.63E-08	6581.56787
795.189916	-0.5495489	0.07499926	-7.3273908	2.35E-13	4.43E-12	959.27185
2665.86356	0.18226069	0.04656905	3.91377316	9.09E-05	0.00048457	2407.43901
496.879151	-0.3300149	0.0894768	-3.6882737	0.00022578	0.00111057	563.89629
5312.48002	-0.1722045	0.03692567	-4.6635444	3.11E-06	2.22E-05	5513.03532
1516.61649	-0.1597301	0.06322773	-2.5262666	0.0115282	0.0355624	1533.35346
39.4589846	-1.3221109	0.32421879	-4.0778354	4.55E-05	0.00025713	49.0747182
47.731938	-1.5060903	0.34400755	-4.3780734	1.20E-05	7.67E-05	73.1491083
5615.3464	-0.1093251	0.03492202	-3.1305478	0.00174481	0.00686484	5696.37106
5315.50075	0.49783608	0.0361221	13.7820368	3.27E-43	3.52E-41	4527.83721
2511.17203	0.36210646	0.04839068	7.48297952	7.27E-14	1.44E-12	2145.39853
192.262507	0.88656553	0.14009559	6.32829026	2.48E-10	3.25E-09	138.890712
507.019047	0.45805423	0.08831607	5.18653299	2.14E-07	1.85E-06	419.44995
94.9249273	0.57915576	0.20454666	2.83141148	0.00463431	0.01618375	70.371294
45.9460493	0.74362756	0.28352075	2.62283291	0.0087202	0.02788997	29.6300185
916.997392	0.50249071	0.06653448	7.55233579	4.28E-14	8.72E-13	775.010172
271.538135	0.52323531	0.12237676	4.27561015	1.91E-05	0.00011742	233.336396
217.960379	1.0535889	0.13543743	7.77915603	7.30E-15	1.59E-13	136.112898
21.0670893	1.18366312	0.42680541	2.77330859	0.00554895	0.01885475	14.8150093
784.824731	0.42954573	0.07702029	5.57704623	2.45E-08	2.44E-07	634.267584
362.529192	0.70433304	0.10267309	6.85995726	6.89E-12	1.10E-10	285.188928
1049.86648	0.6210185	0.07151917	8.68324547	3.85E-18	1.12E-16	806.492067
4963.18735	0.12357706	0.0380065	3.25147149	0.00114809	0.00473729	4568.57848
1042.67505	0.39479121	0.0621614	6.35106696	2.14E-10	2.83E-09	902.789627
1412.16422	0.22403956	0.0612935	3.65519266	0.00025699	0.00124512	1317.60989

766.927084	0.53464579	0.07323307	7.30060583	2.86E-13	5.37E-12	597.230061
2586.0725	-0.6752865	0.04489278	-15.042206	3.88E-51	5.34E-49	3277.8208
3960.5869	-0.2898219	0.04183426	-6.9278602	4.27E-12	6.95E-11	4150.05447
2157.92554	-0.1250804	0.04616666	-2.7093221	0.00674209	0.02236402	2241.69609
999.540102	0.27839788	0.06506352	4.27886296	1.88E-05	0.00011597	892.604308
1366.19074	0.20410398	0.05589347	3.65166073	0.00026055	0.00126048	1249.09047
356.494814	-0.4509963	0.10666477	-4.2281655	2.36E-05	0.0001421	405.560879
186.550484	-0.4127044	0.14311093	-2.8838079	0.00392898	0.01401423	199.076687
2176.1385	-0.7818417	0.04985122	-15.683501	1.96E-55	3.46E-53	2797.25894
1649.8583	0.34058466	0.05159089	6.60164361	4.07E-11	5.89E-10	1471.31561
10622.9883	0.14721448	0.04337573	3.39393636	0.00068896	0.00300773	10345.5062
5498.54652	0.13883327	0.03406317	4.07575846	4.59E-05	0.00025921	5188.957
34425.5117	0.17202589	0.02453894	7.01032391	2.38E-12	4.00E-11	31953.1972
2761.04206	0.35396099	0.04333571	8.16788185	3.14E-16	7.74E-15	2491.69937
9414.73445	0.1152457	0.03582915	3.21653432	0.00129749	0.00528067	9470.49467
23965.7944	-0.1823628	0.07227907	-2.5230379	0.01163459	0.03583926	26867.0193
33630.0959	0.13584675	0.02703795	5.02429992	5.05E-07	4.11E-06	31609.6741
10961.5103	-0.277171	0.04149777	-6.6791786	2.40E-11	3.60E-10	12596.4616
9193.7593	-0.250064	0.04067516	-6.1478308	7.85E-10	9.63E-09	10179.7632
21964.529	0.16376655	0.02571385	6.36880665	1.91E-10	2.55E-09	20379.8971
27799.81	-0.2940442	0.02722112	-10.80206	3.37E-27	1.79E-25	31330.9668
2746.18992	0.2740697	0.04744737	5.7762888	7.64E-09	8.17E-08	2418.55026
15020.4879	0.17336981	0.03019871	5.74096719	9.41E-09	9.94E-08	14286.2986
19014.4797	0.17435836	0.02721099	6.40764513	1.48E-10	2.01E-09	18145.6085
3458.65903	0.33325535	0.03869787	8.61172418	7.20E-18	2.06E-16	3088.00349
2245.29507	-0.1906363	0.04593857	-4.1498097	3.33E-05	0.00019441	2349.10491
369.55615	0.46415251	0.10135837	4.5793211	4.66E-06	3.22E-05	298.152062
528.902039	0.36300934	0.08691855	4.176431	2.96E-05	0.00017485	453.709659
650.569594	0.34243721	0.07683174	4.45697591	8.31E-06	5.50E-05	568.525981
1122.69412	0.65065002	0.06339344	10.2636799	1.03E-24	4.63E-23	843.52959
215.81005	0.43487701	0.13270726	3.27696473	0.0010493	0.00437338	199.076687
119.251496	0.46773547	0.17478223	2.67610428	0.00744835	0.02434843	101.853189
4398.7498	-0.1210198	0.03998082	-3.0269457	0.00247038	0.00932951	4558.39316
1599.62201	0.21024398	0.05871424	3.58080045	0.00034254	0.00161327	1402.79619
206.814215	0.4199797	0.13607066	3.08648253	0.0020254	0.00783367	194.446997
2334.62309	0.37755393	0.04898624	7.70734704	1.28E-14	2.73E-13	2010.21157
397.524063	-0.4105912	0.10131584	-4.0525863	5.07E-05	0.00028339	492.599058
233.777551	-0.5271496	0.13429171	-3.925407	8.66E-05	0.00046404	255.55891
205.700882	0.71665787	0.14616144	4.90319387	9.43E-07	7.35E-06	141.668526
3595.29429	0.15711257	0.04172542	3.76539232	0.00016629	0.00084011	3422.26714
1024.37634	-0.4125244	0.06438591	-6.407059	1.48E-10	2.02E-09	1181.49699
372.104996	-0.3486125	0.10234236	-3.4063362	0.00065841	0.00289351	394.449622
175058.597	0.1548281	0.02480896	6.24081328	4.35E-10	5.53E-09	163803.076

118065.252	0.2504075	0.02289365	10.9378584	7.60E-28	4.28E-26	106206.95
27835.1822	0.1133481	0.02573101	4.40511651	1.06E-05	6.85E-05	26577.2007
80.6507303	-1.1952564	0.22667707	-5.2729481	1.34E-07	1.20E-06	102.779127
92280.9002	0.1810546	0.02793311	6.48172035	9.07E-11	1.27E-09	83603.8751
78804.6959	0.21259667	0.03370977	6.30667787	2.85E-10	3.70E-09	72456.5066
965.302304	-1.5357741	0.07122399	-21.562595	4.03E-103	1.86E-100	1370.38836
43651.5483	0.36586805	0.02474297	14.7867485	1.78E-49	2.34E-47	37557.9004
252.292344	-0.4746935	0.13484166	-3.5203772	0.00043093	0.00197936	304.633628
17979.1262	0.37899254	0.02761255	13.7253752	7.16E-43	7.54E-41	15502.9813
8890.39067	0.20034676	0.03952586	5.06875188	4.00E-07	3.31E-06	8334.36865
10182.6363	0.12908034	0.02997474	4.30630376	1.66E-05	0.00010354	9534.3844
12723.2919	0.18564014	0.03075841	6.03542777	1.59E-09	1.86E-08	11753.858
2825.3416	0.12911172	0.04401119	2.93361109	0.00335044	0.01219456	2637.99759
3981.45926	0.96003535	0.03994396	24.0345595	1.21E-127	9.03E-125	2659.29416
3975.98109	0.21194404	0.03931733	5.39060083	7.02E-08	6.57E-07	3638.93665
4331.16747	0.51493503	0.03818007	13.4870096	1.87E-41	1.84E-39	3554.67629
10231.0391	0.15570502	0.02916263	5.33919595	9.34E-08	8.59E-07	9492.71719
240.547759	-0.6340266	0.12975986	-4.8861533	1.03E-06	7.98E-06	320.374575
1073.92113	-0.7341105	0.06965411	-10.539371	5.69E-26	2.79E-24	1291.68362
4640.03283	0.14618178	0.04531655	3.2257925	0.00125624	0.00513545	4248.20391
37742.4228	-0.3004795	0.0772134	-3.8915466	9.96E-05	0.00052702	38830.1393
7671.46031	-0.153235	0.03156008	-4.8553417	1.20E-06	9.24E-06	8071.40224
120.274689	-1.8729097	0.19491547	-9.6088304	7.34E-22	2.82E-20	191.669182
47.3427485	-0.8309268	0.29959457	-2.7735044	0.00554561	0.0188527	61.1119132
7297.0306	-0.5349036	0.03674363	-14.557724	5.22E-48	6.28E-46	8806.59707
2959.79344	-0.1037775	0.04089677	-2.5375476	0.01116322	0.03455197	3039.85471
798.441373	-0.2284922	0.07065527	-3.2339013	0.00122112	0.00500875	864.826166
2181.89534	0.25355106	0.04966096	5.10564111	3.30E-07	2.76E-06	1916.69182
237.182918	-0.6034524	0.14034466	-4.2997885	1.71E-05	0.00010629	274.077671
36482.7385	0.07980366	0.0299269	2.66662002	0.00766182	0.0249621	36089.3626
7269.97821	0.28351745	0.03722869	7.61556418	2.63E-14	5.44E-13	6624.16102
3513.072	0.25168367	0.04519875	5.56837673	2.57E-08	2.56E-07	3108.37413
1054.71877	1.59549617	0.07129924	22.3774648	6.53E-111	3.51E-108	562.044414
15473.6845	0.11839084	0.02873054	4.12073076	3.78E-05	0.00021789	14865.0099
45506.2713	0.3981733	0.02490386	15.9884187	1.54E-57	3.04E-55	39253.293
54.3315428	-3.8508342	0.39895823	-9.652224	4.81E-22	1.86E-20	106.482879
27.122515	-1.2190676	0.38797595	-3.1421214	0.00167729	0.00663284	39.8153374
11.6504051	-1.7217714	0.61818803	-2.7851905	0.00534963	0.01830261	21.2965758
9227.06508	0.28873783	0.03693278	7.81792872	5.37E-15	1.19E-13	8110.29163
9789.6558	0.34382275	0.03167358	10.8551921	1.88E-27	1.03E-25	8645.48384
1098.89217	0.25683601	0.0638397	4.02313956	5.74E-05	0.00031834	966.679355
825.765806	0.25768639	0.07342592	3.50947438	0.00044899	0.00205228	735.194835
329.114404	-0.4763502	0.12184772	-3.9093893	9.25E-05	0.00049264	407.412755

179.335628	0.74149926	0.14780616	5.01670068	5.26E-07	4.26E-06	142.594464
375.588984	0.91330434	0.10290812	8.87494922	7.00E-19	2.16E-17	257.410786
984.482131	0.17741184	0.06686097	2.65344385	0.0079675	0.02581913	886.122742
2499.80907	0.13249742	0.04473007	2.96215512	0.00305494	0.01124382	2331.51208
1685.89168	-0.2842712	0.05507169	-5.1618388	2.45E-07	2.10E-06	1853.72803
1069.48749	-0.3995974	0.06282156	-6.360832	2.01E-10	2.68E-09	1207.42326
14506.3765	0.19453805	0.03182719	6.11232265	9.82E-10	1.19E-08	13104.8016
2703.04148	0.14363033	0.04486317	3.20151991	0.00136705	0.00553009	2502.81063
1880.1547	-0.1884212	0.04936949	-3.8165527	0.00013533	0.00069733	1992.61875
13188.7719	0.45631233	0.0294984	15.4690555	5.61E-54	9.14E-52	10935.3287
3807.10354	-0.1233895	0.04032223	-3.0600852	0.00221274	0.00846369	4075.97942
3006.25656	0.26563249	0.04141403	6.41406984	1.42E-10	1.93E-09	2712.99857
8304.17244	-0.324458	0.03558194	-9.1186152	7.61E-20	2.52E-18	8986.22906
1783.89332	-0.2224619	0.05199293	-4.2786949	1.88E-05	0.00011602	1933.35871
305.556778	-1.4524063	0.12218367	-11.887074	1.38E-32	9.57E-31	426.857454
681.59941	-0.7698602	0.07866506	-9.7865585	1.29E-22	5.16E-21	835.196147
2498.42163	0.2032258	0.04482368	4.53389337	5.79E-06	3.94E-05	2331.51208
303.351832	0.46584055	0.11123847	4.18776468	2.82E-05	0.00016729	242.595777
6160.54426	0.27353669	0.03254565	8.40470761	4.29E-17	1.15E-15	5497.29438
550.769634	0.27535553	0.08475989	3.24865385	0.00115953	0.00477937	487.04343
2290.11313	0.36084904	0.04533839	7.95901765	1.73E-15	4.00E-14	1973.17405
6158.09536	0.18754689	0.03507141	5.34757259	8.91E-08	8.23E-07	5761.18673
3048.21043	0.19017361	0.04168029	4.56267472	5.05E-06	3.47E-05	2878.74149
3810.98599	0.20016874	0.03853855	5.1939874	2.06E-07	1.79E-06	3507.45344
16519.4099	0.26971174	0.02897746	9.30763841	1.31E-20	4.57E-19	14728.897
4503.67193	-0.1657389	0.037744	-4.3911325	1.13E-05	7.26E-05	4850.06366
7243.24621	-0.1786288	0.03307258	-5.4011159	6.62E-08	6.24E-07	7523.24689
18613.1276	0.09424163	0.02793872	3.37315439	0.00074312	0.00321406	18115.9785
1423.94107	-0.307195	0.05906123	-5.2012965	1.98E-07	1.72E-06	1523.16814
1685.4752	0.30019486	0.05437989	5.52032861	3.38E-08	3.30E-07	1514.8347
3858.39836	0.10030303	0.03990693	2.51342386	0.01195656	0.03667952	3649.12197
49.1942254	0.86004625	0.28373909	3.03111658	0.00243651	0.00921057	43.5190897
3038.55672	0.20041682	0.044167	4.53770562	5.69E-06	3.87E-05	2790.77737
3498.84643	0.66131972	0.04157176	15.9079064	5.59E-57	1.07E-54	2722.25795
97.9767567	-0.7606892	0.20017883	-3.8000483	0.00014467	0.00074034	108.334755
46.6612803	-1.5260892	0.29941016	-5.0969853	3.45E-07	2.88E-06	62.0378513
7087.99355	-0.3362483	0.03199667	-10.508853	7.86E-26	3.83E-24	7967.69717
4308.85052	-0.0988789	0.03689889	-2.6797254	0.00736826	0.02411916	4442.6509
6990.84244	0.47637517	0.04037334	11.7992502	3.94E-32	2.63E-30	5532.48002
269.570316	-0.5847413	0.12651626	-4.6218665	3.80E-06	2.67E-05	333.337709
475.536311	0.39947086	0.09267172	4.31060154	1.63E-05	0.00010168	395.37556
460.496863	0.69070432	0.0908457	7.6030493	2.89E-14	5.98E-13	348.152718
4977.51131	0.13879476	0.03569084	3.8888059	0.00010074	0.00053225	4750.06235

7782.18964	0.09742543	0.03341728	2.9154203	0.0035521	0.012825	7482.50562
1258.7319	0.28399563	0.06014502	4.72184818	2.34E-06	1.71E-05	1156.49666
5174.60923	0.51677372	0.03433088	15.0527367	3.31E-51	4.59E-49	4232.46296
975.273902	-0.5642212	0.0670092	-8.4200568	3.76E-17	1.01E-15	1157.4226
11250.4969	0.2475167	0.0316199	7.82787722	4.96E-15	1.10E-13	10032.5391
1409.33223	-0.2548003	0.05514249	-4.6207608	3.82E-06	2.68E-05	1559.27973
89.0497166	-0.4919823	0.20482967	-2.4019095	0.01630974	0.04792958	108.334755
149.193552	-0.7217295	0.16180124	-4.4605934	8.17E-06	5.42E-05	191.669182
558.735144	0.78318586	0.09049421	8.65454135	4.95E-18	1.44E-16	437.042773
120.900036	0.57519038	0.18084994	3.18048428	0.00147029	0.00590217	88.8900556
864.372693	-0.3041207	0.07067683	-4.3029753	1.69E-05	0.00010497	939.82715
7812.45899	0.13403435	0.03072205	4.36280651	1.28E-05	8.16E-05	7384.35618
6642.50838	-0.4425239	0.03531906	-12.529324	5.16E-36	4.00E-34	7601.02569
9674.46808	0.38058986	0.07703345	4.94057904	7.79E-07	6.15E-06	7823.25083
25435.4068	0.2905318	0.03408177	8.52455198	1.53E-17	4.28E-16	22037.3263
532.014844	0.26726058	0.08619133	3.10078283	0.0019301	0.00751455	495.376872
1966.52154	0.1342358	0.04911139	2.7332925	0.00627046	0.02102264	1887.98774
4701.48059	0.25509861	0.03773942	6.75947373	1.38E-11	2.14E-10	4188.94387
152.531708	0.6058235	0.15915741	3.80644242	0.00014098	0.00072356	137.038836
1065.83997	0.23308646	0.06372972	3.65742192	0.00025477	0.00123558	977.790612
278.578651	0.75000544	0.1237147	6.06237935	1.34E-09	1.59E-08	199.076687
463.138265	0.23637785	0.09383293	2.51913519	0.01176435	0.03618722	419.44995
227.6264	-1.3386742	0.14339481	-9.3355833	1.00E-20	3.55E-19	317.596761
429.661054	-0.2785188	0.09973512	-2.792585	0.00522887	0.01796238	473.154358
849.563967	-0.4524129	0.07154189	-6.3237482	2.55E-10	3.34E-09	1037.05065
324.863576	0.47416407	0.11037815	4.2958145	1.74E-05	0.00010814	291.670495
913.736996	-0.2111592	0.06755353	-3.1258053	0.00177319	0.00696389	946.308717
371.365122	-1.6910711	0.10917168	-15.490016	4.05E-54	6.66E-52	575.933485
2328.54513	-0.5332783	0.05513932	-9.6714695	3.99E-22	1.55E-20	2923.18652
2643.24894	0.22610105	0.04261402	5.30578988	1.12E-07	1.02E-06	2401.88338
1942.59223	0.13717304	0.05325648	2.57570581	0.01000357	0.03147529	1773.17142
6822.06271	-0.1017974	0.0382124	-2.6639895	0.007722	0.02512436	6838.97865
965.097478	-0.3103109	0.06671162	-4.6515269	3.29E-06	2.35E-05	1041.68034
941.216105	0.31982313	0.07099409	4.50492623	6.64E-06	4.48E-05	787.973305
7270.64438	0.18373411	0.03221585	5.70322078	1.18E-08	1.23E-07	6712.12514
3005.63983	0.28248762	0.04361121	6.47740805	9.33E-11	1.30E-09	2733.36921
3255.08868	0.14238635	0.03966509	3.58971442	0.00033104	0.00156632	3102.8185
7063.53066	0.18660404	0.0333387	5.59722011	2.18E-08	2.19E-07	6499.15938
3534.10281	0.40211827	0.03977107	10.1108229	4.95E-24	2.14E-22	3053.74379
484.842991	0.23829963	0.09515936	2.50421644	0.01227229	0.03755895	465.746854
2420.04751	0.16438882	0.04658579	3.52873287	0.00041755	0.00192584	2250.95547
1960.66605	-0.1893223	0.05059283	-3.7420774	0.00018251	0.00091512	2083.36068
15695.1026	0.24218636	0.0279377	8.66880039	4.37E-18	1.27E-16	14227.9645

13516.9889	0.20749991	0.02857543	7.26147897	3.83E-13	7.08E-12	12439.9781
7904.95418	0.20927241	0.02988541	7.00249495	2.51E-12	4.22E-11	7300.09582
15090.8838	0.166645	0.03240611	5.14239361	2.71E-07	2.31E-06	14079.8144
274.713559	0.43120004	0.11946735	3.60935475	0.00030696	0.00146202	237.966086
2973.72586	0.2236933	0.04334787	5.16042223	2.46E-07	2.11E-06	2718.5542
121.294521	-0.9387308	0.18170539	-5.166224	2.39E-07	2.05E-06	148.150093
9348.05114	-0.0936253	0.03405887	-2.7489269	0.00597907	0.02016078	9423.27183
15168.1178	-0.1113577	0.03093023	-3.6002874	0.00031787	0.00150951	15809.4668
1679.09181	0.33035904	0.05266647	6.27266312	3.55E-10	4.56E-09	1474.09342
919.412795	-0.3366572	0.06843	-4.9197316	8.67E-07	6.80E-06	1078.71786
2519.1186	-0.1743951	0.0452044	-3.8579237	0.00011435	0.00059799	2573.18192
5957.16437	0.10419355	0.03283534	3.17321338	0.00150762	0.00603112	5701.00075
1420.57989	0.28702538	0.05479738	5.23793999	1.62E-07	1.44E-06	1269.46111
1443.88549	0.28098347	0.06456219	4.35213692	1.35E-05	8.54E-05	1253.72016
1293.06845	-0.7355989	0.0592423	-12.416785	2.12E-35	1.63E-33	1617.61382
2545.09279	-0.3203735	0.04587189	-6.9840917	2.87E-12	4.78E-11	2920.4087
4922.83033	0.15492346	0.03639067	4.2572308	2.07E-05	0.00012625	4732.46952
5221.16558	0.27569256	0.04315489	6.38844247	1.68E-10	2.25E-09	4650.06103
334.443561	-1.8551449	0.11941511	-15.535262	2.00E-54	3.44E-52	510.191882
3321.79019	0.16555097	0.04470456	3.70322348	0.00021288	0.0010511	3198.19013
1363.59859	0.39774013	0.05576865	7.13196592	9.89E-13	1.75E-11	1193.53418
2992.32827	0.24447502	0.04264167	5.73324295	9.85E-09	1.04E-07	2755.59172
1673.07885	0.34610134	0.05383507	6.42891937	1.29E-10	1.76E-09	1405.574
945.690183	0.35312616	0.0711583	4.96254391	6.96E-07	5.53E-06	881.493051
4205.53931	0.50047471	0.03792012	13.1981307	8.99E-40	8.42E-38	3406.52619
3650.19874	0.30653056	0.04106378	7.46474343	8.35E-14	1.64E-12	3363.0071
2469.90635	0.43337646	0.05151942	8.41190543	4.03E-17	1.08E-15	1949.09966
2514.37824	0.36148915	0.04378146	8.25667108	1.50E-16	3.78E-15	2162.06542
1193.63985	0.58607105	0.05934577	9.8755315	5.32E-23	2.16E-21	929.641831
3389.40778	0.20058475	0.04438505	4.51919654	6.21E-06	4.21E-05	3005.59501
2918.46483	-0.5629945	0.05138598	-10.95619	6.21E-28	3.52E-26	3376.89617
321.832534	-0.3634096	0.10913889	-3.3297905	0.00086911	0.00369955	378.708674
61.2157152	-0.8705806	0.2501516	-3.480212	0.00050102	0.00226384	82.408489
245.370706	-0.3505583	0.12960918	-2.7047331	0.00683593	0.02262884	292.596433
1056.37263	0.19653468	0.06258231	3.1404189	0.00168706	0.006662	973.160921
96.5455992	-1.3164468	0.21053453	-6.2528782	4.03E-10	5.15E-09	136.112898
4801.70449	-0.5202468	0.0351854	-14.78587	1.81E-49	2.35E-47	5598.22163
767.21067	-0.5079329	0.07269888	-6.9868045	2.81E-12	4.69E-11	909.271194
5446.9083	0.19429119	0.03556731	5.46263438	4.69E-08	4.50E-07	4937.10184
73.8157521	-1.3044885	0.23563271	-5.5361095	3.09E-08	3.03E-07	105.556941
3498.44987	0.12975317	0.04378977	2.96309342	0.00304564	0.01121363	3175.96761
414.538855	-0.4465698	0.10072477	-4.4335644	9.27E-06	6.07E-05	511.11782
685.298388	-0.3282411	0.08130587	-4.037115	5.41E-05	0.00030154	762.972977

876.899349	0.22809716	0.07427464	3.07099667	0.00213346	0.00818627	815.751448
696.172565	1.25370082	0.07932956	15.8037027	2.93E-56	5.29E-54	406.486817
1333.81914	0.15537132	0.05692042	2.72962344	0.00634067	0.02122865	1252.79422
1989.70356	0.2362019	0.04980385	4.74264385	2.11E-06	1.56E-05	1776.87517
154.02108	-1.1476126	0.16280239	-7.0491142	1.80E-12	3.08E-11	215.743572
153.289367	-1.2107283	0.16126092	-7.507884	6.01E-14	1.21E-12	208.336068
4587.80889	0.15987347	0.03871832	4.12914267	3.64E-05	0.00021108	4296.35269
5886.74445	0.28965595	0.03454732	8.38432356	5.10E-17	1.35E-15	5366.73711
1922.78394	0.37290143	0.05145218	7.24753373	4.24E-13	7.81E-12	1703.72607
4266.48011	-0.1560607	0.04202646	-3.7133906	0.0002045	0.0010131	4380.61305
27691.3196	0.39016408	0.02456657	15.8819123	8.46E-57	1.59E-54	23671.607
701.04737	-1.0393034	0.07888184	-13.175446	1.22E-39	1.13E-37	918.530574
165.60303	-0.6089689	0.15584723	-3.9074732	9.33E-05	0.00049601	189.817306
594.50302	-1.0582593	0.08298847	-12.751883	3.04E-37	2.48E-35	787.973305
158.826328	-0.6670311	0.15740992	-4.237542	2.26E-05	0.00013677	178.706049
117574.413	-0.387584	0.03063169	-12.653039	1.08E-36	8.62E-35	129161.881
11.0689401	-2.2947319	0.73542652	-3.1202736	0.00180683	0.00708586	10.1853189
290.79019	0.89206512	0.11570264	7.70998105	1.26E-14	2.68E-13	201.854501
332.679728	-0.8635606	0.11202838	-7.7084091	1.27E-14	2.71E-13	439.820588
1298.34091	-1.3165652	0.0604224	-21.789356	2.93E-105	1.38E-102	1832.43146
260.477146	-0.5623128	0.12154305	-4.6264492	3.72E-06	2.62E-05	300.929876
18.3542003	-1.3235816	0.48433703	-2.7327697	0.00628042	0.0210524	19.4446997
6022.99105	-0.4908408	0.03751496	-13.083868	4.07E-39	3.67E-37	7241.76172
3298.85892	0.17156237	0.04141017	4.14300049	3.43E-05	0.00019993	3013.92845
13041.6013	-0.1586228	0.0293936	-5.3965078	6.80E-08	6.38E-07	13957.5906
7061.85843	-0.546596	0.03807373	-14.356248	9.74E-47	1.14E-44	8174.18136
187.633799	-0.8956539	0.15145284	-5.9137481	3.34E-09	3.73E-08	262.966415
866.234755	-0.3170131	0.0731467	-4.3339353	1.46E-05	9.22E-05	1006.49469
112.652412	-0.4525298	0.18922385	-2.3915051	0.01677945	0.04908989	126.853517
2058.39851	0.14856243	0.0514688	2.88645586	0.00389607	0.01391404	1968.54436
437.530217	-1.4088244	0.1019818	-13.814469	2.08E-43	2.26E-41	599.081937
558.380436	-1.0993962	0.09120051	-12.054715	1.83E-33	1.31E-31	745.380154
6447.84621	0.37782982	0.03882272	9.73218273	2.20E-22	8.70E-21	5410.2562
75.2455843	-0.6291862	0.22143851	-2.8413584	0.00449218	0.0157584	92.5938079
1641.37068	0.18357746	0.06921336	2.65234154	0.00799356	0.02588627	1587.05787
3098.11991	-0.1694715	0.05263464	-3.2197708	0.00128293	0.00522581	3385.22962
17165.214	0.54778318	0.02963107	18.4867833	2.64E-76	8.12E-74	13459.4359
20767.6915	0.18197679	0.02844491	6.39751651	1.58E-10	2.13E-09	19833.5937
5993.6054	0.1693302	0.03540416	4.78277666	1.73E-06	1.29E-05	5789.89081
2400.66175	-0.1827835	0.04700406	-3.8886745	0.00010079	0.00053239	2589.84881
1243.17324	0.55019999	0.06035813	9.11559002	7.82E-20	2.58E-18	1000.93906
226.655535	0.55579786	0.13558531	4.09924854	4.14E-05	0.00023688	203.706377
51.9913155	-1.732553	0.28682312	-6.0404927	1.54E-09	1.80E-08	79.6306748

592.753759	0.53433094	0.08284798	6.44953516	1.12E-10	1.55E-09	487.969368
3458.53534	0.19065317	0.04055243	4.70139979	2.58E-06	1.87E-05	3153.7451
25721.4434	-0.6614795	0.02658031	-24.886071	1.05E-136	8.87E-134	30824.4787
284.718778	-0.5587538	0.1225039	-4.5611102	5.09E-06	3.49E-05	350.004594
627.999534	-0.3683634	0.08270502	-4.4539425	8.43E-06	5.57E-05	713.898259
5728.0618	0.2199809	0.03407221	6.456315	1.07E-10	1.48E-09	5244.51328
1536.8581	0.66000281	0.05362406	12.307961	8.21E-35	6.17E-33	1224.09014
6164.15466	0.15718204	0.03522004	4.46285826	8.09E-06	5.37E-05	5870.44742
18.609612	-3.0312247	0.58635641	-5.1695942	2.35E-07	2.02E-06	32.4078328
5893.73239	-0.6591919	0.04030773	-16.353983	4.07E-60	8.78E-58	7092.68569
1701.65234	-0.2098327	0.06795681	-3.0877365	0.00201687	0.00781472	1898.17306
13403.189	-0.7724382	0.03336096	-23.153953	1.33E-118	8.29E-116	17178.9292
2177.05447	-0.5189228	0.04932702	-10.520051	6.98E-26	3.42E-24	2516.6997
1958.61395	0.15592873	0.05241128	2.97509858	0.00292894	0.01084162	1811.13488
1218.95889	-0.3655631	0.0663936	-5.5059991	3.67E-08	3.57E-07	1408.35182
216.789719	-1.0457667	0.13866286	-7.5417937	4.64E-14	9.41E-13	266.670167
259.875326	-1.1964662	0.12561567	-9.5248168	1.65E-21	6.26E-20	370.375232
540.087846	0.43999792	0.08743087	5.03252388	4.84E-07	3.95E-06	472.22842
1483.74245	0.22981935	0.05609554	4.09692696	4.19E-05	0.00023884	1325.94333
174.596906	0.67578538	0.14768269	4.57592811	4.74E-06	3.27E-05	144.44634
12447.2171	-0.0820588	0.03090107	-2.6555332	0.00791832	0.02567261	12600.1654
2308.36056	0.19213269	0.04600656	4.17620175	2.96E-05	0.00017497	2162.06542
509.303827	-0.94446	0.099912	-9.4529188	3.30E-21	1.23E-19	587.044742
143.945996	-0.7659218	0.16654188	-4.5989738	4.25E-06	2.95E-05	182.409802
3186.13786	-1.716338	0.0475851	-36.068808	7.00E-285	6.78E-281	4728.76577
1336.74355	0.24027444	0.05640247	4.25999834	2.04E-05	0.00012496	1212.05295
96.107761	-1.4653009	0.2058084	-7.1197333	1.08E-12	1.90E-11	136.112898
454.069362	-0.5573284	0.10351249	-5.3841655	7.28E-08	6.78E-07	541.673776
204.853901	-0.3813221	0.13816982	-2.7598076	0.00578354	0.01956532	229.632644
417.386499	-0.523902	0.09883204	-5.3009326	1.15E-07	1.04E-06	520.3772
151.27568	-0.411772	0.16173044	-2.5460388	0.01089531	0.03385247	193.521059
58.8641141	-1.3308158	0.26990032	-4.9307678	8.19E-07	6.45E-06	97.2234983
869.838693	-0.218098	0.07556976	-2.886049	0.00390112	0.01392947	924.086203
436.677182	0.52301054	0.0968261	5.40154486	6.61E-08	6.22E-07	336.115523
482.089554	-0.8658794	0.09311034	-9.2994983	1.41E-20	4.92E-19	619.452575
43.3755806	-1.3785164	0.30785309	-4.4778384	7.54E-06	5.03E-05	56.4822228
1317.52862	0.18441298	0.05742702	3.21125832	0.00132155	0.00536845	1209.27513
81.7512604	0.93020968	0.22651969	4.10652909	4.02E-05	0.00023049	53.7044086
91.7655973	-1.379078	0.20965286	-6.5779119	4.77E-11	6.86E-10	129.631331
415.766036	0.35384079	0.09868704	3.58548401	0.00033645	0.00158678	331.485832
387.662459	-0.5921149	0.11812859	-5.0124603	5.37E-07	4.35E-06	466.672792
670.520248	0.28668144	0.07900157	3.62880715	0.00028473	0.00136555	580.563176
2325.93814	0.51194536	0.04670232	10.9618836	5.83E-28	3.31E-26	1862.06148

541.223255	0.370459	0.08513454	4.35145343	1.35E-05	8.56E-05	451.857783
14.6206062	-2.2522274	0.60233305	-3.7391729	0.00018463	0.00092504	30.5559566
560.915095	-0.5778935	0.08521096	-6.7819154	1.19E-11	1.85E-10	707.416692
136.756111	-0.6044384	0.18830258	-3.2099315	0.00132767	0.00538878	174.076359
23.3748554	-1.0275125	0.41723671	-2.4626609	0.01379103	0.04147364	35.185647
165.935562	-1.3466336	0.15625026	-8.6184406	6.79E-18	1.95E-16	239.817963
338.884437	-1.0156657	0.1095731	-9.2692978	1.87E-20	6.47E-19	469.450606
56.8217963	-1.6212933	0.28253565	-5.738367	9.56E-09	1.01E-07	73.1491083
246.042772	-0.751805	0.12815653	-5.8663023	4.46E-09	4.91E-08	333.337709
311.159003	-1.220689	0.11817413	-10.329579	5.18E-25	2.37E-23	394.449622
147.260558	-0.4450466	0.16353332	-2.7214429	0.00649976	0.02166615	185.187616
1660.98319	0.21732133	0.05923053	3.6690764	0.00024343	0.00118684	1443.53747
23806.2337	0.49315008	0.03207831	15.3733166	2.47E-53	3.93E-51	19387.2915
646.108164	-0.6485124	0.08611531	-7.5307448	5.05E-14	1.02E-12	820.381138
12429.9669	-0.1904917	0.03288578	-5.7925259	6.93E-09	7.46E-08	13057.5788
10697.3028	0.24175213	0.02866728	8.43303482	3.37E-17	9.12E-16	9842.72178
7027.40006	0.27287903	0.03386534	8.05776696	7.77E-16	1.85E-14	6388.97275
179.807157	-0.90167	0.14983362	-6.0178082	1.77E-09	2.05E-08	238.892024
1576.94272	-0.1487474	0.05491173	-2.7088451	0.00675179	0.02238851	1636.13259
3228.60858	0.16602697	0.04226611	3.92813414	8.56E-05	0.00045919	3141.7079
1218.57361	-0.3040629	0.06718168	-4.52598	6.01E-06	4.08E-05	1364.83273
2174.4359	0.36979897	0.05386406	6.86541175	6.63E-12	1.06E-10	1907.43244
37.8528074	-1.555007	0.33723352	-4.6110688	4.01E-06	2.80E-05	51.8525324
1443.47982	-0.2876717	0.05543667	-5.1891956	2.11E-07	1.83E-06	1544.46472
2058.27376	0.19279521	0.05396883	3.57234406	0.0003538	0.00166144	1864.83929
1445.34774	0.25564341	0.0546532	4.67755633	2.90E-06	2.08E-05	1333.35083
2031.17018	-0.4270394	0.05101505	-8.3708511	5.72E-17	1.50E-15	2298.17831
915.691083	0.35819434	0.0692226	5.17452923	2.28E-07	1.97E-06	777.787986
2345.88385	0.39126902	0.0529413	7.39062026	1.46E-13	2.81E-12	1945.3959
1474.59353	0.31379206	0.05901776	5.31690867	1.06E-07	9.61E-07	1287.97987
829.361803	-0.3350267	0.07072234	-4.7372111	2.17E-06	1.60E-05	892.604308
13699.9553	0.18750188	0.02908436	6.44682921	1.14E-10	1.57E-09	12532.5719
134.636386	-1.3837679	0.18076588	-7.6550281	1.93E-14	4.04E-13	185.187616
2648.09194	-0.1736864	0.04420102	-3.929466	8.51E-05	0.0004569	2744.48047
15884.3503	0.30935404	0.03330085	9.28967524	1.55E-20	5.39E-19	13927.9606
1320.26857	-0.1580803	0.06068988	-2.6047223	0.00919488	0.02925368	1375.94399
86.8609198	0.80112958	0.22427881	3.57202532	0.00035423	0.00166266	62.9637894
165.382201	0.491285	0.16360068	3.00295213	0.00267375	0.0100059	135.18696
1706.71314	-0.2352548	0.05304836	-4.4347238	9.22E-06	6.04E-05	1811.13488
6161.82786	-0.2031037	0.03386625	-5.9972306	2.01E-09	2.31E-08	6526.93752
13005.9596	0.09779287	0.02753173	3.55200555	0.00038231	0.00178107	12465.9044
839.117132	0.2173183	0.07521844	2.88916232	0.0038627	0.01379992	720.379826
10224.8606	0.20721786	0.04032344	5.13889322	2.76E-07	2.35E-06	9843.64772

2990.84761	0.24868892	0.04783222	5.19919221	2.00E-07	1.74E-06	2679.6648
1648.59975	0.15882144	0.05714435	2.77930283	0.00544757	0.01859503	1552.79816
3014.00385	0.10478629	0.04278214	2.44929995	0.01431342	0.04279236	2829.66677
199.982546	-0.4824117	0.13953398	-3.4573065	0.0005456	0.00244488	224.077015
1988.2974	0.20155191	0.04922595	4.0944241	4.23E-05	0.0002413	1790.76425
6882.12823	0.21552375	0.03557943	6.05753866	1.38E-09	1.63E-08	6607.49413
5804.11064	0.21139256	0.03530724	5.98722918	2.13E-09	2.44E-08	5263.03204
1377.01461	-0.22562	0.07540606	-2.9920675	0.00277095	0.01031986	1607.42851
6817.89004	0.18264642	0.03543754	5.15403818	2.55E-07	2.18E-06	6286.19362
4867.62336	-0.2096389	0.03959015	-5.2952298	1.19E-07	1.07E-06	5289.88425
5636.29321	0.87566043	0.03925971	22.3043003	3.36E-110	1.71E-107	3931.53308
3883.6863	0.32350652	0.04148994	7.7972277	6.33E-15	1.39E-13	3399.11869
5626.12869	0.32287995	0.04051712	7.96897583	1.60E-15	3.70E-14	4859.32304
9931.7208	-0.1942973	0.0326155	-5.9572078	2.57E-09	2.91E-08	10807.5493
1813.65369	0.43092895	0.05321173	8.09838294	5.57E-16	1.34E-14	1571.31692
29.3709973	-1.4755316	0.37752952	-3.9083874	9.29E-05	0.00049428	37.0375232
3123.98152	-0.6361352	0.04124401	-15.423699	1.13E-53	1.83E-51	3801.90175
998.185868	-0.5279327	0.06805666	-7.7572528	8.68E-15	1.89E-13	1197.23794
5446.30569	0.1977946	0.03633563	5.44354398	5.22E-08	5.00E-07	5064.88129
571.306404	0.31738768	0.0849927	3.73429345	0.00018824	0.00094146	543.525652
2066.91635	0.21149896	0.05355463	3.94921866	7.84E-05	0.00042361	1940.76621
624.664678	-0.3538973	0.0804794	-4.3973645	1.10E-05	7.07E-05	713.898259
6738.59537	0.24343137	0.03443341	7.06962661	1.55E-12	2.68E-11	5977.85624
1063.27789	-0.8479487	0.07218615	-11.746694	7.34E-32	4.88E-30	1477.79717
9245.466	0.33113058	0.03677717	9.00369927	2.18E-19	6.97E-18	8167.6998
427.365529	-0.2923964	0.09833225	-2.9735554	0.00294371	0.01089005	475.932173
2760.3291	0.1874254	0.04258029	4.40169398	1.07E-05	6.94E-05	2587.07099
5244.02075	0.29997083	0.04139353	7.24680454	4.27E-13	7.85E-12	4500.05906
163.473275	0.79633967	0.15654161	5.08707992	3.64E-07	3.03E-06	125.001641
5854.49298	0.18755443	0.03354937	5.59040081	2.27E-08	2.27E-07	5440.81215
125.448173	0.65176729	0.1846216	3.53028731	0.00041511	0.00191592	76.8528606
7538.06543	0.26239967	0.0309653	8.47399187	2.37E-17	6.54E-16	6819.53395
11520.9187	0.15757012	0.03527314	4.46714246	7.93E-06	5.27E-05	10435.3222
16320.2133	-0.2693953	0.02838218	-9.4917066	2.27E-21	8.56E-20	17542.8229
49252.8741	-0.6471075	0.23944285	-2.702555	0.00688088	0.02276209	68188.858
344.217633	-0.4225451	0.10876792	-3.8848323	0.0001024	0.00053971	419.44995
663.314566	0.36515496	0.07878345	4.63491941	3.57E-06	2.53E-05	575.933485
4999.74196	-0.2437441	0.037279	-6.5383756	6.22E-11	8.82E-10	5334.32927
4162.68559	-0.2036632	0.04162234	-4.8931217	9.92E-07	7.72E-06	4393.57619
4349.02549	-0.4582093	0.04448991	-10.299173	7.11E-25	3.23E-23	4824.13739
825.067683	0.30473588	0.07185472	4.24100007	2.23E-05	0.00013484	758.343287
922.448784	0.27488898	0.07258562	3.78709943	0.00015242	0.0007765	780.565801
192.600569	-1.2568994	0.14593448	-8.612765	7.13E-18	2.04E-16	265.744229

1836.33916	0.17575492	0.04945008	3.55418901	0.00037915	0.00176805	1690.76293
5248.90687	0.14623937	0.03844437	3.80392143	0.00014242	0.00072981	4941.73153
2775.49732	-0.2771263	0.05037919	-5.5008093	3.78E-08	3.67E-07	2949.11278
320.736625	-0.3531721	0.1168524	-3.0223776	0.00250798	0.00944755	319.448637
3804.35462	0.10542753	0.0441729	2.38670146	0.01700029	0.04965355	3632.45508
932.129105	0.27133896	0.06848235	3.96217345	7.43E-05	0.00040306	805.566129
4958.12422	0.25066556	0.03767507	6.65335354	2.86E-11	4.23E-10	4578.7638
1517.64764	0.24452728	0.05335628	4.58291489	4.59E-06	3.17E-05	1385.20337
1054.89011	0.22241885	0.06672962	3.33313553	0.00085873	0.00366099	928.715893
302.181185	-1.1948807	0.11736402	-10.180979	2.41E-24	1.07E-22	428.709331
885.425485	0.75632297	0.06948634	10.8844849	1.37E-27	7.55E-26	653.712284
2448.31458	-0.247466	0.0504285	-4.9072646	9.24E-07	7.21E-06	2684.29449
5388.37131	-0.1746657	0.03680435	-4.7457899	2.08E-06	1.54E-05	5495.4425
8298.34065	0.21796779	0.0343393	6.34747248	2.19E-10	2.89E-09	7447.31997
1515.16091	0.32498242	0.05466502	5.94497959	2.76E-09	3.11E-08	1309.27644
547.401371	0.32230861	0.08503145	3.79046371	0.00015037	0.00076686	475.006235
8535.99265	0.18433566	0.03293567	5.59683991	2.18E-08	2.19E-07	7907.5112
4396.93001	0.17213297	0.03812312	4.5151859	6.33E-06	4.29E-05	4225.05546
6848.43902	0.19065965	0.03384289	5.63366861	1.76E-08	1.80E-07	6211.19263
860.659495	0.23307715	0.07274441	3.20405606	0.00135506	0.00549009	729.639206
2786.70334	0.43318435	0.045686	9.48177464	2.50E-21	9.39E-20	2352.80866
3168.63994	0.19377333	0.0437773	4.42634305	9.58E-06	6.26E-05	3042.63253
60.055471	-0.787822	0.26316	-2.9936996	0.00275617	0.01027361	95.3716221
1186.35157	0.31630075	0.06234273	5.07357847	3.90E-07	3.24E-06	1065.75473
2424.37997	-0.7664901	0.04994272	-15.347383	3.69E-53	5.81E-51	3058.37348
1088.87558	0.5374359	0.06478062	8.29624558	1.07E-16	2.74E-15	896.308061
701.752613	-0.4900595	0.08243669	-5.9446775	2.77E-09	3.11E-08	857.418661
694.026718	0.20077205	0.07597027	2.64277142	0.00822305	0.0265187	663.897603
3562.50616	0.30921466	0.03803625	8.1294738	4.31E-16	1.06E-14	3156.52291
2268.05596	0.23428757	0.0457272	5.12359345	3.00E-07	2.53E-06	2116.69445
6694.2602	0.21678332	0.03443936	6.294639	3.08E-10	3.98E-09	5987.11562
8061.45408	0.18885792	0.03974673	4.7515335	2.02E-06	1.50E-05	7242.68765
18727.4862	0.12922817	0.02762416	4.67808564	2.90E-06	2.08E-05	17578.9344
6978.92746	0.15266261	0.03174978	4.8083048	1.52E-06	1.15E-05	6558.41941
12035.9981	0.26614699	0.03141516	8.4719297	2.41E-17	6.65E-16	10870.5131
5501.05519	0.79512143	0.03882601	20.479094	3.31E-93	1.36E-90	4043.57159
4356.27275	-0.3748471	0.04620413	-8.1128484	4.94E-16	1.21E-14	5281.5508
5146.66827	-0.211078	0.03879536	-5.4408059	5.30E-08	5.06E-07	5391.73743
4649.11552	0.18808493	0.03655898	5.14469862	2.68E-07	2.28E-06	4331.53833
2826.11799	0.26682372	0.04762997	5.60201274	2.12E-08	2.13E-07	2541.70003
22816.2845	0.22633811	0.03404029	6.64912392	2.95E-11	4.34E-10	21395.6512
4165.72423	0.19952644	0.03812683	5.23322965	1.67E-07	1.47E-06	3837.0874
1490.84464	0.2345788	0.06427446	3.64964228	0.00026261	0.0012698	1319.46176

1166.50819	0.24788556	0.06450664	3.84279155	0.00012164	0.00063228	1075.01411
1179.47985	0.4709545	0.06385724	7.37511551	1.64E-13	3.13E-12	931.493708
2175.82096	0.21236171	0.05214297	4.07268151	4.65E-05	0.00026243	1987.06312
487.715452	0.34908629	0.090647	3.85105168	0.00011761	0.0006132	421.301826
723.139794	-0.7696001	0.08904488	-8.6428341	5.48E-18	1.58E-16	863.900228
593.034763	-1.1957844	0.08396862	-14.240847	5.11E-46	5.86E-44	828.714581
1736.39095	-0.1380691	0.05151374	-2.6802389	0.00735696	0.0240944	1767.61579
4676.01184	0.18529174	0.03770096	4.9147753	8.89E-07	6.97E-06	4258.38923
3139.33263	0.12374854	0.04554859	2.71684675	0.00659071	0.02192568	2997.26156
4806.6741	0.25615971	0.04104593	6.24080593	4.35E-10	5.53E-09	4203.75888
2002.09316	0.444312	0.05278492	8.41740431	3.85E-17	1.03E-15	1674.09605
3883.75308	0.33386227	0.03859935	8.64942662	5.18E-18	1.50E-16	3351.89585
4116.16161	0.13559336	0.03876908	3.4974618	0.00046971	0.0021389	3934.3109
1848.92347	-0.1466943	0.04877369	-3.0076532	0.00263273	0.00986576	1923.17339
382.366265	0.63637592	0.1002866	6.34557276	2.22E-10	2.92E-09	303.70769
455.048329	0.24890129	0.09721938	2.56020247	0.01046112	0.0327026	409.264631
6705.55924	0.18071985	0.03280567	5.50879941	3.61E-08	3.51E-07	6171.3773
1716.32413	0.64404503	0.05250958	12.265286	1.39E-34	1.04E-32	1334.27677
771.162383	0.44316248	0.07500696	5.90828497	3.46E-09	3.85E-08	653.712284
1841.42823	0.35394183	0.04915356	7.2007362	5.99E-13	1.08E-11	1644.46603
420.90014	0.64845336	0.10082388	6.43154529	1.26E-10	1.73E-09	310.189257
160.775998	-0.3978481	0.15412849	-2.5812755	0.0098436	0.03104482	175.002297
1695.70749	0.33087495	0.05185153	6.38119905	1.76E-10	2.36E-09	1500.01969
955.289058	-0.3018324	0.06826346	-4.4215811	9.80E-06	6.38E-05	1025.01345
4028.1294	0.25986575	0.0416111	6.24510594	4.24E-10	5.39E-09	3700.9745
27.9454551	-1.566156	0.38802515	-4.036223	5.43E-05	0.00030249	45.3709659
8162.05785	0.26082981	0.03794926	6.87311981	6.28E-12	1.00E-10	7047.31472
67.1597575	-1.5539027	0.26381657	-5.8900875	3.86E-09	4.28E-08	122.223826
5488.52611	0.19955253	0.03597817	5.54648951	2.91E-08	2.87E-07	4979.69499
10405.2059	-0.1633833	0.02923396	-5.5888191	2.29E-08	2.29E-07	11081.6269
1400.89598	-0.92571	0.05727995	-16.161154	9.48E-59	1.91E-56	1830.57958
3891.42338	0.09470573	0.03943244	2.40172132	0.01631813	0.04794287	3857.45804
9065.94992	-0.420261	0.03175738	-13.23349	5.62E-40	5.34E-38	10366.8027
1302.79133	-0.4125608	0.06126303	-6.7342539	1.65E-11	2.52E-10	1490.76031
1313.94042	0.17429603	0.06272815	2.77859342	0.00545948	0.01861602	1175.01542
1201.99557	0.32887783	0.05913306	5.56165799	2.67E-08	2.65E-07	1039.82846
3197.59842	-0.4943055	0.04449793	-11.108506	1.14E-28	6.68E-27	3728.75264
956.048245	0.27915189	0.06573412	4.24668169	2.17E-05	0.00013197	837.048024
2309.88294	0.23938758	0.04481613	5.34155021	9.22E-08	8.49E-07	2098.17569
1067.77368	0.22263508	0.06573634	3.3867884	0.00070716	0.00307636	961.123726
1145.38291	0.24721892	0.06024703	4.10342059	4.07E-05	0.00023306	1046.31003
1975.58419	0.34004804	0.0488806	6.95670724	3.48E-12	5.73E-11	1780.57893
193.153335	-0.5987426	0.14425423	-4.1506068	3.32E-05	0.00019385	254.632972

206.746885	-2.2061182	0.16166011	-13.646646	2.11E-42	2.15E-40	379.634612
423.309649	-0.5823609	0.0965885	-6.0292987	1.65E-09	1.92E-08	491.67312
446.44655	-0.3570956	0.09375667	-3.8087486	0.00013967	0.00071761	512.969696
1723.2563	-0.4515075	0.05312667	-8.498698	1.92E-17	5.32E-16	1992.61875
292.503973	-2.2283398	0.13203128	-16.877363	6.60E-64	1.51E-61	481.487801
832.089258	-0.3640251	0.06972405	-5.22094	1.78E-07	1.56E-06	943.530903
282.201102	-1.1254958	0.11989427	-9.3874028	6.15E-21	2.23E-19	399.079312
42.8882538	-0.7366459	0.29731612	-2.477652	0.01322501	0.04002603	56.4822228
145.398016	-0.4569138	0.16334637	-2.797208	0.00515463	0.01774193	169.446669
542.407579	2.14680629	0.09230599	23.2574983	1.19E-119	7.98E-117	199.076687
20638.2883	0.34061298	0.03350352	10.1664821	2.80E-24	1.23E-22	18291.9068
1687.7699	-0.3364614	0.05176189	-6.5001749	8.02E-11	1.13E-09	1910.21026
923.518101	0.41308775	0.06604989	6.25417801	4.00E-10	5.11E-09	785.195491
3327.21461	0.26427707	0.03969106	6.65835252	2.77E-11	4.09E-10	3031.52127
22208.6152	-0.4862295	0.02588789	-18.782125	1.06E-78	3.42E-76	25646.6329
1942.0466	0.3039308	0.05007397	6.06963676	1.28E-09	1.52E-08	1784.28268
5301.36324	0.15180517	0.03538698	4.28985957	1.79E-05	0.00011087	5086.17787
440.788945	-0.2762622	0.09409982	-2.935842	0.00332644	0.01211403	464.820916
568.293626	0.40513568	0.08597239	4.71239271	2.45E-06	1.79E-05	519.451262
317.535079	0.88202448	0.11065394	7.97101737	1.57E-15	3.65E-14	223.151077
448.919966	-0.3960139	0.0960492	-4.1230316	3.74E-05	0.00021605	487.04343
76.3847766	-2.4352303	0.25919358	-9.3954114	5.70E-21	2.08E-19	116.668198
153.373464	-0.4953264	0.16049441	-3.0862532	0.00202696	0.00783767	182.409802
611.732625	-0.5995491	0.08519714	-7.0371979	1.96E-12	3.33E-11	675.934798
690.378113	-0.3857623	0.07727575	-4.9920231	5.98E-07	4.81E-06	800.0105
62.0930548	-0.7246307	0.25201215	-2.8753799	0.00403542	0.01433246	77.7787986
692.209653	-0.9207184	0.07732203	-11.907582	1.08E-32	7.56E-31	885.196804
1196.94412	0.1425511	0.05850576	2.43653106	0.0148289	0.0441234	1131.49633
476.781742	-0.299371	0.091172	-3.2835855	0.00102496	0.00428022	543.525652
4253.49624	0.15084537	0.03671693	4.1083327	3.99E-05	0.00022911	3952.82966
4414.81824	0.28005756	0.04143739	6.75857193	1.39E-11	2.15E-10	3971.34842
22291.9693	0.18354181	0.02607561	7.03883126	1.94E-12	3.30E-11	21041.0169
22731.7357	-0.1822265	0.02859487	-6.3727006	1.86E-10	2.49E-09	23717.9039
71.6151668	-0.8356691	0.2310482	-3.6168602	0.0002982	0.00142519	93.519746
1569.72537	0.13881022	0.05498425	2.52454539	0.0115848	0.03571429	1508.35313
1959.44117	0.30479319	0.04811971	6.33406099	2.39E-10	3.14E-09	1752.80078
1110.18501	0.17841114	0.07052504	2.52975613	0.01141418	0.03523463	1087.05131
410.052589	0.29723117	0.0982337	3.02575563	0.00248013	0.00936084	351.85647
313.686068	-0.3396173	0.11113851	-3.0558023	0.00224459	0.00857199	369.449294
3109.13272	0.36932325	0.04112708	8.98004955	2.71E-19	8.56E-18	2661.14604
3002.96601	0.17934884	0.0416153	4.30968474	1.63E-05	0.00010204	2733.36921
2050.48951	-0.2991619	0.05239315	-5.7099436	1.13E-08	1.18E-07	2192.62137
981.134904	0.79315994	0.06873016	11.5402021	8.27E-31	5.33E-29	724.083578

8978.49402	0.21974898	0.03218664	6.82733557	8.65E-12	1.37E-10	8201.9595
3523.22182	0.29332213	0.03944364	7.4364872	1.03E-13	2.02E-12	3182.44918
3730.02541	-0.3459761	0.04126529	-8.3841903	5.11E-17	1.35E-15	4056.53472
4686.39034	0.2117466	0.03518189	6.01862512	1.76E-09	2.04E-08	4267.64861
2583.97343	0.21482978	0.05169963	4.15534481	3.25E-05	0.0001901	2460.21748
7754.9447	0.18474724	0.03223942	5.73047608	1.00E-08	1.05E-07	7103.79694
2279.82674	0.13724339	0.04776296	2.87342718	0.00406045	0.01440816	2202.80669
3633.80117	0.24629944	0.042879	5.74405793	9.24E-09	9.77E-08	3199.11606
3137.54741	0.17130285	0.04525148	3.78557414	0.00015335	0.00078107	2943.55715
2373.73848	0.13420127	0.04972901	2.69865154	0.0069621	0.0229798	2230.58483
3777.33794	0.33246273	0.03889023	8.54874602	1.24E-17	3.49E-16	3268.56142
10057.9597	-0.2546938	0.03054768	-8.3375834	7.58E-17	1.94E-15	10841.809
2574.62555	0.15400971	0.04395483	3.50381722	0.00045864	0.00209145	2441.69871
3501.82482	0.14093933	0.03893135	3.62020115	0.00029437	0.00140796	3289.858
385.005364	-0.3101577	0.10482886	-2.958705	0.00308935	0.01135514	412.042445
809.651343	-0.8102802	0.07921429	-10.228965	1.47E-24	6.54E-23	1025.01345
2035.79718	-0.3127748	0.05110086	-6.1207342	9.31E-10	1.13E-08	2159.2876
77.0696003	-0.6576831	0.24255764	-2.7114508	0.00669895	0.02224377	83.3344271
300.933278	-1.0445154	0.12137466	-8.6057125	7.58E-18	2.16E-16	401.857126
29.1203405	-1.3501853	0.39559693	-3.4130329	0.00064244	0.00283168	47.222842
58.1020898	-1.0387906	0.25822459	-4.0228182	5.75E-05	0.00031868	79.6306748
320.118122	-0.3974438	0.11424803	-3.4787803	0.0005037	0.00227393	328.708018
131999.765	-0.1414855	0.02944765	-4.8046459	1.55E-06	1.17E-05	135115.662
7681.99477	0.21388599	0.0310108	6.8971443	5.31E-12	8.57E-11	7055.64816
1390.49865	0.23406488	0.06004717	3.89801675	9.70E-05	0.00051423	1345.38803
1184.97992	-0.2313264	0.06167798	-3.7505505	0.00017645	0.00088715	1245.38672
7398.69769	0.34533094	0.03194197	10.8111976	3.05E-27	1.63E-25	6389.89868
1093.67012	-0.4145338	0.06318863	-6.5602593	5.37E-11	7.70E-10	1213.90482
1669.61024	-0.992254	0.05398052	-18.381704	1.84E-75	5.58E-73	2270.40017
827.782784	0.24993624	0.06923529	3.60995456	0.00030625	0.00145936	773.158296
1747.04657	-0.34767	0.0525152	-6.6203688	3.58E-11	5.22E-10	1976.8778
473.009435	-1.9104941	0.10267138	-18.607855	2.78E-77	8.82E-75	738.898587
3564.72234	-0.5466507	0.04067482	-13.439534	3.55E-41	3.44E-39	4263.94485
2399.30681	-0.244507	0.04748742	-5.1488786	2.62E-07	2.24E-06	2577.81161
3314.30739	0.22190634	0.04244093	5.22859222	1.71E-07	1.50E-06	2990.78
478.375876	0.23749239	0.09562194	2.48365995	0.01300399	0.03943717	454.635597
2625.70217	0.43523323	0.04662497	9.33476725	1.01E-20	3.57E-19	2205.5845
10705.5106	0.09954226	0.02990472	3.32864706	0.00087269	0.00371314	10068.6507
261.076778	0.40118313	0.11917909	3.36622063	0.00076206	0.00328716	230.558582
145.756891	-0.672391	0.17025688	-3.9492735	7.84E-05	0.00042361	174.076359
4296.71444	0.2069319	0.04046319	5.11407791	3.15E-07	2.65E-06	3925.97746
338.218407	-0.39335	0.10965969	-3.5870059	0.0003345	0.00157959	355.560222
41.1495726	-1.0012259	0.31167871	-3.2123654	0.00131647	0.00535005	62.0378513

1642.18118	-1.7309326	0.06081899	-28.460399	3.62E-178	5.85E-175	2483.36593
10377.2232	0.12346449	0.03075042	4.01505063	5.94E-05	0.00032871	9700.12732
10932.3455	-0.1868662	0.03482517	-5.3658377	8.06E-08	7.48E-07	11533.4847
74.9778366	0.63903386	0.22748426	2.8091344	0.00496749	0.01718016	67.5934798
2875.40479	0.23230419	0.04108162	5.65469852	1.56E-08	1.60E-07	2637.99759
1985.95689	-0.1307996	0.05151047	-2.5392817	0.01110804	0.03442514	2075.02724
1353.96288	0.27200394	0.06454658	4.21407242	2.51E-05	0.00015048	1252.79422
4180.34441	-0.1279943	0.03895432	-3.2857537	0.0010171	0.00425291	4360.24241
3898.79436	-0.2691076	0.04358821	-6.1738626	6.66E-10	8.25E-09	4301.90832
2547.02015	0.17206609	0.0442454	3.88890359	0.0001007	0.00053218	2353.7346
1332.62304	1.05003737	0.05979444	17.560786	4.92E-69	1.25E-66	875.937423
5151.07497	0.13689037	0.03520837	3.88800642	0.00010107	0.00053342	4791.72956
2892.8818	0.16934118	0.04167816	4.06306802	4.84E-05	0.00027166	2755.59172
8172.99751	0.2864061	0.035327	8.10728703	5.18E-16	1.25E-14	7408.43057
3418.67941	0.34583344	0.0421026	8.21406472	2.14E-16	5.32E-15	2924.11245
7193.33713	-0.3383959	0.03571045	-9.4761025	2.64E-21	9.90E-20	7816.76926
1076.08346	0.2455378	0.06435662	3.81526899	0.00013604	0.00070059	930.567769
665.887328	0.29243764	0.07719392	3.78835055	0.00015165	0.00077301	571.303795
487.890405	-0.6097852	0.08987139	-6.7850872	1.16E-11	1.81E-10	588.896618
87.2373587	-1.3997482	0.21676319	-6.4574996	1.06E-10	1.47E-09	125.927579
259.608562	0.39597947	0.12090895	3.275022	0.00105654	0.004396	230.558582
2656.90119	-0.1715726	0.04909223	-3.4949026	0.00047424	0.00215597	2895.40837
1359.23227	0.24314472	0.05615462	4.3299148	1.49E-05	9.38E-05	1274.0908
2537.85433	-0.2174242	0.04428784	-4.9093436	9.14E-07	7.14E-06	2693.55387
6191.83608	0.19447731	0.03618524	5.37449352	7.68E-08	7.14E-07	5682.48199
4475.7177	0.27251696	0.03834518	7.10694114	1.19E-12	2.07E-11	3938.01465
822.409803	0.4407811	0.07178012	6.14071268	8.22E-10	1.01E-08	668.527293
87.5846207	0.59949777	0.22688105	2.6423439	0.00823344	0.02654778	52.7784705
10319.5135	0.34428556	0.031833	10.8153655	2.91E-27	1.57E-25	9164.93511
3237.43731	-0.2881669	0.04177414	-6.8982146	5.27E-12	8.51E-11	3512.08313
837.608131	-0.3533275	0.07551799	-4.6787204	2.89E-06	2.07E-05	1000.01313
710.434682	-0.242575	0.08045038	-3.0152126	0.00256799	0.00965113	720.379826
318.877188	-0.3478493	0.11897074	-2.9238224	0.00345762	0.01252354	411.116507
1420.94261	-0.3567349	0.05818342	-6.1312121	8.72E-10	1.06E-08	1579.65036
5754.73569	0.13841805	0.03329458	4.15737477	3.22E-05	0.00018865	5509.33157
3418.85335	0.84226968	0.04016831	20.9685127	1.27E-97	5.60E-95	2411.14276
7970.9131	0.35057737	0.03308692	10.5956471	3.12E-26	1.56E-24	6845.46022
6191.6836	0.22201745	0.03613307	6.14443886	8.02E-10	9.83E-09	5702.85263
2171.56177	0.30043413	0.04691912	6.40323522	1.52E-10	2.06E-09	1909.28432
2535.28311	0.23118093	0.05034138	4.5922648	4.38E-06	3.05E-05	2247.25172
1302.08756	0.16410847	0.05970696	2.74856515	0.00598567	0.02017953	1250.01641
1849.01081	0.38449802	0.04987062	7.70991027	1.26E-14	2.68E-13	1560.20566
77854.5077	0.28439367	0.02734971	10.3984153	2.52E-25	1.19E-23	70460.1841

7358.08806	0.19000636	0.03723272	5.10320892	3.34E-07	2.80E-06	7032.49971
3060.7746	0.27814916	0.04103931	6.7776271	1.22E-11	1.90E-10	2740.77671
2781.8028	0.17261078	0.04560598	3.78482781	0.00015382	0.0007826	2553.73722
1452.70257	0.2668022	0.05548067	4.80892195	1.52E-06	1.15E-05	1303.72082
1468.54259	0.44591728	0.05621596	7.93221825	2.15E-15	4.94E-14	1291.68362
4035.1179	-0.1881868	0.04084481	-4.6073622	4.08E-06	2.85E-05	4389.87243
2112.07192	-1.5250972	0.05003271	-30.482005	4.51E-204	1.75E-200	3190.78262
654.392282	0.1988464	0.08039307	2.47342705	0.01338241	0.04038893	596.304123
3904.18898	0.3738325	0.0405701	9.21448296	3.13E-20	1.06E-18	3382.4518
23.4031711	0.97949562	0.40083803	2.4436195	0.01454075	0.04338502	16.6668854
558.768484	0.23267499	0.09028198	2.57720285	0.00996035	0.03135965	567.600043
537.50867	-0.2393084	0.08619171	-2.7764662	0.00549533	0.01871194	559.2666
3850.99252	0.19392475	0.03770416	5.14332499	2.70E-07	2.30E-06	3557.4541
1122.70441	-0.2517653	0.07129621	-3.531257	0.00041359	0.00191073	1281.4983
23804.3216	-0.6751849	0.03759607	-17.958923	4.09E-72	1.13E-69	30626.3279
156.059779	0.6495766	0.15544063	4.17893707	2.93E-05	0.00017346	130.557269
8124.29729	-0.5186375	0.03430347	-15.119099	1.21E-51	1.71E-49	9751.05391
1031.50768	-0.3661599	0.06614458	-5.5357513	3.10E-08	3.04E-07	1137.05196
2763.71255	0.14085964	0.04498606	3.13118393	0.00174103	0.00685277	2567.62629
4058.30301	-0.3095826	0.03706586	-8.3522301	6.70E-17	1.73E-15	4528.76315
404.221393	-0.5733788	0.10155201	-5.6461583	1.64E-08	1.68E-07	466.672792
923.529807	0.29642291	0.06717143	4.4129315	1.02E-05	6.62E-05	862.048352
380.306473	-0.3239104	0.10247559	-3.1608545	0.00157307	0.00626674	410.190569
120.957234	-3.0108094	0.22760342	-13.228314	6.02E-40	5.69E-38	197.224811
5885.02689	0.26748201	0.03609367	7.41077338	1.26E-13	2.42E-12	5379.70024
832.161828	0.46833789	0.06879683	6.80755036	9.93E-12	1.56E-10	706.490754
12347.4683	-0.1253973	0.02835087	-4.4230514	9.73E-06	6.35E-05	12920.54
2044.89129	0.21999049	0.0469617	4.68446604	2.81E-06	2.02E-05	1899.099
3816.67188	0.15547249	0.03957319	3.92873333	8.54E-05	0.00045817	3565.78754
1271.35012	0.21574979	0.05821563	3.70604566	0.00021052	0.00104068	1144.45947
2030.85939	0.29518325	0.0575316	5.1308023	2.89E-07	2.44E-06	1703.72607
25.890041	2.48359932	0.44514479	5.57930668	2.41E-08	2.41E-07	7.40750463
3628.08209	-0.1132894	0.04000286	-2.8320312	0.00462533	0.01615533	3863.9396
2005.80422	0.31140655	0.0500997	6.21573719	5.11E-10	6.44E-09	1722.24483
4024.98708	-0.1554267	0.0387206	-4.014057	5.97E-05	0.0003299	4245.42609
394.424916	-0.6569236	0.1061939	-6.1860768	6.17E-10	7.67E-09	537.970024
16073.6387	0.12077375	0.02767065	4.36468757	1.27E-05	8.10E-05	15287.2377
4681.30387	0.15214002	0.03835079	3.96706374	7.28E-05	0.0003951	4272.2783
2556.6022	0.36123733	0.04557354	7.92647025	2.25E-15	5.15E-14	2340.77146
763.207218	0.53914826	0.07394329	7.29137524	3.07E-13	5.72E-12	645.378841
2548.12879	0.26994968	0.04522709	5.96876065	2.39E-09	2.72E-08	2318.54895
2961.68282	-0.2085325	0.04068826	-5.1251256	2.97E-07	2.51E-06	3181.52324
2342.71956	0.23186418	0.04693697	4.93990519	7.82E-07	6.17E-06	2125.02789

1812.68306	0.19127597	0.04977573	3.8427557	0.00012166	0.00063228	1688.91106
643.032437	0.27701765	0.08005376	3.46039529	0.00053938	0.00241868	607.41538
1702.24519	-0.2590675	0.05116387	-5.0634856	4.12E-07	3.40E-06	1845.39459
12082.5704	0.1140704	0.03695185	3.08700062	0.00202187	0.00782628	11758.4877
1315.08522	0.14511291	0.05965698	2.43245477	0.01499687	0.0445539	1271.31298
343.165832	-0.2962552	0.10519181	-2.8163333	0.00485752	0.01686604	387.042117
2080.22598	0.1936892	0.04868566	3.97836238	6.94E-05	0.00037834	1905.58057
2298.96645	0.14118344	0.04507859	3.13193974	0.00173656	0.00683793	2220.39951
7914.73655	0.29898141	0.03320645	9.00371617	2.18E-19	6.97E-18	7022.31439
1160.93423	-0.2477798	0.06085819	-4.071429	4.67E-05	0.00026354	1225.01608
898.784328	0.74861611	0.06951982	10.7683846	4.85E-27	2.54E-25	664.823541
1708.11199	0.23977482	0.05346025	4.48510452	7.29E-06	4.88E-05	1561.1316
98.7797101	-0.6197588	0.20336782	-3.0474772	0.00230771	0.00877328	121.297888
4963.02833	-0.2728037	0.03476619	-7.8468112	4.27E-15	9.48E-14	5469.51623
4279.83224	0.33158727	0.03923704	8.45087425	2.89E-17	7.88E-16	3665.78886
530.972701	0.24195977	0.08949305	2.70367119	0.00685781	0.0226974	486.117492
490.429628	0.55431876	0.09532702	5.81491737	6.07E-09	6.58E-08	424.07964
51727.0705	0.16650244	0.06371226	2.61335	0.00896594	0.02860043	50357.1424
596.152009	-0.491501	0.08484466	-5.7929519	6.92E-09	7.45E-08	748.157968
4799.84939	0.15013112	0.03526059	4.25776011	2.06E-05	0.00012603	4477.83655
3119.16212	0.28950799	0.0401429	7.21193556	5.52E-13	1.00E-11	2834.29646
4893.57062	0.32360448	0.03843677	8.41913941	3.79E-17	1.02E-15	4206.53669
3164.95656	-0.1111221	0.0439272	-2.529687	0.01141643	0.03523463	3269.48736
1276.90025	-0.1789434	0.06057603	-2.9540302	0.00313653	0.01150779	1418.53714
22564.1779	0.11139092	0.02551177	4.36625684	1.26E-05	8.05E-05	21920.6581
3493.85839	0.21724968	0.04044258	5.37180573	7.80E-08	7.24E-07	3280.59861
963.64399	0.18153029	0.06920858	2.62294481	0.00871734	0.02788542	912.049008
2898.60279	-0.1509258	0.04952788	-3.0472902	0.00230915	0.00877702	2947.26091
14681.3089	0.18391544	0.03695413	4.97685786	6.46E-07	5.17E-06	13863.1449
12887.6296	0.2973125	0.04408704	6.74376265	1.54E-11	2.37E-10	11331.6302
755.901699	-0.943416	0.07528492	-12.531275	5.03E-36	3.94E-34	978.71655
582.013987	0.23793953	0.09071175	2.62302876	0.00871519	0.02788315	488.895306
2701.46948	0.15094965	0.04686279	3.22109846	0.001277	0.00520494	2477.8103
25933.2022	-0.2251915	0.02424944	-9.2864638	1.59E-20	5.54E-19	28099.4429
1351.16936	-0.4755024	0.05742319	-8.2806695	1.22E-16	3.11E-15	1594.46537
169.260021	-0.9543427	0.15571084	-6.1289422	8.85E-10	1.08E-08	216.669511
393.319872	-0.3038205	0.10119139	-3.0024342	0.0026783	0.010021	430.561207
2127.96189	-0.5235615	0.04845689	-10.804685	3.27E-27	1.74E-25	2537.99628
1742.36423	-0.4600468	0.05024734	-9.1556454	5.40E-20	1.81E-18	2005.58188
7468.41029	-0.1145295	0.03217359	-3.5597366	0.00037123	0.00173445	7627.8779
1583.85012	0.45826075	0.06178239	7.41733605	1.19E-13	2.31E-12	1406.49994
2824.61626	-0.4862351	0.04305152	-11.294259	1.40E-29	8.65E-28	3366.71086
136.551879	-0.610627	0.16718359	-3.6524339	0.00025977	0.00125764	156.483535

1462.15356	0.2757829	0.05711955	4.82817024	1.38E-06	1.05E-05	1319.46176
20601.6405	-0.4359138	0.02937515	-14.839544	8.13E-50	1.09E-47	23682.7183
1812.2	-0.3394649	0.05120493	-6.6295362	3.37E-11	4.92E-10	2017.61907
4567.45604	-0.227217	0.03908844	-5.8128957	6.14E-09	6.66E-08	4893.58275
25825.038	0.19516512	0.02649482	7.3661628	1.76E-13	3.34E-12	23749.3858
127.907657	-0.4540277	0.17129774	-2.6505177	0.00803685	0.02600039	142.594464
1472.34986	-0.8229389	0.05798091	-14.193272	1.01E-45	1.15E-43	1837.98709
593.605077	0.33175341	0.0843705	3.93210178	8.42E-05	0.00045242	546.303467
1451.50388	0.39873029	0.0599548	6.65051457	2.92E-11	4.30E-10	1188.90449
433.10502	0.38212153	0.09515786	4.01565915	5.93E-05	0.00032795	386.116179
1066.25645	0.27252275	0.06449895	4.22522773	2.39E-05	0.0001437	972.234983
12891.3609	0.34936107	0.02914834	11.9856231	4.23E-33	2.99E-31	11234.4067
1018.06067	-0.903545	0.06596004	-13.69837	1.04E-42	1.08E-40	1350.01772
13137.6702	-0.2360216	0.03131774	-7.5363535	4.83E-14	9.79E-13	14160.371
3953.63624	-0.1834343	0.04092191	-4.482546	7.38E-06	4.93E-05	4146.35072
216.12699	-0.6008335	0.13586984	-4.4221259	9.77E-06	6.37E-05	275.00361
357.9871	0.40280665	0.10273137	3.92097031	8.82E-05	0.00047149	302.781752
14615.2472	0.17477722	0.03150209	5.54811509	2.89E-08	2.85E-07	13528.8813
63.1236128	-1.6931925	0.26468602	-6.396985	1.58E-10	2.13E-09	101.853189
4396.25216	-0.5940911	0.03821206	-15.547212	1.66E-54	2.88E-52	5370.44086
10073.9157	0.23741902	0.03625079	6.54934699	5.78E-11	8.24E-10	9295.49238
1756.07625	-0.1443104	0.0524322	-2.7523248	0.00591738	0.01997013	1850.02428
1070.25892	-0.2293279	0.06427171	-3.5681007	0.00035958	0.00168327	1169.45979
1499.96915	0.19626452	0.05345628	3.67149602	0.00024114	0.00117684	1423.16683
593.35434	-0.4634964	0.09141982	-5.0699774	3.98E-07	3.29E-06	686.120117
1153.73742	0.39248808	0.06036218	6.50221888	7.91E-11	1.12E-09	978.71655
2873.68016	0.28891759	0.04388079	6.58414772	4.58E-11	6.58E-10	2518.55158
958.159322	-0.5831326	0.07385871	-7.8952451	2.90E-15	6.57E-14	1252.79422
414.519685	-1.4913402	0.10711128	-13.92328	4.57E-44	5.07E-42	587.044742
159.965835	-0.6506808	0.16139254	-4.0316658	5.54E-05	0.00030789	218.521387
79.6169053	-1.0041386	0.22248711	-4.5132439	6.38E-06	4.32E-05	114.816322
209.630167	-0.490997	0.14366901	-3.4175566	0.00063186	0.00278884	237.040148
1656.39672	0.18339285	0.05137741	3.56952318	0.00035763	0.00167537	1523.16814
36135.0837	0.19707345	0.02730383	7.21779613	5.28E-13	9.63E-12	32760.6152
7310.39229	0.09844467	0.0335133	2.93748035	0.00330891	0.01206154	7169.53855
5608.85907	0.17342621	0.03612565	4.80063873	1.58E-06	1.19E-05	5254.6986
7979.44298	0.12146989	0.03722025	3.26354293	0.00110029	0.00455943	7448.24591
13524.6525	0.28936504	0.04259572	6.79328966	1.10E-11	1.71E-10	11549.2257
2169.04393	-0.1458483	0.04838292	-3.0144578	0.00257439	0.00966955	2348.17897
3212.92985	0.16068433	0.04218672	3.80888391	0.0001396	0.00071741	2928.74214
2702.74571	0.29656775	0.05029026	5.89712054	3.70E-09	4.10E-08	2310.21551
138.203818	1.70601133	0.18810238	9.06958913	1.19E-19	3.89E-18	63.8897275
160.934405	0.85957275	0.16240953	5.2926252	1.21E-07	1.09E-06	124.075703

4023.07793	0.22014462	0.03813762	5.77237503	7.82E-09	8.36E-08	3651.89978
6524.3273	0.18265016	0.03602103	5.07065393	3.96E-07	3.28E-06	6266.74892
3509.62314	0.1306137	0.04103693	3.18283348	0.00145842	0.005863	3301.89519
469.740467	-0.5494786	0.09328744	-5.890167	3.86E-09	4.28E-08	537.970024
1416.33306	0.4566549	0.05631132	8.10946922	5.08E-16	1.24E-14	1182.42293
833.308509	0.95878935	0.07360808	13.0255998	8.75E-39	7.82E-37	524.080953
164.222808	0.40339877	0.15229663	2.64877008	0.00807853	0.0261265	150.001969
895.946991	-0.264313	0.0687783	-3.8429702	0.00012155	0.00063206	962.975602
553.877807	-0.255924	0.08816178	-2.9028903	0.00369736	0.01328715	579.637238
2457.22094	0.18609666	0.04547627	4.09217109	4.27E-05	0.00024337	2281.51143
784.715563	-0.225098	0.07135041	-3.1548241	0.00160595	0.00637937	837.973962
3112.86166	-0.1366476	0.04195972	-3.256638	0.0011274	0.00466283	3342.63647
4078.51734	0.16095573	0.04155549	3.87327213	0.00010738	0.00056321	3813.01301
4413.86721	0.57188874	0.03789022	15.0933073	1.79E-51	2.50E-49	3440.7859
5756.71077	0.10498395	0.03311091	3.17067548	0.00152085	0.00607744	5548.22097
2105.60677	-0.2416098	0.04655166	-5.1901436	2.10E-07	1.82E-06	2296.32644
6212.18886	0.27168229	0.03598579	7.54971103	4.36E-14	8.88E-13	5426.92308
2456.6484	0.20643134	0.05003428	4.12579852	3.69E-05	0.00021379	2385.21649
608.095675	0.25179679	0.08376099	3.00613455	0.00264592	0.00990559	580.563176
1672.95573	0.24882743	0.05434553	4.57861785	4.68E-06	3.23E-05	1573.1688
1097.35983	-0.3343505	0.06482591	-5.1576676	2.50E-07	2.14E-06	1255.57204
213.977611	0.41264487	0.13242032	3.11617484	0.00183214	0.00717494	186.113554
2111.81908	0.11780812	0.04695556	2.50892789	0.01210982	0.03710858	2054.6566
2809.7634	0.2825566	0.04396963	6.42617602	1.31E-10	1.79E-09	2525.95908
1642.48756	-0.1726856	0.053261	-3.2422525	0.00118589	0.00487767	1694.46669
1825.57595	-0.2554507	0.05498683	-4.6456704	3.39E-06	2.41E-05	1944.46997
2736.99816	0.22551169	0.04326221	5.21267097	1.86E-07	1.63E-06	2458.3656
608.217651	0.39510761	0.08057477	4.9036145	9.41E-07	7.34E-06	539.8219
1720.9229	-0.3412991	0.05474742	-6.2340683	4.54E-10	5.76E-09	1928.72902
4342.35253	-0.2166116	0.03863842	-5.60612	2.07E-08	2.09E-07	4537.09659
1451.67328	-0.4487545	0.05660911	-7.9272498	2.24E-15	5.13E-14	1727.80046
1995.69839	0.19681783	0.05001265	3.93536069	8.31E-05	0.0004467	1790.76425
3368.3355	0.16747146	0.04603247	3.63811575	0.00027464	0.0013235	3248.19078
362.813634	0.57763094	0.10658074	5.41965578	5.97E-08	5.67E-07	273.151733
389.800422	-0.2442527	0.09993653	-2.4440779	0.01452229	0.04334328	433.339021
124.287328	-0.666763	0.18303232	-3.6428702	0.00026962	0.00130142	161.113226
3548.54597	0.15529317	0.04054233	3.83039584	0.00012794	0.00066206	3245.41297
789.412969	-0.3361585	0.0731086	-4.5980706	4.26E-06	2.96E-05	850.011157
1177.45127	0.22840283	0.05964002	3.82969086	0.0001283	0.00066378	1102.79225
3398.23937	-0.32654	0.0394608	-8.2750485	1.28E-16	3.25E-15	3793.56831
2532.59187	-0.2581183	0.04401794	-5.863935	4.52E-09	4.98E-08	2701.88732
28899.4319	0.12895628	0.02757495	4.67657369	2.92E-06	2.09E-05	27203.1348
12556.4247	0.14625179	0.03286844	4.4496115	8.60E-06	5.66E-05	11802.0068

187498.386	0.31107458	0.02195845	14.166509	1.48E-45	1.66E-43	166561.445
2539.80835	-0.2281553	0.04348843	-5.2463437	1.55E-07	1.38E-06	2738.92484
28.5064834	-0.9151502	0.36600097	-2.5004038	0.01240518	0.03789835	33.3337709
9387.32999	0.09791167	0.03776724	2.59250269	0.00952804	0.03018979	8713.07732
367.944663	-0.5481096	0.106377	-5.1525201	2.57E-07	2.19E-06	406.486817
9.6492267	-2.5898491	0.79953852	-3.2391799	0.00119874	0.0049253	12.9631331
15.3869385	-2.6122635	0.59093344	-4.4205714	9.84E-06	6.41E-05	30.5559566
4665.47111	0.19080965	0.03656084	5.2189623	1.80E-07	1.58E-06	4331.53833
3712.07715	0.19440527	0.04129896	4.70726849	2.51E-06	1.83E-05	3463.00842
1731.26081	0.51701182	0.05247022	9.85343288	6.62E-23	2.69E-21	1387.98118
15235.7936	0.32606333	0.0301585	10.8116574	3.03E-27	1.63E-25	13326.1008
661.615193	-0.5524331	0.08942917	-6.1773255	6.52E-10	8.08E-09	833.344271
2460.77076	-0.4949195	0.04860453	-10.182581	2.37E-24	1.05E-22	2726.88764
3012.26262	-0.1747042	0.04109308	-4.2514275	2.12E-05	0.00012936	3177.81949
1100.71581	0.38984087	0.06102218	6.38851089	1.68E-10	2.25E-09	971.309045
822.50571	0.19888346	0.07367523	2.69946187	0.00694517	0.02293172	745.380154
1474.48543	0.27959426	0.05558823	5.02973831	4.91E-07	4.00E-06	1299.09113
140.756091	-0.5862648	0.16564411	-3.5393037	0.00040118	0.00185963	170.372607
976.812894	0.33705235	0.06791975	4.9625088	6.96E-07	5.53E-06	816.677386
3342.0494	0.15397358	0.03998287	3.85098866	0.00011764	0.0006132	3105.59632
3953.12849	-1.1966062	0.03823886	-31.292935	5.82E-215	2.82E-211	5433.40465
2731.86784	-0.2423567	0.04688942	-5.1686869	2.36E-07	2.03E-06	2797.25894
579.828719	-0.8623979	0.08343875	-10.335699	4.86E-25	2.24E-23	750.935782
3695.7152	0.12118908	0.03969403	3.05308091	0.00226505	0.0086348	3620.41789
279.531499	0.43371781	0.1232917	3.51781855	0.00043511	0.00199588	213.891696
12144.917	-0.767726	0.03728918	-20.588438	3.48E-94	1.47E-91	16104.841
1686.50998	0.15697286	0.05364307	2.92624688	0.00343079	0.01244494	1605.57663
9389.67754	0.30641581	0.0293693	10.4332009	1.75E-25	8.35E-24	8536.22315
1477.6992	-0.2266478	0.05469377	-4.1439422	3.41E-05	0.00019921	1616.68789
2479.21935	-0.1541976	0.04877999	-3.1610826	0.00157184	0.0062644	2635.21977
123.225708	-0.5621119	0.18242653	-3.0813056	0.00206095	0.00794739	162.039164
4070.82606	0.25505016	0.04269633	5.973585	2.32E-09	2.65E-08	3651.89978
863.595258	0.23627375	0.07213004	3.27566378	0.00105414	0.00438841	744.454216
1682.10224	-0.3100745	0.05494705	-5.6431513	1.67E-08	1.71E-07	1800.02363
385.267217	0.29849078	0.1014104	2.94339415	0.00324635	0.01186472	336.115523
1679.09799	0.14633199	0.05259252	2.78237245	0.00539631	0.01845284	1553.7241
1722.22727	-0.2141516	0.05277167	-4.0580783	4.95E-05	0.00027721	1856.50585
3065.30375	-0.5352987	0.049581	-10.796448	3.58E-27	1.89E-25	3726.90077
2383.40885	0.12007062	0.04465646	2.68876246	0.00717174	0.02355944	2305.58582
482.521801	-0.4171465	0.09286832	-4.4918055	7.06E-06	4.74E-05	564.822228
3504.33802	0.30803232	0.0397266	7.75380442	8.92E-15	1.93E-13	3073.18848
493.252799	-0.3898785	0.09142434	-4.2644932	2.00E-05	0.00012279	525.932829
1840.88109	0.7309377	0.04989776	14.6487091	1.37E-48	1.68E-46	1413.90745

5665.98955	0.2032913	0.0362078	5.61457212	1.97E-08	1.99E-07	5311.18082
399.398735	0.49416652	0.10533962	4.69117417	2.72E-06	1.96E-05	304.633628
1341.15374	-0.3076607	0.05796814	-5.3074096	1.11E-07	1.01E-06	1487.98249
755.989787	-0.5441828	0.07508349	-7.2477026	4.24E-13	7.81E-12	849.085219
3818.55451	0.11695775	0.04288314	2.72735997	0.00638434	0.02134167	3742.64172
3543.5711	0.12638743	0.03940241	3.20760661	0.00133844	0.00542912	3337.08084
6796.58674	0.21913393	0.03598721	6.08921728	1.13E-09	1.36E-08	6268.6008
8678.58043	0.17858811	0.04032379	4.42885184	9.47E-06	6.19E-05	7970.47498
85.4310954	-1.1249148	0.21895268	-5.1377073	2.78E-07	2.36E-06	125.927579
126.751605	-1.1190115	0.18735888	-5.9725568	2.34E-09	2.66E-08	172.224483
1229.85226	0.18428612	0.06206435	2.96927484	0.00298504	0.01102399	1113.90351
2529.18378	0.21254445	0.0536132	3.96440484	7.36E-05	0.00039942	2216.69576
8474.63075	0.1394986	0.0412633	3.38069433	0.00072303	0.00313906	8356.59116
178.338545	0.50972309	0.14615067	3.48765471	0.00048728	0.00221008	137.038836
1311.90884	-0.1871147	0.06063636	-3.0858502	0.00202971	0.00784409	1454.64872
28.2972394	-2.8622272	0.46159893	-6.2006799	5.62E-10	7.04E-09	50.9265944
141.716673	0.93486938	0.1675935	5.57819592	2.43E-08	2.43E-07	105.556941
56.4850525	-1.3186884	0.2726252	-4.8370011	1.32E-06	1.01E-05	80.5566129
569.644454	-0.2756586	0.08211526	-3.3569716	0.00078801	0.00338705	624.082265
205.707198	0.44882269	0.1343045	3.34182915	0.00083228	0.00356155	175.928235
2335.35015	-0.181822	0.04685497	-3.8805275	0.00010423	0.0005486	2444.47653
96.2788877	-1.1673486	0.22785621	-5.1231811	3.00E-07	2.54E-06	133.335083
10271.8802	0.19565842	0.0289986	6.74716871	1.51E-11	2.32E-10	9502.90251
2007.27213	0.21035311	0.0492655	4.26978521	1.96E-05	0.00012022	1839.83896
744.328217	-1.1132899	0.0765228	-14.548473	5.97E-48	7.15E-46	1017.60595
938.0298	0.37797168	0.0675153	5.59831136	2.16E-08	2.18E-07	801.862377
181.387503	-0.7508947	0.14658452	-5.1226061	3.01E-07	2.54E-06	243.521715
12071.1211	0.26491468	0.03070118	8.6288118	6.20E-18	1.78E-16	10997.3666
2063.41771	-0.7731899	0.04851894	-15.935836	3.57E-57	6.93E-55	2589.84881
234.647086	-0.6204861	0.13271744	-4.6752417	2.94E-06	2.10E-05	280.559238
12157.5595	0.14741405	0.03064403	4.81053069	1.51E-06	1.14E-05	11514.966
2165.32697	-0.3129796	0.04684813	-6.6807279	2.38E-11	3.56E-10	2403.73525
1007.76602	-0.4847346	0.06984081	-6.9405645	3.91E-12	6.39E-11	1136.12602
129.316144	-0.5252289	0.17109515	-3.0698058	0.00214198	0.00821573	152.779783
1191.91212	-0.3174097	0.06008123	-5.2830099	1.27E-07	1.14E-06	1337.98052
2902.62072	0.17147141	0.04214428	4.068676	4.73E-05	0.00026644	2788.92549
1125.01876	-0.1580231	0.06404187	-2.4674962	0.01360617	0.04098129	1162.05229
1417.7397	0.32770806	0.05755612	5.69371327	1.24E-08	1.29E-07	1196.312
3806.82377	-0.1067987	0.03890868	-2.7448543	0.00605378	0.02037542	3966.71873
625.876823	-0.9294507	0.08405693	-11.057395	2.02E-28	1.17E-26	762.972977
2804.27649	0.30912929	0.04184051	7.38827785	1.49E-13	2.85E-12	2483.36593
14462.7866	-0.1685457	0.02825875	-5.9643733	2.46E-09	2.79E-08	15136.3098
432.201253	-0.6076629	0.10868186	-5.5912076	2.25E-08	2.26E-07	567.600043

112.345522	-0.7757105	0.1848013	-4.1975382	2.70E-05	0.0001609	151.853845
14698.7035	-0.1152657	0.02887646	-3.9916821	6.56E-05	0.00035977	15014.086
5163.61812	0.22086102	0.03450944	6.40001777	1.55E-10	2.10E-09	4793.58144
810.0542	0.21318113	0.07065329	3.01728502	0.0025505	0.00959283	738.898587
12531.1102	0.07762475	0.02785707	2.78653707	0.00532745	0.01823961	12124.2332
25641.6066	-0.4229369	0.02552308	-16.570759	1.13E-61	2.50E-59	28817.9708
7711.09387	-0.1093741	0.03298363	-3.3160119	0.00091312	0.00386144	8007.51251
2376.40074	0.26605022	0.04669307	5.69785294	1.21E-08	1.26E-07	2169.47292
10154.7861	0.19074322	0.0293915	6.48974152	8.60E-11	1.21E-09	9476.97624
8061.1186	0.31142057	0.03476792	8.95712437	3.33E-19	1.05E-17	6888.05337
1051.74438	-0.2409199	0.06477711	-3.7192143	0.00019984	0.00099256	1134.27415
3509.81514	0.44168836	0.04256378	10.3770941	3.15E-25	1.48E-23	3054.66972
2409.32509	0.17015246	0.0454895	3.7404776	0.00018367	0.00092073	2292.62268
2010.29307	0.15625576	0.04755237	3.28597199	0.00101631	0.00425053	1862.06148
2230.07824	0.27774902	0.04615282	6.01802949	1.77E-09	2.05E-08	2038.91565
89.923736	-0.9385318	0.22267942	-4.2147216	2.50E-05	0.00015009	105.556941
1386.18978	0.53229179	0.05966798	8.9208955	4.63E-19	1.44E-17	1115.75539
4394.53352	-0.6534461	0.04038753	-16.179402	7.05E-59	1.44E-56	5555.62847
1453.56423	0.33733434	0.05548501	6.07973806	1.20E-09	1.43E-08	1306.49863
847.010735	0.30047963	0.07495756	4.00866366	6.11E-05	0.00033657	787.973305
2198.39968	0.16295821	0.04780996	3.40845766	0.00065331	0.00287437	2112.9907
2528.93882	0.33240368	0.04585816	7.24851718	4.21E-13	7.78E-12	2172.25073
5271.88424	0.12872221	0.03930848	3.27466745	0.00105786	0.00439964	4813.02614
169.472247	-0.5522046	0.15022904	-3.6757515	0.00023715	0.00116119	200.002625
271.825994	-0.323261	0.12447617	-2.5969709	0.00940499	0.02985845	269.447981
7455.79413	0.27424771	0.03299478	8.31185208	9.42E-17	2.41E-15	6549.16003
1632.82016	0.159236	0.05154557	3.0892277	0.00200678	0.00778183	1542.61284
665.893265	-0.3114154	0.07924508	-3.9297764	8.50E-05	0.00045644	709.268569
821.000146	-0.3318744	0.07080195	-4.6873616	2.77E-06	1.99E-05	914.826822
1306.06048	-0.3610454	0.05937649	-6.0806119	1.20E-09	1.43E-08	1446.31528
803.852019	-0.2638782	0.070139	-3.762218	0.00016841	0.00084996	864.826166
1478.43643	0.2070064	0.05570463	3.71614344	0.00020229	0.00100367	1390.759
3287.33858	0.12817401	0.03919724	3.2699756	0.00107557	0.0044656	3138.93009
5863.11704	0.22996975	0.03821486	6.01780996	1.77E-09	2.05E-08	5175.06792
1894.28631	0.37617185	0.05175028	7.26898181	3.62E-13	6.71E-12	1675.94792
3504.20582	-0.403464	0.04020131	-10.036091	1.06E-23	4.48E-22	4105.60944
1548.96574	-0.6839076	0.05339211	-12.809152	1.46E-37	1.22E-35	1925.9512
8315.87244	0.15427387	0.03642916	4.23490084	2.29E-05	0.00013821	7659.35979
200.771273	-0.7928832	0.14026482	-5.652759	1.58E-08	1.62E-07	249.077343
838.968521	0.29206446	0.06992866	4.17660578	2.96E-05	0.00017481	723.15764
3390.3567	-0.3735657	0.05596482	-6.675009	2.47E-11	3.69E-10	3708.38201
999.223041	-0.2172827	0.06544763	-3.3199481	0.00090034	0.00381573	1099.0885
441.209776	-0.4926172	0.09763203	-5.0456508	4.52E-07	3.71E-06	491.67312

7666.75635	0.21836476	0.03160889	6.90833376	4.90E-12	7.94E-11	7160.27917
554.897673	0.48923533	0.08476737	5.77150539	7.86E-09	8.39E-08	442.598402
4852.3081	0.15414623	0.04179193	3.68842123	0.00022565	0.00111021	4541.72628
21.4406207	1.20232931	0.4203718	2.86015691	0.00423431	0.01495248	13.8890712
310.087597	-0.5230594	0.11187721	-4.6752994	2.94E-06	2.10E-05	386.116179
1728.59639	0.22268713	0.05355825	4.15784947	3.21E-05	0.00018832	1587.05787
80.8971901	-1.053601	0.22123099	-4.7624475	1.91E-06	1.42E-05	108.334755
12203.2646	-0.2174003	0.02928395	-7.4238727	1.14E-13	2.21E-12	13185.3583
876.414615	0.16341824	0.06690872	2.44240566	0.01458974	0.0435111	834.270209
230.897534	-1.8686075	0.14537883	-12.853367	8.23E-38	7.00E-36	320.374575
701.114865	-0.6932638	0.07829424	-8.8545948	8.40E-19	2.56E-17	835.196147
377.705117	0.27501943	0.10800385	2.5463854	0.01088449	0.03382971	307.411442
1621.55934	-0.1781936	0.05533337	-3.2203645	0.00128028	0.00521609	1705.57794
1329.81048	0.41757053	0.0621009	6.72406628	1.77E-11	2.70E-10	1154.64479
756.509056	0.55499376	0.07362234	7.53838777	4.76E-14	9.65E-13	635.193522
8034.30754	0.21276393	0.03322337	6.40404364	1.51E-10	2.05E-09	7301.02175
6707.19864	0.12381019	0.03628461	3.41219548	0.00064442	0.0028391	6330.63865
2737.63132	-0.2275969	0.04437	-5.1295225	2.90E-07	2.46E-06	2954.66841
2244.35698	0.15272026	0.0587749	2.59839238	0.00936614	0.02974486	2100.02756
2387.33393	-0.3356378	0.0449733	-7.463046	8.45E-14	1.66E-12	2672.2573
2820.29176	0.21078529	0.04501983	4.68205423	2.84E-06	2.04E-05	2597.25631
308.119779	-0.3391354	0.11017511	-3.0781488	0.00208291	0.00801496	340.745213
2992.16895	0.19388641	0.04339113	4.4683417	7.88E-06	5.24E-05	2730.5914
4482.16864	0.46224672	0.03647619	12.6725603	8.39E-37	6.78E-35	3748.19734
11351.0845	-0.201329	0.0308255	-6.5312499	6.52E-11	9.23E-10	12009.4169
172.660849	-0.7606319	0.15361872	-4.9514273	7.37E-07	5.84E-06	223.151077
1911.19707	0.17869284	0.05251897	3.40244341	0.00066786	0.00292974	1694.46669
6617.07264	0.14351198	0.03690732	3.88844222	0.00010089	0.00053276	6166.74761
3246.80585	-0.1329821	0.04678298	-2.8425323	0.00447567	0.01571346	3342.63647
2067.27775	0.26276006	0.05213974	5.03953519	4.67E-07	3.82E-06	1774.09736
1502.95575	0.14641163	0.05694764	2.57098698	0.01014091	0.03184539	1410.20369
2866.11097	0.12312192	0.04924164	2.50036184	0.01240665	0.03789835	2570.40411
39.7429231	-2.7862114	0.3754548	-7.420897	1.16E-13	2.26E-12	71.2972321
7786.25581	0.16028498	0.04183833	3.83105608	0.00012759	0.00066064	7170.46448
6888.81317	-0.3704117	0.03821268	-9.6934257	3.22E-22	1.26E-20	7657.50791
231.914903	-0.8257777	0.1322004	-6.2464084	4.20E-10	5.35E-09	276.855486
1040.08851	0.21921498	0.0643148	3.40846897	0.00065329	0.00287437	981.494364
991.670257	-0.2320809	0.06515994	-3.5617114	0.00036845	0.00172228	1040.7544
6669.49797	-0.2740999	0.0337886	-8.1121993	4.97E-16	1.21E-14	7444.54216
11915.5753	-0.290767	0.033309	-8.7293805	2.56E-18	7.56E-17	13051.0972
454.856304	-0.5561688	0.10618467	-5.2377501	1.63E-07	1.44E-06	481.487801
5908.35275	-1.0379152	0.03742986	-27.729608	3.07E-169	3.97E-166	7940.84497
1698.61361	0.20019675	0.05322611	3.76125064	0.00016907	0.00085303	1546.31659

1598.55147	-0.1890223	0.05596336	-3.3776072	0.00073119	0.00316813	1733.35608
5846.87183	0.18162498	0.03424207	5.30414762	1.13E-07	1.02E-06	5373.21867
9540.04568	0.12966741	0.03170074	4.09035964	4.31E-05	0.00024499	9306.60363
2695.01765	0.22395642	0.04234961	5.28827548	1.23E-07	1.11E-06	2447.25434
1458.95207	-0.159052	0.05582719	-2.8490062	0.0043856	0.01544883	1548.16847
2956.76525	0.42787091	0.04919031	8.69827657	3.37E-18	9.90E-17	2420.40214
1367.56283	0.32971314	0.05674151	5.81079291	6.22E-09	6.73E-08	1190.75637
17375.0174	0.33669926	0.03531552	9.5340318	1.51E-21	5.74E-20	15002.0488
3837.53025	0.12627038	0.04302184	2.93502988	0.00333516	0.01214122	3694.49294
2139.50856	-0.1837867	0.05889043	-3.1208237	0.00180346	0.00707407	2455.58779
2538.86842	0.11151934	0.04633824	2.40663757	0.01610014	0.04741738	2350.95678
1290.1982	0.29513539	0.05870373	5.02754095	4.97E-07	4.04E-06	1110.19976
4004.53609	0.15953498	0.03848768	4.14509198	3.40E-05	0.00019833	3714.86357
2040.31395	0.18933656	0.05216562	3.62952765	0.00028394	0.0013631	1876.87649
2811.9739	0.24086452	0.04555604	5.28721383	1.24E-07	1.12E-06	2609.29351
4864.85863	-0.1743855	0.04148044	-4.2040402	2.62E-05	0.00015663	5303.77332
4095.96787	0.43900618	0.0415468	10.5665463	4.26E-26	2.11E-24	3309.3027
1492.9861	0.34159728	0.05592588	6.10803601	1.01E-09	1.22E-08	1257.42391
6531.22675	0.11208974	0.04171564	2.68699589	0.00720978	0.02367237	5876.00305
208.761981	-0.6484148	0.14696123	-4.4121488	1.02E-05	6.64E-05	217.595449
444.884953	-0.6238044	0.09546219	-6.5345701	6.38E-11	9.03E-10	567.600043
245.046251	-1.2500251	0.13072194	-9.5624741	1.15E-21	4.37E-20	361.115851
337.225076	-0.5843537	0.10826663	-5.3973573	6.76E-08	6.36E-07	402.783064
1668.25177	0.2960675	0.05962096	4.96582919	6.84E-07	5.45E-06	1439.83371
862.208346	-2.3406392	0.08417131	-27.80804	3.47E-170	4.80E-167	1346.31397
48072.9474	0.1738849	0.02844384	6.11327104	9.76E-10	1.18E-08	45378.3734
13778.4324	0.18084242	0.03640827	4.96706934	6.80E-07	5.42E-06	13164.0617
787.699544	-0.2077509	0.07297056	-2.8470512	0.00441263	0.01551392	844.455528
3107.9277	0.21498913	0.04337131	4.95694318	7.16E-07	5.68E-06	2849.11147
15609.1406	0.13280654	0.02723084	4.87706288	1.08E-06	8.34E-06	14703.8967
889.453776	-0.3346014	0.07000921	-4.7793918	1.76E-06	1.31E-05	967.605293
13781.555	0.35515499	0.03009519	11.8010542	3.85E-32	2.59E-30	12007.565
3508.57997	0.30965585	0.03857968	8.02639715	1.00E-15	2.37E-14	3095.411
1078.85523	-0.2621954	0.06469675	-4.0526832	5.06E-05	0.00028335	1234.27546
409.210641	-0.4196298	0.09789262	-4.286634	1.81E-05	0.00011238	470.376544
13637.8796	0.12605109	0.02861486	4.40509264	1.06E-05	6.85E-05	12768.6861
1571.36113	-0.2862186	0.05978799	-4.7872252	1.69E-06	1.27E-05	1694.46669
643.74573	0.34833058	0.08171014	4.263003	2.02E-05	0.0001235	561.118476
452.860459	0.25865292	0.09571909	2.70220823	0.00688806	0.02277807	425.005578
43.4371233	-1.655289	0.32579595	-5.0807537	3.76E-07	3.13E-06	66.6675417
6029.26634	0.22012763	0.03555424	6.19131851	5.97E-10	7.44E-09	5391.73743
13796.0188	0.22109949	0.03108763	7.11213708	1.14E-12	2.00E-11	12501.09
3609.32873	0.10920095	0.0417868	2.6132883	0.00896756	0.02860089	3338.00678

13977.4261	0.18893987	0.02759174	6.84769703	7.50E-12	1.19E-10	13041.8378
2630.11245	0.28693201	0.04347267	6.60028544	4.10E-11	5.94E-10	2348.17897
1244.01823	-0.4206886	0.06236147	-6.7459694	1.52E-11	2.33E-10	1375.94399
3442.73119	0.3409236	0.04559696	7.47689402	7.61E-14	1.51E-12	2995.40969
9903.80941	0.33709492	0.03161007	10.6641607	1.50E-26	7.62E-25	8797.33769
4291.5562	0.12942408	0.03621862	3.57341314	0.00035236	0.00165627	4122.27633
1765.52389	0.22500945	0.05253728	4.28285287	1.85E-05	0.00011402	1610.20632
1269.30029	0.19917043	0.06124336	3.25211494	0.0011455	0.00472758	1233.34952
2670.70619	0.27974124	0.0515657	5.42494839	5.80E-08	5.51E-07	2418.55026
6827.94883	0.11349355	0.0380817	2.98026462	0.00288	0.0106747	6476.93686
8424.442	0.34126249	0.03682147	9.26802918	1.90E-20	6.54E-19	7001.01782
13279.6017	0.22857244	0.02828796	8.08020384	6.47E-16	1.55E-14	12247.383
7180.24536	0.09783915	0.03469156	2.82025815	0.0047985	0.01667904	6997.31406
17192.42	-0.1523419	0.0326658	-4.6636498	3.11E-06	2.22E-05	17604.8607
95.0532397	-1.7487708	0.22291549	-7.8449947	4.33E-15	9.60E-14	148.150093
5925.78594	-0.293821	0.03456687	-8.5000763	1.89E-17	5.27E-16	6534.34502
60.6049892	-1.41192	0.26334714	-5.3614406	8.26E-08	7.65E-07	94.4456841
5782.60617	0.18579165	0.03277754	5.66826053	1.44E-08	1.49E-07	5404.70057
776.433371	-0.3259886	0.07249429	-4.496749	6.90E-06	4.64E-05	877.789299
612.714192	0.26159963	0.07952593	3.28948869	0.0010037	0.00420412	540.747838
2971.16524	0.36090698	0.04211655	8.56924356	1.04E-17	2.95E-16	2608.36757
5290.29681	0.20660896	0.0347023	5.95375382	2.62E-09	2.96E-08	4968.58373
16284.4304	0.0828	0.02823421	2.93261255	0.00336123	0.01222925	15865.949
3739.25337	0.17224465	0.04242694	4.05979471	4.91E-05	0.00027526	3424.11902
1815.96371	0.29198976	0.05256216	5.55513212	2.77E-08	2.74E-07	1635.20665
4382.89011	0.09714258	0.03534386	2.74849921	0.00598688	0.02018008	4248.20391
365.565722	0.25329905	0.1011227	2.50486835	0.0122497	0.03750164	343.523027
307.705046	-0.4120577	0.1108289	-3.7179621	0.00020084	0.00099698	358.338037
67.5239881	-1.5972106	0.26658819	-5.9913027	2.08E-09	2.39E-08	118.520074
1589.18842	0.20579678	0.05303617	3.88031028	0.00010432	0.00054894	1452.79685
4881.95508	0.30088702	0.04264028	7.05640408	1.71E-12	2.93E-11	4316.72333
1347.12818	0.85491589	0.05810587	14.7130736	5.31E-49	6.73E-47	936.123398
6028.67455	-0.2893228	0.03242488	-8.922864	4.54E-19	1.42E-17	6700.08794
602.828225	-0.2601438	0.08500213	-3.0604393	0.00221013	0.00845702	675.00886
21064.1992	0.1321073	0.0343741	3.84322157	0.00012143	0.00063159	19463.2184
3774.62991	0.28972471	0.04087041	7.08886173	1.35E-12	2.35E-11	3399.11869
884.736079	0.16404918	0.06810587	2.40873756	0.01600781	0.04718846	822.233014
1613.54419	0.42488687	0.06130052	6.93121113	4.17E-12	6.80E-11	1316.68395
22525.64	0.23892003	0.0310641	7.69119347	1.46E-14	3.08E-13	20422.4903
19793.6791	0.29862297	0.03170949	9.41746376	4.62E-21	1.69E-19	17669.6764
555.505924	0.53989837	0.08556194	6.31002971	2.79E-10	3.63E-09	479.635925
26.480162	-3.4077783	0.51812034	-6.5771945	4.79E-11	6.88E-10	40.7412755
932.439987	-0.8867062	0.06765672	-13.10596	3.04E-39	2.76E-37	1241.68296

10443.9443	-0.735691	0.03160635	-23.276685	7.64E-120	5.29E-117	12750.1674
125.584449	-1.0528134	0.18540018	-5.6785996	1.36E-08	1.40E-07	155.557597
2359.29911	-0.4018544	0.04802893	-8.3669247	5.91E-17	1.54E-15	2564.84848
375.207025	-0.3713417	0.1099953	-3.3759776	0.00073554	0.00318269	425.005578
3017.41249	0.28943529	0.04383682	6.60256064	4.04E-11	5.85E-10	2602.81194
25716.995	0.11004817	0.02442055	4.50637664	6.59E-06	4.46E-05	24839.2149
16.1035557	-1.4277551	0.52016226	-2.7448264	0.00605429	0.02037542	25.0003281
87.7226874	-0.8336104	0.21173789	-3.936992	8.25E-05	0.00044429	107.408817
214.803759	-0.4165322	0.13282865	-3.1358612	0.0017135	0.00675539	257.410786
2036.1573	0.12837392	0.04764415	2.69443184	0.00705088	0.02321746	1903.72869
8987.58759	-0.1853808	0.03336814	-5.5556236	2.77E-08	2.73E-07	9613.08914
8173.99372	-0.1306133	0.03423137	-3.8156014	0.00013585	0.00069984	8526.96377
6721.8758	-0.2397613	0.03786458	-6.3320745	2.42E-10	3.18E-09	7431.57902
5348.31558	-0.2481022	0.03901603	-6.3589813	2.03E-10	2.70E-09	5923.22589
51.7847944	-0.8350773	0.27989297	-2.9835595	0.00284917	0.01057864	69.4453559
8461.11077	-0.2310346	0.03281891	-7.0396793	1.93E-12	3.28E-11	9036.22971
7474.77618	-0.1514722	0.03337692	-4.5382325	5.67E-06	3.87E-05	7790.843
19111.6698	-0.0737235	0.02786143	-2.646077	0.00814313	0.02628281	19395.6249
7019.21422	-0.2118702	0.0365552	-5.7958975	6.80E-09	7.33E-08	7527.87658
6239.54042	-0.1778733	0.03351121	-5.3078743	1.11E-07	1.01E-06	6477.8628
4203.77269	-0.1706747	0.03779	-4.5163998	6.29E-06	4.26E-05	4476.91061
13914.4063	-0.149058	0.02909978	-5.1223071	3.02E-07	2.54E-06	14576.1172
2930.1794	-0.2128316	0.04354396	-4.8877409	1.02E-06	7.92E-06	3065.78098
2113.40944	-0.286115	0.0492741	-5.8066006	6.38E-09	6.89E-08	2374.10524
55174.4281	-0.2749652	0.02875047	-9.5638519	1.13E-21	4.32E-20	60085.0479
231.923071	-0.9980091	0.13625134	-7.3247657	2.39E-13	4.51E-12	325.930204
6155.90377	-0.1032889	0.03254123	-3.1740941	0.00150305	0.00601786	6399.15806
6565.77922	0.22594833	0.033347	6.77567126	1.24E-11	1.92E-10	5969.5228
880.569896	0.46055004	0.06936159	6.63984287	3.14E-11	4.60E-10	756.491411
16969.841	0.15788544	0.03270078	4.82818532	1.38E-06	1.05E-05	15539.0928
6618.42687	0.22367142	0.03516663	6.36033133	2.01E-10	2.68E-09	5854.70647
45.5339383	1.02404303	0.29851643	3.43044104	0.0006026	0.00267371	35.185647
2040.71713	1.46863718	0.0499425	29.4065627	4.53E-190	1.10E-186	1057.42129
2191.96127	-0.477559	0.05258222	-9.0821388	1.06E-19	3.48E-18	2642.62728
361.705606	0.30028187	0.10263401	2.92575418	0.00343622	0.01245768	308.33738
557.764743	-0.5283913	0.08765014	-6.0284141	1.66E-09	1.93E-08	703.71294
2979.26983	0.22123354	0.04332212	5.10671115	3.28E-07	2.75E-06	2709.29482
5976.648	0.1181193	0.04124469	2.86386651	0.00418504	0.01479881	5867.66961
322.371718	-0.3379831	0.11720942	-2.8835829	0.00393179	0.01402093	376.856798
19474.7168	0.19713969	0.02594556	7.59820537	3.00E-14	6.19E-13	18133.5713
9427.10244	0.1231991	0.03059594	4.02664894	5.66E-05	0.00031426	9001.04407
3518.6629	0.18683273	0.04834068	3.86491745	0.00011113	0.00058159	3118.55945
2935.25718	0.35898315	0.04198427	8.5504211	1.23E-17	3.45E-16	2542.62597

25812.4813	0.17573806	0.03400341	5.16824898	2.36E-07	2.03E-06	23849.3871
41.1887376	-0.9981101	0.34367205	-2.9042515	0.00368132	0.01323733	36.1115851
2569.01649	-0.397592	0.04521756	-8.7928662	1.46E-18	4.38E-17	2945.40903
38.7170268	-1.3529956	0.3221985	-4.1992609	2.68E-05	0.00015973	59.2600371
1794.1774	0.24635919	0.05411014	4.5529208	5.29E-06	3.62E-05	1618.53976
5380.11131	0.26935478	0.03440154	7.82973078	4.89E-15	1.08E-13	4914.87932
9344.56804	0.13269164	0.03287402	4.03636766	5.43E-05	0.00030239	8768.63361
2477.17773	0.25191691	0.04771103	5.28005557	1.29E-07	1.16E-06	2352.80866
3394.22485	0.11069134	0.04225874	2.61937167	0.00880919	0.02815136	3288.93206
7323.77415	0.10915036	0.03558696	3.067145	0.00216114	0.0082843	6952.86904
49.309881	-0.9755253	0.28732722	-3.3951718	0.00068586	0.00299782	62.0378513
7684.00471	0.14613253	0.03092494	4.7253939	2.30E-06	1.68E-05	7257.50266
1158.47441	-0.4682037	0.06004808	-7.7971477	6.33E-15	1.39E-13	1316.68395
3851.39073	0.16846731	0.04070235	4.13900732	3.49E-05	0.00020299	3587.08412
6445.85804	-0.5181812	0.03418564	-15.157861	6.72E-52	9.80E-50	7599.17382
1989.5296	0.13024151	0.04778433	2.72561134	0.00641825	0.02144396	1945.3959
3865.96374	0.30896975	0.03693045	8.36626125	5.95E-17	1.54E-15	3465.78623
650.454018	-0.5482245	0.07842689	-6.9902615	2.74E-12	4.59E-11	756.491411
590.132039	-0.3447087	0.08171741	-4.2183015	2.46E-05	0.00014791	680.564488
1743.74468	-0.3557203	0.05184409	-6.8613472	6.82E-12	1.09E-10	1916.69182
1576.47588	-0.2524435	0.05727827	-4.4073174	1.05E-05	6.78E-05	1699.09638
4710.18816	0.18408628	0.04375935	4.20678738	2.59E-05	0.00015502	4496.35531
11823.4069	0.16751395	0.02897964	5.78040106	7.45E-09	7.98E-08	11157.5539
548.133077	-0.3213968	0.08627341	-3.7253284	0.00019506	0.00097155	628.711956
1371.67758	-0.2035689	0.05757578	-3.5356694	0.00040674	0.00188135	1450.94497
2732.9468	0.43370191	0.04205191	10.3134884	6.12E-25	2.79E-23	2322.2527
3476.75855	0.13064299	0.03920655	3.33217229	0.00086171	0.00367207	3321.33989
512.84757	0.38540265	0.08721283	4.41910475	9.91E-06	6.45E-05	428.709331
7723.72749	0.16324877	0.03064383	5.32729751	9.97E-08	9.13E-07	7199.16857
101.141098	-0.6495878	0.20132779	-3.2265185	0.00125306	0.00512568	106.482879
5755.99525	0.20577936	0.03379255	6.08948833	1.13E-09	1.36E-08	5296.36581
2043.62704	0.18315182	0.05166671	3.54487079	0.00039281	0.00182384	1869.46898
290.981556	-0.6044313	0.11762961	-5.1384278	2.77E-07	2.35E-06	370.375232
598.92353	-0.3304339	0.09153815	-3.609794	0.00030644	0.0014599	637.045398
1288.33171	-0.5331761	0.06100105	-8.7404415	2.32E-18	6.89E-17	1487.98249
6780.0099	0.31871448	0.03278497	9.72136102	2.44E-22	9.63E-21	5963.04123
21314.3013	0.21758572	0.02771427	7.851035	4.13E-15	9.18E-14	19400.2546
168.990823	-0.4821589	0.1480159	-3.2574806	0.00112406	0.00465099	197.224811
565.806984	0.36403977	0.08439161	4.31369602	1.61E-05	0.00010039	495.376872
1095.59711	0.29650501	0.06377096	4.64953022	3.33E-06	2.37E-05	950.938407
1344.40765	-0.4162007	0.05715112	-7.2824581	3.28E-13	6.09E-12	1513.90876
519.431828	0.60726078	0.09082821	6.68581657	2.30E-11	3.45E-10	391.671807
3689.86474	0.124854	0.04211866	2.96433909	0.00303334	0.01117895	3575.04692

1432.97265	-0.1917968	0.05753954	-3.333304	0.00085821	0.00365957	1459.27841
5838.80791	0.12701335	0.03543804	3.58409621	0.00033825	0.00159458	5580.6288
25989.6661	0.21820178	0.02899955	7.52431715	5.30E-14	1.07E-12	24108.6498
4403.31037	0.12426183	0.03853303	3.22481352	0.00126055	0.00515195	4136.1654
6372.97421	0.20148261	0.03502729	5.75216083	8.81E-09	9.34E-08	5887.11431
846.318555	-0.4227293	0.07051376	-5.9949907	2.03E-09	2.34E-08	931.493708
4157.46721	0.3310397	0.03864755	8.56560651	1.08E-17	3.04E-16	3551.89847
1770.49612	0.52055364	0.05222437	9.96763847	2.11E-23	8.82E-22	1477.79717
1151.46956	-0.5893477	0.06275497	-9.3912518	5.93E-21	2.15E-19	1362.05491
67.1700678	0.74978486	0.24123451	3.10811611	0.00188284	0.00734679	45.3709659
106.529296	0.67124698	0.18741706	3.58156816	0.00034154	0.00160931	85.1863033
3248.5844	1.12472982	0.04351373	25.8477004	2.58E-147	2.78E-144	2016.69314
4049.82613	0.16062784	0.04120368	3.89838559	9.68E-05	0.00051359	3816.71676
2994.6108	0.28656415	0.04053405	7.06971425	1.55E-12	2.68E-11	2708.36888
6502.10812	-0.100119	0.03230869	-3.0988255	0.00194289	0.00756285	6750.0886
2216.0165	0.1370311	0.04608239	2.97361085	0.00294318	0.01089005	2115.76851
3453.44865	-0.1369063	0.04178838	-3.2761818	0.00105221	0.00438364	3533.37971
1112.34728	-0.3066218	0.06111207	-5.0173689	5.24E-07	4.25E-06	1215.7567
5567.36781	0.48599375	0.03848825	12.6270671	1.50E-36	1.18E-34	4504.68876
4815.85384	0.19048836	0.03469622	5.49017632	4.02E-08	3.89E-07	4504.68876
13622.7751	0.13480808	0.0279098	4.83013469	1.36E-06	1.04E-05	12871.4652
3134.79489	0.12412436	0.04453036	2.78740963	0.00531313	0.01820342	3020.41001
5486.03303	-0.4223268	0.03755371	-11.245943	2.42E-29	1.48E-27	6359.34273
4580.4718	0.20091814	0.04813874	4.17373104	3.00E-05	0.00017672	3987.08937
12224.4343	-0.069143	0.02862151	-2.415771	0.01570193	0.04639262	12273.3092
1766.41663	-0.1804788	0.05337727	-3.3811916	0.00072172	0.00313409	1893.54337
3224.79058	-0.1612521	0.0396807	-4.0637412	4.83E-05	0.00027095	3433.3784
1965.62883	-0.1717829	0.04892916	-3.510848	0.00044668	0.00204348	2037.06377
753.5707	0.45136503	0.07641792	5.9065341	3.49E-09	3.88E-08	687.971993
13741.3765	0.12779945	0.02785715	4.5876709	4.48E-06	3.11E-05	13321.4711
2167.52419	-0.260936	0.05160269	-5.0566347	4.27E-07	3.51E-06	2349.10491
200.055612	0.57278606	0.13659235	4.19339781	2.75E-05	0.00016366	151.853845
15016.5047	-0.413731	0.02815201	-14.69632	6.81E-49	8.51E-47	17289.1158
269.053561	-1.1990354	0.13973764	-8.5806184	9.44E-18	2.68E-16	370.375232
7787.20654	0.14936185	0.03370811	4.43103571	9.38E-06	6.13E-05	7297.318
4115.53506	0.34536456	0.03831058	9.01486172	1.97E-19	6.35E-18	3603.751
6296.57827	-0.3990584	0.03483391	-11.456032	2.19E-30	1.40E-28	6976.94343
4931.39818	0.3283781	0.04172357	7.87032605	3.54E-15	7.95E-14	4307.46394
1335.0108	-0.231325	0.0580926	-3.9820039	6.83E-05	0.00037358	1397.24056
6929.83186	0.09684285	0.03343925	2.89608299	0.00377853	0.01353663	6686.19887
1387.83239	0.29944256	0.05765036	5.19411409	2.06E-07	1.79E-06	1227.79389
4350.20383	0.32732499	0.03747601	8.734255	2.45E-18	7.26E-17	3752.82703
1730.37798	0.17803464	0.0513607	3.46635965	0.00052756	0.00237003	1602.79882

368.391335	0.63426599	0.10474852	6.05513116	1.40E-09	1.65E-08	283.337052
6129.52189	-0.2569372	0.03351334	-7.6667128	1.76E-14	3.71E-13	6626.93883
36.7599344	-1.2246833	0.336757	-3.6366973	0.00027616	0.00133001	41.6672136
105.289317	-1.0707614	0.1922177	-5.5705662	2.54E-08	2.53E-07	133.335083
314.953256	-1.2071847	0.11860716	-10.178008	2.49E-24	1.10E-22	420.375888
1667.33386	0.24519724	0.0536693	4.56866869	4.91E-06	3.37E-05	1450.01903
72.1954763	0.66962703	0.24810227	2.69899598	0.0069549	0.02295994	52.7784705
1808.64844	0.20351425	0.05390262	3.7755915	0.00015963	0.00080963	1708.35576
113.677757	-1.0548981	0.18975754	-5.5591892	2.71E-08	2.68E-07	153.705721
218.937177	-0.605943	0.13227542	-4.5809195	4.63E-06	3.20E-05	264.818291
3141.79905	0.32442413	0.04198113	7.72785629	1.09E-14	2.35E-13	2700.03544
3026.70634	-0.477883	0.04387989	-10.890705	1.28E-27	7.09E-26	3456.52685
4536.23284	-0.2846551	0.03830492	-7.4312957	1.08E-13	2.09E-12	4814.87801
2108.16315	0.7183029	0.04846401	14.8213664	1.07E-49	1.42E-47	1624.09539
671.22016	-0.2117364	0.07601786	-2.7853508	0.00534698	0.01829678	721.305764
3354.15187	-0.3753081	0.04365743	-8.5966605	8.21E-18	2.33E-16	3672.27042
503.336185	0.54772103	0.08845646	6.19198432	5.94E-10	7.42E-09	425.931516
267.419365	-0.7910829	0.12308451	-6.4271522	1.30E-10	1.78E-09	346.300842
558.95315	0.51092425	0.08422098	6.06647245	1.31E-09	1.55E-08	455.561535
2138.91373	-0.214039	0.04904956	-4.363729	1.28E-05	8.13E-05	2362.06804
2061.56551	0.12506634	0.05062955	2.47022425	0.01350284	0.04070168	1921.32151
344.233293	0.64394758	0.10766623	5.98096145	2.22E-09	2.54E-08	252.781096
3839.3598	0.15563321	0.04090575	3.80467842	0.00014199	0.00072816	3607.45476
9429.38518	-0.1349986	0.03120294	-4.3264718	1.52E-05	9.51E-05	9891.7965
54.6606601	-0.9600699	0.27870169	-3.444794	0.0005715	0.00255087	66.6675417
3338.25353	0.10908025	0.04408247	2.47445846	0.01334383	0.040329	3243.56109
2984.36555	-0.5771522	0.04327843	-13.335794	1.43E-40	1.38E-38	3591.71381
8587.4261	0.18886106	0.03106456	6.07963153	1.20E-09	1.43E-08	7958.43779
1531.60337	0.36810149	0.05582232	6.5941635	4.28E-11	6.17E-10	1308.35051
1522.67909	0.16240611	0.05608084	2.89592845	0.00378039	0.01353926	1441.68559
5096.05251	0.15859353	0.0346014	4.58344241	4.57E-06	3.17E-05	4784.32205
293.303007	-0.6504414	0.12489429	-5.2079352	1.91E-07	1.67E-06	324.078328
1863.54276	0.17828481	0.05182889	3.43987312	0.00058199	0.00259591	1815.76457
121.851624	-2.2480281	0.20379492	-11.030835	2.71E-28	1.55E-26	194.446997
5500.06231	0.10139122	0.03669598	2.76300579	0.00572718	0.01940592	5174.14199
404.66492	-0.634224	0.09842973	-6.4434185	1.17E-10	1.61E-09	479.635925
1375.94098	-0.2605165	0.05956975	-4.3733024	1.22E-05	7.82E-05	1450.01903
55.453356	-0.8837093	0.26460564	-3.339722	0.00083862	0.00358314	75.9269225
2634.88495	0.18486879	0.04332743	4.26678426	1.98E-05	0.00012165	2458.3656
2983.61557	0.27033151	0.05299579	5.10100001	3.38E-07	2.83E-06	2537.99628
1957.98785	0.20910975	0.05001377	4.18104322	2.90E-05	0.00017207	1861.13554
5363.02613	0.11325802	0.03659164	3.09518841	0.00196688	0.00764394	5116.73383
2602.8524	0.39412291	0.04375436	9.00762569	2.11E-19	6.77E-18	2295.4005

3843.24868	0.17292346	0.03782396	4.57179685	4.84E-06	3.33E-05	3580.60255
4971.10296	0.35037741	0.04124264	8.49551455	1.97E-17	5.46E-16	4239.87046
170.521219	-0.7718404	0.15887192	-4.8582559	1.18E-06	9.13E-06	228.706706
44.3166815	-0.7383146	0.29421213	-2.5094634	0.01209147	0.03706314	63.8897275
13.3573434	-2.0405714	0.58702329	-3.4761336	0.0005087	0.00229329	18.5187616
39.0296517	-0.9341907	0.3159336	-2.9569211	0.00310728	0.01141454	47.222842
7.42547136	-3.7594621	1.04749826	-3.5889912	0.00033196	0.00157029	13.8890712
163.466252	-0.4285893	0.15294607	-2.8022248	0.00507515	0.01750565	194.446997
580.64695	-0.262188	0.10474061	-2.5032123	0.01230717	0.03764191	718.527949
115.795605	-1.0908362	0.19512311	-5.5905021	2.26E-08	2.27E-07	140.742588
14.2945316	-2.2941167	0.60861769	-3.7693888	0.00016365	0.00082806	27.7781424
74.1593811	-0.8959634	0.22716352	-3.9441339	8.01E-05	0.00043198	98.1494364
618.432401	-1.0799734	0.08451451	-12.778556	2.16E-37	1.79E-35	869.455856
43.459775	-1.2843905	0.31540762	-4.0721607	4.66E-05	0.00026279	48.1487801
159.251495	0.98677141	0.159727	6.17786221	6.50E-10	8.06E-09	124.075703
27.2364355	-1.3053394	0.38499002	-3.39058	0.00069745	0.00304026	45.3709659
17.9039754	-2.1333905	0.51503291	-4.1422411	3.44E-05	0.00020039	25.0003281
172.891084	-2.0685095	0.16483476	-12.548988	4.03E-36	3.17E-34	281.485176
147.517034	-0.4111635	0.16584932	-2.4791389	0.01317	0.03987201	169.446669
6124.87721	0.15987846	0.0328132	4.87238202	1.10E-06	8.53E-06	5816.74301
619.493019	-0.4795076	0.09583164	-5.0036463	5.63E-07	4.54E-06	816.677386
10.9928642	-1.9881419	0.6837241	-2.9078131	0.00363966	0.0131094	13.8890712
14.0329527	-2.1492481	0.57635296	-3.7290485	0.0001922	0.00095929	21.2965758
474.132341	-0.2879086	0.09031901	-3.1876853	0.00143417	0.0057715	515.74751
121.821592	0.8588445	0.20487269	4.19208867	2.76E-05	0.00016441	110.186631
388.225721	0.38267352	0.10211794	3.74736824	0.0001787	0.00089697	361.115851
510.34901	-0.3600246	0.09249037	-3.8925628	9.92E-05	0.00052507	612.04507
645.377387	-0.3810185	0.0791881	-4.8115631	1.50E-06	1.13E-05	742.602339
5091.00622	0.20622036	0.03765918	5.47596515	4.35E-08	4.20E-07	4627.83852
103.632688	-1.3027556	0.20726415	-6.2854842	3.27E-10	4.21E-09	127.779455
1443.13537	0.28551532	0.06252441	4.56646154	4.96E-06	3.41E-05	1322.23958
173.128353	-0.633635	0.16071286	-3.9426526	8.06E-05	0.00043453	252.781096
99.6470676	-0.5703491	0.21180146	-2.692848	0.00708446	0.02331616	149.076031
83.3003045	-0.9471675	0.23593959	-4.0144491	5.96E-05	0.00032945	111.11257
522.677437	-0.679788	0.08783488	-7.7393852	9.99E-15	2.15E-13	667.601355
62.7071304	-0.7456248	0.24847793	-3.0007689	0.00269299	0.01007208	83.3344271
1359.42681	-0.5843122	0.0599218	-9.7512448	1.82E-22	7.24E-21	1705.57794
181.646393	-1.2338637	0.14816619	-8.3275663	8.25E-17	2.11E-15	249.077343
1155.78634	-0.8749628	0.06816182	-12.836552	1.02E-37	8.62E-36	1389.83306
146.405006	-0.7504449	0.16851591	-4.4532585	8.46E-06	5.58E-05	195.372935
58.0324	0.82318732	0.26033127	3.16207624	0.00156649	0.00624821	50.9265944
535.784013	-0.3066217	0.08423968	-3.6398722	0.00027277	0.00131535	590.748494
2031.97951	0.26155199	0.05117157	5.11127593	3.20E-07	2.69E-06	1899.099

18.7455005	-2.9392041	0.56352622	-5.2157362	1.83E-07	1.61E-06	37.0375232
57.1268634	-1.0103636	0.2800365	-3.6079711	0.0003086	0.00146839	70.371294
249.044266	-0.4720503	0.12359451	-3.8193466	0.00013381	0.00069021	294.448309
349.209317	-0.5637519	0.10824913	-5.2079115	1.91E-07	1.67E-06	403.709003
101.005152	-0.8152026	0.20734413	-3.9316404	8.44E-05	0.00045317	113.890384
36.4079118	-0.8252898	0.3230282	-2.5548538	0.01062324	0.03313993	50.0006563
1822.07776	0.25693068	0.04968499	5.17119332	2.33E-07	2.00E-06	1633.35477
134.309464	-0.6738258	0.18242148	-3.6937852	0.00022094	0.00108787	186.113554
16.2758629	-1.7145622	0.55278522	-3.1016787	0.00192427	0.00749637	25.0003281
37.3391338	-0.8232164	0.33470178	-2.4595521	0.01391105	0.04175039	42.5931516
1208.69442	0.15546445	0.06452376	2.40941414	0.01597816	0.04711539	1127.79258
279.332351	-0.35955	0.1212048	-2.9664667	0.00301243	0.01111246	325.930204
325.390698	-0.5682378	0.10951169	-5.1888325	2.12E-07	1.83E-06	377.782736
348.640559	-0.5194416	0.11405621	-4.5542597	5.26E-06	3.60E-05	391.671807
127.173781	-0.6383394	0.17270128	-3.6962055	0.00021885	0.00107838	164.816978
216.501777	0.40535584	0.13109524	3.09207137	0.00198765	0.00771255	192.59512
192.580779	-0.6617713	0.14264567	-4.6392667	3.50E-06	2.48E-05	251.855158
324.90812	-0.624414	0.11303505	-5.524074	3.31E-08	3.23E-07	430.561207
428.53247	-0.7863205	0.09898434	-7.943888	1.96E-15	4.51E-14	573.155671
22.1131741	-1.5095888	0.45498244	-3.3179057	0.00090695	0.00383786	25.0003281
105.995545	-0.6673801	0.20457751	-3.2622357	0.00110537	0.00457856	151.853845
193.389797	-0.8639938	0.14270423	-6.0544375	1.41E-09	1.66E-08	269.447981
1700.99912	0.1345727	0.05032375	2.67413881	0.00749214	0.02447509	1626.87321
218.139838	-0.7378285	0.13898294	-5.30877	1.10E-07	1.00E-06	271.299857
332.012709	0.67415078	0.12132779	5.55644171	2.75E-08	2.72E-07	221.299201
1007.56529	0.19887803	0.06573984	3.02522824	0.00248446	0.00936988	925.938079
836.562184	-0.2062721	0.07414468	-2.7820216	0.00540214	0.01846598	927.789955
535.87424	-0.2322916	0.08632053	-2.6910352	0.00712307	0.02341541	589.822556
28912.6555	0.08684906	0.02629186	3.30326861	0.00095565	0.00402415	27590.1769
46.6956541	-1.7801779	0.31405483	-5.6683665	1.44E-08	1.49E-07	76.8528606
10.5464489	-2.3381963	0.68572342	-3.4098242	0.00065005	0.0028626	17.5928235
739.861068	-0.3029549	0.07666896	-3.9514673	7.77E-05	0.00042012	802.788315
818.632302	0.44027247	0.07155523	6.15290394	7.61E-10	9.35E-09	702.787002
9.96970719	-2.4054759	0.70909343	-3.3923258	0.00069302	0.003023	19.4446997
29.4622551	-1.172496	0.3625479	-3.2340443	0.00122051	0.0050073	38.8893993
513.696821	-0.2917398	0.09042407	-3.2263515	0.00125379	0.00512719	531.488457
249.270039	0.92488145	0.13111939	7.05373528	1.74E-12	2.98E-11	165.742916
5896.3482	0.17483008	0.03336351	5.2401581	1.60E-07	1.42E-06	5547.29503
44.8972224	-0.9965101	0.30445467	-3.2730985	0.00106375	0.00442129	67.5934798
396.041563	0.40801904	0.10093666	4.04232752	5.29E-05	0.00029531	334.263647
568.794076	-0.2784284	0.09004622	-3.0920613	0.00198772	0.00771255	562.970352
45.2272485	-0.7452718	0.29963708	-2.4872482	0.01287356	0.03909052	59.2600371
25.3877972	-1.3878167	0.4118464	-3.3697434	0.00075238	0.00324976	37.0375232

315.612559	0.42681923	0.12012955	3.55299127	0.00038088	0.00177526	266.670167
89.0701011	-0.8684553	0.2085515	-4.1642245	3.12E-05	0.00018357	124.075703
673.018077	-0.5435043	0.07963127	-6.8252628	8.78E-12	1.38E-10	779.639863
403.16684	-0.724924	0.1012088	-7.1626578	7.91E-13	1.41E-11	514.821572
4294.95629	0.20216364	0.03991308	5.06509729	4.08E-07	3.37E-06	3881.53243
212.585177	-0.5848883	0.13846854	-4.2239798	2.40E-05	0.00014446	274.077671
126.443849	-0.6118902	0.18362884	-3.332212	0.00086159	0.00367207	139.81665
246.82465	-0.6599893	0.12821365	-5.1475739	2.64E-07	2.25E-06	301.855814
31.2191565	-1.0059143	0.35275814	-2.8515695	0.0043504	0.01533596	45.3709659
9.46862873	-1.8613696	0.70172038	-2.6525803	0.00798791	0.0258723	17.5928235
278.903884	0.56558927	0.12027627	4.70241764	2.57E-06	1.87E-05	232.410458
960.079353	-0.307658	0.07551693	-4.0740269	4.62E-05	0.00026107	1010.19844
217.674149	-0.5292822	0.13154455	-4.0235965	5.73E-05	0.00031781	264.818291
587.080502	-0.5816335	0.0939926	-6.188078	6.09E-10	7.58E-09	762.047039
15.1940158	-1.4956745	0.54939515	-2.7224021	0.00648092	0.02161981	17.5928235
230.280746	-0.5301889	0.13573137	-3.9061635	9.38E-05	0.0004983	309.263318
401.296691	-0.588907	0.10046569	-5.8617724	4.58E-09	5.03E-08	477.784049
55.5093237	-1.7393125	0.28447617	-6.1140887	9.71E-10	1.18E-08	75.0009844
228.669631	-0.7012709	0.1332367	-5.2633466	1.41E-07	1.26E-06	292.596433
243.188282	-1.2510025	0.13264937	-9.430897	4.07E-21	1.50E-19	345.374904
743.077117	0.3943311	0.09746131	4.04602708	5.21E-05	0.00029102	529.636581
84.212491	-0.5642775	0.23455008	-2.4057868	0.01613768	0.04751351	111.11257
932.049568	-0.1797821	0.06962176	-2.5822693	0.0098153	0.03096835	939.82715
245.804808	0.40312605	0.13076668	3.08278865	0.00205071	0.00791466	233.336396
135.194785	-0.5689898	0.17094955	-3.3284074	0.00087344	0.00371552	152.779783
313.513812	-0.9575197	0.11411876	-8.390555	4.84E-17	1.29E-15	430.561207
12.282517	-1.7266954	0.58653935	-2.9438697	0.00324137	0.01184874	20.3706377
596.939502	0.90288437	0.08479929	10.64731	1.79E-26	9.01E-25	404.634941
36.2758771	1.08961741	0.33485053	3.25404113	0.00113776	0.00469965	19.4446997
304.809228	0.29242009	0.11403235	2.56436082	0.0103366	0.032376	260.1886
40.2175916	-1.2888951	0.32149279	-4.009095	6.10E-05	0.00033615	54.6303467
45.8264586	-2.0240846	0.32761947	-6.1781572	6.49E-10	8.05E-09	75.0009844
32.7484763	0.9117618	0.33831802	2.69498441	0.00703919	0.02318451	20.3706377
821.663104	0.2112348	0.0804885	2.62440972	0.00867993	0.02779327	742.602339
341.5515	-0.643552	0.10596633	-6.0731747	1.25E-09	1.49E-08	415.746198
117.772848	-0.5831874	0.18012994	-3.2375926	0.00120543	0.00495068	126.853517
954.742538	-1.3705688	0.06976122	-19.646571	6.19E-86	2.35E-83	1389.83306
469.80361	-0.4393591	0.09451369	-4.6486292	3.34E-06	2.38E-05	581.489114
32.0417436	0.88978031	0.33872857	2.62682395	0.00861859	0.02761511	23.148452
137.295557	-0.5059111	0.17079191	-2.9621489	0.003055	0.01124382	163.89104
3757.90986	-0.1873973	0.04965407	-3.7740565	0.00016061	0.00081399	4014.86751
1196.33997	-0.4772621	0.05905557	-8.0815761	6.39E-16	1.54E-14	1383.35149
71.8908787	0.63502289	0.22880495	2.77538966	0.00551356	0.01876741	49.0747182

2655.81672	-0.6016378	0.0471633	-12.756482	2.87E-37	2.36E-35	3225.96827
205.650615	-0.7625406	0.14747843	-5.170523	2.33E-07	2.01E-06	291.670495
891.169727	-0.5912522	0.07031153	-8.409036	4.13E-17	1.11E-15	1068.53254
573.582299	-0.6491977	0.08693126	-7.4679427	8.15E-14	1.61E-12	647.230717
160.922602	-0.388088	0.15689845	-2.4734982	0.01337975	0.04038718	197.224811
104.277751	-0.7597697	0.20734269	-3.6643185	0.000248	0.00120729	149.076031
553.380799	-1.0143871	0.09089866	-11.159539	6.43E-29	3.84E-27	761.121101
315.674129	0.37761875	0.11062003	3.41365631	0.00064097	0.00282585	289.818619
127.34529	-0.9190491	0.18292474	-5.0241924	5.06E-07	4.11E-06	163.89104
42.4165232	-0.8768155	0.32009664	-2.7392211	0.0061585	0.0206902	45.3709659
55.5430428	-1.1162528	0.26562268	-4.2024004	2.64E-05	0.00015767	73.1491083
46.9024924	-0.933501	0.29872988	-3.1249001	0.00177865	0.00698084	47.222842
55.3494092	-1.0052954	0.26782179	-3.7535982	0.00017431	0.00087723	85.1863033
595.534467	-0.669366	0.08825619	-7.584352	3.34E-14	6.86E-13	705.564816
246.732931	-0.373027	0.12375147	-3.0143237	0.00257553	0.00967019	274.077671
307.70891	0.30212817	0.11056414	2.73260547	0.00628356	0.02105925	287.040805
432.028077	-0.2370747	0.09643804	-2.4583108	0.01395923	0.04186906	438.89465
256.336463	0.54736913	0.13597141	4.02561923	5.68E-05	0.00031545	175.002297
152.171489	-0.8429463	0.17960348	-4.6933743	2.69E-06	1.94E-05	232.410458
33.591374	-1.1653469	0.34253304	-3.4021444	0.00066859	0.00293228	47.222842
6323.70765	0.22460656	0.04193586	5.35595412	8.51E-08	7.87E-07	5730.63077
77.4317646	-0.8595703	0.23061085	-3.7273627	0.00019349	0.00096445	112.038508
657.208471	0.8212932	0.08082581	10.1612738	2.95E-24	1.29E-22	438.89465
182.807389	-0.6054643	0.14640977	-4.1354091	3.54E-05	0.00020571	231.48452
364.479051	-0.2886376	0.10703107	-2.6967645	0.00700168	0.02309469	369.449294
114.452217	-0.5906707	0.20250189	-2.9168653	0.00353569	0.0127705	168.52073
47.0397574	-0.7973202	0.30212759	-2.6390181	0.00831466	0.02677402	57.4081609
1383.81276	-0.162783	0.06109842	-2.6642752	0.00771544	0.02510724	1362.05491
384.802604	-0.3507472	0.10065109	-3.4847834	0.00049254	0.00223132	447.228092
195.335975	-0.838102	0.15344406	-5.4619382	4.71E-08	4.52E-07	237.040148
149.857101	-0.9511477	0.16045678	-5.9277503	3.07E-09	3.44E-08	187.039492
38.4076185	-0.824483	0.31781321	-2.5942377	0.00948009	0.0300575	43.5190897
958.809649	-0.7795124	0.06896333	-11.303288	1.26E-29	7.88E-28	1218.53451
405.285612	-0.2995027	0.09930464	-3.015999	0.00256134	0.00962987	466.672792
36.7618824	-1.2550303	0.35495927	-3.5357024	0.00040669	0.00188135	50.0006563
181.184727	-0.52422	0.15104958	-3.470516	0.00051946	0.00233582	200.002625
59.0777187	-0.9687426	0.25609058	-3.7828123	0.00015507	0.00078855	83.3344271
96.8689018	-0.6437876	0.20414234	-3.1536213	0.00161258	0.00640178	100.001313
350.309823	-1.0901719	0.11167982	-9.7615841	1.65E-22	6.55E-21	463.894978
333.433002	-0.2946174	0.11574752	-2.545345	0.01091698	0.0339099	413.894321
831.801722	0.26317789	0.08066218	3.26271738	0.0011035	0.00457176	684.26824
25.5151203	-1.4495762	0.42561233	-3.4058603	0.00065956	0.0028979	43.5190897
63.6998594	-0.6621617	0.27754816	-2.3857544	0.01704413	0.04975161	85.1863033

240.998834	-0.314554	0.12860899	-2.4458165	0.01445245	0.0431563	276.855486
1709.15734	0.36197696	0.05254925	6.8883379	5.64E-12	9.07E-11	1425.0187
440.591558	0.2866014	0.09530527	3.00719353	0.00263672	0.00987687	383.338365
249.939375	-0.6998361	0.12785196	-5.4738001	4.40E-08	4.24E-07	331.485832
38.1304818	-1.222037	0.33289283	-3.6709623	0.00024164	0.0011787	48.1487801
18.8200093	-1.3112367	0.49522471	-2.647761	0.00810268	0.02618713	30.5559566
136.355209	-0.4947326	0.16530966	-2.9927628	0.00276465	0.01030232	161.113226
2142.16266	-0.2141585	0.04918796	-4.3538808	1.34E-05	8.48E-05	2254.65922
191.46969	-0.6935314	0.1497244	-4.6320533	3.62E-06	2.56E-05	213.891696
1487.86994	0.25280185	0.05414903	4.668631	3.03E-06	2.17E-05	1386.1293
53439.9092	0.18822438	0.03654876	5.14995306	2.61E-07	2.22E-06	51280.3027
71725.0203	0.14725709	0.03040689	4.84288561	1.28E-06	9.79E-06	67877.7428
42550.2874	0.14450113	0.02686902	5.37798296	7.53E-08	7.01E-07	40339.4184
12934.6977	0.17893469	0.03864128	4.63066108	3.65E-06	2.57E-05	12316.8283
61676.131	0.23808776	0.02543062	9.36224944	7.81E-21	2.79E-19	56343.3321
94390.1685	0.27774386	0.02412639	11.5120357	1.15E-30	7.36E-29	83502.9479
35996.0966	0.16846317	0.02702845	6.2328087	4.58E-10	5.80E-09	33367.1046
77392.1607	0.35503603	0.02595122	13.6809016	1.32E-42	1.36E-40	66416.6125
14503.2693	0.13598756	0.02877241	4.72631815	2.29E-06	1.68E-05	13655.7348
16513.6621	0.11741161	0.03210926	3.65662767	0.00025556	0.00123879	15221.4961
1773.19609	0.63260871	0.05152823	12.2769354	1.20E-34	9.02E-33	1379.64774
818.01194	0.22776253	0.07019183	3.24485806	0.00117509	0.00483531	744.454216
5901.23529	0.35570993	0.03332171	10.6750196	1.33E-26	6.80E-25	5190.80887
7633.70476	0.2640008	0.03323726	7.9429174	1.97E-15	4.54E-14	6903.79432
71791.144	0.28233208	0.02605598	10.8355964	2.33E-27	1.27E-25	64400.8453
5023.54667	0.27845989	0.04181917	6.65866557	2.76E-11	4.09E-10	4451.91028
94152.1798	0.22246274	0.03500981	6.35429764	2.09E-10	2.78E-09	89004.8719
32189.0118	0.18628445	0.0298648	6.2375926	4.44E-10	5.64E-09	29705.9455
60260.7706	0.23332645	0.02734547	8.53254563	1.43E-17	4.00E-16	54906.2762
30844.6607	0.28640509	0.0268893	10.6512663	1.72E-26	8.66E-25	27248.5058
60377.8085	0.18350642	0.03431594	5.3475556	8.92E-08	8.23E-07	57451.68
2333.8535	-0.4555397	0.04877589	-9.3394446	9.68E-21	3.43E-19	2742.62859
24834.4346	0.11754473	0.03529859	3.33001197	0.00086842	0.00369742	24432.7281
43592.5484	0.35153748	0.02715351	12.946302	2.47E-38	2.17E-36	38228.2795
448.720948	-0.7091292	0.09437751	-7.5137521	5.75E-14	1.16E-12	542.599714
5037.87023	0.12883123	0.03748549	3.43682921	0.00058857	0.00262224	4623.20883
5237.74632	0.10688557	0.0370435	2.88540687	0.00390908	0.01395021	5105.62257
4490.95461	0.1386014	0.03547931	3.90654137	9.36E-05	0.00049766	4325.05677
579.357047	0.31169677	0.0837253	3.72285043	0.00019699	0.00098013	532.414396
11161.5083	0.16796183	0.03132325	5.36220953	8.22E-08	7.62E-07	10214.9489
3119.63408	-0.5353274	0.04082986	-13.111176	2.84E-39	2.59E-37	3694.49294
302.026515	-1.0172976	0.11707998	-8.6889117	3.66E-18	1.07E-16	424.07964
1820.97425	-0.2651509	0.05256	-5.044729	4.54E-07	3.72E-06	1951.87747

4728.98059	0.17606758	0.03684392	4.77874193	1.76E-06	1.32E-05	4360.24241
12531.3281	-0.7319957	0.08292196	-8.8275251	1.07E-18	3.25E-17	16146.5082
5060.23677	-0.5440827	0.05016537	-10.845783	2.09E-27	1.14E-25	6241.74859
3452.42236	-0.7613601	0.04110045	-18.524376	1.31E-76	4.11E-74	4199.12919
712.156365	-0.8171696	0.2337209	-3.4963479	0.00047167	0.00214583	1068.53254
49.7762879	-2.1370432	0.31423857	-6.800703	1.04E-11	1.63E-10	96.2975602
15.1675944	-2.8880737	0.62461295	-4.6237814	3.77E-06	2.65E-05	26.8522043
1683.78731	-1.1931472	0.06105444	-19.542349	4.79E-85	1.79E-82	2371.32742
1139.14715	-1.1183402	0.16935716	-6.6034423	4.02E-11	5.82E-10	1741.68953
172.212755	0.510779	0.15446375	3.30678875	0.00094372	0.0039761	151.853845
993.035084	0.6285365	0.06979337	9.0056766	2.14E-19	6.87E-18	773.158296
8697.1638	0.21252796	0.0302538	7.02483511	2.14E-12	3.63E-11	7953.8081
661.981486	0.33561151	0.07877138	4.26057652	2.04E-05	0.00012473	552.785033
1180.69295	-0.9000941	0.06292751	-14.303665	2.08E-46	2.40E-44	1522.2422
1743.43532	0.62866933	0.06531915	9.62457846	6.30E-22	2.43E-20	1312.05426
1101.12584	-0.4601708	0.0707123	-6.5076493	7.63E-11	1.08E-09	1372.24023
758.261193	0.19593139	0.07802345	2.51118584	0.01203263	0.03690122	650.008532
571.178647	0.28454504	0.08862528	3.21065306	0.00132434	0.00537639	535.19221
4751.92313	0.17398371	0.03631475	4.7909923	1.66E-06	1.25E-05	4432.46558
3473.36231	0.24851896	0.03881751	6.40223922	1.53E-10	2.07E-09	3184.30105
3998.26275	-0.2759505	0.04095102	-6.7385492	1.60E-11	2.45E-10	4330.6124
3792.20453	0.13593886	0.03995193	3.40256048	0.00066758	0.00292914	3517.63876
1272.53423	-0.6391572	0.0585758	-10.911626	1.01E-27	5.67E-26	1579.65036
6491.28488	0.23397569	0.03604536	6.491145	8.52E-11	1.20E-09	6068.59817
27.9890862	-1.4308877	0.38270625	-3.7388667	0.00018485	0.00092593	42.5931516
7044.92088	0.17212851	0.0333578	5.1600683	2.47E-07	2.11E-06	6411.19526
3154.63157	0.20085038	0.04740394	4.23699776	2.27E-05	0.00013706	2792.62925
1703.62276	0.17578325	0.05521628	3.18354027	0.00145486	0.00585234	1503.72344
3567.3033	0.13134789	0.04500631	2.91843258	0.00351796	0.01271121	3335.22896
824.306351	-0.24285	0.07037239	-3.450927	0.00055867	0.00249936	924.086203
706.295382	-0.340011	0.08767538	-3.878067	0.00010529	0.00055343	699.08325
6474.06879	0.39609217	0.04045632	9.79061208	1.24E-22	4.97E-21	5333.40334
6915.70859	-0.1771275	0.03565928	-4.9672197	6.79E-07	5.42E-06	7084.35224
197.491584	-0.5160227	0.14034317	-3.6768635	0.00023612	0.00115644	248.151405
151.011197	-1.3790515	0.18048936	-7.6406249	2.16E-14	4.51E-13	250.929219
4651.03387	-0.5326793	0.03755469	-14.184095	1.15E-45	1.30E-43	5338.03303
48145.2095	0.71884111	0.02482616	28.9549884	2.43E-184	4.71E-181	36156.0301
48.9805012	-1.2372266	0.30296274	-4.0837584	4.43E-05	0.00025154	91.6678698
794.560639	-0.6145927	0.07560789	-8.1286848	4.34E-16	1.06E-14	903.715565
34.415781	1.30760639	0.33730815	3.87659299	0.00010593	0.00055604	22.2225139
524.564327	-0.2172866	0.08861769	-2.4519553	0.01420823	0.04255664	574.081609
94.5638842	-1.9254193	0.22930428	-8.3967874	4.59E-17	1.22E-15	162.965102
110.034797	-1.0692667	0.23278096	-4.5934456	4.36E-06	3.03E-05	128.705393

33.98459	-1.6556939	0.36297687	-4.561431	5.08E-06	3.49E-05	57.4081609
10570.5604	0.31182586	0.03254071	9.58263927	9.46E-22	3.62E-20	9555.68098
956.157652	-0.4640319	0.06665252	-6.9619555	3.36E-12	5.54E-11	1083.34755
326.781058	-0.8989762	0.11167411	-8.0499968	8.28E-16	1.97E-14	440.746526
5147.32002	0.18183741	0.03646599	4.98649311	6.15E-07	4.94E-06	4698.20981
2507.2614	-0.3383644	0.04473472	-7.5637988	3.91E-14	8.00E-13	2749.11016
7269.14869	-0.1230557	0.03217176	-3.82496	0.00013079	0.00067593	7425.09746
4357.46017	0.18013906	0.04408946	4.08576266	4.39E-05	0.0002496	3975.05217
8999.36436	0.10688741	0.03127321	3.41785878	0.00063116	0.00278638	8441.77747
18050.6365	0.13787242	0.02949013	4.67520624	2.94E-06	2.10E-05	16796.5168
155.164178	-0.6328747	0.15767012	-4.0139165	5.97E-05	0.00033001	193.521059
2891.40973	-0.6333186	0.04289338	-14.764952	2.46E-49	3.19E-47	3439.85996
15.4739581	1.690017	0.51608532	3.2746853	0.0010578	0.00439964	9.25938079
2468.94198	-1.373247	0.04787647	-28.683133	6.19E-181	1.09E-177	3565.78754
3000.75309	-0.3349805	0.04341582	-7.7156332	1.20E-14	2.58E-13	3306.52488
4383.32966	0.17396594	0.03711545	4.68715684	2.77E-06	1.99E-05	4028.75658
6736.88806	-0.7242811	0.04260316	-17.000641	8.12E-65	1.92E-62	8076.03193
1407.89897	0.15783626	0.05751434	2.74429404	0.00606412	0.02039788	1334.27677
1612.93393	0.22953318	0.05853981	3.92097592	8.82E-05	0.00047149	1435.20402
1405.79429	-0.2172426	0.0575721	-3.7734001	0.00016104	0.00081571	1511.13095
6062.94812	0.32298643	0.03268951	9.8804301	5.06E-23	2.06E-21	5355.62585
7119.23724	0.26716825	0.03184185	8.39047552	4.84E-17	1.29E-15	6527.86346
647.890747	0.68041202	0.08036055	8.46699026	2.52E-17	6.92E-16	500.932501
90.0254915	0.6211229	0.20764401	2.99128733	0.00277804	0.01034229	75.9269225
260.714034	1.02934749	0.12161243	8.46416351	2.58E-17	7.08E-16	166.668854
10062.2291	0.26870934	0.0293871	9.1437862	6.03E-20	2.01E-18	9094.56381
868.381699	0.18800463	0.07099318	2.64820668	0.008092	0.02616136	801.862377
886.38382	0.46357387	0.06898837	6.71959413	1.82E-11	2.77E-10	739.824525
1357.16953	0.21473071	0.06320403	3.39742124	0.00068024	0.00297663	1210.20107
1508.04966	0.40310534	0.05756883	7.00214549	2.52E-12	4.23E-11	1246.31265
5404.02103	0.31168689	0.03823773	8.15129086	3.60E-16	8.85E-15	4817.65583
9306.02445	0.26489262	0.03170554	8.35477325	6.56E-17	1.69E-15	8469.55561
4115.38627	0.12070716	0.04022768	3.00059997	0.00269448	0.01007572	3872.27305
11459.7976	0.06942287	0.02855285	2.43138106	0.01504139	0.04467932	11188.1098
5849.3708	0.20870035	0.03387067	6.16168372	7.20E-10	8.86E-09	5361.18148
26958.5648	0.29650275	0.02734857	10.8416162	2.19E-27	1.19E-25	23710.4964
9935.03761	0.10521758	0.02901458	3.62636972	0.00028743	0.00137714	9478.82812
762.478609	-0.3025955	0.07338665	-4.1233049	3.73E-05	0.00021591	846.307404
3906.91783	-0.1702905	0.04083723	-4.1699811	3.05E-05	0.00017949	4241.72234
330.015236	-0.3540981	0.10892809	-3.2507509	0.00115101	0.0047483	370.375232
4899.71826	0.74382801	0.03688973	20.163554	2.05E-90	8.10E-88	3608.38069
1429.50787	-0.6625419	0.05589365	-11.853616	2.06E-32	1.41E-30	1719.46701
1332.70791	0.28892587	0.06056712	4.77034191	1.84E-06	1.37E-05	1175.01542

1240.79086	-0.3292952	0.06303656	-5.2238765	1.75E-07	1.54E-06	1332.4249
2907.97839	0.12745302	0.04646345	2.7430815	0.00608656	0.02046625	2667.62761
1370.83917	0.1638959	0.06020127	2.72246574	0.00647968	0.02161936	1294.46144
2099.48981	0.28710287	0.04908232	5.84941513	4.93E-09	5.39E-08	1934.28465
6183.77027	0.20682678	0.03592143	5.75775455	8.52E-09	9.05E-08	5941.74465
1781.80724	0.54796046	0.05285543	10.3671562	3.50E-25	1.63E-23	1392.61087
6184.62003	-0.1382415	0.03287947	-4.2044946	2.62E-05	0.00015646	6381.56524
2349.77211	0.18384219	0.05320246	3.45552041	0.00054923	0.00246056	2101.87944
359.445196	0.25912118	0.10675592	2.42723014	0.0152146	0.045104	301.855814
4668.8336	0.25302596	0.03792405	6.67191384	2.52E-11	3.76E-10	4159.31385
2574.71437	0.27615331	0.05634534	4.90108531	9.53E-07	7.43E-06	2402.80932
2806.53526	-0.3601993	0.04361338	-8.2589158	1.47E-16	3.71E-15	3098.18881
587.602732	-0.8117738	0.08659329	-9.3745584	6.95E-21	2.49E-19	753.713596
1458.68741	0.1822936	0.05551403	3.28373948	0.0010244	0.0042788	1367.61054
5960.70622	0.19841762	0.03671578	5.4041509	6.51E-08	6.14E-07	5314.88457
746.266068	-0.8163249	0.07805058	-10.458922	1.33E-25	6.43E-24	934.271522
406.40528	-0.3860045	0.0968978	-3.983625	6.79E-05	0.00037114	442.598402
9658.20143	-0.2792662	0.03458309	-8.0752259	6.74E-16	1.61E-14	10320.5058
1202.01761	0.30080356	0.05992538	5.01963534	5.18E-07	4.20E-06	1096.31069
890.276872	0.31802214	0.06946281	4.57830806	4.69E-06	3.23E-05	817.603324
373.315409	0.27846139	0.10451306	2.66436924	0.00771329	0.02510444	362.041789
235.287485	-0.4096304	0.12610902	-3.2482245	0.00116128	0.00478456	270.373919
1837.50847	0.23581913	0.05083367	4.63903419	3.50E-06	2.48E-05	1735.20796
6883.33585	0.21958659	0.03759205	5.84130349	5.18E-09	5.64E-08	6480.64062
596.207142	-0.3888826	0.08426768	-4.6148493	3.93E-06	2.75E-05	673.156984
4098.32014	0.18787795	0.04488675	4.18559964	2.84E-05	0.00016876	3729.67858
6046.58969	0.37210031	0.03512509	10.5935765	3.19E-26	1.59E-24	5125.99321
2826.58664	0.20595823	0.04248474	4.84781633	1.25E-06	9.56E-06	2556.51504
2511.25737	0.29822004	0.04521179	6.5960682	4.22E-11	6.10E-10	2275.9558
115.176969	0.99301098	0.18138804	5.47451176	4.39E-08	4.23E-07	75.0009844
1209.01387	0.19352073	0.0685648	2.82245001	0.00476583	0.01658032	1040.7544
6748.16249	-0.1594369	0.0347477	-4.588415	4.47E-06	3.10E-05	7342.68897
19.1029934	-2.6169686	0.52524594	-4.982368	6.28E-07	5.03E-06	32.4078328
8393.81461	0.26099363	0.02976971	8.76708617	1.83E-18	5.48E-17	7619.54445
2815.21602	-0.1728166	0.04355822	-3.9674849	7.26E-05	0.00039474	3003.74313
3087.87573	0.42672074	0.04493539	9.49631691	2.17E-21	8.20E-20	2566.70036
2058.87155	0.12590584	0.05143479	2.4478731	0.01437023	0.04294232	1901.87681
206.337293	0.639362	0.14067625	4.54491788	5.50E-06	3.75E-05	139.81665
2266.05568	0.19897744	0.04589565	4.33543111	1.45E-05	9.16E-05	2083.36068
28.5621293	-0.9114943	0.38048488	-2.3956123	0.01659263	0.0486314	35.185647
8.94139645	-1.8677536	0.726124	-2.572224	0.01010475	0.0317524	12.9631331
1060.84843	0.15497181	0.06365452	2.4345767	0.01490922	0.04432749	984.272178
56.2453111	-0.8832197	0.29500859	-2.9938778	0.00275456	0.01027067	62.9637894

391.791807	-0.4288149	0.10364685	-4.1372688	3.51E-05	0.00020432	450.931845
1018.0831	0.19683896	0.06715627	2.93105838	0.00337809	0.01228138	943.530903
42.8273224	-0.8960023	0.3001131	-2.9855487	0.0028307	0.01052016	56.4822228
68.6181853	1.08563368	0.23916229	4.53931794	5.64E-06	3.85E-05	44.4450278
1416.86463	0.15798947	0.05590239	2.82616661	0.00471088	0.01642156	1334.27677
2775.09844	-0.283203	0.0422569	-6.7019361	2.06E-11	3.11E-10	3032.44721
1087.28703	0.23484813	0.06389323	3.67563426	0.00023726	0.00116143	969.457169
909.689938	-0.5181161	0.06822764	-7.5939323	3.10E-14	6.39E-13	1114.82945
12826.8312	-0.1379752	0.03274451	-4.2136893	2.51E-05	0.00015069	13225.1736
1004.8502	-0.3880062	0.06423783	-6.0401506	1.54E-09	1.81E-08	1162.05229
869.828963	-0.3141051	0.06943354	-4.5238232	6.07E-06	4.12E-05	976.864673
6195.09756	-0.4060501	0.03677241	-11.04225	2.39E-28	1.37E-26	6910.27588
2187.74984	0.46140724	0.04961295	9.30013773	1.40E-20	4.90E-19	1822.24614
4597.39955	-0.2689527	0.03561987	-7.5506367	4.33E-14	8.83E-13	5028.76971
3083.36701	0.27908549	0.04110033	6.79034692	1.12E-11	1.75E-10	2820.40739
10767.6584	0.15388882	0.02898722	5.30885149	1.10E-07	1.00E-06	10018.65
2536.61618	-0.1876153	0.04471746	-4.1955718	2.72E-05	0.00016225	2646.33103
33948.9642	0.16586019	0.02730874	6.07352121	1.25E-09	1.49E-08	31745.787
21867.2474	0.30683165	0.03019496	10.1616845	2.94E-24	1.29E-22	19494.7003
2302.41776	-0.2746782	0.05099931	-5.3859202	7.21E-08	6.73E-07	2596.33037
21.1615958	-1.9912464	0.47045973	-4.2325543	2.31E-05	0.00013957	33.3337709
1935.22532	0.25488146	0.0536318	4.75243213	2.01E-06	1.49E-05	1730.57827
1197.39489	0.41862464	0.06024026	6.94924973	3.67E-12	6.03E-11	986.124054
81.2981277	-0.7004764	0.23699447	-2.9556654	0.00311995	0.01145243	114.816322
2884.81607	0.11083998	0.04284362	2.58708274	0.00967924	0.03058884	2779.66611
4621.76172	0.16944888	0.03878146	4.36932657	1.25E-05	7.95E-05	4254.68547
922.386318	-0.264975	0.07214854	-3.6726312	0.00024007	0.0011731	1025.93939
43.1143504	-0.7751858	0.30538258	-2.5384087	0.01113579	0.03448909	66.6675417
3878.18629	0.11492365	0.03808013	3.01794268	0.00254497	0.00957389	3748.19734
4247.43567	0.73317482	0.04309812	17.0117589	6.72E-65	1.61E-62	3116.70757
1447.09084	0.21522956	0.06122364	3.51546471	0.00043899	0.00201128	1319.46176
7353.95478	0.17673911	0.0329105	5.37029587	7.86E-08	7.30E-07	6860.27523
3630.15764	0.10391786	0.03856005	2.69496174	0.00703967	0.02318451	3440.7859
793.776515	0.20889038	0.07304028	2.85993396	0.00423729	0.01495901	756.491411
8784.69585	0.19534945	0.03006311	6.49797953	8.14E-11	1.15E-09	8138.99572
1040.48509	0.23661637	0.07194407	3.28889345	0.00100582	0.00421211	971.309045
140818.214	0.33905519	0.02483612	13.6516984	1.97E-42	2.01E-40	122491.423
1411.87815	-0.25796	0.0575012	-4.4861667	7.25E-06	4.86E-05	1512.98282
2823.96874	0.30831022	0.04791262	6.43484393	1.24E-10	1.70E-09	2475.95842
115.646853	0.48496719	0.18885435	2.56794298	0.0102304	0.03209001	94.4456841
1448.2944	0.2134126	0.05587289	3.81960902	0.00013366	0.00068966	1375.01805
911.255369	-0.2544825	0.06830966	-3.7254245	0.00019499	0.00097143	981.494364
151.437338	0.49957858	0.1601393	3.11965007	0.00181066	0.00709944	142.594464

1394.79914	1.10266397	0.06048789	18.2294997	3.01E-74	8.84E-72	895.382122
6720.07942	0.23650855	0.03591857	6.5845768	4.56E-11	6.57E-10	6173.22917
249.022146	0.43234077	0.12598438	3.43170151	0.00059981	0.00266436	226.854829
2630.78848	-0.1365939	0.04602478	-2.9678337	0.00299907	0.01107158	2637.99759
145.035747	-0.677361	0.16612939	-4.07731	4.56E-05	0.00025764	155.557597
1309.27523	0.15893143	0.05822164	2.72976576	0.00633793	0.02122682	1218.53451
3008.17943	-0.4525008	0.04143626	-10.920407	9.21E-28	5.17E-26	3449.11934
1780.10313	-0.5903155	0.05245494	-11.253765	2.22E-29	1.36E-27	2089.84224
1046.24684	-0.5663878	0.06361274	-8.9036852	5.40E-19	1.68E-17	1226.86796
1975.11871	0.62363158	0.04852935	12.8506072	8.53E-38	7.22E-36	1543.53878
75.4481857	1.43388421	0.22764047	6.29889836	3.00E-10	3.88E-09	40.7412755
3317.79931	-0.2673136	0.04177504	-6.3988848	1.57E-10	2.11E-09	3590.78787
56.3661737	-1.8652711	0.2963563	-6.2940153	3.09E-10	4.00E-09	97.2234983
121.09347	-0.6148296	0.17948862	-3.4254518	0.00061378	0.00271708	152.779783
438.990445	0.53518493	0.09824779	5.44729744	5.11E-08	4.90E-07	332.41177
81.99021	0.81926547	0.21953637	3.73179835	0.00019012	0.00094985	54.6303467
1502.19029	0.62393273	0.05739243	10.8713413	1.58E-27	8.69E-26	1164.8301
890.971313	0.51097587	0.06753787	7.56576837	3.86E-14	7.89E-13	750.009844
3980.07997	0.13406229	0.0379336	3.53413009	0.00040912	0.00189143	3795.42019
8120.6321	0.21967327	0.03219775	6.82262861	8.94E-12	1.41E-10	7451.94966
320.471794	0.34834264	0.12105626	2.87752694	0.00400806	0.01424505	321.300513
1218.80833	0.25807426	0.05928086	4.35341641	1.34E-05	8.49E-05	1104.64413
4075.1924	0.2516516	0.04000386	6.29068342	3.16E-10	4.08E-09	3592.63975
8304.32012	0.3679443	0.03287154	11.1934019	4.39E-29	2.64E-27	7150.09385
9564.39076	0.29228345	0.02945916	9.92165099	3.35E-23	1.38E-21	8528.81565
26309.6747	0.21669531	0.02744394	7.89592587	2.88E-15	6.54E-14	23881.7949
2993.61269	0.25849464	0.04536602	5.6979799	1.21E-08	1.26E-07	2784.2958
1987.51981	0.15998049	0.04844922	3.30202399	0.0009599	0.00403837	1901.87681
7993.46291	0.19435046	0.0305121	6.3696196	1.89E-10	2.54E-09	7407.50463
91.446084	0.85098554	0.20851357	4.08119978	4.48E-05	0.00025403	53.7044086
777.329321	0.24939428	0.07195859	3.46580292	0.00052865	0.00237385	682.416364
399.959111	0.84698901	0.09900093	8.55536378	1.17E-17	3.31E-16	269.447981
6577.75508	0.12769207	0.03566356	3.58046326	0.00034299	0.00161496	6308.41613
2137.99253	0.40350797	0.04726423	8.53727949	1.37E-17	3.85E-16	1827.80177
221.440592	-0.6142211	0.13348914	-4.6012814	4.20E-06	2.93E-05	269.447981
4422.41339	0.15840069	0.03708418	4.27138188	1.94E-05	0.00011947	4173.20292
3313.6999	0.23130444	0.03973044	5.82184476	5.82E-09	6.32E-08	3023.18783
136.226708	-0.5804805	0.16681091	-3.4798711	0.00050166	0.00226575	150.001969
1240.97461	0.19365389	0.05834318	3.31922099	0.00090269	0.00382484	1129.64446
10235.9558	0.17488127	0.02889573	6.05214853	1.43E-09	1.68E-08	9614.01508
540.854018	-0.5868206	0.09798109	-5.9891211	2.11E-09	2.42E-08	620.378513
311.592151	-0.6442881	0.11775195	-5.4715708	4.46E-08	4.29E-07	402.783064
538.005435	-0.3216765	0.08688631	-3.7022689	0.00021368	0.00105453	580.563176

2704.14002	0.20890801	0.04743846	4.40376889	1.06E-05	6.88E-05	2601.886
4582.24589	-0.1476223	0.04005946	-3.6850794	0.00022863	0.00112345	4695.432
3046.36396	-0.1405146	0.04534535	-3.0987655	0.00194329	0.00756287	3299.11738
1471.92618	0.21646542	0.06284524	3.44442051	0.00057229	0.00255322	1422.24089
1412.18637	0.18970581	0.05638932	3.36421517	0.00076762	0.00330893	1275.01674
8976.29976	-0.1285355	0.03209979	-4.0042458	6.22E-05	0.00034253	9528.82877
5496.39935	-0.1003799	0.03346484	-2.9995621	0.00270368	0.01010427	5676.92636
6249.08269	0.11373108	0.03414958	3.33038059	0.00086727	0.00369334	5831.55802
4869.9841	0.09425863	0.03579485	2.6333018	0.00845592	0.02713874	4634.32009
1376.25067	0.28827143	0.05934771	4.85733016	1.19E-06	9.16E-06	1319.46176
317.176769	0.35531277	0.11422135	3.11073873	0.0018662	0.00728773	279.6333
1465.8132	0.20414856	0.05397378	3.78236568	0.00015535	0.00078976	1355.57335
2736.24123	0.34995566	0.04218621	8.29549822	1.08E-16	2.75E-15	2427.80964
1805.1106	0.13143842	0.04987561	2.63532472	0.00840568	0.02700434	1751.87485
1354.97708	0.14159387	0.05649089	2.50649044	0.01219364	0.0373418	1301.86894
1617.3114	0.26877621	0.05195283	5.17346574	2.30E-07	1.98E-06	1490.76031
3394.02355	-0.2502868	0.04077212	-6.1386766	8.32E-10	1.02E-08	3810.2352
5229.97284	0.30205259	0.03791185	7.96723404	1.62E-15	3.75E-14	4803.76675
897.171097	-0.5269152	0.06877219	-7.661748	1.83E-14	3.85E-13	1082.42161
152.048819	-0.4123068	0.15910049	-2.5914866	0.00955623	0.03026919	170.372607
486.409485	-0.6685644	0.09518533	-7.0238175	2.16E-12	3.65E-11	554.636909
1552.32286	0.52929287	0.05329755	9.93090445	3.05E-23	1.27E-21	1249.09047
137.141477	0.54584826	0.17547197	3.11074332	0.00186617	0.00728773	108.334755
394.989466	-0.489482	0.10238429	-4.7808314	1.75E-06	1.31E-05	459.265287
683.104574	0.33287234	0.07943633	4.19042948	2.78E-05	0.00016551	632.415708
49.5728902	-2.0257991	0.30630598	-6.6136453	3.75E-11	5.45E-10	77.7787986
4774.09774	0.24922319	0.03573888	6.97344808	3.09E-12	5.12E-11	4293.57487
2444.47335	0.44753016	0.0463439	9.6567222	4.60E-22	1.78E-20	2063.91598
279.428276	-1.2784708	0.12611298	-10.137504	3.77E-24	1.64E-22	418.524012
1908.60168	0.22252441	0.04981573	4.46695059	7.93E-06	5.27E-05	1775.94924
162.872572	0.61418277	0.15190707	4.04314802	5.27E-05	0.00029437	124.075703
2334.9541	0.18550982	0.04608633	4.02526775	5.69E-05	0.00031584	2179.65824
185.922787	-0.4368393	0.14868949	-2.9379301	0.00330412	0.01204632	196.298873
3546.82889	0.28868883	0.03992135	7.23143892	4.78E-13	8.74E-12	3130.59665
6181.43855	0.27379337	0.03790178	7.22375966	5.06E-13	9.22E-12	5653.77791
4833.67411	-0.1782604	0.04049715	-4.4018012	1.07E-05	6.94E-05	5016.73251
1165.69718	0.2584378	0.06139605	4.20935546	2.56E-05	0.00015342	1104.64413
17.114374	3.08600817	0.58342735	5.28944726	1.23E-07	1.10E-06	5.55562847
5741.21577	-0.1077482	0.03747918	-2.8748826	0.00404178	0.01435242	5784.33518
845.259963	-0.2935551	0.07112591	-4.1272588	3.67E-05	0.00021262	949.086531
198.337739	-1.3778738	0.15059498	-9.1495334	5.72E-20	1.91E-18	266.670167
226.105326	-0.3309452	0.13229078	-2.5016496	0.01236162	0.03779057	258.336724
5051.53898	0.33675649	0.0364544	9.23774714	2.52E-20	8.64E-19	4504.68876

3177.07937	0.27000691	0.04218858	6.3999999	1.55E-10	2.10E-09	2815.7777
3450.32226	0.41932001	0.04051533	10.3496638	4.20E-25	1.94E-23	3032.44721
3196.86557	0.17552113	0.04120983	4.25920492	2.05E-05	0.00012527	2959.2981
5627.45103	0.25488162	0.04322263	5.8969487	3.70E-09	4.11E-08	4983.39874
10442.6339	-0.2737926	0.02840241	-9.639768	5.43E-22	2.10E-20	11389.9643
1899.06737	0.45382904	0.05020782	9.0390102	1.58E-19	5.11E-18	1612.98413
1415.74186	0.33739839	0.05510353	6.12299024	9.18E-10	1.12E-08	1234.27546
8599.98998	0.43843772	0.03881869	11.2945022	1.40E-29	8.65E-28	7084.35224
5819.18516	0.1881321	0.03830938	4.91086285	9.07E-07	7.09E-06	5223.2167
1902.12924	-0.1755826	0.05237109	-3.3526621	0.00080038	0.00343338	2099.10163
3671.33056	0.23243353	0.03808149	6.10358363	1.04E-09	1.25E-08	3367.63679
6727.27468	0.10189529	0.04117567	2.47464782	0.01333676	0.04031504	6600.08663
5219.54338	0.12110361	0.0372575	3.25044883	0.00115223	0.00475234	4843.58209
5225.61609	0.26347511	0.03534337	7.45472532	9.01E-14	1.77E-12	4717.65451
5728.27516	0.1815381	0.03371232	5.38491891	7.25E-08	6.76E-07	5275.06924
593.935847	0.27726783	0.08088903	3.42775592	0.00060859	0.00269844	543.525652
6854.51171	-0.2904689	0.03436682	-8.4520161	2.86E-17	7.82E-16	7626.02602
10685.7196	-0.1039179	0.03116131	-3.3348379	0.00085349	0.00364185	10827.9199
4189.50603	-0.2484603	0.0374637	-6.6320278	3.31E-11	4.84E-10	4587.09724
10505.7633	-0.4270154	0.03330883	-12.819885	1.27E-37	1.06E-35	12395.5331
11215.2498	-0.1493709	0.02944803	-5.0723568	3.93E-07	3.25E-06	11800.1549
4018.7583	-0.1290856	0.05259453	-2.4543532	0.01411383	0.04230003	4389.87243
606.579328	-0.7492297	0.08347455	-8.9755467	2.82E-19	8.89E-18	754.639534
19425.1404	-0.6652651	0.02797504	-23.780666	5.29E-125	3.80E-122	23492.9009
326.772242	-0.453263	0.10921768	-4.1500881	3.32E-05	0.00019423	378.708674
7701.42729	0.14351532	0.03341802	4.29454941	1.75E-05	0.00010873	7138.05665
7121.03434	0.19892986	0.03275144	6.07392757	1.25E-09	1.48E-08	6576.01224
9937.59807	0.17037404	0.02955515	5.7646148	8.18E-09	8.71E-08	9333.45584
2980.81382	-0.2091948	0.04370975	-4.7859981	1.70E-06	1.28E-05	3103.74444
1261.89502	-0.4052264	0.0606936	-6.676592	2.45E-11	3.65E-10	1363.90679
1504.50008	0.47899857	0.05646107	8.48369674	2.18E-17	6.03E-16	1227.79389
5550.0977	-0.299635	0.03695392	-8.1083409	5.13E-16	1.24E-14	6235.26702
516.420551	-0.3002433	0.09002634	-3.3350606	0.00085281	0.00363974	554.636909
10246.9494	0.29024103	0.03066797	9.4639809	2.96E-21	1.11E-19	9141.78665
332.603121	-0.3865675	0.11218326	-3.4458574	0.00056925	0.00254261	418.524012
532.744724	-0.2694825	0.089072	-3.0254459	0.00248267	0.00936861	600.007875
10040.026	0.15333452	0.02840478	5.39819444	6.73E-08	6.33E-07	9527.90283
14068.8103	-0.1580343	0.02964062	-5.3316817	9.73E-08	8.92E-07	14793.7127
4811.83358	0.1208732	0.03785677	3.19290856	0.00140848	0.00567754	4643.57947
2809.77648	0.24118457	0.04199254	5.7435099	9.27E-09	9.80E-08	2537.07034
5865.438	0.3713562	0.03519279	10.5520547	4.97E-26	2.45E-24	5203.772
30191.9538	-0.4600592	0.02430913	-18.925366	7.05E-80	2.36E-77	35252.3146
1789.57823	0.19581513	0.05043481	3.88253959	0.00010337	0.00054453	1665.7626

201.450957	0.34126852	0.14268092	2.39183014	0.0167646	0.04906125	158.335412
16961.529	0.21718297	0.02748187	7.90277271	2.73E-15	6.21E-14	15566.871
2399.28115	0.23617303	0.04537161	5.20530387	1.94E-07	1.69E-06	2195.39919
1554.97072	0.19850428	0.06484158	3.06137315	0.00220324	0.00843402	1398.1665
4886.83754	0.21015016	0.0358294	5.86530019	4.48E-09	4.94E-08	4431.53965
1070.33691	0.22665121	0.06188559	3.66242295	0.00024984	0.00121535	975.012797
10272.0327	0.23650476	0.03737047	6.32865316	2.47E-10	3.25E-09	9177.89824
2440.97701	0.42577969	0.04925482	8.6444264	5.41E-18	1.56E-16	2016.69314
4747.28346	-0.3444784	0.03549367	-9.7053479	2.86E-22	1.12E-20	5333.40334
4944.9487	0.17982491	0.0358007	5.02294399	5.09E-07	4.14E-06	4725.98796
348.816867	0.36193796	0.10523895	3.43920162	0.00058343	0.00260116	298.152062
83.1341132	-0.8805666	0.2149061	-4.0974483	4.18E-05	0.00023838	109.260693
82.5473982	-0.9374538	0.23063046	-4.0647443	4.81E-05	0.00026995	117.594136
2491.1886	-0.2444744	0.04946495	-4.9423763	7.72E-07	6.10E-06	2839.85209
5421.16566	0.19832142	0.03382574	5.863033	4.54E-09	5.00E-08	5094.51131
3814.97333	-0.1014123	0.04073765	-2.4894009	0.01279586	0.03890945	4065.79411
1094.74489	0.26852379	0.06423905	4.18007098	2.91E-05	0.00017265	1008.34657
1136.9182	0.36126418	0.0666749	5.41829335	6.02E-08	5.70E-07	933.345584
1767.72173	-0.5542891	0.05722786	-9.6856517	3.47E-22	1.35E-20	1971.32217
89787.8246	-0.2014134	0.02384273	-8.4475831	2.97E-17	8.09E-16	94775.318
583.457941	-0.7528079	0.08287629	-9.0835134	1.05E-19	3.44E-18	711.120445
13311.7822	0.28617458	0.03071895	9.31589686	1.21E-20	4.24E-19	11894.6006
1294.45821	0.23021581	0.06146055	3.74574946	0.00017986	0.00090254	1167.60792
8761.93169	0.1232031	0.03060036	4.02619748	5.67E-05	0.00031477	8479.74093
2928.80292	-0.1627234	0.04382066	-3.713395	0.0002045	0.0010131	3070.41067
15036.9672	0.09817308	0.03092138	3.17492578	0.00149875	0.00600274	14204.8161
89.6768871	-0.8504129	0.21064976	-4.0370942	5.41E-05	0.00030154	101.853189
1373.66745	0.26788354	0.0640794	4.18049395	2.91E-05	0.00017238	1223.1642
5775.96319	0.37617579	0.03626669	10.3724868	3.31E-25	1.55E-23	5063.95535
883.692694	-0.190103	0.0685245	-2.7742342	0.00553318	0.01882098	930.567769
7037.60585	0.15734759	0.03297044	4.7723837	1.82E-06	1.36E-05	6713.97701
564.955507	-0.3423064	0.08352491	-4.098255	4.16E-05	0.00023783	637.045398
165.007616	-0.4155897	0.15676658	-2.6510094	0.00802516	0.02596691	213.891696
567.257834	-0.2985741	0.08885125	-3.3603814	0.00077835	0.00334924	592.600371
2319.94288	0.16955004	0.0467045	3.63027167	0.00028312	0.00135951	2167.62104
320.188226	-0.8726035	0.11858487	-7.3584727	1.86E-13	3.53E-12	399.079312
1324.44408	1.03164438	0.06389222	16.1466343	1.20E-58	2.40E-56	861.122414
2190.94021	-0.9662018	0.05024541	-19.229653	2.09E-82	7.37E-80	2831.51865
49.893609	-0.9459462	0.27966137	-3.38247	0.00071837	0.00312094	62.9637894
2130.76617	-0.6160772	0.04838139	-12.733765	3.84E-37	3.11E-35	2572.25598
23061.1706	-0.7447004	0.02911447	-25.578364	2.66E-144	2.71E-141	29041.1219
3276.02012	0.83821265	0.04238838	19.7745836	4.93E-87	1.91E-84	2325.95646
1296.70433	-0.2177362	0.06155677	-3.53716	0.00040446	0.00187299	1374.09211

1823.65549	-0.3658374	0.05134973	-7.124426	1.05E-12	1.84E-11	2070.39755
5331.99846	0.13954521	0.03385205	4.12220928	3.75E-05	0.00021656	5077.84443
10501.5017	-0.3669281	0.02896095	-12.669752	8.70E-37	7.00E-35	11937.1937
5906.59297	0.09973683	0.03716792	2.68341182	0.00728752	0.02389118	5442.66403
3725.91566	-0.4297846	0.04410996	-9.7434826	1.97E-22	7.80E-21	4088.94256
3955.95901	0.26536448	0.03939343	6.73626223	1.63E-11	2.49E-10	3488.00874
572.594121	0.42849994	0.08324633	5.14737351	2.64E-07	2.25E-06	476.858111
4960.30716	0.192413	0.0387761	4.96215512	6.97E-07	5.54E-06	4539.8744
10212.9369	0.09092978	0.03169557	2.86884843	0.00411969	0.01459434	9601.05194
95.6655316	-0.6951181	0.2044126	-3.4005638	0.00067247	0.00294662	121.297888
25964.9213	0.2548535	0.03413787	7.46541872	8.30E-14	1.64E-12	23557.7166
512.671106	0.63722162	0.09502903	6.70554715	2.01E-11	3.04E-10	352.782408
6253.0007	0.10651207	0.03495522	3.04710093	0.0023106	0.00878083	6046.37566
7533.31107	-0.3416218	0.03609176	-9.4653683	2.93E-21	1.09E-19	8746.4111
1810.56242	-0.4154695	0.05440447	-7.6366795	2.23E-14	4.65E-13	2039.84159
6425.79179	0.23908536	0.03248932	7.3588918	1.85E-13	3.52E-12	5948.22622
3585.64748	0.15507336	0.03808075	4.07222465	4.66E-05	0.00026279	3363.0071
55.1745793	-1.6470095	0.27882343	-5.906998	3.48E-09	3.87E-08	77.7787986
3361.26838	-0.2166599	0.04166987	-5.1994371	2.00E-07	1.74E-06	3629.67727
1327.7686	-0.5415664	0.06404831	-8.4555923	2.78E-17	7.59E-16	1547.24253
2414.70852	0.32481854	0.0458745	7.08058975	1.44E-12	2.48E-11	2064.84192
1037.62726	0.27574589	0.06342996	4.34724986	1.38E-05	8.72E-05	903.715565
4940.00479	0.44053052	0.03899513	11.2970662	1.36E-29	8.43E-28	4185.24012
25.3331301	-1.2144494	0.4093828	-2.9665375	0.00301174	0.01111201	37.9634612
70.8781837	-0.8785784	0.24360408	-3.6065831	0.00031026	0.00147482	99.0753745
992.398896	0.39780271	0.07055018	5.6385779	1.71E-08	1.75E-07	878.715237
960.612711	0.23449933	0.06566644	3.57106806	0.00035553	0.00166794	919.456513
4299.44136	0.13841072	0.0436901	3.16801133	0.00153486	0.00613088	4079.68318
4978.54514	0.27048143	0.03461796	7.81332603	5.57E-15	1.23E-13	4557.46723
3925.62119	0.1488679	0.04061472	3.66536793	0.00024698	0.00120266	3550.97253
5334.03048	0.13634927	0.03593886	3.79392337	0.00014829	0.00075685	5218.58701
1437.55807	0.31817197	0.05602767	5.67883682	1.36E-08	1.40E-07	1243.53484
2410.80693	0.25665223	0.05091954	5.04034906	4.65E-07	3.80E-06	2112.06476
2282.44513	0.29206806	0.04540255	6.43285628	1.25E-10	1.72E-09	2048.17503
6220.59267	0.30565455	0.03375374	9.05542798	1.36E-19	4.42E-18	5463.03467
426.961253	0.58694682	0.09635088	6.09176373	1.12E-09	1.34E-08	364.819603
1174.25803	0.40322051	0.05972832	6.75091024	1.47E-11	2.26E-10	1030.56908
945.79558	0.26374902	0.06604257	3.99362168	6.51E-05	0.00035694	862.97429
5511.53375	0.2156369	0.03392525	6.35623558	2.07E-10	2.75E-09	5151.91947
4506.96061	0.14213756	0.03649288	3.89494	9.82E-05	0.00052038	4240.7964
2877.99477	0.12635338	0.04155949	3.0403018	0.00236341	0.0089622	2766.70298
5505.37612	0.21674384	0.03788777	5.72068124	1.06E-08	1.11E-07	5050.06628
2204.08933	0.54291859	0.04961447	10.9427482	7.20E-28	4.07E-26	1757.43047

1003.21389	0.15444736	0.06462294	2.38997741	0.01684941	0.04928713	916.678698
153.799706	0.60219295	0.16053945	3.75105894	0.00017609	0.0008857	133.335083
399.356586	0.26028111	0.09889267	2.63195564	0.0084895	0.02723748	382.412427
10329.8919	0.40410692	0.03572156	11.3126894	1.14E-29	7.10E-28	8860.30148
6235.82116	0.08130301	0.03349162	2.42756276	0.01520066	0.04507623	6101.93194
15.5831777	-1.3810729	0.50695562	-2.7242481	0.00644481	0.02152155	22.2225139
4810.47751	0.21399615	0.03573685	5.98810937	2.12E-09	2.43E-08	4444.50278
41816.3478	-0.7423461	0.12068732	-6.1509865	7.70E-10	9.46E-09	56619.2617
2202.86148	-0.7297786	0.10837478	-6.7338418	1.65E-11	2.53E-10	2831.51865
2185.94799	0.13063879	0.04593943	2.84371825	0.00445905	0.01566201	2075.95317
651.244454	-1.4481808	0.08299557	-17.448893	3.51E-68	8.72E-66	914.826822
461.494561	0.44316603	0.0955606	4.63753943	3.53E-06	2.49E-05	422.227764
135.64144	0.87046624	0.16945965	5.13671682	2.80E-07	2.37E-06	98.1494364
19641.8892	0.41039707	0.03275172	12.5305503	5.08E-36	3.96E-34	16925.2222
4848.23542	0.42557679	0.04050755	10.506111	8.10E-26	3.93E-24	3978.75593
264.76209	-0.4680307	0.12464728	-3.7548405	0.00017345	0.00087357	312.041133
1332.933	0.69901646	0.05699831	12.2638094	1.42E-34	1.05E-32	1034.27283
2398.44744	0.29398219	0.05172408	5.68366185	1.32E-08	1.37E-07	2055.58254
459.885346	0.37560823	0.09205982	4.08004545	4.50E-05	0.00025514	388.893993
555.169746	0.27601564	0.08333343	3.3121837	0.00092571	0.00390955	499.080625
2476.60971	0.28562674	0.04463568	6.39906717	1.56E-10	2.11E-09	2200.02888
2444.34941	0.18166003	0.04422037	4.10806242	3.99E-05	0.00022924	2266.69642
466.799613	-0.6051366	0.09239878	-6.5491838	5.79E-11	8.24E-10	555.562847
19529.7955	0.58206738	0.03268282	17.8095817	5.96E-71	1.58E-68	15961.3206
1131.57216	-0.4034341	0.06083188	-6.6319522	3.31E-11	4.84E-10	1284.27612
53.2802082	-1.2365397	0.27594312	-4.4811396	7.42E-06	4.96E-05	75.9269225
1351.01056	0.25493541	0.06006519	4.2443121	2.19E-05	0.00013313	1194.46012
1453.81638	0.39835096	0.05975843	6.6660209	2.63E-11	3.90E-10	1268.53517
967.852098	0.19676331	0.07027159	2.80004052	0.00510962	0.01760263	957.419974
2000.98526	0.21261222	0.05501226	3.86481538	0.00011117	0.00058167	1781.50486
713.005372	0.40937104	0.07586229	5.39623899	6.81E-08	6.38E-07	629.637894
9184.45925	0.25477634	0.03020692	8.4343712	3.33E-17	9.03E-16	8408.4437
1767.79436	0.25575005	0.05178177	4.93899798	7.85E-07	6.20E-06	1658.3551
1643.48437	0.21125058	0.05151947	4.10040283	4.12E-05	0.00023591	1529.64971
2875.37483	0.32773189	0.04609976	7.10918871	1.17E-12	2.04E-11	2581.51536
1180.92041	0.26927007	0.05915256	4.55212863	5.31E-06	3.63E-05	1051.86566
970.08048	0.18622508	0.06871454	2.7101262	0.00672576	0.02231751	841.677714
7636.76374	0.21213555	0.03393135	6.25190418	4.05E-10	5.17E-09	6982.49905
3510.01484	-0.3177643	0.04044748	-7.8562217	3.96E-15	8.84E-14	3832.45771
1904.40784	-0.1587419	0.05359246	-2.9620196	0.00305628	0.0112464	2051.87878
1916.55274	-0.3833725	0.04921144	-7.790312	6.68E-15	1.46E-13	2125.95383
1996.61185	-0.1398446	0.05081643	-2.7519553	0.00592406	0.01998848	1998.17438
2943.11989	0.18336447	0.04509219	4.06643547	4.77E-05	0.00026839	2672.2573

5348.94289	0.19769053	0.03986522	4.95897289	7.09E-07	5.63E-06	4800.063
22615.0608	-0.1170773	0.02864286	-4.0874852	4.36E-05	0.00024783	22899.3746
6263.30405	0.23192546	0.03459936	6.70317274	2.04E-11	3.09E-10	5899.1515
59.6501799	-1.0891821	0.26331434	-4.1364328	3.53E-05	0.00020498	89.8159937
7271.51727	0.19544525	0.03096871	6.311055	2.77E-10	3.61E-09	6862.1271
7563.40092	0.19920682	0.03590657	5.54792119	2.89E-08	2.85E-07	6882.49774
32.8978457	-1.4387004	0.3541935	-4.0619052	4.87E-05	0.00027294	50.9265944
8873.51245	0.22558444	0.0348462	6.47371807	9.56E-11	1.33E-09	7947.32653
3945.68086	0.18207978	0.03824739	4.76058036	1.93E-06	1.43E-05	3589.86193
8402.80297	0.14233241	0.03061239	4.64950304	3.33E-06	2.37E-05	7945.47466
46627.7993	0.11429208	0.02460834	4.64444598	3.41E-06	2.42E-05	44061.6894
1705.9729	-0.3291506	0.05297467	-6.2133573	5.19E-10	6.52E-09	1894.46931
5782.46364	0.39908913	0.03731638	10.6947429	1.08E-26	5.57E-25	5194.51262
1291.77482	0.26599876	0.05996234	4.4360972	9.16E-06	6.00E-05	1101.86631
925.321404	0.27948689	0.07118011	3.92647442	8.62E-05	0.00046211	840.751776
153.962811	-0.9269294	0.16677811	-5.5578601	2.73E-08	2.70E-07	197.224811
5286.1603	0.1085	0.0350776	3.09314166	0.0019805	0.00769069	5027.84377
420.620401	-0.4473085	0.09989019	-4.4780022	7.53E-06	5.03E-05	470.376544
1707.99623	-0.1588231	0.05508236	-2.8833754	0.00393438	0.01402759	1886.13587
4194.54854	0.15810047	0.03949692	4.00285544	6.26E-05	0.00034436	3950.97778
1478.17883	-0.4775609	0.05791788	-8.2454825	1.64E-16	4.14E-15	1683.35543
27.6162082	-1.5669699	0.39666155	-3.9503952	7.80E-05	0.00042177	51.8525324
511.107187	-0.2679772	0.09197728	-2.9135153	0.00357384	0.01289151	532.414396
761.410658	0.24294791	0.07199281	3.3746137	0.00073919	0.00319778	685.194179
1480.06372	0.18324904	0.0540951	3.38753464	0.00070524	0.00306939	1352.79553
1412.41456	-0.2242237	0.05526014	-4.0576027	4.96E-05	0.00027769	1525.94595
193.378674	-0.4496794	0.13993942	-3.2133864	0.0013118	0.00533666	233.336396
5861.40076	-0.1233165	0.03785022	-3.2580129	0.00112195	0.00464327	5934.33715
2914.51759	-0.1534214	0.04454151	-3.44446	0.0005722	0.00255322	3135.22634
52.5345749	-1.2137765	0.27523959	-4.4098905	1.03E-05	6.71E-05	75.0009844
708.027478	-0.3888168	0.08204695	-4.7389544	2.15E-06	1.59E-05	778.713925
245.931111	-1.3238747	0.144432	-9.1660756	4.91E-20	1.65E-18	382.412427
2485.83935	-0.2200045	0.04570865	-4.8131911	1.49E-06	1.13E-05	2744.48047
3282.87561	0.28240224	0.03997392	7.06466291	1.61E-12	2.77E-11	2922.26058
1809.34007	0.2125617	0.04918381	4.32178181	1.55E-05	9.70E-05	1672.24417
2084.225	0.15989139	0.04733804	3.37765094	0.00073108	0.00316813	1978.72968
830.042977	-0.6287502	0.07163758	-8.7768213	1.68E-18	5.05E-17	1022.23564
4114.12722	0.14969893	0.04574724	3.27230487	0.00106675	0.00443219	3720.4192
653.597875	0.54159851	0.09174527	5.90328516	3.56E-09	3.96E-08	509.265944
3353.69381	-0.274398	0.04242905	-6.4672213	9.98E-11	1.38E-09	3554.67629
1227.09566	0.72597137	0.05891362	12.3226411	6.84E-35	5.18E-33	917.604636
60.9246475	1.10269213	0.2509404	4.39423919	1.11E-05	7.17E-05	34.2597089
1099.23851	-0.2640691	0.06575028	-4.0162429	5.91E-05	0.00032727	1247.23859

192.153714	-0.6795793	0.14470811	-4.6962075	2.65E-06	1.92E-05	235.188272
124.263066	-0.4587001	0.18406922	-2.4919979	0.01270268	0.03866249	167.594792
169.018322	0.49879168	0.14665029	3.40123222	0.00067083	0.00294075	141.668526
2407.57342	0.24407439	0.04708247	5.1839756	2.17E-07	1.88E-06	2117.62039
159.793228	-0.7695006	0.16395418	-4.6933879	2.69E-06	1.94E-05	222.225139
2119.80839	-0.2065358	0.04909794	-4.2066071	2.59E-05	0.0001551	2175.95449
2973.37728	0.1569068	0.04172829	3.76020188	0.00016978	0.00085595	2765.77704
37045.1644	-0.4608157	0.02819977	-16.341113	5.03E-60	1.07E-57	43299.6424
20.1921815	-2.3129462	0.49728104	-4.6511852	3.30E-06	2.35E-05	33.3337709
4911.18855	0.23828809	0.04385689	5.43331032	5.53E-08	5.28E-07	4360.24241
309.041046	-0.3154597	0.11351851	-2.7789271	0.00545388	0.01860672	331.485832
4287.45882	0.13626738	0.03623852	3.76029055	0.00016972	0.00085595	4097.276
13895.8091	0.19840687	0.02850233	6.96107566	3.38E-12	5.57E-11	12998.3188
795.235833	0.42207233	0.07206794	5.85658952	4.72E-09	5.18E-08	695.379497
47.4422683	-1.2199371	0.30638404	-3.9817254	6.84E-05	0.00037391	63.8897275
417.250045	-0.6930666	0.09749941	-7.1084185	1.17E-12	2.05E-11	506.488129
2960.1693	0.19137532	0.04481282	4.2705485	1.95E-05	0.00011988	2645.40509
627.112271	-0.8898015	0.08350545	-10.65561	1.64E-26	8.31E-25	829.640519
474.521856	0.31901207	0.09061972	3.52033834	0.000431	0.00197936	421.301826
445.323047	-1.6562606	0.11242455	-14.732197	4.00E-49	5.11E-47	670.379169
5257.42798	0.1860544	0.03655156	5.09019096	3.58E-07	2.98E-06	4829.69302
5796.82978	0.28220045	0.03519565	8.01804995	1.07E-15	2.53E-14	5234.32796
5044.74041	0.22524308	0.03546672	6.35082892	2.14E-10	2.83E-09	4644.50541
15184.4492	0.29470952	0.0380868	7.73783968	1.01E-14	2.18E-13	13607.586
5235.37411	0.21343497	0.03590345	5.94469328	2.77E-09	3.11E-08	4899.13838
1329.31908	0.24983002	0.05755122	4.34100343	1.42E-05	8.96E-05	1242.6089
499.364499	-0.4623182	0.09391526	-4.9227165	8.54E-07	6.71E-06	600.007875
4269.80834	0.56730767	0.03661793	15.4926189	3.89E-54	6.45E-52	3461.15654
3849.61621	0.31283469	0.0404493	7.7339962	1.04E-14	2.24E-13	3388.93337
2444.65245	0.30758844	0.04486378	6.85605278	7.08E-12	1.12E-10	2200.95481
16973.0921	0.37821592	0.02571381	14.7086698	5.67E-49	7.14E-47	14712.2301
1052.83427	0.17106616	0.06344307	2.69637293	0.00700991	0.02311151	960.197788
5858.28141	0.12226165	0.03536011	3.45761557	0.00054498	0.00244264	5621.37008
3738.56729	0.21147	0.04198587	5.03669422	4.74E-07	3.87E-06	3467.63811
377.808447	0.54210567	0.10042488	5.39812143	6.73E-08	6.33E-07	293.522371
12182.1974	-0.1353553	0.03039772	-4.4528124	8.48E-06	5.59E-05	12434.4225
1955.58461	-0.3813395	0.05323074	-7.1638954	7.84E-13	1.40E-11	2181.51011
14702.403	0.19736036	0.02707867	7.28840727	3.14E-13	5.84E-12	13470.5472
3315.56165	0.27076848	0.04519096	5.99165091	2.08E-09	2.39E-08	2873.18586
7634.03667	-0.7543936	0.03437873	-21.943613	9.97E-107	4.83E-104	9689.01606
352.757282	-0.917715	0.11537856	-7.9539477	1.81E-15	4.16E-14	494.450934
35741.4624	0.22002068	0.02448766	8.98496316	2.59E-19	8.21E-18	33126.3607
238.649815	-0.7044137	0.12737571	-5.5302044	3.20E-08	3.13E-07	297.226123

750.079399	-0.1856449	0.0736339	-2.5211874	0.01169595	0.03599969	801.862377
9508.5281	0.38372036	0.03232287	11.8714829	1.66E-32	1.15E-30	8123.25477
1211.74759	-0.5309914	0.0602776	-8.8091	1.26E-18	3.81E-17	1441.68559
7604.05466	0.25996682	0.03612855	7.19560575	6.22E-13	1.12E-11	6692.68044
3682.72715	0.24226141	0.03900549	6.21095764	5.27E-10	6.60E-09	3347.26616
8861.4602	0.13964563	0.03724458	3.74942108	0.00017724	0.00089058	8196.40388
2283.81892	0.13308796	0.04588321	2.9005807	0.00372472	0.0133692	2154.65791
1397.43589	0.32859519	0.06188622	5.30966665	1.10E-07	9.98E-07	1285.20205
729.225625	-0.3159842	0.07507414	-4.2089618	2.57E-05	0.00015359	791.677058
2760.20678	0.12286768	0.04416436	2.78205491	0.00540159	0.01846598	2614.84914
1739.22622	0.18152228	0.05486424	3.3085717	0.00093773	0.0039543	1664.83667
2332.53099	-0.135707	0.05113304	-2.6539989	0.00795441	0.02578101	2404.66119
22.86058	-1.2860136	0.42184862	-3.0485192	0.00229972	0.00875321	35.185647
1914.94273	0.22239422	0.05376724	4.13624037	3.53E-05	0.00020505	1819.46833
862.88693	-1.322649	0.07362473	-17.96474	3.68E-72	1.03E-69	1258.34985
1084.71816	-0.5021988	0.06479757	-7.750272	9.17E-15	1.98E-13	1240.75703
4334.50741	0.34644533	0.0367305	9.43208877	4.02E-21	1.48E-19	3755.60485
29494.3904	0.19097894	0.03407477	5.60470241	2.09E-08	2.10E-07	26484.6069
171.215903	-0.786684	0.15018751	-5.2380122	1.62E-07	1.44E-06	206.484192
1520.65281	0.29466435	0.05340238	5.51781339	3.43E-08	3.34E-07	1395.38869
21295.5667	-0.1009691	0.02527447	-3.9949046	6.47E-05	0.00035521	22142.8832
65116.494	0.1690906	0.02628546	6.43285581	1.25E-10	1.72E-09	60642.4626
5945.51268	0.1773816	0.03231664	5.48886311	4.05E-08	3.91E-07	5604.70319
1112.29251	-0.2750559	0.06096032	-4.5120486	6.42E-06	4.34E-05	1227.79389
1620.9245	-0.3360475	0.05250549	-6.4002362	1.55E-10	2.10E-09	1801.8755
1703.4246	0.22592806	0.05132162	4.40220055	1.07E-05	6.93E-05	1564.83535
1267.19637	-0.2659515	0.05876225	-4.5258904	6.01E-06	4.08E-05	1355.57335
1604.04611	-0.4892534	0.0622114	-7.8643691	3.71E-15	8.31E-14	1997.24844
5187.20201	0.32640917	0.03993669	8.17316517	3.00E-16	7.42E-15	4610.2457
12622.7522	0.27535079	0.03030129	9.08709742	1.02E-19	3.34E-18	11239.0364
5215.41098	0.58152081	0.03975459	14.6277645	1.87E-48	2.28E-46	4085.23881
4677.16105	-0.1661368	0.03692332	-4.4995089	6.81E-06	4.59E-05	4924.1387
397.639334	-0.2727422	0.10203613	-2.6729958	0.00751772	0.02455384	475.006235
1838.74164	0.27129075	0.05255882	5.16166035	2.45E-07	2.10E-06	1690.76293
214.814671	0.96825033	0.13317384	7.27057474	3.58E-13	6.64E-12	147.224155
4718.44512	0.52869772	0.0348937	15.1516654	7.39E-52	1.07E-49	3907.45869
367.144685	0.60090613	0.10237581	5.86961068	4.37E-09	4.82E-08	287.040805
11206.2475	0.29778779	0.03938489	7.56096547	4.00E-14	8.17E-13	10096.4288
3746.89503	0.16178327	0.03894174	4.15449565	3.26E-05	0.00019075	3463.00842
2859.62879	-0.1247419	0.04206642	-2.9653554	0.00302333	0.01114632	2973.18717
3350.63414	0.15866098	0.0479321	3.31011953	0.00093256	0.00393421	3018.55814
2517.7768	-0.6453558	0.0489015	-13.197055	9.12E-40	8.50E-38	3015.78032
569.03565	-0.8663303	0.08557082	-10.124132	4.32E-24	1.87E-22	735.194835

3610.47456	0.10702276	0.03788908	2.82463328	0.00473348	0.01648849	3500.04594
517.469166	0.35621784	0.08753363	4.06949695	4.71E-05	0.00026565	430.561207
836.42098	0.33102311	0.07028011	4.71005424	2.48E-06	1.80E-05	707.416692
515.195704	0.41613564	0.08900508	4.67541444	2.93E-06	2.10E-05	438.89465
206.737009	0.57357935	0.14206441	4.03746	5.40E-05	0.00030133	161.113226
4691.01743	0.21436588	0.03668696	5.84310882	5.12E-09	5.59E-08	4268.57454
178.125321	-0.5652526	0.14671837	-3.8526368	0.00011685	0.00060958	202.780439
4041.19832	0.11679874	0.03877694	3.01206704	0.00259475	0.0097366	3851.90241
3288.24133	-0.1378673	0.0397111	-3.4717581	0.00051706	0.00232774	3381.52587
2576.14862	-0.3715592	0.04706432	-7.8947109	2.91E-15	6.59E-14	2895.40837
291.050615	-0.3721738	0.11535573	-3.2263136	0.00125396	0.00512719	338.893337
420.876547	0.46818083	0.09926965	4.71625351	2.40E-06	1.76E-05	348.152718
838.828584	-0.8044891	0.07258152	-11.083938	1.50E-28	8.74E-27	1022.23564
2292.46851	0.16752365	0.04966883	3.37281216	0.00074405	0.00321734	2025.02658
2186.67216	0.34069316	0.04723674	7.21246157	5.49E-13	9.99E-12	1932.43277
2544.9901	0.21680996	0.04518756	4.79800136	1.60E-06	1.21E-05	2316.69707
3579.08392	0.25815431	0.04490337	5.74910707	8.97E-09	9.50E-08	3226.89421
5110.57674	0.40665689	0.03735024	10.8876646	1.32E-27	7.31E-26	4304.68613
11921.6252	0.25457983	0.0423277	6.01449693	1.80E-09	2.09E-08	11164.9614
54607.0925	0.20507519	0.02610074	7.85706536	3.93E-15	8.79E-14	49223.7942
151.943155	-0.699282	0.16390859	-4.2662928	1.99E-05	0.00012188	162.039164
7386.97988	0.33923617	0.03367555	10.0736631	7.22E-24	3.10E-22	6388.97275
404.978751	-0.3578882	0.0983726	-3.6380886	0.00027467	0.0013235	475.006235
1225.73135	-0.2465165	0.06549457	-3.7639231	0.00016727	0.00084459	1284.27612
14.9146138	-4.354612	0.84359592	-5.1619643	2.44E-07	2.10E-06	28.7040805
118.726665	0.45816771	0.18032494	2.5407895	0.01106025	0.03429349	97.2234983
2035.07917	0.14146046	0.04965594	2.84881262	0.00438827	0.01545267	1954.65529
2120.67168	-0.1628192	0.05277391	-3.0852213	0.00203401	0.00785756	2117.62039
1011.82083	0.41032522	0.068028	6.03171102	1.62E-09	1.89E-08	867.60398
1073.31693	0.21302318	0.06265274	3.40006191	0.00067371	0.00295137	959.27185
760.451539	0.69045198	0.07500084	9.20592323	3.39E-20	1.14E-18	548.155343
199.519664	0.43748255	0.14050922	3.11355048	0.00184851	0.00722301	156.483535
1027.36665	-0.1666615	0.06318098	-2.6378433	0.00834351	0.02683574	1070.38442
1710.57071	-0.3624012	0.05232208	-6.9263535	4.32E-12	7.02E-11	1945.3959
12714.9574	0.10947031	0.03088963	3.54391796	0.00039423	0.00182957	12462.2006
78.0389735	-0.5841	0.22451417	-2.6016177	0.00927852	0.02950044	99.0753745
23746.981	0.27424169	0.02976118	9.21474526	3.12E-20	1.06E-18	21166.0186
1192.97235	-0.1580927	0.06083058	-2.5989024	0.00935224	0.02971278	1294.46144
2701.70195	0.13407189	0.0427101	3.13911465	0.00169459	0.00668941	2625.03445
2044.62792	0.16503308	0.04940718	3.34026534	0.00083698	0.00357693	1868.54304
2118.06091	0.19767715	0.05247935	3.76676061	0.00016538	0.00083639	1962.06279
125.451594	-0.9784139	0.17836361	-5.4855017	4.12E-08	3.99E-07	184.261678
10517.0892	0.28713596	0.02886659	9.94699953	2.60E-23	1.08E-21	9360.30804

3354.73063	0.19911984	0.04209207	4.73057868	2.24E-06	1.65E-05	3178.74543
1186.63959	0.32326286	0.06755612	4.78510089	1.71E-06	1.28E-05	1011.12438
7691.58801	-0.2606978	0.03107419	-8.3895273	4.88E-17	1.29E-15	8516.77845
6524.32908	-0.1338435	0.03860023	-3.4674277	0.00052547	0.00236173	7016.75876
5052.20138	0.14535502	0.03515742	4.13440527	3.56E-05	0.00020648	4713.02482
156.67443	-0.9450684	0.15964556	-5.9197912	3.22E-09	3.61E-08	195.372935
3427.04575	0.22259448	0.04185864	5.31776691	1.05E-07	9.58E-07	3105.59632
1658.62374	-2.633982	0.06672444	-39.47552	0	0	2918.55683
13468.6988	0.204052	0.02965788	6.88019572	5.98E-12	9.58E-11	12159.4189
7149.78991	0.15933408	0.03804236	4.18833318	2.81E-05	0.00016694	6687.12481
5307.54519	0.25671981	0.04162944	6.16678447	6.97E-10	8.61E-09	4815.80395
5290.18429	0.31050635	0.03738061	8.30661653	9.85E-17	2.51E-15	4547.28191
3754.18894	0.09329302	0.03882862	2.40268688	0.01627512	0.04786	3577.82474
2135.25207	0.27467916	0.04853128	5.65983733	1.52E-08	1.56E-07	1864.83929
4449.96534	0.37094719	0.03782711	9.80638522	1.06E-22	4.26E-21	3761.16048
2533.2757	0.40159514	0.04503617	8.91716964	4.78E-19	1.49E-17	2148.17634
11247.4364	-0.1629768	0.03080512	-5.2905767	1.22E-07	1.10E-06	11605.7079
2552.35077	-0.1984792	0.04524892	-4.3863846	1.15E-05	7.41E-05	2705.59107
2337.82832	0.25599991	0.04552711	5.62302176	1.88E-08	1.90E-07	2100.02756
65.4159175	-1.1242362	0.25399558	-4.4262038	9.59E-06	6.26E-05	92.5938079
3493.853	0.22056941	0.04182502	5.27362393	1.34E-07	1.19E-06	3273.19111
3240.82715	-0.1302288	0.04038945	-3.2243263	0.00126269	0.00515638	3403.74838
136.991717	0.52948054	0.17967693	2.94684767	0.00321031	0.0117463	112.038508
22878.5126	0.22106994	0.02717486	8.13509121	4.12E-16	1.01E-14	21203.982
628.851096	0.78862788	0.079096	9.97051506	2.05E-23	8.59E-22	475.932173
1930.60771	0.28388961	0.05000983	5.67667634	1.37E-08	1.42E-07	1780.57893
502.143486	0.2355083	0.08696504	2.7080801	0.00676737	0.02243253	450.005906
1692.365	-0.4219463	0.05643707	-7.476404	7.64E-14	1.51E-12	1942.61809
8363.68738	0.12521401	0.03403518	3.67895863	0.00023419	0.00114785	7944.54872
98.4859292	-1.3863556	0.20474305	-6.7711977	1.28E-11	1.98E-10	150.001969
1133.79873	-0.196733	0.06638642	-2.9634523	0.00304209	0.01120695	1127.79258
52.7456198	-0.6864134	0.27122779	-2.5307636	0.01138145	0.03516007	68.5194179
898.022939	-0.3705947	0.07298377	-5.0777693	3.82E-07	3.17E-06	947.234655
2860.47441	0.33852409	0.04586341	7.38113582	1.57E-13	3.00E-12	2430.58746
4384.83091	0.21803352	0.03777924	5.77125306	7.87E-09	8.40E-08	4050.05316
1665.15665	0.24469524	0.0569045	4.30010329	1.71E-05	0.00010617	1469.46373
5397.0817	0.17110383	0.03424284	4.99677664	5.83E-07	4.70E-06	4972.28749
4150.35611	0.24584103	0.03702107	6.64057031	3.12E-11	4.59E-10	3721.34514
10013.2576	0.23416435	0.03174781	7.37576322	1.63E-13	3.11E-12	8951.96935
4956.33899	0.26230553	0.03844619	6.82266638	8.94E-12	1.41E-10	4418.57651
3166.66053	0.23416671	0.04539671	5.15823056	2.49E-07	2.13E-06	2833.37052
1070.54523	0.32535703	0.06231746	5.22096106	1.78E-07	1.56E-06	921.308389
12683.6418	0.23585906	0.02871259	8.21448251	2.13E-16	5.31E-15	11770.5249

28201.7505	0.28628011	0.03166566	9.04071209	1.56E-19	5.05E-18	25262.3686
1726.80662	0.35813462	0.05351194	6.69261166	2.19E-11	3.30E-10	1517.61251
8844.02429	0.0792665	0.03235846	2.44963804	0.01429999	0.04277199	8559.3716
180.986026	-0.5830262	0.14670737	-3.9740756	7.07E-05	0.00038461	208.336068
11314.5231	-0.0857745	0.02897009	-2.9607951	0.00306846	0.01128479	11555.7072
739.766498	-0.5016564	0.07610352	-6.5917633	4.35E-11	6.27E-10	849.085219
6421.84398	0.14194724	0.0356707	3.97937883	6.91E-05	0.0003773	5997.30094
565.366394	4.16468297	0.12751569	32.6601622	5.75E-234	3.71E-230	63.8897275
29.0721481	3.94076566	0.52687456	7.4795141	7.46E-14	1.48E-12	6.48156655
902.962856	-0.3678226	0.06791598	-5.4158478	6.10E-08	5.77E-07	1032.42096
3252.46551	-0.6198545	0.04047479	-15.314583	6.11E-53	9.40E-51	3872.27305
7191.00893	0.17896812	0.03373025	5.30586369	1.12E-07	1.02E-06	6726.94014
4623.88151	0.27999692	0.03840379	7.29086635	3.08E-13	5.74E-12	4062.09035
6221.81725	0.23575138	0.03514564	6.70784071	1.98E-11	3.00E-10	5843.59522
20.2504319	-1.8779203	0.46547383	-4.0344273	5.47E-05	0.00030472	28.7040805
177.750918	-0.9111185	0.15170572	-6.0058287	1.90E-09	2.20E-08	241.669839
296.539732	-1.3564334	0.12273886	-11.051377	2.16E-28	1.25E-26	397.227436
3580.28051	0.20183744	0.03960211	5.09663417	3.46E-07	2.89E-06	3361.15523
71730.9698	-0.1582322	0.02625496	-6.0267572	1.67E-09	1.94E-08	75723.2161
3379.77161	0.13748427	0.04169611	3.29729214	0.00097622	0.00409831	3078.74411
1611.99458	0.2139231	0.05356477	3.99372796	6.50E-05	0.00035688	1559.27973
2706.17035	0.15100004	0.04436357	3.40369429	0.00066481	0.00291767	2530.58877
3127.06586	0.1831328	0.04218218	4.34147257	1.42E-05	8.94E-05	2886.14899
4188.1112	-0.1165286	0.04092983	-2.8470326	0.00441288	0.01551392	4227.83327
2002.32626	0.18146946	0.0478655	3.79123697	0.0001499	0.00076468	1856.50585
954.951564	-0.2179135	0.07163152	-3.0421447	0.00234899	0.00891099	993.531559
2441.92555	0.14188457	0.04847459	2.92698842	0.00342262	0.01241846	2431.5134
82.2112371	-1.8510009	0.24671967	-7.5024457	6.26E-14	1.26E-12	140.742588
230.395935	-1.1774091	0.13576539	-8.6723806	4.23E-18	1.23E-16	306.485504
3537.46348	0.29422274	0.04084422	7.20353379	5.87E-13	1.06E-11	3088.00349
162.205543	0.72896528	0.15497253	4.70383533	2.55E-06	1.86E-05	130.557269
979.961648	0.64927295	0.06649239	9.7646206	1.60E-22	6.37E-21	750.009844
7224.35477	0.15327011	0.03435534	4.46131787	8.15E-06	5.40E-05	6609.34601
3511.97475	0.20386852	0.0388742	5.24431353	1.57E-07	1.39E-06	3305.59894
5049.96974	0.12937272	0.03715611	3.48186908	0.00049793	0.00225363	4728.76577
3354.92868	0.16458878	0.04528859	3.63422156	0.00027882	0.00134118	3074.11442
12349.7498	0.13507345	0.02812442	4.80271144	1.57E-06	1.18E-05	11618.671
1804.10393	-0.3169268	0.05058126	-6.2656959	3.71E-10	4.76E-09	1975.95186
1009.66148	-0.2592396	0.0633206	-4.0940795	4.24E-05	0.00024159	1087.05131
300.586835	-0.3896838	0.11664577	-3.3407455	0.00083554	0.00357153	348.152718
1732.15451	-0.2694402	0.05396331	-4.993026	5.94E-07	4.78E-06	1817.61645
1231.77352	-0.3110264	0.05803303	-5.359472	8.35E-08	7.73E-07	1370.38836
50.2255315	0.92304978	0.28824606	3.20229801	0.00136336	0.00551863	35.185647

933.291668	-0.1838447	0.06951321	-2.6447445	0.00817526	0.02637774	1036.12471
4277.5731	0.26425081	0.03713051	7.11681116	1.10E-12	1.94E-11	3838.01334
337.373897	-0.5239587	0.11072098	-4.7322437	2.22E-06	1.63E-05	420.375888
2712.66375	0.18618504	0.04679762	3.97851475	6.93E-05	0.00037825	2571.33005
1005.63976	-0.9335963	0.06509763	-14.34148	1.20E-46	1.41E-44	1330.57302
1352.45331	-0.6352337	0.05849647	-10.859351	1.80E-27	9.89E-26	1583.35412
4222.35082	0.32338729	0.04617545	7.00344571	2.50E-12	4.20E-11	3747.27141
191.427513	-1.5707468	0.15540552	-10.107407	5.12E-24	2.21E-22	278.707362
7920.33055	-0.9174629	0.03157742	-29.054396	1.35E-185	2.92E-182	10261.2458
5202.19649	0.34087351	0.03637634	9.37074817	7.20E-21	2.58E-19	4546.35597
3996.61113	0.13382141	0.03981657	3.36094787	0.00077676	0.00334386	3704.67825
15346.5897	-0.5953717	0.03754937	-15.855701	1.28E-56	2.37E-54	18256.7211
64.0267633	-1.2805088	0.25143775	-5.0927469	3.53E-07	2.94E-06	98.1494364
517.696703	-0.2470973	0.08783039	-2.8133467	0.00490288	0.01699307	584.266928
4165.22043	0.20511532	0.03681074	5.57216036	2.52E-08	2.51E-07	3827.82802
4282.33799	0.25048001	0.03775714	6.63397741	3.27E-11	4.78E-10	3811.16113
15494.3457	0.25258322	0.03114215	8.11065553	5.03E-16	1.22E-14	14014.0728
23927.1783	0.13644598	0.02887819	4.72487926	2.30E-06	1.69E-05	22147.5129
13933.6945	0.20097858	0.02952702	6.80659898	9.99E-12	1.57E-10	12691.8333
1844.65391	-0.1203475	0.04962176	-2.4252964	0.01529589	0.04531727	1883.35805
7939.59004	0.19314179	0.03346428	5.77158069	7.85E-09	8.39E-08	7147.31603
1626.70771	0.32500094	0.05466858	5.94493106	2.77E-09	3.11E-08	1394.46275
851.391139	0.23328101	0.08808985	2.64821655	0.00809177	0.02616136	737.972649
14585.7483	0.31030467	0.03315845	9.35823919	8.11E-21	2.89E-19	12727.0189
896.146864	0.94360146	0.07323346	12.8848405	5.48E-38	4.68E-36	598.155999
3448.82828	0.18252534	0.04370032	4.17675104	2.96E-05	0.00017478	3193.56044
2896.0978	0.32290896	0.04208189	7.67334771	1.68E-14	3.53E-13	2568.55223
4829.88253	0.3651614	0.03661845	9.97205928	2.02E-23	8.48E-22	4148.20259
1023.15541	0.23954362	0.06430596	3.72506076	0.00019527	0.0009723	899.085875
30565.082	0.20343572	0.02757961	7.37630877	1.63E-13	3.11E-12	28028.1457
9160.80604	0.23262324	0.03137447	7.41441113	1.22E-13	2.36E-12	8194.552
2127.33575	0.13200517	0.04879339	2.7053906	0.00682241	0.02258795	1998.17438
7895.46382	0.24172249	0.03336939	7.24384007	4.36E-13	8.01E-12	7294.54019
1853.54475	0.36339057	0.05093004	7.13509314	9.67E-13	1.71E-11	1691.68887
1832.62028	-0.3841239	0.05423891	-7.0820725	1.42E-12	2.46E-11	2092.62006
3007.81702	-0.1508296	0.04120478	-3.6604869	0.00025174	0.00122334	3238.00546
340.156485	0.2593425	0.10709513	2.42160869	0.01545198	0.04573777	301.855814
1342.51207	0.31431721	0.06318653	4.9744334	6.54E-07	5.23E-06	1258.34985
331.040893	0.42729358	0.10740543	3.97832366	6.94E-05	0.00037834	277.781424
729.983868	0.50347786	0.07642726	6.58767383	4.47E-11	6.44E-10	642.601027
1607.33368	0.22193858	0.05295226	4.19129603	2.77E-05	0.00016493	1475.01936
1922.65004	-0.2872568	0.05413525	-5.3062797	1.12E-07	1.01E-06	2183.36199
3723.00933	0.24039656	0.03929103	6.11835667	9.45E-10	1.15E-08	3356.52554

1170.73126	-0.2504486	0.0608327	-4.1170056	3.84E-05	0.00022131	1279.64643
1976.33706	0.18788477	0.05006801	3.75259076	0.00017502	0.00088053	1831.50552
2429.30436	0.22846713	0.04537107	5.03552443	4.77E-07	3.89E-06	2200.95481
2315.1834	0.2115088	0.04999572	4.23053804	2.33E-05	0.00014074	2262.06673
954.323548	-0.3553446	0.0679819	-5.2270467	1.72E-07	1.51E-06	1055.56941
1320.93967	0.28310158	0.06370593	4.44388091	8.84E-06	5.81E-05	1212.05295
1508.35862	0.19586912	0.0539122	3.6331132	0.00028002	0.00134595	1396.31462
5848.70549	0.11894673	0.03423824	3.47408974	0.00051259	0.00230975	5588.03631
1400.34917	-0.6776377	0.05696647	-11.895379	1.25E-32	8.69E-31	1739.83765
5530.31169	0.16588099	0.03356531	4.94203742	7.73E-07	6.11E-06	5206.54982
3416.03002	0.15109665	0.04025266	3.75370557	0.00017424	0.00087708	3329.67333
4074.72543	0.21798345	0.03791199	5.74972303	8.94E-09	9.47E-08	3810.2352
4382.47557	0.13711699	0.04191987	3.2709308	0.00107194	0.0044515	4093.57225
3538.84254	0.25807641	0.04083199	6.32044682	2.61E-10	3.41E-09	3230.59796
1842.56894	-0.1583395	0.05021717	-3.1530955	0.00161549	0.00641201	1955.58122
1908.47997	0.22563565	0.05016305	4.49804474	6.86E-06	4.61E-05	1775.0233
916.291524	1.06249611	0.07000641	15.1771264	5.01E-52	7.36E-50	612.971008
3560.22645	0.1641685	0.0395337	4.15262177	3.29E-05	0.00019226	3425.97089
7116.55012	0.4067591	0.03113927	13.0625778	5.39E-39	4.83E-37	6040.82003
102.060771	0.48632875	0.20330993	2.39205602	0.01675429	0.04903847	74.0750463
4651.8285	0.17141985	0.03850233	4.4521938	8.50E-06	5.60E-05	4237.09265
5140.45085	-0.1627723	0.0384487	-4.2334931	2.30E-05	0.00013904	5250.06891
342.918607	0.45408722	0.11304304	4.01694108	5.90E-05	0.00032645	274.077671
1405.61966	0.21770708	0.05522963	3.94185258	8.09E-05	0.00043586	1296.31331
36.9599645	1.40916284	0.32578725	4.32540815	1.52E-05	9.55E-05	20.3706377
1332.56172	0.20956985	0.05868996	3.57079578	0.0003559	0.00166927	1202.79357
6027.46276	0.28945396	0.03673844	7.87877614	3.31E-15	7.45E-14	5318.58833
1627.5476	0.23145091	0.05302188	4.36519589	1.27E-05	8.09E-05	1450.01903
1119.5416	-0.1936646	0.06120395	-3.16425	0.00155483	0.00620301	1169.45979
981.476474	0.54593584	0.0684303	7.97798393	1.49E-15	3.46E-14	798.158624
900.050944	-0.3395024	0.0711048	-4.7746766	1.80E-06	1.34E-05	1026.86533
3390.81916	0.22195539	0.04286238	5.17832585	2.24E-07	1.93E-06	3136.15227
260.943731	-0.38693	0.12018478	-3.219459	0.00128433	0.0052304	291.670495
110.128929	-0.6352614	0.18744806	-3.3889998	0.00070148	0.00305509	125.001641
1224.97089	0.28536824	0.05865743	4.86499778	1.14E-06	8.83E-06	1115.75539
973.208576	-0.3701813	0.06872906	-5.386096	7.20E-08	6.72E-07	1077.79192
24.5027716	-2.0771394	0.44221064	-4.697172	2.64E-06	1.91E-05	43.5190897
4113.06146	0.3974259	0.04014777	9.8990777	4.20E-23	1.72E-21	3550.97253
3668.72543	0.33848216	0.04148017	8.16009629	3.35E-16	8.25E-15	3132.44852
1378.95916	-0.1992359	0.05535496	-3.5992418	0.00031915	0.00151485	1447.24122
11216.3746	0.07822171	0.03045173	2.56871194	0.01020773	0.03203356	11000.1444
4344.042	0.26435029	0.0398397	6.63534801	3.24E-11	4.74E-10	4003.75625
30.6172444	-1.2511562	0.38220218	-3.2735454	0.00106207	0.00441619	43.5190897

8135.82067	0.29757814	0.032264	9.22322472	2.88E-20	9.87E-19	7143.61228
2397.23272	-0.4052541	0.04453983	-9.0986897	9.14E-20	3.01E-18	2710.22076
2598.83237	-0.7711885	0.04483714	-17.19977	2.67E-66	6.46E-64	3217.63483
141.485953	-0.9400517	0.16568959	-5.6735712	1.40E-08	1.44E-07	193.521059
504.976713	-0.2695242	0.09394602	-2.8689256	0.00411869	0.01459345	513.895634
39125.828	0.22584611	0.02564386	8.80702469	1.29E-18	3.87E-17	36796.7793
3051.9993	0.20343137	0.05280573	3.85244899	0.00011694	0.00060988	2712.99857
620.337368	-0.3229436	0.08261243	-3.9091411	9.26E-05	0.00049287	654.638222
6015.38349	0.22330444	0.03390672	6.58584648	4.52E-11	6.51E-10	5551.92472
1079.09661	-0.3242649	0.06348977	-5.107357	3.27E-07	2.74E-06	1174.08948
1165.18496	0.31520368	0.05981467	5.26967139	1.37E-07	1.22E-06	1048.16191
2111.42168	0.30371124	0.0502275	6.04671255	1.48E-09	1.74E-08	1873.17273
198.012942	-0.6485835	0.13847007	-4.6839254	2.81E-06	2.02E-05	238.892024
544.454574	0.28823478	0.08770216	3.28651866	0.00101434	0.0042432	450.005906
735.089831	-0.4493666	0.0774319	-5.8033783	6.50E-09	7.02E-08	819.4552
640.554915	0.27764483	0.07877778	3.52440554	0.00042443	0.00195385	608.341318
1383.67493	0.18516504	0.05667503	3.26713632	0.00108641	0.0045058	1290.75768
2293.92655	0.21005173	0.04577175	4.58911336	4.45E-06	3.09E-05	2100.02756
2945.29977	-0.341599	0.04278371	-7.9843241	1.41E-15	3.30E-14	3336.1549
2888.90528	0.11402955	0.04546155	2.50826357	0.01213261	0.03717255	2795.40706
1206.12868	0.28238409	0.0661071	4.27161549	1.94E-05	0.00011939	1044.45815
1334.79335	0.14810576	0.05753183	2.57432737	0.01004352	0.03158047	1232.42358
602.129703	-0.2228336	0.08673131	-2.5692406	0.01019217	0.0319908	593.526309
372.852796	-0.3877432	0.10526722	-3.683418	0.00023013	0.00112937	414.820259
2093.61948	-0.2169014	0.04787476	-4.5306006	5.88E-06	4.00E-05	2210.2142
1268.69884	0.25481595	0.05773023	4.41390877	1.02E-05	6.59E-05	1169.45979
849.10768	-0.2739133	0.07019707	-3.9020612	9.54E-05	0.00050627	898.159937
86.2406895	-0.6886497	0.21534203	-3.1979343	0.00138416	0.00558765	92.5938079
2079.65682	-0.2223704	0.05157466	-4.311622	1.62E-05	0.00010124	2307.43769
1110.27997	-0.4937763	0.06276328	-7.867281	3.62E-15	8.13E-14	1321.31364
1334.92182	0.2218904	0.05653206	3.92503644	8.67E-05	0.00046449	1237.97921
167.596624	-0.3889264	0.15333738	-2.5364093	0.01119958	0.03464237	185.187616
631.095769	0.29571434	0.08694142	3.40130552	0.00067065	0.00294063	586.118804
301.117562	-0.3472121	0.11821801	-2.9370493	0.00331351	0.0120715	330.559894
1404.58687	-0.1783309	0.06032383	-2.9562257	0.00311429	0.01143598	1421.31495
58.5458539	-0.6755133	0.25420723	-2.6573331	0.00787616	0.02556586	75.0009844
540.528312	-0.3956463	0.08597417	-4.6019205	4.19E-06	2.92E-05	627.786018
1495.41164	0.35611789	0.05874499	6.06209785	1.34E-09	1.59E-08	1346.31397
695.074509	0.5071165	0.08028661	6.31632691	2.68E-10	3.50E-09	594.452247
175.964667	-0.3876042	0.15228024	-2.5453351	0.01091729	0.0339099	225.002953
2208.68313	0.26334881	0.04895857	5.37901319	7.49E-08	6.97E-07	2032.43408
2536.3197	0.32524894	0.04667556	6.96829168	3.21E-12	5.30E-11	2237.99234
684.755768	-0.2706937	0.07895348	-3.4285215	0.00060688	0.00269145	775.93611

213.263022	-0.4353942	0.13317674	-3.2692961	0.00107815	0.00447502	253.707034
467.539037	-0.3225846	0.09136047	-3.5308993	0.00041415	0.00191241	535.19221
712.286178	-0.3488488	0.07962582	-4.3811018	1.18E-05	7.58E-05	764.824853
1351.20701	0.31359782	0.05923939	5.29373811	1.20E-07	1.08E-06	1202.79357
4879.44964	0.21954506	0.03732991	5.88120998	4.07E-09	4.50E-08	4429.68777
5533.55051	0.37831112	0.03659177	10.3386931	4.71E-25	2.17E-23	4782.47018
261.777494	0.33965334	0.1198716	2.83347626	0.00460447	0.01609407	225.928891
1308.52589	0.31911792	0.05669796	5.62838445	1.82E-08	1.85E-07	1154.64479
1613.40839	0.19846011	0.05409565	3.66868886	0.0002438	0.00118834	1531.50158
1554.43335	0.18102714	0.05502611	3.28984089	0.00100244	0.00419976	1412.98151
4227.16829	0.15959978	0.03651047	4.37134327	1.23E-05	7.89E-05	4020.42314
9840.19788	0.22364692	0.031263	7.15372528	8.45E-13	1.50E-11	8975.1178
1480.02341	0.25879328	0.05586257	4.63267797	3.61E-06	2.55E-05	1411.12963
1491.47672	0.37823456	0.05525327	6.84546912	7.62E-12	1.21E-10	1306.49863
1309.55394	-0.188736	0.06231092	-3.028939	0.00245414	0.00927179	1364.83273
386.174474	0.28407898	0.1022753	2.77759135	0.00547634	0.01866669	329.633956
360.372746	-0.7843471	0.10584996	-7.4099898	1.26E-13	2.43E-12	453.709659
6605.88936	0.2611818	0.03710293	7.03938528	1.93E-12	3.29E-11	6037.11628
1165.90751	0.3165807	0.0633205	4.99965566	5.74E-07	4.63E-06	1085.19943
1097.75051	-0.2737901	0.06156075	-4.4474782	8.69E-06	5.72E-05	1181.49699
1419.18237	-0.2185726	0.05623656	-3.8866648	0.00010163	0.00053594	1479.64905
974.726753	0.18190156	0.06516916	2.79122157	0.00525095	0.01803034	877.789299
2233.52539	0.30563433	0.05018392	6.09028413	1.13E-09	1.35E-08	2102.80538
454.226681	-0.4477744	0.09506166	-4.7103578	2.47E-06	1.80E-05	522.229077
1353.52503	-0.331632	0.05819274	-5.6988545	1.21E-08	1.26E-07	1563.90942
1113.58719	-0.3677418	0.06168253	-5.9618469	2.49E-09	2.83E-08	1269.46111
33.0970724	-1.6034652	0.37377892	-4.289876	1.79E-05	0.00011087	63.8897275
2759.17987	-0.3090503	0.04493619	-6.877536	6.09E-12	9.75E-11	3066.70692
707.041224	-0.332275	0.07654682	-4.3408074	1.42E-05	8.96E-05	777.787986
2529.85782	-0.1528979	0.04418166	-3.460664	0.00053885	0.00241682	2649.10884
1022.57747	0.17443988	0.06757963	2.58124954	0.00984434	0.03104482	915.75276
391.902974	-0.3008895	0.09997309	-3.0097044	0.00261502	0.00980506	431.487145
1603.10087	0.24554609	0.05358609	4.58227253	4.60E-06	3.18E-05	1503.72344
706.539881	0.31804614	0.07618437	4.17469	2.98E-05	0.00017608	597.230061
359.839759	-0.3624942	0.10466802	-3.4632758	0.00053364	0.00239459	413.894321
5923.13024	-0.1123087	0.03510821	-3.1989283	0.00137939	0.00557306	6074.1538
859.069762	0.26170432	0.07121065	3.67507279	0.00023778	0.00116311	805.566129
3770.11733	-0.1452486	0.03814036	-3.8082647	0.00013995	0.00071882	3887.08806
680.805087	-0.25533	0.07682242	-3.3236394	0.00088851	0.00377135	755.565473
441.66538	-0.4614625	0.09517877	-4.8483768	1.24E-06	9.54E-06	499.080625
448.762146	0.22874953	0.09259307	2.4704822	0.0134931	0.04068499	429.635269
395.398235	-0.2736302	0.10049339	-2.7228673	0.00647181	0.02159682	412.042445
1112.18544	0.20422181	0.0773171	2.64135381	0.00825754	0.02660337	957.419974

1925.53647	0.19842183	0.05224661	3.79779329	0.00014599	0.00074612	1885.20993
2738.77221	0.20921431	0.04249838	4.92287672	8.53E-07	6.71E-06	2524.1072

Control.1	Control.2	shRNA2.0	shRNA2.1	shRNA2.2
12387.9067	12557.8747	11805.2751	11617.1742	10824.0297
732.859692	755.460535	1118.99811	1009.6582	1016.31566
337.336421	323.611019	497.504701	439.553521	499.227114
17042.8547	18064.7844	18847.8339	18773.0679	18789.3368
422.77534	445.10321	522.302443	509.994791	566.207497
26.5155266	26.5073872	12.3988711	11.2706031	1.78614352
5320.04579	5205.38816	5720.52914	5656.90355	5742.45142
189.291398	163.462221	271.225305	297.731766	248.27395
14078.2715	14475.2424	17888.4712	17741.8077	17437.2261
7149.61712	7066.42764	4934.75068	5151.60484	4934.22148
4060.55828	4034.64523	2699.85417	2762.23698	2691.71829
7500.94785	7650.69463	9257.30711	9002.39424	8541.33832
12906.4326	14476.3468	11630.1411	11044.2518	10752.584
288.724623	295.999157	111.58984	143.70019	129.495405
4681.46352	4813.29973	2971.07948	2889.97048	2812.28298
468.440969	432.953991	801.277042	771.097096	750.180279
1783.16916	1772.68152	1565.35747	1520.5922	1480.71298
1744.86896	1704.2041	1509.56255	1507.44317	1511.07742
6159.70413	5999.5053	7161.8979	7025.34261	6966.85281
3547.92476	3711.03421	4167.57054	3939.07579	4090.26867
1546.73905	1513.13002	508.353714	523.143828	440.284378
3646.62144	3683.42235	3256.25351	3288.19846	3374.91819
4696.19437	4871.83687	5697.28125	5476.5739	5205.7153
3711.43718	3538.73619	5523.69706	5575.19167	5276.26796
6153.07525	6190.57939	6696.94023	6586.7283	6484.59406
1901.01595	1697.57726	1895.47741	2198.70682	2237.14476
693.086403	731.162097	480.456254	494.028103	489.403325
6217.89098	6470.01143	6771.33346	6655.29114	6772.16316
1509.91193	1495.45843	1306.53104	1354.35081	1364.61365
87.6485461	83.9400595	40.296331	45.0824124	39.2951575
244.532078	203.223302	156.535747	167.180613	163.432132
1319.88399	1367.33939	1461.51693	1492.4157	1511.97049
962.660923	934.385399	1094.20037	1185.29176	1134.20114
33.1444082	36.4476574	15.4985888	13.149037	17.8614352
211.38767	225.312791	159.635465	159.666877	137.533051
6279.024	6536.2799	7919.77889	7735.3906	7974.23775
5487.97746	5549.9842	6603.9487	6456.17715	6021.98289
9011.59632	9354.89874	11506.1523	11397.3974	11588.4992
36.0905778	33.134234	4.64957665	9.39216926	6.25150233
957.505126	1153.07134	1647.49999	1504.62552	1347.64529
1104.07706	1140.92212	1512.66227	1406.00774	1406.58802
43.4560019	44.1789787	9.2991533	18.7843385	16.9683635

10482.4715	10684.686	11236.4769	11363.5856	11645.6558
3012.45843	3491.24379	3690.214	3924.98753	3850.92543
916.258751	914.504859	734.63311	719.440165	677.841467
3031.60854	3127.87169	3699.51315	3823.55211	3965.23862
598.808975	554.446182	705.185792	702.534261	686.772184
843.341053	860.38561	626.142989	653.69498	630.508663
3783.61833	4067.77946	3583.27374	3611.28908	3537.45725
547.987549	504.744831	443.25964	462.094728	469.755746
1263.17022	1362.92149	1661.44872	1607.00016	1651.28969
26.5155266	25.4029127	7.74929441	8.45295233	2.67921528
75.1273253	71.7908404	35.6467543	41.3255447	29.4713681
700.451827	728.953148	528.501879	504.359489	537.6292
81.7562069	90.5669063	40.296331	46.0216294	58.9427362
4227.7534	4229.03273	4874.30619	4691.38855	4852.05888
912.576039	864.803508	695.886638	675.29697	713.564337
15611.0163	14997.6588	17452.9609	18238.6535	18165.0796
1480.45023	1671.06987	1167.04374	1196.56236	1261.01733
198.866449	173.402491	125.53857	120.219767	110.740898
464.021715	525.729846	336.319378	444.249606	375.983211
1022.32086	879.161676	664.889461	620.822388	599.251152
699.715284	766.50528	540.90075	461.155511	432.246732
2726.67998	2690.4998	2180.65145	2203.40291	2187.13274
4146.73374	3863.45169	3491.83206	3415.93196	3376.70433
9583.88977	9829.82276	6664.3932	6469.32619	6663.20841
136.260345	138.059308	85.2422386	74.1981372	94.6656067
2667.75659	2990.91686	2442.5776	2363.06979	2300.55286
885.32397	825.042427	1236.78739	1385.34497	1412.83953
276.203402	267.282821	395.214015	401.984844	398.310005
549.460634	563.281978	413.812322	424.526051	419.743728
4691.03857	4787.89681	3759.95765	3600.95769	3532.09882
9966.89182	10189.8814	12753.7888	12405.1772	11973.4131
1601.24319	1566.14479	1887.72812	1771.36312	1823.65254
2301.69501	2289.57557	2485.97365	2523.67588	2554.18524
1084.92696	1125.45948	979.510814	1045.34844	927.008488
2314.21624	2380.14248	2132.60582	2036.2223	2011.19761
1964.35859	1969.27797	1881.52868	1698.1042	1735.23843
977.391771	989.609122	804.37676	834.02463	801.08537
66.2888164	54.1192489	20.1481655	21.6019893	15.1822199
278.413029	260.655974	395.214015	326.84749	363.480207
230.537773	198.805404	6.19943553	15.0274708	11.6099329
68.4984436	73.9997893	125.53857	129.611936	137.533051
25898.304	26755.894	28432.1612	28141.7568	27888.845
2784.13029	2784.38013	2571.21589	2499.25624	2561.32981

5059.30978	5114.82126	5674.03337	5508.50727	5596.88073
1421.52684	1438.02576	1601.00423	1832.41222	1651.28969
644.474604	533.461168	743.932264	820.875593	730.532701
396.259814	456.147955	283.624176	332.482792	302.751327
50.8214259	34.2387085	23.2478832	15.9666877	16.0752917
287.98808	321.40207	159.635465	196.296338	154.501415
157.620075	151.313002	266.575728	203.810073	218.802582
2896.08473	2958.8871	3442.23658	3480.73793	3492.80366
36.8271202	26.5073872	10.8490122	12.20982	14.2891482
6076.47484	6213.77335	7120.05171	6836.56	6841.82276
58.18685	69.5818914	85.2422386	99.5569942	112.527042
764.531016	789.699244	1041.50517	934.520841	947.549139
581.131957	563.281978	323.920507	286.461162	317.040475
7665.93335	8176.42448	9940.79487	9682.38729	9350.46134
81.0196645	73.9997893	116.239416	116.462899	102.703253
3816.76274	3727.60133	4682.12368	4769.34355	4842.23509
4615.17471	4688.49411	4844.85887	4961.88302	4960.12056
4685.14623	4436.67393	4979.69659	4901.77314	5128.91112
7458.22839	7612.03803	8556.77089	8235.99322	8414.52213
335.863336	328.028917	196.832078	207.566941	217.016438
140.679599	112.656396	91.4416741	96.7393434	87.5210326
9714.25777	10101.5235	11906.0159	11800.3215	11902.8604
16267.2755	16167.2973	20749.5107	19901.0674	19550.2339
2088.83426	2070.88963	2248.84524	2329.25798	2303.23207
2773.8187	2696.02217	3236.10535	3211.18267	3061.45
450.027409	425.22267	689.687203	576.679193	620.684874
5390.75386	5652.70032	6121.94259	6543.52432	6407.78989
8867.23401	8660.1843	8107.31182	7875.33392	7866.17607
1853.14069	1817.96497	2244.19566	2163.9558	2113.00779
2612.51591	2689.39533	2278.29256	2331.13641	2236.25169
1073.87883	1171.84741	1370.07525	1313.96448	1310.13627
167.195126	188.865134	106.940263	109.88838	111.63397
457.392833	452.834531	358.017402	378.504421	325.078121
474.333308	513.580627	364.216837	321.212189	363.480207
95.0139702	99.402702	58.8946375	57.2922325	58.9427362
374.163541	311.4618	237.128409	221.655195	228.626371
218.753094	188.865134	123.988711	139.004105	131.281549
357.959609	340.178136	584.296799	602.03805	639.439381
2379.76851	2173.60575	2811.44401	2750.02716	2722.08273
1259.48751	1240.32483	1013.60771	973.028735	965.410574
2377.55888	2448.61989	2848.64063	2680.52511	2642.59934
5675.79577	5931.02789	6749.63543	6813.07958	6722.15115
798.411966	832.773748	537.801032	547.563468	576.924358

1030.42282	1178.47426	1332.87864	1238.82713	1203.86073
8535.78992	8554.15475	11775.8278	11285.6306	11183.9377
1122.49062	1282.29486	2124.85653	2086.94001	2025.48675
5965.25693	5819.47597	5092.83629	5278.39912	5365.57514
11642.5258	11186.1174	12857.6293	13334.0627	13690.7901
2852.62873	2836.29043	1900.12699	2020.25561	1996.90846
4791.20834	4599.03168	6298.6265	6224.19057	6387.24924
4424.41022	4289.77883	5015.34334	5027.62821	4951.18984
3055.91444	2926.85734	2321.68861	2290.75008	2146.94451
4130.5298	4364.88309	4651.12651	4374.87244	4647.54544
3511.83418	3444.85586	2952.48117	2701.18788	2767.62939
4424.41022	4480.85291	5223.02443	5200.44412	4702.02282
3523.61886	3410.61715	3050.12228	3072.17857	3101.63823
2451.94966	2508.26151	2775.79726	2799.80566	2774.77396
9394.59837	9062.213	6949.56723	7373.79209	7377.66582
2309.79698	2373.51563	2444.12746	2648.59173	2633.66862
50.0848835	76.2087382	24.7977421	20.6627724	23.2198658
6805.65182	7085.20371	7673.35133	7784.22988	7728.64302
677.619012	711.281557	570.348069	605.794917	584.068932
2474.04594	2380.14248	2039.61429	2132.96164	2182.66738
79517.8545	78490.5825	93592.8782	101276.7	104884.134
6199.47742	6288.87762	7158.79818	7043.18773	7161.54245
669.517046	609.669906	530.051738	594.524314	550.132205
1425.20955	1379.48861	1928.02445	1888.76524	1920.99736
3707.75446	3601.69124	3254.70365	3243.11605	3223.09599
335.863336	369.998946	235.57855	195.357121	186.651998
2254.5563	2279.6353	3026.8744	2907.8156	2795.31461
3869.79379	4073.30183	4265.21165	4334.48611	4229.58786
2601.46777	2552.44049	2871.88851	2846.7665	2822.99984
2633.1391	2481.75413	2957.13075	2770.68993	2831.03748
104662.676	109312.047	68372.0246	68777.9163	69238.9606
807.250475	833.878223	418.461898	419.829966	417.064512
2379.03197	2341.48587	2079.91062	2077.54784	2188.02582
3230.47499	3226.16992	3787.85511	3586.86944	3606.22377
819.035154	884.684048	1004.30856	1049.10531	1069.89997
578.92233	585.371468	396.763874	342.814178	378.662427
525.891277	554.446182	271.225305	270.494475	235.770945
5503.44485	5623.98399	6098.6947	6458.05558	6600.69339
2169.85392	2261.96371	2555.7173	2506.76998	2391.64618
550.933719	517.998525	729.983534	751.373541	719.815839
204.758788	227.52174	122.438852	160.606094	132.174621
5081.40605	5458.31282	6073.89696	6071.09821	5886.23598
702.661454	807.370835	440.159923	407.620146	410.81301

11498.9	11378.296	7025.51032	6727.61084	6623.02018
1926.05839	2055.42698	2210.09877	2215.61273	2291.62214
938.355023	946.534618	829.174502	791.759869	765.362499
4291.83259	4362.67414	3770.80666	3805.70698	3718.75081
181.189432	209.850149	79.042803	50.717714	73.2318844
30.1982386	13.2536936	7.74929441	6.57451848	6.25150233
698.242199	595.311738	485.10583	436.735871	491.189469
12851.1919	12364.5917	14719.0098	14769.1862	15300.1054
156.883532	135.850359	86.7920974	133.368804	122.350831
167.195126	164.566696	92.991533	108.009947	125.923118
3930.92681	3761.84003	4356.65332	4564.59426	4511.79854
30.1982386	30.9252851	103.840545	100.496211	124.136975
961.187838	965.310684	754.781276	804.908906	832.342881
10153.237	10298.1199	11307.7704	11253.6972	10855.2873
657.732367	707.968133	911.317023	935.460058	880.568756
819.035154	850.44534	661.789743	671.540102	718.029696
688.667148	647.222038	517.652867	487.453585	516.195478
1921.63913	2021.18827	1463.06679	1545.95106	1496.78827
170.141295	217.58147	106.940263	99.5569942	97.344822
3439.65303	3368.64712	4181.51927	4247.13894	4104.55781
383.00205	360.058676	328.570083	300.549416	298.285968
273.993774	310.357325	137.937441	145.578624	128.602334
561.981855	573.222248	429.310911	430.161352	389.379288
292.407335	261.760449	195.282219	231.986581	225.947156
33.8809506	30.9252851	13.9487299	23.4804232	21.4337223
374.163541	387.670538	285.174034	337.178876	321.505834
2579.3715	2487.2765	3026.8744	3166.10026	3015.90334
9312.10562	9288.63027	10216.6698	10507.0198	9899.70047
2376.0858	2506.05257	3045.4727	2994.22356	2745.30259
11345.6992	10831.5811	10049.285	10368.9549	10406.0722
3276.14062	3477.9901	2103.1585	2121.69104	2001.37382
6209.78901	5810.64017	7330.83252	6984.01706	7065.0907
1879.65622	1870.97975	2129.50611	2000.53205	2130.86922
371.217372	351.222881	189.082784	235.743448	189.331213
1510.64847	1557.309	863.271398	911.979635	910.040125
8662.47522	8307.85694	9553.33015	10306.9666	10537.3537
313.767064	353.431829	192.182501	225.412062	228.626371
4818.46041	4847.53844	5374.91061	5360.111	5590.62922
1843.56564	1590.44323	1297.23189	1329.93117	1399.44345
1808.2116	1882.02449	1335.97836	1418.21756	1386.94045
2166.17121	2234.35185	2741.70036	2678.64667	2691.71829
1639.54339	1523.07029	1855.18108	1863.40638	1776.31973
9302.53057	8593.91583	5839.86827	6298.38871	7342.83602

33.1444082	28.7163361	13.9487299	15.0274708	21.4337223
883.114343	943.221195	649.390872	640.545944	694.80983
394.786729	467.1927	585.846658	553.198769	495.654827
7302.0814	7835.14187	9522.33298	8944.16279	8955.72362
5399.59237	5705.7151	4221.8156	4184.21141	4178.68277
8140.26665	8430.45361	9314.65189	9579.07343	9718.4069
8551.99386	8139.97682	9423.14201	9774.43055	10000.6176
2347.36064	2448.61989	2644.05925	2627.92896	2597.94575
8966.66723	9046.75036	11436.4087	11048.0087	10633.8055
22682.5599	23269.0681	26036.0794	25508.1925	25990.1744
3668.71772	3765.15346	4469.79302	4473.49022	4423.38443
6498.51363	6735.0853	7929.07804	7667.76698	7799.19569
985.493737	1052.56417	1153.09501	1160.87212	1112.76741
1808.94815	1972.5914	2380.58324	2317.04816	2140.69301
2175.74626	2153.72521	1797.8363	1835.22987	1843.30012
5227.97799	5393.14882	6374.56958	6188.50033	6190.77345
2048.32443	2101.81491	2399.18155	2212.79508	2364.85402
13187.0552	13226.0817	13981.277	14184.9932	13689.897
2612.51591	2631.96266	3003.62652	3074.99622	3026.6202
523.68165	508.058255	641.641577	632.092991	614.433372
103.115937	101.611651	46.4957665	40.3863278	48.2258751
735.06932	801.848463	605.994823	599.220399	575.138214
3313.70428	3226.16992	3756.85793	3620.68125	3578.53855
15771.5825	15862.4623	17961.3146	17536.1192	17473.8421
6718.00327	6762.69716	7349.43082	7842.46133	8042.11121
262.945638	260.655974	323.920507	334.361226	298.285968
2512.34614	2511.57494	1484.76481	1415.39991	1494.10906
12116.8591	11832.235	14410.5879	14019.6911	13815.8201
5867.29679	5982.93819	6287.77749	6365.07311	6333.66493
2640.50452	2656.26109	2975.72906	2938.80976	2889.98022
61.1330196	58.5371468	89.8918152	107.07073	97.344822
2728.88961	2749.03695	2499.92238	2534.94648	2474.70185
3957.44234	4161.65979	4291.55925	4306.30961	4291.20981
1142.37727	1191.72795	1320.47977	1268.88207	1371.75823
3919.87868	3749.69082	5202.87627	4955.3085	5048.53467
3643.67527	3441.54244	3432.93743	3259.08273	3252.56735
668.043961	656.057833	822.975067	733.528419	757.324853
1383.22664	1298.86197	1662.99858	1661.47474	1533.40421
677.619012	691.401016	519.202726	549.441902	446.535881
123.739124	113.76087	72.8433675	77.9550049	58.9427362
1996.02992	1991.36746	2453.42661	2437.26792	2299.65979
955.295499	1052.56417	663.339602	643.363594	629.615592
699.715284	677.042848	768.730006	808.665773	768.041715

3532.45737	3878.91433	4049.78126	3954.10326	4003.64071
6592.05452	6592.60809	6056.84851	6298.38871	6374.74623
15238.3258	16114.2825	16908.9604	16439.1139	16704.9073
95.0139702	123.70114	52.695202	47.9000632	46.4397316
2261.92172	2467.39596	1824.18391	1732.85523	1719.16314
1107.02323	1134.29528	1346.82737	1278.27424	1333.35614
1327.24941	1482.20474	1579.3062	1507.44317	1610.20839
1006.11692	1070.23576	1171.69332	1171.20351	1128.84271
912.576039	800.743989	643.191436	630.214557	609.074941
318.922861	305.939427	232.478832	245.135618	242.022447
6508.08869	6906.27884	7811.28877	7653.67873	7328.54687
4017.10227	4161.65979	5308.26667	5184.47743	5019.95637
1829.57133	1742.86071	1556.05832	1560.97853	1408.37417
3653.25033	3457.00508	2954.03103	2930.35681	2945.35067
2977.1044	3021.84214	3533.67825	3598.14004	3667.84572
2563.16757	2474.02281	2675.05643	2884.33518	3031.08556
2525.60391	2506.05257	2704.50375	2702.1271	2768.52246
3786.5645	3766.25793	4621.67919	4437.79998	4393.01999
1951.10083	1931.72584	2608.4125	2475.77582	2510.42472
5308.26111	5119.23915	5765.47504	5856.01753	5658.50268
3820.44545	3783.92952	4765.81606	4418.07642	4489.47174
368.271202	400.924232	463.407806	427.343701	437.605163
17587.8961	18250.3361	20036.5756	20321.8366	20738.9124
905.210615	981.877801	692.786921	743.859805	735.891131
5453.35996	5886.84891	4307.05784	4342.93907	4442.13894
5543.95468	5277.179	5043.2408	4865.14368	4895.81939
3056.65098	3030.67794	2518.52068	2367.76587	2456.84042
3377.78347	3344.34869	4280.71023	4289.4037	4118.84696
162.039329	212.059098	137.937441	133.368804	138.426123
849.969935	876.952727	491.305266	435.796654	441.17745
4159.9915	4149.51057	4936.30054	4695.14541	4814.54986
117.110242	122.596666	83.6923797	77.0157879	67.8734538
45.6656291	56.3281978	26.347601	32.8725924	25.0060093
103.115937	93.8803297	43.3960487	39.4471109	42.8674445
3983.22132	3916.46646	4071.47929	4264.04484	4184.93427
2041.69555	2141.57599	2916.83442	2838.31355	2973.92896
4557.7244	4432.25604	6382.31888	6194.13563	5939.82028
714.446132	657.162308	471.1571	441.431955	443.856665
1217.50459	1389.42888	1105.04938	1073.52495	1080.61683
100.906309	111.551921	43.3960487	55.4137986	75.9110997
799.148509	823.937952	689.687203	585.132145	632.294807
1834.72713	1709.72648	2140.35512	2167.71267	2119.25929
7071.54362	6659.98104	7182.04606	7433.90197	7734.00145

2433.5361	2455.24674	3167.91156	3605.65378	3457.97386
3989.85021	3799.39217	5089.73657	4883.92802	4528.7669
580.395415	626.237023	519.202726	476.182981	497.440971
3762.2586	3732.01922	4336.50515	4149.46038	4186.72042
1556.3141	1579.39849	778.029159	859.383487	860.921178
895.635564	925.549603	678.838191	651.816547	649.26317
13352.7773	13156.4999	16349.4614	16201.492	16401.2629
7422.13781	7909.14166	9195.31275	8879.35682	8602.0672
4390.52927	4845.32949	5078.88756	5007.90465	4828.83901
5344.35169	5199.86579	6631.84616	6539.76746	6320.26885
270.311062	267.282821	451.008935	410.437797	410.81301
7509.78636	7076.36791	6458.26196	6515.34782	6474.77027
116.3737	115.969819	58.8946375	71.3804864	66.9803821
6330.58197	6890.8162	4973.49715	4876.41428	4822.58751
7000.83555	6983.59205	8434.33204	8247.26383	7928.6911
1319.88399	1435.81681	1680.04703	1781.69451	1656.64812
28590.3665	28379.4714	34137.1918	33970.537	33863.495
1447.30583	1401.5781	1156.19473	1281.09189	1242.26282
145.098854	155.7309	69.7436497	66.6844017	80.3764585
138.469972	161.253272	85.2422386	73.2589202	72.3388126
4403.05049	4646.52408	4906.85322	4859.50838	4890.46096
275.466859	261.760449	99.1909685	100.496211	102.703253
5973.3589	5675.89429	6318.77467	6149.05321	6098.78706
18991.0094	19021.2593	17657.5423	17703.2998	17464.0183
491.273784	544.505912	647.841013	692.202874	680.520682
3412.40096	3400.67688	3998.63592	3816.97759	3580.32469
2254.5563	2248.71002	3274.85182	3188.64146	3199.87612
47.8752563	44.1789787	26.347601	22.5412062	21.4337223
1336.82446	1185.1011	1870.67967	1822.08084	1804.89803
9712.78469	9978.92681	11203.9299	10828.2319	10584.6865
2659.65462	2765.60407	3231.45577	3368.97111	3233.81285
13048.5852	13286.8278	15095.6255	15107.3043	14584.7549
6354.88787	6507.56356	5807.32123	5717.95265	6036.27203
5733.24608	5974.10239	7826.78736	7723.18078	7850.10078
156.14699	141.372732	113.139698	109.88838	91.0933196
95.0139702	113.76087	23.2478832	23.4804232	24.1129376
18500.4721	18192.9034	17129.0404	17033.6382	16728.1272
41.2463747	27.6118617	12.3988711	14.0882539	14.2891482
11827.3979	11589.2506	10490.9948	10624.4219	10815.9921
692.34986	683.669695	787.328312	767.340229	810.909159
617.222535	638.386242	469.607241	427.343701	426.888302
2222.88498	2562.38076	2005.51739	2067.21645	2052.27891
1719.82651	1833.42762	2098.50893	1984.56536	1945.1103

1341.24372	1351.87675	1678.49717	1574.12757	1580.73702
3086.84922	3038.40926	3752.20836	3902.44633	3763.4044
1560.73336	1582.71191	1807.13546	1866.22403	1913.85278
38.300205	41.9700297	65.0940731	88.286391	76.8041715
4897.27045	4779.06102	2720.00234	2720.91143	2699.75593
9067.57354	9409.01798	6289.32735	5994.08242	6350.63329
11784.6785	12171.3086	12843.6806	12828.764	12631.607
1636.59722	1769.3681	2131.05596	1914.1241	1962.97173
1022.32086	1033.7881	1126.74741	1215.3467	1220.8291
858.808444	1053.66864	832.27422	770.157879	782.330863
9461.62373	9627.70393	16284.3673	16515.1904	16942.4644
988.439907	968.624108	1219.73894	1201.25845	1227.0806
3335.06401	3383.00529	3519.72952	3873.3306	3904.50974
8976.24228	8723.13934	10332.9092	10686.4102	10313.1927
7482.53429	7592.15749	8872.9421	8947.91965	8632.43164
1610.0817	1551.78663	1325.12935	1282.97032	1420.87717
114.900615	117.074294	170.484477	168.11983	147.356841
6769.56124	6983.59205	7371.12885	7920.41634	7693.81322
721.075014	693.609965	922.166035	777.671615	918.07777
4480.38745	4485.27081	3945.94072	3805.70698	3681.2418
659.205452	746.62474	440.159923	439.553521	400.096149
905.947157	927.758552	1005.85842	1120.48579	1106.51591
2282.54491	2327.1277	2771.14768	2721.85065	2822.10677
3149.45532	3161.00592	2795.94542	2893.72735	2949.81603
2278.12566	2265.27713	3352.34476	3475.10263	3429.39556
4632.11518	4712.79255	3042.37299	3128.53158	3018.58255
1420.05376	1594.86113	1605.6538	1660.53553	1672.72341
12599.2944	12463.9944	10876.9096	11062.097	11351.8352
771.89644	741.102367	519.202726	508.116357	551.918348
26.5155266	14.3581681	6.19943553	6.57451848	8.93071761
10543.6045	9991.07603	9809.05687	9220.29256	9305.80775
2578.63496	2776.64881	3127.61523	3093.78055	2990.00426
983.28411	978.564378	1371.62511	1375.01358	1271.73419
3490.47445	3919.77988	4488.39132	4377.69009	4262.63152
626.061044	563.281978	353.367825	412.316231	417.064512
3475.74361	3492.34826	2222.49764	2248.48532	2342.52723
2348.83373	2328.23218	2594.46377	2643.89565	2703.32822
95.7505126	113.76087	51.1453431	37.568677	45.5466598
1460.56359	1445.75708	1788.53715	1682.13751	1636.10747
199.602992	239.670959	96.0912507	92.0432587	78.590315
3072.11837	3105.7822	3522.82924	3675.15583	3753.58061
1920.16605	1943.87506	2230.24693	2101.96748	2053.17198
19259.1108	19715.9737	21298.1608	20832.7706	20515.6445

2988.15254	3054.97638	3533.67825	3531.45564	3826.8125
201.812619	185.55171	291.37347	248.892485	253.63238
13403.5987	13985.9602	18068.2549	18057.3846	18247.2422
234.220485	245.193332	430.860769	380.382855	404.561508
4942.93608	4918.2248	5559.34381	5411.76793	5440.59317
592.916636	649.430987	497.504701	507.17714	542.987631
5996.92826	6037.05744	3200.45859	3080.63152	3374.02511
4925.9956	4928.16507	3787.85511	3540.84781	3610.68913
6508.82523	6513.08593	7394.37673	7455.50396	7837.59778
18633.0498	18467.9176	23936.0206	23808.2099	22609.8978
4347.07327	4528.34531	5013.79349	4884.86723	4802.93993
3943.44803	3798.28769	4424.84711	4462.21962	4558.23827
1485.60603	1556.20452	1721.89322	1943.23982	1877.23684
21.3597297	35.3431829	63.5442142	60.1098833	74.1249562
3150.92841	3235.00571	4073.02914	3873.3306	3704.46167
15895.3216	15587.4482	17491.7074	17970.9767	18022.1881
2804.75348	2723.63404	3225.25634	3263.77882	3129.32345
2163.22504	2260.85923	1751.34054	1779.81608	1734.34536
3818.97237	3778.40715	3391.09124	3285.38081	3056.98464
1439.9404	1461.21972	1734.29209	1731.91601	1591.45388
3836.64938	4204.7343	5071.13826	5225.80298	5085.15061
1545.26596	1610.32377	2850.19049	2764.11541	2773.88089
396.996356	364.476574	280.524458	323.090623	288.462179
137.73343	141.372732	89.8918152	94.8609095	92.8794632
3265.82902	3156.58803	4669.72481	4317.58021	4321.57425
2364.30112	2326.02323	3008.27609	2858.03711	2848.89892
11675.6702	11434.6242	9740.86308	9786.64037	10182.8042
101.642852	85.044534	52.695202	59.1706663	70.5526691
1480.45023	1417.04074	1754.44026	1778.87686	1801.32574
1532.74474	1536.32398	1824.18391	1745.06505	1812.93568
95.7505126	98.2982276	142.587017	143.70019	174.148993
2638.29489	2557.96287	3377.14251	3344.55147	3211.48605
13033.8544	13054.8882	15441.2441	15630.4481	15225.0874
85.4389189	97.1937531	150.336312	154.031576	133.067692
150.254651	149.104053	106.940263	103.313862	99.1309655
3748.2643	3885.54118	3171.01127	3084.38839	3096.2798
1391.3286	1422.56311	1800.93602	1811.74945	1804.89803
6678.22998	6505.35461	5542.29536	5552.65047	5420.05252
3995.74254	3970.58571	5441.55454	5361.98943	5241.43817
1154.89849	1145.34002	1360.7761	1255.73303	1369.07901
905.947157	996.235969	1148.44543	1071.64651	1100.26441
3303.39268	3168.73725	3484.08277	3519.24582	3472.26301
466.231342	447.312159	359.567261	373.808337	361.694063

3477.95323	3240.52809	2292.24129	2416.60515	2611.34183
1745.6055	1899.69608	2233.34665	2033.40465	2115.687
1549.68522	1523.07029	1380.92426	1404.1293	1435.16632
473.596766	398.715283	220.079961	258.284655	233.984801
81.7562069	89.4624318	29.4473188	28.1765078	32.1505834
908.893327	966.415159	1115.8984	1109.21519	1025.24638
148.781566	160.148798	201.481655	236.682665	251.846237
1535.69091	1528.59266	2084.5602	1909.42801	1775.42666
271.047605	281.640989	308.421918	357.841649	341.153413
789.573458	725.639725	891.168858	880.985477	850.204317
588.497381	622.923599	745.482123	664.965584	695.702902
5040.89622	5334.61168	6242.83158	6101.15315	6180.05659
710.76342	704.65471	567.248351	507.17714	546.559918
4713.13485	4813.29973	5182.7281	4999.4517	4915.46697
7147.40749	7143.74085	5125.38333	5048.29098	4894.03325
256.316757	248.506755	156.535747	149.335491	138.426123
17.6770177	26.5073872	6.19943553	11.2706031	2.67921528
3686.39473	3669.06418	2546.41814	2504.89154	2559.54367
80.2831221	88.3579574	32.5470365	32.8725924	33.9367269
11270.5719	10813.9095	12798.7347	12980.9171	13229.9651
1709.51492	1748.38308	1208.88993	1190.92706	1248.51432
2918.18101	2915.81259	3538.32783	3584.99101	3442.79164
551.670261	544.505912	844.673091	891.316863	820.732948
2017.38965	2051.00909	2639.40968	2388.42864	2314.84201
32650.1882	31477.5223	29517.0624	29195.5581	29716.0698
2594.10235	2636.38055	2974.1792	2959.47253	3006.97262
903.73753	887.997471	683.487767	816.179509	761.790212
4802.25648	4253.33117	2794.39557	3117.26098	3237.38513
27.9886114	33.134234	4.64957665	3.7568677	2.67921528
1781.69608	1738.44281	1559.15804	1471.75292	1524.4735
975.182143	1010.59414	771.829724	719.440165	849.311245
8195.50733	8271.40928	5618.23845	5577.07011	5612.06295
12072.6666	11802.4142	11062.8927	11359.8287	11517.0534
5959.36459	6120.9975	6464.4614	6493.74583	6740.90565
3229.73844	3584.01965	3679.36499	3768.13831	3649.98429
959.714753	939.907771	531.601597	601.098833	661.766175
321.869031	337.969187	170.484477	184.086518	176.828209
392.577102	340.178136	168.934618	159.666877	192.010429
2220.67535	2393.39617	2582.0649	2626.98974	2722.08273
5221.34911	5403.08909	4240.4139	4763.70825	4720.77733
42.7194595	44.1789787	24.7977421	18.7843385	17.8614352
44.1925443	55.2237233	10.8490122	15.9666877	14.2891482
713.70959	715.699455	840.023514	828.389329	814.481446

2168.38084	2164.76996	1270.88428	1245.40164	1230.65289
1573.99112	1597.07008	1887.72812	1871.85933	1881.7022
10150.2909	10123.613	9186.0136	8860.57248	8669.94066
3554.55364	3496.76616	2918.38428	2880.57831	3031.08556
760.848304	734.475521	1027.55644	941.09536	959.159071
122.266039	139.163783	3.09971777	3.7568677	6.25150233
3490.47445	3620.4673	3975.38803	3884.60121	3875.03837
4355.17524	4711.68808	5782.52349	5637.17999	5380.75736
69.234986	64.0595191	89.8918152	103.313862	96.4517502
6294.49139	6658.87656	8242.14954	8248.20304	7798.30262
431.613849	468.297174	588.946375	511.873225	589.427362
947.930074	954.265939	1185.64205	1132.69561	1125.27042
2236.14274	2208.94893	1574.65663	1511.20003	1535.19036
6831.4308	6684.27947	4671.27467	4221.78008	4329.6119
1543.05634	1600.3835	1827.28362	1801.41806	1957.6133
30052.4032	29823.0196	25882.6433	26258.6268	26788.5805
11196.1811	10958.5957	13153.6523	13365.0569	13698.8277
180.452889	194.387506	122.438852	125.855068	119.671616
2090.30734	2137.15809	1153.09501	1187.17019	1239.5836
25986.6891	24836.3173	26865.2539	28540.924	28729.2255
14557.7606	14665.212	15444.3438	15662.3815	15235.8042
577.449245	557.759606	878.769987	857.505053	881.461828
805.040848	869.221405	404.513168	439.553521	424.209087
2059.37256	1935.03927	928.365471	1023.74645	950.228354
253.370587	312.566274	221.62982	243.257184	205.406505
4185.77048	4283.15198	4688.32312	4658.51595	4543.94912
6167.80609	6147.50488	7474.96939	7344.67636	7056.15998
15521.1581	15321.2698	18587.4576	17741.8077	17413.1132
5532.90654	5789.65516	6338.92283	6116.18062	6106.8247
47644.7185	45641.3029	52802.1423	52413.0006	52184.8622
10100.9425	9762.44981	10652.1801	10507.959	10547.1775
12195.6691	13357.5142	14461.7332	14155.8775	13735.4437
3866.11108	4087.66	2343.38663	2274.7834	2311.26972
548.724091	547.819336	234.028691	247.953268	295.606753
752.746337	773.132127	227.829256	195.357121	192.9035
478.752563	443.998736	249.52728	270.494475	250.060093
15440.875	15452.7023	17809.4284	17334.1876	17390.7864
446.344697	452.834531	389.01458	382.261289	348.297987
991.386076	1009.48966	685.037626	731.649985	748.394136
643.001519	686.983118	491.305266	440.492738	442.070522
112.690988	118.178768	60.4444964	31.9333755	47.3328033
1904.69866	1874.29317	2695.2046	2695.55258	2594.37347
3625.26171	3523.27355	3294.99998	3207.4258	3110.56894

86.9120037	66.268468	23.2478832	14.0882539	8.93071761
1088.60967	1094.5342	1233.68767	1183.41333	1189.57159
4783.10637	4711.68808	3124.51551	3076.87465	3151.65025
1462.03667	1610.32377	1898.57713	1893.46132	1820.97332
1831.78096	1795.87548	2123.30667	2098.21061	2068.3542
262.945638	247.402281	170.484477	154.970793	158.966774
273.257232	267.282821	162.735183	192.53947	198.261931
1452.46162	1401.5781	993.459544	955.183614	996.668085
2924.80989	2962.20052	3228.35605	3247.81213	3452.61543
2455.63238	2307.24716	2912.18484	2735.93891	2512.21086
2395.97244	2454.14227	2930.78315	2907.8156	2921.23773
266.62835	267.282821	370.416273	373.808337	393.844647
1100.39435	1113.31026	995.009403	907.283551	898.430192
552.406803	492.595612	458.758229	405.741712	420.6368
12323.091	11562.7432	14193.6077	15093.216	15266.1687
3969.96356	4024.70496	4506.98963	4398.35286	4397.48535
4360.33103	4431.15156	5050.9901	5408.01106	5161.06171
506.741174	517.998525	680.38805	665.904801	742.142633
3545.71514	3593.95992	4121.07477	3966.31308	4075.08645
204.022246	226.417266	145.686735	158.727661	152.715271
283.568826	281.640989	375.06585	364.416167	356.335633
3258.4636	3355.39343	3981.58747	4190.78592	4072.40723
89.1216309	97.1937531	46.4957665	50.717714	56.263521
4489.22596	4338.37571	5629.08746	5559.22499	5483.46061
1341.24372	1202.77269	1193.39134	1113.91127	1130.62885
2516.7654	2257.54581	1811.78503	1753.518	1904.029
968.553262	1012.80309	776.4793	768.279445	839.487455
153.20082	162.357747	212.330667	218.837544	186.651998
2124.92484	2204.53104	2599.11335	2486.1072	2634.5617
4372.11571	4568.1064	4131.92378	3920.29145	3723.21617
451.500494	381.043691	294.473188	297.731766	347.404915
200.339534	199.909879	116.239416	103.313862	106.27554
284.305368	260.655974	168.934618	153.092359	169.683635
198.129907	213.163572	159.635465	151.213925	170.576706
615.74945	578.744621	365.766696	355.963215	350.08413
1589.45851	1775.99494	1315.83019	1455.78624	1367.29287
2071.15724	2055.42698	1698.64534	1707.49637	1659.32733
285.041911	286.058887	444.809499	401.045627	432.246732
911.102954	914.504859	1098.84995	1034.07784	1110.0882
679.828639	698.027863	821.425208	814.301075	868.065752
296.090047	292.685734	413.812322	397.28876	439.391306
1052.5191	1062.50444	599.795388	606.734134	679.62761
7598.90799	7740.15706	8376.98726	8754.44097	8570.80969

3691.55053	3924.19778	5178.07853	4927.13199	4681.48217
40.5098322	72.8953148	15.4985888	16.9059047	16.0752917
2159.54233	2235.45632	2478.22435	2540.58179	2558.6506
26.5155266	35.3431829	7.74929441	7.51373541	9.82378937
38.300205	38.6566063	6.19943553	17.8451216	8.03764585
3153.13803	3046.14058	3812.65285	3759.68536	3797.34113
7554.71544	7494.96373	9830.75489	9746.25404	10050.6296
2901.24053	2824.14121	4024.98352	3931.56205	4053.65272
1848.72144	1909.63635	2397.63169	2360.25214	2263.04384
3100.10698	3092.52851	3541.42755	3491.06931	3506.19973
7840.4939	7748.99286	5816.62039	5812.81356	5629.92438
8830.40689	9175.97387	6030.50091	6339.71425	6203.27645
3468.37818	3294.64734	4111.77562	4027.36218	3846.46008
1315.46473	1249.16062	945.413918	854.687403	894.857905
4688.82895	4671.927	4173.76997	4063.99164	4187.61349
17062.7413	16856.4893	19318.991	18798.4268	19112.6288
25594.112	25296.8832	30510.522	29572.1841	30461.7847
20447.1537	20156.659	22617.0907	22557.1729	22673.3059
757.165592	720.117352	561.048916	555.077203	555.490635
20078.1459	20221.823	23418.3677	22828.6066	23028.7484
1026.74011	1061.39996	636.992001	548.502685	481.365679
74.3907828	69.5818914	21.6980244	32.8725924	33.9367269
56479.5447	55703.0653	48998.7886	48449.5051	49541.3698
6994.94321	7447.47133	8167.75631	7719.42391	7735.78759
1249.91246	1315.42909	1576.20648	1545.95106	1640.57283
7058.28586	6959.29362	4753.41719	4543.93149	4479.64795
6812.2807	6730.6674	5605.83958	5454.03269	5387.90193
7383.8376	7185.71088	4995.19518	4893.32018	4937.79377
735.805862	707.968133	596.69567	618.943954	635.867094
7462.64764	7879.32085	7178.94634	7030.97791	6925.77151
511.896971	489.282189	615.293976	640.545944	607.288798
3254.78089	3342.13974	4434.14626	4317.58021	4413.56064
673.9363	672.62495	813.675913	863.140355	811.802231
4352.96561	4439.98736	5545.39508	5329.11684	5194.99843
12724.5066	13004.0824	14647.7163	14339.0248	13571.1185
2485.09407	2637.48503	2789.74599	2722.78987	2789.06311
3519.93615	3476.88562	3939.74128	3838.57958	3841.99472
648.157316	628.445972	499.05456	501.541838	519.767765
1973.1971	2037.75539	2681.25587	2659.86233	2564.00903
3344.63906	3450.37824	2620.81137	2612.90149	2573.83282
3331.3813	3294.64734	3904.09453	3829.18741	3811.63028
1592.40468	1842.26341	2126.40639	2023.07326	2017.44911
2709.00296	2730.26088	3110.56678	3055.27266	2972.14282

3900.72857	3839.15325	4317.90685	4023.60531	4213.51257
4266.05361	4400.22628	5050.9901	5164.75388	5138.73491
9488.13925	9838.65855	11434.8588	11273.4208	11506.3366
16217.9272	16755.9821	13037.4129	13061.6898	13120.1172
2725.2069	2827.45464	3118.31607	3019.58242	3025.72713
156.14699	146.895104	120.888993	108.009947	115.206257
5129.2813	5036.40357	6176.18765	6304.96322	6293.4767
542.831752	538.98354	734.63311	703.473478	626.043305
3173.76122	3042.82716	2200.79961	2157.38128	2139.79994
529.573989	531.252219	457.20837	389.775024	429.567517
2321.58166	2348.11272	2971.07948	2851.46259	2913.20009
2052.74368	2054.32251	2318.58889	2265.39123	2239.82398
11625.5853	11890.7721	11075.2916	10677.9572	10803.4891
494.956496	529.04327	420.011757	432.039786	400.989221
2417.33217	2330.44113	3312.04843	3201.7905	3298.11401
4358.12141	4265.48039	6174.63779	5855.07832	5708.5147
690.876775	681.460746	788.878171	788.003001	802.871513
2921.86372	2773.33539	3062.52115	3123.8355	3098.06594
1973.93364	2018.97933	1667.64816	1634.23745	1694.15713
245.268621	249.61123	306.872059	306.184718	273.279959
1028.94974	1073.54918	1238.33725	1177.77803	1178.85473
514.843141	531.252219	643.191436	642.424377	592.106578
257.789842	247.402281	336.319378	383.200506	369.731709
814.615899	853.758763	1115.8984	1077.28181	1043.10782
6463.89614	6745.02557	5943.70882	5799.66452	5645.1066
86.9120037	82.835585	167.384759	117.402116	144.677625
1540.84671	1503.18975	1356.12652	1245.40164	1302.09863
968.553262	956.474888	1210.43979	1124.24266	1118.12585
3067.69911	3209.6028	3662.31654	3474.16341	3556.21175
4422.2006	4277.62961	3592.57289	3528.63799	3606.22377
885.32397	925.549603	1094.20037	1026.5641	1080.61683
631.216841	668.207053	765.630288	728.832335	832.342881
17409.6528	17482.7263	20242.7069	20366.919	20130.7306
2350.30681	2335.9635	2843.99105	2704.94475	2657.78156
2072.63033	2202.32209	1687.79632	1587.27661	1708.44628
1272.74528	1195.04137	1520.41156	1519.65299	1510.18435
42522.8026	41626.5382	42830.3502	46186.9316	47143.4721
849.233392	789.699244	706.735651	740.102938	707.312835
2704.58371	2739.09668	2403.83113	2319.86581	2362.17481
44.1925443	35.3431829	7.74929441	4.69608463	2.67921528
5815.73883	5778.61041	6276.92848	6103.03159	6153.26443
4601.1804	4718.31492	4073.02914	3951.28561	3920.58503
1790.53459	1790.35311	2056.66274	1940.42217	1987.08467

8085.76252	8144.39472	8814.04747	9271.01028	9516.57269
12758.3875	12558.9792	13569.0145	13412.9569	13440.73
18403.2485	18520.9323	23616.7497	23308.5465	22852.8133
561.245312	586.475942	871.020692	892.25608	785.90315
6887.40802	6680.96605	7788.04089	7660.25325	7609.86448
1665.32238	1799.18891	1131.39698	1116.72893	1119.91199
3843.27827	3725.39238	3254.70365	3029.9138	3351.69832
1635.86068	1718.56227	1745.1411	1797.6612	1907.60128
550.933719	540.088014	919.066317	875.350175	885.927187
136.996887	110.447447	83.6923797	92.0432587	66.9803821
3960.38851	3896.58592	4378.35134	4411.5019	4360.86941
1747.07858	1824.59182	1618.05267	1623.90607	1569.12708
3607.5847	3643.66127	4460.49386	4326.97238	4113.48853
739.488574	674.833899	871.020692	893.195297	868.065752
556.826058	476.028495	627.692848	574.800759	607.288798
807.987018	908.982486	1066.30291	1004.02289	1053.82468
2483.62099	2742.4101	2109.35794	2119.8126	2240.71705
6172.96189	6340.78791	8294.84474	7916.65947	7972.45161
2116.82287	2116.17308	2329.4379	2216.55195	2293.40828
715.919217	663.789155	477.356536	511.873225	487.617182
385.211678	423.013721	309.971777	322.151406	271.493815
299.036216	275.014142	221.62982	227.290496	193.796572
27.9886114	23.1939638	9.2991533	5.63530156	7.14457409
184.872144	184.447236	241.777986	247.953268	275.959174
5834.88893	5676.99876	4787.51409	5432.4307	5353.96521
3886.73427	4012.55574	4742.56818	4572.108	4426.95672
607.647484	534.565642	705.185792	767.340229	719.815839
2782.6572	2830.76806	3409.68954	3457.2575	3070.38071
19.8866449	29.8208106	96.0912507	123.976634	107.168611
4301.40764	4311.86832	5771.67448	5585.52306	5770.13665
807.987018	875.848252	1018.25729	991.813074	987.737368
12036.576	11873.1005	16113.8828	15708.4031	14832.1358
332.180624	335.760238	282.074317	276.129776	283.99682
3444.07228	3428.28875	3775.45624	3880.84434	4112.59546
1707.30529	1660.02512	1436.71918	1427.60973	1451.24161
431.613849	407.551078	336.319378	358.780866	336.688054
83.9658341	87.2534829	15.4985888	15.9666877	17.8614352
481.698733	478.237444	330.119942	297.731766	368.838637
8399.52958	8252.63322	9723.81463	9441.94776	9567.47778
115.637158	90.5669063	60.4444964	40.3863278	42.8674445
5841.51781	5609.62582	6224.23327	6017.56284	6159.51594
244.532078	268.387296	187.532925	156.849227	183.079711
1864.92537	1931.72584	1755.99011	1546.89028	1629.85596

2902.71362	2915.81259	2583.61476	2472.95817	2559.54367
5188.94124	5182.1942	5912.71164	6040.10405	6130.93764
8181.51303	8635.88586	9066.67446	8793.88808	8649.40001
1365.54962	1545.15978	926.815612	918.554154	896.644048
966.343635	1020.53441	1128.29727	1100.76224	1119.91199
4534.89158	4635.47934	3888.59594	3991.67194	4210.83335
9865.24896	10364.3884	7203.74409	7127.71725	7379.45196
1212.3488	1243.63825	995.009403	1071.64651	1002.91959
99.4332246	92.7758552	61.9943553	62.927534	57.1565927
3064.75295	2818.61884	2640.95954	2705.88396	2743.51645
3040.44705	3237.21466	3674.71541	3621.62047	3488.3383
201.076076	209.850149	365.766696	308.063152	346.511843
4601.1804	4653.15093	5824.36968	5700.10752	5412.01487
4854.55099	4849.74738	5639.93647	5596.79366	5555.79943
2828.32283	2796.52935	3330.64674	3181.12773	3182.01469
1159.31774	1207.19059	881.869704	874.410958	811.802231
18029.085	17141.4437	16020.8913	16447.5668	17094.2866
3953.75963	3976.10808	4584.48258	4516.6942	4568.95513
16.9404753	13.2536936	30.9971777	30.9941586	25.8990811
262.945638	289.37231	437.060205	354.084781	403.668436
975.182143	1034.89258	861.721539	839.659932	832.342881
1059.14798	1124.35501	1500.2634	1586.33739	1498.57442
2862.94033	2913.60364	3473.23376	3282.56316	3181.12161
11655.047	11714.0562	15303.3066	14379.4111	13797.9587
3962.59814	4206.94324	4443.44542	4485.70004	4363.54862
1598.29702	1549.57768	2000.86782	1866.22403	1942.43108
5425.37135	5228.58213	6311.02537	5992.20399	6059.4919
1879.65622	1894.17371	2507.67167	2284.17556	2516.67622
44.1925443	40.8655553	77.4929441	72.3197033	85.7348891
1754.44401	1740.65176	2208.54891	2023.07326	2123.72465
2328.21054	2332.65007	2658.00798	2625.11131	2620.27255
321.869031	283.849938	478.906395	479.000632	487.617182
4963.55926	5033.09015	5546.94494	5557.34655	5399.51187
5785.54059	6104.43038	6346.67212	6625.2362	6506.02778
395.523271	414.177925	182.883348	169.998264	193.796572
4847.18556	4807.77735	9588.97691	9492.66547	8914.64232
32.4078658	34.2387085	9.2991533	7.51373541	4.46535881
12446.8301	13450.2901	11059.793	10637.5709	10385.5315
1273.48182	1423.66759	1774.58842	1753.518	1737.91765
331.444082	355.640778	449.459076	395.410326	401.882293
1029.68628	1108.89237	568.79821	548.502685	521.553909
5399.59237	5380.9996	4172.22011	4091.22893	3909.86817
571.556906	609.669906	415.362181	368.173035	396.523862

5659.59184	6018.28137	5320.66554	5187.29508	4928.86305
54.5041379	44.1789787	21.6980244	38.507894	33.0436552
468.440969	470.506123	347.16839	390.714241	399.203077
1612.29132	1656.7117	1244.53668	1087.6132	1090.44062
473.596766	492.595612	178.233772	222.594411	196.475787
207.704958	180.029338	282.074317	258.284655	279.531461
13989.1499	13596.0807	14635.3174	14200.9599	14408.8198
942.774278	942.11672	1083.35136	1084.79555	1199.39538
2725.2069	2792.11145	2317.03903	2338.65015	2432.72748
4397.8947	4515.09162	4801.46282	4773.10042	4929.75612
8245.59222	8633.67691	9266.60626	8925.37845	9197.74607
4078.97184	4056.73472	4798.3631	4627.52179	4812.76372
921.414548	934.385399	1012.05785	1110.15441	1077.93762
592.916636	583.162519	441.709782	464.912378	470.648818
63.3426468	81.7311106	40.296331	40.3863278	33.9367269
281.359199	295.999157	209.230949	217.898327	192.010429
11734.5936	11815.6679	13458.9745	13729.473	13176.3808
1015.69198	1026.05678	1334.4285	1324.29587	1309.2432
218.016552	210.954623	125.53857	105.192296	94.6656067
1065.77686	1119.93711	1360.7761	1297.05858	1366.39979
17.6770177	20.9850149	1.54985888	3.7568677	1.78614352
9476.35458	9224.57075	6762.03431	7128.65647	7290.14479
505.268089	553.341708	695.886638	598.281182	579.603573
533.993243	534.565642	350.268108	326.84749	392.058503
16147.9557	16521.8336	18275.9359	18159.7593	17873.0452
307.138183	302.626004	371.966132	363.47695	379.555498
6631.82781	6679.86158	8208.05264	8090.4146	7844.74235
2381.97814	2289.57557	2433.27845	2539.64257	2579.19125
7683.61036	7680.51544	6165.33864	5918.94507	5861.22997
3466.9051	3555.30331	4029.6331	3924.98753	3952.73561
411.727204	377.730268	232.478832	191.600253	215.230294
1474.55789	1444.6526	1114.34854	1117.66814	1115.44663
2158.80579	2153.72521	2399.18155	2449.47774	2474.70185
384.475135	382.148166	550.199903	511.873225	500.120186
1357.44765	1474.47341	1143.79586	1135.51326	1083.29605
237.166654	281.640989	198.381937	157.788444	175.042065
2045.37826	2003.51668	1413.4713	1523.40985	1428.91482
7916.35776	8265.88691	11385.2634	10894.9163	10788.3069
1869.34462	1899.69608	2408.4807	2432.57184	2371.10553
1678.58014	1690.95041	1959.02163	1989.26145	1859.37541
612.80328	609.669906	534.701315	514.690875	547.45299
1321.35707	1381.69756	731.533393	758.887276	833.235953
30.1982386	19.8805404	10.8490122	8.45295233	13.3960764

415.409916	402.028706	323.920507	282.704295	267.921528
7293.24289	7555.70983	8583.11849	8467.04059	8494.00552
16159.7404	15992.7903	15320.3551	14890.3451	14731.2187
8059.98353	8088.06652	8386.28641	8595.71331	8637.79007
2366.51075	2523.72416	2653.35841	2601.63089	2636.34784
2406.28404	2273.00845	2868.78879	2783.83897	2814.96219
195.183737	195.491981	153.436029	135.247237	151.822199
1839.88293	1718.56227	1990.01881	2056.88507	1994.22924
10.3115937	8.83579574	46.4957665	42.2647617	46.4397316
10.3115937	8.83579574	21.6980244	31.9333755	19.6475787
75.1273253	76.2087382	37.1966132	30.9941586	37.509014
26.5155266	15.4626425	9.2991533	9.39216926	9.82378937
20.6231873	23.1939638	80.5926619	62.927534	56.263521
176.033635	159.044323	49.5954842	87.3471741	81.2695303
66.2888164	54.1192489	20.1481655	17.8451216	29.4713681
204.758788	208.745674	271.225305	278.00821	286.676035
117.110242	122.596666	89.8918152	77.9550049	74.1249562
5998.40134	6210.45993	6531.10533	6484.35366	6556.93287
122.266039	118.178768	74.3932264	79.8334387	79.4833867
3793.19338	3714.34763	3116.76621	3014.88633	2917.66544
150.254651	159.044323	99.1909685	123.976634	116.992401
69.9715284	78.4176872	26.347601	56.3530156	41.9743728
1669.00509	1594.86113	1264.68485	1157.11525	1365.50672
38.300205	44.1789787	6.19943553	7.51373541	8.93071761
164.248956	155.7309	240.228127	212.263025	209.871864
60.3964772	56.3281978	91.4416741	88.286391	89.3071761
6408.65546	6515.29488	7406.7756	7540.03348	7177.61774
1549.68522	1519.75687	1199.59078	1138.33091	1141.34571
120.056412	138.059308	182.883348	164.362962	197.368859
123.002582	130.327987	75.9430853	84.5295233	80.3764585
70.7080708	57.4326723	46.4957665	38.507894	37.509014
266.62835	246.297806	144.136876	160.606094	162.539061
2058.63602	1982.53167	1391.77328	1377.83123	1389.61966
8188.87845	8201.82739	9671.11943	9705.86771	9646.96116
72.917698	81.7311106	111.58984	117.402116	113.420114
6599.41994	6537.38437	7733.79582	7902.57122	7640.22892
85.4389189	80.6266361	49.5954842	54.4745817	46.4397316
83.9658341	87.2534829	144.136876	110.827597	110.740898
1177.73131	1305.48882	726.883816	727.893118	764.469428
62.6061044	49.701351	29.4473188	36.6294601	33.0436552
117.846785	121.492191	82.1425208	78.8942218	88.4141043
5910.01625	6223.71362	6613.24785	6686.2853	6546.21601
470.650596	430.745042	722.234239	762.644144	690.344471

10277.7127	10099.3145	13514.7695	13547.2649	13373.7496
94.2774278	96.0892786	68.1937908	38.507894	52.6912339
216.543467	182.238287	158.085606	160.606094	177.72128
76.6004101	70.6863659	167.384759	149.335491	151.822199
159.829702	123.70114	99.1909685	104.253079	96.4517502
2183.84823	2271.90398	2081.46048	2056.88507	1920.10429
28.7251538	20.9850149	6.19943553	5.63530156	8.93071761
391.104017	395.401859	230.928974	250.770919	267.921528
411.727204	398.715283	213.880526	233.865015	302.751327
298.299674	272.805193	82.1425208	118.341333	93.7725349
452.237036	473.819546	385.914862	390.714241	350.977202
864.700783	989.609122	974.861237	1004.02289	1060.96925
4309.50961	4297.51015	4592.23187	4673.54342	4705.59511
33.1444082	33.134234	44.9459076	62.927534	75.0180279
22434.3451	22421.9362	24345.1833	24100.3063	23836.9784
16327.672	16485.3859	20881.2487	20663.7116	20234.3269
3479.42632	3385.21424	4015.68437	4070.56616	3980.42084
2530.7597	2444.202	2930.78315	2832.67825	2803.35226
2616.19862	2645.21635	2936.98258	2848.64494	2723.86887
310.084352	286.058887	193.73236	200.053205	178.614352
4372.11571	4199.21192	3172.56113	3216.81797	3295.4348
273.993774	322.506544	381.265285	393.531892	430.460589
1028.94974	1111.10131	1362.32596	1222.86044	1334.24921
1176.25822	1204.98164	1419.67074	1314.9037	1305.67092
891.216309	1003.96729	1399.52257	1358.10768	1252.97968
1868.60808	1894.17371	2067.51175	2103.84591	2280.01221
37.5636626	28.7163361	12.3988711	14.0882539	8.93071761
53.0310531	39.7610808	29.4473188	33.8118093	28.5782964
483.90836	417.491349	669.539037	585.132145	593.892721
5990.29938	5724.49116	6413.31606	6132.14731	6385.46309
2586.73692	2548.0226	1669.19802	1746.00427	1705.76706
1149.00615	1165.22056	1384.02398	1378.77045	1403.90881
7080.38213	7452.9937	7434.67306	8327.09727	8550.26904
6799.75948	6955.98019	7850.03524	7578.54138	7471.43835
1745.6055	1737.33834	2417.77986	2417.54437	2341.63416
422.038798	404.237655	82.1425208	88.286391	88.4141043
25261.1948	25343.2711	15991.444	15493.3224	15910.0734
458.865918	460.565853	650.940731	584.192928	668.910749
1202.77375	1160.80267	1379.37441	1342.14099	1294.95405
117.110242	99.402702	54.2450609	62.927534	45.5466598
54.5041379	56.3281978	21.6980244	18.7843385	20.5406505
464.021715	438.476363	513.00329	563.530156	588.534291
41.9829171	44.1789787	4.64957665	9.39216926	16.0752917

397.732898	423.013721	381.265285	339.05731	286.676035
332.180624	339.073661	252.626998	259.223872	260.776954
164.248956	182.238287	128.638287	132.429587	104.489396
2230.98694	2422.11251	2005.51739	2148.92833	1965.65095
2060.84565	2184.6505	2600.66321	2539.64257	2444.33741
4330.1328	3984.94388	3366.29349	3674.21661	3778.58662
6386.55919	6556.16044	7146.39931	6956.77977	6832.89204
14733.7943	14688.4059	16664.0827	16301.049	16136.0206
1272.00873	1157.48924	1447.5682	1541.25498	1641.4659
7040.60884	7264.12857	7904.2803	7697.82193	7731.32224
275.466859	275.014142	175.134054	169.059047	141.105338
1481.18678	1645.66696	1793.18673	1807.05337	1778.99895
5513.0199	5534.52155	4801.46282	4618.12963	4893.14018
99.4332246	113.76087	72.8433675	61.0491002	68.7665256
6173.69843	6253.53443	8702.45763	8115.77346	8117.12924
3945.65766	3863.45169	4369.05219	4321.33708	4351.93869
17142.2879	16701.8629	18096.1523	17663.8527	18036.4773
2635.34872	2549.12707	3045.4727	2953.83723	3110.56894
745.380913	778.654499	892.718716	880.985477	883.247972
2955.74467	3276.97574	4392.30007	4427.46859	4502.86782
2238.35237	2264.17266	2909.08512	2920.96464	2995.36269
1151.21578	1272.35459	988.809967	932.642408	987.737368
829.346747	774.236601	1039.95531	1003.08368	987.737368
3407.98171	3388.52766	4181.51927	4228.3546	3952.73561
1675.63397	1651.18933	1481.66509	1511.20003	1546.80029
2431.32648	2472.91833	3205.10817	3291.01611	3411.53413
15299.4588	15492.4634	20076.872	20065.4304	19429.6692
829.346747	836.087171	1032.20602	996.509158	1068.11383
1689.62828	1652.2938	2391.43226	2295.44617	2292.51521
584.078127	589.789365	802.826901	733.528419	726.960414
3804.97806	3841.3622	4358.20318	4212.38791	4262.63152
101.642852	123.70114	63.5442142	60.1098833	49.1189469
1772.12103	1753.90545	2101.60865	2110.42043	1969.22323
7721.17403	7940.06694	8834.19563	8888.74899	8941.43447
4839.0836	4587.98694	5143.98163	5165.69309	5126.23191
6006.50331	5961.95317	6351.3217	6570.76161	6726.6165
3072.11837	2992.02133	2498.37252	2334.89328	2322.87965
2492.4595	2342.59034	2059.76246	2070.97332	2016.55604
15716.3418	15712.2538	18404.5742	18401.138	18107.03
12665.5832	12653.964	14131.6133	13763.2848	13952.4601
17031.07	17935.5609	19244.5978	19278.3666	18676.8097
983.28411	1001.75834	1328.22906	1258.55068	1331.57
2458.57855	2343.69482	2168.25258	2156.44206	2102.29093

7811.76874	7751.20181	8232.85039	8189.03238	8096.58859
1329.45904	1283.39933	1433.61947	1430.42738	1480.71298
6472.73465	6730.6674	7256.43929	7095.78388	7257.9942
654.786198	632.86387	561.048916	537.232082	541.201487
5270.69745	5445.05912	6290.87721	6689.10295	6907.017
968.553262	1111.10131	1156.19473	1176.83881	1169.92401
281.359199	324.715493	212.330667	219.776761	217.016438
5636.75902	5252.88057	6312.57523	6395.12805	6609.6241
1611.55478	1525.27924	2104.70836	2018.37717	2109.4355
1188.77944	1140.92212	1540.55973	1576.006	1574.48552
2509.39997	2435.3662	4348.90403	4345.75672	4404.62993
3248.152	3140.02091	3969.1886	3801.0109	4116.16775
3919.14213	4225.71931	4550.38568	4511.99811	4366.22784
9449.10251	10221.9112	11141.9355	10914.6399	10593.6172
1484.86949	1551.78663	1219.73894	1281.09189	1294.06098
1772.85757	1750.59203	407.612886	397.28876	437.605163
5257.43968	5376.58171	6557.45293	6493.74583	6463.16034
4137.89523	4251.12222	4896.00421	4740.22783	4733.28033
2479.93828	2679.45506	3267.10253	3055.27266	3048.94699
2298.74884	2382.35143	2893.58653	2773.50758	2772.09475
363.115405	342.387085	230.928974	261.102305	255.418524
9517.60095	9737.0469	10523.5418	10183.9291	9988.11458
30.934781	16.567117	3.09971777	2.81765078	4.46535881
4787.52563	4777.95654	4579.833	4589.0139	4219.76407
5591.82993	5716.75984	6650.44447	6869.4326	6595.33496
64.0791892	56.3281978	122.438852	122.0982	118.778544
844.814138	828.35585	968.661802	900.709032	968.089789
205.495331	201.014353	364.216837	367.233818	381.341642
6912.45047	6974.75626	8276.24643	7910.08495	8053.72114
155.410447	135.850359	97.6411096	125.855068	103.596324
3410.92788	3226.16992	4555.03526	4420.89407	4488.57867
3566.33832	3464.7364	3346.14533	3162.34339	3004.2934
5588.88376	5700.19272	5264.87062	5051.10863	4568.95513
375.636626	392.088436	210.780808	217.898327	234.877873
3821.91854	4171.60006	4392.30007	4262.16641	4099.19938
234.220485	213.163572	170.484477	143.70019	154.501415
8413.52389	8524.33394	9942.34473	9945.36803	10203.3449
2754.66859	2744.61905	2958.68061	2933.17446	2998.93497
5521.85841	5656.01375	6272.2789	6311.53774	6502.45549
2362.09149	2419.90356	3133.81466	3059.02953	3084.66986
3602.4289	3538.73619	3132.2648	3045.88049	2990.00426
336.599879	308.148376	271.225305	261.102305	269.707672
8219.81323	8814.81072	9190.66317	9095.37671	8928.93147

522.945107	483.759817	672.638755	727.893118	653.728529
3374.10075	3302.37866	3821.95201	3885.54042	3949.16333
2208.89067	2206.73999	1838.13264	1903.79271	1939.75187
2613.25245	2636.38055	3039.27327	3006.43338	2962.31903
74.3907828	54.1192489	17.0484477	19.7235554	33.0436552
11450.2882	11656.6235	13353.5841	13011.9113	12613.7456
3858.00911	3903.21277	4352.00374	4375.81166	4142.9599
1042.94405	1132.08633	1658.349	1486.78039	1477.14069
6816.69995	6972.54731	7476.51925	7324.95281	7510.73351
1638.07031	1679.90566	1883.07854	2078.48706	2115.687
2242.77162	2205.63551	2055.11288	1936.6653	2175.52281
1878.91967	1944.97954	1650.59971	1645.50805	1644.14511
2088.83426	2165.87443	2530.91956	2449.47774	2366.64017
382.265508	434.058466	553.299621	534.414431	483.151823
234.220485	250.715704	190.632643	186.904168	165.218276
745.380913	679.251797	829.174502	908.222767	869.851895
2147.75765	2385.66485	1573.10677	1498.99021	1431.59403
3760.78552	3899.89934	4355.10346	4295.97822	4114.3816
4118.00858	4177.12243	5480.30101	5265.25009	5237.86588
3184.80936	3220.64755	3876.19707	3600.95769	3583.89698
3332.85438	3376.37845	2743.25022	2718.09378	2719.40351
1726.4554	1717.4578	2148.10441	2014.62031	1962.97173
2706.05679	2658.47004	3152.41297	3053.39423	2858.72271
444.871612	431.849517	299.122764	315.576887	316.147403
19.1501025	19.8805404	94.5413918	90.1648249	95.5586784
1876.71005	1842.26341	2754.09923	2719.033	2643.49241
1320.62053	1336.41411	1221.2888	1156.17604	1161.88636
1774.33065	1737.33834	1951.27233	2004.28892	1979.04702
640.791892	616.296753	542.450609	541.928166	534.949985
7425.08398	7246.45698	8576.91906	8461.40529	8184.10962
55750.3677	53322.9228	42844.299	44428.7175	46919.3111
35.3540354	35.3431829	63.5442142	77.0157879	81.2695303
925.833802	948.743567	1097.30009	1072.58573	1035.96324
2529.28662	2635.27608	2721.5522	2714.33692	2821.21369
212.124213	186.656185	261.926151	246.074835	252.739308
2194.15982	2169.18785	1974.52022	2020.25561	1855.80312
3832.23013	3809.33244	4877.4059	4861.38681	4635.93551
291.670792	288.267836	392.114297	385.07894	352.763346
4464.18351	4375.92784	3806.45342	3795.3756	3757.1529
1864.18883	1979.21825	2062.86217	2107.60278	2245.18241
4541.52047	4345.00255	3378.69236	3475.10263	3526.74038
1303.68006	1442.44365	1549.85888	1528.10594	1543.228
2408.49366	2427.63488	3000.5268	2752.84481	2834.60977

89.8581733	68.477417	111.58984	139.943322	154.501415
1082.71733	1191.72795	976.411096	891.316863	976.127435
1269.06256	1286.71275	1489.41439	1482.08431	1466.42383
3522.14578	3267.03547	3933.54184	4073.38381	3818.77485
2217.72918	2011.248	1878.42897	1912.24566	1990.65696
201.812619	223.103842	133.287864	148.396274	136.639979
114.164073	146.895104	63.5442142	77.0157879	73.2318844
1941.52578	1812.4426	2021.01598	2123.56947	2303.23207
1890.70435	2049.90461	2117.10723	2081.30471	2273.7607
2262.65827	2126.11335	1630.45155	1725.34149	1834.3694
8843.66465	8790.51228	9621.52394	9603.49307	9874.69446
2718.57802	3026.26004	3454.63545	3486.37323	3404.38955
91.3312581	85.044534	41.8461898	41.3255447	26.7921528
5759.02506	5478.19336	6757.38473	6529.43607	6576.58045
504.531547	468.297174	578.097363	562.590939	600.144223
3443.33574	3575.18385	3865.34805	3924.04832	3678.56258
5260.38585	5705.7151	6151.38991	6047.61779	5841.58239
483.90836	547.819336	977.960955	1009.6582	994.881942
71704.6127	77032.6762	102149.649	100096.105	102005.764
14365.5231	13239.3354	15789.9623	17484.4623	17954.3147
25.7789842	44.1789787	7.74929441	23.4804232	19.6475787
67149.8345	67792.6428	82412.1962	82622.913	85362.4781
148576.807	158092.266	202758.739	201687.443	198487.878
850.706477	859.281135	406.063027	371.929903	413.492225
184.135601	178.924864	57.3447787	56.3530156	54.4773774
1238.12778	1296.65302	1157.74459	1148.6623	1097.58519
1277.90107	1262.41432	959.362648	928.88554	970.769004
2301.69501	2390.08275	1732.74223	1677.44143	1659.32733
693.086403	637.281767	833.824079	815.240292	868.065752
1414.16142	1423.66759	1269.33443	1277.33502	1211.89838
5202.199	5372.16381	6490.809	6334.07895	6337.23722
2054.95331	1995.78536	1669.19802	1535.61967	1631.64211
231.274315	251.820179	204.581373	201.931639	187.54507
10194.4834	10245.1052	6822.4788	6890.09537	6694.46592
3111.89166	3089.21508	2228.69707	2102.9067	2032.63133
886.060513	878.057201	1253.83584	1213.46827	1220.8291
5370.86721	5769.77462	6362.17071	6334.07895	6116.64849
3914.72288	4001.51099	4603.08088	4509.18046	4599.31957
12318.6717	11793.5784	10208.9205	10259.0665	10719.5404
16.9404753	22.0894893	6.19943553	13.149037	6.25150233
13614.9863	13729.7221	15315.7055	15556.2499	15578.7438
2840.10751	2805.36515	3324.4473	3151.07279	3245.42278
4134.21252	4329.53991	4936.30054	4689.51011	4658.26231

14025.977	13492.2601	15724.8682	14919.4609	15366.1927
8352.39087	8394.00595	10528.1914	10205.5311	10310.5135
39980.9948	39371.2013	47162.2058	47045.3758	47663.2399
1096.71164	1022.74336	863.271398	852.808969	856.455819
1216.76805	1166.32504	1366.97553	1368.43906	1425.34253
8753.06993	8525.43841	9929.94586	9740.61874	9581.76692
44202.8559	43258.9514	48949.1931	46330.6317	46774.6335
6424.85939	6204.93756	7710.54794	7788.92597	7468.75914
5102.02924	5183.29867	5842.96799	5629.66625	5699.58398
4347.80981	4493.00213	4919.25209	4846.35934	4883.31639
10822.7541	10526.7461	13807.6928	13348.151	12862.9126
5201.46246	5161.20918	3769.2568	3647.91854	3608.00992
2147.02111	2120.59098	2535.56913	2471.07973	2535.43073
1492.97145	1724.08464	1835.03292	1819.26319	1752.2068
2300.95847	2408.85881	3904.09453	3854.54626	3832.17093
3547.18822	3326.67709	2444.12746	2417.54437	2416.65219
1097.44818	1107.78789	1201.14063	1269.82128	1297.63327
1246.96629	1265.72774	995.009403	1011.53663	1120.80506
684.984436	735.579995	652.49059	612.369436	589.427362
1579.14692	1764.9502	1236.78739	1274.51737	1241.36975
486.117987	445.10321	283.624176	289.278813	309.002829
2239.08891	2112.85966	2758.74881	2673.95059	2601.51804
2532.23279	2834.08148	3109.01692	2937.87054	2980.18047
1721.2996	1642.35353	1357.67638	1280.15267	1307.45706
81045.4435	84340.9837	69996.2767	68995.8146	69310.4063
2499.08838	2592.20157	1537.46001	1590.09426	1560.19637
4240.27462	4310.76384	3473.23376	3478.85949	3444.57778
1463.50976	1379.48861	1766.83913	1623.90607	1637.00054
2137.44606	2267.48608	1811.78503	1798.60041	1804.00496
460.339003	492.595612	209.230949	175.633565	154.501415
4808.14882	4863.00108	5994.85416	5794.96843	5261.08574
6938.96599	7192.33773	8155.35744	8022.79098	7524.12959
1420.7903	1438.02576	991.909685	984.299338	1053.82468
499.37575	498.117985	358.017402	374.747553	350.977202
1739.71316	1746.17413	2323.23847	2318.92659	2261.2577
2406.28404	2379.038	2938.53244	3001.7373	2907.84165
2606.62357	2520.41073	1532.81044	1551.58636	1573.59244
1762.54597	1592.65218	2237.99623	2095.39296	2021.0214
1666.79546	1639.04011	2069.06161	1911.30644	1899.56364
9972.78416	10608.4773	12419.0192	11958.1099	11943.0487
6211.2621	6490.99644	7473.41953	7215.06443	7207.98218
1817.78665	1854.41263	2163.603	2132.02242	2037.09669
8437.09324	8936.30291	9712.96562	9734.98344	9691.61475

9174.37219	9078.78012	10060.134	9831.72278	9815.75173
4650.52874	4785.68787	4176.86969	4011.39549	3930.40882
2404.81095	2475.12728	2639.40968	2669.2545	2538.10995
40.5098322	44.1789787	27.8974599	15.0274708	16.0752917
1761.07289	1751.6965	2242.6458	2127.32634	2085.32256
938.355023	985.191225	1427.42003	1347.77629	1343.17993
8166.04564	8264.78244	8860.54323	8758.19783	9017.34557
1105.55015	1161.90714	929.91533	896.012947	893.964833
9149.32975	8887.70604	8259.19799	7725.05922	7537.52566
5877.60839	5749.89408	5119.18389	4920.55748	5132.48341
6069.10941	5864.75942	6456.71211	6816.83645	7143.68102
4634.32481	4409.06207	3718.11146	3688.30487	3666.95265
1834.72713	1820.17392	2241.09594	2119.8126	2091.57406
2804.01693	2992.02133	4113.32548	3920.29145	3625.87135
1999.71263	2174.71023	1518.86171	1498.99021	1507.50513
17569.4825	17410.9355	19749.8517	19661.5671	19457.3545
10830.8561	10810.5961	10388.7041	10380.2255	10333.7334
10953.8586	11476.5942	12482.5634	12649.3736	12497.6462
522.208565	450.625583	520.752585	560.712505	604.609582
1562.94298	1760.5323	1484.76481	1379.70966	1434.27325
27646.8557	28128.7557	17956.665	17908.0491	18066.8417
921.414548	1074.65366	660.239884	661.208716	660.873103
3581.80571	3534.31829	4105.57618	4085.59363	4046.50815
271.047605	314.775223	336.319378	355.963215	348.297987
898.581733	927.758552	985.710249	1068.82886	1066.32768
55.2406803	44.1789787	32.5470365	27.2372909	36.6159422
445.608155	461.670327	706.735651	691.263658	729.639629
1400.90365	1336.41411	1636.65098	1521.53142	1561.98251
8613.86342	8577.34871	7642.35415	7775.77693	7691.13401
11210.9119	11609.1311	12164.8424	12156.2847	12240.4416
626.061044	715.699455	872.570551	888.499212	946.656067
424.248425	478.237444	595.145811	570.104674	567.99364
91.3312581	83.9400595	51.1453431	65.7451848	57.1565927
8812.72987	8870.03444	11188.4313	10805.6907	10885.6517
1003.9073	1031.57915	948.513636	916.67572	812.695303
49.3483411	34.2387085	21.6980244	14.0882539	16.9683635
247.478248	227.52174	381.265285	400.10641	378.662427
3003.61993	3081.48376	2735.50093	2807.31939	2647.0647
486.117987	450.625583	636.992001	591.706663	589.427362
2664.07388	2681.66401	3026.8744	2835.4959	2881.94257
1267.58948	1336.41411	1619.60253	1605.12173	1423.55639
136.260345	104.925074	89.8918152	74.1981372	86.6279608
567.137651	501.431408	624.59313	648.998896	615.326443

2048.32443	2259.75476	1704.84477	1692.4689	1687.90563
2104.30165	2001.30773	1253.83584	1294.24092	1352.11065
96.487055	111.551921	68.1937908	82.6510895	68.7665256
262.209096	308.148376	215.430385	209.445375	215.230294
790.31	936.594348	1007.40827	1011.53663	993.98887
162.775871	147.999579	38.7464721	32.8725924	36.6159422
1453.19816	1632.41326	1258.48541	1260.42912	1179.7478
1381.75355	1528.59266	2555.7173	2558.42691	2445.23048
2321.58166	2469.60491	2007.06725	2086.94001	2031.73826
2745.09354	2668.41031	2317.03903	2336.77171	2369.31938
1220.45076	1188.41453	874.12041	908.222767	893.964833
1238.86432	1230.38456	1486.31467	1367.49984	1431.59403
1799.37309	1842.26341	2278.29256	2235.33628	2148.73066
304.928555	301.52153	235.57855	205.688507	218.802582
154.673905	165.67117	99.1909685	112.706031	114.313185
1744.86896	1652.2938	1999.31796	1978.93006	1888.84678
4132.00289	3973.89913	4336.50515	4380.50774	4438.56665
310.820895	358.954202	485.10583	453.641775	455.466598
2133.0268	2088.56122	1855.18108	1747.8827	1793.2881
716.65576	693.609965	268.125587	271.433692	249.167021
2644.92377	2705.96244	3832.80102	3771.89518	3574.96626
2446.05733	2474.02281	2952.48117	3069.36091	2972.14282
777.788779	804.057412	957.81279	960.818915	998.454229
2066.00144	2117.27755	2569.66603	2601.63089	2396.11154
71.4446132	64.0595191	37.1966132	29.1157247	24.1129376
1174.78514	1203.87717	424.661334	454.580992	426.888302
35.3540354	48.5968765	7.74929441	8.45295233	8.93071761
1741.18624	1813.54708	2430.17873	2490.80329	2382.71546
2068.21107	2218.8892	2499.92238	2510.52684	2304.12514
241.585909	244.088857	150.336312	169.059047	191.117357
2030.64741	2127.21782	1859.83066	1723.46306	1656.64812
1965.09514	1958.23323	2420.87958	2359.31292	2257.68541
41399.5755	45715.3027	54677.4715	53584.2041	50314.77
3318.12353	3450.37824	2980.37863	2964.16862	2859.61578
9201.62426	9182.60072	10298.8123	10346.4137	11064.2661
6485.99241	6615.80206	7036.35933	7054.45833	7111.53043
464.758257	489.282189	596.69567	575.739976	546.559918
783.681118	840.505069	942.314201	941.09536	910.040125
4429.56602	4752.55363	5571.74268	5453.09347	5328.9592
738.015489	777.550025	621.493412	648.059679	651.942386
2696.48174	2900.34995	1501.81326	1511.20003	1587.88159
3635.57331	3734.22817	4054.43084	3957.86013	3979.52777
889.743225	848.236391	1156.19473	1143.027	1202.96766

341.019133	309.252851	158.085606	191.600253	192.9035
965.607092	992.922546	1218.18908	1184.35254	1132.41499
760.848304	724.53525	550.199903	607.673351	618.89873
3989.85021	3977.21256	4477.54231	4419.95485	4406.41607
4443.56033	4233.45063	5252.47175	5072.71062	5032.45937
4965.76889	4881.77714	4500.7902	4503.54516	4392.12692
654.049655	678.147323	872.570551	783.306916	751.966423
108.271733	111.551921	65.0940731	72.3197033	49.1189469
1230.76236	1202.77269	1518.86171	1452.96858	1419.9841
1498.12725	1644.56248	1244.53668	1368.43906	1301.20556
1047.3633	1153.07134	1374.72483	1297.05858	1311.92242
1898.06978	2025.60617	691.237062	654.634197	675.162251
19.1501025	27.6118617	12.3988711	1.87843385	7.14457409
11936.4062	11595.8774	13806.1429	13595.165	13637.2058
462.54863	480.446393	629.242706	606.734134	670.696893
928.779972	961.997261	722.234239	648.059679	728.746557
385.211678	385.461589	505.253996	448.945691	437.605163
1078.29808	1082.38498	1797.8363	1934.78687	1804.00496
380.055881	423.013721	181.333489	199.113988	181.293568
3270.98482	3380.79634	3635.96894	3531.45564	3695.53095
370.480829	393.19291	719.134522	664.965584	644.797812
128.158378	140.268257	229.379115	198.174771	190.224285
19486.7024	19558.0339	25933.7887	26119.6227	26467.0747
9973.5207	10598.537	11290.722	11619.9918	11615.2913
2670.70276	2619.81344	2995.87722	3014.88633	2969.46361
81.7562069	83.9400595	51.1453431	62.927534	58.0496645
638.582265	668.207053	492.855125	531.59678	467.969603
4201.23788	4291.98778	4981.24645	4970.33597	4973.51664
2499.08838	2538.08233	3160.16226	3061.84718	2902.48322
4976.81703	4923.74717	4727.06959	4667.90812	4677.90988
6124.35009	6012.759	5378.01032	5178.84213	5154.81021
3273.93099	3259.30415	3961.4393	3768.13831	3728.5746
553.879888	550.028285	756.331135	693.142091	684.986041
2092.51697	2095.18806	2907.53526	2632.62504	2366.64017
20229.8737	20251.6438	12589.5037	11996.6178	12075.2233
2739.93774	2588.88815	931.465189	1037.8347	934.153062
7214.43285	7412.12815	5064.93883	4997.57326	4719.88426
1275.69144	1394.95125	886.519281	954.244397	978.80665
1163.737	1204.98164	700.536215	831.20698	857.348891
106.062106	82.835585	15.4985888	10.3313862	13.3960764
1385.43626	1348.56332	1653.69943	1663.35318	1589.66774
5006.27872	5104.88099	6067.69753	5951.81766	5917.49349
4909.05513	4960.19483	7084.40495	6793.35603	6693.57285

4232.17266	4572.52429	4988.99574	4591.83155	4649.33159
1439.20386	1386.11546	1007.40827	1032.1994	1096.69212
1948.8912	1916.2632	2769.59782	2624.17209	2367.53324
450.763952	436.267414	525.402161	597.341965	617.112587
1766.96523	1899.69608	2363.5348	2343.34623	2363.96095
6010.92256	5799.59543	7364.92941	7294.89786	6668.56684
2601.46777	2563.48524	2837.79161	2832.67825	2806.03147
108.271733	120.387717	100.740827	72.3197033	73.2318844
262.945638	198.805404	159.635465	142.760973	142.891482
294.616962	248.506755	153.436029	133.368804	157.18063
118.583327	90.5669063	68.1937908	61.9883171	67.8734538
99353.678	98480.4658	80502.7701	85038.5789	86990.548
25295.0758	25515.5691	27072.935	28203.7451	27649.5017
12509.4362	12518.1136	13396.9802	13399.8079	13835.4677
676.88247	651.639936	736.182969	738.224504	726.960414
747.59054	710.177082	522.302443	592.64588	627.829448
7705.70664	7290.63596	8550.57146	8570.35445	8662.79608
474.333308	497.01351	646.291154	670.600885	654.621601
4819.19695	4699.53886	5336.16413	5099.94791	5132.48341
1831.04442	1950.50191	2442.5776	2355.55605	2280.01221
714.446132	701.341287	461.857947	467.730029	461.718101
5295.73989	5230.79108	3854.49904	3903.38554	4046.50815
5032.05771	5348.96984	5602.73986	5646.57216	5462.02689
325.551743	314.775223	193.73236	177.511999	221.481797
3117.784	3041.72268	4778.21494	4499.78829	4577.88585
162.039329	154.626425	289.823611	283.643512	255.418524
104.589021	142.477206	266.575728	274.251342	230.412514
323.342116	312.566274	565.698492	509.055574	618.005659
6099.30765	5776.40146	6493.90872	6178.16894	6256.86076
4694.72129	4716.10597	3085.76904	3121.01785	3207.91377
3332.11784	3323.36367	3797.15426	3676.09505	3611.5822
383.738593	453.939006	213.880526	281.765078	271.493815
3899.25549	3677.89998	3138.46424	3149.19435	3081.99065
19318.0342	19509.437	22361.364	22026.5154	21466.7659
114284.866	114324.152	92664.5127	94236.3303	95765.8711
43.4560019	37.5521319	71.2935086	71.3804864	75.9110997
5429.05406	5320.25351	7208.39366	6825.2894	7049.01541
4021.52153	4015.86916	4775.11522	4913.98296	4757.39327
1118.80791	1160.80267	1366.97553	1318.66056	1398.55038
1214.55843	1206.08612	1509.56255	1526.22751	1428.02175
2233.19657	2330.44113	1697.09548	1812.68867	1910.2805
41.9829171	77.3132127	26.347601	27.2372909	33.0436552
2513.08268	2633.06713	3549.17684	3832.94428	3876.82452

397.732898	437.371889	590.496234	503.420272	534.949985
1985.71832	2158.14311	2681.25587	2391.24629	2547.04066
1437.73077	1465.63762	1046.15475	1014.35428	979.699722
5083.61568	5284.91032	5892.56347	5553.58968	5491.49826
2664.07388	2552.44049	3606.52162	3549.30076	3809.84413
335.126794	387.670538	440.159923	461.155511	437.605163
724.021184	702.445761	632.342424	621.761605	634.08095
2055.68985	2180.2326	1864.48024	1960.14572	1895.99135
1333.87829	1402.68257	1026.00658	1096.06615	1088.65448
10951.649	11675.3996	13927.0319	13738.8652	13070.1052
1996.76646	1932.83032	2399.18155	2225.94411	2303.23207
1076.08845	1049.25074	1408.82172	1362.80376	1387.83352
1724.24577	1605.90588	2642.5094	2665.49764	2600.62497
309.34781	312.566274	176.683913	161.545311	169.683635
2344.41447	2564.58971	2961.78033	2794.17036	2686.35986
3309.28502	3289.12496	4192.36828	4039.572	3838.42243
2311.27007	2377.93353	2792.84571	2675.82902	2706.90051
1411.95179	1397.1602	1707.94449	1638.93354	1763.81673
4068.66024	4023.60048	5086.63685	4868.90054	4828.83901
41045.2986	41373.6135	47255.1973	46315.6043	46250.4004
63.3426468	75.1042638	147.236594	174.694348	139.319195
615.012908	633.968344	816.775631	838.720715	800.192298
12650.1158	12718.0235	14092.8668	13962.3988	13776.525
5423.16172	5721.17774	4702.27185	4800.33771	4629.68401
187.818313	173.402491	72.8433675	64.8059679	61.6219515
3564.1287	3791.66085	4383.00092	4272.4978	4209.94028
1653.5377	1642.35353	1976.07008	1875.6162	1829.90404
3425.65872	3371.96055	3628.21964	3801.95012	3774.12126
373.426999	371.103421	489.755407	446.12804	479.579536
315.976692	333.551289	416.912039	441.431955	429.567517
1459.0905	1475.57789	1300.3316	1267.00363	1203.86073
4825.08929	4834.28474	6851.92612	6626.17541	6721.25807
201.812619	221.999368	151.886171	144.639407	173.255922
515.579683	468.297174	348.718249	309.002369	305.430542
859.544986	876.952727	1018.25729	1058.49748	981.485865
78.0734949	70.6863659	48.0456254	44.1431955	42.8674445
1073.14228	997.340444	1250.73612	1258.55068	1320.85314
901.527903	807.370835	1222.83866	1160.87212	1191.35773
410.990662	408.655553	573.447787	529.718346	545.666846
1395.01131	1573.87612	1932.67403	1747.8827	1692.37099
12379.0682	13221.6638	14232.3541	14193.4462	14711.5711
6208.31593	6140.87804	7044.10862	6824.35018	7014.18561
209.914585	207.6412	316.171212	300.549416	313.468188

4618.85742	4738.19546	4908.40308	5106.52243	5086.04368
2177.21935	2354.73956	2609.96236	2643.89565	2636.34784
1765.49214	1940.56164	2174.45201	2187.43622	2125.51079
5070.35791	5379.89513	6286.22763	6360.37702	6046.09582
1577.67383	1587.12981	1999.31796	1979.86928	2051.38584
101.642852	83.9400595	24.7977421	15.9666877	21.4337223
38.300205	39.7610808	23.2478832	21.6019893	21.4337223
110447.48	112806.604	123078.943	125176.014	126782.253
16730.5607	16889.6236	18947.0248	19262.3999	18951.8758
20038.3727	21501.9089	23370.3221	22524.3003	22650.086
69261.5015	69410.6979	78425.9592	78797.4824	79253.8673
2155.12308	2248.71002	2648.70883	2695.55258	2738.15802
4185.03394	4156.13742	5122.28361	5121.5499	4944.93834
7960.55031	8211.76766	6937.16836	7142.74472	6970.4251
256.316757	235.253061	201.481655	161.545311	167.897491
79.5465797	89.4624318	48.0456254	58.2314494	49.1189469
151.727735	102.716125	52.695202	63.866751	58.9427362
1079.77117	1018.32546	720.68438	743.859805	656.407744
1135.74839	1187.31005	677.288332	685.628356	684.986041
455.919748	451.730057	314.621353	293.974898	284.889892
360.905778	316.984172	156.535747	180.32965	170.576706
49.3483411	53.0147744	29.4473188	15.0274708	22.326794
26.5155266	27.6118617	6.19943553	16.9059047	6.25150233
1311.78202	1232.59351	1531.26058	1576.94522	1522.68735
845.55068	857.072186	424.661334	428.282918	409.919938
2518.97502	2628.64923	3071.82031	2920.96464	2971.24975
2626.51021	2618.70896	3006.72623	3008.31181	3006.07955
5761.97123	5870.28179	6342.02255	6356.62016	6202.38338
1269.06256	1220.44429	1461.51693	1450.15093	1428.02175
2155.12308	2031.12855	2520.07054	2471.07973	2403.25611
6437.38061	6416.99665	7933.72762	7787.04753	7859.92457
408.044492	414.177925	268.125587	258.284655	289.355251
2247.92742	2139.36704	3316.69801	3229.96701	3281.14565
4711.66176	4687.38964	5683.33252	5480.33076	5419.15945
3664.29846	3893.2725	2874.98823	2754.72324	2741.73031
939.091566	829.460325	688.137344	647.120462	654.621601
2772.34561	2827.45464	5219.92472	5073.64983	5016.38408
27352.9753	28631.2916	23753.1372	23685.1724	24212.0685
2945.43308	2931.27524	3234.55549	3087.20604	3102.5313
1092.29239	1066.92234	1242.98682	1213.46827	1180.64087
170.141295	192.178557	514.553149	487.453585	474.221105
1161.52737	1104.47447	1292.58231	1273.57815	1255.6589
163.512414	142.477206	106.940263	121.158983	106.27554

390.367474	456.147955	82.1425208	77.0157879	54.4773774
6141.29057	6064.6693	6644.24503	6599.87734	6522.99614
1155.63503	1202.77269	804.37676	826.510895	814.481446
454.446664	457.252429	706.735651	774.853964	697.489045
11437.0305	11564.9521	13119.5554	13240.141	12872.7364
1168.8928	1157.48924	1463.06679	1382.52732	1310.13627
1464.2463	1418.14522	1810.23518	1798.60041	1740.59686
1570.30841	1484.41368	1966.77092	1850.25734	1806.68417
7145.19787	7145.9498	8135.20928	8751.62332	8776.2162
600.28206	488.177714	691.237062	664.965584	634.08095
14913.5106	15018.6438	16619.1368	16550.8807	16661.1468
849.969935	832.773748	610.6444	683.749922	632.294807
10477.3157	10717.8202	13190.849	12808.1012	12695.9082
499.37575	553.341708	433.960487	455.520209	462.611172
126.685294	155.7309	88.3419563	113.645248	103.596324
539.885582	497.01351	726.883816	588.889013	636.760166
3360.84299	3436.02007	2851.74034	2889.97048	2848.00585
3621.579	3884.4367	4178.41955	4032.99748	4031.32593
12144.1112	13169.7535	14060.3198	13733.2299	13832.7885
13.2577633	18.7760659	35.6467543	32.8725924	29.4713681
167.195126	151.313002	134.837723	118.341333	115.206257
1643.96265	1685.42804	2003.96754	1831.47301	1982.61931
391.104017	379.939217	303.772341	296.792549	276.852246
1160.05429	1232.59351	881.869704	872.532524	975.234363
279.886114	251.820179	137.937441	189.721819	175.042065
6122.87701	5812.84912	4985.89603	5271.82461	5347.71371
3288.66184	3195.24463	3591.02303	3556.8145	3648.19814
4647.58257	4339.48018	5825.91954	5790.27235	5316.45619
377.109711	397.610808	305.3222	250.770919	242.022447
457.392833	498.117985	361.11712	309.941586	320.612762
344.701845	339.073661	215.430385	190.661036	199.155003
8318.50992	8724.24381	10749.8212	10346.4137	9995.25915
2588.21001	2576.73893	2369.73423	2314.23051	2351.45795
1118.80791	1165.22056	954.713072	998.387592	1001.13344
1051.04601	1071.34023	1477.01552	1331.8096	1376.22358
36.0905778	35.3431829	13.9487299	22.5412062	22.326794
1375.86121	1461.21972	759.430853	865.958006	808.229944
12656.0081	12348.0245	11002.4482	11851.0392	11990.3815
7771.25891	8768.42279	9725.36449	9466.3674	9688.04246
14708.7518	15033.002	13618.61	13750.1358	13605.9483
2035.80321	1999.09879	2482.87393	2462.62678	2597.94575
17797.0741	17510.3382	18212.3917	19407.9786	19270.7025
12938.8404	12593.2179	11555.7478	11039.5558	11236.6289

10176.0699	10050.7177	11994.3579	11450.9328	11517.9465
5350.98057	5343.44747	6013.45247	5757.39976	5838.90317
1375.12467	1393.84678	1190.29162	1230.37417	1232.43903
1353.76494	1341.93648	1526.611	1481.14509	1465.53076
3214.27105	3135.60301	3302.74928	3465.71046	3474.94222
1925.32185	2043.27776	2200.79961	2208.09899	2138.0138
7847.85932	8448.1252	9784.25913	9598.79698	9169.16777
1802.31926	1823.48735	1512.66227	1636.11589	1685.22641
391.840559	353.431829	703.635933	613.308653	601.037295
1779.48645	1989.15852	2448.77704	2286.99322	2270.18842
676.88247	690.296542	511.453431	498.724188	495.654827
1228.55273	1351.87675	1086.45108	1115.78971	966.303646
7534.8288	7371.26259	8496.3264	8198.42455	8377.90619
986.23028	1089.01182	1219.73894	1200.31923	1115.44663
1891.44089	1957.12876	1332.87864	1335.56647	1276.19955
24148.2793	23283.4262	27471.2487	27427.0127	28617.5915
34372.9609	35071.4822	42300.2985	41452.339	41386.7316
3536.87663	3325.57262	2879.6378	2879.6391	2909.6278
6597.94686	6595.92152	7921.32875	7508.10011	7379.45196
915.522209	956.474888	683.487767	596.402748	659.980031
2593.36581	2488.38097	2859.48964	2736.87812	2909.6278
533.256701	566.595402	420.011757	435.796654	461.718101
5574.15292	5797.38648	5105.23516	4846.35934	5038.71088
1513.59464	1762.74125	1382.47412	1466.11762	1441.41782
55.2406803	65.1639936	21.6980244	18.7843385	26.7921528
73.6542404	65.1639936	24.7977421	10.3313862	39.2951575
5861.40445	5918.87867	5529.89649	5378.89534	5306.6324
4344.86364	4347.2115	6270.72904	6331.2613	6071.10183
2312.00661	2129.42677	2851.74034	2733.12125	2895.33865
126.685294	140.268257	247.977421	247.014052	252.739308
418.356086	442.894261	610.6444	591.706663	559.062922
80.2831221	78.4176872	100.740827	135.247237	104.489396
33.8809506	39.7610808	58.8946375	56.3530156	57.1565927
757.902134	745.520265	1044.60489	1087.6132	1091.33369
232.7474	202.118827	308.421918	304.306284	348.297987
148.781566	139.163783	302.222482	310.880803	270.600744
11.0481361	13.2536936	23.2478832	30.9941586	33.0436552
648.893858	728.953148	855.522103	941.09536	900.216335
278.413029	262.864923	451.008935	439.553521	458.145813
795.465797	885.788523	1227.48824	1205.95453	1378.00973
4863.3895	4822.13552	5112.98445	5301.87955	5110.15662
892.689394	907.878012	1182.54233	1198.4408	1171.71015
1281.58378	1304.38435	1594.80479	1576.94522	1397.65731

626.061044	658.266782	892.718716	910.101201	917.184699
3149.45532	3113.51352	1996.21824	1961.08494	2018.34218
4407.46975	4512.88267	3601.87204	3476.98106	3614.26142
2266.34098	2246.50107	2058.2126	2033.40465	2101.39785
925.833802	891.310895	1081.8015	1141.14857	1064.54154
1271.27219	1292.23513	1428.96989	1462.36075	1493.21598
407.30795	424.118195	288.273752	330.604358	283.103748
230.537773	208.745674	161.185324	164.362962	155.394486
2793.70534	2654.05214	1670.74788	1602.30408	1538.76264
1473.82135	1422.56311	1822.63405	1869.9809	1838.83476
10438.279	9466.45066	10542.1401	11103.4225	11842.1316
5275.85324	5230.79108	5858.46658	5791.21157	5645.99967
32765.8254	32413.0122	36043.5182	36591.8914	36785.6258
2420.27834	2354.73956	3155.51269	3065.60405	3078.41836
8894.48608	8759.587	9500.63495	10005.4779	9857.7261
25771.6187	23801.4248	20842.5023	23251.2542	23260.9471
32798.2333	31732.6559	35077.9561	34618.5967	35943.4592
12062.355	11387.1318	9339.44963	10019.5662	10364.0978
10226.8913	9572.48021	7856.23468	8539.36029	8787.82613
20819.8441	20952.9851	23440.0657	23126.3384	23068.0436
30702.7701	29838.4822	24676.8531	25270.5706	24979.2172
2620.61788	2408.85881	3101.26762	2919.08621	3008.75876
14089.3197	13997.0049	15328.1044	16216.5194	16205.6802
17414.0721	18047.1128	20174.5131	20183.7717	20121.7999
3082.42996	3010.7974	3848.29961	3872.39139	3850.03236
2412.91292	2422.11251	2055.11288	2132.02242	2100.50478
324.078658	309.252851	424.661334	423.586834	437.605163
476.542936	459.461378	564.148633	628.336123	591.213506
568.610736	584.266993	731.533393	729.771552	720.708911
853.652647	925.549603	1396.42285	1332.74882	1384.26123
171.61438	181.133813	238.678268	249.831702	254.525452
96.487055	101.611651	145.686735	130.551153	139.319195
4718.29064	4477.53949	4079.22858	4177.63689	4381.41006
1458.35396	1593.75666	1684.69661	1784.51216	1673.61648
173.087465	162.357747	243.327845	230.108147	237.557088
2052.00714	2020.0838	2799.04514	2602.5701	2523.8208
436.033103	434.058466	328.570083	344.692612	349.191059
265.155266	305.939427	209.230949	194.417904	172.36285
149.518108	176.715915	255.726716	285.521946	225.054084
3389.56815	3395.15451	3649.91767	3972.8876	3741.97068
1179.94093	1148.65345	864.821257	916.67572	854.669675
435.296561	421.909246	314.621353	329.665141	336.688054
168749.967	164460.666	185451.464	180603.901	187282.507

109166.633	108155.662	128695.632	127307.097	128859.538
27223.3438	26422.3427	28858.3724	28607.6084	29322.2251
119.31987	112.656396	60.4444964	42.2647617	46.4397316
88352.6807	87524.0791	100238.673	96481.0587	97485.0342
70656.5129	75939.2465	80561.6647	86311.2178	86903.0269
1462.03667	1471.15999	513.00329	472.426114	502.799402
38851.1388	38017.1156	49688.4758	48781.9879	49012.6713
296.826589	278.327566	209.230949	247.014052	177.72128
15893.112	15481.4186	20774.3085	20083.2755	20139.6613
8107.12225	8370.81199	9951.64389	9703.05006	8875.34716
9687.0057	9962.35969	10692.4764	10674.2004	10545.3914
12021.8451	11926.1153	13993.6759	13494.6688	13149.5886
2778.97449	2675.03716	2991.22764	2862.73319	3006.07955
2647.86994	2804.26067	5391.95905	5213.59316	5171.77857
3734.26999	3677.89998	4293.10911	4122.22309	4389.44771
3475.74361	3672.3776	5137.7822	4985.36344	5161.06171
9729.72516	9817.67354	10776.1688	10734.3103	10835.6397
285.778453	272.805193	176.683913	200.992422	186.651998
1278.63761	1452.38392	841.573373	805.848123	773.400145
4204.18404	4771.3297	4875.85605	4851.05542	4889.56789
41732.4926	44409.8138	35942.7774	31599.0143	33940.2992
8075.45092	8100.21574	7076.65566	7301.47238	7403.5649
186.345228	187.760659	57.3447787	42.2647617	56.263521
58.9233924	59.6416212	49.5954842	24.4196401	30.3644399
8475.39345	8634.78138	5743.77702	6252.36708	5869.26761
3077.27417	3082.58824	2843.99105	2823.28608	2891.76636
863.227698	858.176661	717.584663	760.76571	726.067342
2021.07236	2026.71065	2492.17308	2302.02069	2332.70344
262.209096	326.924442	156.535747	193.478687	209.871864
34892.9599	35431.5409	38228.8192	38303.1447	35950.6037
6387.29573	6655.56314	8279.34615	8072.56948	7600.93376
3100.10698	3424.97532	3725.86075	3841.39723	3877.71759
499.37575	513.580627	1563.80761	1679.31986	1510.18435
14740.4231	14929.1814	15617.928	16259.7234	16429.8412
39382.1858	39151.4109	52554.1649	52141.5669	50555.0063
90.5947157	108.238498	6.19943553	3.7568677	10.7168611
42.7194595	30.9252851	17.0484477	19.7235554	12.5030047
20.6231873	12.1492191	3.09971777	4.69608463	8.03764585
8333.97731	8461.37889	10721.9238	9973.54454	9761.27435
8537.26301	8696.63195	11197.7304	11075.246	10585.5796
979.601398	1058.08654	1222.83866	1207.83297	1158.31407
729.17698	795.221616	892.718716	855.62662	946.656067
374.900084	363.3721	300.672623	301.488633	226.840227

124.475666	134.745885	246.427562	217.898327	209.871864
259.999469	266.178347	460.308088	518.447743	491.189469
922.15109	960.892786	1094.20037	1039.71314	1003.81266
2381.97814	2446.41094	2577.41532	2590.36028	2671.17764
1884.81201	1805.81575	1608.75352	1525.28829	1436.95246
1188.0429	1256.89194	914.416741	939.216926	910.933196
13958.9517	13512.1406	15848.8569	15121.3925	15492.1158
2527.07699	2681.66401	2805.24458	2891.84892	2809.60376
2049.79751	1965.96455	1735.84195	1747.8827	1788.82274
11335.3876	11085.6102	15298.657	14937.306	15540.3417
4022.99461	3810.43691	3591.02303	3640.40481	3701.78245
2715.63185	2762.29064	3294.99998	3345.49069	3206.12762
9045.47727	9668.56948	7538.51361	7264.84292	7321.4023
1849.45798	1985.84509	1627.35183	1623.90607	1683.44027
446.344697	473.819546	142.587017	161.545311	182.186639
855.125732	883.579574	536.251173	485.575151	493.868684
2309.79698	2320.50086	2768.04796	2663.6192	2597.05268
256.316757	265.073872	361.11712	349.388696	345.618772
5602.14153	5638.34215	6703.13967	6714.4618	6807.88603
504.531547	500.326934	638.54186	595.463531	578.710501
2036.53975	2003.51668	2594.46377	2536.82492	2596.15961
5804.69069	5695.77483	6802.33064	6366.95154	6517.63771
2893.87511	2764.49959	3285.70083	3193.33755	3273.108
3539.8228	3588.43754	4175.31983	4077.14068	3977.74162
14733.0577	15482.5231	18168.9957	18025.4512	17977.5346
4596.76115	4840.91159	4290.00939	4204.87418	4239.41165
7647.51979	7900.30586	6898.42189	6691.9206	6798.06225
17786.026	18113.3813	19331.3898	19604.2749	18727.7148
1526.8524	1675.48777	1297.23189	1285.78797	1235.11825
1470.87518	1555.10005	1766.83913	1941.36139	1863.84077
3759.31243	3762.94451	4028.08324	3851.72861	4099.19938
26.5155266	35.3431829	63.5442142	61.0491002	65.1942386
2786.33992	2918.02154	3112.11664	3257.2043	3366.88054
2610.30628	2803.1562	4347.35417	4197.36044	4312.64353
128.894921	133.641411	65.0940731	74.1981372	77.6972432
78.0734949	67.3729425	24.7977421	25.358857	22.326794
8001.06014	7753.41076	6343.57241	6204.46701	6257.75383
4446.5065	4486.37529	4093.17731	4112.83092	4271.56223
5831.94276	6174.01227	8415.73373	8006.82429	7984.06154
304.928555	328.028917	252.626998	203.810073	194.689644
398.469441	439.580838	519.202726	570.104674	530.484626
353.540354	354.536304	579.647222	564.469373	562.63521
4626.95938	4839.80711	5266.42048	5141.27345	5240.54509

7559.1347	7502.69505	8333.59121	8030.30472	7784.90654
1118.07137	1135.39975	1328.22906	1456.72545	1357.46908
4261.63435	4275.42066	6173.08793	5987.5079	6117.54156
1153.42541	1176.26531	818.32549	805.848123	740.35649
10346.2112	10474.8358	12555.4068	12192.9141	11901.0743
1509.17539	1534.11504	1272.43414	1272.63894	1308.35013
97.2235974	106.029549	80.5926619	75.1373541	66.9803821
194.447195	168.984593	123.988711	108.009947	108.061683
420.565713	374.416844	675.738473	683.749922	760.89714
97.2235974	107.134023	123.988711	146.51784	161.645989
918.468378	1006.17624	802.826901	759.826493	759.110997
7442.02445	7530.30692	8068.56534	8161.79509	8287.70594
7458.96493	7898.09691	5731.37815	5679.44475	5486.13983
8112.27804	9284.21237	11069.0921	10980.3851	10777.59
22433.6086	24179.155	28627.4434	27669.3306	27665.577
477.279478	473.819546	607.544682	551.320336	586.748147
1903.96212	1829.00972	2090.75963	1995.83597	2091.57406
4397.8947	4263.27144	5254.02161	5118.73225	4986.01964
115.637158	110.447447	178.233772	192.53947	181.293568
998.7515	964.20621	1128.29727	1117.66814	1208.32609
192.237568	234.148587	342.518813	376.625987	326.864265
400.679068	456.147955	520.752585	479.000632	502.799402
318.922861	337.969187	156.535747	106.131513	128.602334
458.129376	477.13297	435.510346	380.382855	353.656417
938.355023	968.624108	733.083252	714.744081	705.526691
254.10713	270.596244	382.815144	359.720083	390.27236
1021.58432	973.042005	838.473656	852.808969	850.204317
551.670261	573.222248	179.78363	167.180613	180.400496
2731.09924	2616.50001	1782.33772	1895.33976	2022.80754
2453.42275	2455.24674	2834.6919	2879.6391	2834.60977
1842.09255	1930.62137	2138.80526	2023.07326	1947.78951
7156.98254	7176.87509	6915.47033	6523.80077	6320.26885
1123.22717	1038.206	863.271398	865.958006	858.241962
852.179562	872.534829	1036.85559	1107.33676	990.416583
6852.05399	6853.26407	7876.38284	7771.08085	7558.95939
2741.41083	2650.73872	3453.08559	3219.63562	3235.59899
3096.42427	3085.90166	3387.99152	3479.79871	3377.5974
6667.91839	6647.83182	7650.10345	7617.98849	7298.18243
3064.0164	3007.48397	4152.07195	3988.85428	3938.44647
416.883001	453.939006	520.752585	484.635934	567.100568
2230.2504	2367.99326	2572.76575	2612.90149	2485.41871
2133.76335	2039.96434	1926.47459	1783.57294	1796.86038
14430.3388	14471.9289	17435.9124	16849.5517	16754.9193

12506.49	12704.7698	14235.4538	14400.0739	14815.1674
7385.31069	7311.62097	8462.2295	8495.2171	8475.25101
14111.4159	14460.8842	16416.1053	16229.6685	15247.4142
222.435806	240.775434	336.319378	289.278813	321.505834
2796.65151	2710.38034	3243.85464	3289.13768	3083.77679
156.883532	172.298017	91.4416741	73.2589202	85.7348891
9773.18116	9749.19612	9212.3612	9254.10437	8676.19216
16200.2502	15249.479	14367.1918	14474.2721	14908.0469
1490.02528	1499.87633	1855.18108	1945.11825	1810.25646
984.020652	1018.32546	798.177325	822.754027	814.481446
2735.51849	2701.54455	2396.08183	2347.1031	2361.28174
5722.19794	5804.01332	6193.2361	6088.00411	6234.53396
1260.22405	1309.90672	1577.75634	1563.79618	1542.33493
1361.86691	1286.71275	1698.64534	1618.27076	1444.09704
1577.67383	1650.08485	998.10912	930.763974	984.165081
2840.10751	2720.32061	2227.14721	2251.30297	2311.26972
4654.948	4578.04667	5350.11286	5130.00285	5091.40211
4890.64157	4612.28537	6103.34428	5601.48975	5469.17147
504.531547	554.446182	161.185324	134.30802	141.99841
3057.38752	3130.08064	3690.214	3519.24582	3335.62303
1161.52737	1175.16083	1559.15804	1571.30992	1520.90121
2648.60649	2815.30542	3294.99998	3244.05526	3195.41076
1465.71939	1549.57768	1898.57713	1859.64951	1859.37541
811.66973	793.012667	1136.04656	1010.59741	1041.32167
3482.37249	3558.61673	5012.24363	4956.24772	4817.22908
3225.31919	3198.55806	4122.62463	3899.62868	4092.05481
2208.89067	2140.47152	2935.43272	2786.65662	2798.8869
2234.66966	2207.84446	2788.19613	2857.09789	2836.39591
956.032041	978.564378	1432.06961	1447.33328	1418.19796
3142.82644	3314.52788	3687.11428	3523.00269	3663.38036
3299.70997	3766.25793	2419.32972	2348.04232	2300.55286
355.749981	350.118406	296.023047	278.00821	272.386887
81.0196645	72.8953148	49.5954842	38.507894	42.8674445
257.053299	277.223091	203.031514	241.37875	200.941146
993.595704	982.982276	1168.5936	1121.42501	1098.47827
158.356617	118.178768	55.7949198	56.3530156	54.4773774
5658.11875	5713.44642	4006.38521	3926.86597	3907.18896
880.168173	913.400384	636.992001	648.998896	614.433372
5119.70625	5188.82105	5776.32406	5734.85855	5924.63806
96.487055	112.656396	46.4957665	45.0824124	36.6159422
3496.36679	3349.87106	3618.92049	3736.20493	3613.36835
458.129376	469.401648	319.27093	356.902432	372.410924
723.284641	798.53504	663.339602	581.375277	582.282788

777.052237	826.146901	1022.90686	952.365963	867.17268
436.769646	386.566063	1008.95813	969.271868	968.982861
1257.27788	1272.35459	1455.31749	1404.1293	1361.04136
1819.99628	1882.02449	2211.64863	2167.71267	2079.96413
201.812619	220.894893	88.3419563	90.1648249	107.168611
215.806925	217.58147	96.0912507	85.4687403	96.4517502
4213.7591	4494.10661	4891.35463	4915.86139	4715.4189
5316.36308	5200.97027	6624.09686	6313.41618	6498.88321
1716.1438	1599.27903	2241.09594	2099.14983	2177.30895
4547.41281	4550.4348	4237.31419	4052.72104	3830.38478
24280.1204	23951.6333	31514.8305	31272.1668	31457.5597
933.935769	979.668852	444.809499	483.696717	445.642809
186.345228	224.208317	136.387582	120.219767	136.639979
821.244781	799.639514	389.01458	386.018157	383.127786
189.291398	216.476996	128.638287	119.28055	120.564688
130698.713	139963.422	102803.69	100400.411	102418.363
12.5212209	33.134234	1.54985888	1.87843385	7.14457409
215.806925	192.178557	373.515991	391.653458	369.731709
394.786729	452.834531	247.977421	232.925798	227.733299
1819.99628	1909.63635	729.983534	733.528419	764.469428
303.455471	329.133391	201.481655	219.776761	208.08572
33.1444082	25.4029127	13.9487299	6.57451848	11.6099329
6774.71704	7111.71109	4840.20929	5092.43417	5077.11296
3202.48637	3092.52851	3449.98587	3479.79871	3554.42561
13844.7876	13456.9169	12584.8541	12115.8983	12289.5605
8842.92811	8124.51418	5807.32123	5649.38981	5772.81586
223.908891	248.506755	111.58984	149.335491	129.495405
937.618481	945.430144	711.385227	774.853964	821.62602
139.943057	122.596666	102.290686	77.9550049	106.27554
1942.99886	1956.02428	2022.56584	2186.497	2273.7607
671.726673	637.281767	229.379115	256.406221	231.305586
715.919217	827.251376	337.869236	355.023998	368.838637
5636.02248	5768.67014	7637.70457	7211.30756	7023.11633
93.5408854	87.2534829	63.5442142	59.1706663	55.3704492
1581.35654	1451.27945	1512.66227	1868.10247	1847.76547
3345.3756	3120.14037	2654.90827	3006.43338	3076.63222
14337.5344	14032.3481	20481.3851	20272.9974	20407.5828
19094.8618	19446.4819	22361.364	22365.5727	21504.2749
5663.27455	5463.83519	6476.86027	6226.069	6341.70258
2519.71157	2551.33602	2225.59736	2169.5911	2347.88566
1000.96113	1024.95231	1486.31467	1537.49811	1408.37417
177.50672	167.880119	285.174034	283.643512	242.022447
84.7023765	76.2087382	20.1481655	25.358857	25.8990811

456.656291	511.371678	688.137344	699.71661	712.671265
3248.88855	3294.64734	3608.07148	3660.12836	3785.7312
32038.1215	31693.9993	19731.2534	19866.3164	20174.4911
349.1211	313.670749	261.926151	227.290496	206.299577
698.242199	716.803929	502.154278	545.685034	591.213506
5209.56443	5425.17858	6197.88567	6196.01406	6095.21477
1193.93524	1154.17582	1886.17826	1877.49464	1885.27449
5741.34804	5887.95338	6267.62932	6690.98138	6526.56843
36.0905778	29.8208106	7.74929441	4.69608463	0.89307176
7006.72789	7545.76956	4733.26903	4609.67667	4374.26549
1800.10964	1790.35311	1354.57666	1686.8336	1679.86798
16934.583	16628.9676	9486.68622	9858.02086	10331.9472
2662.60079	2504.94809	1848.98165	1792.96511	1736.1315
1944.47195	1802.50233	2021.01598	2020.25561	2152.30294
1398.69403	1306.59329	1084.90122	1135.51326	979.699722
293.143877	318.088647	133.287864	149.335491	140.212267
369.744287	343.491559	167.384759	148.396274	159.859845
439.715815	461.670327	652.49059	633.032208	581.389716
1336.08792	1438.02576	1605.6538	1626.72372	1570.02016
135.523802	123.70114	201.481655	225.412062	217.016438
12524.9036	13287.9323	12040.8537	12096.1748	12133.273
2108.7209	2191.27734	2532.46941	2417.54437	2438.08591
681.301724	742.206842	356.467543	350.327913	338.474197
179.716347	178.924864	125.53857	103.313862	93.7725349
5149.90449	4771.3297	1515.76199	1461.42154	1489.6437
1255.06826	1206.08612	1473.9158	1442.6372	1430.70096
142.889226	144.686155	49.5954842	49.7784971	53.5843057
511.160429	563.281978	430.860769	337.178876	340.260341
243.795536	223.103842	164.285042	170.937481	197.368859
502.32192	452.834531	345.618531	344.692612	338.474197
155.410447	170.089068	125.53857	138.064888	125.030047
84.7023765	70.6863659	32.5470365	39.4471109	28.5782964
902.264445	974.14648	889.618999	744.799022	784.117006
388.157847	350.118406	500.604419	530.657563	514.409334
643.738061	600.83411	362.666979	319.333755	346.511843
68.4984436	61.8505702	29.4473188	22.5412062	21.4337223
1259.48751	1231.48903	1393.32314	1367.49984	1444.09704
63.3426468	49.701351	131.738005	91.1040418	100.917109
139.206514	129.223513	48.0456254	54.4745817	50.0120186
374.900084	388.775012	471.1571	462.094728	466.183459
444.13507	488.177714	305.3222	365.355384	256.311595
639.318807	590.89384	731.533393	765.461795	715.350481
1923.11222	1973.69587	2653.35841	2765.05463	2778.34625

484.644902	480.446393	598.245529	630.214557	601.930367
27.9886114	13.2536936	7.74929441	2.81765078	5.35843057
663.624706	643.908614	451.008935	440.492738	459.038885
129.631463	189.969608	130.188146	94.8609095	101.810181
36.0905778	22.0894893	18.5983066	12.20982	16.0752917
235.693569	240.775434	85.2422386	96.7393434	97.344822
456.656291	436.267414	210.780808	222.594411	237.557088
92.804343	90.5669063	30.9971777	32.8725924	20.5406505
310.820895	282.745464	175.134054	182.208084	192.010429
450.027409	461.670327	189.082784	185.964951	185.758926
160.566244	164.566696	119.339134	114.584465	139.319195
1520.96007	1641.24906	1872.22953	1791.08668	1696.83635
20093.6133	19829.7346	29185.3926	27278.6164	27062.7536
759.375219	787.490295	508.353714	452.702558	548.346061
12839.4072	13860.0501	11606.8932	11515.7387	11700.1331
9879.24327	9677.40528	11752.5799	11551.429	11480.4375
6235.568	6481.05617	7524.56488	7910.08495	7624.15362
214.33384	251.820179	114.689557	129.611936	129.495405
1715.40726	1614.74167	1560.7079	1454.84702	1479.81991
2939.54074	3049.454	3429.83771	3464.77124	3346.33989
1338.29755	1347.45885	965.562084	1128.93875	1166.35172
1915.74679	1857.72605	2642.5094	2285.11478	2438.08591
50.0848835	68.477417	15.4985888	20.6627724	20.5406505
1613.02787	1601.48798	1311.18062	1325.23508	1265.48269
1830.30788	2069.78515	2256.59453	2150.80676	2177.30895
1306.62623	1309.90672	1611.85324	1576.94522	1533.40421
2298.0123	2398.91854	1680.04703	1823.95927	1687.90563
777.052237	855.967712	1022.90686	1056.61904	1003.81266
1943.73541	2203.42656	2723.10206	2606.32697	2653.3162
1339.77063	1313.22014	1684.69661	1520.5922	1701.30171
917.731836	967.519633	723.784098	746.677456	727.853485
12738.5009	13162.0222	14737.6081	14536.2604	14492.7685
213.597297	182.238287	88.3419563	73.2589202	65.1942386
2867.35958	2802.05172	2565.01645	2462.62678	2447.01663
14403.8233	14212.3774	18454.1697	17331.3699	16976.4011
1378.80738	1429.18996	1184.09219	1332.74882	1220.8291
66.2888164	58.5371468	142.587017	102.374645	88.4141043
153.20082	122.596666	195.282219	222.594411	163.432132
1790.53459	1938.35269	1568.45719	1559.1001	1572.69937
6719.47636	6526.33963	5858.46658	5716.07421	5623.67288
12611.0791	12621.9342	13387.681	13302.1293	13647.0296
759.375219	852.654288	880.319845	911.040418	910.933196
9430.68895	9215.73495	10215.1199	11418.0602	11225.912

2673.64893	2840.70833	3453.08559	3199.91207	3098.06594
1554.84102	1560.62242	1831.9332	1607.93938	1783.46431
2969.73897	2916.91707	3070.27045	3079.6923	3217.73756
221.699264	252.924653	178.233772	156.849227	166.111348
1861.24266	1893.06924	2189.9506	2108.542	2086.21563
6304.80298	6201.62413	7189.79536	7538.15505	7450.8977
5317.09962	5568.76026	6128.14202	6252.36708	6295.26284
1506.96576	1346.35438	1112.79868	1316.78213	1371.75823
6300.38373	6568.30966	7476.51925	7256.38997	7019.54404
5154.32375	5207.59711	4683.67354	4586.19625	4284.06524
3971.43664	4015.86916	7710.54794	7146.50159	7041.87084
3325.48896	3633.721	4294.65896	4357.02732	4292.10288
4894.32428	5245.14924	6360.62085	6411.09474	5986.26001
10803.604	10179.9412	9291.404	9157.36503	9350.46134
1492.97145	1566.14479	2185.30103	2072.85176	1993.33617
44.9290867	48.5968765	10.8490122	17.8451216	16.9683635
3826.33779	3769.57136	2510.77139	2385.61099	2449.69584
1171.10242	1159.69819	889.618999	792.699086	778.758576
5066.6752	5078.3736	6044.44964	5620.27409	5803.1803
498.639208	485.968765	604.444964	642.424377	652.835457
1984.98178	1809.12918	2352.68578	2134.84007	2179.0951
697.505657	689.192067	567.248351	517.508526	562.63521
6384.34956	6148.60936	7281.23703	7294.89786	7344.62216
1361.86691	1257.99642	781.128877	719.440165	781.437791
8084.28943	8306.75247	10687.8269	10505.1413	9721.08612
480.96219	449.521108	424.661334	361.598517	371.517853
2623.56404	2534.7689	2882.73752	2950.08036	2983.75275
4638.00752	4965.7172	5881.71446	5888.89013	5589.73615
107.535191	128.119038	187.532925	226.351279	206.299577
5426.84444	5565.44684	6112.64343	6281.4828	6299.7282
97.9601398	119.283242	145.686735	152.153142	160.752917
6839.53277	6899.652	8347.53994	8096.0499	8226.08399
10962.6972	11271.1619	12567.8057	11791.8685	12096.657
18245.6284	17717.9794	15016.5827	14562.5584	14835.7081
59668.0367	52496.7759	30834.4425	34826.1636	49502.9677
403.625238	360.058676	282.074317	308.063152	292.034466
610.593653	548.92381	767.180147	747.616673	729.639629
5339.93243	5585.32738	4694.52256	4457.52353	4586.81657
4347.07327	4636.58381	3784.75539	4030.17983	3783.94505
4894.32428	5383.20855	3746.00892	3655.43228	3591.04155
733.596235	717.908404	970.211661	878.167826	892.178689
838.185256	882.475099	1072.50235	1013.41506	947.549139
286.514995	260.655974	120.888993	115.523682	106.27554

1721.2996	1760.5323	1979.16979	1930.09078	1936.17958
4787.52563	5228.58213	5501.99903	5519.77787	5513.82505
2981.52365	3190.82674	2599.11335	2344.28545	2588.12196
365.325033	393.19291	299.122764	288.339596	258.990811
3504.46876	3857.92931	4093.17731	3900.56789	3837.52936
833.766002	896.833267	1018.25729	1043.47001	994.881942
4595.28806	4404.64417	5455.50327	5206.07942	5508.46662
1360.39382	1424.77206	1644.40027	1638.93354	1652.18276
941.301193	1054.77312	1134.4967	1124.24266	1145.81107
405.834865	430.745042	165.8349	194.417904	187.54507
645.947689	673.729425	1168.5936	1098.8838	1071.68611
2510.87306	2783.27566	2228.69707	2317.04816	2165.69902
5795.85218	5845.98335	5153.28079	5048.29098	4991.37807
7834.60156	7722.48547	9234.05922	8853.05874	8698.51895
1322.83016	1404.89152	1703.29491	1687.77282	1662.89962
483.171817	503.640357	587.396517	637.728293	597.465008
7895.73458	8178.63343	9015.52912	8868.08622	9350.46134
4106.2239	4073.30183	4641.82735	4796.58084	4538.59069
6388.76882	6589.29467	7388.17729	7214.12521	7299.0755
827.13712	815.102157	946.963777	945.791444	899.323263
2270.02369	2496.1123	3237.65521	3172.67478	3190.9454
2969.73897	2857.27545	3327.54702	3300.40828	3514.23738
64.0791892	69.5818914	38.7464721	47.9000632	44.6535881
1020.11123	1089.01182	1281.7333	1384.40575	1277.09262
3042.65667	3050.55848	1879.97883	1845.56126	1669.15112
914.785666	848.236391	1337.52822	1310.20761	1226.18753
843.341053	763.191857	526.95202	611.430219	608.181869
616.485993	658.266782	745.482123	749.495107	730.532701
3207.64217	3180.88647	3952.14015	3945.65031	3932.19496
2074.10341	2066.47173	2388.33254	2488.92485	2473.80878
6332.79159	6252.42996	7264.18858	7064.78972	7264.2457
7334.48926	8027.32043	8815.59732	8577.86819	8370.76162
17756.5643	18344.2164	19342.2389	19586.4298	19756.5335
6718.00327	6550.63806	7346.3311	7275.17431	7424.99862
10800.6578	11134.2071	12666.9967	13299.3117	13444.3023
4004.58105	4029.12286	6682.9915	6921.08953	7324.97458
4807.41227	4673.03147	3690.214	3773.77361	3911.65431
5739.13842	5421.86516	4939.40026	4687.63168	4700.23668
4345.60019	4352.73387	5120.73375	4867.02211	4877.06489
2495.40567	2654.05214	3250.05408	3085.3276	2930.16845
21079.8436	20595.1354	25788.102	24643.1737	23395.8009
3823.39162	3970.58571	4564.33441	4443.43528	4355.51098
1275.69144	1519.75687	1619.60253	1635.17667	1575.37859

1129.11951	988.504648	1304.98118	1252.91538	1248.51432
1052.5191	977.459903	1394.87299	1386.28418	1334.24921
1937.10652	2120.59098	2461.17591	2302.02069	2246.96855
416.883001	452.834531	513.00329	556.955637	565.314425
844.077595	1024.95231	571.897928	541.928166	492.08254
810.933187	836.087171	368.866414	364.416167	349.191059
1808.94815	1880.92002	1680.04703	1647.38649	1633.42825
4333.07897	4543.80796	4934.75068	4938.4026	5047.64159
2852.62873	3168.73725	3312.04843	3293.83376	3211.48605
4458.29117	4475.33054	5382.6599	4991.93796	5328.06613
1657.22041	1752.80098	2436.37816	2306.71677	2185.3466
3533.19391	3418.34848	4372.15191	4323.21551	4303.71282
3800.55881	4034.64523	4379.9012	4307.24882	4240.30472
1959.2028	1944.97954	1763.73941	1741.30818	1761.13751
308.611267	283.849938	477.356536	452.702558	467.969603
410.990662	428.536093	491.305266	449.884908	540.308415
6415.28434	6272.3105	7059.60721	7083.57406	7231.20205
1366.28616	1320.95146	2005.51739	2149.86754	2121.04543
615.74945	692.505491	914.416741	894.134514	856.455819
1617.44712	1587.12981	2067.51175	2049.37133	2082.64335
357.959609	311.4618	542.450609	517.508526	485.831038
187.081771	185.55171	145.686735	123.976634	147.356841
1548.94868	1452.38392	1912.52586	1883.12994	1877.23684
1113.65212	1020.53441	886.519281	834.02463	851.99046
3696.70633	3588.43754	4593.78173	4403.04895	4185.82734
39.0367474	41.9700297	9.2991533	15.0274708	16.9683635
7406.67042	7824.09712	9086.82263	8826.76067	8780.68156
95.0139702	85.044534	24.7977421	35.6902432	40.1882293
5132.96402	5214.22396	5920.46093	5975.29808	5708.5147
11090.8555	10799.5513	9919.09685	9845.81104	9694.29397
1833.25404	1835.63656	1016.70743	952.365963	936.832277
3632.62714	3801.60112	4073.02914	4012.33471	3971.49012
10506.7774	10270.5081	7499.76713	7831.19073	7920.65345
1405.32291	1571.66717	1106.59924	1143.027	1099.37134
1334.61484	1190.62348	1390.22342	1374.07436	1419.09103
1058.41144	1100.05657	1335.97836	1350.59394	1327.10464
3572.9672	3925.30226	2637.85982	2649.53095	2671.17764
885.32397	869.221405	1036.85559	1076.3426	1031.49788
2123.45175	2137.15809	2470.47506	2495.49937	2534.53766
1030.42282	967.519633	1092.65051	1143.027	1211.89838
1065.77686	1031.57915	1211.98965	1271.69972	1244.94204
1728.66502	1718.56227	2261.24411	2145.17146	2219.28333
229.064688	215.372521	142.587017	168.11983	149.142984

323.342116	313.670749	85.2422386	76.076571	62.5150233
496.429581	536.774591	330.119942	342.814178	342.046485
474.333308	517.998525	392.114297	396.349543	384.913929
1990.87412	1986.94957	1466.1665	1517.77455	1385.1543
480.225648	480.446393	120.888993	90.1648249	101.810181
915.522209	952.056991	723.784098	711.92643	745.714921
369.744287	394.297385	167.384759	184.086518	178.614352
49.3483411	54.1192489	38.7464721	30.0549416	28.5782964
183.399059	152.417476	111.58984	129.611936	125.923118
199.602992	201.014353	883.419563	880.04626	891.285618
18133.674	18199.5303	24029.0121	23348.9328	21826.6738
1888.49473	1856.62158	1442.91862	1526.22751	1502.1467
798.411966	791.908193	1063.20319	1074.46416	1027.9256
2974.15823	3062.7077	3694.86358	3600.95769	3599.0792
25717.1146	26385.895	18558.0103	18602.1304	18341.9078
1718.35343	1709.72648	2172.90215	2197.76761	2069.24727
4951.77459	5028.67225	5627.5376	5684.14084	5429.87631
478.752563	506.95378	389.01458	392.592675	412.599154
485.381445	461.670327	641.641577	680.932271	620.684874
212.124213	235.253061	426.211193	404.802495	403.668436
492.010326	551.132759	402.96331	370.990686	389.379288
130.368006	139.163783	26.347601	22.5412062	23.2198658
195.183737	159.044323	133.287864	132.429587	117.885473
778.525321	756.56501	482.006113	497.784971	479.579536
742.434744	804.057412	610.6444	600.159616	584.962004
69.9715284	85.044534	44.9459076	39.4471109	55.3704492
905.210615	925.549603	492.855125	463.973161	480.472607
1132.80222	1149.75792	1255.3857	1272.63894	1239.5836
521.472022	511.371678	440.159923	440.492738	403.668436
4052.45631	4093.18237	4417.09782	4519.51185	4485.89946
3835.1763	4155.03294	5007.59405	4845.42012	4674.3376
21130.665	20454.8671	23495.8607	23796	23833.4061
24887.0313	23874.3201	21718.1725	21081.6631	21111.3234
92.0678006	89.4624318	52.695202	57.2922325	44.6535881
1515.06773	1464.53314	1560.7079	1656.77866	1712.91164
1775.80374	1727.39807	2191.50046	2182.74014	2126.40386
1016.42852	1028.26573	1084.90122	1147.72308	1296.7402
394.050186	356.745253	447.909217	466.790812	442.963594
346.17493	333.551289	292.923329	266.737607	273.279959
2757.61476	2715.90271	3566.22529	3517.36739	3436.54014
2868.09612	2847.33518	3189.60958	3202.72972	3176.65625
2289.91034	2295.09794	1954.37205	1735.67288	1835.26247
675.409385	760.982908	1194.9412	1281.09189	1250.30047

8456.24335	8228.33478	9624.62366	9429.73794	9930.06491
3164.92271	3154.37908	3835.90074	3813.22072	3988.45849
4107.69699	4360.4652	3346.14533	3247.81213	3261.49807
4389.79273	4371.50994	5026.19236	5018.23604	5044.96238
2426.90722	2296.20242	2585.16462	2781.96054	2953.38831
7196.75583	7479.50109	8297.94446	8205.93828	8245.73157
2152.91345	2154.82969	2451.87675	2276.66183	2439.87205
3430.81452	3337.72184	4014.13451	3788.80108	4032.219
2825.37666	3095.84193	3253.1538	3279.74551	3427.60942
2163.96158	2405.54539	2416.23	2524.6151	2501.494
3323.27933	3442.64691	4218.71588	4250.89581	4159.92826
11231.5351	10752.0589	9168.96515	9085.98454	9267.40566
2449.74004	2418.79908	2718.45248	2779.14288	2639.92013
3358.63336	3347.66211	3614.27091	3737.14415	3663.38036
420.565713	450.625583	309.971777	339.05731	377.769355
995.068788	1083.48945	519.202726	600.159616	634.974022
2264.86789	2347.00824	1788.53715	1776.05921	1879.02299
98.6966822	99.402702	71.2935086	69.5020525	40.1882293
418.356086	396.506334	187.532925	225.412062	175.935137
29.4616962	49.701351	12.3988711	22.5412062	13.3960764
82.4927493	72.8953148	34.0968954	36.6294601	42.8674445
388.89439	375.521319	260.376292	293.035681	274.173031
142928.263	137351.34	125949.282	121642.68	129011.361
7077.43596	7204.48695	8361.48867	8264.16973	8128.73917
1210.13917	1275.66801	1571.55691	1477.38822	1462.85155
1280.1107	1313.22014	1108.1491	1035.95627	1127.05656
6626.67201	6527.4441	8415.73373	8214.39123	8218.04635
1249.17592	1282.29486	976.411096	937.338492	902.89555
2177.21935	2212.26236	1168.5936	1096.06615	1093.11984
762.321389	732.266572	898.918152	900.709032	899.323263
1991.61066	1903.00951	1495.61382	1602.30408	1512.86356
718.865387	779.758974	220.079961	200.053205	180.400496
4339.70785	4092.0779	2865.68907	2882.45675	2944.4576
2563.16757	2675.03716	2114.00752	2172.40875	2293.40828
3030.13545	3169.84172	3437.587	3625.37733	3632.12285
433.823476	429.640568	509.903572	473.365331	568.886712
2239.82545	2243.18764	3192.7093	2909.69404	2963.2121
10373.4632	10569.8207	11067.5423	11052.7048	11100.882
216.543467	228.626215	296.023047	289.278813	305.430542
187.081771	172.298017	139.487299	104.253079	97.344822
3966.28085	4066.67499	4798.3631	4622.82571	4400.16457
405.834865	387.670538	314.621353	278.947427	286.676035
45.6656291	56.3281978	32.5470365	24.4196401	25.8990811

2417.33217	2678.35058	728.433675	793.638302	751.966423
9902.81263	10196.5083	10937.3541	10868.6183	10657.9184
11713.2339	11652.2056	10726.5733	10223.3762	9745.19906
54.5041379	54.1192489	88.3419563	81.7118726	103.596324
2622.09096	2675.03716	3101.26762	3113.50411	3102.5313
2060.10911	2085.24779	1988.46895	1921.63783	1785.25045
1280.84724	1142.0266	1501.81326	1382.52732	1563.76865
4482.59707	4243.3909	4091.62745	3900.56789	4003.64071
4375.06188	4116.37634	3339.94589	3574.65962	3684.81409
2379.03197	2453.03779	2732.40121	2638.26035	2725.65502
859.544986	868.116931	1822.63405	1706.55715	1862.94769
4985.65554	4944.73219	5356.3123	5352.59726	5475.42297
2714.8953	2694.9177	3121.41579	3004.55495	3065.91536
7029.56071	7666.15728	8941.13589	9015.54327	8977.15734
3016.87769	3089.21508	3894.79537	3898.68946	3688.38637
7976.75424	8299.02114	6529.55547	6381.97901	6155.94365
1000.96113	1022.74336	1176.34289	1133.63483	1192.2508
607.647484	617.401227	729.983534	742.920588	726.067342
582.605042	594.207263	409.162745	373.808337	378.662427
123.002582	130.327987	49.5954842	52.5961479	41.9743728
209.914585	234.148587	283.624176	293.974898	305.430542
2767.18981	2791.00698	2379.03339	2443.84244	2664.92614
1244.75666	1212.71297	1503.36312	1456.72545	1463.74462
2708.26642	2779.96223	2413.13028	2323.62268	2308.5905
5909.27971	5717.86432	6881.37344	6484.35366	6475.66334
4062.7679	4157.24189	5030.84193	4774.97885	4890.46096
710.76342	713.490506	950.063495	983.360122	908.253981
78.8100373	77.3132127	102.290686	126.794285	87.5210326
8981.39808	9124.06357	11980.4092	11290.3267	11375.9481
3612.74049	3545.36304	3000.5268	2941.62741	2812.28298
901.527903	912.29591	788.878171	732.589202	690.344471
785.154203	799.639514	702.086074	648.059679	607.288798
321.132488	340.178136	275.874881	283.643512	281.317605
1631.44143	1571.66717	1272.43414	1295.18014	1175.28244
5534.37963	5386.52198	6083.19611	6044.80014	5970.18472
2495.40567	2434.26173	4429.49669	4300.6743	4442.13894
7012.62023	7158.09902	9130.21868	8976.09616	8702.98431
5767.12703	5664.84954	6924.76949	6686.2853	6404.2176
1946.68158	1979.21825	2464.27562	2348.98153	2380.92932
2292.11996	2457.45569	2812.99387	2811.07626	2590.80118
1165.94663	1274.56354	1340.62793	1370.3175	1411.05338
1609.34515	1645.66696	2064.41203	2127.32634	2087.10871
70627.0512	69511.2051	87906.446	86058.5685	82563.5912

6704.74551	6873.14461	8249.89883	7683.73367	7604.50605
2791.49571	2771.12644	3294.99998	3333.28087	3432.96785
2676.5951	2605.45527	3096.61805	2851.46259	2906.94858
1316.20128	1333.10068	1633.55126	1606.06094	1523.58042
1235.18161	1198.3548	1749.79068	1684.95517	1651.28969
4379.48114	4111.95844	3890.1458	3743.71867	3695.53095
3082.42996	3134.49854	1072.50235	1121.42501	1070.79304
643.001519	589.789365	666.43932	693.142091	737.677275
3419.02984	3390.73661	4612.38004	4374.87244	4245.66315
13.2577633	17.6715915	29.4473188	35.6902432	27.6852246
466.231342	510.267204	588.946375	610.491002	609.074941
605.437856	578.744621	509.903572	473.365331	498.334043
3561.18253	3664.64628	4065.27985	4109.07405	4148.31833
1266.85294	1116.62369	946.963777	1029.38175	1094.90598
28992.5187	28225.9495	17271.6274	18949.6407	18759.8654
116.3737	117.074294	198.381937	195.357121	178.614352
9725.30591	9222.3618	6754.28501	6513.46938	6779.30774
1124.70025	1229.28008	866.371115	926.067889	905.574766
2730.36269	2578.94788	2986.57807	2880.57831	2838.18206
4492.90867	4448.82315	3679.36499	3635.70872	3564.2494
457.392833	526.834321	333.21966	326.84749	314.36126
821.244781	800.743989	1047.70461	1000.26603	1009.17109
432.350391	424.118195	353.367825	309.941586	351.870274
232.7474	216.476996	23.2478832	31.9333755	24.1129376
5402.53854	5229.6866	6588.45011	6517.22625	6192.55959
687.194063	701.341287	971.761519	959.879698	966.303646
13017.6505	12700.3519	12012.9562	11669.7703	11763.5412
1887.75818	1883.12897	2172.90215	2189.31465	2237.14476
3536.87663	3729.81028	4113.32548	3991.67194	3962.5594
1184.36019	1197.25032	1411.92144	1348.71551	1341.39379
1788.32496	1980.32272	2296.89086	2283.23635	2132.65537
8.10196645	7.73132127	57.3447787	30.9941586	43.7605163
3703.33521	3738.64607	3567.77515	3495.7654	3399.03112
1826.62516	1817.96497	2265.89369	2256.93827	2145.15837
4180.61469	4295.3012	3893.24551	3681.73035	3853.60465
445.608155	463.879276	314.621353	306.184718	298.285968
15273.6798	15648.1943	16684.2309	17055.2402	16493.2493
4575.40142	4451.0321	4970.39744	4986.30266	4832.4113
2196.36945	2175.8147	2896.68625	2854.28024	2875.69107
592.916636	628.445972	925.265753	912.918852	874.317254
2233.93311	2379.038	2845.54091	2774.4468	2737.26495
3187.75553	3158.79698	2718.45248	2761.29776	2762.27096
2194.15982	2136.05362	2645.60911	2468.26208	2487.20486

1663.84929	1724.08464	1972.97036	1916.00253	1910.2805
567.874194	572.117774	672.638755	696.898959	741.249562
1870.81771	1842.26341	1597.90451	1529.04516	1528.04578
11680.826	11391.5497	11839.372	12601.4735	13223.7136
1202.77375	1280.08591	1315.83019	1439.81955	1380.68894
374.900084	374.416844	296.023047	302.42785	324.185049
1999.71263	1910.74083	2267.44355	2243.78924	2154.08909
2154.38653	2185.75497	2411.58042	2435.38949	2386.28775
7333.01618	6931.68175	8700.90777	8631.40355	8869.09566
1277.16453	1274.56354	1103.49952	1036.89549	1048.46625
667.307418	683.669695	1072.50235	1187.17019	1117.23277
1642.48956	1491.04053	1853.63122	1843.68283	1856.69619
109.008276	131.432462	60.4444964	89.225608	81.2695303
5415.05976	5403.08909	4551.93554	4522.3295	4416.23986
3899.99203	3796.07874	4891.35463	4659.45517	4766.32399
451.500494	524.625372	576.547504	552.259552	594.785793
402.152153	367.789998	534.701315	585.132145	628.72252
48807.719	47078.2242	50559.4965	55648.6029	57911.2384
668.043961	672.62495	509.903572	478.061415	500.120186
4555.51477	4618.91222	5055.63968	4988.18109	5103.01204
2804.75348	2785.48461	3380.24222	3463.83202	3446.36393
4479.6509	4348.31598	5526.79678	5275.58147	5524.54191
3151.66495	3444.85586	3040.82313	3075.93543	3006.97262
1321.35707	1330.89173	1174.79303	1164.62899	1251.19354
21849.5304	21307.5214	23361.0229	23398.7113	23547.6231
3163.44963	3258.19968	3656.1171	3859.24235	3745.54297
916.995293	885.788523	937.664624	1078.22103	1051.14546
3011.72189	3188.61779	2822.29303	2851.46259	2570.26053
13513.3435	13850.1098	16433.1537	15764.7561	14663.3452
11047.3995	12312.6814	14813.5512	14586.9781	13233.5374
980.33794	1028.26573	500.604419	507.17714	540.308415
525.154734	586.475942	681.937908	589.82823	619.791802
2549.17326	2648.52977	2958.68061	2855.21946	2719.40351
27993.7672	27766.4881	23836.8296	24105.0024	23797.6832
1531.27166	1588.23428	1153.09501	1147.72308	1092.22676
222.435806	227.52174	136.387582	108.949163	103.596324
416.883001	460.565853	323.920507	366.294601	361.694063
2468.1536	2515.99284	1827.28362	1701.86107	1716.48393
2021.07236	2028.9196	1449.11806	1483.96274	1465.53076
7702.02392	7969.88775	7137.10016	7244.18015	7129.39187
1298.52426	1305.48882	1701.74505	1812.68867	1978.15395
3227.52882	3292.43839	2380.58324	2378.09726	2302.339
164.985499	174.506966	102.290686	118.341333	102.703253

1263.17022	1388.32441	1619.60253	1624.84528	1557.51715
23992.1323	23412.6498	16941.5075	17522.031	18058.8041
2029.91087	2026.71065	1594.80479	1541.25498	1662.89962
4966.50543	4903.86663	4417.09782	4168.24472	4055.43887
23880.1778	24613.2135	27866.4627	27159.3359	27681.6523
147.308481	152.417476	119.339134	98.6177772	107.168611
1841.35601	1959.3377	1117.44825	1050.04452	1027.9256
519.998938	514.685102	605.994823	695.020525	679.62761
1218.24114	1354.0857	1638.20084	1687.77282	1621.81832
374.163541	365.581049	517.652867	463.033945	492.08254
1009.06309	913.400384	1163.94402	1143.96622	1194.93002
11287.5124	11484.3255	14805.8019	14155.8775	14380.2415
1352.29185	1281.19038	680.38805	716.622515	727.853485
14308.0728	14140.5866	12564.706	11945.9001	11706.3846
4196.81862	4275.42066	3665.41626	3569.02432	3868.78687
253.370587	256.238076	148.786453	179.390433	183.972783
321.869031	300.417055	395.214015	413.255447	414.385297
13936.8554	13709.8416	16062.7375	15493.3224	14959.8451
95.0139702	93.8803297	23.2478832	27.2372909	37.509014
5246.39155	5238.5224	3632.86922	3401.84371	3487.44523
9181.73761	9253.28708	11265.9242	11177.6206	10269.4322
1912.80062	1767.15915	1647.49999	1656.77866	1702.19478
1191.72561	1102.26552	985.710249	955.183614	1017.20874
1403.11328	1366.23492	1608.75352	1608.87859	1589.66774
760.111761	612.983329	514.553149	480.879066	505.478617
1020.11123	996.235969	1272.43414	1323.35665	1331.57
2599.99469	2635.27608	3287.25069	3098.47664	3102.5313
1100.39435	1091.22077	799.727183	726.953901	777.865504
627.534129	620.71465	215.430385	193.478687	242.915519
174.56055	195.491981	111.58984	122.0982	137.533051
103.852479	99.402702	57.3447787	46.0216294	56.263521
230.537773	265.073872	198.381937	179.390433	147.356841
1570.30841	1557.309	1793.18673	1741.30818	1753.09987
33711.5459	34538.0211	39101.3898	38047.6777	38651.2528
7059.75895	6940.51755	7798.8899	7523.12758	7370.52124
5384.12498	5163.41813	6137.44118	5924.58037	5788.89116
7694.6585	7773.2913	8758.25255	8218.1481	7984.06154
12102.8648	12862.7096	15404.0474	15317.6889	13911.3788
2212.57338	2271.90398	2110.9078	2073.79097	1996.90846
3094.21464	3072.64797	3490.2822	3342.67304	3349.0191
2401.12824	2561.27629	3150.86311	2919.08621	2873.90493
79.5465797	49.701351	189.082784	209.445375	237.557088
104.589021	117.074294	172.034336	226.351279	221.481797

3726.16802	3776.1982	4272.96094	4439.67841	4271.56223
6024.18033	6058.04245	6648.89461	7035.67399	7112.42351
3283.50604	3465.84088	3759.95765	3584.05179	3662.48729
594.38972	542.296963	368.866414	394.471109	380.44857
1210.87571	1191.72795	1587.0555	1620.1492	1705.76706
579.658872	593.102789	1142.246	1081.03868	1079.72376
134.78726	138.059308	206.131231	182.208084	174.148993
984.757195	988.504648	790.42803	789.881435	859.135034
589.233924	636.177293	550.199903	489.332018	478.686464
2240.56199	2376.82905	2644.05925	2563.12299	2637.24091
844.814138	858.176661	694.336779	733.528419	739.463418
3245.20583	3191.93121	2967.97976	2896.545	3032.8717
3824.12816	3905.42172	4544.18624	4257.47033	4126.88461
3628.94443	3575.18385	5374.91061	5183.53821	5279.84025
5614.66275	5475.98441	5973.15613	5915.1882	6013.05217
2270.76023	2280.73977	1900.12699	1925.3947	1960.29252
5652.22641	5804.01332	6938.71822	6794.29524	6656.95691
2286.96417	2161.45653	2758.74881	2541.521	2605.9834
573.766533	508.058255	674.188614	637.728293	674.26918
1523.16969	1487.72711	1853.63122	1728.15914	1871.87841
1213.82188	1196.14585	1032.20602	913.858069	972.555148
176.033635	189.969608	234.028691	260.163089	237.557088
2030.64741	1990.26299	2197.6999	2239.09315	2158.55445
2468.1536	2622.02238	2995.87722	3135.1061	3111.46202
1796.42692	1726.29359	1579.3062	1501.80787	1556.62408
2038.01283	1968.1735	1768.38899	1557.22166	1677.18877
2522.65774	2589.99263	2966.4299	2982.95296	2901.59015
522.945107	515.789576	660.239884	697.838176	712.671265
1880.39276	1953.81533	1619.60253	1442.6372	1500.36056
4772.05824	4689.59859	4065.27985	3903.38554	4086.69638
1680.78977	1619.15957	1205.79021	1272.63894	1203.86073
1842.8291	1950.50191	2104.70836	2153.62441	2131.76229
3240.78658	3035.09584	3398.84053	3537.09094	3750.00833
279.886114	320.297595	460.308088	421.7084	421.529871
413.936831	424.118195	330.119942	373.808337	363.480207
151.727735	145.79063	88.3419563	82.6510895	116.099329
3391.77777	3441.54244	3670.06583	3736.20493	3806.27185
863.96424	928.863027	711.385227	704.412694	677.841467
1096.71164	1054.77312	1241.43697	1282.97032	1286.02334
3841.06864	3705.51184	3022.22482	3029.9138	2997.14883
2786.33992	2783.27566	2348.03621	2306.71677	2269.29535
27085.6104	28546.2471	30205.1998	30154.4986	30201.9008
11674.9337	12282.8606	13448.1255	13419.5314	12711.0904

168498.806	167023.047	207877.922	205472.487	209556.61
2709.00296	2777.75328	2303.0903	2345.22466	2364.85402
34.617493	44.1789787	18.5983066	20.6627724	19.6475787
9013.80595	9471.97303	10112.8292	9645.75783	9366.53663
461.075545	441.789787	306.872059	313.698453	277.745318
24.3058993	11.0447447	7.74929441	0.93921693	0.89307176
27.252069	20.9850149	6.19943553	3.7568677	3.57228704
4425.88331	4320.70412	4829.36028	5014.47917	5070.86146
3529.5112	3401.78136	3786.30525	3993.55037	4098.30631
1449.51545	1433.60786	2062.86217	1962.02416	2091.57406
13619.4056	13603.812	17522.7045	16866.4576	16476.2809
774.842609	759.878433	460.308088	544.745817	596.571936
2920.39063	2984.29001	2103.1585	2024.95169	2004.9461
3247.41546	3152.17013	2871.88851	2829.8606	2794.42154
936.145396	953.161465	1233.68767	1245.40164	1264.58961
797.675424	747.729214	946.963777	845.295233	851.99046
1333.14175	1359.60807	1687.79632	1572.24913	1595.02617
158.356617	177.820389	117.789275	119.28055	100.917109
904.474073	863.699033	1134.4967	1089.49163	1052.03854
3139.88027	3246.05046	3561.57571	3493.88696	3505.30666
5574.15292	5506.90969	2391.43226	2401.57768	2411.29376
3069.90874	3017.42424	2459.62605	2513.34449	2533.64459
760.111761	735.579995	393.664156	419.829966	418.850656
3449.22808	3549.78094	3930.44213	3768.13831	3856.28386
237.903197	262.864923	319.27093	295.853332	347.404915
15252.3201	14556.9735	8742.75396	8983.6099	9229.00358
1555.57756	1618.05509	1875.32925	1732.85523	1731.66615
8308.93486	8342.09565	10438.2996	10368.9549	10343.5571
1543.79288	1620.26404	1379.37441	1364.68219	1341.39379
2733.30886	2459.66464	2360.43508	2378.09726	2308.5905
136.996887	141.372732	102.290686	112.706031	83.9487455
3681.97548	3797.18322	4562.78455	4553.32366	4177.7897
818.298611	812.893208	977.960955	887.559995	940.404564
1925.32185	1852.20368	1579.3062	1495.23335	1440.52475
326.288285	375.521319	432.410628	417.951532	423.316015
1567.36224	1661.1296	1796.28645	1757.27487	1738.81072
1865.66191	1819.06945	1669.19802	1596.66877	1526.25964
3624.52517	3545.36304	2307.73988	2515.22293	2672.07071
2298.74884	2244.29212	2520.07054	2440.08557	2491.67021
574.503075	517.998525	385.914862	416.073098	435.819019
3092.00501	3233.90124	3873.09735	3857.36392	3896.47209
559.772227	593.102789	429.310911	442.371172	409.026867
1383.96318	1354.0857	2289.14157	2334.89328	2269.29535

5179.36619	5304.79087	6250.58087	6103.9708	5846.04775
357.959609	328.028917	514.553149	434.857437	456.35967
1518.0139	1447.96603	1149.99529	1244.46243	1198.5023
916.995293	922.23618	633.892283	618.943954	594.785793
3558.23636	3681.2134	4197.01785	3853.60705	3878.61066
3391.04123	3431.60217	3794.05455	3682.66957	3624.97828
6219.36406	6348.51924	7535.41389	7428.26667	6979.35581
7822.81688	8636.99033	9350.29864	9468.24583	8822.65593
113.42753	112.656396	51.1453431	46.0216294	63.408095
155.410447	193.283032	83.6923797	64.8059679	91.0933196
1197.61795	1146.4445	1233.68767	1367.49984	1319.96006
2253.81976	2563.48524	2746.34994	2738.75656	2655.99542
8075.45092	7776.60472	8308.79347	8998.63737	9331.70683
153.20082	152.417476	192.182501	223.533628	211.658007
1403.11328	1337.51858	1157.74459	1271.69972	1246.72818
45.6656291	54.1192489	1.54985888	11.2706031	6.25150233
102.379394	82.835585	189.082784	196.296338	174.148993
89.8581733	72.8953148	23.2478832	36.6294601	35.7228704
631.953383	614.087804	525.402161	504.359489	517.981621
172.350923	172.298017	254.176857	227.290496	232.198658
2564.64065	2439.7841	2129.50611	2245.66767	2188.02582
108.271733	161.253272	43.3960487	57.2922325	74.1249562
9548.53573	9680.7187	10960.602	10819.779	11118.7434
1832.5175	1911.8453	2182.20131	2078.48706	2198.74268
993.595704	1040.41495	489.755407	478.061415	446.535881
812.406272	838.29612	1005.85842	1064.13278	1105.62284
212.860755	227.52174	128.638287	141.821756	133.960764
11076.1247	10807.2827	13600.0117	13114.2859	12831.6551
2570.53299	2650.73872	1525.06114	1560.03931	1484.28527
268.101435	304.834953	189.082784	164.362962	200.941146
11764.7918	11338.5349	12405.0705	12881.3601	13040.6339
2339.25868	2457.45569	1931.12417	1937.60452	1922.7835
1135.74839	1250.2651	902.01787	812.422641	810.016087
140.679599	165.67117	100.740827	107.07073	108.954755
1325.03979	1303.27987	1064.75305	1096.06615	1024.35331
2715.63185	2681.66401	3129.16508	3085.3276	3015.01027
1260.9606	1135.39975	1043.05503	1070.7073	1077.93762
1317.67436	1257.99642	1549.85888	1597.60799	1586.98852
3846.22444	4029.12286	3732.06019	3615.04595	3651.77043
847.760307	849.340865	446.359358	426.404484	422.422943
2503.50763	2524.82863	3161.71212	3069.36091	3082.88372
15100.5924	15693.4777	13502.3706	13685.3298	13658.6395
536.939413	460.565853	333.21966	383.200506	311.682045

131.84109	142.477206	80.5926619	78.8942218	88.4141043
15403.3113	15450.4933	13832.4905	14054.4421	14437.3981
4674.0981	4845.32949	5532.99621	5562.04264	5573.66086
761.584846	749.938163	867.920974	900.709032	841.273599
12161.0516	12301.6366	12812.6834	12716.058	13070.9983
29801.9788	29498.3041	21908.8052	21985.1898	21837.3907
7992.22163	7995.29067	7696.59921	7343.73714	7231.20205
2220.67535	2077.51647	2628.56067	2531.18962	2630.98941
9651.65167	9320.66003	10799.4167	10756.8515	10923.1607
7310.91991	7387.82971	8866.74267	8814.55085	9098.6151
1164.47354	1124.35501	905.117588	973.967952	1008.27802
2977.84094	2901.45443	3935.0917	3970.06995	4219.76407
2214.04647	2297.30689	2555.7173	2607.26619	2488.991
1903.22557	1939.45716	2129.50611	2120.75182	2106.75628
1965.83168	2048.80014	2406.93085	2455.11304	2464.87806
125.212209	121.492191	75.9430853	66.6844017	44.6535881
1086.40005	1199.45927	1683.14675	1570.3707	1662.00655
5351.71711	5216.43291	3352.34476	3347.36912	3543.70875
1270.53565	1271.25011	1692.4459	1590.09426	1590.56081
718.865387	768.714229	981.060673	963.636566	861.814249
2000.44917	2110.65071	2349.58607	2353.67762	2263.04384
2255.29284	2287.36662	2853.2902	2882.45675	2722.9758
5121.17934	5171.14945	5621.33817	5584.58384	5320.02848
206.968416	195.491981	150.336312	139.004105	125.030047
295.353504	342.387085	244.877704	240.439533	238.45016
6852.79053	6840.01037	8293.29488	8035.94002	8163.56897
1524.64278	1565.04032	1690.89604	1738.49053	1735.23843
738.752032	760.982908	629.242706	593.585097	563.528281
914.785666	915.609333	728.433675	695.020525	757.324853
1522.43315	1434.71233	1151.54515	1099.82302	1181.53394
886.060513	879.161676	742.382405	715.683298	734.998059
1412.68833	1314.32462	1543.65945	1589.15504	1620.03218
3141.35336	3145.54328	3409.68954	3476.98106	3411.53413
5356.13637	5658.22269	6472.21069	6266.45533	6250.60926
1583.56617	1684.32356	2230.24693	2081.30471	2110.32857
3934.60952	3930.82463	3037.72341	3050.57658	2965.89132
1878.18313	1926.20347	1177.89275	1203.13688	1182.42701
7963.49648	7977.61907	9172.06487	8721.56837	8401.12606
262.209096	251.820179	148.786453	159.666877	133.067692
771.89644	765.400806	951.613354	906.344334	915.398555
3614.21358	4156.13742	3079.5696	3059.96875	2723.86887
1070.19611	1049.25074	960.912507	932.642408	883.247972
545.041379	505.849306	399.863592	349.388696	355.442561

7131.20356	6972.54731	8169.30617	8158.03822	8409.1637
478.752563	464.983751	621.493412	668.722451	652.835457
4375.06188	4866.3145	5224.57429	5056.74393	5049.42774
11.0481361	14.3581681	27.8974599	32.8725924	28.5782964
346.911473	365.581049	246.427562	258.284655	257.204667
1613.76441	1581.60744	1897.02727	1930.09078	1762.03059
109.744818	108.238498	60.4444964	44.1431955	54.4773774
12935.8943	13237.1265	11441.0583	11406.7896	11013.361
827.13712	818.41558	929.91533	928.88554	919.863914
390.367474	376.625793	99.1909685	102.374645	96.4517502
848.49685	913.400384	561.048916	528.779129	519.767765
361.642321	354.536304	430.860769	435.796654	375.983211
1721.2996	1728.50254	1628.90169	1484.90196	1460.17233
1193.1987	1059.19101	1599.45437	1495.23335	1477.14069
589.233924	616.296753	881.869704	926.067889	890.392546
7586.38677	7433.11316	8747.40353	8776.98217	8360.93783
6574.3775	6341.89239	7197.54465	6705.06963	7093.669
2962.37355	2931.27524	2645.60911	2478.59347	2453.26813
1945.94503	2334.85902	2433.27845	2290.75008	2361.28174
2644.92377	2676.14163	2093.85935	2164.89501	2071.92649
2514.55577	2735.78326	3053.222	3050.57658	2970.35668
341.755676	351.222881	263.47601	282.704295	268.8146
2764.24364	2889.30521	3085.76904	3223.39249	3259.71193
3710.70063	3852.40694	5261.77091	5117.79303	5202.14301
12356.2354	12042.0851	10881.5592	10500.4452	10316.765
215.070382	210.954623	142.587017	135.247237	108.954755
1818.5232	1862.14395	2100.05879	1998.65362	1993.33617
6097.09802	6604.75731	7033.25961	6960.53664	6840.03662
3561.18253	3289.12496	2967.97976	3064.66483	3255.24657
1881.86584	1986.94957	2222.49764	2319.86581	2218.39025
1431.83843	1434.71233	1633.55126	1481.14509	1626.28368
2756.87822	2904.76785	3037.72341	2881.51753	3045.37471
68.4984436	69.5818914	6.19943553	11.2706031	11.6099329
7102.47841	7787.64947	8518.02442	8347.76004	7791.15804
7488.42663	8163.17079	6047.54936	6128.39044	5847.83389
298.299674	314.775223	165.8349	180.32965	155.394486
920.678006	980.773327	1153.09501	1116.72893	1087.76141
1069.45957	1101.16104	943.86406	884.742344	910.040125
7335.22581	7127.17374	5946.80853	5990.32555	6172.91201
13355.7234	12945.5452	10239.9176	10804.7515	11096.4166
524.418192	618.505702	381.265285	383.200506	340.260341
7956.13105	7923.49983	4066.82971	3859.24235	3703.56859
1573.99112	1630.20431	1734.29209	1830.53379	1876.34377

1616.71058	1761.63678	1501.81326	1532.80202	1444.99011
5466.61773	5594.16318	6338.92283	6125.57279	6182.7358
8918.79198	9101.97408	10221.3193	9911.55622	9780.02886
2516.7654	2493.90335	2902.88569	2923.78229	2885.51486
1578.41037	1484.41368	1427.42003	1352.47237	1362.82751
2519.71157	2617.60449	3586.37345	3404.66136	3191.83847
1236.6547	1207.19059	1529.71072	1475.50979	1565.5548
15303.8781	15752.0149	20318.65	19452.1218	18421.3912
3828.54742	3485.72142	3888.59594	4034.87591	4092.94788
2188.26748	2192.38182	1858.2808	2008.98501	2133.54844
2513.08268	2451.93332	2701.40403	2659.86233	2555.97138
1183.62364	1185.1011	1382.47412	1428.54894	1451.24161
3780.67216	3847.98904	4355.10346	4176.69767	4151.89062
1892.91398	1942.77059	2301.54044	2180.8617	2046.92048
2524.13082	2593.30605	3181.86029	3058.09031	2905.16244
5208.82788	4972.34405	4350.45388	4574.92565	4778.82699
3472.79744	3651.39259	4785.96423	4664.15125	4692.19903
1368.49579	1320.95146	1706.39463	1641.75119	1662.89962
6281.97017	6676.54815	6864.32499	6807.44428	6681.06984
273.993774	275.014142	145.686735	182.208084	158.073702
510.423886	540.088014	361.11712	339.996527	350.08413
333.653709	342.387085	133.287864	158.727661	141.105338
394.786729	415.2824	282.074317	248.892485	279.531461
1427.41918	1633.51774	1790.08701	1906.61036	1812.0426
1431.83843	1546.26425	269.675446	281.765078	297.392896
45849.7647	44321.4559	49291.7119	51151.6322	52444.7461
13039.7467	12557.8747	13818.5418	14798.3019	15292.0678
849.233392	843.818493	683.487767	771.097096	734.104988
2988.88908	2782.17118	3418.9887	3337.97696	3270.42879
14953.2839	15009.808	16555.5926	16057.7918	16374.4707
966.343635	1048.14627	751.681558	797.39517	805.550729
12254.5925	12026.6225	15098.7252	15334.5948	15967.23
3166.3958	3138.91644	3862.24834	3926.86597	3861.6423
1129.85605	1169.63846	954.713072	968.332651	1016.31566
472.123681	463.879276	334.769519	374.747553	339.367269
13033.1179	13334.3202	14089.7671	14230.0756	14371.3108
1655.01078	1840.05446	1349.92709	1391.91948	1496.78827
605.437856	532.356693	688.137344	746.677456	728.746557
397.732898	414.177925	500.604419	525.961479	453.680455
57.4503075	72.8953148	26.347601	13.149037	24.1129376
5537.3258	5787.44621	6461.36168	6470.2654	6527.4615
12505.7535	13216.1415	15129.7224	14674.3253	14749.0801
3535.40354	3540.94514	3818.85229	3626.31655	3796.44806

13055.9507	13103.4851	14605.8701	14909.1295	15148.2832
2426.90722	2327.1277	2935.43272	2865.55084	2877.47721
1375.12467	1521.96582	1086.45108	1047.22687	1057.39697
2982.2602	3127.87169	4082.3283	3812.2815	3656.23579
8814.20295	8629.25901	11406.9614	11019.8322	10755.2632
4032.56966	4145.09267	4493.0409	4448.13136	4508.22625
1591.66814	1679.90566	1972.97036	1908.48879	1829.90404
1148.26961	1160.80267	1401.07243	1386.28418	1286.02334
2423.96105	2391.18722	2981.92849	3109.74724	2698.86286
6675.28381	6540.69779	6679.89178	7277.05274	7317.83001
7651.2025	7646.27673	9390.59497	9386.53396	9471.02603
12387.9067	12055.3388	14173.4595	14221.6227	14591.8995
6944.85833	6879.77145	7161.8979	7385.06269	7712.56773
18179.3396	18504.3652	16685.7807	16579.9964	15600.1775
156.883532	134.745885	40.296331	55.4137986	34.8297987
6522.81953	6510.87698	5519.04748	5215.47159	5252.15503
86.1754613	83.9400595	30.9971777	40.3863278	27.6852246
5410.6405	5416.34279	6173.08793	6079.55116	6211.3141
877.958546	833.878223	692.786921	708.169562	668.017677
574.503075	555.550657	672.638755	662.147933	670.696893
2566.11374	2638.5895	3234.55549	3382.12015	3397.24498
4910.52821	4848.64291	5796.47222	5646.57216	5570.98165
15527.787	16073.4169	16425.4044	17043.0303	16770.9946
3447.755	3673.48208	4113.32548	3889.29729	3887.54138
1579.14692	1681.01014	2090.75963	1977.05163	1932.60729
4229.22649	4227.92826	4541.08653	4536.41775	4514.47775
335.126794	321.40207	398.313733	403.863278	391.165431
356.486524	340.178136	252.626998	274.251342	264.349241
99.4332246	83.9400595	46.4957665	28.1765078	28.5782964
1459.82705	1516.44344	1715.69378	1659.59631	1730.77307
4358.12141	4435.56946	5774.7742	5244.58731	5161.95478
944.247362	1001.75834	1704.84477	1715.01011	1780.78509
6621.51622	6572.72755	5405.90778	5425.85618	5445.9516
662.888164	628.445972	584.296799	563.530156	502.799402
20209.987	20613.9115	23027.8033	21996.4604	21073.8144
3295.29072	3494.55721	4268.31136	4165.42707	4025.07443
844.814138	839.400595	895.818434	970.211085	935.939206
1300.73389	1523.07029	1819.53433	1850.25734	1870.98534
20254.1796	21313.0438	25039.5201	24550.1912	23574.4153
17618.0943	17957.6504	22610.8912	22047.1781	20858.5841
457.392833	419.700297	658.690025	661.208716	656.407744
46.4021715	58.5371468	3.09971777	5.63530156	4.46535881
1186.56981	1206.08612	633.892283	658.391065	668.017677

13399.916	13002.9779	7778.74173	7733.51217	7998.35069
168.668211	185.55171	75.9430853	70.4412694	97.344822
2705.32025	2786.58908	2050.4633	2059.70272	1988.87081
424.248425	415.2824	371.966132	333.422009	281.317605
2816.53815	2722.52956	3367.84335	3260.02195	3334.72996
24463.5194	24909.2127	26781.5615	26559.1762	26749.2854
24.3058993	22.0894893	3.09971777	14.0882539	8.03764585
115.637158	112.656396	72.8433675	52.5961479	65.1942386
234.220485	244.088857	195.282219	175.633565	182.186639
1968.77785	1963.7556	2134.15568	2089.75766	2156.7683
9832.8411	9244.45129	8330.49149	8300.79919	8603.85335
8766.3277	8343.20012	7594.30853	7752.29651	8060.86572
7277.03896	7140.42743	5918.91107	6080.49038	6482.80791
5948.31646	5554.40209	4722.42002	4941.22025	5000.30879
69.9715284	60.7460957	29.4473188	32.8725924	48.2258751
9410.06576	8962.8103	7736.89554	7669.64542	7951.01789
7795.56481	8019.5891	7051.85792	6905.12284	7285.67943
19941.1491	19466.3625	18399.9247	18393.6243	19073.3336
7860.38054	7208.90485	6428.81465	6552.91649	6536.39222
6778.39975	6609.17521	5898.76291	5828.78024	5844.2616
4382.42731	4499.62898	3959.88945	3855.48548	4048.29429
15022.5189	14291.8996	13189.2991	13292.7372	13113.8657
3143.56298	3229.48334	2727.75163	2631.68583	2782.81161
2367.24729	2218.8892	1941.97318	1919.7594	1858.48234
62228.9947	58940.2799	48896.4979	49588.7753	51306.9727
276.939944	323.611019	164.285042	159.666877	141.105338
6379.93031	6356.25056	5827.4694	5964.02748	6008.58681
6146.44637	6030.43059	7267.2883	6997.1661	6983.82117
737.278947	727.848674	1039.95531	1052.86217	968.982861
15766.4267	16823.3551	18161.2464	17901.4746	17627.4504
6273.13166	6187.26596	7182.04606	7110.81135	7102.59972
22.8328145	32.0297595	71.2935086	59.1706663	52.6912339
1090.08276	1102.26552	3023.77468	2920.02542	3050.73314
2603.6774	2414.38119	1726.5428	1895.33976	1869.1992
325.551743	340.178136	387.464721	409.49858	399.203077
628.270671	648.326512	427.761052	470.54768	467.969603
2674.38547	2874.94704	3211.30761	3229.02779	3176.65625
5590.35685	5756.52092	5804.22152	6409.2163	6431.90282
319.659404	384.357115	288.273752	304.306284	260.776954
18323.7019	17968.6951	21109.078	20680.6175	20632.6369
9056.5254	9004.78033	10081.832	9823.26983	9595.163
3324.75241	3428.28875	3808.00328	3912.77771	3519.59581
2551.38289	2618.70896	3378.69236	3286.32002	3233.81285

23757.9118	25113.5404	28289.5742	27787.672	26076.8024
58.9233924	68.477417	37.1966132	17.8451216	28.5782964
2846.73639	2967.72289	2264.34383	2244.72845	2145.15837
55.9772227	51.9102999	20.1481655	25.358857	19.6475787
1696.9937	1613.6372	1835.03292	1961.08494	2039.7759
4895.06082	4818.8221	6019.6519	5816.57042	5815.68331
8974.7692	8987.10874	10170.174	9571.55969	9595.163
2255.29284	2168.08338	2794.39557	2620.41522	2672.07071
3122.9398	3385.21424	3539.87769	3542.72625	3485.65908
6852.05399	7340.33731	7647.00373	7731.63373	7418.74712
62.6061044	72.8953148	24.7977421	42.2647617	31.2575116
7226.95407	7401.0834	8142.95857	7966.43797	8109.09159
1369.96887	1347.45885	970.211661	973.967952	972.555148
3580.33263	3711.03421	4169.12039	3906.2032	4154.56983
7467.0669	7722.48547	5139.33206	5341.32666	5405.76337
1882.60239	1872.08422	2067.51175	2086.94001	2082.64335
3458.06659	3434.91559	4297.75868	4283.7684	4255.48694
778.525321	779.758974	551.749762	513.751659	522.44698
637.10918	664.893629	513.00329	515.630092	529.591554
1920.16605	2038.85987	1526.611	1536.55889	1523.58042
1732.34774	1710.83095	1439.8189	1348.71551	1528.04578
4548.14935	4189.27165	4798.3631	4957.18694	5271.80261
11009.8359	11260.1172	12225.2869	12657.8265	12629.8208
569.347279	630.654921	485.10583	485.575151	489.403325
1479.71369	1466.74209	1349.92709	1236.00948	1246.72818
2320.84512	2335.9635	3096.61805	3143.55905	3178.4424
3254.04434	3388.52766	3604.97176	3630.07342	3661.59422
465.4948	439.580838	571.897928	593.585097	577.817429
7383.8376	7275.17331	8187.90448	8096.98912	8199.29184
146.571939	117.074294	77.4929441	77.9550049	81.2695303
5439.36566	5294.85059	6247.48116	6190.37876	6067.52955
1907.64483	1972.5914	2076.8109	2295.44617	2139.79994
349.1211	331.34234	244.877704	237.621882	212.551079
692.34986	679.251797	454.108653	548.502685	582.282788
1546.00251	1537.42846	1053.90404	993.691508	1110.98127
6187.69274	5948.69948	7481.16883	7494.95107	7604.50605
20014.8033	19700.5111	23413.7181	22941.3126	22415.2081
194.447195	199.909879	136.387582	149.335491	136.639979
492.010326	493.700087	678.838191	632.092991	602.823439
953.822414	1048.14627	1213.53951	1205.95453	1201.18152
1541.58325	1558.41347	1131.39698	1127.99953	1193.14387
391.104017	455.04348	624.59313	642.424377	611.754156
3528.77466	3475.78115	4020.33394	3870.51295	3668.73879

1530.53512	1591.54771	1382.47412	1333.68804	1300.31248
5425.37135	5746.58065	6090.94541	5994.08242	6195.23881
24571.7912	23406.0229	27373.6076	27794.2465	28683.6788
4128.32018	4378.13679	4665.07524	4511.0589	4601.10571
5775.96554	6135.35566	6696.94023	6814.0188	6928.45072
960.451295	1016.11651	736.182969	714.744081	718.922768
3701.12558	3799.39217	4586.03243	4646.30613	4660.04845
1434.04806	1446.86155	2171.3523	2089.75766	2003.15996
1413.42487	1367.33939	973.311378	914.797286	877.889541
45.6656291	60.7460957	72.8433675	86.4079572	91.9863914
81.7562069	80.6266361	116.239416	133.368804	141.99841
2069.68416	2033.33749	4655.77608	4409.62347	4306.39203
3931.66336	3711.03421	4454.29443	4257.47033	4127.77768
2694.27212	2689.39533	3327.54702	3255.32587	3292.75558
6711.37439	6715.20476	6382.31888	6157.50617	6296.15592
2129.34409	2085.24779	2337.1872	2267.26966	2361.28174
3567.81141	3757.42214	3246.95436	3238.41996	3376.70433
1234.44507	1237.0114	1024.45672	970.211085	992.202727
4721.97335	4676.34489	6789.93177	6438.33203	6272.93605
4478.91436	4511.7782	5137.7822	5191.99117	5069.96839
12819.5206	13274.6786	14269.5507	14304.2738	14197.1618
2870.30575	3114.618	3265.55267	3356.76129	3181.12161
6300.38373	6201.62413	4514.73893	4641.61005	4898.49861
4197.55516	4599.03168	5122.28361	4790.00632	4786.86464
12686.2064	12585.4866	12053.2525	11956.2315	11792.1195
1905.4352	1826.80077	1693.99576	1568.49227	1710.23242
3363.78916	3416.13953	3071.82031	3046.81971	3016.79641
2056.42639	2154.82969	1866.0301	1835.22987	1844.19319
610.593653	612.983329	849.322668	896.952164	863.600393
13010.285	13076.9777	14113.015	14540.0172	14386.493
2239.82545	2508.26151	1934.22389	1972.35554	2001.37382
167.195126	162.357747	251.077139	233.865015	233.984801
17246.8769	16913.922	13161.4016	12639.9814	12847.7304
422.77534	334.655764	134.837723	159.666877	192.010429
7206.33088	7651.79911	8299.49432	8218.1481	8050.14885
3543.50551	3732.01922	4626.32877	4665.09047	4522.5154
7106.16112	7407.71025	5407.45764	5489.72293	5391.47422
4518.68765	4275.42066	5777.87391	5473.75624	5235.18666
1436.99423	1489.93606	1250.73612	1249.15851	1185.9993
6757.04002	6635.6826	7374.22856	7164.34671	6961.49438
1246.96629	1263.51879	1459.96707	1606.06094	1522.68735
3911.77671	3910.94409	4860.35746	4754.31608	4911.00161
1605.66244	1668.86092	1783.88757	1846.50048	1874.55763

303.455471	280.536515	412.262463	476.182981	454.573526
6566.27554	6830.0701	5618.23845	5614.63878	5520.96963
53.7675955	59.6416212	18.5983066	27.2372909	19.6475787
146.571939	147.999579	68.1937908	70.4412694	65.1942386
453.710121	446.207685	181.333489	173.755131	214.337223
1533.48129	1595.96561	1811.78503	1805.17493	1807.57724
58.18685	54.1192489	120.888993	69.5020525	77.6972432
1588.72197	1748.38308	1982.26951	1872.79855	1951.3618
145.835396	160.148798	79.042803	81.7118726	61.6219515
270.311062	255.133602	189.082784	169.059047	165.218276
2860.7307	2803.1562	3567.77515	3445.04768	3474.04915
3516.98998	3608.31808	2422.42943	2532.12883	2623.84483
4965.03235	5171.14945	4077.67872	4073.38381	4115.27468
1552.63139	1604.8014	2673.50657	2541.521	2652.42313
707.080708	734.475521	613.744118	626.45769	624.257161
3715.85643	3978.31703	2944.73188	2850.52337	2963.2121
413.936831	386.566063	587.396517	592.64588	613.5403
324.078658	344.596034	210.780808	201.931639	176.828209
478.752563	449.521108	627.692848	670.600885	671.589964
2291.38342	2231.03842	2055.11288	1910.36723	1983.51238
1907.64483	2090.77017	2176.00187	2132.96164	2140.69301
287.98808	265.073872	406.063027	412.316231	441.17745
3765.20477	3514.43775	4124.17449	4084.65441	3940.23261
9633.23811	10092.6877	8955.08462	9126.37087	8877.13331
63.3426468	88.3579574	27.8974599	45.0824124	36.6159422
3271.72136	3125.66274	3343.04561	3400.90449	3644.62586
3566.33832	3568.557	2340.28691	2339.58936	2499.70786
7948.76563	8169.79763	9255.75725	9232.50238	8959.29591
1390.59206	1309.90672	1723.44308	1786.39059	1670.93727
1491.49837	1376.17519	1591.70507	1569.43148	1665.57883
4908.31858	4754.76258	5339.26385	5371.3816	5418.26637
410.254119	340.178136	221.62982	221.655195	242.022447
1724.24577	1699.78621	2050.4633	1978.93006	1912.06664
224.645433	185.55171	38.7464721	40.3863278	47.3328033
5239.02612	5510.22312	5740.6773	5573.31324	5762.99207
497.166123	502.535882	297.572906	327.786707	323.291978
1511.38501	1540.74188	1235.23753	1195.62315	1322.63928
67.0253588	72.8953148	38.7464721	45.0824124	33.0436552
2505.71726	2428.73935	2859.48964	2750.96638	2806.03147
2661.12771	2911.3947	3467.03432	3168.91791	3155.22253
1828.83479	1761.63678	2033.41485	2168.65188	2094.25328
4996.70367	5348.96984	5610.48916	5596.79366	5508.46662
2211.83684	2246.50107	2892.03668	3004.55495	2966.78439

3684.92165	3568.557	4108.6759	4081.83676	4034.89822
4243.95733	4629.95697	5695.73139	5420.22088	5596.88073
184.135601	234.148587	119.339134	137.125671	119.671616
56.7137651	45.2834531	34.0968954	32.8725924	33.0436552
27.252069	18.7760659	4.64957665	4.69608463	6.25150233
47.8752563	59.6416212	21.6980244	30.0549416	27.6852246
14.7308481	13.2536936	0	0	2.67921528
172.350923	195.491981	147.236594	140.882539	130.388477
579.658872	605.252008	480.456254	612.369436	487.617182
141.416142	191.074083	75.9430853	73.2589202	72.3388126
25.7789842	16.567117	9.2991533	1.87843385	4.46535881
99.4332246	90.5669063	58.8946375	47.9000632	50.0120186
863.96424	783.072397	406.063027	388.835807	399.203077
62.6061044	73.9997893	26.347601	29.1157247	20.5406505
101.642852	94.9848042	203.031514	208.506158	223.26794
35.3540354	35.3431829	17.0484477	16.9059047	13.3960764
33.8809506	28.7163361	6.19943553	4.69608463	8.93071761
266.62835	288.267836	72.8433675	62.927534	65.1942386
167.195126	171.193542	103.840545	148.396274	125.030047
5771.54628	5762.04329	6586.90025	6453.3595	6358.67094
665.834334	688.087593	457.20837	537.232082	551.918348
27.9886114	9.9402702	7.74929441	2.81765078	3.57228704
25.7789842	22.0894893	3.09971777	6.57451848	5.35843057
538.412498	506.95378	443.25964	418.890749	421.529871
84.7023765	65.1639936	137.937441	141.821756	191.117357
316.713234	335.760238	416.912039	463.033945	435.819019
586.287754	522.416423	438.610064	432.979003	469.755746
724.021184	720.117352	592.046093	534.414431	559.062922
4829.50855	4712.79255	5664.73422	5409.88949	5301.27397
174.56055	139.163783	61.9943553	62.927534	55.3704492
1266.85294	1308.80224	1672.29773	1643.62962	1444.99011
198.129907	181.133813	130.188146	139.004105	137.533051
114.164073	93.8803297	79.042803	74.1981372	87.5210326
109.744818	111.551921	34.0968954	59.1706663	74.1249562
638.582265	622.923599	410.712604	410.437797	385.807001
74.3907828	77.3132127	51.1453431	51.6569309	38.4020857
1629.2318	1562.83137	1035.30573	1124.24266	1099.37134
264.418723	251.820179	103.840545	111.766814	108.954755
1518.0139	1576.08506	855.522103	768.279445	826.984451
167.195126	189.969608	102.290686	97.6785603	125.923118
36.0905778	38.6566063	77.4929441	79.8334387	65.1942386
595.862805	590.89384	477.356536	486.514368	473.328033
1896.59669	1740.65176	2281.39228	2196.82839	2177.30895

33.1444082	28.7163361	6.19943553	4.69608463	2.67921528
61.1330196	99.402702	29.4473188	41.3255447	41.081301
282.095741	291.581259	210.780808	219.776761	195.582716
396.996356	450.625583	277.42474	296.792549	269.707672
120.792954	153.521951	63.5442142	86.4079572	67.8734538
44.1925443	45.2834531	27.8974599	21.6019893	29.4713681
1641.75302	1706.41305	2000.86782	1981.74771	1968.33016
167.931668	141.372732	100.740827	87.3471741	122.350831
21.3597297	29.8208106	1.54985888	5.63530156	14.2891482
48.6117987	50.8058255	35.6467543	16.9059047	29.4713681
1090.8193	1218.23534	1244.53668	1228.49574	1342.28686
286.514995	329.133391	255.726716	254.527787	224.161012
378.582796	410.864502	257.276575	273.312125	254.525452
413.936831	420.804772	337.869236	267.676824	259.883883
159.093159	140.268257	99.1909685	101.435428	98.2378937
191.501025	174.506966	241.777986	242.317967	256.311595
231.274315	224.208317	151.886171	158.727661	137.533051
366.061575	387.670538	244.877704	264.859173	255.418524
504.531547	552.237233	303.772341	324.029839	313.468188
32.4078658	39.7610808	17.0484477	12.20982	6.25150233
123.739124	114.865345	80.5926619	66.6844017	98.2378937
246.005163	233.044113	137.937441	139.943322	133.960764
1612.29132	1625.78642	1786.98729	1792.0259	1762.03059
266.62835	276.118617	195.282219	152.153142	147.356841
253.370587	291.581259	454.108653	401.984844	369.731709
921.414548	964.20621	1131.39698	1075.40338	1027.03253
846.287223	921.131705	731.533393	769.218662	823.412164
601.755144	544.505912	485.10583	492.149669	501.90633
28367.9307	28165.2034	29978.9204	29166.4424	30207.2593
82.4927493	58.5371468	15.4985888	25.358857	21.4337223
19.8866449	15.4626425	3.09971777	1.87843385	5.35843057
863.227698	780.863448	691.237062	633.032208	668.017677
702.661454	673.729425	993.459544	933.581624	905.574766
15.4673905	15.4626425	3.09971777	1.87843385	4.46535881
44.1925443	39.7610808	15.4985888	18.7843385	19.6475787
595.862805	565.490927	488.205548	436.735871	464.397316
164.248956	186.656185	337.869236	288.339596	352.763346
5513.0199	5562.13342	6193.2361	6389.49275	6172.91201
58.18685	51.9102999	40.296331	28.1765078	23.2198658
351.330727	334.655764	452.558794	483.696717	419.743728
645.947689	661.580206	514.553149	540.988949	486.72411
53.7675955	55.2237233	46.4957665	25.358857	31.2575116
30.1982386	41.9700297	20.1481655	13.149037	9.82378937

256.316757	282.745464	409.162745	309.941586	368.838637
117.846785	102.716125	65.0940731	65.7451848	58.9427362
799.148509	810.684259	595.145811	520.326177	533.163841
479.489105	514.685102	297.572906	286.461162	325.971193
4065.71407	4024.70496	4798.3631	4488.51769	4510.90547
235.693569	257.342551	161.185324	159.666877	187.54507
146.571939	171.193542	113.139698	80.7726556	107.168611
301.982386	299.312581	218.530103	186.904168	172.36285
37.5636626	43.0745042	15.4985888	23.4804232	22.326794
8.83850885	18.7760659	1.54985888	4.69608463	5.35843057
227.591603	215.372521	314.621353	368.173035	315.254332
1095.23856	1071.34023	957.81279	846.23445	779.651647
245.268621	262.864923	170.484477	182.208084	180.400496
694.559487	660.475731	415.362181	464.912378	525.126196
15.4673905	34.2387085	9.2991533	5.63530156	8.93071761
262.945638	245.193332	176.683913	200.053205	187.54507
494.956496	470.506123	340.968954	294.914115	328.650408
98.6966822	82.835585	23.2478832	30.0549416	23.2198658
268.837978	286.058887	190.632643	179.390433	154.501415
335.126794	351.222881	120.888993	149.335491	157.18063
718.865387	673.729425	912.866882	776.732398	846.63203
117.846785	72.8953148	57.3447787	64.8059679	81.2695303
1012.00926	1015.01204	911.317023	824.632461	889.499474
198.866449	203.223302	275.874881	309.002369	254.525452
178.979804	153.521951	99.1909685	106.131513	120.564688
413.200289	400.924232	192.182501	225.412062	218.802582
18.4135601	17.6715915	6.19943553	6.57451848	4.46535881
438.979273	400.924232	802.826901	799.273604	734.998059
31.6713234	17.6715915	48.0456254	52.5961479	48.2258751
292.407335	266.178347	362.666979	325.908273	321.505834
47.8752563	68.477417	26.347601	22.5412062	21.4337223
64.0791892	82.835585	13.9487299	14.0882539	25.0060093
25.7789842	22.0894893	38.7464721	40.3863278	49.1189469
710.76342	834.982697	874.12041	947.669878	819.839877
418.356086	417.491349	251.077139	274.251342	272.386887
148.045023	147.999579	100.740827	89.225608	93.7725349
1379.54392	1365.13044	513.00329	515.630092	565.314425
508.950801	534.565642	381.265285	415.133881	397.416934
22.8328145	20.9850149	46.4957665	40.3863278	38.4020857
142.152684	178.924864	108.490122	107.07073	123.243903
4042.14472	3963.95886	3180.31043	3594.38318	3751.79447
1411.21525	1383.90651	985.710249	999.326809	1014.52952
61.869562	57.4326723	91.4416741	92.0432587	79.4833867

3189.96515	3201.87148	1983.81937	2110.42043	2222.85561
260.736011	220.894893	170.484477	142.760973	147.356841
1024.53048	1118.83264	742.382405	699.71661	693.023687
724.021184	727.848674	464.957665	445.188823	432.246732
163.512414	188.865134	125.53857	148.396274	141.99841
127.421836	114.865345	89.8918152	85.4687403	58.9427362
723.284641	733.371046	385.914862	388.835807	327.757336
279.149571	255.133602	340.968954	368.173035	360.800992
187.081771	147.999579	89.8918152	76.076571	99.1309655
49.3483411	71.7908404	20.1481655	35.6902432	32.1505834
79.5465797	75.1042638	35.6467543	39.4471109	30.3644399
67.7619012	70.6863659	26.347601	30.9941586	38.4020857
64.8157316	71.7908404	37.1966132	33.8118093	39.2951575
742.434744	749.938163	423.111475	511.873225	440.284378
282.832283	277.223091	224.729538	200.053205	221.481797
280.622656	258.447025	337.869236	344.692612	337.581126
489.064157	476.028495	376.615709	413.255447	398.310005
247.478248	199.909879	314.621353	290.21803	310.788973
155.410447	201.014353	94.5413918	111.766814	117.885473
45.6656291	47.4924021	15.4985888	20.6627724	25.0060093
5652.22641	6109.95275	7121.60157	6881.64242	6446.19197
95.7505126	93.8803297	40.296331	60.1098833	62.5150233
480.225648	508.058255	822.975067	850.930535	842.166671
222.435806	205.432251	161.185324	134.30802	141.99841
422.77534	407.551078	347.16839	337.178876	302.751327
106.798649	136.954834	100.740827	81.7118726	91.9863914
44.1925443	78.4176872	32.5470365	36.6294601	33.0436552
1501.80996	1526.38371	1253.83584	1311.14683	1347.64529
416.883001	430.745042	336.319378	323.090623	354.549489
218.753094	297.103632	139.487299	147.457057	132.174621
201.812619	204.327776	99.1909685	100.496211	106.27554
50.0848835	54.1192489	24.7977421	33.8118093	24.1129376
1228.55273	1193.9369	654.040449	751.373541	706.419763
452.973579	418.595823	385.914862	350.327913	357.228704
54.5041379	53.0147744	9.2991533	21.6019893	32.1505834
204.758788	238.566485	136.387582	167.180613	140.212267
80.2831221	70.6863659	40.296331	44.1431955	35.7228704
124.475666	131.432462	65.0940731	79.8334387	80.3764585
489.800699	481.550868	190.632643	235.743448	240.236304
335.863336	355.640778	272.775163	308.063152	314.36126
754.219422	827.251376	977.960955	896.012947	851.097388
25.0424418	44.1789787	10.8490122	18.7843385	10.7168611
69.9715284	80.6266361	35.6467543	73.2589202	37.509014

271.047605	251.820179	227.829256	191.600253	226.840227
1529.06203	1533.01056	1929.57431	1905.67114	1932.60729
401.41561	403.13318	522.302443	456.459426	476.90032
287.251538	312.566274	172.034336	195.357121	200.941146
58.18685	55.2237233	13.9487299	30.0549416	23.2198658
35.3540354	14.3581681	10.8490122	7.51373541	14.2891482
160.566244	157.939849	103.840545	123.037417	111.63397
2246.45433	2405.54539	1971.4205	2032.46543	1942.43108
249.687875	249.61123	120.888993	153.092359	161.645989
1316.93782	1372.86176	1613.4031	1609.81781	1628.06982
50248.3959	48363.8324	53248.5016	58123.4395	59374.983
67330.2874	69018.6094	72119.5834	75560.9409	78442.9581
40956.1769	39973.1399	43981.8954	44024.8542	46026.2394
12270.0599	11827.8171	12891.7262	13748.2574	14553.4974
56398.525	57070.4047	65263.0077	66564.182	68417.3345
86758.8029	85735.935	102874.983	103277.232	104191.11
34504.0655	33824.5306	37537.5821	37641.936	39101.3609
68734.1372	68607.7449	85803.2875	85943.0448	88848.1372
13831.5298	13974.9154	15216.5145	14826.4784	15514.4426
15819.4578	16496.4306	16924.459	17363.3033	17256.8256
1369.23233	1422.56311	2199.24975	2082.24393	2186.23967
742.434744	773.132127	902.01787	892.25608	853.776604
5163.8988	5170.04498	6775.98304	6579.21457	6527.4615
6946.33142	6948.24887	8651.31228	8151.4637	8201.07798
63930.4076	66054.2	77190.7217	78549.5292	80621.1602
4665.99613	4507.3603	5300.51738	5840.99006	5374.50586
87540.2744	84192.9841	95232.6289	104321.642	104620.678
30822.09	29815.2882	33793.1231	33441.7579	35555.866
56907.4758	54385.4272	63652.7043	65270.8803	66441.8598
28143.2853	27998.4277	33139.0826	34025.0116	34513.6513
57371.4975	54820.5902	60621.1803	65121.5448	66880.358
2765.71673	2584.47025	1983.81937	2028.70856	1897.77749
23728.4501	23326.5007	24365.3315	26133.711	27019.8861
38661.8474	38046.9364	47269.1461	49391.5397	49957.5412
564.191482	566.595402	320.820789	339.996527	358.121776
4936.30719	4874.04582	5345.46329	5183.53821	5264.65803
5121.17934	4893.92636	5562.44353	5264.31087	5478.99525
4284.46717	4216.88352	4679.02397	4718.62584	4721.6704
483.171817	536.774591	650.940731	640.545944	632.294807
10773.4058	10542.2088	11935.4633	11561.7604	11941.2625
3740.89887	3643.66127	2496.82266	2573.45438	2568.47439
382.265508	404.237655	216.980244	193.478687	191.117357
2045.37826	1963.7556	1667.64816	1580.70209	1716.48393

4533.4185	4417.89787	5161.03008	4940.28103	4961.01363
16310.7315	14479.6603	8623.41482	9610.06759	10017.5859
6125.82318	5656.01375	3759.95765	4197.36044	4380.51699
4416.30826	4416.79339	2526.26998	2569.69751	2586.33582
914.049124	745.520265	384.365003	571.043891	589.427362
80.2831221	66.268468	20.1481655	16.9059047	18.754507
22.0962721	30.9252851	4.64957665	4.69608463	1.78614352
2444.58424	2221.09815	945.413918	1020.9288	1099.37134
1515.80427	1425.87654	573.447787	763.583361	814.481446
129.631463	146.895104	182.883348	191.600253	230.412514
802.831221	757.669484	1286.38287	1220.04279	1118.12585
8128.48198	8095.79784	9218.56063	9300.126	9486.20825
617.222535	585.371468	729.983534	736.34607	750.180279
1488.5522	1608.11482	790.42803	842.477583	832.342881
1259.48751	1542.95083	2151.20413	2039.03995	2155.87523
1181.41402	1277.87696	881.869704	982.420905	910.933196
698.978742	774.236601	802.826901	816.179509	807.336872
550.933719	456.147955	623.043271	633.032208	628.72252
4385.37348	4578.04667	5136.23234	4985.36344	4994.05729
3197.33058	3144.43881	3733.61005	3746.53632	3833.95707
4361.80412	4441.09183	3715.01174	3436.59473	3704.46167
3742.37196	3572.9749	4034.28267	3965.37386	3920.58503
1568.83532	1500.9808	985.710249	1008.71898	991.309655
5985.88012	5830.52071	7254.88943	7053.51911	6754.30173
40.5098322	38.6566063	18.5983066	15.9666877	11.6099329
6721.68598	6739.5032	7527.66459	7485.5589	7383.91732
2890.92894	3131.18511	3304.29914	3325.76714	3482.97987
1605.66244	1690.95041	1833.48306	1772.30234	1815.61489
3297.50034	3584.01965	3790.95483	3829.18741	3566.92861
883.114343	875.848252	731.533393	756.069625	775.186289
782.944576	884.684048	650.940731	621.761605	598.35808
5510.07373	5928.81894	7567.96092	7339.04106	7165.11474
7585.65022	7337.02388	6658.19376	6417.66926	6411.36217
218.016552	233.044113	150.336312	173.755131	161.645989
215.806925	184.447236	100.740827	83.5903064	70.5526691
5668.43034	5489.2381	3825.05172	3774.71283	3810.7372
36849.953	36161.5985	60515.7899	60481.8132	58706.0722
59.6599348	55.2237233	27.8974599	28.1765078	31.2575116
994.332246	987.400173	615.293976	604.8557	661.766175
20.6231873	16.567117	43.3960487	56.3530156	47.3328033
572.293448	541.192489	517.652867	490.271235	451.894311
125.212209	159.044323	49.5954842	36.6294601	33.9367269
142.889226	180.029338	37.1966132	89.225608	82.162602

41.2463747	55.2237233	21.6980244	13.149037	15.1822199
9477.09112	9247.76471	12166.3922	11568.3349	11408.0987
1137.95802	1101.16104	822.975067	818.997159	772.507073
398.469441	436.267414	238.678268	217.898327	228.626371
4917.15709	4845.32949	5612.03901	5464.36408	5346.82063
2775.29178	2881.57388	2172.90215	2222.18725	2242.50319
7752.10881	7557.91878	6938.71822	6929.54248	7011.5064
4115.06241	4156.13742	4883.60534	4676.36107	4338.54262
8718.45244	8833.58679	9469.63777	9318.91034	9213.82136
17197.5286	17562.2485	19447.6293	18610.5834	18689.3127
201.076076	171.193542	117.789275	127.733502	119.671616
3483.10903	3628.19862	2259.69425	2270.08731	2267.5092
5.15579683	7.73132127	24.7977421	25.358857	20.5406505
3452.91079	3665.75076	1413.4713	1343.0802	1372.6513
3270.98482	3465.84088	2682.80573	2696.49179	2581.87046
4090.75651	4244.49538	4606.1806	4668.84734	4660.94152
8202.87276	8894.33288	5272.61992	5141.27345	4834.19744
1283.79341	1373.96624	1528.16086	1502.74708	1424.44946
1523.16969	1487.72711	1855.18108	1755.39644	1620.92525
1537.90054	1477.78684	1366.97553	1239.76634	1301.20556
5444.52145	5362.22354	6682.9915	6699.43433	6832.89204
6448.42875	6415.89218	7636.15472	7767.32398	7919.76038
475.069851	520.207474	787.328312	833.085413	770.72093
72.917698	62.9550446	116.239416	118.341333	93.7725349
168.668211	178.924864	362.666979	339.05731	348.297987
8983.60771	9310.71976	11010.1975	10929.6674	11044.6185
799.885051	840.505069	858.621821	945.791444	963.62443
768.213728	722.326301	1074.05221	1018.11115	995.775014
1357.44765	1192.83242	1521.96142	1419.15678	1441.41782
1294.84155	1350.77227	1813.33489	1659.59631	1683.44027
4902.42624	4735.98651	6162.23892	6095.51785	5710.30084
8459.18951	8423.82676	10382.5047	10284.4253	9816.6448
3856.53603	4104.22712	4330.30572	4165.42707	4363.54862
10989.9492	11377.1915	11797.5258	11713.9135	11692.0955
5503.44485	5406.40252	6419.51549	6198.83171	6206.84874
24318.4206	24554.6764	30395.8324	29624.7803	29147.1831
9724.56937	9515.04753	10225.9689	10345.4744	10320.3373
816.088984	865.907982	675.738473	708.169562	662.659247
4014.1561	4164.97322	3591.02303	3620.68125	3808.95106
375.636626	364.476574	292.923329	267.676824	309.002829
3782.14525	3598.37781	6063.04795	6100.21393	6246.1439
1771.38448	1760.5323	1145.34571	1079.16025	1101.15748
1260.9606	1157.48924	1504.91298	1396.61557	1501.25363

1381.01701	1429.18996	1132.94684	1143.027	1026.13945
2795.91497	2867.21572	3194.25916	2981.07452	2941.77838
1326.51287	1252.47405	1504.91298	1354.35081	1492.32291
1931.21418	1802.50233	2341.83677	2259.75592	2327.34501
5685.37082	5590.84975	6712.43882	6713.52259	6458.69498
1478.97715	1464.53314	2210.09877	2087.87923	2056.74427
6510.29831	6546.22017	5953.00797	5916.12742	5800.50109
2127.87101	2370.20221	2605.31278	2476.71503	2416.65219
357.223066	321.40207	389.01458	407.620146	379.555498
4422.2006	4198.10745	5015.34334	5061.44001	5156.59635
2342.94139	2252.02344	2535.56913	2892.78813	3022.15484
3160.50346	3204.08043	2487.52351	2513.34449	2375.57088
773.369525	716.803929	432.410628	454.580992	394.737718
1348.60914	1379.48861	1619.60253	1498.051	1538.76264
5713.35943	5625.08846	6359.071	6282.42202	6469.41184
1000.22459	922.23618	520.752585	578.557626	521.553909
470.650596	468.297174	350.268108	349.388696	357.228704
10332.2169	11123.1624	8756.70269	8760.07627	8656.54458
1037.05171	1098.9521	1343.72765	1339.32334	1296.7402
779.261864	776.44555	1046.15475	951.426746	970.769004
310.084352	342.387085	395.214015	428.282918	401.882293
278.413029	256.238076	199.931796	208.506158	198.261931
1685.94556	1635.72669	2052.01316	1963.90259	1952.25487
6034.49192	6577.14545	7349.43082	7312.74299	7545.56331
676.145927	677.042848	530.051738	546.624251	474.221105
3681.23894	4084.34658	4524.03808	4322.27629	4248.34237
5258.17623	5430.70095	6876.72386	6686.2853	6901.65857
2668.49313	2647.4253	3064.07101	2983.89217	3039.1232
2191.9502	2295.09794	2733.95107	2845.82729	2724.76194
72.917698	83.9400595	151.886171	147.457057	159.859845
1174.78514	1165.22056	1343.72765	1189.98785	1339.60764
7152.56329	6865.41329	6334.27325	6341.59268	6452.44347
31.6713234	34.2387085	6.19943553	5.63530156	4.46535881
7709.38935	7575.59037	9203.06205	9179.90623	9075.39524
2915.23484	3031.78241	2673.50657	2702.1271	2564.9021
2629.45638	2701.54455	3685.56442	3569.96354	3374.02511
1966.56822	2032.23302	2265.89369	2125.4479	2061.20962
162.039329	182.238287	263.47601	239.500316	250.953165
2145.54802	2098.50149	2442.5776	2366.82665	2459.51963
33.8809506	43.0745042	18.5983066	27.2372909	13.3960764
9.57505126	19.8805404	3.09971777	1.87843385	6.25150233
1020.11123	1001.75834	1177.89275	1094.18772	1086.86833
92.804343	64.0595191	29.4473188	31.9333755	56.263521

482.435275	416.386874	313.071494	332.482792	355.442561
964.134007	931.071976	1170.14346	1072.58573	1027.03253
54.5041379	55.2237233	35.6467543	30.9941586	24.1129376
39.0367474	49.701351	80.5926619	102.374645	95.5586784
1358.92074	1325.36936	1470.81608	1552.52558	1459.27926
3069.1722	3032.88689	2549.51786	2512.40528	2454.1612
1056.20181	968.624108	1170.14346	1195.62315	1163.67251
1053.99218	1048.14627	722.234239	759.826493	759.110997
13620.1421	13453.6035	12780.1364	11857.6137	12024.3182
1109.23286	1146.4445	875.670269	881.924694	853.776604
975.182143	939.907771	774.929441	743.859805	808.229944
6922.76206	7348.06863	5440.00468	5354.4757	5194.99843
1832.5175	1862.14395	2644.05925	2564.06221	2401.46997
5037.95005	4999.95591	4251.26292	4166.36628	4100.09246
2782.6572	2753.45485	3400.39039	3302.28671	3441.0055
10197.4296	10371.0152	11279.873	11297.8404	11441.1423
2780.44758	2670.61926	2417.77986	2359.31292	2345.20645
32505.0894	31759.1633	34913.6711	35988.9142	36781.1605
19257.6377	19920.3015	23316.077	24290.0281	24924.7398
2456.36892	2499.42572	2228.69707	2047.4929	1986.1916
41.2463747	26.5073872	9.2991533	10.3313862	6.25150233
1681.52631	1887.54686	2101.60865	2147.98911	2062.1027
1037.78825	1048.14627	1408.82172	1366.56063	1336.92843
111.217903	77.3132127	46.4957665	62.927534	75.0180279
2767.18981	2766.70854	3118.31607	2938.80976	2938.20609
4464.92006	4319.59964	5071.13826	4872.65741	4747.56948
986.966822	999.549393	926.815612	800.212821	794.833867
43.4560019	53.0147744	34.0968954	32.8725924	28.5782964
3745.31813	3682.31787	3947.49057	4018.90923	4126.88461
3097.89735	3356.49791	5539.19565	5224.86376	5149.45177
1415.6345	1272.35459	1659.89886	1494.29413	1520.90121
6764.40544	7092.93503	7803.53947	7919.47712	7683.09636
3512.57073	3540.94514	3829.7013	3722.11668	3734.82611
693.822945	762.087382	840.023514	872.532524	837.701312
8345.02544	8084.7531	9359.59779	9348.96528	9430.8378
901.527903	1002.86282	1016.70743	1198.4408	1152.06257
126818.608	123712.185	160301.904	156314.812	155270.349
1493.708	1611.42825	1261.58513	1324.29587	1267.26883
2569.79645	2513.78389	3291.90027	3159.52574	2932.84766
83.9658341	111.551921	141.037158	115.523682	147.356841
1282.32033	1370.65281	1542.10959	1566.61383	1553.05179
1014.95543	976.355429	838.473656	791.759869	864.493465
123.002582	110.447447	176.683913	172.815914	183.079711

916.258751	839.400595	2005.51739	1830.53379	1881.7022
6080.15755	6252.42996	7461.02066	7401.96859	6951.67059
221.699264	186.656185	277.42474	293.035681	288.462179
2776.02832	2850.6486	2541.76857	2441.96401	2536.3238
181.189432	198.805404	113.139698	109.88838	111.63397
1230.02582	1259.10089	1444.46848	1330.87038	1372.6513
3532.45737	3447.06481	2516.97083	2590.36028	2513.10394
2138.1826	2182.44155	1494.06396	1390.04105	1386.04737
1290.42229	1227.07113	853.972244	839.659932	839.487455
1593.14122	1523.07029	2425.52915	2363.06979	2402.36304
42.7194595	38.6566063	106.940263	116.462899	107.168611
3581.06917	3695.57157	3110.56678	2902.1803	3026.6202
100.169767	69.5818914	15.4985888	25.358857	30.3644399
145.098854	143.581681	80.5926619	108.949163	95.5586784
366.061575	374.416844	559.499057	517.508526	484.044895
70.7080708	51.9102999	99.1909685	114.584465	100.917109
1157.84466	1222.65324	1889.27798	1854.01421	1724.52157
729.913523	723.430776	1061.65334	1017.17193	1063.64847
3849.17061	3733.1237	4243.51362	4183.27219	4075.97952
7629.84277	7414.3371	8992.28124	8597.59174	8637.79007
246.741706	281.640989	322.370648	375.68677	375.09014
1093.02893	1130.98185	1366.97553	1282.97032	1334.24921
3762.2586	3802.70559	4533.33723	4291.28214	4468.93109
7346.27394	7246.45698	9571.92846	9461.67131	9049.49616
8601.3422	8664.60219	10610.3339	10349.2313	10632.0193
24123.2368	25007.5109	28642.942	27860.9309	28341.6323
2675.85856	2718.11166	3271.7521	3404.66136	3106.99666
1857.55994	1869.87527	2138.80526	2121.69104	2035.31054
7514.20561	7440.84448	8595.51736	8606.04469	8396.6607
72.917698	69.5818914	105.390404	121.158983	125.923118
718.865387	728.953148	857.071962	825.571678	851.097388
288.724623	300.417055	503.704137	521.265394	516.195478
6371.82834	6181.74359	6786.83205	6673.13626	7144.57409
1814.84048	1877.60659	2506.12181	2408.1522	2393.43232
286.514995	248.506755	162.735183	176.572782	184.865855
4221.86106	4138.46583	4725.51973	4743.98469	4531.44612
3077.27417	3045.03611	3564.67543	3648.85776	3523.1681
170.877838	167.880119	116.239416	105.192296	107.168611
1149.00615	1195.04137	1339.07808	1314.9037	1318.17392
9570.632	9664.15159	10907.9068	10675.1396	10983.8896
668.780503	657.162308	441.709782	485.575151	371.517853
356.486524	381.043691	247.977421	216.019893	265.242313
621.641789	586.475942	509.903572	467.730029	461.718101

2482.1479	2450.82884	2741.70036	2935.05289	3013.22412
5042.3693	4699.53886	4455.84429	4308.18804	4292.10288
3107.4724	3189.72226	2743.25022	2956.65488	2981.96661
1375.86121	1293.3396	1442.91862	1671.80613	1625.39061
1312.51857	1375.07071	1473.9158	1529.98437	1506.61206
9206.04351	9401.28666	8471.52865	8424.77583	8825.33514
5695.68241	5685.83456	5367.16131	5222.04611	5330.74534
6151.60216	6026.01269	6439.66366	6482.47522	6563.18437
4660.10379	4842.01606	5029.29207	5070.83218	4983.34043
1207.92954	1189.519	1515.76199	1545.01184	1479.81991
265.155266	288.267836	390.564438	359.720083	319.719691
1381.01701	1346.35438	1602.55409	1557.22166	1552.15872
2386.39739	2402.23197	3101.26762	3018.6432	3081.09758
1679.31668	1739.54729	1886.17826	1900.97506	1872.77148
1305.15314	1253.57852	1461.51693	1416.33912	1391.4058
1438.46732	1472.26446	1779.238	1780.75529	1742.38301
3640.72911	3616.04941	3048.57242	3121.01785	3127.53731
4553.30514	4697.32991	5808.87109	5933.97254	5582.59158
1036.31516	1062.50444	708.285509	769.218662	724.281198
159.829702	192.178557	125.53857	127.733502	136.639979
626.797586	610.77438	364.216837	404.802495	357.228704
1258.01443	1308.80224	1807.13546	1832.41222	1858.48234
103.115937	121.492191	196.832078	148.396274	144.677625
466.231342	453.939006	365.766696	300.549416	324.185049
603.964772	580.95357	705.185792	796.455953	779.651647
78.8100373	80.6266361	26.347601	16.9059047	16.9683635
4394.94853	4405.74865	5078.88756	5274.64226	5196.78458
1995.29337	2153.72521	2772.69754	2825.16451	2856.04349
357.959609	410.864502	167.384759	152.153142	169.683635
1800.10964	1701.99515	2110.9078	2037.16151	2025.48675
139.943057	121.492191	193.73236	193.478687	204.513433
2121.97867	2256.44134	2495.2728	2504.89154	2451.48198
215.806925	228.626215	168.934618	136.186454	169.683635
3195.85749	3248.25941	4004.83535	3808.52464	3892.89981
5550.58356	5577.59606	6961.9661	6934.23856	6410.4691
4989.33825	5388.73092	4643.37721	4564.59426	4399.2715
1054.72872	1023.84783	1280.18344	1232.25261	1298.52634
2.94616962	2.20894893	34.0968954	32.8725924	25.0060093
5918.11822	6156.34068	5729.82829	5449.3366	5409.33566
960.451295	881.370625	757.880994	783.306916	739.463418
280.622656	313.670749	102.290686	125.855068	100.917109
234.957027	265.073872	185.983066	193.478687	218.802582
4506.16643	4372.61441	5794.92236	5481.26998	5649.57196

2950.58887	2868.32019	3549.17684	3511.73209	3366.88054
2924.07335	2905.87232	3845.19989	4026.42296	3967.91783
2963.11009	3083.69271	3462.38474	3388.69467	3324.0131
4955.4573	5453.89492	6386.96846	6106.78845	5878.19833
11471.648	11431.3107	9432.44116	9361.1751	9569.26392
1548.94868	1647.87591	2231.79679	2144.23224	2208.56647
1286.00304	1230.38456	1580.85606	1592.91191	1570.02016
7112.79	7712.5452	10188.7723	9986.69357	9514.78654
5445.258	5646.07348	6419.51549	6098.3355	6082.71176
1983.5087	1962.65113	1876.87911	1755.39644	1735.23843
3391.04123	3365.3337	4023.43366	3982.27977	3898.25824
6632.56435	6246.90759	6548.15378	7023.46417	7312.47158
5101.29269	5046.34384	5615.13873	5386.40907	5324.49384
4754.38122	4768.01627	5878.61474	5656.90355	5578.12622
5416.53284	5413.02936	6084.74597	6173.47285	6006.80067
535.466328	534.565642	621.493412	665.904801	662.659247
7678.45457	7317.14334	6183.93694	6030.71188	6290.79749
11216.0677	11156.2966	10548.3396	10035.5329	10330.1611
4544.46664	4520.61399	3780.10582	3767.19909	3937.55339
12019.6355	11744.9815	8663.71115	8999.57658	9211.14214
12038.7856	11537.3403	10718.824	10547.4061	10648.9877
4277.10174	3937.45147	3541.42755	3827.30897	4139.38761
777.052237	752.147112	432.410628	486.514368	436.712091
24006.1266	23962.678	15394.7483	15027.4708	14666.9175
360.169236	393.19291	289.823611	277.068993	261.670026
7466.33035	7341.44178	8276.24643	7880.96923	8105.5193
6726.84178	6580.45887	7757.04371	7417.93528	7667.91414
9531.59526	9183.70519	10528.1914	10466.6334	10582.0073
3169.34197	3313.4234	2836.24176	2765.05463	2697.07672
1445.0962	1504.29422	1103.49952	1064.13278	1090.44062
1225.60656	1320.95146	1755.99011	1696.22577	1800.43267
6186.21965	5954.22185	4899.10393	4874.53585	5151.23792
607.647484	548.92381	435.510346	486.514368	465.290388
9382.81369	9119.64567	11560.3974	11220.8246	11056.2284
360.169236	352.327355	283.624176	300.549416	280.424533
590.707008	559.968555	443.25964	482.7575	519.767765
9455.73139	9536.03255	10625.8325	10621.6042	10473.0525
15007.788	14696.1373	13691.4534	13279.5881	12944.1821
4548.14935	4652.04646	4813.86169	5184.47743	5028.88709
2588.21001	2597.72395	3093.51833	3045.88049	2996.25576
5196.30666	4940.31429	6566.75209	6728.55006	6556.93287
35119.0784	34528.0808	25230.1528	25539.1867	25482.9096
1708.77838	1627.99536	1906.32643	1945.11825	1883.48834

191.501025	182.238287	232.478832	242.317967	201.834218
15965.2932	15532.2244	17979.9129	18176.6652	18548.2074
2147.75765	2267.48608	2636.30996	2584.72498	2564.00903
1571.78149	1369.54834	1678.49717	1551.58636	1760.24444
4593.07843	4565.89745	5306.71681	5141.27345	5282.51947
1010.53618	974.14648	1132.94684	1168.38586	1160.99329
9935.22049	9181.49624	10867.6105	10948.4517	11521.5188
2038.74938	2192.38182	2918.38428	2776.32523	2703.32822
5381.91535	5212.01501	4229.56489	4176.69767	4150.10447
4629.16901	4561.47955	5142.43177	5253.97948	5356.64442
307.138183	310.357325	398.313733	367.233818	411.706082
116.3737	97.1937531	58.8946375	56.3530156	60.7288798
90.5947157	119.283242	46.4957665	51.6569309	69.6595974
2669.22967	2603.24632	2177.55173	2287.93243	2369.31938
5084.35222	4967.92615	5762.37533	5759.27819	5858.55075
3846.96098	3933.03358	3733.61005	3557.75372	3752.68754
990.649534	982.982276	1154.64487	1272.63894	1159.20715
989.176449	1064.71339	1287.93273	1330.87038	1215.47067
2149.96728	2183.54602	1481.66509	1462.36075	1357.46908
95667.2833	97688.5577	84481.2578	82589.1012	83525.4295
736.542404	748.833689	438.610064	441.431955	424.209087
12054.253	12050.9209	14206.0065	14518.4152	15146.4971
1161.52737	1253.57852	1312.73047	1455.78624	1415.51874
8396.58341	8276.93166	9323.95104	9103.82966	8990.55342
3111.89166	3088.11061	2901.33583	2667.37607	2733.69266
14328.6959	15040.7333	15864.3555	15521.4989	15261.7033
123.739124	120.387717	63.5442142	71.3804864	57.1565927
1338.29755	1175.16083	1469.26622	1592.91191	1443.20397
4886.22231	5128.07495	6717.0884	6511.59095	6348.84715
922.15109	977.459903	795.077607	830.267763	846.63203
6778.39975	6466.698	7383.52772	7361.58227	7521.45037
646.684231	612.983329	475.806677	509.055574	508.157832
175.297092	177.820389	133.287864	135.247237	154.501415
593.653178	693.609965	505.253996	515.630092	502.799402
2177.21935	2199.00866	2569.66603	2410.03063	2396.11154
392.577102	456.147955	195.282219	241.37875	236.664017
874.275834	868.116931	1937.3236	1719.70619	1686.11949
2854.10182	3019.63319	1416.57102	1524.34907	1499.46749
59.6599348	73.9997893	38.7464721	30.0549416	33.9367269
2504.98072	2658.47004	1715.69378	1677.44143	1655.75505
28963.057	28623.5603	17905.5197	17044.9088	16788.856
2447.53041	2268.59056	4248.1632	4208.63105	4157.24905
1366.28616	1438.02576	1264.68485	1217.22514	1119.91199

2074.83995	2006.83011	1669.19802	1550.64715	1570.02016
5064.46557	5080.58255	5604.28972	5500.05432	5664.75418
11739.7494	11823.3992	9004.68011	9242.83377	9261.15416
5780.38479	5883.53549	6166.88849	6011.92754	6154.15751
4285.94025	4448.82315	3290.35041	3194.27677	3047.16085
3625.99826	3660.22838	4418.64767	4339.1822	4203.68878
506.004632	479.341919	674.188614	631.153774	668.017677
4517.95111	4830.97132	5435.3551	5260.554	5177.137
10159.8659	9912.65834	10455.348	10430.9432	10717.7542
123.002582	112.656396	55.7949198	82.6510895	78.590315
23738.0252	23721.9026	29397.7233	28795.4517	26578.7087
413.936831	439.580838	595.145811	639.606727	634.974022
5854.77557	6164.072	6620.99715	6491.86739	6339.91643
8152.05133	8378.54331	6433.46422	6716.34024	6773.05624
2063.05528	2096.29254	1652.14957	1557.22166	1454.8139
5940.21449	5797.38648	6858.12556	6985.8955	7024.90247
3379.25655	3439.33349	3769.2568	3772.83439	3790.19655
86.1754613	88.3579574	20.1481655	29.1157247	29.4713681
3666.50809	3550.88541	2988.12793	3119.13941	3213.2722
1498.12725	1683.21909	1044.60489	1145.84465	1047.57318
2152.91345	2217.78473	2659.55784	2688.03884	2705.11436
959.714753	954.265939	1114.34854	1151.47995	1142.23878
4172.51272	4210.25667	5855.36686	5809.9959	5406.65644
29.4616962	37.5521319	23.2478832	11.2706031	12.5030047
82.4927493	96.0892786	37.1966132	47.9000632	62.5150233
875.748919	808.47531	1190.29162	1158.05447	1043.10782
858.808444	869.221405	1052.35418	1035.01705	1028.81867
3875.68613	4332.85333	4488.39132	4617.19041	4402.84378
4455.345	4531.65874	5398.15849	5419.28166	5509.35969
3782.88179	3833.63088	4184.61898	4082.77598	4118.84696
5104.23886	4924.85165	5503.54889	5585.52306	5667.4334
1301.47043	1288.9217	1659.89886	1553.4648	1578.0578
2292.11996	2176.91917	2740.15051	2661.74077	2481.84642
2082.94192	2026.71065	2467.37534	2552.79161	2516.67622
5514.49298	5714.55089	6966.61568	6870.37181	6794.48996
327.024828	333.551289	495.954842	526.900695	513.516263
998.7515	1001.75834	1374.72483	1323.35665	1316.38778
870.593122	843.818493	1039.95531	995.569942	1061.86232
5067.41174	5086.10492	5863.11615	5862.59205	6038.05818
4385.37348	4225.71931	4728.61945	4672.60421	4788.65078
2759.82439	2724.73851	3054.77186	3013.0079	2948.92296
4961.34964	5261.71636	6138.99103	5860.71362	5759.41979
1814.10394	1819.06945	2464.27562	2682.40354	2687.25293

958.978211	976.355429	1022.90686	1074.46416	1069.89997
123.739124	110.447447	162.735183	191.600253	200.941146
352.803812	354.536304	444.809499	413.255447	448.322024
8946.78059	8856.78075	12248.5348	11938.3864	11128.5672
6066.89979	6000.60978	6585.35039	6379.16136	6280.9737
22.8328145	22.0894893	10.8490122	6.57451848	8.93071761
4365.48683	4554.8527	5210.62556	5182.599	5104.79819
51653.7188	48762.5477	26890.0516	32831.2669	34141.2404
2806.22656	2613.18659	1432.06961	1696.22577	1837.94168
2077.78612	2105.12833	2321.68861	2292.62852	2242.50319
990.649534	954.265939	345.618531	348.44948	353.656417
398.469441	352.327355	514.553149	542.867383	538.522272
93.5408854	94.9848042	195.282219	156.849227	175.042065
17127.5571	16528.4604	23316.077	22533.6925	21420.3262
4068.66024	4370.40547	5632.18718	5601.48975	5437.91395
285.041911	322.506544	249.52728	212.263025	207.192649
1014.95543	1001.75834	1601.00423	1675.563	1670.04419
2152.91345	2249.81449	2800.595	2621.35444	2510.42472
405.098322	407.551078	516.103008	497.784971	543.880703
508.950801	499.222459	595.145811	631.153774	597.465008
2256.76593	2239.87422	2707.60347	2791.3527	2664.03306
2262.65827	2345.90377	2585.16462	2616.65836	2589.01504
563.454939	569.908825	376.615709	386.957374	348.297987
15835.6617	15111.4197	24337.434	23239.9836	22692.9535
1297.78772	1280.08591	1005.85842	972.089518	949.335282
72.917698	75.1042638	34.0968954	36.6294601	25.0060093
1306.62623	1187.31005	1523.51128	1448.2725	1445.88318
1302.94351	1181.78768	1754.44026	1602.30408	1612.8876
887.533597	854.863237	1105.04938	999.326809	1002.91959
1775.0672	2006.83011	2208.54891	2106.66357	2127.29694
598.808975	606.356482	853.972244	809.60499	779.651647
8333.24076	8387.3791	9977.99149	9802.60706	10197.0934
1627.75871	1544.05531	1952.82219	1883.12994	1940.64494
1520.22352	1517.54792	1794.73659	1775.11999	1723.6285
2510.87306	2552.44049	3316.69801	3270.35334	3020.3687
1080.50771	1079.07155	1311.18062	1267.94285	1294.95405
934.672311	944.325669	1061.65334	1003.08368	1035.07017
7014.82986	7223.26301	8474.62837	8162.7343	7962.62782
3881.57847	3981.63045	3019.1251	3163.28261	3182.01469
1917.95642	2054.32251	1879.97883	1709.37481	1812.93568
2183.84823	2194.59077	1711.04421	1637.0551	1646.82433
2132.29026	2143.78494	1968.32078	1882.19072	1854.91005
2703.84717	2891.51415	3223.70648	3105.05116	3062.34307

4894.32428	5259.50741	5753.07617	5580.82697	5805.85952
23400.6887	24307.2741	21485.6937	21835.8543	21761.4796
5577.83563	5808.43122	6805.43035	6816.83645	6672.13913
69.234986	86.1490084	29.4473188	40.3863278	42.8674445
6709.16476	6766.01058	7823.68764	7745.72199	7722.39152
6986.84125	7251.97935	8297.94446	8201.2422	7759.90053
48.6117987	45.2834531	13.9487299	22.5412062	16.0752917
8020.21024	8581.76661	9609.12507	9659.84608	9422.80015
3776.98945	3723.18343	4184.61898	4218.96243	4180.46891
8132.90123	7880.42532	8877.59168	8713.11542	8867.30952
45047.67	45227.1249	49133.6263	48485.1954	47811.4898
1842.09255	1970.38245	1461.51693	1538.43733	1528.93886
4975.34394	4789.00129	6605.49856	6462.75167	6667.67377
1200.56412	1214.92191	1438.26904	1393.79792	1401.22959
771.89644	899.042216	1019.80715	1005.90133	1014.52952
182.662516	228.626215	89.8918152	115.523682	109.847827
5160.95263	5064.01543	5618.23845	5371.3816	5474.5299
512.633513	476.028495	320.820789	380.382855	363.480207
1713.19763	1812.4426	1573.10677	1595.72956	1667.36498
3948.60383	3983.8394	4638.72764	4348.57437	4296.56824
1655.01078	1826.80077	1233.68767	1253.8546	1216.36374
33.1444082	38.6566063	15.4985888	13.149037	13.3960764
574.503075	563.281978	508.353714	462.094728	425.99523
694.559487	714.59498	809.026337	844.356016	820.732948
1411.95179	1393.84678	1566.90733	1556.28245	1598.59845
1525.37932	1515.33897	1289.48259	1342.14099	1276.19955
208.4415	228.626215	162.735183	169.059047	158.073702
6295.96447	6106.63933	5540.74551	5463.42486	5827.29324
3100.10698	2974.34974	2698.30432	2874.00379	2705.11436
81.0196645	64.0595191	30.9971777	32.8725924	31.2575116
760.848304	875.848252	568.79821	641.48516	622.471018
345.438388	332.446815	102.290686	154.031576	158.966774
2669.22967	2617.60449	2213.19848	2321.74424	2348.77873
2941.75036	3029.57346	3550.7267	3623.4989	3629.44364
1667.532	1687.63699	1969.87064	1922.57705	1936.17958
1970.98747	1951.60638	2258.14439	2203.40291	2142.47916
991.386076	1016.11651	612.194259	682.810705	655.514673
3913.98634	4058.94367	4582.93272	4294.09979	4114.3816
517.78931	577.640146	664.889461	814.301075	837.701312
3624.52517	3836.9443	3065.62087	3046.81971	2993.57654
950.876244	904.564588	1511.11241	1545.01184	1533.40421
44.1925443	37.5521319	77.4929441	84.5295233	87.5210326
1199.09103	1158.59372	923.715894	1040.65235	1026.13945

242.322451	228.626215	172.034336	139.004105	135.746908
120.792954	143.581681	105.390404	111.766814	96.4517502
142.152684	135.850359	201.481655	200.053205	192.9035
2194.15982	2307.24716	2565.01645	2642.01721	2619.37948
185.608686	199.909879	94.5413918	130.551153	125.923118
2281.80837	2356.94851	1972.97036	1996.77519	1934.39343
2811.38236	2853.96202	3211.30761	3124.77471	3073.05993
42530.9046	42893.3704	31865.0986	30032.4004	31649.5701
30.934781	36.4476574	7.74929441	3.7568677	8.93071761
4350.01944	4813.29973	5387.30948	5438.066	5118.19426
342.492218	352.327355	291.37347	287.400379	249.167021
4062.7679	4099.80922	4440.3457	4454.70588	4569.8482
13083.9393	12729.0682	15151.4204	14589.7957	14822.312
667.307418	679.251797	883.419563	892.25608	953.800641
67.7619012	65.1639936	41.8461898	27.2372909	18.754507
503.058462	540.088014	303.772341	325.908273	324.185049
2764.24364	2882.67836	3195.80902	3198.03363	3074.84607
816.825527	798.53504	426.211193	476.182981	415.278369
429.404222	416.386874	519.202726	506.237923	554.597564
754.955965	606.356482	187.532925	234.804232	217.90951
4906.10896	5015.41855	5732.92801	5647.51138	5412.90794
5208.09134	5259.50741	6227.33299	6588.60674	6263.11226
4596.0246	4709.47913	5574.8424	5415.5248	5328.06613
13041.9564	14265.3922	17341.371	16951.9263	15898.4635
4796.36414	4854.16528	5573.29254	5797.78608	5491.49826
1178.46785	1227.07113	1404.17215	1483.96274	1439.63168
547.987549	588.684891	420.011757	454.580992	384.913929
3465.43201	3398.46794	5016.8932	5096.19104	5180.70929
3579.59609	3328.88604	4223.36546	4323.21551	4253.7008
2189.74057	2168.08338	2644.05925	2679.58589	2785.49082
14898.0432	14666.3165	19277.1448	19171.2959	19113.5218
1025.26703	987.400173	1081.8015	1130.81718	1131.52192
5676.53231	5517.95444	6334.27325	6003.47459	5996.0838
3455.85696	3461.42298	4195.468	4035.81513	3815.20256
317.449776	311.4618	449.459076	456.459426	438.498235
12700.2007	13124.4701	11723.1326	11670.7095	11440.2493
2155.12308	2295.09794	1788.53715	1707.49637	1605.74303
13817.5355	13807.0353	15701.6203	15602.2716	15815.4078
3150.19186	2982.08106	3758.40779	3528.63799	3600.86534
9575.7878	9507.31621	5478.75115	5646.57216	5906.77663
444.871612	447.312159	227.829256	221.655195	280.424533
33464.0676	32470.4449	38258.2665	38398.0056	38731.6292
299.772759	292.685734	164.285042	185.025734	192.9035

773.369525	820.624529	706.735651	677.175404	720.708911
8383.32565	8232.75268	11145.0352	10526.7433	10640.057
1461.30013	1397.1602	950.063495	1020.9288	999.347301
6883.72531	7180.18851	8566.07004	8224.72262	8076.94101
3393.9874	3373.06502	4116.42519	3951.28561	3914.33353
8487.91467	8601.64715	9753.26195	8855.8764	9273.65717
2174.27318	2203.42656	2439.47788	2409.09142	2321.98658
1154.16195	1276.77248	1625.80197	1562.85697	1479.81991
819.771696	818.41558	615.293976	680.932271	649.26317
2702.37408	2616.50001	2766.49811	2904.05874	2956.9606
1540.84671	1692.05488	1797.8363	1827.71614	1912.06664
2508.66343	2398.91854	2392.98212	2137.65772	2152.30294
26.5155266	35.3431829	15.4985888	10.3313862	14.2891482
1754.44401	1729.60702	2053.56302	1951.69277	2180.88124
1173.31205	1271.25011	474.256818	507.17714	492.975612
1235.91816	1340.832	895.818434	870.65409	924.329273
3820.44545	3868.97406	4956.44871	4810.6691	4794.90229
26993.5426	29159.2304	32051.0817	31218.6314	31059.2497
214.33384	229.730689	125.53857	132.429587	118.778544
1344.18989	1356.29465	1706.39463	1660.53553	1661.11348
22158.8782	21808.9528	20778.958	20481.5035	20402.2244
61490.9792	61764.4211	70918.4428	68637.0337	67245.6244
5591.09339	5546.67077	6273.82876	6347.22799	6309.55199
1208.66609	1216.02639	1024.45672	979.603254	1017.20874
1829.57133	1792.56206	1452.21777	1390.04105	1459.27926
1548.94868	1601.48798	1799.38616	1883.12994	1822.75946
1390.59206	1405.996	1139.14628	1193.74471	1118.12585
1767.70177	1865.45738	1233.68767	1411.64304	1348.53836
4392.7389	4806.67288	5940.6091	5701.04674	5671.89875
11478.2768	11537.3403	14184.3085	13867.5379	13430.0131
4099.59502	4364.88309	5997.95388	6320.92991	6423.86518
5070.35791	4834.28474	4489.94118	4329.79003	4414.45372
410.254119	420.804772	358.017402	348.44948	373.303996
1698.46678	1600.3835	2109.35794	1989.26145	1944.21722
136.996887	152.417476	288.273752	281.765078	282.210677
3838.12247	3845.78009	5560.89367	5551.71125	5606.70452
284.305368	305.939427	427.761052	460.216294	437.605163
9785.70239	10273.8215	12798.7347	12763.958	11518.8396
3503.73222	3645.87022	3987.78691	3918.41302	3962.5594
2924.07335	3053.8719	2754.09923	2715.27613	2737.26495
3352.00448	3115.72247	3721.21118	3435.65552	3460.65307
2949.85233	3257.0952	1929.57431	1977.99085	1976.36781
715.182675	758.773959	379.715426	402.924061	422.422943

3454.38388	3477.9901	3716.5616	3771.89518	3741.97068
448.554324	483.759817	582.74694	581.375277	577.817429
771.89644	741.102367	943.86406	931.703191	922.543129
416.146459	469.401648	613.744118	582.314494	570.672855
190.764483	145.79063	230.928974	256.406221	255.418524
4347.80981	4405.74865	5188.92754	4990.99874	4944.04527
209.178043	227.52174	133.287864	153.092359	142.891482
3770.36057	4016.97364	4197.01785	4180.45454	4230.48093
3469.11473	3487.93037	3099.71777	3152.012	3139.14724
3014.66806	2796.52935	2335.63734	2203.40291	2211.24568
332.917167	312.566274	257.276575	237.621882	267.028457
369.007745	337.969187	533.151456	457.398643	479.579536
1089.34622	1083.48945	649.390872	596.402748	592.106578
2207.41759	2246.50107	2428.62887	2446.66009	2400.57689
1970.25093	1879.81554	2501.47224	2382.79334	2453.26813
2348.09719	2403.33644	2665.75728	2833.61747	2702.43515
3164.18617	3381.90082	4114.87533	3853.60705	3733.03996
4445.03341	4430.04709	5948.35839	5607.12505	5928.21035
10668.8167	10800.6558	11995.9078	13889.1399	13010.2694
51551.3394	51416.5999	58979.8798	58150.6768	58320.2652
203.285704	198.805404	114.689557	122.0982	110.740898
6679.70307	6485.47407	8490.12696	8116.71267	8160.88975
457.392833	434.058466	336.319378	366.294601	360.800992
1288.94921	1417.04074	1149.99529	1167.44664	1046.6801
27.252069	29.8208106	0	2.81765078	0.89307176
97.9601398	104.925074	139.487299	153.092359	119.671616
1944.47195	1912.94978	2045.81373	2121.69104	2230.89326
2244.98125	2352.53061	2101.60865	1946.99669	1960.29252
843.341053	891.310895	1246.08654	1121.42501	1101.15748
1006.85347	1019.42993	1120.54797	1167.44664	1166.35172
595.126263	601.938585	945.413918	967.393434	904.681694
174.56055	175.61144	254.176857	209.445375	226.840227
1092.29239	1096.74315	971.761519	989.93464	943.08378
1909.85446	1925.099	1428.96989	1504.62552	1549.47951
11883.3752	12369.0096	12865.3786	13392.2942	13317.4861
83.9658341	99.402702	52.695202	73.2589202	59.835808
21184.4326	22135.8773	26594.0286	26136.5286	25265.0001
1270.53565	1212.71297	1081.8015	1154.2976	1144.02493
2526.34045	2577.84341	2843.99105	2797.92722	2839.07513
1965.83168	1943.87506	2227.14721	2085.06158	2177.30895
2101.35548	1848.89026	2278.29256	2266.33044	2251.43391
164.985499	150.208528	82.1425208	83.5903064	87.5210326
9483.72	9574.68915	11667.3377	11619.9918	11396.4887

3136.9341	3062.7077	3432.93743	3619.74203	3697.31709
1118.80791	1029.3702	1339.07808	1223.79965	1397.65731
8315.56375	8317.79721	7054.95763	7034.73478	6909.69622
6805.65182	6672.13026	5887.9139	6302.14557	6461.37419
4858.97024	4817.71763	5362.51173	5207.01864	5353.96521
198.129907	225.312791	110.039981	99.5569942	111.63397
3090.53193	3297.96076	3704.16273	3612.2283	3751.79447
2947.6427	2713.69377	420.011757	450.824124	501.013258
12661.1639	12729.0682	14545.4256	14531.5643	14185.5519
6791.65751	6770.42848	7989.52254	7457.38239	7202.62375
4688.0924	4998.85144	6081.64626	5764.91349	5495.96362
4752.17159	4869.62793	5900.31277	5947.12158	5724.58999
3709.96409	3609.42256	3863.79819	3812.2815	3951.84254
2004.13188	1927.30795	2320.13875	2385.61099	2309.48357
4005.3176	3870.07853	5074.23798	4998.51248	4990.485
2183.11169	2213.36683	2971.07948	2789.47427	2894.44558
11997.5392	12031.0404	10884.6589	10577.461	10388.2107
2791.49571	2680.55953	2416.23	2297.3246	2422.90369
2163.22504	2125.00887	2596.01363	2480.4719	2562.22288
72.1811556	104.925074	40.296331	43.2039786	39.2951575
3270.98482	3141.12538	3656.1171	3712.72451	3908.9751
3382.20272	3381.90082	3012.92567	3093.78055	3170.40475
92.804343	134.745885	137.937441	175.633565	168.790563
21268.3985	20898.8659	25231.7026	24070.2514	24597.8755
454.446664	452.834531	799.727183	791.759869	798.406154
1751.49784	1685.42804	2185.30103	2061.58115	2119.25929
469.177512	463.879276	547.100186	551.320336	531.377698
1828.83479	2042.17329	1494.06396	1441.69798	1404.80188
7792.61864	8263.67796	8938.03618	8739.4135	8503.82931
148.045023	130.327987	48.0456254	58.2314494	56.263521
1245.49321	1254.68299	1115.8984	1007.77976	1051.14546
53.7675955	72.8953148	43.3960487	40.3863278	37.509014
1020.11123	1066.92234	829.174502	786.124567	738.570346
2577.89842	2567.90314	3228.35605	3296.65141	3061.45
4160.72804	3942.97385	4782.86451	4626.58258	4745.78334
1554.84102	1539.63741	1934.22389	1780.75529	1712.01857
5124.12551	5139.11969	5664.73422	5707.62126	5774.60201
3815.28966	3854.61589	4545.7361	4445.31371	4519.83618
9143.43741	9518.36096	10797.8668	10728.6749	10939.236
4391.26582	4719.4194	5378.01032	5335.69136	5495.07055
2830.53246	3064.91665	3552.27656	3386.81624	3332.05074
953.085871	977.459903	1191.84148	1172.14272	1207.43302
11472.3845	11711.8473	13590.7125	13991.5146	13564.867

25738.4743	25217.361	32313.0078	31055.2077	29624.0834
1585.03925	1433.60786	1932.67403	1961.08494	1930.82115
8758.96227	8469.11021	9368.89695	8869.02543	9038.77929
208.4415	235.253061	144.136876	135.247237	154.501415
11733.1205	11674.2951	10725.0235	11104.3617	11094.6305
846.287223	905.669063	623.043271	634.910642	579.603573
6024.91687	6291.08656	6940.26808	6729.48927	6548.00215
62.6061044	50.8058255	1094.20037	1101.70145	1018.99488
2.94616962	1.10447447	46.4957665	62.927534	54.4773774
1026.74011	989.609122	810.576196	800.212821	758.217925
4003.10797	3942.97385	2589.81419	2527.43275	2579.19125
6861.62904	6632.36917	7894.98115	7538.15505	7491.979
4123.16438	4345.00255	5151.73093	5088.67731	4972.62357
5780.38479	5521.26786	6588.45011	6838.43844	6758.76709
33.1444082	34.2387085	6.19943553	9.39216926	9.82378937
227.591603	228.626215	111.58984	141.821756	115.206257
452.237036	426.327144	185.983066	154.031576	163.432132
3351.26794	3270.3489	3919.59311	3849.85018	3729.46767
77155.0265	74108.0278	66727.6243	67324.0085	69347.9153
3279.08678	3299.06523	3536.77797	3567.14589	3517.80967
1452.46162	1465.63762	1749.79068	1713.13167	1731.66615
2555.0656	2604.35079	2930.78315	2738.75656	2877.47721
2977.84094	2912.49917	3453.08559	3290.07689	3242.74356
4484.8067	4351.6294	4107.12604	3853.60705	4103.66474
1874.50042	1903.00951	2084.5602	2120.75182	2174.62974
969.289804	1118.83264	909.767164	880.04626	858.241962
2302.43156	2232.1429	2527.81984	2657.04468	2500.60093
109.744818	138.059308	26.347601	31.9333755	46.4397316
301.245843	352.327355	136.387582	148.396274	137.533051
3273.93099	3163.21487	3997.08606	3867.6953	3834.85014
123.739124	112.656396	182.883348	219.776761	203.620362
746.117456	793.012667	1233.68767	1147.72308	1209.21916
7027.35108	6876.45803	7764.793	7567.27077	7500.90972
3279.82333	3204.08043	3775.45624	3793.49716	3713.39238
4744.06963	5007.68723	5201.32641	5239.89123	5378.07815
3049.28555	3373.06502	3575.52444	3482.61636	3574.96626
11722.0724	11982.4435	12832.8316	13043.8447	12898.6354
1985.71832	2038.85987	1655.24929	1604.18251	1564.66173
1093.76547	1118.83264	931.465189	919.493371	907.360909
323.342116	352.327355	255.726716	285.521946	238.45016
1867.135	1994.68089	1610.30338	1540.31576	1562.87558
1367.75925	1352.98122	1106.59924	1081.03868	1111.87434
42.7194595	25.4029127	61.9943553	78.8942218	57.1565927

933.935769	1011.69861	858.621821	854.687403	904.681694
3872.73996	3958.43649	4556.58512	4742.10626	4697.55746
403.625238	366.685523	297.572906	257.345438	278.638389
2418.80526	2624.23133	2952.48117	2920.96464	2788.17004
1314.72819	1313.22014	702.086074	702.534261	670.696893
1682.26285	1675.48777	1018.25729	1080.09947	1075.2584
3517.72652	3990.46625	4849.50844	4692.32776	4536.80455
259.262926	323.611019	88.3419563	98.6177772	100.024037
10430.9135	10376.5376	5517.49762	5364.80708	5570.98165
4484.8067	4740.40441	5932.8598	5738.61542	5770.13665
3780.67216	3946.28727	4286.90967	4094.04658	4167.07284
18699.3386	18430.3654	12851.4299	12438.0498	11403.6333
81.7562069	92.7758552	35.6467543	34.7510263	41.081301
561.981855	540.088014	464.957665	458.33786	496.547899
3900.72857	3880.0188	4449.64485	4538.29619	4394.80614
3998.68871	3921.98883	4671.27467	4568.35113	4722.56347
14383.2001	14006.9452	17518.055	16653.2553	16390.546
23293.8901	22935.5168	25586.6203	24559.5834	25039.946
13292.3808	12898.0528	15143.6711	14658.3586	14917.8707
1937.10652	1941.66611	1793.18673	1784.51216	1728.09386
7594.48873	7477.29214	8606.36638	8379.69341	8432.38357
1439.9404	1505.3987	1734.29209	1826.77692	1859.37541
749.063625	855.967712	1057.00376	888.499212	819.839877
12728.1893	13613.7523	16560.2422	16049.3388	15835.9485
657.732367	577.640146	1216.63922	1205.01532	1121.69813
3112.6282	3397.36346	3578.62416	3764.38144	3646.412
2621.35442	2522.61968	3288.80055	3191.45911	3183.80083
4259.42473	4248.91327	5613.58887	5332.87371	5376.292
973.709059	939.907771	1123.64769	1096.06615	1106.51591
29139.0906	28074.6365	32274.2614	32419.8899	33454.4682
8422.36239	8654.66192	9987.29064	9858.96007	9847.00924
1982.77215	2108.44176	2279.84242	2235.33628	2159.44752
7235.79258	7165.83034	8772.20128	8659.58006	8244.8385
1613.76441	1557.309	2078.36076	2101.96748	2078.17799
2077.78612	2066.47173	1464.61664	1629.54137	1664.68576
3146.50915	3111.30457	2830.04232	2854.28024	2866.76035
306.40164	320.297595	379.715426	388.835807	343.832628
1095.23856	1239.22035	1521.96142	1460.48232	1479.81991
269.57452	300.417055	387.464721	380.382855	370.624781
588.497381	583.162519	816.775631	877.228609	871.638039
1470.87518	1504.29422	1769.93884	1668.98848	1754.88601
2175.74626	1978.11377	1729.64251	1679.31986	1789.71581
3410.19133	3470.25878	4122.62463	3921.23067	4057.22501

1315.46473	1218.23534	1074.05221	1069.76808	1067.22075
1896.59669	1806.92023	2183.75117	2105.72435	2033.5244
2226.56769	2281.84425	2678.15615	2644.83486	2543.46838
2088.83426	2081.93437	2554.16744	2513.34449	2390.7531
1042.2075	1112.20579	888.06914	814.301075	813.588374
1091.55584	1275.66801	1469.26622	1446.39407	1430.70096
1388.38243	1431.39891	1655.24929	1607.00016	1571.8063
5533.64308	5701.2972	6163.78878	5941.48627	6163.9813
1701.41295	1722.98017	1128.29727	1038.77392	1070.79304
5185.25853	5250.67162	5805.77137	5789.33313	5944.28564
3198.06712	3179.78199	3659.21682	3545.5439	3583.89698
3804.24152	3680.10892	4466.6933	4293.16057	4393.91306
4088.54689	4335.06228	4793.71352	4591.83155	4392.12692
3137.67064	3297.96076	3980.03761	3801.95012	3784.83812
1986.45487	1886.44239	1742.04138	1711.25324	1773.64052
1697.73024	1805.81575	2106.25822	2015.55952	2050.49276
578.92233	588.684891	1246.08654	1180.59568	1290.4887
3380.72964	3268.13995	3704.16273	3766.25987	3816.09564
6125.82318	6194.99729	8107.31182	8081.96165	8148.38675
81.7562069	101.611651	96.0912507	123.976634	134.853836
4491.43558	4388.07706	5088.18671	4840.72404	4865.45495
5647.80716	5380.9996	4971.9473	4822.87892	4769.0032
266.62835	330.237866	389.01458	418.890749	378.662427
1281.58378	1319.84699	1548.30902	1479.26666	1508.3982
17.6770177	23.1939638	49.5954842	58.2314494	52.6912339
1208.66609	1302.1754	1388.67356	1462.36075	1430.70096
5529.96037	5411.92489	6898.42189	6599.87734	6406.00374
1507.7023	1530.80161	1808.68532	1750.70035	1717.377
1183.62364	1229.28008	1069.40263	1032.1994	1033.28403
835.239087	757.669484	1157.74459	1221.92122	1118.12585
1042.94405	941.012246	824.524926	805.848123	759.110997
3033.81816	3221.75202	3716.5616	3727.75198	3508.87895
298.299674	297.103632	227.829256	213.202242	237.557088
135.523802	142.477206	80.5926619	79.8334387	97.344822
1093.76547	1100.05657	1387.1237	1341.20177	1311.92242
1135.01185	1075.75813	866.371115	890.377646	793.940796
33.8809506	40.8655553	12.3988711	5.63530156	10.7168611
3594.32693	3493.45274	4855.70788	4698.90228	4485.00638
3377.04692	3205.1849	4125.72435	4151.33881	4020.60907
1473.82135	1500.9808	1291.03245	1277.33502	1283.34412
10738.7883	10988.4165	11867.2695	11305.3541	11398.2749
3959.65197	3868.97406	4872.75633	4829.45343	4529.65997
50.8214259	33.134234	27.8974599	13.149037	15.1822199

7424.34744	7320.45677	9179.81416	8952.61574	8794.07763
2778.23795	2703.7535	2075.26104	2067.21645	2048.70662
3289.39838	3317.8413	1986.91909	1870.92012	1910.2805
190.02794	175.61144	88.3419563	100.496211	100.917109
539.885582	606.356482	433.960487	450.824124	484.937966
36046.3853	35354.2277	42690.8629	42173.6576	41693.0552
2697.21829	3102.46878	3397.29067	3227.14936	3174.87011
718.865387	690.296542	587.396517	527.839912	542.987631
5635.28594	5453.89492	6627.19658	6369.76919	6454.22962
1251.38555	1175.16083	929.91533	977.72482	966.303646
1014.21889	1052.56417	1312.73047	1297.05858	1266.37576
1933.42381	1855.5171	2428.62887	2215.61273	2362.17481
240.112824	246.297806	156.535747	143.70019	162.539061
517.78931	501.431408	607.544682	607.673351	582.282788
875.748919	855.967712	571.897928	629.27534	658.193888
561.245312	568.804351	694.336779	699.71661	710.885122
1308.83585	1278.98143	1540.55973	1455.78624	1427.12867
2149.23074	2134.94915	2414.68014	2528.37197	2436.29976
3325.48896	3209.6028	2634.7601	2641.078	2524.71387
2792.9688	2726.94746	3132.2648	2845.82729	3040.01628
1177.73131	1034.89258	1387.1237	1308.32918	1284.23719
1287.47612	1283.39933	1346.82737	1434.18425	1424.44946
683.511351	671.520476	522.302443	556.955637	584.962004
413.936831	436.267414	354.917684	292.096464	325.078121
2322.3182	2214.47131	1963.6712	1929.15157	1921.89043
1168.8928	1134.29528	1342.17779	1405.06852	1392.29888
968.553262	917.818282	791.977889	761.704927	756.431782
111.217903	117.074294	55.7949198	65.7451848	75.0180279
2222.14843	2189.06839	1923.37487	1821.14162	2014.76989
1297.78772	1272.35459	948.513636	942.034577	879.675685
1249.91246	1211.60849	1394.87299	1457.66467	1457.49311
175.297092	209.850149	151.886171	132.429587	150.929128
550.197176	557.759606	784.228595	652.755764	655.514673
359.432693	316.984172	297.572906	241.37875	260.776954
1548.21213	1495.45843	1405.72201	1289.54484	1267.26883
72.1811556	68.477417	46.4957665	50.717714	38.4020857
598.808975	619.610176	441.709782	465.851595	489.403325
1297.05117	1284.50381	1790.08701	1675.563	1578.95087
549.460634	579.849095	810.576196	872.532524	763.576356
184.872144	188.865134	151.886171	139.943322	165.218276
2082.20538	1900.80056	2448.77704	2383.73256	2404.14918
2235.4062	2277.42635	2899.78597	2888.09205	2679.21528
697.505657	775.341076	616.843835	631.153774	611.754156

253.370587	227.52174	184.433207	176.572782	183.972783
523.68165	501.431408	396.763874	431.100569	417.064512
816.825527	818.41558	568.79821	637.728293	667.124606
1207.193	1197.25032	1602.55409	1442.6372	1454.8139
4491.43558	4597.92721	5433.80524	5215.47159	5108.37047
4863.3895	4782.37444	6386.96846	6381.97901	6004.12145
237.166654	229.730689	292.923329	308.063152	276.852246
1165.94663	1169.63846	1492.5141	1433.24503	1435.16632
1465.71939	1506.50317	1794.73659	1725.34149	1656.64812
1507.7023	1443.54813	1717.24364	1611.69625	1633.42825
3980.27515	3973.89913	4555.03526	4417.1372	4416.23986
9320.20759	8927.46712	10813.3654	10560.5551	10444.4743
1322.09362	1306.59329	1650.59971	1592.91191	1596.81231
1283.79341	1301.07092	1687.79632	1747.8827	1621.81832
1376.59775	1436.92128	1322.02963	1147.72308	1209.21916
379.319338	334.655764	416.912039	437.675088	418.850656
446.344697	471.610597	241.777986	281.765078	267.028457
5812.05611	6176.22122	7433.1232	7238.54485	6938.27451
978.128313	1054.77312	1297.23189	1254.79381	1325.31849
1188.0429	1238.11588	979.510814	994.630725	1004.70573
1555.57756	1547.36873	1278.63358	1301.75466	1352.11065
943.51082	917.818282	1035.30573	1040.65235	1033.28403
1996.76646	1895.27819	2405.38099	2466.38365	2534.53766
514.106598	540.088014	358.017402	374.747553	416.171441
1504.75613	1451.27945	1238.33725	1195.62315	1167.24479
1238.86432	1255.78747	974.861237	1002.14446	940.404564
39.7732898	46.3879276	13.9487299	13.149037	21.4337223
3093.4781	2989.81238	2563.46659	2477.65425	2363.96095
815.352442	772.027652	605.994823	659.330282	611.754156
2731.09924	2609.87317	2375.93367	2425.0581	2388.07389
1019.37469	942.11672	1123.64769	1038.77392	1095.79905
425.72151	438.476363	371.966132	356.902432	326.864265
1486.34257	1405.996	1774.58842	1704.67872	1743.27608
626.061044	663.789155	798.177325	782.367699	771.614002
408.044492	395.401859	286.723893	334.361226	320.612762
6043.33043	6352.93713	5618.23845	5654.08589	5796.03573
740.961659	801.848463	906.667446	977.72482	921.650057
4036.98892	3955.12307	3556.92614	3595.32239	3589.25541
723.284641	744.415791	623.043271	596.402748	642.118596
503.058462	530.147744	399.863592	359.720083	358.121776
409.517577	402.028706	464.957665	500.602622	485.831038
430.140764	453.939006	382.815144	354.084781	339.367269
1028.2132	1112.20579	1269.33443	1254.79381	1051.14546

1792.00767	1697.57726	2101.60865	2016.49874	2060.31655
2549.17326	2544.70917	2981.92849	2964.16862	2868.5465

Gene	baseMean	log2FoldChai	lfcSE	stat	pvalue	padj
44263	3066.70416	0.29698181	0.06655816	4.46198925	8.12E-06	7.43E-05
AACS	3825.18104	-0.1623376	0.06223424	-2.6084932	0.00909418	0.03479957
ABCC5	6472.91843	0.71105581	0.05633982	12.6208402	1.62E-36	2.82E-34
ABCD3	2891.30914	-0.2319528	0.06888823	-3.3670897	0.00075966	0.00416266
ABHD11	3282.70594	0.35833353	0.06586494	5.44042894	5.32E-08	7.26E-07
ABHD12	4360.00334	0.18424425	0.06600137	2.79152147	0.00524609	0.02182451
ABHD14B__PCBP4	6397.48197	0.1709623	0.05546616	3.08228094	0.00205421	0.0097448
ABI2	3348.65508	-0.1989159	0.06437473	-3.0899693	0.00200177	0.00951768
ABTB2	1265.87538	0.24211516	0.09135467	2.65027683	0.00804258	0.03142346
AC002310.11__AC00:	6178.33488	0.22304079	0.0559912	3.98349736	6.79E-05	0.00049295
AC009948.5	329.748435	-0.4391235	0.16566559	-2.650662	0.00803342	0.03139499
AC010646.3__NR2F6	4216.09333	0.64106072	0.08407324	7.62502699	2.44E-14	8.01E-13
AC010761.8	1866.61832	-0.4808309	0.08132926	-5.912151	3.38E-09	5.60E-08
AC113189.5	351.778606	0.56251484	0.16387726	3.43253756	0.00059796	0.00338443
AC234582.1	2838.40415	2.26204717	0.0990551	22.8362504	2.00E-115	6.69E-112
AC234582.2	489.191205	2.28619179	0.16844478	13.5723518	5.84E-42	1.39E-39
AC240274.1	608.766864	0.42491849	0.13441061	3.16134641	0.00157042	0.00774451
ACAD10__ALDH2__R	5564.24735	0.18425803	0.05676154	3.24617715	0.00116966	0.00605981
ACAP3	1915.24862	0.35473475	0.07918671	4.47972563	7.47E-06	6.90E-05
ACO2	5287.69713	-0.1926349	0.05727218	-3.3634988	0.00076961	0.00419818
ACTA2	924.384554	-0.8377288	0.10556059	-7.9359997	2.09E-15	8.04E-14
ACTL6A	10055.5831	-0.1359547	0.05514207	-2.4655341	0.01368091	0.04876252
ACTN1	13513.2566	-0.1444207	0.05008258	-2.8836513	0.00393094	0.01705414
ACVR1B	3947.70224	0.34292107	0.06210834	5.52133676	3.36E-08	4.74E-07
ACVR2B	815.144689	-0.4409811	0.11162401	-3.950594	7.80E-05	0.00055718
ADAM15__DCST1	22268.9823	0.16160333	0.0450603	3.58637937	0.0003353	0.00202478
ADAR	23035.9693	-0.2720806	0.05194377	-5.2379832	1.62E-07	2.03E-06
ADCY9	4513.26285	-0.3465038	0.06454897	-5.3680757	7.96E-08	1.06E-06
ADGRG1	11190.3478	0.1623711	0.05452182	2.97809407	0.00290047	0.01309012
ADI1	3663.78522	-0.4475319	0.07140616	-6.2674125	3.67E-10	7.07E-09
ADSS	4241.17119	0.3416548	0.0642164	5.32036691	1.04E-07	1.35E-06
AF011889.5__IDS	2935.03931	-0.5601668	0.06816315	-8.21803	2.07E-16	8.81E-15
AGL	2443.9694	-0.3760025	0.07960485	-4.7233622	2.32E-06	2.35E-05
AGR2	47585.7535	-0.1672557	0.05102265	-3.2780681	0.0010452	0.00547616
AGR3	4080.11002	-0.3181234	0.07282707	-4.3682024	1.25E-05	0.00010932
AHCY	17814.0231	-0.1308123	0.04596492	-2.8459165	0.00442838	0.01887883
AHCYL1	9550.64186	-0.2730264	0.05136749	-5.3151596	1.07E-07	1.38E-06
AK2	5573.94624	-0.3892604	0.05786018	-6.7276052	1.72E-11	3.92E-10
AK4	4782.73102	-0.2723356	0.06010773	-4.5307914	5.88E-06	5.56E-05
AKAP9	5218.86262	0.38068824	0.0673526	5.65216839	1.58E-08	2.34E-07
AL139099.2__RN7SL	1332.13602	-0.6835192	0.09315806	-7.3371989	2.18E-13	6.47E-12
ALCAM	12206.3888	0.42897176	0.05113345	8.38925884	4.89E-17	2.26E-15

ALDH16A1__CTD-314	45972.4926	-0.2488891	0.04855587	-5.1258298	2.96E-07	3.55E-06
ALDH5A1	2919.91718	-0.2960629	0.06711425	-4.4113269	1.03E-05	9.17E-05
ALDH6A1	7538.2849	-0.1615424	0.05731659	-2.8184232	0.00482601	0.02033459
ALDH7A1	5993.5263	-0.209943	0.05999361	-3.4994233	0.00046627	0.00270982
ALDOA	63023.2174	-0.3125555	0.0425212	-7.3505812	1.97E-13	5.91E-12
ALDOC	3008.71952	-0.5258111	0.06749384	-7.7905045	6.67E-15	2.40E-13
ALG10B	2347.92843	-0.6232367	0.0791386	-7.8752558	3.40E-15	1.26E-13
ALG9__FDXACB1__R	2789.47029	-0.2712446	0.07487354	-3.622703	0.00029154	0.00178898
ALOX15	385.378245	0.43079663	0.15333525	2.80950809	0.00496173	0.02082828
AMZ1	4338.76152	-0.4103939	0.0827111	-4.9617759	6.99E-07	7.84E-06
ANAPC7__ARPC3	17729.9216	0.16398816	0.05055198	3.24395123	0.00117884	0.0060998
ANGPTL4	1079.90163	-0.2968703	0.09873351	-3.0067833	0.00264028	0.01206913
ANKFN1	620.986616	-0.3985951	0.12670621	-3.145821	0.00165621	0.00810065
ANKRD22	1226.34335	0.7379181	0.09544462	7.73137448	1.06E-14	3.65E-13
ANKRD40	3130.63299	0.36593088	0.06621964	5.52601741	3.28E-08	4.63E-07
ANKRD9	2584.03954	0.44590446	0.08475651	5.26100568	1.43E-07	1.81E-06
ANKS3__ZNF500	1887.27897	0.23242311	0.08349465	2.78368861	0.00537446	0.02229747
ANLN	16195.2191	-0.2398987	0.050953	-4.7082342	2.50E-06	2.52E-05
ANP32E	6949.43079	-0.4472161	0.0576739	-7.75422	8.89E-15	3.11E-13
ANXA3	703.011478	0.76751193	0.12283253	6.24844208	4.15E-10	7.94E-09
AP000648.5	489.515566	-0.603526	0.14280165	-4.2263233	2.38E-05	0.00019364
AP000769.1	12807.8851	16.9650257	1.44374864	11.7506782	7.01E-32	1.00E-29
AP000769.7	40.8203322	8.67411069	1.50528795	5.76242616	8.29E-09	1.29E-07
AP001610.5	86.9898123	-1.3747659	0.32323279	-4.2531758	2.11E-05	0.00017445
AP1B1	8237.53566	-0.1791982	0.05689002	-3.1499053	0.00163323	0.00800232
AP1G1	10364.5164	-0.1334857	0.05268741	-2.5335412	0.01129165	0.04162533
AP1S2	3175.94066	-0.5833356	0.06546304	-8.9109146	5.06E-19	2.95E-17
AP3M1	8852.06314	-0.2632767	0.05080819	-5.1817773	2.20E-07	2.69E-06
AP5Z1	2073.2039	0.32565818	0.07645125	4.25968441	2.05E-05	0.00016972
APAF1	2286.53585	-0.3429312	0.07804903	-4.3937922	1.11E-05	9.87E-05
APBB2	4877.96953	-0.7352582	0.05961172	-12.334121	5.93E-35	9.44E-33
APC2	221.242341	0.60758084	0.20069026	3.02745558	0.00246622	0.01134801
API5	10346.7775	-0.1994805	0.0508301	-3.9244562	8.69E-05	0.00061392
APOC1__APOE	10349.7886	0.74916326	0.14883336	5.03357101	4.81E-07	5.57E-06
ARAP2	1612.64288	0.2866399	0.09274531	3.09061344	0.00199743	0.00949976
ARFGAP1	4351.59474	0.20212336	0.06096601	3.31534512	0.0009153	0.00488131
ARHGAP19__ARHGA	2372.35604	-0.5668576	0.07246849	-7.8221255	5.19E-15	1.88E-13
ARHGAP27	3389.40493	0.22572922	0.06426703	3.51236404	0.00044414	0.00259841
ARHGAP39__C8orf82	4012.81754	0.59435232	0.0727241	8.17270144	3.02E-16	1.27E-14
ARHGEF18__CTB-13:	2463.03157	0.23388104	0.07343889	3.18470296	0.00144903	0.0072486
ARHGEF38	1294.13354	0.30475255	0.09326381	3.26763981	0.00108448	0.00565713
ARL15	910.611011	0.34387237	0.10648722	3.22923609	0.00124121	0.00637383
ARL3	4068.3182	-0.5409713	0.06540352	-8.2712874	1.33E-16	5.85E-15

ARL4D	1844.05768	0.43908543	0.08550894	5.13496494	2.82E-07	3.41E-06
ARL6IP1__RP11-1035	66278.3975	-0.2384285	0.04478411	-5.3239531	1.02E-07	1.32E-06
ARMT1	5733.48531	-0.5292426	0.05574961	-9.4932067	2.24E-21	1.58E-19
ARRDC1	9676.6838	0.53233678	0.05024224	10.5954028	3.13E-26	3.21E-24
ARSJ	234.282279	0.5097969	0.19555338	2.60694491	0.00913541	0.03494131
ASAP1	3987.02491	0.24149535	0.0665075	3.63109938	0.00028222	0.00173751
ATAD2	13678.4523	-0.5335974	0.0515732	-10.346409	4.34E-25	4.08E-23
ATAD5	1202.50464	-0.7367503	0.09512837	-7.7448006	9.57E-15	3.31E-13
ATF2	2250.44117	-0.2255185	0.07388646	-3.05223	0.00227148	0.01062165
ATF4	9053.21175	0.24020351	0.05349432	4.49026182	7.11E-06	6.61E-05
ATM	4989.74468	-0.5459045	0.06803924	-8.0233768	1.03E-15	4.10E-14
ATP13A2	3782.63776	-0.2093178	0.06691985	-3.1278886	0.00176067	0.00856141
ATP5A1	25316.6064	-0.2141578	0.04816216	-4.446599	8.72E-06	7.93E-05
ATP5G1	3775.92075	-0.2976339	0.07255469	-4.1022001	4.09E-05	0.00031399
ATP5G3	8563.71816	-0.1882128	0.05662904	-3.3236095	0.00088861	0.00474804
ATP6V1D	4432.61173	0.19074862	0.05993945	3.18235547	0.00146082	0.00729233
AVL9	5181.82743	-0.2448552	0.05919798	-4.1362088	3.53E-05	0.00027572
AZIN1	15478.1517	-0.2252813	0.04754241	-4.7385321	2.15E-06	2.20E-05
B3GALT6	1203.45359	0.29836823	0.09280068	3.21515143	0.00130376	0.00664081
B4GALNT4	1985.77913	0.33393537	0.07998089	4.17518954	2.98E-05	0.00023693
BAMBI	5451.11577	0.21971283	0.06521434	3.36908781	0.00075417	0.00413829
BBX	6394.06304	-0.3700129	0.0656315	-5.6377331	1.72E-08	2.53E-07
BCAR1	4497.30866	0.32264092	0.06348535	5.08213172	3.73E-07	4.43E-06
BCAS1	2144.96533	1.50858375	0.07937467	19.0058592	1.53E-80	1.59E-77
BCKDHA__CTC-435M	1845.61464	-0.382139	0.08888331	-4.2993331	1.71E-05	0.00014444
BCL2	150.866861	-0.9372814	0.24542993	-3.818937	0.00013403	0.0009016
BCL3	1205.51775	0.3692314	0.09958952	3.7075328	0.00020929	0.00133527
BCL6	6535.94175	-0.6376312	0.05441869	-11.717135	1.04E-31	1.46E-29
BCL7A	2492.39781	-0.5931604	0.07156141	-8.2888295	1.14E-16	5.12E-15
BCL7B	2381.5199	0.21408168	0.07407346	2.89012671	0.00385087	0.01676773
BDKRB1__BDKRB2__	558.164539	0.68928187	0.13105344	5.25954801	1.44E-07	1.82E-06
BEST1	265.654665	1.22513487	0.18759551	6.53072582	6.55E-11	1.38E-09
BHLHE40	10509.5055	-0.438646	0.04926677	-8.9034871	5.41E-19	3.13E-17
BIVM__BIVM-ERCC5	4531.6154	-0.1835825	0.05936674	-3.0923462	0.00198581	0.00946672
BLM	1019.28192	-0.6917978	0.09985777	-6.9278315	4.27E-12	1.07E-10
BLOC1S5__BLOC1S5-	16550.8008	-0.2582605	0.04643481	-5.5617864	2.67E-08	3.80E-07
BLVRA	5111.36114	0.38894635	0.0612958	6.34539925	2.22E-10	4.42E-09
BMP4	1613.81554	-0.4443342	0.08311163	-5.3462339	8.98E-08	1.18E-06
BMS1	6790.73423	-0.1815758	0.05447699	-3.3330735	0.00085892	0.00460121
BNIP3	2673.57791	-0.6093293	0.07641729	-7.9737097	1.54E-15	6.04E-14
BNIP3L	5425.87975	-0.2044803	0.05707039	-3.5829488	0.00033974	0.00204786
BNIPL	4043.17227	0.31574898	0.06405615	4.92925355	8.25E-07	9.17E-06
BRCA2	1084.71984	-0.736898	0.11161704	-6.6020211	4.06E-11	8.78E-10

BRI3BP	6279.66528	-0.2849509	0.0567593	-5.0203382	5.16E-07	5.94E-06
BRICD5__PGP	2373.73731	0.29461728	0.08401735	3.50662409	0.00045383	0.00264676
BRK1	8207.3189	0.21282655	0.06097911	3.49015524	0.00048274	0.00279215
BRPF3	3908.84265	0.31935909	0.06682448	4.7790735	1.76E-06	1.83E-05
BSDC1	3758.81547	0.20330316	0.0649154	3.13181725	0.00173728	0.00846493
BTBD3	2043.68799	-0.2312824	0.07988473	-2.8952014	0.00378915	0.01654214
BTF3	22577.1128	-0.2084621	0.05591184	-3.7284066	0.00019269	0.00123744
BTG2	1131.41989	0.50628493	0.09858581	5.13547464	2.81E-07	3.41E-06
BUB3	7027.50927	-0.1619268	0.05591388	-2.896003	0.00377949	0.01650426
C14orf132	5060.16482	0.67052216	0.05956931	11.2561684	2.16E-29	2.65E-27
C14orf79	1790.3493	0.27822296	0.08069285	3.44792599	0.00056491	0.00321269
C19orf33__CTB-102L	22857.2451	0.23035239	0.05156777	4.46698363	7.93E-06	7.27E-05
C1orf204__CFAP45__	485.440781	0.41375142	0.144355	2.86620781	0.00415421	0.01786045
C1orf21	867.442886	-0.8029777	0.10675021	-7.5220241	5.39E-14	1.72E-12
C20orf96	620.286867	0.56694316	0.1238986	4.57586402	4.74E-06	4.56E-05
C2orf54	209.551653	1.35813481	0.21177455	6.41311628	1.43E-10	2.90E-09
C4orf46	799.234228	-0.6817553	0.11167619	-6.1047508	1.03E-09	1.87E-08
C4orf48	259.736074	1.55890881	0.47578958	3.27646691	0.00105115	0.00550213
C5orf34	779.677762	-0.6641191	0.11346179	-5.8532407	4.82E-09	7.73E-08
C5orf38	993.562219	0.79800036	0.1125267	7.09165356	1.33E-12	3.52E-11
C6orf132	4255.67349	0.40295691	0.06085193	6.62192492	3.55E-11	7.75E-10
C6orf89	7030.3952	-0.2056405	0.05469403	-3.7598354	0.00017003	0.00110719
C7orf50	6080.96824	0.21298952	0.06162386	3.4562833	0.00054768	0.00312641
CA11	536.01754	-0.3649282	0.1336259	-2.7309694	0.00631483	0.02558871
CA12	57219.7933	-0.5257711	0.04323521	-12.160717	5.03E-34	7.78E-32
CAB39	2349.00116	0.25462898	0.0726524	3.50475688	0.00045702	0.00266353
CABLES1	2951.68204	-0.19283	0.06855418	-2.8128117	0.00491104	0.02065184
CACUL1	3990.74558	0.33994587	0.06388657	5.32108524	1.03E-07	1.34E-06
CADM1	5275.57945	-0.7809909	0.05704179	-13.691558	1.14E-42	2.76E-40
CADM4	912.684202	0.34344925	0.10443457	3.28865473	0.00100667	0.00529924
CADPS2	1017.86964	0.43506935	0.1050244	4.1425551	3.43E-05	0.00026895
CAMK1D	601.513072	0.72768964	0.12684304	5.73693015	9.64E-09	1.48E-07
CAPN2	14934.3532	0.21073203	0.04678082	4.50466707	6.65E-06	6.22E-05
CARD14	2470.55426	0.32753788	0.07416791	4.41616701	1.00E-05	9.00E-05
CARD9__DNLZ	728.893712	0.88798776	0.12974919	6.8438788	7.71E-12	1.85E-10
CASC4	6784.38638	-0.2518915	0.05689641	-4.4271943	9.55E-06	8.60E-05
CASP8AP2	2222.05948	-0.3267586	0.07907322	-4.1323542	3.59E-05	0.00027999
CATSPERB__TC2N	5817.5501	0.41723189	0.05582	7.4745945	7.74E-14	2.43E-12
CBX3	22447.3853	-0.1269722	0.05144151	-2.4682822	0.01357632	0.04847485
CBX4	3562.97097	0.52365911	0.06683094	7.83557901	4.67E-15	1.71E-13
CBX5	24654.1066	-0.4130138	0.05607406	-7.365506	1.76E-13	5.35E-12
CCBL1	336.26485	-0.5937057	0.16389879	-3.6223923	0.00029189	0.00178981
CCDC106__ZNF580__	2422.67835	0.21308331	0.08076691	2.63825011	0.00833351	0.03240098

CCDC125	3055.11525	-0.5872481	0.06656053	-8.8227668	1.12E-18	6.20E-17
CCDC129	48.740698	1.24752566	0.42925687	2.90624506	0.00365795	0.0160617
CCDC183__RABL6__F	13913.0006	0.28653484	0.04842669	5.91687915	3.28E-09	5.46E-08
CCDC30__PPCS	2049.25903	0.30088759	0.08502984	3.53861175	0.00040224	0.00238078
CCDC6	14218.9802	0.42500973	0.05163354	8.23127185	1.85E-16	7.97E-15
CCDC64	2110.23401	0.68475612	0.07619882	8.98643954	2.55E-19	1.50E-17
CCDC78	489.885767	0.59050193	0.13767667	4.2890487	1.79E-05	0.00015031
CCDC80	191.056666	-0.9980728	0.21579928	-4.625005	3.75E-06	3.69E-05
CCDC82	365.21503	-0.9419682	0.1596576	-5.8999273	3.64E-09	6.00E-08
CCDC85C	7745.04278	0.13803416	0.05255459	2.62649085	0.00862703	0.03337904
CCDC90B	3027.83517	0.42145528	0.06663865	6.32448731	2.54E-10	5.02E-09
CCNC	2642.02324	-0.2575201	0.07207683	-3.5728554	0.00035311	0.00211777
CCNG1	5386.79536	-1.1068761	0.05949094	-18.605794	2.88E-77	2.68E-74
CCNJL	865.323553	0.39333893	0.10941027	3.59508232	0.00032429	0.00196967
CCNL1	6496.79392	-0.3555658	0.05784055	-6.1473446	7.88E-10	1.45E-08
CCNT1	5089.13211	-0.2411894	0.06607534	-3.6502184	0.00026202	0.00162876
CCNY	4252.82167	-1.0594569	0.06097117	-17.376358	1.25E-67	8.33E-65
CCSAP	1470.28608	-0.5734872	0.08903222	-6.4413444	1.18E-10	2.44E-09
CCT2	18734.3512	-0.1502356	0.04751767	-3.1616792	0.00156862	0.00774253
CCT4	10378.9063	-0.2061898	0.05292251	-3.8960689	9.78E-05	0.00068155
CCT8	16256.5291	-0.1240288	0.04883494	-2.539756	0.01109298	0.04100157
CD109	230.276873	-0.8853356	0.20070387	-4.4111538	1.03E-05	9.17E-05
CD2BP2	4789.87584	-0.1687907	0.05832073	-2.8941799	0.0038015	0.0165744
CD34	129.551746	-0.7901127	0.2592433	-3.0477652	0.0023055	0.01075063
CD63	16946.7912	-0.1961975	0.05931182	-3.3078988	0.00093999	0.00499258
CDC123	5690.21086	-0.1525345	0.05948982	-2.5640437	0.01034605	0.03878144
CDC23	4034.93103	-0.1667688	0.06165685	-2.7047889	0.00683478	0.02735714
CDC42BPG	5085.27668	0.45211864	0.06037327	7.48872252	6.95E-14	2.20E-12
CDC42EP1	3046.47725	0.56823809	0.07091083	8.01341792	1.12E-15	4.42E-14
CDC6	3768.36348	-0.5344424	0.06211695	-8.6038103	7.71E-18	3.91E-16
CDCA7L	1920.20619	-0.3634651	0.07844511	-4.6333687	3.60E-06	3.56E-05
CDH19	372.471524	0.5184474	0.15716182	3.29881269	0.00097095	0.00514032
CDK6	369.79078	0.7152359	0.16762624	4.26684927	1.98E-05	0.00016467
CDKN1B	2498.00005	0.29242385	0.07323336	3.99304134	6.52E-05	0.00047704
CDR2L	2834.77121	0.5595785	0.06882735	8.13017591	4.29E-16	1.79E-14
CDV3	39776.0797	-0.3827407	0.04432371	-8.6351239	5.87E-18	3.02E-16
CEBPA	1035.77876	1.03084342	0.11791594	8.74218868	2.29E-18	1.24E-16
CEBPB	2382.71981	0.79126593	0.25973048	3.0464885	0.00231531	0.01077834
CEBPD	555.347547	0.56423047	0.16030706	3.51968562	0.00043206	0.00253573
CELSR2	5291.40307	-0.4373042	0.07614263	-5.7432249	9.29E-09	1.43E-07
CENPK	1575.03697	-0.7863283	0.08409516	-9.3504589	8.73E-21	5.83E-19
CENPQ	806.690183	-0.3756628	0.11069748	-3.3935982	0.00068981	0.00383679
CEP126	1643.76577	0.66120881	0.09383373	7.04660056	1.83E-12	4.82E-11

CEP135	1301.63428	-0.416377	0.10034262	-4.1495529	3.33E-05	0.00026221
CEP19	501.743493	0.46504891	0.13874947	3.35171674	0.00080312	0.00435537
CEP250	4980.6191	-0.2728391	0.06430485	-4.2429011	2.21E-05	0.00018182
CEP295NL__TIMP2	9183.4085	0.31092906	0.05308408	5.85729368	4.70E-09	7.57E-08
CEP55	5386.44977	-0.4129485	0.05671675	-7.2808909	3.32E-13	9.52E-12
CEP68	738.443431	-0.4929845	0.1153346	-4.2743855	1.92E-05	0.00015959
CETN3	1534.79627	-0.2647376	0.09471283	-2.7951606	0.00518739	0.02162342
CFAP157__TTC16	867.430923	0.28839611	0.11144971	2.58767943	0.00966249	0.03659855
CFAP73	63.6138876	1.32854913	0.39810358	3.33719462	0.00084629	0.00454811
CFAP97	2516.74589	-0.2043689	0.07389985	-2.7654849	0.00568382	0.02337572
CFL2	2362.72045	-0.3999467	0.07275765	-5.496971	3.86E-08	5.39E-07
CGGBP1	4773.13827	-0.1516903	0.05832939	-2.6005809	0.00930661	0.03545
CHCHD10	1890.76052	0.52423378	0.09951474	5.26790075	1.38E-07	1.75E-06
CHEK2	1373.84281	-0.6531593	0.0885133	-7.3792218	1.59E-13	4.85E-12
CHMP2A	3919.35696	-0.254406	0.06557514	-3.8796099	0.00010462	0.00072272
CHMP4B	9697.33612	0.22286864	0.05481358	4.06593811	4.78E-05	0.00036059
CHST1	229.979792	2.07431521	0.21890866	9.47571092	2.65E-21	1.85E-19
CISH	2690.19048	0.34053206	0.07148097	4.76395422	1.90E-06	1.96E-05
CITED2	13581.8096	-0.4426147	0.05294214	-8.3603492	6.25E-17	2.86E-15
CITED4	964.56959	0.60199192	0.14030338	4.29064449	1.78E-05	0.00014953
CLASP1	2130.38401	-0.2083184	0.08248245	-2.5256089	0.0115498	0.04242715
CLDN3	9871.2085	0.55689271	0.06757806	8.24073249	1.71E-16	7.41E-15
CLDN4	16764.0614	0.72539475	0.05090486	14.2500083	4.48E-46	1.31E-43
CLIC3	4484.97791	0.23304966	0.0775377	3.00563024	0.00265031	0.01210591
CLIP1	6471.34856	0.16855106	0.06466597	2.60648772	0.00914761	0.03497998
CLSTN2	3632.87544	-0.2617352	0.07510504	-3.4849211	0.00049228	0.00284027
CLSTN3	2050.35611	0.21898904	0.07866502	2.78381721	0.00537233	0.02229417
CMTM7	820.055447	-0.6752748	0.11253428	-6.0006145	1.97E-09	3.42E-08
CNIH1	6796.42402	-0.1709261	0.05889502	-2.9022166	0.00370532	0.01623232
CNIH2	572.429138	0.44985478	0.1373811	3.27450267	0.00105848	0.00553532
CNNM4	4330.38446	0.16710914	0.0636783	2.62427128	0.00868346	0.03353522
CNOT1	12362.8808	-0.1591147	0.05711407	-2.7859117	0.00533774	0.02215613
CNOT6	6691.34341	-0.2452299	0.0567702	-4.3196933	1.56E-05	0.00013268
CNOT6L	1724.52302	-0.480142	0.0873391	-5.4974459	3.85E-08	5.38E-07
CNTRL	2089.94193	-0.3554442	0.08129877	-4.372073	1.23E-05	0.00010763
COA3	2967.52245	0.30860338	0.07314461	4.21908583	2.45E-05	0.00019957
COL18A1	2852.60948	0.86486079	0.06880965	12.5688888	3.13E-36	5.34E-34
COL6A1	1026.98309	0.593825	0.09963657	5.95991009	2.52E-09	4.29E-08
COMTD1	1096.1457	0.2898944	0.10457486	2.77212331	0.00556919	0.02299685
COPA	23950.1201	-0.2569005	0.04748909	-5.4096741	6.31E-08	8.50E-07
COPG2__TSGA13	2387.40589	-0.4834984	0.07226951	-6.690213	2.23E-11	5.00E-10
COPS6	4657.49109	-0.2116474	0.06628791	-3.1928501	0.00140876	0.00708111
COQ10B	1324.22645	0.24672793	0.09049661	2.72637747	0.00640337	0.02589095

CORO2A	975.25115	0.29583455	0.10181481	2.90561415	0.00366533	0.01608566
COX15	7019.64969	-0.2824114	0.05371324	-5.2577627	1.46E-07	1.84E-06
CP	1156.98001	-0.2335279	0.0942847	-2.4768374	0.01325523	0.0474806
CPAMD8	8083.21961	0.51468373	0.0578481	8.89715958	5.73E-19	3.29E-17
CPD	16711.8419	-0.2095356	0.05172396	-4.0510366	5.10E-05	0.00038176
CPE	6846.00384	0.40899177	0.05485337	7.45609197	8.91E-14	2.78E-12
CPEB1__RP11-152F1	27531.1623	-0.426059	0.05937254	-7.176029	7.18E-13	1.97E-11
CPN2	18.6894809	-2.055226	0.71796032	-2.8625899	0.00420194	0.01804706
CPNE3	13191.4849	-0.2366186	0.05047758	-4.6875973	2.76E-06	2.77E-05
CPSF2	7655.93758	-0.3363393	0.05439347	-6.18345	6.27E-10	1.17E-08
CRACR2B	3261.63669	0.70797799	0.07078623	10.0016337	1.50E-23	1.24E-21
CRIM1	11761.0538	-0.5062256	0.0541253	-9.3528466	8.53E-21	5.72E-19
CRIP2	10671.4291	0.21491164	0.04938693	4.35158932	1.35E-05	0.00011667
CRTAP	10547.7999	0.16681057	0.05062781	3.29484051	0.00098478	0.00520035
CRY2	2176.86552	0.30635503	0.07540959	4.06254722	4.85E-05	0.00036505
CSE1L	21974.5081	-0.4291143	0.04523132	-9.4871066	2.38E-21	1.67E-19
CSNK2A2	2588.39602	-0.4853117	0.06995981	-6.9370072	4.00E-12	1.01E-10
CSRNP2	2076.41313	0.30859179	0.08580214	3.59655126	0.00032246	0.0019593
CTC-432M15.3__FNII	5344.18796	-0.1662648	0.06397762	-2.5987961	0.00935513	0.03561048
CTC-490E21.12__EGL	7522.309	0.23556695	0.05452547	4.32030997	1.56E-05	0.00013247
CTD-2008P7.9	201.008365	0.95667643	0.21311599	4.48899412	7.16E-06	6.64E-05
CTD-2031P19.5	2341.76232	-0.2604411	0.09125281	-2.8540617	0.00431641	0.01847695
CTD-2349B8.1__ITPR	4251.75649	0.43117754	0.06466898	6.66745569	2.60E-11	5.78E-10
CTPS2	3944.93357	-0.4010117	0.0652251	-6.1481201	7.84E-10	1.45E-08
CTSO	486.696217	0.68183891	0.13879301	4.91263142	8.99E-07	9.89E-06
CTU1	217.809117	0.97966459	0.21792742	4.49537094	6.94E-06	6.47E-05
CTXN1	1404.29206	1.01370532	0.09774269	10.3711628	3.35E-25	3.18E-23
CUEDC2	3838.82786	-0.2584302	0.06471422	-3.9934071	6.51E-05	0.00047672
CUX1	2766.16055	-0.1987515	0.07424109	-2.6771094	0.00742604	0.02933013
CWF19L1	2505.98647	-0.2841334	0.07541891	-3.7674023	0.00016496	0.00107718
CXCL12	16422.7126	-0.9137356	0.04723461	-19.344619	2.26E-83	2.70E-80
CYB561	11646.7439	0.16241193	0.04902486	3.31284841	0.00092351	0.00491568
CYB561A3	3442.65004	0.29491325	0.06463489	4.56275604	5.05E-06	4.84E-05
CYBRD1	4165.82645	-0.2847955	0.06154732	-4.6272613	3.71E-06	3.66E-05
CYFIP2	5824.43923	0.40595348	0.05931447	6.84408846	7.70E-12	1.85E-10
CYP1A1	524.714372	1.46981605	0.13963503	10.526127	6.55E-26	6.43E-24
CYP26B1	40.3896365	-1.2202601	0.48018417	-2.5412335	0.01104621	0.04087392
CYP2T1P	777.610426	1.35539512	0.11573171	11.7115273	1.11E-31	1.55E-29
CYP4V2__KLKB1	316.324236	-0.9698862	0.17942443	-5.4055416	6.46E-08	8.68E-07
DAB2IP	9191.27061	0.17686204	0.05096475	3.47028143	0.00051991	0.00298423
DAGLB__KDELR2	21114.4246	-0.1483654	0.0472181	-3.1421292	0.00167724	0.0081915
DAP	4763.47987	-0.4323956	0.06102511	-7.0855361	1.39E-12	3.67E-11
DARS	4188.48132	-0.2544235	0.06141502	-4.1426916	3.43E-05	0.00026892

DBN1	6227.02793	0.25129292	0.05585854	4.49873758	6.84E-06	6.38E-05
DCLRE1B	763.369538	-0.4900891	0.11590398	-4.2284062	2.35E-05	0.00019214
DCUN1D3__ERI2	4421.70764	-0.2702621	0.05949095	-4.5429109	5.55E-06	5.29E-05
DCXR	5382.60619	-0.2246684	0.06309445	-3.5608269	0.00036969	0.00220218
DDAH1	3964.70566	-0.2455472	0.06134753	-4.0025606	6.27E-05	0.00046046
DDX60	437.398095	-0.7952429	0.14635882	-5.4335151	5.53E-08	7.53E-07
DECR2__NME4	5668.8537	0.19468936	0.06001375	3.24407948	0.00117831	0.00609894
DEPDC1	3087.3326	-0.3588737	0.06684148	-5.3690265	7.92E-08	1.05E-06
DGAT2	547.649034	1.10732548	0.13360402	8.28811468	1.15E-16	5.14E-15
DHCR24	54228.3045	0.14987897	0.04221009	3.55078541	0.00038408	0.0022798
DHTKD1	14077.5199	-0.4772522	0.04919686	-9.7008684	2.99E-22	2.32E-20
DHX40	4875.05424	-0.2055314	0.05832306	-3.5240164	0.00042506	0.00249991
DHX58	834.240305	-0.433209	0.11155826	-3.8832535	0.00010307	0.00071345
DHX9	18406.8773	-0.1994958	0.04655646	-4.2850287	1.83E-05	0.00015275
DIP2B	9973.29849	0.18523844	0.06081628	3.04586925	0.00232009	0.01079154
DKK1	106.660407	0.94463507	0.31699489	2.97996936	0.00288277	0.01302785
DLG2	1151.64097	0.32162574	0.09915675	3.24360911	0.00118026	0.00610524
DLG5	23206.0485	-0.2170988	0.0536965	-4.0430722	5.28E-05	0.00039408
DLST	10122.2319	-0.1594749	0.05088389	-3.1340944	0.00172385	0.00840687
DLX1	876.94151	0.47343395	0.10812222	4.3786923	1.19E-05	0.00010474
DLX2	311.435825	0.87725609	0.17380311	5.04741305	4.48E-07	5.23E-06
DNAJB9	1462.23146	0.25590874	0.08843122	2.89387338	0.00380522	0.01658365
DNAJC8	4305.07793	-0.2671117	0.06020791	-4.4364887	9.14E-06	8.26E-05
DNAJC9__MRPS16	10255.6346	-0.3472689	0.04974084	-6.9815654	2.92E-12	7.47E-11
DNASE1L2__E4F1	2273.53448	0.32219223	0.0744488	4.32770198	1.51E-05	0.0001286
DOCK1	1818.00937	-0.2724146	0.0850418	-3.2033028	0.00135861	0.00686622
DOHH	896.723949	0.47545452	0.10912133	4.35711825	1.32E-05	0.0001141
DSCC1	1258.60625	-0.2877055	0.0938551	-3.0654219	0.00217363	0.0102444
DSP	52298.4517	-0.1525897	0.06185408	-2.4669305	0.01362768	0.04863743
DTL	4537.25923	-0.5808543	0.05981309	-9.7111566	2.70E-22	2.11E-20
DUSP10	385.362064	0.73736443	0.15692358	4.698876	2.62E-06	2.63E-05
DUSP22	1101.76632	0.34171136	0.09619042	3.55244685	0.00038167	0.00226787
DUSP4	2737.23358	-1.5305772	0.07062905	-21.670646	3.88E-104	1.08E-100
DUSP8	1191.09416	0.34940158	0.09480107	3.68562911	0.00022814	0.00143959
DVL1	3209.39723	0.21646785	0.06687836	3.23673962	0.00120904	0.00623096
DYNC1I1	157.561523	1.03384455	0.23889912	4.32753595	1.51E-05	0.00012863
DYRK1B	730.888112	0.35892771	0.11753709	3.05374005	0.00226008	0.01058018
DZIP1L	165.290015	-0.7898365	0.23173175	-3.4084085	0.00065343	0.00365663
E2F1	2891.24281	-0.2268875	0.07117796	-3.1876092	0.00143454	0.00719555
E2F2	1163.29959	-0.3003046	0.09436408	-3.1824036	0.00146058	0.00729233
ECT2	18943.5247	-0.1796487	0.04635737	-3.8753	0.00010649	0.00073351
EDARADD	799.216743	0.36796055	0.1103551	3.33433206	0.00085505	0.00458633
EEA1	5759.58481	-0.1808781	0.06258285	-2.890218	0.00384975	0.01676723

EEF1G__RP11-864I4.	89704.8657	-0.3374657	0.04578989	-7.3698727	1.71E-13	5.19E-12
EFEMP1	15824.8754	-0.5804975	0.04853568	-11.960221	5.74E-33	8.64E-31
EFNA1	3441.55312	0.15775664	0.06397595	2.46587434	0.01366793	0.04874982
EFNA3__EFNA4__RP	4389.67026	0.17764605	0.06112183	2.90642565	0.00365584	0.01606088
EFNB3	389.69932	0.56016247	0.15912875	3.52018392	0.00043125	0.00253186
EFTUD2	9179.35642	-0.2333551	0.054422	-4.287881	1.80E-05	0.00015095
EGFL7	1058.52755	1.15251322	0.1012772	11.3797901	5.27E-30	6.67E-28
EGR1	185.156022	-1.2080767	0.22246179	-5.4304907	5.62E-08	7.64E-07
EIF1AX	9884.22487	-0.3057869	0.05083428	-6.0153683	1.79E-09	3.14E-08
EIF2AK2	2984.58636	-0.3995196	0.07289051	-5.4810913	4.23E-08	5.85E-07
EIF2S3	19095.906	-0.3244237	0.04690821	-6.9161387	4.64E-12	1.15E-10
EIF3D	9374.71456	-0.1792475	0.05222267	-3.4323689	0.00059833	0.00338443
EIF3E	20311.8547	-0.2578559	0.04921015	-5.2398924	1.61E-07	2.01E-06
EIF3M	9677.02717	-0.2179417	0.05420732	-4.0205214	5.81E-05	0.00043013
EIF4A2	58187.1279	-0.157302	0.04391222	-3.5821919	0.00034072	0.00205291
EIF4B	41051.6681	-0.2039965	0.0429375	-4.7510111	2.02E-06	2.08E-05
EIF4G3	4389.32196	0.22902024	0.06292695	3.63946169	0.00027321	0.00168952
ELAVL2	41.9078048	1.19844365	0.46346906	2.58581154	0.009715	0.03677084
ELF3	4415.36281	0.37814839	0.05948693	6.35683116	2.06E-10	4.13E-09
ELK3	354.321291	-0.5046337	0.15917803	-3.1702475	0.00152309	0.00755575
ELMOD2	1888.47332	-0.2920074	0.07783649	-3.7515483	0.00017575	0.00114132
EMID1	237.778479	0.59124865	0.20698212	2.85652035	0.00428313	0.01834386
EMP1	276.476931	0.88005198	0.18417575	4.77832711	1.77E-06	1.83E-05
ENDOG	354.066541	0.46911042	0.1821162	2.57588523	0.00999838	0.03771522
ENO1	71511.0368	-0.4241181	0.04493893	-9.4376546	3.81E-21	2.60E-19
ENSA	19701.8986	0.13776982	0.05236219	2.63109364	0.00851106	0.03300678
ENTPD3	101.420088	1.31361431	0.30223182	4.34637992	1.38E-05	0.00011916
EPAS1	6566.82984	0.25865609	0.05703637	4.53493282	5.76E-06	5.46E-05
EPHA1	1126.61253	0.36337734	0.09864345	3.68374541	0.00022983	0.00144918
EPHB3	3837.32072	0.41181484	0.0655787	6.27970459	3.39E-10	6.56E-09
EPS8L1	5801.41439	0.46657665	0.06663337	7.00214746	2.52E-12	6.52E-11
ERAL1	4195.93177	-0.2522338	0.06046293	-4.1717093	3.02E-05	0.00024012
ERBB2	17611.8555	0.84337623	0.04746853	17.7670593	1.27E-70	1.06E-67
ERBB3__PA2G4__RP	35275.1326	-0.347555	0.04325236	-8.0355164	9.32E-16	3.73E-14
ERBB4	1092.18604	-0.5167973	0.10315343	-5.009986	5.44E-07	6.24E-06
ESR1	11914.2188	-0.6765346	0.05384662	-12.564106	3.33E-36	5.61E-34
ESRRA	2289.3754	0.71721598	0.08266789	8.67587127	4.10E-18	2.17E-16
ETNK2	2719.53091	-0.9029541	0.06909939	-13.067468	5.05E-39	9.70E-37
EXOC3	4365.52368	0.17380409	0.06183187	2.81091462	0.00494009	0.0207531
EZH1	2020.70335	-0.2320681	0.07834079	-2.96229	0.0030536	0.01371451
FAM102B	1311.11471	-0.3481474	0.09050685	-3.8466411	0.00011975	0.00081406
FAM110A	2433.62995	0.41191549	0.07339066	5.61264155	1.99E-08	2.90E-07
FAM129A	4266.75607	0.59229918	0.07046687	8.40535648	4.27E-17	1.98E-15

FAM134B	1563.63069	-0.3698778	0.08727641	-4.2380038	2.26E-05	0.00018519
FAM162A	1817.07697	-0.3336649	0.08128018	-4.10512	4.04E-05	0.00031076
FAM171A2	402.155415	0.48206845	0.15829773	3.04532769	0.00232427	0.01080197
FAM192A	3323.47009	-0.4411729	0.06548468	-6.7370396	1.62E-11	3.71E-10
FAM20C	3053.12246	0.29449013	0.0692619	4.2518345	2.12E-05	0.00017541
FAM214A	5778.76642	0.29615538	0.05907064	5.01357986	5.34E-07	6.14E-06
FAM217B	2436.62676	-0.2401198	0.07167568	-3.3500871	0.00080786	0.00437114
FAM43A	491.753437	0.55853527	0.13866752	4.02787389	5.63E-05	0.0004182
FAM69B	965.051857	0.79863163	0.10321242	7.73774705	1.01E-14	3.49E-13
FAM83H	12247.7249	0.36242467	0.04966156	7.29789127	2.92E-13	8.48E-12
FAM89B__SSSCA1	2991.55844	0.32346629	0.07397633	4.37256479	1.23E-05	0.0001075
FAM8A1	4087.37748	-0.258743	0.06091101	-4.2478853	2.16E-05	0.00017826
FANCC	611.039916	-0.5335436	0.12961297	-4.116437	3.85E-05	0.00029771
FANCD2	3539.36064	-0.5076894	0.06420631	-7.9071573	2.63E-15	1.00E-13
FANCI	6967.24467	-0.4327631	0.05586854	-7.7460967	9.48E-15	3.28E-13
FANCM	1332.96475	-0.416144	0.10091815	-4.1235797	3.73E-05	0.00028899
FAR1	4542.2613	-0.4420473	0.06132409	-7.20838	5.66E-13	1.57E-11
FBLIM1	1179.52067	0.40776777	0.0943881	4.32011867	1.56E-05	0.00013249
FBP1	1662.00699	0.5744036	0.08663221	6.63036986	3.35E-11	7.34E-10
FBRSL1	3382.96242	0.18854887	0.06701995	2.81332454	0.00490321	0.02063451
FBXL17	1534.36073	-0.2534276	0.08551921	-2.9633996	0.00304261	0.0136762
FBXL2	839.015009	0.30035322	0.10910402	2.75290697	0.00590687	0.02415237
FBXL20	1755.58087	-0.5251371	0.08099877	-6.4832731	8.98E-11	1.87E-09
FBXL4	1025.33295	-0.3997861	0.0996427	-4.0121968	6.02E-05	0.0004446
FBXL8__HSF4__RP11	1946.94449	0.24282655	0.07850734	3.0930426	0.00198116	0.00945195
FBXO21	4282.12063	-0.2866036	0.06057274	-4.7315615	2.23E-06	2.27E-05
FBXO33	1178.47636	-0.4876301	0.09354845	-5.2125946	1.86E-07	2.30E-06
FDFT1	12509.4258	-0.2387918	0.05279219	-4.5232419	6.09E-06	5.74E-05
FDPS	4690.77195	-0.3003991	0.06013153	-4.9957007	5.86E-07	6.67E-06
FEZ1	933.1818	0.47705477	0.10350792	4.60887233	4.05E-06	3.96E-05
FGD1	1880.30285	-0.3019488	0.07808638	-3.8668554	0.00011025	0.00075594
FGD3	597.661899	0.37024192	0.12616613	2.93455866	0.00334022	0.01484613
FGF12	208.124666	1.12090494	0.21390251	5.24026074	1.60E-07	2.00E-06
FGFBP3	309.922021	1.74961253	0.2065708	8.46979605	2.46E-17	1.17E-15
FGFR3	1851.74901	0.24057164	0.07914923	3.03946893	0.00236996	0.01097384
FHL2	10617.5186	-0.2014044	0.05236219	-3.8463706	0.00011988	0.00081463
FITM2	2238.49988	-0.1832415	0.07372254	-2.4855562	0.01293492	0.04648277
FKBP4	40572.5602	-0.1897605	0.04643027	-4.086999	4.37E-05	0.00033238
FKRP	507.451887	0.37928236	0.13760658	2.75628068	0.00584628	0.02393393
FKTN	5112.95353	-0.2408899	0.06196607	-3.8874492	0.0001013	0.00070239
FLNB	40912.7792	-0.155628	0.05328055	-2.9209155	0.00349005	0.01542983
FLNC	369.534855	1.43051559	0.16787286	8.52142286	1.58E-17	7.79E-16
FLOT2	5427.68099	-0.1778827	0.05679698	-3.1319042	0.00173677	0.00846489

FMN1	10516.4361	-0.2435302	0.06761462	-3.6017383	0.0003161	0.00192411
FN3KRP	2931.67014	-0.4102531	0.06994608	-5.8652773	4.48E-09	7.27E-08
FOS	1634.71018	-0.8613046	0.08305944	-10.369738	3.40E-25	3.21E-23
FOSB	358.009242	-1.2267749	0.16742644	-7.3272471	2.35E-13	6.95E-12
FOXD2	533.465185	0.61878801	0.13775217	4.4920382	7.05E-06	6.56E-05
FOXN2	756.452806	-0.3313699	0.11599214	-2.8568309	0.00427894	0.01833532
FOXO6	301.940984	0.67668829	0.17331488	3.90438652	9.45E-05	0.00066102
FOXRED1	4970.29913	-0.2346617	0.05877953	-3.9922352	6.55E-05	0.00047825
FOXRED2	4762.54496	-0.2193674	0.05854743	-3.7468324	0.00017908	0.00115983
FRAT2	2548.76194	0.21764467	0.07742165	2.81116022	0.00493632	0.02074248
FSTL3	1444.13717	0.89282978	0.0874785	10.2062762	1.86E-24	1.67E-22
FUBP1	7427.21319	-0.3265795	0.0545548	-5.9862666	2.15E-09	3.70E-08
FUT11	860.094168	-0.4766955	0.10772494	-4.4251174	9.64E-06	8.67E-05
FUT7__NPDC1__RP1	10492.0879	0.30703345	0.05097396	6.02333904	1.71E-09	3.00E-08
FYTTD1	16852.3272	-0.1399748	0.04605333	-3.0394064	0.00237045	0.01097384
FZD4	3236.45868	0.33417966	0.06602338	5.06153481	4.16E-07	4.89E-06
FZD7	713.564127	-0.3615236	0.11602039	-3.1160349	0.00183301	0.00886156
G6PD	6854.65851	0.34591878	0.05384278	6.42460838	1.32E-10	2.71E-09
GABARAPL1	1605.45583	0.26806715	0.08283301	3.23623562	0.00121117	0.00623812
GABRD	65.8169284	1.01073046	0.38793112	2.60543792	0.00917569	0.03507129
GALK1	1647.38629	-0.2548873	0.08321617	-3.0629543	0.00219164	0.0102973
GALNT5	60.0068621	-1.4816067	0.38886168	-3.8101124	0.0001389	0.00093033
GANAB	44902.6643	-0.1462833	0.04279919	-3.4179001	0.00063106	0.00354665
GAPDH	51252.2825	-0.1815923	0.04611254	-3.9380238	8.22E-05	0.00058319
GART	9328.26725	-0.1579962	0.05110153	-3.0918101	0.0019894	0.00947774
GAS6	4328.09323	-0.2840263	0.05986191	-4.7446917	2.09E-06	2.14E-05
GAS6-AS2	431.0872	-0.4136324	0.14575776	-2.837807	0.00454246	0.01929132
GATA4	296.697163	-0.5913959	0.17593624	-3.3614216	0.00077542	0.00422437
GCN1	16265.0839	-0.245774	0.05193686	-4.73217	2.22E-06	2.26E-05
GDF15	717.058945	1.6399396	0.12369043	13.2584194	4.03E-40	8.53E-38
GEMIN7__MARK4__	5300.74949	0.27513073	0.05994538	4.58969008	4.44E-06	4.29E-05
GGH	4247.34031	-0.2175764	0.06585842	-3.3036991	0.00095418	0.00505959
GINS1	3124.81485	-0.4440886	0.06645687	-6.6823575	2.35E-11	5.24E-10
GK5	4533.07985	-0.1739035	0.06336305	-2.7445575	0.00605925	0.02467879
GLB1L2	1315.78156	0.29504572	0.09342743	3.15802027	0.00158845	0.00781773
GLI4__RP13-582O9.6	2204.35657	0.22927701	0.0790575	2.90012983	0.00373008	0.01631837
GLS2	738.835564	-0.3521955	0.11668706	-3.0182909	0.00254205	0.01166158
GLT8D2	238.148857	-0.8423665	0.19636366	-4.2898291	1.79E-05	0.00014993
GMNN	1987.32897	-0.3262689	0.08110443	-4.0228249	5.75E-05	0.00042632
GMPS	10721.1005	-0.2309197	0.04992735	-4.6251148	3.74E-06	3.69E-05
GNA13	6138.37763	-0.2494006	0.05653707	-4.4112767	1.03E-05	9.17E-05
GNB2L1	46394.9122	-0.276001	0.04685934	-5.8899901	3.86E-09	6.34E-08
GOLGA7B	560.83446	0.90573281	0.14229336	6.36524988	1.95E-10	3.92E-09

GPI__PDCD2L__RP11	11263.2403	-0.3133719	0.05047299	-6.208705	5.34E-10	1.01E-08
GPN3	2498.22245	-0.1920231	0.07372462	-2.6046	0.00919816	0.03514109
GPR158	868.482556	0.30687078	0.11610156	2.64312364	0.0082145	0.03199041
GPR37	250.656245	0.81805829	0.20013414	4.08755003	4.36E-05	0.00033204
GPR37L1	463.735084	-1.1301457	0.14271779	-7.9187448	2.40E-15	9.13E-14
GRAMD2	484.080192	-0.4003072	0.13773702	-2.906315	0.00365713	0.0160617
GREB1	32157.3596	-0.246033	0.05457039	-4.508544	6.53E-06	6.11E-05
GRIN1	353.067496	0.60581837	0.16220795	3.73482543	0.00018785	0.00121095
GYS1	3130.40353	-0.3692618	0.06973824	-5.2949681	1.19E-07	1.53E-06
H19__MIR675	845.246344	0.47894156	0.11281042	4.24554364	2.18E-05	0.00018005
H2AFX	6348.09564	0.30699258	0.07231905	4.24497536	2.19E-05	0.00018041
H3F3B	20012.5995	-0.2466063	0.04959671	-4.9722309	6.62E-07	7.45E-06
HAUS6	1483.3616	-0.4009515	0.09140073	-4.3867424	1.15E-05	0.00010147
HDAC1	7613.47571	-0.16828	0.05996196	-2.8064457	0.00500914	0.02100931
HELLS	3824.95452	-0.5839465	0.06305295	-9.2612087	2.02E-20	1.31E-18
HELZ2	2816.86925	-0.2442936	0.07840742	-3.1156948	0.00183512	0.00886922
HERPUD1	2873.46375	0.39210928	0.06803062	5.76371785	8.23E-09	1.28E-07
HERPUD2	2040.36516	-0.2591217	0.07649721	-3.3873358	0.00070575	0.00390983
HES4	1067.90366	0.7027225	0.13321116	5.27525243	1.33E-07	1.69E-06
HIST1H1C	1816.53159	0.43107585	0.08408198	5.1268515	2.95E-07	3.54E-06
HIST1H2AC	2753.90427	0.46626557	0.07654844	6.09111787	1.12E-09	2.01E-08
HIST1H2AD__HIST1H	435.372935	0.56826064	0.14637358	3.88226235	0.00010349	0.00071577
HIST1H2BB__HIST1H	692.405598	0.39335277	0.11923086	3.29908536	0.00097	0.00513847
HIST1H2BD	1885.11493	0.25789597	0.08103522	3.18251718	0.00146001	0.00729233
HIST1H2BG	1543.88047	0.85418657	0.0866041	9.86311926	6.02E-23	4.83E-21
HIST1H3E	746.906073	0.60740474	0.11451782	5.30401959	1.13E-07	1.46E-06
HIST1H4H	1035.12052	1.14324471	0.10086318	11.3346089	8.84E-30	1.10E-27
HIST2H2AA3__HIST2	2399.24593	0.6226729	0.0750583	8.29585702	1.08E-16	4.84E-15
HIST3H2A	1174.18423	0.98549661	0.10368303	9.50489791	2.00E-21	1.44E-19
HK1	14370.7689	-0.1606349	0.04732401	-3.3943639	0.00068788	0.0038299
HLA-F	525.996921	-0.3636007	0.13575893	-2.6782823	0.00740008	0.02924192
HMGCS2	558.577953	-0.3445701	0.13615739	-2.530675	0.01138433	0.04191149
HNRNPC	25013.476	-0.2904025	0.04765201	-6.0942345	1.10E-09	1.98E-08
HNRNPH1	19150.4905	-0.3362103	0.04551761	-7.3863776	1.51E-13	4.62E-12
HNRNPH3	12325.7667	-0.3017349	0.05224628	-5.7752417	7.68E-09	1.20E-07
HORMAD2-AS1__RP1	2148.97933	0.2276401	0.07714324	2.95087546	0.00316875	0.0141707
HOXB2	2586.12621	0.30917839	0.07370613	4.19474459	2.73E-05	0.00021927
HOXC13	2172.43703	0.23125658	0.07473744	3.09425354	0.00197309	0.00942961
HP1BP3	7791.08723	-0.2428259	0.05398331	-4.4981661	6.85E-06	6.40E-05
HPCAL1	1454.71007	0.35553815	0.08787121	4.04612778	5.21E-05	0.00038933
HPS3	2461.23278	-0.3025261	0.07772538	-3.8922442	9.93E-05	0.00069008
HPS4	1870.02238	-0.2209266	0.0796491	-2.7737495	0.00554143	0.02289919
HS6ST3	677.25116	0.63099823	0.11963722	5.27426369	1.33E-07	1.70E-06

HSD17B12	6652.57935	-0.1568738	0.05441187	-2.8830797	0.00393808	0.01707666
HSP90AB1	105548.532	-0.2270651	0.04182483	-5.4289548	5.67E-08	7.70E-07
HSP90B1	87061.7663	-0.1360342	0.04145612	-3.2814023	0.00103292	0.00541523
HSPA13	3766.72909	-0.3655463	0.06312798	-5.7905587	7.02E-09	1.10E-07
HSPA4	14532.9138	-0.1720979	0.04714198	-3.6506305	0.0002616	0.001628
HSPB1	42564.8752	0.3896636	0.06059853	6.43024826	1.27E-10	2.62E-09
HSPD1	31848.8691	-0.1481479	0.04389044	-3.3754032	0.00073708	0.00405781
HYAL2__TUSC2	3501.47292	0.23102748	0.06859523	3.36798185	0.00075721	0.00415084
HYOU1	20958.5658	-0.1777969	0.04682785	-3.7968191	0.00014656	0.00097377
IARS2	23783.3702	-0.1207461	0.04485811	-2.6917338	0.00710817	0.02825504
ID1	1644.63244	0.38397179	0.08896341	4.31606431	1.59E-05	0.00013474
ID2	1188.86485	0.92634876	0.09642339	9.6070963	7.46E-22	5.54E-20
ID3	2856.53176	0.36618032	0.06955325	5.26474783	1.40E-07	1.77E-06
IDI1	9843.58444	-0.1379343	0.05146832	-2.679985	0.00736255	0.02913447
IER3	14599.0287	-0.5502302	0.05265028	-10.45066	1.46E-25	1.40E-23
IFFO1	16786.3023	-0.1660895	0.04942308	-3.3605652	0.00077783	0.00423472
IFI27	344.075155	-0.7354729	0.16404867	-4.4832608	7.35E-06	6.81E-05
IFI27L1	529.007427	-0.3848633	0.13269362	-2.9003904	0.00372698	0.01630908
IFI44	49.2803921	-1.0674428	0.4250383	-2.5114039	0.0120252	0.04388397
IFI6	1853.10283	-0.882372	0.09228628	-9.5612481	1.16E-21	8.54E-20
IFIT1	713.994266	-1.4441252	0.11979807	-12.054662	1.83E-33	2.78E-31
IFIT2	292.848749	-0.6632108	0.17609168	-3.7662816	0.0001657	0.00108153
IFIT3	286.006585	-0.862241	0.18251439	-4.724236	2.31E-06	2.35E-05
IFIT5	614.027986	-0.5330763	0.12415644	-4.2935855	1.76E-05	0.00014786
IFRD1__LSMEM1	1757.63964	0.61354871	0.08324804	7.37012813	1.70E-13	5.19E-12
IGF1R	9415.30325	-0.2732769	0.0690283	-3.9589108	7.53E-05	0.00054044
IGF2__INS__INS-IGF	619.87616	1.84216882	0.13576432	13.5688734	6.13E-42	1.44E-39
IGFALS__SPSB3	1827.76797	0.22342939	0.07915177	2.82279726	0.00476067	0.02008016
IGFBP4	36603.4807	-0.2583419	0.05165067	-5.0017141	5.68E-07	6.49E-06
IGSF9	2810.28998	0.49086984	0.06856559	7.15912851	8.12E-13	2.22E-11
IKZF2	1347.73511	0.30913881	0.10129376	3.05190374	0.00227395	0.01062725
IL17RC	3681.26688	0.46546164	0.06363486	7.31457006	2.58E-13	7.54E-12
IL17RE	1607.35313	0.63235253	0.0850867	7.43186134	1.07E-13	3.31E-12
IL1R1	831.809248	0.39574819	0.10962427	3.61004181	0.00030615	0.00186832
IL6ST	29510.8287	-0.2170106	0.05366517	-4.0437888	5.26E-05	0.00039306
IMPAD1	7722.25317	-0.3126364	0.05215148	-5.9947765	2.04E-09	3.52E-08
INHBB	7266.26629	0.56737162	0.05312581	10.6797732	1.27E-26	1.35E-24
INHBE	71.3751929	1.32012608	0.35612276	3.70694109	0.00020978	0.00133788
INO80B__INO80B-W	3578.72095	0.16913534	0.0668127	2.53148506	0.01135807	0.04183325
INPP4B	2838.7832	0.25154986	0.07022378	3.58211813	0.00034082	0.00205291
INPPL1	10073.2399	0.38702023	0.05038048	7.68194781	1.57E-14	5.27E-13
INSIG1	2577.90782	-0.4362051	0.07206111	-6.0532659	1.42E-09	2.51E-08
IPO5	8446.43441	-0.1459992	0.05288678	-2.7605997	0.00576953	0.02367777

IPO7	13272.5151	-0.2482701	0.04983321	-4.98202	6.29E-07	7.13E-06
IPO8	5744.36383	-0.1533443	0.05630248	-2.7235791	0.00645787	0.02605456
IPPK	1398.7498	0.38174727	0.08923682	4.27791233	1.89E-05	0.00015724
IRAK1	5301.24062	0.21449556	0.060648	3.5367294	0.00040511	0.00239696
IRX2	1189.66586	0.64105874	0.09863521	6.49928876	8.07E-11	1.68E-09
IRX4	185.136301	0.92343654	0.22019223	4.19377432	2.74E-05	0.00021989
ISG15	310.71293	-1.049216	0.1810976	-5.7936496	6.89E-09	1.08E-07
ITGA5	1352.27673	0.23597339	0.09212577	2.56142651	0.01042433	0.03899461
ITPK1	10058.5418	-0.2035426	0.04979837	-4.0873354	4.36E-05	0.0003322
ITPR3	3844.82146	-0.2672802	0.08129805	-3.2876581	0.00101024	0.00531301
IVNS1ABP	5383.13482	-0.1764746	0.05796516	-3.0444943	0.00233072	0.01082049
JADE2	2165.60697	0.42183849	0.07739855	5.45021196	5.03E-08	6.91E-07
JAG2__NUDT14	4569.64651	0.35097632	0.05901465	5.94727438	2.73E-09	4.60E-08
JAK1	21434.4651	-0.5401877	0.04915612	-10.989224	4.31E-28	4.86E-26
JUP	50976.0677	0.18478257	0.04455798	4.14701441	3.37E-05	0.00026439
KATNAL1	660.610924	-0.908605	0.12046248	-7.5426392	4.61E-14	1.49E-12
KCNC3	2621.86914	0.18953519	0.07223365	2.62391809	0.00869247	0.03354674
KCNK5	1225.54551	-0.2889412	0.09437028	-3.0617811	0.00220024	0.01033192
KCNS3	1049.45856	-0.4432486	0.09841937	-4.5036725	6.68E-06	6.24E-05
KCTD6	3411.50127	-0.3650517	0.06990326	-5.2222412	1.77E-07	2.19E-06
KDELC2	5203.19274	0.14780282	0.05907487	2.50195774	0.01235087	0.04486658
KDM1A	5636.94826	-0.228248	0.05699746	-4.0045293	6.21E-05	0.00045765
KDM2B	3416.49871	-0.2750064	0.0654592	-4.2011878	2.66E-05	0.00021394
KDM4A	2453.11488	0.37566171	0.07328596	5.12597107	2.96E-07	3.55E-06
KHNYN	2406.98778	-0.529862	0.07239378	-7.3191647	2.50E-13	7.32E-12
KIAA0391__PSMA6__	16040.2862	-0.181926	0.05285666	-3.4418754	0.0005777	0.00328206
KIAA0513	2523.63496	0.43450192	0.07884848	5.5105938	3.58E-08	5.02E-07
KIAA1147	3745.69817	0.41915431	0.0650438	6.44418545	1.16E-10	2.40E-09
KIAA1161	11083.6878	0.3311855	0.05008228	6.61282783	3.77E-11	8.19E-10
KIAA1429	6545.73891	-0.2269959	0.05505028	-4.1234291	3.73E-05	0.00028905
KIF13B	1981.6072	-0.4218863	0.08089826	-5.215023	1.84E-07	2.27E-06
KIF14	2675.62957	-0.4630813	0.07626431	-6.0720584	1.26E-09	2.26E-08
KIF1C	12226.898	0.41457497	0.04869476	8.51374873	1.68E-17	8.22E-16
KIFC2	2854.19171	0.26052349	0.0678546	3.83943741	0.00012332	0.00083662
KLF16	1347.32952	0.38419079	0.09541616	4.02647515	5.66E-05	0.0004205
KLF4	888.736678	0.32695137	0.10538967	3.10230936	0.00192017	0.00921098
KLHL12	4510.25875	-0.2203036	0.05939617	-3.7090537	0.00020804	0.00132829
KLHL15	763.594059	-0.4645247	0.11625736	-3.9956586	6.45E-05	0.00047304
KMO	475.802346	1.21288034	0.1432776	8.46524723	2.56E-17	1.21E-15
KNTC1	6443.36027	-0.5105677	0.06573225	-7.7673855	8.01E-15	2.82E-13
KRBA2__RP11-849F2	22788.408	-0.4093255	0.0542377	-7.5468821	4.46E-14	1.44E-12
KREMEN2	1670.23004	0.65472253	0.08962645	7.30501468	2.77E-13	8.05E-12
KRR1	4046.02857	-0.224358	0.06118071	-3.6671368	0.00024528	0.00153501

KRT13	71.7817464	-1.8811859	0.3654046	-5.1482273	2.63E-07	3.19E-06
KRT15	2391.65398	-0.5022153	0.07327364	-6.8539694	7.18E-12	1.74E-10
KRT80	7745.86083	0.20263108	0.05535398	3.66064157	0.00025158	0.00156975
KYNU	7999.26202	0.36434595	0.05477817	6.65129876	2.91E-11	6.43E-10
LA16c-312E8.4	116.740582	2.00058997	0.29773656	6.71932921	1.83E-11	4.13E-10
LAMB3	560.369142	-0.4233194	0.13325752	-3.1767017	0.0014896	0.00742268
LANCL1	4792.35294	-0.3327272	0.05886618	-5.6522644	1.58E-08	2.34E-07
LAP3	2384.63938	-0.2403796	0.07481169	-3.2131289	0.00131297	0.00667486
LBR	3775.96719	-1.073867	0.06347386	-16.918258	3.30E-64	2.04E-61
LDHA	33915.611	-0.4168516	0.04892839	-8.5196275	1.60E-17	7.89E-16
LDHAP4	400.957057	-0.5108861	0.15361575	-3.3257405	0.00088184	0.00471642
LDLRAD1	201.69145	0.97006967	0.21447624	4.52297024	6.10E-06	5.74E-05
LHFP	764.649359	-0.6101085	0.11571305	-5.2725986	1.35E-07	1.71E-06
LHX4	659.6159	-0.4435057	0.12773521	-3.4720708	0.00051646	0.00296747
LIMA1	5369.7429	0.2004813	0.05654542	3.54549167	0.00039188	0.0023236
LIME1__RP4-583P15.	9367.13523	0.29426326	0.05165676	5.69651049	1.22E-08	1.84E-07
LIMK1	1673.41139	0.32151876	0.08160587	3.93989761	8.15E-05	0.00057945
LIMK2	6612.95844	0.37177532	0.05416102	6.86426008	6.68E-12	1.62E-10
LINC00472__RP11-15	141.598107	0.69452173	0.25385159	2.73593609	0.00622031	0.02526084
LINC00657	37690.5584	-0.1300804	0.0444023	-2.9295858	0.00339414	0.01506573
LINC00885	1169.59751	0.44919828	0.09466741	4.74501501	2.08E-06	2.14E-05
LINC01588	311.110995	-0.6268251	0.17182522	-3.6480392	0.00026425	0.0016408
LIPH	2031.50779	0.62316121	0.07986694	7.80249288	6.07E-15	2.18E-13
LITAF	8342.5957	-0.5654143	0.05155836	-10.966491	5.54E-28	6.13E-26
LIX1L	334.421699	-0.8319509	0.18949653	-4.3903228	1.13E-05	0.00010018
LLNLR-284B4.1	774.522088	0.35116858	0.11530424	3.04558245	0.0023223	0.01079582
LMCD1	2565.25532	0.26036163	0.07471379	3.48478674	0.00049253	0.00284072
LMO4	5760.07053	0.98452893	0.05648816	17.4289433	4.98E-68	3.46E-65
LMTK3	1546.89635	0.51167222	0.08517653	6.00719709	1.89E-09	3.29E-08
LPGAT1	3323.9265	0.28512471	0.06907689	4.12764277	3.67E-05	0.00028504
LPP	5458.67993	-0.3475454	0.07922464	-4.3868349	1.15E-05	0.00010147
LRCH4__SAP25	2365.0573	0.27228447	0.07195224	3.7842387	0.00015418	0.00101829
LRIG1	13292.2922	-0.3942229	0.04998665	-7.8865639	3.11E-15	1.17E-13
LRRC26__RP11-350O	1398.16735	1.80612949	0.25369583	7.11927157	1.08E-12	2.92E-11
LRRC40	1166.0836	-0.2460963	0.09609989	-2.5608383	0.01044199	0.03904469
LRRC56	443.772685	0.77291772	0.14490838	5.33383717	9.62E-08	1.26E-06
LRRC58	5657.07165	-0.5309874	0.06419826	-8.2710565	1.33E-16	5.85E-15
LRRC8B	1877.21228	-0.2653221	0.08293037	-3.1993362	0.00137744	0.00694567
LRRCC1	2517.17628	-0.2417033	0.07205799	-3.354289	0.00079569	0.00431929
LRRN1	802.443237	0.64959017	0.11103058	5.85055192	4.90E-09	7.84E-08
LRRN3	1506.86846	-0.5918253	0.08653824	-6.8388881	7.98E-12	1.91E-10
LSS	5600.79765	0.25189554	0.05781359	4.35702976	1.32E-05	0.0001141
LTV1__PHACTR2__RI	2833.86164	0.27413943	0.07120741	3.84987219	0.00011818	0.00080504

LUZP6__MTPN	6927.17019	-0.9042841	0.05813563	-15.55473	1.48E-54	5.88E-52
LYPD3	2186.20903	1.05267716	0.07634346	13.7887016	2.98E-43	7.66E-41
LYRM1	1006.9759	-0.3916943	0.10034447	-3.9034969	9.48E-05	0.0006629
MADD	4338.02984	0.18127405	0.05991104	3.02572023	0.00248042	0.01140705
MAF1	4704.70323	0.19588384	0.06721819	2.91414928	0.00356659	0.01572257
MAFA	284.553752	1.02962952	0.19549887	5.26667769	1.39E-07	1.76E-06
MAFB	436.353874	0.57068156	0.1540603	3.70427406	0.000212	0.00134946
MAFK	2184.21739	0.22670297	0.07799519	2.90662744	0.00365348	0.01605897
MAGED2	20064.6646	0.29323599	0.04720321	6.2122047	5.22E-10	9.92E-09
MAL2	10739.4311	-0.5195837	0.05223387	-9.9472576	2.59E-23	2.12E-21
MALAT1	102699.587	19.9685288	1.449076	13.7801805	3.35E-43	8.36E-41
MAP1A	1975.47426	0.51307714	0.08746657	5.86597999	4.46E-09	7.25E-08
MAP2	1378.02243	0.38545252	0.093638	4.1164115	3.85E-05	0.00029771
MAP7D1	3427.31807	0.45781113	0.06678561	6.85493626	7.13E-12	1.73E-10
MAPK1	7012.96385	-0.2348483	0.05683928	-4.1317965	3.60E-05	0.00028041
MAPK10	172.654789	0.89207601	0.22925827	3.89114002	9.98E-05	0.00069266
MAPK11	1009.7472	0.30854391	0.10259528	3.00738897	0.00263502	0.0120517
MAPK15	815.409253	0.34063983	0.1123117	3.03298624	0.00242147	0.0111692
MAPK8	1447.53303	-0.4363473	0.08648051	-5.045614	4.52E-07	5.28E-06
MAPK8IP1	1616.86197	0.49081042	0.08657319	5.66931193	1.43E-08	2.13E-07
MAPT	8833.6442	-0.6617671	0.05319521	-12.440351	1.58E-35	2.53E-33
MARCKS	72.7542029	1.14197606	0.36665406	3.11458726	0.00184202	0.00888972
MARCKSL1	5666.78525	0.55601225	0.06136471	9.06078179	1.30E-19	7.87E-18
MARK2	7147.05478	0.19906603	0.05886742	3.3815993	0.00072065	0.00397919
MARVELD3	2230.64295	0.33156297	0.07925204	4.18365221	2.87E-05	0.00022882
MASTL	2003.71627	-0.5168962	0.07861568	-6.5749765	4.87E-11	1.04E-09
MAT2A	10087.9924	0.26219061	0.04989095	5.25527436	1.48E-07	1.86E-06
MATN2	1294.63061	0.42670143	0.0913672	4.67018155	3.01E-06	3.00E-05
MATR3	21080.6738	-0.2727299	0.04536891	-6.0113824	1.84E-09	3.21E-08
MB	833.489032	0.37830843	0.10959436	3.45189683	0.00055666	0.00317443
MBNL3	652.893261	-0.5668865	0.12511957	-4.5307581	5.88E-06	5.56E-05
MBOAT1	1377.15607	-0.8948151	0.09049592	-9.8879054	4.70E-23	3.83E-21
MC1R__RP11-566K1:	6276.89465	0.15878282	0.05597259	2.83679616	0.00455687	0.01933284
MCC	1256.1417	0.38252136	0.10604739	3.60707945	0.00030966	0.00188839
MCFD2	4076.7365	0.17100321	0.06419806	2.66368177	0.00772906	0.03034044
MCM2	7309.47111	-0.4534051	0.05324554	-8.515363	1.66E-17	8.16E-16
MCM3	9580.46056	-0.5465374	0.05046344	-10.830365	2.47E-27	2.70E-25
MCM4	15464.1096	-0.3190342	0.04810132	-6.6325451	3.30E-11	7.25E-10
MCM5	2953.19591	-0.4267653	0.06751753	-6.3208076	2.60E-10	5.13E-09
MCM6	4834.98256	-0.3316565	0.05798045	-5.7201445	1.06E-08	1.62E-07
MCM7	6382.14026	-0.3064996	0.05970013	-5.1339856	2.84E-07	3.42E-06
MDC1	4335.54781	-0.7903926	0.07039153	-11.228519	2.95E-29	3.60E-27
MDGA1	274.269003	-0.4908449	0.18244509	-2.6903704	0.00713727	0.028364

MDGA2	656.382228	0.57844567	0.13117684	4.40966316	1.04E-05	9.22E-05
MDK	7095.84348	0.89939035	0.06005829	14.97529	1.07E-50	4.04E-48
MED12L	181.680173	-0.6301846	0.22920756	-2.7494057	0.00597034	0.02437611
MED14	5985.58665	-0.3034325	0.06204786	-4.8902982	1.01E-06	1.10E-05
MEGF9	4759.5735	0.19898314	0.05922352	3.35986703	0.0007798	0.00424267
MESDC1	1112.56031	0.48320857	0.09913587	4.87420499	1.09E-06	1.18E-05
METRN	2908.28625	0.83043156	0.09325447	8.9050051	5.34E-19	3.10E-17
METTL21A	557.232864	-0.3356018	0.12925494	-2.5964327	0.00941974	0.0358319
METTL21B__RP11-57	4555.7567	-0.2699163	0.06959226	-3.8785386	0.00010509	0.00072561
METTL7A	10289.5748	0.33730184	0.0496353	6.79560337	1.08E-11	2.53E-10
MFAP1	4535.18599	-0.179897	0.06126797	-2.9362327	0.00332225	0.01478591
MFI2	876.890276	0.4219642	0.10979213	3.84330082	0.00012139	0.00082422
MFN1	9565.73561	-0.1528209	0.0506892	-3.0148616	0.00257097	0.01177807
MGST1	11498.9318	0.48040332	0.05261321	9.1308499	6.80E-20	4.20E-18
MICB	4233.70674	-0.7670392	0.06043376	-12.69223	6.53E-37	1.16E-34
MISP	3347.78474	0.66508769	0.07038011	9.44993765	3.39E-21	2.34E-19
MLXIP	4525.56703	0.18291468	0.06608937	2.76768661	0.00564557	0.0232693
MOB1A	7214.24317	-0.1701438	0.05353846	-3.1779738	0.00148308	0.00739681
MOK	1114.62923	0.25879151	0.10180129	2.54212407	0.01101811	0.04080608
MORF4L2	11517.5192	-0.1445948	0.04920629	-2.9385426	0.00329759	0.01468792
MPP2	2912.062	-0.1932159	0.06727085	-2.8722089	0.00407613	0.01756091
MPZL1	5909.09403	-0.1575896	0.05516622	-2.8566323	0.00428162	0.01834209
MROH6	454.766729	0.45834102	0.14379664	3.18742502	0.00143546	0.00719581
MRPL22	2826.26822	-0.2056711	0.076554	-2.6866147	0.00721802	0.02864399
MRPS10	4433.88287	0.21226913	0.06105442	3.47672032	0.00050759	0.00292151
MRPS23	3460.52504	-0.2617412	0.07170138	-3.6504345	0.0002618	0.001628
MSH2	4102.06669	-0.4597147	0.06329369	-7.2631994	3.78E-13	1.07E-11
MSH6	5715.41113	-0.2801198	0.05625494	-4.9794713	6.38E-07	7.21E-06
MSS51	104.108139	-0.8108947	0.29201822	-2.7768632	0.00548863	0.02270345
MT-ND6	154147.624	-0.350035	0.04287524	-8.1640363	3.24E-16	1.36E-14
MTDH	16021.3822	-0.2018375	0.04668476	-4.3234139	1.54E-05	0.00013079
MTERF2	2279.68905	0.6392238	0.07392851	8.64651283	5.31E-18	2.75E-16
MTHFD1	12747.88	-0.2450348	0.04812713	-5.0914069	3.55E-07	4.23E-06
MUM1L1	253.695426	1.19176129	0.19472638	6.120184	9.35E-10	1.71E-08
MX1	2420.68064	-1.2882092	0.07363269	-17.495073	1.56E-68	1.13E-65
MXD3__RAB24	3576.92011	0.23656297	0.06945157	3.4061573	0.00065884	0.00368294
MXD4	4002.34392	0.33267005	0.06387035	5.20852115	1.90E-07	2.35E-06
MXRA7	2193.08219	-0.3586895	0.0826196	-4.3414574	1.42E-05	0.00012143
MYB	2158.6206	-0.6790948	0.07675646	-8.8473952	8.96E-19	5.07E-17
MYC	8192.15076	-0.1827055	0.05144522	-3.5514562	0.00038311	0.0022748
MYH14	3214.54328	0.19294052	0.06692185	2.88307214	0.00393817	0.01707666
MYO18A__TIAF1	4517.22765	0.21550962	0.06351886	3.39284437	0.00069171	0.00384608
MYO1B	2776.28257	0.41581007	0.06925864	6.00372847	1.93E-09	3.36E-08

Metazoa_SRP__RN7!	1301.80852	-0.5447947	0.10265113	-5.3072446	1.11E-07	1.44E-06
NAALADL2	1301.87486	1.35783827	0.09698536	14.0004462	1.55E-44	4.24E-42
NACC1	5459.27361	0.16299763	0.05718728	2.85024287	0.00436859	0.01867157
NACC2	2651.18557	0.38212469	0.07005489	5.45464683	4.91E-08	6.76E-07
NANOS1	747.240647	1.62544586	0.12440205	13.0660694	5.15E-39	9.77E-37
NAP1L1	36364.9794	-0.2585371	0.04535705	-5.7000426	1.20E-08	1.80E-07
NARF	2393.68815	-0.2969875	0.07446194	-3.9884475	6.65E-05	0.00048404
NCBP1	2723.52196	-0.1828882	0.0708653	-2.5807858	0.00985757	0.03723451
NCL	32967.7839	-0.1894371	0.04341435	-4.3634674	1.28E-05	0.00011114
NCOA3	7240.14197	0.27089377	0.05992618	4.52045753	6.17E-06	5.79E-05
NCOA5	2399.73123	0.26333393	0.07380241	3.56809393	0.00035959	0.00215042
NCR3LG1	538.309508	-0.9742585	0.15785398	-6.1718973	6.75E-10	1.25E-08
NDC1	6113.63251	-0.2779935	0.0563968	-4.9292419	8.25E-07	9.17E-06
NDUFS5	4328.78335	-0.292141	0.06629644	-4.4065861	1.05E-05	9.34E-05
NEAT1__mascRNA-n	47016.7276	0.19840993	0.04732127	4.19282743	2.75E-05	0.0002206
NECAB3	3842.06255	0.263132	0.06729521	3.91011458	9.23E-05	0.00064798
NEDD1	2903.82453	-0.3833011	0.06955289	-5.5109301	3.57E-08	5.02E-07
NEIL2	837.823551	-0.4243218	0.1086056	-3.9069971	9.35E-05	0.00065457
NEK1	837.709927	-0.7828724	0.11448395	-6.8382726	8.02E-12	1.92E-10
NEK11	770.535031	0.4786033	0.11282914	4.24184106	2.22E-05	0.00018259
NEMP1	6843.77842	-0.4001882	0.05712524	-7.005454	2.46E-12	6.39E-11
NF1	8407.14125	-0.5055418	0.06956086	-7.2676188	3.66E-13	1.04E-11
NFE2L1	19292.0522	0.13949081	0.04605608	3.02871669	0.00245595	0.01131009
NFKBIB	810.070999	0.36234829	0.11414876	3.17435141	0.00150172	0.00746079
NIPAL2	1801.34617	0.20705067	0.08009502	2.58506314	0.00973612	0.03683406
NIPAL3	5545.82806	0.16180833	0.06277168	2.57772817	0.00994522	0.03753313
NKAIN1	1360.57354	-0.5201141	0.09464559	-5.4953864	3.90E-08	5.43E-07
NKTR	8076.57745	-0.4951024	0.06155482	-8.0432757	8.75E-16	3.52E-14
NME1__NME1-NME2	28051.3793	-0.1741587	0.05144812	-3.3851337	0.00071144	0.00393611
NME6	1041.99129	0.29833468	0.09954318	2.99703771	0.00272617	0.01241757
NOM1	1008.95168	-0.2610484	0.10142427	-2.5738263	0.01005808	0.03789414
NOXA1	671.898229	0.51789093	0.12370805	4.18639631	2.83E-05	0.00022629
NPEPPS	12019.3169	0.39762244	0.04923755	8.07559367	6.71E-16	2.76E-14
NPIPB5	1356.42887	-0.26723	0.08928563	-2.9929788	0.00276269	0.01256678
NPM1	75340.7844	-0.1899816	0.04829909	-3.9334406	8.37E-05	0.00059317
NPTN	6753.15847	0.16099809	0.05405668	2.97831995	0.00289833	0.01308495
NQO1	14634.6882	0.21929092	0.05498971	3.987854	6.67E-05	0.00048504
NR1D2	2653.60462	0.23240807	0.07143096	3.25360419	0.00113951	0.00591278
NR2C2	3922.83581	-0.3166511	0.06980677	-4.5361097	5.73E-06	5.44E-05
NRIP2	100.108326	-0.8906727	0.31008921	-2.8723113	0.00407481	0.01756091
NTN4	1430.40234	0.65235453	0.08719016	7.48197408	7.32E-14	2.31E-12
NUCKS1	41327.2105	-0.2608005	0.04434139	-5.8816499	4.06E-09	6.65E-08
NUDCD1	2623.5618	-0.2038057	0.06967635	-2.9250334	0.00344419	0.01526241

NUDT1	1165.06189	-0.2982299	0.1019214	-2.9260777	0.00343265	0.01521901
NUDT21	7206.81937	-0.6882339	0.05450247	-12.627572	1.49E-36	2.62E-34
NUDT3__RPS10__RP	31073.6616	-0.1694927	0.04734451	-3.5799863	0.00034361	0.002066
NUDT5	5907.11902	-0.1869055	0.05927448	-3.1532207	0.0016148	0.00792593
NUFIP2	11816.7887	-0.1478547	0.05191831	-2.8478338	0.00440179	0.01879905
NUP155	6427.0831	-0.1632113	0.05981694	-2.7285135	0.00636205	0.0257488
NUPR1	12488.9655	0.76087426	0.05459952	13.9355474	3.85E-44	1.02E-41
NXPH4	2020.07659	0.41186801	0.08451499	4.87331307	1.10E-06	1.18E-05
OAS1	486.864325	-0.6041055	0.13928628	-4.33715	1.44E-05	0.00012357
OAS2	1456.43151	-0.8031019	0.0888389	-9.03998	1.57E-19	9.41E-18
OAS3	7110.11019	-0.7625686	0.05398305	-14.126075	2.62E-45	7.30E-43
OASL	240.650918	-0.857446	0.192211	-4.4609623	8.16E-06	7.44E-05
OBSL1	2065.32596	0.34349969	0.07582511	4.53015768	5.89E-06	5.57E-05
OCIAD1	5943.61661	-0.2779747	0.05793537	-4.7980134	1.60E-06	1.67E-05
ODC1	3724.87193	0.52600469	0.07000133	7.51420955	5.73E-14	1.82E-12
OLA1	4587.09871	-0.2613292	0.06617544	-3.9490357	7.85E-05	0.00056034
OLFM1	13137.2085	-0.4819352	0.04817915	-10.002983	1.48E-23	1.23E-21
ORC6	1053.19998	-0.3950709	0.09960901	-3.9662163	7.30E-05	0.00052528
ORMDL3	5161.00476	0.14281748	0.05731522	2.49178974	0.01271013	0.0459021
OSBPL2	5506.87119	0.31570844	0.05752888	5.48782506	4.07E-08	5.65E-07
P4HA1	3482.78721	-0.4652196	0.06530828	-7.1234408	1.05E-12	2.84E-11
PABPC4	10773.9286	-0.3483842	0.04931091	-7.0650523	1.61E-12	4.24E-11
PAICS	11333.5528	-0.2699206	0.04988419	-5.4109442	6.27E-08	8.45E-07
PAK2	33471.4621	0.14950895	0.04497253	3.32445039	0.00088593	0.00473642
PALM3	146.555051	0.68395863	0.25502301	2.68194866	0.00731947	0.02898461
PAPSS2	2863.81517	-0.2578463	0.06912968	-3.7298931	0.00019156	0.00123172
PARP1	20427.6117	-0.1333936	0.04699898	-2.8382239	0.00453653	0.01927104
PARP4	1375.92014	-0.3136565	0.08849051	-3.5445215	0.00039333	0.00233135
PAWR	5619.72638	-0.2306559	0.05637575	-4.0914034	4.29E-05	0.00032702
PBLD	1038.45581	0.29707225	0.1031797	2.87917343	0.00398719	0.01724891
PBRM1	3428.83141	-0.2464197	0.0721394	-3.4158827	0.00063576	0.00356583
PBX2	5017.71985	-0.4430557	0.05878525	-7.5368509	4.81E-14	1.54E-12
PBXIP1	5671.55511	0.35169078	0.05679662	6.19210763	5.94E-10	1.12E-08
PCDH1	6708.09139	0.56521133	0.05591142	10.10905	5.04E-24	4.27E-22
PCDHA1__PCDHA10_	7458.41401	0.22272253	0.05397826	4.12615255	3.69E-05	0.00028658
PCGF5	2906.14936	-0.2057887	0.06778657	-3.0358328	0.00239872	0.01108323
PCNA	10585.6905	-0.4003629	0.05421413	-7.384845	1.53E-13	4.66E-12
PCNP	7461.8795	-0.2222008	0.05442855	-4.0824314	4.46E-05	0.00033806
PCNT	6723.56855	0.26125158	0.06985505	3.73990986	0.00018409	0.0011881
PCP4L1	783.469897	0.62373467	0.11185563	5.57624763	2.46E-08	3.53E-07
PDCD4-AS1	281.317496	1.13230094	0.18711392	6.05139867	1.44E-09	2.54E-08
PDGFA	667.140566	0.46174208	0.12086803	3.82021692	0.00013333	0.00089802
PDHA1	7652.45511	-0.2952364	0.05623273	-5.2502596	1.52E-07	1.91E-06

PDHB	5903.35695	-0.1746621	0.06252611	-2.7934268	0.00521528	0.02172341
PDK1	890.661269	-0.6513571	0.10973864	-5.9355311	2.93E-09	4.91E-08
PFAS	1733.6536	-0.5873915	0.08377058	-7.0119068	2.35E-12	6.12E-11
PFKFB4__UCN2	1242.72043	-0.5348375	0.09167643	-5.8339697	5.41E-09	8.62E-08
PFKP	10384.4218	-0.1296258	0.04933971	-2.6272097	0.00860883	0.03331632
PGK1	24432.9344	-0.5399012	0.04820849	-11.199297	4.11E-29	4.90E-27
PGPEP1	1046.57855	-0.3068266	0.0981691	-3.125491	0.00177508	0.00860895
PGR	49373.0068	-0.837502	0.05729609	-14.61709	2.19E-48	7.77E-46
PGRMC2	6607.15446	0.33046889	0.05701853	5.79581523	6.80E-09	1.07E-07
PHACTR1	2329.18155	-0.641524	0.07257884	-8.838994	9.66E-19	5.41E-17
PHB	7264.87088	-0.1554851	0.06062923	-2.5645234	0.01033176	0.03874526
PHC2	1807.83816	0.19558469	0.07914663	2.47116876	0.01346722	0.04811616
PHF20	8647.2459	0.34749314	0.05300997	6.55524146	5.56E-11	1.18E-09
PHLDA1	3149.08192	-0.4730598	0.06556443	-7.2151906	5.39E-13	1.50E-11
PHLDA3	2080.43794	0.53765947	0.08018637	6.7051229	2.01E-11	4.53E-10
PHYH	3883.22985	-0.2065293	0.06275443	-3.2910704	0.00099807	0.00526057
PIEZO1	21210.7921	0.14696523	0.05650143	2.60108854	0.00929285	0.03542087
PIGA	1012.64178	-0.2750908	0.10123859	-2.7172527	0.00658263	0.02651947
PIH1D1	1413.80374	-0.2991779	0.0929216	-3.2196813	0.00128333	0.00654919
PIK3C2A__RPS13	15504.4808	-0.1802672	0.0524548	-3.4366202	0.00058902	0.00333845
PIK3CB	5504.30842	-0.2459749	0.05942458	-4.1392792	3.48E-05	0.00027244
PIK3IP1	783.942384	0.91781067	0.11447398	8.01763539	1.08E-15	4.28E-14
PIK3R3__RP11-322N1	3912.14184	0.48352584	0.064392	7.50909818	5.95E-14	1.89E-12
PIM3	2114.31837	0.33089549	0.08314653	3.97966675	6.90E-05	0.00050009
PINK1	512.883665	0.34505101	0.13454725	2.56453413	0.01033144	0.03874526
PITPNA	4713.07647	-0.3850258	0.05840662	-6.59216	4.33E-11	9.33E-10
PITX1	6512.55187	0.1908346	0.06171321	3.09228136	0.00198624	0.00946672
PJA2	9024.02716	-0.2269477	0.05534878	-4.1003208	4.13E-05	0.00031611
PKM	68118.0739	-0.2174386	0.04330048	-5.0216197	5.12E-07	5.91E-06
PKN2	3401.09057	-0.3961257	0.06647363	-5.959141	2.54E-09	4.30E-08
PKP3	5894.06808	0.46167516	0.0579989	7.96006693	1.72E-15	6.71E-14
PLCB1	2165.69254	0.26705714	0.07977926	3.34745089	0.00081558	0.00440294
PLCH1	1203.57392	0.28443748	0.10117624	2.81130712	0.00493407	0.02073823
PLEKHA1	1394.45539	-0.2369163	0.08853164	-2.6760638	0.00744925	0.02940093
PLEKHA2	2029.43991	-0.3606249	0.07583289	-4.7555209	1.98E-06	2.04E-05
PLEKHA5	1089.90344	-0.3700898	0.0981197	-3.7718191	0.00016206	0.00106029
PLEKHF1	615.337159	0.59530858	0.12870239	4.62546651	3.74E-06	3.69E-05
PLEKHH1	3043.64604	-0.3903182	0.07117955	-5.4835719	4.17E-08	5.77E-07
PLEKHH3	5154.48575	0.24542855	0.05984043	4.10138329	4.11E-05	0.00031481
PLEKHM1	1940.39727	0.2459149	0.07989873	3.07783241	0.00208512	0.00988022
PLOD2	10836.9948	-0.1883649	0.04956316	-3.8005028	0.0001444	0.00096209
PLP2	8563.68432	-0.1655608	0.05335875	-3.1027856	0.00191708	0.00919881
PLSCR1	1214.92417	-0.6656968	0.09772766	-6.8117541	9.64E-12	2.28E-10

PLXNB2	26938.3716	0.16952484	0.05293821	3.20231549	0.00136328	0.00688564
PLXNB3__SRPK3	2442.3596	0.25660017	0.07371829	3.4808208	0.00049988	0.00287832
PMEPA1	3595.118	0.49575325	0.06338819	7.82090915	5.24E-15	1.89E-13
PNMA1	6468.75844	-0.1473057	0.05600011	-2.6304547	0.00852707	0.03305354
PNPLA2	5960.59236	0.48668082	0.05691311	8.55129608	1.22E-17	6.09E-16
PNPLA7	762.247114	0.51106761	0.1237804	4.12882515	3.65E-05	0.0002838
PNRC1	2630.08211	0.34256612	0.07079488	4.83885428	1.31E-06	1.39E-05
PODXL2	2127.56057	0.45844208	0.07718871	5.93923773	2.86E-09	4.81E-08
POLA2	3143.95419	-0.4410454	0.06637501	-6.6447506	3.04E-11	6.70E-10
POLD2	8260.99697	-0.3986829	0.05179188	-7.6977885	1.38E-14	4.68E-13
POLE	6670.03109	-0.1811091	0.06137893	-2.9506728	0.00317083	0.01417621
POLE2	1028.50285	-0.2845028	0.09899845	-2.8738101	0.00405553	0.01749922
POLR1B	2154.38842	0.2660788	0.07684575	3.46250529	0.00053517	0.00306129
POMGNT2	1792.09883	-0.2606578	0.08121345	-3.2095396	0.00132948	0.00674143
PP14571	1187.87129	0.69813929	0.09534596	7.32216977	2.44E-13	7.19E-12
PPDPF	14805.7874	0.22553861	0.05263544	4.28491909	1.83E-05	0.00015275
PPFIA3	3504.78163	0.33430576	0.06447804	5.1848004	2.16E-07	2.65E-06
PPFIA4	333.095042	-1.4981729	0.17022981	-8.8008845	1.36E-18	7.48E-17
PPIA	78560.1008	-0.1436954	0.050847	-2.8260358	0.0047128	0.01989333
PPIG	4193.98113	-0.1911221	0.06052747	-3.1576091	0.00159069	0.00782369
PPIP5K2	3784.66933	-0.2256223	0.07527078	-2.9974758	0.00272225	0.0124065
PPL	5876.85742	0.23900591	0.06239428	3.83057388	0.00012784	0.00086418
PPP1CA	10884.3363	-0.1747565	0.05067547	-3.4485426	0.00056362	0.00320755
PPP1R10	5176.88156	0.22500514	0.05928776	3.79513613	0.00014756	0.00097924
PPP1R13L	5074.40047	0.16555243	0.05745365	2.88149547	0.00395793	0.01714453
PPP1R16A	2507.66575	0.41317587	0.07102358	5.81744664	5.98E-09	9.47E-08
PPP1R18	1695.52934	-0.459671	0.08798439	-5.2244613	1.75E-07	2.16E-06
PPP1R3F	464.407465	0.85661456	0.14311999	5.98528939	2.16E-09	3.72E-08
PPP2R1A	11188.0948	0.16717658	0.04942125	3.38268644	0.00071781	0.00396741
PPP2R1B	4148.09161	-0.2096704	0.0635342	-3.3001185	0.00096644	0.00512134
PPP2R2C	3101.14519	-0.1766069	0.06648234	-2.6564483	0.00789686	0.03090635
PPP2R5B	1273.73328	0.30025089	0.09306809	3.22614209	0.00125471	0.00643458
PREX1	32377.3462	-0.218944	0.04603708	-4.7558177	1.98E-06	2.04E-05
PRICKLE2	675.286836	0.40666038	0.1196768	3.39798833	0.00067883	0.00378456
PRKAA2	929.754935	-0.6054072	0.11134639	-5.4371518	5.41E-08	7.39E-07
PRLR	172966.743	-0.2355339	0.06042242	-3.8981208	9.69E-05	0.00067608
PRMT2	3023.68903	0.17868038	0.06897316	2.5905785	0.00958148	0.03638913
PRPF8	20506.482	-0.1978871	0.05456724	-3.6264818	0.00028731	0.00176561
PRPS2	3501.3707	-0.3348485	0.06348245	-5.2746631	1.33E-07	1.70E-06
PRPSAP1	3465.78154	-0.2696061	0.06372325	-4.2308899	2.33E-05	0.00019031
PRR7	509.745775	0.78855962	0.14913433	5.28757927	1.24E-07	1.59E-06
PRSS22	1633.33148	0.31710903	0.08714115	3.63902741	0.00027367	0.00169116
PRSS23	335.513038	-0.6101098	0.16518224	-3.6935557	0.00022114	0.00140072

PRSS53__RP11-196G	2958.38963	-0.2638921	0.07026333	-3.7557582	0.00017282	0.00112405
PSAP	30022.4105	0.29738408	0.04584598	6.48659004	8.78E-11	1.83E-09
PSMB3	6125.92409	-0.19275	0.06007183	-3.2086581	0.00133356	0.00675802
PSMG1	1682.35607	-0.2882942	0.08976908	-3.2115086	0.0013204	0.00670966
PTAR1	1796.39974	-0.2901211	0.08149499	-3.5599875	0.00037087	0.00220844
PTBP3	11065.2846	-0.2017099	0.0542037	-3.7213304	0.00019818	0.0012702
PTGES3	31785.9933	-0.1959936	0.04826415	-4.0608523	4.89E-05	0.00036721
PTK7	6911.49661	0.23082613	0.05350913	4.31377062	1.60E-05	0.00013608
PTMA	36956.3198	-0.1578214	0.04711367	-3.3497994	0.0008087	0.00437427
PTMS	3683.22792	0.44609412	0.08510232	5.2418562	1.59E-07	1.99E-06
PTP4A1	8453.13124	0.13235208	0.05235606	2.52792311	0.01147395	0.04220422
PTP4A2	25531.2007	-0.2321527	0.04740957	-4.8967473	9.74E-07	1.06E-05
PTPN18	1310.41184	-0.3862618	0.08981974	-4.3004107	1.70E-05	0.00014389
PTRF	1457.63948	-1.2258187	0.08919822	-13.742636	5.64E-43	1.38E-40
PVRL4	6389.52815	0.30519626	0.05542062	5.50690777	3.65E-08	5.12E-07
PWAR6	369.950175	-0.3909479	0.15702153	-2.4897726	0.01278249	0.0460539
PWP1	4720.6352	-0.1536438	0.06006061	-2.5581461	0.01052319	0.03931308
PWWP2B	1233.34084	0.32907385	0.09479856	3.47129575	0.00051795	0.00297502
PXN	14116.1969	0.20722361	0.04827479	4.29258419	1.77E-05	0.00014846
QSER1	5648.54963	-0.3135246	0.07132342	-4.395815	1.10E-05	9.79E-05
QSOX1	9003.71831	-0.1520844	0.05159062	-2.9479084	0.00319932	0.01429031
QSOX2	1717.81171	0.33460073	0.08242009	4.05969852	4.91E-05	0.0003687
RAB15	4405.87088	0.52093747	0.05964519	8.73393879	2.46E-18	1.33E-16
RAB27B	2557.67905	0.48768875	0.07911767	6.1640941	7.09E-10	1.31E-08
RAB6B	2297.16317	-0.2297164	0.0800578	-2.8693823	0.00411274	0.01769126
RAB9A	1683.73549	0.28191028	0.08855486	3.18345338	0.0014553	0.00727778
RAD18	2086.16452	-0.3857048	0.08184894	-4.7123984	2.45E-06	2.47E-05
RAD21	22738.823	-0.3517685	0.04527136	-7.7702214	7.83E-15	2.76E-13
RAD23A	3320.56773	-0.3831672	0.06579339	-5.8237952	5.75E-09	9.13E-08
RAD51	1590.16675	-0.3712645	0.08506862	-4.3642936	1.28E-05	0.00011083
RAET1E-AS1	255.882519	1.3180598	0.2190247	6.01785913	1.77E-09	3.09E-08
RAPGEFL1	9395.38037	-0.6013332	0.05040526	-11.92997	8.26E-33	1.22E-30
RAVER2	1543.06738	-0.2735415	0.08903295	-3.0723624	0.00212372	0.01004885
RB1	4717.97617	-0.2524402	0.0630655	-4.0028253	6.26E-05	0.00046035
RBBP7	22481.8779	-0.1965754	0.04716856	-4.1675086	3.08E-05	0.00024412
RBBP8	6316.10317	-0.6026308	0.05698653	-10.57497	3.89E-26	3.92E-24
RBL1	1682.45417	-0.2769203	0.0854481	-3.2408011	0.00119194	0.00615426
RBM22	3995.35349	-0.2772576	0.06205586	-4.467872	7.90E-06	7.25E-05
RDX	8688.91115	-0.465378	0.05485439	-8.483879	2.18E-17	1.05E-15
RECQL	3721.22715	-0.4108768	0.06291573	-6.5305903	6.55E-11	1.38E-09
REST	4238.39106	-0.2825712	0.06293961	-4.4895612	7.14E-06	6.63E-05
RFC1	4455.3406	-0.3118037	0.06362381	-4.9007401	9.55E-07	1.04E-05
RFC2	3865.08081	-0.2102031	0.06452027	-3.2579395	0.00112224	0.00583224

RFC3	2781.31653	-0.5566552	0.06930114	-8.032411	9.56E-16	3.82E-14
RFC4	4980.60317	-0.4293316	0.05829841	-7.3643787	1.78E-13	5.39E-12
RHBDF2	1755.36492	-0.2585796	0.08147883	-3.1735805	0.00150571	0.00747841
RHOA	15370.9486	-0.1725861	0.04769841	-3.6182782	0.00029657	0.00181384
RHOB	16625.0695	0.59936888	0.0570236	10.5108908	7.70E-26	7.52E-24
RHOV	3468.43654	0.44155003	0.07193076	6.1385429	8.33E-10	1.53E-08
RHPN1	5670.38449	0.33869467	0.05961084	5.68176312	1.33E-08	1.99E-07
RLIM	2223.80988	-0.4179205	0.08524416	-4.9026284	9.46E-07	1.04E-05
RLTPR	167.529134	-0.6451094	0.22825918	-2.8262147	0.00471017	0.01988725
RMND5A	4817.93479	0.26644101	0.05817065	4.58033433	4.64E-06	4.47E-05
RMND5B	4073.46444	0.3286265	0.06197884	5.30223682	1.14E-07	1.48E-06
RMRP	68.3987522	-2.3051283	0.41642336	-5.5355403	3.10E-08	4.40E-07
RNASEH2A	2272.28128	-0.4329728	0.07634695	-5.6711207	1.42E-08	2.11E-07
RNF145	5931.97653	-0.2564424	0.05635681	-4.5503354	5.36E-06	5.12E-05
RNF168	4253.79078	-0.2957591	0.06541381	-4.5213556	6.14E-06	5.77E-05
RNF222	96.7683539	0.80129198	0.30657396	2.61369873	0.0089568	0.03439204
RNF223	1251.75087	0.31076452	0.09763848	3.18280796	0.00145854	0.00728966
RNF26	6833.03782	-0.2695049	0.05443412	-4.9510284	7.38E-07	8.26E-06
RNF7	3294.91864	0.20015857	0.07077812	2.8279724	0.00468438	0.01980341
ROBO1	5491.941	-0.363867	0.06356272	-5.7245342	1.04E-08	1.58E-07
RP1-239B22.5	125.025143	-0.692508	0.28088215	-2.4654751	0.01368317	0.04876252
RP1-257A7.5	238.43961	-0.6429974	0.19886844	-3.2332802	0.00122377	0.00629526
RP1-41C23.2	318.851572	-0.4403286	0.16945304	-2.5985286	0.00936243	0.03563012
RP11-126H7.4	203.102042	-0.7570054	0.21265805	-3.5597309	0.00037124	0.00220981
RP11-127O4.3	51.0008404	1.93667352	0.44953986	4.30812411	1.65E-05	0.0001391
RP11-148K1.12	758.904352	0.29515964	0.11313041	2.60902119	0.00908016	0.03476185
RP11-211G3.2	65.0794911	-1.6045712	0.40207797	-3.9906966	6.59E-05	0.00048073
RP11-316M1.12	6011.16866	0.16922014	0.05980609	2.82948026	0.00466237	0.01972531
RP11-342K6.1	199.991207	-0.7105657	0.22469832	-3.16231	0.00156523	0.00773647
RP11-343C2.11__SN	6312.16389	-0.3302866	0.05683984	-5.8108301	6.22E-09	9.80E-08
RP11-352M15.2	1134.21599	0.4162457	0.0958312	4.34353028	1.40E-05	0.00012047
RP11-386G11.10	31445.6585	-0.1261054	0.04934987	-2.5553331	0.01060862	0.03956147
RP11-400F19.6	3481.99613	0.39010101	0.06461964	6.03688034	1.57E-09	2.76E-08
RP11-400K9.4	578.570948	1.20959212	0.13440194	8.99981159	2.26E-19	1.34E-17
RP11-402D21.2	29.2921851	1.51475378	0.56086466	2.70074742	0.00691839	0.02761567
RP11-442H21.2	1049.12598	0.57754419	0.0992901	5.81673497	6.00E-09	9.48E-08
RP11-469A15.2	347.351609	2.36720068	0.18103731	13.0757613	4.53E-39	8.80E-37
RP11-47A8.5	195.118271	0.61399029	0.24306529	2.52603034	0.01153595	0.04238561
RP11-54O7.3	896.063966	0.87050758	0.10733454	8.11022786	5.05E-16	2.09E-14
RP11-580I16.2	1178.83365	0.48256345	0.09631225	5.01040577	5.43E-07	6.23E-06
RP11-618K13.2	171.649799	0.68044223	0.24289612	2.80137133	0.00508859	0.02129661
RP11-89K21.1	644.342024	0.34088804	0.13246685	2.57338377	0.01007095	0.03792898
RP4-568C11.4	1118.00426	0.74858642	0.09642861	7.76311499	8.29E-15	2.90E-13

RP4-734P14.4__SNR	14746.6419	-0.1263314	0.05122145	-2.4663777	0.01364873	0.04870216
RP5-850E9.3__SCRT2	4266.48318	0.25768879	0.06105376	4.22068659	2.44E-05	0.00019835
RP5-899B16.1	358.661753	-0.8000856	0.15944983	-5.0177889	5.23E-07	6.02E-06
RPA1	7791.43697	-0.7459363	0.05213202	-14.308604	1.93E-46	5.98E-44
RPA3	2067.29713	-0.3122369	0.08272588	-3.7743562	0.00016042	0.00105162
RPL11	25846.6329	-0.2994037	0.06174146	-4.8493137	1.24E-06	1.32E-05
RPL14	32321.9975	-0.3588403	0.04561551	-7.8666295	3.64E-15	1.35E-13
RPL15	31589.3194	-0.2960479	0.0485617	-6.0963261	1.09E-09	1.95E-08
RPL18	16641.4517	-0.3164782	0.05296946	-5.9747284	2.30E-09	3.95E-08
RPL18A	12605.513	-0.2939939	0.05231266	-5.619938	1.91E-08	2.79E-07
RPL19	45367.8896	-0.3892673	0.05197411	-7.4896377	6.91E-14	2.18E-12
RPL22	13072.6311	-0.2792626	0.05730948	-4.872886	1.10E-06	1.18E-05
RPL23	36029.8153	-0.3427963	0.05726223	-5.9864293	2.14E-09	3.70E-08
RPL23A	27542.7378	-0.3646122	0.05405331	-6.7454181	1.53E-11	3.51E-10
RPL24	22470.431	-0.2820926	0.0554036	-5.0915929	3.55E-07	4.23E-06
RPL27	25739.4827	-0.2959055	0.05975889	-4.9516559	7.36E-07	8.24E-06
RPL27A	12256.5381	-0.2110354	0.06476309	-3.2585746	0.00111973	0.00582102
RPL28	16969.1937	-0.3974779	0.05989297	-6.6364696	3.21E-11	7.07E-10
RPL29	16907.5151	-0.2606323	0.04866171	-5.3560042	8.51E-08	1.12E-06
RPL3	62402.2089	-0.2355546	0.0470011	-5.0116832	5.40E-07	6.19E-06
RPL31	15936.198	-0.2972461	0.05977526	-4.9727282	6.60E-07	7.44E-06
RPL32	34768.3349	-0.3744627	0.0613792	-6.1008072	1.06E-09	1.91E-08
RPL35	19575.6745	-0.5430598	0.0577454	-9.4043825	5.23E-21	3.55E-19
RPL35A	38856.746	-0.310196	0.12449509	-2.4916325	0.01271575	0.0459125
RPL37	25072.8542	-0.33065	0.124876	-2.6478266	0.00810111	0.03159625
RPL37A	21239.2099	-0.3088927	0.06349996	-4.8644558	1.15E-06	1.23E-05
RPL39	11233.654	-0.3540483	0.07032116	-5.0347342	4.79E-07	5.55E-06
RPL4	61193.271	-0.3668612	0.04548255	-8.0659765	7.27E-16	2.97E-14
RPL6	39197.4313	-0.2932848	0.0472275	-6.2100424	5.30E-10	1.00E-08
RPL7	43925.9281	-0.3720661	0.05648166	-6.5873801	4.48E-11	9.61E-10
RPL7A	78462.4484	-0.3993779	0.04422903	-9.0297692	1.72E-19	1.03E-17
RPL8	67612.1615	-0.3542822	0.05126327	-6.9110348	4.81E-12	1.19E-10
RPL9	22455.3571	-0.362463	0.05472188	-6.6237304	3.50E-11	7.67E-10
RPL9P9	195.404767	-0.7331191	0.2167876	-3.3817391	0.00072029	0.00397919
RPLP0	74155.7021	-0.4777411	0.04634299	-10.308812	6.43E-25	5.97E-23
RPS11	19882.9346	-0.143261	0.05447619	-2.6297908	0.00854374	0.03311047
RPS12	23236.2582	-0.1993829	0.05843269	-3.4121797	0.00064446	0.00361099
RPS14	45039.6875	-0.2891279	0.05662264	-5.1062254	3.29E-07	3.93E-06
RPS16	15134.3774	-0.2745928	0.06548217	-4.1933985	2.75E-05	0.00022015
RPS18	61315.2378	-0.311703	0.05198989	-5.9954541	2.03E-09	3.51E-08
RPS19	22873.643	-0.2759245	0.05796212	-4.7604275	1.93E-06	1.99E-05
RPS20	23129.2815	-0.3234221	0.05579335	-5.7967863	6.76E-09	1.06E-07
RPS24	47129.9918	-0.4021079	0.05721131	-7.0284685	2.09E-12	5.47E-11

RPS25	25561.0207	-0.271488	0.06147641	-4.4161326	1.00E-05	9.00E-05
RPS26	7217.58492	-0.2306832	0.05904419	-3.9069587	9.35E-05	0.00065457
RPS27	16836.9829	-0.3420548	0.06539821	-5.2303383	1.69E-07	2.11E-06
RPS27A	17258.2839	-0.2949045	0.06518607	-4.5240425	6.07E-06	5.72E-05
RPS3	62712.1425	-0.3178092	0.0534355	-5.947529	2.72E-09	4.60E-08
RPS3A	21574.2087	-0.3528081	0.05252283	-6.7172329	1.85E-11	4.19E-10
RPS5	18809.8111	-0.2040531	0.05011563	-4.0716463	4.67E-05	0.00035282
RPS6KA5	1270.58597	0.38187869	0.09452918	4.03979688	5.35E-05	0.00039945
RPS7	19178.9095	-0.2763418	0.05478805	-5.0438336	4.56E-07	5.31E-06
RPS8	30039.8069	-0.2536864	0.05287832	-4.7975506	1.61E-06	1.67E-05
RPS9	16547.4934	-0.2293622	0.0541109	-4.2387434	2.25E-05	0.00018476
RPSA	33593.9037	-0.2765501	0.04832912	-5.7222252	1.05E-08	1.60E-07
RQCD1	2525.43697	-0.4837354	0.07196255	-6.7220432	1.79E-11	4.06E-10
RRM2	10768.7781	-0.1799193	0.05043959	-3.5670245	0.00036106	0.00215615
RRM2B	3610.2884	-0.2227448	0.06332083	-3.5177175	0.00043528	0.00255102
RSPH1	293.032932	0.76225033	0.19080656	3.99488543	6.47E-05	0.00047438
RTKN2	1558.19912	-0.2087579	0.08385184	-2.4896042	0.01278854	0.04605897
RWDD3__TMEM56_	2505.85408	-0.221628	0.0711808	-3.1135923	0.00184825	0.0089146
RXRA	7749.35685	0.23488292	0.05600346	4.19407858	2.74E-05	0.0002197
S100A11	18357.0059	0.24371113	0.06159818	3.95646655	7.61E-05	0.0005453
SACS	839.449424	-0.416396	0.11609923	-3.5865524	0.00033508	0.00202417
SAMD11	887.655626	0.54581767	0.10819039	5.04497383	4.54E-07	5.29E-06
SAMD9	439.087943	-0.7142864	0.16227195	-4.4017863	1.07E-05	9.54E-05
SAMD9L	117.16486	-0.7957528	0.27291727	-2.9157291	0.00354858	0.01566032
SAMHD1	3183.18603	-0.3102566	0.06771862	-4.5815554	4.62E-06	4.45E-05
SAPCD2	6754.70653	0.22686732	0.06078633	3.7322097	0.00018981	0.00122172
SATB2	563.607018	-0.7587699	0.13196684	-5.7497014	8.94E-09	1.38E-07
SBNO2	4347.81373	0.3139459	0.06250013	5.02312401	5.08E-07	5.86E-06
SC5D	6789.58017	0.43348893	0.05582608	7.76498957	8.17E-15	2.87E-13
SCAND1	2576.34813	0.39499355	0.07542865	5.23665106	1.64E-07	2.04E-06
SCARA3	2349.85127	-0.9462579	0.07350856	-12.872757	6.41E-38	1.16E-35
SCD	51189.0957	-0.4758483	0.04289032	-11.094539	1.33E-28	1.54E-26
SCFD1	4406.3917	0.36011146	0.06158132	5.84773809	4.98E-09	7.96E-08
SCNN1G	196.439612	-0.974247	0.21573432	-4.5159576	6.30E-06	5.91E-05
SCYL1	6426.45641	-0.4743875	0.05619411	-8.441942	3.12E-17	1.46E-15
SCYL3	1718.60337	0.51354678	0.08253924	6.22184994	4.91E-10	9.36E-09
SDC1	3638.01152	0.38756509	0.06353505	6.10001982	1.06E-09	1.91E-08
SDC3	1406.62073	0.49419125	0.08970533	5.509051	3.61E-08	5.06E-07
SDC4	10008.0509	0.3470957	0.05156048	6.73181663	1.68E-11	3.83E-10
SDF4	7709.59707	0.29017701	0.05941262	4.88409692	1.04E-06	1.13E-05
SDHC	13195.6317	-0.2954741	0.05129006	-5.760845	8.37E-09	1.29E-07
SEC22C	4493.69839	-0.2247068	0.06102937	-3.6819449	0.00023146	0.00145836
SEMA3C	22157.4492	-0.1270726	0.05089043	-2.4969847	0.01252544	0.04538229

SEMA3F	8681.63891	-0.2110956	0.05137518	-4.1089027	3.98E-05	0.00030614
SEPW1	3956.95005	-0.1831337	0.0679364	-2.6956635	0.00702486	0.02795051
SERBP1	14618.2659	-0.2691329	0.04741434	-5.6761922	1.38E-08	2.05E-07
SERPINI1	887.071142	0.55195928	0.10873918	5.07599246	3.85E-07	4.56E-06
SERTAD1	1085.26389	0.31573332	0.10306399	3.06346881	0.00218787	0.01028803
SERTAD4	3105.30459	-0.6515874	0.06765567	-9.6309365	5.92E-22	4.43E-20
SESN2	1903.96162	0.6124987	0.08191671	7.4770909	7.60E-14	2.39E-12
SET	34101.8945	-0.1699373	0.04734968	-3.5889859	0.00033197	0.00200973
SETD7	7769.91114	0.20300039	0.06127184	3.31311084	0.00092264	0.00491263
SETX	8429.48434	-0.2614538	0.06636098	-3.9398724	8.15E-05	0.00057945
SEZ6L2	7516.86329	0.333858	0.05263339	6.3430834	2.25E-10	4.48E-09
SF3B3	14966.2751	-0.4906402	0.04839429	-10.138389	3.73E-24	3.21E-22
SFXN3	2255.82274	-0.5047455	0.07355596	-6.8620614	6.79E-12	1.65E-10
SGPP1	2615.09244	0.25778745	0.06970933	3.69803368	0.00021728	0.00137957
SH2D3A	1393.43593	0.24399688	0.08791749	2.77529402	0.00551518	0.02279636
SH3BP5L	2555.23174	0.30481823	0.07070704	4.31100242	1.63E-05	0.00013744
SH3D19	3675.11011	-0.301261	0.06284483	-4.7937269	1.64E-06	1.70E-05
SHC2	290.733651	1.43134552	0.18420586	7.7703584	7.83E-15	2.76E-13
SHISA8	51.8649986	1.95946845	0.45305855	4.32497844	1.53E-05	0.00012993
SIGMAR1	4508.32149	-0.5338209	0.06568097	-8.1274813	4.38E-16	1.82E-14
SKAP2	3893.58501	1.89331569	0.06478338	29.22533	9.24E-188	7.72E-184
SLC11A2	5845.06712	0.34812206	0.05558592	6.26277456	3.78E-10	7.27E-09
SLC12A7	5733.75366	0.40903221	0.05666412	7.21853946	5.25E-13	1.47E-11
SLC18B1	619.935857	-0.3943681	0.12415068	-3.1765281	0.00149049	0.00742269
SLC19A2	1503.22549	0.51907266	0.08885669	5.84168366	5.17E-09	8.24E-08
SLC25A36	5832.40752	-0.2741959	0.0561359	-4.8845022	1.04E-06	1.13E-05
SLC25A48	104.80631	-2.1123245	0.30471045	-6.9322352	4.14E-12	1.04E-10
SLC2A6	1714.52957	0.24563585	0.08388451	2.92826242	0.00340862	0.01512599
SLC35D2	659.400015	0.38505368	0.12654146	3.04290521	0.00234306	0.01086813
SLC37A1	1886.25032	0.39446799	0.07821618	5.04330408	4.58E-07	5.33E-06
SLC38A1	30330.8081	0.27641603	0.05262293	5.25276802	1.50E-07	1.88E-06
SLC38A10	5125.57252	0.18410466	0.0572628	3.21508289	0.00130407	0.00664081
SLC3A2	19059.3037	0.27767604	0.04784008	5.80425484	6.47E-09	1.02E-07
SLC46A1	2372.9089	-0.2126515	0.07304848	-2.9111012	0.00360157	0.01585585
SLC47A1	450.985213	-0.557476	0.14288715	-3.9015126	9.56E-05	0.0006678
SLC4A2	5306.29556	0.14475251	0.05813116	2.49010196	0.01277065	0.04604103
SLC5A6	5716.25712	0.39636202	0.05585312	7.09650669	1.28E-12	3.41E-11
SLC6A4	43.7269211	1.1606722	0.45527037	2.54941301	0.01079044	0.04014987
SLC6A6	4342.95496	-0.5378625	0.06369146	-8.4448129	3.05E-17	1.43E-15
SLC7A5	38118.0829	0.334667	0.04344656	7.7029569	1.33E-14	4.51E-13
SLC9A1	3271.51558	0.67614917	0.06640156	10.1827302	2.37E-24	2.09E-22
SLC9A8	1966.73501	-0.2447143	0.07730704	-3.1654847	0.00154825	0.00766461
SLITRK4	1518.96576	-0.2277187	0.08501709	-2.6785049	0.00739516	0.02923584

SMAD2	3493.63597	-0.2116611	0.06490848	-3.2609152	0.00111053	0.00578037
SMAD6	443.87608	0.46220291	0.14779777	3.12726586	0.0017644	0.00857208
SMC5	1490.13284	-0.3013331	0.08714274	-3.4579251	0.00054435	0.00311061
SMIM14	5638.86463	0.2933636	0.05616483	5.22326183	1.76E-07	2.18E-06
SNCB	88.7634283	-1.1632607	0.31714521	-3.6679118	0.00024454	0.00153151
SNHG1__SNORD22_	5869.76939	-0.4794664	0.05576138	-8.5985385	8.07E-18	4.09E-16
SNHG17__SNORA71	1099.8087	0.39778568	0.09679572	4.10953793	3.96E-05	0.00030572
SNRNP200	18741.1073	-0.2248119	0.05354855	-4.1982818	2.69E-05	0.00021648
SNRNP48	1360.78173	-0.3271438	0.08974805	-3.6451357	0.00026725	0.00165821
SNRPD2	4812.84739	-0.2010923	0.06809305	-2.953199	0.00314499	0.01407578
SNX10	1293.82709	-0.6563664	0.09410934	-6.9745085	3.07E-12	7.80E-11
SNX18	3216.37064	0.22338136	0.06517748	3.42727801	0.00060966	0.0034368
SOCS1	24.9066289	1.94210813	0.6252823	3.1059701	0.00189656	0.00911079
SOCS3	142.819907	-0.6301663	0.24587332	-2.5629716	0.01037805	0.03886651
SOD2	4859.41115	-0.3309227	0.05843541	-5.663052	1.49E-08	2.20E-07
SOD3	5001.03832	1.14760261	0.07721516	14.8623996	5.78E-50	2.15E-47
SORL1	18682.2094	0.50821987	0.06100128	8.33129814	8.00E-17	3.61E-15
SOWAHA	2570.53055	1.32235097	0.07834922	16.8776537	6.57E-64	3.92E-61
SOX12	4630.52711	0.18400731	0.05981426	3.07631158	0.00209579	0.00992513
SOX4	4481.17537	0.5300249	0.06965297	7.60950912	2.75E-14	8.99E-13
SPAG4	498.890111	-0.9170155	0.13848977	-6.62154	3.55E-11	7.76E-10
SPAG9	5556.7854	-0.3268493	0.05874677	-5.5636982	2.64E-08	3.76E-07
SPEG	468.575701	0.73158483	0.14090458	5.19205871	2.08E-07	2.55E-06
SPICE1	1458.31594	-0.6269785	0.08704617	-7.2028272	5.90E-13	1.63E-11
SPIDR	3253.79919	-0.1803301	0.06537894	-2.7582293	0.00581154	0.02380338
SPINT1	29837.391	0.41604855	0.04404964	9.4449931	3.55E-21	2.44E-19
SPIRE2	2620.5343	0.30513382	0.07274019	4.19484507	2.73E-05	0.00021927
SPNS2	1799.36325	0.74546794	0.08153247	9.14320328	6.06E-20	3.76E-18
SPRY1	285.841192	0.59889175	0.17754803	3.37312522	0.0007432	0.0040848
SRGAP2	4885.45733	-0.5397986	0.06106525	-8.8397024	9.60E-19	5.40E-17
SRGAP2C	508.574851	-0.3992078	0.14695106	-2.7166036	0.00659556	0.02655872
SRP54	7463.47977	-0.1485049	0.05565541	-2.668292	0.0076238	0.02998647
SRPK1	4981.87731	-0.2452478	0.0584944	-4.192672	2.76E-05	0.00022064
SRPK2	2939.00844	0.17957428	0.06935472	2.58921486	0.00961951	0.03650866
SRSF10	10532.4988	-0.1959306	0.04952113	-3.9565056	7.61E-05	0.0005453
SRSF11	8509.43125	-0.1935247	0.05112886	-3.785039	0.00015368	0.00101542
SSBP1	3658.6343	-0.2369115	0.06441622	-3.677823	0.00023523	0.00147877
SSH3	6296.25669	0.19722926	0.05493916	3.58995739	0.00033073	0.00200298
SSR3	12243.6099	-0.2232087	0.04857	-4.5956077	4.31E-06	4.19E-05
SSRP1	16617.372	-0.317869	0.04698182	-6.7657861	1.33E-11	3.08E-10
ST13	16141.6982	-0.1965197	0.04782584	-4.1090697	3.97E-05	0.00030606
STAG3L3	1078.65755	0.34196545	0.09768955	3.50053248	0.00046433	0.00270045
STARD4	1420.28283	-0.378442	0.08828391	-4.286647	1.81E-05	0.00015172

STAT1	7299.14437	-0.4415367	0.05273491	-8.372758	5.63E-17	2.58E-15
STAT6	7889.84537	0.19843398	0.0527571	3.76127515	0.00016905	0.00110169
STAU1	11847.5807	-0.4714933	0.04956989	-9.5116884	1.88E-21	1.35E-19
STC1	3611.63319	-0.3556399	0.06420459	-5.5391667	3.04E-08	4.31E-07
STC2	69549.4629	-0.7826147	0.04462496	-17.537602	7.40E-69	5.62E-66
STK17A	2054.78523	0.20588692	0.0763712	2.69587128	0.00702048	0.02793973
STK24	5145.2107	-0.5262441	0.05916784	-8.8940886	5.89E-19	3.37E-17
STMN1	13154.0549	-0.4234971	0.05306422	-7.980842	1.45E-15	5.73E-14
STMN3	223.786099	1.74163466	0.22049995	7.89857167	2.82E-15	1.06E-13
STOM	7422.75009	0.43271602	0.05368454	8.0603473	7.61E-16	3.09E-14
SUB1	7713.44652	-0.2323646	0.05889894	-3.9451397	7.98E-05	0.00056857
SUMO3	9461.72691	0.28637896	0.05734555	4.99391775	5.92E-07	6.72E-06
SYN1	327.946835	-0.4444365	0.17409667	-2.5528145	0.01068564	0.03979537
SYNPO	971.985468	0.56590815	0.10193682	5.55155762	2.83E-08	4.02E-07
SYP	442.176875	2.05226055	0.16382229	12.5273585	5.29E-36	8.66E-34
SYTL1	2579.03196	0.21949666	0.07045381	3.11546919	0.00183653	0.00887344
TACC1	8526.99846	-0.3897428	0.05808583	-6.709773	1.95E-11	4.40E-10
TACSTD2	16256.4727	0.37612567	0.05516748	6.81788765	9.24E-12	2.19E-10
TAGLN2	27291.1285	0.27212681	0.04765105	5.71082523	1.12E-08	1.70E-07
TAOK3	3348.03984	0.41113804	0.06645784	6.18644888	6.15E-10	1.15E-08
TARS	6142.13663	0.45400857	0.05506616	8.24478345	1.65E-16	7.18E-15
TAX1BP1	13075.4659	0.1265683	0.04789462	2.64264111	0.00822622	0.03202856
TBC1D13	2136.97091	-0.3782145	0.07568353	-4.997316	5.81E-07	6.62E-06
TBC1D4	1284.93708	0.41784802	0.0918662	4.54844124	5.40E-06	5.16E-05
TBC1D8	327.131265	0.55789778	0.16857053	3.30958073	0.00093436	0.00497025
TBC1D9	2199.48101	0.4292174	0.07817076	5.49076627	4.00E-08	5.56E-07
TBX1	391.981131	0.54736389	0.16216006	3.37545443	0.00073694	0.00405781
TCAF1	7275.88513	0.30829177	0.05386488	5.72342791	1.04E-08	1.59E-07
TCAIM	1713.3216	0.30750256	0.08317911	3.69687235	0.00021827	0.00138466
TCEB2	4652.73398	-0.2201442	0.07036597	-3.1285609	0.00175665	0.00854932
TCTN1	3856.56091	0.16658392	0.06289217	2.64872262	0.00807966	0.03154115
TCTN3	5861.09937	-0.1691651	0.05543685	-3.0514928	0.00227707	0.01063883
TEAD2	2116.68244	0.27015331	0.07486973	3.60831164	0.0003082	0.00188013
TECR	4905.29176	-0.1999619	0.05829995	-3.4298814	0.00060385	0.00341205
TEF	775.996301	0.54967284	0.11547083	4.76027426	1.93E-06	1.99E-05
TESK2	1025.61656	0.80305172	0.10126102	7.93051205	2.18E-15	8.36E-14
TEX14	357.607058	1.26604164	0.16584924	7.63368972	2.28E-14	7.52E-13
TFAP2A	10933.3725	0.17479391	0.05021652	3.4808047	0.00049991	0.00287832
TGFB1	713.003737	0.34791012	0.11607885	2.99718797	0.00272483	0.01241483
TGFBR3	289.541686	-0.5603691	0.18110596	-3.0941504	0.00197377	0.00943019
THBS1	33795.4462	-0.1650925	0.05603651	-2.9461602	0.00321746	0.01436162
THRB	2009.43007	0.35377941	0.07936648	4.45754207	8.29E-06	7.55E-05
THRIL	2502.17333	-0.4446848	0.08489994	-5.2377519	1.63E-07	2.03E-06

THSD7A	144.121544	1.21834506	0.27047867	4.50440345	6.66E-06	6.22E-05
THUMPD3	4294.8782	-0.1633148	0.06015135	-2.7150639	0.0066263	0.02666966
TLE1	2997.81413	0.58214408	0.06810198	8.54812318	1.25E-17	6.24E-16
TLE3	4657.75515	0.21708329	0.06204717	3.49868132	0.00046757	0.00271549
TLK1	2842.43146	0.18593746	0.06821715	2.72567017	0.00641711	0.02593394
TLR5	410.426025	-0.7025083	0.15207267	-4.6195565	3.85E-06	3.78E-05
TM7SF2__VPS51	13479.239	0.24021833	0.05458524	4.40079246	1.08E-05	9.58E-05
TMED2	31360.7171	-0.2397528	0.04507592	-5.3188661	1.04E-07	1.36E-06
TMED4	8128.68131	-0.1517917	0.05937694	-2.5564089	0.01057587	0.0394746
TMEM123	17126.1157	-0.1778994	0.04598944	-3.8682657	0.00010961	0.00075189
TMEM132A	15116.4258	0.34963552	0.04778071	7.31750438	2.53E-13	7.40E-12
TMEM159	3672.10879	0.26906534	0.06375238	4.22047526	2.44E-05	0.00019844
TMEM165	2926.59731	0.36335223	0.0737496	4.92683652	8.36E-07	9.28E-06
TMEM167A	8708.92681	-0.5268484	0.05143877	-10.242245	1.28E-24	1.16E-22
TMEM184A	6472.45045	0.57311266	0.0559554	10.2423119	1.28E-24	1.16E-22
TMEM209	1604.76798	-0.2090252	0.08269516	-2.5276601	0.01148255	0.04221725
TMEM237	1473.87522	-0.2567327	0.09058485	-2.8341684	0.00459451	0.01947769
TMEM238	234.55014	1.88533758	0.55308324	3.4087773	0.00065255	0.00365387
TMEM243	835.591625	-0.3614878	0.11217691	-3.2224795	0.00127086	0.00650145
TMEM245	9339.8887	0.15353535	0.05290314	2.90219722	0.00370555	0.01623232
TMEM259	5425.95252	0.19893315	0.05837484	3.40785752	0.00065475	0.00366253
TMEM57	1748.13529	0.21530937	0.08076681	2.66581492	0.0076802	0.03016278
TMEM59	9520.69678	0.18952529	0.05218438	3.63183982	0.00028141	0.00173381
TMEM65	1458.18136	-0.2653822	0.08585032	-3.09122	0.00199336	0.00948578
TMEM80	1248.06327	0.31953212	0.0946407	3.37626538	0.00073477	0.00405045
TMEM86A	888.203138	1.19014929	0.11328053	10.5062125	8.09E-26	7.85E-24
TMEM97	4511.70965	-0.3604034	0.06104258	-5.9041317	3.55E-09	5.85E-08
TMOD3	3858.39556	-0.2591887	0.06419548	-4.0374917	5.40E-05	0.00040321
TMPO	17549.193	-0.3318845	0.04600355	-7.2143231	5.42E-13	1.51E-11
TMTC2	1582.04106	0.28014189	0.08834556	3.17097872	0.00151926	0.00753899
TMX1	5669.09764	-0.2695152	0.05788874	-4.6557454	3.23E-06	3.20E-05
TNFRSF12A	2501.09189	0.53771693	0.09490403	5.66590173	1.46E-08	2.17E-07
TNNC1	223.12829	-0.7811	0.20950984	-3.7282258	0.00019283	0.00123785
TNPO1	9939.52453	-0.1720767	0.05161189	-3.3340514	0.00085591	0.00458801
TNRC18	18322.725	0.34107371	0.0554935	6.14619207	7.94E-10	1.46E-08
TOB1	23554.5816	-0.4306646	0.04679849	-9.2025328	3.50E-20	2.20E-18
TOLLIP	2135.92252	0.29986452	0.07687306	3.90077539	9.59E-05	0.00066927
TOM1L2	3917.95772	0.22513934	0.07139309	3.1535172	0.00161316	0.00792021
TOMM20	19836.7674	-0.3197787	0.04629487	-6.907432	4.94E-12	1.22E-10
TOMM22	2883.36624	-0.2099655	0.07391641	-2.8405803	0.00450315	0.01913897
TOP2A	34988.4899	-0.4965702	0.04902891	-10.12811	4.15E-24	3.55E-22
TOP3A	4100.76813	-0.1861013	0.06169425	-3.0165094	0.00255703	0.01171869
TOPBP1	6996.8906	-0.3533279	0.06015237	-5.873881	4.26E-09	6.94E-08

TOR1AIP2	6669.84737	-0.4376696	0.05536963	-7.9045063	2.69E-15	1.02E-13
TP53I11	12844.4976	0.37991183	0.04918095	7.7247766	1.12E-14	3.83E-13
TP53I3	10988.8962	0.64328528	0.05753902	11.1799825	5.11E-29	6.05E-27
TPBG	6432.9952	-0.1928858	0.05970985	-3.2303856	0.00123623	0.00635153
TPI1	10374.9091	-0.1804892	0.05645505	-3.1970423	0.00138845	0.00699373
TPR	16463.3974	-0.2700306	0.06076137	-4.4441156	8.83E-06	8.01E-05
TPT1	50624.0097	-0.2605258	0.04914902	-5.3007319	1.15E-07	1.49E-06
TRAPPC4	6337.984	-0.3468584	0.06873356	-5.0464198	4.50E-07	5.26E-06
TRIM17	895.144889	0.27108049	0.10599664	2.55744404	0.01054445	0.03937491
TRIM2	920.563373	-0.3519771	0.10794415	-3.2607333	0.00111125	0.00578228
TRIM21	390.135008	-0.5459618	0.15893924	-3.435035	0.00059248	0.00335576
TRIM25	7120.19252	-0.3534553	0.05529563	-6.3921013	1.64E-10	3.31E-09
TRIM29	655.567481	0.93698138	0.12847237	7.29325211	3.03E-13	8.73E-12
TRIM34__TRIM6__TI	997.67726	0.44407626	0.10045255	4.42075664	9.84E-06	8.84E-05
TRIM44	12137.9599	-0.2921739	0.051775	-5.6431453	1.67E-08	2.46E-07
TRIP6	958.15458	0.39627542	0.11358004	3.48895302	0.00048492	0.00280332
TRMT112	6979.17834	-0.2170551	0.06330436	-3.4287539	0.00060636	0.00342047
TRPV6	5485.41494	0.15094212	0.05672928	2.66074478	0.0077968	0.03056826
TSC22D1	5644.61074	0.41341426	0.0564383	7.32506586	2.39E-13	7.05E-12
TSPAN12	424.93834	0.5072215	0.15429773	3.2872907	0.00101156	0.00531827
TSPAN13	26603.7447	-0.4235353	0.05169049	-8.1936798	2.53E-16	1.07E-14
TSPAN3	5606.64362	-0.1822487	0.06268162	-2.9075297	0.00364296	0.01601693
TTC39B	2253.8662	-0.2428693	0.08340619	-2.9118857	0.00359254	0.0158286
TTYH3	3904.25845	0.38293863	0.06196944	6.17947559	6.43E-10	1.20E-08
TUBGCP3	1352.07346	-0.5430363	0.090233	-6.018157	1.76E-09	3.09E-08
TUSC1	501.881904	0.54830803	0.14028911	3.90841472	9.29E-05	0.00065147
TXNRD1	19848.0052	0.42259816	0.04569528	9.24817906	2.28E-20	1.46E-18
UAP1	2548.55557	-0.4161215	0.074336	-5.597846	2.17E-08	3.14E-07
UBA6	3245.23891	-0.3923824	0.06706287	-5.8509637	4.89E-09	7.83E-08
UBE2E2	1789.53203	0.2825041	0.08294691	3.40584243	0.0006596	0.00368596
UBE2L6	3829.27515	-0.2938513	0.06559116	-4.4800437	7.46E-06	6.90E-05
UBE2S	2367.95252	0.30191453	0.09534752	3.16646439	0.00154304	0.00764337
UBE2T	2025.54451	-2.0225341	0.08570968	-23.5975	4.09E-123	1.71E-119
UBE3C	5224.89198	0.15616148	0.06214597	2.51281769	0.01197712	0.04375631
UBXN2B	3098.31232	-0.2475758	0.06716775	-3.6859333	0.00022787	0.00143896
UBXN7	7958.14808	-0.4038693	0.06525381	-6.1892068	6.05E-10	1.13E-08
UCP2	5636.65219	0.25379651	0.05685681	4.46378405	8.05E-06	7.37E-05
UNG	5786.65753	-0.1693145	0.06122651	-2.7653799	0.00568565	0.02337572
UPK1A	107.726247	3.00530996	0.34748882	8.64865215	5.21E-18	2.71E-16
UPK3B	1576.76698	0.31346762	0.08799287	3.56242067	0.00036745	0.00219119
USP16	3106.91278	-0.1648214	0.0659122	-2.5006198	0.01239762	0.04499727
USP3	1941.06811	0.3990196	0.0777245	5.13376881	2.84E-07	3.42E-06
USP54	5726.48538	0.43188518	0.05791872	7.45674573	8.87E-14	2.77E-12

UTP20	3415.82907	-0.203094	0.08159535	-2.4890387	0.0128089	0.04610929
VASH2	552.510495	-0.5178433	0.13762131	-3.7628133	0.00016801	0.00109579
VASN	1305.06356	0.56941874	0.09127883	6.23823452	4.43E-10	8.47E-09
VCL	8824.21069	-0.4744599	0.05369376	-8.8364061	9.88E-19	5.50E-17
VGLL4	3145.55786	0.22631791	0.06752534	3.35159967	0.00080346	0.0043558
VMP1	10585.7834	0.43401723	0.0575576	7.54057145	4.68E-14	1.50E-12
VPS35	8046.67058	-0.202199	0.05250873	-3.8507691	0.00011775	0.00080275
VRK1	1504.3894	-0.509979	0.08700363	-5.8615833	4.58E-09	7.41E-08
WARS	10433.469	0.30731907	0.05139744	5.97926777	2.24E-09	3.84E-08
WBP11	4328.49199	-0.1827893	0.0598019	-3.0565794	0.00223878	0.01049814
WBSCR16	5292.17294	-0.2044496	0.05836584	-3.5028977	0.00046023	0.00267939
WDFY1	2101.25432	-0.2595	0.07778464	-3.3361344	0.00084952	0.00456109
WDHD1	3521.35362	-0.6054405	0.06545345	-9.2499398	2.25E-20	1.44E-18
WDR20	1986.93589	0.24403605	0.07855663	3.10649856	0.00189317	0.00909976
WDR76	2048.59307	-0.3463061	0.07761738	-4.4617084	8.13E-06	7.43E-05
WDYHV1	640.905047	-0.6425748	0.12812015	-5.0154075	5.29E-07	6.09E-06
WIPI1	3859.15194	-0.2757486	0.06206628	-4.442809	8.88E-06	8.05E-05
WISP2	658.7283	-0.8843418	0.1238472	-7.1405878	9.29E-13	2.51E-11
WNT7B	1418.13747	0.36902286	0.08729735	4.22719423	2.37E-05	0.00019302
WWC3	19835.2932	-0.2511828	0.05746567	-4.3710064	1.24E-05	0.0001081
XRCC2	737.808832	-0.4838227	0.11846744	-4.0840145	4.43E-05	0.00033607
XRCC6	19082.878	-0.2175605	0.04922667	-4.4195658	9.89E-06	8.88E-05
XRN1	3898.39748	-0.4141665	0.07782558	-5.321727	1.03E-07	1.34E-06
YAP1	11884.2967	0.17563538	0.05069116	3.46481277	0.0005306	0.00303827
YEATS4	1863.91106	-0.4966122	0.08336132	-5.9573458	2.56E-09	4.34E-08
YTHDC2	3198.6149	-0.3162956	0.07163398	-4.4154414	1.01E-05	9.02E-05
YY1	10900.2303	-0.2074156	0.04974154	-4.1698668	3.05E-05	0.00024184
ZBED5-AS1	606.791936	0.69176592	0.14286084	4.84223601	1.28E-06	1.37E-05
ZBTB25	1726.74782	-0.3140389	0.08318421	-3.7752222	0.00015987	0.00104879
ZBTB37	1244.07292	-0.6907888	0.09710439	-7.113879	1.13E-12	3.02E-11
ZBTB41	4086.74767	-0.2135476	0.07627068	-2.7998652	0.0051124	0.0213748
ZFAND5	4403.84845	0.165061	0.0607104	2.71882561	0.00655141	0.02640006
ZFC3H1	5370.85228	-0.3361547	0.06125759	-5.487559	4.08E-08	5.65E-07
ZFP36L1	13318.9725	-0.2230976	0.0505689	-4.4117547	1.03E-05	9.16E-05
ZGRF1	748.787264	-0.5623686	0.11446266	-4.913118	8.96E-07	9.88E-06
ZKSCAN2	589.192882	-0.4999932	0.12952537	-3.8601956	0.0001133	0.00077557
ZMIZ1	15036.5348	-0.2262426	0.0567795	-3.9845826	6.76E-05	0.00049113
ZMIZ2	5236.96659	0.28302161	0.06101932	4.63822956	3.51E-06	3.48E-05
ZNF107	2354.1567	-0.3784081	0.0781544	-4.8418011	1.29E-06	1.37E-05
ZNF217	12192.4408	-0.280536	0.05420057	-5.1758875	2.27E-07	2.77E-06
ZNF219	741.99542	0.53990626	0.11540958	4.67817563	2.89E-06	2.89E-05
ZNF292	2019.47915	-0.2980931	0.08384509	-3.5552843	0.00037757	0.00224438
ZNF296	412.423706	0.43369631	0.15135937	2.86534174	0.00416559	0.0178996

ZNF302	1370.22324	0.25189895	0.09084909	2.77271846	0.00555902	0.02296051
ZNF37BP	1792.34103	-0.2807119	0.08032228	-3.49482	0.00047438	0.00274934
ZNF382	467.520109	-0.3454787	0.13974949	-2.4721281	0.01343114	0.04800776
ZNF431	1042.36154	-0.2722241	0.10281175	-2.6477918	0.00810194	0.03159625
ZNF444	2141.14449	0.34351956	0.08118965	4.23107558	2.33E-05	0.00019024
ZNF467	1718.61165	0.27699046	0.0847233	3.26935395	0.00107793	0.00562648
ZNF528-AS1	447.351299	0.36940935	0.14420441	2.56170629	0.01041594	0.03897343
ZNF579	608.792473	0.53456027	0.13545495	3.94640618	7.93E-05	0.00056628
ZNF613	749.760574	0.98683577	0.11595925	8.51019441	1.74E-17	8.46E-16
ZNF615	954.194299	0.25791249	0.1039244	2.48173194	0.01307456	0.0469341
ZNF710	1451.19685	0.30536799	0.09091396	3.35886803	0.00078262	0.00425388
ZNF736	3060.32899	0.18251232	0.07148543	2.55314004	0.01067566	0.03976706
ZNF85	388.599785	-0.8009108	0.15475493	-5.1753494	2.27E-07	2.78E-06
ZNF865	892.02177	0.32197562	0.11655791	2.76236604	0.00573841	0.02356741
ZNF92	1175.33358	-0.4440606	0.09422859	-4.7125883	2.45E-06	2.47E-05
ZNHIT2	854.515448	0.53481337	0.11065402	4.83320334	1.34E-06	1.43E-05
ZNRF2P1	43.3867937	1.67872223	0.46877532	3.58108063	0.00034218	0.00205959
ZSCAN18	3322.22541	0.23179703	0.06486714	3.57341211	0.00035236	0.00211479
ZYG11B	2379.0643	-0.360781	0.07674156	-4.7012462	2.59E-06	2.60E-05

Control.0	Control.1	shRNA1.0	shRNA1.1	Gene	baseMean	log2FoldChar
2764.74443	2738.85501	3340.83563	3422.38157	44263	2768.50526	0.27091677
4025.98362	4053.59655	3565.96148	3655.1825	AACS	3196.80154	-0.4431807
4904.588	4912.62179	7850.56314	8223.9008	ABCC5	5848.13579	0.68973199
3081.87382	3166.08904	2715.93112	2601.34256	ABCD3	2701.44958	-0.1565184
2826.09077	2929.11656	3724.59109	3651.02534	ABHD11	2905.00097	0.2799312
4082.13112	4084.3574	4853.425	4420.09985	ABHD12	4355.61373	0.42297496
5982.82793	6056.46972	6893.97856	6656.65167	ABHD14B__F	5791.54429	0.15166252
3605.91714	3552.30861	3123.72137	3112.67318	ABI2	3017.81596	-0.2351766
1146.86465	1173.46949	1390.01195	1353.15542	ABTB2	1255.9961	0.46055514
5708.32904	5696.45384	6781.81621	6526.74043	AC002310.11	5403.53246	0.10965238
367.038279	391.916024	273.195431	286.844006	AC009948.5	300.04292	-0.43829
3323.10011	3268.62521	5663.39737	4609.25061	AC010646.3_	3560.87593	0.45268434
2229.26366	2121.35941	1604.72272	1511.12748	AC010761.8	1850.91715	-0.2066558
284.896568	283.683401	444.643591	393.890864	AC113189.5	305.903921	0.44871391
930.592804	1029.91885	5220.3561	4172.74886	AC234582.1	1871.02733	1.6724371
155.965274	177.72936	910.117332	712.952856	AC234582.2	329.46143	1.73537217
543.798923	494.452193	643.331177	753.485161	AC240274.1	531.265906	0.31819898
5154.13244	5265.80193	5980.65658	5856.39846	ACAD10__AL	5201.71483	0.25259016
1656.35121	1706.65757	2197.58085	2100.40484	ACAP3	1711.63522	0.30162815
5651.14178	5628.09639	4869.44819	5002.10218	ACO2	4490.54259	-0.4212779
1219.64845	1150.68368	653.746252	673.459841	ACTA2	988.692114	-0.2697011
10783.4391	10276.4027	9867.08192	9295.40867	ACTL6A	9304.87592	-0.0914672
14478.7763	13897.0688	12655.9186	13021.2629	ACTN1	11589.5531	-0.3367943
3454.11094	3506.73698	4352.70023	4477.26079	ACVR1B	3409.45848	0.20197045
909.797434	967.257862	670.570604	712.952856	ACVR2B	759.925785	-0.3641483
21040.7553	21006.2432	23703.1086	23325.822	ADAM15__D	19758.5011	0.0865163
25228.9428	25170.3509	20005.7569	21738.8264	ADAR	22241.5829	-0.0944052
5104.22355	4998.0686	3807.11053	4143.64874	ADCY9	4179.28835	-0.2963496
10375.8498	10744.6512	11415.7235	12225.1668	ADGRG1	10546.7694	0.25281204
4222.49986	4235.88308	3281.54982	2915.20811	ADI1	3478.2809	-0.3131668
3796.19478	3687.88422	4901.49457	4579.1112	ADSS	3667.76587	0.20135648
3510.25844	3483.95117	2323.36291	2422.5847	AF011889.5_	3036.07919	-0.145056
2824.01124	2694.42267	2005.30254	2252.14116	AGL	2355.08957	-0.1971551
51017.281	49668.5203	46704.4014	42952.8114	AGR2	43454.5482	-0.1616883
4342.07324	4717.80307	3831.94648	3428.61731	AGR3	3602.71554	-0.4231167
18661.765	18579.5538	16902.8657	17111.9078	AHCY	16561.654	-0.0719971
10361.2931	10540.7182	8512.32099	8788.23521	AHCYL1	9215.93894	-0.0975819
6400.81486	6243.31341	4923.92704	4727.72965	AK2	5499.06842	-0.1383998
5244.59229	5219.09101	4225.31585	4441.92494	AK4	3792.3258	-0.7595504
4454.36824	4611.84903	5567.25822	6241.97501	AKAP9	4446.73201	0.20538717
1702.10103	1582.47488	1053.5249	990.443253	AL139099.2_	1201.92506	-0.7285565
10468.3892	10339.0637	13650.9588	14367.1433	ALCAM	10850.9145	0.36536243

49080.1923	50780.4681	43330.7182	40698.5916	ALDH16A1__	44613.5633	-0.0577416
3218.0835	3218.49642	2605.37109	2637.71771	ALDH5A1	2320.41931	-0.7798087
7990.62089	7926.04587	6852.31825	7384.15458	ALDH6A1	6594.10393	-0.2888735
6474.63843	6385.72475	5770.75276	5342.98926	ALDH7A1	5349.37151	-0.2760943
69211.1502	70435.5124	55905.7196	56540.4872	ALDOA	56139.3649	-0.3880397
3484.26423	3618.38748	2476.38439	2455.84198	ALDOC	2718.17231	-0.5594398
2868.72128	2823.16252	1744.92566	1954.90426	ALG10B	2254.68631	-0.4385688
3101.62942	2997.47401	2400.27423	2658.50351	ALG9__FDXA	2610.44889	-0.1894322
331.68615	324.697869	438.234314	446.894647	ALOX15	355.311405	0.4578181
5049.11582	4849.9608	3352.85302	4103.11644	AMZ1	3800.28473	-0.5487064
16832.8122	16616.5559	19336.7887	18133.5298	ANAPC7__AI	16802.5358	0.26863453
1190.53493	1191.69814	1003.05185	934.3216	ANGPTL4	923.652433	-0.5089311
689.366513	722.310347	507.935201	564.334404	ANKFN1	493.361702	-0.9096774
953.467711	885.228927	1563.86358	1502.81316	ANKRD22	1114.1155	0.729746
2773.06258	2697.84054	3513.88611	3537.74274	ANKRD40	2659.90808	0.18033119
2128.40611	2250.09927	3211.84893	2745.80386	ANKRD9	2476.1596	0.56509133
1816.47556	1653.1109	1988.47818	2091.05123	ANKS3__ZNI	1694.26814	0.19164946
17597.042	17476.7204	14367.1955	15339.9186	ANLN	15056.86	-0.1782443
8161.14293	7877.05637	6063.17603	5696.34782	ANP32E	6369.29367	-0.4285919
538.600081	502.427229	933.350961	837.667641	ANXA3	548.943231	0.39629147
600.986191	578.75971	360.52183	417.794531	AP000648.5	435.813723	-0.6913188
0	0	24623.641	26607.8994	AP000769.1	11280.109	16.9150751
0	0	90.5310374	72.7502914	AP000769.7	19.847154	7.76943475
122.692683	128.739857	52.8765351	43.6501749	AP001610.5	70.314086	-2.1324883
8870.26504	8623.29182	7446.77869	8009.80709	AP1B1	7723.34356	-0.0939953
10911.3306	10773.1335	9608.30736	10165.2943	AP1G1	9522.50227	-0.1095414
3794.11524	3824.59911	2535.67021	2549.37807	AP1S2	3249.12027	-0.1994353
9609.54044	9705.61805	8039.63682	8053.45726	AP3M1	7898.90295	-0.3312891
1805.03811	1875.2726	2279.29913	2333.20578	AP5Z1	1850.00535	0.27021513
2522.47837	2589.60792	1921.18078	2112.87632	APAF1	1969.94065	-0.5319356
6112.79899	6074.69838	3578.78004	3745.60072	APBB2	4282.15074	-0.8852892
180.919718	169.754324	272.394272	261.901049	APC2	208.716166	0.68462486
11099.5287	11020.3596	9455.28587	9811.93573	API5	8496.46419	-0.5442317
7599.66794	7844.01694	14307.1085	11648.3609	APOC1__APC	8982.45702	0.63357435
1478.5508	1425.25275	1647.98534	1898.78261	ARAP2	1448.0089	0.24846215
3976.07473	4119.67542	4707.61394	4603.01487	ARFGAP1	3944.24526	0.18682094
2810.49424	2853.92337	1886.73091	1938.27562	ARHGAP19__	2243.23702	-0.4394863
3147.37924	3102.28876	3672.51571	3635.43599	ARHGAP27	3332.9671	0.42266295
3179.61206	3218.49642	5142.64362	4510.51807	ARHGAP39__	3473.27601	0.46482845
2204.30921	2323.01388	2610.17805	2714.62516	ARHGEF18__	2241.03123	0.22863007
1182.21678	1132.45502	1378.79571	1483.06666	ARHGEF38	1180.6686	0.30949918
777.746835	828.264388	1043.91099	992.521833	ARL15	810.982082	0.2801726
4886.91193	4759.95683	3440.98058	3185.42347	ARL3	3813.68181	-0.4438409

1560.69251	1572.22126	2241.64462	2001.6723	ARL4D	1621.32439	0.34678101
71324.9995	72163.8165	62194.0215	59430.7524	ARL6IP1__RF	62621.3278	-0.1276496
6769.93268	6777.64078	4722.03482	4664.33297	ARMT1	5511.73656	-0.3488686
7870.00775	7953.38885	11495.8394	11387.4992	ARRDC1	7607.15509	0.14987675
184.039024	202.793757	278.002389	272.293948	ARSJ	378.145955	1.72063748
3657.90557	3648.00904	4134.78481	4507.4002	ASAP1	4434.64853	0.73355945
15917.8159	16438.8265	10870.9349	11486.2317	ATAD2	13787.7067	-0.2008691
1518.062	1486.77445	870.86051	934.3216	ATAD5	1281.53853	-0.1988781
2441.37643	2409.59997	2038.95124	2111.83703	ATF2	2075.48768	-0.1893846
8312.94913	8291.75821	10070.5765	9537.56321	ATF4	8179.16358	0.21593131
5955.79395	5885.57611	3808.71285	4308.89583	ATM	5212.22422	-0.1026401
3914.72839	4200.56506	3590.79743	3424.46015	ATP13A2	3394.5433	-0.2594183
27624.5694	26761.9401	24005.1458	22874.7702	ATP5A1	23583.2392	-0.1476699
4143.47746	4188.03286	3604.41714	3167.75555	ATP5G1	3595.46186	-0.1609421
9026.23031	9220.28019	8307.22413	7701.13799	ATP5G3	7942.51958	-0.1350856
4136.19908	4144.73981	4789.33223	4660.17581	ATP6V1D	4238.19301	0.31639801
5703.1302	5535.81384	4641.1177	4847.24799	AVL9	4255.16014	-0.595925
16521.9214	16845.5533	14115.6314	14429.5007	AZIN1	14768.4481	-0.0854951
1085.51831	1073.2119	1322.71454	1332.36962	B3GALT6	1139.03427	0.39328296
1706.2601	1809.19374	2275.29333	2152.36934	B4GALNT4	1706.06633	0.17614652
4928.50267	5148.45498	6156.9117	5570.59374	BAMBI	5097.67569	0.28732211
7130.73235	7284.62517	5237.98161	5922.91301	BBX	5965.22278	-0.2929929
3859.62066	4133.34691	4907.90385	5088.36324	BCAR1	4063.98296	0.30021738
1136.46697	1093.71914	3085.26571	3264.40951	BCAS1	2032.59352	1.58586415
2123.20727	2056.41984	1717.68623	1485.14524	BCKDHA__CT	1789.8512	-0.1870982
213.152542	183.425814	108.156549	98.7325384	BCL2	142.794988	-0.7823839
1072.00132	1033.33673	1440.485	1276.24797	BCL3	1202.2694	0.59024484
7953.18923	7960.2246	5060.92534	5169.42785	BCL6	6830.36153	-0.178891
3007.01049	2987.22039	1950.82368	2024.53668	BCL7A	2549.58669	-0.2076584
2211.58759	2199.97047	2635.81516	2478.70636	BCL7B	2144.31128	0.17986555
414.86763	439.766236	678.582201	699.442088	BDKRB1__BI	582.50704	0.99333991
163.243654	154.943544	366.129948	378.301515	BEST1	180.657372	0.57387087
12121.6211	12068.5071	8940.14023	8907.75354	BHLHE40	9171.25754	-0.5919435
4809.96906	4828.31428	4194.87179	4293.30648	BIVM__BIVM	4205.47559	-0.127698
1263.31872	1254.35914	767.510919	791.938887	BLM	1066.18611	-0.2185926
18133.5626	17923.3224	15148.3261	14997.9922	BLOC1S5__B	15595.2993	-0.1553711
4530.27134	4323.60846	5953.41716	5638.14759	BLVRA	4206.72845	0.11752566
1863.26514	1858.18324	1390.01195	1343.80181	BMP4	1550.09564	-0.2731079
7308.53276	7125.12446	6297.11464	6432.16505	BMS1	6197.23893	-0.1770821
3205.60627	3257.23231	2236.03651	1995.43657	BNIP3	2474.53239	-0.5549081
5726.00511	5893.55114	5018.46388	5065.49886	BNIP3L	5036.86473	-0.1489069
3680.78048	3524.96564	4594.65044	4372.29252	BNIPL	3737.79997	0.35197257
1356.89789	1352.33814	730.657576	898.985744	BRCA2	1072.26544	-0.440382

7010.1192	6783.33723	5548.83155	5776.37314	BRI3BP	4859.20643	-0.8723349
2128.40611	2138.44877	2809.6668	2418.42755	BRICD5__PG	2088.10339	0.19677373
7784.74673	7423.61864	9231.76233	8389.14789	BRK1	7204.15073	0.10995322
3493.62215	3460.02606	4155.61496	4526.10742	BRPF3	3414.76583	0.20724314
3562.24687	3424.70805	3926.48331	4121.82365	BSDC1	3437.90034	0.21220083
2202.22967	2210.22409	1797.00103	1965.29716	BTBD3	1709.46016	-0.513517
24451.196	23963.842	22057.5267	19835.8866	BTF3	20947.4906	-0.1541912
955.547248	914.850487	1377.99455	1277.28726	BTG2	1269.1432	0.98209044
7487.37294	7357.53978	6809.05563	6456.06872	BUB3	6512.47826	-0.1129235
3850.26274	3959.03542	6089.61429	6341.74683	C14orf132	3998.06714	0.31821541
1653.23191	1582.47488	1939.60745	1986.08296	C14orf79	1599.55942	0.22535066
20804.7278	21271.6979	25623.4882	23729.0665	C19orf33__C	19717.1057	0.07967723
423.185778	410.144677	596.062759	512.36991	C1orf204__C	524.42957	0.81786987
1108.39322	1097.13701	631.313783	632.927535	C1orf21	823.291894	-0.6444601
502.208184	497.870066	751.487726	729.581494	C20orf96	519.8487	0.3572209
112.294998	123.043403	300.434859	302.433354	C2orf54	136.365183	0.63242475
962.825627	1007.13304	627.307985	599.670259	C4orf46	683.942002	-0.9380563
115.414303	148.1078	503.128243	272.293948	C4orf48	178.456351	0.97952663
989.859608	921.686231	596.062759	611.102448	C5orf34	791.427193	-0.2893992
714.320957	738.260418	1365.97716	1155.69034	C5orf38	742.901381	0.31312625
3674.54187	3654.84478	4773.30903	4919.99828	C6orf132	3772.46135	0.33055952
7604.86678	7454.3795	6410.8793	6651.45522	C6orf89	6360.21511	-0.2295763
5497.25604	5771.64703	6781.81621	6273.1537	C7orf50	5460.29458	0.17212793
622.821329	584.456164	484.701572	452.091097	CA11	489.078338	-0.3631478
67827.2183	67235.2447	46262.9624	47553.7476	CA12	52893.4473	-0.4770858
2127.36634	2156.67742	2522.05049	2589.91038	CAB39	2158.87809	0.27763509
3229.52095	3066.97075	2741.56823	2768.66823	CABLES1	2492.22505	-0.4397323
3621.51367	3422.42947	4399.16749	4519.87168	CACUL1	3429.62622	0.1855582
6705.46703	6634.09014	3897.64157	3865.11905	CADM1	5873.13413	-0.1011691
817.258038	791.807084	1026.28548	1015.38621	CADM4	835.898393	0.35677388
849.490861	880.671764	1105.60028	1235.71566	CADPS2	882.406095	0.3068961
460.617444	445.46269	768.312078	731.660074	CAMK1D	542.407815	0.70300655
13862.1936	13828.7113	15996.7542	16049.7536	CAPN2	13812.8391	0.25002256
2169.99685	2211.36338	2654.24183	2846.61497	CARD14	2256.59358	0.33348063
464.776518	559.391767	1003.85301	887.553555	CARD9__DNI	530.44979	0.34978435
7359.48142	7388.30063	6002.28789	6387.47559	CASC4	6286.2704	-0.2003373
2441.37643	2501.88253	1874.71352	2070.26544	CASP8AP2	2130.2054	-0.165394
5006.48531	4958.19342	6708.10952	6597.41214	CATSPERB__	5028.72575	0.27998252
23726.4773	23145.8312	22263.4248	20653.8077	CBX3	20501.1416	-0.1215185
2911.35179	2937.0916	4347.09211	4056.34839	CBX4	3032.23988	0.35001772
28408.5548	27904.6488	19996.9442	22306.2786	CBX5	24087.2438	-0.1888071
413.827861	395.333897	273.996591	261.901049	CCBL1	317.565786	-0.4759897
2269.81463	2220.47771	2766.40418	2434.01689	CCDC106__ZI	2209.72156	0.21262211

3707.81446	3628.6411	2432.32062	2451.68482	CCDC125	3193.98036	-0.1361869
27.0339809	30.7608507	66.4962487	70.6717117	CCDC129	44.8483627	1.27427988
12414.8358	12658.6597	15488.0178	15090.489	CCDC183__R	12468.6559	0.2417458
1839.35047	1835.39743	2411.49046	2110.79774	CCDC30__PP	1829.27787	0.24530099
12060.2748	12216.6149	15781.2423	16817.7888	CCDC6	11545.9558	0.12021501
1588.76626	1648.55374	2577.33051	2626.28552	CCDC64	1637.3461	0.2872235
386.793881	395.333897	591.255802	586.159491	CCDC78	446.172274	0.59045971
262.021661	247.226097	128.18554	126.793365	CCDC80	197.944152	-0.5059872
478.293508	481.919995	238.745568	261.901049	CCDC82	388.112954	-0.3714736
7361.56095	7387.16134	8025.21594	8206.23287	CCDC85C	6962.04704	0.09917124
2573.42703	2604.4187	3488.249	3445.24594	CCDC90B	2847.15472	0.49782133
2961.26068	2793.54096	2449.94613	2363.34518	CCNC	2414.25173	-0.2493652
7439.54359	7277.78943	3508.27799	3321.57045	CCNG1	5930.95356	-0.3795761
768.388919	726.86751	948.572994	1017.46479	CCNJL	773.412855	0.34427185
7222.23197	7362.09694	5518.38748	5884.45929	CCNL1	6430.5176	-0.0972554
5536.76724	5486.82434	4408.7814	4924.15544	CCNT1	4651.38993	-0.2326604
5676.09622	5819.49724	2751.18215	2764.51107	CCNY	4789.34087	-0.2710609
1826.87325	1689.56821	1163.28377	1201.4191	CCSAP	1268.66208	-0.7785284
19570.5226	19849.8631	18100.5994	17416.4198	CCT2	17422.4276	-0.0899797
10844.7854	11396.3256	9801.38683	9473.12723	CCT4	9698.11036	-0.1293137
16996.0558	16916.1893	15924.6498	15189.2216	CCT8	14906.8582	-0.1058143
276.57842	321.279997	164.237723	159.011351	CD109	190.216797	-1.3325493
5106.30308	5033.38661	4532.15999	4487.65369	CD2BP2	4348.77599	-0.1815165
166.362959	161.779289	91.332197	98.7325384	CD34	110.911564	-1.0565741
17785.2401	18414.3567	16722.6048	14864.9631	CD63	15283.9672	-0.2285369
5989.06654	5994.94802	5572.06518	5204.76371	CDC123	5187.66154	-0.1522397
4202.74426	4333.86208	3826.33836	3776.77942	CDC23	2926.60414	-0.988122
4296.32343	4291.70832	5700.25072	6052.82425	CDC42BPG	4118.80322	0.14317743
2350.91657	2558.84707	3678.12383	3598.02156	CDC42EP1	2466.86646	0.27073576
4455.40801	4461.46265	3074.04947	3082.53378	CDC6	3560.49585	-0.4102424
2116.96866	2204.52764	1663.20738	1696.12108	CDCA7L	1826.25474	-0.2260067
317.129391	295.076309	431.825037	445.855358	CDH19	348.685131	0.58014367
289.055642	270.011912	415.000685	505.094881	CDK6	311.845498	0.532859
2285.41115	2206.80622	2825.68999	2674.09286	CDKN1B	2358.34136	0.38254563
2335.32004	2247.82069	3379.29129	3376.65281	CDR2L	2293.99136	0.25808423
45216.4126	44828.8131	35086.7857	33972.3075	CDV3	38968.8264	-0.1530686
735.116327	626.609922	1506.18009	1275.20868	CEBPA	744.026678	0.47879649
1712.49871	1778.43289	3564.35916	2475.58849	CEBPB	2258.44721	0.87819684
428.38462	469.387796	770.715557	552.902215	CEBPD	488.178711	0.46848325
5993.22561	6177.23454	4101.13611	4894.01603	CELSR2	4648.72231	-0.5652081
2017.15088	1970.97303	1165.68725	1146.33674	CENPK	1713.37336	-0.1751771
905.63836	915.989778	681.786839	723.345755	CENPQ	749.299702	-0.3135943
1285.15386	1260.05559	1861.89496	2167.95868	CEP126	1292.44428	0.29805949

1455.67589	1518.67459	1028.68896	1203.49768	CEP135	1206.67357	-0.3580669
406.549482	436.348364	559.209417	604.866709	CEP19	540.805619	0.8635187
5427.59155	5469.73498	4297.42022	4727.72965	CEP250	4630.73218	-0.2097307
7986.46182	8410.24445	10223.5979	10113.3298	CEP295NL__	8094.2082	0.22154589
6200.13954	6104.31994	4643.52117	4597.81842	CEP55	4976.01635	-0.3691647
855.729472	870.418147	593.659281	633.966825	CEP68	710.953212	-0.3062217
1706.2601	1646.27516	1498.96965	1287.68016	CETN3	1130.73475	-1.0552867
748.633317	814.592899	995.040252	911.457223	CFAP157__T	787.147436	0.27338324
25.9942124	46.7109215	91.332197	90.4182194	CFAP73	59.5687918	1.38025995
2704.43786	2683.02976	2249.65622	2429.85973	CFAP97	2265.25606	-0.2434614
2679.48341	2697.84054	2075.00342	1998.55443	CFL2	2215.98557	-0.307003
5053.27489	4994.65073	4546.58086	4498.04659	CGGBP1	4247.83878	-0.2266143
1537.81761	1566.52481	2471.57744	1987.12225	CHCHD10	1646.62909	0.40800529
1677.14658	1682.73246	1084.77013	1050.72207	CHEK2	1422.74334	-0.2205556
4292.16435	4238.16166	3710.17021	3436.93163	CHMP2A	3686.00032	-0.1575803
9057.42337	8844.31423	10794.0236	10093.5833	CHMP4B	8511.81309	0.11995002
100.857544	75.1931907	364.527628	379.340805	CHST1	130.287879	1.16454957
2299.96791	2449.47515	3034.79265	2976.52621	CISH	2430.02109	0.31661597
15343.8637	15958.0458	11874.7879	11150.5411	CITED2	13374.4103	-0.1928634
740.315169	794.085665	1363.57368	960.303847	CITED4	949.985196	0.77659214
2307.24629	2258.0743	1857.88917	2098.32626	CLASP1	1956.91357	-0.1830225
7789.94557	8192.63991	12609.4513	10892.7972	CLDN3	8797.23904	0.50049881
12308.7795	12966.2682	21319.6587	20461.5391	CLDN4	15749.7817	0.79497599
3987.51218	4263.22605	5234.77698	4454.39642	CLIC3	4941.84639	0.70348257
6161.66811	6022.291	6456.5454	7244.88974	CLIP1	6156.75232	0.28347366
3971.91565	3947.64251	3070.04367	3541.8999	CLSTN2	3073.7726	-0.5076963
1841.43001	1948.18721	2150.31243	2261.49477	CLSTN3	1945.17998	0.32516453
1017.93336	1000.29729	663.360168	598.63097	CMTM7	660.218543	-1.2035136
7244.06711	7155.88531	6645.61907	6140.1246	CNIH1	6260.26254	-0.1402817
447.100453	521.795172	696.207712	624.613216	CNIH2	534.481896	0.5088846
4141.39792	4015.99996	4431.21387	4732.9261	CNNM4	4004.71429	0.20601635
12850.4988	13234.0016	11127.306	12239.7169	CNOT1	11461.7964	-0.1076999
7245.10688	7269.81439	5943.00208	6307.45027	CNOT6	6289.8634	-0.1494362
2053.54278	1961.8587	1361.1702	1521.52038	CNOT6L	1674.85762	-0.2681919
2284.37139	2406.1821	1746.52798	1922.68627	CNTRL	1949.02035	-0.2783755
2577.5861	2727.4621	3420.15043	3144.89117	COA3	2574.6901	0.17689727
2052.50301	1991.48026	3664.50412	3701.95054	COL18A1	2012.18406	0.24069526
824.536417	812.314318	1224.97306	1246.10856	COL6A1	930.867602	0.58099383
976.342618	998.018713	1289.86699	1120.35449	COMTD1	1053.71838	0.42136802
26057.6383	26093.1765	21274.7938	22374.8718	COPA	22615.9861	-0.14763
2826.09077	2741.13359	1990.0805	1992.3187	COPG2__TS	2321.41057	-0.2694239
5237.31391	4759.95683	4407.98025	4224.71335	COPS6	4172.93721	-0.26733
1189.49516	1233.8519	1465.32095	1408.23778	COQ10B	1225.43125	0.28768142

884.84299	865.860984	1055.92838	1094.37224	CORO2A	1049.05183	0.70553938
7801.38303	7607.04446	6321.14942	6349.02186	COX15	6477.5925	-0.2425149
1244.60289	1256.63772	1075.95737	1050.72207	CP	980.289766	-0.4729766
6537.02453	6774.22291	9155.65217	9865.97881	CPAMD8	6688.05058	0.26853894
17793.5583	18049.7836	14948.0362	16055.9893	CPD	14885.4285	-0.282512
5829.98196	5935.7049	7941.09418	7677.23433	CPE	6529.58324	0.52060908
31361.4974	31778.2374	25033.0336	21951.8808	CPEB1__RP1	27441.1195	-0.1396324
24.9544439	35.3180138	7.21043661	7.27502914	CPN2	17.4222306	-1.8903507
14262.5045	14276.4526	11797.0754	12429.9069	CPNE3	12083.9776	-0.220946
8478.27232	8608.48104	6611.16921	6925.82774	CPSF2	7459.41735	-0.1264393
2368.59263	2587.32933	4126.77322	3963.85159	CRACR2B	2486.50834	0.26628779
14063.9087	13539.3315	9395.1989	10045.776	CRIM1	11216.2148	-0.353223
9913.15284	9843.47224	11544.7102	11384.3813	CRIP2	10727.615	0.46717442
9994.25478	9884.4867	11350.0284	10962.4296	CRTAP	9637.03532	0.17267481
1899.65704	1993.75884	2391.46147	2422.5847	CRY2	1915.10481	0.21210538
25222.7042	25215.9226	18904.9636	18554.4422	CSE1L	21558.218	-0.1913709
3005.97072	3033.93132	2172.7449	2140.93715	CSNK2A2	2551.11052	-0.2278326
1966.20223	1741.97558	2203.99012	2393.48459	CSRNP2	1820.92153	0.20602477
5618.90895	5681.64306	4789.33223	5286.86761	CTC-432M15	4878.46183	-0.1602982
6852.07439	6969.04163	8314.43457	7953.68543	CTC-490E21.[illegible]	7122.11215	0.33506751
144.527821	128.739857	266.786154	263.979629	CTD-2008P7.	158.034613	0.6236367
2520.39883	2581.63288	1920.37962	2344.63796	CTD-2031P1[illegible]	2097.14077	-0.3162176
3592.40015	3648.00904	4698.00003	5068.61673	CTD-2349B8.	3568.51668	0.21749973
4568.74277	4412.47314	3512.28379	3286.23459	CTPS2	3916.48487	-0.1316435
364.958742	382.801698	602.472036	596.55239	CTSO	411.670845	0.49949033
132.050599	161.779289	319.66269	257.74389	CTU1	181.459586	0.77569758
898.359981	963.83999	1996.48978	1758.47847	CTXN1	1124.93628	0.72073217
4143.47746	4222.21159	3603.61598	3386.00642	CUEDC2	3555.04224	-0.2067738
2859.36336	3052.15997	2477.98671	2675.13215	CUX1	2511.39293	-0.2097601
2762.66489	2743.41217	2363.42089	2154.44792	CWF19L1	2387.6557	-0.1465034
21749.8774	21162.326	11400.5014	11378.1456	CXCL12	19047.9104	-0.0770544
10949.802	11035.1704	12433.9973	12168.0059	CYB561	10291.6839	0.07614658
3112.02711	3072.6672	3852.77663	3733.12924	CYB561A3	3287.06846	0.41442985
4496.99875	4654.00279	3715.77833	3796.52592	CYBRD1	3790.45303	-0.2892306
5027.28068	4991.23285	6418.8909	6860.35248	CYFIP2	5564.51562	0.52266793
292.174947	264.315458	779.528313	762.83877	CYP1A1	942.282143	2.6834831
68.6247207	44.43234	25.6371079	22.8643773	CYP26B1	35.0119731	-1.4470311
424.225546	450.019853	1109.60608	1126.59023	CYP2T1P	465.593513	0.42037581
374.316659	463.691343	216.313098	210.975845	CYP4V2__KL[illegible]	313.043009	-0.6425902
8586.40824	8670.00274	9649.96766	9858.70378	DAB2IP	8301.85374	0.15235422
22581.6922	21817.4182	20293.3732	19765.2149	DAGLB__KD[illegible]	19491.4302	-0.1103581
5402.63711	5543.78888	4164.42772	3943.0658	DAP	4628.71095	-0.2249507
4550.02694	4565.13811	3895.23809	3743.52214	DARS	3882.07516	-0.2027873

5576.27844	5796.71143	6781.81621	6753.30562	DBN1	5958.0458	0.37776012
885.882759	896.621834	601.670877	669.302681	DCLRE1B	732.583163	-0.3177053
4802.69068	4867.05016	4021.02015	3996.06958	DCUN1D3__I	3841.54838	-0.4272258
5808.14682	5796.71143	5194.71899	4730.84752	DCXR	5018.13122	-0.1549604
4318.15856	4284.87258	3655.69136	3600.10014	DDAH1	3710.84061	-0.163594
562.514756	546.859569	307.645295	332.572761	DDX60	370.330123	-1.1173031
5162.45058	5413.90973	6214.59519	5884.45929	DECR2__NM	5159.14131	0.19012489
3405.24182	3534.07996	2715.12996	2694.87865	DEPDC1	2836.45946	-0.3327897
337.924761	356.59801	739.470332	756.603031	DGAT2	378.374886	0.4742731
51251.2289	51576.8324	56989.6886	57095.468	DHCR24	50064.997	0.18438415
16472.0125	16295.2759	11525.4823	12017.3089	DHTKD1	13795.2039	-0.2388763
5207.16063	5237.31966	4582.63304	4473.10363	DHX40	4589.12867	-0.1082174
926.43373	990.043677	682.587999	737.895813	DHX58	792.782596	-0.292743
19664.1018	19689.2231	16897.2576	17376.9268	DHX9	17455.8484	-0.0794131
9278.89406	9385.47735	10009.6883	11219.1342	DIP2B	10219.9458	0.48790486
54.0679618	92.2825522	151.419169	128.871945	DKK1	249.336551	2.70471433
973.223312	1074.35119	1247.40553	1311.58383	DLG2	1301.62436	0.84225327
24581.167	25312.7623	20499.2713	22430.9934	DLG5	20101.2873	-0.3805256
10502.7016	10860.8589	9628.33635	9497.0309	DLST	8894.0472	-0.2744748
763.190076	705.220985	1027.08664	1012.26834	DLX1	915.947411	0.79611237
205.874162	233.554607	406.187929	400.126603	DLX2	330.291083	1.2023848
1322.58553	1344.36311	1647.98534	1533.99186	DNAJB9	1312.16638	0.21346427
4702.87291	4702.99229	3954.5239	3859.92261	DNAJC8	3502.34141	-0.658773
11526.8735	11441.8972	9097.16752	8956.60017	DNAJC9__MI	9975.61888	-0.1423064
2073.29838	1967.55516	2523.65281	2529.63156	DNASE1L2__	1980.2012	0.20256779
2011.95204	1964.13728	1558.25547	1737.69268	DOCK1	1497.30965	-0.6158653
750.712854	750.792616	1095.98636	989.403963	DOHH	753.499622	0.26444458
1334.02298	1434.36708	1156.07334	1109.96159	DSCC1	1028.00429	-0.6671451
54213.5294	55904.998	45851.1664	53224.1132	DSP	46389.6353	-0.2365605
5522.21048	5354.66661	3646.07744	3626.08238	DTL	4334.75261	-0.4175092
299.453327	277.986947	463.070262	500.937721	DUSP10	336.51792	0.63740167
974.263081	969.536444	1233.78582	1229.47993	DUSP22	1009.80683	0.35528182
4108.12533	4026.25357	1426.06413	1388.49128	DUSP4	3342.42842	-0.3167885
1016.89359	1078.90836	1350.75512	1317.81956	DUSP8	1083.19034	0.3437272
2901.99387	3037.34919	3491.45364	3406.79222	DVL1	2865.09654	0.15891003
100.857544	105.954041	208.301502	215.133005	DYNC1I1	128.640146	0.78954694
660.252995	619.774178	792.346867	851.17841	DYRK1B	878.196682	1.00556299
210.033236	208.490211	113.764666	128.871945	DZIP1L	156.781265	-0.6319789
3018.44794	3219.63571	2725.54504	2601.34256	E2F1	2668.07461	-0.187824
1261.23919	1306.76651	1040.70635	1044.48633	E2F2	1077.22289	-0.2492381
19936.5211	20305.5794	17588.6584	17943.3397	ECT2	17185.1427	-0.1943917
703.923272	691.549496	894.094139	907.300063	EDARADD	733.920417	0.38585077
6093.04339	6144.19511	5144.24594	5656.8548	EEA1	5140.94576	-0.2452842

100983.356	99317.6727	81449.8931	77068.5409	EEF1G__RP1	89094.1478	-0.0709896
19310.5805	18622.8469	12824.9632	12541.111	EFEMP1	16849.2172	-0.0755946
3282.54914	3224.19287	3618.83802	3640.63244	EFNA1	3245.35444	0.25018771
4042.61991	4198.28648	4722.03482	4595.73984	EFNA3__EFN	4242.44694	0.33170238
291.135179	339.508649	482.298093	445.855358	EFNB3	363.598275	0.61454567
10117.9872	9719.28954	8223.90353	8656.24539	EFTUD2	8731.7986	-0.1024836
661.292764	652.81361	1501.37313	1418.63068	EGFL7	757.618329	0.61249131
260.981893	256.340423	119.372784	103.928988	EGR1	196.093508	-0.5978722
10796.9561	11057.9562	8743.05496	8938.93224	EIF1AX	8531.38877	-0.4871145
3406.28159	3381.415	2444.33801	2706.31084	EIF2AK2	2873.40353	-0.221806
21541.9237	20924.2142	16793.1069	17124.3793	EIF2S3	18170.1866	-0.1879309
10161.6575	9751.18968	8873.64398	8712.36704	EIF3D	8592.43848	-0.1625071
22127.3134	22120.4696	19053.1782	17946.4576	EIF3E	18546.7629	-0.2528004
10513.0993	10302.6064	9230.96118	8661.44184	EIF3M	8852.45116	-0.2066151
61944.2082	60769.7696	55886.4918	54148.0419	EIF4A2	53111.5707	-0.1531498
44022.7584	43876.3661	38375.5459	37932.002	EIF4B	37463.9584	-0.1999047
4139.31838	3941.94606	4626.69682	4849.32657	EIF4G3	4147.56769	0.32319336
28.0737494	22.7858154	61.689291	55.0823635	ELAVL2	38.5213297	1.21416322
3846.10367	3833.71343	4981.61054	5000.0236	ELF3	4173.68144	0.46972305
408.629019	422.676875	290.820943	295.158325	ELK3	316.327848	-0.5835385
2074.33815	2083.76281	1704.86768	1690.92463	ELMOD2	1677.66768	-0.3756351
213.152542	166.336452	307.645295	263.979629	EMID1	224.108658	0.66720095
189.237866	200.515175	377.346182	338.8085	EMP1	279.154274	1.09830586
280.737494	314.444252	471.883018	349.201399	ENDOG	353.235871	0.67906789
82255.046	81642.7157	62554.5434	59591.8423	ENO1	68242.3505	-0.2739675
18841.6449	18685.5079	21479.8906	19800.5507	ENSA	17656.5068	0.090982
59.2668043	56.9645384	134.594817	154.854192	ENTPD3	90.1892308	1.26168341
6055.61172	5901.52618	6964.4806	7345.70086	EPAS1	6408.20503	0.43496515
957.626785	1012.82949	1223.37074	1312.62312	EPHA1	1004.71517	0.30754381
3384.44645	3200.26777	4278.99355	4485.57511	EPHB3	3534.16554	0.43783565
4778.77601	4967.30775	7119.90557	6339.66825	EPS8L1	4751.30685	0.1872932
4537.54972	4586.78463	3860.78822	3798.6045	ERAL1	3982.46381	-0.1275293
12617.5907	12592.5809	22227.3726	23009.8779	ERBB2	13263.4913	0.38768919
39667.1681	39339.7102	30975.2345	31118.4175	ERBB3__PA2	34449.9178	-0.1306376
1338.18205	1231.57332	853.234998	945.753789	ERBB4	1055.80809	-0.3156168
14752.2354	14558.9967	8831.98368	9513.65954	ESR1	11116.4472	-0.5904763
1690.66357	1775.01502	3018.76946	2673.05357	ESRRA	1974.79563	0.58699684
3530.01404	3558.00507	1902.7541	1887.35042	ETNK2	2632.91306	-0.6670487
4189.22727	4014.86067	4548.98434	4709.02244	EXOC3	3971.97869	0.16817229
2229.26366	2135.0309	1811.42191	1907.09693	EZH1	1864.12499	-0.1945845
1459.83497	1476.52084	1126.43043	1181.67259	FAM102B	1249.34122	-0.2060107
2052.50301	2125.91657	2842.51434	2713.58587	FAM110A	2082.56676	0.24648311
3408.36113	3395.08649	4819.77629	5443.80038	FAM129A	3389.13415	0.24638471

1797.75973	1729.44339	1418.85369	1308.46596	FAM134B	1252.69411	-0.8405451
2019.23042	2034.77331	1661.60506	1552.69908	FAM162A	1675.02435	-0.2982792
335.845224	336.090777	509.53752	427.14814	FAM171A2	406.61506	0.72254282
3899.13186	3756.24166	2834.50275	2804.00409	FAM192A	3336.42101	-0.1318505
2700.27878	2786.70522	3464.21421	3261.29164	FAM20C	2913.5021	0.4121076
5250.83091	5122.25129	6180.94649	6561.037	FAM214A	5490.29624	0.40358558
2617.0973	2660.24394	2205.59244	2263.57335	FAM217B	1852.34237	-0.8879549
385.754112	410.144677	598.466238	572.648723	FAM43A	415.770876	0.36648669
699.764198	709.778149	1248.20669	1202.45839	FAM69B	795.048386	0.56202182
10573.4058	10863.1375	13959.4053	13594.9509	FAM83H	10652.7585	0.23870077
2565.10888	2752.5265	3464.21421	3184.38419	FAM89B__S[illegible]	2607.95696	0.20554711
4483.48176	4422.72676	3748.62588	3694.67551	FAM8A1	3753.54697	-0.2348429
729.917484	714.335312	464.672581	535.234287	FANCC	586.123012	-0.3581106
4138.27861	4172.08279	2870.55493	2976.52621	FANCD2	3613.37129	-0.137654
7992.70043	8013.77126	5769.15044	6093.35655	FANCI	6978.11163	-0.1311219
1455.67589	1590.44991	1058.33186	1227.40135	FANCM	1254.5606	-0.3061639
5197.80271	5265.80193	3747.82472	3957.61585	FAR1	4351.7742	-0.277449
1006.4959	1021.94382	1370.78411	1318.85885	FBLIM1	1056.52334	0.36389572
1313.22761	1359.17389	2075.80458	1899.8219	FBP1	1364.59241	0.31209095
3120.34526	3205.96422	3709.36905	3496.17115	FBRSL1	3106.07504	0.20511098
1713.53848	1623.48934	1393.21658	1407.19849	FBXL17	1323.11008	-0.4359424
775.667298	728.006801	925.339364	927.046571	FBXL2	779.103734	0.34970708
2073.29838	2068.95203	1408.43862	1471.63447	FBXL20	1734.53931	-0.2562058
1175.97817	1157.51942	902.105735	865.728468	FBXL4	955.258966	-0.3266263
1829.99255	1736.27913	2098.23705	2123.26922	FBXL8__HSF[illegible]	1859.72811	0.36361236
4683.11731	4728.05669	3802.30357	3915.00497	FBXO21	3995.19734	-0.2126672
1366.2558	1385.37757	982.221698	980.050355	FBXO33	1116.35459	-0.3556345
13897.5457	13188.4299	11737.7896	11213.9378	FDFT1	11470.893	-0.2203052
5186.36526	5170.10151	4301.42602	4105.19502	FDPS	4548.84736	-0.1090173
773.587761	787.249921	1095.1852	1076.70431	FEZ1	798.485424	0.31530294
2059.78139	2092.87714	1677.62825	1690.92463	FGD1	1782.71388	-0.179795
515.725174	527.491626	690.599595	656.831203	FGD3	538.493673	0.3413148
141.408515	120.764821	298.03138	272.293948	FGF12	260.435336	1.74482451
144.527821	140.132764	563.215215	391.812284	FGFBP3	218.146996	1.23465497
1668.82844	1727.1648	2019.72341	1991.27941	FGFR3	1921.28101	0.56717495
11415.6183	11301.7644	10150.6924	9601.99918	FHL2	7919.98671	-0.917516
2362.35402	2399.34636	2123.073	2069.22615	FITM2	2018.26234	-0.2158889
43212.7787	43264.5669	38924.3403	36888.5549	FKBP4	38087.2666	-0.1005587
441.901611	439.766236	546.390863	601.748839	FKRP	484.477756	0.49243874
5432.79039	5642.90717	4525.75071	4850.36586	FKTN	4571.232	-0.3031768
43094.2451	43135.8271	36769.2209	40651.8236	FLNB	35226.6792	-0.3354436
191.317403	208.490211	501.525924	576.805882	FLNC	236.273059	0.67419134
5728.08464	5796.71143	5147.45058	5038.47733	FLOT2	4859.44173	-0.2352265

11357.3913	11441.8972	8870.43934	10396.0166	FMN1	9426.28011	-0.296734
3252.39586	3440.65812	2565.31311	2468.31346	FN3KRP	2681.98191	-0.398675
2129.44588	2088.31998	1173.69885	1147.37602	FOS	1680.41815	-0.4182843
462.696981	541.163115	220.318896	207.857976	FOSB	386.972715	-0.5297498
411.748324	430.65191	686.593797	604.866709	FOXD2	464.447772	0.50504905
884.84299	800.92141	676.979881	663.066942	FOXN2	660.507611	-0.4748954
238.106986	226.718863	378.147342	364.790747	FOXO6	251.268164	0.45336949
5471.26183	5276.05555	4599.4574	4534.42174	FOXRED1	4657.57671	-0.1492117
5070.95096	5176.93725	4386.34894	4415.94269	FOXRED2	3952.30791	-0.531239
2267.73509	2448.33586	2853.73058	2625.24623	FRAT2	2438.7742	0.34497429
999.217525	1023.08311	1883.52627	1870.72178	FSTL3	1011.27816	0.25560926
8286.95491	8239.35083	6436.51641	6746.0306	FUBP1	7167.537	-0.1469292
1017.93336	983.207933	703.418149	735.817233	FUT11	801.280286	-0.4001095
9251.86008	9509.66004	11774.643	11432.1887	FUT7__NPD(	8838.24666	0.09285193
17678.144	17660.1462	16019.1867	16051.8322	FYTTD1	15184.0892	-0.1755137
2847.92591	2877.84848	3545.13133	3674.92901	FZD4	2869.99655	0.26348792
786.064983	819.150062	626.506825	622.534637	FZD7	653.54308	-0.3486565
5997.38469	6075.83767	7611.01642	7734.39527	G6PD	5902.24028	0.19585245
1448.39752	1465.12793	1752.93725	1755.3606	GABARAPL1	1935.76112	0.93860942
50.9486563	36.4573046	104.150751	71.7110016	GABRD	69.2474701	1.29640477
1776.96436	1809.19374	1539.02764	1464.35944	GALK1	1494.57589	-0.271198
81.1019427	95.7004245	32.0463849	31.1786963	GALNT5	46.8605689	-2.6610926
47102.5526	47250.9453	42339.6838	42917.4755	GANAB	41954.5345	-0.0722697
55238.7411	53710.724	49157.5521	46902.1129	GAPDH	43681.6223	-0.3972496
9867.40303	9808.15422	8693.38307	8944.12869	GART	8659.40712	-0.1020855
4763.17948	4742.86747	3932.89259	3873.43337	GAS6	3367.53471	-0.8522176
479.333277	505.845101	373.340384	365.830037	GAS6-AS2	331.832595	-1.0605849
338.96453	374.826663	244.353685	228.643773	GATA4	265.874411	-0.6524007
17430.6791	17860.6614	14368.7978	15400.1974	GCN1	15271.6005	-0.154246
362.879205	333.812195	1120.82231	1050.72207	GDF15	395.411744	0.57746907
4651.92425	4943.38264	5867.69308	5739.99799	GEMIN7__M	4810.09249	0.26273608
4651.92425	4484.24846	4089.91987	3763.26865	GGH	3991.05347	-0.1249687
3617.3546	3587.62663	2691.89633	2602.38185	GINS1	3022.94243	-0.2490309
4776.69647	4832.87144	4099.53379	4423.21772	GK5	4021.93667	-0.2555634
1231.0859	1132.45502	1486.1511	1413.43423	GLB1L2	1253.70065	0.40752365
1945.40686	2113.38437	2312.14667	2446.48837	GLI4__RP13-	2094.86098	0.33808994
867.166926	789.528502	644.933496	653.713333	GLS2	663.297373	-0.4015084
321.288465	290.519146	178.658596	162.129221	GLT8D2	214.148826	-0.8974169
2210.54782	2213.64196	1846.67293	1678.45315	GMNN	1897.30313	-0.1813923
11706.7535	11446.4543	9778.95436	9952.23987	GMPS	9815.88339	-0.2176845
6637.88208	6695.61184	5483.13646	5736.88012	GNA13	5191.43121	-0.4972508
51217.9563	50423.8701	43166.4805	40771.3419	GNB2L1	45365.0805	-0.0612584
407.58925	373.687372	797.954984	664.106232	GOLGA7B	540.098639	1.02333334

12634.227	12330.544	10210.7794	9877.411	GPI__PDCD2	10298.0354	-0.3042055
2743.94906	2585.05075	2382.64872	2281.24128	GPN3	2302.03402	-0.1580874
775.667298	775.857013	882.076745	1040.32917	GPR158	782.038649	0.2738186
202.754857	159.500708	303.639497	336.72992	GPR37	207.54731	0.59075176
622.821329	650.535029	291.622103	289.961876	GPR37L1	481.473695	-0.5985693
560.435219	541.163115	419.006483	415.715951	GRAMD2	435.562543	-0.4470519
35159.7717	34620.7679	27891.5711	30957.3276	GREB1	29041.8058	-0.275203
266.180735	293.937018	419.807642	432.344589	GRIN1	339.767763	0.73487335
3459.30979	3596.74095	2636.61632	2828.94705	GYS1	3037.49075	-0.1690117
693.525587	720.031765	1038.30287	929.125151	H19__MIR67	926.496032	0.90970799
5425.51201	5928.86916	7506.06451	6531.93688	H2AFX	5967.00465	0.38530572
21626.1449	21815.1396	18870.5138	17738.5996	H3F3B	17232.8894	-0.4318358
1666.7489	1707.79686	1205.74523	1353.15542	HAUS6	1388.67062	-0.3107161
8342.06264	7773.38091	7404.31723	6934.14206	HDAC1	6803.95859	-0.2307905
4655.04356	4522.98435	3103.69238	3018.0978	HELLS	3833.73997	-0.2632513
2912.39156	3195.7106	2449.94613	2709.42871	HELZ2	2289.00142	-0.635877
2456.97296	2514.41473	3287.15793	3235.30939	HERPUD1	2441.45932	0.20915078
2261.49648	2184.0204	1841.06481	1874.87894	HERPUD2	1766.93035	-0.4262028
776.707067	851.050204	1531.8172	1112.04017	HES4	923.710901	0.57230804
1483.74964	1612.09644	2144.70431	2025.57597	HIST1H1C	1844.54	0.69277073
2394.58685	2231.87061	3344.04027	3045.11934	HIST1H2AC	2384.97363	0.33428019
359.7599	341.78723	532.771149	507.17346	HIST1H2AD_	417.891104	0.6921667
579.151052	618.634887	802.761942	769.074509	HIST1H2BB_	645.138901	0.44582089
1671.94774	1763.62211	2109.45329	1995.43657	HIST1H2BD	1807.96471	0.39007791
1091.75692	1108.52992	2042.15588	1933.07917	HIST1H2BG	1170.40916	0.41752661
589.548737	594.709781	918.930087	884.435686	HIST1H3E	685.702244	0.62234496
649.85531	640.281412	1414.04673	1436.29861	HIST1H4H	735.09835	0.58727468
1958.92385	1819.44736	2941.85814	2876.75438	HIST2H2AA3	1875.61817	0.23567038
807.900121	769.021268	1664.8097	1455.00583	HIST3H2A	797.321216	0.28587849
15078.7227	15260.7998	13488.3234	13655.2297	HK1	13393.579	-0.0938587
572.912441	611.799142	480.695774	438.580328	HLA-F	442.869334	-0.6447817
664.412069	584.456164	467.87722	517.566359	HMGCS2	395.592584	-1.3612495
27267.9288	27780.4661	23062.9821	21942.5272	HNRNPC	23823.0658	-0.1526659
21336.0495	21408.4128	17005.4142	16852.0854	HNRNPH1	18619.1135	-0.1327439
13679.1943	13543.8887	11390.8875	10689.0964	HNRNPH3	11665.9692	-0.1830946
2009.8725	1948.18721	2246.45158	2391.40601	HORMAD2-A	2018.71603	0.30742658
2354.03588	2267.18863	2961.88713	2761.3932	HOXB2	2504.77049	0.46116375
2025.46903	1972.11232	2371.43248	2320.7343	HOXC13	2366.41746	0.67475419
8328.54565	8560.63083	7005.33974	7269.83269	HP1BP3	7175.94963	-0.2097437
1317.38668	1234.99119	1639.97375	1626.48866	HPCAL1	1354.37108	0.40754501
2753.30698	2680.75118	2087.02082	2323.85217	HPS3	2326.98659	-0.1864196
2028.58834	1996.03743	1682.43521	1773.02853	HPS4	1624.82107	-0.3726853
526.122859	536.605952	807.5689	838.706931	HS6ST3	647.992959	0.74551275

6964.36939	7064.74205	6365.2132	6215.99276	HSD17B12	5421.6851	-0.5224014
114931.85	112743.075	97485.9041	97033.2994	HSP90AB1	96185.3358	-0.227807
91182.4983	91144.4007	83097.0772	82823.0889	HSP90B1	78665.0284	-0.1624967
4226.65894	4257.5296	3353.65418	3229.07365	HSPA13	3692.91605	-0.1367281
15308.5116	15488.658	13611.702	13722.7835	HSPA4	13581.417	-0.0961318
36871.2306	36835.5491	51726.0699	44826.651	HSPB1	37754.2893	0.31920259
33726.9707	33238.8082	30387.9845	30041.7132	HSPD1	28305.786	-0.2257535
3272.15146	3172.92479	3933.69375	3627.12167	HYAL2__TUS	3193.49814	0.2310052
22215.6937	22278.831	19288.7191	20051.0196	HYOU1	19616.5875	-0.0976023
24777.6833	24777.2956	22586.2921	22992.21	IARS2	21058.7251	-0.2099876
1447.35775	1408.16339	1967.64803	1755.3606	ID1	2014.30232	1.06628009
812.059195	828.264388	1607.1262	1508.00961	ID2	838.73263	0.31528559
2491.28532	2501.88253	3301.57881	3131.3804	ID3	3003.41542	0.71344766
10377.9294	10251.3383	9554.62966	9190.44039	IDI1	8836.00409	-0.1849846
16865.045	17837.8756	12138.3694	11554.8249	IER3	14331.5839	-0.3000119
18233.3803	17268.2302	15778.8388	15864.76	IFFO1	14171.3725	-0.4128932
425.265315	435.209073	271.593112	244.233121	IFI27	228.949809	-2.5986677
606.185033	592.431199	469.479539	447.933937	IFI27L1	474.967577	-0.4340631
58.2270358	75.1931907	30.4440657	33.2572761	IFI44	35.4894304	-2.5992151
2369.6324	2439.22153	1415.64905	1187.90833	IFI6	1370.75124	-1.9892452
1065.76271	1021.94382	373.340384	394.930153	IFIT1	593.563898	-2.0142639
343.123604	374.826663	222.722375	230.722353	IFIT2	238.87666	-1.1214767
351.441752	387.358861	217.114258	188.111468	IFIT3	205.965405	-2.1571558
723.678873	729.146092	512.742159	490.544822	IFIT5	555.458051	-0.562038
1444.23844	1334.10949	2138.29503	2113.91561	IFRD1__LSM	1386.1336	0.2506227
10356.0942	10247.9205	7834.53995	9222.65837	IGF1R	8827.29544	-0.1834103
271.379577	268.872621	909.316172	1029.93627	IGF2__INS__	286.815417	0.41115626
1708.33964	1664.50381	1970.85267	1967.37574	IGFALS__SPS	1662.69519	0.21831802
39200.3121	40548.4977	34703.8314	31961.2816	IGFBP4	30074.6078	-0.6107524
2304.12699	2369.7248	3283.15213	3284.15601	IGSF9	2774.99915	0.68288455
1218.60868	1187.14098	1365.97716	1619.21363	IKZF2	1182.59443	0.21056394
3086.0329	3100.01018	4345.48979	4193.53466	IL17RC	3088.13275	0.251446
1265.39826	1256.63772	2011.71181	1895.66474	IL17RE	1368.34582	0.46460976
717.440262	718.892475	918.128928	972.775325	IL1R1	1131.4893	1.29751199
31436.3607	32011.792	25975.1973	28619.9647	IL6ST	25847.9842	-0.3442859
8587.44801	8524.17353	6910.80291	6866.58822	IMPAD1	7480.74595	-0.1230839
5858.05571	5853.67597	8743.85612	8609.47735	INHBB	6652.18395	0.57786312
39.5112029	42.1537584	100.946112	102.889698	INHBE	58.85527	1.11775349
3378.20784	3362.04706	3925.68215	3648.94676	INO80B__IN	3240.708	0.15152419
2634.77337	2547.45416	3005.14975	3167.75555	INPP4B	2580.03781	0.24513133
8677.90787	8781.65324	11282.731	11550.6677	INPPL1	8413.97564	0.1566742
3004.93095	2925.69869	2250.45738	2130.54425	INSIG1	2398.79876	-0.3691698
8785.00402	8960.52189	7871.39329	8168.81844	IPO5	7512.21167	-0.2216621

14483.9752	14336.835	11861.1682	12408.0818	IPO7	11733.7094	-0.346321
6123.19667	5974.44079	5393.40658	5486.41126	IPO8	5302.54015	-0.1160103
1177.01794	1253.21984	1597.51229	1567.24914	IPPK	1226.3236	0.28076221
4734.06596	5082.37612	5660.9939	5727.52652	IRAK1	4793.50218	0.19281409
929.553035	930.800558	1525.40792	1372.90193	IRX2	948.678166	0.30678122
124.77222	131.018438	246.757164	237.997382	IRX4	157.188455	0.76345643
375.356427	462.552052	197.085267	207.857976	ISG15	244.654893	-1.8231536
1273.71641	1208.7875	1409.23978	1517.36322	ITGA5	1382.38198	0.52760101
10762.6437	10771.9942	9417.63137	9281.8979	ITPK1	9014.10691	-0.2575099
4094.60834	4301.96194	3175.79675	3806.91882	ITPR3	3448.19007	-0.3195363
5637.62479	5785.31852	4967.18966	5142.40631	IVNS1ABP	4962.24781	-0.1422462
1862.22538	1838.8153	2392.26263	2569.12458	JADE2	1866.16787	0.27624378
4002.06894	4030.81074	5100.18216	5145.52418	JAG2__NUD	4210.55749	0.37730045
25642.7707	25155.5402	16962.9527	17976.597	JAK1	18522.9263	-0.7369925
47107.7515	48323.0179	53362.0378	55111.4636	JUP	45918.7958	0.15238819
866.127157	857.885948	459.064464	459.366126	KATNAL1	641.223203	-0.6651397
2365.47333	2534.92196	2787.23433	2799.84693	KCNC3	2425.6046	0.22993095
1314.26738	1380.82041	1071.15042	1135.94384	KCNK5	1047.27531	-0.5052467
1227.96659	1190.55885	884.480224	894.828585	KCNS3	918.319831	-0.5850297
3982.31334	3700.41641	3074.04947	2889.22586	KCTD6	3149.76043	-0.3238458
4963.8548	4908.06463	5334.92193	5605.9296	KDELC2	5100.80614	0.3415193
6133.59436	6031.40533	5278.84076	5103.95259	KDM1A	5027.56815	-0.2978966
3830.50714	3651.42691	3100.48774	3083.57307	KDM2B	3052.96049	-0.3387027
2119.04819	2150.98097	2692.69749	2849.73284	KDM4A	2242.29264	0.38349451
2859.36336	2827.71969	1933.99933	2006.86875	KHNYN	2458.55911	-0.1572946
16867.1245	17237.4693	15604.186	14452.365	KIAA0391__I	15099.5673	-0.0839343
2152.32079	2138.44877	2729.55084	3074.21946	KIAA0513	2418.27962	0.55910195
3232.64025	3176.34266	4162.02424	4411.78553	KIAA1147	3098.57742	0.1647928
9712.47752	9920.94401	12187.2402	12514.0894	KIAA1161	9728.45814	0.23158208
6934.2161	7185.50687	6023.9192	6039.31348	KIAA1429	5817.30475	-0.3074599
2261.49648	2275.16366	1616.74012	1773.02853	KIF13B	1941.08703	-0.1893782
3119.30549	3080.64224	2124.67532	2377.89524	KIF14	2551.75351	-0.3097654
10406.0031	10557.8075	13852.0499	14091.7315	KIF1C	9863.28312	0.09053779
2574.4668	2620.36877	3126.92601	3095.00526	KIFC2	2846.79128	0.48884503
1135.4272	1204.23034	1607.1262	1442.53435	KLF16	1157.93141	0.22885786
795.422899	781.553467	995.841411	982.128934	KLF4	788.343924	0.25390991
4907.7073	4799.83201	4162.8254	4170.67028	KLHL12	4138.69197	-0.2008195
900.439518	869.278856	608.080154	676.57771	KLHL15	647.70044	-0.7274465
280.737494	292.797727	648.939295	680.73487	KMO	352.800972	0.76248984
7541.4409	7597.93013	4982.41169	5651.65835	KNTC1	6324.13706	-0.2635392
25791.4575	26212.802	20520.1014	18629.2711	KRBA2__RP1	22612.6159	-0.1381701
1267.4778	1329.55233	2159.12518	1924.76485	KREMEN2	1371.93409	0.39817255
4409.65819	4309.93698	3726.19341	3738.32569	KRR1	3756.96315	-0.1667007

102.937081	123.043403	32.0463849	29.1001166	KRT13	64.511329	-1.9779729
2833.36915	2775.31231	2026.93385	1931.00059	KRT15	2179.95041	-0.5021324
6985.16476	7421.34006	8271.97311	8304.96541	KRT80	7643.81538	0.41068332
7030.91457	6959.9273	9250.18901	8756.01722	KYNU	6867.97863	0.20689439
46.7895823	46.7109215	205.096863	168.36496	LA16c-312E8	61.8089456	0.92529042
602.025959	682.43517	483.099253	473.916184	LAMB3	522.927121	-0.3443405
5296.58072	5387.70604	4187.66135	4297.46364	LANCL1	4653.59843	-0.1342244
2627.49499	2539.47912	2257.66782	2113.91561	LAP3	2078.58201	-0.3853149
5201.96179	5036.80449	2415.49626	2449.60624	LBR	4006.67384	-0.4791123
38925.8132	38641.325	30056.3044	28039.0016	LDHA	28361.841	-0.7265569
494.929804	447.741272	342.095159	319.061992	LDHAP4	342.099675	-0.7620477
136.209673	136.714892	284.411666	249.429571	LDLRAD1	154.308654	0.56638773
878.604379	969.536444	602.472036	607.984578	LHFP	761.847881	-0.3017087
782.945678	735.981836	516.747957	602.788129	LHX4	598.138163	-0.4570159
4988.80924	5006.04363	5765.94581	5718.17291	LIMA1	5538.28254	0.51726046
8390.93176	8441.0053	10505.6061	10130.9977	LIME1__RP4-	8453.78784	0.26802141
1478.5508	1497.02807	1861.89496	1856.17172	LIMK1	1565.66922	0.38851033
5721.84603	5809.24363	7424.34623	7496.39789	LIMK2	5598.52555	0.17789726
112.294998	103.67546	160.231925	190.190048	LINC00472__	132.469923	0.75424881
38794.8024	39982.2702	35756.5551	36228.6058	LINC00657	35040.3107	-0.0706767
970.104007	1008.27233	1360.36904	1339.64465	LINC00885	1065.8604	0.44688838
363.918974	391.916024	254.76876	233.840222	LINC01588	237.840265	-1.3923886
1570.05043	1628.04651	2375.43828	2552.49594	LIPH	1974.30305	0.77453517
10021.2888	9892.46174	6752.1733	6704.459	LITAF	7043.71767	-0.8567622
390.952955	467.109215	278.002389	201.622236	LIX1L	285.682555	-1.1188786
716.400494	644.838575	880.474426	856.374859	LLNLR-284B4	713.674233	0.37403357
2409.14361	2260.35288	2884.17464	2707.35013	LMCD1	2427.13962	0.35546522
3856.50135	3879.28507	7731.19036	7573.30534	LMO4	4626.82426	0.69995124
1266.43803	1285.11999	1854.68453	1781.34285	LMTK3	1419.55669	0.52670741
3029.8854	2961.01671	3508.27799	3796.52592	LPGAT1	3094.22854	0.34080999
6076.40709	6145.3344	4330.26776	5282.71045	LPP	4930.76696	-0.3745943
2139.84356	2145.28452	2589.3479	2585.75322	LRCH4__SAP	2200.69871	0.32616041
15208.6938	14982.8129	11227.451	11750.2114	LRIG1	11572.578	-0.5526217
595.787348	648.256447	2551.6934	1796.9322	LRRC26__RP	774.451362	0.79110509
1302.82993	1228.15545	1091.98057	1041.36846	LRRC40	969.529089	-0.5544052
329.606613	325.83716	568.823332	550.823635	LRRC56	394.711163	0.71261478
6630.6037	6738.90489	4385.54778	4873.23024	LRRC58	4794.5254	-0.8022111
2035.86672	2060.977	1622.34824	1789.65717	LRRC8B	1698.27466	-0.2885693
2751.22744	2702.3977	2249.65622	2365.42376	LRRCC1	2262.72832	-0.284333
636.33832	612.938433	974.210101	986.286094	LRRN1	720.032328	0.61117925
1817.51533	1804.63658	1166.48841	1238.83353	LRRN3	1461.90705	-0.3767086
5073.03049	5151.87285	5955.01948	6223.26779	LSS	4964.43556	0.17584472
2570.30772	2558.84707	2990.72887	3215.56288	LTV1__PHAC	2753.35861	0.43859561

8713.26	9347.88075	4867.84587	4779.69415	LUZP6__MTI	7560.71735	-0.2565991
1388.09094	1457.15289	2977.10916	2922.48314	LYPD3	1643.12477	0.61870728
1166.62025	1118.78353	868.457031	874.042787	LYRM1	927.46794	-0.3550014
4083.17088	4047.9001	4575.42261	4645.62575	MADD	3937.74365	0.16990055
4371.18676	4403.35882	5298.86975	4745.39758	MAF1	4167.62696	0.11579458
164.283422	210.768792	415.000685	348.162109	MAFA	264.220314	1.06451739
342.083835	361.155173	567.221013	474.955474	MAFB	369.844027	0.38492645
1924.61149	2101.99147	2349.00001	2361.2666	MAFK	2344.41706	0.63896042
18264.5734	17801.4183	22444.4868	21748.18	MAGED2	18362.2741	0.30310183
12542.7274	12765.7531	9059.51302	8589.73084	MAL2	10989.4371	-0.143771
0	0	224750.911	186047.438	MALAT1	73861.6989	19.6268337
1577.32881	1677.03601	2171.94374	2475.58849	MAP1A	1794.8893	0.50540025
1208.21099	1180.30524	1477.33834	1646.23517	MAP2	1459.50409	0.75033461
2796.97725	2980.38465	4003.39464	3928.51574	MAP7D1	3019.35776	0.37126641
7648.53706	7513.62262	6243.43694	6646.25877	MAPK1	6146.32514	-0.3608474
128.931294	112.789786	227.529333	221.368744	MAPK10	177.487073	1.14561605
873.405537	931.939848	1149.66406	1083.97934	MAPK11	926.629227	0.32503681
687.286976	751.931907	893.29298	929.125151	MAPK15	772.246001	0.43774759
1666.7489	1663.36452	1248.20669	1211.812	MAPK8	1390.12496	-0.2630821
1316.34692	1373.98467	1960.4376	1816.67871	MAPK8IP1	1505.62713	0.54146062
10862.4615	10784.5264	6674.46082	7013.12809	MAPT	7094.43446	-1.1912295
36.3918974	54.6859569	110.560028	89.3789295	MARCKS	101.416615	1.96286544
4491.7999	4687.04222	6981.30495	6506.99392	MARCKSL1	4801.40604	0.37249837
6373.78088	6937.14149	7647.86976	7629.42699	MARK2	6426.44044	0.1621204
2056.66209	1894.64055	2565.31311	2405.95607	MARVELD3	1946.74533	0.21650492
2435.13782	2281.99941	1643.17839	1654.54949	MASTL	1967.04629	-0.2672878
9151.00253	9197.49437	11088.8503	10914.6223	MAT2A	9475.13298	0.34085016
1101.11484	1107.39063	1448.4966	1521.52038	MATN2	1265.25312	0.59857019
22875.9467	23258.621	19026.7399	19161.3875	MATR3	20584.4914	-0.0618107
714.320957	735.981836	966.999665	916.653672	MB	762.617111	0.3834001
752.792391	805.478573	498.321285	554.980795	MBNL3	638.772177	-0.3272508
1841.43001	1739.697	945.368355	982.128934	MBOAT1	1468.05707	-0.3260672
6002.58353	5859.37242	6511.02426	6734.59841	MC1R__RP1	5777.45791	0.18577089
1042.8878	1137.01219	1300.28207	1544.38476	MCC	1114.73491	0.31559119
3701.57585	3970.42833	4337.4782	4297.46364	MCFD2	3681.82892	0.1462367
8505.3063	8390.87651	6108.04096	6233.66069	MCM2	7100.04606	-0.2446043
11434.3342	11312.018	7721.57645	7853.91361	MCM3	9569.56387	-0.2413932
17302.7875	17028.9791	13534.7907	13989.881	MCM4	14409.6341	-0.2478029
3439.55419	3333.56479	2510.0331	2529.63156	MCM5	2765.10231	-0.3395209
5403.67687	5372.89526	4284.60166	4278.75643	MCM6	4477.14637	-0.2813128
7008.03966	7110.31368	5934.18933	5476.01837	MCM7	6196.38686	-0.111067
5657.38039	5327.32363	2999.54163	3357.94559	MDC1	4569.55562	-0.2780273
300.493095	340.64794	228.330493	227.604483	MDGA1	241.386976	-0.6113905

532.36147	519.51659	716.236703	857.414149	MDGA2	584.334033	0.52104737
5006.48531	4901.22888	9632.34215	8843.31757	MDK	5902.54051	0.69114757
239.146754	201.654466	128.986699	156.932772	MED12L	150.627166	-1.0022709
6648.27976	6573.70773	5108.19376	5612.16534	MED14	5793.2409	-0.1154287
4427.33426	4435.25896	5000.03721	5175.66359	MEGF9	4462.66581	0.27492714
945.149563	911.432614	1347.55049	1246.10856	MESDC1	943.131109	0.29346357
2076.41769	2113.38437	4143.59757	3299.74536	METRN	2272.97605	0.46319293
615.54295	627.749213	484.701572	500.937721	METTL21A	501.006641	-0.3794969
4955.53665	5009.46151	4388.75241	3869.27621	METTL21B__	4166.18166	-0.2604119
9060.54267	9123.44047	11420.5304	11553.7856	METTL7A	9087.4982	0.25522176
4934.74128	4700.71371	4308.63645	4196.65253	MFAP1	3886.37322	-0.3759682
754.871928	745.096162	1053.5249	954.068108	MFI2	756.924878	0.27934641
10120.0668	10022.3409	8952.95879	9167.57601	MFN1	8758.25715	-0.138803
9681.28447	9524.47082	13796.7699	12993.2021	MGST1	9104.71911	0.11074101
5373.52359	5293.14491	3135.73876	3132.41969	MICB	4351.08787	-0.3403781
2475.68879	2703.53699	4019.41783	4192.49537	MISP	2502.79811	0.1640963
4221.46009	4254.11173	4577.82609	5048.87023	MLXIP	4180.55868	0.22066851
7587.19072	7690.21268	6705.70604	6873.86325	MOB1A	6560.60638	-0.1777761
1078.23993	951.307791	1233.78582	1195.18336	MOK	1087.38179	0.43420092
11941.7412	12247.3758	10959.0625	10921.8973	MORF4L2	10415.249	-0.1682825
3130.74294	3082.92082	2730.35199	2704.23226	MPP2	2299.40626	-0.6802092
6219.89514	6243.31341	5595.29881	5577.86877	MPZL1	5321.59359	-0.1952262
400.310871	365.712337	527.163032	525.880678	MROH6	434.971454	0.57307392
2998.69234	3058.99571	2783.22853	2464.1563	MRPL22	2626.99413	-0.1456005
4042.61991	4175.50066	4840.60644	4676.80445	MRPS10	4006.051	0.1889678
3723.41098	3826.87769	3318.40316	2973.40834	MRPS23	3195.33049	-0.2216579
4671.67985	4827.17498	3366.47274	3542.93919	MSH2	4018.96665	-0.2223896
6282.28125	6253.56703	5092.97172	5232.82453	MSH6	5415.6067	-0.1572963
122.692683	142.411346	71.3032064	80.0253206	MSS51	95.480794	-0.7858787
174918.175	170596.26	137344.396	133731.664	MT-ND6	144416.098	-0.2617364
17134.345	17145.1868	14775.7869	15030.2102	MTDH	13917.6931	-0.3550442
1754.08945	1811.47232	2754.38678	2798.80764	MTERF2	1911.54415	0.43826019
13915.2218	13740.986	11740.9943	11594.3179	MTHFD1	12016.2424	-0.1406929
167.402728	141.272055	351.709074	354.397848	MUM1L1	191.807973	0.78690238
3507.13914	3362.04706	1398.02354	1415.51281	MX1	1944.03522	-2.0467144
3138.02132	3432.68308	3952.92158	3784.05444	MXD3__RAB	3166.30211	0.15806464
3441.63372	3645.73046	4518.54027	4403.47121	MXD4	3920.64643	0.51405896
2459.05249	2472.26097	2046.16168	1794.85362	MXRA7	2053.60703	-0.2710816
2715.87531	2597.58295	1620.74592	1700.27824	MYB	1814.97556	-1.0047058
8705.98162	8714.43508	7675.10919	7673.07717	MYC	7476.85766	-0.1780468
2941.50508	3057.85642	3368.87621	3489.93541	MYH14	3014.26292	0.26970095
4193.38634	4165.24705	4670.7606	5039.51662	MYO18A__TI	4050.14584	0.17243709
2345.71773	2411.87856	3130.93181	3216.60217	MYO1B	3020.59496	0.83692597

1574.2095	1517.5353	1152.06754	963.421717	Metazoa_SR	1150.29012	-0.6646816
722.639105	738.260418	1783.38132	1963.21858	NAALADL2	1050.43077	1.11037627
5117.74054	5183.77299	5680.22173	5855.35917	NACC1	4993.06063	0.17278693
2287.49069	2316.17813	2953.87553	3047.19792	NACC2	2549.67429	0.51618185
375.356427	356.59801	1196.93248	1060.07568	NANOS1	463.293368	0.82792904
39865.7639	39365.9139	33778.492	32449.7478	NAP1L1	34590.3369	-0.126764
2704.43786	2574.79714	2202.3878	2093.12981	NARF	2217.33033	-0.2471304
2913.43133	2876.70919	2475.58323	2628.3641	NCBP1	2338.48861	-0.3740864
35065.1528	35192.6918	30689.2205	30924.0703	NCL	29818.687	-0.2127498
6525.58708	6596.49355	7560.54336	8277.94388	NCOA3	6852.01742	0.36878786
2213.66713	2147.5631	2550.09108	2687.60362	NCOA5	2226.24566	0.30941733
681.048365	743.956872	305.241816	422.99098	NCR3LG1	436.847115	-1.5375479
6571.3369	6830.04815	5471.11906	5582.02593	NDC1	5842.88701	-0.1305811
4742.38411	4791.85697	4077.90248	3702.98983	NDUFS5	4129.8061	-0.1496332
42894.6095	44686.4018	51437.6524	49048.2465	NEAT1__ma	45452.3894	0.35346178
3434.35534	3553.44791	4361.51299	4018.93396	NECAB3	3367.32704	0.15765351
3330.37849	3242.42153	2451.54845	2590.94966	NEDD1	2411.53319	-0.7125628
963.865396	957.004245	733.061055	697.363508	NEIL2	739.767623	-0.5342388
1037.68896	1080.04765	575.232609	657.870493	NEK1	887.979093	-0.2529497
642.576931	644.838575	878.070947	916.653672	NEK11	704.198854	0.48668627
7678.69034	7893.00644	5726.68898	6076.72791	NEMP1	6386.69264	-0.3217213
9892.35747	9833.21862	6386.84451	7516.14439	NF1	8629.70336	-0.1213151
18193.8691	18526.0072	20073.0544	20375.2781	NFE2L1	17225.831	0.08240982
725.75841	692.688787	958.186909	863.649888	NFKBIB	746.240127	0.38725283
1678.18635	1665.6431	1893.14019	1968.41503	NIPAL2	1645.48277	0.21229485
5123.97915	5344.41299	5615.3278	6099.59229	NIPAL3	5455.54833	0.36427883
1535.73807	1672.47885	1165.68725	1068.38999	NKAIN1	1213.34462	-0.6010893
9453.57517	9440.1633	6333.96798	7078.60336	NKTR	8009.76655	-0.216449
29398.4145	30090.9478	27415.6823	25300.4728	NME1__NMI	26057.1321	-0.1166256
924.354193	944.472047	1121.62347	1177.51543	NME6	930.883813	0.2452192
1077.20016	1122.20141	891.69066	944.714499	NOM1	921.980504	-0.2526111
550.037534	555.973895	836.410646	745.170842	NOXA1	605.731426	0.490877
10528.6958	10217.1596	13632.5321	13698.8799	NPEPPS	12120.1415	0.64504651
1454.63613	1508.42098	1211.35335	1251.30501	NPIPB5	1246.07851	-0.239426
79740.8857	80852.0479	72947.186	67823.0181	NPM1	67884.4902	-0.2256017
6392.49671	6361.79965	7208.83429	7049.50324	NPTN	6386.93734	0.25889275
13277.8437	13775.1647	16397.334	15088.4104	NQO1	12968.1837	0.1429119
2401.86523	2477.95742	2794.44476	2940.15106	NR1D2	2665.80917	0.48350058
4425.25472	4274.61896	3307.18692	3684.28262	NR2C2	3726.10938	-0.1860568
120.613146	138.993474	57.6834928	83.1431902	NRIP2	91.9805342	-0.8511914
1121.91021	1102.83346	1752.93725	1743.92841	NTN4	1275.33177	0.5976879
44742.2782	45360.8619	37005.563	38200.1387	NUCKS1	39688.2283	-0.0997837
2782.4205	2834.55543	2423.50786	2453.7634	NUDCD1	2373.97893	-0.2253553

1332.98321	1238.40906	1108.80492	980.050355	NUDT1	1079.07957	-0.2476996
8726.77699	9060.77948	5474.3237	5565.39729	NUDT21	7708.48851	-0.1491666
33379.688	32416.2402	29947.3467	28551.3715	NUDT3__RP!	29225.4126	-0.0753299
6285.40056	6295.72078	5717.87623	5329.47849	NUDT5	5337.38173	-0.214284
12306.6999	12534.477	10898.1744	11527.8033	NUFIP2	10279.801	-0.2931603
6864.55161	6712.70121	5823.6293	6307.45027	NUP155	5782.56211	-0.2019897
9330.88248	9211.16586	16331.6389	15082.1747	NUPR1	9126.46228	0.21449229
1662.58983	1806.91516	2417.89974	2192.90164	NXPH4	1803.45055	0.35610421
610.344107	563.94893	381.35198	391.812284	OAS1	298.308044	-3.1248232
1899.65704	1802.358	1033.49591	1090.21508	OAS2	994.858288	-2.484158
8960.7249	8930.90033	5192.31552	5356.50003	OAS3	6505.71854	-0.7475259
304.652169	315.583543	169.84584	172.52212	OASL	168.393366	-2.3942773
1828.95278	1812.61161	2335.3803	2284.35915	OBSL1	1871.62105	0.32547038
6671.15467	6358.38178	5459.10167	5285.82832	OCIAD1	5511.73192	-0.2239472
3061.07845	3047.6028	4624.29334	4166.51312	ODC1	3500.77128	0.59835789
4871.3154	5134.78349	4353.50139	3988.79455	OLA1	4276.15162	-0.1919316
15448.8803	15174.2137	11039.1784	10886.5615	OLFM1	12259.2406	-0.4022468
1229.00636	1163.21587	892.49182	928.085861	ORC6	975.270127	-0.3420025
4884.83239	4927.43257	5460.70399	5371.05009	ORMDL3	4847.55361	0.22485561
4808.92929	5004.90434	6080.80154	6132.84957	OSBPL2	4689.48699	0.13406639
4105.00602	3974.98549	2982.71728	2868.44006	P4HA1	3082.53246	-0.5689177
12001.008	12136.8646	9534.60067	9423.24132	PABPC4	10413.1233	-0.1619028
12488.6594	12294.0867	10431.8994	10119.5655	PAICS	10438.76	-0.2372713
32071.6593	31403.4107	34696.6209	35714.1574	PAK2	32814.3945	0.34303002
97.7382386	127.600566	191.47715	169.40425	PALM3	178.234889	1.30892136
3175.45299	3061.27429	2562.10847	2656.42493	PAPSS2	2257.00447	-0.7653504
21440.0264	21300.1802	19105.2535	19864.9867	PARP1	17837.5149	-0.2660844
1504.54501	1544.87828	1208.14871	1246.10856	PARP4	1262.77278	-0.2948259
6135.6739	5999.50518	5141.0413	5202.68513	PAWR	5333.90082	-0.1071071
965.944933	896.621834	1099.191	1192.06549	PBLD	1019.51023	0.48670001
3707.81446	3731.17727	2957.88133	3318.45258	PBRM1	2722.34727	-0.7229428
5724.96534	5841.14377	4327.86428	4176.90602	PBX2	4201.56471	-0.7490286
4929.54244	5039.08307	6441.32337	6276.27157	PBXIP1	5000.27395	0.26499993
5418.23363	5401.37753	7816.91444	8195.83997	PCDH1	5391.23874	0.24601461
6904.06281	6861.9483	7877.00141	8190.64353	PCDHA1__PC	6942.3395	0.27789064
3104.74873	3120.51741	2656.64531	2742.68599	PCGF5	2603.91953	-0.2595075
11984.3717	12109.5216	9451.28007	8797.58881	PCNA	10251.3022	-0.2052343
8062.36492	8010.35339	7045.39772	6729.40196	PCNP	6817.59585	-0.2150562
6041.05496	6188.62745	6782.61737	7881.97443	PCNT	5937.67216	0.17706827
623.861098	609.520561	955.78343	944.714499	PCP4L1	640.702997	0.35501888
182.999255	169.754324	415.000685	357.515718	PDCD4-AS1	232.854254	0.92100287
543.798923	578.75971	760.300482	785.703147	PDGFA	585.454794	0.36610535
8451.23834	8417.08019	7101.4789	6640.02303	PDHA1	7372.69494	-0.1231622

6354.02528	6169.25951	5801.19683	5288.94619	PDHB	5414.36667	-0.156394
1114.63183	1060.67971	657.75205	729.581494	PDK1	815.991518	-0.6274876
2070.17908	2091.73785	1329.12381	1443.57364	PFAS	1758.51845	-0.2264359
1483.74964	1457.15289	1016.67156	1013.30763	PFKFB4__UC	1093.20889	-0.6625845
10833.348	10867.6946	9892.71902	9943.92555	PFKP	8298.04284	-0.5611965
29093.7623	28813.8028	20473.6342	19350.5382	PGK1	22443.7709	-0.5131636
1166.62025	1148.40509	942.163717	929.125151	PGPEP1	962.798569	-0.276303
62619.0179	64004.2161	33236.107	37632.6865	PGR	49368.3657	-0.4925123
5910.04413	5798.99001	7566.15148	7153.43223	PGRMC2	6017.65917	0.32886272
2819.85216	2857.34125	1811.42191	1828.11089	PHACTR1	2251.48122	-0.4323581
7678.69034	7636.66602	7208.03313	6536.09404	PHB	6507.29709	-0.2095136
1679.22612	1691.84679	1932.39701	1927.88272	PHC2	1673.38377	0.23852938
7591.34979	7628.69098	9469.70674	9899.23608	PHF20	7295.58941	0.14291106
3646.46812	3675.35202	2643.0256	2631.48197	PHLDA1	2536.69186	-0.942833
1680.26589	1715.7719	2571.72239	2353.99157	PHLDA3	1660.1817	0.19300126
4189.22727	4133.34691	3675.72035	3534.62487	PHYH	3270.45962	-0.4654435
19655.7836	20604.0735	21182.6604	23400.6509	PIEZO1	19536.9302	0.17574877
1079.2797	1138.15148	895.696458	937.43947	PIGA	872.437041	-0.4612542
1480.63034	1640.57871	1303.48671	1230.51922	PIH1D1	1320.77467	-0.2198122
16581.1882	16365.9119	15077.8241	13992.9989	PIK3C2A__RF	14420.007	-0.1186467
5946.43603	5996.08731	4878.26094	5196.44939	PIK3CB	5134.00256	-0.1731202
521.963785	563.94893	1040.70635	1009.15047	PIK3IP1	606.10672	0.53778126
3355.33294	3168.36763	4496.90896	4627.95783	PIK3R3__RP1	3494.04646	0.43296354
1835.1914	1912.8692	2492.40759	2216.80531	PIM3	1928.16353	0.33132797
447.100453	456.855598	583.244205	564.334404	PINK1	503.839541	0.53084161
5316.33632	5360.36306	4089.11872	4086.4878	PITPNA	4527.03836	-0.2148412
6104.48084	6063.30547	7277.73401	6604.68717	PITX1	5787.54736	0.11902653
9531.5578	9931.19763	8071.6832	8561.67001	PJA2	8318.49906	-0.1912175
72973.0326	73512.7368	64002.2388	61984.2876	PKM	61838.5308	-0.2301483
3837.78552	3890.67797	2850.52594	3025.37283	PKN2	3350.58789	-0.1485052
4939.94012	4979.83995	7013.35134	6643.1409	PKP3	4782.29066	0.15755794
1984.91806	1944.76934	2251.25854	2481.82423	PLCB1	1966.65327	0.25799686
1057.44456	1111.94779	1234.58698	1410.31636	PLCH1	1173.04125	0.45669185
1547.17552	1469.68509	1272.24148	1288.71945	PLEKHA1	1170.28165	-0.5081371
2279.17254	2284.27799	1772.96625	1781.34285	PLEKHA2	1915.95906	-0.2446302
1191.5747	1266.89133	960.590388	940.557339	PLEKHA5	1028.46785	-0.2594246
518.84448	461.412761	761.902801	719.188595	PLEKHF1	646.692189	0.91959612
3382.36692	3521.54776	2530.06209	2740.60741	PLEKHH1	2996.79759	-0.143378
4708.07175	4728.05669	5751.52493	5430.28961	PLEKHH3	4878.30078	0.34216721
1765.52691	1784.12934	2032.54196	2179.39087	PLEKHM1	1746.61728	0.21318223
11555.9871	11529.6226	10012.893	10249.4768	PLOD2	10075.4667	-0.1272446
8997.1168	9114.32614	8262.35919	7880.93514	PLP2	7618.00345	-0.2420321
1417.20446	1563.10693	914.924289	964.461006	PLSCR1	1089.13672	-0.7271981

25120.8069	25590.7492	27233.0179	29808.9123	PLXNB2	24402.3675	0.15228354
2238.62157	2211.36338	2575.72819	2743.72528	PLXNB3__SR	2407.62671	0.45751097
2989.33443	2976.96678	4167.63236	4246.53844	PMEPA1	3075.94961	0.3347311
6812.56319	6786.75511	6281.09144	5994.62401	PNMA1	5970.3695	-0.1102007
5068.87142	4860.21442	7016.55598	6896.72763	PNPLA2	4810.36002	0.16933809
663.3723	592.431199	830.802529	962.382427	PNPLA7	724.325798	0.61304888
2330.1212	2309.34239	3005.14975	2875.71509	PNRC1	2466.34836	0.41541201
1779.0439	1806.91516	2538.07369	2386.20956	PODXL2	1758.49583	0.20357885
3654.78626	3587.62663	2707.11837	2626.28552	POLA2	2981.83333	-0.3114712
9469.17169	9321.67706	7170.37862	7082.76052	POLD2	8033.99676	-0.1911722
7018.43735	7154.74602	5954.21832	6552.72268	POLE	6153.19802	-0.1442992
1135.4272	1123.3407	915.725449	939.518049	POLE2	948.79504	-0.2467898
1900.69681	2013.12679	2395.46727	2308.26282	POLR1B	1897.56622	0.17386009
1945.40686	1962.99799	1679.23057	1580.7599	POMGNT2	1639.90318	-0.2512535
905.63836	906.875451	1510.98705	1427.98429	PP14571	922.510075	0.30136814
13175.9464	14128.3448	16195.4418	15723.4166	PPDPF	13754.3079	0.27637491
3041.32285	3160.39259	3920.07403	3897.33704	PPFIA3	3127.04087	0.27925773
498.04911	485.337867	165.038882	183.954308	PPFIA4	368.246097	-0.636478
83217.8716	81724.7447	77964.0475	71333.7393	PPIA	69827.9914	-0.2211833
4450.20916	4493.36279	3953.72274	3878.62982	PPIG	3892.94753	-0.1340921
4091.48903	4064.98946	3233.48024	3748.71859	PPIP5K2	3195.71421	-0.4765188
5306.9784	5473.15285	6080.00038	6647.29806	PPL	5273.93523	0.19697024
11369.8685	11717.6055	10364.602	10085.269	PPP1CA	10190.5474	-0.0946371
4783.97485	4762.23541	5435.86804	5725.44794	PPP1R10	4553.71316	0.12756571
4786.05439	4781.60335	5404.62282	5325.32133	PPP1R13L	5161.92141	0.4511074
2161.6787	2140.72735	2896.19204	2832.06492	PPP1R16A	2189.19936	0.3025077
1886.14005	2042.74835	1491.75922	1361.46974	PPP1R18	1642.37573	-0.2614351
318.16916	342.926521	596.863919	599.670259	PPP1R3F	373.466919	0.56593379
10599.4	10482.6144	11966.9213	11703.4433	PPP2R1A	9898.2001	0.0835351
4410.69796	4485.38775	3726.19341	3970.08733	PPP2R1B	3677.18278	-0.2981777
3240.9584	3340.40053	2891.38508	2931.83674	PPP2R2C	2472.14893	-0.6256786
1174.9384	1107.39063	1373.18759	1439.41648	PPP2R5B	1155.11409	0.28747072
34611.8137	35044.584	29268.7645	30584.2225	PREX1	27945.2058	-0.3951473
563.554525	598.127653	768.312078	771.153089	PRICKLE2	654.677775	0.55918252
1130.22836	1111.94779	681.786839	795.056756	PRKAA2	795.641548	-0.8421501
184541.232	189563.173	147258.747	170503.819	PRLR	146996.297	-0.4653771
2900.9541	2773.03373	3283.15213	3137.61614	PRMT2	2744.44183	0.16711929
22047.2512	21769.568	18179.9142	20029.1945	PRPF8	18410.5577	-0.2460051
3903.29093	3908.90663	3114.90861	3078.37662	PRPS2	3310.68468	-0.2186691
3806.59246	3771.05244	3161.37587	3124.10537	PRPSAP1	3121.9477	-0.3074924
354.561057	394.194606	708.225107	582.002331	PRR7	419.489903	0.54057434
1469.19288	1441.20282	1898.74831	1724.18191	PRSS22	1566.85543	0.44609124
401.350639	409.005386	253.166441	278.529687	PRSS23	321.989305	-0.4335615

3280.46961	3177.48195	2778.42157	2597.1854	PRSS53__RP	2730.50957	-0.2262465
26925.845	26954.4803	33789.7083	32419.6084	PSAP	25977.485	0.15790415
6678.43305	6392.5605	5910.9557	5521.74712	PSMB3	5729.70156	-0.114574
1768.64621	1933.37643	1591.10301	1436.29861	PSMG1	1539.47987	-0.2762544
2007.79297	1943.63005	1570.27286	1663.90309	PTAR1	1464.55489	-0.676672
11968.7752	11702.7948	9929.57237	10659.9963	PTBP3	10276.1323	-0.1435384
33728.0105	34159.3551	30551.4211	28705.1864	PTGES3	30197.976	-0.0705891
6312.43454	6407.37128	7462.00073	7464.1799	PTK7	6375.03463	0.26246474
39260.6186	38693.7324	35907.9743	33962.9539	PTMA	33336.2614	-0.1898666
3134.90202	3104.56734	4677.16988	3816.27243	PTMS	3158.4557	0.28852644
7938.63247	8192.63991	8773.49903	8907.75354	PTP4A1	7667.34764	0.11888956
27585.0582	27579.9509	24086.0629	22873.7309	PTP4A2	23025.3634	-0.2655315
1488.94849	1481.078	1151.26638	1120.35449	PTPN18	1079.53191	-0.7500828
1989.07713	2096.29501	890.889501	854.296279	PTRF	1755.94405	-0.1730168
5741.60164	5691.89668	7183.19718	6941.41709	PVRL4	5618.46322	0.21058822
433.583463	405.587513	313.253413	327.376311	PWAR6	309.644209	-0.6945091
4997.12739	4947.93981	4571.41681	4366.05678	PWP1	4136.55611	-0.277145
1107.35345	1080.04765	1426.06413	1319.89814	PWWP2B	1095.64887	0.25918177
13252.8893	12953.736	14960.8548	15297.3077	PXN	13731.0618	0.37735079
6167.90672	6348.12816	4660.34553	5417.81813	QSER1	4950.49223	-0.4420627
9344.39947	9611.05692	8463.45026	8595.96658	QSOX1	7716.69568	-0.3464863
1475.4315	1564.24622	1927.59005	1903.97906	QSOX2	1641.32672	0.4551059
3629.83182	3608.13386	5206.73639	5178.78146	RAB15	3901.48511	0.44948258
2026.5088	2231.87061	2861.74217	3110.5946	RAB27B	2187.78616	0.32699528
2622.29615	2336.68537	2140.69851	2088.97265	RAB6B	2090.56021	-0.2351425
1518.062	1523.23176	1958.03412	1735.6141	RAB9A	1575.26129	0.34800871
2384.18916	2340.10324	1708.07232	1912.29337	RAD18	1999.07698	-0.2232709
25745.7077	25247.8227	19874.3668	20087.3948	RAD21	22383.9877	-0.1103969
3715.09284	3801.81329	2827.29231	2938.07248	RAD23A	3273.39396	-0.1336613
1774.88482	1813.7509	1429.26877	1342.76252	RAD51	1451.32371	-0.3670967
119.573377	174.311488	405.386769	324.258442	RAET1E-AS1	273.595683	1.62537686
11365.7094	11284.6751	7424.34623	7506.79079	RAPGEFL1	8716.17242	-0.5379538
1612.68094	1764.7614	1359.56788	1435.25932	RAVER2	1383.54187	-0.3253948
5095.9054	5160.98718	4138.79061	4476.2215	RB1	4444.74392	-0.1487928
24223.4867	23800.9234	21388.5585	20514.5429	RBBP7	20290.8494	-0.2271032
7441.62313	7790.47027	4943.95603	5088.36324	RBBP8	5399.7447	-0.8471809
1780.08367	1906.03345	1473.33255	1570.36701	RBL1	1544.22171	-0.2538222
4456.44777	4299.68336	3632.45773	3592.82511	RBM22	3494.42788	-0.4127408
9801.89761	10353.8745	7175.18558	7424.68689	RDX	8803.08975	-0.1254715
4228.73847	4265.50464	3150.15964	3240.50584	RECQL	3568.37267	-0.2438202
4739.26481	4562.85953	3730.1992	3921.24071	REST	3681.9086	-0.439852
4762.13971	5107.44051	3912.06244	4039.71975	RFC1	4313.04623	-0.1232135
4176.75005	4117.39684	3694.94818	3471.22819	RFC2	3577.05522	-0.1641623

3333.4978	3290.27174	2296.12348	2205.37312	RFC3	2770.47321	-0.258044
5727.04488	5706.70746	4308.63645	4180.02389	RFC4	4893.09503	-0.1870272
1941.24778	1882.10835	1562.26126	1635.84227	RHBDF2	1620.19057	-0.2185899
16295.2519	16285.0222	14675.642	14227.8784	RHOA	13886.0945	-0.2001916
12931.6008	13513.1278	21006.4053	19049.1442	RHOB	12841.3687	0.17718756
2923.82901	2962.156	4214.09962	3773.66155	RHOV	2913.80488	0.22970986
4868.1961	5149.59427	6458.14772	6205.59986	RHPN1	5205.63804	0.35566427
2550.55212	2533.78267	1759.34653	2051.55822	RLIM	2170.68644	-0.1952353
202.754857	206.211629	135.395976	125.754075	RLTPR	152.4418	-0.6580508
4396.1412	4352.09073	5245.99321	5277.514	RMND5A	4196.48118	0.14479914
3677.66117	3544.33358	4496.90896	4574.95404	RMND5B	3462.81699	0.14318076
146.607358	80.8896445	23.2336291	22.8643773	RMRP	64.4072515	-2.0346937
2597.3417	2626.06522	2008.50717	1857.21101	RNASEH2A	2258.08579	-0.1551099
6451.76352	6465.47511	5507.17125	5303.49625	RNF145	5569.04016	-0.1653585
4546.90763	4828.31428	3698.95398	3940.98722	RNF168	3874.99405	-0.2968963
75.9031002	64.9395738	112.162347	134.068394	RNF222	93.5450775	0.94448139
1188.45539	1045.86893	1395.62006	1377.05909	RNF223	1253.62111	0.54505803
7593.42933	7345.00758	6205.78244	6187.93193	RNF26	6465.8076	-0.1545572
3130.74294	3004.30976	3677.32267	3367.2992	RNF7	2983.59848	0.18277408
6221.97468	6136.22008	4569.01333	5040.55591	ROBO1	5316.25007	-0.1716556
180.919718	127.600566	89.7298778	101.850408	RP1-239B22.	108.185655	-0.8841704
317.129391	264.315458	191.47715	180.836439	RP1-257A7.5	221.86602	-0.5676099
354.561057	379.383826	258.774558	282.686847	RP1-41C23.2	261.183146	-0.8326187
255.78305	255.201132	164.237723	137.186264	RP11-126H7.	158.470507	-1.4732311
24.9544439	17.0893615	70.5020468	91.4575092	RP11-127O4.	36.4709826	1.48206079
689.366513	673.320844	822.790933	850.13912	RP11-148K1.	689.762243	0.28791491
90.4598592	104.814751	22.4324694	42.610885	RP11-211G3.	62.5188392	-1.287583
5723.92557	5596.19625	6592.74254	6131.81028	RP11-316M1	5578.43102	0.21627326
262.021661	233.554607	129.787859	174.600699	RP11-342K6.	188.895112	-0.5786159
7109.93698	6950.81298	5458.30051	5729.6051	RP11-343C2.	5640.56229	-0.3940589
973.223312	970.675734	1321.91338	1271.05152	RP11-352M1	1024.32521	0.39385186
32509.4018	33132.8541	31102.6189	29037.7592	RP11-386G1	28937.3333	-0.097432
2961.26068	3066.97075	3919.27288	3980.48023	RP11-400F1	2931.25237	0.18002606
348.322446	349.762266	761.902801	854.296279	RP11-400K9.	366.66953	0.38849976
15.5965274	14.81078	47.2684178	39.4930153	RP11-402D2	26.055482	1.47015542
828.695491	855.607367	1275.44612	1236.75495	RP11-442H2	845.358539	0.26698264
121.652914	103.67546	597.665079	566.412983	RP11-469A1	145.843266	0.87504766
149.726663	159.500708	285.212826	186.032888	RP11-47A8.5	192.778838	0.79231178
650.895079	616.356306	1176.90349	1140.101	RP11-54O7.3	681.638609	0.44092123
1004.41637	961.561408	1330.72613	1418.63068	RP11-580I16	1167.41551	0.68315113
117.49384	146.968509	237.143248	184.993598	RP11-618K1	163.953491	0.78373312
597.866885	540.023824	779.528313	659.949072	RP11-89K21.	612.336309	0.44348506
840.132945	828.264388	1396.42122	1407.19849	RP4-568C11.	884.919244	0.40808954

15627.7205	15158.2637	14498.5857	13701.9977	RP4-734P14.	13172.273	-0.1888538
3940.7226	3832.57414	4705.21047	4587.42552	RP5-850E9.3	3723.47538	0.14051221
450.219759	461.412761	268.388474	254.62602	RP5-899B16.	342.429795	-0.6205275
9787.34085	9736.3789	5811.6119	5830.41621	RPA1	8585.93087	-0.1049697
2292.68953	2289.97444	1954.02832	1732.49623	RPA3	1927.50628	-0.2389838
28398.1572	28645.1878	24842.3576	21500.829	RPL11	24827.4447	-0.1352525
36095.5633	36549.5871	28883.4067	27759.4326	RPL14	30605.9264	-0.2358644
34917.5056	34724.4433	29286.39	27428.9385	RPL15	29647.5143	-0.2040808
18673.2024	18249.1595	15403.0949	14240.3499	RPL18	16286.5919	-0.0959561
14020.2384	13753.5182	11681.7085	10966.5868	RPL18A	11981.4208	-0.1634026
52051.8507	50855.6613	41060.2318	37503.8145	RPL19	44589.4045	-0.1492894
14335.2883	14336.835	12417.9742	11200.427	RPL22	12531.4419	-0.1222678
40216.1659	40369.6291	33715.2004	29818.2659	RPL23	34651.317	-0.1723949
31437.4005	30576.2856	25235.727	22921.5383	RPL23A	27101.5832	-0.1235301
24282.7535	25042.7504	21294.0216	19262.1986	RPL24	21704.8987	-0.1021928
28169.4081	28575.691	24634.0561	21578.7757	RPL27	24471.9545	-0.1633588
12899.368	13408.313	12162.4042	10556.0673	RPL27A	11479.3787	-0.1276509
18789.6565	19799.7343	15485.6144	13801.7696	RPL28	16456.1893	-0.1980604
18536.9927	18326.6313	15740.3831	15026.0531	RPL29	16151.2929	-0.1163804
68315.9095	66657.6243	58998.9969	55636.305	RPL3	60213.6806	-0.0624131
17791.4787	17357.0949	15167.554	13428.6645	RPL31	15458.5423	-0.1037506
38888.3815	39627.9508	32470.1984	28086.8089	RPL32	34139.2806	-0.138488
23238.8259	23200.5172	16861.2054	15002.1494	RPL35	19827.5641	-0.1942753
42366.4071	43671.2937	37647.2918	31741.9914	RPL35A	37356.3508	-0.1431828
27899.0683	27969.5884	24138.9394	20283.8205	RPL37	22331.8717	-0.4068851
22995.5201	24018.528	20348.6533	17594.1383	RPL37A	20239.1123	-0.1685226
12490.7389	12724.7386	10725.1239	8994.0146	RPL39	10916.4726	-0.1510699
69353.5985	68513.5289	54800.1194	52105.8373	RPL4	60885.3318	-0.0924917
43916.702	42421.4917	36077.019	34374.5127	RPL6	36514.4936	-0.2242687
49046.9197	50076.3864	40555.5013	36024.905	RPL7	43014.4374	-0.144471
89690.4305	88820.2476	69117.643	66221.4724	RPL7A	77687.1677	-0.1365259
75096.2399	76653.7615	62020.971	56677.6735	RPL8	66743.9743	-0.1041589
25535.6745	24991.4823	20613.0359	18681.2356	RPL9	22126.8468	-0.1173039
262.021661	225.579572	140.202934	153.814902	RPL9P9	187.588359	-0.545736
86913.2088	85736.1875	63809.9605	60163.4517	RPLP0	77055.0363	-0.061253
21080.2665	20662.1774	19784.6369	18004.6578	RPS11	17945.6985	-0.1739525
24348.2589	25335.5481	22896.3409	20364.8852	RPS12	21616.6587	-0.136802
49826.7461	49253.8185	42983.0149	38095.1705	RPS14	41641.191	-0.2436423
16313.9677	16831.8818	14744.5417	12647.1185	RPS16	13962.1958	-0.2359163
68232.728	67598.6784	57330.9826	52098.5623	RPS18	58862.992	-0.1488951
24747.53	25366.309	21911.7157	19469.0173	RPS19	21610.1272	-0.1635988
25489.9247	25937.0936	21637.7191	19452.3886	RPS20	22003.4914	-0.1873717
53450.3393	53866.8068	43133.6329	38069.1882	RPS24	45889.9871	-0.189668

28057.1131	27865.9129	24811.9135	21509.1433	RPS25	24160.8108	-0.1577341
7961.50737	7627.55169	6887.56928	6393.71133	RPS26	6701.88933	-0.1741023
18232.3406	19420.3504	15924.6498	13770.5909	RPS27	16020.6259	-0.2052243
19096.3882	18940.709	16716.1955	14279.8429	RPS27A	16532.2236	-0.139419
70552.4515	68635.433	58656.9018	53003.7838	RPS3	60783.6424	-0.126005
24358.6566	24043.5924	19748.5847	18146.0013	RPS3A	21382.7768	-0.0909926
20157.9918	20121.0143	18043.717	16916.5213	RPS5	17733.3599	-0.1013035
1132.30789	1073.2119	1384.40383	1492.42026	RPS6KA5	1157.08851	0.37786343
20963.8124	21060.9291	18191.1304	16499.7661	RPS7	18086.6802	-0.1693294
33330.8189	32020.9063	28568.551	26238.9515	RPS8	28310.6297	-0.1499086
17487.8663	18236.6273	15833.3176	14632.1622	RPS9	15485.1034	-0.1478771
37512.7678	36097.2887	31181.1325	29584.4257	RPSA	32658.383	-0.07917
2994.53327	2896.07713	2147.10779	2064.0297	RQCD1	2430.17165	-0.3024836
11304.3631	11576.3335	10238.0188	9956.39703	RRM2	9689.20853	-0.2208052
3934.48399	3841.68847	3311.19272	3353.78844	RRM2B	3325.22832	-0.1910143
249.544439	184.565104	359.720671	378.301515	RSPH1	269.339943	0.78293164
1671.94774	1669.06098	1428.46761	1463.32015	RTKN2	1403.72718	-0.2447725
2741.86952	2653.4082	2300.12928	2328.00933	RWDD3__TM	2262.80983	-0.2518971
6920.69911	7318.80389	8229.51165	8528.41274	RXRA	7164.01665	0.27231541
17289.2706	16334.0117	21147.4094	18657.3319	S100A11	16025.4925	0.12642749
959.706322	958.143536	662.559008	777.388828	SACS	710.318624	-0.6724017
718.480031	725.728219	1095.1852	1011.22905	SAMD11	752.703786	0.36122884
589.548737	500.148647	295.627901	371.026486	SAMD9	406.145089	-0.6570408
155.965274	141.272055	84.1217604	87.3003497	SAMD9L	97.9314974	-1.1580766
3497.78122	3548.89074	2758.39258	2927.67959	SAMHD1	2931.77087	-0.2769394
6022.33913	6430.15709	7529.29814	7037.03176	SAPCD2	5924.44257	0.12156487
726.798179	689.270915	399.778652	438.580328	SATB2	554.680108	-0.4755409
3770.20057	3982.96053	4749.27424	4888.81958	SBNO2	4007.93404	0.34181992
5770.71515	5785.31852	7991.56724	7610.71977	SC5D	6456.27561	0.53740026
2175.19569	2278.58154	3047.61121	2804.00409	SCAND1	2156.25233	0.16813516
3102.66919	3084.06011	1572.67634	1639.99943	SCARA3	2259.25722	-0.7323412
59650.4789	59458.4459	42379.7417	43267.7162	SCD	47286.0731	-0.43015
3781.63802	3938.52819	5035.28823	4870.11237	SCFD1	3972.34695	0.33220613
276.57842	243.808224	128.18554	137.186264	SCNN1G	165.751136	-1.3329461
7466.57757	7482.86176	5507.97241	5248.41388	SCYL1	6552.33821	-0.1153583
1453.59636	1377.40254	1998.0921	2045.32248	SCYL3	1512.88412	0.42872736
3123.46456	3182.03912	4175.64395	4070.89845	SDC1	3304.1688	0.37698787
1141.66581	1193.97672	1599.91577	1690.92463	SDC3	1238.66052	0.40835887
8856.74805	8764.56388	11434.9513	10975.9404	SDC4	8546.74548	0.17463813
6950.8524	6925.74858	8868.83703	8092.95028	SDF4	6563.91178	0.10466211
14326.9701	14760.6512	12153.5915	11541.3141	SDHC	12730.1446	-0.1185108
4843.24165	4840.84647	4041.04914	4249.65631	SEC22C	4054.90008	-0.2563858
23253.3826	23007.9771	20399.9275	21968.5094	SEMA3C	19663.7465	-0.2095538

9302.80873	9329.6521	8134.17365	7959.92117	SEMA3F	7764.0672	-0.2720173
4375.34583	4041.06435	3817.5256	3593.8644	SEPW1	3620.7977	-0.1727867
15813.8391	16142.6109	13296.8463	13219.7672	SERBP1	13402.5391	-0.2501633
752.792391	685.853042	1070.34926	1039.28988	SERPINI1	747.035175	0.35251925
930.592804	1004.85446	1263.42873	1142.17958	SERTAD1	962.294569	0.24176384
3699.49631	3890.67797	2414.6951	2416.34897	SERTAD4	3176.67811	-0.257942
1481.67011	1528.92821	2212.80288	2392.4453	SESN2	1566.6849	0.36103049
36650.7997	35567.5185	32890.006	31299.254	SET	31170.5548	-0.1619082
7335.56674	7109.17439	7898.63272	8736.27071	SETD7	6816.06367	0.09792555
9027.27008	9351.29862	7138.33224	8201.03642	SETX	8056.06154	-0.1143775
6652.43884	6648.90092	8320.84384	8445.26955	SEZ6L2	6371.36341	0.1395784
17571.0478	17400.3879	12218.4854	12675.1793	SF3B3	15534.4022	-0.0749004
2681.56295	2611.25444	1869.1054	1861.36817	SFXN3	2105.90255	-0.4230713
2399.78569	2364.02834	2838.50854	2858.04716	SGPP1	2523.06463	0.40552715
1270.5971	1281.70211	1534.22068	1487.22381	SH2D3A	1265.39172	0.233166
2298.92814	2272.88508	2784.83085	2864.2829	SH3BP5L	2241.85782	0.20409367
4012.46663	4102.58606	3288.76025	3296.62749	SH3D19	3500.41999	-0.1633871
165.323191	149.247091	443.041271	405.323052	SHC2	189.035769	0.71041768
18.7158329	23.9251061	99.3437932	65.4752623	SHISA8	45.6806249	1.88917864
5297.62049	5371.75597	3858.38474	3505.52476	SIGMAR1	4576.02457	-0.1810283
1680.26589	1622.35005	6133.67807	6138.04602	SKAP2	1690.12294	0.31702304
5182.20618	5104.02264	6545.47412	6548.56552	SLC11A2	4970.69464	0.16416801
4872.35517	4979.83995	6457.34656	6625.47297	SLC12A7	4825.22633	0.20093371
694.565355	714.335312	551.197821	519.644939	SLC18B1	565.078892	-0.394062
1292.43224	1178.02665	1756.94305	1785.50001	SLC19A2	1253.80156	0.29540882
6444.48514	6324.20305	5217.95262	5342.98926	SLC25A36	5288.82733	-0.2920294
168.442496	172.032906	39.2568215	39.4930153	SLC25A48	107.612868	-1.375786
1631.39677	1506.1424	1877.91816	1842.66095	SLC2A6	1634.07758	0.36089964
572.912441	571.923966	799.557304	693.206348	SLC35D2	596.002668	0.36400156
1629.31723	1630.32509	2124.67532	2160.68366	SLC37A1	1657.58212	0.30156222
27381.2636	27483.1112	31701.8863	34756.9714	SLC38A1	25626.4683	0.07017149
4829.72466	4767.93186	5446.28312	5458.35044	SLC38A10	4782.53129	0.24654851
17245.6003	17218.1014	21352.5063	20421.0068	SLC3A2	18243.3038	0.40379368
2533.91582	2559.98636	2148.71011	2249.0233	SLC46A1	2130.32941	-0.2613787
548.997766	525.213044	370.135746	359.594298	SLC47A1	373.677304	-0.925779
4932.66175	5148.45498	5556.84314	5587.22238	SLC4A2	4866.46696	0.16205662
4968.01387	4903.50747	6488.59179	6504.91534	SLC5A6	4769.93963	0.16469934
31.1930549	22.7858154	61.689291	59.239523	SLC6A4	47.7559174	1.52806153
5286.18303	4998.0686	3459.40725	3628.16096	SLC6A6	4448.51675	-0.1557468
33451.4321	33981.6257	42286.0061	42753.2677	SLC7A5	40118.0923	0.6875989
2554.71119	2481.37529	3959.33086	4090.64496	SLC9A1	2721.65143	0.45464092
2103.45167	2163.51317	1818.63234	1781.34285	SLC9A8	1787.66727	-0.2534757
1614.76047	1662.22523	1384.40383	1414.47352	SLITRK4	1401.08763	-0.1900529

3832.58668	3666.23769	3259.11735	3216.60217	SMAD2	3266.74114	-0.1334962
382.634807	364.573046	543.987384	484.309083	SMAD6	408.36932	0.48166075
1595.00487	1695.26466	1317.10642	1353.15542	SMC5	1393.46329	-0.2204555
5066.79188	5067.56534	6152.10474	6268.99654	SMIM14	4928.59249	0.18243561
113.334766	132.157729	54.4788544	55.0823635	SNCB	73.8774653	-1.6357995
6798.00643	6875.61979	4951.96763	4853.48373	SNHG1__SN	5821.91382	-0.2029603
954.507479	943.332756	1231.38234	1270.01223	SNHG17__SI	947.118136	0.25317918
20402.3374	19989.9958	16538.3381	18033.758	SNRNP200	16876.2582	-0.2636702
1558.61298	1469.68509	1200.93827	1213.89058	SNRNP48	1261.6187	-0.2724793
5259.14905	5039.08307	4728.44409	4224.71335	SNRPD2	4339.5775	-0.2350279
1646.9933	1519.81388	1029.49012	979.011065	SNX10	1134.27443	-0.8057449
2972.69813	2963.29529	3484.2432	3445.24594	SNX18	2909.24637	0.20047047
11.4374535	9.11432614	41.6603004	37.4144356	SOCS1	19.8663449	1.69635115
170.522033	176.590069	112.963507	111.204017	SOCS3	129.609941	-0.6537028
5348.56914	5479.98859	4284.60166	4324.48518	SOD2	4716.0019	-0.1327546
2971.65836	3252.67514	7413.12999	6366.68979	SOD3	4383.18464	1.063487
15591.3286	15255.1034	20495.2655	23387.1401	SORL1	14910.4051	0.16443794
1536.77784	1400.18835	3799.09893	3546.05706	SOWAHA	1481.67396	0.27725033
4281.76667	4390.82662	4996.03141	4853.48373	SOX12	4745.69763	0.48724028
3579.92293	3757.38095	5590.49185	4996.90573	SOX4	3752.35784	0.3161337
645.696236	659.649355	359.720671	330.494181	SPAG4	530.122791	-0.3578653
6188.70209	6176.09525	4788.53107	5073.81318	SPAG9	5226.68829	-0.2261044
353.521289	350.901557	581.641886	588.238071	SPEG	386.653358	0.4950041
1774.88482	1764.7614	1119.21999	1174.39756	SPICE1	1485.76309	-0.2477922
3506.09937	3407.61869	3063.6344	3037.84431	SPIDR	2975.12414	-0.1712284
25755.0656	25374.284	34255.182	33965.0325	SPINT1	24309.4824	0.11923526
2419.54129	2269.46721	2950.67089	2842.45782	SPIRE2	2391.49596	0.30759161
1320.50599	1368.28821	2205.59244	2303.06637	SPNS2	1346.48868	0.26043883
222.510458	232.415317	351.709074	336.72992	SPRY1	258.084082	0.57261196
5758.23793	5817.21866	3855.98126	4110.39147	SRGAP2	5044.98123	-0.1321349
625.940635	529.770207	407.790248	470.798315	SRGAP2C	470.553831	-0.3476459
8014.53557	7681.09836	7227.26096	6931.02419	SRP54	6838.23814	-0.1323169
5404.71664	5402.51682	4480.88577	4639.39001	SRPK1	4630.76092	-0.1826474
2743.94906	2767.33728	3029.18453	3215.56288	SRPK2	2708.3975	0.2104462
11244.0565	11248.2178	9733.28826	9904.43253	SRSF10	9721.29572	-0.1570067
9111.49133	9047.10799	7948.30462	7930.82106	SRSF11	7786.02987	-0.1817278
3948.00098	3970.42833	3443.38406	3272.72382	SSBP1	3421.99693	-0.1578865
5838.30011	5895.82972	6799.44172	6651.45522	SSH3	5577.84006	0.11948262
13178.0259	13200.9621	11418.9281	11176.5233	SSR3	10962.1685	-0.279558
18491.2429	18391.5709	14985.6907	14600.9835	SSRP1	15508.3014	-0.2423348
17077.1578	17403.8058	15274.9094	14810.92	ST13	14325.6754	-0.2803227
940.990489	961.561408	1185.71624	1226.36206	STAG3L3	943.097981	0.23508998
1652.19214	1558.54977	1237.79162	1232.59779	STARD4	1287.67163	-0.3964579

8378.45454	8436.44814	6183.34997	6198.32483	STAT1	6288.87953	-0.6407411
7435.38452	7259.56077	8353.69139	8510.74481	STAT6	7192.12568	0.19963019
13826.8415	13707.9465	10091.4066	9764.1284	STAU1	12186.015	-0.086054
3985.43265	4123.09329	3124.52253	3213.4843	STC1	2538.0283	-1.4205178
86603.3578	89328.3713	52000.8676	50265.2549	STC2	71486.029	-0.3525262
1881.98098	1935.65501	2230.42839	2171.07655	STK17A	2160.83302	0.56901531
6073.28779	6071.2805	4110.75003	4325.52447	STK24	5258.30118	-0.151342
15044.4104	15101.2991	11650.4632	10820.0469	STMN1	13406.4969	-0.0714628
106.056387	100.257588	385.357779	303.472644	STMN3	225.988203	1.92715854
6348.82644	6285.46717	8392.14705	8664.55971	STOM	6024.93733	0.12832835
8407.56806	8262.13665	7402.71492	6781.36645	SUB1	7335.03326	-0.1024305
8505.3063	8549.23792	10855.7129	9936.65052	SUMO3	8282.47314	0.17738596
414.86763	340.64794	268.388474	287.883296	SYN1	259.200891	-0.9758943
775.667298	791.807084	1151.26638	1169.20111	SYNPO	826.976251	0.39280873
165.323191	178.868651	773.920196	650.595463	SYP	238.786114	1.03115932
2352.99611	2414.15714	2800.05288	2748.92173	SYTL1	2334.46656	0.19851413
9757.18757	9582.57465	7059.8186	7708.41302	TACC1	7558.88268	-0.4837521
13973.4488	14328.86	19202.1938	17521.388	TACSTD2	13658.5858	0.16008635
24633.1554	24819.4494	30669.9927	29041.9163	TAGLN2	23107.423	0.07099942
2802.1761	2946.20593	3773.46182	3870.3155	TAOK3	2806.12881	0.19258335
5215.47878	5151.87285	7142.33804	7058.85685	TARS	4941.34111	0.12619716
12537.5285	12466.1196	13548.4104	13749.8051	TAX1BP1	11691.7409	0.07328387
2398.74592	2431.2465	1817.03002	1900.86119	TBC1D13	2088.0814	-0.1572865
1099.0353	1100.55488	1430.06993	1510.08819	TBC1D4	1103.0325	0.26537512
280.737494	248.365387	391.767056	387.655124	TBC1D8	285.295479	0.45190306
1886.14005	1861.60112	2418.7009	2631.48197	TBC1D9	1873.74342	0.25568247
324.407771	313.304961	510.33868	419.873111	TBX1	368.573988	0.61677533
6470.47935	6533.83255	8179.83975	7919.38887	TCAF1	7703.1453	0.67877703
1577.32881	1485.63516	1929.99353	1860.32888	TCAIM	1686.28101	0.50193801
5000.2467	5017.43654	4582.63304	4010.61964	TCEB2	4321.03613	-0.1635155
3564.3264	3704.97358	4109.94887	4046.99478	TCTN1	3596.52488	0.22931096
6212.61676	6196.60249	5556.04198	5479.13623	TCTN3	5411.60428	-0.13135
1928.77056	1909.45133	2323.36291	2305.14495	TEAD2	1874.47272	0.19248116
5264.3479	5225.92675	4623.49218	4507.4002	TECR	4495.88427	-0.1840347
663.3723	595.849072	912.52081	932.24302	TEF	650.16898	0.33684456
725.75841	769.021268	1289.86699	1317.81956	TESK2	753.055805	0.27730504
200.67532	219.883118	525.560713	484.309083	TEX14	277.98722	0.9253778
10373.7703	10168.1701	11430.9455	11760.6043	TFAP2A	10113.2986	0.21437142
628.020172	626.609922	786.73875	810.646105	TGFB1	722.901626	0.61089157
367.038279	322.419287	220.318896	248.390281	TGFBR3	238.380846	-0.9584082
35927.1208	35521.9469	30048.2928	33684.4242	THBS1	28408.2048	-0.4249809
1733.29408	1794.38296	2179.95533	2330.08791	THRB	1926.67312	0.48310809
2901.99387	2864.17699	1943.61325	2298.90921	THRIL	1992.72205	-0.9544403

86.3007852	86.5860984	166.641202	236.958092	THSD7A	119.299684	1.01738816
4584.3393	4491.08421	4067.48741	4036.60188	THUMPD3	3933.72187	-0.148209
2368.59263	2434.66437	3656.49252	3531.507	TLE1	2650.75243	0.50722329
4245.37477	4369.1801	4857.43079	5159.03495	TLE3	4566.39001	0.40773447
2620.21661	2698.97983	3014.76366	3035.76573	TLK1	2671.90685	0.26916195
534.441007	481.919995	305.241816	320.101282	TLR5	403.545541	-0.428904
12149.6949	12573.2129	15166.7528	14027.2955	TM7SF2__VF	12217.0685	0.22406033
33683.3004	34240.2447	29203.8706	28315.4527	TMED2	29962.6534	-0.0955454
8219.36996	8895.58232	7910.65012	7489.12286	TMED4	6997.41864	-0.3326382
18162.6761	18199.0307	16106.5131	16036.2428	TMEM123	14237.738	-0.477845
13187.3838	13401.4773	17153.6287	16723.2134	TMEM132A	15423.7524	0.62748971
3361.57155	3300.52536	4084.31176	3942.02651	TMEM159	3241.43469	0.18420894
2560.94981	2561.12565	3460.20841	3124.10537	TMEM165	2479.76981	0.16825052
10176.2143	10386.9139	7101.4789	7171.10016	TMEM167A	7325.14018	-0.8283295
5183.24595	5222.50888	7578.16887	7905.8781	TMEM184A	5586.01213	0.43925831
1713.53848	1728.3041	1494.1627	1483.06666	TMEM209	1451.46904	-0.233037
1532.61876	1678.1753	1380.39803	1304.3088	TMEM237	1218.9336	-0.5869305
75.9031002	124.182694	478.292295	259.822469	TMEM238	128.031684	0.85821096
930.592804	950.168501	771.516717	690.088479	TMEM243	715.098842	-0.5786147
8887.9411	8796.46402	9589.88069	10085.269	TMEM245	8834.29359	0.25376344
5034.55906	5072.1225	5938.19512	5658.93338	TMEM259	4988.3264	0.21971789
1592.92534	1642.85729	1861.0938	1895.66474	TMEM57	1677.12889	0.34903008
8918.09439	8876.21437	10372.6136	9915.86472	TMEM59	8383.73881	0.09446461
1590.8458	1592.72849	1314.70294	1334.4482	TMEM65	1317.63184	-0.2910559
1060.56387	1160.93729	1391.61426	1379.13767	TMEM80	1105.50914	0.24433982
502.208184	581.038292	1226.57538	1242.99069	TMEM86A	666.773086	0.76969646
5057.43396	5089.21186	4050.66305	3849.52971	TMEM97	4437.85258	-0.1199922
4311.91995	4094.61102	3460.20841	3566.84286	TMOD3	3438.85157	-0.3298073
19460.3072	19657.3229	15496.0294	15583.1124	TMPO	16971.0065	-0.14597
1366.2558	1491.33162	1684.03753	1786.5393	TMTC2	1398.47009	0.19855979
6136.71366	6255.84561	5020.86736	5262.96394	TMX1	5194.09745	-0.2526491
1876.78214	2206.80622	3203.03617	2717.74303	TNFRSF12A	2294.36342	0.55277879
285.936336	279.126238	185.067873	142.382713	TNNC1	194.755715	-0.9622872
10545.3321	10515.6538	9146.03825	9551.07398	TNPO1	9311.89879	-0.0886941
16011.3951	16319.201	19472.9858	21487.3182	TNRC18	16919.7668	0.3743152
27234.6562	26856.5013	20475.2365	19651.9323	TOB1	20545.9194	-0.5848477
1854.947	1975.53019	2381.0464	2332.16649	TOLLIP	1919.96846	0.26259517
3445.7928	3779.02748	4046.65726	4400.35334	TOM1L2	3514.60121	0.18342373
21873.6099	22180.852	17873.07	17419.5376	TOMM20	18390.9717	-0.2654338
3038.20355	3150.13897	2804.05868	2541.06375	TOMM22	2676.86964	-0.1545402
40338.8586	41560.1879	28094.2645	29960.6486	TOP2A	32482.4878	-0.4330941
4377.42537	4350.95144	3763.84791	3910.84781	TOP3A	3799.05367	-0.1379027
7763.95136	7931.74233	5870.09656	6421.77215	TOPBP1	6846.03225	-0.1299332

7682.84942	7662.86971	5544.82575	5788.84462	TOR1AIP2	6636.44625	-0.1538192
10962.2793	11364.4254	14500.188	14551.0976	TP53I11	11642.3605	0.36561111
8417.96574	8742.91735	13990.6505	12804.0513	TP53I3	8177.52317	0.12652889
6899.90374	6827.76957	6246.64158	5757.66592	TPBG	5934.84618	-0.1578479
11060.0175	10989.5987	10151.4936	9298.52654	TPI1	8955.77456	-0.3550202
17793.5583	18201.3093	13969.0192	15889.7029	TPR	15991.9047	-0.0736917
55211.7071	55157.6232	47822.8202	44303.8882	TPT1	49220.9734	-0.0631004
7155.68679	7040.81695	5976.65079	5178.78146	TRAPPC4	6100.99921	-0.1749538
813.098964	810.035736	1005.45533	951.989528	TRIM17	812.177999	0.25622401
1059.5241	1003.71517	769.914398	849.09983	TRIM2	830.447799	-0.3826801
501.168415	424.955456	323.668488	310.747673	TRIM21	371.579489	-0.3947487
7845.0533	8129.97892	6140.08735	6365.6505	TRIM25	6951.28257	-0.1357946
412.788093	487.616449	864.451233	857.414149	TRIM29	482.358637	0.43645622
847.411324	843.075168	1142.45362	1157.76892	TRIM34__TR	899.534337	0.41688102
13248.7302	13474.3919	10603.3476	11225.37	TRIM44	11731.5339	-0.1106006
774.62753	881.811054	1157.67566	1018.50408	TRIP6	869.145975	0.38326242
7329.32813	7681.09836	6789.02664	6117.26022	TRMT112	6184.11601	-0.3067585
5233.15484	5163.26576	5710.66579	5834.57337	TRPV6	5002.85631	0.1519412
4779.81578	4903.50747	6462.95468	6432.16505	TSC22D1	5094.76961	0.38855391
333.765687	369.130209	534.373468	462.483996	TSPAN12	398.300709	0.57327418
30283.2574	30683.379	23671.0622	21777.2801	TSPAN13	26904.0185	-0.0946711
5951.63487	5972.16221	5497.55733	5005.22005	TSPAN3	5175.83565	-0.1432513
2530.79652	2352.63544	1941.20977	2190.82306	TTC39B	2056.10198	-0.2385057
3378.20784	3400.78294	4468.86838	4369.17465	TTYH3	3530.50985	0.36228821
1606.44233	1599.56424	1066.34346	1135.94384	TUBGCP3	1363.92568	-0.2043212
415.907398	399.89106	630.512623	561.216534	TUSC1	434.980919	0.42169502
17034.5273	16887.7071	22553.4445	22916.3418	TXNRD1	17261.3339	0.3017277
2974.77767	2853.92337	2264.07709	2101.44413	UAP1	2365.77648	-0.3566832
3659.98511	3706.11287	2728.74968	2886.10799	UBA6	3211.21934	-0.1308834
1558.61298	1672.47885	2008.50717	1918.52911	UBE2E2	1598.08957	0.22765108
4170.51144	4267.78322	3560.35336	3318.45258	UBE2L6	3263.67664	-0.5203892
2183.51384	2060.977	2896.19204	2331.1272	UBE2S	2177.48463	0.32109823
3216.00396	3289.13245	850.83152	746.210132	UBE2T	1758.27637	-2.4289886
4900.42892	4982.11853	5288.45467	5728.56581	UBE3C	4720.77596	0.13293682
3287.74798	3439.51883	2822.48535	2843.49711	UBXN2B	2458.76777	-0.7259513
9042.86661	9082.426	6394.85611	7312.44358	UBXN7	7537.15215	-0.2780008
5211.3197	5073.26179	6197.77084	6064.25644	UCP2	4925.59695	0.13902869
5947.4758	6307.11369	5614.52664	5277.514	UNG	5345.01755	-0.1293282
28.0737494	19.3679431	171.448159	212.015135	UPK1A	64.925542	2.32305627
1348.57974	1465.12793	1796.19987	1697.16037	UPK3B	2109.49293	1.1959717
3275.27076	3293.68961	2953.87553	2904.81521	USP16	2813.44194	-0.1836549
1651.15237	1697.54324	2193.57505	2222.00176	USP3	1721.93332	0.33030978
4945.13897	4804.38917	6440.52221	6715.89119	USP54	5074.86784	0.35931126

3814.91061	3493.06549	2929.84074	3425.49944	UTP20	3155.35051	-0.1617783
612.423644	688.131624	426.216919	483.269793	VASH2	480.747499	-0.680044
1079.2797	1021.94382	1559.05663	1559.97411	VASN	1112.53608	0.40189585
10335.2989	10186.3988	7190.40762	7584.73753	VCL	9047.72384	-0.0973764
2821.9317	2976.96678	3364.06926	3419.2637	VGLL4	2844.06507	0.20472215
8968.00328	9045.9687	12740.0403	11589.1214	VMP1	8882.82195	0.21896792
8657.1125	8560.63083	7381.08361	7587.8554	VPS35	7416.77388	-0.1684228
1819.59487	1715.7719	1261.02525	1221.16561	VRK1	1497.52108	-0.2176552
9520.12035	9132.5548	11644.8551	11436.3458	WARS	8807.31376	0.10051074
4595.77675	4608.43116	4027.42942	4082.33064	WBP11	3918.35388	-0.2039719
5690.65298	5644.04646	5027.27663	4806.71568	WBSCR16	4846.49994	-0.1910652
2256.29764	2321.87459	1849.07641	1977.76864	WDFY1	1944.4685	-0.2114487
4231.85778	4265.50464	2717.53344	2870.51864	WDHD1	3607.10407	-0.2131598
1766.56667	1872.99402	2180.75649	2127.42638	WDR20	1834.60889	0.27714008
2247.97949	2337.82466	1761.75001	1846.81811	WDR76	1956.89081	-0.1968672
817.258038	746.235453	530.36767	469.759025	WDYHV1	640.149752	-0.3280795
4226.65894	4227.90804	3534.71626	3447.32452	WIPI1	3464.2261	-0.3250823
822.45688	887.507508	479.093454	445.855358	WISP2	683.595768	-0.4071775
1255.00057	1220.18041	1592.70533	1604.66357	WNT7B	1257.32453	0.29768383
21448.3445	21662.4747	17078.3197	19152.0339	WWC3	18014.9177	-0.2628559
811.019427	910.293324	620.898708	609.023868	XRCC2	693.128111	-0.3813926
20673.717	20367.1011	18138.2539	17152.4401	XRCC6	17476.8491	-0.2029183
4515.71458	4388.54804	3068.44136	3620.88593	XRN1	3694.60843	-0.2862309
11305.4029	11015.8024	12357.8872	12858.0944	YAP1	10977.2686	0.21138622
2183.51384	2181.74182	1623.95056	1466.43802	YEATS4	1860.37958	-0.2008169
3562.24687	3530.66209	2706.31721	2995.23343	YTHDC2	3013.05488	-0.2103254
11806.5713	11556.9656	10030.5185	10206.8659	YY1	10276.3566	-0.10454
401.350639	527.491626	745.879609	752.445871	ZBED5-AS1	504.181177	0.4698273
1933.9694	1892.36197	1485.34994	1595.30996	ZBTB25	1600.21729	-0.2614694
1566.93112	1503.86381	900.503416	1004.99331	ZBTB37	1107.08318	-0.7806346
4368.06745	4405.6374	3482.64088	4090.64496	ZBTB41	3664.3152	-0.2651062
4221.46009	4083.21811	4710.81858	4599.897	ZFAND5	4194.51638	0.28178321
6055.61172	5928.86916	4570.61565	4928.3126	ZFC3H1	5221.42982	-0.1331064
14558.8385	14131.7627	12018.1955	12567.0932	ZFP36L1	12711.485	-0.0832203
894.200907	890.925381	586.448844	623.573927	ZGRF1	734.533837	-0.3101765
691.44605	688.131624	460.666783	516.527069	ZKSCAN2	530.449834	-0.5364975
16259.8997	16161.9788	13143.0236	14581.237	ZMIZ1	14346.0531	-0.0873341
4638.40726	4810.08562	5571.26402	5928.10946	ZMIZ2	4527.07889	0.14025083
2743.94906	2575.93643	1962.03992	2134.70141	ZNF107	2262.64153	-0.2065176
13283.0425	13461.8597	10583.3186	11441.5423	ZNF217	11310.7629	-0.2243061
593.707811	616.356306	900.503416	857.414149	ZNF219	664.678779	0.49944604
2200.15014	2252.37785	1701.66304	1923.72556	ZNF292	1849.97037	-0.2795894
330.646382	371.40879	472.684178	474.955474	ZNF296	395.945241	0.56189631

1300.75039	1200.81247	1504.57777	1474.75234	ZNF302	1256.98055	0.27017046
1939.16825	1992.61955	1590.30185	1647.27446	ZNF37BP	1650.25801	-0.2469592
518.84448	527.491626	406.989088	416.755241	ZNF382	426.271637	-0.346317
1133.34766	1146.12651	893.29298	996.678993	ZNF431	939.796826	-0.300957
1867.42422	1909.45133	2522.05049	2265.65193	ZNF444	1967.23483	0.36154316
1596.04464	1512.97814	1950.82368	1814.60013	ZNF467	1678.76133	0.45234224
375.356427	405.587513	502.327084	506.13417	ZNF528-AS1	424.906087	0.47500036
532.36147	462.552052	769.914398	670.341971	ZNF579	564.965584	0.57201148
489.730962	516.098718	983.022857	1010.18976	ZNF613	542.566956	0.45334816
892.12137	845.35375	1015.06924	1064.23283	ZNF615	877.346924	0.27932284
1338.18205	1256.63772	1539.8288	1670.13883	ZNF710	1417.34942	0.48058708
2984.13558	2747.96933	3181.40486	3327.80619	ZNF736	2855.40966	0.24691554
512.605869	475.08425	289.218624	277.490397	ZNF85	396.622823	-0.3862194
781.905909	805.478573	1077.55969	903.142904	ZNF865	818.278921	0.33358374
1380.81256	1328.41304	990.233294	1001.87544	ZNF92	1146.07677	-0.2269097
667.531374	729.146092	1035.09823	986.286094	ZNHIT2	715.944715	0.32042874
19.7556014	21.6465246	72.9055257	59.239523	ZNRF2P1	35.6879344	1.46964467
3044.44216	3068.11004	3629.25309	3547.09635	ZSCAN18	3143.06182	0.32872051
2653.4892	2694.42267	1986.87586	2181.46945	ZYG11B	2261.64529	-0.2229761

lfcSE	stat	pvalue	padj	Control.0	Control.1	shRNA2.0
0.05606722	4.83199965	1.35E-06	1.24E-05	2522.31139	2494.26665	2973.59593
0.05322584	-8.3264204	8.33E-17	2.29E-15	3672.95588	3691.59765	2642.91375
0.04158018	16.5880008	8.51E-62	1.34E-59	4474.51779	4473.90923	7046.92209
0.05634137	-2.7780363	0.00546885	0.02215316	2811.63255	2883.34735	2531.83845
0.05660478	4.94536334	7.60E-07	7.23E-06	2578.27843	2667.53725	3099.08558
0.0447344	9.45524968	3.22E-21	1.16E-19	3724.17996	3719.61146	5006.01949
0.03943318	3.84606314	0.00012003	0.00077456	5458.20975	5515.60795	6059.11506
0.05341093	-4.4031546	1.07E-05	8.53E-05	3289.72392	3235.0763	2742.9663
0.08109211	5.67940727	1.35E-08	1.63E-07	1046.29916	1068.67498	1466.87224
0.04050894	2.70686871	0.00679211	0.02667808	5207.78094	5187.74262	5584.28936
0.16977879	-2.5815357	0.00983618	0.03675426	334.853674	356.916692	275.568485
0.05587006	8.10245013	5.39E-16	1.38E-14	3031.70635	2976.72671	3918.1599
0.06821753	-3.0293648	0.00245069	0.01118777	2033.78549	1931.91535	1711.91622
0.167273	2.68252451	0.00730688	0.02843487	259.91475	258.349582	379.860558
0.30681077	5.45103783	5.01E-08	5.63E-07	848.99161	937.943865	2254.57416
0.37381833	4.64228755	3.45E-06	2.97E-05	142.289097	161.85757	384.100073
0.12772098	2.49136032	0.0127255	0.04572052	496.11465	450.296059	615.5776
0.0419609	6.01965589	1.75E-09	2.39E-08	4702.18035	4795.54927	5719.95384
0.07109736	4.24246633	2.21E-05	0.00016731	1511.11021	1554.24769	1842.49329
0.0443924	-9.4898664	2.31E-21	8.41E-20	5155.60827	5125.4897	3803.69299
0.09244201	-2.9175162	0.00352831	0.01525531	1112.70074	1047.92401	881.819152
0.03479218	-2.62896	0.00856464	0.03264258	9837.86815	9358.68768	8979.29309
0.03262019	-10.324718	5.45E-25	2.41E-23	13209.1711	12656.0169	10089.1982
0.0508936	3.96848417	7.23E-05	0.00049128	3151.22919	3193.57435	3707.87995
0.10446428	-3.4858642	0.00049055	0.00273119	830.019731	880.878696	664.755976
0.02461134	3.51530299	0.00043925	0.00247749	19195.7477	19130.3197	20134.3054
0.02290156	-4.1222168	3.75E-05	0.00027029	23016.6843	22922.5595	21561.3262
0.04909534	-6.0362071	1.58E-09	2.17E-08	4656.64784	4551.72537	3629.02497
0.03163506	7.99151451	1.33E-15	3.31E-14	9466.01931	9785.12012	11447.5388
0.04970251	-6.3008247	2.96E-10	4.51E-09	3852.24014	3857.60541	3125.37057
0.04958757	4.06062433	4.89E-05	0.00034305	3463.31661	3358.54457	3867.28572
0.05326567	-2.7232549	0.00646422	0.02554283	3202.45327	3172.82338	2845.56257
0.06105083	-3.2293607	0.00124067	0.00617705	2576.38124	2453.80226	2178.26289
0.02083458	-7.7605713	8.45E-15	1.95E-13	46543.7121	45232.9654	41282.7027
0.05422341	-7.8032108	6.04E-15	1.41E-13	3961.32845	4296.48843	3126.21847
0.02569597	-2.8018819	0.00508055	0.02073873	17025.3647	16920.3413	16046.5649
0.03281544	-2.9736573	0.00294274	0.01306381	9452.73899	9599.39894	8813.1041
0.04081271	-3.3910948	0.00069614	0.00372317	5839.54453	5685.76591	5253.60718
0.04817527	-15.766395	5.30E-56	6.70E-54	4784.70803	4753.00978	2838.77935
0.04531321	4.53261102	5.83E-06	4.87E-05	4063.7766	4199.99642	4805.91438
0.09507561	-7.6629166	1.82E-14	4.08E-13	1552.84834	1441.1549	830.097067
0.03032813	12.0469832	2.01E-33	1.31E-31	9550.44417	9415.75285	12167.4085

0.02135201	-2.704269	0.00684549	0.02685562	44776.4815	46245.6128	44070.6079
0.06088237	-12.808449	1.47E-37	1.09E-35	2935.89836	2931.07458	1731.41799
0.03815358	-7.5713327	3.69E-14	8.08E-13	7289.94472	7218.22508	6031.98216
0.04168675	-6.6230696	3.52E-11	5.86E-10	5906.8947	5815.45947	4912.75016
0.01821783	-21.299989	1.14E-100	3.45E-98	63142.2096	64145.3999	48536.5132
0.05904473	-9.4748475	2.67E-21	9.67E-20	3178.73842	3295.25411	2127.3887
0.06134753	-7.1489232	8.75E-13	1.70E-11	2617.17079	2571.04524	1894.21537
0.05977803	-3.1689274	0.00153003	0.0074067	2829.65584	2729.79016	2363.95365
0.15168002	3.01831519	0.00254184	0.01155046	302.601479	295.701329	397.666521
0.0518538	-10.581799	3.62E-26	1.67E-24	4606.37236	4416.84406	2987.16238
0.02633749	10.1997021	1.99E-24	8.49E-23	15356.7879	15132.6452	18559.7494
0.0958279	-5.3108866	1.09E-07	1.18E-06	1086.1401	1085.27576	790.245624
0.13221363	-6.8803597	5.97E-12	1.07E-10	628.917807	657.805764	325.594764
0.08811435	8.28180644	1.21E-16	3.28E-15	869.860678	806.175203	1396.49629
0.05671598	3.17954817	0.00147505	0.00717863	2529.90014	2456.9149	2812.49435
0.07224847	7.82149914	5.22E-15	1.23E-13	1941.77187	2049.15833	2733.63937
0.07401186	2.5894425	0.00961315	0.03601095	1657.19368	1505.48291	1799.25023
0.02645434	-6.73781	1.61E-11	2.78E-10	16054.0045	15915.9943	14099.7795
0.03960815	-10.820799	2.74E-27	1.33E-25	7445.51413	7173.61049	5496.95535
0.12624181	3.139146	0.00169441	0.00809278	491.371681	457.558899	658.820655
0.13949965	-4.9557024	7.21E-07	6.90E-06	548.287319	527.07465	316.26783
1.44305768	11.7216902	9.87E-32	5.92E-30	0	0	21948.8178
1.56105434	4.9770431	6.46E-07	6.22E-06	0	0	44.9388606
0.36607353	-5.8253005	5.70E-09	7.29E-08	111.934089	117.242983	27.132897
0.03597578	-2.6127398	0.00898196	0.03401099	8092.45523	7853.20477	7393.71443
0.03193559	-3.4300727	0.00060342	0.00326769	9954.54521	9811.05884	9125.13241
0.0518709	-3.8448391	0.00012063	0.00077725	3461.41943	3483.05039	2975.29173
0.03445917	-9.6139595	6.98E-22	2.64E-20	8766.90554	8838.87587	7033.35564
0.0675195	4.00203115	6.28E-05	0.00043154	1646.75915	1707.80487	2012.92179
0.06580419	-8.083613	6.29E-16	1.60E-14	2301.28899	2358.34779	1633.06124
0.04632377	-19.110905	2.05E-81	4.61E-79	5576.784	5532.20873	2958.33367
0.20074906	3.41035153	0.00064879	0.00349153	165.055352	154.59473	240.804461
0.03354883	-16.222075	3.52E-59	5.03E-57	10126.2407	10036.2069	6953.65275
0.03856569	16.4284439	1.20E-60	1.79E-58	6933.27339	7143.52158	10548.7616
0.07585157	3.27563636	0.00105424	0.00533617	1348.90064	1297.9732	1571.16432
0.04751877	3.93151879	8.44E-05	0.00056149	3627.42337	3751.77546	4199.66371
0.06168785	-7.1243583	1.05E-12	2.02E-11	2564.04952	2599.05905	1874.7136
0.0508272	8.31568466	9.12E-17	2.49E-15	2871.39397	2825.24463	3835.06541
0.0603509	7.70209673	1.34E-14	3.05E-13	2900.80038	2931.07458	3774.01639
0.06329439	3.61216955	0.00030365	0.00177667	2011.01923	2115.56144	2369.04107
0.08504518	3.63923241	0.00027345	0.00161612	1078.55135	1031.32323	1340.53469
0.10407668	2.6919826	0.00710287	0.02773291	709.548296	754.297776	848.750934
0.04869263	-9.1151549	7.86E-20	2.62E-18	4458.3917	4334.87773	3275.44941

0.07161309	4.84242487	1.28E-06	1.18E-05	1423.83956	1431.81696	1838.25377
0.01798883	-7.0960464	1.28E-12	2.46E-11	65070.7011	65719.361	60318.1258
0.04022121	-8.6737475	4.18E-18	1.24E-16	6176.29539	6172.37616	4825.41615
0.03509027	4.27117691	1.94E-05	0.00014852	7179.90782	7243.12624	7943.15559
0.1553719	11.0743159	1.67E-28	8.66E-27	167.901134	184.683637	596.923733
0.04559302	16.0892936	3.03E-58	4.15E-56	3337.15361	3322.23037	5446.08116
0.02857729	-7.0289762	2.08E-12	3.91E-11	14522.0252	14970.7876	12805.8795
0.08007476	-2.4836558	0.01300414	0.04652833	1384.94721	1354.00082	1186.21634
0.06366256	-2.9748189	0.00293162	0.01303481	2227.29866	2194.41513	1915.41295
0.03378087	6.39211852	1.64E-10	2.55E-09	7584.00885	7551.27815	8762.22992
0.04137087	-2.4809751	0.01310235	0.04679818	5433.54631	5359.97567	4998.38836
0.05400752	-4.803373	1.56E-06	1.42E-05	3571.45633	3825.4414	3043.97188
0.02443819	-6.0425875	1.52E-09	2.10E-08	25202.2448	24372.0148	22323.591
0.04951075	-3.2506495	0.00115142	0.00577466	3780.147	3814.02837	3448.42163
0.03489842	-3.8708225	0.00010847	0.00070608	8234.74432	8396.8802	7637.9105
0.04610589	6.86242111	6.77E-12	1.21E-10	3773.50684	3774.60153	4624.46313
0.04638148	-12.84834	8.79E-38	6.62E-36	5203.03797	5041.44827	3417.89712
0.02701765	-3.1644176	0.00155394	0.00750917	15073.1583	15341.1925	14371.1085
0.08755284	4.49194974	7.06E-06	5.79E-05	990.332113	977.370709	1241.33004
0.07104143	2.47949007	0.01315704	0.04691193	1556.64272	1647.62705	1800.09813
0.04299723	6.68233976	2.35E-11	4.01E-10	4496.33546	4688.68178	5616.50967
0.03919084	-7.4760573	7.66E-14	1.64E-12	6505.4575	6634.08526	5387.57586
0.04953684	6.06048654	1.36E-09	1.89E-08	3521.18085	3764.22604	4442.16398
0.07137566	22.2185558	2.27E-109	8.49E-107	1036.81322	996.046582	3178.78846
0.06907412	-2.708658	0.00675559	0.02656639	1937.0289	1872.77508	1644.93188
0.24381318	-3.2089483	0.00133221	0.00656862	194.461765	167.045312	111.075297
0.08326409	7.08882833	1.35E-12	2.58E-11	978.000391	941.056511	1443.13096
0.03654779	-4.894716	9.84E-07	9.25E-06	7255.79534	7249.35153	6375.38289
0.05784028	-3.5902036	0.00033042	0.00192306	2743.33378	2720.45223	2332.58124
0.0622719	2.88839026	0.00387219	0.01648189	2017.65939	2003.5062	2268.14061
0.12049407	8.24389065	1.67E-16	4.47E-15	378.488997	400.49373	762.264824
0.21278693	2.69692728	0.00699826	0.02737651	148.929255	141.106599	206.888339
0.03276644	-18.065545	5.95E-73	1.16E-70	11058.7086	10990.7515	7241.93978
0.0458718	-2.7838011	0.0053726	0.02180088	4388.19574	4397.13064	4072.47826
0.08848262	-2.4704581	0.01349401	0.04793656	1152.54168	1142.34092	1012.39622
0.02629087	-5.9096972	3.43E-09	4.52E-08	16543.479	16322.7134	14717.0529
0.04713445	2.493413	0.01265216	0.04547691	4133.02396	3937.49665	4395.52931
0.07292448	-3.7450792	0.00018034	0.00111682	1699.88041	1692.24164	1392.25678
0.03936489	-4.4984783	6.84E-06	5.63E-05	6667.66707	6488.82846	5891.23026
0.05882313	-9.4335023	3.97E-21	1.41E-19	2924.51523	2966.35123	2028.18405
0.04297191	-3.465214	0.00052981	0.00292696	5223.90704	5367.23851	4714.34085
0.0494751	7.11413523	1.13E-12	2.16E-11	3358.02268	3210.17513	4221.70919
0.08730004	-5.0444644	4.55E-07	4.50E-06	1237.91514	1231.5701	913.191564

0.04472679	-19.503632	1.02E-84	2.40E-82	6395.4206	6177.56391	3486.57726
0.06555137	3.00182475	0.00268367	0.01209011	1941.77187	1947.47858	2156.21741
0.03981266	2.76176524	0.00574898	0.02312258	7102.12311	6760.66618	7642.15001
0.0502984	4.12027306	3.78E-05	0.00027246	3187.27577	3151.03486	3623.93755
0.05133631	4.1335428	3.57E-05	0.00025821	3249.88297	3118.87086	3646.83093
0.06986943	-7.3496658	1.99E-13	4.10E-12	2009.12205	2012.84413	1423.62919
0.02418403	-6.3757462	1.82E-10	2.82E-09	22307.136	21823.7956	19858.7369
0.08331327	11.787923	4.51E-32	2.72E-30	871.757866	833.151464	1643.23607
0.03796179	-2.9746634	0.0029331	0.01303513	6830.82524	6700.48836	6196.47535
0.04694484	6.77849592	1.21E-11	2.12E-10	3512.6435	3605.48112	4427.74963
0.07260719	3.10369646	0.00191119	0.00900513	1508.26443	1441.1549	1726.33057
0.02516242	3.16651666	0.00154277	0.00746321	18980.4169	19372.0685	20459.0522
0.12607269	6.48728805	8.74E-11	1.39E-09	386.077749	373.517468	685.953552
0.10315067	-6.2477547	4.16E-10	6.23E-09	1011.20118	999.159228	675.778715
0.12447779	2.86975614	0.00410788	0.01733245	458.170891	453.408705	583.357285
0.25213444	2.50828385	0.01213192	0.04391438	102.44815	112.055241	184.842861
0.11485796	-8.1670989	3.16E-16	8.19E-15	878.398024	917.192894	440.061673
0.22471244	4.35902271	1.31E-05	0.0001029	105.293932	134.881308	222.998497
0.10587441	-2.73342	0.00626803	0.0248573	903.061467	839.376755	746.154667
0.10758771	2.91042775	0.00360934	0.01558106	651.684063	672.331443	785.158206
0.04982769	6.6340524	3.27E-11	5.46E-10	3352.33112	3328.45566	4094.52373
0.03864866	-5.9400845	2.85E-09	3.80E-08	6938.01636	6788.67999	5785.24238
0.04214236	4.08443961	4.42E-05	0.00031317	5015.21636	5256.22082	5792.8735
0.12950202	-2.8041861	0.00504438	0.02063723	568.207793	532.262392	428.19103
0.01862077	-25.621169	8.86E-145	6.07E-142	61879.631	61230.9261	43895.9399
0.06258437	4.43617296	9.16E-06	7.40E-05	1940.82328	1964.07935	2398.71767
0.06044028	-7.2754845	3.45E-13	7.01E-12	2946.3329	2793.08062	2149.43418
0.05392783	3.44086185	0.00057986	0.00315261	3303.95283	3116.79576	3743.49188
0.0392505	-2.5775232	0.00995112	0.03712463	6117.48256	6041.64505	5703.84369
0.09957981	3.58279314	0.00033994	0.00197148	745.594867	721.096224	959.826231
0.09740987	3.15056468	0.00162955	0.00782394	775.00128	802.025008	948.803491
0.12351716	5.69156975	1.26E-08	1.54E-07	420.227132	405.681473	686.801455
0.0274901	9.0950048	9.46E-20	3.13E-18	12646.6549	12593.764	15103.6967
0.06102379	5.46476398	4.64E-08	5.24E-07	1979.71563	2013.88168	2501.31394
0.1317096	2.65572397	0.00791384	0.03047207	424.021508	509.436325	594.380024
0.03793471	-5.2811081	1.28E-07	1.36E-06	6714.14818	6728.50217	5817.46269
0.06302636	-2.6242032	0.00868519	0.03300896	2227.29866	2278.45656	2032.42356
0.04210507	6.64961518	2.94E-11	4.94E-10	4567.48	4515.41117	5474.90987
0.02474238	-4.9113491	9.05E-07	8.53E-06	21645.966	21078.8358	19595.8869
0.05349652	6.54281253	6.04E-11	9.85E-10	2656.06314	2674.80009	3348.36907
0.02446687	-7.7168455	1.19E-14	2.73E-13	25917.4847	25412.676	22845.0514
0.16631975	-2.8618955	0.00421116	0.01768643	377.540403	360.029338	241.652364
0.06201472	3.42857464	0.00060676	0.0032847	2070.78065	2022.18207	2342.75607

0.05224359	-2.6067675	0.00914014	0.03452241	3382.68613	3304.59205	3008.35995
0.43990158	2.89673859	0.00377064	0.01612483	24.6634434	28.0138101	72.0717576
0.02874959	8.40866948	4.15E-17	1.17E-15	11326.2121	11528.2016	13526.597
0.06790101	3.61262641	0.00030311	0.00177481	1678.06275	1671.49067	2020.55292
0.02959769	4.0616348	4.87E-05	0.00034186	11002.7416	11125.6328	11946.9537
0.07166741	4.00772809	6.13E-05	0.00042233	1449.4516	1501.33271	1817.9041
0.13638393	4.32939354	1.50E-05	0.00011637	352.87696	360.029338	556.224388
0.20296288	-2.4930038	0.01266675	0.0455194	239.045682	225.14803	157.709964
0.14428238	-2.5746288	0.01003477	0.03737731	436.35323	438.883025	331.530085
0.03644527	2.72110068	0.00650649	0.02568518	6716.04536	6727.46462	7139.34352
0.05581566	8.91902604	4.70E-19	1.48E-17	2347.7701	2371.83592	3264.42667
0.06156101	-4.0507011	5.11E-05	0.00035626	2701.59565	2544.06898	2241.00771
0.03971003	-9.5586946	1.19E-21	4.42E-20	6787.18991	6627.85997	5201.88509
0.10332637	3.33188742	0.00086259	0.00449204	701.01095	661.955958	868.252703
0.03782077	-2.5714812	0.01012645	0.03764199	6588.93377	6704.63856	6181.21309
0.04413779	-5.2712296	1.36E-07	1.44E-06	5051.26293	4996.83369	4213.23016
0.0434529	-6.2380395	4.43E-10	6.59E-09	5178.37453	5299.79786	4377.72335
0.08393419	-9.275462	1.77E-20	6.12E-19	1666.67962	1538.68446	934.38914
0.02516179	-3.576046	0.00034883	0.00201735	17854.4359	18077.2079	16868.1829
0.03573398	-3.6187882	0.00029599	0.00173804	9893.83519	10378.5979	9449.03137
0.02708307	-3.9070259	9.34E-05	0.00061507	15505.7172	15405.5205	14505.0771
0.2154859	-6.183928	6.25E-10	9.07E-09	252.325998	292.588684	110.227394
0.04498184	-4.0353285	5.45E-05	0.00037816	4658.54503	4583.88938	4049.58487
0.27592171	-3.8292531	0.00012853	0.0008246	151.775036	147.33189	66.1364364
0.03113156	-7.3410043	2.12E-13	4.37E-12	16225.7	16769.8968	14436.397
0.04130103	-3.6860996	0.00022772	0.00137653	5463.90131	5459.58033	4942.42676
0.05559903	-17.772288	1.16E-70	2.14E-68	3834.21686	3946.83458	1933.21891
0.04589625	3.11958868	0.00181104	0.00859236	3919.59032	3908.44529	4294.62885
0.06333356	4.27476012	1.91E-05	0.0001467	2144.77098	2330.33398	2759.92436
0.04918781	-8.3403263	7.41E-17	2.06E-15	4064.7252	4063.04002	3077.888
0.06813291	-3.3171451	0.00090942	0.00470461	1931.33734	2007.65639	1695.80606
0.15768441	3.67914407	0.00023402	0.00140943	289.321163	268.725068	390.035394
0.1616902	3.2955553	0.00098227	0.00503078	263.709126	245.899	367.142012
0.0603078	6.34321934	2.25E-10	3.45E-09	2085.00956	2009.73149	2677.67777
0.061235	4.21465204	2.50E-05	0.00018691	2130.54207	2047.08324	2483.50798
0.0205092	-7.4634124	8.43E-14	1.79E-12	41251.5063	40825.4593	37307.7333
0.11741582	4.07778519	4.55E-05	0.00032171	670.655942	570.651688	810.595297
0.14466574	6.0705239	1.27E-09	1.78E-08	1562.33428	1619.61324	2630.1952
0.13592574	3.44661179	0.00056766	0.00309755	390.820719	427.469992	527.395685
0.04487485	-12.595207	2.24E-36	1.59E-34	5467.69569	5625.58809	3694.3135
0.06989849	-2.5061646	0.01220488	0.04410902	1840.27232	1794.95895	1628.82172
0.10412165	-3.0118066	0.00259698	0.01176648	826.225355	834.189013	659.668558
0.08100851	3.67936008	0.00023382	0.00140891	1172.46216	1147.52867	1463.48063

0.08312677	-4.3074796	1.65E-05	0.00012779	1328.03157	1383.05218	1048.85605
0.12719681	6.78883923	1.13E-11	1.98E-10	370.900245	397.381084	730.892412
0.04344449	-4.8275556	1.38E-06	1.27E-05	4951.66057	4981.27046	4280.2145
0.0377758	5.86475691	4.50E-09	5.81E-08	7286.15035	7659.1832	8555.34158
0.04271794	-8.6419134	5.53E-18	1.62E-16	5656.46589	5559.18499	4295.47675
0.10798122	-2.8358791	0.00456997	0.01897515	780.692844	792.687072	656.276946
0.08963358	-11.773341	5.36E-32	3.22E-30	1556.64272	1499.25762	767.352243
0.10546951	2.5920594	0.00954033	0.03581875	682.987664	741.847194	829.249164
0.39987602	3.45171978	0.00055703	0.0030537	23.7148495	42.5394895	80.5507879
0.06091982	-3.9964242	6.43E-05	0.00044115	2467.29294	2443.42677	2053.62114
0.06134803	-5.0042842	5.61E-07	5.48E-06	2444.52668	2456.9149	1973.91825
0.04587373	-4.9399585	7.81E-07	7.43E-06	4610.16673	4548.61273	3861.3504
0.07174127	5.68717659	1.29E-08	1.57E-07	1402.97049	1426.62922	1840.79748
0.07584232	-2.9080811	0.00363654	0.01567787	1530.08209	1532.45917	1313.40179
0.04860284	-3.242203	0.00118609	0.00593409	3915.79594	3859.68051	3518.79758
0.03415653	3.51177366	0.00044513	0.00250546	8263.20214	8054.48919	8937.74584
0.26152808	4.45286635	8.47E-06	6.89E-05	92.0136159	68.4782025	178.907539
0.06089104	5.19971364	2.00E-07	2.06E-06	2098.28988	2230.72932	2674.28616
0.03011499	-6.4042321	1.51E-10	2.36E-09	13998.4013	14532.9422	12344.6202
0.1099682	7.06197013	1.64E-12	3.12E-11	675.398912	723.171321	1076.83685
0.06530575	-2.8025484	0.00507006	0.02071137	2104.93004	2056.42117	1829.77474
0.03993136	12.5339787	4.87E-36	3.40E-34	7106.86608	7461.01143	9953.53367
0.02926887	27.1611478	1.87E-162	1.62E-159	11229.4555	11808.3397	20011.3594
0.0461763	15.2347115	2.08E-52	2.29E-50	3637.85791	3882.50657	6035.37377
0.03880978	7.30418064	2.79E-13	5.71E-12	5621.36791	5484.48149	6729.80635
0.05273126	-9.6279933	6.09E-22	2.31E-20	3623.629	3595.10563	2549.64441
0.06676573	4.87023081	1.11E-06	1.04E-05	1679.95994	1774.20797	2156.21741
0.11557227	-10.413515	2.15E-25	9.60E-24	928.673504	910.967603	380.708461
0.03848823	-3.644794	0.00026761	0.00158385	6608.85424	6516.84227	5893.77396
0.12898458	3.94531342	7.97E-05	0.00053423	407.895411	475.197224	635.927273
0.04786757	4.30388168	1.68E-05	0.00012982	3778.24981	3657.35854	4225.9487
0.0309127	-3.4840017	0.00049398	0.00274561	11723.673	12052.1636	10910.8162
0.03779291	-3.9540802	7.68E-05	0.00051776	6609.80284	6620.59713	5957.36669
0.07130675	-3.7611006	0.00016917	0.00105441	1873.47311	1786.65856	1527.07336
0.06764882	-4.1150093	3.87E-05	0.00027778	2084.06097	2191.30248	1801.79394
0.05886983	3.00488831	0.00265678	0.01199532	2351.56447	2483.89116	2748.90162
0.06847282	3.51519442	0.00043943	0.00247765	1872.52451	1813.63482	2090.08097
0.09391034	6.18668657	6.14E-10	8.92E-09	752.235025	739.772097	1124.31942
0.09549552	4.41243765	1.02E-05	8.20E-05	890.729745	908.892506	1125.16732
0.02393393	-6.1682304	6.91E-10	9.97E-09	23772.7137	23762.9738	21179.7698
0.06057149	-4.4480308	8.67E-06	7.03E-05	2578.27843	2496.34175	2103.64742
0.05339548	-5.0066042	5.54E-07	5.42E-06	4778.06787	4334.87773	3718.05479
0.08340822	3.44907762	0.0005625	0.00307348	1085.19151	1123.66505	1383.77775

0.08988671	7.84920654	4.19E-15	9.91E-14	807.253475	788.536878	1332.90356
0.03915942	-6.1930156	5.90E-10	8.61E-09	7117.30062	6927.71149	6036.22167
0.09158976	-5.1640775	2.42E-07	2.47E-06	1135.46699	1144.41602	833.488679
0.03855084	6.96583817	3.26E-12	5.99E-11	5963.81034	6169.26352	7214.80689
0.02748808	-10.277619	8.89E-25	3.87E-23	16233.2887	16437.8813	13274.7698
0.03954729	13.1642177	1.41E-39	1.10E-37	5318.76643	5405.62781	7525.1394
0.02752012	-5.0738296	3.90E-07	3.89E-06	28611.4916	28940.341	26915.8338
0.72528322	-2.6063622	0.00915096	0.03454741	22.7662555	32.1640042	7.63112727
0.02874633	-7.6860603	1.52E-14	3.43E-13	13011.8636	13001.5205	11122.792
0.03543707	-3.5679959	0.00035972	0.00207504	7734.8353	7839.71664	7178.34706
0.06233468	4.27190439	1.94E-05	0.00014818	2160.89708	2356.2727	2737.03098
0.03182117	-11.100253	1.25E-28	6.55E-27	12830.6821	12330.2266	9790.73629
0.03060732	15.2634852	1.34E-52	1.50E-50	9043.89499	8964.41924	12350.5555
0.03177533	5.43424177	5.50E-08	6.17E-07	9117.88532	9001.77099	10161.2699
0.06708304	3.16183321	0.00156779	0.00756945	1733.0812	1815.70992	2031.57566
0.02326088	-8.2271561	1.92E-16	5.10E-15	23010.9927	22964.0615	20225.8789
0.057509	-3.9616865	7.44E-05	0.00050352	2742.38519	2762.99172	2370.73687
0.07492913	2.74959498	0.0059669	0.02385786	1793.79121	1586.41169	1919.65246
0.04269019	-3.7549193	0.0001734	0.00107791	5126.20186	5174.25449	4648.20441
0.03604935	9.29468847	1.48E-20	5.15E-19	6251.23432	6346.68432	7939.76398
0.23129907	2.69623518	0.00701281	0.02741388	131.854563	117.242983	206.040436
0.06407249	-4.9353097	8.00E-07	7.60E-06	2299.3918	2351.08495	1831.47055
0.04911851	4.42806012	9.51E-06	7.66E-05	3277.39219	3322.23037	3817.25944
0.04885929	-2.6943399	0.00705282	0.02755716	4168.12194	4018.42543	3669.72432
0.14316789	3.48884329	0.00048512	0.00270551	332.956486	348.616304	457.867636
0.21711749	3.57270882	0.00035331	0.00204037	120.471435	147.33189	226.390109
0.09719935	7.41498937	1.22E-13	2.56E-12	819.585197	877.766051	1291.35632
0.05690747	-3.6335083	0.00027959	0.00164945	3780.147	3845.15483	3478.94613
0.06018665	-3.4851599	0.00049184	0.00273561	2608.63344	2779.59249	2332.58124
0.05953843	-2.4606521	0.01386848	0.04906526	2520.4142	2498.41684	2296.12141
0.02588481	-2.9768208	0.00291254	0.01297183	19842.6888	19272.4638	18682.6954
0.03069817	2.48049242	0.01312011	0.04683105	9989.64318	10049.695	10527.564
0.05144157	8.05632188	7.86E-16	1.99E-14	2839.14178	2798.26837	3798.60558
0.04895083	-5.9085947	3.45E-09	4.54E-08	4102.66895	4238.38572	3457.74856
0.0409302	12.7697384	2.42E-37	1.75E-35	4586.45188	4545.50008	6467.80432
0.10745417	24.9732811	1.19E-137	7.26E-135	266.554908	240.711257	1647.47559
0.54144767	-2.6725226	0.00752833	0.02919294	62.6072025	40.4643924	26.2849939
0.13247794	3.17317586	0.00150781	0.00730993	387.026343	409.831667	539.266327
0.17113979	-3.7547682	0.0001735	0.00107815	341.493832	422.282249	248.435588
0.0336165	4.53212571	5.84E-06	4.88E-05	7833.48907	7895.74426	8770.70895
0.02605584	-4.2354437	2.28E-05	0.00017199	20601.564	19869.0542	18743.7444
0.04391798	-5.1220636	3.02E-07	3.06E-06	4928.89431	5048.71111	4254.77741
0.04705756	-4.3093448	1.64E-05	0.00012678	4151.04725	4157.45693	3600.19627

0.03997243	9.45051617	3.37E-21	1.21E-19	5087.3095	5279.04689	6743.3728
0.10646132	-2.9842321	0.00284291	0.0127134	808.202069	816.550688	630.839855
0.04763954	-8.9678826	3.02E-19	9.68E-18	4381.55558	4432.40729	3300.8865
0.0426805	-3.6307067	0.00028265	0.00166567	5298.84596	5279.04689	4823.72034
0.04808686	-3.4020521	0.00066882	0.0035946	3939.51079	3902.22	3498.4479
0.17216965	-6.4895473	8.61E-11	1.38E-09	513.189342	498.023291	193.321891
0.0430519	4.41617839	1.00E-05	8.08E-05	4709.7691	4930.43058	5486.78051
0.05539503	-6.0075729	1.88E-09	2.56E-08	3106.64528	3218.47552	2516.57619
0.14798159	3.20494653	0.00135088	0.0066412	308.293043	324.752688	423.103612
0.02033675	9.06654793	1.23E-19	4.03E-18	46757.1458	46970.8592	52429.236
0.02748293	-8.6918068	3.57E-18	1.06E-16	15027.6258	14840.0565	12632.0593
0.04401247	-2.4587903	0.0139406	0.04927799	4750.55864	4769.61056	4365.0048
0.10295371	-2.8434427	0.0044629	0.01859637	845.197234	901.629667	725.804994
0.02493338	-3.1850106	0.00144749	0.00705913	17939.8093	17930.9136	16930.9277
0.03184695	15.3203025	5.60E-53	6.34E-51	8465.25266	8547.32473	11770.5899
0.2241679	12.0655739	1.61E-33	1.06E-31	49.3268869	84.0414304	443.453285
0.08577053	9.81984459	9.25E-23	3.67E-21	887.883963	978.408257	1730.57009
0.02509863	-15.16121	6.39E-52	6.95E-50	22425.7102	23052.2531	17525.3077
0.0342536	-8.01302	1.12E-15	2.80E-14	9581.74777	9890.95007	7962.65736
0.09693006	8.21326561	2.15E-16	5.70E-15	696.26798	642.242536	1165.86667
0.16188126	7.42757247	1.11E-13	2.33E-12	187.821608	212.697447	462.107152
0.07900287	2.70198124	0.00689277	0.02700886	1206.61154	1224.30726	1415.99806
0.05066987	-13.001278	1.20E-38	9.19E-37	4290.49056	4283.0003	2663.26342
0.03127136	-4.5506955	5.35E-06	4.49E-05	10516.1128	10420.0998	9538.90909
0.06661878	3.04070091	0.00236028	0.01082317	1891.49639	1791.8463	2150.28209
0.07475603	-8.2383354	1.75E-16	4.66E-15	1835.52935	1788.73365	1185.36844
0.10412606	2.53965799	0.01109609	0.04068484	684.884852	683.744477	805.507879
0.09158156	-7.2847097	3.22E-13	6.56E-12	1217.04607	1306.27359	792.789334
0.02029659	-11.655188	2.16E-31	1.28E-29	49459.69	50912.506	42608.8231
0.04570241	-9.1353877	6.52E-20	2.18E-18	5037.98262	4876.47806	3708.72786
0.15650406	4.0727485	4.65E-05	0.00032791	273.195066	253.16184	402.753939
0.08989309	3.95227064	7.74E-05	0.00052063	888.832557	882.953793	1123.47152
0.05093019	-6.2200541	4.97E-10	7.35E-09	3747.89481	3666.69648	2959.18158
0.0880738	3.90271808	9.51E-05	0.00062487	927.72491	982.558451	1222.67617
0.05626859	2.82413359	0.00474086	0.01954639	2647.52579	2766.10436	2967.66061
0.2519078	3.13426958	0.00172283	0.00821417	92.0136159	96.4920127	158.557867
0.10015159	10.0404091	1.01E-23	4.16E-22	602.357176	564.426397	1137.88587
0.22776189	-2.7747352	0.00552467	0.02235171	191.615984	189.87138	128.033358
0.05945209	-3.1592501	0.00158176	0.0076277	2753.76832	2932.11213	2542.86119
0.08746147	-2.8496899	0.00437619	0.01827676	1150.6445	1190.06816	992.894449
0.02565464	-7.577252	3.53E-14	7.75E-13	18188.3409	18492.2273	15948.2081
0.10582539	3.64610771	0.00026624	0.00157635	642.198123	629.791954	813.139006
0.04142063	-5.9217869	3.18E-09	4.21E-08	5558.76071	5595.49919	4710.10133

0.017432	-4.0723716	4.65E-05	0.0003283	92128.3957	90448.2925	87486.6347
0.02749023	-2.7498719	0.00596186	0.02384532	17617.2874	16959.7682	16349.2662
0.05218257	4.79446841	1.63E-06	1.48E-05	2994.71119	2936.26232	3584.08611
0.0476353	6.96337338	3.32E-12	6.08E-11	3688.13339	3823.36631	4627.00684
0.15394317	3.99202959	6.55E-05	0.00044903	265.606314	309.18946	434.126352
0.03475496	-2.9487469	0.00319065	0.01400272	9230.768	8851.32645	8423.91661
0.10422509	5.87662041	4.19E-09	5.43E-08	603.30577	594.515304	901.320921
0.20649873	-2.8952827	0.00378817	0.01619138	238.097088	233.448418	144.143515
0.03426178	-14.217432	7.14E-46	6.45E-44	9850.19987	10070.446	7178.34706
0.05424517	-4.0889545	4.33E-05	0.00030807	3107.59387	3079.44402	2642.06584
0.0270078	-6.9583928	3.44E-12	6.28E-11	19652.97	19055.6162	17202.2567
0.03511081	-4.628408	3.68E-06	3.16E-05	9270.60895	8880.37781	8131.39006
0.02451004	-10.314154	6.08E-25	2.68E-23	20187.0284	20145.0421	16848.6811
0.03385316	-6.1032725	1.04E-09	1.47E-08	9591.23371	9382.5513	8321.32034
0.01887155	-8.1153803	4.84E-16	1.24E-14	56512.4862	55342.8382	50391.725
0.02066611	-9.6730708	3.92E-22	1.50E-20	40162.5204	39958.0687	35279.5493
0.04857309	6.65375305	2.86E-11	4.82E-10	3776.35263	3589.91789	4526.95428
0.46732677	2.59810326	0.00937403	0.03528415	25.6120374	20.7509705	54.265794
0.04562815	10.2945881	7.45E-25	3.26E-23	3508.84912	3491.35078	4846.61372
0.16501336	-3.5363107	0.00040576	0.00231572	372.797433	384.930502	233.173333
0.0701845	-5.3521092	8.69E-08	9.49E-07	1892.44499	1897.67625	1467.72015
0.19958163	3.3429978	0.00082879	0.00433799	194.461765	151.482084	271.32897
0.17468314	6.28741776	3.23E-10	4.88E-09	172.644104	182.60854	364.598303
0.16106698	4.21605906	2.49E-05	0.00018583	256.120374	286.363392	397.666521
0.0176909	-15.486345	4.29E-54	5.00E-52	75042.321	74351.7647	61294.9101
0.02622576	3.46918451	0.00052204	0.00289182	17189.4715	17016.8333	18469.0238
0.30864057	4.08787282	4.35E-05	0.00030934	54.0698567	51.8774262	117.010618
0.03838191	11.3325555	9.05E-30	4.94E-28	5524.61133	5374.50135	7408.97668
0.09111012	3.37551755	0.00073677	0.00391299	873.655054	922.380637	1101.42604
0.05150753	8.50041985	1.89E-17	5.42E-16	3087.6734	2914.4738	4044.49746
0.04611877	4.06110592	4.88E-05	0.00034249	4359.73792	4523.71156	4930.55612
0.04652809	-2.7409101	0.00612693	0.02439782	4139.66412	4177.17035	3804.5409
0.0285213	13.5929709	4.41E-42	3.70E-40	11511.1879	11468.0238	14858.6527
0.02010328	-6.4983221	8.12E-11	1.31E-09	36188.8603	35826.5505	32868.1131
0.09165143	-3.4436644	0.00057389	0.00312321	1220.84045	1121.58995	964.913649
0.03021305	-19.543753	4.66E-85	1.11E-82	13458.6514	13258.8326	8831.75797
0.06704566	8.75518089	2.04E-18	6.17E-17	1542.41381	1616.5006	2419.06735
0.05705935	-11.690437	1.43E-31	8.49E-30	3220.47656	3240.26404	2014.6176
0.04881151	3.44534126	0.00057034	0.00310802	3821.88514	3656.321	4134.37518
0.06778015	-2.870818	0.00409411	0.01728765	2033.78549	1944.36593	1723.78686
0.08088747	-2.5468796	0.01086909	0.04001326	1331.82595	1344.66289	1153.14812
0.06517411	3.78191737	0.00015563	0.00097779	1872.52451	1936.06554	2202.85207
0.050197	4.90835561	9.18E-07	8.65E-06	3109.49106	3091.8946	3695.16141

0.08238869	-10.20219	1.94E-24	8.29E-23	1640.11899	1574.99866	898.777212
0.07014253	-4.2524724	2.11E-05	0.00016052	1842.16951	1853.06166	1503.33207
0.15243987	4.73985467	2.14E-06	1.91E-05	306.395855	306.076814	454.476024
0.05171977	-2.5493256	0.01079315	0.03976932	3557.22742	3420.79748	3210.16087
0.05447365	7.56526442	3.87E-14	8.43E-13	2463.49856	2537.84369	3340.73794
0.04102204	9.83826098	7.70E-23	3.08E-21	4790.39959	4664.81816	6299.07161
0.06773382	-13.109476	2.91E-39	2.25E-37	2387.61104	2422.6758	1300.68325
0.14620093	2.50673297	0.01218527	0.04405899	351.928366	373.517468	431.582643
0.10171964	5.52520449	3.29E-08	3.78E-07	638.403747	646.39273	964.065746
0.03629066	6.57747176	4.79E-11	7.85E-10	9646.25216	9893.02517	11135.5105
0.0597018	3.44289646	0.00057552	0.00313002	2340.18134	2506.71723	2821.82129
0.04826325	-4.8658751	1.14E-06	1.06E-05	4090.33723	4027.76337	3483.18565
0.11779015	-3.0402424	0.00236388	0.01083141	665.912973	650.542924	505.350206
0.05032383	-2.7353638	0.00623114	0.02474083	3775.40403	3799.50269	3523.88499
0.03609701	-3.6324853	0.0002807	0.00165482	7291.84191	7298.11631	6659.4304
0.08539431	-3.5852968	0.0003367	0.00195473	1328.03157	1448.41774	1154.84393
0.04478294	-6.1954169	5.81E-10	8.49E-09	4742.0213	4795.54927	3923.24732
0.08861555	4.10645449	4.02E-05	0.00028713	918.238971	930.681025	1214.19714
0.07803475	3.99938414	6.35E-05	0.00043585	1198.07419	1237.79539	1524.52965
0.06130274	3.34587002	0.00082025	0.00430153	2846.73053	2919.66154	3132.15379
0.08053435	-5.4131239	6.19E-08	6.91E-07	1563.28288	1478.50665	1109.05716
0.10397778	3.3632866	0.0007702	0.00406574	707.651108	662.993506	892.841891
0.06903813	-3.7110762	0.00020638	0.00126288	1891.49639	1884.18812	1569.46851
0.09341783	-3.4964024	0.00047158	0.00263807	1072.85979	1054.1493	870.796412
0.06991945	5.2004468	1.99E-07	2.06E-06	1669.5254	1581.22395	2033.27147
0.04646746	-4.5766914	4.72E-06	3.99E-05	4272.46728	4305.82637	3698.55302
0.08687686	-4.0935468	4.25E-05	0.00030255	1246.45249	1261.659	1008.1567
0.03303596	-6.6686469	2.58E-11	4.38E-10	12678.9071	12010.6617	10648.8142
0.04385102	-2.4860831	0.01291578	0.04630292	4731.58676	4708.3952	4350.59045
0.10114156	3.11744183	0.00182428	0.00863773	705.75392	716.94603	870.796412
0.06988855	-2.5725965	0.01009388	0.0375464	1879.16467	1905.97664	1622.0385
0.12346263	2.76451923	0.00570067	0.02296202	470.502613	480.384966	619.817115
0.18805097	9.27846565	1.72E-20	5.96E-19	129.008781	109.980143	391.7312
0.20977669	5.88556799	3.97E-09	5.17E-08	131.854563	127.618468	266.241552
0.06807664	8.3314183	7.99E-17	2.20E-15	1522.49333	1572.92356	2231.68078
0.03603953	-25.458597	5.67E-143	3.73E-140	10414.6133	10292.4814	5382.48844
0.06544071	-3.2990005	0.0009703	0.00497937	2155.20552	2185.07719	1912.86924
0.01943858	-5.1731503	2.30E-07	2.35E-06	39423.5657	39400.9052	36621.7798
0.13904075	3.54168639	0.00039758	0.00227207	403.152441	400.49373	512.981333
0.04715559	-6.4292871	1.28E-10	2.01E-09	4956.40354	5138.97784	3978.36102
0.02041153	-16.434025	1.09E-60	1.65E-58	39315.426	39283.6622	30882.3242
0.18656867	3.61363647	0.00030193	0.00176876	174.541292	189.87138	296.766061
0.04460648	-5.2733708	1.34E-07	1.42E-06	5225.80422	5279.04689	4352.28626

0.03215332	-9.2287194	2.74E-20	9.41E-19	10361.492	10420.0998	8388.30468
0.05803065	-6.8700773	6.42E-12	1.15E-10	2967.20196	3133.39654	2303.75253
0.07035894	-5.9450057	2.76E-09	3.69E-08	1942.72047	1901.82644	1439.73935
0.15057611	-3.5181531	0.00043456	0.00245271	422.12432	492.835549	320.507346
0.13251086	3.81137865	0.00013819	0.00088176	375.643215	392.193342	537.570521
0.11440881	-4.1508645	3.31E-05	0.00024101	807.253475	729.396612	561.311806
0.1809065	2.50609839	0.01220716	0.04410902	217.228021	206.472156	277.264291
0.04452965	-3.3508386	0.00080567	0.00423455	4991.50151	4804.88721	4436.22866
0.04802369	-11.06202	1.92E-28	9.84E-27	4626.29283	4714.62049	3175.39685
0.06171704	5.58961165	2.28E-08	2.68E-07	2068.88347	2229.69178	2742.1184
0.0901988	2.83384326	0.00459919	0.01907235	911.598813	931.718574	1082.77217
0.0357446	-4.110529	3.95E-05	0.00028273	7560.294	7503.55092	6795.94279
0.10174338	-3.9325364	8.41E-05	0.00055957	928.673504	895.404376	706.303224
0.03749895	2.47612044	0.01328188	0.04723403	8440.58922	8660.41752	8837.69329
0.02631928	-6.6686347	2.58E-11	4.38E-10	16127.9948	16083.0397	14210.8548
0.05482558	4.80593037	1.54E-06	1.41E-05	2598.19891	2620.84757	3177.09266
0.11371706	-3.0659997	0.00216944	0.01004617	717.137047	745.997388	551.13697
0.03898774	5.02343688	5.08E-07	4.99E-06	5471.49007	5533.24627	6299.91952
0.0667368	14.0643467	6.29E-45	5.62E-43	1321.39141	1334.2874	2578.47312
0.3727292	3.47814116	0.0005049	0.00280161	46.4811049	33.2015527	79.7028849
0.07436232	-3.6469812	0.00026534	0.00157157	1621.14711	1647.62705	1342.2305
0.48913481	-5.4404074	5.32E-08	5.96E-07	73.9903303	87.1540759	8.4790303
0.01928302	-3.7478401	0.00017836	0.00110586	42972.2558	43031.2875	40616.251
0.02080644	-19.092627	2.91E-81	6.46E-79	50395.0037	48914.1876	37557.0168
0.03345908	-3.0510544	0.00228039	0.01050374	9002.15685	8932.25524	8440.02677
0.05202121	-16.382117	2.57E-60	3.80E-58	4345.50901	4319.3145	2348.69139
0.1601203	-6.6236753	3.50E-11	5.84E-10	437.301824	460.671544	223.8464
0.17759431	-3.6735449	0.00023921	0.00143805	309.241637	341.353464	214.519467
0.02856557	-5.3997173	6.67E-08	7.42E-07	15902.2294	16265.6482	14210.8548
0.14669363	3.93656532	8.27E-05	0.00055093	331.059298	304.001717	495.17537
0.04594327	5.71870697	1.07E-08	1.32E-07	4244.00946	4501.92304	5177.2959
0.04767303	-2.6213705	0.0087577	0.03326146	4244.00946	4083.79099	3841.00073
0.05313855	-4.6864446	2.78E-06	2.44E-05	3300.15845	3267.2403	2786.20936
0.04862469	-5.2558369	1.47E-07	1.55E-06	4357.84073	4401.28084	3767.23316
0.08386327	4.85938208	1.18E-06	1.09E-05	1123.13527	1031.32323	1413.45435
0.06911999	4.89134789	1.00E-06	9.40E-06	1774.81933	1924.65251	2255.42206
0.11364407	-3.5330342	0.00041082	0.00233879	791.127378	719.021127	579.965673
0.19847875	-4.5214762	6.14E-06	5.11E-05	293.115539	264.574873	155.166255
0.06597985	-2.7492066	0.00597397	0.02387554	2016.7108	2015.95678	1774.66104
0.03273407	-6.650091	2.93E-11	4.93E-10	10680.2196	10424.25	8959.79132
0.04147248	-11.989898	4.01E-33	2.57E-31	6055.82396	6097.67267	4281.0624
0.0195885	-3.1272653	0.00176441	0.00839294	46726.7908	45920.8601	44102.8282
0.12579188	8.13513001	4.11E-16	1.06E-14	371.848839	340.315916	728.348703

0.03172179	-9.5897951	8.83E-22	3.33E-20	11526.3654	11229.3877	9153.96112
0.06352478	-2.4885941	0.01282493	0.04603749	2503.33951	2354.1976	2226.59336
0.10759844	2.54481948	0.01093342	0.04021405	707.651108	706.570544	800.420461
0.21614717	2.73309975	0.00627413	0.02487548	184.975826	145.256793	219.606885
0.13104405	-4.5676951	4.93E-06	4.16E-05	568.207793	592.440207	393.427006
0.13784286	-3.2431994	0.00118195	0.00591517	511.292154	492.835549	354.423467
0.02156381	-12.762264	2.66E-37	1.91E-35	32076.7054	31529.0245	26297.7125
0.156783	4.6872006	2.77E-06	2.43E-05	242.840058	267.687519	431.582643
0.0537184	-3.1462532	0.00165377	0.00792863	3155.97216	3275.54069	2870.99966
0.0964642	9.43052426	4.08E-21	1.45E-19	632.712183	655.730667	1243.87375
0.04596351	8.38286063	5.17E-17	1.45E-15	4949.76338	5399.40251	6634.84121
0.02743517	-15.740229	8.02E-56	9.98E-54	19729.8061	19866.9791	14962.0969
0.07712688	-4.0286361	5.61E-05	0.00038827	1520.59615	1555.28524	1248.11326
0.04195005	-5.5015533	3.76E-08	4.30E-07	7610.56949	7079.19357	6189.69212
0.04826902	-5.4538356	4.93E-08	5.55E-07	4246.85524	4119.06764	3514.55806
0.07024226	-9.0526271	1.40E-19	4.55E-18	2657.01173	2910.32361	1703.43719
0.05936247	3.52328317	0.00042624	0.00241568	2241.52757	2289.86959	2659.0239
0.06921723	-6.1574666	7.39E-10	1.06E-08	2063.1919	1988.98052	1515.20272
0.11151019	5.13233858	2.86E-07	2.90E-06	708.599702	775.048747	991.198643
0.07082274	9.7817561	1.35E-22	5.30E-21	1353.64361	1468.13116	2314.77527
0.06308831	5.29860729	1.17E-07	1.25E-06	2184.61193	2032.55756	2604.75811
0.14177225	4.88224405	1.05E-06	9.82E-06	328.213516	311.264557	536.722618
0.11541814	3.86265867	0.00011216	0.00072665	528.366846	563.388848	713.086449
0.06928175	5.6303125	1.80E-08	2.14E-07	1525.33912	1606.12511	2077.36242
0.08458284	4.93630376	7.96E-07	7.56E-06	996.023677	1009.53471	1369.36339
0.11014362	5.65030395	1.60E-08	1.92E-07	537.852786	541.600329	808.051588
0.10756847	5.45954293	4.77E-08	5.39E-07	592.871236	583.10227	917.431079
0.06934991	3.39827967	0.00067811	0.0036374	1787.15105	1656.96499	2010.37809
0.10187499	2.80616947	0.00501343	0.02054079	737.057521	700.345253	889.450279
0.02817101	-3.3317466	0.00086303	0.00449289	13756.5099	13897.9625	12837.2519
0.13757593	-4.6867338	2.78E-06	2.43E-05	522.675282	557.163557	334.073794
0.15179915	-8.9674382	3.03E-19	9.70E-18	606.151552	532.262392	217.063176
0.02495105	-6.1186154	9.44E-10	1.34E-08	24876.8771	25299.5832	22958.6704
0.0277457	-4.784305	1.72E-06	1.55E-05	19465.1484	19496.5743	18184.1284
0.02977787	-6.1486812	7.81E-10	1.12E-08	12479.7024	12334.3768	11033.7621
0.06480709	4.74371829	2.10E-06	1.87E-05	1833.63216	1774.20797	2214.72272
0.06162149	7.48381416	7.22E-14	1.55E-12	2147.61677	2064.72156	2804.86322
0.06115958	11.0326824	2.66E-28	1.35E-26	1847.86107	1795.99649	2853.1937
0.03638075	-5.7652399	8.15E-09	1.02E-07	7598.23776	7796.1396	6659.4304
0.08168329	4.9893318	6.06E-07	5.88E-06	1201.86857	1124.7026	1493.15724
0.06041428	-3.085687	0.00203082	0.00949529	2511.87685	2441.35168	2163.00063
0.07220842	-5.161244	2.45E-07	2.50E-06	1850.70685	1817.78501	1445.67467
0.11262295	6.61954565	3.60E-11	5.99E-10	479.988553	488.685354	819.074327

0.04087739	-12.779715	2.13E-37	1.55E-35	6353.68246	6433.83839	4483.71123
0.01717625	-13.262905	3.80E-40	3.02E-38	104853.784	102674.764	88908.5681
0.01719822	-9.4484614	3.44E-21	1.23E-19	83186.9489	83004.9194	74891.8831
0.04879028	-2.8023629	0.00507298	0.02071813	3856.03452	3877.31883	3466.22759
0.02784391	-3.4525269	0.00055536	0.00304661	13966.1491	14105.4722	13227.2873
0.04429021	7.20706937	5.72E-13	1.13E-11	33638.0911	33546.0189	40612.8594
0.0229461	-9.8384286	7.69E-23	3.08E-21	30769.5429	30270.4782	26438.4644
0.05257773	4.39359399	1.11E-05	8.88E-05	2985.22525	2889.57264	3410.26599
0.02401087	-4.064922	4.80E-05	0.00033737	20267.6589	20289.2614	18865.8424
0.02421128	-8.6731287	4.20E-18	1.24E-16	22604.9945	22564.6053	19304.2083
0.06621173	16.1040976	2.39E-58	3.35E-56	1320.44282	1282.40997	2685.3089
0.09839592	3.20425469	0.00135413	0.0066528	740.851897	754.297776	934.38914
0.05342206	13.3549262	1.11E-40	8.93E-39	2272.83117	2278.45656	3735.86075
0.03288903	-5.6245066	1.86E-08	2.21E-07	9467.91649	9335.86161	8292.49164
0.03133562	-9.5741481	1.03E-21	3.83E-20	15386.1943	16244.8972	12716.0017
0.03364229	-12.273041	1.26E-34	8.48E-33	16634.544	15726.123	11870.6424
0.2203545	-11.793123	4.24E-32	2.57E-30	387.974937	396.343536	54.265794
0.13181494	-3.2929736	0.00099134	0.00506931	553.030289	539.525232	418.864097
0.5457183	-4.7629245	1.91E-06	1.71E-05	53.1212628	68.4782025	8.4790303
0.08563757	-23.228649	2.34E-119	9.61E-117	2161.84568	2221.39139	578.269867
0.12594258	-15.99351	1.42E-57	1.91E-55	972.308827	930.681025	234.869139
0.18873318	-5.942128	2.81E-09	3.76E-08	313.036013	341.353464	146.687224
0.21660663	-9.9588635	2.31E-23	9.38E-22	320.624765	352.766498	78.0070788
0.12106725	-4.6423621	3.44E-06	2.97E-05	660.221409	664.031055	442.605382
0.08044579	3.11542346	0.00183681	0.00869205	1317.59704	1214.96932	1538.944
0.03434164	-5.3407545	9.26E-08	1.01E-06	9447.99602	9332.74897	8419.67709
0.1672649	2.45811434	0.01396687	0.04934961	247.583028	244.861451	328.138473
0.0707567	3.08547499	0.00203227	0.00949666	1558.53991	1515.85839	1799.25023
0.02328867	-26.225298	1.37E-151	9.77E-149	35762.9416	36927.3895	23613.2515
0.05596826	12.2012834	3.06E-34	2.03E-32	2102.08426	2158.10093	3392.46003
0.08330049	2.52776367	0.01147916	0.04182803	1111.75214	1081.12556	1273.55035
0.05248886	4.79046398	1.66E-06	1.51E-05	2815.42693	2823.16953	3333.10681
0.0780802	5.95041685	2.67E-09	3.58E-08	1154.43887	1144.41602	1560.14158
0.08751266	14.8265641	9.87E-50	1.01E-47	654.529845	654.693118	1629.66962
0.0224566	-15.331172	4.73E-53	5.40E-51	28679.7903	29153.0384	22667.8396
0.03523748	-3.4929838	0.00047766	0.00266935	7834.43766	7762.93805	7123.23336
0.03705792	15.5935121	8.06E-55	9.59E-53	5344.37847	5330.92431	7983.85494
0.37816611	2.95572094	0.00311939	0.01373301	36.0465712	38.3892954	87.3340121
0.05235285	2.89428712	0.0038002	0.01623015	3081.98183	3061.80569	3479.79404
0.05828732	4.20556863	2.60E-05	0.00019405	2403.73714	2319.9585	2840.47515
0.03347789	4.67993033	2.87E-06	2.51E-05	7916.96534	7997.42402	8836.84538
0.06028411	-6.1238331	9.14E-10	1.30E-08	2741.4366	2664.42461	2057.01275
0.03551623	-6.2411487	4.34E-10	6.46E-09	8014.67052	8160.31913	6890.06003

0.02925824	-11.836701	2.52E-32	1.55E-30	13213.9141	13056.5106	10340.1775
0.04235	-2.739323	0.00615659	0.02448032	5586.26994	5440.90446	5006.01949
0.08304373	3.38089591	0.0007225	0.00384786	1073.80838	1141.30338	1343.0784
0.0467082	4.12805656	3.66E-05	0.00026409	4318.94838	4628.50396	5066.22061
0.0929658	3.29993625	0.00096707	0.00496529	848.043016	847.677143	1031.05009
0.22744737	3.35662897	0.00078899	0.00415617	113.831277	119.31808	198.409309
0.20165731	-9.0408507	1.55E-19	5.03E-18	342.442426	421.2447	112.771103
0.08019916	6.57863525	4.75E-11	7.80E-10	1162.02762	1100.83898	1582.18705
0.03293899	-7.817783	5.38E-15	1.26E-13	9818.89627	9810.02129	8120.36732
0.05420274	-5.8952058	3.74E-09	4.91E-08	3735.56309	3917.78322	2967.66061
0.04286049	-3.3188187	0.00090399	0.00467798	5143.27655	5268.6714	4671.09779
0.06931131	3.98555114	6.73E-05	0.00046012	1698.93181	1674.60332	1973.07035
0.04872087	7.74412411	9.62E-15	2.21E-13	3651.13822	3670.84668	4605.80926
0.02543527	-28.975219	1.35E-184	1.48E-181	23394.2247	22909.0714	13826.7547
0.02108649	7.22681721	4.94E-13	9.87E-12	42976.9988	44007.6206	47720.8305
0.11524977	-5.7712884	7.87E-09	9.89E-08	790.178784	781.274038	473.129891
0.06124543	3.75425477	0.00017386	0.00107955	2158.0513	2308.54546	2625.95569
0.09034646	-5.592324	2.24E-08	2.64E-07	1199.02279	1257.50881	842.815612
0.09510086	-6.1516758	7.67E-10	1.10E-08	1120.28949	1084.23821	745.306764
0.05580339	-5.8033358	6.50E-09	8.25E-08	3633.11494	3369.9576	2809.10274
0.04180598	8.16914866	3.11E-16	8.09E-15	4528.58765	4469.75904	5670.77547
0.0421542	-7.0668312	1.59E-12	3.02E-11	5595.75588	5492.78188	4498.12558
0.0546232	-6.2007102	5.62E-10	8.23E-09	3494.62021	3325.34302	2722.61663
0.06109349	6.27717465	3.45E-10	5.20E-09	1933.23453	1958.89161	2536.07796
0.05854235	-2.6868506	0.00721292	0.02810917	2608.63344	2575.19543	2305.44834
0.02856735	-2.9381198	0.00330209	0.01441376	15388.0915	15698.1092	14912.0706
0.05905669	9.46720733	2.87E-21	1.04E-19	1963.58953	1947.47858	2889.65353
0.05284864	3.11820353	0.00181957	0.00862538	2949.17868	2892.68528	3238.14167
0.03247844	7.13033276	1.00E-12	1.94E-11	8860.81635	9034.97254	10382.5726
0.04028908	-7.6313472	2.32E-14	5.19E-13	6326.17324	6543.81854	5186.62284
0.06588182	-2.8745135	0.00404651	0.01711302	2063.1919	2071.9844	1783.14007
0.05832245	-5.3112559	1.09E-07	1.17E-06	2845.78193	2805.53121	2322.4064
0.03133413	2.88943029	0.00385941	0.01643173	9493.52853	9614.96216	10174.8364
0.05814347	8.40756473	4.19E-17	1.18E-15	2348.71869	2386.3616	3205.92136
0.08523916	2.6848911	0.00725534	0.0282678	1035.86462	1096.68879	1238.78633
0.10220804	2.48424595	0.01298261	0.04648165	725.674393	711.758287	836.880291
0.0474772	-4.2298095	2.34E-05	0.00017579	4477.36358	4371.19193	3770.62478
0.11750049	-6.1910082	5.98E-10	8.70E-09	821.482385	791.649523	456.17183
0.15571823	4.89659971	9.75E-07	9.17E-06	256.120374	266.64997	418.016194
0.03780115	-6.9717229	3.13E-12	5.77E-11	6880.15212	6919.4111	5728.43287
0.0260166	-5.3108456	1.09E-07	1.18E-06	23529.8736	23871.9164	21984.4298
0.07870109	5.0593017	4.21E-07	4.19E-06	1156.33606	1210.81913	1534.70449
0.04831138	-3.4505466	0.00055945	0.00306122	4022.98706	3925.04606	3561.19273

0.38063318	-5.1965331	2.03E-07	2.09E-06	93.9108038	112.055241	27.132897
0.06273577	-8.0039259	1.21E-15	3.01E-14	2584.91859	2527.4682	1831.47055
0.0388676	10.5662123	4.27E-26	1.96E-24	6372.65434	6758.59108	8847.02022
0.03651471	5.66605603	1.46E-08	1.76E-07	6414.39248	6338.38393	7374.21266
0.36369442	2.54414248	0.01095464	0.04026203	42.686729	42.5394895	78.8549818
0.12866263	-2.6763056	0.00744387	0.02892004	549.235913	621.491565	467.19457
0.04365671	-3.0745416	0.00210826	0.00980435	4832.13772	4906.56697	4403.16044
0.0643059	-5.9919058	2.07E-09	2.81E-08	2397.09698	2312.69566	1823.83942
0.04766756	-10.051119	9.08E-24	3.77E-22	4745.81567	4587.00202	3377.19777
0.02213928	-32.817547	3.31E-236	9.06E-233	35512.5128	35190.5333	21152.6369
0.16250258	-4.6894496	2.74E-06	2.41E-05	451.530734	407.75657	232.32543
0.22825441	2.48138788	0.01308719	0.04676435	124.265811	124.505823	183.147055
0.10986901	-2.7460761	0.00603128	0.02405191	801.561911	882.953793	719.02177
0.11797729	-3.8737615	0.00010717	0.00069872	714.291265	670.256346	513.829236
0.0404239	12.7959056	1.73E-37	1.27E-35	4551.35391	4558.98821	6465.26061
0.03329247	8.05051159	8.24E-16	2.08E-14	7655.1534	7687.19701	9255.70948
0.07256773	5.35376196	8.61E-08	9.42E-07	1348.90064	1363.33876	1772.11733
0.04055893	4.3861429	1.15E-05	9.18E-05	5220.11266	5290.45992	6017.56781
0.25052319	3.01069461	0.00260651	0.01180247	102.44815	94.4169156	156.014158
0.02183879	-3.2362944	0.00121092	0.00603992	35392.9899	36411.7279	34410.4487
0.0894409	4.9964656	5.84E-07	5.69E-06	885.038181	918.230443	1193.84747
0.19065013	-7.3033708	2.81E-13	5.74E-12	332.007892	356.916692	130.577067
0.06670452	11.6114352	3.61E-31	2.09E-29	1432.37691	1482.65684	2538.62167
0.03660507	-23.405563	3.75E-121	1.58E-118	9142.54876	9009.03383	5016.19433
0.17879402	-6.2579198	3.90E-10	5.86E-09	356.671336	425.394895	172.972218
0.12275148	3.04707999	0.00231076	0.01061684	653.581251	587.252464	724.109188
0.06254679	5.68318914	1.32E-08	1.60E-07	2197.89225	2058.49627	2658.176
0.04380901	15.9773355	1.84E-57	2.46E-55	3518.33506	3532.85272	5697.06046
0.07687689	6.85131025	7.32E-12	1.30E-10	1155.38747	1170.35473	1646.62769
0.05275299	6.4604864	1.04E-10	1.65E-09	2764.20285	2696.58861	3468.7713
0.04456116	-8.4062966	4.23E-17	1.19E-15	5543.58321	5596.53673	4407.39995
0.06150849	5.30268938	1.14E-07	1.22E-06	1952.20641	1953.70387	2446.20024
0.03006517	-18.380793	1.87E-75	3.80E-73	13875.0841	13644.8006	9301.49624
0.24810416	3.18860063	0.00142963	0.00698242	543.544349	590.36511	813.986909
0.09338489	-5.9367768	2.91E-09	3.86E-08	1188.58825	1118.47731	789.397721
0.14800077	4.81493963	1.47E-06	1.35E-05	300.704291	296.738878	458.715539
0.0436436	-18.380954	1.87E-75	3.80E-73	6049.1838	6137.09951	3457.74856
0.06998357	-4.1233865	3.73E-05	0.00026904	1857.34701	1876.92528	1510.9632
0.06127631	-4.6401783	3.48E-06	3.00E-05	2509.97967	2461.0651	2066.33969
0.10759817	5.6802011	1.35E-08	1.63E-07	580.539515	558.201105	850.44674
0.07612097	-4.9488147	7.47E-07	7.13E-06	1658.14227	1643.47686	1240.48213
0.04509226	3.89966484	9.63E-05	0.0006323	4628.19002	4691.79442	5113.70318
0.0555152	7.90045961	2.78E-15	6.66E-14	2344.92431	2330.33398	3147.41605

0.03991116	-6.4292587	1.28E-10	2.01E-09	7949.21754	8513.08563	6826.4673
0.07240148	8.54550591	1.28E-17	3.72E-16	1266.37296	1327.02456	2015.4655
0.09579353	-3.7059015	0.00021064	0.00128655	1064.32244	1018.87265	840.271903
0.04682856	3.62813924	0.00028547	0.00168111	3725.12855	3686.4099	4155.57275
0.04670459	2.4792976	0.01316414	0.04692707	3987.88908	4010.12504	4252.2337
0.18952489	5.61676826	1.95E-08	2.31E-07	149.877849	191.946477	334.921697
0.15052326	2.55725558	0.01055017	0.03897015	312.087419	328.902882	397.666521
0.0655475	9.74805202	1.88E-22	7.34E-21	1755.84745	1914.27703	2933.74449
0.02611904	11.6046296	3.90E-31	2.24E-29	16663.0018	16211.6957	20108.8683
0.03104175	-4.6315372	3.63E-06	3.12E-05	11442.8892	11625.7312	10301.1739
1.4428581	13.602747	3.86E-42	3.25E-40	0	0	151247.247
0.07127648	7.09070121	1.33E-12	2.55E-11	1439.01706	1527.27143	2047.68582
0.0763502	9.82753887	8.57E-23	3.42E-21	1102.2662	1074.90027	1861.14715
0.05556772	6.681332	2.37E-11	4.03E-10	2551.7178	2714.22694	3371.26245
0.03867634	-9.3299269	1.06E-20	3.73E-19	6977.8573	6842.63251	5362.13876
0.2267727	5.05182514	4.38E-07	4.34E-06	117.625653	102.717304	218.758982
0.09498636	3.42193148	0.00062178	0.00335386	796.818942	848.714692	1025.11476
0.10541573	4.15258329	3.29E-05	0.00023963	627.020619	684.782025	870.796412
0.07944889	-3.3113379	0.00092851	0.00478677	1520.59615	1514.82084	1313.40179
0.07534394	7.18651889	6.65E-13	1.31E-11	1200.91998	1251.28352	1755.15927
0.03710253	-32.106418	3.59E-226	5.89E-223	9909.96129	9821.43432	4275.97498
0.31104264	6.31059925	2.78E-10	4.24E-09	33.2007892	49.8023291	155.166255
0.04730118	7.87503288	3.41E-15	8.11E-14	4097.92598	4268.47462	5247.67186
0.04353578	3.72384256	0.00019621	0.00120425	5814.88109	6317.63296	6745.06861
0.06891933	3.14142502	0.00168128	0.00803855	1876.31889	1725.44319	2114.67016
0.07005115	-3.8156087	0.00013585	0.00086746	2221.6071	2078.20969	1851.82022
0.03204779	10.6356844	2.03E-26	9.49E-25	8348.5756	8376.12923	10673.4033
0.08070419	7.41684179	1.20E-13	2.52E-12	1004.56102	1008.49716	1526.22545
0.02384751	-2.5919126	0.0095444	0.03581875	20870.0161	21181.5531	20130.9137
0.11083829	3.45909424	0.000542	0.00298424	651.684063	670.256346	799.572558
0.11799381	-2.7734574	0.00554641	0.02240657	686.78204	733.546806	532.483103
0.07779361	-4.1914401	2.77E-05	0.00020459	1679.95994	1584.33659	1266.76713
0.03995627	4.64935556	3.33E-06	2.88E-05	5476.23304	5336.11205	6182.061
0.08820295	3.57801186	0.00034622	0.00200395	951.43976	1035.47343	1229.45939
0.05157109	2.83563322	0.00457349	0.01898497	3376.99456	3615.8566	3892.72281
0.03611463	-6.7729963	1.26E-11	2.20E-10	7759.49874	7641.54487	6511.89527
0.03197657	-7.5490628	4.38E-14	9.50E-13	10431.688	10301.8193	8711.35574
0.02827429	-8.7642492	1.88E-18	5.70E-17	15785.5524	15508.2378	13353.6248
0.0584683	-5.8069219	6.36E-09	8.10E-08	3137.94888	3035.86698	2362.25784
0.04429963	-6.3502291	2.15E-10	3.31E-09	4929.8429	4893.07884	4012.27714
0.03826311	-2.9027171	0.00369941	0.01590218	6393.52341	6475.34033	5931.9296
0.04771727	-5.8265545	5.66E-09	7.24E-08	5161.29983	4851.57689	4058.91181
0.18820423	-3.248548	0.00115996	0.00581394	274.14366	310.227008	201.800921

0.12076847	4.31443223	1.60E-05	0.00012413	485.680117	473.122127	656.276946
0.03986973	17.3351476	2.55E-67	4.46E-65	4567.48	4463.53375	7353.01508
0.23877524	-4.1975497	2.70E-05	0.00019994	218.176615	183.646089	100.900461
0.03980744	-2.8996775	0.00373547	0.01600722	6065.3099	5986.65498	5623.2929
0.04421167	6.21842935	5.02E-10	7.42E-09	4039.11316	4039.1764	4910.20645
0.10395658	2.82294361	0.00475849	0.01960924	862.271926	830.038819	950.499297
0.06714526	6.89837164	5.26E-12	9.50E-11	1894.34217	1924.65251	2497.92233
0.12694524	-2.9894537	0.00279477	0.01254591	561.567635	571.689236	438.365867
0.045644	-5.7052822	1.16E-08	1.42E-07	4520.9989	4562.10086	3802.84509
0.0327275	7.79838936	6.27E-15	1.46E-13	8266.04792	8308.68857	9983.21028
0.05085917	-7.3923375	1.44E-13	3.02E-12	4502.02702	4280.92521	3469.6192
0.10366535	2.69469413	0.00704533	0.02753443	688.679228	678.556734	818.226424
0.03304185	-4.2008229	2.66E-05	0.00019736	9232.66519	9127.31436	8384.91307
0.03330024	3.32553226	0.0008825	0.00458119	8832.35853	8673.90565	9337.95607
0.04498573	-7.5663568	3.84E-14	8.38E-13	4902.33368	4820.45044	3823.19476
0.06279507	2.61320363	0.00896978	0.03397359	2258.60226	2462.10265	2596.27908
0.04628114	4.76800057	1.86E-06	1.67E-05	3851.29155	3874.20619	4569.34943
0.03800265	-4.6779918	2.90E-06	2.53E-05	6921.89026	7003.45253	6070.9857
0.09324617	4.65650132	3.22E-06	2.79E-05	983.691955	866.353017	1279.48567
0.03150814	-5.3409219	9.25E-08	1.00E-06	10894.6018	11153.6466	9890.78885
0.06087509	-11.173852	5.48E-29	2.91E-27	2856.21647	2807.6063	1778.05265
0.04101151	-4.7602777	1.93E-06	1.73E-05	5674.48918	5685.76591	4917.83758
0.1421546	4.0313428	5.55E-05	0.00038415	365.208682	333.053076	489.240049
0.05785612	-2.5165959	0.01184946	0.04302468	2735.74503	2785.81778	2548.79651
0.04758971	3.97077034	7.16E-05	0.00048719	3688.13339	3802.61534	4323.45755
0.05211138	-4.2535403	2.10E-05	0.00015991	3396.91504	3485.12549	2971.05222
0.04723144	-4.708507	2.50E-06	2.21E-05	4262.03274	4396.09309	3737.55656
0.04309462	-3.6500215	0.00026222	0.00155589	5731.40482	5695.10384	5262.08621
0.29478497	-2.6659389	0.00767736	0.02970775	111.934089	129.693565	67.8322424
0.01930896	-13.555177	7.38E-42	6.13E-40	159580.068	155361.478	129294.189
0.02730955	-13.000732	1.21E-38	9.22E-37	15631.8802	15614.0677	12275.9401
0.07088485	6.18270642	6.30E-10	9.13E-09	1600.27804	1649.70215	2299.51302
0.03023873	-4.6527385	3.28E-06	2.84E-05	12695.0332	12513.8727	11605.2488
0.21000831	3.74700594	0.00017896	0.00110912	152.72363	128.656017	245.891879
0.07149371	-28.627895	3.02E-180	3.10E-177	3199.60749	3061.80569	763.96063
0.05725254	2.76083181	0.00576544	0.02317742	2862.85663	3126.1337	3391.61212
0.04909411	10.4708897	1.18E-25	5.26E-24	3139.84607	3320.15527	4607.50507
0.06585722	-4.1162016	3.85E-05	0.00027683	2243.42476	2251.4803	1922.19617
0.07025356	-14.301136	2.15E-46	2.04E-44	2477.72747	2365.61063	1187.06424
0.03563883	-4.9958674	5.86E-07	5.70E-06	7942.57738	7936.20865	7099.49207
0.05405986	4.98893176	6.07E-07	5.89E-06	2683.57236	2784.78024	3325.47569
0.04691981	3.67514487	0.00023771	0.00143012	3825.67951	3793.2774	4357.37367
0.05387373	15.534954	2.01E-54	2.36E-52	2140.02801	2196.49022	3855.41508

0.09022809	-7.366681	1.75E-13	3.63E-12	1436.17128	1382.01463	838.576097
0.09412781	11.7964741	4.07E-32	2.48E-30	659.272815	672.331443	1507.57159
0.04205027	4.10905644	3.97E-05	0.00028441	4668.97956	4720.84578	5310.41668
0.05767891	8.94923062	3.58E-19	1.14E-17	2086.90675	2109.33615	2980.37915
0.13448519	6.1562842	7.45E-10	1.07E-08	342.442426	324.752688	576.574061
0.02048442	-6.1883119	6.08E-10	8.84E-09	36370.0417	35850.4141	33219.9928
0.0629432	-3.9262443	8.63E-05	0.00057231	2467.29294	2344.85966	2010.37809
0.06074103	-6.1587113	7.33E-10	1.05E-08	2657.96033	2619.81002	1998.50744
0.02371745	-8.9701773	2.96E-19	9.52E-18	31990.3833	32049.8739	28172.4261
0.03670248	10.0480363	9.37E-24	3.87E-22	5953.37581	6007.40595	7673.52243
0.06211666	4.98122962	6.32E-07	6.11E-06	2019.55658	1955.77897	2432.63379
0.14457512	-10.634941	2.05E-26	9.54E-25	621.329056	677.519186	214.519467
0.04031752	-3.2388175	0.00120026	0.00599038	5995.11394	6220.1034	5576.65823
0.04653948	-3.2151878	0.00130359	0.00644151	4326.53713	4363.92909	3977.51312
0.04515839	7.82715711	4.99E-15	1.18E-13	39133.296	40695.7657	52258.8075
0.05143967	3.06482338	0.00217799	0.01008009	3133.20591	3236.11384	3596.80466
0.05997513	-11.880971	1.49E-32	9.25E-31	3038.34651	2952.8631	1843.34119
0.10515645	-5.0804187	3.77E-07	3.77E-06	879.346618	871.540759	613.033891
0.0971538	-2.6036004	0.00922502	0.03479503	946.69679	983.596	787.701915
0.10765174	4.52093261	6.16E-06	5.12E-05	586.231078	587.252464	834.336582
0.03831087	-8.3976509	4.55E-17	1.28E-15	7005.36653	7188.13617	5698.75627
0.03382788	-3.5862445	0.00033547	0.00194834	9024.92311	8955.0813	8156.82715
0.02545293	3.23773407	0.00120483	0.00601135	16598.4974	16871.5765	17747.4583
0.1049323	3.69050158	0.00022381	0.00135691	662.118597	630.829502	840.271903
0.07145011	2.97123192	0.00296608	0.01314966	1531.03068	1516.89594	1733.11379
0.04168511	8.73882356	2.36E-18	7.10E-17	4674.67112	4867.14012	6132.88262
0.08628804	-6.9660787	3.26E-12	5.99E-11	1401.07331	1523.12123	942.020267
0.03438009	-6.295764	3.06E-10	4.64E-09	8624.61645	8597.12706	7475.96102
0.02268034	-5.1421443	2.72E-07	2.76E-06	26820.5461	27403.7316	25103.0171
0.09383166	2.6133951	0.00896476	0.03396933	843.300046	860.127726	993.742352
0.0945002	-2.673128	0.00751476	0.02915406	982.743361	1021.9853	832.640776
0.11711991	4.19123427	2.77E-05	0.00020469	501.806214	506.323679	731.740315
0.03029835	21.2898224	1.41E-100	4.21E-98	9605.46262	9304.73516	14895.9604
0.08392666	-2.8528002	0.00433359	0.01812648	1327.08298	1373.71424	1187.06424
0.01988542	-11.34508	7.85E-30	4.30E-28	72748.6208	73631.706	63635.1224
0.03985058	6.49658724	8.22E-11	1.32E-09	5831.95578	5793.67095	6793.39908
0.03006095	4.75407186	1.99E-06	1.79E-05	12113.5451	12544.9992	13747.8997
0.05703118	8.47782816	2.29E-17	6.57E-16	2191.25209	2256.66804	3139.78492
0.04914999	-3.7854895	0.00015341	0.00096607	4037.21597	3892.88206	3456.90066
0.30047008	-2.8328657	0.00461328	0.01910865	110.036901	126.58092	63.5927273
0.08350765	7.15728303	8.23E-13	1.61E-11	1023.5329	1004.34697	1474.50337
0.01998748	-4.9923113	5.97E-07	5.79E-06	40818.9475	41309.9945	38615.1998
0.06000121	-3.7558458	0.00017276	0.00107434	2538.43749	2581.42073	2222.35384

0.09034126	-2.7418213	0.00610996	0.02433615	1216.09748	1127.81524	1014.93993
0.03604234	-4.1386496	3.49E-05	0.00025304	7961.54926	8251.6234	7275.8559
0.02277073	-3.3081898	0.00093901	0.00483484	30452.7125	29521.3681	28476.8233
0.04086684	-5.2434687	1.58E-07	1.66E-06	5734.2506	5733.49314	4978.03869
0.03101536	-9.4520998	3.32E-21	1.19E-19	11227.5583	11415.1089	9252.31787
0.04043983	-4.9948204	5.89E-07	5.73E-06	6262.61744	6113.2359	5450.32068
0.0330116	6.49748325	8.17E-11	1.31E-09	8512.68236	8388.57981	9911.13852
0.07426267	4.79519807	1.63E-06	1.48E-05	1516.80177	1645.55196	1941.69794
0.19972901	-15.645314	3.58E-55	4.35E-53	556.824665	513.586519	61.0490182
0.10312244	-24.089402	3.23E-128	1.52E-125	1733.0812	1641.40176	292.526546
0.0375804	-19.891379	4.83E-88	1.26E-85	8174.9829	8133.34287	4850.00533
0.24382576	-9.8196241	9.27E-23	3.67E-21	277.938036	287.400941	50.0262788
0.07299842	4.4585946	8.25E-06	6.72E-05	1668.57681	1650.7397	1967.13503
0.04237121	-5.2853626	1.25E-07	1.34E-06	6086.17896	5790.55831	5100.13673
0.05037422	11.8782551	1.54E-32	9.52E-31	2792.66067	2775.4423	4151.33324
0.04715366	-4.0703446	4.69E-05	0.00033046	4444.16279	4676.23119	3969.03409
0.02912211	-13.81242	2.15E-43	1.85E-41	14094.2093	13819.1088	10554.6969
0.09259488	-3.6935362	0.00022116	0.0013423	1121.23808	1059.33704	864.013188
0.04327489	5.19598381	2.04E-07	2.10E-06	4456.49451	4487.39736	5300.24184
0.0443501	3.0229104	0.00250356	0.01139434	4387.24715	4557.95066	4928.01241
0.0534782	-10.63831	1.98E-26	9.28E-25	3745.04903	3620.0068	2489.4433
0.03294247	-4.9147149	8.89E-07	8.40E-06	10948.6717	11053.0044	10047.6509
0.03083821	-7.6940675	1.43E-14	3.23E-13	11393.5623	11196.1861	9584.69586
0.0213148	16.0935152	2.83E-58	3.94E-56	29259.3813	28598.9875	36587.8637
0.22559286	5.80213992	6.55E-09	8.31E-08	89.1678339	116.205435	256.914618
0.06245613	-12.254208	1.60E-34	1.07E-32	2897.00601	2787.89288	1690.71864
0.02498235	-10.650895	1.73E-26	8.13E-25	19560.0078	19398.0072	16275.4987
0.08766132	-3.3632383	0.00077034	0.00406574	1372.61549	1406.9158	1059.87879
0.04138914	-2.5878058	0.00965894	0.036166	5597.65306	5463.73052	5098.44092
0.09150386	5.31890137	1.04E-07	1.13E-06	881.243806	816.550688	1188.76005
0.05602661	-12.90356	4.30E-38	3.25E-36	3382.68613	3397.97141	2047.68582
0.04839588	-15.477115	4.95E-54	5.73E-52	5222.95844	5319.51128	3226.27103
0.04237799	6.25324463	4.02E-10	6.03E-09	4497.28405	4589.07712	5491.86793
0.04146457	5.93312789	2.97E-09	3.95E-08	4943.12322	4919.01755	5757.26158
0.03717047	7.47611371	7.66E-14	1.64E-12	6298.66401	6249.15476	7498.8544
0.05682771	-4.566566	4.96E-06	4.18E-05	2832.50162	2841.84541	2357.17042
0.03099666	-6.6211763	3.56E-11	5.93E-10	10933.4942	11028.1033	9588.93537
0.03661325	-5.8737256	4.26E-09	5.52E-08	7355.39771	7295.00367	6334.68354
0.03928968	4.50673751	6.58E-06	5.43E-05	5511.33101	5635.96358	6275.33033
0.11248829	3.15605187	0.0015992	0.00770526	569.156387	555.08846	713.934352
0.18940363	4.86264637	1.16E-06	1.08E-05	166.95254	154.59473	295.070255
0.11842115	3.09155377	0.00199112	0.00933888	496.11465	527.07465	654.58114
0.03536978	-3.4821305	0.00049744	0.002763	7710.17185	7665.40849	7091.01304

0.04135255	-3.7819675	0.00015559	0.00097779	5796.8578	5618.32525	5142.53188
0.10328576	-6.0752575	1.24E-09	1.73E-08	1016.89274	965.957675	668.147588
0.06882353	-3.2900942	0.00100154	0.0051151	1888.65061	1904.93909	1641.54027
0.08738942	-7.5819757	3.40E-14	7.50E-13	1353.64361	1327.02456	860.621576
0.03401845	-16.496829	3.87E-61	5.94E-59	9883.40066	9897.17536	6641.62444
0.02324374	-22.077499	5.20E-108	1.90E-105	26542.6081	26240.6397	18407.9748
0.09242283	-2.9895539	0.00279385	0.01254522	1064.32244	1045.84891	886.90657
0.0208875	-23.579286	6.29E-123	2.79E-120	57128.1237	58288.4385	40423.777
0.04063117	8.09385404	5.78E-16	1.47E-14	5391.80817	5281.12198	6839.18584
0.06176397	-7.0001664	2.56E-12	4.75E-11	2572.58687	2602.1717	1951.87278
0.03758552	-5.5743163	2.49E-08	2.91E-07	7005.36653	6954.68775	6087.94376
0.07203846	3.31113923	0.00092917	0.00478866	1531.97927	1540.75956	1866.23457
0.0363982	3.9263227	8.63E-05	0.00057231	6925.68463	6947.42491	7771.87918
0.05836353	-16.154487	1.06E-58	1.50E-56	3326.71908	3347.13154	1725.48267
0.07703359	2.5054169	0.01223071	0.04418439	1532.92787	1562.54808	1669.52107
0.05130962	-9.0712721	1.18E-19	3.87E-18	3821.88514	3764.22604	2736.18308
0.02739212	6.41603354	1.40E-10	2.19E-09	17932.2206	18764.065	20564.1922
0.09779619	-4.7164841	2.40E-06	2.13E-05	984.640549	1036.51097	724.957091
0.08517026	-2.5808569	0.00985554	0.03681823	1350.79782	1494.06987	1263.37552
0.02848956	-4.164567	3.12E-05	0.0002283	15127.2282	14904.3845	14045.5137
0.04176156	-4.1454433	3.39E-05	0.00024635	5425.00896	5460.61788	4873.74662
0.11827463	4.54688619	5.44E-06	4.57E-05	476.194177	513.586519	738.52354
0.05179692	8.35886685	6.33E-17	1.76E-15	3061.11277	2885.42244	4027.53939
0.06639159	4.99051129	6.02E-07	5.84E-06	1674.26837	1742.04397	2160.45692
0.12754417	4.16202171	3.15E-05	0.00023065	407.895411	416.056958	580.813576
0.04606842	-4.6635244	3.11E-06	2.70E-05	4850.16101	4881.6658	4300.56417
0.04435595	2.68343997	0.00728691	0.0283694	5569.19524	5521.83324	5798.80883
0.03522005	-5.4292219	5.66E-08	6.34E-07	8695.761	9044.31048	7786.29353
0.01719003	-13.388475	7.06E-41	5.77E-39	66574.2226	66947.8185	56922.2741
0.05075276	-2.9260526	0.00343293	0.01489779	3501.26037	3543.22821	3145.72024
0.04288101	3.67430548	0.0002385	0.0014343	4506.76999	4535.12459	5013.65062
0.06516212	3.95930734	7.52E-05	0.0005076	1810.8659	1771.09533	2133.32402
0.08501417	5.37194947	7.79E-08	8.59E-07	964.720076	1012.64736	1332.05566
0.08642003	-5.8798528	4.11E-09	5.33E-08	1411.50784	1338.43759	996.286061
0.06995613	-3.4969084	0.00047068	0.00263397	2079.318	2080.28479	1834.01425
0.09079678	-2.8572007	0.00427395	0.0179297	1087.0887	1153.75396	919.126885
0.11762361	7.81812512	5.36E-15	1.26E-13	473.348395	420.207152	822.46594
0.05425304	-2.6427644	0.00822322	0.03149361	3085.77621	3207.06249	2871.84756
0.044539	7.68241793	1.56E-14	3.51E-13	4295.23353	4305.82637	5320.59152
0.068712	3.1025474	0.00191863	0.00903723	1610.71257	1624.80099	1870.47409
0.03140944	-4.0511574	5.10E-05	0.00035587	10542.6735	10499.9911	9729.68727
0.03527528	-6.8612401	6.83E-12	1.22E-10	8208.18369	8300.38819	6915.49712
0.09105553	-7.9863153	1.39E-15	3.44E-14	1292.93359	1423.51657	821.618037

0.02456584	6.19899511	5.68E-10	8.31E-09	22918.0305	23305.4149	25268.3582
0.06008604	7.6142646	2.65E-14	5.92E-13	2042.32283	2013.88168	2732.79147
0.05463008	6.12723006	8.94E-10	1.27E-08	2727.20769	2711.11429	3337.34633
0.03871019	-2.8468148	0.00441591	0.01843326	6215.18774	6180.67655	5730.12868
0.04832579	3.50409358	0.00045816	0.00257199	4624.39564	4426.182	4909.35855
0.10986634	5.57995158	2.41E-08	2.82E-07	605.202958	539.525232	869.948509
0.05926279	7.00965998	2.39E-12	4.46E-11	2125.7991	2103.11086	2870.99966
0.06879897	2.95903941	0.003086	0.01361152	1623.0443	1645.55196	1861.14715
0.05503234	-5.6597841	1.52E-08	1.82E-07	3334.30783	3267.2403	2597.12698
0.03455109	-5.5330284	3.15E-08	3.63E-07	8638.84536	8489.22202	7447.98022
0.03903823	-3.6963554	0.00021872	0.00133143	6403.00935	6515.80473	5773.37173
0.09272717	-2.6614611	0.00778023	0.03002801	1035.86462	1023.02284	857.229964
0.06753406	2.57440579	0.01004124	0.03739293	1734.02979	1833.34824	2012.92179
0.0726557	-3.4581387	0.00054392	0.00299184	1774.81933	1787.69611	1451.60999
0.09469498	3.18251464	0.00146002	0.00711395	826.225355	825.888624	993.742352
0.03313815	8.34008313	7.42E-17	2.06E-15	12020.5829	12866.6392	15185.9433
0.05337698	5.23180098	1.68E-07	1.76E-06	2774.63739	2878.1596	3472.16291
0.14857818	-4.283792	1.84E-05	0.00014126	454.376515	441.995671	289.134933
0.01845771	-11.983247	4.35E-33	2.77E-31	75920.719	74426.4682	65012.9649
0.04959802	-2.7035767	0.00685976	0.02689236	4059.98223	4092.09138	3821.49896
0.05256896	-9.0646432	1.25E-19	4.09E-18	3732.7173	3701.97313	2624.25988
0.04308158	4.57202911	4.83E-06	4.08E-05	4841.62366	4984.38311	5519.00083
0.03349449	-2.8254525	0.00472139	0.01948077	10372.8751	10671.1866	9663.55084
0.04512706	2.826812	0.00470139	0.01941777	4364.48089	4336.95283	4658.37925
0.04175984	10.8024209	3.35E-27	1.62E-25	4366.37808	4354.59115	5903.9488
0.06192438	4.88511462	1.03E-06	9.69E-06	1972.12688	1949.55368	2440.26492
0.07426324	-3.5203828	0.00043092	0.00243721	1720.74948	1860.3245	1470.26385
0.14824731	3.81749776	0.00013481	0.00086118	290.269757	312.302105	449.388606
0.03330688	2.50804311	0.01214019	0.04393464	9669.96701	9546.48396	9977.27496
0.04960715	-6.0107808	1.85E-09	2.52E-08	4023.93565	4084.82854	3232.20635
0.05931718	-10.548017	5.19E-26	2.37E-24	2956.76743	3042.09227	1921.34827
0.08541217	3.36568797	0.00076353	0.00403759	1071.9112	1008.49716	1270.15874
0.02186929	-18.068592	5.63E-73	1.12E-70	31576.7963	31914.9926	23965.1313
0.11234963	4.97716377	6.45E-07	6.22E-06	514.137936	544.712975	775.831273
0.10256911	-8.2105631	2.20E-16	5.79E-15	1031.12165	1012.64736	581.661479
0.01769285	-26.303113	1.77E-152	1.32E-149	168359.305	172634.586	122335.449
0.05707654	2.92798588	0.00341166	0.01483682	2646.5772	2525.39311	2940.52771
0.02934938	-8.3819496	5.21E-17	1.45E-15	20113.9867	19825.4772	16366.2243
0.0508954	-4.2964406	1.74E-05	0.00013419	3561.02179	3559.82898	3032.10124
0.05222859	-5.8874349	3.92E-09	5.12E-08	3472.80255	3434.28561	2799.77581
0.15267691	3.54064232	0.00039915	0.00227949	323.470546	358.991789	444.301188
0.07273475	6.13312398	8.62E-10	1.23E-08	1340.36329	1312.49888	1807.72926
0.16566576	-2.6170857	0.00886841	0.03364303	366.157276	372.47992	247.587685

0.05653129	-4.0021469	6.28E-05	0.00043151	2992.814	2893.72283	2488.59539
0.02176866	7.25373901	4.05E-13	8.17E-12	24564.7897	24547.3605	27309.2608
0.04145016	-2.7641387	0.00570733	0.02298318	6092.81912	5821.68476	5541.0463
0.07696797	-3.5892132	0.00033168	0.00192901	1613.55836	1760.71984	1409.21484
0.07685011	-8.805089	1.31E-18	4.01E-17	1831.73497	1770.05778	1100.57813
0.03200034	-4.4855279	7.27E-06	5.96E-05	10919.2653	10657.6984	9878.07031
0.02124275	-3.3229736	0.00089063	0.00462049	30770.4915	31108.8174	29640.9941
0.03774732	6.95320104	3.57E-12	6.51E-11	5758.91404	5835.17289	6957.89227
0.02087527	-9.0952872	9.43E-20	3.13E-18	35817.96	35238.2605	31305.4278
0.05190123	5.55914452	2.71E-08	3.16E-07	2860.01084	2827.31973	3478.94613
0.03560735	3.33890485	0.00084109	0.00439262	7242.51502	7461.01143	7959.26575
0.02322281	-11.43408	2.83E-30	1.57E-28	25166.1982	25116.9746	21106.8501
0.08785003	-8.5382192	1.36E-17	3.94E-16	1358.38658	1348.81308	793.637237
0.0702062	-2.4644087	0.01372395	0.04861676	1814.66028	1909.08928	1673.76058
0.04120213	5.11110019	3.20E-07	3.24E-06	5238.13595	5183.59242	6142.20955
0.16382271	-4.2393948	2.24E-05	0.00016938	395.563689	369.367274	228.933818
0.04581529	-6.0491825	1.46E-09	2.02E-08	4558.94266	4506.07324	3739.25236
0.08725592	2.97036321	0.00297448	0.0131798	1010.25259	983.596	1167.56247
0.02822044	13.3715425	8.87E-41	7.21E-39	12090.7788	11796.9267	15452.1848
0.04301351	-10.277299	8.92E-25	3.88E-23	5627.05948	5781.22037	4227.64451
0.03542619	-9.7805119	1.37E-22	5.35E-21	8525.01408	8752.75934	6773.89731
0.0728769	6.24485855	4.24E-10	6.33E-09	1346.05485	1424.55412	1927.28359
0.04711636	9.5398406	1.43E-21	5.27E-20	3311.54158	3285.91617	4519.32315
0.06728274	4.86001733	1.17E-06	1.09E-05	1848.80966	2032.55756	2383.45542
0.07035742	-3.342114	0.00083143	0.00435044	2392.35401	2128.01202	1928.13149
0.07229261	4.81389077	1.48E-06	1.36E-05	1384.94721	1387.20238	1757.70298
0.06464242	-3.4539373	0.00055247	0.00303345	2175.12599	2131.12467	1835.71006
0.02375614	-4.6470897	3.37E-06	2.91E-05	23488.1355	22993.1128	21625.7668
0.05151206	-2.5947585	0.00946574	0.03558723	3389.32628	3462.29942	3153.35137
0.07573798	-4.8469296	1.25E-06	1.16E-05	1619.24992	1651.77725	1284.57309
0.3739156	4.34690842	1.38E-05	0.00010828	109.088307	158.744924	338.313309
0.03306969	-16.267275	1.68E-59	2.43E-57	10369.0808	10276.9181	7129.16868
0.08055029	-4.0396473	5.35E-05	0.00037189	1471.26926	1607.16266	1228.61149
0.0444635	-3.3464025	0.00081867	0.00429464	4649.05909	4700.09481	4247.14628
0.02511162	-9.0437484	1.51E-19	4.91E-18	22099.3939	21675.4262	18911.6292
0.04278871	-19.79917	3.03E-87	7.77E-85	6789.0871	7094.7568	3869.82943
0.07508179	-3.3806096	0.00072325	0.00385063	1623.99289	1735.81868	1405.82322
0.05056832	-8.1620441	3.29E-16	8.52E-15	4065.67379	3915.70813	3001.57673
0.03587936	-3.4970394	0.00047045	0.00263357	8942.39543	9429.24098	8375.58614
0.05107807	-4.773482	1.81E-06	1.63E-05	3857.93171	3884.58167	3356.8481
0.04996367	-8.8034378	1.33E-18	4.06E-17	4323.69135	4155.38184	3163.52621
0.04842206	-2.544574	0.01094111	0.04022632	4344.56042	4651.33003	4137.76679
0.04904182	-3.3473948	0.00081575	0.00428203	3810.50201	3749.70036	3369.56664

0.05647954	-4.568805	4.91E-06	4.14E-05	3041.19229	2996.44013	2579.32102
0.04279715	-4.370085	1.24E-05	9.82E-05	5224.85563	5197.08055	4630.39845
0.07175117	-3.0464989	0.00231523	0.01063441	1771.02496	1714.03016	1496.54885
0.02719349	-7.3617452	1.82E-13	3.76E-12	14866.3648	14830.7186	12955.9583
0.03167507	5.59391268	2.22E-08	2.62E-07	11797.6633	12306.363	13401.9553
0.05387608	4.26367085	2.01E-05	0.00015332	2667.44627	2697.62616	3151.65556
0.04692146	7.57999238	3.46E-14	7.60E-13	4441.31701	4689.71932	5658.05692
0.0618996	-3.1540641	0.00161014	0.00775339	2326.90103	2307.50792	2020.55292
0.23222631	-2.8336618	0.0046018	0.01907837	184.975826	187.796283	111.075297
0.04608877	3.14174457	0.00167944	0.00803298	4010.65534	3963.43536	4467.60107
0.05097359	2.80892064	0.00497079	0.02038185	3355.1769	3227.81346	3674.81173
0.41786833	-4.869222	1.12E-06	1.04E-05	133.751751	73.6659451	28.828703
0.06203826	-2.5002297	0.01241128	0.04473834	2369.58776	2391.54935	2187.58982
0.04006296	-4.1274661	3.67E-05	0.00026466	5886.02563	5888.08787	5214.60364
0.04972772	-5.9704381	2.37E-09	3.19E-08	4148.20147	4397.13064	3470.4671
0.31917185	2.95916254	0.00308476	0.01360974	69.2473604	59.1402658	148.38303
0.09046013	6.0253954	1.69E-09	2.31E-08	1084.24292	952.469544	1422.78129
0.04134924	-3.7378484	0.0001856	0.00114511	6927.58182	6689.07533	5950.58347
0.05675169	3.22059259	0.00127926	0.00634417	2856.21647	2736.01546	3267.81828
0.04099869	-4.1868548	2.83E-05	0.00020849	5676.38636	5588.23635	5017.04223
0.29988193	-2.948395	0.00319429	0.01401018	165.055352	116.205435	87.3340121
0.19753845	-2.873415	0.0040606	0.01716381	289.321163	240.711257	183.994958
0.17790973	-4.6800063	2.87E-06	2.51E-05	323.470546	345.503658	185.690764
0.23336297	-6.3130459	2.74E-10	4.18E-09	233.354119	232.410869	81.3986909
0.49133159	3.01641664	0.00255782	0.01160909	22.7662555	15.5632278	51.7220849
0.10836405	2.6569226	0.00788575	0.03038224	628.917807	613.191177	755.4816
0.37716765	-3.4138214	0.00064059	0.00345076	82.5276761	95.4544641	42.3951515
0.04126506	5.24107462	1.60E-07	1.68E-06	5222.00985	5096.43835	5904.7967
0.21256172	-2.7221075	0.0064867	0.02561936	239.045682	212.697447	139.904
0.04189889	-9.4049965	5.20E-21	1.84E-19	6486.48562	6330.08354	4979.7345
0.09066193	4.34418153	1.40E-05	0.00010937	887.883963	883.991342	1198.08698
0.02166515	-4.497176	6.89E-06	5.67E-05	29658.7393	30173.9862	28062.1987
0.05648463	3.18716898	0.00143673	0.00701291	2701.59565	2793.08062	3025.31801
0.15471838	2.5110124	0.01203855	0.04363409	317.778983	318.527397	450.236509
0.57663884	2.54952549	0.01078696	0.03976297	14.2289097	13.4881308	43.2430546
0.09829149	2.71623343	0.00660293	0.02601588	756.0294	779.198941	932.693334
0.24210734	3.61429628	0.00030116	0.00176593	110.985495	94.4169156	177.211733
0.21038704	3.76597239	0.0001659	0.00103602	136.597533	145.256793	225.542206
0.11307627	3.89932601	9.65E-05	0.00063293	593.81983	561.313751	747.00257
0.08486879	8.04949789	8.31E-16	2.10E-14	916.341783	875.690954	1447.37047
0.22951622	3.41471786	0.00063848	0.00344055	107.19112	133.843759	200.953018
0.11801109	3.75799482	0.00017128	0.00106677	545.441537	491.798	696.976291
0.09603793	4.24925383	2.14E-05	0.00016262	766.463934	754.297776	999.677673

0.02938951	-6.4258913	1.31E-10	2.06E-09	14257.3675	13804.5831	12233.5449
0.04889451	2.87378287	0.00405588	0.01714824	3595.17118	3490.31323	3945.2928
0.15671352	-3.9596298	7.51E-05	0.00050734	410.741192	420.207152	285.743321
0.03306548	-3.1746001	0.00150043	0.00728274	8929.11511	8866.88968	8273.83777
0.06972386	-3.4275756	0.000609	0.00329355	2091.64972	2085.47253	1848.42861
0.026753	-5.0556027	4.29E-07	4.26E-06	25907.9987	26087.0825	24297.5092
0.02237091	-10.543353	5.45E-26	2.47E-24	32930.4399	33285.5942	28500.5646
0.02095873	-9.7372698	2.09E-22	8.12E-21	31855.683	31623.4414	27488.1683
0.02650572	-3.6202056	0.00029437	0.00173041	17035.7993	16619.4522	15770.1485
0.02941873	-5.5543738	2.79E-08	3.24E-07	12790.8412	12525.2858	11272.0229
0.02357101	-6.3336047	2.39E-10	3.67E-09	47487.5631	46314.091	43221.0091
0.03305152	-3.699309	0.00021619	0.001317	13078.2652	13056.5106	12374.2968
0.02443078	-7.0564627	1.71E-12	3.24E-11	36689.7179	36764.4944	33395.5088
0.02373727	-5.2040543	1.95E-07	2.02E-06	28680.7389	27845.7273	26199.3557
0.0280819	-3.6391001	0.00027359	0.00161637	22153.4638	22806.3541	21453.6425
0.02423389	-6.7409232	1.57E-11	2.72E-10	25699.3081	26023.7921	23456.3894
0.03150882	-4.051275	5.09E-05	0.00035584	11768.2569	12210.9086	11079.5489
0.02940053	-6.7366285	1.62E-11	2.80E-10	17142.0418	18031.5558	15432.6831
0.02601317	-4.4739024	7.68E-06	6.28E-05	16911.5334	16690.0055	15433.531
0.01901691	-3.2819801	0.00103081	0.0052434	62325.4701	60704.889	59017.4425
0.03218365	-3.2237061	0.00126543	0.00629079	16231.3916	15807.0518	15376.7215
0.0231921	-5.971342	2.35E-09	3.17E-08	35478.3634	36089.0503	33104.678
0.02528408	-7.6837004	1.55E-14	3.48E-13	21201.0754	21128.6381	18770.8773
0.04698413	-3.0474713	0.00230776	0.01060896	38651.4102	39771.31	36556.4913
0.07262613	-5.6024612	2.11E-08	2.50E-07	25452.6736	25471.8162	20176.7005
0.03003776	-5.6103594	2.02E-08	2.39E-07	20979.1044	21873.598	19560.275
0.03292823	-4.5878526	4.48E-06	3.80E-05	11395.4595	11588.3795	10589.4609
0.01779667	-5.1971365	2.02E-07	2.09E-06	63272.1669	62395.0555	58757.1363
0.02317826	-9.675824	3.82E-22	1.47E-20	40065.7638	38633.1193	34062.8084
0.02473642	-5.8404146	5.21E-09	6.68E-08	44746.1265	45604.4078	41965.2647
0.01687049	-8.0925852	5.84E-16	1.49E-14	81825.7165	80888.3204	73745.5182
0.01946304	-5.3516232	8.72E-08	9.51E-07	68511.2515	69808.3397	65180.8497
0.02452558	-4.7829211	1.73E-06	1.56E-05	23296.5195	22759.6644	21439.2281
0.2147765	-2.5409485	0.01105522	0.04057121	239.045682	205.434608	140.751903
0.01744136	-3.5119401	0.00044485	0.00250475	79292.022	78079.6766	74969.8902
0.02553552	-6.8121777	9.61E-12	1.70E-10	19231.7943	18816.98	16797.8069
0.05514518	-2.4807605	0.01311024	0.04680602	22213.2252	23073.0041	21287.4535
0.02304424	-10.572806	3.98E-26	1.83E-24	45457.572	44855.2978	38971.3191
0.03046511	-7.7438177	9.65E-15	2.21E-13	14883.4395	15328.7419	13081.448
0.02146276	-6.9373711	3.99E-12	7.24E-11	62249.5826	61561.9041	56996.0417
0.02551079	-6.4129271	1.43E-10	2.23E-09	22577.4853	23101.0179	20695.6172
0.02932851	-6.3887235	1.67E-10	2.60E-09	23254.7814	23620.8297	21238.2751
0.02119416	-8.9490673	3.58E-19	1.14E-17	48763.422	49056.3317	43633.0899

0.02754031	-5.7273915	1.02E-08	1.26E-07	25596.8599	25377.3993	23498.7846
0.03874793	-4.4932009	7.02E-06	5.76E-05	7263.38409	6946.38736	6286.35307
0.07672955	-2.6746451	0.00748084	0.02904992	16633.5954	17686.0521	15538.6709
0.02895572	-4.8149045	1.47E-06	1.35E-05	17421.877	17249.2442	16116.9408
0.01941021	-6.4916859	8.49E-11	1.36E-09	64365.8958	62506.0732	58290.7896
0.02430776	-3.7433567	0.00018158	0.00112366	22222.7111	21896.424	20926.2468
0.02498537	-4.0545143	5.02E-05	0.00035139	18390.3915	18324.1445	17209.0399
0.08990577	4.20288277	2.64E-05	0.00019596	1033.01884	977.370709	1238.78633
0.02833356	-5.9762842	2.28E-09	3.08E-08	19125.5518	19180.122	17454.0839
0.02740785	-5.4695489	4.51E-08	5.11E-07	30408.1286	29161.3388	27468.6666
0.02887904	-5.1205686	3.05E-07	3.08E-06	15954.4021	16608.0392	14830.6719
0.02456601	-3.2227464	0.00126968	0.00630236	34223.3735	32873.6874	31322.3858
0.06008596	-5.0341808	4.80E-07	4.74E-06	2731.95066	2637.44835	2213.02691
0.03249098	-6.7958918	1.08E-11	1.89E-10	10313.1137	10542.5305	8856.34715
0.05134784	-3.7200058	0.00019922	0.00122133	3589.47961	3498.61362	3071.95268
0.18915402	4.13912229	3.49E-05	0.00025267	227.662555	168.082861	350.183952
0.07640306	-3.2037006	0.00135673	0.00666203	1525.33912	1520.00859	1291.35632
0.06183407	-4.0737596	4.63E-05	0.00032677	2501.44232	2416.45051	2036.66308
0.03903472	6.97623579	3.03E-12	5.60E-11	6313.84152	6665.21171	7746.44209
0.03091774	4.08915667	4.33E-05	0.00030807	15773.2207	14875.3332	16913.9697
0.11288832	-5.9563442	2.58E-09	3.46E-08	875.552242	872.578308	585.900994
0.1081712	3.33941773	0.00083954	0.00438591	655.478439	660.918409	798.724655
0.14909759	-4.4067834	1.05E-05	8.40E-05	537.852786	455.483802	306.940897
0.29606131	-3.9116107	9.17E-05	0.0006052	142.289097	128.656017	66.1364364
0.05423434	-5.1063483	3.28E-07	3.31E-06	3191.07014	3231.96365	2615.78085
0.04256534	2.85595908	0.0042907	0.01797916	5494.25632	5855.92386	6125.25149
0.12187472	-3.9018833	9.54E-05	0.00062678	663.067191	627.716857	470.586182
0.05362889	6.37380142	1.84E-10	2.85E-09	3439.60176	3627.26964	4290.38933
0.03776907	14.2285811	6.09E-46	5.53E-44	5264.69658	5268.6714	7584.49261
0.06523885	2.57722452	0.00995972	0.0371483	1984.4586	2075.09705	2212.17901
0.06192347	-11.826553	2.85E-32	1.74E-30	2830.60443	2808.64385	1666.97736
0.01833739	-23.457537	1.11E-121	4.79E-119	54419.8879	54148.6199	40349.1615
0.04831578	6.87572781	6.17E-12	1.11E-10	3450.0363	3586.80524	4490.49445
0.22920652	-5.8154808	6.05E-09	7.71E-08	252.325998	222.035384	92.4214303
0.03730227	-3.0925288	0.00198459	0.00931357	6811.85336	6814.6187	6246.50163
0.07675584	5.58559963	2.33E-08	2.73E-07	1326.13438	1254.39616	1786.53169
0.0525213	7.17780937	7.08E-13	1.39E-11	2849.57631	2897.87303	3647.67884
0.08217246	4.96953456	6.71E-07	6.45E-06	1041.55619	1087.35085	1399.04
0.03352928	5.20852571	1.90E-07	1.98E-06	8080.1235	7981.86079	9133.61144
0.03774452	2.77290878	0.00555577	0.02243886	6341.35074	6307.25747	6717.08781
0.03049436	-3.8863203	0.00010178	0.00066541	13070.6764	13442.4787	12402.2776
0.04630381	-5.5370352	3.08E-08	3.55E-07	4418.55075	4408.54368	3667.18061
0.02633467	-7.9573363	1.76E-15	4.29E-14	21214.3557	20953.2924	17897.5372

0.03527419	-7.7115107	1.24E-14	2.84E-13	8487.07032	8496.48486	6939.2384
0.05365674	-3.2202233	0.00128091	0.00634851	3991.68346	3680.18461	3362.78342
0.02842755	-8.8000304	1.37E-18	4.18E-17	14427.1658	14701.025	12343.7723
0.10750799	3.27900512	0.00104174	0.0052908	686.78204	624.604211	819.074327
0.09378957	2.57772632	0.00994527	0.03711247	848.99161	915.117797	1042.07282
0.05405108	-4.7721899	1.82E-06	1.64E-05	3375.09737	3543.22821	2844.71467
0.07300158	4.945516	7.59E-07	7.23E-06	1351.74642	1392.39012	1776.35685
0.02281152	-7.0976507	1.27E-12	2.43E-11	33436.9891	32391.2273	29233.1528
0.03779012	2.59130065	0.00956139	0.03585795	6692.33051	6474.30278	6992.65629
0.03515437	-3.2535786	0.00113961	0.00572244	8235.69292	8516.19828	7741.35467
0.03801096	3.67205682	0.00024061	0.00144593	6069.10427	6055.13318	6610.25203
0.02632937	-2.8447457	0.00444469	0.01852518	16030.2896	15846.4786	15200.3576
0.06339069	-6.6740294	2.49E-11	4.23E-10	2446.42387	2378.06122	1795.85862
0.05811619	6.97786836	3.00E-12	5.54E-11	2189.3549	2152.91319	2904.91578
0.08038699	2.90054392	0.00372516	0.0159719	1159.18184	1167.24209	1358.34065
0.06102839	3.34424134	0.00082508	0.00432272	2097.34129	2069.9093	2408.89251
0.04966521	-3.289769	0.0010027	0.00511942	3660.62416	3736.21223	3294.10327
0.20850701	3.4071645	0.00065642	0.0035314	150.826443	135.918857	236.564946
0.44798742	4.21703507	2.48E-05	0.00018513	17.0746916	21.788519	67.8322424
0.04376481	-4.1363904	3.53E-05	0.00025525	4833.08632	4892.04129	4275.12708
0.07054439	4.49395089	6.99E-06	5.74E-05	1532.92787	1477.4691	1887.43215
0.04222743	3.88771038	0.00010119	0.00066187	4727.79239	4648.21738	5256.15089
0.0428974	4.68405286	2.81E-06	2.46E-05	4445.11138	4535.12459	5166.27316
0.12020116	-3.2783542	0.00104414	0.00529811	633.660777	650.542924	498.566982
0.0847852	3.48420274	0.00049361	0.00274448	1179.10231	1072.82517	1406.67113
0.04350729	-6.7121954	1.92E-11	3.29E-10	5879.38548	5759.43185	4632.94216
0.28270515	-4.866505	1.14E-06	1.06E-05	153.672224	156.669827	55.9616
0.07433872	4.85480058	1.21E-06	1.12E-05	1488.34395	1371.63915	1813.66458
0.11937597	3.049203	0.00229449	0.0105539	522.675282	520.849359	703.759515
0.07125028	4.23243579	2.31E-05	0.00017423	1486.44676	1484.73194	1865.38667
0.0218496	3.21156907	0.00132012	0.00651535	24980.2738	25028.783	26325.6933
0.04303018	5.72966516	1.01E-08	1.24E-07	4406.21903	4342.14057	5162.03365
0.02456052	16.4407653	9.77E-61	1.49E-58	15733.3797	15680.4708	20801.605
0.0641309	-4.0757064	4.59E-05	0.00032446	2311.72352	2331.37153	1884.88844
0.1490398	-6.2116231	5.24E-10	7.72E-09	500.85762	478.309869	257.762521
0.04385814	3.69501786	0.00021987	0.00133598	4500.12983	4688.68178	5165.42526
0.04360514	3.77706226	0.00015869	0.00099514	4532.38203	4465.60884	5110.31156
0.4341961	3.51928895	0.00043271	0.0024456	28.4578193	20.7509705	78.8549818
0.04702679	-3.3118733	0.00092674	0.00478061	4822.65178	4551.72537	4177.61823
0.02034022	33.8048913	1.67E-250	5.49E-247	30518.1655	30946.9598	49044.4071
0.05706707	7.96678258	1.63E-15	3.99E-14	2330.6954	2259.78068	3092.30235
0.06835888	-3.7080144	0.00020889	0.00127729	1919.00562	1970.30465	1639.84446
0.07676799	-2.4756791	0.0132983	0.04728221	1473.16645	1513.7833	1314.2497

0.05248831	-2.5433517	0.01097946	0.04033824	3496.5174	3338.83115	3128.76218
0.14185118	3.39553572	0.00068494	0.00366807	349.082584	332.015527	462.955055
0.07857357	-2.8057204	0.00502042	0.02055459	1455.14316	1543.8722	1269.31084
0.04297363	4.24529178	2.18E-05	0.00016529	4622.49845	4615.01583	5318.89571
0.3471068	-4.7126692	2.44E-06	2.17E-05	103.396744	120.355629	37.3077333
0.03939263	-5.1522419	2.57E-07	2.62E-06	6201.90743	6261.60534	5451.16858
0.09525392	2.65793975	0.00786199	0.03032932	870.809272	859.090177	1072.59733
0.02789938	-9.4507546	3.36E-21	1.21E-19	18613.311	18204.8264	15062.1494
0.08207162	-3.3200188	0.00090011	0.00466163	1421.94237	1338.43759	1142.12538
0.04750491	-4.9474438	7.52E-07	7.17E-06	4797.98834	4589.07712	4066.54293
0.08897102	-9.0562619	1.35E-19	4.41E-18	1502.57286	1384.08973	840.271903
0.05837854	3.43397572	0.0005948	0.00322525	2712.03018	2698.66371	2982.07496
0.66568704	2.54827125	0.01082583	0.03987185	10.4345338	8.30038819	32.2203152
0.25385471	-2.5751061	0.01002094	0.0373427	155.569412	160.820021	91.5735273
0.04500574	-2.9497255	0.00318056	0.0139649	4879.56742	4990.6084	4597.33023
0.11717159	9.07632102	1.12E-19	3.70E-18	2711.08159	2962.20103	5514.76131
0.02791431	5.89081127	3.84E-09	5.03E-08	14224.1667	13892.7747	15588.6972
0.08000842	3.4652646	0.00052971	0.00292696	1402.0219	1275.14713	1574.55593
0.04349799	11.2014429	4.01E-29	2.15E-27	3906.31	3998.71201	5562.24388
0.0495566	6.37924533	1.78E-10	2.76E-09	3266.00907	3421.83503	4197.12
0.12642356	-2.830685	0.00464484	0.01922287	589.07686	600.740595	440.061673
0.04107812	-5.504255	3.71E-08	4.24E-07	5646.03136	5624.55054	4814.39341
0.14455156	3.42441207	0.00061613	0.00332776	322.521953	319.564945	457.867636
0.07436142	-3.3322675	0.00086141	0.00448733	1619.24992	1607.16266	1365.97178
0.05391037	-3.1761683	0.00149234	0.00725206	3198.65889	3103.30763	2787.90516
0.02304565	5.17387292	2.29E-07	2.35E-06	23496.6728	23108.2807	25137.7811
0.06148478	5.00272769	5.65E-07	5.52E-06	2207.37819	2066.79666	2646.30536
0.07839414	3.3221721	0.0008932	0.00463086	1204.71435	1246.09578	1466.87224
0.17912533	3.19671123	0.00139004	0.00680523	202.999111	211.659899	293.374449
0.04182525	-3.1592149	0.00158195	0.0076277	5253.31345	5297.72276	4794.04373
0.13832312	-2.5132888	0.01196114	0.04337266	571.053575	482.460063	412.928776
0.04007159	-3.3020128	0.00095994	0.0049333	7311.76238	6995.15214	6664.51782
0.04477726	-4.079022	4.52E-05	0.00032014	4930.7915	4920.0551	4427.74963
0.05593289	3.76247653	0.00016824	0.00104922	2503.33951	2520.20536	2932.89658
0.03153275	-4.9791638	6.39E-07	6.16E-06	10258.0953	10243.7166	9250.62206
0.03452891	-5.2630635	1.42E-07	1.50E-06	8312.52903	8239.17282	7308.07622
0.04990641	-3.1636509	0.00155804	0.00752677	3601.81133	3615.8566	3223.72732
0.04007984	2.98111536	0.00287201	0.01280865	5326.35519	5369.31361	5847.1393
0.03004321	-9.3051958	1.34E-20	4.70E-19	12022.4801	12022.0747	9964.55641
0.02642011	-9.1723609	4.63E-20	1.56E-18	16869.7953	16749.1458	14310.0594
0.02738815	-10.235182	1.38E-24	5.93E-23	15579.7075	15849.5912	12991.5702
0.09443215	2.48951216	0.01279185	0.04593883	858.47755	875.690954	1052.24766
0.08141108	-4.8698276	1.12E-06	1.04E-05	1507.31583	1419.36638	1113.29668

0.03808277	-16.824959	1.60E-63	2.69E-61	7643.77027	7683.04681	4929.70822
0.03812249	5.23654585	1.64E-07	1.72E-06	6783.39554	6611.25919	7850.73416
0.02871935	-2.9963757	0.0027321	0.01229481	12614.4027	12483.7838	11824.8557
0.0601369	-23.621401	2.32E-123	1.06E-120	3635.96072	3754.88811	1376.14662
0.03774557	-9.339539	9.68E-21	3.41E-19	79009.341	81351.0671	64175.2367
0.06295108	9.03900734	1.58E-19	5.10E-18	1716.9551	1762.79494	2551.34022
0.04129036	-3.6653106	0.00024704	0.00147779	5540.73743	5529.09608	5032.30449
0.02753407	-2.5954312	0.00944723	0.03552712	13725.2063	13752.7057	13097.5581
0.20122404	9.57717823	9.97E-22	3.72E-20	96.7565858	91.30427	353.575564
0.03905529	3.28581235	0.00101689	0.00518382	5792.11483	5724.1552	6357.57692
0.03692175	-2.7742602	0.00553274	0.02237886	7670.33091	7524.30189	7196.15302
0.03394078	5.22633766	1.73E-07	1.80E-06	7759.49874	7785.76412	8709.65993
0.19782002	-4.9332434	8.09E-07	7.67E-06	378.488997	310.227008	198.409309
0.10283432	3.81982131	0.00013355	0.00085444	707.651108	721.096224	891.993988
0.18763954	5.49542648	3.90E-08	4.44E-07	150.826443	162.895118	311.180412
0.06372375	3.11523011	0.00183801	0.00869524	2146.66817	2198.56532	2397.02187
0.03542963	-13.653885	1.91E-42	1.62E-40	8901.60589	8726.82063	6307.55064
0.02993047	5.34860851	8.86E-08	9.66E-07	12748.1545	13049.2478	14150.6537
0.02268597	3.12966274	0.00175007	0.00832957	22473.1399	22602.9946	23596.2934
0.0562582	3.42320469	0.00061887	0.00334058	2556.46077	2683.10048	3009.20786
0.04227276	2.98530712	0.00283294	0.01268261	4758.14739	4691.79442	5146.7714
0.0292149	2.50844126	0.01212651	0.0439045	11438.1462	11352.8559	12038.5272
0.06343427	-2.4795197	0.01315595	0.04691193	2188.40631	2214.12855	1949.32907
0.08714798	3.04510911	0.00232596	0.01068069	1002.66383	1002.27187	1237.09052
0.17319338	2.60923982	0.00907436	0.03431137	256.120374	226.185578	347.640243
0.06650622	3.84448936	0.0001208	0.00077805	1720.74948	1695.35429	2039.20679
0.15031266	4.10328272	4.07E-05	0.00029085	295.961321	285.325844	427.343127
0.03876602	17.509586	1.21E-68	2.21E-66	5903.10033	5950.34078	9766.99501
0.07222556	6.94958956	3.66E-12	6.66E-11	1439.01706	1352.96327	2006.13857
0.04476374	-3.6528547	0.00025934	0.00154104	4561.78844	4569.3637	4072.47826
0.05087742	4.50712683	6.57E-06	5.42E-05	3251.78016	3374.1078	3958.85925
0.04195206	-3.1309544	0.00174239	0.00830264	5667.84902	5643.22642	5058.58948
0.06652855	2.89321163	0.00381324	0.01626173	1759.64183	1738.93132	1985.7889
0.04452022	-4.1337329	3.57E-05	0.00025811	4802.73131	4759.23508	4158.11646
0.11694894	2.88027037	0.00397334	0.01686438	605.202958	542.637878	696.128388
0.10480113	2.64601198	0.00814469	0.03124383	662.118597	700.345253	836.032388
0.17485286	5.29232286	1.21E-07	1.29E-06	183.078638	200.246865	348.488146
0.03147315	6.81124749	9.68E-12	1.71E-10	9464.12212	9260.12057	10822.6343
0.1068896	5.71516406	1.10E-08	1.35E-07	572.950763	570.651688	854.686255
0.19325122	-4.9593899	7.07E-07	6.78E-06	334.853674	293.626232	149.230933
0.02156477	-19.707188	1.87E-86	4.59E-84	32776.7677	32349.7254	24335.6649
0.06661587	7.2521468	4.10E-13	8.25E-12	1581.30616	1634.13892	2275.77173
0.06603116	-14.454393	2.35E-47	2.29E-45	2647.52579	2608.39699	1334.59937

0.26453822	3.84590233	0.00012011	0.00077456	78.7333002	78.8536878	150.926739
0.04740846	-3.1262139	0.00177073	0.00841813	4182.35085	4090.01628	3768.08107
0.05733061	8.8473375	8.96E-19	2.78E-17	2160.89708	2217.24119	3068.56107
0.0443437	9.1948673	3.75E-20	1.28E-18	3873.10921	3978.99859	5228.17009
0.05689058	4.73122191	2.23E-06	1.98E-05	2390.45682	2457.95245	2954.94206
0.14464657	-2.965186	0.003025	0.01338164	487.577305	438.883025	352.727661
0.03265818	6.86077295	6.85E-12	1.22E-10	11084.3206	11450.3855	12854.2099
0.02121251	-4.5042033	6.66E-06	5.49E-05	30729.7019	31182.4833	29033.8956
0.04248458	-7.8296213	4.89E-15	1.16E-13	7498.6354	8101.17887	6105.74972
0.02740055	-17.439249	4.15E-68	7.50E-66	16570.0396	16573.8001	11796.027
0.03013972	20.8193619	2.89E-96	8.19E-94	12031.0174	12204.6833	18255.3522
0.05373011	3.42841183	0.00060712	0.00328559	3066.80433	3005.77807	3546.77838
0.05892845	2.85516598	0.00430143	0.01801034	2336.38697	2332.40908	2575.0815
0.03618998	-22.888366	6.07E-116	2.43E-113	9283.88926	9459.32989	5254.45508
0.03997923	10.987162	4.41E-28	2.23E-26	4728.74098	4756.12243	6443.21513
0.07517554	-3.099905	0.00193583	0.00910554	1563.28288	1573.96111	1340.53469
0.08616201	-6.811941	9.63E-12	1.70E-10	1398.22752	1528.30897	957.282521
0.27829297	3.08383987	0.00204348	0.00954358	69.2473604	113.092789	161.101576
0.10785945	-5.3645253	8.12E-08	8.91E-07	848.99161	865.315468	590.140509
0.03289609	7.71409071	1.22E-14	2.78E-13	8108.58132	8010.91215	9652.5281
0.04820379	4.55810455	5.16E-06	4.35E-05	4593.09204	4619.16603	5134.05285
0.07149509	4.88187507	1.05E-06	9.84E-06	1453.24597	1496.14497	1838.25377
0.03345592	2.82355438	0.00474944	0.01957683	8136.09055	8083.54054	8691.00606
0.08006213	-3.6353758	0.00027758	0.00163814	1451.34879	1450.49284	1217.58875
0.08873796	2.75349809	0.00589621	0.02363952	967.565858	1057.26195	1199.78279
0.11804029	6.5206247	7.00E-11	1.13E-09	458.170891	529.149747	870.796412
0.0458833	-2.6151597	0.00891858	0.03381772	4613.96111	4634.72925	4349.74255
0.05326721	-6.1915623	5.96E-10	8.68E-09	3933.81923	3728.94939	3116.89154
0.02538444	-5.7503725	8.90E-09	1.11E-07	17753.8849	17901.8622	16044.0211
0.08005393	2.48032524	0.01312626	0.04684284	1246.45249	1358.15102	1471.11176
0.04146818	-6.0926019	1.11E-09	1.56E-08	5598.60166	5697.17894	4737.23423
0.07268332	7.60530464	2.84E-14	6.33E-13	1712.21213	2009.73149	2732.79147
0.20604969	-4.6701707	3.01E-06	2.62E-05	260.863344	254.199388	135.664485
0.03198705	-2.7728109	0.00555744	0.02244009	9620.64012	9576.57287	9058.99598
0.02687706	13.9269418	4.35E-44	3.80E-42	14607.3987	14861.845	18839.5574
0.02414561	-24.221701	1.31E-129	6.54E-127	24846.5221	24458.1313	16370.4638
0.06770496	3.87852171	0.00010509	0.00068573	1692.29166	1799.10914	2073.97081
0.0547842	3.34811395	0.00081364	0.00427258	3143.64044	3441.54845	3762.14575
0.02472499	-10.735444	6.94E-27	3.31E-25	19955.5715	20200.0322	16668.0778
0.05672626	-2.724315	0.0064435	0.02547323	2771.7916	2868.82167	2531.83845
0.02214729	-19.555173	3.73E-85	9.01E-83	36801.652	37848.7326	27793.4134
0.05086504	-2.7111494	0.00670504	0.02638654	3993.58065	3962.39781	3495.90419
0.03708926	-3.5032573	0.00045961	0.002579	7083.15123	7223.41282	6482.21867

0.04234126	-3.6328445	0.00028031	0.00165311	7009.1609	6978.55137	6524.61382
0.03065343	11.9272487	8.53E-33	5.37E-31	10001.0263	10349.5465	13060.2504
0.03538958	3.57531433	0.00034981	0.00202157	7679.81685	7962.14737	8605.36786
0.04086946	-3.8622454	0.00011235	0.00072759	6294.86964	6218.0283	5481.69309
0.03375356	-10.518009	7.14E-26	3.22E-24	10090.1941	10008.1931	7731.17983
0.0278588	-2.6451875	0.00816457	0.03129084	16233.2887	16575.8752	15829.5017
0.0186845	-3.3771553	0.0007324	0.00389301	50370.3402	50231.8742	48521.2509
0.03862216	-4.5298805	5.90E-06	4.92E-05	6528.22376	6412.04987	5728.43287
0.10105985	2.53536906	0.01123289	0.04109746	741.800491	737.697	858.077867
0.10092183	-3.7918462	0.00014953	0.00094492	966.617264	914.080249	740.219346
0.15403615	-2.5627017	0.01038612	0.038468	457.222297	387.005599	327.29057
0.03814377	-3.5600741	0.00037075	0.0021336	7157.14156	7403.94626	6718.78361
0.1361109	3.20662209	0.00134303	0.00661452	376.591809	444.070768	564.703418
0.0950692	4.38502694	1.16E-05	9.23E-05	773.104092	767.785907	1028.50638
0.03000317	-3.6862964	0.00022754	0.00137597	12086.9845	12271.0864	11153.3165
0.10214923	3.75198532	0.00017544	0.00108814	706.702514	803.062557	996.286061
0.0404569	-7.5823531	3.39E-14	7.48E-13	6686.63895	6995.15214	5584.28936
0.04266952	3.56088333	0.00036961	0.00212778	4774.27349	4702.16991	5197.64558
0.04205594	9.23897846	2.49E-20	8.57E-19	4360.68652	4465.60884	5775.91544
0.14455599	3.96575883	7.32E-05	0.00049652	304.498667	336.165722	477.369406
0.02180913	-4.3408936	1.42E-05	0.00011071	27627.7996	27943.2568	25923.7873
0.04212537	-3.4005941	0.0006724	0.00360792	5429.75193	5438.82936	4997.54046
0.06677961	-3.5715347	0.0003549	0.00204882	2308.87774	2142.5377	1904.39021
0.04923495	7.35835425	1.86E-13	3.85E-12	3081.98183	3097.08234	3968.18618
0.07870082	-2.5961765	0.00942676	0.03546639	1465.5777	1456.71813	1301.53115
0.13642858	3.09095805	0.00199512	0.00935229	379.437591	364.179532	498.566982
0.02575104	11.7171085	1.04E-31	6.22E-30	15540.8151	15379.5818	18885.3442
0.06060758	-5.8851255	3.98E-09	5.18E-08	2713.92737	2599.05905	2074.81872
0.05146874	-2.542969	0.0109915	0.0403644	3339.0508	3375.14535	3075.34429
0.07385522	3.08239669	0.00205341	0.0095818	1421.94237	1523.12123	1721.24315
0.05160387	-10.084306	6.48E-24	2.71E-22	3804.81045	3886.65677	2666.65503
0.06671543	4.81295288	1.49E-06	1.36E-05	1992.04735	1876.92528	2330.88543
0.07714838	-31.484634	1.41E-217	2.11E-214	2934.00117	2995.40259	535.874715
0.04320077	3.07718636	0.00208965	0.00972328	4470.72342	4537.19969	4955.99321
0.06067102	-11.965371	5.40E-33	3.41E-31	2999.45416	3132.35899	1885.73634
0.03499348	-7.9443601	1.95E-15	4.74E-14	8249.92183	8271.33683	6790.85537
0.04309972	3.22574446	0.00125645	0.00625172	4754.35302	4620.20357	5118.7906
0.04348055	-2.974393	0.00293569	0.01304309	5425.95755	5743.86862	5095.04931
0.40049844	5.80041272	6.62E-09	8.38E-08	25.6120374	17.6383249	123.793842
0.06698787	17.8535562	2.71E-71	5.12E-69	1230.32639	1334.2874	2914.24272
0.05632937	-3.2603749	0.00111265	0.00560247	2988.07103	2999.55278	2701.41906
0.06976227	4.7347913	2.19E-06	1.95E-05	1506.36724	1545.9473	1938.30633
0.04565615	7.86994255	3.55E-15	8.44E-14	4511.51296	4375.34212	5530.02356

0.05750843	-2.8131227	0.00490629	0.0201526	3480.39131	3181.12377	2941.37561
0.13633801	-4.9879266	6.10E-07	5.91E-06	558.721853	626.679308	388.339588
0.08904222	4.51354239	6.38E-06	5.28E-05	984.640549	930.681025	1220.98036
0.03261125	-2.9859756	0.00282675	0.01266306	9429.02414	9276.72135	8756.2946
0.0560908	3.64983481	0.00026241	0.00155645	2574.48406	2711.11429	3032.94914
0.03262117	6.71244898	1.91E-11	3.29E-10	8181.62306	8238.13527	9538.06119
0.03608419	-4.6674972	3.05E-06	2.66E-05	7897.99346	7796.1396	6891.75583
0.07861519	-2.7686156	0.0056295	0.0227199	1660.03946	1562.54808	1440.58725
0.03511697	2.86216994	0.00420751	0.01768016	8685.32646	8316.98896	9202.29159
0.04683529	-4.3550904	1.33E-05	0.00010466	4192.78538	4196.88378	3639.19981
0.0428938	-4.4543786	8.41E-06	6.85E-05	5191.65484	5140.01538	4482.01542
0.0657535	-3.2157782	0.00130091	0.00643021	2058.44893	2114.52389	1787.37959
0.04880108	-4.3679322	1.25E-05	9.91E-05	3860.77749	3884.58167	3321.23617
0.06917931	4.0061122	6.17E-05	0.00042505	1611.66117	1705.72977	1979.00567
0.06599158	-2.9832163	0.00285236	0.01273511	2050.86018	2129.04957	1805.18555
0.11507603	-2.8509808	0.00435846	0.01821198	745.594867	679.594283	572.334546
0.04969156	-6.5420015	6.07E-11	9.89E-10	3856.03452	3850.34257	3088.06284
0.11096177	-3.6695293	0.000243	0.0014571	750.337837	808.2503	578.269867
0.0809925	3.67544914	0.00023743	0.00142894	1144.95293	1111.21447	1393.95258
0.02599161	-10.113108	4.83E-24	2.03E-22	19567.5966	19727.9476	16147.4653
0.11229903	-3.3962234	0.00068323	0.00366125	739.903303	829.00127	603.706958
0.02546674	-7.9679754	1.61E-15	3.96E-14	18860.8941	18548.2549	16318.7417
0.04987425	-5.7390523	9.52E-09	1.18E-07	4119.74365	3996.63691	3266.97038
0.03084559	6.85304598	7.23E-12	1.29E-10	10314.0623	10032.0567	11732.4342
0.06664014	-3.0134518	0.00258294	0.01171019	1992.04735	1986.90542	1724.63476
0.05311929	-3.9594925	7.51E-05	0.00050742	3249.88297	3215.36287	2810.79855
0.03129307	-3.3406755	0.00083575	0.00436887	10771.2846	10524.8922	9910.29062
0.14396654	3.26344785	0.00110065	0.00554716	366.157276	480.384966	595.227927
0.07302617	-3.5804893	0.00034295	0.00198825	1764.3848	1723.3681	1421.08548
0.08739048	-8.9327198	4.16E-19	1.31E-17	1429.53112	1369.56405	819.074327
0.04939284	-5.3673002	7.99E-08	8.80E-07	3985.0433	4012.20014	3266.12247
0.04674132	6.02856777	1.65E-09	2.27E-08	3851.29155	3718.57391	4551.54347
0.0415414	-3.2041859	0.00135445	0.0066528	5524.61133	5399.40251	4989.06143
0.02963054	-2.808597	0.00497579	0.02039726	13282.2129	12869.7519	12274.2443
0.10555877	-2.9384252	0.00329884	0.01440387	815.790821	811.362945	670.691297
0.12773386	-4.20012	2.67E-05	0.00019788	630.814995	626.679308	460.411346
0.02825287	-3.0911587	0.00199377	0.00934864	14834.1126	14718.6633	13698.7214
0.04586011	3.05823143	0.00222648	0.01028134	4231.67774	4380.52986	4660.07506
0.06353408	-3.2505014	0.00115202	0.0057759	2503.33951	2345.89721	2130.78032
0.02965967	-7.5626631	3.95E-14	8.58E-13	12118.2881	12259.6733	10452.9486
0.1148849	4.34736005	1.38E-05	0.00010811	541.647161	561.313751	821.618037
0.06732174	-4.1530331	3.28E-05	0.00023926	2007.22486	2051.23343	1689.02284
0.14563911	3.85814172	0.00011425	0.00073933	301.652885	338.240819	472.281988

0.08532255	3.16646032	0.00154306	0.00746321	1186.69107	1093.57614	1421.93338
0.07148046	-3.4549191	0.00055046	0.00302476	1769.12777	1814.67237	1534.70449
0.13844074	-2.5015541	0.01236496	0.0446203	473.348395	480.384966	362.902497
0.10008865	-3.0069038	0.00263923	0.01192919	1033.96744	1043.77381	902.168824
0.06632864	5.45078542	5.01E-08	5.64E-07	1703.67478	1738.93132	2162.15273
0.07204247	6.27882755	3.41E-10	5.15E-09	1456.09176	1377.86444	1910.32553
0.13986509	3.39613248	0.00068345	0.00366127	342.442426	369.367274	507.893915
0.13383709	4.27393853	1.92E-05	0.00014717	485.680117	421.2447	616.425503
0.12369068	3.66517648	0.00024717	0.00147779	446.787764	470.009481	645.254206
0.09945886	2.80842587	0.00497843	0.02040301	813.893633	769.861004	924.214303
0.07880142	6.09871093	1.07E-09	1.51E-08	1220.84045	1144.41602	1616.10318
0.06102863	4.04589669	5.21E-05	0.00036318	2722.46472	2502.56704	3198.29023
0.15234717	-2.5351269	0.01124066	0.04111399	467.656831	432.657734	377.316849
0.10291887	3.24123009	0.00119015	0.00594745	713.342671	733.546806	869.948509
0.08849898	-2.5639803	0.01034794	0.03835251	1259.7328	1209.78158	1006.4609
0.10982505	2.91762904	0.00352704	0.01525381	608.997334	664.031055	769.895952
0.49989959	2.93987971	0.0032834	0.01435503	18.0232856	19.7134219	43.2430546
0.05247955	6.26378348	3.76E-10	5.65E-09	2777.48317	2794.11817	3453.50904
0.06201739	-3.5953806	0.00032392	0.00188856	2420.81183	2453.80226	2135.86773

shRNA2.1
3083.84708
2779.73889
7397.19405
2578.97997
3275.10262
4972.64402
6133.2444
2803.49734
1442.13804
5634.31691
232.83283
4316.91074
1726.05154
325.490793
3442.5997
629.59898
563.075314
5589.17585
1938.68969
3877.37938
912.324559
9043.65478
10403.8262
3585.15042
664.048735
20573.6316
21465.7614
3879.75522
11488.3995
3077.90747
3981.91657
2923.47753
2211.91189
40758.8124
3026.82679
16254.345
8998.51372
5217.35607
2792.80604
4717.24066
983.599916
12270.0526

43361.5509
1683.28633
5836.26375
4762.38172
48733.3369
2271.30802
1936.31384
2518.39592
425.276292
3190.76011
18160.9608
732.948246
361.128471
1383.92983
2840.32294
3180.06881
1815.14574
14157.6616
5361.09471
588.021689
351.625091
23171.6183
34.4497555
24.9463747
7553.99983
9199.27264
3076.71954
6956.47477
2032.53557
1587.0646
3061.27655
274.410121
6869.75642
11304.2715
1573.99745
4198.11848
1935.12592
3800.16441
4287.21268
2468.50317
1272.26511
931.331321
3186.00842

1791.38729
59377.1234
4872.85852
8062.43071
563.075314
5633.12899
12852.1346
1200.98975
1964.82399
8819.13741
5056.98652
3137.3036
22435.1063
3339.25044
7500.54332
4780.20056
3358.2572
14288.3331
1347.10423
1819.89743
5589.17585
5333.77249
4528.36096
2918.72584
1704.66894
98.5975761
1446.88973
6440.91636
2401.9795
2287.93893
788.780609
225.705295
7393.63028
3964.09773
957.465618
14797.9519
4360.86388
1416.00374
5741.22994
1979.07906
4841.97253
4161.29288
906.384946

3377.26396
2306.9457
7311.66362
3696.81514
3736.01659
1392.24529
19800.294
1728.42739
6322.1241
4446.3943
1722.48777
20056.8852
652.169509
607.02845
584.457921
146.11448
500.115416
250.651669
677.115884
862.43181
4314.5349
5928.92171
5776.86762
427.652137
44567.2923
2331.89207
2080.05248
3554.26443
5629.56522
917.07625
1003.7946
656.9212
14907.2407
2531.46307
593.961302
5884.96858
1982.64282
5557.10194
19683.8775
3449.72724
22173.7633
291.041038
2403.16743

3080.28331
54.6444398
13493.6129
1947.00515
12108.4951
1780.69598
515.55841
169.872932
345.685478
7265.33464
3404.58618
2170.3346
5106.87927
862.43181
6247.28497
4344.23296
4301.46775
934.895089
16889.8836
9070.977
14211.1181
105.725112
4103.08467
78.4028918
13703.8752
4884.73774
1992.14621
4352.54842
2632.43649
3036.33017
1670.21918
446.658899
370.631852
2660.94663
2514.83215
36490.6065
924.203785
3221.6461
607.02845
3807.29194
1589.44044
677.115884
1386.30568

1066.7545
664.048735
4309.7832
8876.15769
4392.93779
614.155986
699.686413
894.50572
91.4700405
2096.68339
1988.58244
3971.22526
1916.11916
1315.03032
3449.72724
8791.81519
181.752158
2716.77899
12621.6777
1324.5337
1836.52834
10667.545
19949.9722
6211.64729
6791.35352
2526.71138
2170.3346
420.524602
6021.57968
618.907676
4357.30011
11160.5329
5971.68693
1512.22547
1718.92401
2714.40315
2272.49594
1107.14387
1290.08395
21748.487
2107.3747
3860.74846
1309.09071

1267.51342
5829.13621
807.78737
7404.32159
13595.7742
7868.79932
25296.8118
7.12753562
11199.7343
7084.77041
2691.83262
9913.21412
12551.5902
10267.2151
2080.05248
20031.9389
2328.3283
1983.83075
4565.18656
7950.76598
177.000468
1906.61578
3857.18469
3809.66779
507.242952
231.644908
1511.03755
3115.92099
2324.76453
2235.67034
18393.7936
10599.8334
3712.25814
3363.00889
6658.30619
1614.38682
10.6913034
526.249713
239.960366
8707.47268
18751.3583
4282.46099
3619.60017

6722.45401
674.740039
3251.34417
4670.91168
3503.18376
276.785967
5509.58503
2504.14085
457.350202
54102.747
12681.0738
4471.34068
698.498491
17021.743
12096.6159
420.524602
1609.63513
17401.8782
8140.8336
1159.41246
458.538125
1401.74867
2772.61136
9427.35378
2087.18001
1179.60715
839.861281
795.908144
42577.5219
3715.8219
416.960834
1143.96947
2995.94081
1199.80183
3079.09539
167.497087
1208.11729
117.604338
2443.5568
975.284457
16111.7943
850.552584
4699.42182

86313.2684
16470.5469
3466.35816
4831.28123
445.470976
8421.18334
931.331321
168.68501
7026.5622
2664.5104
16769.9034
8087.37708
17006.3
8114.6993
50199.2334
34455.6951
4697.04597
53.4565172
4847.91214
274.410121
1452.82934
279.161812
396.76615
472.793196
62280.4062
17950.6985
137.799022
7324.73077
1121.39894
4090.01752
5191.22178
3808.47987
15216.1006
32916.1474
915.888327
8916.54706
2321.20077
2056.29403
4275.33345
1754.56169
1167.72792
2318.82492
3659.98954

896.881566
1501.53417
559.511546
3157.49828
3311.92822
6206.8956
1298.39941
506.055029
931.331321
11936.2463
2763.10798
3412.90164
522.685945
3354.69343
6663.05788
1086.94918
3946.27889
1162.97623
1497.9704
3525.75429
1141.59362
852.928429
1593.00421
823.230364
2154.8916
3703.94268
949.15016
10545.1889
4404.81701
900.445333
1723.6757
583.269998
411.021221
346.8734
2358.02637
5590.36377
1819.89743
36902.8157
621.283522
4211.18563
31425.3045
283.913502
4580.62956

8535.22391
2323.57661
1437.38635
312.423645
552.384011
544.068552
304.108186
4397.68948
3292.92146
2714.40315
1119.02309
6810.36029
674.740039
9414.28663
14314.4674
3083.84708
599.900915
6304.30526
2508.89254
117.604338
1367.29892
17.8188391
41198.3438
37860.2813
8263.18963
2456.62394
205.51061
198.383075
14707.6698
451.410589
5317.14157
3795.41272
2738.1616
3561.39196
1446.88973
2424.55003
563.075314
143.738635
1781.88391
9199.27264
4331.16581
44709.843
719.881098

9282.42722
2124.00561
913.512482
280.349734
371.819775
383.699001
26263.7808
416.960834
2847.45048
1173.66753
6884.01149
14372.6756
1230.68782
6336.37917
3454.47893
1885.23317
2575.4162
1500.34625
1219.99651
2241.60995
2717.96692
495.363726
775.71346
2023.03219
1306.71486
855.304274
846.988816
2047.97857
862.43181
13082.5916
357.564704
226.893217
22157.1324
17330.6029
10816.0353
2252.30126
3001.88042
2968.61859
6649.99073
1597.7559
2191.7172
1385.11776
804.223602

4415.50832
88304.2267
73576.3623
3572.08327
13026.7593
43220.1881
25744.6587
3488.92869
19043.5873
19761.0925
2769.04759
925.391708
3726.51321
8247.74664
12979.2424
12454.1806
77.2149692
388.450691
11.879226
521.498023
236.396598
154.429938
72.4632788
454.974357
1473.02403
8108.75969
326.678716
1777.13221
23994.8487
3447.35139
1263.94965
3380.82773
1614.38682
1587.0646
22891.2686
7202.37474
7949.57806
73.6512014
3339.25044
2755.98044
8904.66783
2132.32107
6983.79699

10324.2353
5176.96671
1347.10423
5160.33579
1067.94242
197.195152
102.161344
1684.47425
8307.14277
3171.75335
4765.94548
2118.066
4914.43581
13961.6544
48969.7335
520.3101
2609.86596
889.75403
723.444865
2786.86643
5734.10241
4523.60927
2669.26209
2540.96645
2344.95922
14399.9978
2872.39685
3314.30406
10635.4711
5212.60438
1846.03173
2233.29449
10169.8054
3446.16347
1260.38588
879.062726
3935.58758
521.498023
470.417351
5768.55216
21064.2436
1585.87668
3518.62675

24.9463747
1775.94429
8596.99588
7344.92546
83.1545822
453.786434
4472.5286
1780.69598
3316.67991
21591.6812
276.785967
185.315926
643.854051
494.175803
6577.52745
9217.09148
1778.32014
5865.96182
177.000468
33946.0763
1266.3255
131.859409
2443.5568
5007.09377
187.691771
889.75403
2793.99396
5759.04878
1705.85686
3447.35139
4175.54795
2450.68433
9468.93107
1149.90908
781.653073
522.685945
3534.06974
1547.86315
2013.52881
890.941953
1305.52694
5424.05461
3190.76011

6954.09892
1963.63606
786.404763
4183.86341
4420.26001
380.135233
440.719286
2773.79928
20465.5306
10587.9542
144199.549
2165.58291
1799.70274
3440.22386
5402.672
270.846354
1035.86851
906.384946
1211.68106
1815.14574
4370.36726
167.497087
5591.55169
6828.17912
2070.5491
1716.54816
10502.4237
1521.72885
20155.4828
928.955476
602.27676
1341.16462
6115.42556
1242.56704
3841.7417
6487.24534
8833.39248
12991.1216
2524.33553
4073.38661
5984.75408
4206.43394
179.376313

722.256943
7226.1332
99.7854987
5497.70581
4862.16722
1129.7144
2774.9872
432.403828
3778.7818
9792.04602
3292.92146
842.237126
8288.136
9574.65618
3858.37262
2694.20846
4427.38754
6246.09705
1219.99651
9721.95859
1755.74961
5008.2817
552.384011
2437.61718
4209.99771
2928.22922
3680.18423
4973.83194
72.4632788
133428.655
12148.8845
2096.68339
11250.815
239.960366
750.767085
3284.606
4615.07931
1797.3269
1229.49989
6929.15255
3263.22339
4224.25278
3890.44653

944.39847
1362.54723
5272.00051
3022.0751
609.404296
32920.8991
2046.79065
2077.67663
27062.0648
7773.76552
2497.01331
234.020753
5579.67247
3851.24508
49721.6885
3503.18376
1811.58197
595.149224
833.921668
808.975293
5654.51159
8381.98189
17685.7917
851.740507
1800.89067
6147.49947
987.163683
7341.36169
24901.2336
1026.36513
850.552584
683.055497
14674.4079
1096.45256
61522.5116
7128.72354
13466.2906
3075.53162
3517.43883
67.7115884
1598.94382
38008.7716
2153.70368

957.465618
7344.92546
28450.7464
4903.74451
9224.21902
5304.07442
9693.44844
2109.75054
61.7719754
312.423645
4864.54306
58.2082076
2200.03266
5070.05367
4283.64891
4015.1784
10568.9474
856.492197
5146.08072
4884.73774
2475.63071
9603.16633
9580.5958
36811.3456
250.651669
1652.40034
16116.546
1211.68106
5175.77878
1191.48637
2061.04572
3037.5181
5422.86668
5945.55263
7722.68484
2384.16066
9454.676
6285.29849
6328.06371
724.632788
314.79949
664.048735
7024.18635

5099.75174
612.968063
1598.94382
831.545822
6769.97092
18583.8612
854.116352
41633.1235
6558.52069
1879.29356
5981.19031
1754.56169
7537.36892
1747.43415
1875.72979
2759.54421
20887.2431
743.63955
1174.85545
13602.9017
4776.63679
696.122646
4002.11125
2135.88484
610.592218
4075.76245
6260.35212
7747.63122
56909.8082
3212.14272
5073.61744
2151.32783
1382.74191
934.895089
1670.21918
953.90185
870.747268
2822.50411
5591.55169
1880.48148
9529.51512
7047.94481
818.478674

26117.6664
2841.51087
3528.13013
5755.48501
5281.50389
882.626494
2765.48382
1904.23993
2728.65822
7559.93945
5920.60626
879.062726
2009.96504
1545.48731
1044.18397
14944.0664
3383.20357
287.47727
63951.8134
3598.21757
2723.90653
5750.73332
10054.5769
4855.03968
6022.7676
2394.85197
1518.16509
441.907208
10399.0745
3367.76058
1968.38775
1269.88926
24323.9032
784.028918
557.135701
124655.846
2865.26932
17336.5425
3089.78669
2780.92681
551.196088
1806.83028
301.732341

2546.90606
27488.529
5463.25605
1374.42645
1155.84869
9649.49531
29271.6009
6948.15931
30983.3973
3467.54608
8006.59835
20711.4306
817.290751
1626.26604
5909.91495
244.712056
3741.9562
1221.18444
15584.3566
4166.04457
6815.11198
1867.41433
4489.15952
2486.32201
1913.74331
1771.1926
1854.34718
21428.9358
3088.59877
1249.69458
488.23619
7089.5221
1227.12405
4182.67549
18476.9482
3845.30547
1411.25205
2994.75288
8465.13647
3174.1292
3085.035
4118.52767
3378.45188

2464.9394
4520.04551
1499.15833
12891.3361
13859.493
3138.49152
6033.4589
2027.78388
125.919796
4344.23296
3593.46588
21.3826069
2083.61625
5287.44351
3484.177
97.4096535
1554.99069
6295.9898
3074.3437
4983.33532
64.1478206
173.4367
190.067617
86.71835
55.8323624
761.458389
29.6980651
6090.47919
163.933319
4765.94548
1127.33855
27854.4092
3205.01518
380.135233
33.2618329
913.512482
200.75892
263.718818
824.418287
1430.25881
213.826069
715.129407
1019.23759

12393.5965
3863.12431
253.027515
8273.88093
1684.47425
23017.1884
27707.1068
27622.7643
15720.9677
11337.5333
41334.9549
11616.6951
31755.547
25680.5108
20406.1345
22708.3285
10858.8005
15218.4765
15570.1016
58806.9206
14419.0046
31885.0306
18209.6656
34446.1917
18226.2965
18543.4718
10092.5904
59116.9684
33296.2826
39741.9507
74289.1158
63475.4564
21011.975
165.121242
75878.5563
16936.2126
19892.9519
37280.5751
12555.154
54644.4398
20066.3886
19900.0795
42107.1046

22170.1995
6311.43279
14224.1853
15340.8325
57971.811
20485.7253
17009.8638
1379.17814
16586.9633
26204.3847
14547.3002
32214.0852
2138.26069
9044.8427
3140.86736
331.430406
1278.20472
2096.68339
7930.5713
16539.4464
507.242952
895.693643
324.302871
54.6444398
2688.26885
6222.3386
457.350202
4674.47544
7707.24185
2353.27468
1730.80323
40226.6231
4362.0518
96.2217309
6336.37917
1684.47425
3821.54701
1426.69505
8991.38618
6889.9511
12005.1458
3725.32528
18589.8008

7133.47523
3448.53932
12138.1932
857.68012
1042.99605
2943.67221
1746.24623
29620.8501
7104.96509
7731.0003
6750.96415
15060.4828
1803.26651
2845.07464
1376.8023
2391.2882
3310.7403
232.83283
76.0270466
4303.84359
1862.66264
5250.61791
5154.39618
477.544887
1356.60761
4883.54982
64.1478206
1862.66264
636.726515
1793.76313
26171.1229
5219.73192
20757.7596
1993.33413
257.779205
5111.63096
4971.4561
62.959898
4242.07162
49962.8368
3203.82726
1621.51435
1303.1511

3102.85384
489.424113
1305.52694
5157.95994
34.4497555
5372.97393
985.975761
15624.746
1143.96947
3904.7016
810.163215
3244.21663
28.5101425
110.476802
4396.50156
6344.69462
15935.9817
1674.97087
5515.52465
4124.46728
490.612035
4821.77785
446.658899
1350.668
2810.62488
25495.1949
2645.50364
1468.27234
324.302871
4834.845
415.772911
6381.52023
4244.44746
2877.14855
9132.74897
7284.3414
3246.59247
5768.55216
9839.56292
14104.2051
12881.8327
985.975761
1110.70763

4898.99282
7523.11385
11821.0178
1385.11776
61408.4711
2612.2418
4931.06673
13050.5177
362.316394
6225.90236
6949.34723
8874.96977
149.678248
987.163683
330.242484
2595.61089
6299.55357
14686.2871
23757.2641
2975.74612
5168.65125
11937.4342
2000.46166
1170.10376
311.235722
2039.66311
465.665661
9192.1451
1947.00515
4080.51414
3801.35233
5276.7522
2013.52881
4263.45422
756.706698
813.726983
380.135233
10906.3174
893.317798
175.812545
24170.6612
2215.47566
1380.36607

168.68501
3694.4393
3156.31036
5185.28216
2884.27608
334.994174
13479.3578
28904.5328
6284.11057
12011.0854
19203.9568
3346.37797
2675.2017
5302.8865
6415.96998
1328.09747
991.915374
168.68501
555.947778
9565.1528
5606.99469
1920.87085
8624.3181
1151.097
1197.42598
808.975293
4152.97742
2975.74612
16184.2575
1518.16509
4743.37496
2722.71861
128.295641
8991.38618
19370.266
16508.5604
2114.50223
3711.07021
16740.2053
2535.02684
27486.1532
3744.33205
6595.34629

6033.4589
13158.6187
8462.76063
5744.79371
7993.5312
15328.9533
47760.4283
5735.29033
911.136637
700.874336
314.79949
6525.25886
544.068552
1028.74097
11414.7483
970.532767
5470.38359
5337.33626
5776.86762
475.169041
26121.2301
4837.22084
1868.60226
3974.78903
1231.87574
497.739571
19239.5945
2075.30079
3055.33694
1726.05154
2696.58431
2510.08046
567.827004
4919.1875
1817.52158
6836.49458
5209.04062
5115.19473
92.6579631
2959.1152
2564.7249
1897.1124
5882.59273

3018.51134
349.249245
1313.8424
8728.85529
3057.71278
9573.46826
7081.20664
1326.90955
9024.64802
3644.54655
4572.3141
1817.52158
3361.82097
2042.03896
1842.46796
563.075314
3062.46447
597.525069
1379.17814
16616.6614
599.900915
16179.5059
3395.0828
11830.5212
1737.93077
2776.17512
9898.95905
574.95454
1492.03079
810.163215
3393.89488
4656.65661
4972.64402
12419.7308
640.290283
403.893685
14132.7152
4836.03292
2070.5491
10412.1416
734.136169
1652.40034
471.605274

1325.72163
1482.52741
388.450691
779.277228
2264.18048
1970.7636
479.920732
736.512014
608.216373
1001.41875
1688.03802
2998.31665
308.859877
956.277696
1108.33179
820.854519
61.7719754
3547.13689
2036.09934

Gene Set_F	baseMean	log2FoldChai	lfcSE	stat	pvalue	padj
STMN3	2737.43215	0.52606477	0.05025221	10.4684908	1.21E-25	8.17E-24
TMEM238	832.780709	0.92102468	0.09283894	9.92067185	3.38E-23	1.95E-21
NAALADL2	1072.99167	0.37235344	0.06492636	5.7350121	9.75E-09	1.42E-07
FGF12	594.599395	0.33411914	0.09021535	3.70357317	0.00021258	0.00127929
NUPR1	93.7193926	0.74807138	0.21742689	3.44056517	0.0005805	0.00308416
NANOS1	283.753296	0.4250791	0.123488	3.4422706	0.00057685	0.00306836
BTG2	2428.9314	0.91125981	0.04903274	18.5847225	4.27E-77	1.95E-74
ID1	3968.57449	0.78538761	0.05385142	14.5843438	3.53E-48	7.24E-46
MDK	5952.79853	0.80194356	0.03820904	20.9883185	8.39E-98	6.12E-95
PDCD4-AS1	195.932552	0.54844679	0.14141161	3.87837175	0.00010516	0.00067995
IL1R1	176.728138	0.53848257	0.14910569	3.61141518	0.00030453	0.00176224
HIST1H2AD_	178.786391	0.54331317	0.15828533	3.43249223	0.00059806	0.00316272
CEP19	123.011639	0.66851579	0.17865472	3.74194297	0.0001826	0.00111506
CTXN1	1144.14635	0.34750878	0.0687002	5.05833689	4.23E-07	4.60E-06
SKAP2	2387.17443	0.1375124	0.04926286	2.79140103	0.00524804	0.0203645
CAMK1D	697.11482	0.34207005	0.07966063	4.29409172	1.75E-05	0.00013616
RP11-47A8.5	282.440995	0.40978466	0.12973867	3.15853908	0.00158562	0.007344
FAM43A	485.770945	0.84197664	0.11809169	7.12985485	1.00E-12	2.45E-11
CNIH2	407.265083	0.75775446	0.1015556	7.46147401	8.56E-14	2.37E-12
MAPK8IP1	827.173235	0.41549993	0.0732657	5.67113869	1.42E-08	2.00E-07
HIST1H2BG	950.796331	0.40440479	0.07194105	5.62133594	1.89E-08	2.61E-07
ODC1	4446.13493	0.40538119	0.041212	9.83648316	7.84E-23	4.43E-21
HIST1H3E	847.576227	0.43029913	0.07640565	5.63177145	1.78E-08	2.47E-07
EFNB3	882.818437	0.55887804	0.07290021	7.66634354	1.77E-14	5.32E-13
PPP1R3F	509.467079	0.3694963	0.09758101	3.78655946	0.00015275	0.00095188
SESN2	1777.44301	0.62554623	0.0560609	11.1583344	6.52E-29	5.19E-27
PRR7	1625.53016	0.24597278	0.08051166	3.05512008	0.0022497	0.00989176
HOXC13	1099.01073	0.41652613	0.06632779	6.27981355	3.39E-10	6.05E-09
HIST3H2A	1072.1286	0.27213684	0.06464073	4.20999049	2.55E-05	0.00018999
MATN2	1093.48384	0.36391399	0.06545057	5.56013463	2.70E-08	3.62E-07
SAMD11	1691.68955	0.36806703	0.07451612	4.93942832	7.84E-07	8.07E-06
FAM171A2	1007.11569	0.38126423	0.09342598	4.08092294	4.49E-05	0.00031568
BAMBI	12730.7209	0.57297916	0.03344139	17.1338302	8.30E-66	3.03E-63
ID3	4258.67394	0.28093304	0.04967372	5.6555665	1.55E-08	2.17E-07
TRIP6	5411.19873	0.48582493	0.04616004	10.5247955	6.64E-26	4.60E-24
SMAD6	1399.29543	0.26041963	0.06237224	4.17524925	2.98E-05	0.0002185
CYFIP2	2134.34032	0.39877907	0.05929646	6.7251747	1.75E-11	3.72E-10
ID2	4505.26446	0.3433069	0.04134329	8.30381284	1.01E-16	3.68E-15
IGSF9	1204.20479	0.30111696	0.06612807	4.55354225	5.28E-06	4.62E-05
TGFB1	5061.48026	0.3439823	0.04833496	7.11663544	1.11E-12	2.69E-11
DYRK1B	1562.41392	0.16495336	0.06565657	2.51236633	0.01199245	0.04024855
ZNF219	3608.00608	0.44340647	0.06493339	6.82863608	8.57E-12	1.87E-10
METTL7A	380.817124	0.44665018	0.10274015	4.34737693	1.38E-05	0.00010882
TEAD2	2476.52476	0.63891736	0.04785562	13.350936	1.17E-40	1.69E-38

KDM4A	3914.87091	0.4728228	0.04355503	10.8557564	1.87E-27	1.41E-25
CEP126	292.724736	0.36335066	0.1181408	3.075573	0.00210099	0.00932456
THRB	2124.15336	0.53362551	0.05737403	9.30081974	1.39E-20	6.67E-19
JADE2	5334.97321	0.40673156	0.04149033	9.80304526	1.09E-22	6.04E-21
SGPP1	1813.41443	0.40847938	0.05487445	7.44389055	9.78E-14	2.68E-12
ASAP1	4011.35141	0.2913957	0.04329461	6.73053047	1.69E-11	3.60E-10
NACC2	3680.87644	0.34414171	0.04582785	7.50944487	5.94E-14	1.68E-12
DBN1	3831.52418	0.39009454	0.05003926	7.79577013	6.40E-15	2.02E-13
RP11-400F19	897.467902	0.48619753	0.07400154	6.57010037	5.03E-11	1.01E-09
CCDC90B	2623.55576	0.32275706	0.05072308	6.36312026	1.98E-10	3.66E-09
ARHGAP39_	4303.18472	0.25384636	0.05772896	4.39720986	1.10E-05	8.90E-05
TTYH3	4143.80323	0.44400676	0.05217366	8.51017019	1.74E-17	6.75E-16
CADPS2	1176.6943	0.40047313	0.0642821	6.22993232	4.67E-10	8.17E-09
HIST1H2AC	2596.8453	0.25412655	0.05311986	4.78402199	1.72E-06	1.65E-05
H2AFX	10303.9132	0.45564573	0.09666842	4.71349099	2.44E-06	2.27E-05
TCAIM	1516.7276	0.26507371	0.06465967	4.0995214	4.14E-05	0.00029425
MARCKSL1	9413.87576	0.24494961	0.03747713	6.53597538	6.32E-11	1.25E-09
TNRC18	16658.7509	0.34088585	0.04352284	7.83234319	4.79E-15	1.54E-13
PODXL2	2042.49436	0.47888593	0.05221506	9.17141373	4.67E-20	2.16E-18
FRAT2	2335.07736	0.42541842	0.0603833	7.04529916	1.85E-12	4.39E-11
TEF	1138.12446	0.28916318	0.06961088	4.15399407	3.27E-05	0.00023844
MXD4	3612.59639	0.24815856	0.05598354	4.43270595	9.31E-06	7.67E-05
USP54	1722.6056	0.15369772	0.05842728	2.63058134	0.0085239	0.03052332
CADM4	524.888545	0.25896809	0.08907587	2.9072756	0.00364592	0.01489908
STAT6	3566.01883	0.65119266	0.04317041	15.0842365	2.06E-51	4.87E-49
CDKN1B	6099.07779	0.3036416	0.03786602	8.01884201	1.07E-15	3.67E-14
TBC1D4	989.559831	0.19891819	0.07238043	2.74823178	0.00599176	0.02269502
MESDC1	3720.40314	0.37457197	0.06228521	6.01381918	1.81E-09	2.95E-08
TCAF1	5767.15667	0.14422045	0.03984727	3.61933058	0.00029537	0.00171683
UBE2S	3980.90044	0.41635619	0.04605709	9.04000252	1.57E-19	7.04E-18
PBXIP1	2935.64665	0.4703975	0.04482785	10.4934206	9.26E-26	6.32E-24
STK17A	1697.90252	0.23306044	0.06160111	3.78338045	0.00015471	0.00096284
LIMK1	2574.96917	0.28468879	0.05000142	5.693614	1.24E-08	1.77E-07
TARS	7189.11563	0.33604724	0.03539503	9.49419267	2.22E-21	1.13E-19
ARHGEF18_	1435.42717	0.45584988	0.06559221	6.94975698	3.66E-12	8.32E-11
TLE3	6908.87907	0.29297737	0.03805186	7.69942412	1.37E-14	4.17E-13
POLR1B	3006.19967	0.2807372	0.05066544	5.54100013	3.01E-08	4.00E-07
SLC35D2	1533.20482	0.17266163	0.0634261	2.72224901	0.00648393	0.0242718
HSPB1	84885.8757	0.2102958	0.0403505	5.2117274	1.87E-07	2.16E-06
HIST2H2AA3_	5374.72809	0.13584308	0.04106062	3.30835419	0.00093846	0.00466518
MAGED2	13315.4583	0.34330211	0.04035753	8.50651875	1.79E-17	6.92E-16
SLC37A1	4198.06195	0.29479963	0.04206521	7.00815704	2.41E-12	5.60E-11
SOX12	4989.10712	0.16420422	0.0448128	3.66422598	0.00024809	0.00147062
NCOA3	80110.8538	0.16009974	0.03959491	4.04344235	5.27E-05	0.00036463
QSOX2	2170.92821	0.16346171	0.04962226	3.29412052	0.0009873	0.00486596

DIP2B	6522.96516	0.17270276	0.04363952	3.95748546	7.57E-05	0.00050681
ANKRD40	2999.29588	0.25265465	0.04456941	5.66879023	1.44E-08	2.03E-07
PPFIA3	1707.80477	0.15535415	0.06287513	2.47083621	0.01347975	0.04418297
SERTAD1	985.206238	0.31629377	0.0747561	4.23100962	2.33E-05	0.00017479
CRTAP	9472.50553	0.25703958	0.0381808	6.73216762	1.67E-11	3.56E-10
VGLL4	5081.31663	0.33085754	0.04161138	7.95113128	1.85E-15	6.25E-14
BLVRA	3611.26309	0.27279934	0.04229414	6.45005103	1.12E-10	2.14E-09
ATF4	8708.21818	0.4503677	0.03671538	12.2664576	1.37E-34	1.48E-32
KLF16	1146.96335	0.2183753	0.0640396	3.41000423	0.00064962	0.00339695
BRPF3	3707.81673	0.21498809	0.0429973	5.00003692	5.73E-07	6.09E-06
PTMS	6557.86294	0.16468045	0.03851074	4.27622144	1.90E-05	0.00014594
SH3BP5L	1657.51557	0.32440815	0.05984687	5.42063699	5.94E-08	7.46E-07
PAK2	15561.2246	0.18395943	0.03171318	5.80072488	6.60E-09	9.91E-08
EIF4G3	7212.89285	0.16456298	0.04256754	3.86592629	0.00011067	0.00071179
PKP3	5338.21429	0.20922496	0.05437171	3.84804853	0.00011906	0.00076015
SUMO3	14050.9059	0.22959471	0.03191108	7.19482774	6.25E-13	1.56E-11
MADD	5707.76846	0.32563901	0.04961228	6.56367831	5.25E-11	1.05E-09
DAB2IP	2641.34709	0.38734616	0.04949795	7.82549947	5.06E-15	1.62E-13
PLEKHH3	2482.4035	0.22213575	0.06937922	3.20176183	0.0013659	0.00645897
PTK7	3226.54011	0.23334507	0.05135426	4.54383115	5.52E-06	4.81E-05
CLSTN3	1163.9187	0.23219352	0.06404938	3.62522642	0.00028871	0.00168243
TM7SF2__VI	9664.92065	0.25281371	0.04414117	5.72738967	1.02E-08	1.48E-07
CRY2	1910.15992	0.19520494	0.05414487	3.60523428	0.00031187	0.00179787
CCDC106__ZI	3808.38286	0.19291982	0.04740929	4.06924078	4.72E-05	0.00033028
PCDHA1__PC	6134.83615	0.21662843	0.0377714	5.73525011	9.74E-09	1.42E-07
SNX18	1438.32008	0.20697859	0.06335273	3.26708226	0.00108662	0.00527655
DECR2__NM	6368.74173	0.23954956	0.04172806	5.74073079	9.43E-09	1.38E-07
ADGRG1	9009.89357	0.18768406	0.04359983	4.30469701	1.67E-05	0.00013052
JUP	27260.9042	0.27542209	0.04298985	6.40667706	1.49E-10	2.80E-09
ANAPC7__AI	14577.2335	0.21210301	0.03301758	6.42394107	1.33E-10	2.52E-09
YAP1	2953.31425	0.17773909	0.04591433	3.87110254	0.00010834	0.00069881
NACC1	8686.56544	0.24826365	0.04304416	5.76765015	8.04E-09	1.19E-07
BSDC1	3149.98573	0.19082005	0.04607129	4.14184328	3.45E-05	0.00024998
LSS	7421.9918	0.21028018	0.04108871	5.11771162	3.09E-07	3.45E-06
PSAP	29097.2373	0.24181413	0.03060595	7.90088737	2.77E-15	9.20E-14
SRPK2	5395.0215	0.14924109	0.03829133	3.89751672	9.72E-05	0.00063378
MLXIP	5917.83805	0.24680351	0.04176206	5.9097539	3.43E-09	5.37E-08
IRAK1	16873.0768	0.12390239	0.03956658	3.13149102	0.00173921	0.00794607
PHF20	2160.88656	0.14921749	0.05083901	2.93509812	0.00333442	0.01380873
SEZ6L2	4556.46655	0.19398187	0.04518721	4.29284906	1.76E-05	0.00013672
PCNT	7075.2014	0.12565198	0.04553822	2.75926432	0.00579317	0.02210812
MARK2	4830.40965	0.22323813	0.0392234	5.6914533	1.26E-08	1.79E-07
SETD7	9686.40083	0.17224401	0.03960378	4.34918161	1.37E-05	0.000108
MAF1	6413.0171	0.2291231	0.04084881	5.60905149	2.03E-08	2.79E-07
MCFD2	7138.50119	0.18812761	0.03777551	4.98014793	6.35E-07	6.68E-06

RMND5A	4135.46907	0.15489737	0.04261207	3.63505895	0.00027792	0.00162578
PPP2R1A	14977.2579	0.24022823	0.03808249	6.30810158	2.82E-10	5.09E-09
CNNM4	3221.10002	0.12730527	0.04481436	2.84072492	0.00450111	0.01783814
C7orf50	6911.4472	0.14705279	0.0402231	3.65592896	0.00025625	0.00151214
NFE2L1	18414.5214	0.28489023	0.03650405	7.80434555	5.98E-15	1.90E-13
PRMT2	4827.90828	0.15729401	0.03852774	4.08261706	4.45E-05	0.00031399
UBE3C	7644.90302	0.13938069	0.03672	3.79577074	0.00014719	0.00092163
AC002310.11	7152.23356	0.13709055	0.0377246	3.63398276	0.00027908	0.00163205
PTP4A1	20104.668	0.07862223	0.03052667	2.57552587	0.01000878	0.03483491
CCDC85C	8610.35786	0.09011568	0.03584661	2.51392481	0.01193959	0.04012283
KIAA0391__F	16143.4972	-0.1501098	0.03641725	-4.1219419	3.76E-05	0.00027013
AZIN1	18829.6348	-0.1394693	0.03034919	-4.5954869	4.32E-06	3.83E-05
TPBG	13147.3035	-0.1318656	0.04201373	-3.1386316	0.00169739	0.00778229
MORF4L2	24841.6368	-0.1398977	0.03502862	-3.9938125	6.50E-05	0.00044106
BUB3	11531.7227	-0.1965049	0.03480183	-5.646396	1.64E-08	2.29E-07
SSBP1	4298.16134	-0.1555519	0.05262426	-2.9558976	0.00311761	0.0130445
ABCD3	1786.62906	-0.1691228	0.05442289	-3.1075668	0.00188634	0.00851631
CTC-432M15	3883.45918	-0.1329852	0.04226941	-3.1461342	0.00165444	0.0076141
PLP2	3349.8027	-0.2705881	0.04961213	-5.4540706	4.92E-08	6.28E-07
DDAH1	2356.13123	-0.2438161	0.05370025	-4.5403163	5.62E-06	4.88E-05
DARS	5479.42522	-0.2253562	0.03819562	-5.9000522	3.63E-09	5.68E-08
WDR76	1008.23587	-0.178937	0.06971345	-2.5667494	0.01026568	0.03555933
NARF	2788.81479	-0.1561282	0.04646118	-3.3604017	0.00077829	0.0039769
XRN1	1957.36531	-0.1655247	0.06118369	-2.7053731	0.00682277	0.02536748
NR2C2	4867.01266	-0.2600684	0.04424086	-5.8784658	4.14E-09	6.42E-08
SRSF11	7305.90031	-0.3267958	0.03829829	-8.5329078	1.43E-17	5.57E-16
E2F2	1170.35635	-0.1688609	0.06737726	-2.5062	0.01220366	0.04081465
PFKP	15726.3515	-0.1425912	0.03369386	-4.2319648	2.32E-05	0.00017412
MT-ND6	137358.157	-0.2506292	0.03901398	-6.4240869	1.33E-10	2.52E-09
GK5	1439.76656	-0.3393215	0.06024261	-5.6325822	1.78E-08	2.46E-07
RBL1	1243.09841	-0.1885707	0.0657525	-2.8678861	0.00413224	0.01658171
PLEKHA1	1759.5833	-0.1530255	0.05492633	-2.7860127	0.00533608	0.02067532
SEMA3F	3312.36532	-0.2584699	0.04718079	-5.4782861	4.29E-08	5.52E-07
GPI__PDCD2I	27506.0599	-0.253067	0.03367664	-7.5146165	5.71E-14	1.61E-12
CFL2	833.844135	-0.1846794	0.0728121	-2.5363839	0.01120039	0.03813068
GAPDH	111152.661	-0.2792176	0.02888192	-9.6675582	4.14E-22	2.21E-20
KNTC1	4540.28364	-0.1921428	0.04613804	-4.1645197	3.12E-05	0.00022839
PLEKHH1	2825.32634	-0.3633983	0.0465836	-7.8009925	6.14E-15	1.94E-13
PRSS53__RP	2675.65074	-0.2375303	0.05536773	-4.2900486	1.79E-05	0.00013819
SLC25A36	4913.46313	-0.294533	0.03930993	-7.4925857	6.75E-14	1.88E-12
HNRNPH1	29288.1608	-0.3687639	0.03674176	-10.036643	1.05E-23	6.19E-22
PREX1	17242.2645	-0.3811367	0.03904715	-9.7609343	1.66E-22	8.99E-21
ENO1	81297.799	-0.2271108	0.03552144	-6.3936263	1.62E-10	3.03E-09
BBX	4468.3274	-0.2541479	0.04401661	-5.7739093	7.75E-09	1.15E-07
SOD2	3427.78287	-0.4657054	0.04486856	-10.379326	3.08E-25	2.02E-23

ZBTB25	2053.52	-0.3173693	0.05605363	-5.6618867	1.50E-08	2.10E-07
BNIP3L	4561.29523	-0.4231573	0.04722962	-8.9595757	3.26E-19	1.44E-17
DCUN1D3__I	3058.69961	-0.1627349	0.04853017	-3.3532716	0.00079862	0.00406422
IL6ST	1777.45045	-0.3508416	0.0536654	-6.5375744	6.25E-11	1.24E-09
IFFO1	40146.0595	-0.3437468	0.09509496	-3.6147741	0.00030061	0.00174453
HELLS	3549.61738	-0.126145	0.05203292	-2.4243299	0.01533667	0.04897526
SNHG1__SN	4541.63844	-0.4141483	0.04866702	-8.5098347	1.74E-17	6.76E-16
ZNF292	3165.3872	-0.3493771	0.04641222	-7.5276972	5.16E-14	1.47E-12
MAL2	17203.8869	-0.3408973	0.03617901	-9.4225177	4.40E-21	2.18E-19
CP	572.012244	-0.2635463	0.08569531	-3.0753871	0.0021023	0.00932456
FBXL20	764.808868	-0.2595894	0.07700719	-3.3709755	0.00074903	0.00384616
AF011889.5_	7945.95149	-0.5221348	0.03772039	-13.842242	1.42E-43	2.33E-41
PIGA	896.597156	-0.289919	0.06996733	-4.1436343	3.42E-05	0.00024834
QSOX1	7307.45992	-0.3491704	0.03680713	-9.4864879	2.39E-21	1.21E-19
SLC9A8	2063.32748	-0.3369503	0.05079424	-6.6336324	3.28E-11	6.76E-10
NKTR	5060.34546	-0.4547944	0.05196701	-8.7515992	2.10E-18	8.68E-17
HSD17B12	1126.76705	-0.3473562	0.06404555	-5.4235801	5.84E-08	7.37E-07
CCNL1	7033.19757	-0.5782322	0.03560722	-16.239182	2.66E-59	8.30E-57
AGR3	810.264182	-0.2080762	0.07526463	-2.7645948	0.00569936	0.02181861
CRIM1	1458.00056	-0.237433	0.0594038	-3.9969321	6.42E-05	0.00043561
NPIPB5	1842.66241	-0.5271231	0.05251961	-10.03669	1.05E-23	6.19E-22
FUT11	767.640858	-0.2317564	0.08348747	-2.7759426	0.00550419	0.02119161
ZGRF1	979.341082	-0.3050572	0.07123318	-4.2825151	1.85E-05	0.00014223
ZNF37BP	1188.29747	-0.5140727	0.065892	-7.8017464	6.11E-15	1.93E-13
FAM217B	2788.65857	-0.1907328	0.04733069	-4.0297913	5.58E-05	0.00038442
CCDC125	2284.37559	-0.433593	0.05791339	-7.486921	7.05E-14	1.96E-12
AK4	3400.52334	-0.2719289	0.04402914	-6.1761119	6.57E-10	1.13E-08
TACC1	1945.40926	-0.3949623	0.05804325	-6.8046207	1.01E-11	2.20E-10
RDX	15100.9577	-0.6582099	0.03134137	-21.001312	6.38E-98	4.85E-95
TMEM167A	6070.62018	-0.2473162	0.04042858	-6.1173611	9.51E-10	1.60E-08
FAM162A	1944.17966	-0.4293394	0.05560673	-7.7209971	1.15E-14	3.53E-13
HLA-F	406.222193	-0.2737087	0.10382278	-2.6363071	0.00838139	0.03011342
STAT1	13497.0259	-0.5390268	0.03285999	-16.403744	1.80E-60	5.85E-58
CA12	9050.98952	-0.6014816	0.03347769	-17.966641	3.56E-72	1.51E-69
ATAD5	752.009297	-0.4949922	0.07683299	-6.4424437	1.18E-10	2.24E-09
LRRC58	6504.87901	-0.2669487	0.03735772	-7.1457428	8.95E-13	2.19E-11
STC2	34978.587	-0.2242844	0.02959681	-7.5779951	3.51E-14	1.02E-12
KCNK5	883.843751	-0.5758099	0.07509063	-7.6681993	1.74E-14	5.26E-13
PGR	7584.41299	-0.3967383	0.04387385	-9.0427052	1.53E-19	6.90E-18
PGK1	28060.9843	-0.3724471	0.0306631	-12.146427	5.99E-34	6.24E-32
P4HA1	3480.88076	-0.4979172	0.04628532	-10.757562	5.46E-27	4.01E-25
ALDOA	95839.8394	-0.5453123	0.0370497	-14.718399	4.91E-49	1.03E-46
CCBL1	184.802468	-0.4651274	0.15454185	-3.0097178	0.00261491	0.01123473
PBX2	3654.98995	-0.2053551	0.05057202	-4.0606461	4.89E-05	0.00034202
BHLHE40	3318.14033	-0.3491991	0.044238	-7.8936458	2.93E-15	9.69E-14

CLSTN2	1291.39701	-0.5733086	0.06939625	-8.2613771	1.44E-16	5.19E-15
BCL6	639.677651	-0.7068502	0.08482117	-8.3334176	7.85E-17	2.88E-15
SFXN3	1638.78683	-0.4406395	0.05622542	-7.8370163	4.61E-15	1.49E-13
ZBTB37	1214.81574	-0.371431	0.06875692	-5.4020884	6.59E-08	8.22E-07
CYP4V2__KLI	325.200196	-0.2703225	0.11021242	-2.452741	0.01417724	0.04591612
DUSP4	4406.69219	-0.2239591	0.03988146	-5.6156197	1.96E-08	2.69E-07
SCARA3	774.29051	-0.2823402	0.08688071	-3.2497456	0.00115508	0.0055778
LDHAP4	312.264552	-0.4828267	0.11523932	-4.1897739	2.79E-05	0.00020597
C1orf21	3446.24086	-0.4240895	0.04337867	-9.7764533	1.42E-22	7.73E-21
PRSS23	11422.7922	-0.6809301	0.03470501	-19.620514	1.03E-85	6.28E-83
LDHA	27760.003	-0.657292	0.03205582	-20.504609	1.96E-93	1.32E-90
GPR37L1	1148.38459	-0.2791475	0.06333075	-4.4077718	1.04E-05	8.50E-05
PHLDA1	4968.48956	-0.4837876	0.04162758	-11.621804	3.19E-31	2.99E-29
ALDOC	749.38081	-0.5425046	0.07629789	-7.1103495	1.16E-12	2.80E-11
PAPSS2	5094.66307	-0.480036	0.04000089	-12.000633	3.53E-33	3.55E-31
FOS	17253.108	-0.7292092	0.0372962	-19.551838	3.98E-85	2.34E-82
LAMB3	629.923311	-0.9082621	0.08840427	-10.273962	9.23E-25	5.81E-23
KRT15	814.379877	-0.7336456	0.07477122	-9.8118713	1.00E-22	5.55E-21
BNIP3	5036.81991	-0.8704427	0.03940234	-22.091144	3.85E-108	3.69E-105
EFEMP1	670.77593	-1.1723336	0.0815121	-14.382326	6.68E-47	1.27E-44
FHL2	340.462758	-0.7950149	0.11047919	-7.1960607	6.20E-13	1.55E-11
WISP2	1293.25927	-1.132114	0.06695892	-16.907591	3.96E-64	1.36E-61
PFKFB4__UC	666.315404	-0.9770156	0.0851997	-11.46736	1.92E-30	1.69E-28
MSS51	78.660822	-0.8507772	0.22851038	-3.7231447	0.00019676	0.0011931
EGR1	8385.21649	-0.9315206	0.03416498	-27.265366	1.09E-163	1.99E-160
PDK1	828.336024	-0.9684479	0.07401313	-13.084812	4.02E-39	5.47E-37
PRKAA2	76.207868	-0.7339746	0.22755261	-3.2255161	0.00125746	0.00602114
CD109	967.825345	-0.5209039	0.07016562	-7.4239195	1.14E-13	3.10E-12
SPAG4	309.013147	-1.1555124	0.12354169	-9.3532186	8.50E-21	4.15E-19
DDX60	476.10267	-1.6087077	0.10412138	-15.450311	7.51E-54	2.01E-51
IFI6	6364.4924	-1.4470091	0.04346924	-33.288121	5.74E-243	2.61E-239
IFI27	6009.7513	-1.397802	0.03784726	-36.932717	1.38E-298	2.52E-294
OAS1	803.864435	-0.9328525	0.07484762	-12.463356	1.18E-35	1.36E-33
SCNN1G	41.2349822	-1.0227778	0.32383899	-3.1582912	0.00158697	0.0073481
FOSB	1787.48831	-1.9927567	0.05870585	-33.94477	1.46E-252	8.86E-249
OAS2	425.967035	-1.672458	0.11230819	-14.891683	3.73E-50	8.10E-48
PPFIA4	935.112111	-2.2942784	0.08288942	-27.678783	1.26E-168	2.55E-165
IFI44	111.561328	-3.3550208	0.24650636	-13.610281	3.48E-42	5.42E-40
UBE2T	1873.20292	-2.2146889	0.06337759	-34.944352	1.58E-267	1.44E-263

Control.0	Control.1	Control.2	shRNA1.0	shRNA1.1	shRNA1.2	Gene Set_F
2173.24565	2195.79589	2366.00486	3325.6383	3168.24604	3195.66217	STMN3
548.799405	557.675039	623.385664	934.649337	1118.47703	1213.69778	TMEM238
943.934977	925.756431	936.866409	1182.86409	1209.28958	1239.23856	NAALADL2
561.771028	498.179125	518.494768	714.990269	656.074348	618.086832	FGF12
68.8493799	85.674117	53.6373898	120.812488	105.63909	127.703891	NUPR1
248.456458	226.084475	252.691703	351.45451	292.824144	331.008485	NANOS1
1659.36984	1664.29905	1737.85143	3200.43263	3120.05979	3191.57564	BTG2
2865.73071	2851.83751	3026.34073	5365.17275	4625.88015	5076.48507	ID1
4357.46728	4313.05717	4348.2044	7763.84978	7466.08901	7468.12354	MDK
147.676931	165.002003	164.487996	241.624976	227.958036	228.845372	PDCD4-AS1
153.663834	134.064128	145.416924	203.184639	218.691449	205.347857	IL1R1
149.672565	136.443964	151.376634	177.923846	213.131497	244.169839	HIST1H2AD_
101.777344	93.6069056	89.3956497	161.449416	152.898683	138.941833	CEP19
980.85421	959.867422	1083.47528	1336.62543	1255.62252	1248.43324	CTXN1
2245.08848	2310.82132	2264.68979	2432.72419	2577.96446	2491.75832	SKAP2
620.642236	621.930627	601.930708	764.413559	822.872911	750.898878	CAMK1D
250.452092	245.123168	232.428689	269.082359	341.010395	356.549263	RP11-47A8.5
322.294923	334.763679	387.381149	744.644243	541.168671	584.373005	FAM43A
314.312387	283.200554	312.288803	508.510744	522.635497	502.642514	CNIH2
704.458873	695.705561	727.084618	966.499902	909.978827	959.311628	MAPK8IP1
835.172913	816.283948	804.560848	1010.43172	1137.0102	1101.31836	HIST1H2BG
3707.88835	3908.48495	3858.31624	5110.36823	5013.22348	5078.52833	ODC1
683.504714	698.878677	786.681718	997.252172	961.871713	957.268366	HIST1H3E
705.45669	746.475408	687.750532	1013.7266	1055.46424	1088.03715	EFNB3
457.998049	428.370585	446.978249	639.20789	525.415473	558.832226	PPP1R3F
1343.06182	1394.58424	1457.74506	2206.47535	2097.95526	2164.83636	SESN2
1418.89592	1400.93047	1643.68801	1958.2606	1536.4001	1795.00589	PRR7
909.011378	919.4102	998.847393	1245.46692	1256.54917	1264.77934	HOXC13
972.871673	961.45398	979.776321	1196.04363	1151.83674	1170.78927	HIST3H2A
965.886953	985.252346	915.411453	1211.41976	1232.45605	1250.4765	MATN2
1413.90683	1356.50685	1665.14297	2001.09412	1745.82496	1967.66155	SAMD11
764.327899	851.188218	1010.76681	1236.68056	1010.98462	1168.74601	FAM171A2
10126.8458	10190.4603	10390.1584	15550.7638	15039.6704	15086.4269	BAMBI
3830.61985	3743.48295	3963.20714	5012.61995	4530.4343	4471.67944	ID3
4402.36905	4415.39014	4710.55477	6629.31069	6021.42812	6288.13959	TRIP6
1255.25391	1247.03437	1319.47979	1494.77996	1465.04738	1614.17718	SMAD6
1768.1319	1880.0709	1874.92476	2339.36908	2313.86673	2629.67852	CYFIP2
3921.4212	4021.92383	3969.16685	5201.52675	4958.55061	4958.99749	ID2
1041.72105	1055.06089	1141.88043	1361.88623	1264.8891	1359.79103	IGSF9
4287.62008	4382.07243	4716.51448	5680.38352	5382.96029	5919.33075	TGFB1
1460.80424	1404.10358	1556.67625	1690.27653	1519.72024	1742.9027	DYRK1B
2923.6041	2904.98719	3348.16507	4494.22455	3746.48106	4230.5745	ZNF219
327.284009	332.383843	306.329093	430.531775	442.942851	445.431171	METTL7A
1893.85686	1953.84584	1963.12847	3008.23095	2988.47425	3051.61218	TEAD2

3290.8008	3300.04006	3245.65806	4398.67285	4544.33418	4709.7195	KDM4A
234.487019	264.161861	269.378891	339.373261	316.91727	332.030116	CEP126
1787.09043	1753.14628	1666.33491	2340.46738	2623.37073	2574.51044	THRB
4561.02197	4589.91149	4614.00747	5857.20907	6056.64115	6331.04809	JADE2
1500.71692	1592.90395	1580.51509	2066.99184	2016.4093	2122.94948	SGPP1
3576.17649	3637.97686	3609.20037	4248.20639	4445.1817	4551.36667	ASAP1
3151.1064	3249.27022	3333.86176	3972.53426	4106.02463	4272.46137	NACC2
3234.92304	3183.42807	3536.4919	4513.99386	4187.57059	4332.73761	DBN1
757.343179	756.788034	727.084618	1048.87205	983.184863	1111.53467	RP11-400F19
2256.06446	2325.89362	2412.4906	2986.26504	2784.60934	2976.01147	CCDC90B
3787.71371	3804.56542	4186.10029	4992.85063	4253.36336	4794.51488	ARHGAP39_
3477.3926	3407.92599	3649.72639	4959.90177	4400.70209	4967.17054	TTYH3
992.828015	1020.94989	1029.83789	1298.1851	1317.70865	1400.65628	CADPS2
2311.94222	2490.10235	2298.06417	2955.51277	2706.77001	2818.68028	HIST1H2AC
8238.9758	8369.88527	9462.82751	12801.7305	10774.2605	12175.7998	H2AFX
1340.06837	1401.72375	1390.99631	1749.58448	1705.97864	1512.01407	TCAIM
8521.35804	8503.94939	8826.33048	10575.4859	10003.2805	10052.8503	MARCKSL1
14663.9201	14046.5888	15395.1228	18415.118	17801.1133	19630.6421	TNRC18
1666.35456	1695.23693	1759.30639	2441.51055	2330.54659	2362.01117	PODXL2
2027.56435	1903.07599	2050.14023	2856.66619	2447.30558	2725.71185	FRAT2
987.838929	1032.84908	1053.67673	1170.78284	1237.08934	1346.50983	TEF
3222.94923	3263.54924	3420.87353	4176.81719	3562.07598	4029.31316	MXD4
1635.42223	1707.13611	1547.14071	1741.89642	1857.95066	1846.08745	USP54
464.982769	464.861413	505.383407	567.818693	568.041773	578.243218	CADM4
2734.01886	2787.58192	2803.44758	4255.89446	4400.70209	4414.4681	STAT6
5416.15122	5524.39399	5435.2555	6909.37601	6673.79584	6635.49417	CDKN1B
946.928428	943.208566	872.501541	1000.54706	1045.271	1128.9024	TBC1D4
3229.93395	3072.36903	3419.68159	4576.5967	3758.52762	4265.30996	MESDC1
5623.69718	5497.42251	5314.86936	5934.08974	6181.74008	6051.12117	TCAF1
3346.67856	3328.5981	3559.1388	4680.93475	4374.75564	4595.29681	UBE2S
2484.56458	2475.82333	2422.02614	3376.15989	3417.51722	3437.78874	PBXIP1
1506.70382	1692.85709	1478.00808	1829.76004	1812.54438	1867.5417	STK17A
2329.90293	2271.15738	2364.81292	2952.21788	2725.30319	2806.42071	LIMK1
6409.97705	6397.79402	6256.50354	7997.78669	8021.15756	8051.47491	TARS
1215.34123	1192.29813	1224.12443	1633.16518	1547.52	1800.11405	ARHGEF18__
6357.09275	6007.50082	6268.42296	7681.47763	7523.54185	7615.23842	TLE3
2608.2939	2713.80698	2824.90253	3145.51786	3300.75823	3443.91853	POLR1B
1421.88937	1524.68197	1373.11718	1729.81517	1584.58635	1565.13889	SLC35D2
78081.1837	76099.2412	81953.1639	95618.6909	86539.7278	91023.2469	HSPB1
4923.22957	5238.02032	5204.01876	5656.22102	5710.07081	5516.80809	HIST2H2AA3_
11245.3987	11807.1626	12163.7681	15260.8138	14256.6438	15158.9627	MAGED2
3707.88835	3783.14689	3821.36604	4564.51545	4542.48087	4768.9741	SLC37A1
4626.87789	4733.49497	4754.65662	5284.99719	4998.39694	5536.21908	SOX12
75929.8901	76067.51	75016.0615	78842.2295	87798.1303	87011.3015	NCOA3
2096.41373	2027.62077	2020.34168	2280.06113	2305.5268	2295.60514	QSOX2

6211.41145	6022.57311	6169.49177	6488.72889	7117.66535	7127.92037	DIP2B
2746.99048	2778.06257	2684.25338	3282.80478	3259.98525	3243.67883	ANKRD40
1551.60559	1537.37443	1762.88221	1853.92254	1733.7784	1807.26546	PPFIA3
864.109609	861.500843	907.067859	1182.86409	1003.57135	1092.12368	SERTAD1
8448.51739	8526.1612	8920.4939	10636.9904	10057.9534	10244.9169	CRTAP
4377.42362	4440.77507	4689.09981	5753.9693	5572.92532	5653.70666	VGLL4
3298.78333	3218.33234	3296.91156	4007.67971	3929.95948	3915.91211	BLVRA
7269.09758	7616.27035	7187.41024	10117.4967	10074.6332	9984.401	ATF4
1048.70577	1070.13318	1060.82838	1282.80896	1195.3897	1223.91409	KLF16
3413.5323	3393.64697	3491.19811	3922.01267	3931.81279	4094.69756	BRPF3
6323.16697	6020.98656	6212.40169	6775.38397	7042.60599	6972.63244	PTMS
1473.77586	1452.4936	1488.73555	1961.55548	1734.70506	1833.82787	SH3BP5L
14360.5837	14668.5194	14681.1496	16657.8455	16332.3593	16666.8902	PAK2
6789.14755	6919.77151	6696.33014	7196.03109	7723.70013	7952.37669	EIF4G3
4894.29288	4712.07644	5251.69644	6179.0096	5328.28743	5663.92297	PKP3
12817.9585	12982.8018	13005.2791	15264.1087	15300.0615	14935.2254	SUMO3
4849.39111	4995.27699	5358.97122	6065.88518	6216.95311	6760.13317	MADD
2292.9837	2219.59425	2357.66127	2940.13663	2931.94807	3105.75863	DAB2IP
2263.04918	2095.84275	2519.76538	2896.20482	2430.62573	2688.93313	PLEKHH3
2919.61284	2880.39555	3102.62502	3367.37352	3387.86415	3701.36957	PTK7
1071.65557	1040.78187	1101.35441	1216.91124	1258.40249	1294.40664	CLSTN3
8564.26417	8638.01352	9261.38931	10805.0296	9927.29447	10793.5329	TM7SF2__VI
1715.2476	1805.50269	1823.67126	1973.63673	2051.62233	2091.27892	CRY2
3531.27472	3509.46568	3621.11979	4232.83025	3826.1737	4129.43302	CCDC106__Z
5723.47889	5578.33695	5724.89741	6668.84933	6451.39775	6662.05658	PCDHA1__PC
1421.88937	1300.97733	1283.72153	1465.12599	1571.61313	1586.59314	SNX18
5974.9288	5676.70353	5876.27404	6910.4743	7152.87838	6621.19133	DECR2__NM
8198.0653	8256.44639	8823.9466	9410.1945	9264.73352	10105.9751	ADGRG1
23748.047	24161.6876	26093.9942	30644.6367	28253.8233	30663.2366	JUP
13333.8299	13841.1296	13343.7907	15504.6354	15816.2104	15623.8048	ANAPC7__AI
2767.94464	2823.27947	2722.39552	3067.53889	3214.57897	3124.14799	YAP1
7983.53462	7571.05345	8273.2694	9798.99105	9071.06185	9421.48225	NACC1
2862.73726	3020.8059	2939.32896	3384.94625	3265.5452	3426.5508	BSDC1
6774.18029	6785.70738	7088.47905	7984.60715	7642.15416	8256.82277	LSS
26767.4415	26347.9641	26881.8679	30977.4202	31510.1019	32098.6284	PSAP
5083.87813	5120.61505	5145.6136	5655.12272	5788.83679	5576.06269	SRPK2
5481.00933	5333.21378	5426.91191	6163.63347	6391.16494	6711.09487	MLXIP
15872.2766	15867.9571	16708.6429	17980.1931	16703.0228	18106.3685	IRAK1
2006.61019	2049.83258	2093.05015	2206.47535	2285.14031	2324.21081	PHF20
4172.87111	4153.60812	4429.25646	4800.64895	4712.0594	5070.35528	SEZ6L2
6755.22177	6698.4467	6851.2826	6911.5726	7375.27646	7859.40826	PCNT
4553.03943	4422.52965	4395.88208	5233.37731	5174.46209	5203.16733	MARK2
9127.03302	9110.80772	9090.94161	9718.81549	10449.93	10620.8772	SETD7
5863.17328	5844.87865	6006.19572	7213.60382	6655.26266	6894.98848	MAF1
6451.88537	6820.61165	6747.58364	7536.50265	7585.62798	7688.79586	MCFD2

3887.49542	4055.24154	3792.75943	4369.01888	4346.95588	4361.34328	RMND5A
13471.5287	13469.0818	14262.7779	16422.8103	15612.3455	16625.0033	PPP2R1A
3010.41419	3083.47493	3144.34299	3334.42466	3310.02482	3443.91853	CNNM4
6654.44224	6418.41927	6605.74254	7568.35321	7031.48609	7190.23987	C7orf50
16473.9603	16450.2237	16887.4342	19348.6691	20396.6843	20930.1569	NFE2L1
4577.98486	4555.00722	4562.75396	5030.19267	5117.00925	5124.50173	PRMT2
7206.2351	7314.82438	7306.60444	7865.99125	7922.00508	8253.75787	UBE3C
6761.20867	6696.86015	6982.39622	7647.43048	7320.6036	7504.90226	AC002310.11
19541.2501	19735.1915	19391.7043	20824.778	20752.5212	20382.5626	PTP4A1
8488.43007	8184.25801	8353.12951	8903.88035	8702.25169	9030.19753	CCDC85C
17036.7292	17448.1686	16457.1431	15578.2212	15537.2861	14803.435	KIAA0391__I
19655.0012	19925.5784	19633.6686	18076.8431	17841.8863	17844.8309	AZIN1
13399.6858	13678.5074	14163.8467	13140.0055	11822.3115	12679.4639	TPBG
25559.085	26804.0994	25765.0182	24431.5799	23079.3612	23410.6773	MORF4L2
12029.683	12575.0565	12340.1755	10914.8591	10786.3071	10544.2549	BUB3
4730.65087	4606.57035	4249.27322	4104.3297	4289.50305	3808.64084	SSBP1
1840.97255	1920.52812	1913.0669	1639.75495	1730.99842	1674.45342	ABCD3
4093.04575	4085.38614	4008.50093	3599.11384	3752.04101	3762.66744	CTC-432M15
3602.11973	3835.50329	3549.60327	2909.38437	3171.02601	3031.17955	PLP2
2447.64535	2684.45567	2528.10897	2248.21057	2124.82836	2103.53849	DDAH1
5808.29334	5937.69228	5972.82134	5131.23584	4991.91033	5034.59819	DARS
1116.55734	1005.8776	1091.81887	958.811835	950.751809	925.597801	WDR76
2902.64995	2916.88637	3000.11801	2640.30201	2578.89112	2694.04128	NARF
2029.55998	2086.32341	2095.43403	1673.8021	1970.07636	1888.99595	XRN1
5273.46338	5349.07936	5292.22246	4185.60355	4475.76144	4625.94574	NR2C2
8218.02164	8269.93213	7896.61573	6646.88342	6470.85759	6333.09136	SRSF11
1267.22772	1166.11993	1286.10541	1120.26125	1134.23023	1048.19354	E2F2
16311.3161	16263.8032	16936.3038	15217.9803	14689.3935	14939.312	PFKP
145604.465	153938.143	148228.715	132924.489	121976.083	121477.049	MT-ND6
1662.36329	1571.48542	1592.43451	1215.81295	1280.6423	1315.86089	GK5
1310.13385	1329.53537	1334.97504	1076.32944	1229.67607	1177.94069	RBL1
1837.9791	1853.8927	1865.38922	1739.69982	1619.79938	1640.73959	PLEKHA1
3563.20487	3522.95142	3740.31399	3081.81673	2894.88173	3071.02317	SEMA3F
29381.7223	29459.9971	30898.7124	25349.7548	24535.142	25411.031	GPI__PDCD2I
893.046305	914.650527	852.238527	779.789694	795.07315	768.266607	CFL2
119160.316	123088.321	123377.916	100329.28	100477.601	100482.53	GAPDH
4720.6727	4897.70369	4910.80103	3981.32062	4362.70908	4368.4947	KNTC1
3184.03437	3190.56758	3163.41406	2399.77533	2462.13212	2552.03456	PLEKHH1
2763.95337	2913.71326	3007.26966	2624.92587	2316.64671	2427.39556	PRSS53__RP
5419.14467	5426.02741	5395.92142	4347.05297	4522.09437	4370.53796	SLC25A36
33763.1372	33067.8293	32201.505	25769.3036	26476.4919	24450.6978	HNRNPH1
18971.4965	19579.7089	19969.7962	14631.4906	14538.3481	15762.7467	PREX1
84743.6085	87288.4395	91020.2667	76499.5656	72545.3283	75689.5853	ENO1
4918.24049	4825.51531	4840.47645	3889.06381	4075.44489	4261.22343	BBX
4106.01737	3921.17741	3902.4181	2812.73437	2900.44168	2923.90829	SOD2

2352.85272	2343.34576	2134.76812	1893.46117	1808.83775	1787.85447	ZBTB25
5049.95235	5305.44902	5318.44519	4147.16322	3788.1807	3758.58092	BNIP3L
3153.10204	3308.76613	3228.97087	3004.93606	2904.14831	2752.27426	DCUN1D3__I
1997.62984	2030.00061	1948.82516	1538.71178	1586.43967	1563.09562	IL6ST
41844.4579	44947.1803	47926.7957	38265.7081	31851.1123	36041.1029	IFFO1
3710.8818	3809.32509	3588.93735	3654.02861	3327.63133	3206.90011	HELLS
5059.93052	5430.78708	5071.7132	4098.83822	3908.64633	3679.91532	SNHG1__SN
3595.13501	3581.65406	3463.78344	2708.39632	2892.10175	2751.25263	ZNF292
18904.6428	19651.104	19121.1335	15726.491	15175.8893	14644.0606	MAL2
603.679346	638.589483	629.345374	540.361309	513.368911	506.729039	CP
868.100877	794.865419	839.127166	686.43459	720.940456	679.384699	FBXL20
9205.86057	9550.28421	9346.01719	6478.84423	6365.2185	6729.48423	AF011889.5_
985.843295	985.252346	988.119915	822.623212	818.239617	779.50455	PIGA
8189.08494	8042.2611	8334.05844	6365.71981	6347.61198	6566.02325	QSOX1
2252.0732	2302.88853	2355.27739	1836.34981	1809.76441	1823.61156	SLC9A8
5894.10561	5832.18619	5830.98025	3863.80302	4409.04202	4531.95568	NKTR
1273.21462	1270.83274	1239.61968	1022.51297	966.505007	987.9173	HSD17B12
8464.48246	8444.45348	8362.66505	5672.69545	5685.97768	5568.91127	CCNL1
849.142352	859.914286	898.724265	728.169813	794.146492	731.487887	AGR3
1586.52919	1588.14428	1559.06013	1268.53112	1369.60153	1376.13713	CRIM1
2161.27184	2176.7572	2189.59745	1500.27144	1535.47344	1492.60308	NPIPB5
914.998281	805.178044	766.418704	732.562994	714.453845	672.233282	FUT11
1106.57916	1123.28287	1015.53458	844.589119	874.765797	911.294965	ZGRF1
1423.885	1397.75735	1373.11718	924.764679	1039.71104	970.549571	ZNF37BP
3080.26139	2956.55032	2879.73186	2604.05826	2581.67109	2629.67852	FAM217B
2725.0385	2710.63387	2436.32944	1982.4231	2010.84934	1840.97929	CCDC125
3658.99531	3782.35361	3717.66709	3124.65025	3120.98645	2998.48736	AK4
2231.11904	2234.66655	2165.75861	1552.98962	1773.62472	1714.29703	TACC1
18631.2409	18575.4178	18253.3997	11657.3068	11712.0391	11776.342	RDX
6395.0098	6780.94771	6590.2473	5673.79374	5590.53184	5393.19072	TMEM167A
2268.03827	2266.39771	2159.7989	1591.42995	1745.82496	1633.58817	FAM162A
437.04389	438.68321	457.705727	390.993142	330.81715	382.090042	HLA-F
15942.1238	16163.8501	15859.9802	10922.5472	10856.7331	11236.9208	STAT1
10875.2086	10932.176	10925.3403	7155.39416	7181.6048	7236.21327	CA12
891.050671	860.707564	889.188729	601.865848	654.22103	615.021938	ATAD5
7033.61274	7237.08305	7043.18526	5782.52498	5978.80182	5954.06621	LRRC58
37988.8927	37824.3294	37262.4907	31764.8979	32546.1063	32484.8049	STC2
1117.55515	1010.63727	1045.33313	728.169813	670.900887	730.466256	KCNK5
8629.12228	8607.86892	8628.46811	6095.53916	6711.78884	6833.69061	PGR
31055.0616	32097.6493	31834.3868	24518.3453	24163.5518	24696.9109	PGK1
4088.05666	4136.94926	3998.9654	3010.42754	2888.39512	2762.49057	P4HA1
110530.196	112724.926	117968.883	75963.5974	76403.9351	81447.4983	ALDOA
179.607078	219.738245	244.348109	151.564757	160.311952	153.244669	CCBL1
4016.21383	3903.72528	3826.13381	3249.85592	3646.40192	3287.60897	PBX2
3642.03242	3743.48295	3768.92059	2851.17471	2928.24144	2974.98984	BHLHE40

1498.72129	1470.73901	1667.52685	999.448763	1004.49801	1107.44814	CLSTN2
747.365008	831.356247	799.79308	506.314153	467.962635	485.274785	BCL6
1852.94636	1907.83566	1899.95554	1433.27542	1398.32795	1340.38004	SFXN3
1378.98323	1416.79605	1314.71202	970.893084	1088.82395	1118.68608	ZBTB37
343.249083	357.768766	365.926193	300.932924	299.310755	284.013453	CYP4V2__KLI
4711.69235	4808.06318	4722.47419	4057.103	4022.62534	4118.19507	DUSP4
753.351911	843.25543	954.745539	697.417543	683.874108	713.098527	SCARA3
377.174864	376.01418	338.511527	249.313043	276.144288	256.429413	LDHAP4
3917.42994	3970.3607	3959.63131	2979.67527	2985.69428	2864.65368	C1orf21
13739.9415	14432.1223	14032.7331	8851.16217	8685.57184	8795.22237	PRSS23
33217.3313	34506.0439	34203.9675	21964.8086	21506.8214	21161.0455	LDHA
1236.29539	1244.65453	1298.02483	1034.59421	1048.97763	1027.76091	GPR37L1
5986.9026	5763.96421	5629.54205	4176.81719	4207.03042	4046.68089	PHLDA1
898.03539	869.433632	899.916207	589.784599	629.201246	609.913783	ALDOC
5855.19075	6003.53442	5943.02279	4238.32173	4153.28422	4374.62449	PAPSS2
20921.2311	21788.1972	21855.4485	13383.8271	12434.8329	13135.1114	FOS
803.242766	771.067053	892.764555	454.694272	422.55636	435.21486	LAMB3
989.834564	1005.8776	1057.25255	598.570962	631.981222	602.762365	KRT15
6561.64525	6458.87649	6515.15495	3622.17804	3583.38913	3479.67562	BNIP3
944.932794	916.237085	927.330873	401.976096	423.483018	410.695713	EFEMP1
431.056987	409.331892	456.513785	246.018157	246.49121	253.36452	FHL2
1706.26724	1811.84892	1808.17601	846.78571	748.740216	837.737524	WISP2
822.201291	913.063969	916.603395	427.236889	465.182659	453.60422	PFKFB4__UC
89.803539	106.299367	107.27478	65.8977206	54.6728623	48.016663	MSS51
10970.0012	10979.7727	11056.454	5774.83692	5740.65054	5789.5836	EGR1
1096.60099	1070.13318	1122.80936	565.622102	545.801964	569.048538	PDK1
101.777344	92.8136268	90.5875917	63.7011299	49.1129102	59.2546054	PRKAA2
1172.43509	1105.83073	1145.45626	754.528901	805.266396	823.434688	CD109
451.013329	385.533527	443.402423	195.496571	173.285174	205.347857	SPAG4
763.330082	705.224908	682.982764	211.971001	232.59133	260.515937	DDX60
9490.23844	9128.25986	9318.60253	3618.88316	3353.57778	3277.39266	IFI6
8697.97166	8697.50943	8741.7026	3365.17693	3256.27861	3299.86854	IFI27
1037.72978	1060.61384	1066.78809	543.656195	540.242012	574.156693	OAS1
61.8646602	44.4236162	59.5970998	35.145451	25.9464431	20.4326225	SCNN1G
2825.81803	2950.99736	2793.91204	704.007315	722.793773	727.401362	FOSB
624.633505	634.623089	686.55859	186.710208	186.258395	237.018421	OAS2
1450.82606	1542.13411	1668.7188	304.22781	317.843928	326.921961	PPFIA4
206.54814	200.699552	202.630139	18.6710208	20.386491	20.4326225	IFI44
3145.1195	3174.702	2928.60149	632.618118	713.527186	644.649241	UBE2T

baseMean	log2FoldChar	lfcSE	stat	pvalue	padj	Control.0
2440.97701	0.42577969	0.04925482	8.6444264	5.41E-18	1.56E-16	2016.69314
653.597875	0.54159851	0.09174527	5.90328516	3.56E-09	3.96E-08	509.265944
1332.62304	1.05003737	0.05979444	17.560786	4.92E-69	1.25E-66	875.937423
561.828649	0.38345087	0.08718525	4.39811618	1.09E-05	7.05E-05	521.303139
138.203818	1.70601133	0.18810238	9.06958913	1.19E-19	3.89E-18	63.8897275
259.608562	0.39597947	0.12090895	3.275022	0.00105654	0.004396	230.558582
2180.60193	0.83711881	0.0471656	17.7485049	1.77E-70	4.57E-68	1539.83503
3981.45926	0.96003535	0.03994396	24.0345595	1.21E-127	9.03E-125	2659.29416
5501.05519	0.79512143	0.03882601	20.479094	3.31E-93	1.36E-90	4043.57159
178.338545	0.50972309	0.14615067	3.48765471	0.00048728	0.00221008	137.038836
179.335628	0.74149926	0.14780616	5.01670068	5.26E-07	4.26E-06	142.594464
192.262507	0.88656553	0.14009559	6.32829026	2.48E-10	3.25E-09	138.890712
115.609629	0.68623918	0.18923169	3.62644964	0.00028735	0.00137705	94.4456841
1135.99844	0.51557243	0.06765957	7.62009636	2.53E-14	5.26E-13	910.197132
2266.05568	0.19897744	0.04589565	4.33543111	1.45E-05	9.16E-05	2083.36068
721.479867	0.61158606	0.07386497	8.27978453	1.23E-16	3.13E-15	575.933485
278.903884	0.56558927	0.12027627	4.70241764	2.57E-06	1.87E-05	232.410458
393.84455	0.52641701	0.10323461	5.09922997	3.41E-07	2.85E-06	299.078
339.800311	0.49731727	0.11284068	4.40725146	1.05E-05	6.78E-05	291.670495
885.425485	0.75632297	0.06948634	10.8844849	1.37E-27	7.55E-26	653.712284
916.997392	0.50249071	0.06653448	7.55233579	4.28E-14	8.72E-13	775.010172
4413.86721	0.57188874	0.03789022	15.0933073	1.79E-51	2.50E-49	3440.7859
784.824731	0.42954573	0.07702029	5.57704623	2.45E-08	2.44E-07	634.267584
750.356086	0.34057737	0.07443242	4.5756587	4.75E-06	3.27E-05	654.638222
452.860459	0.25865292	0.09571909	2.70220823	0.00688806	0.02277807	425.005578
1508.04966	0.40310534	0.05756883	7.00214549	2.52E-12	4.23E-11	1246.31265
1613.54419	0.42488687	0.06130052	6.93121113	4.17E-12	6.80E-11	1316.68395
1122.69412	0.65065002	0.06339344	10.2636799	1.03E-24	4.63E-23	843.52959
1042.67505	0.39479121	0.0621614	6.35106696	2.14E-10	2.83E-09	902.789627
1088.87558	0.5374359	0.06478062	8.29624558	1.07E-16	2.74E-15	896.308061
1743.43532	0.62866933	0.06531915	9.62457846	6.30E-22	2.43E-20	1312.05426
908.184448	0.31003875	0.08350157	3.71296926	0.00020484	0.00101453	709.268569
13038.1159	0.80500823	0.0289346	27.8216477	2.37E-170	3.54E-167	9397.34556
4331.16747	0.51493503	0.03818007	13.4870096	1.87E-41	1.84E-39	3554.67629
5215.41098	0.58152081	0.03975459	14.6277645	1.87E-48	2.28E-46	4085.23881
1502.19029	0.62393273	0.05739243	10.8713413	1.58E-27	8.69E-26	1164.8301
2050.11075	0.48508534	0.0489964	9.90042862	4.14E-23	1.70E-21	1640.76228
3975.98109	0.21194404	0.03931733	5.39060083	7.02E-08	6.57E-07	3638.93665
1098.89217	0.25683601	0.0638397	4.02313956	5.74E-05	0.00031834	966.679355
4848.23542	0.42557679	0.04050755	10.506111	8.10E-26	3.93E-24	3978.75593
1453.81498	0.17441145	0.05703891	3.05776256	0.00222996	0.00852364	1355.57335
3051.9993	0.20343137	0.05280573	3.85244899	0.00011694	0.00060988	2712.99857
382.366265	0.63637592	0.1002866	6.34557276	2.22E-10	2.92E-09	303.70769
2204.08933	0.54291859	0.04961447	10.9427482	7.20E-28	4.07E-26	1757.43047

3534.10281	0.40211827	0.03977107	10.1108229	4.95E-24	2.14E-22	3053.74379
266.468672	0.31141758	0.12197151	2.55319942	0.01067384	0.03326565	217.595449
1767.79436	0.25575005	0.05178177	4.93899798	7.85E-07	6.20E-06	1658.3551
5174.60923	0.51677372	0.03433088	15.0527367	3.31E-51	4.59E-49	4232.46296
1781.80724	0.54796046	0.05285543	10.3671562	3.50E-25	1.63E-23	1392.61087
3788.27585	0.3365653	0.03852205	8.73695301	2.39E-18	7.10E-17	3318.56208
3418.67941	0.34583344	0.0421026	8.21406472	2.14E-16	5.32E-15	2924.11245
3759.54136	0.53143445	0.04370571	12.159383	5.11E-34	3.74E-32	3001.89125
818.632302	0.44027247	0.07155523	6.15290394	7.61E-10	9.35E-09	702.787002
2344.33356	0.22608303	0.04631719	4.88119028	1.05E-06	8.17E-06	2093.546
3822.29298	0.13720471	0.04367552	3.14145545	0.0016811	0.00664387	3514.86095
3579.08392	0.25815431	0.04490337	5.74910707	8.97E-09	9.50E-08	3226.89421
1044.51875	0.28369753	0.06296258	4.50581167	6.61E-06	4.47E-05	921.308389
2511.17203	0.36210646	0.04839068	7.48297952	7.27E-14	1.44E-12	2145.39853
8844.15446	0.25670858	0.04331119	5.92707222	3.08E-09	3.45E-08	7645.47072
1437.55807	0.31817197	0.05602767	5.67883682	1.36E-08	1.40E-07	1243.53484
8535.99265	0.18433566	0.03293567	5.59683991	2.18E-08	2.19E-07	7907.5112
15184.4492	0.29470952	0.0380868	7.73783968	1.01E-14	2.18E-13	13607.586
1698.61361	0.20019675	0.05322611	3.76125064	0.00016907	0.00085303	1546.31659
2103.16824	0.35173833	0.04866704	7.22744465	4.92E-13	8.98E-12	1881.50618
1003.21389	0.15444736	0.06462294	2.38997741	0.01684941	0.04928713	916.678698
3314.30739	0.22190634	0.04244093	5.22859222	1.71E-07	1.50E-06	2990.78
1726.80662	0.35813462	0.05351194	6.69261166	2.19E-11	3.30E-10	1517.61251
503.607522	0.3435991	0.09276371	3.70402514	0.00021221	0.00104805	431.487145
2809.77648	0.24118457	0.04199254	5.7435099	9.27E-09	9.80E-08	2537.07034
5661.05072	0.30585092	0.03333691	9.17454366	4.53E-20	1.53E-18	5025.99189
992.398896	0.39780271	0.07055018	5.6385779	1.71E-08	1.75E-07	878.715237
3139.33263	0.12374854	0.04554859	2.71684675	0.00659071	0.02192568	2997.26156
5334.03048	0.13634927	0.03593886	3.79392337	0.00014829	0.00075685	5218.58701
3427.04575	0.22259448	0.04185864	5.31776691	1.05E-07	9.58E-07	3105.59632
2383.40885	0.12007062	0.04465646	2.68876246	0.00717174	0.02355944	2305.58582
1554.97072	0.19850428	0.06484158	3.06137315	0.00220324	0.00843402	1398.1665
2308.36056	0.19213269	0.04600656	4.17620175	2.96E-05	0.00017497	2162.06542
6425.79179	0.23908536	0.03248932	7.3588918	1.85E-13	3.52E-12	5948.22622
1221.26147	0.23172509	0.05881446	3.93993399	8.15E-05	0.00043912	1127.79258
6263.30405	0.23192546	0.03459936	6.70317274	2.04E-11	3.09E-10	5899.1515
2956.76525	0.42787091	0.04919031	8.69827657	3.37E-18	9.90E-17	2420.40214
1447.09084	0.21522956	0.06122364	3.51546471	0.00043899	0.00201128	1319.46176
78804.6959	0.21259667	0.03370977	6.30667787	2.85E-10	3.70E-09	72456.5066
4963.18735	0.12357706	0.0380065	3.25147149	0.00114809	0.00473729	4568.57848
11520.9187	0.15757012	0.03527314	4.46714246	7.93E-06	5.27E-05	10435.3222
3630.15764	0.10391786	0.03856005	2.69496174	0.00703967	0.02318451	3440.7859
4774.09774	0.24922319	0.03573888	6.97344808	3.09E-12	5.12E-11	4293.57487
77854.5077	0.28439367	0.02734971	10.3984153	2.52E-25	1.19E-23	70460.1841
1989.5296	0.13024151	0.04778433	2.72561134	0.00641825	0.02144396	1945.3959

6197.96106	0.23737786	0.03392513	6.99711095	2.61E-12	4.37E-11	5763.96454
2804.10559	0.27393761	0.04335755	6.3181059	2.65E-10	3.46E-09	2549.10753
1668.25177	0.2960675	0.05962096	4.96582919	6.84E-07	5.45E-06	1439.83371
868.381699	0.18800463	0.07099318	2.64820668	0.008092	0.02616136	801.862377
9515.10816	0.46054897	0.03356668	13.72042	7.66E-43	8.03E-41	7839.91772
4623.88151	0.27999692	0.03840379	7.29086635	3.08E-13	5.74E-12	4062.09035
3332.70443	0.26087827	0.04366011	5.97520943	2.30E-09	2.62E-08	3061.15129
7137.82218	0.12695466	0.03715725	3.41668655	0.00063388	0.0027965	6745.45891
1056.37263	0.19653468	0.06258231	3.1404189	0.00168706	0.006662	973.160921
3534.06495	0.28398816	0.04288083	6.62272984	3.53E-11	5.14E-10	3167.63417
5976.648	0.1181193	0.04124469	2.86386651	0.00418504	0.01479881	5867.66961
1458.68741	0.1822936	0.05551403	3.28373948	0.0010244	0.0042788	1367.61054
15235.7936	0.32606333	0.0301585	10.8116574	3.03E-27	1.63E-25	13326.1008
6983.01878	0.27793344	0.03321691	8.36722647	5.90E-17	1.53E-15	6300.08269
4852.3081	0.15414623	0.04179193	3.68842123	0.00022565	0.00111021	4541.72628
13311.7822	0.28617458	0.03071895	9.31589686	1.21E-20	4.24E-19	11894.6006
5244.02075	0.29997083	0.04139353	7.24680454	4.27E-13	7.85E-12	4500.05906
2326.32417	0.25041523	0.04735579	5.28795371	1.24E-07	1.11E-06	2127.80571
2244.35698	0.15272026	0.0587749	2.59839238	0.00936614	0.02974486	2100.02756
2979.26983	0.22123354	0.04332212	5.10671115	3.28E-07	2.75E-06	2709.29482
1055.05117	0.16861881	0.06250577	2.69765182	0.00698304	0.02304107	994.457497
8873.51245	0.22558444	0.0348462	6.47371807	9.56E-11	1.33E-09	7947.32653
1794.81277	0.22627744	0.05258931	4.30272732	1.69E-05	0.00010506	1591.68756
3689.25542	0.31019596	0.03933744	7.88551376	3.13E-15	7.07E-14	3276.89486
5665.98955	0.2032913	0.0362078	5.61457212	1.97E-08	1.99E-07	5311.18082
1376.25067	0.28827143	0.05934771	4.85733016	1.19E-06	9.16E-06	1319.46176
6024.54516	0.29306291	0.03927822	7.46120608	8.57E-14	1.68E-12	5544.51722
8737.25143	0.30492735	0.03635796	8.38681233	5.00E-17	1.32E-15	7607.50726
25435.4068	0.2905318	0.03408177	8.52455198	1.53E-17	4.28E-16	22037.3263
13729.5659	0.25285281	0.030091	8.40293753	4.35E-17	1.16E-15	12373.3106
2896.0978	0.32290896	0.04208189	7.67334771	1.68E-14	3.53E-13	2568.55223
8172.99751	0.2864061	0.035327	8.10728703	5.18E-16	1.25E-14	7408.43057
2967.21041	0.23152036	0.04290323	5.39633827	6.80E-08	6.38E-07	2656.51635
6817.89004	0.18264642	0.03543754	5.15403818	2.55E-07	2.18E-06	6286.19362
25716.995	0.11004817	0.02442055	4.50637664	6.59E-06	4.46E-05	24839.2149
5225.61609	0.26347511	0.03534337	7.45472532	9.01E-14	1.77E-12	4717.65451
5301.36324	0.15180517	0.03538698	4.28985957	1.79E-05	0.00011087	5086.17787
16519.4099	0.26971174	0.02897746	9.30763841	1.31E-20	4.57E-19	14728.897
2010.29307	0.15625576	0.04755237	3.28597199	0.00101631	0.00425053	1862.06148
4115.38627	0.12070716	0.04022768	3.00059997	0.00269448	0.01007572	3872.27305
6796.58674	0.21913393	0.03598721	6.08921728	1.13E-09	1.36E-08	6268.6008
4396.93001	0.17213297	0.03812312	4.5151859	6.33E-06	4.29E-05	4225.05546
9306.02445	0.26489262	0.03170554	8.35477325	6.56E-17	1.69E-15	8469.55561
5854.49298	0.18755443	0.03354937	5.59040081	2.27E-08	2.27E-07	5440.81215
6694.2602	0.21678332	0.03443936	6.294639	3.08E-10	3.98E-09	5987.11562

3839.3598	0.15563321	0.04090575	3.80467842	0.00014199	0.00072816	3607.45476
13796.0188	0.22109949	0.03108763	7.11213708	1.14E-12	2.00E-11	12501.09
3084.50295	0.21075372	0.0442998	4.75744126	1.96E-06	1.46E-05	2793.55519
6325.75934	0.11117749	0.03418529	3.25220238	0.00114515	0.00472714	6175.08105
16073.6387	0.12077375	0.02767065	4.36468757	1.27E-05	8.10E-05	15287.2377
4382.89011	0.09714258	0.03534386	2.74849921	0.00598688	0.02018008	4248.20391
7149.78991	0.15933408	0.03804236	4.18833318	2.81E-05	0.00016694	6687.12481
6526.80777	0.09159467	0.03291969	2.78236706	0.0053964	0.01845284	6274.15642
19474.7168	0.19713969	0.02594556	7.59820537	3.00E-14	6.19E-13	18133.5713
8153.04303	0.14805273	0.03212009	4.60934934	4.04E-06	2.82E-05	7876.95524
15168.1178	-0.1113577	0.03093023	-3.6002874	0.00031787	0.00150951	15809.4668
17637.2183	-0.1107861	0.02625282	-4.2199717	2.44E-05	0.00014687	18239.1283
12182.1974	-0.1353553	0.03039772	-4.4528124	8.48E-06	5.59E-05	12434.4225
22731.7357	-0.1822265	0.02859487	-6.3727006	1.86E-10	2.49E-09	23717.9039
10663.9519	-0.2056436	0.03168681	-6.4898794	8.59E-11	1.20E-09	11163.1095
4018.7583	-0.1290856	0.05259453	-2.4543532	0.01411383	0.04230003	4389.87243
1638.47818	-0.2079038	0.05281315	-3.9365909	8.26E-05	0.00044491	1708.35576
3392.5095	-0.3241946	0.04095241	-7.9163739	2.45E-15	5.58E-14	3798.198
3246.80585	-0.1329821	0.04678298	-2.8425323	0.00447567	0.01571346	3342.63647
2208.94463	-0.2110916	0.04973697	-4.2441588	2.19E-05	0.00013317	2271.32611
5125.02661	-0.1998823	0.03647031	-5.4806856	4.24E-08	4.09E-07	5389.88556
933.291668	-0.1838447	0.06951321	-2.6447445	0.00817526	0.02637774	1036.12471
2537.85433	-0.2174242	0.04428784	-4.9093436	9.14E-07	7.14E-06	2693.55387
1844.65391	-0.1203475	0.04962176	-2.4252964	0.01529589	0.04531727	1883.35805
4567.45604	-0.227217	0.03908844	-5.8128957	6.14E-09	6.66E-08	4893.58275
6854.51171	-0.2904689	0.03436682	-8.4520161	2.86E-17	7.82E-16	7626.02602
1049.04034	-0.2787778	0.06791052	-4.1050761	4.04E-05	0.00023188	1175.94136
14462.7866	-0.1685457	0.02825875	-5.9643733	2.46E-09	2.79E-08	15136.3098
131999.765	-0.1414855	0.02944765	-4.8046459	1.55E-06	1.17E-05	135115.662
1374.7462	-0.2504932	0.0579478	-4.3227394	1.54E-05	9.66E-05	1542.61284
1112.34728	-0.3066218	0.06111207	-5.0173689	5.24E-07	4.25E-06	1215.7567
1621.55934	-0.1781936	0.05533337	-3.2203645	0.00128028	0.00521609	1705.57794
3000.75309	-0.3349805	0.04341582	-7.7156332	1.20E-14	2.58E-13	3306.52488
25816.6363	-0.2160694	0.0280215	-7.7108437	1.25E-14	2.67E-13	27265.1727
770.299773	-0.1968125	0.0728929	-2.7000226	0.00693348	0.02289701	828.714581
103642.031	-0.2628526	0.02515572	-10.44902	1.48E-25	7.10E-24	110576.451
4266.48011	-0.1560607	0.04202646	-3.7133906	0.0002045	0.0010131	4380.61305
2737.63132	-0.2275969	0.04437	-5.1295225	2.90E-07	2.46E-06	2954.66841
2359.29911	-0.4018544	0.04802893	-8.3669247	5.91E-17	1.54E-15	2564.84848
4597.39955	-0.2689527	0.03561987	-7.5506367	4.33E-14	8.83E-13	5028.76971
27799.81	-0.2940442	0.02722112	-10.80206	3.37E-27	1.79E-25	31330.9668
17192.42	-0.1523419	0.0326658	-4.6636498	3.11E-06	2.22E-05	17604.8607
75387.9867	-0.2283363	0.02818937	-8.1000867	5.49E-16	1.33E-14	78638.9951
4160.41609	-0.2453801	0.03874866	-6.3326078	2.41E-10	3.17E-09	4563.94879
3394.02355	-0.2502868	0.04077212	-6.1386766	8.32E-10	1.02E-08	3810.2352

1922.65004	-0.2872568	0.05413525	-5.3062797	1.12E-07	1.01E-06	2183.36199
4246.62089	-0.4130275	0.04023066	-10.266487	9.98E-25	4.51E-23	4686.17262
2691.04158	-0.3333629	0.04574899	-7.2867811	3.17E-13	5.91E-12	2925.96433
1685.89168	-0.2842712	0.05507169	-5.1618388	2.45E-07	2.10E-06	1853.72803
37742.4228	-0.3004795	0.0772134	-3.8915466	9.96E-05	0.00052702	38830.1393
3162.48628	-0.2505873	0.04172603	-6.0055392	1.91E-09	2.20E-08	3443.56372
4582.24589	-0.1476223	0.04005946	-3.6850794	0.00022863	0.00112345	4695.432
2945.29977	-0.341599	0.04278371	-7.9843241	1.41E-15	3.30E-14	3336.1549
16320.2133	-0.2693953	0.02838218	-9.4917066	2.27E-21	8.56E-20	17542.8229
518.923778	-0.3307792	0.08671646	-3.8144916	0.00013646	0.00070243	560.192538
707.10452	-0.2680667	0.07633363	-3.5117779	0.00044512	0.00203794	805.566129
8319.82414	-0.1298414	0.03154269	-4.1163698	3.85E-05	0.00022179	8542.70472
821.000146	-0.3318744	0.07080195	-4.6873616	2.77E-06	1.99E-05	914.826822
6445.85804	-0.5181812	0.03418564	-15.157861	6.72E-52	9.80E-50	7599.17382
1780.10313	-0.5903155	0.05245494	-11.253765	2.22E-29	1.36E-27	2089.84224
4963.02833	-0.2728037	0.03476619	-7.8468112	4.27E-15	9.48E-14	5469.51623
1024.37634	-0.4125244	0.06438591	-6.407059	1.48E-10	2.02E-09	1181.49699
6783.92963	-0.4428243	0.0322985	-13.710368	8.80E-43	9.17E-41	7854.73273
680.241313	-0.5368184	0.07742137	-6.9337233	4.10E-12	6.69E-11	787.973305
1289.81923	-0.3920662	0.05685665	-6.895697	5.36E-12	8.64E-11	1472.24155
1742.36423	-0.4600468	0.05024734	-9.1556454	5.40E-20	1.81E-18	2005.58188
674.938436	-0.3970893	0.0890825	-4.4575456	8.29E-06	5.49E-05	849.085219
900.050944	-0.3395024	0.0711048	-4.7746766	1.80E-06	1.34E-05	1026.86533
1110.27997	-0.4937763	0.06276328	-7.867281	3.62E-15	8.13E-14	1321.31364
2549.16747	-0.2358124	0.04528055	-5.207808	1.91E-07	1.67E-06	2858.37085
2128.73002	-0.4182861	0.05511944	-7.5887208	3.23E-14	6.65E-13	2528.73689
3128.90057	-0.3018124	0.04388417	-6.8774779	6.09E-12	9.75E-11	3395.41494
1823.65549	-0.3658374	0.05134973	-7.124426	1.05E-12	1.84E-11	2070.39755
15016.5047	-0.413731	0.02815201	-14.69632	6.81E-49	8.51E-47	17289.1158
5861.40076	-0.1233165	0.03785022	-3.2580129	0.00112195	0.00464327	5934.33715
1685.07567	-0.6686652	0.05389873	-12.405954	2.43E-35	1.86E-33	2104.65725
356.494814	-0.4509963	0.10666477	-4.2281655	2.36E-05	0.0001421	405.560879
14068.8103	-0.1580343	0.02964062	-5.3316817	9.73E-08	8.92E-07	14793.7127
9513.70495	-0.1862359	0.03120989	-5.9672101	2.41E-09	2.74E-08	10091.7991
726.17722	-0.3682604	0.07829411	-4.7035516	2.56E-06	1.86E-05	826.862705
6161.82786	-0.2031037	0.03386625	-5.9972306	2.01E-09	2.31E-08	6526.93752
30191.9538	-0.4600592	0.02430913	-18.925366	7.05E-80	2.36E-77	35252.3146
849.563967	-0.4524129	0.07154189	-6.3237482	2.55E-10	3.34E-09	1037.05065
7711.09387	-0.1093741	0.03298363	-3.3160119	0.00091312	0.00386144	8007.51251
25641.6066	-0.4229369	0.02552308	-16.570759	1.13E-61	2.50E-59	28817.9708
3398.23937	-0.32654	0.0394608	-8.2750485	1.28E-16	3.25E-15	3793.56831
87155.2728	-0.6169161	0.02745268	-22.471984	7.80E-112	4.45E-109	102568.013
175.706339	-0.3785047	0.15513138	-2.4398975	0.01469143	0.04377882	166.668854
3065.30375	-0.5352987	0.049581	-10.796448	3.58E-27	1.89E-25	3726.90077
2860.21267	-0.6007881	0.04327709	-13.882359	8.10E-44	8.93E-42	3379.67399

1173.9137	-0.6482701	0.06551238	-9.8953835	4.36E-23	1.78E-21	1390.759
630.960643	-0.4820549	0.08249798	-5.8432327	5.12E-09	5.59E-08	693.527621
1429.50787	-0.6625419	0.05589365	-11.853616	2.06E-32	1.41E-30	1719.46701
1170.73126	-0.2504486	0.0608327	-4.1170056	3.84E-05	0.00022131	1279.64643
293.734135	-0.3546578	0.1126975	-3.1469891	0.00164961	0.0065354	318.522699
4095.22881	-0.2189359	0.0366556	-5.9727829	2.33E-09	2.66E-08	4372.27961
706.295382	-0.340011	0.08767538	-3.878067	0.00010529	0.00055343	699.08325
284.718778	-0.5587538	0.1225039	-4.5611102	5.09E-06	3.49E-05	350.004594
3100.25753	-0.5305154	0.04044652	-13.116467	2.65E-39	2.42E-37	3635.2329
10443.9443	-0.735691	0.03160635	-23.276685	7.64E-120	5.29E-117	12750.1674
25721.4434	-0.6614795	0.02658031	-24.886071	1.05E-136	8.87E-134	30824.4787
991.835685	-0.5172533	0.06469123	-7.9957258	1.29E-15	3.01E-14	1147.23728
4394.53352	-0.6534461	0.04038753	-16.179402	7.05E-59	1.44E-56	5555.62847
621.638224	-0.978165	0.08081898	-12.10316	1.02E-33	7.32E-32	833.344271
3953.12849	-1.1966062	0.03823886	-31.292935	5.82E-215	2.82E-211	5433.40465
16092.8343	-0.7092423	0.03052459	-23.235114	2.01E-119	1.30E-116	19414.1437
558.380436	-1.0993962	0.09120051	-12.054715	1.83E-33	1.31E-31	745.380154
701.04737	-1.0393034	0.07888184	-13.175446	1.22E-39	1.13E-37	918.530574
4629.67829	-0.9076751	0.03990849	-22.743911	1.65E-114	9.68E-112	6088.96881
629.722688	-1.1202522	0.08267592	-13.549922	7.93E-42	7.88E-40	876.863361
294.135983	-1.0925987	0.11854995	-9.2163574	3.07E-20	1.04E-18	400.00525
1352.45331	-0.6352337	0.05849647	-10.859351	1.80E-27	9.89E-26	1583.35412
625.876823	-0.9294507	0.08405693	-11.057395	2.02E-28	1.17E-26	762.972977
77.0696003	-0.6576831	0.24255764	-2.7114508	0.00669895	0.02224377	83.3344271
8504.39865	-0.5855661	0.03015022	-19.421619	5.07E-84	1.82E-81	10179.7632
744.328217	-1.1132899	0.0765228	-14.548473	5.97E-48	7.15E-46	1017.60595
60.6049892	-1.41192	0.26334714	-5.3614406	8.26E-08	7.65E-07	94.4456841
807.162947	-0.9431467	0.08489202	-11.109958	1.12E-28	6.59E-27	1087.97724
279.428276	-1.2784708	0.12611298	-10.137504	3.77E-24	1.64E-22	418.524012
605.91253	-0.287243	0.08260066	-3.4774907	0.00050613	0.00228383	708.342631
7297.0306	-0.5349036	0.03674363	-14.557724	5.22E-48	6.28E-46	8806.59707
7671.46031	-0.153235	0.03156008	-4.8553417	1.20E-06	9.24E-06	8071.40224
895.946991	-0.264313	0.0687783	-3.8429702	0.00012155	0.00063206	962.975602
33.98459	-1.6556939	0.36297687	-4.561431	5.08E-06	3.49E-05	57.4081609
1809.08925	-1.4509213	0.05678204	-25.55247	5.15E-144	5.00E-141	2622.25664
553.877807	-0.255924	0.08816178	-2.9028903	0.00369736	0.01328715	579.637238
862.208346	-2.3406392	0.08417131	-27.80804	3.47E-170	4.80E-167	1346.31397
120.274689	-1.8729097	0.19491547	-9.6088304	7.34E-22	2.82E-20	191.669182
1658.62374	-2.633982	0.06672444	-39.47552	0	0	2918.55683

Control.1	Control.2	shRNA2.0	shRNA2.1	shRNA2.2	Gene Set_C	baseMean
2038.74938	2192.38182	2918.38428	2776.32523	2703.32822	STMN3	223.786099
517.78931	577.640146	664.889461	814.301075	837.701312	TMEM238	234.55014
859.544986	868.116931	1822.63405	1706.55715	1862.94769	NAALADL2	1301.87486
462.54863	480.446393	629.242706	606.734134	670.696893	FGF12	208.124666
79.5465797	49.701351	189.082784	209.445375	237.557088	NUPR1	12488.9655
209.914585	234.148587	283.624176	293.974898	305.430542	NANOS1	747.240647
1545.26596	1610.32377	2850.19049	2764.11541	2773.88089	BTG2	1131.41989
2647.86994	2804.26067	5391.95905	5213.59316	5171.77857	ID1	1644.63244
4004.58105	4029.12286	6682.9915	6921.08953	7324.97458	MDK	7095.84348
153.20082	152.417476	192.182501	223.533628	211.658007	PDCD4-AS1	281.317496
124.475666	134.745885	246.427562	217.898327	209.871864	IL1R1	831.809248
126.685294	140.268257	247.977421	247.014052	252.739308	HIST1H2AD_	435.372935
86.9120037	82.835585	167.384759	117.402116	144.677625	CEP19	501.743493
891.216309	1003.96729	1399.52257	1358.10768	1252.97968	CTXN1	1404.29206
2145.54802	2098.50149	2442.5776	2366.82665	2459.51963	SKAP2	3893.58501
577.449245	557.759606	878.769987	857.505053	881.461828	CAMK1D	601.513072
227.591603	215.372521	314.621353	368.173035	315.254332	RP11-47A8.5	195.118271
310.820895	358.954202	485.10583	453.641775	455.466598	FAM43A	491.753437
262.945638	289.37231	437.060205	354.084781	403.668436	CNIH2	572.429139
645.947689	673.729425	1168.5936	1098.8838	1071.68611	MAPK8IP1	1616.86197
757.902134	745.520265	1044.60489	1087.6132	1091.33369	HIST1H2BG	1543.88047
3628.94443	3575.18385	5374.91061	5183.53821	5279.84025	ODC1	3724.87193
648.893858	728.953148	855.522103	941.09536	900.216335	HIST1H3E	746.906073
693.086403	637.281767	833.824079	815.240292	868.065752	EFNB3	389.69932
397.732898	414.177925	500.604419	525.961479	453.680455	PPP1R3F	464.407465
1294.84155	1350.77227	1813.33489	1659.59631	1683.44027	SESN2	1903.96162
1300.73389	1523.07029	1819.53433	1850.25734	1870.98534	PRR7	509.745775
853.652647	925.549603	1396.42285	1332.74882	1384.26123	HOXC13	2172.43703
892.689394	907.878012	1182.54233	1198.4408	1171.71015	HIST3H2A	1174.18423
914.785666	848.236391	1337.52822	1310.20761	1226.18753	MATN2	1294.63061
1259.48751	1542.95083	2151.20413	2039.03995	2155.87523	SAMD11	887.655626
790.31	936.594348	1007.40827	1011.53663	993.98887	FAM171A2	402.155415
9461.62373	9627.70393	16284.3673	16515.1904	16942.4644	BAMBI	5451.11578
3475.74361	3672.3776	5137.7822	4985.36344	5161.06171	ID3	2856.53176
4099.59502	4364.88309	5997.95388	6320.92991	6423.86518	TRIP6	958.15458
1157.84466	1222.65324	1889.27798	1854.01421	1724.52157	SMAD6	443.87608
1745.6055	1737.33834	2417.77986	2417.54437	2341.63416	CYFIP2	5824.43923
3734.26999	3677.89998	4293.10911	4122.22309	4389.44771	ID2	1188.86485
979.601398	1058.08654	1222.83866	1207.83297	1158.31407	IGSF9	2810.28998
4068.66024	4370.40547	5632.18718	5601.48975	5437.91395	TGFB1	713.003737
1303.68006	1442.44365	1549.85888	1528.10594	1543.228	DYRK1B	730.888112
2697.21829	3102.46878	3397.29067	3227.14936	3174.87011	ZNF219	741.995421
308.611267	283.849938	477.356536	452.702558	467.969603	METTL7A	10289.5748
1814.10394	1819.06945	2464.27562	2682.40354	2687.25293	TEAD2	2116.68244

3064.0164	3007.48397	4152.07195	3988.85428	3938.44647	KDM4A	2453.11488
245.268621	249.61123	306.872059	306.184718	273.279959	CEP126	1643.76578
1627.75871	1544.05531	1952.82219	1883.12994	1940.64494	THRB	2009.43007
4261.63435	4275.42066	6173.08793	5987.5079	6117.54156	JADE2	2165.60697
1478.97715	1464.53314	2210.09877	2087.87923	2056.74427	SGPP1	2615.09244
3377.78347	3344.34869	4280.71023	4289.4037	4118.84696	ASAP1	3987.02491
3016.87769	3089.21508	3894.79537	3898.68946	3688.38637	NACC2	2651.18557
2955.74467	3276.97574	4392.30007	4427.46859	4502.86782	DBN1	6227.02793
702.661454	673.729425	993.459544	933.581624	905.574766	RP11-400F19	3481.99613
2159.54233	2235.45632	2478.22435	2540.58179	2558.6506	CCDC90B	3027.83517
3532.45737	3878.91433	4049.78126	3954.10326	4003.64071	ARHGAP39_	4012.81754
3164.18617	3381.90082	4114.87533	3853.60705	3733.03996	TTYH3	3904.25845
947.930074	954.265939	1185.64205	1132.69561	1125.27042	CADPS2	1017.86964
2312.00661	2129.42677	2851.74034	2733.12125	2895.33865	HIST1H2AC	2753.90427
7771.25891	8768.42279	9725.36449	9466.3674	9688.04246	H2AFX	6348.09564
1301.47043	1288.9217	1659.89886	1553.4648	1578.0578	TCAIM	1713.3216
7895.73458	8178.63343	9015.52912	8868.08622	9350.46134	MARCKSL1	5666.78525
13041.9564	14265.3922	17341.371	16951.9263	15898.4635	TNRC18	18322.725
1573.99112	1630.20431	1734.29209	1830.53379	1876.34377	PODXL2	2127.56058
1766.96523	1899.69608	2363.5348	2343.34623	2363.96095	FRAT2	2548.76194
958.978211	976.355429	1022.90686	1074.46416	1069.89997	TEF	775.996301
3030.13545	3169.84172	3437.587	3625.37733	3632.12285	MXD4	4002.34392
1585.03925	1433.60786	1932.67403	1961.08494	1930.82115	USP54	5726.48538
431.613849	468.297174	588.946375	511.873225	589.427362	CADM4	912.684202
2588.21001	2597.72395	3093.51833	3045.88049	2996.25576	STAT6	7889.84537
5129.2813	5036.40357	6176.18765	6304.96322	6293.4767	CDKN1B	2498.00005
875.748919	808.47531	1190.29162	1158.05447	1043.10782	TBC1D4	1284.93708
2852.62873	3168.73725	3312.04843	3293.83376	3211.48605	MESDC1	1112.56031
5104.23886	4924.85165	5503.54889	5585.52306	5667.4334	TCAF1	7275.88513
3090.53193	3297.96076	3704.16273	3612.2283	3751.79447	UBE2S	2367.95252
2298.74884	2244.29212	2520.07054	2440.08557	2491.67021	PBXIP1	5671.55511
1571.78149	1369.54834	1678.49717	1551.58636	1760.24444	STK17A	2054.78523
2108.7209	2191.27734	2532.46941	2417.54437	2438.08591	LIMK1	1673.41139
5940.21449	5797.38648	6858.12556	6985.8955	7024.90247	TARS	6142.13663
1107.02323	1134.29528	1346.82737	1278.27424	1333.35614	ARHGEF18_	2463.03158
5577.83563	5808.43122	6805.43035	6816.83645	6672.13913	TLE3	4657.75515
2519.71157	2617.60449	3586.37345	3404.66136	3191.83847	POLR1B	2154.38842
1415.6345	1272.35459	1659.89886	1494.29413	1520.90121	SLC35D2	659.400015
70656.5129	75939.2465	80561.6647	86311.2178	86903.0269	HSPB1	42564.8752
4863.3895	4822.13552	5112.98445	5301.87955	5110.15662	HIST2H2AA3	2399.24593
10962.6972	11271.1619	12567.8057	11791.8685	12096.657	MAGED2	20064.6646
3512.57073	3540.94514	3829.7013	3722.11668	3734.82611	SLC37A1	1886.25032
4394.94853	4405.74865	5078.88756	5274.64226	5196.78458	SOX12	4630.52711
70627.0512	69511.2051	87906.446	86058.5685	82563.5912	NCOA3	7240.14197
1882.60239	1872.08422	2067.51175	2086.94001	2082.64335	QSOX2	1717.81171

5591.82993	5716.75984	6650.44447	6869.4326	6595.33496	DIP2B	9973.29849
2579.3715	2487.2765	3026.8744	3166.10026	3015.90334	ANKRD40	3130.63299
1427.41918	1633.51774	1790.08701	1906.61036	1812.0426	PPFIA3	3504.78163
799.885051	840.505069	858.621821	945.791444	963.62443	SERTAD1	1085.26389
7916.35776	8265.88691	11385.2634	10894.9163	10788.3069	CRTAP	10547.7999
4123.16438	4345.00255	5151.73093	5088.67731	4972.62357	VGLL4	3145.55786
2988.15254	3054.97638	3533.67825	3531.45564	3826.8125	BLVRA	5111.36114
7071.54362	6659.98104	7182.04606	7433.90197	7734.00145	ATF4	9053.21175
993.595704	982.982276	1168.5936	1121.42501	1098.47827	KLF16	1347.32952
3150.92841	3235.00571	4073.02914	3873.3306	3704.46167	BRPF3	3908.84265
5590.35685	5756.52092	5804.22152	6409.2163	6431.90282	PTMS	3683.22792
1348.60914	1379.48861	1619.60253	1498.051	1538.76264	SH3BP5L	2555.23175
13619.4056	13603.812	17522.7045	16866.4576	16476.2809	PAK2	33471.4621
6424.85939	6204.93756	7710.54794	7788.92597	7468.75914	EIF4G3	4389.32196
4375.06188	4866.3145	5224.57429	5056.74393	5049.42774	PKP3	5894.06808
12054.253	12050.9209	14206.0065	14518.4152	15146.4971	SUMO3	9461.72691
4638.00752	4965.7172	5881.71446	5888.89013	5589.73615	MADD	4338.02984
2060.84565	2184.6505	2600.66321	2539.64257	2444.33741	DAB2IP	9191.27061
1945.94503	2334.85902	2433.27845	2290.75008	2361.28174	PLEKHH3	5154.48575
2674.38547	2874.94704	3211.30761	3229.02779	3176.65625	PTK7	6911.49661
966.343635	1020.53441	1128.29727	1100.76224	1119.91199	CLSTN3	2050.35611
8020.21024	8581.76661	9609.12507	9659.84608	9422.80015	TM7SF2__VI	13479.239
1678.58014	1690.95041	1959.02163	1989.26145	1859.37541	CRY2	2176.86552
3258.4636	3355.39343	3981.58747	4190.78592	4072.40723	CCDC106__Z	2422.67835
5179.36619	5304.79087	6250.58087	6103.9708	5846.04775	PCDHA1__PC	7458.41401
1207.92954	1189.519	1515.76199	1545.01184	1479.81991	SNX18	3216.37064
5270.69745	5445.05912	6290.87721	6689.10295	6907.017	DECR2__NM	5668.8537
7665.93335	8176.42448	9940.79487	9682.38729	9350.46134	ADGRG1	11190.3478
22433.6086	24179.155	28627.4434	27669.3306	27665.577	JUP	50976.0677
12851.1919	12364.5917	14719.0098	14769.1862	15300.1054	ANAPC7__AI	17729.9216
2621.35442	2522.61968	3288.80055	3191.45911	3183.80083	YAP1	11884.2967
7029.56071	7666.15728	8941.13589	9015.54327	8977.15734	NACC1	5459.27361
2804.75348	2723.63404	3225.25634	3263.77882	3129.32345	BSDC1	3758.81547
6300.38373	6568.30966	7476.51925	7256.38997	7019.54404	LSS	5600.79765
24463.5194	24909.2127	26781.5615	26559.1762	26749.2854	PSAP	30022.4105
4754.38122	4768.01627	5878.61474	5656.90355	5578.12622	SRPK2	2939.00844
4951.77459	5028.67225	5627.5376	5684.14084	5429.87631	MLXIP	4525.56703
14733.0577	15482.5231	18168.9957	18025.4512	17977.5346	IRAK1	5301.24062
1903.22557	1939.45716	2129.50611	2120.75182	2106.75628	PHF20	8647.2459
3856.53603	4104.22712	4330.30572	4165.42707	4363.54862	SEZ6L2	7516.86329
6219.36406	6348.51924	7535.41389	7428.26667	6979.35581	PCNT	6723.56855
4106.2239	4073.30183	4641.82735	4796.58084	4538.59069	MARK2	7147.05478
8459.18951	8423.82676	10382.5047	10284.4253	9816.6448	SETD7	7769.91114
5426.84444	5565.44684	6112.64343	6281.4828	6299.7282	MAF1	4704.70323
6332.79159	6252.42996	7264.18858	7064.78972	7264.2457	MCFD2	4076.7365

3765.20477	3514.43775	4124.17449	4084.65441	3940.23261	RMND5A	4817.93479
12505.7535	13216.1415	15129.7224	14674.3253	14749.0801	PPP2R1A	11188.0948
2862.94033	2913.60364	3473.23376	3282.56316	3181.12161	CNNM4	4330.38446
5959.36459	6120.9975	6464.4614	6493.74583	6740.90565	C7orf50	6080.96825
15273.6798	15648.1943	16684.2309	17055.2402	16493.2493	NFE2L1	19292.0522
4229.22649	4227.92826	4541.08653	4536.41775	4514.47775	PRMT2	3023.68903
6791.65751	6770.42848	7989.52254	7457.38239	7202.62375	UBE3C	5224.89198
6217.89098	6470.01143	6771.33346	6655.29114	6772.16316	AC002310.11	6178.33488
18323.7019	17968.6951	21109.078	20680.6175	20632.6369	PTP4A1	8453.13124
7598.90799	7740.15706	8376.98726	8754.44097	8570.80969	CCDC85C	7745.04278
16200.2502	15249.479	14367.1918	14474.2721	14908.0469	KIAA0391__F	16040.2862
18500.4721	18192.9034	17129.0404	17033.6382	16728.1272	AZIN1	15478.1517
12700.2007	13124.4701	11723.1326	11670.7095	11440.2493	TPBG	6432.9952
24887.0313	23874.3201	21718.1725	21081.6631	21111.3234	MORF4L2	11517.5192
11675.6702	11434.6242	9740.86308	9786.64037	10182.8042	BUB3	7027.50927
4277.10174	3937.45147	3541.42755	3827.30897	4139.38761	SSBP1	3658.6343
1783.16916	1772.68152	1565.35747	1520.5922	1480.71298	ABCD3	2891.30914
3793.19338	3714.34763	3116.76621	3014.88633	2917.66544	CTC-432M15	5344.18796
3561.18253	3289.12496	2967.97976	3064.66483	3255.24657	PLP2	8563.68432
2492.4595	2342.59034	2059.76246	2070.97332	2016.55604	DDAH1	3964.70566
5513.0199	5534.52155	4801.46282	4618.12963	4893.14018	DARS	4188.48132
933.935769	1011.69861	858.621821	854.687403	904.681694	WDR76	2048.59307
2708.26642	2779.96223	2413.13028	2323.62268	2308.5905	NARF	2393.68815
1937.10652	1941.66611	1793.18673	1784.51216	1728.09386	XRN1	3898.39748
4966.50543	4903.86663	4417.09782	4168.24472	4055.43887	NR2C2	3922.83581
7678.45457	7317.14334	6183.93694	6030.71188	6290.79749	SRSF11	8509.43125
1082.71733	1191.72795	976.411096	891.316863	976.127435	E2F2	1163.29959
15100.5924	15693.4777	13502.3706	13685.3298	13658.6395	PFKP	10384.4218
142928.263	137351.34	125949.282	121642.68	129011.361	MT-ND6	154147.624
1459.0905	1475.57789	1300.3316	1267.00363	1203.86073	GK5	4533.07986
1234.44507	1237.0114	1024.45672	970.211085	992.202727	RBL1	1682.45417
1721.2996	1728.50254	1628.90169	1484.90196	1460.17233	PLEKHA1	1394.45539
3270.98482	3465.84088	2682.80573	2696.49179	2581.87046	SEMA3F	8681.63892
27352.9753	28631.2916	23753.1372	23685.1724	24212.0685	GPI__PDCD2	11263.2403
849.233392	789.699244	706.735651	740.102938	707.312835	CFL2	2362.72045
114284.866	114324.152	92664.5127	94236.3303	95765.8711	GAPDH	51252.2825
4547.41281	4550.4348	4237.31419	4052.72104	3830.38478	KNTC1	6443.36027
2962.37355	2931.27524	2645.60911	2478.59347	2453.26813	PLEKHH1	3043.64605
2705.32025	2786.58908	2050.4633	2059.70272	1988.87081	PRSS53__RP	2958.38963
5037.95005	4999.95591	4251.26292	4166.36628	4100.09246	SLC25A36	5832.40752
30702.7701	29838.4822	24676.8531	25270.5706	24979.2172	HNRNPH1	19150.4905
18179.3396	18504.3652	16685.7807	16579.9964	15600.1775	PREX1	32377.3462
81045.4435	84340.9837	69996.2767	68995.8146	69310.4063	ENO1	71511.0368
4480.38745	4485.27081	3945.94072	3805.70698	3681.2418	BBX	6394.06304
3640.72911	3616.04941	3048.57242	3121.01785	3127.53731	SOD2	4859.41115

2175.74626	1978.11377	1729.64251	1679.31986	1789.71581	ZBTB25	1726.74782
4925.9956	4928.16507	3787.85511	3540.84781	3610.68913	BNIP3L	5425.87975
3072.11837	2992.02133	2498.37252	2334.89328	2322.87965	DCUN1D3__I	4421.70764
1884.81201	1805.81575	1608.75352	1525.28829	1436.95246	IL6ST	29510.8287
41732.4926	44409.8138	35942.7774	31599.0143	33940.2992	IFFO1	16786.3023
3536.87663	3325.57262	2879.6378	2879.6391	2909.6278	HELLS	3824.95452
5042.3693	4699.53886	4455.84429	4308.18804	4292.10288	SNHG1__SN	5869.76939
3325.48896	3209.6028	2634.7601	2641.078	2524.71387	ZNF292	2019.47915
18245.6284	17717.9794	15016.5827	14562.5584	14835.7081	MAL2	10739.4311
592.916636	583.162519	441.709782	464.912378	470.648818	CP	1156.98001
738.015489	777.550025	621.493412	648.059679	651.942386	FBXL20	1755.58088
8867.23401	8660.1843	8107.31182	7875.33392	7866.17607	AF011889.5_	2935.03931
914.785666	915.609333	728.433675	695.020525	757.324853	PIGA	1012.64178
7467.0669	7722.48547	5139.33206	5341.32666	5405.76337	QSOX1	9003.71831
2138.1826	2182.44155	1494.06396	1390.04105	1386.04737	SLC9A8	1966.73501
5415.05976	5403.08909	4551.93554	4522.3295	4416.23986	NKTR	8076.57745
1179.94093	1148.65345	864.821257	916.67572	854.669675	HSD17B12	6652.57935
7840.4939	7748.99286	5816.62039	5812.81356	5629.92438	CCNL1	6496.79392
798.411966	832.773748	537.801032	547.563468	576.924358	AGR3	4080.11002
1474.55789	1444.6526	1114.34854	1117.66814	1115.44663	CRIM1	11761.0538
2021.07236	2028.9196	1449.11806	1483.96274	1465.53076	NPIPB5	1356.42887
747.59054	710.177082	522.302443	592.64588	627.829448	FUT11	860.094168
1042.94405	941.012246	824.524926	805.848123	759.110997	ZGRF1	748.787264
1297.78772	1272.35459	948.513636	942.034577	879.675685	ZNF37BP	1792.34103
2745.09354	2668.41031	2317.03903	2336.77171	2369.31938	FAM217B	2436.62676
2516.7654	2257.54581	1811.78503	1753.518	1904.029	CCDC125	3055.11525
3511.83418	3444.85586	2952.48117	2701.18788	2767.62939	AK4	4782.73102
2074.83995	2006.83011	1669.19802	1550.64715	1570.02016	TACC1	8526.99846
17246.8769	16913.922	13161.4016	12639.9814	12847.7304	RDX	8688.91115
6295.96447	6106.63933	5540.74551	5463.42486	5827.29324	TMEM167A	8708.92681
2104.30165	2001.30773	1253.83584	1294.24092	1352.11065	FAM162A	1817.07697
407.30795	424.118195	288.273752	330.604358	283.103748	HLA-F	525.996922
15007.788	14696.1373	13691.4534	13279.5881	12944.1821	STAT1	7299.14437
10150.2909	10123.613	9186.0136	8860.57248	8669.94066	CA12	57219.7933
799.148509	823.937952	689.687203	585.132145	632.294807	ATAD5	1202.50464
6719.47636	6526.33963	5858.46658	5716.07421	5623.67288	LRRC58	5657.07165
35119.0784	34528.0808	25230.1528	25539.1867	25482.9096	STC2	69549.4629
938.355023	968.624108	733.083252	714.744081	705.526691	KCNK5	1225.54551
7992.22163	7995.29067	7696.59921	7343.73714	7231.20205	PGR	49373.0069
29801.9788	29498.3041	21908.8052	21985.1898	21837.3907	PGK1	24432.9344
3841.06864	3705.51184	3022.22482	3029.9138	2997.14883	P4HA1	3482.78721
104662.676	109312.047	68372.0246	68777.9163	69238.9606	ALDOA	63023.2174
204.022246	226.417266	145.686735	158.727661	152.715271	CCBL1	336.26485
3624.52517	3545.36304	2307.73988	2515.22293	2672.07071	PBX2	5017.71985
3475.74361	3492.34826	2222.49764	2248.48532	2342.52723	BHLHE40	10509.5055

1365.54962	1545.15978	926.815612	918.554154	896.644048	CLSTN2	3632.87544
771.89644	741.102367	519.202726	508.116357	551.918348	BCL6	6535.94175
1771.38448	1760.5323	1145.34571	1079.16025	1101.15748	SFXN3	2255.82274
1315.46473	1218.23534	1074.05221	1069.76808	1067.22075	ZBTB37	1244.07292
332.180624	339.073661	252.626998	259.223872	260.776954	CYP4V2__KLI	316.324236
4464.18351	4375.92784	3806.45342	3795.3756	3757.1529	DUSP4	2737.23358
782.944576	884.684048	650.940731	621.761605	598.35808	SCARA3	2349.85127
349.1211	313.670749	261.926151	227.290496	206.299577	LDHAP4	400.957057
3686.39473	3669.06418	2546.41814	2504.89154	2559.54367	C1orf21	867.442886
13399.916	13002.9779	7778.74173	7733.51217	7998.35069	PRSS23	335.513038
32038.1215	31693.9993	19731.2534	19866.3164	20174.4911	LDHA	33915.6111
1155.63503	1202.77269	804.37676	826.510895	814.481446	GPR37L1	463.735084
5351.71711	5216.43291	3352.34476	3347.36912	3543.70875	PHLDA1	3149.08193
807.250475	833.878223	418.461898	419.829966	417.064512	ALDOC	3008.71952
5574.15292	5506.90969	2391.43226	2401.57768	2411.29376	PAPSS2	2863.81517
20229.8737	20251.6438	12589.5037	11996.6178	12075.2233	FOS	1634.71018
715.919217	827.251376	337.869236	355.023998	368.838637	LAMB3	560.369142
933.935769	979.668852	444.809499	483.696717	445.642809	KRT15	2391.65398
5996.92826	6037.05744	3200.45859	3080.63152	3374.02511	BNIP3	2673.57791
850.706477	859.281135	406.063027	371.929903	413.492225	EFEMP1	15824.8754
380.055881	423.013721	181.333489	199.113988	181.293568	FHL2	10617.5186
1682.26285	1675.48777	1018.25729	1080.09947	1075.2584	WISP2	658.7283
847.760307	849.340865	446.359358	426.404484	422.422943	PFKFB4__UC	1242.72043
98.6966822	99.402702	71.2935086	69.5020525	40.1882293	MSS51	104.108139
10194.4834	10245.1052	6822.4788	6890.09537	6694.46592	EGR1	185.156022
993.595704	1040.41495	489.755407	478.061415	446.535881	PDK1	890.661269
86.1754613	83.9400595	30.9971777	40.3863278	27.6852246	PRKAA2	929.754935
1026.74011	1061.39996	636.992001	548.502685	481.365679	CD109	230.276873
357.959609	410.864502	167.384759	152.153142	169.683635	SPAG4	498.890111
654.786198	632.86387	561.048916	537.232082	541.201487	DDX60	437.398095
8475.39345	8634.78138	5743.77702	6252.36708	5869.26761	IFI6	1853.10283
8075.45092	8100.21574	7076.65566	7301.47238	7403.5649	IFI27	344.075155
984.757195	988.504648	790.42803	789.881435	859.135034	OAS1	486.864325
41.2463747	55.2237233	21.6980244	13.149037	15.1822199	SCNN1G	196.439612
2739.93774	2588.88815	931.465189	1037.8347	934.153062	FOSB	358.009242
589.233924	636.177293	550.199903	489.332018	478.686464	OAS2	1456.43151
1431.83843	1546.26425	269.675446	281.765078	297.392896	PPFIA4	333.095042
186.345228	187.760659	57.3447787	42.2647617	56.263521	IFI44	49.2803921
2947.6427	2713.69377	420.011757	450.824124	501.013258	UBE2T	2025.54451

log2FoldChai	lfcSE	stat	pvalue	padj	Control.0	Control.1
1.74163466	0.22049995	7.89857167	2.82E-15	1.06E-13	106.056387	100.257588
1.88533758	0.55308324	3.4087773	0.00065255	0.00365387	75.9031002	124.182694
1.35783827	0.09698536	14.0004462	1.55E-44	4.24E-42	722.639105	738.260418
1.12090494	0.21390251	5.24026074	1.60E-07	2.00E-06	141.408516	120.764821
0.76087426	0.05459952	13.9355474	3.85E-44	1.02E-41	9330.88248	9211.16586
1.62544586	0.12440205	13.0660694	5.15E-39	9.77E-37	375.356427	356.59801
0.50628493	0.09858581	5.13547464	2.81E-07	3.41E-06	955.547248	914.850487
0.38397179	0.08896341	4.31606431	1.59E-05	0.00013474	1447.35775	1408.16339
0.89939035	0.06005829	14.97529	1.07E-50	4.04E-48	5006.48531	4901.22888
1.13230094	0.18711392	6.05139867	1.44E-09	2.54E-08	182.999255	169.754324
0.39574819	0.10962427	3.61004181	0.00030615	0.00186832	717.440262	718.892475
0.56826064	0.14637358	3.88226235	0.00010349	0.00071577	359.7599	341.78723
0.46504891	0.13874947	3.35171674	0.00080312	0.00435537	406.549482	436.348364
1.01370532	0.09774269	10.3711628	3.35E-25	3.18E-23	898.359981	963.83999
1.89331569	0.06478338	29.22533	9.24E-188	7.72E-184	1680.26589	1622.35005
0.72768964	0.12684304	5.73693015	9.64E-09	1.48E-07	460.617444	445.46269
0.61399029	0.24306529	2.52603034	0.01153595	0.04238562	149.726663	159.500708
0.55853527	0.13866752	4.02787389	5.63E-05	0.0004182	385.754112	410.144677
0.44985478	0.1373811	3.27450267	0.00105848	0.00553532	447.100453	521.795172
0.49081042	0.08657319	5.66931193	1.43E-08	2.13E-07	1316.34692	1373.98467
0.85418657	0.0866041	9.86311926	6.02E-23	4.83E-21	1091.75692	1108.52992
0.52600469	0.07000133	7.51420955	5.73E-14	1.82E-12	3061.07845	3047.60281
0.60740474	0.11451782	5.30401959	1.13E-07	1.46E-06	589.548737	594.709781
0.56016247	0.15912875	3.52018392	0.00043125	0.00253186	291.135179	339.508649
0.85661457	0.14311999	5.98528939	2.16E-09	3.72E-08	318.16916	342.926521
0.6124987	0.08191671	7.47709091	7.60E-14	2.39E-12	1481.67011	1528.92821
0.78855962	0.14913434	5.28757927	1.24E-07	1.59E-06	354.561057	394.194606
0.23125658	0.07473744	3.09425354	0.00197309	0.00942961	2025.46903	1972.11232
0.98549661	0.10368303	9.50489791	2.00E-21	1.44E-19	807.900121	769.021268
0.42670143	0.0913672	4.67018155	3.01E-06	3.00E-05	1101.11484	1107.39063
0.54581767	0.10819039	5.04497383	4.54E-07	5.29E-06	718.480031	725.728219
0.48206845	0.15829773	3.04532769	0.00232427	0.01080197	335.845224	336.090777
0.21971283	0.06521434	3.36908781	0.00075417	0.00413829	4928.50267	5148.45498
0.36618032	0.06955325	5.26474783	1.40E-07	1.77E-06	2491.28532	2501.88253
0.39627542	0.11358004	3.48895302	0.00048492	0.00280332	774.62753	881.811055
0.46220292	0.14779777	3.12726586	0.0017644	0.00857208	382.634807	364.573046
0.40595348	0.05931447	6.84408846	7.70E-12	1.85E-10	5027.28068	4991.23286
0.92634876	0.09642339	9.6070963	7.46E-22	5.54E-20	812.059195	828.264388
0.49086984	0.06856559	7.15912851	8.12E-13	2.22E-11	2304.12699	2369.7248
0.34791012	0.11607885	2.99718797	0.00272483	0.01241483	628.020172	626.609922
0.35892771	0.11753709	3.05374005	0.00226008	0.01058018	660.252995	619.774178
0.53990626	0.11540958	4.67817563	2.89E-06	2.89E-05	593.707811	616.356306
0.33730184	0.0496353	6.79560337	1.08E-11	2.53E-10	9060.54268	9123.44047
0.27015331	0.07486973	3.60831164	0.0003082	0.00188013	1928.77056	1909.45133

0.37566171	0.07328596	5.12597107	2.96E-07	3.55E-06	2119.0482	2150.98097
0.66120882	0.09383373	7.04660057	1.83E-12	4.82E-11	1285.15386	1260.05559
0.35377941	0.07936648	4.45754207	8.29E-06	7.55E-05	1733.29408	1794.38296
0.42183849	0.07739855	5.45021196	5.03E-08	6.91E-07	1862.22538	1838.8153
0.25778745	0.06970933	3.69803368	0.00021728	0.00137957	2399.78569	2364.02834
0.24149535	0.0665075	3.63109938	0.00028222	0.00173751	3657.90557	3648.00904
0.38212469	0.07005489	5.45464683	4.91E-08	6.76E-07	2287.49069	2316.17813
0.25129292	0.05585854	4.49873758	6.84E-06	6.38E-05	5576.27844	5796.71143
0.39010101	0.06461964	6.03688034	1.57E-09	2.76E-08	2961.26068	3066.97075
0.42145528	0.06663865	6.32448731	2.54E-10	5.02E-09	2573.42703	2604.4187
0.59435232	0.0727241	8.17270144	3.02E-16	1.27E-14	3179.61206	3218.49642
0.38293863	0.06196944	6.17947559	6.43E-10	1.20E-08	3378.20784	3400.78294
0.43506935	0.1050244	4.1425551	3.43E-05	0.00026895	849.490861	880.671764
0.46626557	0.07654844	6.09111787	1.12E-09	2.01E-08	2394.58685	2231.87062
0.30699258	0.07231905	4.24497536	2.19E-05	0.00018041	5425.51201	5928.86916
0.30750256	0.08317911	3.69687235	0.00021827	0.00138466	1577.32881	1485.63516
0.55601225	0.06136471	9.06078179	1.30E-19	7.87E-18	4491.7999	4687.04222
0.34107371	0.0554935	6.14619207	7.94E-10	1.46E-08	16011.3951	16319.201
0.45844208	0.07718871	5.93923773	2.86E-09	4.81E-08	1779.0439	1806.91516
0.21764467	0.07742165	2.81116022	0.00493632	0.02074248	2267.73509	2448.33586
0.54967284	0.11547083	4.76027426	1.93E-06	1.99E-05	663.372301	595.849072
0.33267006	0.06387035	5.20852115	1.90E-07	2.35E-06	3441.63372	3645.73046
0.43188518	0.05791872	7.45674573	8.87E-14	2.77E-12	4945.13897	4804.38917
0.34344925	0.10443457	3.28865473	0.00100667	0.00529924	817.258038	791.807084
0.19843399	0.0527571	3.76127515	0.00016905	0.00110169	7435.38452	7259.56077
0.29242385	0.07323336	3.99304134	6.52E-05	0.00047704	2285.41115	2206.80622
0.41784802	0.0918662	4.54844124	5.40E-06	5.16E-05	1099.0353	1100.55488
0.48320857	0.09913587	4.87420499	1.09E-06	1.18E-05	945.149563	911.432615
0.30829177	0.05386488	5.72342791	1.04E-08	1.59E-07	6470.47935	6533.83256
0.30191453	0.09534752	3.16646439	0.00154304	0.00764337	2183.51384	2060.977
0.35169078	0.05679662	6.19210764	5.94E-10	1.12E-08	4929.54244	5039.08307
0.20588692	0.0763712	2.69587128	0.00702048	0.02793973	1881.98098	1935.65502
0.32151876	0.08160587	3.93989761	8.15E-05	0.00057945	1478.5508	1497.02807
0.45400857	0.05506616	8.24478345	1.65E-16	7.18E-15	5215.47878	5151.87285
0.23388104	0.07343889	3.18470296	0.00144903	0.00724861	2204.30921	2323.01388
0.21708329	0.06204717	3.49868132	0.00046757	0.00271549	4245.37477	4369.1801
0.2660788	0.07684575	3.46250529	0.00053517	0.00306129	1900.69681	2013.12679
0.38505368	0.12654147	3.04290521	0.00234306	0.01086813	572.912441	571.923966
0.3896636	0.06059853	6.43024826	1.27E-10	2.62E-09	36871.2306	36835.5491
0.6226729	0.0750583	8.29585702	1.08E-16	4.84E-15	1958.92385	1819.44736
0.29323599	0.04720321	6.2122047	5.22E-10	9.92E-09	18264.5734	17801.4183
0.39446799	0.07821618	5.04330409	4.58E-07	5.33E-06	1629.31723	1630.32509
0.18400731	0.05981426	3.07631158	0.00209579	0.00992513	4281.76667	4390.82662
0.27089377	0.05992618	4.52045753	6.17E-06	5.79E-05	6525.58708	6596.49355
0.33460073	0.08242009	4.05969852	4.91E-05	0.0003687	1475.4315	1564.24623

0.18523844	0.06081628	3.04586925	0.00232009	0.01079154	9278.89406	9385.47735
0.36593088	0.06621964	5.52601741	3.28E-08	4.63E-07	2773.06258	2697.84054
0.33430576	0.06447804	5.1848004	2.16E-07	2.65E-06	3041.32285	3160.39259
0.31573332	0.10306399	3.06346881	0.00218787	0.01028803	930.592804	1004.85446
0.16681057	0.05062781	3.29484051	0.00098478	0.00520035	9994.25478	9884.4867
0.22631791	0.06752534	3.35159967	0.00080346	0.0043558	2821.9317	2976.96678
0.38894635	0.0612958	6.34539925	2.22E-10	4.42E-09	4530.27134	4323.60847
0.24020351	0.05349432	4.49026182	7.11E-06	6.61E-05	8312.94913	8291.75821
0.38419079	0.09541616	4.02647515	5.66E-05	0.0004205	1135.4272	1204.23034
0.31935909	0.06682448	4.7790735	1.76E-06	1.83E-05	3493.62215	3460.02606
0.44609412	0.08510232	5.2418562	1.59E-07	1.99E-06	3134.90202	3104.56734
0.30481823	0.07070704	4.31100242	1.63E-05	0.00013744	2298.92815	2272.88508
0.14950895	0.04497253	3.3244504	0.00088593	0.00473642	32071.6593	31403.4107
0.22902024	0.06292696	3.63946169	0.00027321	0.00168952	4139.31838	3941.94606
0.46167516	0.0579989	7.96006693	1.72E-15	6.71E-14	4939.94013	4979.83995
0.28637896	0.05734555	4.99391775	5.92E-07	6.72E-06	8505.3063	8549.23792
0.18127405	0.05991104	3.02572023	0.00248042	0.01140705	4083.17088	4047.9001
0.17686204	0.05096475	3.47028143	0.00051991	0.00298423	8586.40824	8670.00275
0.24542855	0.05984043	4.10138329	4.11E-05	0.00031481	4708.07175	4728.05669
0.23082613	0.05350913	4.31377062	1.60E-05	0.00013608	6312.43454	6407.37128
0.21898904	0.07866502	2.78381721	0.00537233	0.02229417	1841.43001	1948.18721
0.24021833	0.05458524	4.40079246	1.08E-05	9.58E-05	12149.6949	12573.2129
0.30635503	0.07540959	4.06254722	4.85E-05	0.00036505	1899.65704	1993.75884
0.21308331	0.08076691	2.63825011	0.00833351	0.03240098	2269.81463	2220.47771
0.22272253	0.05397826	4.12615256	3.69E-05	0.00028658	6904.06281	6861.9483
0.22338136	0.06517748	3.42727801	0.00060967	0.0034368	2972.69813	2963.29529
0.19468936	0.06001375	3.24407948	0.00117831	0.00609894	5162.45058	5413.90973
0.1623711	0.05452182	2.97809407	0.00290047	0.01309012	10375.8498	10744.6512
0.18478257	0.04455798	4.14701441	3.37E-05	0.00026439	47107.7515	48323.0179
0.16398816	0.05055198	3.24395123	0.00117884	0.0060998	16832.8122	16616.5559
0.17563538	0.05069116	3.46481277	0.0005306	0.00303827	11305.4029	11015.8024
0.16299763	0.05718728	2.85024288	0.00436859	0.01867157	5117.74054	5183.773
0.20330316	0.0649154	3.13181725	0.00173728	0.00846493	3562.24687	3424.70805
0.25189554	0.05781359	4.35702976	1.32E-05	0.0001141	5073.03049	5151.87285
0.29738408	0.04584598	6.48659004	8.78E-11	1.83E-09	26925.845	26954.4803
0.17957428	0.06935472	2.58921486	0.00961951	0.03650866	2743.94906	2767.33728
0.18291468	0.06608938	2.76768661	0.00564557	0.0232693	4221.46009	4254.11173
0.21449556	0.060648	3.5367294	0.00040512	0.00239696	4734.06596	5082.37612
0.34749314	0.05300997	6.55524146	5.56E-11	1.18E-09	7591.34979	7628.69098
0.333858	0.05263339	6.3430834	2.25E-10	4.48E-09	6652.43884	6648.90092
0.26125158	0.06985505	3.73990986	0.00018409	0.0011881	6041.05496	6188.62745
0.19906603	0.05886742	3.3815993	0.00072065	0.00397919	6373.78088	6937.14149
0.20300039	0.06127184	3.31311084	0.00092264	0.00491263	7335.56674	7109.17439
0.19588384	0.06721819	2.91414928	0.00356659	0.01572257	4371.18676	4403.35882
0.17100321	0.06419806	2.66368177	0.00772906	0.03034044	3701.57585	3970.42833

0.26644101	0.05817065	4.58033433	4.64E-06	4.47E-05	4396.1412	4352.09073
0.16717658	0.04942125	3.38268644	0.00071781	0.00396741	10599.4001	10482.6144
0.16710914	0.0636783	2.62427128	0.00868346	0.03353522	4141.39792	4015.99996
0.21298952	0.06162386	3.4562833	0.00054768	0.00312641	5497.25604	5771.64703
0.13949081	0.04605608	3.02871669	0.00245595	0.01131009	18193.8691	18526.0072
0.17868038	0.06897316	2.5905785	0.00958148	0.03638913	2900.9541	2773.03373
0.15616148	0.06214597	2.51281769	0.01197712	0.04375631	4900.42892	4982.11853
0.22304079	0.0559912	3.98349737	6.79E-05	0.00049295	5708.32904	5696.45384
0.13235208	0.05235606	2.52792311	0.01147395	0.04220422	7938.63247	8192.63991
0.13803416	0.0525546	2.62649085	0.00862703	0.03337904	7361.56095	7387.16134
-0.181926	0.05285666	-3.4418754	0.0005777	0.00328206	16867.1245	17237.4693
-0.2252813	0.04754241	-4.7385321	2.15E-06	2.20E-05	16521.9214	16845.5533
-0.1928858	0.05970985	-3.2303856	0.00123623	0.00635153	6899.90374	6827.76957
-0.1445948	0.04920629	-2.9385426	0.00329759	0.01468792	11941.7412	12247.3758
-0.1619268	0.05591388	-2.896003	0.00377949	0.01650426	7487.37294	7357.53978
-0.2369115	0.06441622	-3.677823	0.00023523	0.00147877	3948.00098	3970.42833
-0.2319528	0.06888823	-3.3670897	0.00075966	0.00416266	3081.87382	3166.08904
-0.1662648	0.06397762	-2.5987961	0.00935513	0.03561048	5618.90895	5681.64306
-0.1655608	0.05335875	-3.1027856	0.00191709	0.00919881	8997.1168	9114.32615
-0.2455472	0.06134753	-4.0025606	6.27E-05	0.00046046	4318.15856	4284.87258
-0.2544235	0.06141502	-4.1426916	3.43E-05	0.00026892	4550.02694	4565.13811
-0.3463061	0.07761738	-4.4617084	8.13E-06	7.43E-05	2247.97949	2337.82466
-0.2969875	0.07446194	-3.9884475	6.65E-05	0.00048404	2704.43786	2574.79714
-0.4141665	0.07782558	-5.321727	1.03E-07	1.34E-06	4515.71458	4388.54804
-0.3166511	0.06980677	-4.5361097	5.73E-06	5.44E-05	4425.25472	4274.61896
-0.1935247	0.05112886	-3.785039	0.00015368	0.00101542	9111.49133	9047.10799
-0.3003046	0.09436408	-3.1824036	0.00146058	0.00729233	1261.23919	1306.76651
-0.1296258	0.04933971	-2.6272097	0.00860883	0.03331632	10833.348	10867.6946
-0.350035	0.04287524	-8.1640363	3.24E-16	1.36E-14	174918.175	170596.26
-0.1739035	0.06336305	-2.7445575	0.00605926	0.02467879	4776.69647	4832.87144
-0.2769203	0.0854481	-3.2408011	0.00119194	0.00615426	1780.08367	1906.03346
-0.2369163	0.08853164	-2.6760638	0.00744925	0.02940093	1547.17552	1469.68509
-0.2110956	0.05137518	-4.1089027	3.98E-05	0.00030614	9302.80873	9329.6521
-0.3133719	0.05047299	-6.208705	5.34E-10	1.01E-08	12634.227	12330.544
-0.3999467	0.07275765	-5.496971	3.86E-08	5.39E-07	2679.48341	2697.84054
-0.1815923	0.04611254	-3.9380238	8.22E-05	0.00058319	55238.7411	53710.724
-0.5105677	0.06573225	-7.7673855	8.01E-15	2.82E-13	7541.4409	7597.93013
-0.3903182	0.07117955	-5.4835719	4.17E-08	5.77E-07	3382.36692	3521.54776
-0.2638921	0.07026333	-3.7557582	0.00017282	0.00112405	3280.46961	3177.48195
-0.2741959	0.0561359	-4.8845022	1.04E-06	1.13E-05	6444.48514	6324.20305
-0.3362103	0.04551761	-7.3863776	1.51E-13	4.62E-12	21336.0495	21408.4128
-0.218944	0.04603708	-4.7558177	1.98E-06	2.04E-05	34611.8137	35044.584
-0.4241181	0.04493893	-9.4376546	3.81E-21	2.60E-19	82255.046	81642.7157
-0.3700129	0.0656315	-5.6377331	1.72E-08	2.53E-07	7130.73235	7284.62517
-0.3309227	0.05843541	-5.663052	1.49E-08	2.20E-07	5348.56914	5479.98859

-0.3140389	0.08318421	-3.7752222	0.00015987	0.00104879	1933.9694	1892.36197
-0.2044803	0.05707039	-3.5829488	0.00033974	0.00204786	5726.00511	5893.55114
-0.2702621	0.05949095	-4.5429109	5.55E-06	5.29E-05	4802.69068	4867.05016
-0.2170106	0.05366517	-4.0437888	5.26E-05	0.00039306	31436.3607	32011.792
-0.1660895	0.04942308	-3.3605652	0.00077783	0.00423472	18233.3804	17268.2302
-0.5839465	0.06305295	-9.2612087	2.02E-20	1.31E-18	4655.04356	4522.98435
-0.4794664	0.05576138	-8.5985385	8.07E-18	4.09E-16	6798.00643	6875.61979
-0.2980931	0.08384509	-3.5552843	0.00037757	0.00224438	2200.15014	2252.37785
-0.5195837	0.05223387	-9.9472576	2.59E-23	2.12E-21	12542.7274	12765.7531
-0.2335279	0.0942847	-2.4768374	0.01325523	0.0474806	1244.60289	1256.63772
-0.5251371	0.08099877	-6.4832731	8.98E-11	1.87E-09	2073.29838	2068.95204
-0.5601668	0.06816315	-8.21803	2.07E-16	8.81E-15	3510.25844	3483.95117
-0.2750908	0.10123859	-2.7172527	0.00658263	0.02651947	1079.2797	1138.15148
-0.1520844	0.05159062	-2.9479084	0.00319932	0.01429031	9344.39947	9611.05692
-0.2447143	0.07730704	-3.1654847	0.00154825	0.00766461	2103.45167	2163.51317
-0.4951024	0.06155482	-8.0432757	8.75E-16	3.52E-14	9453.57517	9440.1633
-0.1568738	0.05441187	-2.8830797	0.00393808	0.01707666	6964.36939	7064.74205
-0.3555658	0.05784055	-6.1473446	7.88E-10	1.45E-08	7222.23197	7362.09694
-0.3181234	0.07282707	-4.3682024	1.25E-05	0.00010932	4342.07324	4717.80307
-0.5062256	0.0541253	-9.3528466	8.53E-21	5.72E-19	14063.9087	13539.3315
-0.26723	0.08928563	-2.9929788	0.00276269	0.01256678	1454.63613	1508.42098
-0.4766955	0.10772494	-4.4251174	9.64E-06	8.67E-05	1017.93336	983.207933
-0.5623686	0.11446266	-4.913118	8.96E-07	9.88E-06	894.200907	890.925381
-0.2807119	0.08032228	-3.49482	0.00047438	0.00274934	1939.16825	1992.61955
-0.2401198	0.07167568	-3.3500871	0.00080786	0.00437114	2617.09731	2660.24394
-0.5872481	0.06656053	-8.8227668	1.12E-18	6.20E-17	3707.81446	3628.6411
-0.2723356	0.06010774	-4.5307914	5.88E-06	5.56E-05	5244.59229	5219.09101
-0.3897428	0.05808583	-6.709773	1.95E-11	4.40E-10	9757.18757	9582.57465
-0.465378	0.05485439	-8.483879	2.18E-17	1.05E-15	9801.89761	10353.8745
-0.5268484	0.05143877	-10.242245	1.28E-24	1.16E-22	10176.2143	10386.9139
-0.3336649	0.08128018	-4.10512	4.04E-05	0.00031076	2019.23042	2034.77331
-0.3636008	0.13575893	-2.6782823	0.00740008	0.02924192	572.912441	611.799143
-0.4415367	0.05273491	-8.372758	5.63E-17	2.58E-15	8378.45454	8436.44814
-0.5257711	0.04323521	-12.160717	5.03E-34	7.78E-32	67827.2183	67235.2447
-0.7367503	0.09512837	-7.7448006	9.57E-15	3.31E-13	1518.062	1486.77445
-0.5309874	0.06419826	-8.2710565	1.33E-16	5.85E-15	6630.6037	6738.90489
-0.7826147	0.04462496	-17.537602	7.40E-69	5.62E-66	86603.3578	89328.3713
-0.2889412	0.09437028	-3.0617811	0.00220024	0.01033192	1314.26738	1380.82041
-0.837502	0.05729609	-14.61709	2.19E-48	7.77E-46	62619.0179	64004.2161
-0.5399012	0.04820849	-11.199297	4.11E-29	4.90E-27	29093.7623	28813.8028
-0.4652196	0.06530828	-7.1234408	1.05E-12	2.84E-11	4105.00602	3974.98549
-0.3125555	0.0425212	-7.3505812	1.97E-13	5.91E-12	69211.1502	70435.5124
-0.5937057	0.16389879	-3.6223923	0.00029189	0.00178982	413.827861	395.333897
-0.4430557	0.05878525	-7.5368509	4.81E-14	1.54E-12	5724.96534	5841.14377
-0.438646	0.04926677	-8.9034871	5.41E-19	3.13E-17	12121.6211	12068.5071

-0.2617352	0.07510505	-3.4849211	0.00049228	0.00284027	3971.91566	3947.64251
-0.6376312	0.05441869	-11.717135	1.04E-31	1.46E-29	7953.18923	7960.2246
-0.5047455	0.07355596	-6.8620614	6.79E-12	1.65E-10	2681.56295	2611.25444
-0.6907888	0.09710439	-7.113879	1.13E-12	3.02E-11	1566.93112	1503.86381
-0.9698862	0.17942443	-5.4055416	6.46E-08	8.68E-07	374.316659	463.691343
-1.5305772	0.07062905	-21.670646	3.88E-104	1.08E-100	4108.12533	4026.25357
-0.9462579	0.07350856	-12.872757	6.41E-38	1.16E-35	3102.66919	3084.06011
-0.5108861	0.15361575	-3.3257405	0.00088184	0.00471642	494.929804	447.741272
-0.8029777	0.10675021	-7.5220241	5.39E-14	1.72E-12	1108.39322	1097.13701
-0.6101098	0.16518224	-3.6935557	0.00022114	0.00140072	401.35064	409.005386
-0.4168517	0.04892839	-8.5196275	1.60E-17	7.89E-16	38925.8132	38641.325
-1.1301457	0.14271779	-7.9187448	2.40E-15	9.13E-14	622.821329	650.535029
-0.4730598	0.06556443	-7.2151906	5.39E-13	1.50E-11	3646.46812	3675.35202
-0.5258111	0.06749384	-7.7905045	6.67E-15	2.40E-13	3484.26423	3618.38748
-0.2578463	0.06912969	-3.7298931	0.00019156	0.00123172	3175.45299	3061.27429
-0.8613046	0.08305944	-10.369738	3.40E-25	3.21E-23	2129.44588	2088.31998
-0.4233194	0.13325752	-3.1767017	0.0014896	0.00742268	602.025959	682.43517
-0.5022153	0.07327364	-6.8539694	7.18E-12	1.74E-10	2833.36915	2775.31231
-0.6093293	0.07641729	-7.9737097	1.54E-15	6.04E-14	3205.60627	3257.23231
-0.5804975	0.04853568	-11.960221	5.74E-33	8.64E-31	19310.5805	18622.8469
-0.2014044	0.05236219	-3.8463706	0.00011988	0.00081463	11415.6183	11301.7644
-0.8843418	0.1238472	-7.1405878	9.29E-13	2.51E-11	822.45688	887.507508
-0.5348375	0.09167643	-5.8339697	5.41E-09	8.62E-08	1483.74964	1457.15289
-0.8108947	0.29201822	-2.7768632	0.00548863	0.02270345	122.692683	142.411346
-1.2080767	0.22246179	-5.4304907	5.62E-08	7.64E-07	260.981893	256.340423
-0.6513571	0.10973864	-5.9355311	2.93E-09	4.91E-08	1114.63183	1060.67971
-0.6054072	0.11134639	-5.4371518	5.41E-08	7.39E-07	1130.22836	1111.94779
-0.8853356	0.20070387	-4.4111538	1.03E-05	9.17E-05	276.57842	321.279997
-0.9170155	0.13848977	-6.62154	3.55E-11	7.76E-10	645.696236	659.649355
-0.7952429	0.14635882	-5.4335151	5.53E-08	7.53E-07	562.514756	546.859569
-0.882372	0.09228628	-9.5612481	1.16E-21	8.54E-20	2369.6324	2439.22153
-0.7354729	0.16404867	-4.4832608	7.35E-06	6.81E-05	425.265315	435.209073
-0.6041055	0.13928628	-4.33715	1.44E-05	0.00012358	610.344107	563.94893
-0.974247	0.21573432	-4.5159576	6.30E-06	5.91E-05	276.57842	243.808224
-1.2267749	0.16742644	-7.3272471	2.35E-13	6.95E-12	462.696981	541.163115
-0.8031019	0.0888389	-9.03998	1.57E-19	9.41E-18	1899.65704	1802.358
-1.4981729	0.17022981	-8.8008845	1.36E-18	7.48E-17	498.04911	485.337867
-1.0674428	0.4250383	-2.5114039	0.0120252	0.04388397	58.2270358	75.1931907
-2.0225341	0.08570968	-23.5975	4.09E-123	1.71E-119	3216.00396	3289.13245

shRNA1.0	shRNA1.1	Gene Set_C	baseMean	log2FoldChai	lfcSE	stat
385.357779	303.472644	STMN3	225.988203	1.92715854	0.20122404	9.57717823
478.292295	259.822469	TMEM238	128.031684	0.85821096	0.27829297	3.08383987
1783.38132	1963.21858	NAALADL2	1050.43077	1.11037627	0.09412781	11.7964741
298.03138	272.293948	FGF12	260.435336	1.74482451	0.18805097	9.27846565
16331.6389	15082.1747	NUPR1	9126.46228	0.21449229	0.0330116	6.49748325
1196.93248	1060.07568	NANOS1	463.293368	0.82792904	0.13448519	6.1562842
1377.99455	1277.28726	BTG2	1269.1432	0.98209044	0.08331327	11.787923
1967.64803	1755.3606	ID1	2014.30232	1.06628009	0.06621173	16.1040976
9632.34215	8843.31757	MDK	5902.54051	0.69114757	0.03986973	17.3351476
415.000685	357.515718	PDCD4-AS1	232.854254	0.92100287	0.18940363	4.86264637
918.128928	972.775326	IL1R1	1131.4893	1.29751199	0.08751266	14.8265641
532.771149	507.17346	HIST1H2AD_	417.891104	0.6921667	0.14177225	4.88224405
559.209417	604.866709	CEP19	540.805619	0.8635187	0.12719681	6.78883923
1996.48978	1758.47847	CTXN1	1124.93628	0.72073217	0.09719935	7.41498937
6133.67807	6138.04602	SKAP2	1690.12294	0.31702304	0.07054439	4.49395089
768.312078	731.660074	CAMK1D	542.407815	0.70300655	0.12351716	5.69156975
285.212826	186.032888	RP11-47A8.5	192.778838	0.79231178	0.21038704	3.76597239
598.466238	572.648723	FAM43A	415.770876	0.36648669	0.14620093	2.50673297
696.207712	624.613217	CNIH2	534.481896	0.5088846	0.12898458	3.94531342
1960.4376	1816.67871	MAPK8IP1	1505.62713	0.54146062	0.07534394	7.18651889
2042.15588	1933.07917	HIST1H2BG	1170.40916	0.41752661	0.08458284	4.93630377
4624.29334	4166.51312	ODC1	3500.77128	0.59835789	0.05037422	11.8782551
918.930088	884.435686	HIST1H3E	685.702244	0.62234496	0.11014362	5.65030395
482.298093	445.855358	EFNB3	363.598275	0.61454567	0.15394317	3.99202959
596.863919	599.670259	PPP1R3F	373.466919	0.56593379	0.14824732	3.81749776
2212.80288	2392.4453	SESN2	1566.6849	0.36103049	0.07300158	4.945516
708.225107	582.002332	PRR7	419.489903	0.54057434	0.15267691	3.54064232
2371.43248	2320.7343	HOXC13	2366.41746	0.67475419	0.06115958	11.0326824
1664.8097	1455.00583	HIST3H2A	797.321216	0.28587849	0.10187499	2.80616947
1448.4966	1521.52038	MATN2	1265.25312	0.59857019	0.08070419	7.4168418
1095.1852	1011.22905	SAMD11	752.703786	0.36122884	0.1081712	3.33941773
509.53752	427.14814	FAM171A2	406.61506	0.72254282	0.15243987	4.73985467
6156.9117	5570.59374	BAMBI	5097.67569	0.28732211	0.04299723	6.68233976
3301.57881	3131.3804	ID3	3003.41542	0.71344766	0.05342206	13.3549262
1157.67566	1018.50408	TRIP6	869.145975	0.38326242	0.10214923	3.75198532
543.987384	484.309083	SMAD6	408.36932	0.48166075	0.14185118	3.39553572
6418.8909	6860.35248	CYFIP2	5564.51562	0.52266793	0.0409302	12.7697384
1607.1262	1508.00961	ID2	838.73263	0.31528559	0.09839592	3.20425469
3283.15214	3284.15601	IGSF9	2774.99915	0.68288455	0.05596826	12.2012834
786.73875	810.646105	TGFB1	722.901626	0.61089157	0.1068896	5.71516406
792.346867	851.17841	DYRK1B	878.196682	1.00556299	0.1001516	10.0404091
900.503416	857.414149	ZNF219	664.67878	0.49944604	0.1148849	4.34736006
11420.5304	11553.7856	METTL7A	9087.4982	0.25522176	0.0327275	7.79838936
2323.36291	2305.14495	TEAD2	1874.47272	0.19248117	0.06652855	2.89321163

2692.69749	2849.73284	KDM4A	2242.29264	0.38349451	0.06109349	6.27717465
1861.89496	2167.95869	CEP126	1292.44428	0.29805949	0.08100851	3.67936008
2179.95533	2330.08791	THRB	1926.67312	0.48310809	0.06661587	7.2521468
2392.26263	2569.12458	JADE2	1866.16787	0.27624378	0.06931131	3.98555114
2838.50854	2858.04716	SGPP1	2523.06463	0.40552715	0.05811619	6.97786836
4134.78481	4507.4002	ASAP1	4434.64853	0.73355945	0.04559302	16.0892936
2953.87553	3047.19792	NACC2	2549.67429	0.51618185	0.05767891	8.94923062
6781.81621	6753.30563	DBN1	5958.0458	0.37776012	0.03997243	9.45051617
3919.27288	3980.48023	RP11-400F19	2931.25237	0.18002606	0.05648463	3.18716898
3488.249	3445.24594	CCDC90B	2847.15472	0.49782133	0.05581566	8.91902604
5142.64362	4510.51807	ARHGAP39_	3473.27601	0.46482845	0.0603509	7.70209673
4468.86838	4369.17465	TTYH3	3530.50985	0.36228821	0.04923495	7.35835425
1105.60028	1235.71566	CADPS2	882.406095	0.3068961	0.09740987	3.15056468
3344.04027	3045.11934	HIST1H2AC	2384.97363	0.33428019	0.06308831	5.29860729
7506.06451	6531.93688	H2AFX	5967.00465	0.38530572	0.04596351	8.38286063
1929.99353	1860.32888	TCAIM	1686.28101	0.50193801	0.07222556	6.94958956
6981.30495	6506.99392	MARCKSL1	4801.40604	0.37249838	0.04730118	7.87503288
19472.9858	21487.3182	TNRC18	16919.7668	0.3743152	0.02687706	13.9269418
2538.07369	2386.20956	PODXL2	1758.49584	0.20357885	0.06879897	2.95903941
2853.73058	2625.24623	FRAT2	2438.7742	0.34497429	0.06171704	5.58961165
912.520811	932.24302	TEF	650.168981	0.33684456	0.11694894	2.88027037
4518.54027	4403.47121	MXD4	3920.64643	0.51405896	0.04909411	10.4708897
6440.52221	6715.89119	USP54	5074.86785	0.35931126	0.04565615	7.86994255
1026.28548	1015.38621	CADM4	835.898393	0.35677388	0.09957981	3.58279314
8353.69139	8510.74481	STAT6	7192.12568	0.19963019	0.03812249	5.23654585
2825.68999	2674.09286	CDKN1B	2358.34136	0.38254563	0.0603078	6.34321934
1430.06993	1510.08819	TBC1D4	1103.0325	0.26537512	0.08714799	3.04510911
1347.55049	1246.10856	MESDC1	943.131109	0.29346357	0.10395658	2.82294361
8179.83975	7919.38887	TCAF1	7703.14531	0.67877703	0.03876602	17.509586
2896.19204	2331.1272	UBE2S	2177.48463	0.32109823	0.06671543	4.81295288
6441.32337	6276.27157	PBXIP1	5000.27395	0.26499993	0.04237799	6.25324463
2230.42839	2171.07655	STK17A	2160.83302	0.56901531	0.06295108	9.03900734
1861.89496	1856.17172	LIMK1	1565.66922	0.38851033	0.07256773	5.35376196
7142.33804	7058.85685	TARS	4941.34111	0.12619717	0.04227276	2.98530712
2610.17805	2714.62516	ARHGEF18_	2241.03123	0.22863007	0.06329439	3.61216955
4857.43079	5159.03495	TLE3	4566.39001	0.40773447	0.0443437	9.1948673
2395.46727	2308.26282	POLR1B	1897.56622	0.17386009	0.06753407	2.57440579
799.557304	693.206348	SLC35D2	596.002668	0.36400156	0.11937597	3.049203
51726.0699	44826.651	HSPB1	37754.2893	0.31920259	0.04429021	7.20706937
2941.85814	2876.75438	HIST2H2AA3	1875.61818	0.23567038	0.06934991	3.39827967
22444.4868	21748.18	MAGED2	18362.2741	0.30310183	0.02611904	11.6046296
2124.67532	2160.68366	SLC37A1	1657.58213	0.30156222	0.07125028	4.23243579
4996.03141	4853.48373	SOX12	4745.69763	0.48724028	0.04349799	11.2014429
7560.54336	8277.94388	NCOA3	6852.01742	0.36878786	0.03670248	10.0480363
1927.59005	1903.97906	QSOX2	1641.32672	0.4551059	0.0728769	6.24485855

10009.6883	11219.1342	DIP2B	10219.9458	0.48790486	0.03184695	15.3203025
3513.88611	3537.74274	ANKRD40	2659.90809	0.18033119	0.05671598	3.17954817
3920.07404	3897.33704	PPFIA3	3127.04087	0.27925773	0.05337698	5.23180098
1263.42873	1142.17958	SERTAD1	962.29457	0.24176384	0.09378957	2.57772632
11350.0284	10962.4296	CRTAP	9637.03532	0.17267481	0.03177533	5.43424177
3364.06926	3419.2637	VGLL4	2844.06507	0.20472216	0.0560908	3.64983481
5953.41716	5638.14759	BLVRA	4206.72845	0.11752566	0.04713446	2.493413
10070.5765	9537.56321	ATF4	8179.16358	0.21593131	0.03378087	6.39211852
1607.1262	1442.53435	KLF16	1157.93141	0.22885786	0.08523916	2.6848911
4155.61496	4526.10742	BRPF3	3414.76583	0.20724314	0.0502984	4.12027306
4677.16988	3816.27243	PTMS	3158.4557	0.28852644	0.05190123	5.55914452
2784.83085	2864.2829	SH3BP5L	2241.85783	0.20409367	0.06102839	3.34424134
34696.621	35714.1574	PAK2	32814.3945	0.34303002	0.0213148	16.0935152
4626.69682	4849.32657	EIF4G3	4147.56769	0.32319336	0.04857309	6.65375305
7013.35134	6643.1409	PKP3	4782.29066	0.15755794	0.04288101	3.67430548
10855.7129	9936.65052	SUMO3	8282.47314	0.17738596	0.03394078	5.22633766
4575.42261	4645.62575	MADD	3937.74365	0.16990055	0.04682857	3.62813924
9649.96766	9858.70378	DAB2IP	8301.85374	0.15235422	0.0336165	4.53212571
5751.52493	5430.28961	PLEKHH3	4878.30078	0.34216721	0.044539	7.68241793
7462.00073	7464.1799	PTK7	6375.03463	0.26246474	0.03774733	6.95320104
2150.31243	2261.49477	CLSTN3	1945.17998	0.32516453	0.06676573	4.87023081
15166.7528	14027.2955	TM7SF2__VF	12217.0685	0.22406033	0.03265818	6.86077295
2391.46147	2422.58471	CRY2	1915.10481	0.21210538	0.06708304	3.16183321
2766.40418	2434.01689	CCDC106__ZI	2209.72156	0.21262211	0.06201472	3.42857464
7877.00141	8190.64353	PCDHA1__PC	6942.3395	0.27789064	0.03717047	7.47611371
3484.2432	3445.24594	SNX18	2909.24637	0.20047048	0.05837854	3.43397572
6214.5952	5884.45929	DECR2__NM	5159.14131	0.19012489	0.04305191	4.41617839
11415.7235	12225.1668	ADGRG1	10546.7694	0.25281204	0.03163506	7.99151451
53362.0378	55111.4636	JUP	45918.7958	0.15238819	0.02108649	7.22681721
19336.7887	18133.5298	ANAPC7__AF	16802.5358	0.26863453	0.02633749	10.1997021
12357.8872	12858.0944	YAP1	10977.2686	0.21138622	0.03084559	6.85304598
5680.22173	5855.35917	NACC1	4993.06063	0.17278693	0.04205027	4.10905644
3926.48331	4121.82366	BSDC1	3437.90034	0.21220083	0.05133631	4.1335428
5955.01948	6223.26779	LSS	4964.43556	0.17584472	0.04509226	3.89966484
33789.7083	32419.6084	PSAP	25977.485	0.15790415	0.02176866	7.25373901
3029.18453	3215.56288	SRPK2	2708.3975	0.2104462	0.0559329	3.76247653
4577.82609	5048.87023	MLXIP	4180.55868	0.22066851	0.04628114	4.76800057
5660.9939	5727.52652	IRAK1	4793.50219	0.19281409	0.0467082	4.12805656
9469.70674	9899.23608	PHF20	7295.58941	0.14291106	0.0363982	3.9263227
8320.84384	8445.26955	SEZ6L2	6371.36341	0.1395784	0.03801096	3.67205683
6782.61737	7881.97443	PCNT	5937.67216	0.17706827	0.03928968	4.50673751
7647.86976	7629.42699	MARK2	6426.44044	0.1621204	0.04353578	3.72384256
7898.63272	8736.27071	SETD7	6816.06367	0.09792555	0.03779012	2.59130065
5298.86975	4745.39758	MAF1	4167.62696	0.11579458	0.04670459	2.4792976
4337.4782	4297.46364	MCFD2	3681.82892	0.1462367	0.05157109	2.83563322

5245.99321	5277.514	RMND5A	4196.48118	0.14479914	0.04608877	3.14174457
11966.9213	11703.4433	PPP2R1A	9898.2001	0.0835351	0.03330688	2.50804312
4431.21387	4732.9261	CNNM4	4004.71429	0.20601635	0.04786757	4.30388168
6781.81621	6273.1537	C7orf50	5460.29458	0.17212793	0.04214236	4.08443961
20073.0544	20375.2781	NFE2L1	17225.831	0.08240982	0.02545293	3.23773407
3283.15214	3137.61614	PRMT2	2744.44183	0.16711929	0.05707654	2.92798588
5288.45467	5728.56581	UBE3C	4720.77596	0.13293682	0.04320077	3.07718636
6781.81621	6526.74043	AC002310.11	5403.53246	0.10965238	0.04050894	2.70686871
8773.49903	8907.75354	PTP4A1	7667.34764	0.11888956	0.03560735	3.33890485
8025.21594	8206.23287	CCDC85C	6962.04704	0.09917125	0.03644527	2.72110068
15604.186	14452.365	KIAA0391__F	15099.5673	-0.0839343	0.02856735	-2.9381198
14115.6314	14429.5007	AZIN1	14768.4481	-0.0854951	0.02701765	-3.1644176
6246.64158	5757.66592	TPBG	5934.84619	-0.1578479	0.04086946	-3.8622454
10959.0625	10921.8973	MORF4L2	10415.249	-0.1682825	0.03150814	-5.3409219
6809.05564	6456.06872	BUB3	6512.47826	-0.1129235	0.03796179	-2.9746634
3443.38406	3272.72383	SSBP1	3421.99693	-0.1578865	0.04990641	-3.1636509
2715.93112	2601.34256	ABCD3	2701.44958	-0.1565184	0.05634137	-2.7780363
4789.33223	5286.86761	CTC-432M15	4878.46183	-0.1602982	0.04269019	-3.7549193
8262.35919	7880.93514	PLP2	7618.00345	-0.2420321	0.03527528	-6.8612401
3655.69136	3600.10014	DDAH1	3710.84061	-0.163594	0.04808687	-3.4020521
3895.23809	3743.52214	DARS	3882.07516	-0.2027873	0.04705757	-4.3093448
1761.75001	1846.81811	WDR76	1956.89082	-0.1968672	0.06599158	-2.9832163
2202.3878	2093.12981	NARF	2217.33033	-0.2471304	0.0629432	-3.9262443
3068.44136	3620.88593	XRN1	3694.60843	-0.2862309	0.04987425	-5.7390523
3307.18692	3684.28262	NR2C2	3726.10938	-0.1860568	0.04914999	-3.7854895
7948.30462	7930.82106	SRSF11	7786.02987	-0.1817278	0.03452891	-5.2630635
1040.70635	1044.48633	E2F2	1077.22289	-0.2492381	0.08746147	-2.8496899
9892.71902	9943.92555	PFKP	8298.04284	-0.5611965	0.03401845	-16.496829
137344.397	133731.664	MT-ND6	144416.098	-0.2617364	0.01930897	-13.555177
4099.53379	4423.21772	GK5	4021.93668	-0.2555634	0.04862469	-5.2558369
1473.33255	1570.36701	RBL1	1544.22171	-0.2538222	0.0750818	-3.3806096
1272.24148	1288.71945	PLEKHA1	1170.28165	-0.5081371	0.08642003	-5.8798528
8134.17365	7959.92117	SEMA3F	7764.0672	-0.2720173	0.03527419	-7.7115107
10210.7794	9877.411	GPI__PDCD2L	10298.0354	-0.3042055	0.03172179	-9.5897951
2075.00342	1998.55444	CFL2	2215.98557	-0.307003	0.06134803	-5.0042842
49157.5521	46902.1129	GAPDH	43681.6223	-0.3972496	0.02080644	-19.092627
4982.4117	5651.65836	KNTC1	6324.13707	-0.2635392	0.03780115	-6.9717229
2530.06209	2740.60741	PLEKHH1	2996.79759	-0.143378	0.05425304	-2.6427644
2778.42157	2597.1854	PRSS53__RP	2730.50957	-0.2262465	0.05653129	-4.0021469
5217.95262	5342.98926	SLC25A36	5288.82733	-0.2920294	0.04350729	-6.7121954
17005.4142	16852.0854	HNRNPH1	18619.1135	-0.1327439	0.0277457	-4.784305
29268.7645	30584.2225	PREX1	27945.2059	-0.3951473	0.02186929	-18.068592
62554.5434	59591.8423	ENO1	68242.3505	-0.2739675	0.0176909	-15.486345
5237.98161	5922.91301	BBX	5965.22278	-0.2929929	0.03919084	-7.4760573
4284.60166	4324.48518	SOD2	4716.0019	-0.1327546	0.04500574	-2.9497255

1485.34994	1595.30996	ZBTB25	1600.21729	-0.2614694	0.07302617	-3.5804893
5018.46388	5065.49886	BNIP3L	5036.86473	-0.1489069	0.04297191	-3.465214
4021.02015	3996.06958	DCUN1D3__I	3841.54839	-0.4272258	0.04763954	-8.9678826
25975.1973	28619.9647	IL6ST	25847.9842	-0.3442859	0.0224566	-15.331172
15778.8388	15864.76	IFFO1	14171.3725	-0.4128932	0.03364229	-12.273041
3103.69238	3018.09781	HELLS	3833.73997	-0.2632513	0.04826902	-5.4538356
4951.96763	4853.48373	SNHG1__SN	5821.91382	-0.2029603	0.03939263	-5.1522419
1701.66304	1923.72556	ZNF292	1849.97037	-0.2795894	0.06732174	-4.1530331
9059.51302	8589.73084	MAL2	10989.4371	-0.143771	0.03104175	-4.6315372
1075.95737	1050.72207	CP	980.289766	-0.4729766	0.09158976	-5.1640775
1408.43862	1471.63447	FBXL20	1734.53931	-0.2562058	0.06903813	-3.7110762
2323.36291	2422.58471	AF011889.5_	3036.07919	-0.145056	0.05326567	-2.7232549
895.696458	937.43947	PIGA	872.437041	-0.4612542	0.09779619	-4.7164841
8463.45026	8595.96658	QSOX1	7716.69568	-0.3464863	0.03542619	-9.7805119
1818.63234	1781.34285	SLC9A8	1787.66727	-0.2534757	0.06835888	-3.7080144
6333.96798	7078.60336	NKTR	8009.76656	-0.216449	0.0343801	-6.295764
6365.2132	6215.99276	HSD17B12	5421.6851	-0.5224014	0.04087739	-12.779715
5518.38748	5884.45929	CCNL1	6430.5176	-0.0972554	0.03782077	-2.5714812
3831.94648	3428.61731	AGR3	3602.71554	-0.4231167	0.05422341	-7.8032108
9395.1989	10045.776	CRIM1	11216.2148	-0.353223	0.03182117	-11.100253
1211.35335	1251.30501	NPIPB5	1246.07851	-0.239426	0.08392666	-2.8528002
703.418149	735.817233	FUT11	801.280286	-0.4001095	0.10174338	-3.9325364
586.448844	623.573927	ZGRF1	734.533837	-0.3101765	0.10555877	-2.9384252
1590.30185	1647.27446	ZNF37BP	1650.25801	-0.2469592	0.07148046	-3.4549191
2205.59244	2263.57335	FAM217B	1852.34238	-0.8879549	0.06773382	-13.109476
2432.32062	2451.68482	CCDC125	3193.98036	-0.1361869	0.05224359	-2.6067675
4225.31585	4441.92494	AK4	3792.3258	-0.7595504	0.04817527	-15.766395
7059.8186	7708.41302	TACC1	7558.88268	-0.4837521	0.03542963	-13.653885
7175.18558	7424.68689	RDX	8803.08975	-0.1254715	0.03587936	-3.4970394
7101.4789	7171.10016	TMEM167A	7325.14018	-0.8283295	0.03618998	-22.888366
1661.60506	1552.69908	FAM162A	1675.02435	-0.2982792	0.07014253	-4.2524724
480.695774	438.580328	HLA-F	442.869334	-0.6447817	0.13757593	-4.6867338
6183.34997	6198.32483	STAT1	6288.87953	-0.6407411	0.03808277	-16.824959
46262.9624	47553.7476	CA12	52893.4473	-0.4770858	0.01862077	-25.621169
870.86051	934.3216	ATAD5	1281.53853	-0.1988781	0.08007476	-2.4836558
4385.54778	4873.23024	LRRC58	4794.5254	-0.8022111	0.0436436	-18.380954
52000.8676	50265.2549	STC2	71486.029	-0.3525262	0.03774557	-9.339539
1071.15042	1135.94384	KCNK5	1047.27531	-0.5052467	0.09034646	-5.592324
33236.107	37632.6865	PGR	49368.3657	-0.4925123	0.0208875	-23.579286
20473.6342	19350.5382	PGK1	22443.771	-0.5131636	0.02324374	-22.077499
2982.71728	2868.44006	P4HA1	3082.53246	-0.5689177	0.0534782	-10.63831
55905.7197	56540.4872	ALDOA	56139.3649	-0.3880397	0.01821783	-21.299989
273.996591	261.901049	CCBL1	317.565786	-0.4759897	0.16631975	-2.8618955
4327.86428	4176.90602	PBX2	4201.56471	-0.7490286	0.04839588	-15.477115
8940.14023	8907.75354	BHLHE40	9171.25754	-0.5919435	0.03276644	-18.065545

3070.04368	3541.8999	CLSTN2	3073.77261	-0.5076963	0.05273126	-9.6279933
5060.92534	5169.42785	BCL6	6830.36153	-0.178891	0.03654779	-4.894716
1869.1054	1861.36817	SFXN3	2105.90255	-0.4230713	0.06339069	-6.6740294
900.503416	1004.99331	ZBTB37	1107.08318	-0.7806346	0.08739048	-8.9327198
216.313098	210.975845	CYP4V2__KLI	313.043009	-0.6425902	0.17113979	-3.7547682
1426.06413	1388.49128	DUSP4	3342.42842	-0.3167885	0.05093019	-6.2200541
1572.67634	1639.99943	SCARA3	2259.25722	-0.7323412	0.06192347	-11.826553
342.095159	319.061992	LDHAP4	342.099675	-0.7620477	0.16250258	-4.6894496
631.313783	632.927536	C1orf21	823.291894	-0.6444601	0.10315067	-6.2477547
253.166441	278.529687	PRSS23	321.989305	-0.4335615	0.16566576	-2.6170857
30056.3044	28039.0016	LDHA	28361.841	-0.7265569	0.02213928	-32.817547
291.622103	289.961876	GPR37L1	481.473695	-0.5985693	0.13104405	-4.5676951
2643.0256	2631.48197	PHLDA1	2536.69186	-0.942833	0.05836353	-16.154487
2476.38439	2455.84198	ALDOC	2718.17231	-0.5594398	0.05904473	-9.4748475
2562.10847	2656.42493	PAPSS2	2257.00447	-0.7653504	0.06245613	-12.254208
1173.69885	1147.37603	FOS	1680.41815	-0.4182843	0.07035894	-5.9450057
483.099253	473.916184	LAMB3	522.927121	-0.3443405	0.12866263	-2.6763056
2026.93385	1931.00059	KRT15	2179.95041	-0.5021324	0.06273577	-8.0039259
2236.03651	1995.43657	BNIP3	2474.53239	-0.5549081	0.05882313	-9.4335023
12824.9632	12541.111	EFEMP1	16849.2172	-0.0755946	0.02749023	-2.7498719
10150.6924	9601.99918	FHL2	7919.98671	-0.917516	0.03603953	-25.458597
479.093455	445.855358	WISP2	683.595768	-0.4071775	0.11096177	-3.6695293
1016.67156	1013.30763	PFKFB4__UC	1093.20889	-0.6625845	0.08738942	-7.5819757
71.3032064	80.0253206	MSS51	95.480794	-0.7858787	0.29478497	-2.6659389
119.372784	103.928988	EGR1	196.093508	-0.5978722	0.20649873	-2.8952827
657.75205	729.581494	PDK1	815.991518	-0.6274876	0.10328576	-6.0752575
681.786839	795.056756	PRKAA2	795.641548	-0.8421501	0.10256911	-8.2105631
164.237723	159.011351	CD109	190.216797	-1.3325493	0.2154859	-6.183928
359.720671	330.494181	SPAG4	530.122791	-0.3578653	0.12642356	-2.830685
307.645295	332.572761	DDX60	370.330123	-1.1173031	0.17216965	-6.4895473
1415.64905	1187.90833	IFI6	1370.75124	-1.9892452	0.08563757	-23.228649
271.593112	244.233121	IFI27	228.949809	-2.5986677	0.2203545	-11.793123
381.351981	391.812284	OAS1	298.308044	-3.1248232	0.19972902	-15.645314
128.18554	137.186264	SCNN1G	165.751136	-1.3329461	0.22920652	-5.8154808
220.318896	207.857976	FOSB	386.972715	-0.5297498	0.15057611	-3.5181531
1033.49591	1090.21508	OAS2	994.858288	-2.484158	0.10312244	-24.089402
165.038882	183.954308	PPFIA4	368.246097	-0.636478	0.14857818	-4.283792
30.4440657	33.2572761	IFI44	35.4894304	-2.5992151	0.5457183	-4.7629245
850.83152	746.210132	UBE2T	1758.27637	-2.4289886	0.07714838	-31.484634

pvalue	padj	Control.0	Control.1	shRNA2.0	shRNA2.1	
9.97E-22	3.72E-20	96.7565858	91.30427	353.575564	362.316394	
0.00204348	0.00954358	69.2473604	113.092789	161.101576	168.68501	
4.07E-32	2.48E-30	659.272815	672.331443	1507.57159	1362.54723	
1.72E-20	5.96E-19	129.008781	109.980144	391.7312	411.021221	
8.17E-11	1.31E-09	8512.68236	8388.57981	9911.13852	9693.44844	
7.45E-10	1.07E-08	342.442426	324.752688	576.574061	609.404296	
4.51E-32	2.72E-30	871.757866	833.151464	1643.23607	1728.42739	
2.39E-58	3.35E-56	1320.44282	1282.40998	2685.3089	2769.04759	
2.55E-67	4.46E-65	4567.48	4463.53375	7353.01508	7226.1332	
1.16E-06	1.08E-05	166.95254	154.59473	295.070255	314.79949	
9.87E-50	1.01E-47	654.529845	654.693118	1629.66963	1587.0646	
1.05E-06	9.82E-06	328.213516	311.264557	536.722618	495.363726	
1.13E-11	1.98E-10	370.900245	397.381084	730.892412	664.048735	
1.22E-13	2.56E-12	819.585197	877.766051	1291.35632	1511.03755	
6.99E-06	5.74E-05	1532.92787	1477.4691	1887.43215	1862.66264	
1.26E-08	1.54E-07	420.227132	405.681473	686.801455	656.9212	
0.0001659	0.00103602	136.597533	145.256793	225.542206	263.718818	
0.01218527	0.04405899	351.928366	373.517468	431.582643	506.055029	
7.97E-05	0.00053423	407.895411	475.197224	635.927273	618.907676	
6.65E-13	1.31E-11	1200.91998	1251.28352	1755.15927	1815.14574	
7.96E-07	7.56E-06	996.023677	1009.53471	1369.36339	1306.71486	
1.54E-32	9.52E-31	2792.66067	2775.4423	4151.33324	4283.64891	
1.60E-08	1.92E-07	537.852786	541.600329	808.051588	855.304274	
6.55E-05	0.00044903	265.606314	309.18946	434.126352	445.470976	
0.00013481	0.00086118	290.269757	312.302106	449.388606	441.907208	
7.59E-07	7.23E-06	1351.74642	1392.39012	1776.35685	1746.24623	
0.00039915	0.00227949	323.470547	358.991789	444.301188	551.196088	
2.66E-28	1.35E-26	1847.86107	1795.99649	2853.1937	2968.61859	
0.00501343	0.02054079	737.057521	700.345253	889.450279	862.43181	
1.20E-13	2.52E-12	1004.56102	1008.49717	1526.22546	1521.72886	
0.00083954	0.00438591	655.478439	660.918409	798.724655	895.693643	
2.14E-06	1.91E-05	306.395855	306.076814	454.476024	559.511546	
2.35E-11	4.01E-10	4496.33546	4688.68178	5616.50967	5589.17585	
1.11E-40	8.93E-39	2272.83117	2278.45656	3735.86075	3726.51321	
0.00017544	0.00108814	706.702514	803.062557	996.286061	970.532767	
0.00068494	0.00366807	349.082584	332.015527	462.955055	489.424113	
2.42E-37	1.75E-35	4586.45188	4545.50008	6467.80432	6658.30619	
0.00135413	0.0066528	740.851897	754.297776	934.38914	925.391708	
3.06E-34	2.03E-32	2102.08426	2158.10093	3392.46003	3447.3514	
1.10E-08	1.35E-07	572.950763	570.651688	854.686255	893.317798	
1.01E-23	4.16E-22	602.357176	564.426397	1137.88587	1208.11729	
1.38E-05	0.00010811	541.647161	561.313751	821.618037	734.136169	
6.27E-15	1.46E-13	8266.04792	8308.68857	9983.21028	9792.04602	
0.00381324	0.01626173	1759.64183	1738.93133	1985.7889	2013.52881	

3.45E-10	5.20E-09	1933.23453	1958.89161	2536.07796	2540.96645
0.00023382	0.00140891	1172.46216	1147.52867	1463.48063	1386.30568
4.10E-13	8.25E-12	1581.30616	1634.13892	2275.77173	2215.47566
6.73E-05	0.00046012	1698.93182	1674.60332	1973.07035	2118.066
3.00E-12	5.54E-11	2189.3549	2152.91319	2904.91578	2845.07464
3.03E-58	4.15E-56	3337.15362	3322.23037	5446.08117	5633.12899
3.58E-19	1.14E-17	2086.90675	2109.33615	2980.37915	3022.0751
3.37E-21	1.21E-19	5087.3095	5279.04689	6743.3728	6722.45401
0.00143673	0.00701291	2701.59565	2793.08062	3025.31801	3205.01518
4.70E-19	1.48E-17	2347.7701	2371.83592	3264.42667	3404.58618
1.34E-14	3.05E-13	2900.80039	2931.07458	3774.01639	4287.21268
1.86E-13	3.85E-12	3081.98184	3097.08234	3968.18618	3974.78903
0.00162955	0.00782394	775.00128	802.025008	948.803491	1003.7946
1.17E-07	1.25E-06	2184.61193	2032.55756	2604.75811	2717.96692
5.17E-17	1.45E-15	4949.76338	5399.40252	6634.84121	6884.01149
3.66E-12	6.66E-11	1439.01707	1352.96327	2006.13857	1947.00515
3.41E-15	8.11E-14	4097.92599	4268.47462	5247.67186	5591.55169
4.35E-44	3.80E-42	14607.3987	14861.8451	18839.5574	19370.266
0.003086	0.01361152	1623.0443	1645.55196	1861.14715	1904.23993
2.28E-08	2.68E-07	2068.88347	2229.69178	2742.1184	2714.40315
0.00397334	0.01686438	605.202958	542.637878	696.128388	756.706698
1.18E-25	5.26E-24	3139.84607	3320.15527	4607.50507	4615.07931
3.55E-15	8.44E-14	4511.51296	4375.34212	5530.02357	5882.59273
0.00033994	0.00197148	745.594867	721.096224	959.826231	917.07625
1.64E-07	1.72E-06	6783.39554	6611.25919	7850.73416	7523.11385
2.25E-10	3.45E-09	2085.00956	2009.73149	2677.67777	2660.94663
0.00232596	0.01068069	1002.66384	1002.27187	1237.09052	1170.10376
0.00475849	0.01960924	862.271926	830.038819	950.499297	1129.7144
1.21E-68	2.21E-66	5903.10033	5950.34078	9766.99501	9192.14511
1.49E-06	1.36E-05	1992.04735	1876.92528	2330.88543	2510.08046
4.02E-10	6.03E-09	4497.28405	4589.07712	5491.86793	5422.86668
1.58E-19	5.10E-18	1716.9551	1762.79494	2551.34022	2612.24181
8.61E-08	9.42E-07	1348.90064	1363.33876	1772.11733	1778.32014
0.00283294	0.01268261	4758.14739	4691.79442	5146.7714	5168.65125
0.00030365	0.00177668	2011.01923	2115.56144	2369.04107	2468.50317
3.75E-20	1.28E-18	3873.10921	3978.99859	5228.17009	5185.28216
0.01004124	0.03739293	1734.02979	1833.34824	2012.92179	2009.96505
0.00229449	0.0105539	522.675282	520.849359	703.759515	636.726515
5.72E-13	1.13E-11	33638.0911	33546.0189	40612.8594	43220.1881
0.00067811	0.0036374	1787.15106	1656.96499	2010.37809	2047.97857
3.90E-31	2.24E-29	16663.0018	16211.6957	20108.8683	20465.5306
2.31E-05	0.00017423	1486.44676	1484.73194	1865.38667	1793.76313
4.01E-29	2.15E-27	3906.31	3998.71201	5562.24388	5515.52465
9.37E-24	3.87E-22	5953.37581	6007.40595	7673.52243	7773.76552
4.24E-10	6.33E-09	1346.05486	1424.55412	1927.28359	1867.41433

5.60E-53	6.34E-51	8465.25266	8547.32473	11770.5899	12096.6159
0.00147505	0.00717863	2529.90014	2456.9149	2812.49435	2840.32295
1.68E-07	1.76E-06	2774.63739	2878.1596	3472.16291	3383.20357
0.00994527	0.03711247	848.99161	915.117798	1042.07282	1042.99605
5.50E-08	6.17E-07	9117.88532	9001.77099	10161.2699	10267.2151
0.00026241	0.00155646	2574.48406	2711.11429	3032.94914	3057.71278
0.01265216	0.04547691	4133.02396	3937.49665	4395.52931	4360.86388
1.64E-10	2.55E-09	7584.00885	7551.27815	8762.22992	8819.13741
0.00725534	0.0282678	1035.86462	1096.68879	1238.78633	1260.38588
3.78E-05	0.00027246	3187.27577	3151.03487	3623.93755	3696.81514
2.71E-08	3.16E-07	2860.01084	2827.31973	3478.94613	3467.54608
0.00082508	0.00432272	2097.34129	2069.9093	2408.89251	2391.2882
2.83E-58	3.94E-56	29259.3813	28598.9875	36587.8637	36811.3456
2.86E-11	4.82E-10	3776.35263	3589.91789	4526.95428	4697.04597
0.0002385	0.0014343	4506.76999	4535.1246	5013.65062	5073.61744
1.73E-07	1.80E-06	7759.49874	7785.76412	8709.65993	8874.96977
0.00028547	0.00168111	3725.12855	3686.4099	4155.57275	4183.86341
5.84E-06	4.88E-05	7833.48907	7895.74426	8770.70895	8707.47268
1.56E-14	3.51E-13	4295.23353	4305.82637	5320.59152	5591.55169
3.57E-12	6.51E-11	5758.91404	5835.17289	6957.89227	6948.15931
1.11E-06	1.04E-05	1679.95994	1774.20798	2156.21741	2170.3346
6.85E-12	1.22E-10	11084.3206	11450.3855	12854.2099	13479.3578
0.00156779	0.00756945	1733.0812	1815.70992	2031.57566	2080.05248
0.00060676	0.0032847	2070.78065	2022.18207	2342.75607	2403.16743
7.66E-14	1.64E-12	6298.66401	6249.15476	7498.8544	7722.68484
0.0005948	0.00322526	2712.03018	2698.66371	2982.07496	3244.21663
1.00E-05	8.08E-05	4709.7691	4930.43058	5486.78051	5509.58503
1.33E-15	3.31E-14	9466.01931	9785.12012	11447.5388	11488.3995
4.94E-13	9.87E-12	42976.9988	44007.6206	47720.8305	48969.7335
1.99E-24	8.49E-23	15356.7879	15132.6452	18559.7494	18160.9608
7.23E-12	1.29E-10	10314.0623	10032.0567	11732.4342	11830.5212
3.97E-05	0.00028441	4668.97956	4720.84578	5310.41668	5272.00051
3.57E-05	0.00025821	3249.88297	3118.87086	3646.83093	3736.01659
9.63E-05	0.0006323	4628.19002	4691.79442	5113.70318	5424.05461
4.05E-13	8.17E-12	24564.7897	24547.3605	27309.2608	27488.529
0.00016824	0.00104922	2503.33951	2520.20536	2932.89658	2877.14855
1.86E-06	1.67E-05	3851.29155	3874.20619	4569.34943	4427.38754
3.66E-05	0.00026409	4318.94838	4628.50396	5066.22061	5160.33579
8.63E-05	0.00057231	6925.68463	6947.42491	7771.87918	7537.36892
0.00024061	0.00144593	6069.10427	6055.13318	6610.25203	6750.96416
6.58E-06	5.43E-05	5511.33101	5635.96358	6275.33033	6328.06371
0.00019621	0.00120425	5814.88109	6317.63296	6745.06861	6828.17912
0.00956139	0.03585795	6692.33052	6474.30279	6992.65629	7104.96509
0.01316414	0.04692707	3987.88908	4010.12504	4252.2337	4420.26001
0.00457349	0.01898497	3376.99456	3615.8566	3892.72281	3841.7417

0.00167945	0.00803298	4010.65534	3963.43536	4467.60107	4344.23296
0.01214019	0.04393464	9669.96701	9546.48396	9977.27496	10399.0745
1.68E-05	0.00012982	3778.24981	3657.35854	4225.9487	4357.30011
4.42E-05	0.00031317	5015.21636	5256.22082	5792.8735	5776.86762
0.00120483	0.00601135	16598.4974	16871.5765	17747.4583	17685.7917
0.00341166	0.01483682	2646.5772	2525.39311	2940.52771	2865.26932
0.00208965	0.00972328	4470.72342	4537.19969	4955.99321	4919.1875
0.00679211	0.02667808	5207.78094	5187.74262	5584.28936	5634.31691
0.00084109	0.00439262	7242.51502	7461.01143	7959.26575	8006.59835
0.00650649	0.02568518	6716.04536	6727.46462	7139.34352	7265.33464
0.00330209	0.01441376	15388.0915	15698.1092	14912.0706	14399.9978
0.00155394	0.00750917	15073.1583	15341.1925	14371.1085	14288.3331
0.00011235	0.00072759	6294.86964	6218.0283	5481.69309	5744.79371
9.25E-08	1.00E-06	10894.6018	11153.6466	9890.78885	9721.95859
0.0029331	0.01303513	6830.82524	6700.48836	6196.47535	6322.1241
0.00155804	0.00752677	3601.81133	3615.8566	3223.72732	3246.59248
0.00546885	0.02215316	2811.63255	2883.34735	2531.83845	2578.97997
0.0001734	0.00107791	5126.20186	5174.25449	4648.20441	4565.18657
6.83E-12	1.22E-10	8208.18369	8300.38819	6915.49712	7047.94481
0.00066882	0.0035946	3939.51079	3902.22	3498.4479	3503.18376
1.64E-05	0.00012678	4151.04725	4157.45693	3600.19627	3619.60017
0.00285236	0.01273511	2050.86018	2129.04957	1805.18555	1842.46796
8.63E-05	0.00057231	2467.29294	2344.85966	2010.37809	2046.79065
9.52E-09	1.18E-07	4119.74365	3996.63691	3266.97038	3395.0828
0.00015341	0.00096607	4037.21597	3892.88206	3456.90066	3517.43883
1.42E-07	1.50E-06	8312.52903	8239.17282	7308.07622	7284.3414
0.00437619	0.01827676	1150.6445	1190.06816	992.894449	975.284457
3.87E-61	5.94E-59	9883.40066	9897.17536	6641.62444	6769.97092
7.38E-42	6.13E-40	159580.068	155361.478	129294.189	133428.655
1.47E-07	1.55E-06	4357.84074	4401.28084	3767.23316	3561.39197
0.00072325	0.00385063	1623.99289	1735.81868	1405.82323	1411.25205
4.11E-09	5.33E-08	1411.50784	1338.4376	996.286061	934.895089
1.24E-14	2.84E-13	8487.07032	8496.48486	6939.2384	7133.47523
8.83E-22	3.33E-20	11526.3654	11229.3877	9153.96112	9282.42722
5.61E-07	5.48E-06	2444.52668	2456.9149	1973.91826	1988.58244
2.91E-81	6.46E-79	50395.0037	48914.1876	37557.0168	37860.2813
3.13E-12	5.77E-11	6880.15212	6919.4111	5728.43287	5768.55216
0.00822322	0.03149361	3085.77621	3207.06249	2871.84756	2822.50411
6.28E-05	0.00043151	2992.814	2893.72283	2488.59539	2546.90606
1.92E-11	3.29E-10	5879.38548	5759.43185	4632.94216	4883.54982
1.72E-06	1.55E-05	19465.1484	19496.5743	18184.1284	17330.6029
5.63E-73	1.12E-70	31576.7963	31914.9926	23965.1313	24323.9032
4.29E-54	5.00E-52	75042.321	74351.7647	61294.9101	62280.4063
7.66E-14	1.64E-12	6505.4575	6634.08526	5387.57586	5333.77249
0.00318056	0.0139649	4879.56742	4990.6084	4597.33023	4396.50156

0.00034295	0.00198825	1764.3848	1723.3681	1421.08548	1492.03079
0.00052981	0.00292696	5223.90704	5367.23851	4714.34085	4841.97253
3.02E-19	9.68E-18	4381.55558	4432.40729	3300.8865	3251.34417
4.73E-53	5.40E-51	28679.7903	29153.0384	22667.8396	22891.2686
1.26E-34	8.48E-33	16634.544	15726.123	11870.6424	12454.1806
4.93E-08	5.55E-07	4246.85524	4119.06764	3514.55806	3454.47893
2.57E-07	2.62E-06	6201.90743	6261.60534	5451.16858	5372.97394
3.28E-05	0.00023927	2007.22486	2051.23343	1689.02284	1652.40034
3.63E-06	3.12E-05	11442.8892	11625.7312	10301.1739	10587.9542
2.42E-07	2.47E-06	1135.46699	1144.41602	833.488679	807.78737
0.00020638	0.00126288	1891.49639	1884.18812	1569.46851	1593.00421
0.00646422	0.02554283	3202.45327	3172.82338	2845.56257	2923.47753
2.40E-06	2.13E-05	984.640549	1036.51098	724.957091	743.63955
1.37E-22	5.35E-21	8525.01408	8752.75934	6773.89731	6815.11198
0.00020889	0.00127729	1919.00562	1970.30465	1639.84446	1621.51435
3.06E-10	4.64E-09	8624.61645	8597.12706	7475.96102	7341.36169
2.13E-37	1.55E-35	6353.68247	6433.83839	4483.71123	4415.50832
0.01012645	0.03764199	6588.93377	6704.63856	6181.21309	6247.28497
6.04E-15	1.41E-13	3961.32845	4296.48844	3126.21847	3026.82679
1.25E-28	6.55E-27	12830.6822	12330.2267	9790.73629	9913.21413
0.00433359	0.01812648	1327.08298	1373.71425	1187.06424	1096.45256
8.41E-05	0.00055957	928.673505	895.404376	706.303224	674.740039
0.00329884	0.01440387	815.790821	811.362945	670.691297	640.290283
0.00055046	0.00302476	1769.12777	1814.67237	1534.70449	1482.52741
2.91E-39	2.25E-37	2387.61104	2422.6758	1300.68325	1298.39941
0.00914014	0.03452241	3382.68613	3304.59205	3008.35995	3080.28331
5.30E-56	6.70E-54	4784.70803	4753.00979	2838.77935	2792.80604
1.91E-42	1.62E-40	8901.60589	8726.82063	6307.55064	6299.55357
0.00047045	0.00263357	8942.39543	9429.24098	8375.58614	8465.13647
6.07E-116	2.43E-113	9283.88926	9459.32989	5254.45508	5302.8865
2.11E-05	0.00016052	1842.16951	1853.06166	1503.33207	1501.53417
2.78E-06	2.43E-05	522.675282	557.163557	334.073794	357.564704
1.60E-63	2.69E-61	7643.77028	7683.04681	4929.70822	4898.99282
8.86E-145	6.07E-142	61879.631	61230.9261	43895.9399	44567.2923
0.01300415	0.04652833	1384.94721	1354.00082	1186.21634	1200.98975
1.87E-75	3.80E-73	6049.1838	6137.09952	3457.74856	3534.06975
9.68E-21	3.41E-19	79009.341	81351.0671	64175.2367	61408.4711
2.24E-08	2.64E-07	1199.02279	1257.50881	842.815612	889.75403
6.29E-123	2.79E-120	57128.1237	58288.4385	40423.777	41633.1235
5.20E-108	1.90E-105	26542.6081	26240.6397	18407.9748	18583.8612
1.98E-26	9.28E-25	3745.04903	3620.0068	2489.4433	2475.63071
1.14E-100	3.45E-98	63142.2096	64145.3999	48536.5132	48733.3369
0.00421116	0.01768643	377.540403	360.029338	241.652364	291.041038
4.95E-54	5.73E-52	5222.95844	5319.51128	3226.27103	3037.5181
5.95E-73	1.16E-70	11058.7086	10990.7515	7241.93978	7393.63028

6.09E-22	2.31E-20	3623.629	3595.10563	2549.64441	2526.71138
9.84E-07	9.25E-06	7255.79534	7249.35153	6375.38289	6440.91636
2.49E-11	4.23E-10	2446.42387	2378.06122	1795.85862	1803.26651
4.16E-19	1.31E-17	1429.53113	1369.56405	819.074328	810.163216
0.0001735	0.00107815	341.493832	422.282249	248.435588	239.960366
4.97E-10	7.35E-09	3747.89481	3666.69648	2959.18158	2995.94081
2.85E-32	1.74E-30	2830.60443	2808.64385	1666.97736	1730.80323
2.74E-06	2.41E-05	451.530734	407.75657	232.32543	276.785967
4.16E-10	6.23E-09	1011.20118	999.159228	675.778715	607.02845
0.00886841	0.03364303	366.157276	372.47992	247.587685	301.732341
3.31E-236	9.06E-233	35512.5128	35190.5333	21152.6369	21591.6812
4.93E-06	4.16E-05	568.207793	592.440207	393.427006	371.819775
1.06E-58	1.50E-56	3326.71908	3347.13154	1725.48267	1747.43415
2.67E-21	9.67E-20	3178.73842	3295.25411	2127.3887	2271.30802
1.60E-34	1.07E-32	2897.00601	2787.89288	1690.71864	1652.40034
2.76E-09	3.69E-08	1942.72047	1901.82644	1439.73935	1437.38635
0.00744387	0.02892004	549.235913	621.491565	467.19457	453.786435
1.21E-15	3.01E-14	2584.91859	2527.4682	1831.47055	1775.94429
3.97E-21	1.41E-19	2924.51523	2966.35123	2028.18405	1979.07906
0.00596186	0.02384532	17617.2874	16959.7682	16349.2662	16470.5469
5.67E-143	3.73E-140	10414.6133	10292.4814	5382.48844	5590.36377
0.000243	0.0014571	750.337837	808.2503	578.269867	597.52507
3.40E-14	7.50E-13	1353.64361	1327.02456	860.621576	831.545822
0.00767736	0.02970775	111.934089	129.693565	67.8322424	72.4632788
0.00378817	0.01619138	238.097089	233.448418	144.143515	168.68501
1.24E-09	1.73E-08	1016.89274	965.957675	668.147588	612.968063
2.20E-16	5.79E-15	1031.12165	1012.64736	581.661479	557.135701
6.25E-10	9.07E-09	252.325998	292.588684	110.227394	105.725112
0.00464484	0.01922287	589.07686	600.740595	440.061673	490.612035
8.61E-11	1.38E-09	513.189342	498.023291	193.321891	276.785967
2.34E-119	9.61E-117	2161.84568	2221.39139	578.269867	521.498023
4.24E-32	2.57E-30	387.974937	396.343536	54.265794	77.2149692
3.58E-55	4.35E-53	556.824665	513.586519	61.0490182	61.7719754
6.05E-09	7.71E-08	252.325998	222.035384	92.4214303	96.2217309
0.00043456	0.00245271	422.12432	492.835549	320.507346	312.423645
3.23E-128	1.52E-125	1733.0812	1641.40176	292.526546	312.423645
1.84E-05	0.00014126	454.376516	441.995671	289.134933	287.47727
1.91E-06	1.71E-05	53.1212628	68.4782025	8.47903031	11.879226
1.41E-217	2.11E-214	2934.00117	2995.40259	535.874715	567.827004

av log2 FC
1.15515941
1.05154293
0.97265134
0.89582486
0.85736232
0.81860837
0.8091885
0.79891871
0.79690073
0.77786842
0.7433105
0.67257651
0.67083065
0.64937967
0.63670714
0.59608807
0.595419
0.5733539
0.55345278
0.55102349
0.54465217
0.52540813
0.52239864
0.51854089
0.51267439
0.50054519
0.4999984
0.49329673
0.48457579
0.48165538
0.47594572
0.47397856
0.47125558
0.46887401
0.4617209
0.45705401
0.45312145
0.44922132
0.43292684
0.4320902
0.42596388
0.42154754
0.41888742
0.41111761

0.40852432
0.40850914
0.40656576
0.40539689
0.40493861
0.40075395
0.39707042
0.38764551
0.37414927
0.36702917
0.36255796
0.36184698
0.35653403
0.35419469
0.35116315
0.34817156
0.33944897
0.33774607
0.3352759
0.33494393
0.33253198
0.32919848
0.3257572
0.32569758
0.32261035
0.3211155
0.31998601
0.31874816
0.31690963
0.31549086
0.30178971
0.30161674
0.29671264
0.28883458
0.28752152
0.28743015
0.28713675
0.28423661
0.28293966
0.27944085
0.27430251
0.27368692
0.27116875
0.27104376
0.27085246

0.27080598
0.26821358
0.26624628
0.26544889
0.26426848
0.26047363
0.26003741
0.25836429
0.25698966
0.25639462
0.25435508
0.25390341
0.25064043
0.2486775
0.24565107
0.24488355
0.24419611
0.24174441
0.24061294
0.23696737
0.23624147
0.2356692
0.2349857
0.2322053
0.23013322
0.22977546
0.22935668
0.22694864
0.22578116
0.22439463
0.22191741
0.21761358
0.2094611
0.20516671
0.20178763
0.20068417
0.20054797
0.20023094
0.19896936
0.19703136
0.19577644
0.18913938
0.18451564
0.18208899
0.18053771

0.18044268
0.17800985
0.17779612
0.16083693
0.15689115
0.15005907
0.14695327
0.1403446
0.13175089
0.11884345
-0.131832
-0.140258
-0.1544887
-0.1587504
-0.1692497
-0.1698588
-0.1913744
-0.1959357
-0.2027908
-0.2160122
-0.2206123
-0.2264887
-0.2294176
-0.2465674
-0.2474983
-0.2481293
-0.2492953
-0.2504898
-0.2509715
-0.2548204
-0.2564838
-0.2690681
-0.2691408
-0.2716785
-0.2721104
-0.280228
-0.2805776
-0.2811728
-0.2823808
-0.2824278
-0.2829406
-0.2868924
-0.2883832
-0.2906335
-0.2949174

-0.2950336
-0.297393
-0.2983964
-0.2991023
-0.3058022
-0.3059825
-0.3110493
-0.3171647
-0.3184118
-0.3252075
-0.3272498
-0.3392997
-0.3395346
-0.3414806
-0.356364
-0.3597874
-0.3597889
-0.3684694
-0.3715337
-0.372237
-0.3734565
-0.3764127
-0.3792762
-0.38388
-0.388655
-0.3938285
-0.4014068
-0.4085736
-0.4156976
-0.4314527
-0.4324872
-0.4332719
-0.4448347
-0.4476436
-0.4497203
-0.4508127
-0.4548711
-0.4556027
-0.4590317
-0.4621122
-0.4646487
-0.4657059
-0.4783319
-0.4831845
-0.4951442

-0.4977525
-0.5013568
-0.5077496
-0.5233258
-0.5593642
-0.5725652
-0.5752376
-0.5786286
-0.6005107
-0.6150731
-0.615545
-0.6312789
-0.6382816
-0.6514801
-0.6749597
-0.6795101
-0.6938296
-0.6943242
-0.7355888
-0.7371695
-0.7516335
-0.7647167
-0.7759721
-0.7763084
-0.8307589
-0.8401456
-0.898363
-0.9204839
-0.927216
-0.9521242
-1.2133825
-1.2212944
-1.2315235
-1.2464162
-1.3000507
-1.3039105
-1.6923921
-2.2236471
-2.3250484

Supplementary Table S5: List of reagents, chemical primers, and antibodies used for the study.

REAGENT or RESOURCE	SOURCE	IDENTIFIER
Antibodies		
UBE2T	Cell Signaling	Cat# 12992
TFAP2A	Abcam	Cat# ab108311
IF16	Cell Signaling	Cat# 54355
ELK1	Cell Signaling	Cat# 9182
GTF2I	ThermoFischer	Cat# PA5-17642
FOXP3	Cell Signaling	Cat# 12632
ACTINB	Cell Signaling	Cat#4970
BrdU	BD Biosciences	Cat# 347580
BrdU	Abcam	Cat# ab6326
sheep anti-mouse Cy3	Sigma-Aldrich	Cat# C218-M
anti-rat Alexa Fluor 488	Invitrogen	Cat# A11006
Chemicals		
DMEM	GIBCO	Cat# 11965-092
RPMI	GIBCO	Cat# 11875-093
Trypsin-EDTA	GIBCO	Cat# 25200-056
Fetal Bovine Serum	GIBCO	Cat# 10438-026
Penicillin-Streptomycin	GIBCO	Cat# 15140-122
Effectene Transfection Reagent	QIAGEN	Cat# 301427
Agarose, Low gelling	Sigma-Aldrich	Cat# A9045
IdU	Sigma-Aldrich	Cat# I7125
CldU	Sigma-Aldrich	Cat# C6891
Hydroxyurea	Sigma-Aldrich	Cat# H8627
Aphidicolin	MedChemExpress	Cat# HY-N6733
Deposited Data		
RNA-Seq performed with MCF7 cells expressing either NS shRNA or UBE2T shRNAs	This paper	GEO: GSE156603
RNA-Seq performed with T47D cells expressing either NS shRNA or UBE2T shRNAs	This paper	GEO: GSE156603
Experimental Models: Cell Lines		
HEK293T	ATCC	ATCC CRL-3216
MCF7	ATCC	ATCC HTB-22
T47D	ATCC	ATCC HTB-133
Experimental Models: Organisms/Strains		
Mouse: female NSG	Jackson Laboratory	Stock No. 005557
shRNA and Oligonucleotides		
	Clone ID	Catalog number
h. shRNA targeting sequence: UBE2T 1#:	TRCN0000004386	RHS3979-9573127
h. shRNA targeting sequence: UBE2T 2#:	TRCN0000004389	RHS3979-9573130
h. shRNA targeting sequence: TFAP2A 1#:	TRCN0000004923	RHS3979-9573708
h. shRNA targeting sequence: TFAP2A 2#:	TRCN0000004925	RHS3979-9573710
h. shRNA targeting sequence: IFI6 1#:	V3LHS_402972	RHS4430-101099309
h. shRNA targeting sequence: IFI6 2#:	V3LHS_402973	RHS4430-101099309

h. ACTINB qPCR Forward primer:	gcatggagtcctgtggcatc	
h. ACTINB qPCR Reverse primer:	ttctgcatcctgtcggcaat	
h. UBE2T qPCR Forward primer:	gtgcatcccaggcagctctt	
h. UBE2T qPCR Reverse primer:	ggggtggctctgtggctaac	
h. IFI6 qPCR Forward primer:	ctggtctgcgatcctgaatg	
h. IFI6 qPCR Reverse primer:	agaggttctgggagctgctg	
h. TFAP2A qPCR Forward primer:	ctctcgctcctcagctccac	
h. TFAP2A qPCR Reverse primer:	tgagcagggtaacgttggca	
Recombinant DNA		
IFI6-ORF	Open biosystems	Cat# OHS6085-213573285
Commercial assays and kits		
CUT&RUN Assay Kit	Cell Signaling	Cat# 86652
Real Time-Glo Annexin V apoptosis reagent	Promega	Cat# JA1011
Software and Algorithms		
Prism 8.0	GraphPad	www.graphpad.com/scientific software/prism
ImageJ	https://imagej.nih.gov/ij	N/A
Other		
UBE2T and TFP2A mRNA expression in normal and breast cancer samples were analyzed and represented as box plot.	Oncomine Research Premium Edition	https://www.oncomine.org/resource/login.html
UBE2T and IFI6 mRNA expression in normal and breast cancer samples	Gene Expression Profiling Interactive Analysis (GEPIA)	http://gepia.cancer-pku.cn
